# Application of m^6^A and TME in Predicting the Prognosis and Treatment of Clear Cell Renal Cell Carcinoma

**DOI:** 10.1155/2022/2910491

**Published:** 2022-03-04

**Authors:** Dongchen Pei, Chaojie Xu, Dong Wang, Xiaoxue Shi, Yurui Zhang, Yi Liu, Nan Liu, Jianhua Guo, Yang Yu, Zhengjun Kang, Haipeng Zhu

**Affiliations:** Department of Urology, The Fifth Affiliated Hospital of Zhengzhou University, Zhengzhou University, Zhengzhou, Henan Province, China

## Abstract

**Background:**

Previous studies have shown that RNA N6-methyladenosine (m^6^A) plays an important role in the construction of the tumor microenvironment (TME). However, how m^6^A plays a role in the TME of clear cell renal cell carcinoma remains unclear.

**Methods:**

Based on 23 m^6^A modulators, we applied consensus cluster analysis to explore the different m^6^A modification profiles of ccRCC. The CIBERSORT method was employed to reveal the correlation between TME immune cell infiltration and different m^6^A modification patterns. A m^6^A score was constructed using a principal component analysis algorithm to assess and quantify the m^6^A modification patterns of individual tumors.

**Results:**

Three distinct m^6^A modification patterns of ccRCC were identified. The characteristics of TME cell infiltration in these three patterns were consistent with immune rejection phenotype, immune inflammation phenotype, and immune desert phenotype. In particular, when m^6^A scores were high, TME was characterized by immune cell infiltration and patient survival was higher (*p* < 0.05). When m^6^A scores were low, TME was characterized by immunosuppression and patient survival was lower (*p* < 0.05). The immunotherapy cohort confirmed that patients with higher m^6^A scores had significant therapeutic advantages and clinical benefits.

**Conclusions:**

The m^6^A modification plays an important role in the formation of TME. The m^6^A scoring system allows the identification of m^6^A modification patterns in individual tumors, discriminates the immune infiltrative features of TME, and provides more effective prognostic indicators and treatment strategies for immunotherapy.

## 1. Introduction

Renal cell carcinoma is the most common tumor of the urinary system, and the most common histologic subtype is clear cell renal cell carcinoma (ccRCC) accounting for approximately 80%–90% of cases [1, 2]. Studies have shown that approximately 100,000 people die from renal cell carcinoma each year worldwide, and the incidence is increasing every year [3]. Notably, the prognosis of advanced clear cell renal cell carcinoma is poor, but the 5-year survival rate of early-stage patients is relatively favorable, reaching 90% [4]. Therefore, a new method for early diagnosis and better treatment of ccRCC is urgently needed to further improve the survival rate and survival time of patients.

The tumor microenvironment (TME) refers to the internal and external environment, in which tumors occur, grow, metastasize, and tumor cells reside. It includes not only the tumor cells but also their surrounding fibroblasts, immune and inflammatory cells, glial cells, and various other cells, and the extracellular matrix (ECM), microvasculature, tumor-infiltrating immune cells (TIC), and biomolecules infiltrating in the nearby area [5, 6]. TME plays an important role in tumor progression, tumor metastasis, and tumor drug resistance [7, 8]. A number of studies have pointed out the close association of TME with the development and prognosis of ccRCC [9].

The m^6^A is one of the most common modifications of RNA [10], which has been shown to have a wide range of effects on splicing, transport, localization, stability, and translation efficiency of precursor or mature mRNAs, specifically including RNA stabilization [11], translation [12], strange splicing [13, 14], and nuclear export [15]. In the post-transcriptional regulation of the transcriptome, m^6^A is known as a key regulator that intervenes and controls endocytosis. The m^6^A structure and function are mediated by a combination of methylation transferase (writer), demethylase (eraser), and methylation reading protein (reader). The main role of the m^6^A writer is to catalyze the m^6^A modification of adenosine on mRNA, including METTL3, METTL14, METTL16, RBM15, VIRMA, WTAP, and ZC3H13 [16]. The m^6^A eraser mainly serves to demethylate the bases that have undergone m^6^A modification, including FTO and ALKHB5[17, 18]. The reader, also known as m^6^A-binding protein, includes YTHDF1, YTHDF2, YTHDC1, YTHDC2, IGFBP1, IGFBP2, IGF2BP3, RBMX, and HNRNPC, which is mainly responsible for binding to m^6^A sites and exerting specific regulatory effects on the modified RNA [19]. Notably, m^6^A modification during tumor development enables TME remodeling, affecting immune cell survival, proliferation, polarization, migration, and function. These processes help to protect tumor cells from clearance, inhibit cell death, and promote proliferation to further aid tumor cell proliferation and immune escape [20–24].

In particular, there is a correlation between the infiltration of immune cells in TME and m^6^A modification [25]. For example, METTL3 activates dendritic cells and initiates activation of cytotoxic T lymphocytes by increasing m^6^A levels of CD40, CD80, and TLR4 [26]; METTL3 overexpression promotes gastric cancer progression (GC) and liver metastasis through angiogenic and glycolytic pathways [27]. These studies suggest that m^6^A-regulated post-transcriptional modifications in TME interfere with immune cell infiltration.

Based on the above background, this study aimed to investigate the characteristics of immune cell infiltration in TME mediated by multiple m^6^A regulators, deepen the understanding of post-transcriptional modifications interfering with TME immune regulation, and further confirm the interaction between m^6^A modifications and TME in tumorigenesis and progression.

In this study, we analyzed genomic information from 569 patients with clear cell renal cell carcinoma, comprehensively assessed m^6^A modification patterns, and regressed the correlation between m^6^A modification patterns and the characteristics of TME cell infiltration. After combining the m^6^A features in TME, we found three distinct m^6^A modification patterns and high concordance with immune rejection phenotype, immunoinflammatory phenotype, and immune inert phenotype, respectively. This further confirms that m^6^A modifications play a significant role in shaping the TME profile. To further validate the relationship between m^6^A features and clinical phenotypes, we developed a scoring system to quantify the m^6^A modification pattern in individual patients and initially achieved to discriminate the immune response phenotype of tumors by m^6^A scoring.

## 2. Methods

### 2.1. Source and Preprocessing of Renal Clear Cell Carcinoma Dataset

**Figure 1:**
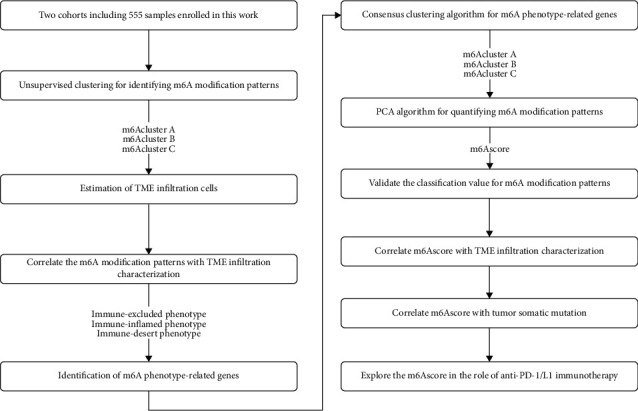
The flow chart of our study of identifying hallmark genes and candidate agents.

As shown in Figure 1, two cohorts of the Comprehensive Gene Expression (GEO) and The Cancer Genome Atlas (TCGA) databases, GSE29609 and TCGA-KIRC (Cancer Genome Atlas-Kidney Renal Clear Cell Carcinoma), were used in this study, with all cases containing survival information. We use the GSE29609 microarray data of Affymetrix Human Genome U133 Plus 2.0 Array and directly download the normalized matrix file. For TCGA-KIRC, we downloaded the transcriptome data (FPKM value) and clinical annotations from the genome data sharing (GDC, https://portal.gdc.cancer.gov/). Then, we use the “limma” *R* package to convert the FPKM value to a million parts per million (TPM) value. Next, we combined the mRNA expression matrices of our two cohorts and used the “limma” and “sva” packages to correct for batch effects caused by nonbiotech deviations. We downloaded simple nucleotide variation data from the TCGA database to further analyze copy number variation (CNV).

### 2.2. Unsupervised Clustering of 23 m^6^A Regulators

We selected 23 recognized m^6^A regulators, including 8 writers (METTL3, METL14, METL16, RBM15, RBM15B, WTAP, VIRMA, and ZC3H13), 2 erasers (ALKBH5 and FTO), and 13 readers (YTHDC1, YTHDC2, YTHDF1, YTHBDF3DF2, IGFBP2, IGFBP3, HNRNPA2B1, HNRNPC, FMR1, LRPPRC, and RBMX). We extracted the m^6^A-related gene expression matrix of the combined sample through the “limma” package based on 23 m^6^A regulatory factors. We use unsupervised cluster analysis to classify all patients and determine different m^6^A modification patterns based on the expression differences of 23 m^6^A regulatory factors for further analysis. In sample clustering, the Euclidean distance is selected as the clustering statistics, and the K-means clustering method is used to achieve the clustering purpose. We used the “ConsensusClusterPlus” package to do the above and repeated it by 1,000 times to ensure the stability of the clustering.

### 2.3. Genomic Variation Analysis (GSVA) and Functional Annotation

We use the “GSVA” package to perform GSVA enrichment analysis to study the changes in biological processes under m^6^A modification. GSVA is a nonparametric and unsupervised method. GSVA analyzed the “c2.cp.kegg” downloaded from the “MSigDB database.v6.2.symbols” gene set. The difference is significant when the adjusted *p* value is less than 0.05. The first 20 paths are selected to pull the path heatmap. These analyses were completed by the following packages: “limma,” “GSEABase,” “GSVA,” and “pheatmap.”.

### 2.4. Estimation of TME Cell Infiltration

The relative abundance of each cell infiltration in the sample was quantified by the ssGSEA (single-sample gene-set enrichment analysis) algorithm. We obtained genes related to TME-infiltrating immune cells from Charoentong's study, including activated B cell, CD4 T cell, CD8 T cell, type 17 T helper cell, eosinophil monocyte, and so on. The relative abundance of each immune cell in sample TME was evaluated by ssGSEA score. The differential immune-infiltrating cells were shown by box diagram. The above analysis is performed by *R* software. The above analysis was performed by “reshape2,” “ggpubr,” “limma,” “GSEABase,” and “GSVA” packages.

### 2.5. Screening of Differentially Expressed Genes (DEGs) with Different m^6^A Modification Patterns

In order to identify the different m^6^A modification patterns of DEGs, we use the empirical Bayes method of the “limma” package to determine DEGs, calculate the expression level and *p* value of DEGs, and adjust *p* < 0.001 as the significance standard of DEGs. We use Gene Ontology (GO) data and Kyoto Encyclopedia of Genes and Genomes (KEGG) to analyze the differential pathways of cell composition (CC), biological process (BP), molecular function (MF), and DEG, respectively. The threshold used in screening GO and KEGG was set as *p* < 0.05.

### 2.6. Generation of m^6^A Gene Signature

We built a scoring system to quantify the m^6^A modification pattern of a single ccRCC patient through the m^6^A gene signature, which we call m^6^Ascore. The specific process is as follows.

First of all, we used univariate Cox regression analysis of the DEGs screened above to screen out the DEGs that are significant for prognosis. Next, we conduct an unsupervised cluster analysis of DEGs with significant prognosis in order to divide the patients into several groups for further analysis. In the sample clustering, the Euclidean distance is selected as the clustering statistics, and the K-means clustering method is used to achieve the clustering purpose. In addition, we also used principal component analysis (PCA) to construct the m^6^A gene signature for DEGs with significant prognosis. We choose the first and second principal components as the signature score, and they are not related to each other. We use the following formula to define the m^6^Ascore:

(1) $$\begin{array}{c} \text{m}6\text{Ascore}=\sum \left(\text{PC}1_{i}+\text{PC}2_{i}\right), \end{array}$$

where *i* represents the expression of m^6^A-related genes.

### 2.7. The Role of m^6^A Score

In order to prove the clinical guidance of grouping according to m^6^A score, we analyzed the relationship between m^6^A score and immune-infiltrating cells, clinical stage, patient age, tumor grade, m^6^A modification pattern, m^6^A gene cluster classification, and immunotherapy-related genes. *p* < 0.05 is considered statistically different.

### 2.8. Statistical Analysis

When analyzing differences between groups, a one-way analysis of variance was used for parametric tests, and the Kruskal-Wallis test was used for nonparametric tests. Spearman's correlation analysis was used to calculate the correlation between m^6^A scores and TMB. The Kaplan-Meier method was used to draw the survival curve, and then, the log-rank test was used to determine the significance of the difference. The samples were scored by the PCA method and then combined with the patient's survival information, the best cutoff value was determined by the “survminer” R software package and divided into two groups (high m^6^A score group and low m^6^A score group). Univariate and multivariate Cox regression models were used to calculate hazard ratios (HRs) and independent prognostic factors for m^6^A regulatory genes and m^6^A phenotype-related genes. The “RCircos” *R* package draws a circle diagram of the 23 chromosomal mutation positions of the 23 m^6^A regulatory factors. The waterfall is drawn by the “maftools” package. The block diagram is drawn by the “ggpubr” package. All *p*-values are bilateral, and *p* < 0.05 is statistically significant. All data processing is completed by *R* 4.0.5 software.

## 3. Results

### 3.1. Genetic Variation of m^6^A Regulatory Factor in Renal Clear Cell Carcinoma

**Figure 2:**
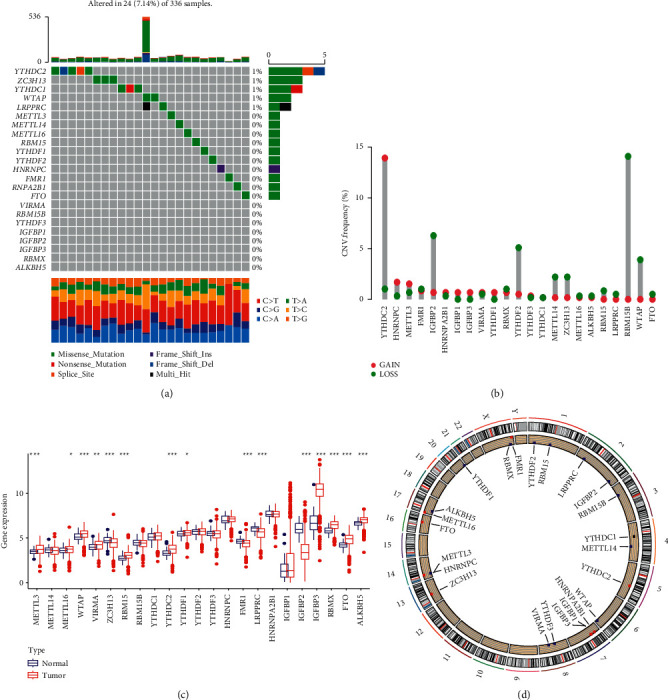
(a) The mutation frequency of 23 m^6^A regulatory factors in 336 patients with clear cell renal cell carcinoma from the TCGA-STAD cohort. Each column represents an individual patient. The bar graph above shows TMB, and the numbers on the right indicate the mutation frequency of each regulator. The bar graph on the right shows the proportions of each variant type. The stacked bar chart below shows the conversion rate in each sample. (b) Frequency of CNV changes in m^6^A modulators in the GSE29609 cohort. The height of the column represents the frequency of change. Delete frequency, blue dot; zoom in frequency, red dot. (c) The expression of 23 m^6^A expression factors between normal tissues and clear cell renal cell carcinoma tissues. Tumor, red; normal, blue. The upper and lower ends of the box represent the interquartile range of values. The line in the box represents the median value, and the red or blue dots represent outliers. The asterisk represents the statistical *p* value (^∗^*p* < 0.05; ^*∗∗*^*p* < 0.01; and ^*∗∗∗*^*p* < 0.001) (d) The position of the CNV change in the m^6^A regulatory factor on the 23 chromosomes of the GSE29609 cohort.

In this study, we used 23 m^6^A regulators to assess the m^6^A signature of TME. First, we summarized the copy number variation and the incidence of somatic mutations in 23 m^6^A regulators in clear cell renal cell carcinoma. In 336 samples, 24 m^6^A regulators were mutated with a mutation frequency of 7.14%, with mutations mainly occurring in YTHDC2, ZC3H13, YTHDC1, WTAP, and LRPPRC (Figure 2(a)). The study of the frequency of CNV changes showed that 23 regulators had changes in CNV. Among them, the copy number of regulator YTHDC2 increased, while the copy numbers of RBM15B, IGFBP2, YTHDF2, WTAP, METL14, and ZC3H13 decreased (Figure 2(b)). Meanwhile, the location of CNV changes in the m^6^A regulator on the chromosome is shown in Figure 2(d).

To determine whether the above gene mutations affect the expression of the m^6^A regulator in patients with clear cell renal cell carcinoma, we also investigated the differences in the expression of m^6^A regulators between normal and clear cell renal cell carcinoma patients and found that CNV changes may be important for the disruption of m^6^A regulator factor expression. Compared with normal kidney tissue, the expression of m^6^A CNV amplification regulator YTHDC2 in clear cell renal cell carcinoma tissue was significantly increased (*p* < 0.05). The expression of CNV-deficient m^6^A regulators IGFBP2 and ZC3H13 clear cell renal cell carcinoma was significantly reduced (Figure 2(c)). This indicates that there are differences in the gene expression levels of m^6^A regulatory factors in normal kidney tissue and clear cell renal cell carcinoma tissue. At the same time, we conducted a survival analysis on the high and low expression of each regulator in ccRCC, and the results showed that the difference in expression of the 20 regulators had a significant impact on the prognosis of ccRCC (Figures S1A-T). Therefore, we believe that the imbalance in the expression of m^6^A regulatory factors may play an important role in the occurrence and development of clear cell renal cell carcinoma.

### 3.2. Exploring the 23 Modulator-Mediated m^6^A Methylation Modification Patterns

**Figure 3:**
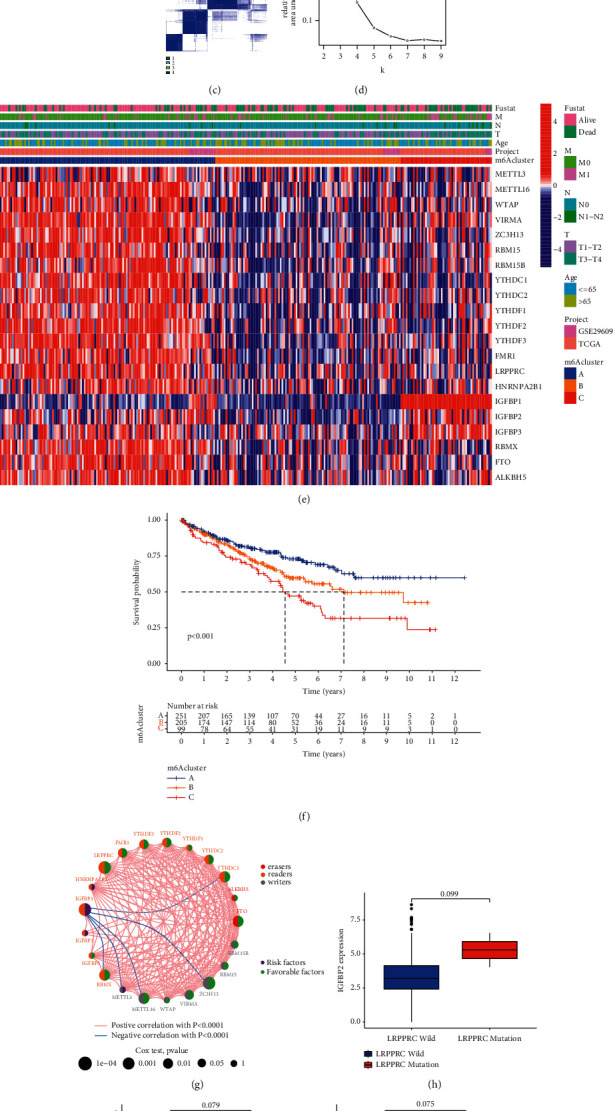
The patterns of m^6^A methylation modification and the biological characteristics of each pattern. (a–d) Using unsupervised cluster analysis to show that ccRCC can be divided into three different genotypes (*k* = 3). (e) Unsupervised clustering of 23 m^6^A regulatory factors in the clear cell renal cell carcinoma cohort. Survival status, clinical stage, age, project, and m^6^A cluster are used as patient annotations. Red represents the high expression of regulatory factors, and blue represents low expression. (f) Survival analysis based on three m^6^A modification patterns of 555 gastric cancer patients from 1 GEO cohort, including 251 cases in m^6^A cluster-A, 205 cases in m^6^A cluster-B, and 99 cases in m^6^A cluster-C. The Kaplan-Meier curve with a log-rank *p*-value of 0.011 shows a significant difference in survival between the three m^6^A modification modes. The overall survival rate of m^6^A cluster-A is significantly better than the other two m^6^A clusters. (g) Interaction between m^6^A regulatory factors in clear cell renal cell carcinoma. The size of the circle represents the influence of each adjusting factor on the prognosis, and the numerical ranges calculated by the log-rank test are *p* < 0.001, *p* < 0.01, *p* < 0.05, and *p* < 0.1, respectively. The purple dots in the circle are prognostic risk factors; the green dots in the circle are prognostic protective factors. The lines connecting the regulators show their interaction, and the thickness shows the relative strength between the regulators. Negative correlations are marked in blue, and positive correlations are marked in red. Writers, erasers, and readers are marked in gray, red, and orange. (h–j) Correlation between the regulators.

There were two cohorts GSE 29609 and TCGA-KIRC in this study. Their basic information and clinical features are shown in Table S1. The m^6^A regulator network depicts a comprehensive map of m^6^A regulator interactions, regulator connections, and their impact on the prognosis of ccRCC patients (Figure 3(g) and Table S2). We separately analyzed the gene expression differences between the mutant genes of the three regulatory factors YTHDC2, WTAP, and LRPPRC with higher mutation frequency and the wild-type genes. Among them, compared with the mutant YTHDC2 subgroup, the wild-type YTHDC2 subgroup has a higher level of gene expression of the regulatory factor FMR1. In contrast, the gene expression level of regulator METL16 was higher in the mutant WTAP subgroup compared to the wild-type WTAP subgroup. Similarly, the regulatory factor IGFBP2 was significantly upregulated in LRPPRC mutants (Figures 3(h)–3(n)). This indicates that not only the expression of m^6^A regulators in the same functional category is correlated but also between writers, erasers, and readers.

The above results indicate that the crosstalk between writer, reader, and erase regulators may form different m^6^A modification patterns between different tumors, and it also plays a key role in the formation of TME cell infiltration characteristics.

According to the expression of 23 m^6^A regulatory factors, the *R* package “ConensusClusterPlus” was used to qualitatively classify the m^6^A modification patterns, and finally, three different modification patterns were identified through unsupervised clustering (Figures 3(a)–3(d)). We refer to these patterns as m^6^A clusters A, B, and C (Figure 3(e) and Table S3). We found that most regulatory factors are highly expressed in the m^6^A cluster-A modification mode, low in the m^6^A cluster-B modification mode, and moderately expressed in the m^6^A cluster-C modification mode.

Then, we analyzed the survival of the three modification modes and found that ccRCC patients showed a particularly significant survival advantage in the m^6^A cluster-A modification mode, while the m^6^A cluster-C modification mode had the worst prognosis (Figure 3(f)).

### 3.3. Infiltration Characteristics of TME Cells in Different m^6^A Modes

**Figure 4:**
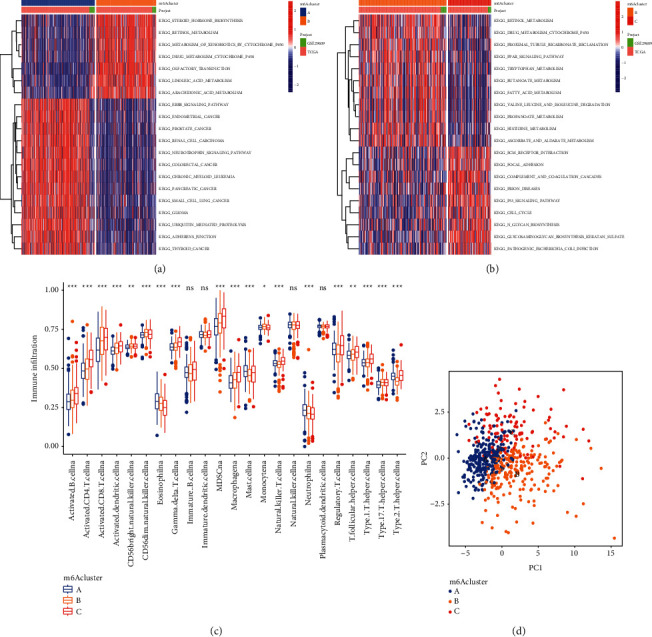
(a-b) GSVA enrichment analysis shows the activation status of biological pathways in different m^6^A modification modes. Heatmaps are used to visualize these biological processes. Red represents activated pathways, and blue represents inhibited pathways. The clear cell renal cell carcinoma cohort was used as sample annotation. A m^6^A cluster A and m^6^A cluster B; B m^6^A cluster B and m^6^A cluster C. (c) The abundance of each TME-infiltrating cell in the three m^6^A modification modes. The upper and lower ends of the box represent the interquartile range of values. The lines in the boxes represent the median value, and the black dots represent the outliers. The asterisk represents the statistical *p* value (^*∗*^*p* < 0.05; ^*∗∗*^*p* < 0.01; and ^*∗∗∗*^*p* < 0.001). (d) The principal component analysis of the transcriptome profile of the three m^6^A modification patterns shows significant differences in the transcriptome among different modification patterns.

In order to explore whether there are differences in biological behavior between different m^6^A modification modes, we performed GSVA enrichment analysis (Figures 4(a) and 4(b)). As shown in the figure, m^6^A cluster-A has rich oncogenic activation pathways, m^6^A cluster-B is rich in metabolic pathways, and m^6^A cluster-C is rich in interstitial activation pathways.

Subsequent TME cell ssGSVA differential analysis showed (Figure 4(c) and Table S4) that in m^6^A cluster-A, activated B cells, activated CD4+ T cells, activated CD8 T cells, activated dendritic cells, etc. were significantly reduced, but at the same time, this type also shows the best survival advantage. The m^6^A cluster-C has a large number of immune cell infiltration.

Usually, we divide the characteristics of TME infiltration into three categories, namely, immunoinflammatory phenotype, immune rejection phenotype, and immune desert phenotype. The immunoinflammatory phenotype is characterized by the presence of a large number of CD4 and CD8 T cells in the tumor parenchyma, usually accompanied by myeloid cells and monocytes; the immune rejection phenotype has obvious immune rejection reactions, characterized by the presence of more immune cells, but these immune cells can only stay in the matrix surrounding the tumor cell nest and cannot penetrate the tumor tissue; the immune desert phenotype refers to the absence of immune cell infiltration, which is characterized by the lack of T cells in the tumor tissue or matrix. We usually think that the immune desert phenotype and immune rejection phenotype are both noninflammatory tumors.

Therefore, we classified the m^6^A modification patterns with different TME characteristics into three types. Among them, A is the immune desert phenotype, characterized by the lack of immune cell infiltration; B is the immune rejection phenotype, characterized by a certain degree of immune cell infiltration with immunosuppressive effects; and C is the immunoinflammatory phenotype, characterized by a large infiltration of immune CD4^+^and CD8^+^ T cells.

Then, we performed principal component analysis on the transcriptome profile of the m^6^A modification pattern, and the results showed that there are significant differences between the m^6^A cluster A, m^6^A cluster B, and m^6^A cluster C transcriptomes (Figure 4(d)).

### 3.4. Generation of m^6^A Gene Cluster and Gene Function Analysis of DEGs

**Figure 5:**
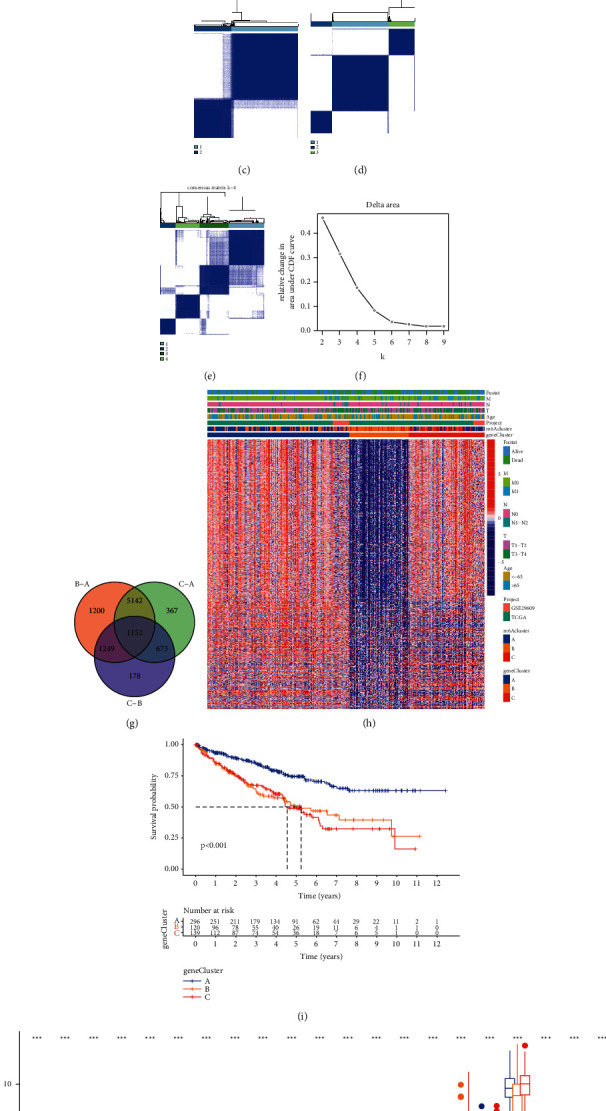
(a-b) Function annotation, m^6^A-related genes using GO enrichment analysis, and KEGG enrichment analysis. The color depth of the bubble chart represents the number of enriched genes, and the size of the bubble chart represents the proportion of gene expression. Unsupervised clustering of overlapping m^6^A phenotype-related genes in the C clear cell renal cell carcinoma cohort to classify patients into different genomic subtypes, respectively, is called m^6^A gene clusters A-C. Survival status, clinical stage, age, m^6^A clusters, and gene clusters were used as patient annotations. (c–f) Using unsupervised cluster analysis to show that ccRCC can be divided into three different genotypes (*k* = 3). (g) A total of 1,152 DEGs were obtained from the three types. (h) Three different m^6^A modified genome phenotypes were discovered through an unsupervised clustering algorithm, and these three clusters were named m^6^A gene clusters A, B, and C. (i) Kaplan-Meier curve showed that the m^6^A modified genome phenotype was significantly correlated with the overall survival of 555 patients in the clear cell renal cell carcinoma cohort, including 296 gene cluster A 120 gene cluster B and 139 gene cluster C (*p* < 0.0001, log-rank test). (j) The expression of 23 m^6^A regulatory factors in 3 gene clusters. The upper and lower ends of the box represent the interquartile range of values. The line in the box represents the median value, and the red or yellow dots represent the outliers. The asterisk represents the statistical *p* value (^*∗*^*p* < 0.05; ^*∗∗*^*p* < 0.01; and ^*∗∗∗*^*p* < 0.001). One-way analysis of variance is used to test the statistical differences between the three gene clusters.

We used the limma software package to identify 1,152 DEGs (Figure 5(g)) associated with the m^6^A phenotype to study the potential biological behavior of each m^6^A modification mode in one step (Table S5). The clusterProfiler software package is used for the GO and KEGG enrichment analysis of DEG.

The results indicate that these genes are highly related to biological processes that are significantly related to m^6^A modification and immunity (Figures 5(a) and 5(b)). For example, GO enrichment analysis is performed on DEGs, and the results of significant enrichment are shown in Table S6. DEGs show the enrichment of biological processes, indicating that m^6^A is modified in CC, BP, and MF, such as cell substrate linkage and mRNA catabolism (Figure 5(a)). Using KEGG to analyze the differential pathways of DEGs, the results showed that the ERbB signaling pathway, p53 signaling pathway, mTOR signaling pathway, and mRNA monitoring pathway were enhanced (Figure 5(b)).

In order to further verify this regulatory mechanism, we performed an unsupervised cluster analysis of the 1,152 m^6^A phenotype-related genes obtained, hoping to divide patients into different genomic subtypes. We discovered three different m^6^A modified genome phenotypes through an unsupervised clustering algorithm (Figures 5(c)–5(f)) and named these three clusters as m^6^A gene clusters A, B, and C (Figure 5(h)). We found that the surviving patients were mainly concentrated in m^6^A gene cluster A (Figure 5(h)). At the same time, the survival curve shows that the three modified genome phenotypes are significantly related to the survival rate of patients (Figure 5(i)). Among 555 patients with clear cell renal cell carcinoma, the number of A gene clusters is the largest, 296 cases, which should be related to the better prognosis of patients with this gene cluster. The prognosis of patients with B gene cluster (129 cases) and C gene cluster (139 cases) was poor (Figure 5(i)). In the three m^6^A gene clusters, significant differences in the expression of m^6^A regulatory factors were observed. Most genes are highly expressed in the A gene cluster, the B gene cluster is low, and the C gene cluster is in the middle (Figure 5(j)).

### 3.5. Relationship between m^6^A Score and m^6^A-Related Phenotypes

From the above, we can find that the methylation modification of m^6^A plays an important role in the formation of the uniqueness of the TME landscape. However, the above analysis is based on the patient population and cannot accurately predict the m^6^A methylation modification pattern of individual patients. Due to the complexity of m^6^A modification patterns and individual heterogeneity, we constructed a scoring system to quantify the m^6^A modification patterns of individuals with clear cell renal cell carcinoma, which we call m^6^Acore, and the m^6^A score was constructed according to the m^6^A modification pattern of DEGs by the PCA Table S7.

**Figure 6:**
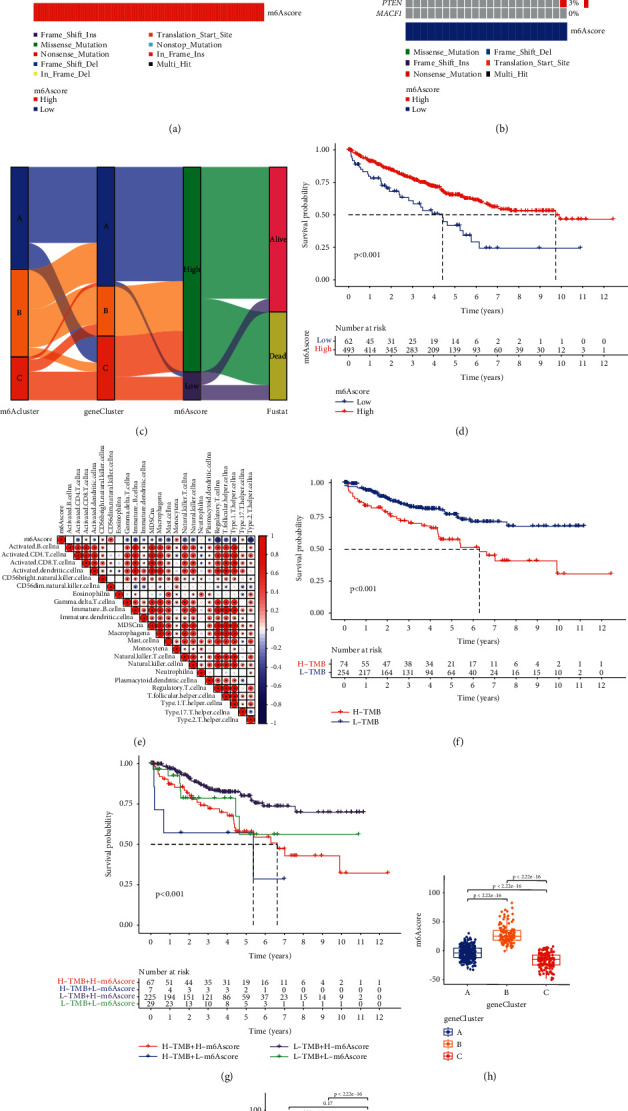
(a-b) Waterfall plots of tumor somatic mutations established by those with high m^6^A score (a) and low m^6^A score (b). Each column represents a single patient. The bar graph above shows TMB, and the numbers on the right indicate the mutation frequency of each gene. The bar graph on the right shows the proportion of each variant type. (c) The Sankey diagram shows the changes in m^6^A clusters, gene clusters, m^6^A score, and survival status. (d) Survival analysis of low (62 cases) and high (493 cases) m^6^A score patient groups in the TCGA-KIRC cohort, using Kaplan-Meier curve (HR, 1.81 (1.26–2.62); *p* < 0.001, log-rank test). (e) Spearman's correlation analysis was used to analyze the correlation between m^6^A score and immune cells in the clear cell renal cell carcinoma cohort. Negative correlations are marked in blue, and positive correlations are marked in red. (f) Using the Kaplan-Meier curve to analyze the survival rate of patients with low (254 cases) and high (74 cases) tumor mutations in the clear cell renal cell carcinoma cohort (HR, 1.81 (1.26–2.62); *p* < 0.001, log-level test). (g) Kaplan-Meier curve is used to analyze survival by m^6^A score and TMB score. H high; L low. (h-i) Differences in m^6^A score between the three gene clusters and the three types in the clear cell renal cell carcinoma cohort. The Kruskal-Wallis test was used to compare the statistical differences between the three gene clusters (*p* < 0.001).

We use the Sankey chart to show the changes in the attributes of a single patient (Figure 6(c)). We found that most patients with m^6^A cluster-A were genotyped as m^6^A gene cluster A and had a higher m^6^Ascore, and most patients survived. We found that most genotypes of m^6^A cluster-B patients are m^6^A gene clusters A and B, with high m^6^Acore, and most patients survived. We found that most genotypes of m^6^A cluster-C patients were m^6^A gene cluster C, with a low m^6^Acore, and most patients died.

The Kruskal-Wallis test showed that there are significant differences in the m^6^A value between the m^6^A gene clusters. Gene cluster C has the lowest median score, and gene cluster B has the highest median score (Figures 6(h)–6(i)).

We use the survminer software package to determine the best cutoff value, divide patients into low m^6^A value group and high m^6^A value group according to m^6^A value, and predict the prognosis of high and low groups. The results of the Kruskal-Wallis test showed that patients with high m^6^A scores showed a significant survival benefit (Figure 6(d)). Then, we used the maftools software package to analyze the difference in mutation distribution in the population with low m^6^A value and high m^6^A value in the TCGA-KIRC cohort. As shown in Figures 6(a) and 6(b), the high m^6^A value group showed a broader tumor mutation burden than the low m^6^A value group. Survival analysis showed that ccRCC patients with high TMB had a better prognosis (Figure 6(f)). The subgroup with low TBM and high m^6^A score had the best prognosis, while the subgroup with high TBM and low m^6^A score had the worst prognosis (Figure 6(g)). We speculate that this is because patients with low TMB have better clinical effects in immunotherapy.

We studied the correlation between immune cells and m^6^A scores and found that most immune cells are negatively correlated with m^6^A scores (Figure 6(e)), such as activated CD4 T cells and activated CD8 T cells. In contrast, the m^6^A score is positively correlated with activated CD8 T cells, CD56 bright natural killer cells, CD56 dim natural killer cells, monocytes, and type 17 T helper cells.

### 3.6. Clinical Application of m^6^A Score and the Role of m^6^A Modification Mode in Anti-PD-1/L1 Immunotherapy

**Figure 7:**
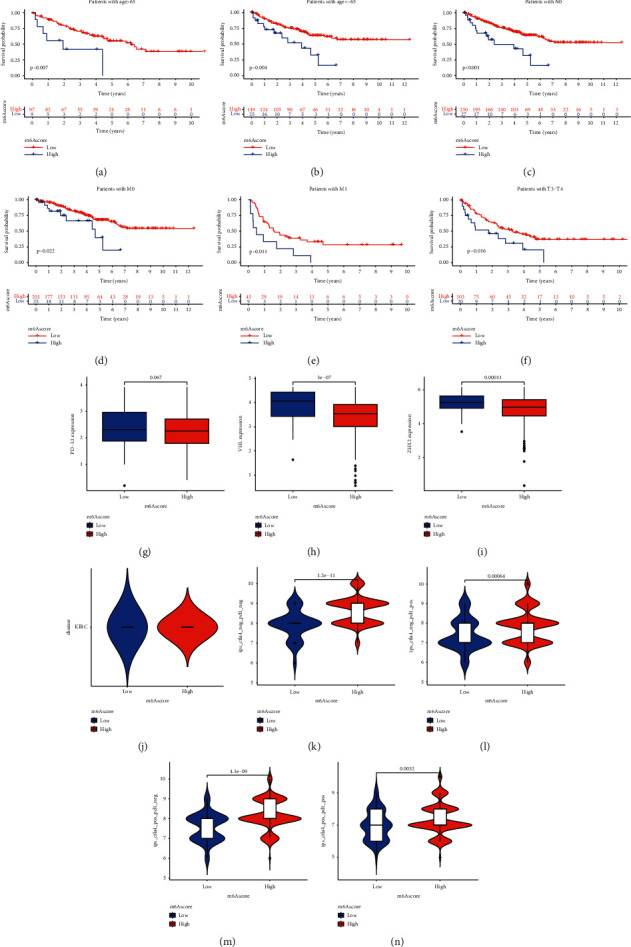
(a–f) Kaplan-Meier curve is used to show the difference in survival rate between the high group and low group in age and clinical stage (*p* < 0.05 is meaningful). (g–i) The difference in the expression levels of the three immune markers in the high- and low-score groups (*p* < 0.1 is meaningful). (j) The distribution of scoring scores between the high and low groups in ccRCC, and the difference between the high and low groups, and the black bars represent the median. (k–n) No anti-PD-1/L1 immunotherapy and anti-CTLa4 immunotherapy, anti-PD-1/L1 immunotherapy alone, anti-CTLa4 immunotherapy alone, anti-PD-1/L1 immunotherapy and anti-PD-1/L1 immunotherapy, and anti-PD-1/L1 immunotherapy alone were separately received. The different efficacy of CTLa4 immunotherapy in the high- and low-score groups when the two combined immunotherapy is combined, and the black bars represent the median.

First, we use the violin chart to show the distribution and difference of the scores of patients in the m^6^A high and low groups (Figure 5(j)). We found that the distribution of the scores of patients in the high- and low-score groups was different, but the median was about the same. Subsequently, we used the m^6^A score to study the clinical characteristics of KIRC. It was found that patients in the m^6^A high-level group of all ages had significantly better survival rates than those in the m^6^A low group, as were the clinical stages and tumor grades, such as M0 stage, M1 stage, N0 stage, and T3-T4 stage (Figures 7(a)–7(f)).

Based on two immunotherapy cohorts, we investigated whether m^6^A modification signals can predict patient response to immune checkpoint blockade therapy. We found whether they receive anti-PD-1/L1 immunotherapy and anti-CTLa4 immunotherapy alone or receive combined immunotherapy at the same time, and patients in high groups can obtain greater clinical benefits (Figures 7(k)–7(n)).

## 4. Discussion

Immune cells recruited during tumor development are remodeled by aberrantly functioning TME, affecting immune cell survival, proliferation, differentiation, migration, and function. These processes help to protect tumor cells from clearance, inhibit cell death, and promote proliferation to further aid tumor cell migration and metastasis. Most of the current studies have focused on immunomodulation of tumors [28], focusing on addressing the sustained suppression of adaptive immune responses by TME [29]. Encouragingly, previous studies have demonstrated a strong association between m^6^A methylation and tumor immunotherapy; for example, studies have reported a broad regulatory mechanism of m^6^A on the tumor microenvironment in gastric cancer [25]. However, the role of m^6^A modification models in regulating tumor immunity in ccRCC has not been fully elucidated, and it is unclear whether the overall m^6^A-associated regulators in TME can lead to a series of transformations in “tumor immunity.”

A limitation of previous studies on TME may be that they have focused on trends in only a few immune cells. For example, baseline levels of CD4+/CD8+ T cells, macrophage M1, and NK cells, and baseline levels of inflammatory cytokines in tumor invasion have been associated with immune responses [29–31]. In contrast, most studies targeting m^6^A modulators in TME have also focused on a single TME cell type (macrophages and CD8 T cells) or a single m^6^A modulator, which has led to the characterization of TME immune cell infiltration mediated by the combined effects of multiple m^6^A modulators not being adequately studied [25]. Therefore, it is important to investigate the role of the m^6^A modification patterns in ccRCC TME cell infiltration.

In this study, we used 23 m^6^A regulators to reveal three different m^6^A modification patterns. These three types of TME cell infiltration were markedly different. Class A was characterized by immunosuppression and corresponded to the immune desert phenotype. Class B was characterized by natural immunity and stromal activation and corresponded to the immune rejection phenotype. Class C was characterized by adaptive immune activation and corresponded to the immunoinflammatory phenotype.

Overall, the immunoinflammatory phenotype is referred to as a “hot” tumor, which is characterized by a large infiltration of immune cells in the TME [32–34], while conversely the immune rejection phenotype and the immune desert phenotype are considered as “cold” tumors. Although the immune rejection phenotype also shows a large number of immune cells, the immune cells cannot penetrate the parenchyma of the tumor cells remaining in the stroma surrounding the tumor cell nests [35–37]. Previous studies of gastric cancer have shown that the immunoinflammatory phenotype has the best prognosis [25], but this time we came to a different conclusion for clear cell renal cell carcinoma. In our study, we found a better prognosis for ccRCC patients with m^6^A cluster A characterized by immune desert behavior. Because the immune desert phenotype has the strongest degree of immune tolerance and immunosuppression and lacks activated T cells [38], the immunosuppressive TME will accordingly change. An abnormal TME will strongly reshape the local immune cell level, thus affecting tumor cell survival, proliferation, differentiation/immune cell polarization, migration, and function. These processes can inhibit tumor cell death, promote tumor cell proliferation, and further support tumor migration and metastasis [39, 40]. All of the above studies suggest that the TME and immune characteristics of each tumor are different, so this may explain the better prognosis of the m^6^A cluster with immune desert type in ccRCC patients. Also, combined with the infiltration characteristics of TME cells in each cluster, we can confirm the correctness of the m^6^A score to determine the immunophenotypic classification.

Several studies have identified subtypes of ccRCC based on genomic analysis [41–43] and improved individualized treatment strategies for ccRCC based on different subtypes. However, genotyping based on differential gene expression associated with m^6^A has not been elucidated. In this study, we have demonstrated that mRNA transcriptome differences in different m^6^A modification patterns are significantly correlated with multiple immune-related biological pathways and that these differential genes are likely to result from m^6^A-related post-transcriptional modifications. Based on GO and KEGG analysis of these m^6^A signature genes, we found that differentially expressed genes were significantly associated with immune activation, which further demonstrates the importance of m^6^A modifications in the formation of different TME immune cell infiltration profiles.

To promote the significance of m^6^A features of TME in clinical diagnosis and treatment, we established a m^6^A scoring system to assess the m^6^A modification pattern in individual ccRCC patients. Through the evaluation, we found that m^6^A modification patterns characterized by immune rejection phenotype and immune desert phenotype had higher m^6^A scores and correspondingly better prognosis.

This study also noted that m^6^A modification in TME may affect the therapeutic effect of immune checkpoint blockade. Therefore, we investigated the immunotherapy of ccRCC. We found that in two cohorts receiving anti-PD-1 and anti-PD-L1 immunotherapy, the m^6^A score had predictive value for whether patients could receive immunotherapy. Furthermore, the m^6^A score can also be used to assess the clinicopathological characteristics of patients, such as tumor grade and clinical stage.

Of course, there remain some shortcomings in our study. First, our data were all from the TCGA database and the GEO database, and the sample size was not large enough, which may lead to bias in the results. Meanwhile, due to technical limitations, this study only started from the overall m^6^A regulators in the tumor TME for comprehensive scoring and did not analyze the impact of tumor driving or tumor suppression brought by the main targets of m^6^A modifications in it. Our overall m^6^A scoring mechanism may have limitations and may not be applicable for other types of samples, so the generalization of the conclusions in this study needs to be performed with caution. We will then examine one or two m^6^A regulators in more depth to explore how the antitumor effects of these regulators are regulated by numerous tumor suppressors and how they act in a highly coordinated manner. The findings of this study provide an ideal resource for a comprehensive analysis of m^6^A regulators and immune regulation, bringing to light that the characterization of TME mediated by multiple m^6^A regulators will help improve our understanding of cancer immunity. For further external validation, future multicenter, large sample, prospective double-blind trials are necessary to carry out.

## 5. Conclusions

In conclusion, the m^6^A score can comprehensively evaluate the modification patterns of m^6^A methylation in ccRCC patients and the corresponding characteristics of TME immune cell infiltration. Similarly, the m^6^A score can be used to assess m^6^A genotyping, clinical characteristics, and OS of ccRCC patients. More importantly, we can predict the clinical benefits of new immune checkpoint-blocking strategies (PD-1/L1 and CTLA4) based on the m^6^A score, and discover more effective immune targets, thereby improving the immunotherapy effect of ccRCC, and help to develop new immunotherapy drugs.

## Data Availability

All data used in this work can be acquired from the Gene Expression Omnibus (GEO; https://www.ncbi.nlm.nih.gov/geo/) and the GDC portal (https://portal.gdc.cancer.gov/).

## Abbreviations

m^6^A: N6-methyladenosine methylation
TME: Tumor microenvironment
ccRCC: Clear cell renal clear cell carcinoma
DEGs: Differentially expressed genes
PCA: Principal component analysis
CNV: Copy number variation
OS: Overall survival
GEO: Gene Expression Omnibus
TCGA: The Cancer Genome Atlas
TCGA-ACC: The Cancer Genome Atlas-Adrenocortical Carcinoma
GDC: Genomic data commons
FPKM: Fragments per kilobase million
TPM: Transcripts per kilobase million
GSVA: Gene set variation analysis
ssGSEA: Single-sample gene-set enrichment analysis algorithm
CC: Cellular component
BP: Biological process
MF: Molecular function
GO: Gene Ontology
KEGG: Kyoto Encyclopedia of Genes and Genomes
TMB: Tumor mutation burden
HR: Hazard ratios.

## Supplementary Materials

Supplementary Materials Figure S1. (A-T) Survival analysis results of the high and low expression of 20 regulatory factors in ccRCC. S1: Basic information of datasets included in this study for identifying distinct m^6^A methylation modification patterns. S2: Spearman's correlation analysis of the 21 m^6^A modification regulators. S3: Estimating relative abundance of tumor microenvironment cells in KIRC patients by the single-sample gene-set enrichment analysis (ssGSEA). S4: The activation states of biological pathways in distinct m^6^A modification patterns by GSVA enrichment analysis. S5: Prognostic analysis of m^6^A phenotype-related genes using a univariate Cox regression model. S6: Functional annotation for m^6^A phenotype-related genes (Gene Ontology-Biological process). S7: The changes in m^6^Aclusters, survival time, and m^6^A score.
