# Supplementary material for: Application of m6A and TME in Predicting the Prognosis and Treatment of Clear Cell Renal Cell Carcinoma
Source: J Oncol. 2022 Mar 4;2022:2910491. doi: 10.1155/2022/2910491 (PMC8916893; doi:10.1155/2022/2910491)
Supplement: Supplementary Materials — Figure S1. (A-T) Survival analysis results of the high and low expression of 20 regulatory factors in ccRCC. S1: Basic information of datasets included in this study for identifying distinct m6A methylation modification patterns. S2: Spearman's correlation analysis of the 21 m6A modification regulators. S3: Estimating relative abundance of tumor microenvironment cells in KIRC patients by the single-sample gene-set enrichment analysis (ssGSEA). S4: The activation states of biological pathways in distinct m6A modification patterns by GSVA enrichment analysis. S5: Prognostic analysis of m6A phenotype-related genes using a univariate Cox regression model. S6: Functional annotation for m6A phenotype-related genes (Gene Ontology-Biological process). S7: The changes in m6Aclusters, survival time, and m6A score. [file 2910491.f1.pdf]

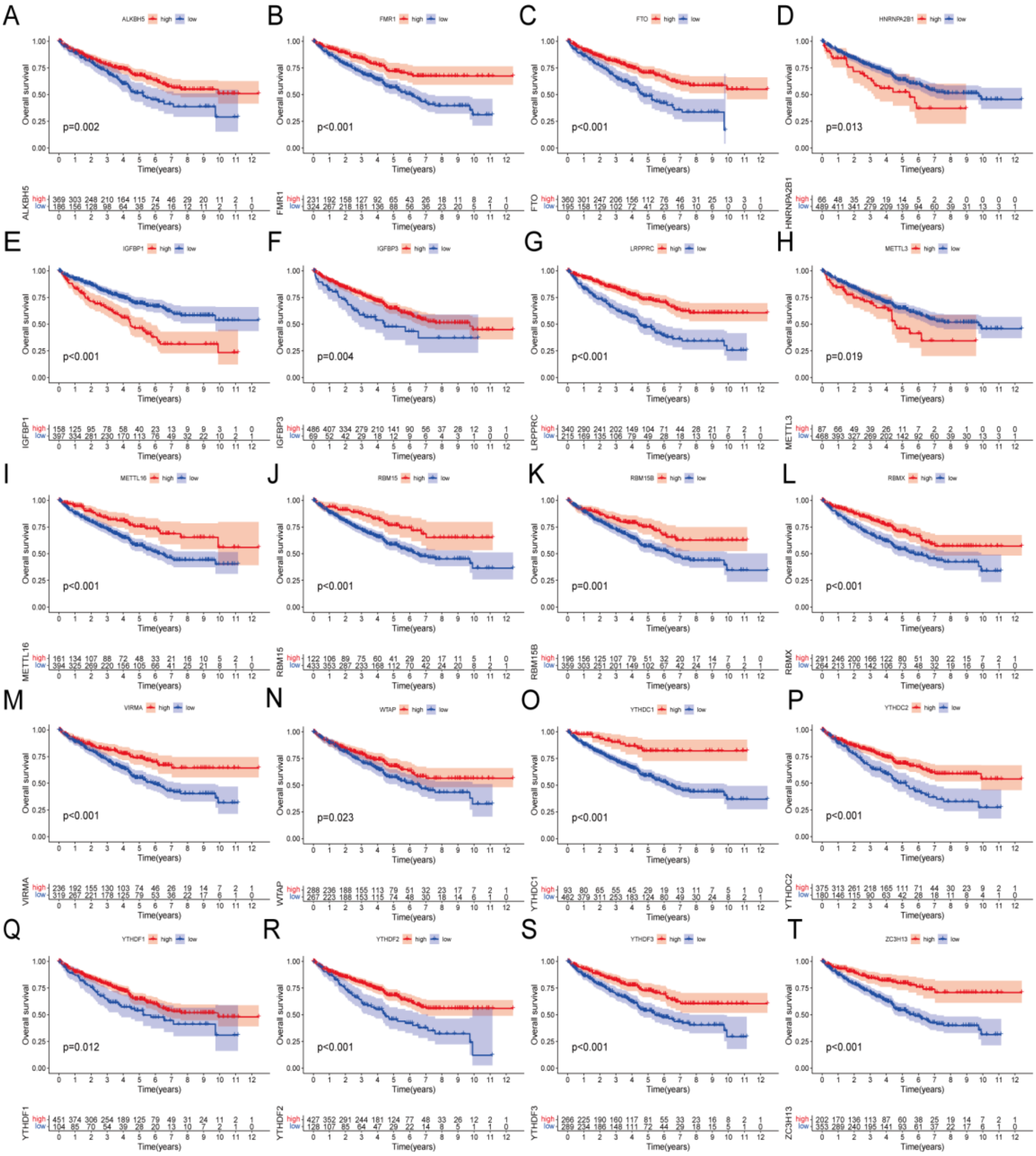
A
p=0.002
B
p<0.001
C
p<0.001
D
p=0.013
E
p<0.001
F
p=0.004
G
p<0.001
H
p=0.019
I
p<0.001
J
p<0.001
K
p=0.001
L
p<0.001
M
p<0.001
N
p=0.023
O
p<0.001
P
p<0.001
Q
p=0.012
R
p<0.001
S
p<0.001
T
p<0.001

Table

Table-S1: Basic information of datasets included in this study for identifying distinct m6A methylation modification patterns

"Accession number/Source"	Platform	"Number of patients"	Survival data
GEO: GSE29609	"Affymetrix Human Genome U133 Plus 2.0 Array"	39	OS
TCGA: KIRC	Illumina RNAseq	530	OS

Table-S2: Spearman correlation analysis of the 21 m6A modification

**regulators From**	**To**	**P value（<0.00001）**	**CorWeight**	
FTO	ALKBH5	0.682159463	3.98E-79	4.09295678
FTO	YTHDC1	7.09E-01	4.96E-88	4.253552975
FTO	YTHDC2	6.59E-01	4.28E-72	3.95267041
FTO	YTHDF1	5.07E-01	1.76E-38	3.041601167
FTO	YTHDF2	6.49E-01	2.10E-69	3.895417341
FTO	YTHDF3	5.58E-01	5.84E-48	3.350405466
FTO	FMR1	5.07E-01	1.99E-38	3.039734516
FTO	LRPPRC	6.33E-01	5.89E-65	3.796079544
FTO	HNRNPA2B1	4.41E-01	1.70E-28	2.646899231
FTO	IGFBP3	3.83E-01	2.79E-21	2.295871159

FTO	RBMX	6.43E-01	8.31E-68	3.860448827
FTO	METTL3	2.22E-01	9.02E-08	1.33068914
FTO	METTL16	6.00E-01	7.86E-57	3.597454868
FTO	WTAP	5.83E-01	4.50E-53	3.497057786
FTO	VIRMA	6.81E-01	7.87E-79	4.087297909
FTO	ZC3H13	6.85E-01	6.43E-80	4.107965297
FTO	RBM15	6.35E-01	1.09E-65	3.812863574
FTO	RBM15B	4.99E-01	3.90E-37	2.993161629
ALKBH5	YTHDC1	6.57E-01	1.71E-71	3.94006902
ALKBH5	YTHDC2	4.72E-01	6.50E-33	2.832150339
ALKBH5	YTHDF1	6.56E-01	3.34E-71	3.933911825

ALKBH5	YTHDF2	6.93E-01	1.81E-82	4.155331236
ALKBH5	YTHDF3	4.62E-01	2.01E-31	2.771710621
ALKBH5	FMR1	4.25E-01	2.16E-26	2.551440011
ALKBH5	LRPPRC	5.14E-01	1.10E-39	3.083848439
ALKBH5	HNRNPA2B1	5.22E-01	4.76E-41	3.130636998
ALKBH5	IGFBP2	1.65E-01	7.37E-05	0.992373114
ALKBH5	IGFBP3	3.41E-01	5.53E-17	2.04747364
ALKBH5	RBMX	5.81E-01	1.44E-52	3.48315148
ALKBH5	METTL3	3.49E-01	9.02E-18	2.095829985
ALKBH5	METTL16	6.47E-01	9.14E-69	3.881532664
ALKBH5	WTAP	5.03E-01	7.39E-38	3.019345571

ALKBH5	VIRMA	5.87E-01	5.59E-54	3.521807087
ALKBH5	ZC3H13	6.04E-01	8.11E-58	3.622861516
ALKBH5	RBM15	6.39E-01	1.64E-66	3.831503742
ALKBH5	RBM15B	6.25E-01	6.41E-63	3.748485615
YTHDC1	YTHDC2	6.86E-01	1.89E-80	4.117977662
YTHDC1	YTHDF1	6.55E-01	4.59E-71	3.930986359
YTHDC1	YTHDF2	6.92E-01	2.95E-82	4.151449917
YTHDC1	YTHDF3	6.22E-01	3.43E-62	3.731126092
YTHDC1	FMR1	6.87E-01	1.23E-80	4.121493196
YTHDC1	LRPPRC	6.59E-01	3.97E-72	3.95334847
YTHDC1	HNRNPA2B1	7.11E-01	1.24E-88	4.263785405

YTHDC1	IGFBP1	-2.60E-01	3.04E-10	1.559971438
YTHDC1	IGFBP3	2.72E-01	4.40E-11	1.629942535
YTHDC1	RBMX	7.85E-01	3.55E-120	4.71196275
YTHDC1	METTL3	5.62E-01	1.29E-48	3.369896379
YTHDC1	METTL16	7.57E-01	7.48E-107	4.541771278
YTHDC1	WTAP	6.23E-01	1.90E-62	3.737248279
YTHDC1	VIRMA	7.18E-01	1.97E-91	4.310422493
YTHDC1	ZC3H13	8.10E-01	2.64E-133	4.857659416
YTHDC1	RBM15	7.21E-01	2.99E-92	4.323778204
YTHDC1	RBM15B	6.15E-01	1.78E-60	3.689506291
YTHDC2	YTHDF1	5.13E-01	1.34E-39	3.080993583

YTHDC2	YTHDF2	5.96E-01	4.88E-56	3.57676268
YTHDC2	YTHDF3	6.10E-01	3.00E-59	3.659091904
YTHDC2	FMR1	6.77E-01	1.91E-77	4.060558366
YTHDC2	LRPPRC	5.88E-01	4.20E-54	3.525161471
YTHDC2	HNRNPA2B1	6.27E-01	1.49E-63	3.763419463
YTHDC2	IGFBP3	2.55E-01	6.99E-10	1.528722698
YTHDC2	RBMX	6.36E-01	9.82E-66	3.813909481
YTHDC2	METTL3	4.17E-01	2.74E-25	2.499324356
YTHDC2	METTL16	5.29E-01	2.33E-42	3.174453802
YTHDC2	WTAP	6.02E-01	1.69E-57	3.614666338
YTHDC2	VIRMA	6.21E-01	4.59E-62	3.728068907

YTHDC2	ZC3H13	6.69E-01	5.82E-75	4.011408386
YTHDC2	RBM15	6.23E-01	1.34E-62	3.740834055
YTHDC2	RBM15B	3.91E-01	3.36E-22	2.344685472
YTHDF1	YTHDF2	7.16E-01	1.83E-90	4.294477097
YTHDF1	YTHDF3	6.02E-01	2.75E-57	3.609247683
YTHDF1	FMR1	5.40E-01	2.43E-44	3.238712187
YTHDF1	LRPPRC	5.28E-01	4.27E-42	3.165719731
YTHDF1	HNRNPA2B1	6.73E-01	2.89E-76	4.037429247
YTHDF1	IGFBP2	1.89E-01	5.37E-06	1.136578491
YTHDF1	RBMX	5.61E-01	1.85E-48	3.365286806
YTHDF1	METTL3	4.09E-01	2.22E-24	2.455212107

YTHDF1	METTL16	6.67E-01	1.34E-74	4.004132402
YTHDF1	WTAP	5.72E-01	8.25E-51	3.433655581
YTHDF1	VIRMA	5.50E-01	2.47E-46	3.30108164
YTHDF1	ZC3H13	6.17E-01	4.47E-61	3.704173754
YTHDF1	RBM15	6.14E-01	3.72E-60	3.681605692
YTHDF1	RBM15B	7.06E-01	4.51E-87	4.237100073
YTHDF2	YTHDF3	7.12E-01	5.91E-89	4.269236766
YTHDF2	FMR1	5.61E-01	1.41E-48	3.368799565
YTHDF2	LRPPRC	6.23E-01	1.42E-62	3.740302477
YTHDF2	HNRNPA2B1	5.41E-01	1.35E-44	3.246781047
YTHDF2	IGFBP3	1.89E-01	5.82E-06	1.13241349

YTHDF2	RBMX	6.49E-01	1.81E-69	3.896830859
YTHDF2	METTL3	2.63E-01	1.94E-10	1.576502995
YTHDF2	METTL16	6.47E-01	6.72E-69	3.884450844
YTHDF2	WTAP	6.67E-01	2.26E-74	3.999509593
YTHDF2	VIRMA	7.09E-01	3.76E-88	4.255610632
YTHDF2	ZC3H13	0.709097602	4.32E-88	4.254585614
YTHDF2	RBM15	0.737560471	9.43E-99	4.425362823
YTHDF2	RBM15B	0.679569486	2.58E-78	4.077416917
YTHDF3	FMR1	0.72321558	3.27E-93	4.339293481
YTHDF3	LRPPRC	0.741330214	2.87E-100	4.447981286
YTHDF3	HNRNPA2B1	0.473536165	3.85E-33	2.841216988

YTHDF3	RBMX	0.652281611	2.98E-70	3.913689665
YTHDF3	METTL3	0.235812209	1.25E-08	1.414873255
YTHDF3	METTL16	0.56677168	1.16E-49	3.40063008
YTHDF3	WTAP	0.590150097	1.10E-54	3.540900584
YTHDF3	VIRMA	0.800517024	3.54E-128	4.803102147
YTHDF3	ZC3H13	0.71527244	2.72E-90	4.291634638
YTHDF3	RBM15	0.617366701	4.46E-61	3.704200205
YTHDF3	RBM15B	0.528886886	2.52E-42	3.173321318
FMR1	LRPPRC	0.672379863	4.16E-76	4.03427918
FMR1	HNRNPA2B1	0.557988679	7.06E-48	3.347932072
FMR1	RBMX	0.65497621	5.19E-71	3.929857261

FMR1	METTL3	0.427575396	1.08E-26	2.565452376
FMR1	METTL16	0.567759133	7.23E-50	3.406554797
FMR1	WTAP	0.539899821	2.31E-44	3.239398924
FMR1	VIRMA	0.688605844	3.50E-81	4.131635064
FMR1	ZC3H13	0.720122872	4.60E-92	4.320737231
FMR1	RBM15	0.540260898	1.97E-44	3.241565386
FMR1	RBM15B	0.505518723	3.05E-38	3.033112335
LRPPRC	HNRNPA2B1	0.38599961	1.17E-21	2.31599766
LRPPRC	RBMX	0.765415361	1.26E-110	4.592492164
LRPPRC	METTL3	0.242096642	4.93E-09	1.452579854
LRPPRC	METTL16	0.684360409	8.02E-80	4.106162454

LRPPRC	WTAP	0.527479485	4.53E-42	3.16487691
LRPPRC	VIRMA	0.762311506	3.20E-109	4.573869036
LRPPRC	ZC3H13	0.731009506	3.54E-96	4.386057039
LRPPRC	RBM15	0.572131916	8.85E-51	3.432791494
LRPPRC	RBM15B	0.539865147	2.34E-44	3.239190881
HNRNPA2B1	IGFBP3	0.172569881	3.50E-05	1.035419287
HNRNPA2B1	RBMX	0.622827255	1.95E-62	3.736963533
HNRNPA2B1	METTL3	0.675348092	5.19E-77	4.052088553
HNRNPA2B1	METTL16	0.550176645	2.47E-46	3.301059869
HNRNPA2B1	WTAP	0.591922269	4.38E-55	3.551533616
HNRNPA2B1	VIRMA	0.488378628	1.93E-35	2.93027177

HNRNPA2B1	ZC3H13	0.646758616	1.01E-68	3.880551693
HNRNPA2B1	RBM15	0.591808224	4.65E-55	3.550849346
HNRNPA2B1	RBM15B	0.573189882	5.30E-51	3.439139294
IGFBP1	IGFBP2	0.231483497	2.33E-08	1.388900981
IGFBP1	IGFBP3	0.246282601	2.62E-09	1.477695604
IGFBP1	RBMX	-0.179452152	1.66E-05	1.076712914
IGFBP1	METTL3	-0.186042391	7.93E-06	1.116254344
IGFBP1	METTL16	-0.198611558	1.80E-06	1.191669349
IGFBP1	ZC3H13	-0.180281698	1.52E-05	1.081690187
IGFBP2	VIRMA	0.175683489	2.51E-05	1.054100932
IGFBP2	RBM15B	0.36623831	1.68E-19	2.197429861

IGFBP3	RBMX	0.217391085	1.63E-07	1.304346507
IGFBP3	METTL16	0.164138811	8.37E-05	0.984832869
IGFBP3	WTAP	0.227891625	3.87E-08	1.36734975
IGFBP3	VIRMA	0.220527214	1.07E-07	1.323163282
IGFBP3	ZC3H13	0.176732793	2.24E-05	1.060396756
IGFBP3	RBM15	0.244773969	3.30E-09	1.468643813
RBMX	METTL3	0.542378964	7.84E-45	3.254273784
RBMX	METTL16	0.704713808	1.46E-86	4.22828285
RBMX	WTAP	0.66444474	9.67E-74	3.986668439
RBMX	VIRMA	0.753431191	2.54E-105	4.520587148
RBMX	ZC3H13	0.723124185	3.54E-93	4.338745108

RBMX	RBM15	0.624944202	5.71E-63	3.74966521
RBMX	RBM15B	0.564676052	3.12E-49	3.388056313
METTL3	METTL16	0.418536007	1.54E-25	2.511216039
METTL3	WTAP	0.344015203	2.98E-17	2.064091221
METTL3	VIRMA	0.289791067	1.80E-12	1.738746405
METTL3	ZC3H13	0.410666378	1.47E-24	2.463998271
METTL3	RBM15	0.427262533	1.18E-26	2.563575198
METTL3	RBM15B	0.394917382	1.12E-22	2.369504294
METTL16	WTAP	0.537376692	6.87E-44	3.22426015
METTL16	VIRMA	0.679753396	2.26E-78	4.078520375
METTL16	ZC3H13	0.739807663	1.18E-99	4.438845977

METTL16	RBM15	0.623274501	1.51E-62	3.739647008
METTL16	RBM15B	0.677706402	9.76E-78	4.066238414
WTAP	VIRMA	0.577336561	6.97E-52	3.464019369
WTAP	ZC3H13	0.582541942	5.24E-53	3.495251649
WTAP	RBM15	0.548384315	5.52E-46	3.290305887
WTAP	RBM15B	0.493697387	2.71E-36	2.962184324
VIRMA	ZC3H13	0.791008371	4.33E-123	4.746050225
VIRMA	RBM15	0.623699472	1.18E-62	3.742196832
VIRMA	RBM15B	0.621617901	3.93E-62	3.729707405
ZC3H13	RBM15	0.661394448	7.51E-73	3.968366687
ZC3H13	RBM15B	0.703194825	4.86E-86	4.219168947

RBM15 RBM15B 0.597315863 2.61E-56 3.583895176

Table-S3: Estimating relative abundance of tumor microenvironment cells in KIRC patients by the Single-Sample Gene-Set Enrichment Analysis (ssGSEA)

ID Activated.B.cell Activated.CD4.T.cell Activated.CD8.T.cell Activated.dendritic.cell CD56bright.natural.killer.cell CD56dim.natural.killer.cell Eosinophil Gamma.delta.T.cell Immature.B.cell Immature.dendritic.cell Macrophage Mast.cell MDSC Monocyte Natural.killer.cell Natural.killer.T.cell Neutrophil Plasmacytoid.dendritic.cell Regulatory.T.cell T.follicular.helper.cell Type.1.T.helper.cell Type.17.T.helper.cell Type.2.T.helper.cell

TCGA_TCGA-CZ-4859 0.185345235 0.397844277 0.568678868
0.563491113 0.637059662 0.716391605 0.304972583
0.625425554 0.379872867 0.705348919 0.332448044
0.400713714 0.648794705 0.755924759 0.745038257
0.49653793 0.255769987 0.744999816 0.511576989
0.514857258 0.470244041 0.377723424 0.419574339
TCGA_TCGA-CW-5587 0.365249711 0.536476728 0.776370458
0.646920923 0.660627012 0.713307303 0.264457135
0.637456003 0.575846467 0.730382462 0.475192872
0.530115767 0.860172085 0.763362139 0.821746803
0.551979938 0.274521787 0.769952218 0.734097848
0.652480549 0.562790053 0.4313219 0.436538565
TCGA_TCGA-BP-4332 0.18540966 0.365127472 0.544611543
0.569847082 0.63503712 0.725261136 0.258347601
0.641069917 0.414348382 0.728349515 0.408003536
0.421990156 0.700361385 0.755209265 0.777108798
0.530262834 0.191966585 0.779028586 0.586288631
0.567408659 0.51544878 0.328578004 0.439778986
TCGA_TCGA-CJ-4643 0.263429517 0.472057354 0.619235331
0.627361933 0.616450727 0.728960341 0.366702392
0.608150548 0.494559426 0.724841666 0.443833943
0.480415559 0.81344906 0.748193973 0.773481995

0.531346275 0.272332849 0.777557243 0.642824129
0.588305962 0.535911940.387229591 0.460335339
TCGA_TCGA-B0-4817 0.284666148 0.556724165 0.653892586
0.719441138 0.64512745 0.627870408 0.231565335
0.663010231 0.479007306 0.809661313 0.491365392
0.421579901 0.844284627 0.757083699 0.800939268
0.494517223 0.619889846 0.78189285 0.521280252
0.524588608 0.511858082 0.47413208 0.4149794
TCGA_TCGA-BP-4964 0.266091795 0.474845764 0.666186988
0.634308293 0.633223453 0.735358248 0.247136609
0.639894554 0.472027168 0.7040161 0.372184102 0.41250976
0.748636645 0.766042525 0.769315636 0.524256398
0.243810252 0.783042711 0.603687033 0.5894867 0.533294355
0.40248043 0.439864408
TCGA_TCGA-B0-5402 0.251993888 0.465772892 0.587257082
0.583143173 0.626685959 0.690571575 0.321393815
0.630776901 0.470432942 0.676881333 0.365613829
0.481580223 0.721467081 0.739370439 0.766291777
0.541538345 0.339604477 0.779238134 0.550708436 0.5575006
0.529354087 0.410929422 0.424081708
TCGA_TCGA-CJ-4891 0.292256193 0.590015921 0.652228435
0.731583954 0.677526148 0.767595128 0.251575279

0.727636552 0.453690404 0.781291277 0.57580614
0.502854583 0.889124558 0.798300167 0.797558297
0.567149142 0.373780231 0.79019968 0.750658428
0.616255648 0.559299057 0.415814378 0.37622248
TCGA_TCGA-B0-4690 0.366618697 0.692707432 0.764423622
0.676394076 0.63126627 0.681796715 0.200553534
0.670784953 0.549576709 0.756876116 0.52954026
0.513119774 0.920306269 0.728474346 0.808491115
0.577601004 0.198028901 0.765872139 0.782059828
0.641786012 0.613912757 0.370852747 0.526273042
TCGA_TCGA-B0-4815 0.615976535 0.709827733 0.835071423
0.648218632 0.649003236 0.719880279 0.205248991
0.690385038 0.660887038 0.710809832 0.516165769
0.441556110.908896027 0.766459364 0.826301663 0.579868062
0.197408693 0.786462984 0.732559337 0.678413795
0.599160552 0.397715334 0.427294056
TCGA_TCGA-B2-5635 0.276190999 0.507181796 0.658279222
0.675671517 0.599702571 0.689413717 0.262028366
0.583473682 0.55781391 0.741467951 0.434733973
0.452106884 0.815431312 0.722314812 0.832126141
0.495069604 0.154959312 0.779444033 0.722949804
0.618553411 0.553705212 0.456571941 0.457558469

TCGA_TCGA-A3-3328 0.080175342 0.324493434 0.4692265
0.496293416 0.64018459 0.644176289 0.351572517
0.548958216 0.296715954 0.610672286 0.229925831
0.358671558 0.499532591 0.763603451 0.591827973
0.362877461 0.220403068 0.731225961 0.322164675
0.47310841 0.399138203 0.424147466 0.345650679
TCGA_TCGA-CJ-4872 0.35093085 0.551693033 0.733869941
0.600033176 0.659776725 0.720214595 0.199788733
0.685328366 0.467093986 0.702395794 0.429607206
0.540469588 0.857086468 0.763543517 0.790337374
0.577859795 0.311061587 0.779832815 0.626110591
0.601993037 0.584802209 0.375385636 0.484524078
TCGA_TCGA-B8-A54K0.179831626 0.31696549 0.435885946
0.513343533 0.636567171 0.700304167 0.207328372
0.53564959 0.290848832 0.730553851 0.331570925
0.348858322 0.537559972 0.780471888 0.700068949
0.385798019 0.19197986 0.766088835 0.564832577
0.497565236 0.498810766 0.376967804 0.466020023
TCGA_TCGA-CJ-4873 0.376982608 0.542168404 0.805468351
0.680556055 0.66403829 0.772219893 0.182728742
0.668750802 0.534004352 0.748272677 0.517086483
0.49248674 0.937464254 0.7851434 0.77708631 0.534058513

0.141907164 0.778868674 0.669141845 0.674373544
0.596088656 0.411846064 0.379420841
TCGA_TCGA-G6-A8L70.25904999 0.618789036 0.724450089
0.578386689 0.65457934 0.765544734 0.18686143
0.668077054 0.300620782 0.662856767 0.392631301
0.369386862 0.722184592 0.753118308 0.667628987
0.550640944 0.252643833 0.753206793 0.503075288
0.505996171 0.490145065 0.417045006 0.486007415
TCGA_TCGA-CZ-5467 0.282825605 0.423477933 0.517117447
0.60705684 0.648597723 0.695347792 0.280188297
0.622717399 0.443825241 0.740372067 0.397871391
0.458859238 0.728303454 0.800202407 0.774838110.545577959
0.261673357 0.777829388 0.570927886 0.568884028
0.50509575 0.355270785 0.419964683
TCGA_TCGA-B2-5641 0.3576592 0.548885928 0.763892939
0.648532267 0.662203228 0.70406316 0.147118533
0.633377989 0.55667596 0.747081277 0.460951276
0.471341761 0.877520737 0.778694731 0.827277921
0.522822272 0.188807244 0.759464183 0.686600115
0.636550811 0.571855504 0.442727385 0.446397429
TCGA_TCGA-CJ-4882 0.520405441 0.697490601 0.809173648
0.657835087 0.643766502 0.752264053 0.320681276

0.640824467	0.515407303	0.696971951	0.479133676	
0.554147221	0.877741653	0.783006888	0.789764236	
0.551558443	0.124785571	0.754498237	0.654704121	
0.61598349	0.645339532	0.328842777	0.473221418	
TCGA_TCGA-B8-A54G	0.383382641	0.631069901	0.845117336	
0.705366239	0.660392361	0.677711256	0.311513251	
0.665495369	0.622737879	0.74381313	0.533809049	
0.526889485	0.948598491	0.783341254	0.819037417	
0.514446514	0.067727987	0.775802772	0.654101572	
0.673262947	0.598145064	0.44889748	0.409150327	
TCGA_TCGA-BP-4961	0.256868599	0.423114433	0.514204282	
0.572229174	0.630894736	0.72866608	0.244319366	
0.655144265	0.438667933	0.683666582	0.397174972	
0.470055959	0.694774746	0.789827115	0.772045487	
0.554873178	0.284715648	0.757859761	0.588916338	
0.534809083	0.514043609	0.370464693	0.44421532	
TCGA_TCGA-B0-5698	0.36063927	0.5607346	0.685361491	
0.671422551	0.633074812	0.715579447	0.369311140	0.653968512
0.52643426	0.745263392	0.507382708	0.489326547	
0.880622625	0.782391706	0.82233515	0.566406377	
0.347448003	0.773361569	0.656790807	0.613226319	
0.56238831	0.421495801	0.448532363		

TCGA_TCGA-BP-5199 0.380832146 0.600300061 0.830343437
0.642589424 0.631962461 0.747166442 0.284248306
0.660261685 0.552050068 0.699365035 0.468806324
0.471725157 0.834349194 0.776846893 0.776706648
0.573835437 0.189118491 0.767345072 0.695686047
0.629711733 0.58249374 0.360990956 0.477128897
TCGA_TCGA-CW-6093 0.200324568 0.45719174 0.541051738
0.532339625 0.632387177 0.743966815 0.417518996
0.631780944 0.442881371 0.688543623 0.348574388
0.540255928 0.70753383 0.778359444 0.766813618
0.554316457 0.349192978 0.767157244 0.621735664
0.549335456 0.531415195 0.413420823 0.457907302
TCGA_TCGA-B0-4700 0.467714145 0.677510755 0.859282938
0.642527222 0.650581718 0.774461469 0.284038849
0.736720066 0.532450149 0.665508498 0.511712141
0.486699193 0.911565936 0.774721298 0.808600307
0.593298998 0.247246789 0.758308779 0.724736169
0.653744638 0.62613507 0.386239062 0.503212265
TCGA_TCGA-AK-3460 0.266722368 0.464795526 0.613336631
0.635631759 0.631184185 0.727438585 0.293994429
0.64759968 0.428429884 0.707254756 0.399157415
0.51028314 0.763609631 0.759958393 0.762806472

0.532764964 0.298418075 0.770827219 0.610097375
0.567347589 0.523383436 0.372679519 0.456376933
TCGA_TCGA-AK-3458 0.282054167 0.549440134 0.73921535
0.622847139 0.648173795 0.729175691 0.209422259
0.62848806 0.398257322 0.729693632 0.347854979
0.43978996 0.728337704 0.739820196 0.746693279
0.478527371 0.262926865 0.734318431 0.490168702
0.551579509 0.510646471 0.419705461 0.381922977
TCGA_TCGA-CZ-5984 0.312139969 0.551462613 0.658118918
0.673819164 0.649531104 0.684040771 0.210223506
0.622271863 0.51373226 0.737031233 0.429353726
0.377488583 0.813260418 0.750419449 0.810163745
0.522670895 0.149864627 0.77041212 0.650437541
0.59653261 0.541778005 0.45897173 0.468410731
TCGA_TCGA-A3-3324 0.254672266 0.464016642 0.658390236
0.641954831 0.630203873 0.69040111 0.277744219 0.643255334
0.493158925 0.724936582 0.405518912 0.45546193
0.788608067 0.74419724 0.788546351 0.506941791
0.160209704 0.7786104 0.660141772 0.598525905 0.523781189
0.390750029 0.412444786
TCGA_TCGA-CJ-4912 0.278332709 0.394636551 0.601577879
0.579013111 0.57282854 0.730370643 0.368282542

0.668301636 0.394541697 0.695489933 0.396833244
0.417741038 0.755433314 0.741093747 0.696199799
0.496927027 0.298390164 0.768827706 0.502833503
0.549075077 0.482923152 0.439741153 0.433050882
TCGA_TCGA-CZ-5453 0.17362385 0.366533637 0.455493982
0.518459504 0.632791206 0.752465929 0.332977711
0.650977282 0.358457399 0.711741057 0.323354223
0.429295543 0.612728326 0.761984847 0.747403739
0.514035236 0.277863743 0.741663545 0.517818255
0.553802105 0.467457187 0.359177264 0.406057288
TCGA_TCGA-A3-3378 0.406697064 0.546320882 0.709190841
0.730352449 0.642093536 0.693867584 0.285655442
0.70141781 0.586898653 0.779336197 0.56183726
0.497312052 0.929890734 0.744465158 0.827493932
0.546770457 0.248302715 0.797693379 0.798839551
0.640814639 0.587537119 0.429664492 0.483009534
TCGA_TCGA-A3-3343 0.320021197 0.50145773 0.652427034
0.6275234 0.652469902 0.706710449 0.282823335 0.626198105
0.504488124 0.73965415 0.490987143 0.448325595
0.829027123 0.770767524 0.797736258 0.535916043
0.256225681 0.788178189 0.633231184 0.619976264
0.549655281 0.426315656 0.386168917

TCGA_TCGA-CJ-4887 0.326270098 0.589000166 0.816145709
0.673056498 0.669792671 0.738871312 0.314861936
0.675236979 0.558679595 0.716177438 0.501244051
0.490205087 0.882552417 0.775447805 0.816549974
0.528799732 0.187825953 0.771271318 0.664809073
0.638209793 0.56921934 0.412892365 0.422645975
TCGA_TCGA-DV-5574 0.424010042 0.579318406 0.728851731
0.648503325 0.676382622 0.696920348 0.345020579
0.697988953 0.554247489 0.750374868 0.565410792
0.600530184 0.86445518 0.771904374 0.786761333
0.599337192 0.221019218 0.76924146 0.689847232
0.658939424 0.600691571 0.354958257 0.498063933
TCGA_TCGA-CJ-4920 0.243443053 0.494723476 0.558388569
0.626387205 0.616014482 0.729554029 0.346903417
0.643498918 0.42087607 0.747307368 0.455193777
0.515619769 0.780060338 0.756067737 0.799738845
0.519026898 0.199078821 0.777706961 0.608829414
0.597142668 0.537825526 0.40938167 0.465758488
TCGA_TCGA-BP-5177 0.358848343 0.366425146 0.655197647
0.605313834 0.64571998 0.759809008 0.264728374
0.66290278 0.396700513 0.674407233 0.35907613
0.436342139 0.693521513 0.755743049 0.76678661

0.515963792 0.26855855 0.759739434 0.574396898
0.520839074 0.529161507 0.34646466 0.473610928
TCGA_TCGA-B0-4842 0.418066838 0.531836227 0.767696285
0.679237505 0.690588422 0.743997326 0.171224985
0.708627915 0.499225457 0.747234263 0.509023055
0.42414967 0.874615271 0.809724138 0.820453114
0.554551361 0.273411377 0.759234685 0.668897492
0.629848736 0.579363136 0.471275382 0.396917853
TCGA_TCGA-BP-4355 0.249771255 0.347369225 0.473949253
0.563165702 0.638811603 0.749802357 0.360914418
0.621021174 0.386980753 0.683427772 0.364210804
0.461591075 0.702910366 0.785036317 0.741220769
0.540022564 0.271533393 0.775375477 0.585170054
0.537780637 0.510041268 0.360078762 0.463376812
TCGA_TCGA-A3-3359 0.29655012 0.495797317 0.689154007
0.650598691 0.6292738 0.698954029 0.413669393 0.617967109
0.509601893 0.721053282 0.436514879 0.482758833
0.83180359 0.754298965 0.789775239 0.506317351
0.329400902 0.771814551 0.650602425 0.601731120.524703777
0.388084626 0.414211023
TCGA_TCGA-AK-3433 0.216531074 0.353058045 0.479205359
0.538250336 0.645547258 0.675094249 0.215382476

0.513395929 0.381552781 0.674224875 0.261980994
0.290892841 0.523726309 0.785978954 0.657128742
0.385724908 0.188578813 0.760804716 0.369389865
0.433347585 0.409527477 0.380162493 0.408521629
TCGA_TCGA-B0-4827 0.373512435 0.564850672 0.704906431
0.640021834 0.630308774 0.711120559 0.30945015
0.704516816 0.48973661 0.716781982 0.458154362
0.45793651 0.874479122 0.762226731 0.784810991
0.542685981 0.151583164 0.7625657 0.658160317 0.583970178
0.560079512 0.357371754 0.423388508
TCGA_TCGA-B2-A4SR 0.234098275 0.478864658 0.605431673
0.600688978 0.639435267 0.734431406 0.204419827
0.651798154 0.47236955 0.6837083 0.374722592 0.454744375
0.748305461 0.752943451 0.775853723 0.494399259
0.200431923 0.779126508 0.62721489 0.610802045
0.516788632 0.356153809 0.473403205
TCGA_TCGA-CJ-4874 0.268420348 0.543816905 0.763744997
0.632434153 0.669821607 0.716864364 0.27574137
0.648603154 0.530480254 0.717539372 0.397555093
0.481803526 0.804518529 0.774184358 0.798727586
0.529830964 0.271794628 0.761401805 0.620598495
0.594810658 0.546861466 0.406779492 0.457047653

TCGA_TCGA-B0-4699 0.195285398 0.639909914 0.768520929
0.639585118 0.641156722 0.723956115 0.296979869
0.626931434 0.491548144 0.779324827 0.483130852
0.415382134 0.832232236 0.74240781 0.758198329
0.512896874 0.091584718 0.766138741 0.703929904
0.592388156 0.496789189 0.427985292 0.473704042
TCGA_TCGA-B8-5550 0.207282844 0.541934541 0.581216646
0.633466925 0.638525616 0.737778484 0.145291076
0.676648731 0.468069668 0.700382355 0.420608828
0.430575355 0.777995659 0.766069203 0.734081217
0.534236694 0.158499844 0.753205597 0.659198215
0.56384249 0.573405988 0.409592488 0.411645094
TCGA_TCGA-A3-A8OW 0.273899964 0.43659918 0.64130029
0.602101629 0.637616251 0.741915565 0.32333429
0.627651562 0.439967107 0.648185575 0.356630764
0.46608629 0.730417773 0.773402568 0.756833757
0.507981934 0.274957892 0.760502551 0.536125141
0.567106649 0.513799309 0.394062247 0.454457065
TCGA_TCGA-B2-3923 0.158779564 0.340046971 0.456989694
0.559480667 0.636138529 0.665186024 0.370541431
0.500071349 0.453081421 0.658724268 0.277095168
0.399417528 0.504027719 0.728923559 0.597604879

0.379408545 0.217090243 0.724454011 0.430794089
0.494098713 0.421080631 0.391731013 0.499943375
TCGA_TCGA-BP-4967 0.295520316 0.438112030.562054566
0.578027577 0.607938527 0.7634192 0.227967076 0.653979042
0.442646914 0.71737356 0.385109983 0.416923466
0.696117571 0.75841761 0.764861329 0.521781313
0.115688950.771454769 0.609423803 0.58582695 0.501565746
0.401409589 0.436562555
TCGA_TCGA-BP-5201 0.372853516 0.567887332 0.795079664
0.644715232 0.630976727 0.743236408 0.298996479
0.639081449 0.486723349 0.689665301 0.385424196
0.391444185 0.787391683 0.757457505 0.797694537
0.51801466 0.201180137 0.776008436 0.605309791
0.602538583 0.556677822 0.410492507 0.432295168
TCGA_TCGA-AK-3440 0.130662795 0.417295736 0.440138035
0.53154085 0.639731807 0.742391964 0.336866387
0.562292663 0.306034446 0.730908036 0.235879008
0.358683366 0.507399708 0.773720338 0.607290877
0.422196729 0.208955369 0.729601708 0.393261913
0.471741015 0.405654321 0.433170287 0.380538218
TCGA_TCGA-A3-3365 0.335222581 0.488495612 0.625786697
0.603014948 0.638812965 0.718646252 0.479220871

1550		0.655302205	0.462116349	0.754598131	0.391715255
1551		0.602988698	0.720773183	0.738338252	0.743632668
1552		0.553875032	0.211505826	0.792230651	0.609398253
1553		0.602891603	0.547613836	0.34661075	0.508694447
1554	TCGA_TCGA-DV-5569	0.291268312	0.54395521	0.638988622	
1555		0.614133868	0.643758872	0.743305301	0.364182506
1556		0.648966265	0.486096578	0.738395045	0.43570191
1557		0.475381649	0.824038231	0.768954261	0.796797185
1558		0.575692779	0.223882199	0.762882769	0.61822796
1559		0.598361994	0.565110505	0.387587067	0.444296415
1560	TCGA_TCGA-BP-4974	0.2593445	0.467867054	0.56902488	
1561		0.603543254	0.617992027	0.744693856	0.388009073
1562		0.623099316	0.399868397	0.668772755	0.367046711
1563		0.445705179	0.686473728	0.77108178	0.737014428
1564		0.567039106	0.199439891	0.770132279	0.558803649
1565		0.556193585	0.523309373	0.361491269	0.477871896
1566	TCGA_TCGA-CW-5584	0.252540764	0.414142662	0.496342787	
1567		0.607594125	0.637507038	0.706617098	0.295720437
1568		0.603782723	0.464479746	0.724156301	0.422146249
1569		0.488112025	0.775010749	0.777090654	0.811412223
1570		0.518669915	0.264415473	0.768506592	0.633502779
1571		0.578810867	0.526231543	0.471930633	0.395707601

TCGA_TCGA-B4-5838 0.30053095 0.543785651 0.572911308
0.595483783 0.628506628 0.725415476 0.172120734
0.612493625 0.41356485 0.68564205 0.342775472
0.405737624 0.67487915 0.749192526 0.713358122
0.532778247 0.176157376 0.744226734 0.542630286
0.520932706 0.525368133 0.378197494 0.453149884
TCGA_TCGA-AK-3454 0.328281077 0.535441945 0.724107959
0.580857396 0.6417863 0.765609659 0.25693613 0.689898537
0.404746525 0.68324821 0.455169246 0.440656451
0.824625973 0.780418716 0.772480128 0.588107781
0.112751460.769427283 0.547326323 0.588446231 0.545556709
0.383223753 0.517635467
TCGA_TCGA-BP-4782 0.302393308 0.460717228 0.703354172
0.63913899 0.637812502 0.708490887 0.205016706
0.673334977 0.483039851 0.746647623 0.476574259
0.464465979 0.801880238 0.789546993 0.800446096
0.537321537 0.255309591 0.76230177 0.654285623
0.610204167 0.551505798 0.428758831 0.46279991
TCGA_TCGA-CZ-5451 0.271186886 0.473332163 0.657094766
0.612145085 0.644668237 0.721814705 0.289880366
0.660525479 0.442442244 0.697612135 0.391414347
0.481551933 0.740650518 0.774848527 0.76573185

	0.524159935	0.246282363	0.753200573	0.577594896
	0.577910276	0.509841967	0.408183018	0.432743265
TCGA_TCGA-CJ-4907	0.323109016	0.396747748	0.527837102	
	0.604590815	0.622430707	0.704122899	0.242951083
	0.621712408	0.44484446	0.728557256	0.396106953
	0.457927687	0.740799534	0.781888612	0.773621769
	0.533834342	0.232451225	0.772911628	0.643009575
	0.569022475	0.519606961	0.411026631	0.448092218
TCGA_TCGA-BP-5196	0.333316435	0.521586884	0.753177893	
	0.611058805	0.636760173	0.707402869	0.320223956
	0.592974872	0.470224443	0.701235066	0.418259458
	0.398976063	0.757134577	0.738946514	0.77633369
	0.536733809	0.104892986	0.779071653	0.571460259
	0.605967231	0.582667373	0.424847866	0.43178988
TCGA_TCGA-CJ-4889	0.691637239	0.512450406	0.74794792	
	0.638697759	0.650489978	0.704458868	0.386296337
	0.677701667	0.663088988	0.717197136	0.484462654
	0.551725125	0.832024177	0.761633572	0.78663253
	0.585101235	0.195211835	0.774059045	0.677690792
	0.627988466	0.609348773	0.347677432	0.43124257
TCGA_TCGA-AK-3428	0.212043654	0.541153945	0.527991822	
	0.647702902	0.654163145	0.695256164	0.244692167

0.655645072 0.433924469 0.759603889 0.439035699

0.506889971 0.781237231 0.794606425 0.764075606

0.543192294 0.471807252 0.781574709 0.62006658

0.575971368 0.527825702 0.382735758 0.459479371

TCGA_TCGA-A3-A8CQ 0.211227604 0.344183094 0.511860117

0.570304305 0.594590518 0.711219015 0.355996019

0.607437724 0.439889779 0.682455217 0.339930604

0.462738077 0.688823316 0.758687693 0.754893502

0.507315865 0.317066321 0.778419165 0.564938529

0.557639828 0.512559286 0.408291746 0.446143544

TCGA_TCGA-A3-3380 0.257884541 0.429611548 0.587882699

0.594372146 0.624924262 0.72456342 0.184402889

0.653511244 0.456912905 0.697387287 0.40646077

0.487384467 0.740032464 0.759674813 0.748571325

0.547339673 0.275983664 0.773827809 0.584799742

0.54904424 0.529774565 0.347195046 0.399431054

TCGA_TCGA-A3-3323 0.274113294 0.43505405 0.630544154

0.628986263 0.62804896 0.692361822 0.222070195

0.597944229 0.510709048 0.732368522 0.438151130.507834148

0.821686176 0.763000495 0.804152969 0.520045862

0.271778709 0.788594995 0.658640529 0.595972012

0.544226313 0.384672274 0.418215055

TCGA_TCGA-CJ-4895 0.25870942 0.555904688 0.67773584
0.67927602 0.653084773 0.748620461 0.284973542
0.663058469 0.509462608 0.790343389 0.494290345
0.471364041 0.860990918 0.749971594 0.829653246
0.545874749 0.196623214 0.772504363 0.692163336
0.594411791 0.573522334 0.366071301 0.423658446
TCGA_TCGA-CW-5591 0.232549767 0.323219442 0.428127114
0.586493373 0.627535766 0.72343898 0.203030847
0.565585396 0.345187092 0.739390836 0.389658414
0.402865966 0.588432895 0.784511020.746677346 0.507043845
0.182700478 0.726864161 0.543830836 0.520051205
0.507960063 0.445123635 0.419855053
TCGA_TCGA-BP-4799 0.301768578 0.603425315 0.759506582
0.624447124 0.665373102 0.772413722 0.460609299
0.719710885 0.497305935 0.715322884 0.488539776
0.511318228 0.815743199 0.785756566 0.786803328
0.625779933 0.291377896 0.769654173 0.623713663
0.61286293 0.607316889 0.386795242 0.510674675
TCGA_TCGA-B0-4847 0.419388604 0.597295826 0.840561152
0.675614036 0.628418128 0.717185159 0.346518024
0.609419946 0.516048543 0.707611679 0.462861121
0.493005871 0.924305296 0.777619507 0.783247628

0.506915733 0.244984067 0.764511460.622206745 0.628431515
0.556889297 0.483388288 0.38843387
TCGA_TCGA-CW-6087 0.469771529 0.759763253 0.86248255
0.706883572 0.644186157 0.694744356 0.254475075
0.744618153 0.698449826 0.730533852 0.611118808
0.567252434 0.951233521 0.76557758 0.822309707
0.610580166 0.146696694 0.755174097 0.818763674
0.712643962 0.647623637 0.501850284 0.563047172
TCGA_TCGA-BP-4173 0.485241643 0.686165793 0.867769952
0.671741043 0.64471312 0.697559405 0.431587095
0.639330092 0.694058977 0.742014929 0.555805187
0.580317246 0.965794572 0.766137349 0.810528467
0.594793209 0.188988118 0.766042721 0.773078379
0.70828414 0.648078382 0.465405296 0.507662159
TCGA_TCGA-B8-4619 0.315412773 0.369116469 0.454006858
0.525416561 0.635355499 0.659200715 0.305885649
0.567816926 0.332050991 0.740682282 0.273476482
0.363808918 0.553123443 0.781481868 0.663441415
0.38316505 0.169107771 0.756816995 0.411860935
0.477648782 0.436398022 0.40923262 0.383624671
TCGA_TCGA-BP-4176 0.324244543 0.647337688 0.669810442
0.660909374 0.656495269 0.690894988 0.388191056

1682		0.70605467	0.556910657	0.754671092	0.54496459	
1683		0.613710197	0.914147369	0.746637665	0.800934195	
1684		0.548775348	0.281627564	0.799466983	0.685923415	
1685		0.686363852	0.587873068	0.425167952	0.481009006	
1686	TCGA_TCGA-BP-4768	0.175767318	0.439604419	0.541066124		
1687		0.565700206	0.594807863	0.698524112	0.214958904	
1688		0.611747850.442125986	0.704982316	0.362790341	0.36104269	
1689		0.655398997	0.738457978	0.767172622	0.488340979	
1690		0.207613504	0.768737691	0.555052197	0.566165727	
1691		0.49031717	0.421002585	0.360719239		
1692	TCGA_TCGA-B8-A54I	0.54722841	0.584450808	0.725420709		
1693		0.691779229	0.642216016	0.734620482	0.207741986	
1694		0.715602241	0.572192518	0.750510124	0.555819607	
1695		0.502137844	0.913916347	0.757707993	0.773545097	
1696		0.544585459	0.179985232	0.796463811	0.688524616	
1697		0.611786665	0.570418406	0.408901066	0.440158207	
1698	TCGA_TCGA-CJ-4884	0.353947422	0.560989594	0.742452354		
1699		0.657681735	0.648306678	0.748544093	0.258112133	
1700		0.678589943	0.540948629	0.708797531	0.473368859	
1701		0.453386859	0.853730542	0.750045923	0.757405743	
1702		0.518340222	0.124230983	0.793645218	0.660207476	
1703		0.616066919	0.560477915	0.372675778	0.418085183	

TCGA_TCGA-BP-4164 0.208970724 0.452091954 0.534138166
0.62320333 0.637356757 0.727065868 0.285272042
0.570883428 0.446657798 0.698340901 0.358075479
0.386832868 0.67960618 0.719151548 0.750210493
0.49039534 0.219589874 0.777936289 0.59147834
0.553462303 0.470530772 0.447408742 0.380786247
TCGA_TCGA-CZ-5466 0.456632778 0.561945741 0.746218881
0.618333516 0.655813915 0.7064836 0.287140945 0.668781977
0.557320319 0.734833437 0.45387166 0.531710272
0.868506378 0.796489113 0.77815232 0.546935066
0.207522334 0.764961864 0.669763244 0.678104749
0.592670286 0.408437333 0.41034006
TCGA_TCGA-AK-3450 0.275478428 0.40996441 0.580912804
0.621173025 0.663661766 0.687697334 0.254453984
0.609017744 0.386962907 0.735307272 0.40565913
0.435986309 0.705055549 0.751044128 0.760294127
0.50308985 0.257193572 0.764590672 0.508882555
0.51853825 0.482912189 0.416039533 0.423486912
TCGA_TCGA-BP-4327 0.242113650.416079859 0.594529195
0.587859196 0.630859604 0.758640881 0.235456058
0.64070459 0.384044701 0.70734465 0.364018804
0.379662101 0.737062286 0.730957008 0.771378609

	0.519040832	0.187888896	0.784536697	0.591638879
	0.575094485	0.49893051	0.415099871	0.384067967
TCGA_TCGA-BP-5200	0.35232617	0.563370827	0.698641098	
	0.601345876	0.624434729	0.72607934	0.40182983
	0.650571177	0.503248384	0.71448313	0.428499876
	0.562986329	0.840955869	0.760856215	0.791573986
	0.546251759	0.204952012	0.771250308	0.679771471
	0.60499648	0.565690672	0.425424599	0.455063479
TCGA_TCGA-BP-4160	0.401094513	0.638190929	0.806148342	
	0.665427547	0.651267776	0.707203242	0.345095896
	0.673465336	0.573103028	0.748070156	0.473008583
	0.52000178	0.870206482	0.751261206	0.806142305
	0.57824028	0.162135847	0.752436289	0.704538251
	0.641520118	0.594304502	0.402167927	0.470958639
TCGA_TCGA-BP-5170	0.296503125	0.515735776	0.617072562	
	0.589809317	0.660974253	0.706212039	0.365428599
	0.680791109	0.486832112	0.706299468	0.469823112
	0.570451505	0.763278524	0.77107308	0.765674026
	0.547274026	0.229890937	0.763116192	0.633939168
	0.613710308	0.561148253	0.406375218	0.446764468
TCGA_TCGA-CJ-6027	0.367157391	0.698822652	0.85624969	
	0.627100506	0.65030552	0.695495694	0.177207110.721899013

1748		0.550520214	0.706878057	0.486407826	0.410206682
1749		0.880664318	0.741938635	0.773370009	0.57707285
1750		0.130845345	0.764274643	0.677806095	0.636771743
1751		0.588137718	0.429024572	0.477560053	
1752	TCGA_TCGA-BP-4983	0.474563546	0.743661179	0.827862704	
1753		0.680288718	0.606922004	0.701577693	0.184729623
1754		0.650591888	0.650269028	0.681683067	0.479425258
1755		0.454926572	0.938517429	0.736483791	0.783049174
1756		0.612129453	0.081833151	0.744402768	0.798084226
1757		0.635033905	0.59067403	0.383911937	0.472690794
1758	TCGA_TCGA-B4-5378	0.221731063	0.371356117	0.481038795	
1759		0.542523887	0.642310207	0.688293582	0.38134951
1760		0.608688809	0.386486024	0.729256616	0.347814283
1761		0.517514498	0.658217217	0.766303594	0.715022857
1762		0.464323327	0.303431843	0.751351909	0.614278953
1763		0.52494417	0.517843872	0.36877075	0.531177375
1764	TCGA_TCGA-A3-3317	0.310242245	0.538792886	0.595120031	
1765		0.64271478	0.587938918	0.726549552	0.241385506
1766		0.627657917	0.484635617	0.692379423	0.390623987
1767		0.46934009	0.754806816	0.751292018	0.766678661
1768		0.531066021	0.189003029	0.79231065	0.646623751
1769		0.580362798	0.544788797	0.339086446	0.417073231

TCGA_TCGA-BP-4771 0.459706001 0.633589919 0.874027761
0.665406613 0.651231082 0.737392986 0.238880578
0.662989449 0.615438517 0.71945506 0.49200091
0.511501322 0.916734377 0.756424993 0.832427487
0.559735134 0.136503797 0.765650616 0.711049092
0.638153948 0.626574822 0.399776995 0.428163008
TCGA_TCGA-A3-3352 0.256866991 0.492070924 0.616152138
0.635347765 0.655813032 0.723537618 0.249750547
0.630590886 0.489750526 0.716848125 0.379748097
0.44606356 0.764811690.763495959 0.772477002 0.521651229
0.253597417 0.798222778 0.606267674 0.582119789
0.544438001 0.402608504 0.378616533
TCGA_TCGA-B0-5084 0.240495698 0.55246731 0.561232748
0.662166017 0.615260139 0.681427002 0.165541332
0.64794123 0.360036351 0.689931651 0.411973652
0.353339935 0.724760329 0.756351561 0.724838853
0.533539319 0.179273977 0.737920032 0.523304971
0.510721099 0.481664864 0.431276634 0.417469276
TCGA_TCGA-BP-4159 0.239516547 0.486696674 0.658346664
0.606057295 0.658582997 0.756980596 0.303321324
0.667199333 0.456813089 0.716542062 0.407442502
0.482508835 0.776222067 0.793567544 0.783604182

1792 0.567085723 0.332326465 0.763163504 0.603365456

1793 0.573347556 0.554228001 0.389577063 0.476278459

1794 TCGA_TCGA-B8-A54J 0.289700368 0.489216754 0.706413135

1795 0.618835057 0.653341405 0.777341888 0.328567912

1796 0.63424402 0.441467171 0.691063787 0.380063875

1797 0.469613873 0.802823524 0.801050066 0.786845564

1798 0.536159752 0.336688078 0.771315361 0.605565444

1799 0.576558432 0.543101585 0.376227988 0.427275282

1800 TCGA_TCGA-CJ-4644 0.297763535 0.50933155 0.728042811

1801 0.649900002 0.660205895 0.718628768 0.219841895

1802 0.651240623 0.517021786 0.742956277 0.425204706

1803 0.487910049 0.834645396 0.783620716 0.769264259

1804 0.548554812 0.319547739 0.759855386 0.661687585

1805 0.599530807 0.538420766 0.414507485 0.444785211

1806 TCGA_TCGA-CZ-4863 0.318506167 0.557015382 0.773951066

1807 0.67396956 0.638326084 0.697369856 0.227053529

1808 0.642825437 0.591389434 0.699996621 0.417451692

1809 0.441266736 0.836543983 0.744859365 0.801217376

1810 0.539598322 0.222544391 0.759589835 0.684800796

1811 0.629240055 0.566673078 0.42748584 0.462624586

1812 TCGA_TCGA-BP-4330 0.486989671 0.524818247 0.791829676

1813 0.650078065 0.646512865 0.726643568 0.271680726

1814		0.617870706	0.590031548	0.717820975	0.403144695
1815		0.497857896	0.827501976	0.752424188	0.811609553
1816		0.536489666	0.213035036	0.773993412	0.691381236
1817		0.612157506	0.561519486	0.407151541	0.408110712
1818	TCGA_TCGA-BP-4995	0.296012689	0.447744395	0.606818442	
1819		0.597558454	0.665247019	0.71724834	0.400359765
1820		0.596147667	0.441679708	0.742393697	0.317031857
1821		0.544370128	0.708915525	0.735289094	0.713468188
1822		0.481552526	0.322938522	0.795006859	0.530674476
1823		0.540785887	0.499083304	0.364782779	0.400388125
1824	TCGA_TCGA-BP-4798	0.4912389	0.660019184	0.841032481	
1825		0.705775133	0.647966076	0.702275677	0.236602235
1826		0.618329515	0.609086716	0.690086843	0.559916575
1827		0.556247675	0.929516737	0.746107991	0.833760401
1828		0.552490756	0.159683108	0.769722942	0.726452774
1829		0.659841411	0.626124336	0.429179035	0.46339429
1830	TCGA_TCGA-B0-5700	0.266351893	0.498233781	0.610267365	
1831		0.576524542	0.634229161	0.749235173	0.316443672
1832		0.640506158	0.425754403	0.711204062	0.384377429
1833		0.48639801	0.758902721	0.792097138	0.739546932
1834		0.539848128	0.233578709	0.749051333	0.574483182
1835		0.570196602	0.54450974	0.39784986	0.429858129

TCGA_TCGA-AK-3456	0.240773282	0.391961117	0.486692456	
	0.641348867	0.662400587	0.669422931	0.282114408
	0.615926824	0.450383263	0.717149923	0.428013019
	0.424064522	0.70549263	0.822379123	0.722481102
	0.506555167	0.151456262	0.766719413	0.511071051
	0.580690334	0.479540089	0.389024395	0.334830507
TCGA_TCGA-CJ-4641	0.453690596	0.663132339	0.823825115	
	0.680544384	0.644257411	0.741812727	0.251736575
	0.664422886	0.598130072	0.716486479	0.502333487
	0.473192279	0.887119894	0.765627344	0.808988683
	0.554434002	0.129780692	0.759625633	0.681172878
	0.648736661	0.616119207	0.428280767	0.407232115
TCGA_TCGA-BP-4335	0.411442461	0.535130071	0.729954435	
	0.649260459	0.634245391	0.765738692	0.376243939
	0.62899946	0.509597133	0.757959734	0.412057931
	0.433445881	0.790908635	0.749390435	0.786520705
	0.544684776	0.25512381	0.787073573	0.616596886
	0.594863208	0.547505314	0.383211837	0.448298454
TCGA_TCGA-B8-4146	0.221774773	0.659861487	0.659931427	
	0.597935372	0.657764411	0.571001686	0.368513998
	0.667620094	0.583258618	0.683571692	0.386575553
	0.455561747	0.71099342	0.683990441	0.775268025

0.539466012 0.248253554 0.725093653 0.632966045
0.581180015 0.521127755 0.44501987 0.586194646
TCGA_TCGA-B0-4694 0.398524459 0.609507655 0.806639802
0.688389486 0.665573034 0.725539646 0.219369206
0.726918156 0.572803913 0.71245328 0.54965938
0.565904713 0.926530511 0.774673309 0.79968302
0.572094333 0.21479805 0.75641425 0.779963956
0.644870591 0.606805217 0.412928078 0.442337755
TCGA_TCGA-B0-4836 0.405880689 0.626674389 0.736772722
0.614960716 0.611912222 0.683635495 0.203680494
0.674553138 0.478558645 0.683240868 0.413831092
0.480179992 0.785354164 0.732400753 0.77400677
0.558613214 0.237395719 0.760694855 0.653204742
0.601941564 0.560432301 0.399994305 0.478667056
TCGA_TCGA-CJ-4635 0.320322804 0.521454843 0.792365995
0.631671106 0.627883479 0.71900195 0.169303753
0.639744438 0.518737349 0.722164662 0.451741192
0.442992903 0.785823354 0.720851180.80576041 0.530404912
0.157936911 0.788405735 0.622491486 0.634849814 0.5593585
0.395379481 0.424371612
TCGA_TCGA-BP-4975 0.299330279 0.410938972 0.525433008
0.590217246 0.627110811 0.711549759 0.269733069

1880		0.675492215	0.449514861	0.718441099	0.39701242
1881		0.480572118	0.750402004	0.777939674	0.772738892
1882		0.524262641	0.251919166	0.769099176	0.609375244
1883		0.593004348	0.520831624	0.388553193	0.440209229
1884	TCGA_TCGA-B8-A54E	0.231178615	0.412988398	0.5387256	
1885		0.584984145	0.637310031	0.660945419	0.26249769
1886		0.615726506	0.404496087	0.709293885	0.370459368
1887		0.299364282	0.603705338	0.765669272	0.601173613
1888		0.508697743	0.129528517	0.742015939	0.393436126
1889		0.495323326	0.420097689	0.358985194	0.361678743
1890	TCGA_TCGA-A3-3376	0.356321199	0.398467907	0.544267245	
1891		0.596134975	0.627367135	0.690092186	0.278370182
1892		0.634323677	0.497886472	0.707114572	0.452641314
1893		0.569846993	0.823777771	0.797297341	0.788598515
1894		0.566609129	0.253815424	0.774001458	0.665546023
1895		0.589059896	0.554735545	0.378593135	0.483982022
1896	TCGA_TCGA-BP-4758	0.410139301	0.510866589	0.809798611	
1897		0.619568658	0.670849093	0.755909424	0.376294286
1898		0.631851633	0.501109149	0.693436348	0.403401645
1899		0.518466191	0.797984935	0.786393482	0.79354358
1900		0.547297052	0.222261042	0.768721226	0.614431424
1901		0.619495488	0.560752945	0.398340199	0.44765507

TCGA_TCGA-B0-5121 0.253718255 0.451290902 0.718791541
0.622131486 0.660535563 0.768753479 0.302586868
0.645729546 0.450388197 0.709456642 0.431462997
0.525572385 0.82339889 0.804582971 0.792723478
0.539606622 0.355141403 0.777041938 0.576408153
0.572216443 0.551349766 0.386154392 0.452916477
TCGA_TCGA-BP-5180 0.322038155 0.551814011 0.728951356
0.59408449 0.633625163 0.670362276 0.289767297
0.618476015 0.506158586 0.719910695 0.451842046
0.427239387 0.811576952 0.748110367 0.766348664
0.510185473 0.271184556 0.765272336 0.614705014
0.625258273 0.522990449 0.427862268 0.415139967
TCGA_TCGA-DV-5566 0.235946349 0.524503188 0.552379074
0.611532453 0.639234964 0.756438891 0.270028688
0.628533313 0.413665363 0.702900769 0.396179201
0.451120284 0.722702401 0.767160708 0.782502393
0.516113166 0.231836816 0.742117145 0.537668308
0.557039282 0.519310856 0.450411281 0.426260088
TCGA_TCGA-BP-4784 0.310090709 0.424524231 0.457948994
0.579004509 0.681486743 0.698025753 0.316463791
0.656208731 0.391535917 0.707673038 0.430303096
0.475762565 0.618866092 0.780594576 0.708846962

0.476498097 0.242752709 0.768521972 0.568085897

0.520122378 0.521936081 0.395148712 0.532479671

TCGA_TCGA-A3-3331 0.240445092 0.517940617 0.615735158

0.623546684 0.635925099 0.684342739 0.337445803

0.632446977 0.516036387 0.720021263 0.432876762

0.565367422 0.797495806 0.742525628 0.792788062

0.535229219 0.247172419 0.784113687 0.653330353

0.610506001 0.547556026 0.406830371 0.453101776

TCGA_TCGA-CZ-5455 0.338829823 0.578935659 0.732027133

0.67273822 0.641804412 0.638015678 0.314675116

0.688276616 0.558889879 0.72143653 0.470159971

0.524866989 0.859347303 0.738853401 0.826127284

0.546178041 0.257578915 0.782500362 0.693576967

0.650238552 0.557279193 0.445149792 0.484446929

TCGA_TCGA-A3-3383 0.296451386 0.503747642 0.754047151

0.631262277 0.674616564 0.748273849 0.283136884

0.657024766 0.48903633 0.730146313 0.439260646

0.525431768 0.867993405 0.788869498 0.790727346

0.517803948 0.269066339 0.761791170.63679216 0.631753729

0.541365611 0.397681093 0.393856514

TCGA_TCGA-CJ-4875 0.245228965 0.469572244 0.807824699

0.619384832 0.637829162 0.736811744 0.220575565

0.634786876 0.456339431 0.707941577 0.370783632
0.40900567 0.812850244 0.755980922 0.764069117
0.515299397 0.232445433 0.767938409 0.583724577
0.586375048 0.529553559 0.452047555 0.416060096
TCGA_TCGA-B0-4811 0.272878103 0.483199716 0.60992399
0.614000895 0.635833559 0.775336802 0.230698133
0.589714784 0.374734302 0.720389314 0.317901003
0.409460059 0.753552367 0.74480968 0.74209047
0.533631182 0.236125662 0.755847708 0.511035432
0.540876598 0.521770931 0.41745848 0.396974273
TCGA_TCGA-T7-A92I 0.243985208 0.388418974 0.530057907
0.583545447 0.574560008 0.588853741 0.224451641
0.556237604 0.416457781 0.719712509 0.391899535
0.402056938 0.635173868 0.725498242 0.590206471
0.503029745 0.020953364 0.770746957 0.420413683
0.562259746 0.444830036 0.338485582 0.351140484
TCGA_TCGA-B0-5085 0.221119583 0.428245768 0.649281611
0.605819052 0.612548891 0.791147060.328737367 0.641957689
0.378999653 0.690939518 0.384706416 0.42787506
0.731338283 0.768966422 0.742557245 0.532727598
0.29447404 0.758874467 0.481744441 0.543022957
0.511910440.396543487 0.355993684

1968	TCGA_TCGA-CZ-4853	0.264769144	0.44543263	0.593615134	
1969		0.622834867	0.640799653	0.715413601	0.194614883
1970		0.60617356	0.448586119	0.697137759	0.360863175
1971		0.358412533	0.705169994	0.750604624	0.760854883
1972		0.500408122	0.175552325	0.771135585	0.573989578
1973		0.53930468	0.509602954	0.430111054	0.378929376
1974	TCGA_TCGA-A3-A6NI	0.229713209	0.401546082	0.53701378	
1975		0.603772002	0.612687438	0.709185084	0.186088665
1976		0.628208626	0.464067946	0.741732879	0.439354237
1977		0.419492899	0.796705451	0.76617481	0.782163105
1978		0.517564931	0.208060625	0.761998463	0.595789088
1979		0.596707229	0.50772794	0.451227417	0.425938529
1980	TCGA_TCGA-AK-3465	0.250532545	0.349468685	0.462615569	
1981		0.514119744	0.653389088	0.689367019	0.279657064
1982		0.535523701	0.353197633	0.670725362	0.296396543
1983		0.35135049	0.485364567	0.774290395	0.625334186
1984		0.42366938	0.083055103	0.741315011	0.358573686
1985		0.427619763	0.426549986	0.372043563	0.406705001
1986	TCGA_TCGA-B2-4101	0.367314264	0.541825889	0.736282228	
1987		0.639029662	0.635138987	0.717386691	0.244037619
1988		0.674255482	0.532548865	0.707365806	0.447641709
1989		0.490115535	0.853502241	0.760075374	0.811498327

1990		0.533459351	0.176417327	0.79221151	0.661697393	0.60971299
1991		0.565043167	0.421016812	0.436915197		
1992	TCGA_TCGA-BP-5185	0.275597424	0.468521736	0.719739034		
1993		0.581131057	0.625559228	0.781359163	0.286581879	
1994		0.633015438	0.387155642	0.667419722	0.356864328	
1995		0.464458186	0.714072136	0.776521823	0.730398398	
1996		0.504683969	0.195620738	0.761691602	0.479145886	
1997		0.534627346	0.508129811	0.408860517	0.3952435	
1998	TCGA_TCGA-AK-3427	0.175033997	0.349592562	0.473839197		
1999		0.540616834	0.655469529	0.710211406	0.257220263	
2000		0.602686716	0.333969231	0.703401058	0.25432463	
2001		0.375568782	0.498774425	0.758597987	0.568913504	
2002		0.374385617	0.203280271	0.701114359	0.397717365	
2003		0.486380416	0.403536093	0.445727976	0.394293694	
2004	TCGA_TCGA-BP-4998	0.371257443	0.504252405	0.677639073		
2005		0.668647075	0.635093732	0.748382484	0.307617224	
2006		0.631506889	0.507256869	0.751273816	0.492457267	
2007		0.522245073	0.852561043	0.741372034	0.828994921	
2008		0.55431602	0.20564692	0.772566341	0.686868185	
2009		0.640407951	0.5679647	0.389953463	0.443421309	
2010	TCGA_TCGA-BP-5202	0.243154606	0.433034705	0.557604508		
2011		0.607163757	0.620117401	0.730058601	0.322977495	

0.656247774 0.46914666 0.690048805 0.389128376
0.448723272 0.70538608 0.749402402 0.76489582
0.541111774 0.288105449 0.761803372 0.604320629
0.55079192 0.507992893 0.438784716 0.420895153
TCGA_TCGA-B8-5164 0.400793095 0.599369495 0.831009653
0.668912 0.656868672 0.644858413 0.383065842 0.66056322
0.630055157 0.73188689 0.506840636 0.512624012
0.896535433 0.75717698 0.819443841 0.554463737
0.195331104 0.768401056 0.715348352 0.683283865
0.582616357 0.48834472 0.450730345
TCGA_TCGA-DV-5573 0.377042393 0.593351643 0.81440177
0.662371422 0.659902543 0.682320192 0.314405261
0.646611751 0.524427507 0.767059964 0.437836929
0.490761617 0.828254753 0.770055344 0.803600059
0.537040431 0.09277932 0.761271052 0.621208093
0.643588135 0.579028912 0.429885312 0.452304221
TCGA_TCGA-BP-4989 0.467692066 0.636935316 0.86760715
0.702892025 0.643297399 0.700875769 0.273277887
0.673525505 0.607662216 0.712247465 0.485417166
0.465829157 0.929630423 0.74070102 0.756487083
0.564023476 0.199382351 0.792990806 0.730599099
0.690431294 0.580136878 0.408225982 0.450297014

TCGA_TCGA-G6-A8L80.213551056 0.347295739 0.591423475
0.571624495 0.607132358 0.746841229 0.245472438
0.621790634 0.340314813 0.698121546 0.311971706
0.36023191 0.693772128 0.780608987 0.728764733
0.479174303 0.241843065 0.783251503 0.459301446
0.545930608 0.469752796 0.422159246 0.367121527
TCGA_TCGA-BP-4162 0.354599814 0.581132621 0.753288065
0.663786528 0.652406663 0.732220287 0.358731437
0.670015377 0.56665022 0.703056651 0.413668247
0.56666818 0.832878584 0.729750111 0.789046004
0.564787693 0.299974627 0.765851262 0.686966356
0.624040352 0.56508476 0.384520841 0.470716082
TCGA_TCGA-BP-5173 0.31290489 0.523624992 0.745311931
0.574867673 0.667120436 0.713302381 0.259069797
0.649373387 0.522856743 0.691118126 0.383293087
0.495780428 0.762192832 0.739118807 0.768649674
0.51564294 0.207236633 0.747195749 0.617180032 0.5925214
0.531449764 0.377121104 0.48687115
TCGA_TCGA-B8-A8YJ 0.496909315 0.554973569 0.654484951
0.601999371 0.643785952 0.712264939 0.368127308
0.665398158 0.520703258 0.710425726 0.529005916
0.676159429 0.856498483 0.783318984 0.752488021

	0.606545849	0.290854163	0.770593597	0.687023801	
	0.604975017	0.610250097	0.334522289	0.466822118	
TCGA_TCGA-B0-5080	0.520837807	0.580353814	0.740196466		
	0.589233703	0.629709099	0.705282407	0.330493563	
	0.68079544	0.499326486	0.645424851	0.440742868	
	0.506769837	0.799607898	0.805797895	0.789725565	
	0.563455854	0.127509126	0.760777523	0.613045339	
	0.551010324	0.591383756	0.351363239	0.468811079	
TCGA_TCGA-BP-4976	0.244740379	0.395415714	0.486484508		
	0.581479992	0.602292108	0.709539909	0.220190994	
	0.615187117	0.448718081	0.696863345	0.337105278	
	0.420623385	0.674957483	0.771646992	0.754013754	
	0.519017019	0.23203568	0.75464859	0.604155433	
	0.548028938	0.498300815	0.429728519	0.438291007	
TCGA_TCGA-BP-4163	0.337420168	0.487221659	0.581256097		
	0.625925228	0.642035577	0.717053607	0.198258697	
	0.65203448	0.515448704	0.735211343	0.4933023	0.460001265
	0.829848913	0.747531736	0.801711372	0.520624354	
	0.156366793	0.756963059	0.666202073	0.595078113	
	0.54371643	0.402102838	0.358676121		
TCGA_TCGA-CJ-4899	0.322977569	0.470919434	0.539462985		
	0.583812083	0.628023535	0.706127683	0.327315778	

0.656371043 0.474984829 0.716844784 0.442387188

0.550271276 0.747466414 0.758821114 0.77487416

0.563964532 0.242727777 0.774252055 0.652080493

0.588812645 0.534955344 0.366252433 0.463895563

TCGA_TCGA-BP-4977 0.199616462 0.425500841 0.588771524

0.635895261 0.65104324 0.725961672 0.224261746

0.597212615 0.460759252 0.748232379 0.452781685

0.451239154 0.789996534 0.76657744 0.778193802

0.50184418 0.144007591 0.78296332 0.620177499

0.605549376 0.528100846 0.423810123 0.420571718

TCGA_TCGA-A3-3335 0.278494944 0.562773164 0.567458346

0.58496507 0.62646292 0.716209023 0.243579817

0.602983564 0.454382482 0.713711195 0.403862965

0.43056406 0.752796551 0.737799213 0.753308275

0.504649184 0.224081632 0.772692944 0.528569073

0.566014098 0.523654019 0.398402121 0.365378245

TCGA_TCGA-CJ-4634 0.202301377 0.34828705 0.502968874

0.595252352 0.607226996 0.700029918 0.263940119

0.613817543 0.421372019 0.6912463 0.380554643 0.498144166

0.73034746 0.740575976 0.784945343 0.514090548

0.305022385 0.772699664 0.581951296 0.582114813

0.511058681 0.400772365 0.395292352

TCGA_TCGA-A3-3325 0.323779061 0.478090807 0.546092448
0.586834858 0.626576929 0.743535819 0.33077188
0.626211643 0.483995651 0.735518883 0.410679623
0.483879827 0.806397489 0.741401203 0.791206088
0.539028365 0.234643295 0.768807431 0.620819569
0.602311528 0.541015148 0.386831373 0.436444516
TCGA_TCGA-CW-6090 0.362546873 0.509846158 0.696878729
0.629068029 0.673674726 0.689069221 0.297544265
0.696759096 0.526617641 0.758213445 0.54808995
0.630232647 0.861691140.775918686 0.79518357 0.57180148
0.183111398 0.805010355 0.714182279 0.659872381
0.578082204 0.374882294 0.478396492
TCGA_TCGA-BP-4177 0.274482077 0.378809414 0.468328078
0.55775532 0.65517099 0.675309746 0.341698683
0.576637431 0.352165192 0.733527193 0.366732815
0.442890278 0.585228808 0.769953585 0.716971786
0.487629706 0.199171005 0.749922725 0.542666257
0.523472269 0.528055307 0.390651038 0.513034045
TCGA_TCGA-BP-4170 0.306054928 0.54089819 0.722113215
0.622482193 0.645167605 0.731452028 0.352360732
0.64716378 0.527895506 0.72728 0.453121671 0.556877681
0.834703774 0.766790706 0.79985363 0.554570351

0.283887868 0.764507256 0.659371033 0.621031644
0.558823723 0.414197261 0.458617011
TCGA_TCGA-A3-3357 0.224453454 0.550742512 0.679004301
0.656599558 0.649730929 0.723568219 0.226026627
0.681032941 0.513605914 0.747956042 0.471601741
0.460856654 0.803733961 0.738152012 0.745935297
0.505623012 0.232278182 0.769148501 0.610312773
0.606294821 0.547295983 0.367663277 0.374105095
TCGA_TCGA-BP-4960 0.372231119 0.534015155 0.722191741
0.630600845 0.639979696 0.786085597 0.299784473
0.665848407 0.471276463 0.705549016 0.472820938
0.536821759 0.872895185 0.780278902 0.798463369
0.547896276 0.256065597 0.773983064 0.637267006
0.596952164 0.563590583 0.410385216 0.404307895
TCGA_TCGA-CJ-5677 0.270886779 0.511499048 0.623955014
0.639970728 0.646385432 0.745670776 0.24918647
0.669201024 0.48673297 0.697879386 0.526105123
0.453664975 0.851552169 0.789249096 0.815275444
0.539068442 0.19674333 0.770651589 0.653885517
0.596768427 0.55273235 0.408320646 0.39289654
TCGA_TCGA-BP-4167 0.428970605 0.608288569 0.845751595
0.651048359 0.697129788 0.720906189 0.270232436

0.687655585 0.550027035 0.740201402 0.580589475
0.579756144 0.944993626 0.773198164 0.799703169
0.60152647 0.228301099 0.770154841 0.720842429
0.679211848 0.613284277 0.42364902 0.476111208
TCGA_TCGA-CW-6097 0.565736693 0.707483074 0.752770508
0.699830195 0.638892064 0.669205038 0.264705293
0.656666549 0.637546823 0.698522747 0.47942241
0.53213196 0.866859304 0.754720014 0.776147751
0.606669614 0.131962252 0.763109244 0.741685017
0.637462212 0.644372558 0.361466334 0.464476628
TCGA_TCGA-B8-5162 0.579835136 0.687513054 0.812741347
0.689562363 0.654678815 0.70059572 0.364520961
0.720844351 0.683178065 0.736946007 0.593026336
0.611909685 0.959386252 0.796347543 0.816563206
0.624102504 0.221344785 0.783408458 0.789065573
0.705750647 0.635265544 0.413743096 0.515153033
TCGA_TCGA-CJ-6031 0.405698639 0.562472058 0.759729489
0.638580506 0.661712509 0.736624398 0.201299922
0.702866417 0.526437377 0.721274285 0.56628914
0.566571991 0.870814456 0.791056028 0.781083094
0.575402272 0.190653517 0.772074089 0.687678599
0.637799667 0.607631515 0.357812532 0.464640293

TCGA_TCGA-CJ-4639 0.289638298 0.504054616 0.780516701
0.585916869 0.657834536 0.691840962 0.325800628
0.624544759 0.509119744 0.691766072 0.342201142
0.435525271 0.751156384 0.773348611 0.758111951
0.521649465 0.216948213 0.778286555 0.628724549
0.582976535 0.53172702 0.36969499 0.450920648
TCGA_TCGA-BP-4759 0.227893308 0.442075008 0.68771616
0.601728463 0.648576244 0.747805409 0.195816811
0.67602166 0.421726968 0.711149575 0.374515327
0.462581069 0.733121108 0.791661023 0.757186251
0.549946729 0.179369379 0.760396784 0.564469404
0.549877312 0.528270943 0.393570504 0.443759638
TCGA_TCGA-BP-4986 0.33930969 0.516777673 0.571608881
0.647153781 0.617963588 0.690711429 0.338933295
0.622171997 0.491003552 0.747713043 0.474040197
0.47802418 0.775485928 0.737979483 0.766854191
0.535463843 0.174904215 0.77581492 0.658722554
0.626967337 0.550882249 0.401851052 0.501624026
TCGA_TCGA-B0-5075 0.257111488 0.520586602 0.676414622
0.692529515 0.634973237 0.731276405 0.316099541
0.682491932 0.551500993 0.743404183 0.523379401
0.499219982 0.905119638 0.757551557 0.750010214

0.513247956 0.092801945 0.780575823 0.664130354
0.647542658 0.552540316 0.417531398 0.378093991
TCGA_TCGA-BP-4775 0.35563698 0.594039951 0.587897255
0.625481591 0.59177663 0.714991422 0.327791756
0.636277581 0.480434412 0.704333112 0.363491014
0.50687647 0.756582048 0.72477909 0.75233637
0.501075303 0.258695134 0.7830898 0.603615426 0.565439163
0.518468 0.411509752 0.447185593
TCGA_TCGA-CJ-4888 0.509520905 0.728446588 0.85460262
0.666572042 0.654186389 0.695448688 0.236648474
0.736615178 0.620813188 0.732324344 0.538752 0.515009052
0.927067192 0.762237263 0.828353167 0.591509713
0.211170942 0.787816421 0.745293461 0.642162331
0.619679558 0.412207792 0.503645519
TCGA_TCGA-AK-3431 0.268290685 0.507065215 0.626615674
0.606872577 0.639529736 0.702792453 0.318701177
0.667746215 0.420290887 0.718159555 0.437080741
0.525689114 0.782247617 0.79888395 0.750052145
0.546108873 0.295280863 0.765225417 0.636001 0.573195688
0.575414729 0.359516498 0.504990382
TCGA_TCGA-BP-4338 0.280797535 0.535574111 0.579533459
0.682867622 0.670314656 0.755398595 0.111906959

0.733522364 0.470553778 0.699478916 0.486189527
0.451965526 0.807286524 0.708867246 0.693862411
0.537852412 0.192910284 0.729196983 0.626712518
0.599991394 0.524133014 0.419993378 0.41582834
TCGA_TCGA-B0-4701 0.379804883 0.593041267 0.713726115
0.690126286 0.639955687 0.758574166 0.232811237
0.625516548 0.496908621 0.714092734 0.46644636
0.467903177 0.841053041 0.739050462 0.785270807
0.534544454 0.271810565 0.768875593 0.619467405
0.610600635 0.561415731 0.416825365 0.420000335
TCGA_TCGA-B0-4718 0.295872319 0.477447052 0.705433979
0.616803546 0.663021882 0.761217502 0.368547868
0.631285159 0.438708443 0.7399714 0.394961669 0.509367938
0.772346294 0.764739411 0.799269786 0.522746479
0.229355575 0.787372951 0.561284456 0.588282316
0.510799444 0.39384752 0.433437209
TCGA_TCGA-B0-5696 0.235150863 0.444436727 0.618713997
0.612150645 0.66014923 0.775989589 0.240228139
0.642121259 0.416662638 0.709156412 0.388307637
0.430221567 0.717174828 0.768910629 0.753750115
0.526285203 0.240509385 0.761884461 0.562081002
0.561230456 0.502463145 0.384557966 0.44026716

TCGA_TCGA-BP-4326 0.35774072 0.509025744 0.530623708
0.645044543 0.616429712 0.719460566 0.321919964
0.631373144 0.421997961 0.678982528 0.435746022
0.537930784 0.772327657 0.776692924 0.73274457
0.506136576 0.245362486 0.783471911 0.598643624
0.545866179 0.559326796 0.33801381 0.540449497
TCGA_TCGA-CZ-4856 0.320340965 0.436483995 0.710196874
0.614789274 0.648670611 0.730049633 0.265076038
0.67029333 0.505264873 0.696994734 0.39191513
0.493244236 0.792878136 0.775462111 0.786052573
0.547097396 0.269195556 0.768562255 0.632023403
0.591193897 0.542764494 0.437687003 0.477046225
TCGA_TCGA-BP-4972 0.27041999 0.459669051 0.589926454
0.618938706 0.644654411 0.710004006 0.303868989
0.698801638 0.529375722 0.772469493 0.526011429
0.609322563 0.844418464 0.776343171 0.794502158
0.588273005 0.204681117 0.780144522 0.717961477
0.651970108 0.555717246 0.371859519 0.434428452
TCGA_TCGA-BP-5169 0.665429835 0.613807806 0.780630064
0.645381286 0.622809123 0.763614452 0.238094501
0.690919429 0.571098917 0.669775423 0.522073902
0.439859039 0.930627863 0.743991347 0.772654621

0.552315069 0.194835559 0.796493114 0.626949035
0.567970036 0.582982362 0.425544242 0.385612973
TCGA_TCGA-B2-5633 0.275047927 0.485876389 0.58105926
0.629875895 0.618370678 0.664434073 0.271582158
0.639893641 0.522354296 0.758424979 0.45564758
0.506371671 0.756855718 0.753859482 0.813940226
0.515931214 0.186328741 0.773774652 0.691493289
0.614345145 0.534799198 0.426475357 0.491041993
TCGA_TCGA-B0-5693 0.205428846 0.391419473 0.463425535
0.583910097 0.634446274 0.724827467 0.372835703
0.614400688 0.397102338 0.685923648 0.32791865
0.449067454 0.66653456 0.786122376 0.761689338 0.5262069
0.310792104 0.753499086 0.539703778 0.533693099
0.50162847 0.362911074 0.450795506
TCGA_TCGA-BP-4969 0.213423834 0.410195948 0.491984669
0.559423721 0.616403313 0.739204338 0.283919748
0.620740958 0.341314669 0.677573412 0.331406338
0.376880301 0.589726345 0.747831169 0.707009110.504614528
0.200132854 0.748415411 0.467937794 0.508956204
0.485115088 0.416088171 0.400170913
TCGA_TCGA-BP-4165 0.301100582 0.425579544 0.76173118
0.621233662 0.656458092 0.764621150.324485014 0.648993619

0.441362965 0.694167182 0.396117361 0.465003312
0.774427936 0.780029982 0.781970132 0.514091239
0.248426487 0.768899157 0.548806628 0.558673319
0.531547164 0.377545812 0.424118268
TCGA_TCGA-CJ-4870 0.240568263 0.379551054 0.563382781
0.579191613 0.655260228 0.754891184 0.312678093
0.681034575 0.411746496 0.712932761 0.426680613
0.528255769 0.756497828 0.799571424 0.747690613
0.516453816 0.356922742 0.761833046 0.578185615
0.572203763 0.521977095 0.370656078 0.425723699
TCGA_TCGA-B8-5552 0.391770864 0.522938947 0.634177604
0.620579959 0.633585478 0.690476561 0.273212828
0.655889346 0.527170682 0.721184805 0.481641887
0.551158913 0.812545762 0.774632199 0.820925538
0.566504677 0.262037009 0.773565243 0.707529825
0.626995293 0.573079313 0.393595993 0.468621545
TCGA_TCGA-BP-4760 0.260896902 0.384636599 0.402413067
0.581152471 0.631109427 0.687697802 0.370850458
0.611153976 0.35231377 0.732619031 0.404394809
0.619480031 0.651650823 0.772017082 0.742712903
0.502554603 0.21289626 0.764503764 0.662150093
0.530520614 0.530256033 0.386977784 0.519101813

TCGA_TCGA-B0-5113 0.388315061 0.50077591 0.648599225
0.629498818 0.636941886 0.675693952 0.3035769 0.609566363
0.541069757 0.716294627 0.398499663 0.486766513
0.756415911 0.729458127 0.791953262 0.510677711
0.180827679 0.791391855 0.665991572 0.608215282
0.548783829 0.413294073 0.441797132
TCGA_TCGA-BP-4340 0.23462986 0.463729055 0.530169041
0.614112358 0.621951774 0.732833598 0.323640549
0.602644678 0.428779949 0.711597490.318937545 0.358972131
0.649930716 0.715355492 0.765229356 0.503481563
0.201277232 0.763102092 0.492827531 0.530614066
0.485834773 0.377714565 0.394928968
TCGA_TCGA-B8-A7U6 0.343520907 0.573761181 0.593154265
0.626756373 0.611287280.765268243 0.38064793 0.636223817
0.434879262 0.702908417 0.397380309 0.595630452
0.772631519 0.776507078 0.792888478 0.54501481
0.292205237 0.760908695 0.605100167 0.56036215
0.544070787 0.374217117 0.452884195
TCGA_TCGA-B8-5551 0.532105571 0.719454163 0.809254327
0.725726128 0.6567536 0.74035444 0.21998317 0.715455472
0.676491834 0.728215261 0.59626559 0.498620841
0.986557207 0.773176149 0.827730135 0.570525039

0.111257298 0.768368611 0.860804925 0.693275485
0.652627901 0.437649215 0.463840545
TCGA_TCGA-B0-5400 0.343765282 0.547213198 0.755056148
0.691394484 0.687860225 0.751089951 0.257138278
0.704996696 0.433051461 0.763760463 0.491099141
0.464017482 0.839851607 0.773543518 0.776322008
0.584598018 0.334705059 0.767691316 0.615809494
0.590278758 0.555000801 0.397944146 0.419062624
TCGA_TCGA-BP-4994 0.284076083 0.419762177 0.547856026
0.583313452 0.659809762 0.71357942 0.414224807
0.571339881 0.414510529 0.735398743 0.288884571
0.517452029 0.661021366 0.730642333 0.681176298
0.453331150.30498433 0.787268804 0.48740856 0.526874286
0.476282452 0.38375557 0.395155233
TCGA_TCGA-B0-4824 0.269631827 0.515241366 0.785654839
0.645667442 0.646759359 0.727057638 0.309159504
0.634045732 0.530744429 0.70958616 0.443430952
0.450537039 0.834721238 0.743603263 0.793399732
0.525585199 0.212700765 0.773602041 0.642207788
0.63937019 0.548034123 0.405540093 0.450509185
TCGA_TCGA-CJ-4871 0.241911576 0.519565948 0.605005908
0.621832565 0.652692665 0.702132024 0.289172079

0.666100778 0.425558419 0.703426272 0.42376646
0.442430397 0.79356894 0.764620985 0.780060389
0.543695955 0.297996482 0.768309414 0.587511220.572211929
0.537374755 0.416487924 0.445684642
TCGA_TCGA-B0-5690 0.33984449 0.473331534 0.58551542
0.568481397 0.639217612 0.727262522 0.377887231
0.653066256 0.472053053 0.688690554 0.406122518
0.531598667 0.755727536 0.822704675 0.78887617
0.535061344 0.261733465 0.761244046 0.608604922
0.577319559 0.551758535 0.384112387 0.484225942
TCGA_TCGA-B8-5549 0.271761575 0.488825553 0.672101785
0.597167205 0.665893901 0.719097971 0.288688273
0.632038361 0.488144743 0.747037659 0.426578507
0.514298429 0.773479023 0.782352451 0.804881419
0.531219986 0.302565462 0.776811906 0.624936782
0.582137512 0.528499841 0.384073866 0.405607681
TCGA_TCGA-DV-A4W0 0.235720064 0.423299897 0.468643785
0.574394981 0.646724095 0.74137909 0.312040743
0.602007902 0.3364768 0.740744177 0.345513589 0.39412336
0.60884504 0.769335428 0.760066932 0.499694482
0.201935519 0.76691855 0.547410949 0.514220272
0.51435737 0.344133416 0.478028805

TCGA_TCGA-CZ-5989 0.240764726 0.627841366 0.673654506
0.600776661 0.637914551 0.561612657 0.439393044
0.646506136 0.622525819 0.682769537 0.419687948
0.495131119 0.685436636 0.706095145 0.802680323
0.537764308 0.295519259 0.713083716 0.615882908
0.618993515 0.534084733 0.447801029 0.616695562
TCGA_TCGA-B0-5081 0.523293827 0.684196743 0.848428187
0.667813392 0.665160029 0.73205162 0.31712081
0.631106280.605597447 0.679807025 0.511355599 0.505500558
0.931966412 0.784494697 0.808526401 0.563053757
0.170199408 0.777970176 0.720172361 0.646816591
0.618195447 0.423145124 0.499306282
TCGA_TCGA-A3-3373 0.289085785 0.4285786 0.527575786
0.626176839 0.62672761 0.734220004 0.334871643
0.66137365 0.502578051 0.717299663 0.431212207
0.487805106 0.739076435 0.744799446 0.772114687
0.548297721 0.253476484 0.764059664 0.607217707
0.564765206 0.511820559 0.438782524 0.427927794
TCGA_TCGA-CZ-4860 0.252568976 0.586968729 0.609568756
0.631011115 0.623775926 0.714131564 0.14601834
0.660654746 0.518656576 0.682349373 0.39387452
0.366209393 0.813715034 0.751197372 0.71002532

2386	0.558936145	0.040289254	0.771266711	0.733632544
2387	0.566741598	0.560257649	0.332783109	0.419848201
2388	TCGA_TCGA-AK-3451	0.304207186	0.562743638	0.772651185
2389	0.636977608	0.669287063	0.733863051	0.175247232
2390	0.719116797	0.43810605	0.718383935	0.441827637
2391	0.474294866	0.785519091	0.799333035	0.715432776
2392	0.525966172	0.190476335	0.744000015	0.562753297
2393	0.596471112	0.554668272	0.422402353	0.404590034
2394	TCGA_TCGA-B0-5104	0.246778681	0.47169083	0.503930718
2395	0.616190689	0.639347105	0.746836035	0.248047274
2396	0.578351056	0.431430823	0.722891418	0.403632698
2397	0.420652805	0.778091167	0.782512956	0.791616549
2398	0.514905178	0.318032508	0.762842586	0.558938903
2399	0.579900131	0.530384262	0.445756389	0.376292766
2400	TCGA_TCGA-BP-4351	0.221072265	0.490614042	0.628142222
2401	0.606676823	0.582918164	0.749949087	0.241675125
2402	0.59029245	0.389506058	0.720034547	0.366506311
2403	0.362990933	0.714301035	0.757486635	0.755596389
2404	0.525662002	0.234728787	0.769919703	0.539428611
2405	0.548991512	0.491681819	0.360935	0.437231097
2406	TCGA_TCGA-B2-4099	0.257987681	0.540594959	0.69403637
2407	0.664009034	0.664965575	0.723642232	0.353908343

2408		0.667817474	0.523973265	0.738778181	0.463910633
2409		0.541359676	0.836331035	0.775635004	0.784661278
2410		0.543107062	0.226146342	0.774715179	0.656739563
2411		0.638731416	0.551225596	0.403385465	0.405657491
2412	TCGA_TCGA-B0-5694	0.251899389	0.405579602	0.594234407	
2413		0.600069899	0.602608075	0.719363695	0.347185002
2414		0.627047024	0.467483277	0.747906166	0.371520903
2415		0.456846448	0.780269474	0.787965463	0.794619635
2416		0.546009012	0.231574642	0.773089494	0.636475591
2417		0.594279237	0.545493347	0.402140931	0.430601991
2418	TCGA_TCGA-CJ-4903	0.342597033	0.523724367	0.720780408	
2419		0.599630846	0.647007773	0.698086963	0.325392778
2420		0.623220863	0.513561742	0.722982792	0.407042811
2421		0.459234934	0.823152167	0.74318149	0.79823677
2422		0.526450185	0.227489255	0.775285473	0.620411435
2423		0.634969219	0.553207141	0.411015135	0.404614798
2424	TCGA_TCGA-CZ-5457	0.314221321	0.50521125	0.637948593	
2425		0.608325423	0.651893004	0.713859377	0.151468433
2426		0.666300648	0.502118649	0.707119584	0.442787027
2427		0.539644646	0.769188553	0.765531625	0.785273567
2428		0.539268588	0.219864512	0.756073491	0.616413275
2429		0.618610985	0.539728155	0.402177009	0.421596782

TCGA_TCGA-BP-4158 0.197286877 0.393500159 0.590847518

0.60247231 0.649826109 0.748397809 0.236052315

0.62675034 0.419155125 0.709251151 0.316452919

0.433912939 0.692171028 0.758674904 0.768437731

0.491221369 0.243981736 0.784438827 0.585427865

0.544855065 0.505666683 0.348446492 0.440334752

TCGA_TCGA-A3-3320 0.204892766 0.45397282 0.530997768

0.594206492 0.617755641 0.734360769 0.373733713

0.607907498 0.469583042 0.714072901 0.363667881

0.503719612 0.760356289 0.716486088 0.770438013

0.510972611 0.283964962 0.772431981 0.629048404

0.576226374 0.51029519 0.368926877 0.426545035

TCGA_TCGA-DV-5568 0.247276496 0.550190748 0.748563682

0.625043124 0.651109781 0.735445647 0.239994278

0.673736532 0.443179342 0.712639914 0.458398425

0.451166173 0.815469983 0.772219793 0.766529512

0.568529354 0.191728883 0.759179256 0.596092351

0.599730342 0.559161065 0.380301407 0.422855792

TCGA_TCGA-CZ-5985 0.35110303 0.561260044 0.678144087

0.664662623 0.649383569 0.648340072 0.249624474

0.674015494 0.543086085 0.753858482 0.464990766

0.486059605 0.854633267 0.756612538 0.812607548

2452	0.523646563	0.252941888	0.767666751	0.69767164
2453	0.626368507	0.54847677	0.428476379	0.400911426
2454	TCGA_TCGA-BP-4166	0.356778454	0.468674823	0.680195952
2455	0.60643705	0.63271372	0.759361333	0.250516598
2456	0.705738147	0.449660998	0.702224873	0.416906208
2457	0.483526886	0.756771951	0.785383798	0.740072079
2458	0.546311287	0.177453072	0.774880048	0.637431406
2459	0.579679017	0.532724993	0.350182898	0.459602338
2460	TCGA_TCGA-AS-3777	0.228440128	0.453667368	0.525613093
2461	0.572817296	0.691750906	0.757534478	0.226729863
2462	0.652312395	0.331511467	0.736815833	0.370240875
2463	0.391798041	0.635634712	0.838348575	0.694359374
2464	0.453903872	0.316236646	0.701172404	0.469810668
2465	0.460366014	0.502009902	0.436994006	0.441308813
2466	TCGA_TCGA-CZ-5456	0.36715628	0.598906502	0.683491564
2467	0.653845889	0.613383069	0.636912547	0.278980659
2468	0.641175885	0.522956353	0.687948013	0.481609795
2469	0.474962333	0.801470066	0.752077817	0.795329186
2470	0.54275605	0.15778609	0.757281867	0.649259463
2471	0.641059211	0.55774628	0.439523469	0.426511088
2472	TCGA_TCGA-BP-5183	0.342189057	0.487935555	0.727331166
2473	0.632855945	0.623885359	0.68247838	0.207246276

2474		0.622898172	0.527151267	0.724663673	0.408812764
2475		0.368237244	0.783035659	0.756073736	0.803051076
2476		0.512307812	0.206864119	0.780021533	0.632784058
2477		0.604581374	0.53081104	0.411457286	0.370570254
2478	TCGA_TCGA-CZ-4854	0.341541171	0.514272404	0.636520409	
2479		0.619320175	0.615170325	0.74641055	0.144278165
2480		0.634436864	0.45548893	0.704212715	0.392334209
2481		0.380202354	0.725902396	0.720363577	0.774334122
2482		0.523461149	0.050508563	0.784389282	0.591994299
2483		0.605557331	0.518840822	0.411155394	0.456720436
2484	TCGA_TCGA-A3-3306	0.248258378	0.557294577	0.601664309	
2485		0.632225225	0.593981873	0.692678424	0.272529757
2486		0.618364169	0.462540446	0.700598504	0.36208503
2487		0.424616733	0.729297087	0.737122683	0.780574987
2488		0.496760564	0.222261212	0.770082995	0.557469692
2489		0.552805153	0.523885287	0.463014988	0.370545644
2490	TCGA_TCGA-BP-4789	0.198148432	0.383661735	0.523759952	
2491		0.581831492	0.639380644	0.716625711	0.319058763
2492		0.632934218	0.395081955	0.667218418	0.328310775
2493		0.524556658	0.624663683	0.78183958	0.748914459
2494		0.551197518	0.21827471	0.746164335	0.584308668
2495		0.534439236	0.521234971	0.378366009	0.485477398

TCGA_TCGA-CZ-5462 0.305761067 0.594060271 0.639676032
0.672255285 0.63884105 0.600818827 0.288365001
0.655067334 0.542116713 0.736551574 0.456429698
0.474934765 0.841934724 0.732619305 0.778076978
0.540145404 0.259697659 0.764269535 0.597083691
0.59201005 0.52459172 0.473272619 0.421675101
TCGA_TCGA-B0-5705 0.325284978 0.4675459 0.649831766
0.586297161 0.637327671 0.71388121 0.280000893
0.62272281 0.499714562 0.684862363 0.366325319
0.427427133 0.745416365 0.77422679 0.74913186
0.518440107 0.266995533 0.756480062 0.610346289
0.586047151 0.528257915 0.427083811 0.425651325
TCGA_TCGA-B0-4710 0.331808365 0.524799151 0.652653294
0.596998063 0.607951794 0.692435877 0.35904221
0.610894598 0.4655368 0.706114147 0.400230708 0.545205167
0.771748977 0.759836085 0.771370936 0.514809503
0.353101453 0.795530846 0.554051181 0.561514058
0.515419208 0.353652842 0.422503031
TCGA_TCGA-BP-5004 0.204736469 0.506344593 0.53587553
0.600724324 0.613742517 0.707097925 0.299589333
0.615149663 0.467456735 0.697648065 0.422704698
0.453226779 0.703863331 0.715506609 0.761436065

2518	0.540498297	0.208996759	0.791595958	0.593294006
2519	0.556075571	0.513482284	0.381180404	0.492159587
2520	TCGA_TCGA-A3-3387	0.346253718	0.531272736	0.559879288
2521	0.700770022	0.650364001	0.684809179	0.227791535
2522	0.67845244	0.49888531	0.738076003	0.497710256
2523	0.496352641	0.850493877	0.747898614	0.805043665
2524	0.545297323	0.188074692	0.768377911	0.680822301
2525	0.633968768	0.570149399	0.431632184	0.405762998
2526	TCGA_TCGA-BP-5175	0.196160239	0.482168379	0.772488854
2527	0.643426144	0.674574074	0.79422125	0.192024375
2528	0.675983969	0.432959076	0.724658869	0.486462799
2529	0.383251373	0.843795693	0.745760616	0.785011482
2530	0.509764532	0.167551336	0.765259924	0.585771683
2531	0.573846964	0.517211376	0.444897663	0.440201414
2532	TCGA_TCGA-BP-4331	0.232740612	0.435383616	0.623326575
2533	0.659462146	0.617059502	0.716740078	0.262434422
2534	0.645404316	0.500828399	0.743055508	0.423713652
2535	0.476771613	0.839573446	0.752578955	0.79532519
2536	0.517673814	0.204068538	0.771462849	0.655162569
2537	0.609562863	0.537066649	0.42763053	0.413011847
2538	TCGA_TCGA-BP-4174	0.231869019	0.506688277	0.663603073
2539	0.666212696	0.667547718	0.639139461	0.242666295

0.657473279 0.517673187 0.733300729 0.449580733
0.542892112 0.819045457 0.730639558 0.725044588
0.515330905 0.261110544 0.776169694 0.644384568
0.61295304 0.522753794 0.394370896 0.436684941
TCGA_TCGA-CJ-6028 0.425306957 0.699327804 0.794233029
0.665295897 0.652225595 0.656298432 0.268111785
0.70133548 0.617306323 0.740568074 0.501014951
0.486107358 0.841727778 0.746995548 0.823511475
0.559745694 0.209361599 0.793909456 0.690654552
0.646136587 0.57306855 0.389812289 0.522577944
TCGA_TCGA-A3-3367 0.255549132 0.464407659 0.549468494
0.626451556 0.639785071 0.701427126 0.248579079
0.606845594 0.4922122 0.726796766 0.36781606 0.447388233
0.779549788 0.746120637 0.786213937 0.50972405
0.231189854 0.780244216 0.612822509 0.602997994
0.524543328 0.426654458 0.408108396
TCGA_TCGA-A3-3351 0.342271632 0.533124116 0.727238611
0.666113913 0.634465776 0.673426698 0.299595654
0.572778609 0.577762509 0.742865133 0.481077368
0.510051553 0.858694835 0.748269647 0.8476535 0.515811088
0.139362455 0.781109842 0.70494524 0.644195861
0.572729849 0.427054436 0.438797762

TCGA_TCGA-CJ-4890 0.511870399 0.699139514 0.831721679
0.683578007 0.671000365 0.685913164 0.307928952
0.708600519 0.614547529 0.762815088 0.586298472
0.621705922 0.957031306 0.788370844 0.816559959
0.605190943 0.180744298 0.791155183 0.780767365 0.7002258
0.650085383 0.394404267 0.512843031
TCGA_TCGA-CW-5581 0.209893363 0.494082622 0.616647369
0.601155260.641579132 0.719741205 0.208262673 0.648881324
0.442981871 0.708110926 0.347406574 0.372388676
0.672821188 0.767082858 0.75149781 0.5128987 0.196776036
0.752166012 0.54570475 0.543921794 0.499979005
0.370164031 0.383582114
TCGA_TCGA-B8-4153 0.220765262 0.428622321 0.6125893
0.591442254 0.658725203 0.748462633 0.374084572
0.635793213 0.385242684 0.730610723 0.366931325
0.478934881 0.710004027 0.80138187 0.787218081
0.533398598 0.241862102 0.780524303 0.537604231
0.575127018 0.521292455 0.409122776 0.41408317
TCGA_TCGA-B0-4698 0.352773901 0.778904110.859507727
0.658072486 0.634777678 0.634276919 0.191698193
0.686324594 0.534023411 0.723970049 0.501802263
0.367146769 0.884680372 0.739479316 0.716975054

	0.577709256	0.259661369	0.743161278	0.691518131
	0.583969844	0.575488583	0.377225459	0.54352115
TCGA_TCGA-B0-5106	0.335796521	0.4593598	0.710198682	
	0.646311348	0.647126917	0.704576921	0.302673045
	0.609131033	0.513341959	0.75290443	0.488440312
	0.501023421	0.841662714	0.788867276	0.785397103
	0.530722653	0.199530062	0.791508281	0.679403128
	0.620538646	0.546295111	0.41663273	0.428575183
TCGA_TCGA-BP-4329	0.264926695	0.481272593	0.632907539	
	0.622171162	0.669576725	0.716187124	0.334995491
	0.654812468	0.473518096	0.743723287	0.433890449
	0.534479135	0.802252422	0.756153226	0.78688136
	0.559817252	0.279897525	0.778727663	0.616926866
	0.599518991	0.548661263	0.39024542	0.454397953
TCGA_TCGA-B0-4712	0.378104394	0.644581078	0.757306558	
	0.644804418	0.642429308	0.748044398	0.194734141
	0.69580299	0.496310651	0.671084593	0.502945887
	0.413234004	0.872122968	0.759014591	0.731903672
	0.582513407	0.235062673	0.763809981	0.700133085
	0.63129039	0.596462402	0.444116296	0.48489851
TCGA_TCGA-B0-4691	0.421510026	0.590858971	0.824852903	
	0.629688337	0.641172366	0.781659307	0.283381117

2606		0.618396593	0.483300439	0.663737541	0.388558097
2607		0.389919648	0.799384317	0.757918606	0.759851803
2608		0.547175211	0.087065324	0.749573454	0.571589872
2609		0.595740843	0.601400587	0.420655234	0.422225672
2610	TCGA_TCGA-CZ-5982	0.233405828	0.436643424	0.506652085	
2611		0.611449697	0.612482078	0.737250661	0.215921342
2612		0.602814415	0.417843555	0.67919625	0.359509432
2613		0.457249763	0.704936546	0.760819065	0.786298752
2614		0.524379802	0.368925026	0.757571913	0.583256836
2615		0.562178493	0.511938796	0.398019715	0.426882924
2616	TCGA_TCGA-BP-4962	0.406946456	0.584852511	0.761669352	
2617		0.636448325	0.639407451	0.734514027	0.216140684
2618		0.665519637	0.51541259	0.709775028	0.485637819
2619		0.505597194	0.871026163	0.77956703	0.765049561
2620		0.530401082	0.105768503	0.741861581	0.651691892
2621		0.609524566	0.586135042	0.430188015	0.392462226
2622	TCGA_TCGA-DV-A4VX	0.265758288	0.586265993	0.645792075	
2623		0.659232003	0.645275723	0.661179882	0.128916635
2624		0.60960788	0.394939458	0.701259392	0.415108982
2625		0.359694551	0.766325008	0.726397036	0.739380756
2626		0.506993761	0.265747993	0.739044284	0.514211967
2627		0.520989798	0.480673878	0.441983555	0.39210141

TCGA_TCGA-CJ-6030 0.344905017 0.588421839 0.776735795
0.666406259 0.657500863 0.689936309 0.282317367
0.696244991 0.569815202 0.753140419 0.588862984
0.564549242 0.906878892 0.795725867 0.836176518
0.546696117 0.204292349 0.783325315 0.732432383
0.674557446 0.595328807 0.406410584 0.453927317
TCGA_TCGA-B0-4818 0.388793629 0.510748584 0.720604423
0.635647862 0.639337017 0.729779304 0.31305009
0.611594882 0.502139238 0.69038597 0.407192655
0.46201684 0.83582355 0.754696278 0.804013578
0.517627056 0.338640265 0.770147976 0.580128503
0.578233553 0.539887975 0.403729101 0.417200144
TCGA_TCGA-B0-5706 0.573436005 0.642162631 0.781193403
0.676386966 0.646692683 0.712781992 0.405236081
0.654463442 0.637282456 0.719403209 0.447180171
0.53987451 0.841515555 0.774159575 0.781315569
0.556884861 0.201675481 0.771350173 0.65314777
0.601196984 0.5713908 0.343144359 0.502722436
TCGA_TCGA-BP-4973 0.25320377 0.496124851 0.680422943
0.639159822 0.640591371 0.735700721 0.250037521
0.600272473 0.507445123 0.71792184 0.396673418
0.46575916 0.79352504 0.735998594 0.797465767

0.517040768 0.231695634 0.77785315 0.616003694
0.589051583 0.53128351 0.409681588 0.37898878
TCGA_TCGA-B8-5165 0.341651666 0.443304636 0.564266693
0.583281426 0.659006298 0.694323281 0.340958585
0.657851427 0.468015512 0.720097672 0.405382296
0.556199941 0.691931394 0.764133065 0.765787134
0.562723868 0.301167694 0.752663681 0.626110179
0.572919848 0.541297342 0.390936863 0.452425669
TCGA_TCGA-CJ-4901 0.484641419 0.705817718 0.883169904
0.68897572 0.651051954 0.749360228 0.277780979
0.664810481 0.625634134 0.689615832 0.544311668
0.520770822 0.910456049 0.751534492 0.840588785
0.585965706 0.104338832 0.786225741 0.750178489
0.670101317 0.647398489 0.431639782 0.49095472
TCGA_TCGA-B0-5110 0.245545157 0.410144278 0.558170174
0.591558022 0.623700145 0.693687926 0.326208769
0.616517915 0.486467717 0.705455349 0.363322409
0.506249627 0.74847701 0.749921181 0.795749225
0.534001848 0.237239296 0.782113036 0.628627886
0.575677993 0.52978833 0.397372356 0.46882427
TCGA_TCGA-B0-4838 0.362080006 0.576616224 0.765199145
0.684456897 0.636117757 0.717262474 0.244135133

0.608266545 0.561350531 0.697123493 0.431543753
0.447205059 0.841106109 0.731492925 0.813227301
0.527117556 0.265653831 0.762876275 0.656644827
0.632890952 0.56599548 0.429923349 0.437137326
TCGA_TCGA-BP-4968 0.305630677 0.553910908 0.787644885
0.626387439 0.651698873 0.754948909 0.255663784
0.649548827 0.463192587 0.691123932 0.443295952
0.433870384 0.816577254 0.769229443 0.778014524
0.52071025 0.19273422 0.759587331 0.618996143
0.580597242 0.545028313 0.380978393 0.430543984
TCGA_TCGA-A3-A8OX 0.388042877 0.528257255 0.706507247
0.622175677 0.632709971 0.676371805 0.195757936
0.59936407 0.476094067 0.738454742 0.445814621
0.417866807 0.778383367 0.767765675 0.783372185
0.541227482 0.157101153 0.776411398 0.62183812
0.598293245 0.557594636 0.388265775 0.465884119
TCGA_TCGA-A3-A6NN 0.252677825 0.424670276 0.651719131
0.58277021 0.645272103 0.728608516 0.191250669
0.624858469 0.466653584 0.729323247 0.404751140.471672778
0.805335666 0.774647521 0.789392481 0.52182872
0.228493496 0.778542143 0.625110793 0.613102591
0.528842224 0.379824186 0.419764053

TCGA_TCGA-B0-4814 0.287186945 0.459107036 0.552212824
0.592341535 0.636453773 0.706682049 0.279982883
0.593276872 0.436792926 0.700793496 0.346645569
0.395600943 0.7111329900.75071525 0.774094653 0.521556191
0.252464161 0.779698754 0.585263847 0.559609923
0.505207483 0.386075566 0.379063889
TCGA_TCGA-B0-4823 0.318160434 0.529661474 0.697259157
0.610596124 0.659247782 0.714810942 0.288268579 0.6603325
0.479535492 0.75989386 0.459172413 0.513140835
0.805325369 0.769008121 0.773427314 0.569352767
0.199999419 0.759183849 0.672471584 0.584057823
0.55904593 0.372222758 0.473814405
TCGA_TCGA-B0-5098 0.18357509 0.46475604 0.461541848
0.555510342 0.653449528 0.736471715 0.070978534
0.600297015 0.218802356 0.739897327 0.355695865
0.243542443 0.493121150.747715301 0.697155529 0.502037457
0 0.709124715 0.414967245 0.422792947 0.456351229
0.380739987 0.455057387
TCGA_TCGA-B0-5711 0.296726795 0.468785387 0.64228666
0.618296363 0.649645596 0.692912854 0.264048193
0.644740541 0.493961598 0.717908544 0.388869904
0.525351791 0.767953895 0.7639669 0.798495416 0.525695595

0.260455843 0.773322395 0.637063445 0.595476058
0.533730455 0.406252417 0.469586671
TCGA_TCGA-A3-3319 0.25216276 0.561053168 0.611144246
0.676286282 0.632563485 0.645839753 0.185963632
0.612380595 0.468258354 0.741180645 0.467068572
0.337799168 0.839818705 0.732830689 0.780648618
0.553457997 0.224905461 0.751733661 0.603121018
0.563006014 0.510558591 0.476834821 0.437649655
TCGA_TCGA-BP-5007 0.286326483 0.453454451 0.665717441
0.630518789 0.636151509 0.715799373 0.211407276
0.619748158 0.45290347 0.696641181 0.372428304
0.411434567 0.719868532 0.770004878 0.791502110.510395043
0.175301301 0.774385071 0.599609344 0.570389649
0.501304718 0.425867915 0.427242308
TCGA_TCGA-B8-A54D 0.357526321 0.598870259 0.802917916
0.672004651 0.630733271 0.7043686 0.197336148 0.631436455
0.532523286 0.753416699 0.471714677 0.449205259
0.876695255 0.755582786 0.805993514 0.505270221
0.119549298 0.766861048 0.564256801 0.627361259
0.553112188 0.431745963 0.375734463
TCGA_TCGA-B0-4833 0.31796186 0.494266368 0.762913268
0.649105459 0.645785517 0.755787188 0.284772715

0.61604812 0.453294913 0.686983683 0.38733579
0.466927675 0.830259325 0.76822196 0.786319867
0.511541853 0.293748199 0.756525603 0.583848928
0.595263299 0.532220168 0.425482027 0.406293785
TCGA_TCGA-BP-4347 0.249886624 0.45443657 0.590284877
0.582768471 0.648602211 0.7406961 0.306600653 0.61881945
0.440891473 0.695382978 0.319913776 0.466837867
0.69547814 0.760403563 0.757442084 0.49995518
0.206230835 0.78441569 0.571054857 0.55867481
0.487722731 0.369134293 0.470670472
TCGA_TCGA-CW-5590 0.215943417 0.532124155 0.703163101
0.603290029 0.664441636 0.715785078 0.218687396
0.641483239 0.467109365 0.696223885 0.381517257
0.458474796 0.761354352 0.77036009 0.773199331
0.528388278 0.243578928 0.771595292 0.605744951
0.593021101 0.544014126 0.416896646 0.447909131
TCGA_TCGA-B0-4822 0.339926898 0.535639395 0.739163481
0.579589205 0.647058512 0.781676484 0.273471179
0.696653978 0.416447318 0.649165823 0.477583902
0.457364923 0.833961114 0.782224232 0.768592346
0.592999421 0.150199066 0.782627395 0.586481416
0.60644131 0.555362183 0.38965184 0.472403662

TCGA_TCGA-B0-4703 0.327738146 0.562499278 0.70206403
0.641419732 0.605894886 0.704847351 0.196806493
0.619367146 0.463753958 0.714709193 0.474557223
0.447946074 0.806583276 0.750565457 0.763291198
0.56166298 0.12475556 0.770447247 0.680191998
0.605754731 0.621898077 0.393730759 0.489412139
TCGA_TCGA-B0-5702 0.452817095 0.383106185 0.673126201
0.55646983 0.626505043 0.724535305 0.269768836
0.645026251 0.369577604 0.696397539 0.560978718
0.599685004 0.83974593 0.828521149 0.756227512
0.500663058 0.209397234 0.755394654 0.590723241
0.539781329 0.531683856 0.409602863 0.373750301
TCGA_TCGA-CJ-4918 0.370082847 0.48486617 0.679603546
0.62222399 0.637099963 0.700935766 0.37707312
0.679227891 0.486996509 0.700056119 0.464349232
0.563496914 0.838804866 0.777457566 0.759493169
0.544212345 0.278697421 0.786313131 0.690130836
0.613986247 0.57978537 0.35806685 0.483622874
TCGA_TCGA-CJ-4897 0.305920105 0.407668611 0.583140252
0.595024163 0.632377728 0.716548163 0.226666282
0.639151914 0.449323709 0.701730927 0.367342807
0.447278873 0.70892382 0.75537596 0.763038864

0.517913869 0.24343962 0.76337747 0.58884132
0.545880948 0.496517435 0.412818467 0.408444448
TCGA_TCGA-BP-4776 0.403358765 0.515900825 0.697143401
0.6039806 0.631058569 0.797093882 0.417461401 0.622527143
0.397666242 0.684834831 0.372294935 0.508925726
0.77743916 0.769081436 0.764678199 0.565042911
0.296549769 0.758718563 0.533557272 0.569506189
0.548333247 0.366246349 0.432150354
TCGA_TCGA-B4-5377 0.285534855 0.500447075 0.627262813
0.636552351 0.616131851 0.727337449 0.322002087
0.619540146 0.50390136 0.699492535 0.398751307
0.417776771 0.767955641 0.779834056 0.76477849
0.517600638 0.249528739 0.75435707 0.627289033
0.586172428 0.534746596 0.468157737 0.40096699
TCGA_TCGA-BP-4774 0.220062406 0.516957529 0.599006715
0.588425063 0.604979542 0.772630531 0.391121164
0.612239674 0.428012615 0.698326695 0.361904826
0.506265403 0.717518236 0.76582201 0.754482024
0.526988179 0.226351268 0.76649472 0.540916362
0.557016388 0.533971065 0.40420581 0.43590341
TCGA_TCGA-B8-4143 0.371423301 0.693663546 0.785689787
0.655697214 0.648836193 0.753940576 0.193665406

2804 0.642384762 0.568789349 0.684394103 0.447508847

2805 0.424656154 0.862375901 0.741217025 0.788778355

2806 0.515857418 0.197156205 0.761754768 0.693149221

2807 0.599580645 0.591839633 0.395816184 0.457470713

2808 TCGA_TCGA-CJ-5675 0.389451261 0.565585842 0.836116328

2809 0.665008944 0.670372471 0.656735909 0.208199218

2810 0.635037266 0.586293938 0.736921753 0.44983142

2811 0.46381516 0.895054168 0.770541829 0.829189875

2812 0.519324264 0.203035443 0.753632514 0.657016829

2813 0.668548415 0.5779465 0.452393829 0.403436399

2814 TCGA_TCGA-B0-5399 0.351016173 0.515175728 0.706080467

2815 0.647538795 0.648055199 0.732818556 0.273347406

2816 0.601981946 0.492953099 0.73457976 0.385317686

2817 0.432371221 0.762959309 0.750149374 0.797545014

2818 0.537042723 0.194080424 0.783291203 0.591352477

2819 0.603266308 0.555005917 0.426402179 0.425220137

2820 TCGA_TCGA-B0-4707 0.319121372 0.545611060.699401037

2821 0.565929049 0.590049888 0.770121177 0.121580265

2822 0.599536136 0.326244077 0.646872408 0.310663516

2823 0.312228407 0.682778982 0.733381916 0.705061886

2824 0.491215447 0.119537114 0.740764614 0.429473474

2825 0.527283663 0.535483265 0.393486864 0.445678668

TCGA_TCGA-CZ-4865 0.333541167 0.469705305 0.719053677
0.633329821 0.655785387 0.746466136 0.232428029
0.676898614 0.503489363 0.750808148 0.48694641
0.491731472 0.817611980.766816777 0.80154701 0.548590815
0.191398872 0.770265723 0.633957317 0.602343853
0.556150012 0.398079193 0.468173155
TCGA_TCGA-BP-4344 0.290844592 0.426801657 0.533751454
0.581778536 0.628353686 0.699373679 0.36515185
0.631529564 0.505574939 0.719774418 0.421374292
0.533592371 0.769235231 0.775497049 0.778167179
0.529290648 0.226710261 0.788711379 0.688121021
0.612487101 0.550564645 0.355048536 0.480905721
TCGA_TCGA-DV-5565 0.276461908 0.422415072 0.573123571
0.632064045 0.665234174 0.742713204 0.27381372
0.674648849 0.40821052 0.703293418 0.425520665
0.488597737 0.798904828 0.788701581 0.768864076
0.580451138 0.337596699 0.759044961 0.591150015
0.587481264 0.531802705 0.39181742 0.434673205
TCGA_TCGA-AK-3436 0.251489084 0.510017972 0.638356515
0.576079644 0.627828851 0.68768334 0.295507778
0.675700421 0.379432578 0.6815001 0.362112450.422005736
0.711569018 0.763793676 0.707465078 0.504976129

0.203771357 0.762743859 0.551410719 0.567446248
0.527437218 0.420966902 0.465682073
TCGA_TCGA-A3-A6NJ0.426312213 0.552442745 0.787966529
0.625341045 0.625950272 0.709104819 0.336651418
0.612673755 0.535442168 0.727851716 0.473515818 0.4866985
0.880793752 0.759193583 0.780895767 0.54503722
0.297774222 0.767944521 0.603325744 0.599991120.567848065
0.41418188 0.446956844
TCGA_TCGA-BP-5191 0.459909658 0.636202761 0.874232912
0.686896981 0.678142102 0.71346126 0.213450487
0.687390393 0.569589213 0.740600379 0.484811715
0.501936074 0.91925616 0.781347344 0.750967448
0.549521767 0.270452177 0.775933198 0.645303238
0.640656704 0.592380414 0.470233146 0.467643808
TCGA_TCGA-B8-4154 0.266456073 0.522102053 0.757825341
0.615358639 0.667740713 0.72330239 0.320668637
0.675894382 0.481361661 0.727094586 0.431884171
0.441886489 0.792497497 0.77096972 0.771323321
0.526461404 0.286115223 0.758901498 0.588929241
0.613343775 0.550528516 0.40370016 0.42049802
TCGA_TCGA-B0-4688 0.179921644 0.642284745 0.588559445
0.614236449 0.596985332 0.688178813 0.179006977

2870 0.603900943 0.385481653 0.660872932 0.557838641

2871 0.444707904 0.793286756 0.71690644 0.660775408

2872 0.520779214 0.191610854 0.751374916 0.648235789

2873 0.566649255 0.498266377 0.306721335 0.54054586

2874 TCGA_TCGA-B0-5697 0.346051426 0.52216964 0.764168957

2875 0.682571338 0.646324226 0.704624036 0.27828498

2876 0.650409829 0.544876434 0.760511130.485370221 0.491882282

2877 0.88758662 0.788933446 0.824241218 0.532182589

2878 0.206558268 0.771579825 0.63529415 0.631995797

2879 0.558042478 0.488278304 0.427788082

2880 TCGA_TCGA-BP-43430.406116409 0.593155744 0.733236482

2881 0.662464339 0.668578518 0.713161907 0.241673554

2882 0.706388643 0.565028235 0.762697715 0.484025025

2883 0.574730294 0.851026184 0.769504143 0.791232217

2884 0.561245431 0.099587921 0.763578367 0.708831001

2885 0.656863819 0.599846547 0.400765707 0.544468482

2886 TCGA_TCGA-MW-A4EC 0.244231879 0.431550626 0.581254689

2887 0.597825055 0.632193661 0.748481682 0.302333565

2888 0.615733608 0.419345784 0.7307224 0.335544873 0.508266868

2889 0.703980318 0.763611976 0.730415328 0.510235281

2890 0.214116738 0.77710553 0.598836528 0.559063403

2891 0.508921115 0.390269486 0.421567013

TCGA_TCGA-CZ-5452 0.431652976 0.669799512 0.849895243
0.662465144 0.651390884 0.679679503 0.25293375
0.648251208 0.608249138 0.695864634 0.478098808
0.45883953 0.864130806 0.777566044 0.797971464
0.530493966 0.12715542 0.772706827 0.673575156
0.626462279 0.601765471 0.391121780.395525942
TCGA_TCGA-B0-5699 0.187320021 0.477919871 0.58956749
0.618184489 0.637426257 0.734234562 0.22556489
0.617986238 0.482636903 0.694089575 0.419925705
0.387849917 0.739017459 0.775884802 0.795337041
0.504078228 0.214422929 0.749132764 0.561651342
0.57300219 0.519408083 0.419484396 0.388311256
TCGA_TCGA-CZ-5460 0.332102095 0.571249385 0.743467935
0.609347286 0.658797586 0.758993346 0.330834421
0.640440125 0.509260048 0.709744796 0.468410681
0.474083802 0.82558923 0.776069478 0.795378282
0.533047356 0.306205993 0.776291942 0.623641589
0.60470474 0.567055774 0.411621870.410590913
TCGA_TCGA-B0-4845 0.191767847 0.449982594 0.490333042
0.593673545 0.623203135 0.767023409 0.270410302
0.604555737 0.378302594 0.698921886 0.327960243
0.437383205 0.718648194 0.753421301 0.736081193

0.488069912 0.296617815 0.777603994 0.500871493
0.540626675 0.496142366 0.377773261 0.415077265
TCGA_TCGA-MM-A563 0.457374451 0.561083874 0.770801746
0.632590429 0.660396422 0.734823464 0.167069516
0.665355128 0.513161312 0.762859137 0.510012209
0.526295712 0.904243302 0.789037818 0.797719082
0.548074467 0.255557287 0.788527384 0.67578508
0.643444343 0.577332117 0.380664587 0.415373655
TCGA_TCGA-BP-4971 0.379315411 0.538598662 0.690986108
0.682578994 0.637846138 0.770467271 0.284469549
0.617723796 0.539178612 0.704445522 0.525068814
0.500426031 0.852825651 0.746295484 0.755312418
0.54599935 0.146938548 0.775966824 0.71013905
0.619088388 0.593271821 0.39954271 0.437037529
TCGA_TCGA-AK-3425 0.361613762 0.523068138 0.754561528
0.633292192 0.655587206 0.716657116 0.260936753
0.713300649 0.526695183 0.716537263 0.559121508
0.511852479 0.869609188 0.779028153 0.797853615
0.569110217 0.198343569 0.776915241 0.681398439
0.675364576 0.590285961 0.411460070.453316362
TCGA_TCGA-CZ-4858 0.364497825 0.715936569 0.770457444
0.654769231 0.635964281 0.690979811 0.215867076

2936		0.683532515	0.520486695	0.669588865	0.488088428
2937		0.470668992	0.836653109	0.750143616	0.737182626
2938		0.499586087	0.161451427	0.757623438	0.686341199
2939		0.619533856	0.581056526	0.391288211	0.537718786
2940	TCGA_TCGA-B0-4841	0.248333435	0.461181192	0.665006846	
2941		0.551171585	0.670816059	0.758707404	0.320456794
2942		0.678035492	0.353589942	0.692469267	0.420594165
2943		0.485067714	0.782021202	0.824883767	0.756131457
2944		0.574296404	0.285655788	0.756695864	0.525019127
2945		0.564309734	0.531549922	0.357793785	0.481090356
2946	TCGA_TCGA-B0-4819	0.540545082	0.716838565	0.89626659	
2947		0.701757691	0.671150238	0.733856458	0.387920609
2948		0.649089618	0.618247841	0.753367251	0.539180776
2949		0.533497527	0.949737436	0.764697924	0.832184023
2950		0.598616535	0.272638918	0.782701735	0.681852133
2951		0.673048266	0.660845928	0.393914654	0.502134965
2952	TCGA_TCGA-G6-A5PC	0.323652597	0.482563843	0.764420448	
2953		0.595545898	0.677967254	0.760975764	0.220508584
2954		0.607174133	0.417374482	0.688082844	0.429405252
2955		0.416460348	0.812179411	0.794263249	0.736530903
2956		0.514814299	0.243844319	0.76040195	0.455142475
2957		0.557121632	0.543592628	0.449032222	0.334664045

TCGA_TCGA-BP-4992 0.395694862 0.581407328 0.822659314
0.675257867 0.602046955 0.765001747 0.244070801
0.574480419 0.502156245 0.680535358 0.440755424
0.436287562 0.867459773 0.728463774 0.757531508
0.513063555 0.187392549 0.736665571 0.568548541
0.591111369 0.573038697 0.422889194 0.379333122
TCGA_TCGA-CZ-4864 0.3334065 0.505658627 0.772966865
0.676302129 0.650566811 0.655391564 0.299393961
0.618911884 0.578477768 0.72006588 0.455550789
0.480061359 0.874315054 0.739368931 0.839581707
0.528800479 0.196450907 0.776262654 0.681232582
0.642523585 0.541726037 0.447225503 0.426024255
TCGA_TCGA-BP-5198 0.366386304 0.625299617 0.644704397
0.658390784 0.621309665 0.720258413 0.285834426
0.662936055 0.487741256 0.696956902 0.530307019
0.480619217 0.865993237 0.770493058 0.822067159
0.568578463 0.197852156 0.753363212 0.705250272
0.604552979 0.597502473 0.432169157 0.438752303
TCGA_TCGA-BP-4349 0.277742609 0.428688959 0.602767443
0.608126525 0.634749872 0.763314835 0.294103908
0.630862892 0.427180676 0.69405728 0.398315591
0.406995431 0.712549423 0.740934817 0.784976804

2980 0.531651884 0.251613053 0.778129761 0.54122762

2981 0.526974016 0.532065321 0.389759236 0.413512025

2982 TCGA_TCGA-CJ-4878 0.204763527 0.468016619 0.46720048

2983 0.625335565 0.632940759 0.746252622 0.262216504

2984 0.616137304 0.383232731 0.712058128 0.348499205

2985 0.421341521 0.721365116 0.750953209 0.773832478

2986 0.516451585 0.348512551 0.773653672 0.525807702

2987 0.548727212 0.500217053 0.418597932 0.421396969

2988 TCGA_TCGA-CJ-5672 0.373939799 0.546534843 0.80235289

2989 0.658911485 0.646843468 0.726466493 0.19908469

2990 0.664361494 0.570254971 0.725402563 0.509120848

2991 0.513844595 0.877816701 0.8025701 0.810385124 0.53993003

2992 0.194292142 0.764768639 0.683991291 0.669019317

2993 0.57332027 0.445098889 0.447355469

2994 TCGA_TCGA-BP-4765 0.293219651 0.445600519 0.500145466

2995 0.613458415 0.621086556 0.719511168 0.359828135

2996 0.612103752 0.407024457 0.714680589 0.344215671

2997 0.509545412 0.699382205 0.739228772 0.775763933

2998 0.536608185 0.324252691 0.769374182 0.559828732

2999 0.538753471 0.519860966 0.356042123 0.44976813

3000 TCGA_TCGA-BP-4970 0.353266261 0.51394659 0.693470883

3001 0.653179981 0.632808782 0.693732188 0.209942805

3002		0.628837367	0.537251008	0.745370963	0.482385537	
3003		0.479430719	0.827164221	0.748810636	0.811011555	
3004		0.563748002	0.1742603	0.766402322	0.679658713	0.631977496
3005		0.572179926	0.440707761	0.490593549		
3006	TCGA_TCGA-BP-5009	0.336719097	0.503776758	0.736492734		
3007		0.670952356	0.639298927	0.718926368	0.264495846	
3008		0.644597215	0.498965607	0.693308267	0.466964457	
3009		0.453076938	0.849215012	0.769837051	0.801221762	
3010		0.524647051	0.14685994	0.79458691	0.653882525	
3011		0.615906969	0.551935241	0.463536471	0.426237652	
3012	TCGA_TCGA-CJ-4900	0.573950693	0.638873683	0.810858011		
3013		0.698630276	0.631195594	0.74493235	0.250250088	
3014		0.694216612	0.584824991	0.72796373	0.531007967	
3015		0.501029082	0.951873723	0.761202556	0.82019245	
3016		0.566358256	0.225623537	0.794694425	0.71250531	
3017		0.611715794	0.595710181	0.365725851	0.410130546	
3018	TCGA_TCGA-BP-5186	0.330589542	0.348323202	0.593623965		
3019		0.608378074	0.627531332	0.718069027	0.324508573	
3020		0.605956067	0.447256507	0.697051726	0.371949046	
3021		0.50755951	0.663873006	0.743717144	0.775687861	
3022		0.512919947	0.260728849	0.765248551	0.567429602	
3023		0.563532771	0.523295966	0.390747564	0.447882099	

TCGA_TCGA-A3-3370 0.355687111 0.510500754 0.713135246
0.660220879 0.626300808 0.711000383 0.255370746
0.604666995 0.523682441 0.710527449 0.397122978
0.494813874 0.818589581 0.736544315 0.794840283
0.525561703 0.237242603 0.777039324 0.669147198
0.595820717 0.5316096 0.42359374 0.44353328
TCGA_TCGA-B0-4810 0.35906149 0.58986276 0.815129873
0.639980576 0.643122829 0.738194474 0.18415074
0.680226132 0.451182972 0.720175469 0.434639110.4770993
0.877883432 0.747435949 0.78976379 0.57000888
0.254922485 0.775340814 0.659000374 0.610208576
0.572963404 0.385926496 0.484031996
TCGA_TCGA-CJ-4916 0.423378237 0.578291776 0.843099734
0.68970316 0.662223402 0.715146483 0.295123084
0.649312549 0.58544035 0.728530394 0.490428594
0.470218688 0.911849986 0.762607768 0.821447425
0.546676255 0.238087397 0.778188986 0.668148616
0.651464735 0.577245858 0.41090533 0.435461302
TCGA_TCGA-B0-4846 0.289604193 0.54692125 0.688641827
0.659197788 0.637297769 0.693155723 0.392336034
0.587656007 0.532523231 0.692703712 0.433997455
0.50703392 0.799466125 0.735810321 0.829618618

	0.525204582	0.206329337	0.767520815	0.646075013
	0.617649834	0.571967478	0.414463289	0.461213898
TCGA_TCGA-BP-4756	0.263491132	0.407343641	0.570059137	
	0.633611758	0.656113875	0.672260524	0.292192105
	0.609779188	0.428527405	0.710764363	0.343205456
	0.336623696	0.648994578	0.785653842	0.62784698
	0.497203256	0.111050666	0.788446479	0.461301058
	0.53895406	0.464640762	0.337264752	0.311574794
TCGA_TCGA-B0-5088	0.365760394	0.552752643	0.722210999	
	0.639044994	0.634528595	0.777479729	0.32686364
	0.679447947	0.499428965	0.713073557	0.488204889
	0.534219988	0.852261457	0.784860588	0.776941509
	0.565818253	0.194432997	0.782353861	0.6277102	0.59842065
	0.556332372	0.366940802	0.40270845	
TCGA_TCGA-B0-4837	0.215144605	0.498702092	0.597662712	
	0.556637274	0.620509411	0.738087932	0.257622209
	0.713205041	0.420640779	0.701620494	0.418643354
	0.44641637	0.776950721	0.80962197	0.779093859
	0.527369756	0.243220402	0.757207498	0.580313452
	0.542249483	0.530569513	0.383784368	0.432330021
TCGA_TCGA-BP-5174	0.257107951	0.417136005	0.60244309	
	0.617964227	0.631077333	0.728328739	0.184364268

3068		0.658341853	0.436448529	0.704920983	0.420125724
3069		0.407856097	0.749141614	0.772492747	0.750926018
3070		0.505663469	0.167814672	0.773693017	0.563329836
3071		0.550130036	0.498218733	0.431430018	0.374116387
3072	TCGA_TCGA-BP-4803	0.269717201	0.43340667	0.59974348	
3073		0.584066946	0.628518619	0.702195328	0.31051761
3074		0.624198879	0.458134396	0.744913756	0.398178619
3075		0.466115720.713315767	0.778022403	0.770513051	0.529269814
3076		0.241055146	0.784852436	0.597656711	0.588068093
3077		0.520993109	0.392150432	0.420409012	
3078	TCGA_TCGA-B0-5097	0.262287093	0.663997342	0.604763739	
3079		0.681933044	0.618652725	0.699759303	0.391519747
3080		0.735809027	0.495224817	0.721385197	0.544817079
3081		0.592231461	0.854993543	0.770641228	0.810035059
3082		0.594238368	0.182887347	0.780262735	0.711641045
3083		0.603741042	0.585156186	0.395417574	0.502614559
3084	TCGA_TCGA-B0-5119	0.160367675	0.452989825	0.659337419	
3085		0.596652188	0.638536268	0.698946891	0.334278221
3086		0.629217137	0.469019689	0.687836247	0.344627668
3087		0.492098326	0.709224898	0.771706968	0.775842862
3088		0.527546644	0.295531835	0.770241473	0.588060469
3089		0.56438374	0.504477642	0.408438561	0.440157682

TCGA_TCGA-BP-4345	0.343034277	0.533928682	0.698278056	
	0.656599502	0.663166406	0.747861502	0.334168384
	0.699467341	0.523181483	0.680710634	0.518825763
	0.563280388	0.879479792	0.789494972	0.743340685
	0.579782191	0.172283957	0.785930228	0.782533635
	0.596349502	0.591410621	0.370007088	0.528775782
TCGA_TCGA-AK-3453	0.401119614	0.5905215	0.830028203	
	0.671693778	0.623163017	0.716411579	0.323181684
	0.605879585	0.509870871	0.738721543	0.451716318
	0.479812933	0.91300604	0.771372652	0.782907946
	0.509677796	0.211165644	0.760318476	0.593878142
	0.616531054	0.551322966	0.47432767	0.428564678
TCGA_TCGA-BP-4342	0.362536587	0.509820059	0.60877229	
	0.593210846	0.586661167	0.696494525	0.295309372
	0.629519041	0.478924397	0.661958071	0.393896177
	0.430516557	0.759789459	0.757986246	0.754899798
	0.52800067	0.19182555	0.753266427	0.607454651
	0.582947213	0.537722745	0.399134615	0.427780713
TCGA_TCGA-B0-4844	0.345473983	0.530644878	0.685815671	
	0.631509697	0.62937147	0.724043197	0.137478218
	0.585043861	0.425550926	0.673131702	0.400808599
	0.347801486	0.77195715	0.74946585	0.745788539

0.530958064 0.2550545 0.748908532 0.541021725 0.54073963
0.515252219 0.435907138 0.371959202
TCGA_TCGA-BP-4804 0.441714775 0.56514672 0.798398636
0.645185577 0.664109052 0.718851247 0.265478398
0.662858759 0.567958534 0.717506185 0.476048325
0.549334009 0.861455919 0.785193987 0.815585674
0.561470009 0.187339319 0.775918562 0.711343553
0.649368235 0.580098523 0.441871188 0.470994821
TCGA_TCGA-CW-6088 0.276463514 0.389427616 0.495272364
0.573799024 0.634322504 0.715466285 0.272891673
0.645762199 0.436243037 0.700236213 0.335793238
0.467178822 0.685209264 0.757325985 0.791224118
0.513753154 0.3009445 0.770841491 0.600245244 0.567492594
0.491683497 0.390126375 0.457994417
TCGA_TCGA-B0-5701 0.272114142 0.541532954 0.657414444
0.648938333 0.630614049 0.671568504 0.196445804
0.696295987 0.467621676 0.734091392 0.426958104
0.398068183 0.749211420.752821832 0.761161148 0.5165889
0.230332021 0.730691245 0.607776715 0.568960672
0.521689857 0.433714367 0.497046132
TCGA_TCGA-CJ-5681 0.179104876 0.395545467 0.399234817
0.599079311 0.623036952 0.623247051 0.111740719

0.649783125 0.377956407 0.713910345 0.314289787
0.329630231 0.620546657 0.747449937 0.564627468
0.429179681 0.049504921 0.795890243 0.475593275
0.538828429 0.473344667 0.299824196 0.394130253
TCGA_TCGA-B0-4843 0.285855422 0.491097431 0.581323511
0.62868137 0.631265439 0.78355434 0.328649082
0.691359106 0.380966612 0.674270673 0.426514359
0.461215827 0.775954507 0.804470486 0.773250766
0.529940905 0.175679486 0.76641607 0.586407099
0.549072953 0.549909409 0.394835743 0.456264676
TCGA_TCGA-BP-4766 0.20302194 0.391128638 0.498517882
0.625420823 0.600656506 0.742861154 0.306339797
0.638072877 0.435481073 0.714119650.387206495 0.467919797
0.767543084 0.745016842 0.751742166 0.53901017
0.339651583 0.772114850.655654827 0.58426625 0.498095607
0.382444291 0.431187635
TCGA_TCGA-B8-A54H 0.254211216 0.425861262 0.724536746
0.610686304 0.632721236 0.727021459 0.226751073
0.600927961 0.449928497 0.679357783 0.341982671
0.37271114 0.772898295 0.777398029 0.780525721 0.521662741
0.21372765 0.778852295 0.561710008 0.5672973 0.528253792
0.382099101 0.382751091

TCGA_TCGA-B4-5836 0.302680082 0.517733949 0.659523659 0.624971199 0.633264377 0.683650991 0.316230796 0.648015925 0.472516467 0.69545261 0.408514085 0.524142571 0.78957664 0.745174146 0.781151801 0.5345558 0.335873487 0.76191425 0.624454745 0.570452411 0.526816351 0.422453468 0.483684416

TCGA_TCGA-B0-5117 0.183364935 0.313683209 0.476471135 0.534658026 0.652240483 0.792209487 0.304746364 0.529837029 0.323323291 0.669406729 0.231906602 0.368638836 0.457035247 0.781270535 0.637359762 0.408777765 0.148722907 0.753430248 0.350153981 0.42715186 0.404921644 0.444880066 0.426722103

TCGA_TCGA-CJ-5680 0.222229819 0.422337706 0.503667748 0.59088509 0.616601627 0.739360211 0.169370906 0.607101968 0.418062158 0.726025483 0.351227271 0.412135279 0.753086054 0.794004622 0.732551208 0.489941114 0.209744234 0.770452829 0.567023145 0.539143405 0.497550019 0.412554211 0.337501605

TCGA_TCGA-CW-5589 0.335117206 0.466178743 0.577836669 0.606055506 0.667167971 0.722516284 0.223699872 0.672191438 0.464057783 0.737974518 0.431505918 0.525771353 0.790576174 0.765676707 0.802705854

0.560841975 0.232743831 0.757772236 0.651281253
0.601704411 0.533438254 0.363353488 0.434584242
TCGA_TCGA-BP-5000 0.34155945 0.531194386 0.768262415
0.630238992 0.640274556 0.742740239 0.292595478 0.6154502
0.45837079 0.715213554 0.451900603 0.438268799
0.797805012 0.780279338 0.805374991 0.545566123
0.20556612 0.767218383 0.583629301 0.577517189
0.549027657 0.393713236 0.434963406
TCGA_TCGA-A3-3346 0.23800434 0.628650618 0.687048081
0.699837961 0.629601011 0.662519877 0.132612845
0.686960737 0.460887841 0.753421932 0.51563581
0.479786112 0.919987775 0.712546129 0.724404802
0.516184717 0.24849099 0.782820321 0.733214382
0.591846571 0.564863166 0.390806912 0.462257039
TCGA_TCGA-CJ-5689 0.303697169 0.541441072 0.730580152
0.614115153 0.691272257 0.747739787 0.370614598
0.767650704 0.459684167 0.74348512 0.498852162
0.547377552 0.843145073 0.783729827 0.787387177
0.576642333 0.292792613 0.764196525 0.703532791
0.58025577 0.574004791 0.388151787 0.486212581
TCGA_TCGA-3Z-A93Z 0.351207013 0.402529781 0.624923426
0.570928556 0.642023052 0.711763269 0.254740602

3200		0.616484779	0.413512841	0.719177532	0.340453471
3201		0.423026828	0.735014069	0.792856294	0.75471573
3202		0.492182736	0.194851746	0.777224992	0.561564456
3203		0.562218802	0.508618761	0.390003564	0.461520977
3204	TCGA_TCGA-CJ-4638	0.28624686	0.442831255	0.629375399	
3205		0.583980059	0.633991307	0.773217086	0.158559832
3206		0.605652865	0.342056576	0.674938417	0.32588455
3207		0.34754082	0.642384718	0.764135052	0.722440437
3208		0.486155981	0.127572842	0.732547224	0.404701644
3209		0.497905729	0.493306516	0.438669206	0.347751212
3210	TCGA_TCGA-BP-5184	0.358791997	0.486714487	0.658582324	
3211		0.604575393	0.631330956	0.724505866	0.383972341
3212		0.670036986	0.471950668	0.709930025	0.414608639
3213		0.518209447	0.747020848	0.771683589	0.757383847
3214		0.55799921	0.291906935	0.751721379	0.614032939
3215		0.58807172	0.554142319	0.4030211	0.476159795
3216	TCGA_TCGA-B0-5116	0.152516935	0.423677786	0.549114851	
3217		0.54027816	0.627189702	0.756302293	0.258166524
3218		0.623219613	0.359863157	0.66255666	0.325082685
3219		0.434913912	0.575908318	0.782326947	0.772528016
3220		0.50343385	0.168287282	0.775173081	0.54070301
3221		0.539764588	0.52704697	0.422177859	0.46417034

TCGA_TCGA-CZ-4857 0.315321323 0.582540609 0.598999854
0.59794038 0.62031673 0.706974312 0.154747717
0.664667589 0.481152385 0.699431246 0.457512891
0.415756117 0.805909696 0.730574549 0.777380952
0.55566758 0.190141825 0.777277312 0.675985046
0.591603078 0.54965033 0.397748908 0.437519474
TCGA_TCGA-BP-5192 0.206585393 0.372003994 0.513935421
0.551409236 0.619905226 0.712694041 0.339854 0.60143524
0.376851388 0.71618362 0.283572527 0.423724818
0.562629341 0.766537829 0.754547572 0.479706575
0.174584342 0.748925454 0.51827769 0.512682297
0.49309441 0.415902883 0.423144036
TCGA_TCGA-B8-4620 0.213525963 0.603815223 0.565725906
0.643230364 0.636581191 0.705030869 0.386450096
0.655180468 0.512536383 0.750883141 0.518082409
0.574264629 0.865016554 0.774530934 0.768771024
0.554098923 0.446792197 0.810319824 0.686266747
0.62885179 0.55600374 0.39480444 0.462885392
TCGA_TCGA-BP-4959 0.281320712 0.526494476 0.615456658
0.621081263 0.619417973 0.721921616 0.28184395
0.689404802 0.512990378 0.710599332 0.507782666
0.520962956 0.849166529 0.780030637 0.768517363

0.518956538	0.167507124	0.763061343	0.650988579
0.597446707	0.572295549	0.434276542	0.39059183
TCGA_TCGA-CJ-4905	0.242931306	0.376117171	0.609674622
0.567325366	0.625462289	0.700997226	0.280374412
0.648980956	0.444102114	0.683135529	0.364111598
0.500489926	0.737016824	0.804861472	0.784618703
0.537354212	0.342015985	0.772633978	0.61276013
0.562645203	0.536136629	0.377837079	0.461574786
TCGA_TCGA-BP-5181	0.237217574	0.406725924	0.605257433
0.579184688	0.655920419	0.718372544	0.256720685
0.62259368	0.451575906	0.727043826	0.360442107
0.456323702	0.732025058	0.769723124	0.742091314
0.526026672	0.285199973	0.759222583	0.585044609
0.561669575	0.533034904	0.370581438	0.393274942
TCGA_TCGA-EU-5906	0.217640958	0.498908633	0.553959734
0.619523497	0.644873909	0.699926921	0.380077811
0.644913842	0.482238339	0.703781358	0.40267747
0.569471979	0.76776476	0.768054666	0.798025972
0.555937976	0.321267758	0.760233042	0.63376704
0.579734363	0.533274336	0.37430915	0.474425115
TCGA_TCGA-CJ-4868	0.349319676	0.60769581	0.75626203
0.643935881	0.648259175	0.746282726	0.238016403

	0.743262037	0.511740809	0.722137406	0.591289975
	0.520256801	0.870380136	0.780051963	0.813974335
	0.612437989	0.228234696	0.773515241	0.773182802
	0.657082591	0.595940059	0.364183865	0.514959254
TCGA_TCGA-B8-4148	0.395046126	0.575398888	0.770205147	
	0.676346438	0.599456628	0.758960503	0.344110126
	0.636232923	0.571008918	0.685683585	0.377529488
	0.470985425	0.840008979	0.740876176	0.825899583
	0.505412392	0.176231314	0.766963346	0.652161853
	0.617348808	0.570627137	0.449328252	0.415127175
TCGA_TCGA-CJ-4886	0.338845833	0.520105594	0.707580717	
	0.617749091	0.661868523	0.731832795	0.313638921
	0.643209337	0.500023301	0.698995194	0.412853223
	0.495216558	0.760923481	0.757190626	0.787466335
	0.535235877	0.200964467	0.763929347	0.633730317
	0.597072741	0.56563985	0.397502435	0.470028921
TCGA_TCGA-AK-3429	0.389000191	0.565854445	0.826090781	
	0.635945191	0.66834743	0.728178978	0.322259255
	0.653401367	0.522346266	0.707551502	0.390922696
	0.524637467	0.837033256	0.766265894	0.764940136
	0.516903736	0.189482847	0.774642154	0.64219958
	0.618772859	0.574520054	0.436973558	0.475531115

TCGA_TCGA-CZ-5465 0.225734769 0.482158425 0.489717321
0.580587148 0.644003804 0.685068387 0.299811307
0.59382895 0.444549452 0.734080645 0.358528998
0.470150856 0.658581943 0.757302813 0.760835392
0.496122166 0.175556556 0.780909268 0.584182936
0.588204814 0.507307938 0.422405221 0.401031215
TCGA_TCGA-B0-4714 0.34933733 0.515509792 0.83252429
0.654714282 0.639574104 0.764864548 0.221826358
0.637962325 0.533638701 0.717919164 0.453279836
0.454120444 0.892813082 0.757193091 0.799525142
0.539639739 0.215104215 0.784645712 0.635001035
0.624842163 0.575051558 0.413838568 0.423490809
TCGA_TCGA-AK-3461 0.284768498 0.511922640.616842274
0.634974992 0.649593237 0.754419392 0.276350176
0.626152595 0.411237933 0.728490362 0.367316804
0.485739491 0.777330342 0.763240815 0.806742109
0.549919926 0.361791485 0.771087538 0.573379805
0.555671138 0.545088786 0.399246685 0.456645546
TCGA_TCGA-B8-5159 0.253456444 0.480687751 0.650784692
0.628277812 0.630052555 0.689551436 0.220751373
0.608381629 0.491341327 0.713893894 0.408364158
0.454294885 0.738996243 0.755336126 0.757642885 0.5135891

0.210856137 0.767547837 0.58483777 0.555786765
0.501557397 0.436591866 0.436549699
TCGA_TCGA-A3-3358 0.369967444 0.587686472 0.758074139
0.681134617 0.680317518 0.714498533 0.293357903
0.683283729 0.551889276 0.731065711 0.510466021
0.546554133 0.894754718 0.769646046 0.800944101
0.546410895 0.260785008 0.774753582 0.668542242
0.660987354 0.563339796 0.400537921 0.44099219
TCGA_TCGA-A3-3372 0.244836872 0.454638344 0.599754273
0.641512976 0.618650215 0.709780137 0.320184414
0.614187701 0.485786575 0.712127761 0.397585039
0.478339757 0.763048957 0.757319722 0.755245835
0.515857138 0.245192643 0.791312127 0.654377918
0.624415162 0.52518263 0.402105556 0.453037917
TCGA_TCGA-CJ-6032 0.245131111 0.489818647 0.605684396
0.60555426 0.631894181 0.737180469 0.352932459
0.610123548 0.45480142 0.685720367 0.344931032
0.441721647 0.713649698 0.759903324 0.784957484
0.543912838 0.246459511 0.784927757 0.594842705
0.561219627 0.515348262 0.452761948 0.440265064
TCGA_TCGA-B0-5703 0.253140642 0.511451222 0.769819813
0.529432301 0.626626702 0.766749588 0.185884394

3332	0.637722721	0.371236973	0.646500693	0.285514615
3333	0.433740082	0.724898653	0.768847871	0.725800262
3334	0.53709342	0.313194987	0.724530661	0.535057125
3335	0.502650849	0.546973579	0.404445014	0.41081398
3336	TCGA_TCGA-B8-A54F	0.250366846	0.425515422	0.516376835
3337	0.622812564	0.640118617	0.706924223	0.234303721
3338	0.590452193	0.461294908	0.718059381	0.495158118
3339	0.48152319	0.82633213	0.728045419	0.800167961
3340	0.516539574	0.208855732	0.764180868	0.619556655
3341	0.599689221	0.526833931	0.377818483	0.421641474
3342	TCGA_TCGA-B0-5095	0.331815131	0.492413512	0.626454027
3343	0.604678128	0.631326196	0.727735424	0.234061093
3344	0.659118313	0.452735879	0.67419527	0.431938884
3345	0.419129194	0.776752512	0.747860008	0.785015002
3346	0.542675952	0.172856804	0.776614376	0.659026567
3347	0.539814329	0.553050431	0.345834096	0.43859843
3348	TCGA_TCGA-CZ-4861	0.309994357	0.65306084	0.68149813
3349	0.69161931	0.63308351	0.740276584	0.353441037
3350	0.695896525	0.566603696	0.748133901	0.54401995
3351	0.557357966	0.935598934	0.789917703	0.825908695
3352	0.549888724	0.185642407	0.774059619	0.830017675
3353	0.686911579	0.606251774	0.429306972	0.488933728

TCGA_TCGA-A3-3349 0.290452907 0.350809467 0.574290298
0.601207934 0.636809995 0.713244713 0.230897006
0.625863091 0.447985277 0.720292423 0.406535695
0.492495344 0.777691963 0.774788529 0.775512719
0.538519983 0.256254916 0.780060646 0.646964022
0.567674556 0.537450476 0.364559258 0.454413283
TCGA_TCGA-AK-3455 0.358976918 0.552098413 0.850867261
0.643628863 0.65408959 0.704829706 0.246439477
0.656872579 0.505114988 0.674333365 0.32278261
0.369797896 0.776110970.751277016 0.750175856 0.517762611
0.240012708 0.760708625 0.539924267 0.552412572
0.571224092 0.414103867 0.394723205
TCGA_TCGA-CW-5585 0.160988761 0.325652056 0.484267002
0.542720106 0.600762407 0.719562828 0.18587771
0.620835085 0.383698547 0.678173538 0.365661336
0.368720025 0.63165155 0.775334687 0.754494929
0.495383557 0.245972382 0.766854768 0.540263187
0.530003723 0.467711301 0.408554545 0.404600558
TCGA_TCGA-BP-4981 0.299908184 0.473188572 0.698876246
0.665007022 0.658218182 0.736495112 0.226227407
0.626879238 0.415298781 0.750798848 0.463209833
0.435375973 0.797399298 0.781518055 0.792818456

0.532683163 0.197813708 0.770583019 0.504019279
0.554869286 0.516359110.402066238 0.344247185
TCGA_TCGA-BP-4337 0.388463982 0.473456209 0.598005851
0.587207153 0.628915307 0.779497098 0.214549141
0.633182626 0.385092729 0.701346197 0.40416169
0.474908405 0.740764648 0.752324231 0.729122102
0.510976158 0.214921124 0.7778999 0.545784283 0.530168654
0.555949985 0.362990632 0.368019417
TCGA_TCGA-CJ-5686 0.336511683 0.562812423 0.712194899
0.678811952 0.642794524 0.654771402 0.257364042
0.657152598 0.504358027 0.728653986 0.476913281
0.443267988 0.841534457 0.759000432 0.806514997
0.520589033 0.190162637 0.773373146 0.646440796
0.606244355 0.532688785 0.433648671 0.444516152
TCGA_TCGA-A3-A8OV 0.145335597 0.363831073 0.589638166
0.571922531 0.645725235 0.743523447 0.148244433
0.621215884 0.450977692 0.711933538 0.377441593
0.418037976 0.750206971 0.767296315 0.726889186
0.497069553 0.256607124 0.755727771 0.584162343
0.561370594 0.495664434 0.391195853 0.333125848
TCGA_TCGA-AK-3447 0.151306312 0.38723363 0.572387519
0.554062491 0.698475724 0.751347879 0.261108512

3398		0.591901036	0.344088467	0.691936302	0.288773624
3399		0.375314829	0.569936712	0.779996479	0.569761422
3400		0.423105228	0.260307135	0.690453813	0.38142012
3401		0.457642151	0.419446106	0.40684441	0.430969524
3402	TCGA_TCGA-CJ-4876	0.322557955	0.52035408	0.730475233	
3403		0.608549826	0.630849253	0.756199091	0.287243312
3404		0.603583955	0.401421134	0.66427954	0.307560471
3405		0.344979625	0.660141927	0.732740978	0.761895689
3406		0.483765327	0.176512233	0.771462458	0.424940907
3407		0.519133034	0.497700904	0.443073977	0.399821869
3408	TCGA_TCGA-B0-4834	0.160049526	0.301123188	0.419570907	
3409		0.51754406	0.628268758	0.729666931	0.327800322
3410		0.564103935	0.279061692	0.687911423	0.229408948
3411		0.382945826	0.421882184	0.776115879	0.582929277
3412		0.386427559	0.19159767	0.769774355	0.366558564
3413		0.490169829	0.412705018	0.388492214	0.37237903
3414	TCGA_TCGA-B0-5713	0.244979113	0.453154078	0.631516554	
3415		0.60696218	0.62858603	0.740571179	0.415571725
3416		0.641326945	0.460620771	0.702752183	0.335648142
3417		0.455282759	0.707100495	0.765897726	0.772354129
3418		0.52172282	0.274204102	0.76528664	0.621182221
3419		0.562323152	0.540805026	0.420205673	0.46755126

TCGA_TCGA-CJ-5671 0.418246311 0.608576546 0.741662199
0.616829289 0.663364904 0.721356564 0.260358915
0.685480192 0.570681574 0.706049029 0.565049536
0.591700889 0.897252786 0.798242711 0.792561559
0.597729719 0.20642186 0.764612535 0.715934278
0.67147998 0.631291657 0.401717618 0.455785549
TCGA_TCGA-CZ-5454 0.279951181 0.576813135 0.659111007
0.669296911 0.679829196 0.651486051 0.336290948
0.738175411 0.549016273 0.777323748 0.544784455
0.534052135 0.877341328 0.787778381 0.788448932 0.5811719
0.248663371 0.776053307 0.789222935 0.678505062
0.571225197 0.392415559 0.420321736
TCGA_TCGA-BP-4790 0.37566279 0.522313617 0.714740754
0.646593205 0.653842002 0.706194049 0.30185389
0.65778191 0.545688897 0.735136101 0.427819514
0.522660037 0.785564324 0.777647423 0.80822316
0.564450599 0.143132119 0.770447033 0.679487051
0.636908341 0.601886904 0.39340684 0.48136757
TCGA_TCGA-B0-5107 0.250679395 0.638968928 0.744230539
0.685392885 0.633088287 0.648344213 0.134427629
0.629829069 0.476063061 0.660362491 0.409822822
0.352930525 0.784697774 0.751157425 0.760665968

0.515040044 0.103194576 0.729021762 0.555327104
0.563378493 0.518163499 0.52357609 0.33757233
TCGA_TCGA-B4-5834 0.249780273 0.435120764 0.572939348
0.555296225 0.650045717 0.674888284 0.3519463 0.628582199
0.423149987 0.699924223 0.367831387 0.493760678
0.686043288 0.78251032 0.778199863 0.525497067
0.235790969 0.751598434 0.601221635 0.563220031
0.539502548 0.35821624 0.471735735
TCGA_TCGA-G6-A8L6 0.328617934 0.460592764 0.676692463
0.583428805 0.665620637 0.736910743 0.259874715
0.646855884 0.451894715 0.746062839 0.46605804
0.49494885 0.846444062 0.827161987 0.784432913
0.517445099 0.346365743 0.783853519 0.594260225
0.598804166 0.530828006 0.409783805 0.399504418
TCGA_TCGA-BP-4991 0.189135357 0.407470715 0.536367068
0.597745719 0.62491738 0.707401936 0.313749189
0.634403373 0.46243349 0.681618229 0.407201373
0.46895969 0.734951291 0.778135188 0.787451407
0.523150128 0.292746438 0.771530902 0.626225717
0.566186136 0.53545359 0.385168581 0.470344631
TCGA_TCGA-A3-3347 0.377224106 0.592074893 0.677253757
0.701481568 0.628059692 0.672143087 0.182717326

0.655091279 0.530301224 0.751744031 0.529936504
0.478006958 0.882047116 0.722310402 0.782773149
0.573765092 0.079087524 0.77100756 0.739590108
0.663392389 0.614764441 0.440862126 0.468832847
TCGA_TCGA-B2-5636 0.354115761 0.362460121 0.439245265
0.561959215 0.607442692 0.698791120.262870101 0.621423936
0.452699837 0.68087724 0.396676238 0.495494258
0.702552894 0.784157687 0.766776776 0.50846595
0.338845116 0.775241691 0.653611437 0.582096888
0.513636894 0.37177177 0.498508072
TCGA_TCGA-B4-5844 0.205273803 0.379481361 0.534265783
0.590386408 0.656357695 0.710551466 0.36252645
0.636684749 0.38489147 0.712346431 0.384992124
0.520697663 0.718228349 0.787393846 0.779734794
0.517071885 0.33173966 0.766988062 0.546374027
0.541709304 0.529860198 0.361866554 0.458899121
TCGA_TCGA-BP-4801 0.324676043 0.450221097 0.619561837
0.629064448 0.659411467 0.668199114 0.257660448
0.673817526 0.531290269 0.755081772 0.50198901
0.696165032 0.845355742 0.768996642 0.799251175
0.571976312 0.17496737 0.77951802 0.708960443
0.652442831 0.566711994 0.387003858 0.455604366

TCGA_TCGA-A3-3307 0.217504824 0.51659036 0.647662438
0.652913527 0.644943015 0.725070879 0.35125068
0.621395384 0.480577275 0.721729498 0.419041425
0.520371343 0.806920223 0.778861643 0.805397537
0.537992638 0.269001975 0.782904378 0.622659418
0.574596573 0.535929879 0.412433082 0.422576794
TCGA_TCGA-B0-4821 0.283860544 0.483826789 0.751892277
0.634955614 0.606328554 0.813574353 0.200177601
0.625928049 0.302727898 0.633368228 0.430806611
0.318938932 0.741706348 0.758908267 0.744639651
0.486847225 0.176147459 0.748588075 0.459620298
0.564761624 0.515982996 0.387790933 0.367211897
TCGA_TCGA-B0-4813 0.18687908 0.497444767 0.536039688
0.592760206 0.5975742 0.808926094 0.369927854 0.628238055
0.358527967 0.650020398 0.357631079 0.491847477
0.784420361 0.752169518 0.711852843 0.491926488
0.212168114 0.75149216 0.539739499 0.540089882
0.508555333 0.377041297 0.492179813
TCGA_TCGA-CZ-5459 0.293733965 0.550351166 0.682559089
0.594100187 0.667797744 0.732870293 0.23573715
0.618171774 0.440345702 0.754392475 0.406988084
0.408740964 0.746924953 0.785673948 0.779664556

0.527345762 0.213084969 0.752885494 0.551066863
0.560613163 0.522555282 0.38210065 0.429352319
TCGA_TCGA-B0-5108 0.348156938 0.584520183 0.711079241
0.6827721 0.668732317 0.723763062 0.318887119 0.726811431
0.538600455 0.736467326 0.567926426 0.570943604
0.946387366 0.787979091 0.824017806 0.56833572
0.22489669 0.784699173 0.812993946 0.65431573
0.608568155 0.400395429 0.48280655
TCGA_TCGA-CZ-4862 0.437131833 0.583574971 0.798225448
0.66609321 0.620799841 0.741599531 0.263587246
0.654715077 0.585592859 0.723072695 0.45866368
0.432086778 0.849077408 0.759451122 0.824820093
0.546224858 0.137145002 0.771441421 0.674109657
0.661587097 0.589425461 0.436291112 0.450748784
TCGA_TCGA-MM-A564 0.259978509 0.374546338 0.631175797
0.504681594 0.626797803 0.768255151 0.199200618
0.631816324 0.380988515 0.715253308 0.376114712
0.438470747 0.744345681 0.795333178 0.759962041
0.496500855 0.303920665 0.790063841 0.540669135
0.541107183 0.492734779 0.327941019 0.400979858
TCGA_TCGA-CJ-4881 0.313307915 0.58520522 0.718474441
0.672503679 0.669996472 0.72183019 0.24486929

	0.667381133	0.470306095	0.732782726	0.522151872	
	0.507976321	0.866655734	0.761388964	0.788665008	
	0.54147596	0.238500777	0.777149233	0.641389315	
	0.637982613	0.555893417	0.413655045	0.455573083	
TCGA_TCGA-B0-5077	0.22093404	0.468080202	0.674369461		
	0.634008187	0.631226213	0.73374652	0.235893153	
	0.64376928	0.443319523	0.755430949	0.390981361	0.405277
	0.723554107	0.74342708	0.755112918	0.539360033	
	0.148839786	0.784487278	0.621442069	0.579090651	
	0.512218844	0.357878967	0.415499334		
TCGA_TCGA-B0-5092	0.412542344	0.639674394	0.859477824		
	0.714114704	0.641497567	0.734196427	0.190453591	
	0.61963201	0.531818333	0.689690084	0.428904414	
	0.424416329	0.845348485	0.756833151	0.793279279	
	0.535462614	0.125998061	0.754741348	0.637304838	
	0.636786083	0.590988186	0.428617198	0.436297011	
TCGA_TCGA-EU-5905	0.247853897	0.459878937	0.553673303		
	0.597297378	0.605657519	0.703256092	0.139371203	
	0.590634289	0.453527316	0.745597314	0.458816064	
	0.41670803	0.72817731	0.766468267	0.741237417	
	0.554457624	0.125575884	0.783939837	0.619681247	
	0.566180722	0.539219364	0.420789458	0.434621566	

TCGA_TCGA-BP-5168 0.251962384 0.427600826 0.626625159
0.583748293 0.620237851 0.743821743 0.336697937
0.624626282 0.427933825 0.704959478 0.368444157
0.442635444 0.676033778 0.819655669 0.771455518
0.523024766 0.260107623 0.761203722 0.589502625
0.551441045 0.532815887 0.360402169 0.44291693
TCGA_TCGA-B4-5832 0.236422171 0.633455733 0.611686088
0.589074388 0.678242745 0.573892535 0.355339086
0.693573824 0.565585091 0.66163999 0.468877071
0.464624528 0.675599918 0.673695142 0.783007767
0.537367521 0.320122082 0.712949501 0.630536034
0.594334641 0.523015104 0.475224105 0.650503414
TCGA_TCGA-B8-5163 0.362712316 0.626756148 0.778436414
0.663042601 0.651050233 0.718767911 0.285828436
0.683581821 0.569151499 0.731038424 0.513856497
0.529039873 0.905659111 0.759943655 0.790312299
0.56371373 0.2349494 0.764206276 0.74240342 0.649790438
0.627681927 0.392497491 0.550059856
TCGA_TCGA-DV-5575 0.36442536 0.541539316 0.673158599
0.612894569 0.640653282 0.73438396 0.323412449
0.64466006 0.47782983 0.701354012 0.413425433
0.501731011 0.796468343 0.742857498 0.80146831

0.547680159 0.284083562 0.760449054 0.625171234
0.609567242 0.532766246 0.38439463 0.465838135
TCGA_TCGA-CW-5580 0.243824463 0.501989266 0.487014697
0.628034259 0.634971046 0.672342027 0.315959319
0.618241694 0.479503814 0.698430012 0.428827753 0.4626743
0.751035144 0.761409466 0.805298913 0.541226858
0.273599227 0.76634994 0.643964803 0.605971894
0.531684551 0.372756963 0.447533483
TCGA_TCGA-B0-5692 0.401015549 0.558366487 0.831526314
0.661810914 0.661051235 0.689542739 0.277589539
0.63550604 0.553267336 0.715782439 0.436710564
0.408021961 0.820417483 0.785286739 0.809696141
0.538204806 0.244484253 0.762413982 0.62314683
0.616917361 0.548890887 0.441948035 0.423317551
TCGA_TCGA-CJ-4640 0.342954184 0.584212396 0.795515783
0.656661924 0.674073516 0.718366023 0.225316187
0.662229732 0.548413523 0.699070764 0.492372563
0.461332704 0.839455431 0.750808361 0.845953479
0.550868269 0.13724515 0.778949992 0.653962092
0.626195602 0.574516641 0.459671767 0.429742338
TCGA_TCGA-A3-3316 0.203602479 0.500068447 0.506944889
0.641439954 0.631060366 0.678098007 0.36968495

3596	0.661834682	0.47040412	0.701231072	0.464150103	
3597	0.520611481	0.795925773	0.740222608	0.786124491	
3598	0.558717972	0.241568342	0.782265964	0.655815795	
3599	0.623216791	0.57408281	0.406652681	0.454253515	
3600	TCGA_TCGA-BP-4797	0.336195565	0.571142587	0.670936323	
3601	0.645539737	0.625884286	0.739047159	0.281680677	
3602	0.651139732	0.510212022	0.7185136	0.463225551	0.500203283
3603	0.827638037	0.786380574	0.780998194	0.539059553	
3604	0.195989544	0.768645866	0.645208848	0.604812556	
3605	0.577936984	0.417295069	0.402671817		
3606	TCGA_TCGA-BP-5195	0.26153941	0.454883055	0.610317621	
3607	0.570403252	0.649776055	0.715073045	0.340968096	
3608	0.63901607	0.488510742	0.74702056	0.418626762	
3609	0.489225337	0.783060843	0.778059808	0.796996028	
3610	0.540471495	0.283851965	0.776521405	0.662097173	
3611	0.593152111	0.569340421	0.406800433	0.440828408	
3612	TCGA_TCGA-B0-5691	0.391149479	0.462517846	0.547815432	
3613	0.585066434	0.631662125	0.703425365	0.316452214	
3614	0.60191465	0.464284309	0.736904451	0.383695773	
3615	0.534621869	0.754480861	0.761414988	0.781684392	
3616	0.526955585	0.225317738	0.75684848	0.639101964	
3617	0.565299912	0.534289807	0.391420747	0.452061204	

TCGA_TCGA-BP-5006	0.219333442	0.469942632	0.547788373
0.636677041	0.632448928	0.752020534	0.361869502
0.637659916	0.464713568	0.70028324	0.404299246
0.495328175	0.762252696	0.774341274	0.789794101
0.542570636	0.32150178	0.766761171	0.586700469
0.589537807	0.537564672	0.416439197	0.430315408
TCGA_TCGA-CZ-5469	0.206201645	0.501021072	0.579992805
0.602536758	0.638155454	0.716359012	0.088186702
0.645011179	0.398181711	0.663765244	0.462816448
0.375393463	0.738366662	0.776062148	0.756550788
0.543226657	0.067881838	0.764154278	0.532401253
0.586990688	0.522117604	0.404243765	0.418251504
TCGA_TCGA-BP-4999	0.210909644	0.408703306	0.500970562
0.586364315	0.596600103	0.682180271	0.377247504
0.602558968	0.440750073	0.702596989	0.366326118
0.54206872	0.709515908	0.759675902	0.764399299
0.519311377	0.247667937	0.768656338	0.620775363
0.549903533	0.505927085	0.330846135	0.491606884
TCGA_TCGA-EU-5904	0.275367523	0.516946155	0.570925235
0.611352696	0.643829765	0.710927713	0.400497634
0.632525038	0.441150278	0.701678806	0.378490811
0.576057625	0.769749893	0.779499297	0.785415027

0.536470108 0.330972535 0.768062831 0.609442862
0.570473064 0.538263273 0.387797657 0.454456751
TCGA_TCGA-B2-3924 0.379170278 0.620769199 0.758495459
0.673970099 0.631428829 0.716740908 0.30106036
0.627466772 0.589250649 0.697830802 0.407657273
0.48704294 0.801080447 0.715190318 0.792583101
0.508492872 0.24452425 0.760474085 0.689006648
0.639264031 0.552843396 0.407974704 0.493896394
TCGA_TCGA-BP-5189 0.284139237 0.52548377 0.569081924
0.668957463 0.631048647 0.678036693 0.332617447
0.635519733 0.503066178 0.737042011 0.481365833
0.496728562 0.834586035 0.77162475 0.81594175
0.538055016 0.263469761 0.792991825 0.666402589
0.588284447 0.548007435 0.419622702 0.445185247
TCGA_TCGA-B0-5812 0.329781768 0.423623205 0.634360213
0.606126402 0.657857255 0.691304332 0.327508745
0.627621170.478505852 0.726392872 0.386617514 0.516267251
0.784711503 0.777156998 0.790190735 0.559029107
0.232059029 0.775339745 0.622595553 0.576574119
0.568848256 0.377927353 0.479471442
TCGA_TCGA-A3-3385 0.11192849 0.365476192 0.450320110.624256556
0.599038536 0.704284297 0.169187681 0.61256748

3662 0.427209583 0.694136133 0.313209661 0.381854221
3663 0.639350497 0.72424458 0.721973678 0.465152335
3664 0.225495962 0.747041065 0.519827682 0.532180706
3665 0.486283422 0.410114599 0.362965033
3666 TCGA_TCGA-BP-4787 0.230306409 0.560011387 0.624874805
3667 0.658174027 0.638454754 0.706667437 0.297063778
3668 0.718973411 0.502868845 0.7143287 0.528528924 0.537287006
3669 0.876224916 0.755933169 0.774428725 0.553584281
3670 0.317813535 0.769534884 0.731258224 0.650573443
3671 0.587458832 0.451107186 0.468091109
3672 TCGA_TCGA-BP-4325 0.244652367 0.494661106 0.726119763
3673 0.635279465 0.626311883 0.700403909 0.311237048
3674 0.618772283 0.528455592 0.688560632 0.367391735
3675 0.417962797 0.781515271 0.722212304 0.777312521
3676 0.493351811 0.231815382 0.758229814 0.618155831
3677 0.608061231 0.530963375 0.424176473 0.391020763
3678 TCGA_TCGA-A3-3322 0.203625038 0.407682133 0.443345122
3679 0.603361419 0.617794552 0.741649665 0.248419892
3680 0.604439141 0.467429491 0.723716287 0.362358678
3681 0.464235268 0.71510889 0.73049501 0.757743052
3682 0.521241073 0.199061853 0.774885308 0.624463854
3683 0.58977663 0.516456233 0.371400541 0.409662341

TCGA_TCGA-BP-5194 0.312826443 0.450641419 0.596213616
0.585791154 0.646298443 0.716900166 0.307611355
0.668293085 0.478938086 0.69810616 0.407708937
0.504283614 0.704495633 0.770414947 0.780312562
0.517027122 0.26176106 0.783751574 0.621595677
0.585117331 0.531359413 0.412605923 0.453098085
TCGA_TCGA-B0-5096 0.324839561 0.57382694 0.565990464
0.652480383 0.623906107 0.678593522 0.310566567
0.695349186 0.525337688 0.733689867 0.496349714
0.502328199 0.845347423 0.783868611 0.750928345
0.556743894 0.177953848 0.760876512 0.761470415
0.638170524 0.587760308 0.44613341 0.471989096
TCGA_TCGA-CZ-5986 0.208875742 0.492844333 0.519050811
0.601457038 0.634087455 0.674137208 0.416223611
0.619608167 0.4943327 0.715420993 0.360957442 0.552385015
0.696154101 0.725943525 0.79190705 0.534265977
0.181895396 0.772619962 0.638592868 0.621255819
0.493201374 0.395345062 0.466426018
TCGA_TCGA-B8-4151 0.18732932 0.396947716 0.53179979
0.592899151 0.647313113 0.727523979 0.320029567
0.663237099 0.436238752 0.703740989 0.379492287
0.408542564 0.679576821 0.75756779 0.730377962

0.514314097 0.253399604 0.74683565 0.544727667

0.560234669 0.497739528 0.396817409 0.380717257

TCGA_TCGA-BP-4982 0.272700301 0.448376657 0.588871828

0.638188716 0.617385752 0.704337942 0.337447894

0.633665213 0.503117365 0.696404662 0.40546188

0.515841498 0.768038635 0.754420694 0.815029002

0.536559552 0.268688082 0.770031728 0.662506619

0.584627239 0.537539881 0.391276414 0.468533592

TCGA_TCGA-B0-4713 0.324282772 0.500705046 0.755998711

0.610766428 0.640653566 0.721984946 0.096842405

0.630862896 0.411678030.692445295 0.374619679 0.313719539

0.758165913 0.745619524 0.763371205 0.49351648

0.205434738 0.730391691 0.509729389 0.518727493

0.525641187 0.412019885 0.391909356

TCGA_TCGA-CJ-4902 0.384130004 0.494355231 0.719643085

0.619862538 0.651715891 0.704515753 0.258255705

0.698859669 0.482077558 0.713466123 0.48637042

0.515163253 0.824468631 0.791091904 0.791497328

0.567792045 0.186480421 0.794800767 0.651698853

0.601288144 0.558620499 0.377826419 0.43603816

TCGA_TCGA-MM-A84U 0.336186353 0.390539017 0.676186968

0.656542445 0.61944381 0.765928852 0.164860976

3728		0.646699492	0.420623567	0.749156255	0.485923132
3729		0.425416105	0.823814822	0.77583853	0.79053869
3730		0.524375659	0.221423785	0.760966034	0.572175674
3731		0.58931752	0.549332347	0.391447171	0.342269794
3732	TCGA_TCGA-B0-4816	0.241309379	0.46754269	0.654441734	
3733		0.664519872	0.644968657	0.701159748	0.316368199
3734		0.631263812	0.516789347	0.77356304	0.520102494
3735		0.498388828	0.906193824	0.799124573	0.853820145
3736		0.550683877	0.272588564	0.777613382	0.679343458
3737		0.651538561	0.571229424	0.394191067	0.41796804
3738	TCGA_TCGA-A3-3311	0.309994068	0.494647666	0.638848638	
3739		0.643529522	0.648533707	0.693787204	0.273726822
3740		0.621540069	0.507864297	0.704978769	0.471012476
3741		0.479131863	0.795814209	0.758143852	0.810599657
3742		0.52709197	0.226439117	0.773965587	0.638977374
3743		0.599322291	0.539714676	0.432945319	0.429878235
3744	TCGA_TCGA-CZ-4866	0.231963787	0.459350811	0.512618027	
3745		0.647727958	0.617217403	0.698074484	0.159623946
3746		0.689570738	0.478134793	0.695169527	0.442772321
3747		0.391453808	0.751716997	0.753463524	0.704199184
3748		0.482153812	0.12159872	0.761235043	0.610264649
3749		0.573122182	0.515561711	0.389378629	0.337825396

TCGA_TCGA-BP-5182 0.39884712 0.527965678 0.713009966
0.633854148 0.656058053 0.754067868 0.284240562
0.691798524 0.548319648 0.743149028 0.545493195
0.620562523 0.894434544 0.759725835 0.785548424
0.568382447 0.093873404 0.759548773 0.720096209
0.660652086 0.613587656 0.382154091 0.419995933
TCGA_TCGA-CJ-6033 0.231029361 0.491105733 0.643205421
0.583785579 0.64090041 0.703052883 0.225782782
0.67252615 0.417889444 0.714683474 0.43055537
0.53416622 0.8042547 0.777784152 0.774491653 0.535682212
0.2681386 0.764873922 0.626176486 0.584595426 0.559668914
0.414083998 0.495702269
TCGA_TCGA-CJ-4637 0.486829495 0.71639276 0.865588497
0.698764512 0.639298311 0.683558926 0.245003491
0.635135898 0.664331337 0.719954967 0.522602715
0.472948297 0.910627415 0.726607245 0.819179741
0.583972661 0.169840416 0.778118913 0.789652557
0.686939666 0.631535053 0.417113595 0.495463462
TCGA_TCGA-CJ-4885 0.223558414 0.421902127 0.599030148
0.598832039 0.64452664 0.713433657 0.233886732
0.668719477 0.406419802 0.74399073 0.352524272
0.406221170.688601159 0.757379713 0.742418721 0.514534823

0.185691377 0.768270206 0.532149881 0.591075419
0.512032389 0.411034491 0.393026559
TCGA_TCGA-B8-5546 0.202935633 0.327265314 0.485476729
0.521068973 0.658138932 0.641647661 0.194125097
0.579047648 0.346068181 0.758078434 0.300644421
0.263807964 0.53710205 0.801132528 0.630032458
0.470900499 0.144131845 0.756416007 0.344811970.509351044
0.478222498 0.378933868 0.40265883
TCGA_TCGA-B8-5545 0.360677466 0.443206983 0.591104394
0.595373994 0.635590444 0.736689713 0.268863532
0.667662388 0.501814658 0.739081071 0.518305568
0.543936803 0.812901042 0.796980185 0.813772018
0.555149933 0.264894158 0.768528296 0.689468074
0.626140385 0.565637402 0.38975688 0.463747268
TCGA_TCGA-CJ-5676 0.385820175 0.540900608 0.733676781
0.616888912 0.68497379 0.722643064 0.294401293
0.668089507 0.475855502 0.669135976 0.490550993
0.529449491 0.833623368 0.792767043 0.820811533
0.575565019 0.25519394 0.758385876 0.600221171
0.604322009 0.554802682 0.427522582 0.443794793
TCGA_TCGA-B0-5707 0.320631946 0.361219169 0.530931129
0.631144243 0.635163742 0.699798258 0.190496078

3794		0.622083147	0.41501699	0.740450126	0.468483042
3795		0.474451134	0.741436667	0.789026797	0.726978971
3796		0.513106696	0.137748353	0.756017435	0.471975269
3797		0.56558389	0.497003517	0.484835745	0.366379691
3798	TCGA_TCGA-BP-5190	0.257551912	0.481344262	0.634594975	
3799		0.619305503	0.640227523	0.681397599	0.127271925
3800		0.638253363	0.429133502	0.694681609	0.413664264
3801		0.390159573	0.689279316	0.753350214	0.764731538
3802		0.505051193	0.176532559	0.759100726	0.496743186
3803		0.537605613	0.483639211	0.452227685	0.359678509
3804	TCGA_TCGA-B0-5709	0.50199773	0.578801381	0.746606891	
3805		0.66451906	0.621426843	0.719685792	0.32055616
3806		0.645331958	0.590769516	0.732389486	0.520109092
3807		0.569502736	0.881778604	0.767526184	0.816768288
3808		0.570009494	0.239612975	0.750762254	0.730953005
3809		0.648296393	0.628661252	0.400583016	0.455453493
3810	TCGA_TCGA-B0-5109	0.482344035	0.715906911	0.815797936	
3811		0.673798499	0.674224521	0.795262167	0.173600641
3812		0.646625467	0.492653056	0.740951535	0.48192493
3813		0.520704539	0.854534966	0.750414191	0.767685712
3814		0.53366627	0.261423149	0.744804862	0.660504581
3815		0.594430535	0.614481597	0.356353953	0.551321899

TCGA_TCGA-B4-5843 0.189139399 0.41517087 0.464879541
0.598814409 0.64028708 0.688142848 0.250589698
0.645840292 0.420125059 0.751501886 0.361160048
0.433578728 0.653825217 0.738826882 0.776791952
0.519576006 0.254880446 0.772934983 0.576446449
0.552905868 0.492080641 0.42604463 0.464524367
TCGA_TCGA-B2-4098 0.379547052 0.476281393 0.537814133
0.696088818 0.628197984 0.691851062 0.146475989
0.698068885 0.506292432 0.753078153 0.536785121
0.449117820.862411783 0.737828347 0.688205687 0.502072643
0.127046662 0.774782319 0.669685216 0.608558343
0.517880871 0.415451186 0.296468148
TCGA_TCGA-CJ-4893 0.305982271 0.527404571 0.759762546
0.64441734 0.663768011 0.670181077 0.278875388
0.637711118 0.537091081 0.717860642 0.420339591
0.487724026 0.805576411 0.784550419 0.794661162
0.518128241 0.216632375 0.784331365 0.66176289
0.591448806 0.552475027 0.397643808 0.468521934
TCGA_TCGA-BP-4985 0.211256618 0.60004432 0.577250765
0.636203838 0.649245765 0.713484363 0.310120538
0.690860177 0.429570018 0.700429841 0.413009328
0.485645603 0.782467179 0.761792887 0.77593428

0.55240284 0.208745916 0.75752321 0.703805772
0.583685037 0.574381746 0.386977787 0.465615449
TCGA_TCGA-BP-5001 0.287860349 0.433300807 0.740833551
0.590743816 0.606334147 0.773417241 0.289083296
0.605063876 0.385508033 0.678942418 0.312717471
0.375627667 0.703428821 0.763125849 0.737758528
0.515041399 0.251016588 0.737700228 0.456626635
0.528293359 0.513098759 0.396099616 0.371535607
TCGA_TCGA-A3-3313 0.285274736 0.371683398 0.453122688
0.580660416 0.628614846 0.709989312 0.334300735
0.59367439 0.382595175 0.700289948 0.368573971
0.39927804 0.614988625 0.726018816 0.619448007
0.463915974 0.037283945 0.778871797 0.452103823
0.529506713 0.452344243 0.311224739 0.395307541
TCGA_TCGA-BP-4169 0.411318227 0.647265232 0.661860801
0.669115417 0.636961659 0.706453118 0.43317944
0.681811840.534019921 0.768825733 0.492189833 0.67312318
0.903823537 0.746815271 0.789821001 0.567671343
0.390415868 0.789282912 0.733793491 0.636758397
0.601729805 0.348071079 0.504169612
TCGA_TCGA-B8-5553 0.348655558 0.551283241 0.660280706
0.645235247 0.61865728 0.691835466 0.262798305

3860	0.626175584	0.520779081	0.699638208	0.433311944	
3861	0.52478178	0.830016494	0.778444904	0.810398287	
3862	0.532750437	0.288458871	0.760228862	0.676679818	
3863	0.599348207	0.545770887	0.475822597	0.454026408	
3864	TCGA_TCGA-AK-3443	0.128429441	0.261908678	0.41964448	
3865	0.490173289	0.672662094	0.631456499	0.273744496	
3866	0.543824153	0.282340769	0.697786608	0.185113262	
3867	0.292607502	0.349193314	0.763248818	0.602352879	
3868	0.352815487	0.163782164	0.721607106	0.310731534	
3869	0.436210428	0.361373846	0.395098983	0.363503251	
3870	TCGA_TCGA-B0-4696	0.077098213	0.527118310.417907849		
3871	0.555706521	0.614196991	0.698435881	0.183086836	
3872	0.648546402	0.339013303	0.630157647	0.420393136	
3873	0.372786257	0.617558752	0.658423617	0.624623847	
3874	0.470373776	0.036015131	0.715324426	0.502196755	
3875	0.52744057	0.387258866	0.300431183	0.481712752	
3876	TCGA_TCGA-DV-5576	0.189466863	0.27901422	0.365366351	
3877	0.495809688	0.611692290.7288268	0.289615005	0.558004322	
3878	0.282307503	0.714818101	0.295212163	0.391928514	
3879	0.553064317	0.779292589	0.706385143	0.458102868	
3880	0.234038405	0.77015076	0.538008982	0.489690841	
3881	0.477712271	0.388502833	0.509884671		

TCGA_TCGA-BP-4987 0.25997181 0.387023244 0.645238612
0.570627148 0.641088637 0.74197852 0.194254121
0.618065925 0.487377578 0.686644249 0.358249454
0.448200488 0.738168383 0.75195715 0.779426785
0.512680663 0.277861741 0.764553986 0.627326902
0.569146759 0.526431589 0.379175846 0.403976819
TCGA_TCGA-BP-4769 0.208423027 0.361845947 0.387566651
0.547187415 0.652674006 0.728834258 0.377459269
0.575894606 0.329096701 0.711509380.380994812 0.458978893
0.537725456 0.752337407 0.723894386 0.491563801
0.253770688 0.77186018 0.538055172 0.516326212
0.523849379 0.354082644 0.508303634
TCGA_TCGA-BP-4161 0.362568915 0.576267103 0.788252281
0.674115620.643568539 0.689644606 0.314188391 0.674784454
0.560009153 0.730365791 0.484745659 0.456211920.844017357
0.747129322 0.843594195 0.533406511 0.221595924
0.786424783 0.69997741 0.64748342 0.566889697
0.394804936 0.395766178
TCGA_TCGA-BP-5010 0.304589531 0.462737249 0.684646896
0.607964066 0.609326173 0.76550841 0.312082342
0.617672947 0.388160955 0.703420893 0.395349554
0.440828623 0.756304273 0.765623071 0.808953597

3904		0.552903763	0.278915657	0.770200355	0.543963564
3905		0.531098049	0.563611416	0.37721077	0.424048355
3906	TCGA_TCGA-AK-3445	0.228391399	0.507575567	0.733222577	
3907		0.632437447	0.637709276	0.752073725	0.196185738
3908		0.679442927	0.496416298	0.751651226	0.42944056
3909		0.426888126	0.819879214	0.756377565	0.754764856
3910		0.546601455	0.124071506	0.788623609	0.644132866
3911		0.609520008	0.535716216	0.382226418	0.495709579
3912	TCGA_TCGA-B2-4102	0.322908063	0.459703248	0.595808521	
3913		0.665879312	0.618943906	0.67207716	0.221223762
3914		0.626344388	0.545978578	0.736139608	0.455231757
3915		0.482407653	0.833021483	0.756466771	0.821086018
3916		0.525564843	0.252521073	0.769009914	0.670580086
3917		0.611235198	0.540292952	0.472983389	0.412845978
3918	TCGA_TCGA-B0-5083	0.360638112	0.432931967	0.671413466	
3919		0.621568608	0.687171691	0.733286854	0.275037304
3920		0.588053833	0.523802216	0.767131632	0.468372768
3921		0.497011407	0.82450541	0.806601167	0.702595882
3922		0.510680994	0.205306562	0.790333333	0.649818941
3923		0.593646422	0.517312229	0.401868309	0.416289092
3924	TCGA_TCGA-B0-4697	0.800524773	0.732381507	0.86105592	
3925		0.704421603	0.628951888	0.726870457	0.245301187

0.665393792 0.696334771 0.694001954 0.531748428
0.459847304 0.936228851 0.787679379 0.792473875
0.579469002 0.220970052 0.778685731 0.665622377
0.655188758 0.603732691 0.388440125 0.45933122
TCGA_TCGA-DV-A4VZ 0.219724936 0.328521011 0.385727072
0.610478682 0.614265372 0.755776993 0.235497546
0.601207468 0.290937049 0.687794827 0.360737838
0.384819205 0.577419293 0.777009135 0.708684746
0.476387564 0.20891234 0.808187246 0.495133844
0.515401466 0.49102201 0.38281096 0.469857516
TCGA_TCGA-BP-4963 0.270220273 0.504118482 0.625241788
0.635108721 0.636295756 0.767736624 0.233348628
0.645719562 0.509403246 0.732778549 0.459817946
0.479809638 0.84682474 0.76046953 0.808578068
0.532486179 0.278903456 0.781879665 0.695155538
0.623491901 0.551090867 0.421164298 0.422964117
TCGA_TCGA-BP-4354 0.336618738 0.693758958 0.690202222
0.777897008 0.640455473 0.659884851 0.286520658
0.770754869 0.554040017 0.739499781 0.68369484
0.589745318 0.968468251 0.7788981 0.849052825 0.583904597
0.410172974 0.792446382 0.869239395 0.702900793
0.639205312 0.415683296 0.507253726

TCGA_TCGA-BP-4341 0.213769692 0.367267947 0.549225927
0.538809971 0.626080531 0.766451444 0.363398341
0.645969043 0.346663791 0.700816778 0.385444043
0.509503815 0.700807098 0.75328541 0.77121209
0.527478988 0.35293105 0.762482155 0.502354373
0.53953657 0.478301649 0.391934467 0.386699346
TCGA_TCGA-B0-5099 0.183263967 0.318851222 0.46240558
0.54091089 0.610512798 0.746643095 0.341931449
0.606785919 0.288055669 0.66010052 0.297642865
0.440604463 0.559239485 0.736904102 0.722556687
0.525753889 0.197810933 0.789352457 0.456625076
0.480683212 0.450610536 0.404060826 0.451746851
TCGA_TCGA-B0-4839 0.320890496 0.430007619 0.697990021
0.613919584 0.688455141 0.742344216 0.218516449
0.680608693 0.478861814 0.736186969 0.482059566
0.494292301 0.861662144 0.813563169 0.781195287
0.558314726 0.281668719 0.768846981 0.620044825
0.606146833 0.53884585 0.411392125 0.41361792
TCGA_TCGA-CJ-5678 0.42289736 0.628259235 0.854908586
0.656764519 0.675118171 0.691169964 0.247677463
0.686445381 0.562048177 0.729762251 0.484735582
0.514578812 0.890454096 0.782006867 0.80172062

	0.536798569	0.244412998	0.763218752	0.623720939
	0.655627474	0.583493149	0.431903737	0.44242958
TCGA_TCGA-A3-3308	0.272349226	0.507248678	0.525510539	
	0.595392982	0.621784452	0.711908316	0.223899984
	0.606236554	0.439804662	0.673063962	0.435229137
	0.445815202	0.747708792	0.744498227	0.73436028
	0.534183853	0.117028201	0.748308092	0.608016725
	0.572467104	0.539696972	0.361097642	0.441779552
TCGA_TCGA-B0-5120	0.303590799	0.471734637	0.566759845	
	0.584503034	0.623626506	0.683782651	0.351894754
	0.637888601	0.474485173	0.721975008	0.459735929
	0.618909711	0.796315129	0.738427993	0.765354256
	0.530895903	0.258337269	0.782012014	0.658983787
	0.639737993	0.556602695	0.376634002	0.475180507
TCGA_TCGA-B0-5100	0.287852261	0.326167191	0.553801401	
	0.553109703	0.655429438	0.721528779	0.235063916
	0.570294017	0.314869429	0.678963561	0.413865349
	0.443601213	0.697568932	0.768363169	0.741816172
	0.504394326	0.212603583	0.73249918	0.489321831
	0.518624409	0.51076709	0.375847521	0.522792597
TCGA_TCGA-B0-4945	0.276595042	0.428851493	0.542337148	
	0.598614502	0.631713191	0.715235337	0.381462889

3992	0.630999061	0.416066528	0.68956164	0.326157487	
3993	0.438118425	0.674691439	0.750222843	0.748552932	
3994	0.535150477	0.13628669	0.785167797	0.554805631	
3995	0.557050233	0.521902226	0.432303274	0.488714131	
3996	TCGA_TCGA-BP-4781	0.249455824	0.504752962	0.503435492	
3997	0.602339273	0.631146158	0.692759043	0.341617532	
3998	0.66132754	0.472531412	0.763197134	0.450795585	
3999	0.544284382	0.768775031	0.736952576	0.764510315	
4000	0.560997816	0.150294282	0.756331394	0.658787056	
4001	0.629596966	0.556874341	0.386533787	0.429230928	
4002	TCGA_TCGA-CJ-5684	0.321338055	0.45895772	0.741090538	
4003	0.597674811	0.665859281	0.734228209	0.2676461	0.660857165
4004	0.472280884	0.730201216	0.369328988	0.479269317	
4005	0.750145413	0.771735337	0.787320601	0.515778912	
4006	0.238627997	0.757143241	0.577230219	0.574552823	
4007	0.543067951	0.418340685	0.444413027		
4008	TCGA_TCGA-BP-4346	0.451213911	0.59924759	0.84880815	
4009	0.676336129	0.618396354	0.756273839	0.276954973	
4010	0.606527761	0.59608609	0.712565512	0.464229761	
4011	0.43896706	0.863988216	0.734571212	0.827477827	
4012	0.544016409	0.099895053	0.774386153	0.684917077	
4013	0.649611365	0.596172767	0.396702891	0.413396241	

TCGA_TCGA-AK-3426 0.47232417 0.68062755 0.888082185
0.692206053 0.649813134 0.73595165 0.227343094
0.662049031 0.579894972 0.733173822 0.605926479
0.592057567 1 0.800373461 0.798722511 0.584964299
0.251640245 0.759568835 0.741116234 0.706122002
0.638909831 0.437363338 0.456778117
TCGA_TCGA-CJ-5682 0.331643819 0.43218271 0.674124315
0.584338755 0.654159401 0.692188913 0.207000494
0.600698684 0.432862862 0.696694768 0.410557169
0.416158525 0.736244773 0.780453677 0.790903121
0.541693638 0.187033983 0.769098467 0.544895007
0.561384878 0.54913313 0.401244977 0.422327694
TCGA_TCGA-A3-A6NL 0.319969204 0.441338533 0.65992968
0.583957967 0.623901129 0.697149644 0.279213139
0.642634576 0.483065136 0.738924833 0.424175899
0.47314242 0.833637616 0.805937987 0.790201469
0.528678365 0.258690523 0.786360618 0.669856011
0.594019387 0.543691418 0.385370143 0.45240354
TCGA_TCGA-CZ-5987 0.321368505 0.577590461 0.69540781
0.639809806 0.626324291 0.721151176 0.301217286
0.615038468 0.459730803 0.731434507 0.455446916
0.399956701 0.829023273 0.764316278 0.799921432

0.514020646 0.23288803 0.757446881 0.589544075

0.603435152 0.54024975 0.415865031 0.448399896

TCGA_TCGA-A3-3382 0.250871826 0.573723295 0.681183082

0.622457291 0.617197997 0.668295344 0.174682383

0.647709825 0.473581016 0.713921787 0.399505779

0.406876883 0.804577639 0.727744404 0.727843669

0.547235196 0.188063768 0.787617674 0.634244828

0.606257946 0.550426445 0.411230236 0.459808873

TCGA_TCGA-CJ-4636 0.352752985 0.569994071 0.743467501

0.682744853 0.654986744 0.73488598 0.177993882

0.656892707 0.541605596 0.72499741 0.509578185

0.506760615 0.894682446 0.763472978 0.785191437

0.57305023 0.148320784 0.772623715 0.736300221

0.658337733 0.578725802 0.390112450.417668105

TCGA_TCGA-CW-5583 0.301892183 0.343763477 0.514103109

0.543363616 0.619394166 0.699289136 0.305571673

0.578919876 0.423874299 0.697578821 0.312141339

0.453796571 0.668793467 0.752095724 0.763999062

0.521054175 0.280474728 0.757652676 0.599694229

0.53351321 0.480433988 0.365655745 0.444949123

TCGA_TCGA-B0-4849 0.235444706 0.462845636 0.610927319

0.658909666 0.635644511 0.758416332 0.383611473

0.604711208 0.451444544 0.726233243 0.40780533
0.510411639 0.829182933 0.745608276 0.80292207
0.506553439 0.26269662 0.776438288 0.614212637
0.570832601 0.515753042 0.391468736 0.4252309
TCGA_TCGA-B0-4852 0.295731303 0.545771501 0.606409742
0.630355552 0.635174578 0.696680301 0.383970091
0.621778977 0.474090273 0.723575438 0.372944395
0.516561283 0.755647581 0.737741416 0.76684653
0.52068596 0.306833073 0.795924826 0.540906675
0.562811913 0.522053159 0.402714189 0.449036177
TCGA_TCGA-B8-4621 0.2703666 0.416863796 0.543443751
0.651348569 0.675232439 0.665217107 0.150369503
0.666169365 0.4659116 0.815793061 0.490236003 0.50010648
0.786372964 0.789709629 0.749051485 0.492372244
0.17725763 0.753110405 0.566618931 0.602551147
0.508544207 0.460605044 0.362361877
TCGA_TCGA-BP-4807 0.226527331 0.425320992 0.498688486
0.595067072 0.643772674 0.71601377 0.274408613
0.683900967 0.479293471 0.767585261 0.46267747
0.547873934 0.803613689 0.750718025 0.789568549
0.553399326 0.211919613 0.756726821 0.685750827
0.619002421 0.539116502 0.402462273 0.429671869

TCGA_TCGA-CJ-4908 0.32186264 0.463502365 0.60422636
0.622553074 0.63071062 0.710815026 0.348873156
0.657369142 0.458194978 0.697371387 0.396598928
0.518394843 0.740901073 0.780346918 0.783192622
0.547890888 0.229160899 0.775065186 0.608414445
0.572851415 0.550687594 0.363863257 0.446469634
TCGA_TCGA-BP-4352 0.205713643 0.483606378 0.496555681
0.60949497 0.579872098 0.735894099 0.108864976
0.598382633 0.289485988 0.700616858 0.351232361
0.254658478 0.638506355 0.709272084 0.651361528
0.531507577 0.034532773 0.710630283 0.444540057
0.517067782 0.480422636 0.442501467 0.403805128
TCGA_TCGA-A3-A8OU 0.343921112 0.400119887 0.631965277
0.625677892 0.588071942 0.737997674 0.322164657
0.607351131 0.45060348 0.717570204 0.411821102
0.492837254 0.79562373 0.760549974 0.786163914
0.520891726 0.278620427 0.77851623 0.598430082
0.593302369 0.520688134 0.385788053 0.430747922
TCGA_TCGA-AK-3434 0.264070888 0.503418422 0.730972244
0.585468856 0.657886457 0.740571736 0.256702675
0.646176723 0.447588135 0.683010014 0.364078752
0.454633148 0.737053575 0.763098612 0.79264601

0.535471355 0.263841965 0.768537063 0.549510023
0.593634434 0.542884568 0.430914169 0.433679263
TCGA_TCGA-CJ-5683 0.183597963 0.501264964 0.602983773
0.619227531 0.66139073 0.704703096 0.287234068
0.671863709 0.40987066 0.740360404 0.389406649
0.393612967 0.709725747 0.777340422 0.772035985
0.559526529 0.282831386 0.762380067 0.55332548
0.552488021 0.494773484 0.395735491 0.445936005
TCGA_TCGA-A3-3326 0.356755194 0.530570662 0.679780437
0.678236305 0.629010111 0.737089167 0.258183202
0.626300887 0.558556635 0.719930731 0.46413374
0.532364275 0.85650217 0.7477834 0.78364966 0.529084418
0.258026237 0.787143676 0.660127563 0.651337108
0.554978611 0.393231278 0.430917386
TCGA_TCGA-BP-4353 0.167501406 0.57288632 0.692885331
0.608333219 0.651962313 0.704841018 0.25468485
0.685839573 0.532153138 0.742088372 0.508588694
0.551164654 0.815143595 0.76812721 0.764032733
0.569977826 0.212754371 0.783692909 0.685823292
0.620996476 0.592544364 0.364935241 0.523783627
TCGA_TCGA-GK-A6C7 0.306235338 0.433346427 0.593954261
0.604317682 0.640633119 0.716979266 0.384203387

0.603722476 0.435053629 0.681855338 0.340688414
0.500221227 0.740951281 0.784563751 0.778682485
0.508832514 0.186695496 0.737913876 0.58839308
0.558179041 0.50649134 0.386494971 0.448802523
TCGA_TCGA-BP-4761 0.409840522 0.518959419 0.726179983
0.602409048 0.626899582 0.70958122 0.167617464
0.597721118 0.407589364 0.657725675 0.436852902
0.377621241 0.80148872 0.751220156 0.714251885
0.482737096 0.228471025 0.728635522 0.544538715
0.606894994 0.529720758 0.436204358 0.414531935
TCGA_TCGA-A3-3329 0.232654345 0.504239224 0.670491443
0.647991293 0.647975586 0.705969009 0.302919821
0.609382408 0.500368622 0.731473102 0.47765747
0.452734437 0.782209186 0.772263624 0.778034567
0.502808946 0.206492704 0.7773582 0.623295725 0.612311119
0.536867538 0.420754865 0.405255925
TCGA_TCGA-A3-3374 0.343547852 0.439846307 0.567773608
0.66795818 0.703175434 0.700330588 0.352912708
0.664685741 0.569743288 0.794660019 0.537353308
0.596726968 0.848554514 0.809356047 0.65388706
0.490111627 0.25194367 0.757375104 0.661780738
0.613930417 0.515505693 0.405737964 0.396159901

TCGA_TCGA-CZ-5470 0.327453498 0.569896712 0.7678374
0.663944563 0.655591243 0.739271787 0.273480229
0.676770184 0.52399977 0.703709016 0.492279184
0.488229287 0.858828162 0.780487622 0.810580801
0.509440354 0.15347552 0.767418098 0.621916568
0.643796734 0.566709953 0.412177793 0.415373021
TCGA_TCGA-CZ-5463 0.246337596 0.483423403 0.626520426
0.577687763 0.646747274 0.676461203 0.319841206
0.623350676 0.505453782 0.755510669 0.377663249
0.414932939 0.699476987 0.743628096 0.761245161
0.51060269 0.176181874 0.78274161 0.611175311
0.591833824 0.510981501 0.420140742 0.441886056
TCGA_TCGA-BP-4965 0.216746283 0.444604116 0.535568867
0.607258922 0.621353891 0.697667163 0.202580254
0.65230463 0.47264557 0.688724017 0.424994128
0.485366646 0.72847332 0.761397441 0.793562563
0.529592005 0.232780011 0.761295986 0.642756092
0.574352651 0.514093885 0.42259859 0.491844314
TCGA_TCGA-B4-5835 0.310900436 0.557392829 0.629713135
0.646005874 0.651126656 0.689655414 0.200771248
0.670022818 0.450114036 0.7091877 0.454958783 0.406772009
0.726254965 0.724530681 0.657185864 0.526847343

0.085730439 0.734813091 0.478007063 0.542284756
0.534244343 0.419681156 0.377618784
TCGA_TCGA-B0-5094 0.22761512 0.443199286 0.607988714
0.60171921 0.605514766 0.692559355 0.154922639
0.626022054 0.412070828 0.639144392 0.36870521
0.384531804 0.706506737 0.696465313 0.733501309
0.505468547 0.252804582 0.765789663 0.595213161
0.562559085 0.511050724 0.420146054 0.407623865
TCGA_TCGA-DV-5567 0.165623197 0.270815663 0.400359207
0.536786969 0.630289715 0.686802495 0.384449802
0.588138579 0.296315989 0.721346537 0.31428779
0.437322782 0.564116408 0.776416465 0.709621308
0.415098262 0.211191441 0.75331474 0.594107951
0.511623501 0.496495194 0.400505376 0.513577471
TCGA_TCGA-BP-4334 0.214085403 0.361266631 0.453691526
0.548679778 0.689683716 0.693786369 0.396908092
0.545701231 0.325220248 0.682452477 0.335810559
0.394438645 0.616076721 0.775177775 0.624728165
0.407794211 0.233699318 0.720042844 0.358336279
0.46371218 0.405617835 0.446842501 0.344527126
TCGA_TCGA-BP-5187 0.312329662 0.550320451 0.623802586
0.593260469 0.601422789 0.688628429 0.327142203

0.630337805 0.442660345 0.733304488 0.354371559
0.421993443 0.655941501 0.742530797 0.770327678
0.544622798 0.25863932 0.768596148 0.580766 0.541958525
0.540990561 0.395624087 0.498824135
TCGA_TCGA-CZ-5988 0.258131707 0.52690503 0.622319661
0.622522317 0.634911240.718663255 0.273412276 0.654960774
0.427576647 0.752304146 0.440291715 0.460229611
0.713546385 0.745988196 0.80777076 0.549622855
0.267903877 0.769162162 0.663477458 0.589353098
0.538625423 0.395472864 0.492064434
TCGA_TCGA-CJ-4894 0.368075657 0.496225166 0.753640381
0.654089233 0.62825661 0.705901063 0.232179221
0.629832867 0.541825764 0.689920374 0.381488075
0.412129833 0.800194609 0.743623348 0.829454766
0.523415796 0.169384209 0.770676055 0.651202322
0.61735688 0.551116528 0.422146168 0.44512843
TCGA_TCGA-BP-4763 0.260592294 0.458873674 0.590805733
0.58343632 0.633229446 0.669772803 0.267277018
0.633795072 0.482911270.775392733 0.364279381 0.507303691
0.678070326 0.743913399 0.765381553 0.523435332
0.220769581 0.772942818 0.563183655 0.554395881
0.528634716 0.368246186 0.525620372

TCGA_TCGA-B0-4828 0.248986108 0.484149346 0.670352614
0.584649628 0.616711239 0.723082514 0.264488837
0.573727788 0.396666722 0.696659409 0.321420158
0.449076308 0.756709471 0.757399485 0.759332996
0.476199849 0.207201301 0.766283576 0.49634079
0.533124608 0.496649293 0.430132612 0.378070346
TCGA_TCGA-CJ-5679 0.149445941 0.425663856 0.574183276
0.580211275 0.658986785 0.753240925 0.133369643
0.66727462 0.306577583 0.703280725 0.410138434
0.303137024 0.65474616 0.765575621 0.738526046
0.543283276 0.207065511 0.749458163 0.521552621
0.526930391 0.509155037 0.411424512 0.452234724
TCGA_TCGA-CJ-4869 0.427155255 0.676620925 0.876632183
0.657025305 0.663854201 0.73640752 0.227712121
0.661873526 0.577769263 0.672538659 0.486005236
0.487403691 0.875324971 0.784866283 0.803046112
0.549767917 0.123538779 0.752524231 0.635487492
0.642214275 0.616130788 0.401354912 0.442715286
TCGA_TCGA-A3-3363 0.11138071 0.339778142 0.522096893
0.63037948 0.644426958 0.693451551 0.121934407
0.629489864 0.461153972 0.798369253 0.473234853
0.538579666 0.752426641 0.788533323 0.727655701

4234	0.507903874	0.123180987	0.747952362	0.490199278
4235	0.602436924	0.50145709	0.438667913	0.355777611
4236	TCGA_TCGA-BP-4777	0.471019695	0.703251152	0.834629384
4237	0.664323914	0.652367848	0.726555802	0.368520371
4238	0.696635195	0.582916885	0.750904104	0.472487455
4239	0.534690743	0.885111121	0.759720313	0.803444552
4240	0.557045508	0.162285385	0.763454781	0.666321924
4241	0.645974261	0.581850095	0.415252924	0.494308767
4242	TCGA_TCGA-B8-5158	0.272689237	0.607780982	0.681104864
4243	0.680819658	0.636879153	0.675284484	0.168210794
4244	0.657939604	0.521650292	0.733892137	0.488073569
4245	0.477179163	0.866438475	0.744413036	0.807146599
4246	0.546022042	0.248391129	0.76802124	0.751249108
4247	0.624632126	0.562659918	0.435267139	0.482949791
4248	TCGA_TCGA-B0-5695	0.289065448	0.453983274	0.594092331
4249	0.571713555	0.649741663	0.731801413	0.380628145
4250	0.623659303	0.410080447	0.707848996	0.338047009
4251	0.471840334	0.692847413	0.772990815	0.775666084
4252	0.511245691	0.282765772	0.770006007	0.540178782
4253	0.559773387	0.503790781	0.402211519	0.430094206
4254	TCGA_TCGA-BP-5176	0.271749305	0.40156687	0.67035321
4255	0.608860171	0.649568795	0.747661751	0.296545658

0.662688844 0.425307252 0.71591767 0.402628304

0.425458492 0.720643384 0.780285855 0.790058363

0.537402662 0.322871628 0.753621268 0.560748217

0.547426463 0.515388291 0.3851543 0.430303844

TCGA_TCGA-BP-4993 0.280910747 0.471242655 0.68589573

0.60327997 0.670285991 0.736541069 0.312739366

0.632746813 0.446912809 0.729548266 0.385920948

0.502841683 0.757772952 0.772871739 0.795160161

0.569649579 0.266082646 0.764978799 0.588780862

0.573210151 0.557350363 0.38225855 0.443895542

TCGA_TCGA-BP-4795 0.415513712 0.39280399 0.577950012

0.598617766 0.654020933 0.701659231 0.297613157

0.635399216 0.479148951 0.731304226 0.444115065

0.599575934 0.763934284 0.785124355 0.735910791

0.527450894 0.201998765 0.773192277 0.702138804

0.579369662 0.579420695 0.361411940.518456479

TCGA_TCGA-CW-5588 0.333632427 0.517439818 0.596789657

0.6124106 0.635622044 0.711374776 0.221100843 0.685144757

0.483952981 0.746234936 0.476645537 0.541332203

0.811334747 0.760062498 0.780172046 0.555137416

0.213774926 0.751825065 0.67303323 0.622458304

0.557414571 0.420405489 0.468353813

TCGA_TCGA-CZ-5464 0.389146908 0.585067376 0.748402636
0.669860338 0.653402492 0.719017349 0.218957851
0.647961158 0.531371578 0.716270016 0.463425313
0.482686444 0.850704152 0.77882214 0.807043827
0.556511029 0.190456449 0.771030846 0.688728061
0.625499198 0.585182866 0.422927585 0.455848604
TCGA_TCGA-BP-4762 0.407458393 0.479690291 0.654531477
0.618778677 0.644350282 0.72267018 0.292293935
0.66573842 0.504377152 0.744501846 0.479622472
0.564711144 0.807285459 0.769569927 0.794479785
0.563455698 0.220648508 0.757245618 0.665224489
0.607797048 0.585034709 0.390432341 0.4821986
TCGA_TCGA-BP-5178 0.411659786 0.635601237 0.727631845
0.665440134 0.657809257 0.715519327 0.200212355
0.709006052 0.512398232 0.690666229 0.511253089
0.460449956 0.915698591 0.796255098 0.794000516
0.56621442 0.29273227 0.768270041 0.660916534
0.591072308 0.557028865 0.435974386 0.445268114
TCGA_TCGA-B0-5115 0.277644219 0.463058509 0.60277881
0.622542722 0.655900932 0.706872259 0.352187992
0.604422545 0.480837209 0.729740825 0.414542531
0.446696451 0.736228709 0.739941131 0.793328358

4300 0.50848763 0.166711159 0.782105556 0.622000009

4301 0.588424931 0.535636126 0.396524179 0.465049317

4302 TCGA_TCGA-B8-4622 0.283521339 0.465301936 0.640291738

4303 0.61045578 0.652521263 0.747368181 0.238812567

4304 0.645177619 0.499018761 0.72175671 0.45356893

4305 0.483431828 0.832051949 0.779655513 0.810165961

4306 0.527018088 0.233856096 0.787205057 0.63735673

4307 0.609373572 0.544510735 0.415425992 0.403199248

4308 TCGA_TCGA-B0-5102 0.27460322 0.453126328 0.659570845

4309 0.585007951 0.602233476 0.708888854 0.239479166

4310 0.669921809 0.442330495 0.703226586 0.396407439

4311 0.38528849 0.75885839 0.724401618 0.736232112

4312 0.502423694 0.115971731 0.78467419 0.553567752

4313 0.596605307 0.491275096 0.430460685 0.385055749

4314 TCGA_TCGA-BP-5008 0.359436717 0.587548344 0.698107975

4315 0.641280821 0.635826108 0.704847307 0.384852878

4316 0.642769734 0.512821565 0.748324474 0.432072377

4317 0.623286802 0.821803892 0.755794701 0.793343885

4318 0.572515681 0.259276513 0.761356355 0.640527662

4319 0.600412864 0.5864849 0.369931638 0.484884106

4320 TCGA_TCGA-CJ-4642 0.357021195 0.524086591 0.604691826

4321 0.563161383 0.617639394 0.73519255 0.323045256

4322		0.560721719	0.368978395	0.757711457	0.468793803
4323		0.440980482	0.691769347	0.736011389	0.795637145
4324		0.533026709	0.24225058	0.756919371	0.41838578
4325		0.508834677	0.480963826	0.401046597	0.48880871
4326	TCGA_TCGA-A3-3362	0.23084967	0.439275054	0.533850584	
4327		0.575549455	0.625656264	0.737224636	0.221882261
4328		0.633383734	0.429886053	0.744512726	0.37721793
4329		0.446246372	0.747311685	0.764985786	0.765965833
4330		0.523972918	0.305261264	0.759965966	0.572788473
4331		0.550090439	0.503255811	0.378619112	0.426044274
4332	TCGA_TCGA-B2-5639	0.322986297	0.49919779	0.638604362	
4333		0.655092781	0.645783992	0.648504073	0.293248114
4334		0.627567709	0.516195043	0.737904299	0.474095992
4335		0.447412436	0.810589098	0.791227565	0.826735874
4336		0.524393177	0.192006906	0.746682875	0.627018756
4337		0.620030923	0.537523505	0.457123929	0.410588809
4338	TCGA_TCGA-CZ-5458	0.301401159	0.523430459	0.657397298	
4339		0.609575764	0.636588692	0.665449945	0.237501868
4340		0.601454317	0.452648666	0.711067806	0.446537988
4341		0.499467852	0.783875584	0.806473034	0.799025406
4342		0.524356476	0.203834865	0.766229296	0.595422681
4343		0.586109947	0.533955622	0.438022082	0.442961821

TCGA_TCGA-AS-3778 0.203380417 0.410031003 0.534116167
0.584671575 0.626886923 0.735505688 0.224301190.618421925
0.435613906 0.694661372 0.386647007 0.455084231
0.760395579 0.751925105 0.785466064 0.527975803
0.227910745 0.76120065 0.573915467 0.572390501
0.510908467 0.393216408 0.387860665
TCGA_TCGA-B0-4693 0.323078919 0.548469224 0.719491732
0.646461749 0.635775151 0.72648917 0.354769767 0.6133237
0.521711596 0.730893393 0.423052431 0.461482098
0.840007529 0.753433972 0.778788082 0.536595264
0.307950068 0.769834721 0.625112218 0.585490158
0.538468548 0.384251691 0.448097579
TCGA_TCGA-CJ-4892 0.324406204 0.491555361 0.806578183
0.633480881 0.649261568 0.734951155 0.264240091
0.617419538 0.450771949 0.71046284 0.339433704
0.425944712 0.8154622 0.746712945 0.783565775 0.533808001
0.211435665 0.760801672 0.596194613 0.58802895
0.547148553 0.421210223 0.419489102
TCGA_TCGA-B0-5712 0.183072068 0.438755192 0.634148687
0.610953455 0.657796432 0.702283526 0.314370325
0.599134569 0.38207859 0.706288954 0.340834371
0.356638624 0.640440217 0.762740493 0.752589075

0.460774659 0.178961594 0.760431282 0.478641854
0.551265068 0.47621328 0.417250168 0.43921216
TCGA_TCGA-B0-4706 0.234447723 0.474347889 0.719187629
0.559008963 0.618456222 0.7396823 0.232496249 0.610994226
0.348222668 0.717516658 0.315486732 0.394740714
0.722048076 0.762418359 0.770878541 0.470986425
0.212820412 0.765528976 0.533201911 0.553768873
0.529010016 0.412712964 0.39272365
TCGA_TCGA-CZ-5461 0.265380205 0.49451183 0.626544592
0.624324911 0.651339274 0.720313497 0.333199986
0.626369843 0.499129116 0.737130027 0.453680243
0.511978906 0.792053084 0.783636579 0.797367407
0.51840665 0.240458724 0.760250849 0.673347912
0.64762694 0.562518705 0.39020941 0.459272541
TCGA_TCGA-6D-AA2E 0.307476505 0.4003565 0.589573952
0.599810111 0.619860409 0.678603261 0.332840728
0.569229878 0.38112323 0.707541254 0.428020849 0.425779406
0.704143327 0.772515737 0.685934392 0.46919988
0.054449286 0.782184682 0.513393385 0.564310978
0.505756719 0.305659585 0.336494226
TCGA_TCGA-EU-5907 0.170275656 0.478493169 0.494147474
0.584208584 0.621527294 0.650538243 0.247092564

4388		0.607522987	0.466494752	0.659315483	0.375180104
4389		0.367533451	0.674124249	0.727301102	0.724767652
4390		0.536580151	0.202017162	0.747901205	0.556735281
4391		0.571627246	0.504308998	0.463090363	0.401594531
4392	TCGA_TCGA-BP-4770	0.224865924	0.552621584	0.621725803	
4393		0.603225188	0.670123531	0.711023767	0.23149066
4394		0.639040622	0.453160723	0.709032537	0.488853877
4395		0.455455877	0.782935874	0.758928293	0.703470978
4396		0.59758783	0.119934464	0.755250546	0.581206118
4397		0.579653651	0.537755355	0.303611245	0.556479034
4398	TCGA_TCGA-B0-4848	0.329569723	0.539458934	0.684099659	
4399		0.649556328	0.655364831	0.678888774	0.171766916
4400		0.660613626	0.495042442	0.735981639	0.476752444
4401		0.474415235	0.860420181	0.755780953	0.811850206
4402		0.554492479	0.192628129	0.773243461	0.663093741
4403		0.615902376	0.562723373	0.384048172	0.437312494
4404	TCGA_TCGA-CZ-5468	0.148746736	0.545441469	0.660096514	
4405		0.635216495	0.630529122	0.762231975	0.192363944
4406		0.698360997	0.403838594	0.67928547	0.443092709
4407		0.425667759	0.796513039	0.763699086	0.702082212
4408		0.588378297	0.260575798	0.771864043	0.595837536
4409		0.612329546	0.529519052	0.394745259	0.530303292

TCGA_TCGA-CJ-4904 0.265530561 0.476066126 0.638666762
0.618247963 0.662936619 0.715211540.323416494 0.649325352
0.47876731 0.713944321 0.389838426 0.504086295
0.77568543 0.786983528 0.792013241 0.538791271
0.259331504 0.772124521 0.638269996 0.579473761
0.545650641 0.392039883 0.469476527
TCGA_TCGA-B0-5710 0.310666741 0.47184243 0.626521764
0.621498138 0.662781594 0.668353244 0.335871026
0.652701728 0.458836191 0.688795243 0.411486865
0.492455017 0.727363696 0.775995631 0.761194168
0.557142195 0.280368751 0.776911003 0.610118155
0.573138493 0.539830456 0.417113189 0.484147303
GSE29609_GSM733579 0.289279891 0.424571474 0.563389618
0.642097101 0.669445015 0.725640073 0.280244829
0.603357349 0.501634606 0.765635575 0.377713868
0.491289539 0.739102329 0.777980274 0.739032727
0.551950998 0.179397632 0.761481869 0.563684267
0.592065471 0.536017094 0.430536119 0.405785864
GSE29609_GSM733580 0.256824666 0.403466905 0.500124199
0.601416367 0.638280772 0.721831534 0.245090312
0.669207251 0.419474964 0.676602637 0.377989332
0.424399687 0.737341338 0.745012889 0.736167983

	0.532841241	0.257310387	0.78259663	0.573446518
	0.537968474	0.496938631	0.353782896	0.463972102
GSE29609_GSM733581	0.297626542	0.610531104	0.829955827	
	0.673238278	0.663895116	0.741812155	0.203576175
	0.665371588	0.474923488	0.723117765	0.477663494
	0.454778737	0.839108619	0.768566185	0.791808171
	0.535107338	0.24539756	0.776807758	0.659919819
	0.611410753	0.570143666	0.404654324	0.40245718
GSE29609_GSM733582	0.318766215	0.450101583	0.691060037	
	0.631216988	0.630215175	0.721391687	0.165442452
	0.585500641	0.496054572	0.754755094	0.316208935
	0.421862667	0.716816411	0.775707506	0.757556211
	0.505138526	0.13106076	0.78175502	0.604172953
	0.581470158	0.529203555	0.432890513	0.384608788
GSE29609_GSM733583	0.309173211	0.489254211	0.557467159	
	0.551280355	0.650267775	0.732438379	0.197486734
	0.644934872	0.389568973	0.735723654	0.423007463
	0.440401613	0.76855761	0.751346248	0.747608224
	0.517106464	0.163298219	0.776627637	0.606104795
	0.572662781	0.512791203	0.378552781	0.43322992
GSE29609_GSM733584	0.298142969	0.469726687	0.504713762	
	0.621572974	0.666118598	0.653792803	0.190590803

4454	0.652731761	0.497236473	0.694362765	0.427072912
4455	0.437001854	0.81019406	0.788684815	0.791256202
4456	0.529821932	0.227523123	0.77260335	0.629020408
4457	0.545494451	0.506607659	0.409761556	0.434574615
4458	GSE29609_GSM733585	0.249026966	0.346425997	0.49715349
4459	0.576829061	0.63165718	0.732062496	0.251901457
4460	0.600731452	0.423791188	0.733825594	0.327714514
4461	0.406180846	0.643294272	0.746857909	0.757969783
4462	0.527172517	0.227870241	0.776676877	0.469333876
4463	0.538127241	0.513600715	0.375717836	0.410744234
4464	GSE29609_GSM733586	0.175409389	0.393863753	0.562145814
4465	0.58154621	0.640003418	0.734641728	0.348171273
4466	0.666371581	0.450130846	0.728125147	0.388006078
4467	0.428483166	0.729573101	0.778437627	0.769335742
4468	0.527906249	0.228224647	0.773481013	0.574241155
4469	0.593452588	0.515248117	0.335405783	0.456979259
4470	GSE29609_GSM733587	0.346375915	0.499901122	0.718935309
4471	0.61506795	0.615666806	0.737135976	0.205798996
4472	0.693347152	0.455661799	0.705255803	0.407824477
4473	0.446118725	0.814935094	0.790814815	0.763050885
4474	0.549967608	0.211455512	0.782331684	0.642529627
4475	0.566000082	0.536707059	0.375817574	0.477551865

GSE29609_GSM733588 0.371873391 0.559695073 0.741031307
0.608030457 0.622703125 0.76709228 0.171162826
0.634543784 0.466227188 0.685021725 0.400413504
0.434903929 0.798875738 0.788743679 0.760119935
0.493144516 0.171326404 0.782293019 0.603814331
0.56676169 0.551845868 0.361402124 0.436715844
GSE29609_GSM733589 0.297066003 0.4430188 0.540610131
0.582296474 0.635718168 0.719503504 0.314780381
0.605767448 0.462892793 0.685684405 0.403845591
0.477823319 0.759485101 0.773156638 0.715414271
0.526546485 0.2470844 0.751551633 0.565648351 0.558649654
0.515492035 0.360928962 0.443190798
GSE29609_GSM733590 0.298103922 0.598544547 0.71930526
0.637170254 0.618943159 0.72576137 0.21736848
0.665770393 0.458143477 0.712890644 0.483691294
0.426682469 0.82586958 0.777567366 0.794582074
0.529587852 0.174715087 0.772915926 0.654205421
0.570721142 0.567389524 0.395627841 0.451926102
GSE29609_GSM733591 0.356605377 0.538411724 0.68845693
0.672524848 0.622077689 0.700993539 0.275216877
0.633066488 0.518938845 0.738508848 0.459087593
0.461444829 0.769201977 0.766757894 0.774078316

0.54774459 0.158895424 0.788267413 0.611219467
0.575898226 0.552061221 0.441855669 0.399542501
GSE29609_GSM733592 0.329368139 0.512112377 0.73791851
0.656982548 0.654724072 0.699415728 0.374866899
0.674454808 0.548063567 0.744131856 0.496810528
0.524070522 0.831899363 0.749435473 0.797842669
0.575737405 0.266649239 0.768562177 0.689084737
0.663932767 0.573818526 0.434308669 0.449409678
GSE29609_GSM733593 0.351496978 0.552237305 0.671759094
0.671317553 0.636972567 0.76548945 0.401327366
0.677507925 0.524282362 0.746648351 0.4770137 0.546794006
0.89247557 0.801223931 0.779881444 0.515114532
0.248164913 0.769556235 0.708289354 0.655242779
0.56727087 0.436420765 0.433121904
GSE29609_GSM733594 0.328533939 0.536463455 0.747047046
0.631235395 0.682113360.746959892 0.306496769 0.647602214
0.495894686 0.690725977 0.433123129 0.524973418
0.860322452 0.783560607 0.767967984 0.524786972
0.289075589 0.753627603 0.580982892 0.561501794
0.541475798 0.426067071 0.442590084
GSE29609_GSM733595 0.319818005 0.533693269 0.737180738
0.624699352 0.627928531 0.76302694 0.304857959

0.657958757 0.473344787 0.735377284 0.382397888
0.507684597 0.827524558 0.764508645 0.729749703
0.555172287 0.25339812 0.758133355 0.58379551
0.557843156 0.545095052 0.382701706 0.433670671
GSE29609_GSM733596 0.294418959 0.554638882 0.689336604
0.645467457 0.658321135 0.742808014 0.271049516
0.659474675 0.468827508 0.72281426 0.474829208
0.447647711 0.817415631 0.771721461 0.779003748
0.528870138 0.221996746 0.775596647 0.618506612
0.587193441 0.55351761 0.383655941 0.434127196
GSE29609_GSM733597 0.351795364 0.465507322 0.645667706
0.617437505 0.656626448 0.694462703 0.317983337
0.652389411 0.480229551 0.7069009 0.439567452 0.551908992
0.805420612 0.767038939 0.820556735 0.536331355
0.297250179 0.760639064 0.627159959 0.620800134
0.550588865 0.359677 0.458384059
GSE29609_GSM733598 0.129896476 0.492560399 0.55935211
0.593599201 0.620096782 0.731771701 0.295834783
0.606911315 0.411793696 0.698815325 0.377796915
0.401049452 0.672084177 0.723990789 0.762766414
0.531750718 0.227017342 0.747366812 0.527780852
0.557673016 0.512308373 0.42520975 0.426363326

GSE29609_GSM733599 0.316990835 0.502609722 0.650969021
0.668626452 0.649419547 0.722410813 0.286203818
0.639264099 0.536496194 0.759124991 0.449081586
0.458443677 0.738674813 0.796899708 0.768119801
0.574152634 0.170984972 0.776748557 0.595300271
0.588662329 0.561448822 0.438291346 0.386641352
GSE29609_GSM733600 0.318357427 0.55759202 0.709159244
0.624815102 0.646145831 0.725056576 0.397498772
0.672939674 0.449258514 0.74788063 0.41948154
0.47853871 0.780637788 0.794074506 0.791913596
0.503055639 0.231023413 0.79903777 0.61250386
0.601586646 0.544377546 0.344660084 0.494078774
GSE29609_GSM733601 0.152063512 0.453308578 0.55548664
0.592770046 0.654110661 0.762429755 0.317685429
0.639408118 0.400269008 0.718812966 0.446771824
0.478053814 0.774659297 0.7564566 0.77422463 0.516640115
0.149370068 0.767935339 0.590787494 0.563354841
0.534169168 0.356787734 0.431566986
GSE29609_GSM733602 0.337782906 0.610872581 0.751374414
0.689848103 0.612705528 0.714843717 0.338986343
0.675312528 0.538855796 0.710350053 0.494644173
0.546097314 0.884153538 0.756566138 0.782495555

0.579031827 0.207993983 0.774334322 0.711046111
0.622277382 0.582879953 0.396955919 0.466744806
GSE29609_GSM733603 0.335513388 0.536802703 0.716481549
0.652437045 0.636128279 0.729304357 0.268398102
0.643303837 0.46622143 0.70796663 0.436743168
0.495516849 0.828704627 0.772841347 0.784993892
0.543776382 0.267881651 0.759421242 0.585671043
0.612209732 0.536238814 0.392949248 0.422484462
GSE29609_GSM733604 0.354231901 0.707361279 0.752905678
0.585712605 0.643121231 0.674177691 0.331137770.65822305
0.482567132 0.69916482 0.334709073 0.495313033
0.769867743 0.75408168 0.719038026 0.536987378
0.231980501 0.771782838 0.563648875 0.595741032
0.553724599 0.410349299 0.471289583
GSE29609_GSM733605 0.188317681 0.424896336 0.580715331
0.594873082 0.625046579 0.722742182 0.28860033
0.639654309 0.448265451 0.725029367 0.40160075
0.453751254 0.740258712 0.763466474 0.768568176
0.516378951 0.162707813 0.778102301 0.611534254
0.580697179 0.505228802 0.405159335 0.41602317
GSE29609_GSM733606 0.285869557 0.609894448 0.729390556
0.644948709 0.647971905 0.687451914 0.27560101

0.68959812 0.52234039 0.758599998 0.500379337
0.515224853 0.88575079 0.778335794 0.806399313
0.554250667 0.191881773 0.768451911 0.681202488
0.623451009 0.607432013 0.43957866 0.465778952
GSE29609_GSM733607 0.27345498 0.534682126 0.691861538
0.646701244 0.659983548 0.76568557 0.278337404
0.621028434 0.41391083 0.711194799 0.340145515
0.346666203 0.696648677 0.7656276 0.792115331 0.481293584
0.06308477 0.779314415 0.531450214 0.517232238
0.520652733 0.439180984 0.411206715
GSE29609_GSM733608 0.346005886 0.613947055 0.772296244
0.664485971 0.630063171 0.772839517 0.316554615
0.643419217 0.554290721 0.731671766 0.474325444
0.437873589 0.839926686 0.772828932 0.818803871
0.539266297 0.23064948 0.783420327 0.694210516
0.645868854 0.593870657 0.442802665 0.447207569
GSE29609_GSM733609 0.259047948 0.459614155 0.535363774
0.581832195 0.645127438 0.75129285 0.245952881
0.662239014 0.441529824 0.721147315 0.477780094
0.465176965 0.7485132 0.786377332 0.772780893 0.548518606
0.144707453 0.812544972 0.633478765 0.591837631
0.539345876 0.397541567 0.447945057

GSE29609_GSM733610 0.255891414 0.42665232 0.51717565
0.616219157 0.626866553 0.721589525 0.28366753
0.613343824 0.472225047 0.736926579 0.379315027
0.485690385 0.701858646 0.752823331 0.729968532 0.5542832
0.208020815 0.781774054 0.563142369 0.566469516
0.528355847 0.367391542 0.425937463
GSE29609_GSM733611 0.294959887 0.448035445 0.689326888
0.593937943 0.644157604 0.714600109 0.336100043
0.63318805 0.510211158 0.665188871 0.367362465
0.459569514 0.750225145 0.748158542 0.759981929
0.49888505 0.180786908 0.747753364 0.587072304
0.589033628 0.533431993 0.39541249 0.434421504
GSE29609_GSM733612 0.367374622 0.568246902 0.794825194
0.638884758 0.663082521 0.67621329 0.357187728
0.671107560.516020965 0.737482301 0.454469626 0.47255822
0.855201425 0.758278342 0.773450136 0.562115773
0.314643698 0.762243924 0.699063509 0.627306373
0.552182675 0.368899772 0.501832298
GSE29609_GSM733613 0.324780771 0.551555215 0.670440289
0.670780731 0.63729913 0.677026572 0.23975839
0.655661029 0.572992262 0.736443882 0.508476878
0.466204448 0.816712471 0.797830608 0.780174978

0.537498268 0.19987442 0.77135041 0.618064691
0.594507659 0.565571678 0.45019143 0.392698212
GSE29609_GSM733614 0.294075385 0.494581872 0.550167528
0.595511897 0.597507407 0.764984733 0.287060692
0.631762078 0.437126657 0.683796027 0.372143255
0.456145097 0.77592498 0.761299132 0.755255316
0.471981114 0.249631949 0.742704604 0.550372339
0.541722869 0.509882238 0.386873454 0.460918005
GSE29609_GSM733615 0.356628894 0.511069754 0.760420867
0.613245602 0.655716635 0.691014846 0.315252808
0.654287683 0.510977304 0.706280841 0.373504398
0.487740626 0.79758183 0.75137027 0.825528389
0.552668761 0.234830424 0.771834371 0.590272177
0.610711903 0.560142342 0.364327586 0.503745964
GSE29609_GSM733616 0.399394136 0.523269787 0.627117299
0.672625959 0.623507408 0.694792793 0.243699673
0.638225159 0.541201045 0.748221565 0.434322021
0.46101706 0.741225362 0.750615069 0.784064412
0.559532484 0.207830285 0.767172302 0.633250485
0.582693617 0.550213018 0.440124752 0.389335532
GSE29609_GSM733617 0.323127132 0.484052422 0.689423528
0.614325635 0.643747925 0.717692498 0.185686556

0.615890488 0.515674606 0.734108988 0.439488574

0.474248354 0.7667838 0.768268372 0.789864745 0.535660529

0.215400114 0.76671955 0.643345544 0.616183012

0.516057848 0.428082403 0.375147214

Table-S4: The activation states of biological pathways in distinct m6A

modification patterns by GSVA enrichment analysis

id TCGA_TCGA-CZ-4859 TCGA_TCGA-CW-5587 TCGA_TCGA-BP-4332 TCGA_TCGA-CJ-4643 TCGA_TCGA-B0-4817 TCGA_TCGA-BP-4964 TCGA_TCGA-B0-5402 TCGA_TCGA-CJ-4891 TCGA_TCGA-B0-4690 TCGA_TCGA-B0-4815 TCGA_TCGA-B2-5635 TCGA_TCGA-A3-3328 TCGA_TCGA-CJ-4872 TCGA_TCGA-B8-A54K TCGA_TCGA-CJ-4873 TCGA_TCGA-G6-A8L7 TCGA_TCGA-CZ-5467 TCGA_TCGA-B2-5641 TCGA_TCGA-CJ-4882 TCGA_TCGA-B8-A54G TCGA_TCGA-BP-4961 TCGA_TCGA-B0-5698 TCGA_TCGA-BP-5199 TCGA_TCGA-CW-6093 TCGA_TCGA-B0-4700 TCGA_TCGA-AK-3460 TCGA_TCGA-AK-3458 TCGA_TCGA-CZ-5984 TCGA_TCGA-A3-3324 TCGA_TCGA-CJ-4912 TCGA_TCGA-CZ-5453 TCGA_TCGA-A3-3378 TCGA_TCGA-A3-3343 TCGA_TCGA-CJ-4887 TCGA_TCGA-DV-5574 TCGA_TCGA-CJ-4920 TCGA_TCGA-BP-5177 TCGA_TCGA-B0-4842 TCGA_TCGA-BP-4355 TCGA_TCGA-A3-3359 TCGA_TCGA-AK-3433 TCGA_TCGA-B0-4827 TCGA_TCGA-B2-A4SR TCGA_TCGA-CJ-4874 TCGA_TCGA-B0-4699 TCGA_TCGA-B8-5550 TCGA_TCGA-A3-A8OW TCGA_TCGA-B2-3923 TCGA_TCGA-BP-4967 TCGA_TCGA-BP-5201 TCGA_TCGA-AK-3440 TCGA_TCGA-A3-3365 TCGA_TCGA-DV-5569 TCGA_TCGA-BP-4974 TCGA_TCGA-CW-5584 TCGA_TCGA-B4-5838 TCGA_TCGA-AK-3454 TCGA_TCGA-BP-4782 TCGA_TCGA-CZ-5451 TCGA_TCGA-CJ-4907 TCGA_TCGA-BP-5196 TCGA_TCGA-CJ-4889 TCGA_TCGA-AK-

3428 TCGA_TCGA-A3-A8CQ TCGA_TCGA-A3-3380 TCGA_TCGA-A3-
3323 TCGA_TCGA-CJ-4895 TCGA_TCGA-CW-5591 TCGA_TCGA-BP-
4799 TCGA_TCGA-B0-4847 TCGA_TCGA-CW-6087 TCGA_TCGA-BP-
4173 TCGA_TCGA-B8-4619 TCGA_TCGA-BP-4176 TCGA_TCGA-BP-
4768 TCGA_TCGA-B8-A54I TCGA_TCGA-CJ-4884 TCGA_TCGA-BP-
4164 TCGA_TCGA-CZ-5466 TCGA_TCGA-AK-3450 TCGA_TCGA-BP-
4327 TCGA_TCGA-BP-5200 TCGA_TCGA-BP-4160 TCGA_TCGA-BP-
5170 TCGA_TCGA-CJ-6027 TCGA_TCGA-BP-4983 TCGA_TCGA-B4-
5378 TCGA_TCGA-A3-3317 TCGA_TCGA-BP-4771 TCGA_TCGA-A3-
3352 TCGA_TCGA-B0-5084 TCGA_TCGA-BP-4159 TCGA_TCGA-B8-
A54J TCGA_TCGA-CJ-4644 TCGA_TCGA-CZ-4863 TCGA_TCGA-BP-
4330 TCGA_TCGA-BP-4995 TCGA_TCGA-BP-4798 TCGA_TCGA-B0-
5700 TCGA_TCGA-AK-3456 TCGA_TCGA-CJ-4641 TCGA_TCGA-BP-
4335 TCGA_TCGA-B8-4146 TCGA_TCGA-B0-4694 TCGA_TCGA-B0-
4836 TCGA_TCGA-CJ-4635 TCGA_TCGA-BP-4975 TCGA_TCGA-B8-
A54E TCGA_TCGA-A3-3376 TCGA_TCGA-BP-4758 TCGA_TCGA-B0-
5121 TCGA_TCGA-BP-5180 TCGA_TCGA-DV-5566 TCGA_TCGA-BP-
4784 TCGA_TCGA-A3-3331 TCGA_TCGA-CZ-5455 TCGA_TCGA-A3-
3383 TCGA_TCGA-CJ-4875 TCGA_TCGA-B0-4811 TCGA_TCGA-T7-A92I
TCGA_TCGA-B0-5085 TCGA_TCGA-CZ-4853 TCGA_TCGA-A3-A6NI
TCGA_TCGA-AK-3465 TCGA_TCGA-B2-4101 TCGA_TCGA-BP-5185
TCGA_TCGA-AK-3427 TCGA_TCGA-BP-4998 TCGA_TCGA-BP-5202

TCGA_TCGA-B8-5164 TCGA_TCGA-DV-5573 TCGA_TCGA-BP-4989 TCGA_TCGA-G6-A8L8 TCGA_TCGA-BP-4162 TCGA_TCGA-BP-5173 TCGA_TCGA-B8-A8YJ TCGA_TCGA-B0-5080 TCGA_TCGA-BP-4976 TCGA_TCGA-BP-4163 TCGA_TCGA-CJ-4899 TCGA_TCGA-BP-4977 TCGA_TCGA-A3-3335 TCGA_TCGA-CJ-4634 TCGA_TCGA-A3-3325 TCGA_TCGA-CW-6090 TCGA_TCGA-BP-4177 TCGA_TCGA-BP-4170 TCGA_TCGA-A3-3357 TCGA_TCGA-BP-4960 TCGA_TCGA-CJ-5677 TCGA_TCGA-BP-4167 TCGA_TCGA-CW-6097 TCGA_TCGA-B8-5162 TCGA_TCGA-CJ-6031 TCGA_TCGA-CJ-4639 TCGA_TCGA-BP-4759 TCGA_TCGA-BP-4986 TCGA_TCGA-B0-5075 TCGA_TCGA-BP-4775 TCGA_TCGA-CJ-4888 TCGA_TCGA-AK-3431 TCGA_TCGA-BP-4338 TCGA_TCGA-B0-4701 TCGA_TCGA-B0-4718 TCGA_TCGA-B0-5696 TCGA_TCGA-BP-4326 TCGA_TCGA-CZ-4856 TCGA_TCGA-BP-4972 TCGA_TCGA-BP-5169 TCGA_TCGA-B2-5633 TCGA_TCGA-B0-5693 TCGA_TCGA-BP-4969 TCGA_TCGA-BP-4165 TCGA_TCGA-CJ-4870 TCGA_TCGA-B8-5552 TCGA_TCGA-BP-4760 TCGA_TCGA-B0-5113 TCGA_TCGA-BP-4340 TCGA_TCGA-B8-A7U6 TCGA_TCGA-B8-5551 TCGA_TCGA-B0-5400 TCGA_TCGA-BP-4994 TCGA_TCGA-B0-4824 TCGA_TCGA-CJ-4871 TCGA_TCGA-B0-5690 TCGA_TCGA-B8-5549 TCGA_TCGA-DV-A4W0 TCGA_TCGA-CZ-5989 TCGA_TCGA-B0-5081 TCGA_TCGA-A3-3373 TCGA_TCGA-CZ-4860 TCGA_TCGA-AK-3451 TCGA_TCGA-B0-5104 TCGA_TCGA-BP-4351 TCGA_TCGA-B2-

4099 TCGA_TCGA-B0-5694 TCGA_TCGA-CJ-4903 TCGA_TCGA-CZ-5457 TCGA_TCGA-BP-4158 TCGA_TCGA-A3-3320 TCGA_TCGA-DV-5568 TCGA_TCGA-CZ-5985 TCGA_TCGA-BP-4166 TCGA_TCGA-AS-3777 TCGA_TCGA-CZ-5456 TCGA_TCGA-BP-5183 TCGA_TCGA-CZ-4854 TCGA_TCGA-A3-3306 TCGA_TCGA-BP-4789 TCGA_TCGA-CZ-5462 TCGA_TCGA-B0-5705 TCGA_TCGA-B0-4710 TCGA_TCGA-BP-5004 TCGA_TCGA-A3-3387 TCGA_TCGA-BP-5175 TCGA_TCGA-BP-4331 TCGA_TCGA-BP-4174 TCGA_TCGA-CJ-6028 TCGA_TCGA-A3-3367 TCGA_TCGA-A3-3351 TCGA_TCGA-CJ-4890 TCGA_TCGA-CW-5581 TCGA_TCGA-B8-4153 TCGA_TCGA-B0-4698 TCGA_TCGA-B0-5106 TCGA_TCGA-BP-4329 TCGA_TCGA-B0-4712 TCGA_TCGA-B0-4691 TCGA_TCGA-CZ-5982 TCGA_TCGA-BP-4962 TCGA_TCGA-DV-A4VX TCGA_TCGA-CJ-6030 TCGA_TCGA-B0-4818 TCGA_TCGA-B0-5706 TCGA_TCGA-BP-4973 TCGA_TCGA-B8-5165 TCGA_TCGA-CJ-4901 TCGA_TCGA-B0-5110 TCGA_TCGA-B0-4838 TCGA_TCGA-BP-4968 TCGA_TCGA-A3-A8OX TCGA_TCGA-A3-A6NN TCGA_TCGA-B0-4814 TCGA_TCGA-B0-4823 TCGA_TCGA-B0-5098 TCGA_TCGA-B0-5711 TCGA_TCGA-A3-3319 TCGA_TCGA-BP-5007 TCGA_TCGA-B8-A54D TCGA_TCGA-B0-4833 TCGA_TCGA-BP-4347 TCGA_TCGA-CW-5590 TCGA_TCGA-B0-4822 TCGA_TCGA-B0-4703 TCGA_TCGA-B0-5702 TCGA_TCGA-CJ-4918 TCGA_TCGA-CJ-4897 TCGA_TCGA-BP-4776 TCGA_TCGA-B4-5377 TCGA_TCGA-BP-4774 TCGA_TCGA-B8-

4143	TCGA_TCGA-CJ-5675	TCGA_TCGA-B0-5399	TCGA_TCGA-B0-
4707	TCGA_TCGA-CZ-4865	TCGA_TCGA-BP-4344	TCGA_TCGA-DV-
5565	TCGA_TCGA-AK-3436	TCGA_TCGA-A3-A6NJ	TCGA_TCGA-BP-
5191	TCGA_TCGA-B8-4154	TCGA_TCGA-B0-4688	TCGA_TCGA-B0-
5697	TCGA_TCGA-BP-4343	TCGA_TCGA-MW-A4EC	TCGA_TCGA-CZ-
5452	TCGA_TCGA-B0-5699	TCGA_TCGA-CZ-5460	TCGA_TCGA-B0-
4845	TCGA_TCGA-MM-A563	TCGA_TCGA-BP-4971	TCGA_TCGA-AK-
3425	TCGA_TCGA-CZ-4858	TCGA_TCGA-B0-4841	TCGA_TCGA-B0-
4819	TCGA_TCGA-G6-A5PC	TCGA_TCGA-BP-4992	TCGA_TCGA-CZ-
4864	TCGA_TCGA-BP-5198	TCGA_TCGA-BP-4349	TCGA_TCGA-CJ-
4878	TCGA_TCGA-CJ-5672	TCGA_TCGA-BP-4765	TCGA_TCGA-BP-
4970	TCGA_TCGA-BP-5009	TCGA_TCGA-CJ-4900	TCGA_TCGA-BP-
5186	TCGA_TCGA-A3-3370	TCGA_TCGA-B0-4810	TCGA_TCGA-CJ-
4916	TCGA_TCGA-B0-4846	TCGA_TCGA-BP-4756	TCGA_TCGA-B0-
5088	TCGA_TCGA-B0-4837	TCGA_TCGA-BP-5174	TCGA_TCGA-BP-
4803	TCGA_TCGA-B0-5097	TCGA_TCGA-B0-5119	TCGA_TCGA-BP-
4345	TCGA_TCGA-AK-3453	TCGA_TCGA-BP-4342	TCGA_TCGA-B0-
4844	TCGA_TCGA-BP-4804	TCGA_TCGA-CW-6088	TCGA_TCGA-B0-
5701	TCGA_TCGA-CJ-5681	TCGA_TCGA-B0-4843	TCGA_TCGA-BP-
4766	TCGA_TCGA-B8-A54H	TCGA_TCGA-B4-5836	TCGA_TCGA-B0-
5117	TCGA_TCGA-CJ-5680	TCGA_TCGA-CW-5589	TCGA_TCGA-BP-
5000	TCGA_TCGA-A3-3346	TCGA_TCGA-CJ-5689	TCGA_TCGA-3Z-

A93Z	TCGA_TCGA-CJ-4638	TCGA_TCGA-BP-5184	TCGA_TCGA-B0-
5116	TCGA_TCGA-CZ-4857	TCGA_TCGA-BP-5192	TCGA_TCGA-B8-
4620	TCGA_TCGA-BP-4959	TCGA_TCGA-CJ-4905	TCGA_TCGA-BP-
5181	TCGA_TCGA-EU-5906	TCGA_TCGA-CJ-4868	TCGA_TCGA-B8-
4148	TCGA_TCGA-CJ-4886	TCGA_TCGA-AK-3429	TCGA_TCGA-CZ-
5465	TCGA_TCGA-B0-4714	TCGA_TCGA-AK-3461	TCGA_TCGA-B8-
5159	TCGA_TCGA-A3-3358	TCGA_TCGA-A3-3372	TCGA_TCGA-CJ-
6032	TCGA_TCGA-B0-5703	TCGA_TCGA-B8-A54F	TCGA_TCGA-B0-
5095	TCGA_TCGA-CZ-4861	TCGA_TCGA-A3-3349	TCGA_TCGA-AK-
3455	TCGA_TCGA-CW-5585	TCGA_TCGA-BP-4981	TCGA_TCGA-BP-
4337	TCGA_TCGA-CJ-5686	TCGA_TCGA-A3-A8OV	TCGA_TCGA-AK-
3447	TCGA_TCGA-CJ-4876	TCGA_TCGA-B0-4834	TCGA_TCGA-B0-
5713	TCGA_TCGA-CJ-5671	TCGA_TCGA-CZ-5454	TCGA_TCGA-BP-
4790	TCGA_TCGA-B0-5107	TCGA_TCGA-B4-5834	TCGA_TCGA-G6-
A8L6	TCGA_TCGA-BP-4991	TCGA_TCGA-A3-3347	TCGA_TCGA-B2-
5636	TCGA_TCGA-B4-5844	TCGA_TCGA-BP-4801	TCGA_TCGA-A3-
3307	TCGA_TCGA-B0-4821	TCGA_TCGA-B0-4813	TCGA_TCGA-CZ-
5459	TCGA_TCGA-B0-5108	TCGA_TCGA-CZ-4862	TCGA_TCGA-MM-
A564	TCGA_TCGA-CJ-4881	TCGA_TCGA-B0-5077	TCGA_TCGA-B0-
5092	TCGA_TCGA-EU-5905	TCGA_TCGA-BP-5168	TCGA_TCGA-B4-
5832	TCGA_TCGA-B8-5163	TCGA_TCGA-DV-5575	TCGA_TCGA-CW-
5580	TCGA_TCGA-B0-5692	TCGA_TCGA-CJ-4640	TCGA_TCGA-A3-

3316	TCGA_TCGA-BP-4797	TCGA_TCGA-BP-5195	TCGA_TCGA-B0-
5691	TCGA_TCGA-BP-5006	TCGA_TCGA-CZ-5469	TCGA_TCGA-BP-
4999	TCGA_TCGA-EU-5904	TCGA_TCGA-B2-3924	TCGA_TCGA-BP-
5189	TCGA_TCGA-B0-5812	TCGA_TCGA-A3-3385	TCGA_TCGA-BP-
4787	TCGA_TCGA-BP-4325	TCGA_TCGA-A3-3322	TCGA_TCGA-BP-
5194	TCGA_TCGA-B0-5096	TCGA_TCGA-CZ-5986	TCGA_TCGA-B8-
4151	TCGA_TCGA-BP-4982	TCGA_TCGA-B0-4713	TCGA_TCGA-CJ-
4902	TCGA_TCGA-MM-A84U	TCGA_TCGA-B0-4816	TCGA_TCGA-A3-
3311	TCGA_TCGA-CZ-4866	TCGA_TCGA-BP-5182	TCGA_TCGA-CJ-
6033	TCGA_TCGA-CJ-4637	TCGA_TCGA-CJ-4885	TCGA_TCGA-B8-
5546	TCGA_TCGA-B8-5545	TCGA_TCGA-CJ-5676	TCGA_TCGA-B0-
5707	TCGA_TCGA-BP-5190	TCGA_TCGA-B0-5709	TCGA_TCGA-B0-
5109	TCGA_TCGA-B4-5843	TCGA_TCGA-B2-4098	TCGA_TCGA-CJ-
4893	TCGA_TCGA-BP-4985	TCGA_TCGA-BP-5001	TCGA_TCGA-A3-
3313	TCGA_TCGA-BP-4169	TCGA_TCGA-B8-5553	TCGA_TCGA-AK-
3443	TCGA_TCGA-B0-4696	TCGA_TCGA-DV-5576	TCGA_TCGA-BP-
4987	TCGA_TCGA-BP-4769	TCGA_TCGA-BP-4161	TCGA_TCGA-BP-
5010	TCGA_TCGA-AK-3445	TCGA_TCGA-B2-4102	TCGA_TCGA-B0-
5083	TCGA_TCGA-B0-4697	TCGA_TCGA-DV-A4VZ	TCGA_TCGA-BP-
4963	TCGA_TCGA-BP-4354	TCGA_TCGA-BP-4341	TCGA_TCGA-B0-
5099	TCGA_TCGA-B0-4839	TCGA_TCGA-CJ-5678	TCGA_TCGA-A3-
3308	TCGA_TCGA-B0-5120	TCGA_TCGA-B0-5100	TCGA_TCGA-B0-

4945 TCGA_TCGA-BP-4781 TCGA_TCGA-CJ-5684 TCGA_TCGA-BP-4346 TCGA_TCGA-AK-3426 TCGA_TCGA-CJ-5682 TCGA_TCGA-A3-A6NL TCGA_TCGA-CZ-5987 TCGA_TCGA-A3-3382 TCGA_TCGA-CJ-4636 TCGA_TCGA-CW-5583 TCGA_TCGA-B0-4849 TCGA_TCGA-B0-4852 TCGA_TCGA-B8-4621 TCGA_TCGA-BP-4807 TCGA_TCGA-CJ-4908 TCGA_TCGA-BP-4352 TCGA_TCGA-A3-A8OU TCGA_TCGA-AK-3434 TCGA_TCGA-CJ-5683 TCGA_TCGA-A3-3326 TCGA_TCGA-BP-4353 TCGA_TCGA-GK-A6C7 TCGA_TCGA-BP-4761 TCGA_TCGA-A3-3329 TCGA_TCGA-A3-3374 TCGA_TCGA-CZ-5470 TCGA_TCGA-CZ-5463 TCGA_TCGA-BP-4965 TCGA_TCGA-B4-5835 TCGA_TCGA-B0-5094 TCGA_TCGA-DV-5567 TCGA_TCGA-BP-4334 TCGA_TCGA-BP-5187 TCGA_TCGA-CZ-5988 TCGA_TCGA-CJ-4894 TCGA_TCGA-BP-4763 TCGA_TCGA-B0-4828 TCGA_TCGA-CJ-5679 TCGA_TCGA-CJ-4869 TCGA_TCGA-A3-3363 TCGA_TCGA-BP-4777 TCGA_TCGA-B8-5158 TCGA_TCGA-B0-5695 TCGA_TCGA-BP-5176 TCGA_TCGA-BP-4993 TCGA_TCGA-BP-4795 TCGA_TCGA-CW-5588 TCGA_TCGA-CZ-5464 TCGA_TCGA-BP-4762 TCGA_TCGA-BP-5178 TCGA_TCGA-B0-5115 TCGA_TCGA-B8-4622 TCGA_TCGA-B0-5102 TCGA_TCGA-BP-5008 TCGA_TCGA-CJ-4642 TCGA_TCGA-A3-3362 TCGA_TCGA-B2-5639 TCGA_TCGA-CZ-5458 TCGA_TCGA-AS-3778 TCGA_TCGA-B0-4693 TCGA_TCGA-CJ-4892 TCGA_TCGA-B0-5712 TCGA_TCGA-B0-4706 TCGA_TCGA-CZ-5461 TCGA_TCGA-6D-AA2E TCGA_TCGA-EU-

5907 TCGA_TCGA-BP-4770 TCGA_TCGA-B0-4848 TCGA_TCGA-CZ-5468 TCGA_TCGA-CJ-4904 TCGA_TCGA-B0-5710 GSE29609_GSM733579 GSE29609_GSM733580 GSE29609_GSM733581 GSE29609_GSM733582 GSE29609_GSM733583 GSE29609_GSM733584 GSE29609_GSM733585 GSE29609_GSM733586 GSE29609_GSM733587 GSE29609_GSM733588 GSE29609_GSM733589 GSE29609_GSM733590 GSE29609_GSM733591 GSE29609_GSM733592 GSE29609_GSM733593 GSE29609_GSM733594 GSE29609_GSM733595 GSE29609_GSM733596 GSE29609_GSM733597 GSE29609_GSM733598 GSE29609_GSM733599 GSE29609_GSM733600 GSE29609_GSM733601 GSE29609_GSM733602 GSE29609_GSM733603 GSE29609_GSM733604 GSE29609_GSM733605 GSE29609_GSM733606 GSE29609_GSM733607 GSE29609_GSM733608 GSE29609_GSM733609 GSE29609_GSM733610 GSE29609_GSM733611 GSE29609_GSM733612 GSE29609_GSM733613 GSE29609_GSM733614 GSE29609_GSM733615 GSE29609_GSM733616 GSE29609_GSM733617

KEGG_N_GLYCAN_BIOSYNTHESIS 0.462851059 0.404763762 - 1.96E-01 -0.154644359 -0.244646886 -0.384903547 0.124079655 - 0.301015594 0.308465387 0.004773554 -0.032977358 0.385147381 -0.322156089 0.163301786 -0.495905129 -0.233499839 0.287231354 0.330422895 -0.396750003 0.12325009 0.026430006 -0.206821169 -0.432924503 0.162943441 0.002224971 -0.377516834 -0.164486217 0.251027753 - 0.072539523 -0.43668479 0.031845745 0.287042156 -0.105523199 -0.352940218 -0.172293495 0.155286342 -0.477729886 0.224526995 -0.49860503 0.028835826 -0.101655918 0.334332557 -0.206176939 0.396292033 0.281588862 0.370372141 0.06497158 0.026006232 -0.269198531 - 0.51339507 0.166869003 -0.127042293 0.306496834 -0.327500323 0.479348135 0.503742504 -0.533396116 0.379531688 0.28333703 0.139928451 -0.03749593 0.010306263 - 0.029392708 0.196435133 -0.343597181 -0.27391573 0.26807854 0.314545724 0.292550354 -0.486734443 0.333935824 - 0.018964009 -0.1262715 0.239853584 -0.14034968 -0.016547344 - 0.216012133 0.086958154 0.114961902 0.506661623 -0.424265355 0.046075209 0.328582903 -0.099902339 0.277351792 0.218389884 0.214411699 -0.294340382 -0.50024595 - 0.16086854 -0.314041731 -0.449639157 0.003810338 0.532689337

0.234782174 -0.403914642 -0.041225461 0.212472757
0.147366762 0.228251285 0.081483129 -0.59787623 -
0.051370383 0.277345192 0.408689308 -0.295087244 0.019992855
0.065460414 -0.135452625 -0.497725832 -0.635701493
0.031866047 0.281705964 0.307402787 0.031932664
0.28255368 -0.088552409 -0.555858361 -0.715276913
0.31138042-0.548907296 -0.024822417 0.27381424 -0.059051256
-0.344761642 -0.502909118 0.125115087 -0.249259026
0.229482172 0.350871377 0.042285049 -0.60963343
0.084994773 -0.23548523 0.017836041 -0.116449934
0.301621961 0.315574842 0.30471984 -0.32695225 -
0.479653755 -0.313215496 0.230027656 -0.343215388 0.11152086
0.300980439 0.223036857 0.035053794 -0.498027178
0.322359189 -0.272397368 0.23042144 0.330839469 -
0.257139172 -0.250804099 -0.521056638 -0.23002414 -0.035314433
-0.127056591 -0.195241282 0.214954558 0.367093417 -
0.150637861 -0.599057278 0.346242824 0.330149397 0.410272932
-0.034540864 -0.213003834 -0.05583602 0.106861447 -
0.451761269 -0.433670705 0.278333746 0.224613548 0.431374826
-0.052640536 -0.309332478 -0.048787947 0.377284805
0.392788484 -0.038178696 -0.381554692 0.55254021
0.065970055 0.306513767 0.014440425 -0.02625359 -

0.348851777 0.355762778 0.291134715 0.031499908 -0.212099789
-0.562239428 0.224307675 -0.182224596 -0.041404808
0.293696336 -0.454758972 -0.065441777 -0.095482674
0.241398185 -0.206901747 0.131211251 0.507173849 -
0.054417076 -0.360596639 0.139970786 0.167066568 0.108749674
0.2693669 -0.439946034 -0.241769671 0.387845341 -0.119179274
0.277065292 0.408620123 -0.143235146 -0.246216805 -
0.180263465 -0.124371827 0.323371701 -0.39478143 0.167096677
-0.413061868 -0.021508101 0.324817379 -0.339566507
0.313661431 -0.026714168 -0.005699075 0.224898538
0.015205795 0.307756963 -0.444455415 0.03448488 -
0.578242033 0.015877009 -0.197628707 -0.457902544 0.35627644
0.129439718 -0.271013973 0.320818918 0.047442822
0.113272899 0.349570467 -0.22313443 -0.409427528 -
0.472460658 -0.321138561 -0.405130556 -0.529005301 0.213634547
-0.139486037 -0.054473243 0.116585386 -0.443272194
0.485278517 -0.450619064 -0.161013257 0.44165823 -
0.236822071 -0.13554565 0.261252324 -0.22502021 -0.054509621
0.160896028 0.185269344 -0.504261011 0.012927583
0.428885518 0.472937116 0.252765306 0.291247108
0.228692207 -0.026509916 -0.304656995 -0.54467466 -
0.404683551 -0.618654056 0.187658187 0.515105768 -0.557767185

-0.53102576 -0.344500267 -0.565368155 0.070470118
0.292638964 -0.413817532 -0.276445355 -0.133106908
0.066950757 0.37567833 -0.402143729 -0.412515631
0.165629315 -0.204400323 0.159918545 -0.455879189
0.243856674 -0.271870591 -0.545491749 0.111065709
0.177224337 -0.045298158 0.209002333 0.36171974 -
0.218891757 -0.499619723 -0.299401875 0.234826706 -0.476908807
0.052735264 0.273576713 0.286925495 -0.450467735 -
0.082019651 0.323848257 0.565963012 -0.285306676 0.174770577
0.34760243 -0.458141254 0.326230802 -0.092786601
0.13901652 -0.397957502 0.131859048 -0.058040642
0.100298586 0.360212131 0.077350297 0.366730538
0.210418097 0.212901901 0.419630347 0.149371737 -
0.12288425 0.152069795 0.471381437 0.25201083 -0.6014313 -
0.291900684 0.403585453 -0.071891709 -0.057351611 0.332218228
-0.234158367 -0.028667166 -0.102956166 0.242556275 -
0.00767121 0.173599426 0.318217034 -0.533886323 -0.399197033
0.381691853 0.09591968 0.098575841 -0.242061856 -
0.03593276 0.061240326 -0.055726242 0.265232357 -0.068296296
-0.084515872 0.181607306 -0.360774636 -0.265241092
0.107938176 0.295969053 0.298779878 0.307512524
0.294638419 -0.264392475 -0.365327032 -0.272338359 -

0.123390901 0.128432097 -0.49630394 0.066631307 -0.509632769
-0.506144067 0.441395114 0.155970943 0.09986898
0.375402882 0.164389837 0.41374009 0.010535999
0.293805152 -0.30030382 0.287846759 0.275388794
0.200105631 0.018536886 0.017372868 -0.200235033
0.169153926 -0.409147429 0.218995647 0.078637396
0.04542946 0.17754767 -0.276218241 -0.400633195 -
0.163946916 0.425744135 0.106315092 0.269484038 -0.192357998
0.054651824 -0.420921383 -0.437767289 -0.171222105
0.484319088 0.315826923 0.028288314 0.40707654
0.207493877 0.164011272 0.256223482 0.405291893 -
0.217455945 0.469023072 -0.239544543 0.221435765 0.108500873
0.219080887 -0.23046018 0.521156211 -0.026372429 -
0.556755023 -0.092067204 -0.037582736 0.569699976 0.190463913
0.316427126 0.334786571 -0.179089302 0.142163232 -
0.313604123 -0.564478877 0.184737071 0.223040786 0.015858581
-0.298955156 -0.114112549 -0.484032576 0.283662356 -
0.483886004 -0.117486212 -0.374570292 0.046034358 0.353143125
-0.003379637 -0.366934977 -0.190922364 0.416717424 -
0.145277288 -0.355606595 -0.572335698 0.095460543 0.04552417
-0.058091721 0.257723442 -0.394122653 0.315298602 -
0.346582534 -0.394444159 0.335529366 0.394631481 -0.309060464

-0.116869206 0.22145198 -0.326292241 -0.255210337 -
0.462205553 -0.191848127 0.300816057 0.139729365 0.267453907
0.14187958 0.018899682 0.086545626 0.399207724
0.341802741 0.124667382 0.425224607 -0.297331851 -
0.431272646 0.137230352 -0.196814407 0.135965311 -0.462184621
0.096578751 -0.140499212 0.296942433 -0.208345915
0.518443216 0.239972954 0.347114485 -0.126742644
0.094704748 0.309202861 -0.154400985 0.429175848 -
0.33984997 -0.253501963 -0.281253096 -0.248367584 -0.323813703
-0.42946233 -0.117005207 0.424165044 0.091046014
0.057003823 0.089385204 -0.2119501 0.211975187 -0.59408656
0.463146184 0.243032802 0.56002941 0.389139915
0.582835976 0.343926697 0.188758537 0.348158055
0.063174297 0.058411763 0.234321557 -0.409607096
0.084355875 -0.316404427 -0.201494921 -0.320113801
0.073319069 -0.129375322 -0.202340619 0.261554293
0.191283179 -0.137784926 -0.056209574 -0.406080598 -
0.23949995 0.324819413 -0.079010254 0.082148305 0.031719326
-0.350349819 0.262235069 0.123295032 0.11889585-0.453091945
0.128898084 -0.375485127 0.328087548 0.283784295
0.297803941 -0.063297214 -0.269181395 -0.265666109 -
0.191567156 0.330193893 -0.440799783 0.052969108 -0.102370684

KEGG_OTHER_GLYCAN_DEGRADATION 0.089542627 0.459139055 -2.06E-01 -0.28847757 -0.006082814 -0.399419304 -0.405607164 0.569978356 0.289725272 0.211993372 -0.492588636 0.297427765 -0.498172814 0.107055201 0.02144772 0.029087843 -0.041875934 0.532337435 -0.526288414 0.339455185 -0.444350405 0.162539605 -0.425676622 -0.434775257 -0.127162062 -0.019043688 -0.187634549 -0.17981793 0.033548617 -0.221124178 0.029797542 0.634244883 0.671314161 0.448192344 0.084746718 -0.06302675 -0.619370656 0.475175609 -0.639285351 -0.069485981 -0.10923037 0.560017528 -0.244989681 0.278937195 0.014297616 0.412956267 -0.301860255 -0.082058418 0.560508849 -0.511734996 0.224841906 0.011699315 0.400852118 -0.562785774 0.313869912 0.174358931 -0.574103273 0.452292249 0.408831291 -0.172149829 -0.100915088 0.244247401 -0.075279487 -0.071382267 -0.396591088 0.453026612 0.578171352 0.449320219 0.028355369 -0.520027211 0.287993838 0.23227708 -0.095613491 0.711690997 0.070731337 -0.0003865 0.167838616 0.273802771 0.628721336 0.414202064 -0.187659381 -0.212267008 0.432483353 -0.107673155 0.198216843 0.117993879 -0.222732131 -0.40245588 -0.395051015 -0.237074842 -0.27407573 -0.545614139 -0.265931958 0.42629775

5026 0.280055577 -0.196550098 0.279666811 -0.050587318

5027 0.260338217 0.653624216 0.445586832 -0.384274991 -

5028 0.250752427 0.569191679 0.025627969 0.421843409 0.149278207

5029 0.646691675 -0.287293945 -0.584757696 -0.560173608

5030 0.390039039 -0.16600218 -0.264201482 0.145939815

5031 0.301427624 0.090917489 -0.229175517 -0.453963679

5032 0.642646147 -0.393906089 -0.244250949 0.147989174 -

5033 0.225961477 -0.356271919 -0.484325733 -0.074025363 0.266961973

5034 0.051954852 0.688532636 -0.153902172 -0.406681314

5035 0.192388133 -0.266290539 0.167076721 0.002461662 -

5036 0.323627973 0.07589283 0.130489049 -0.317660125 -0.13983144

5037 0.165370678 -0.230628031 0.223496838 0.357663587

5038 0.303225626 0.211296354 0.591427462 -0.156647001

5039 0.250967539 -0.136363215 0.469733943 0.485816232

5040 0.071378247 -0.376175814 -0.418947074 -0.314073289

5041 0.548859384 -0.152876916 0.5299091 -0.364117876 0.218161948

5042 -0.478091344 -0.387699892 0.137351792 0.436030878

5043 0.239931324 0.563090571 -0.128622284 -0.006595823 -

5044 0.307258896 -0.529203617 -0.316644607 0.327079964 0.17342608

5045 0.111379912 -0.316593272 -0.264203706 -0.241506312

5046 0.467563215 0.621225345 0.169255481 0.06372447

5047 0.115783419 -0.358869214 0.593331315 -0.301214296 -

0.294931268 -0.387452659 0.600513395 0.45721305 -0.077416577
-0.340918642 -0.55089413 -0.067542282 0.285888185
0.281037421 0.020622294 -0.229688835 -0.289235189 -
0.094063447 0.594116286 -0.34043421 0.228696775 0.293302329
0.381121888 -0.481542532 0.40166133 -0.298489269 -
0.021016615 0.239297687 -0.470315258 0.363851422 -0.111133316
-0.181517598 0.187406873 0.509947921 -0.025336823 -
0.089943874 0.167620372 0.291031073 0.161084646 -0.338268513
-0.339772036 -0.118581247 -0.062795346 -0.122123888 -
0.287989852 -0.217105271 -0.130252205 -0.165646403 0.37330694
-0.444732039 0.385846113 0.283278453 -0.367972808 -
0.228361947 -0.350023894 -0.418547398 -0.185098514 0.444303537
-0.185260706 -0.235822416 0.274268942 0.33122018 -
0.188420667 -0.243216767 -0.128529058 0.042448952 -0.650354334
-0.406434838 -0.211139612 -0.366721367 -0.256006952 -
0.271308163 -0.589591818 -0.10744827 -0.505686198 0.242389385
-0.629489093 -0.355038883 0.497872984 0.119903898 -
0.603589014 0.340794776 -0.558902814 -0.456250477 -0.272200601
0.169754612 -0.304223461 0.143304643 0.346359142
0.43039203 0.289188331 -0.048550994 0.416995736 -
0.098953514 0.341431002 -0.487993824 -0.052475475 -0.432116936
0.581737113 0.019448856 -0.621597397 -0.596364927 -

0.398823787 -0.630322455 0.091787679 0.248732021 -0.629316665
-0.28125727 0.35993074 -0.252334558 0.201908691 -
0.378714851 -0.02486376 -0.064287191 0.105642832 -0.021736935
-0.273921315 -0.251047982 0.495189411 0.337887345 -
0.112557396 0.394368392 0.430680774 -0.144668377 -0.061151454
-0.39950235 -0.486890224 -0.442435199 -0.257856466 -
0.433951019 -0.154195676 0.635328323 0.496241868 -0.645807154
0.453453327 -0.30463751 0.167511279 -0.185428331
0.324073246 0.254087552 -0.512613231 0.311514153
0.497414748 -0.287857756 -0.198461946 0.047766358
0.111535609 0.226522993 -0.162359291 -0.091903163
0.489411329 -0.319222074 0.347205918 0.000809924
0.331555394 -0.392099428 -0.434095071 0.028027535 -
0.026243413 -0.293593996 -0.528906602 0.291746446 0.311292584
-0.302883552 0.41785274 -0.320254961 -0.27771481 -
0.429496473 0.215610922 -0.400288051 0.149815599 0.106659965
-0.487546446 -0.398284056 0.157841243 0.361021463
0.096455259 -0.19050498 -0.092901459 -0.253332768
0.212235193 0.510406271 -0.051531429 -0.126268116 -
0.239970372 -0.441811246 -0.094864006 0.304129327 -0.102023635
0.105278158 0.494941784 -0.291267911 -0.539315326 -
0.47314622 0.245476266 0.247403241 0.214873126 -0.529862886

-0.374490844 -0.37556408 -0.430745773 -0.081146051 -
0.044716849 -0.334584972 -0.153061067 -0.315816042 0.297164551
0.059965239 0.523202616 0.0588938 0.317237789 0.219545291
0.128018173 -0.270006665 -0.17170477 -0.467509821 -
0.214371638 -0.214138209 0.557909059 -0.312183496 0.200482622
0.223244656 0.207605575 -0.039624399 -0.183128972
0.344272299 0.283458676 0.416322648 -0.426885315 -
0.252177435 0.152579016 -0.139871693 0.363474155 0.015531928
0.51359704 0.602973351 -0.090651875 -0.15975651
0.352179583 0.473455628 0.370461273 -0.379543119
0.331699126 0.020852094 0.26758992 -0.450020547
0.291311595 0.444963661 0.011056315 0.362096316 -
0.183021018 0.121114984 0.020463572 0.378109996 0.504070816
-0.033204228 0.090704497 -0.315796545 -0.071581815
0.416670662 -0.532670035 0.479006242 0.290672689
0.349993818 -0.480082807 0.060377444 -0.217220759
0.223843015 -0.534755311 -0.293424872 -0.471323073 -
0.074916217 -0.186279517 -0.126910484 -0.636916736 -0.356524203
0.381754063 -0.414500174 -0.12377712 -0.34055718 -
0.546138926 -0.114932193 0.314048248 0.187659374 0.184108729
-0.277216872 -0.457479563 -0.466453565 0.593651342
0.58877652 -0.582533061 -0.330842548 0.324359608 -

0.484326799 -0.340653247 0.254933836 0.290634018 -0.19429207
-0.433769796 0.163356541 0.609075631 -0.116293129
0.363451711 0.174423066 0.515136158 0.432314514 -
0.057989858 -0.388778764 -0.536479978 0.365339969 -0.291869254
-0.329480849 -0.607962724 0.46281836 -0.29832704
0.519999186 0.272009908 0.370095971 0.082256556
0.358378797 -0.401995525 -0.063391637 0.154415746 -
0.568545125 0.26858989 -0.026499583 0.003083816 0.378507373
-0.284061313 -0.013181602 -0.602973561 -0.166196951
0.405606333 0.188694221 0.490337062 -0.309775529 -
0.491548536 0.200088959 -0.578586788 0.191981337 0.529286345
0.437498315 0.024017049 0.438926789 0.419543183 -
0.127540518 -0.398332628 -0.293945411 0.288308775 0.60578953
-0.37723073 0.472308697 -0.175128644 -0.533288821
0.397309682 0.339771798 -0.083676189 -0.270603242
0.334117632 -0.100245749 0.404632174 0.169322676 -
0.18680179 -0.021971228 0.375621648 0.371947539 0.274521765
0.158075473 -0.40804585 0.288921057 0.016179424
0.034153947 -0.167266022 0.476686128 -0.329798069
0.237184544 0.151428616 0.518815332 -0.314271015 -
0.657548618 -0.396827977 -0.476413598 0.084447768 -0.376080434
0.062667958 0.276326801

KEGG_O_GLYCAN_BIOSYNTHESIS -0.370008359 0.102498629 - 1.84E-01 -0.108543513 0.095655149 0.01121269-0.329759485 - 0.401230629 0.108947542 -0.350125522 0.254767704 0.028816943 -0.304328864 -0.207853529 0.026446799 -0.121527794 - 0.136338826 0.355603069 -0.016975618 0.033089977 -0.025366008 0.007991779 -0.287496457 -0.269302004 0.04044271 - 0.151012364 -0.115500534 0.291906708 0.310695052 -0.092054041 -0.093715438 0.465508191 -0.067575286 0.070438371 0.005254839 0.003455642 -0.177297756 0.195531885 - 0.397745638 -0.315199038 0.17869689 0.328834329 0.004623809 0.309805501 0.074675413 0.245925825 -0.296717849 - 0.029252705 0.501263805 -0.035247829 -0.15513572 -0.041459216 0.140501449 -0.339353855 -0.143274251 0.460264011 0.038124427 0.277534934 0.003538932 0.13671062 - 0.105530922 0.407271228 -0.085589946 0.017748019 -0.276156223 0.107179674 0.063070188 0.404320514 0.259029518 - 0.197992775 0.141832191 0.28435941 0.231113921 0.475538335 -0.026953957 0.190468126 0.051053556 -0.05537571 - 0.131093855 -0.237290904 -0.236276837 0.232511961 0.146136056 -0.131252828 0.247359997 -0.031812999 -0.062417761 - 0.010201429 -0.056566347 -0.219867476 -0.047716466 -0.192673687 -0.391910712 0.313691333 -0.101457566 -0.18059134 -

0.121480331 -0.035954449 -0.250559482 0.173981097 0.334865114
-0.189905543 0.076585102 0.148708653 0.052805024
0.110751429 -0.248317881 0.036431667 -0.09906711 -
0.306040821 -0.200380526 -0.049912843 -0.356663173 0.309429082
0.440596627 0.002098262 -0.371385534 -0.028664009 -
0.142702728 -0.055146679 -0.286108923 0.048817486 -0.334137997
0.083674185 -0.10235227 -0.091138579 -0.204709346
0.086108089 -0.272128509 0.406088277 -0.139078585 -
0.091316502 -0.239794483 0.230284458 -0.132655602 0.098476055
0.272569707 0.115114619 0.122695101 -0.133478311 -
0.040046344 -0.061259917 -0.154937927 -0.526212865 -0.128314176
0.262893455 0.202427623 0.167508351 0.158305071
0.037916366 -0.05142747 0.195876475 0.223026624
0.078990187 -0.161023371 -0.227181295 0.147256071
0.213361679 0.307954101 -0.077120034 -0.008290641
0.288063525 -0.356033859 -0.244783926 -0.207700721
0.146191207 0.161591262 0.287187004 0.070613874
0.174999087 -0.183848821 -0.305725704 -0.451106617 -
0.172340027 0.140652072 0.324587046 -0.157929045 -0.317624772
-0.301479956 0.103374765 0.323582021 -0.071745199 -
0.313252358 0.243353013 -0.12680619 0.121315599 -0.058010559
0.235653246 -0.020010539 0.361340222 -0.078461983

0.107079121 -0.145567759 -0.356496492 -0.544953537 -
0.178040167 0.079110401 0.099805816 -0.090732836 -0.04855333
0.016143187 0.377559915 0.177165204 -0.046226633
0.119160937 -0.061870624 -0.019294327 -0.123400098
0.036001827 0.060343094 -0.017234686 -0.410887434 -
0.033847368 0.082946524 0.124923787 -0.094676989 0.274343297
0.284667244 -0.399666367 -0.006066923 0.126323476 -
0.165942811 -0.23863206 -0.106022879 -0.200292787 -0.048765896
0.167606018 -0.087246089 0.031430879 0.450209042
0.034664666 0.269201094 -0.440802376 0.417467029
0.064226446 0.172294088 0.268366428 -0.046179312
0.209299218 -0.053915186 0.244012055 -0.061724123 -
0.533784119 0.148140589 0.198072243 0.281243542 0.066185949
-0.162436016 0.168273707 -0.344393547 -0.080418307
0.130737141 -0.096273455 -0.156973365 -0.04663577
0.087495012 0.258877914 -0.47256845 0.320151357 -
0.335202678 -0.513129408 0.038891352 -0.285485432 0.183171145
0.393452879 -0.165305793 -0.117487742 -0.228243962 -
0.087076047 0.166585563 -0.246886068 0.167488155 0.432110108
0.148697229 -0.398112998 -0.1596937 -0.240973412 -0.295231713
-0.438713548 -0.108140705 -0.279519067 0.274275576 -
0.054680484 -0.097838235 0.159511177 -0.075704675 0.056771152

0.039123884 0.292376179 -0.294095463 -0.187960749
0.386257588 -0.443561364 0.305472115 0.110141402 -
0.239552124 -0.308531824 -0.021073981 -0.041267271 -0.057605099
-0.19089014 -0.142688407 -0.373401283 -0.022891328 -
0.051631905 0.163648505 0.186927913 0.098948823 0.126379608
-0.142738506 -0.064560879 -0.108889862 0.040727038
0.13878958 0.227122947 0.165319188 -0.169474437 -
0.084542115 0.060383294 -0.046466872 0.047836802 0.041824126
-0.292648068 -0.177196101 -0.0183862 0.309265299 -0.319119604
-0.049819796 -0.297867733 -0.002168205 -0.088246766 -
0.18172927 0.26635566 0.429415681 -0.12946749 0.326479083
0.036161156 0.101103493 0.01496923 0.278975294
0.20888173 -0.256511153 -0.061380427 -0.123855414
0.213878032 0.149613007 0.078695182 0.215656457
0.050022915 0.018893179 -0.102988863 0.11905301-0.091360177
-0.200753729 -0.377669903 -0.193917547 -0.372111302
0.001328478 -0.272563252 -0.250217529 -0.323994122
0.091691762 0.076047904 0.123279999 0.147334436
0.054747061 -0.107495832 -0.17744323 -0.446728025
0.263982674 0.032831203 -0.003780517 0.133280964
0.32069601 -0.198478674 -0.306633311 -0.190249656
0.030879993 0.069516647 0.357301811 -0.428554938 -

0.260110040.066853447 -0.244706829 0.322320771 -0.177493589
0.327043312 0.239035283 -0.14026323 0.138153792
0.004113884 0.219076947 0.248504769 0.539847788
0.329153124 0.279066704 -0.226876401 -0.051482067
0.096069015 0.254652785 0.096106562 0.421050149 -
0.296381641 0.152206156 0.066955812 -0.015436659 0.046239321
-0.189634588 0.181015317 0.158399234 -0.337186436
0.069492191 0.337850841 0.129215179 -0.153848854 -
0.039465361 0.131535498 0.056576745 0.442123614 -0.091554495
0.259211915 -0.131686937 0.057835346 0.329984219 -
0.225860693 0.280203664 0.070775895 0.272912347 0.273135325
0.208646911 0.319661136 0.370387054 0.480896572 -
0.077804049 -0.21679573 -0.13672123 0.429077966 -0.086790912
0.060371097 -0.077396839 -0.082842965 -0.164053701 -
0.201708567 -0.011697417 -0.272472421 0.157536083 0.102085673
-0.056924457 0.234790458 0.118623730.128883299 -0.357841185
-0.188523173 0.233075802 -0.162128414 0.135253056
0.21922559 -0.40346251 0.147999923 0.185720258 -
0.294342674 0.14783065 -0.024402451 -0.297976881 -0.188900588
0.201674897 -0.011990694 0.327776011 -0.12728628 -
0.289183582 -0.008116141 0.434655244 0.240645578 -0.045203789
0.091952811 -0.3223351-0.015313983 -0.146065958 -0.440171529

0.064286465 -0.383401868 -0.117898234 -0.118205824
0.208289708 0.264711173 0.110254324 -0.21907596
0.12858626 0.226191293 0.152815927 -0.088552397 -
0.068626928 0.335198985 -0.114389259 0.020054982 -0.195865488
0.268321628 0.36560115-0.035113544 0.148669821 0.256787095
-0.165334929 -0.118753785 -0.281396261 0.252071389
0.170321794 -0.110402531 0.071652006 0.126172792 -
0.430710577 -0.215748431 0.005435584 -0.028663597 -0.070836415
-0.017238866 0.165579339 0.336375423 -0.213864851 -
0.137606194 -0.099259288 -0.081175959 -0.448729531 0.253388254
-0.105892881 0.43145302 -0.052781404 0.108267999
0.122770269 0.338147695 0.170911568 0.151988213
0.054781816 0.116232393 0.020481551 0.201739645 -
0.128460904 -0.156761451 0.135411046 0.0141078 0.14282875 -
0.277439699 -0.043782197 -0.123956895 -0.170477607 0.143905897
0.027218958 0.089571453 0.112748499 0.25446082
0.160331884 0.041793171 -0.24470633 -0.16279633
0.09364451 0.168286826 0.276532457 0.103021233 -
0.19567952 0.013143536 0.076285703 -0.154532726 -0.006181297
-0.394196817 0.066311177 -0.01050851 0.205012832 -
0.19389061 0.118330697 0.25482159
KEGG_GLYCOSAMINOGLYCAN_DEGRADATION 0.093504075

0.066780267 -3.36E-01 -0.166068935 -0.059915003 -0.244835514
-0.113396702 0.368876863 0.276755568 -0.071163536 -
0.410173304 0.208012747 -0.087685886 0.172720712 0.358098303
0.215742255 -0.177730009 0.276496943 -0.144671356 -
0.04191392 -0.39931417 0.145030127 -0.203352482 -0.295675556
0.019558117 0.265185555 -0.06766396 -0.041984604 -
0.322003565 -0.168038736 -0.106981217 0.324818365 0.308364755
0.346099419 0.225121684 0.323995253 -0.352750766
0.471502929 -0.281762238 -0.24381043 -0.13107159 0.4362642
-0.190525965 0.231548571 0.214601481 0.239579559
0.11387155-0.163300819 0.26240508 -0.35104147 0.362320863
-0.14789871 0.02953248 -0.471889565 0.336948542
0.217180837 0.211963602 0.253579765 0.436403919 -
0.221386946 -0.032783867 -0.079453894 -0.204813625 -0.294870713
0.238306185 0.129316349 0.489671672 0.309945869
0.211605683 -0.287871836 0.109372603 -0.033582292
0.057771150.265156409 -0.128659506 0.132213643 -0.259390658
0.036361013 0.433688503 0.090906552 -0.00858827 -
0.051973843 0.012159882 -0.020380006 0.158676669 0.19215992
-0.224575914 -0.170156351 -0.012316523 -0.168485022 -
0.262579639 -0.306433826 0.114563672 0.276653484 -0.206935649
-0.219545165 0.244061533 -0.121620981 0.31390979

0.293537126 -0.257134813 -0.031281586 -0.359662773

0.484826497 0.376343784 0.25927825 -0.236770327

0.287435921 -0.114589443 -0.207358286 -0.327926766

0.278210854 0.098745285 -0.007235409 0.037130018

0.134135842 0.355068561 -0.274567359 -0.308731372

0.390141022 -0.170241463 -0.129884932 -0.060853539 -

0.180766288 -0.351686666 -0.199236455 -0.04518539 0.031987175

-0.040074268 0.133067047 -0.295741351 -0.485594492

0.117109034 -0.191649789 0.252942655 -0.01325843

0.09454606 -0.031093049 -0.218208644 -0.21021492 -

0.142541881 -0.28679779 -0.421408357 0.222580764 0.119440838

0.159152389 -0.076992832 -0.089599507 0.058199471 -

0.031736243 0.092442049 0.309494257 0.197575831 0.000986804

-0.306923014 -0.249672678 -0.064123614 0.147828977 -

0.281586096 0.32138282 -0.248098999 0.278971572 -0.528987769

-0.201848474 0.22260873 0.438022826 0.02390278

0.143990995 -0.118912831 -0.345569323 -0.208008447 -

0.387397871 -0.286207081 0.587102979 0.077463132 -0.001596789

-0.276597004 -0.397414058 -0.16445049 0.175606162

0.470925863 0.313131103 -0.096818089 0.045181665 -

0.164791993 0.114097594 -0.132491444 -0.380710965 -0.261772196

0.222085359 0.266766598 -0.178062798 -0.052842631 -

0.054836565 -0.294252469 -0.01457418 0.218567561 0.014349624
0.169863558 -0.339836955 -0.028141954 0.251826867 -
0.414121871 0.272417778 0.528547025 0.271730494 -0.159625398
-0.08796545 -0.310199713 -0.130506815 -0.00383116 -
0.554985642 0.082478006 -0.249467937 0.09007531 -0.009332293
0.023752352 -0.176759111 -0.19873307 -0.096938246
0.378114155 -0.197354469 0.187780927 -0.271471055
0.054799118 -0.169329359 0.335029253 -0.212689317 -
0.29780349 0.129443038 -0.243861115 0.364360376 -0.220971649
0.286838939 0.127377892 -0.273297742 -0.283832742 -
0.342152774 -0.445921232 -0.291735762 0.502802655 0.039960845
-0.271071914 0.391972958 0.179357655 0.034279927 -
0.247682159 0.099131436 -0.135677858 -0.202768582 -0.17362319
-0.050091093 0.186732007 0.061710729 0.028759264 -
0.329792762 -0.213829625 -0.084320497 0.12594457 -0.229883968
-0.304301223 0.067218284 -0.192083813 -0.221773578 -
0.114477663 -0.567008078 0.066615852 -0.159846142 0.236603797
-0.066055718 -0.0329131 0.379984769 0.29735019 0.04414164
0.027279858 0.108903781 -0.05946397 0.251812339 -
0.184082915 0.083224874 -0.345448214 0.388052406 0.154045821
-0.305029961 -0.088212552 -0.159308606 -0.167698381 -
0.006590311 -0.077133202 -0.157072401 -0.051415047 0.197350972

-0.142918579 0.223289958 -0.421828913 -0.153744564 -0.151318969 -0.178017172 0.358903604 -0.38403313 -0.327330969

-0.010396996 0.109098092 -0.02661166 0.118364325

0.22931733 -0.143449659 -0.208956553 0.047845754 -0.183453452 0.079273819 -0.29796607 -0.062113294 -0.256203148

0.445864515 -0.192878631 -0.254821053 0.421174151 -0.330435411 0.224489337 0.044033806 0.289534124 0.129802615

-0.159871975 0.498143607 0.606236643 0.125281835 -0.402042438 0.02905081 0.137293459 -0.05262386 -0.049244418

0.049948836 0.201027876 -0.104000812 0.207264809 -0.056452805 0.371595728 -0.092968884 -0.269075823 -0.075962636

-0.028126172 -0.106864672 -0.030348151 -0.260117584

0.051867763 -0.171955762 -0.043373211 -0.053272389 -0.243290305 -0.347211733 -0.213760262 -0.066356432 -0.367637696

0.200962372 -0.396163897 -0.057599325 0.058367626

0.300408972 0.200276734 -0.376346424 0.095057997 -0.364977065 0.429598804 0.285964069 0.029234188 -0.24933594

-0.021584619 0.062980009 -0.284109435 0.403912382 -0.171373236 0.252349972 0.216601133 -0.179315089 -0.311288876

-0.307721683 0.128511222 0.046295024 -0.373666898 -0.419576732 0.088438453 -0.223681672 -0.189815584 0.101247395

0.00273807 -0.255509476 0.037461976 -0.023253418

5356 0.294697958 -0.174380614 -0.036550064 0.017087717

5357 0.221141309 0.180930835 -0.007764476 -0.06554752 -

5358 0.14466643 -0.329790076 0.012990446 -0.362483136 0.323066266

5359 -0.442918735 0.27204313 0.060701193 -0.103245369 -

5360 0.048333973 0.070187314 0.259595157 -0.239043058 0.215127933

5361 -0.356941272 -0.151854713 -0.102846231 0.02378519

5362 0.07582603 -0.130044627 0.318819795 0.410648626

5363 0.277129117 -0.403103416 -0.015893782 -0.001509828

5364 0.040693146 0.019487619 0.337105036 -0.030017195

5365 0.313311178 -0.27413862 0.104398827 -0.04994242 -

5366 0.062916377 0.36275732 -0.0767595 0.004350856 0.003184197

5367 0.299663189 0.399320334 0.304638524 0.089547049 -

5368 0.211796498 -0.155672935 -0.032723212 -0.265347979 0.227302685

5369 -0.04264215 0.355224852 -0.337090782 0.052712267 -

5370 0.047005388 -0.031738521 0.138131553 0.050315594 0.266810031

5371 0.128120599 -0.3596468 -0.257455088 -0.088229487 -0.354056549

5372 0.24496145 -0.092559829 -0.390160444 -0.034119961 -

5373 0.11181284 0.456683903 0.230776286 0.095642326 0.258581187 -

5374 0.269616783 -0.112729332 -0.528627583 0.278150973 0.128165088

5375 -0.350334659 -0.119883687 0.235293599 0.011308883

5376 0.260487076 0.00500257 0.142945404 -0.043977689 -

5377 0.203024369 -0.020664921 0.587390471 -0.384569931 -0.07136951

-0.199017017 0.223367453 -0.05190994 -0.0260275 0.013097857
-0.365645425 0.240070085 -0.519583546 -0.16109031 -
0.324809006 0.672555125 -0.399655361 0.331082386 0.05114986
0.398560993 -0.231594287 0.379477607 0.08896308
0.079921661 0.088890625 -0.298745163 -0.123047047
0.165616588 -0.288640541 0.354773545 -0.154485681 -
0.256317627 -0.005246142 -0.054627398 0.119500835 -0.032108499
0.184786701 -0.383115708 -0.328091913 0.214970014 -
0.378599103 -0.080952066 0.262549136 0.184114469 0.259330403
0.161067843 0.323424081 -0.15628907 -0.339730899
0.245244548 -0.057097983 0.055598885 -0.396770294
0.054311376 0.092993715 -0.152644073 0.131730077
0.433510409 -0.029129817 -0.208376095 0.355434419
0.218995065 0.107888769 0.393238671 -0.280680141
0.293794509 -0.080486724 -0.090931331 0.20527062
0.382553609 -0.30397441 0.154510597 0.013152668
0.343580189 -0.429903565 -0.116497575 -0.343832531
0.232202324 0.323615988 0.337538342 0.232983636 -
0.423163326 -0.390348687 -0.104382675 -0.006100118 -0.322853688
0.112273197 -0.127394727
KEGG_GLYCOSAMINOGLYCAN_BIOSYNTHESIS_KERATAN_SULFATE -
0.247626325 -0.105668095 -2.98E-01 -0.327995687 -0.277191166 -

0.163556622 -0.523139629 -0.253027495 0.598973788 0.019011033
-0.35545249 0.086209405 -0.02310741 -0.026791032 -
0.314292674 0.037704247 0.043807321 -0.294499057 0.362919425
-0.086422716 0.210077671 -0.498205416 -0.050939882
0.031201288 0.624629892 -0.297839522 -0.388034343
0.018608591 -0.289935336 -0.045868423 -0.194152357
0.367458201 -0.185819922 -0.413377202 0.213337367
0.303001589 0.329727502 0.60761517 -0.048144707 -
0.280830849 -0.087986847 0.543551309 0.009682596 -0.038409005
0.237384803 0.466804383 -0.066737008 -0.46235468 -
0.244271909 -0.130398651 -0.040443661 0.065448791 0.059022977
0.348019629 -0.049726069 0.432687074 0.052323025
0.244245081 -0.085684037 0.20771956 0.520079144
0.182028892 -0.340496056 0.179809629 0.007624462 -
0.348168722 0.366002846 0.358273417 0.00905765 -0.515130127
0.206288729 0.20980402 -0.074300853 0.025828619 -
0.237525117 0.358949804 -0.377356415 -0.514584658 0.089079526
-0.055672812 0.153813604 0.254737047 0.12659351
0.033114614 0.350542854 0.238942602 0.212051788
0.061805482 0.392056444 -0.368154077 0.053594362 -
0.107972109 0.001376734 -0.325239198 -0.220215231 -0.50578698
-0.16719618 0.330190306 -0.046232928 -0.003332628

0.232291156 -0.398910458 -0.398478679 0.39905249
0.305711843 -0.305535725 0.082790804 -0.131282022
0.349226241 -0.325175158 -0.361044662 -0.507961524 -
0.310229566 0.526599764 -0.066789461 -0.197444894 -0.319276266
-0.595892276 -0.453347308 0.076685424 -0.469065055 -
0.329422398 -0.402133419 0.015908591 -0.016324464 -0.356020663
0.094310806 -0.031757735 -0.272566899 -0.565913664 -
0.219577653 -0.355022435 -0.109920533 -0.257387076 -0.125673746
0.532143727 0.615482098 -0.049367903 -0.2964689 0.018247105
-0.411516208 0.118982052 -0.085959255 -0.438574268
0.040044151 -0.132992995 -0.106664422 -0.463372739
0.183017678 0.452281196 0.352940639 0.399125472
0.368998853 0.35998399 -0.217734297 -0.21738953
0.11645136-0.260858033 -0.205322174 0.112187838 0.198680808
0.400509575 -0.485165422 -0.266066911 0.341742549
0.550673295 0.188694947 0.064176708 -0.148599018 -
0.200665161 -0.082593913 -0.461809355 -0.346830327 0.126802959
0.264933602 0.374311007 -0.326332881 -0.466068205
0.004837328 0.525237845 0.68498808 -0.122864454 -
0.516633961 0.461962102 0.204327798 -0.284386918 -0.288811526
-0.33564838 0.352554512 0.013320066 0.255797597 -
0.385626689 -0.599185864 -0.086843811 -0.29076472 -0.292092101

-0.331432842 -0.188342266 -0.236858362 -0.374469539
0.548460514 -0.336859506 0.164362574 -0.155555646
0.502675106 -0.275607765 -0.086319517 -0.478214277
0.28994667 -0.352316765 -0.505789096 -0.110267653 -
0.022197831 0.176463226 0.360826146 -0.03710014 0.28083248
0.189283915 -0.585134521 -0.367311211 0.485527418
0.050068193 -0.517609974 0.056336189 -0.174865112 -
0.090680771 0.456188937 0.178097529 -0.211048724 0.22775905
0.081714763 0.242206918 -0.278174066 0.405322495 -
0.436483196 0.023722145 -0.20841285 0.121197843 -0.316243039
0.019517809 0.490079341 0.046140047 -0.445667808
0.114295581 -0.04860042 0.129973513 0.226396518 -
0.349497073 -0.367311585 -0.406835518 -0.18534483 -0.229834943
0.057086326 0.462574563 0.122572956 0.505194986 -
0.038520413 -0.125907296 -0.374347831 -0.068837477 0.137422629
-0.070522734 -0.426329382 0.531999075 0.338522031 -
0.118298471 0.041554257 0.248549065 -0.178387317 -0.174270957
-0.211078292 0.394448921 -0.041474554 0.299959377 -
0.360153738 0.093583173 -0.485041466 -0.425940662 -0.481091734
-0.073812945 0.005659818 0.398090681 0.533767583 -
0.232534226 0.075196507 -0.465188633 -0.444843973 -0.546070581
0.442780087 -0.221600661 -0.280053377 -0.23501044 -

0.001934915 0.135204456 -0.038208911 -0.116042065 -0.323756539
-0.195807118 0.388953697 -0.306903538 -0.392672481 -
0.090330646 -0.155450211 0.269827583 -0.228465853 0.097816288
0.583539411 -0.231589752 0.58851495 -0.430422617
0.163023323 0.096922381 0.256541153 -0.341489082
0.263284215 0.381356304 0.062454158 -0.340112361 -
0.381629641 0.235010703 0.018169829 0.079371974 0.347412089
0.146582742 0.381843202 0.428160181 -0.069633198 -
0.10631651 0.017068546 0.097515022 0.565190772 -0.142849233
0.050377534 0.111783653 0.352485368 -0.025661797
0.107881201 0.749055946 -0.304708607 0.061583423 -
0.25548815 -0.476127454 -0.496082749 -0.472854856 -0.302311012
-0.326687358 -0.436268104 -0.360302598 -0.339950534 -
0.176198601 0.53748823 -0.132850063 0.14381964 -0.439355122
0.012495523 0.011996920.06696524 -0.212143355 -0.440723953
-0.268425019 -0.325585126 -0.006668006 -0.291954609
0.386523683 0.028950811 0.376281074 -0.22392221
0.225338649 -0.189936802 -0.010405683 0.534610205
0.14062729 0.327614532 -0.006320933 -0.189474733
0.244857388 0.044885499 -0.197484647 0.617278816 -
0.219927125 -0.124491248 0.561664779 -0.016656023 -0.298468509
0.317278091 0.287279999 -0.004366768 0.482769818 -

0.18028205 -0.383430002 -0.023539966 0.222387225 0.461673815
0.315885808 0.248786688 0.11716015-0.231276565 0.328243194
0.331032573 -0.138188901 -0.391354076 0.409071574
0.055315203 -0.472407653 0.200554304 -0.584091257 -
0.385294926 -0.081931524 0.353165295 -0.146471647 -0.440888597
0.073292739 -0.072865454 0.327006008 -0.169518521
0.202970084 -0.296327848 -0.31632585 0.13441233
0.498096233 0.245628359 -0.2640509 0.272949522 0.397526844
-0.029162422 -0.1190807 -0.450373961 0.152866388 0.3775065 -
0.126088105 0.122547791 -0.057883677 0.372049632 -0.454439017
-0.252232211 0.327292267 -0.060877992 -0.05556795
0.277882488 0.278354007 -0.207296829 0.50350603 -
0.389157357 0.250895387 0.291234721 -0.433539263 0.219136004
0.294190971 0.423583198 -0.438889675 0.461745847 -
0.320745721 -0.128625099 -0.014284271 -0.397712224 0.622646057
-0.015475483 0.287264308 -0.312578235 0.493063531 -
0.313957983 -0.53688391 -0.024553717 -0.041712234 0.268701037
-0.406756789 0.180958806 -0.184374386 -0.264453507 -
0.561165936 -0.544017941 0.233821716 0.176840227 0.430374484
0.260086668 0.200636942 -0.055257607 0.111647625 -
0.395643301 -0.274333589 0.373792928 0.511295834 -0.284675218
0.383600635 -0.350177297 -0.432899644 0.075768926 -

0.356648227 2.39E-05 0.218458871 0.234010967 -0.238306269
0.188753467 -0.10135024 0.005789955 -0.509145904
0.493245896 0.357159134 -0.053242073 -0.367475876
0.53992391 0.238125031 0.164036262 -0.288588008
0.548608452 -0.053981295 -0.36963186 0.275510278 -
0.016634911 -0.232715511 -0.350184761 0.04350251 0.258341862
-0.083018948 -0.391612061 -0.518550523 -0.319718777 -
0.048175754 -0.482905858 -0.273377931 -0.080985919 -0.184942477
0.052862039 0.115485397 -0.218188291 0.626824533
0.155033436 0.616896352 -0.057871815 0.135863004 -
0.157050034 -0.148364693 -0.068137346 -0.335329707 0.14150148
0.442228902 0.200355053 -0.19362349 0.35718341
0.271918779 -0.108152811 0.414424762 0.123816452 -
0.127453346 -0.174132562 0.122368478 -0.204165796 0.284186627
-0.469518258 -0.092823321 -0.086051422 -0.367741336
0.466693599 0.354780149 0.129813739 -0.119321696
0.250848229 -0.40616803 0.274559422 0.219294013
0.490392359 0.097387532 -0.241188037 -0.227638953
0.176614602 0.52466574 -0.47951638 -0.045396532
0.100486151
KEGG_GLYCEROLIPID_METABOLISM 0.208240511 0.362646507 -
2.42E-01 0.210653494 0.289977872 0.125395886 -0.070579226 -

0.169002543 -0.294616046 0.002118666 -0.118311333 -0.290704855
-0.236696448 -0.205364194 0.092293886 0.137855408 -
0.311302325 0.126707084 -0.012938584 0.031327182 -0.043636597
0.05975854 -0.211311739 0.051871978 -0.24655535 -
0.181739519 -0.050054854 -0.014454467 -0.003563396 -0.172075967
0.193275654 -0.133560794 0.102806635 0.203609425 -
0.335426807 -0.123676045 -0.090742669 0.224482409 0.158399039
0.176085415 -0.170482231 0.202893412 0.054889291
0.21868246 -0.174640124 -0.016436399 0.183640571 -
0.044714729 -0.24906122 -0.147413458 -0.113680907 -0.268103375
-0.005044049 0.278796868 0.154826032 0.129720722
0.165250929 -0.147075694 -0.006384852 -0.180087551 -
0.32832839 -0.196938211 -0.134032759 0.047768743 0.036225032
0.194333345 -0.250672736 0.043576474 0.008338873
0.189320156 0.00141861 -0.248764782 -0.186765529 -
0.14119088-0.079385969 -0.050481182 0.143402507 0.082093155
0.186237979 0.227467625 0.12527412 -0.093557293
0.015774732 -0.218946871 -0.308525918 -0.110513896 -
0.189200595 -0.102573054 -0.085135476 0.193475557 0.011098992
-0.042663346 0.098413683 0.315475337 0.077332441
0.107136303 -0.110613559 -0.292624444 0.128146652
0.121073597 0.136258217 0.066399976 -0.006851812

0.145973965 -0.167739698 -0.220318741 -0.081547204
0.251287135 -0.059836684 -0.135031383 0.061334158 -
0.158181603 0.16511157 -0.288292086 0.168807462 -0.245163637
0.081805459 -0.01848293 -0.002304464 -0.031422169
0.344086735 0.176382452 0.042219764 0.049234485
0.022461973 0.173911288 -0.130961912 -0.224241677
0.022092325 0.012928358 -0.209548192 -0.171346618
0.057926869 0.101279719 -0.194726342 -0.277413116 -
0.312250146 0.202141431 -0.177648043 -0.248464104 -0.263964947
-0.04957761 0.195752523 -0.019635235 -0.315277444 -
0.148934753 0.249504711 0.127680567 -0.018841716 0.055732543
0.150347741 -0.259209974 -0.175480938 -0.161506089
0.16613704 -0.036814795 -0.375407262 0.202543732
0.244526541 -0.021980309 -0.295298077 0.129518382 -
0.16058488 -0.149675315 0.277578751 -0.309530886 0.261897552
0.116959449 0.079635577 -0.127524651 0.122129485
0.391218143 0.191478699 0.080739751 -0.179380945 -
0.150348857 -0.146353524 0.152946149 -0.261343838 0.054056629
0.029252802 -0.063258982 0.135431355 0.075592334
0.237304737 0.062341927 -0.023402521 -0.083428786 -
0.138906783 0.331195245 -0.275358593 0.198229333 0.015902121
0.218062586 -0.138682644 0.115749494 -0.130826339 -

0.04199313 -0.065056928 0.201409119 0.011709109 -0.016933921
-0.328652925 0.268275072 -0.16430504 -0.062791121 -
0.033876208 -0.25434076 0.011337948 0.11571103 0.15765858 -
0.195166458 -0.029688954 -0.11831199 0.237788827 0.112408094
-0.293569708 -0.037182393 0.14451214 -0.084898256 -
0.25554015 0.347088455 0.184648399 -0.174783246 -0.238048289
-0.046195036 -0.200205565 -0.032440015 -0.064826198
0.093094762 0.164480804 -0.201864441 0.16382576 -
0.219958249 0.195004132 0.191585461 -0.156901246 0.122970784
-0.072983294 0.168074669 0.029288659 -0.133349112
0.075661281 -0.218316273 -0.145246169 0.102102778 -
0.07220282 -0.009624062 0.216300611 0.373227266 -0.012832331
0.085514896 0.275463872 -0.33784482 0.114427918 -
0.160234347 0.208607546 0.159537109 0.109936251 0.020967667
-0.252040856 0.00169981 0.193125763 0.010827925 -
0.121328843 -0.105318568 -0.132198902 -0.109433555 0.018623716
0.208893043 -0.031296389 -0.254116729 0.001183075 -
0.139081634 -0.093354115 0.203831 -0.049303789 0.15632549
0.241199581 -0.253531604 -0.050658055 -0.112765568 -
0.123189574 0.045266469 -0.071534433 0.232815525 -0.020577368
-0.280273134 -0.423136277 0.23186929 -0.053079136
0.252076739 0.078628161 -0.398027351 -0.209383092 -

0.037519595 0.040938769 0.305872691 0.037711480.160750054
0.131385443 0.269143211 0.317855818 -0.021518421
0.227591313 0.022033376 -0.096506268 0.06592665 -
0.110271991 0.205248203 -0.228366572 0.013965242 -0.153812425
0.234058446 0.056821815 0.236650604 0.235303679
0.116252428 0.179622984 0.145301238 -0.031937445
0.12070039 0.439306572 0.307289133 -0.08450954
0.051800973 -0.049823941 0.272550498 0.029105145
0.036801485 -0.00729441 0.157062898 -0.096660349
0.141086406 -0.021586607 0.178253246 0.121300253 -
0.079048315 -0.02098682 0.211746428 0.173267657 -0.128945222
0.088718295 -0.229939091 0.140063989 -0.065063878 -
0.079144948 0.25031957 0.029927484 -0.218301917 -0.207182746
-0.243283828 -0.062162162 0.234084963 0.293856521
0.199613367 0.101200616 -0.199115829 0.051938737
0.051898816 0.155059274 -0.113540354 0.122559266 -
0.29200456 -0.056464305 0.113733639 0.043013483 -0.092883787
-0.08330946 -0.169772722 0.018621525 -0.211158246
0.282118197 0.220480954 0.129566681 0.130719328 -
0.329658145 -0.038077205 -0.043025263 -0.259294727 -0.071075937
-0.121133033 0.163251796 0.050469277 -0.166978543
0.074948299 -0.196990226 -0.268274703 -0.159260449 -

0.217191517 0.005279281 0.127282846 0.290044426 0.10892171
-0.117516227 0.091424998 0.083717214 -0.250601234
0.004638761 0.186285189 0.006386182 -0.222249669 -
0.264906317 0.200810215 0.022398481 0.004342983 0.041547793
-0.021141614 -0.135223237 -0.269685137 -0.095776861
0.025937776 0.259110745 0.042589479 -0.046044467 -
0.17404324 0.235209695 -0.008958129 -0.078764899 -0.123282034
-0.216075509 -0.112155952 0.074915283 -0.332344878
0.006112426 0.063443056 0.099608757 -0.195695667 -
0.129201982 -0.222355743 0.035756097 0.012201357 -0.122632241
0.455092052 0.086355129 -0.270066226 -0.044549035 -
0.124381409 -0.221008434 0.046850199 -0.059079988 -0.154167821
0.31810797 0.135669808 0.087197329 0.094689646 -
0.086730508 -0.09204757 -0.034869137 0.115888498 -0.290235524
0.210682928 0.245853683 -0.080395311 0.168558067
0.059912752 -0.299389242 -0.155887994 0.256454267 -
0.095261619 0.119047382 0.043854908 0.028590083 -0.200896231
0.096707629 -0.153595209 0.069682466 -0.091135942
0.202142501 0.137625949 -0.224064913 0.103466017
0.059754008 -0.107729902 -0.03766464 -0.279041651
0.152251428 0.187220401 0.227459217 -0.226341865
0.079768192 0.09459234 0.05238106 0.186412404

0.04421019 -0.030662984 0.022842577 0.053017106 -
0.163253371 -0.052516923 -0.091705982 -0.090353916 -0.25495721
-0.187174512 -0.321891124 0.093039331 0.115197082 -
0.059532743 -0.142348392 0.177187567 0.043075076 0.16565159
0.128379799 -0.29895909 -0.171190117 -0.117776642 -
0.002658838 -0.247263587 0.062922183 0.076698719 0.07489172
-0.102547349 -0.159430205 -0.095590318 0.396980929
0.039370314 0.069806707 0.150342569 -0.072863588
0.17582506 0.176031238 -0.099458703 -0.202268556
0.065708562 -0.049531698 -0.149046173 0.069830936
0.101432256 0.132167 -0.056393622 0.110406943 0.12172293
0.112604291 -0.080204594 0.18273372 -0.191424955
0.129295768 0.265163973 -0.008013887 0.026976006
0.067543657 -0.20575143 -0.154265464 0.084483898 -
0.126936687 -0.013123448 0.025285117 0.064081797 0.109506638
0.206503623 0.179898251 -0.197907953 0.080236961 -
0.078926434 0.076037489 -0.105560358 -0.061913759 -0.202488888
0.150556182 -0.372706347 0.220618998 0.164967676 -
0.141563076 0.178372615 -0.075122364 -0.153824096 -0.025134575
0.008760856 0.251402557
KEGG_GLYCOSYLPHOSPHATIDYLINOSITOL_GPI_ANCHOR_BIOSYNTHE
SIS 0.508317355 0.080815957 9.91E-02 0.346685402 -0.349564756

-0.051990657 0.040825916 -0.452777085 0.010193288 -
0.39346305 -0.170268102 0.448891844 -0.376465851 0.301140482
-0.423508492 -0.111967391 0.179363477 0.264724388 -
0.402023894 0.298025055 -0.007776596 -0.122907982 -0.317223028
0.088533741 -0.412330701 -0.059138544 -0.121241434
0.054481024 0.031220442 -0.455851558 0.352964317
0.431843222 0.293860165 -0.211904917 -0.466291991 -
0.241217182 -0.379417619 -0.090290792 -0.332914098 0.288528886
0.227917646 0.078020011 0.348512939 0.227273483 -
0.040995621 0.137688943 0.172856365 -0.01238141 -0.050063619
-0.392140331 -0.095441205 0.118199043 0.042748194 -
0.146403682 0.221209337 0.565864839 -0.471628933 0.205145787
0.417465407 -0.313754754 0.330311923 -0.378014829
0.333893077 0.352645846 0.332164551 0.008330277
0.008803431 0.226208384 0.031164984 -0.463253701 -
0.183705198 -0.614046959 0.121427477 0.269402889 0.297790598
-0.281520741 -0.302332322 0.44266475 -0.329551006
0.518743704 -0.186272339 -0.096485779 0.051951832 -
0.409679923 -0.263952252 -0.288062229 0.297752489 -0.074139037
-0.520311699 0.149717282 -0.156097257 -0.431145686
0.032194192 0.458744207 0.084577233 -0.249284649 -
0.205757996 -0.20648933 -0.048360081 0.085998182 -0.057811743

-0.419651034 0.258331023 -0.281966627 0.301797042
0.078357464 0.067121961 0.259769051 -0.068805967 -
0.485206733 -0.617402294 -0.129307702 0.34368573 0.05342028
0.412479854 0.331441508 0.027786459 -0.275562187 -
0.531187827 0.512989921 -0.388554218 0.163233202 0.116614418
0.181383227 -0.284246022 -0.429832758 0.159577136 -
0.068447158 0.14098856 0.100993689 0.00326052 -0.473890093
0.404560564 -0.287554167 -0.004969651 -0.20343236 -
0.260030646 0.185616352 -0.066620981 -0.18655903 -0.22698309
0.125007943 0.436125837 0.31839514 0.143125669
0.467273592 0.163609655 0.116182143 -0.480762901 -
0.44980998 -0.513975398 -0.215589408 -0.359512429 -0.574477622
0.126301266 -0.499398735 -0.266417018 -0.065957418
0.208918009 -0.351027917 -0.004608782 -0.108319425 -
0.085372055 -0.39533454 0.191432164 0.288506526 0.271764264
0.024329881 -0.604659139 -0.020534957 0.305088915 -
0.176319824 -0.290118423 0.30985101 -0.253642809 0.390901697
0.137882215 0.025497522 0.016394928 -0.100109864
0.006659211 -0.258287279 0.260047665 0.163473031 -
0.167070011 0.110164678 0.328199029 0.275741206 -0.505506833
0.325806394 0.26377538 -0.051644753 0.199470096 -
0.184563972 0.296348577 -0.293600931 0.053310566 -0.014886535

-0.055650273 0.413656043 -0.046429104 0.278811655 -
0.405978422 -0.141873743 0.34586841 -0.042553475 -0.269273315
0.285175835 0.094745183 0.358620072 0.353289348 -
0.283187155 0.243564097 0.103927679 -0.388114392 0.400090814
0.228993283 -0.397873219 0.243122568 0.085888446 -
0.595808573 0.108869978 -0.270593162 -0.018372589 -0.307155205
0.252515942 -0.201517398 -0.400311748 0.458443782 -
0.274493052 -0.028373376 -0.09284388 0.246823101 0.319371878
-0.089360967 -0.196637564 -0.53874184 0.092094544 -
0.0701797 -0.23107804 0.054900717 0.118349133 0.153427828 -
0.019607657 0.16981967 0.306917163 0.138727015 0.162734486
0.070554797 -0.268671871 -0.133175959 -0.229009648 -
0.468909103 -0.228734144 -0.047346113 -0.436476192 0.107780589
-0.417264503 0.533369751 -0.316976675 0.002033114
0.293762861 0.141935376 -0.109944216 0.152998097 -
0.176287274 -0.187540912 -0.126984218 0.049069606 -0.527171807
0.263319673 0.373721837 0.28959318 -0.048568318
0.432426364 -0.13279962 0.349653131 -0.313021119 -
0.220048576 -0.450493786 -0.496495227 -0.257069932 0.22630814
-0.542104856 -0.555925743 -0.343085878 -0.457782056
0.295759308 -0.475939736 -0.344262177 0.200970217 -
0.217738683 0.271307797 0.307149983 -0.543561307 -0.574398356

0.362643029 0.018139425 0.072584615 -0.498646749
0.193729018 0.277988227 -0.462541045 0.233767188
0.151531803 0.14769265 -0.142243683 0.442337082 -
0.500086577 -0.375195848 -0.145430385 0.186158684 -0.563380348
0.253085924 0.448280168 0.412549866 -0.373680697
0.313882947 0.306274106 0.668009472 -0.397636909
0.373633416 0.337489441 -0.464405836 0.251570222 -
0.406349945 0.021920987 -0.194936234 0.150360094 0.047483724
0.069389117 0.379759478 -0.209513568 0.100092339
0.077904255 0.385653824 0.441254989 -0.128841828
0.172477066 0.054193975 0.413724778 0.473128003 -
0.29535573 0.051269679 0.077547615 -0.1095758 0.163357065
0.511642370.234131578 0.261197677 -0.13101293 -0.32611792
0.10314317 0.20332391 0.44862246 -0.489882501 -
0.214218403 0.231014986 0.466780578 0.216630221 -0.127023001
0.18673435 0.225413071 -0.476027245 0.018344583 -
0.359278535 -0.138258373 0.10355083 -0.330660085 -0.001934665
-0.290255571 -0.04352036 0.462299402 0.018468065
0.270325306 -0.334665241 0.088757759 -0.160951413 -
0.367249297 -0.072244224 -0.336228216 -0.196055395 -0.418785129
-0.427426489 0.369119694 -0.022962513 0.176720792 -
0.110365519 0.319579537 0.561263771 -0.157303719 0.074849812

0.0380365 -0.033494877 0.023677371 0.206306353 -0.134630348
-0.131716262 0.110687238 0.219486229 -0.272963994
0.095253229 -0.017699301 0.183733245 -0.116188849
0.151133021 -0.093707591 -0.0318588 -0.013416216 0.277315014
0.490902275 0.034282982 -0.340743 -0.488599137 -0.37874581
-0.013711917 0.204762389 0.49162937 -0.103044443
0.260353998 -0.146637026 0.092261689 0.296346263
0.065464441 -0.461196066 0.429696459 0.038349737 -
0.150376821 -0.325759117 0.274092085 -0.124480791 0.429174851
0.012796265 -0.299414147 0.154465358 0.001956655
0.279469857 0.369167442 0.398599098 0.244481908
0.31224179 0.155764154 0.033083342 -0.503856522
0.215187994 0.253994933 -0.166899796 -0.496071394
0.278764257 -0.481039733 -0.168268045 -0.220841628
0.55661821 -0.44006127 -0.12136012 0.334004366
0.070549627 -0.314676569 0.050547161 0.379965137 -
0.335089745 -0.212618278 -0.597199825 -0.427326143 0.089103801
-0.102192475 0.390440791 -0.391247718 0.38669033
0.142234435 0.003192126 0.231571559 0.377261283 -
0.404298134 0.064985294 0.366948972 -0.116460437 -0.301714217
-0.1630748 -0.096531767 0.224552975 -0.063878615 0.299332252
0.277759213 -0.23030966 0.158447249 0.216534001

0.409068594 0.313577014 0.476843395 -0.275511219 -
0.379688077 0.079964842 -0.201139045 -0.001877293 -0.006083081
-0.012687593 -0.413879705 0.512390427 -0.428837302
0.329848333 0.084341638 0.251945319 0.011293634
0.124095268 -0.121093035 -0.572328746 0.148832543 -
0.441177949 0.020670388 0.145203126 0.373896536 -0.266279709
-0.236972326 0.204848009 0.251541529 0.172923895
0.37658852 0.038258377 0.201531215 0.380388138 -
0.400511967 0.125425809 0.263502817 0.63219726 -0.04785365
0.286855662 0.306953031 -0.062324601 0.279860764 -
0.311899196 0.486738441 0.214371429 0.06610909 -0.094988016
0.081018905 -0.272374517 0.300327952 -0.18752985 -
0.031557829 0.029645419 -0.100335542 0.074333301 -0.463338423
-0.145772991 -0.241527681 -0.016963584 0.158048015 -
0.155783855 0.30732446 -0.265415799 -0.073999679 -0.094366975
-0.384742643 0.337119836 -0.238278673 0.402445494 -
0.424415571 0.025877947 0.17976211-0.223757849 0.28387393 -
0.398604052 -0.05225332 -0.106701122 0.416868213 0.011164579
-0.103245188 0.262927899
KEGG_GLYCEROPHOSPHOLIPID_METABOLISM -0.059944983 -
0.011528849 -3.58E-02 0.239198486 0.153537802 -0.052636277 -
0.075631532 -0.039763011 -0.031163207 0.146926663 -0.048612554

-0.008513974 -0.227852798 -0.099347977 0.052975886

0.18881355 -0.112877859 -0.056183241 -0.044002222

0.077159651 0.116787358 -0.041206835 -0.08560618

0.001939816 -0.116238176 0.017243317 -0.016332551

0.019846738 0.209588618 0.154521824 -0.181980871

0.176075166 0.15720224 0.115186178 -0.427232124 -

0.325927894 -0.24734484 0.083146896 0.136308657 0.09062879

0.155325404 -0.227994223 0.147507611 -0.188159238 -

0.05488693 0.037284283 0.216970306 0.060455299 -0.246091289

-0.137080176 -0.008249797 -0.151242084 -0.295726027

0.303966913 0.141256346 -0.002779974 0.170188734 -

0.20746713 -0.1431339 -0.179305997 -0.328494061 -0.275980536 -

0.354194801 0.121716502 -0.036449451 0.07805829 -0.205381146

0.033897191 -0.09856339 0.227503223 -0.133694345 -

0.125541165 0.010754971 0.070517993 0.117816772 0.113031308

0.09625009 0.244324671 0.09314346 0.072651376

0.069034785 0.031647171 -0.243932588 -0.26079668 -

0.192885785 -0.016687233 -0.176454952 0.233871301 0.073478946

-0.014848692 0.218127043 -0.236022732 0.241466694

0.054145108 0.293303147 0.110445927 -0.045607489 -

0.073997587 0.086767613 -0.004284196 0.176879443 0.01444823

-0.118464648 -0.051068776 -0.213993613 0.024827178 -

0.092373373 0.090859505 -0.033919366 -0.021076345 -0.168459023
-0.120584964 -0.240924578 -0.133865276 -0.027399147 -
0.086256695 -0.113064117 0.094493414 0.09320117-0.044020589
0.153098445 0.180539119 0.049786929 0.059627291 -
0.015985333 0.081432704 -0.055872356 0.117914065 -0.163077018
-0.09772204 -0.119297606 -0.204620529 0.126944505
0.154106527 -0.181004232 -0.128798207 -0.163165889 -
0.077225806 -0.011964014 0.013684428 -0.141377837 0.061019734
0.019620929 0.011593077 -0.310706988 -0.196581764 -
0.043763171 0.171507719 0.095313404 0.023107602 0.099815487
-0.144673482 -0.358619427 -0.195183758 0.097271678 -
0.27262828 -0.176248018 0.299777016 0.133661623 0.076696833
-0.195448121 -0.032624654 -0.044985594 0.018427307 -
0.074581886 -0.059605848 -0.019162582 -0.1201809-0.061939087 -
0.109381572 -0.070759755 0.18794097 -0.039305026 -0.003472115
-0.070778887 -0.142930306 0.109605617 0.004987795 -
0.099627628 0.100238634 0.080785434 -0.000378009 0.197483297
-0.332501021 -0.004937102 -0.041098424 -0.043468822 -
0.09630743 -0.146298457 0.193422273 0.055858982 0.101000523
0.162375332 0.034153641 -0.097482064 -0.166404885
0.172369485 -0.076795922 0.137631444 0.255569794 -
0.303704786 0.117830082 -0.211039298 0.144227106 0.095572243

0.029291193 0.215811049 0.064865689 -0.01475227
0.115969065 -0.044702517 -0.067765201 0.140204205 -
0.112714424 -0.019711275 0.278214296 0.005224008 -0.092721387
0.020541739 0.076774897 -0.159187017 0.002863136 -
0.01516828 -0.057367255 -0.037715609 -0.186207141 -0.089381779
-0.026984075 0.064092423 0.098366023 0.250284491 -
0.229562371 0.178918473 -0.1956716 0.301370927 -0.068736276 -
0.135914254 0.018014331 0.093934171 -0.229238097 -0.029998957
-0.216026464 0.115546569 -0.160211024 0.005299517
0.047682845 0.231122010.047135956 0.169861879 0.205452323
0.127053417 -0.060008031 0.183011036 -0.187498841 -
0.009999998 -0.241854479 -0.048792819 0.021034747 0.160521833
-0.039402515 -0.054512204 -0.007982952 -0.060229375
0.315244995 -0.329978472 0.001069681 -0.191285319 -
0.119681877 -0.021163726 0.051637994 -0.022545754 -0.166991345
0.013802727 -0.167868844 -0.208315202 0.052162138
0.020252559 0.083488824 0.139202938 0.107175044
0.05325879 -0.131167194 -0.036281256 -0.006691907
0.007007136 0.124007539 0.155280585 0.060310988 -
0.353395747 0.044844887 0.111394444 0.077933157 -0.068994886
-0.230970483 -0.040113519 0.015788756 -0.039842248
0.189186046 0.088821476 0.077534905 -0.024987087

0.237463805 0.082668917 0.036521174 -0.024325904
0.048933452 0.024010877 -0.069536941 -0.180232357
0.17270304 0.196445266 0.049396054 -0.067325923 -
0.004288753 0.044191633 0.071004178 0.124522593 0.047463879
0.143809345 -0.138742944 0.124346301 0.098180934 -
0.261064365 0.108654287 0.033324379 -0.198915276 0.079765288
0.221525355 -0.178056561 -0.179486061 0.055170824 -
0.168219093 -0.057260129 0.176807806 -0.164711007 -0.107450293
-0.058999457 -0.155588282 0.212434213 -0.081174996 -
0.024756969 -0.127932703 0.152614652 0.169865994 -0.021467172
-0.078817905 0.007860054 -0.035495244 -0.025595364 -
0.129117883 -0.054439041 -0.251190828 -0.088216199 0.123355964
-0.029264899 0.010830587 0.33185939 -0.148471822 -
0.022223066 0.204043057 0.15443883 -0.007456869 -0.05464812
-0.129084083 -0.294128498 -0.03942062 0.269184876 -
0.222218322 -0.03761073 0.050640346 0.077173993 -0.129974971
-0.002092094 -0.094643467 0.178693716 0.133053275
0.106417147 -0.273543424 -0.170823217 -0.119869593
0.192819788 0.084118342 -0.069165354 0.009965279 -
0.137094646 -0.10395113 -0.170332103 -0.320928272 -0.293680565
-0.140282707 0.056946887 0.109267678 0.049336767
0.002325144 -0.243149643 -0.152090912 -0.028610528 -

0.162054109 -0.011962248 0.016012386 -0.017671743 -0.203780189
-0.246980502 0.232468985 -0.000382823 0.051650118
0.161522709 -0.221983462 -0.124284842 -0.167702761
0.01677448 0.16990222 0.300080801 0.058732164
0.006802666 -0.120510649 0.108794951 0.117640785 -
0.203055966 -0.046093402 -0.062986722 -0.299938485 -0.062951053
-0.246913394 -0.167837881 0.129075916 -0.007451421 -
0.111265348 0.046792841 -0.158945015 0.261565026 -0.049690361
-0.148000457 0.231184172 0.070206613 -0.135160473
0.037686944 0.186620464 -0.189008447 -0.076783346
0.110523297 -0.000130788 0.189504275 -0.101414939
0.119180029 0.215782038 0.01708026 -0.013282276
0.155726977 0.251322943 -0.060607527 0.234494868
0.158416471 -0.249720492 -0.200188805 -0.044252315 -
0.273910999 -0.028202113 0.048714979 -0.144011898 -0.185028541
0.132772707 0.059250042 -0.038797496 -0.102800845
0.10657767 0.213943781 0.080100777 -0.108132461
0.25652071 -0.155648028 0.276803551 -0.037215629
0.013358654 0.033387646 -0.062928236 0.072994095 -
0.135137405 0.058491462 -0.105852338 0.100963082 0.040643836
0.095800346 0.085845825 -0.075033617 -0.02220775 -
0.062650473 -0.021977455 0.070603765 -0.142753183 0.232901577

-0.398664836 -0.248377166 -0.044384461 -0.164718671
0.16431515 -0.046992926 -0.09869289 0.167581241
0.09951794 -0.001844909 -0.084290986 -0.261334861 -
0.191139287 -0.107750744 -0.225482342 -0.097892278 -0.148831741
0.027052645 0.039876492 0.086954743 -0.278972529 -
0.207721809 -0.050282312 0.149123859 -0.138633575 -0.15608092
0.278205833 0.140796714 0.251292859 0.012359962
0.032268028 -0.105450371 0.084595851 0.002466385 -
0.139040823 -0.052852045 0.192192396 -0.108254546 -0.030920813
0.02403039 -0.024971228 -0.130740947 -0.000340933
0.040294971 -0.199038404 0.180101036 -0.110636861
0.007588062 -0.105366745 0.108682454 0.054341118
0.080076396 -0.141935389 0.052443807 -0.027502054
0.144712214 0.065613434 -0.215740493 0.01522977
0.012481117 -0.120421422 0.183952948 0.044788935 -
0.008223307 -0.193727832 -0.071056843 -0.031605799 0.260801513
0.027079761 0.339711679 0.130780203 -0.250129467 -
0.149461976 0.095079747 0.026918418 -0.25555306 0.035699618
0.180071773
KEGG_ETHER_LIPID_METABOLISM -4.60E-02 0.181522975 -1.46E-01
0.158660369 -0.114489051 0.364950077 0.122200023
0.256760865 -0.122201511 0.080442609 0.001731081

0.108243957 0.102931008 -0.378465378 0.187476474 -
0.172962047 0.05169092 0.068549003 0.022985019 -0.15563388
-0.181606777 0.123741641 -0.24380456 -0.236861953
0.063799812 0.334973979 -0.142669164 0.148554033
0.483956956 0.115070061 0.061824839 0.217431764
0.319120737 0.19751337 -0.275010886 -0.307594253 -
0.140412473 -0.086242403 -0.051965269 -0.182881885 0.279021122
-0.33924952 -0.300914952 0.111850806 -0.062040709 -
0.086146663 -0.301595986 0.114270959 -0.09919118 0.084586167
0.181846516 0.034732111 -0.251826348 -0.142303331
0.264474402 0.156140933 0.110569959 -0.147349964 -
0.12874984 -0.137522477 -0.473186588 -0.214991702 -0.235349695
-0.17100212 0.076176831 0.116725962 -0.30344472
0.040086476 0.227423692 0.200271705 -0.132321873 -
0.091627525 0.229664063 0.282307368 0.236956956 0.193641098
0.277681812 0.073438615 0.126665054 0.145864331
0.158610383 0.304705934 0.138379966 0.110488177
0.171888362 0.027509062 -0.483833907 -0.007932976 -
0.142376226 0.357508415 -0.174362524 0.06237167 -0.074077954
-0.011138126 0.181049489 0.019403125 0.118789704 -
0.077404689 0.294963904 0.171900702 0.101961824 0.151249325
0.222285858 0.145853778 0.105814369 0.122185189

0.254037047 -0.019786812 -0.099242225 0.238571503
0.072544299 0.13540796 -0.29875972 -0.197914758
0.081195475 0.144534098 0.008768466 0.009908184
0.215536947 -0.314893478 0.123611971 0.423117522 -
0.255243202 0.033496484 -0.030491997 0.350703364 0.055833015
0.012325425 -0.079773811 -0.012126798 -0.091060027 -
0.25035568 -0.176648078 0.021227399 -0.164697941 -0.371317613
-0.198977776 -0.050131384 0.001495999 -0.134245521
0.060234616 0.342398303 -0.21931192 -0.08554093 -
0.054782187 -0.368473398 0.187054093 0.288764857 0.271256737
-0.040116631 0.206714906 -0.104552736 0.023881289
0.128950198 0.051363519 0.117254875 -0.112686575
0.450453034 0.23226973 0.100445134 -0.051380086
0.017082891 -0.071285725 0.045138611 -0.181634946
0.161838279 0.060880241 -0.027236277 0.091739323
0.207408173 -0.292527284 -0.079251089 -0.088676144 -
0.078983419 -0.104310961 -0.253037313 -0.011601161 -0.128158461
-0.348982797 0.100858801 -0.046224747 0.140364106 -
0.051206197 -0.142821024 -0.296209076 0.090633312 -0.407968876
0.204507398 -0.041512773 0.167324786 0.120574053
0.389295995 0.203539592 -0.189046224 0.018407158
0.248921292 0.378382379 0.216703444 0.171919312

0.02594166 -0.192123529 0.273835846 0.224352987
0.28403664 0.322615243 0.222462932 0.02649834
0.102343764 -0.316515268 0.174646901 -0.015932905 -
0.091034407 -0.024351749 -0.273273895 0.09190942 0.125668272
-0.047083836 0.080385081 0.271531465 -0.002606534
0.190143391 0.005491153 0.144299366 -0.230117716 -
0.030279197 0.283883985 -0.146439705 0.151292007 -0.111429665
0.401112329 0.073249326 0.223825382 0.088245494
0.240109983 0.387174376 -0.046072083 0.026397348 -
0.084025773 -0.244838284 -0.098388235 -0.086505082 -0.042681092
0.215042748 0.026520246 -0.142519307 0.236626791
0.394100131 0.020840016 -0.203799974 -0.020643588 -
0.007104217 0.09430136 0.153017099 -0.462590864 -0.088136998
-0.086745435 0.161575337 -0.214898005 0.138691959 -
0.226175336 -0.362383822 -0.002940193 0.108812655 0.051366298
-0.315175465 -0.273394552 -0.183606957 -0.082893389 -
0.316614735 0.338054429 0.259660258 -0.374442115 -0.112717376
0.030431306 -0.425908925 0.357085212 0.157063127
0.175356295 -0.045698018 0.20729067 0.084339893
0.060742182 -0.232748616 0.173082539 0.164947034
0.113302706 0.015483104 0.225636529 -0.078095773 -
0.175150324 -0.372698132 0.212954673 -0.201713316 -0.288435721

5994 0.338226205 0.087321582 -0.108420945 -0.035420034 -

5995 0.255164929 0.160855983 0.071447984 0.20555761 0.085663513

5996 -0.024508297 -0.165994293 0.160941346 0.334459351 -

5997 0.048859783 0.011809616 0.09258939 0.239249168 -0.105731283

5998 0.044449679 -0.016828841 0.174142732 -0.249539052

5999 0.105927991 -0.226862862 -0.195385625 -0.199696445

6000 0.192074668 -0.331353028 -0.20678611 0.395134666 -

6001 0.015896216 0.109586241 -0.059941045 0.133500805 -0.140551052

6002 0.078077368 0.227127376 -0.233531647 0.218589217

6003 0.371531557 -0.20293376 0.06754437 -0.098906021 -

6004 0.004258897 -0.080700567 0.044611380.036083798 0.141682853 -

6005 0.050531462 -0.087525276 0.036021837 0.292553733 0.20595942

6006 -0.127539461 -0.0754843-0.364145265 -0.34917034 0.196728877

6007 -0.441882434 0.04881791 -0.187836114 0.162552577

6008 0.312767872 0.127792419 -0.107639802 0.029601069 -

6009 0.058782218 0.036007657 -0.043518816 0.309076277 -0.055415491

6010 0.014036581 0.166284945 -0.191775513 -0.054535235 -

6011 0.024386873 -0.006981251 -0.297261227 -0.066365469 0.017067926

6012 0.065236102 0.115320739 -0.093791046 -0.081167153

6013 0.210466792 -0.203967942 0.062658386 -0.139812231 -

6014 0.254673352 -0.171462542 -0.039572325 0.196081867 0.08567645

6015 -0.051756209 -0.322135855 0.168469822 -0.079585884

0.079560184 0.150235543 0.235956254 -0.171515963 -
0.13616658 -0.008828456 -0.149418336 -0.151758423 -0.09475629
0.256829381 -0.10858321 0.132346681 0.204663262
0.024679348 0.067356816 0.193901428 0.101238077 -
0.133189696 0.244557209 0.027484669 0.161220037 -0.092514678
0.148733744 -0.112293689 -0.187666855 0.295077301
0.224632665 0.07791698 -0.019415673 -0.056025238 -
0.191932701 -0.357181132 -0.047773177 0.346899823 0.107705572
0.269810867 -0.224090744 -0.004072255 -0.215468284
0.189569426 0.090826725 -0.184729209 0.200356052
0.194395722 -0.103076843 -0.18150525 0.322996661 -
0.311231472 -0.455112922 -0.121402737 -0.100760656 0.381950617
-0.078685299 0.180719762 0.093822379 0.263719543
0.079823096 -0.274797715 0.417027362 -0.083881856
0.074140858 -0.052555804 -0.017177834 0.074296742 -
0.249361661 -0.461758846 -0.340409021 -0.266508176 -0.250386887
0.085445468 -0.048766821 0.096407379 0.087978297 -
0.235115244 0.209077958 -0.036696616 0.178791005 -0.327066572
-0.083344011 0.167445983 0.382390923 0.167056241
0.000694974 -0.305994668 -0.426013459 -0.057267356 -
0.011107724 0.193404146 0.168995651 -0.34307338 -0.01712968
0.142203544 0.316935567 0.122249594 0.319723502 -

0.149720139 0.146117431 -0.11116213 -0.418619693 0.244712633
-0.096845143 0.11956191-0.248269755 0.180835336 -0.027999179
0.221357927 -0.121884788 0.134406436 0.207901119
0.050672614 -0.120709445 -0.232039299 -0.257646023 -
0.336780181 0.044092533 0.035682671 -0.033049878 0.432340864
-0.016819245 0.247898241 0.029024335 -0.174423936
0.211003656 -0.086477326 0.063541136 -0.255074308
0.057488815 -0.114610608 -0.259402011 -0.18504404 -
0.222356922 0.124979814 -0.121249006 -0.029990643 -0.266794971
0.089483127 -0.164489466 0.171197109 0.087417483
0.138965736 -0.198009174 -0.320591376 0.146382854
0.053709485 -0.15474478 0.181577294 0.031029721 -
0.248227121 -0.001772614 0.236294916 -0.195764867 0.276939868
0.111592263 -0.093617903 0.02652701 0.172020331 -
0.055032411 -0.228236381 0.178526217 0.120759528 0.098411067
0.144598059 -0.079245207 -0.365489597 -0.304753513
0.006818752 0.203592976 0.425917187 0.206312061
0.416154857 0.16804582 -0.133401919 -0.200940582
0.363832712 -0.089467058 -0.113950239 0.173625452
0.010665261
KEGG_ARACHIDONIC_ACID_METABOLISM -0.251788383 0.032350448
-3.33E-01 -0.34285084 0.46471616 0.283703569 0.044927067

0.423982924 -0.254370484 0.06729896 0.154922097 -
0.151567455 0.265043538 -0.224217788 0.432854624 0.224697665
-0.337143068 0.046809262 0.309288034 0.046818647 -
0.343506526 -0.243783426 -0.307134901 -0.581822576 0.163563627
0.29978621 0.354742294 0.007538246 -0.237115612
0.327223984 0.084139011 -0.096045365 0.097429516
0.450242823 -0.119635796 -0.402001833 -0.012359218
0.386080984 -0.151555284 -0.190140313 0.070881275
0.245702317 -0.428264452 0.051819134 -0.276966681 -
0.010729763 -0.235277924 0.052847958 -0.100364101 0.202180291
0.081727038 -0.350168684 0.019060829 -0.214787424
0.091105256 -0.046940956 0.357262494 -0.054538433 -
0.168597816 -0.250777101 -0.362084902 -0.036769064 -0.084095737
-0.289529318 0.218377491 -0.157041844 -0.005412446 -
0.193822239 0.32988513 0.274237958 -0.33075608 -0.048610357
0.139661216 0.131258273 -0.143622635 0.505172629
0.355599726 -0.28983972 0.189201453 0.130586838
0.169721303 -0.266388516 0.160215268 0.096527544 -
0.030349812 -0.222696589 -0.507024064 -0.205887506 0.294576807
0.037224478 0.141105811 0.412678916 -0.093829403 -
0.213550681 -0.154899512 -0.116834505 0.016800453 0.209502718
0.417365278 0.094164942 0.304356727 0.213652749 -

0.268028634 0.408627231 0.069076777 -0.110808549 -0.029914856
0.058980119 -0.376646065 0.421421308 0.288621298
0.300692392 0.019747778 -0.230183822 -0.33165765 -
0.389294913 0.126200451 0.259024426 0.282086273 -0.351513792
0.441785017 0.226775602 0.032674525 0.116618403
0.007437762 0.483656557 0.219056908 -0.341010334 -
0.063870459 -0.298691115 0.039969309 0.03129145 -0.156723028
-0.243579245 -0.170362281 -0.402594073 -0.257195898 -
0.079798935 0.207316015 -0.373106424 0.267375781 0.280536486
-0.437443464 -0.012230594 -0.241233455 -0.219201974 -
0.054818097 0.399062056 0.410820231 0.369339406 0.443454376
-0.303470515 -0.124120497 0.462983266 -0.225032429
0.439497726 -0.17032891 0.445102179 -0.071177987
0.237655826 -0.288159471 0.306860245 0.042320298
0.025484961 0.073490652 -0.047158584 0.084191144 -
0.165537857 0.49219638 0.013733956 -0.405065906 0.347428093
0.077388853 -0.027897713 -0.358855112 -0.388236306 -
0.451226104 -0.163983521 -0.482143405 0.254247718 0.354583814
0.030514252 0.121873789 -0.019553662 -0.475921705
0.133997033 -0.393608287 -0.368886967 0.183843683 -
0.066155381 -0.035234722 0.512874931 0.282597989 0.252048325
0.058874289 0.122691577 0.119426783 -0.073054837

0.063358648 -0.257440347 0.223828462 0.138009895

0.094755765 0.489701283 0.04468109 0.178385233

0.071313264 0.051704086 -0.394359323 0.181489808 -

0.00665257 -0.254850674 -0.083885117 -0.293452932 0.504357229

-0.23614745 -0.16951803 -0.041805565 0.000711832 -

0.233264871 0.186186963 -0.012016627 0.337837173 -0.428970106

-0.061501294 0.251490903 0.011215910.44844072 -0.406680909

0.583977661 0.325202436 -0.209092233 -0.148983409 -

0.023199413 -0.024727412 0.050380552 0.200539319 -0.328028749

-0.286325317 0.248789225 -0.114102259 -0.21586975

0.108600785 0.026510986 -0.273331234 -0.307576371

0.342159651 -0.172802259 0.130740293 0.316909196 -

0.150139021 -0.065729075 0.325958104 -0.483252361 0.367845775

-0.01293018 0.176420642 0.363968236 -0.198490451 -

0.08172082 -0.01958646 -0.076861655 0.136476324 0.402866923

-0.116834926 -0.293653099 -0.1198587 0.112137186 -0.136070942

0.599415814 0.263058095 -0.430566834 -0.058763784

0.129400124 -0.162210362 0.444091346 -0.149582447

0.289982687 -0.081872382 0.343749874 0.39547012

0.180501842 -0.272365879 0.34844423 0.420914338

0.466022543 0.380129103 -0.02894756 -0.10186211

0.197182491 -0.215539931 0.473976351 -0.315809025 -

0.491116082 -0.007478493 0.368644027 -0.3477326 -0.01510704 -
0.044129248 0.392649589 -0.351014254 0.12584835 0.520997925
0.365329761 0.363721418 -0.167845893 0.048646834 -
0.233065219 -0.097275829 0.351865648 0.204696177 0.310267629
0.300058131 -0.377460402 0.248742662 -0.233718033 0.3551517
-0.186943767 -0.356427333 -0.447793451 0.333408097 -
0.22000171 -0.197758231 0.522061859 -0.067481777 -0.130380191
0.043470915 0.421345579 -0.002650902 0.110106147 -
0.177028773 -0.177393139 0.074731217 0.403339689 -0.533240488
-0.218756095 -0.168577004 -0.232812715 -0.355838683 -
0.163989668 0.104930584 -0.243673025 0.17952559 -0.308090652
-0.09890777 0.366832543 -0.415676722 -0.286814858 -
0.00946685 -0.245131967 -0.297899329 0.229932194 -0.501841625
0.050441982 -0.17379902 0.299586472 0.350443982
0.131400612 -0.01313253 0.398310576 0.021368395 -
0.063886136 -0.39753368 0.010318491 -0.21212384 -0.023328242
0.15692675 -0.448103834 0.076647262 -0.4024308 0.02277841
-0.518255348 -0.511275208 -0.05749398 -0.147428886
0.187523257 0.148937627 0.401103965 -0.045130549 -
0.260301404 0.258816873 0.256842873 0.013318363 0.174114956
0.096438372 -0.043633615 -0.145357113 -0.030778269 -
0.372927774 -0.217703436 0.196874119 0.237231414 0.074385321

0.355957587 -0.299799204 -0.084710983 0.127889353
0.095764309 -0.315028481 -0.208822106 0.037812034 -
0.304936024 -0.411230971 0.098921076 0.231540552 -0.097178131
0.013274741 -0.307848919 -0.156808906 -0.394327088
0.070842584 -0.329241649 0.476991792 0.066671241
0.561291151 -0.054559714 -0.048624897 0.292126991
0.241575699 -0.200139981 0.168858446 -0.300925984 -
0.362658494 -0.357415589 0.396698239 0.225377043 0.457284077
-0.044931558 -0.054721337 -0.371218131 0.205207602 -
0.049638785 -0.051395376 0.33546338 0.106061706 -0.262330255
-0.286528937 0.030946896 -0.356495931 -0.340563449 -
0.458599862 -0.249881052 0.038412691 0.305287737 0.139771313
-0.138287075 0.337846818 0.10493167 0.033616305
0.090414783 -0.270377732 0.132363045 -0.168036997
0.409894558 0.307823016 -0.192127183 -0.282593262 -
0.049209847 -0.305956225 -0.112547185 0.205995046 0.129440107
0.250107287 -0.208809423 -0.48071008 0.109367068 -
0.405036803 0.280692696 -0.327234668 -0.20348398 -0.079519981
0.285431599 -0.01277036 -0.416495965 -0.003953678 -
0.380060218 0.015179852 0.023355702 -0.023688022 -0.060120255
-0.511695217 0.433273497 -0.003060351 0.374896798
0.324810663 -0.177803752 -0.385075343 0.409790904 -

0.053182406 -0.399950844 0.335065071 0.074811997 -0.330859872
-0.344525014 0.177733497 0.245782503 0.324785467
0.414099007 0.014910375 0.510374557 -0.171829694 -
0.251219418 -0.134453077 -0.265331648 -0.354925612 0.093134987
0.380644125 -0.114432749 0.527585629 -0.384191126
0.013104229 0.103147415 -0.183293234 0.233067977
0.153394783 0.124956403 -0.188947592 -0.015885964 -
0.120901439 0.035869761 -0.142304548 0.09624924 -0.336076951
0.256898967 -0.379983483 -0.255035393 -0.332167768 -
0.082363634 -0.47897005 -0.542070676 0.335095937 -0.245116865
-0.180281346 -0.137066275 0.26989157 0.131365733
0.082017807 -0.050799706 -0.279833686 -0.102573872
0.194146462 -0.136195151 0.112286724 0.137071126 -
0.018105996 0.206660476 0.096726987 0.045741806 0.147780663
0.15817349 0.092238887 -0.022163906 0.246414913
0.007646672 0.058581324 0.085628388 -0.139231293
0.05763306 0.055790983 0.03853463 0.134120527
0.159997515 0.062889495 -0.006608898 0.003475412
0.240318884 -0.167448588 0.043128876 0.078044901
KEGG_LINOLEIC_ACID_METABOLISM -0.504302375 -0.306103863
1.12E-01 -0.234671303 0.497268697 0.379069263 0.188657758
0.487971679 -0.347862996 0.300224061 0.304204586 0.2313261

0.20657627 -0.093178909 0.455888609 0.25411067-0.515462031
-0.128303541 0.526580506 0.014439456 -0.363913023 -
0.309742452 -0.355970516 -0.566643043 0.210812464 0.421709564
0.368802642 -0.021374681 0.196279601 0.552577075
0.154599369 0.164387958 0.140835816 0.526000817 -
0.470398898 -0.293614027 0.447960352 0.221309432 0.08210656
-0.075875397 0.365968685 -0.329199619 -0.393362388
0.188432212 -0.111406675 -0.287883457 -0.159489959
0.29936648 0.180248417 0.360499661 0.372323672 -
0.157890284 -0.509349852 0.005885121 -0.393096615 -0.285292058
0.592712951 -0.292505995 -0.453030624 -0.365509117 -
0.40078022 -0.172215018 -0.383201785 -0.321757066 0.284792546
-0.107120055 -0.463350804 -0.468239469 0.404319215
0.540314451 -0.41342491 -0.103808082 0.296386186
0.263343461 0.215202625 0.597919981 0.495742755
0.119368770.073041492 -0.095426323 0.156468644 -0.235335836
-0.12620736 0.018027429 -0.057708023 -0.159421746 -
0.598854948 -0.128982669 0.505507143 0.254570946 0.018858326
0.340236439 0.201767873 -0.478525799 -0.112059174
0.053912829 0.330611195 0.344830969 0.153723242
0.035448418 0.164043699 0.577052399 0.138408154
0.096011064 0.149306843 0.104110662 -0.014732538

0.311874951 -0.391721078 0.580660189 0.482551645
0.077917459 -0.391397822 -0.356674822 -0.106510084 -
0.317821798 -0.088367702 0.516595891 0.624835088 -0.309265139
0.540427692 0.201445134 -0.088101972 0.322721902
0.285937874 0.532956976 0.322592542 -0.305569632 -
0.425832956 -0.284797702 -0.012174351 0.303624216 -0.274560488
0.217325431 0.013517498 -0.534225398 -0.439893517 -
0.390329987 0.023402157 -0.391928484 0.459072872 0.401549676
-0.538996328 0.180212445 -0.295980763 -0.382845799 -
0.174924258 0.503264464 0.550907749 0.155770327 0.321202218
-0.352757757 -0.193030821 -0.069723422 -0.08120296
0.411039564 -0.093692838 0.6493656 0.066194944 0.121689779
-0.387839774 0.242936086 0.061312891 0.277811074 -
0.341350568 0.001883803 -0.301281309 -0.363910544 0.628890472
0.307062341 -0.44870598 0.543596017 0.275377144 -
0.189798505 -0.438077441 -0.534591411 -0.395698442 0.0915187 -
0.466933591 0.202711713 0.139944296 0.331627593 0.264148049
-0.403181027 -0.517634181 -0.048529248 -0.203365836 -
0.007554643 0.399480484 -0.189738683 0.023764768 0.462913652
0.398120588 0.191150566 -0.227587694 0.062582594
0.311589253 -0.205401406 0.436952067 -0.199085829 -
0.066193711 0.098516324 -0.046204078 0.623343027 0.137441541

0.095615225 0.28359854 -0.24550445 -0.380700683 -
0.089318921 -0.053283897 -0.031826331 0.176804456 -0.100040137
0.631910792 -0.371528259 -0.208932633 0.028229826
0.130409308 -0.155252483 0.104469176 -0.159030548
0.382779911 -0.565955629 0.018030311 -0.016637385
0.051754398 0.599850732 -0.328902271 0.418056783
0.507659468 -0.177310273 -0.050808823 0.263720183
0.462015076 -0.210729518 0.291781855 -0.134489834
0.11800363-0.001854776 -0.354756686 -0.534033896 0.091373313
-0.390623373 -0.219493146 -0.174219622 0.488311430.215518952
0.376401201 0.379792082 -0.159597448 -0.25887598
0.449329745 -0.514469289 -0.095643245 -0.351476289
0.146664196 0.342741311 -0.249682292 -0.077074765 -
0.024130317 -0.377488072 0.155833643 0.523792846 -0.242136806
-0.380425564 -0.175223451 0.061482588 -0.400807855
0.647024059 0.30958063 -0.548777277 -0.305098775
0.153719833 -0.346050899 0.225067121 0.19137671
0.216955831 0.079444059 0.19390471 0.525445783
0.109988985 -0.349217229 0.513282837 0.555248056
0.466605991 0.536480463 0.209714302 -0.226483344
0.258858049 -0.209284976 0.398826882 -0.408488315 -
0.515226481 0.501839098 0.43477334 -0.356158168 0.158908366

-0.213172414 0.579013516 -0.209881362 -0.107003443
0.486278071 0.342071719 0.324930261 -0.201126915
0.12904712 -0.334843541 -0.328273283 0.491391561
0.310603379 0.162431763 0.368692265 -0.24061768
0.168300075 -0.263215165 0.491529647 -0.199524532 -
0.498035249 -0.587575619 0.551182475 -0.534173344 -0.577028924
0.592487551 -0.081871342 -0.188343004 -0.215138021
0.544691146 -0.253508445 0.059501411 0.168830461 -
0.233451648 0.039101938 0.230054022 -0.555701382 -0.399915514
-0.375712627 -0.317796626 -0.073674402 -0.275013181 -
0.063839095 -0.251421931 0.436088895 -0.010624393 -0.26149185
0.472967928 -0.289745566 -0.34718507 0.290517233 -
0.297138581 -0.255190813 0.118935591 -0.643825691 0.459177098
-0.159142878 0.388746926 0.549940529 0.043488074 -
0.107442305 0.379395898 0.223321914 0.117414838 -0.256087236
0.036681934 -0.385817143 -0.092467533 0.294534097 -
0.502098574 -0.126910464 -0.131987629 0.096872321 -0.6288443 -
0.51865687 -0.493117634 -0.142069632 0.388566256 0.087747141
0.042863672 -0.290195546 -0.272504655 0.358309312
0.472654012 0.058918618 0.43821438 -0.366872879 -
0.036087675 0.012664172 -0.283782741 -0.562011275 -0.215344891
-0.250393269 0.214984629 0.221628757 -0.044508852 -

0.482868918 -0.341436154 -0.053448419 0.032076312 -0.161352474
-0.349379793 0.452858565 -0.441890245 -0.522691499
0.34626622 0.393947857 0.221234615 0.351965382 -
0.177444526 -0.352242887 -0.289732335 0.281630008 -0.13426462
0.602037127 0.164920917 0.364071184 -0.447717188
0.118623235 0.446618744 -0.167094037 -0.45435135
0.141941598 -0.094531433 -0.485904001 -0.598256293
0.365835457 -0.090066872 0.562537571 -0.393647831 -
0.232295747 -0.509713469 0.174351219 -0.130833692 -0.091605071
0.537110873 0.444310722 -0.350819218 -0.604708195
0.282471486 -0.363581711 -0.529821256 -0.312630271 -
0.317526106 0.306865284 0.470464673 0.222855797 0.191375264
0.299264681 0.390120847 0.101367758 0.498757932 -
0.232559256 0.401939485 -0.034458784 0.225890454 0.234374434
-0.218761986 -0.511366683 -0.24718684 0.106123674 -
0.44067204 0.20796204 0.239104763 0.334615576 -0.008259532
-0.510956576 0.169624233 -0.485413139 0.534856292 -
0.437958951 -0.005879212 0.406104252 0.252355771 -0.394202085
-0.325702697 0.259229712 -0.488696557 -0.010805939
0.000741419 0.056545929 -0.007372056 -0.496925552
0.16960827 0.136036288 0.323908365 0.451374672
0.111492657 -0.354116775 0.518580161 -0.008867485 -

0.578751181 0.61309097 0.32942553 -0.137596275 -0.219918028
0.037811242 0.366616638 0.325077684 0.399391921
0.201652642 0.518380757 -0.382781925 -0.400529833 -
0.492806714 -0.39609128 -0.534078549 -0.103473734 0.359057623
-0.394310915 0.551743605 -0.182638672 0.336030612
0.076577321 -0.298086405 0.473219556 0.018958164
0.160459266 -0.348943515 -0.22466912 -0.255872226
0.350190872 -0.22339477 0.174457127 -0.503186212
0.298729618 -0.426500103 -0.421638178 -0.519861091
0.082405047 -0.513619154 -0.305182231 0.447642219 -
0.136003911 -0.463219135 -0.131622765 0.236208721 0.316802855
0.295814627 0.088460526 -0.29046301 -0.109376004
0.224941632 -0.30550019 0.365030904 0.012634741
0.051487891 0.306394443 0.087424623 -0.233984276 -
0.014611406 0.177407147 0.315307848 -0.142493516 0.277592177
0.085134694 -0.151498276 0.399078795 -0.282235882
0.036525694 0.254199789 -0.020298492 -0.000775198
0.364518161 -0.028622898 -0.123915219 0.341076796
0.060705347 -0.055090916 0.315665169 0.10671718
KEGG_ALPHA_LINOLENIC_ACID_METABOLISM -0.260214249 -
0.040921272 -0.236918195 -0.020525783 0.39268503 0.356480835
0.186417573 0.449759491 0.085754062 0.31307886 -

0.020146467 0.322447403 0.087122338 -0.333081107 0.184118928
0.208735522 -0.355578827 0.226054334 0.450312799
0.053037416 -0.197252929 -0.246759811 -0.134565107 -
0.447094516 0.323863787 0.192936413 0.074654018 -0.02279429
0.203894153 0.232090422 -0.046798306 0.344098573
0.159477487 0.439322999 -0.428019088 -0.247140242
0.134185019 0.108885664 -0.049543628 -0.241754314
0.324743576 -0.18715579 -0.445565864 -0.175110333
0.122581856 -0.037224516 -0.33613115 0.357727277 -
0.124593835 0.354021237 0.408767285 -0.280906145 -0.352931254
-0.044304486 0.2124514 -0.1034474 0.502657302 -0.43762703 -
0.385382164 -0.158947364 -0.311455399 -0.021781975 -0.415417183
-0.071255376 0.077363098 0.119715665 -0.081540657 -
0.309228315 0.221646001 0.26066225 -0.060606205 0.08592362
0.403561186 0.176791489 -0.012895557 0.488745244
0.169648949 0.155254131 0.198337558 -0.119713647
0.08521534 0.127129568 -0.033497736 -0.079949652
0.449164343 -0.010085217 -0.514799334 0.057508986 0.3643051
0.140704537 -0.289034316 -0.061199502 -0.083873815 -
0.198253429 0.171834819 -0.045182539 0.307300237 0.162504895
0.278467857 0.121532559 0.185863568 0.405852954 -
0.056115506 0.233717228 0.341483241 0.195317704 -0.125965287

-0.113932306 -0.213463334 0.269738862 0.150809269 0.04807537 -0.511698077 -0.176411847 -0.326743314 - 0.248798157 -0.108564422 0.1194802 0.341982095 -0.40370921 0.29321325 0.480527373 -0.235277146 0.311469663 0.000371499 0.471034839 0.105027851 -0.223797911 - 0.181177097 -0.095970082 -0.168571594 0.036761007 -0.229972072 0.170633441 -0.369485314 -0.295560365 -0.092516033 - 0.162586254 -0.009011292 -0.177708718 -0.012826703 0.515022774 -0.350203174 0.020916742 -0.174773422 -0.365024321 - 0.0401401 0.230028755 0.475565955 0.325282194 0.362800758 - 0.201989742 -0.182644955 0.087864183 0.076509091 -0.041696751 0.033837818 0.589827005 0.098845805 0.47174722 - 0.136846724 0.058885976 -0.001559043 0.19732251 -0.255328658 0.261247454 -0.246026779 -0.273698019 0.518117590.075883247 -0.419153201 0.123535136 -0.145824617 -0.391931677 - 0.378863228 -0.394389544 -0.263121884 -0.293774424 -0.23932125 0.274469484 0.372449048 0.251164642 0.148631272 - 0.253128094 -0.561078335 -0.039569214 -0.458586995 -0.164501749 0.159603637 -0.191610162 0.195777272 0.35157343 0.318084485 -0.015462239 -0.050425095 -0.13634879 0.16288579 -0.133030908 0.000290492 -0.053754618 0.012461647 0.093713706 0.020886856 0.586362967

0.39452741 0.072467537 0.129175959 0.00016899 -
0.459203843 0.260724668 0.038892568 -0.168298525 0.173330725
-0.361536507 0.560162757 -0.0146993 -0.435185132 0.040707374
-0.03103552 -0.104051202 0.24598259 -0.047384249 -
0.028032833 -0.426577095 -0.221117846 -0.122869445 0.168502799
0.524350858 -0.205986884 0.516714676 0.397222974 -
0.236677075 -0.026825893 0.326159219 0.449600218 -0.215609298
0.189423827 -0.15258176 0.003458017 0.169378254 -
0.11497656 -0.450836058 0.193608541 -0.195061814 -0.243291358
0.092724642 0.576221132 -0.157124808 -0.184252492
0.129208508 0.145933 0.232587243 0.426455188 -0.434842947
0.033581196 -0.14192347 0.127818644 -0.073834971
0.134348593 -0.071273825 -0.255302892 -0.196127839 0.0732426
0.511416587 -0.452788241 -0.268862027 -0.342163511 -
0.17499764 -0.279277219 0.477581641 0.06404858 -0.538806388
-0.452409829 -0.076088777 -0.496621065 0.531231165 -
0.209120841 0.304546805 -0.042223019 0.31268463 0.407320492
-0.040334986 -0.262722186 0.123592374 0.371319353
0.343511372 0.372901495 -0.010340284 0.10792204
0.047265089 -0.393635419 0.264319018 -0.215668243 -
0.338137064 0.342019756 0.249867138 -0.148090561 0.172094432
-0.285737152 0.377832763 -0.077217426 -0.001444015

6390 0.409803576 0.268547724 0.141827139 -0.150438015

6391 0.434812129 -0.114768453 -0.029499998 0.299876672

6392 0.536873661 0.052270988 0.038899088 -0.054683956

6393 0.325470273 -0.370851785 0.465361607 -0.410076436 -

6394 0.438460629 -0.367639746 0.442409449 -0.242400943 -0.658539977

6395 0.576246103 0.264370862 0.04465293 -0.131851376

6396 0.34309509 -0.125479584 0.080823742 0.198170013 -

6397 0.434071884 0.146663157 0.463378107 -0.43718019 -0.138198927

6398 -0.312462157 0.020448847 0.144870128 -0.029314897 -

6399 0.169550548 -0.152979991 0.078359047 0.021375811 -0.037702993

6400 0.280534812 0.004176336 -0.250335397 0.179608682 -

6401 0.515537576 0.02127775 0.056598516 -0.597708115 -0.024209799

6402 -0.331323125 0.18298001 0.604144634 0.21803674 -

6403 0.3280031 0.311593134 0.261000773 0.142406451 -0.426822654

6404 0.222271999 -0.399780707 -0.167633685 0.258064027 -

6405 0.530339807 -0.138620734 -0.055355938 0.256411939 -0.511398305

6406 -0.285613146 -0.228150434 -0.233682314 0.355335914

6407 0.257456503 -0.050674171 -0.054054362 -0.233146276 -

6408 0.030523927 0.167068841 0.015310858 0.076976136 -0.280343978

6409 0.022557754 -0.130305663 0.177906865 -0.469140953 -

6410 0.211533152 -0.374579553 0.108819308 0.228510998 0.368475195

6411 -0.334418763 -0.204810746 -0.085671348 -0.350640368 -

0.004679321 -0.496460621 0.248953664 -0.201846512 -0.316996478
0.055694628 0.209976354 0.052920522 0.262403817 -
0.342458733 -0.329661638 -0.26824495 -0.228249322 0.169902843
0.417216923 0.068258328 0.342635137 -0.492981324
0.219016374 -0.086700527 0.112947759 -0.245424765
0.127284543 -0.591200656 -0.532252116 -0.365132283
0.44461736 -0.145243338 0.475077482 -0.345842569
0.101078954 -0.572675424 0.013703086 -0.219343168 -
0.274452122 0.347119648 0.201377874 -0.16800516 -0.450933911
0.272160784 -0.22954133 -0.575775272 -0.220376576 -
0.070774184 0.302583166 0.240500526 0.103691081 -0.024127087
0.293587803 0.19301488 -0.064140915 0.509617302 -
0.044731501 0.182516816 -0.041874108 -0.075121898 0.039426447
-0.330457663 -0.487707875 -0.210994159 -0.224541264 -
0.531872797 0.033742705 0.288034605 0.300806741 0.003117703
-0.514157617 0.306562493 -0.414844643 0.083113736 -
0.440626034 -0.110317884 -0.099624622 0.040107383 -0.242651083
-0.121520896 -0.126629067 -0.443851075 -0.068050639 -
0.110957996 0.026657004 -0.099704351 -0.375511124 0.244825898
0.092392057 0.390558766 0.176398286 -0.001188139 -
0.240460788 0.133363116 -0.017403025 -0.525635675 0.500924359
0.104331989 0.108266585 -0.269153609 0.253977163

0.079649056 0.266868558 0.171591245 -0.191381012
0.46440143 0.150881797 -0.414643608 -0.27787219 -
0.227423575 -0.2737481 0.018215454 0.182858398 -0.342375022
0.56411504 -0.154349476 0.121498128 -0.309644441 -0.136202603
0.563046219 -0.117159729 0.03986521 -0.432440556 -
0.144536991 -0.342707184 -0.019934674 -0.407641814 -0.221316788
-0.185518516 -0.103617515 -0.053887921 -0.277600613 -
0.078747857 0.169874314 -0.109485006 -0.253396489 0.274808405
-0.321427665 -0.436382458 0.166047521 0.0382964 0.190468823
0.14617066 -0.155370295 -0.181649973 0.094711415
0.132838223 -0.07679678 0.346843199 0.096693753 -
0.187313774 0.431369873 0.168338669 0.049275532 0.043002306
-0.010883385 0.141360077 0.084526386 0.435565886 -
0.063750339 -0.287654218 -0.220581257 -0.083183289 0.133034437
0.391682043 0.270090847 0.528260292 0.264773047 -
0.045748476 -0.128395122 0.407927155 0.20024664 -0.365695684
0.048335635 -0.061184244
KEGG_SPHINGOLIPID_METABOLISM 0.299171724 0.42105777 -
3.73E-01 -0.097247543 0.209667051 -0.296476517 -0.265206297
0.037654206 -0.107819911 -0.004469742 -0.415021004
0.207451623 -0.572408274 -0.319489258 -0.152213808 -
0.029921497 -0.030434274 0.358210452 -0.138904857 0.210188249

-0.245806321 0.29924258 -0.538502117 -0.256324094 -
0.010472078 -0.391385112 -0.228262982 0.290203739 0.016676552
-0.458866008 0.213415666 0.522716382 0.226470369
0.17003968 -0.279189891 -0.070683726 -0.480383369
0.462717731 -0.455492993 0.082951566 0.072648856
0.45285338 -0.236815476 0.286275628 0.000811385
0.264605144 -0.211582974 -0.198167021 0.044185243 -
0.25600839 0.172188192 -0.107224119 0.152468512 -0.349217236
0.352661246 0.421675883 -0.427002367 0.405758621
0.248041008 -0.313393133 -0.162867417 -0.031030897 -
0.034535898 0.105552399 -0.379511419 -0.322771711 0.182011923
0.343420737 0.095189714 -0.217274976 0.332026518
0.125482087 -0.017382665 0.262754543 -0.046436865
0.032125927 -0.012868388 0.131478162 0.246268554
0.363154792 -0.218727708 0.38532427 0.319319841 -
0.558317008 0.084013581 0.117743209 -0.300109822 -0.148461237
-0.047606146 0.104870062 -0.216264215 -0.473632986 -
0.050876469 0.437523767 0.142450239 -0.05781706 -0.130442609
0.105712354 0.292044331 0.401797041 0.45956735 -
0.450667974 -0.068471003 0.331377921 -0.324723196 -0.224348341
0.042507621 0.213226039 -0.442975884 -0.278324909 -
0.410864537 -0.224888896 0.119629753 -0.292692415 0.057746974

6478 0.257446406 0.064213621 -0.31291101 -0.496957813

6479 0.486817282 -0.271340712 0.140164721 0.061428859 -

6480 0.037098966 -0.242004595 -0.158055259 0.065031966 0.118082031

6481 0.100855149 0.498323856 0.099241909 -0.303429075

6482 0.176458254 0.244698309 -0.269708939 -0.238738739 -

6483 0.1490901 0.249511006 -0.070674515 -0.361827983 0.157652181 -

6484 0.004291852 -0.061413418 -0.168962377 0.084650531 -0.141847652

6485 0.235370219 0.437016886 -0.303760134 -0.023721996 -

6486 0.17699048 0.059858485 0.1542219 -0.182503639 -0.172323641 -

6487 0.515657973 -0.302698796 0.439999429 0.249620476 0.290181129

6488 -0.239794333 0.15720776 -0.142277473 -0.292534388

6489 0.175685421 0.13283438 0.413981542 0.415687281 -

6490 0.344686831 -0.290077369 -0.159849364 -0.314190637 -0.370068054

6491 0.036561829 0.111026616 0.044485612 -0.132971712

6492 0.38804183 -0.0820203 0.424709751 0.337446418 -0.101315136

6493 0.022492187 0.107102491 -0.148168371 0.199836852 -

6494 0.118699775 -0.097450411 -0.194980864 0.452788383 0.29689164

6495 0.273731788 0.179787408 -0.568233614 0.173252967 -

6496 0.40992419 0.077787065 0.098625037 -0.462309451 -0.155356132

6497 0.012542561 0.134727805 -0.411870235 0.159645998

6498 0.229474596 0.077616415 -0.266453153 0.132491135 -

6499 0.15747433 0.310802146 0.24795332 -0.032414698 0.276183953

0.024303714 -0.056029683 0.244241915 0.392311856
0.268642144 -0.179848337 -0.111923417 0.093409353
0.301307001 0.203658263 -0.053752682 -0.282585234 -
0.362062694 0.121941022 -0.014274502 0.092160172 0.336562598
0.168148018 0.170995648 -0.259071068 0.043308032
0.03246869 -0.311425863 -0.071954515 -0.314457447 -
0.277129936 -0.150264575 0.276123219 -0.221805755 -0.174759577
0.006455835 0.022256872 -0.029897218 0.183280112 -
0.291008887 0.339213281 -0.458256938 -0.170140286 -0.052090115
-0.31326401 -0.313016809 -0.045192342 -0.41426344 -
0.05689973 -0.386019202 0.446657053 -0.19924327 -0.302666407
0.451546038 0.013398112 0.113200403 0.352459803 -
0.365033701 -0.137647056 -0.157374506 0.122638534 -0.175996821
0.179777944 0.029212082 0.434646147 0.167870385 -
0.015950714 0.254481596 0.352498293 0.171654989 -0.438971189
-0.315854593 -0.439280374 -0.025164334 -0.108513491 -
0.370713012 -0.28216522 -0.096099074 -0.269186405 0.239636134
0.119013592 -0.456500203 -0.237258181 0.268756117 -
0.228987663 0.1035513 -0.467686033 -0.130102963 -0.00389175 -
0.03668299 0.04377774 -0.159286987 0.360411514 0.333399601
-0.069480296 -0.227128873 0.238858043 -0.252876492
0.079388605 0.192047984 -0.378248533 -0.311534373 -

0.262485536 -0.030857515 -0.362721631 -0.09039702 0.39661407

0.438653348 -0.45942023 0.083115424 0.167609176

0.058348375 -0.152555172 0.431372136 -0.198604088 -

0.317277994 0.06024036 0.257133407 -0.11310251 0.064017004

-0.343405415 -0.290840862 -0.061387903 0.0980391 -0.28958938

0.509472699 -0.215442858 0.308647896 0.354527591 -

0.086738159 0.119357138 -0.275340245 0.172760587 -0.081032252

-0.350138661 -0.253710737 0.145504784 0.26175318 -

0.307656799 0.379519534 0.035985181 -0.457949118 -0.358632002

0.21468152 -0.260791344 0.174745617 0.174634041 -

0.27119383-0.354867112 0.377337845 0.229193201 0.181119613

0.329721152 0.1143739 -0.193322533 -0.186210385 0.174897341

0.292386975 0.005783261 -0.188862459 -0.458929575 -

0.2496674 0.029166616 -0.20057252 0.330630491 0.153349349

0.165050028 -0.215275704 -0.337191513 0.205899676 -

0.085073074 0.222107816 -0.492942254 -0.371733039 -0.32181369

-0.230326295 0.252004615 -0.183483323 -0.054137876

0.016206133 -0.049652762 -0.014061831 0.238906867

0.415500953 -0.244056119 0.536711475 -0.260661491

0.085327268 0.284525489 -0.202865578 -0.441102751

0.167616691 -0.224237157 0.215377646 -0.179850212

0.228885329 0.092542653 0.039594559 -0.30547704 -

0.235426223 0.150690058 0.193367976 0.370252038 -0.312324325
0.020971022 -0.412455974 0.057283953 -0.191416837
0.244624475 0.369959098 0.32740319 -0.067279303
0.175450191 0.289721423 0.228467262 0.364607656 -
0.081884818 0.185597847 0.158523191 0.346325471 -0.372033044
0.050554411 0.214073881 0.212919159 0.043137055 -
0.000882521 0.05111249-0.193725363 0.291446234 0.22295219 -
0.292358538 0.000271115 -0.229338544 -0.253707608 0.173427665
-0.446426597 -0.20483311 0.017660819 0.114416272 -
0.249632049 -0.168131986 0.018673801 -0.029069249 -0.436134631
0.10082434 -0.31754754 0.264407721 -0.088734203 -
0.419549662 -0.549346227 0.212532365 0.126957337 0.001794179
0.110050516 -0.196273091 -0.219267716 -0.044560001
0.186134832 -0.2135992-0.241791169 -0.101614064 -0.271586278
-0.172982376 0.447401014 0.447744633 -0.482392851 -
0.21428539 -0.105132428 -0.336908324 -0.158968492 -0.056810269
-0.289136992 0.044653164 -0.115778724 0.374441999
0.410528656 0.036990545 0.241594323 0.111820737
0.48242375 0.095056112 0.004039956 0.057445259 -
0.44930062 -0.043134057 0.011360603 -0.220683021 -0.104498614
0.19092503 0.086922194 0.338664115 0.18484191
0.292507972 0.122493635 0.363682408 -0.332852834 -

0.281314122 0.084815887 -0.18192055 0.170196097 0.014239898
-0.317327195 -0.05812742 -0.086326968 -0.244610898 -
0.437716995 -0.104467045 0.405806395 0.350610062 0.232283476
0.066549374 -0.170242287 0.020342865 -0.355701719
0.204632705 0.17028714 0.539639488 -0.042609994
0.126831846 0.186625091 -0.017945753 -0.168654142 -
0.281907797 0.316473458 0.23774229 -0.031545807 0.205411087
-0.16366199 -0.326473156 0.197702254 0.255214986
0.001209757 0.128041764 0.060948742 -0.287184417 -
0.075961023 -0.112533761 -0.35237771 0.217185883 0.282813189
-0.130921524 0.3827808 -0.111651356 -0.158517721 0.030881384
0.01739609 0.082409123 -0.005579317 0.246774195 -
0.332551135 0.291274169 0.218605022 0.236765561 -0.413689571
-0.310273214 -0.274231255 -0.3687591 0.349253362 -0.248503408
-0.101106277 0.178949872
KEGG_GLYCOSPHINGOLIPID_BIOSYNTHESIS_LACTO_AND_NEOLACTO
_SERIES 0.041068196 -0.261245436 -2.39E-01 -0.289567479
0.211916910.361359647 -0.043049138 -0.145960732 0.011181826
0.243560263 0.163325602 -0.090384608 -0.087141191 -
0.481090697 -0.048055956 0.366615582 -0.303196541 0.020720433
0.181834828 -0.137895833 -0.276759052 -0.119125826 -
0.192431705 0.07952399 0.225093671 -0.325001965 -0.078073068

-0.01692201 -0.11879039 0.093585669 -0.002539866 -
0.143575874 -0.027963888 0.361991786 -0.059108383 0.361980777
0.349813392 0.441208789 -0.237220436 -0.04080351
0.097822603 -0.031793204 -0.233047728 0.34164175 -
0.329263676 -0.122557042 -0.177338362 -0.058014226 0.159021802
-0.050976295 0.01922142 0.059322419 -0.054565638 -
0.045973085 -0.135444141 -0.025897846 0.081923468 0.018421101
-0.030896738 0.05376777 -0.103075195 0.084117913 -
0.184871364 -0.273827911 -0.413426992 -0.047692408 -0.157668462
0.181120437 0.143940594 -0.044195583 -0.135716928 -
0.119277308 0.040078165 0.066174235 0.014809506 0.340387478
0.023389413 -0.435469745 0.387340773 0.041633738
0.024872513 0.047546439 0.063979422 0.255219282
0.206601958 0.097123929 -0.401687358 -0.067155246 -
0.1398481 -0.149156615 -0.273698054 -0.070702553 -0.153900387 -
0.008292764 -0.033685963 -0.193107522 -0.000265828 -0.176186022
0.272732995 -0.052297246 0.075066515 -0.218352712 -
0.115492381 0.216689623 -0.008716967 -0.216005313 0.258606362
0.030899582 -0.15059627 0.202752546 -0.034130934 -
0.001101711 -0.114468745 0.219907465 0.19968737 -0.233204272
0.078148959 -0.060069411 -0.171232444 -0.213515595
0.243966463 0.027216017 -0.095752379 -0.136352372

0.154082986 0.148580773 0.15662247 -0.134259402 0.1022146
0.033124739 -0.191935044 -0.166690706 -0.160897947 -
0.118010987 -0.1032349 -0.026279428 0.003554432 0.081425219 -
0.286005053 0.158211028 -0.068252686 0.10943931 -0.279266298
-0.062333728 -0.219241209 -0.08346168 0.236449816 -
0.167036263 0.14723078 0.229091506 0.366810903 -0.063882907
-0.018881655 0.2965226 -0.138490996 -0.016477319 -0.05282455
-0.003675677 -0.021366492 -0.091436205 -0.202172257
0.139746504 -0.325238847 -0.13049111 0.299503444
0.141267421 0.276056367 0.290984635 0.223454447 -
0.021099437 -0.077104338 0.107222818 -0.131340562 -0.058265333
0.182917758 -0.074718646 -0.386914557 -0.078644381 -
0.079733832 -0.121751295 0.294573405 -0.033486351 -0.079950252
0.210407947 0.17077208 -0.00394373 -0.523067168 -
0.064955859 0.296640776 0.177297725 -0.161499063 0.167591915
0.024512362 -0.381920514 -0.118431914 0.339858201 -
0.2547009 -0.224314081 -0.261537484 0.110558960.106870914 -
0.061482562 0.207661664 0.022295166 0.194923217 -0.185793036
-0.079802429 -0.176064677 0.111845793 -0.172919891 -
0.221150261 -0.349809794 -0.205905927 -0.202683608 0.297282043
0.27914075 0.02694363 0.189145091 -0.152384088 -
0.391603167 -0.036239647 0.367139641 0.296687158 -0.18202482

-0.234825239 0.071027622 0.272756718 0.16222017 -
0.195027004 0.269227675 0.132580847 0.255145613 0.011455101
0.260019512 -0.11739392 0.023576251 -0.176397268 -
0.146802488 -0.342070843 0.146031249 0.148694968 -0.309661608
-0.477142427 0.271458451 -0.233652796 0.081775688
0.130617664 -0.222560184 0.049374081 0.109337155 -
0.041056248 -0.228178273 0.14956645 -0.07662121 0.277586859
0.054348659 -0.151194934 0.310228985 -0.206242778
0.036945412 -0.125871395 0.201450107 -0.051638798
0.198073832 0.279892393 0.106325101 -0.10469786
0.209312655 -0.053220076 0.289239069 0.267637493 -
0.316961053 0.259639394 0.316909613 -0.023358654 -0.024258443
0.075916664 0.357163192 -0.094086265 0.02602643
0.023777133 0.206403356 -0.128740908 0.21760352
0.156867762 0.070881211 0.051832593 -0.467320074
0.300484911 -0.167562416 -0.096055878 0.168185312
0.075918442 0.099810156 0.300062824 -0.082253633 -
0.079077613 -0.174698526 -0.132364878 0.074085159 -0.15385146
0.007594407 0.238883403 -0.161462715 0.110982781
0.168970869 0.135716371 0.115453298 0.148833563 -
0.012701247 -0.209848466 -0.108154533 0.177783568 -0.178886525
0.420326926 0.087957257 0.113341323 0.146486348 -

0.013689383 0.142108939 0.228546632 -0.010858086 0.136535283
0.359558244 -0.272010383 0.285504478 -0.139741994 -
0.098780726 0.369582696 0.186385026 0.275171583 0.028669593
-0.048966463 0.103937579 0.18320718 0.068332389
0.115507736 0.235864229 -0.257623185 0.107819125 -
0.257073286 -0.347186372 -0.029039643 -0.244461164 -0.116188755
0.015903245 -0.443746224 0.080743298 -0.069413907 -
0.228069424 -0.224473061 -0.043994625 -0.25939104 -0.13897464
-0.082740746 0.234785853 -0.153256487 -0.055088267 -
0.084086073 0.129649022 -0.179030839 0.021139435 -0.075919341
0.069160567 -0.005963797 0.032063981 -0.03803533 -
0.149305114 -0.098392594 -0.271591964 0.243368275 -0.242959982
0.078783447 -0.013880626 -0.258940399 -0.030878816
0.091605535 -0.030877724 0.012507593 0.085403796 -
0.079140703 0.359228555 -0.241414013 -0.112539105 -0.066469349
0.044919077 0.011950086 0.23835426 -0.068555073 -
0.029109396 0.418071617 0.116161402 0.143188429 0.32707749
0.097298159 -0.106601971 0.082331015 -0.015950024
0.002053044 -0.012981135 -0.087558733 -0.16320101 -
0.175125715 -0.148220387 0.196390626 0.0715371 -0.003198842
0.038924897 0.024545667 0.066913032 -0.220937544
0.247891997 0.235033589 0.247312766 0.056517647

0.00488217 0.265218374 -0.327848945 0.315719823 -
0.041062439 -0.031029958 -0.129966648 0.08401116 -0.087343564
0.269673332 -0.032051887 0.090221889 -0.080706885
0.220181757 -0.012128828 -0.044094687 0.26637962
0.184023354 0.032061833 0.048515933 -0.106287636
0.100053188 0.168305253 -0.350193272 -0.23696025 -
0.052304668 -0.018446698 -0.047329495 0.248537111 0.025251659
0.080905437 0.148656799 -0.15027206 0.123161831 -
0.02666525 0.025657928 0.177317773 0.076852039 0.105847169
-0.034667014 -0.196573064 -0.029746694 -0.056676953 -
0.128104764 0.275390063 0.156506978 -0.162660213 0.120014471
0.089062132 -0.082418088 0.075831874 -0.207254763 -
0.013767594 -0.260505078 -0.507502925 -0.331918919 0.147582251
0.267391963 -0.212929688 -0.040515804 0.222316808
0.063660636 0.038208134 0.025811479 -0.370362958 -
0.12994498 0.240557064 0.033195481 0.148468038 0.224600921
-0.295804745 0.191120206 0.241124090.044984632 -0.268271954
0.370826908 -0.294685902 0.238782976 0.045579087
0.062852458 -0.159230231 0.13251445 0.229276852
0.203012891 0.31944512 0.094408038 0.086222961
0.288119712 -0.088776639 0.163803387 -0.032277022
0.071403627 0.324039663 0.235721559 0.072091879 -

0.203931844 -0.227954341 0.032713828 -0.213814083 -0.246619131
0.050711464 0.087341723 0.095055313 -0.275008071 -
0.063106467 0.294469111 -0.29589796 -0.067099438 0.150675659
-0.243139206 -0.06766904 -0.01538501 0.219989506 -
0.238458693 -0.159700231 0.001011797 -0.218906074 -0.026200988
-0.219943978 0.286344768 0.362336387 -0.021641285
0.252334909 0.063639595 0.01001641 0.244346469 -
0.08573524 -0.166220962 0.285685143 0.072568714 0.101138872
-0.096991443 0.040160536 -0.065750686 0.199154517 -
0.262673494 -0.016689334 0.368609066 0.227982561 -0.11255442
-0.123355811 0.052750464 -0.175607361 0.413467776 -
0.428416967 0.387117542 -0.169553904 -0.245006544 -0.007255575
-0.409212786 0.077917633 0.049129733 -0.202503498 0.240083
KEGG_GLYCOSPHINGOLIPID_BIOSYNTHESIS_GLOBO_SERIES -
0.106448284 0.293570607 -0.345887822 -0.254999408 -0.151211573
0.018509127 -0.488898845 -0.068443609 0.10981703
0.167460598 -0.111653552 -0.010648606 0.157828991 -
0.319043533 0.108991452 0.13353562 -0.103334863 0.388078963
-0.168600978 0.13641689 -0.276518938 -0.192514378
0.242559149 -0.112153355 0.140843449 -0.326897993 -
0.371884547 0.127113988 0.031375414 -0.497106354 -0.252886438
0.35914841 -0.068868175 0.292716053 0.176848161

0.310564957 0.018077475 0.399663793 -0.207273512

0.043202362 0.478478273 -0.144726727 -0.09731095

0.401632684 0.048202284 0.221003444 -0.371118893 -

0.030857383 -0.139041877 -0.175436551 0.286547038 0.075858369

0.007266538 -0.314243005 0.261815967 0.119904055 -

0.14510094 0.241209375 0.081082922 0.247855106 0.104264097

0.185453048 0.092433043 -0.093890858 -0.178444793

0.063949151 0.083193543 -0.018613513 0.037917358 -

0.220141774 0.42747957 0.173933295 0.312522592 0.473971812

-0.164903472 0.071187545 -0.476819118 -0.221931633

0.331247141 0.128375091 0.097848291 0.212182599

0.324915918 0.326269074 0.173315465 0.014323672 -

0.365169592 -0.184407676 -0.117327965 -0.411641095 0.069501483

-0.062411352 -0.092603333 0.416835869 0.181022833 -

0.247442131 -0.03732516 0.145106513 0.287722287 0.046306162

-0.001236265 -0.337913334 -0.240536937 0.322974748

0.253467333 0.358076541 0.105970808 -0.238821108

0.048919378 0.093948017 -0.352397409 0.164628228 -

0.239589187 0.254251079 0.170070342 0.320173041 0.123300727

-0.160335714 -0.433794919 0.099482816 -0.511668441 -

0.204151485 -0.368586848 -0.091074761 -0.379302761 0.068846901

0.455191099 0.03861951 -0.39240243 0.344137363 -

0.087178232 -0.252246162 0.005071924 -0.411453771 0.425598672
0.29855444 0.240241819 -0.376483164 -0.215675757 -
0.061405021 -0.161835459 -0.117029374 -0.150676021 0.092038261
0.411164547 -0.022766201 0.068677617 -0.315554907 -
0.12981324 0.133170585 0.449626928 0.151036172 0.406138297
0.350939454 -0.297842286 -0.332189631 -0.084496199 -
0.112332146 -0.123182194 0.532438312 0.376392003 0.066950816
-0.386056291 -0.081679687 0.216291522 0.389212273
0.278169841 0.276985693 -0.088964537 0.273758713 -
0.209068062 -0.503392596 -0.36727489 -0.054574684 0.31502689
0.108630998 -0.174256585 -0.11967598 -0.099572288
0.206317905 0.43188808 -0.038853812 -0.186599995 0.2688387
0.309352110.159916748 -0.641713869 -0.225666849 -0.116166821
0.177757134 0.019075789 -0.105784644 -0.408385457 -
0.356626638 -0.205679295 -0.011158944 0.249097435 -0.06158632
-0.193396762 -0.045033836 0.204497664 -0.129739563
0.225994113 -0.088235392 0.130897112 0.005075504 -
0.311753125 -0.014042429 0.191364037 -0.145163559 -0.009194164
-0.416465037 -0.313353904 -0.319420632 0.1248014 -0.050637705
0.234580985 0.051772617 0.068020189 -0.218898718
0.501811244 -0.284711474 0.081409078 0.009688755 -
0.131131731 0.385479748 0.28432335 -0.346484057 -0.232118276

0.113181023 -0.273692833 0.389980764 -0.397969115
0.17594584 -0.39663163 -0.180226467 -0.07233311 -
0.292064616 -0.389581136 -0.116541562 0.208489793 -0.111271512
-0.195028181 0.419308875 0.006907953 0.097614697 -
0.178849318 -0.289377114 -0.040973424 -0.321645529 -0.35281212
-0.023315289 0.033709487 0.365032081 0.202257072
0.200282464 -0.24018358 -0.399709655 0.194128657 -
0.487691332 -0.383295111 0.292323008 -0.025986294 0.067448795
0.3435932 0.36480574 -0.139692541 -0.035417057 0.158471162
0.309610742 -0.065336994 0.126113322 0.280506007
0.190214886 -0.157819032 -0.09107344 -0.468957247
0.283870708 -0.486772302 0.221770512 -0.152061824
0.573177568 0.094473536 -0.295205025 -0.196880701 -
0.138563642 -0.456480843 0.122072716 0.287860544 -0.26445055
-0.429030823 0.246588383 -0.103002404 0.068863469 -
0.19726748 -0.092818041 -0.059012536 0.003691444 0.259110992
-0.210289611 -0.024575586 -0.180629881 -0.091291731
0.144091304 -0.265390436 0.119560414 0.151836027 -
0.03224977 0.44170783 -0.264764981 0.272388265 0.006576383
0.208224592 -0.096343387 0.063928009 0.193179166
0.093774837 -0.196561226 -0.067581478 0.052165634
0.045540996 -0.276245727 0.341164619 -0.093407828

0.368305814 0.338715033 0.335402622 -0.519237093
0.226685581 0.004309448 -0.122530811 -0.006951317
0.469073256 0.177302272 0.359181804 0.101483787
0.057603128 0.285711637 -0.099837243 0.069664893
0.299038201 -0.028444964 -0.061768465 -0.15338071 -
0.139760937 0.027812112 -0.139050979 0.001306973 -0.003171724
-0.073015665 -0.088437034 0.145315505 0.105756145 -
0.100086743 -0.131491475 -0.344040767 -0.118604159 0.083843198
0.086734799 0.19130883 -0.126527357 0.182748214 -
0.226350211 0.507541115 0.395535022 0.07778144 -0.41482976
0.272105959 0.136832525 -0.18510588 0.186988072 -
0.094837093 0.060151777 0.535642911 -0.292446046 -0.256502185
0.210432846 0.419712385 0.481400796 -0.106502854 -
0.157678824 -0.112776466 -0.483941881 -0.379501355 0.39338954
0.036960486 -0.338908484 0.524433272 0.173187265 -
0.026263605 0.17358592 0.179641855 0.257133359 0.222959905
0.445022599 0.090082122 -0.3718898 -0.285530595 0.081615368
-0.164605164 -0.068557433 0.396827709 -0.009464798 -
0.273295606 0.151372406 -0.389161479 0.064866979 0.029164822
-0.085689047 -0.099025029 -0.308091576 -0.160996263 -
0.455756185 0.376039842 -0.151095846 0.210028119 0.099674159
-0.261260313 0.483407805 0.354581395 -0.021233693 -

0.062556336 0.105496867 0.291813142 0.304644702 -0.089973998
-0.357699886 0.175283789 -0.115631526 0.119608922
0.258989365 0.052840722 -0.0937319 -0.315027392 -0.119033262
0.295982795 -0.016139337 0.126558903 -0.017871458 -
0.186847342 -0.146720234 0.175117566 0.237703002 -0.140739302
0.336165754 -0.202504414 0.259667148 -0.037085137 -
0.004613872 -0.08422403 0.105239669 -0.357600554 -0.328200303
-0.055000811 0.060599951 0.004675682 0.21265353 -
0.097762076 -0.295396088 0.369778627 -0.12461664 -0.251685831
0.214764463 0.200734791 0.1260832 -0.028009095 0.033429969
0.042638883 -0.417902205 -0.451317136 -0.503666343
0.225145221 0.157735024 -0.334669329 -0.186909645
0.353502569 0.079763262 -0.352888123 -0.206191382
0.284299311 0.19856379 -0.243450375 -0.205033958
0.544857916 -0.114475657 -0.235007393 0.284865702
0.150418815 0.032838531 -0.133764556 0.088621415 -
0.439456054 0.131944461 0.13392821 -0.113691798 -0.509304177
0.077099499 -0.190141041 0.236065648 0.08026835
0.412092302 0.076416359 0.172228238 0.051593783
0.404379198 0.366397387 0.06294158 0.389869082
0.11284317 -0.100296369 0.171507053 0.213330504 0.006888465
0.150989402 -0.39566798 -0.298176432 -0.176315934 -

0.107408713 -0.477476614 0.188411143 -0.161073233 -0.336493085
0.273670496 0.126523881 0.10685574 0.439607089
0.289819403 0.281162408 0.014092248 0.026237323
0.075071884 -0.338985321 0.254046619 -0.386089112
0.003779733 0.102991586 -0.405975325 0.353569932
0.473701859 -0.002585256 -0.24861144 0.302482707 -
0.28952412 0.058468479 0.077649753 0.321224868 0.032316436
0.099243169 0.118623498 -0.27621387 -0.275568232
0.055384566 -0.018027261 0.196283174 0.142778997 -
0.07422424 0.175215843 0.348795423 -0.092829269 -0.192021267
0.235979841 0.013113939 -0.407166765 0.262486982 -
0.196037252 -0.181724837 -0.24503976 -0.234415171 0.004243333
KEGG_GLYCOSPHINGOLIPID_BIOSYNTHESIS_GANGLIO_SERIES -
0.295662095 0.305577278 -0.231804834 -0.227669464 -0.338681141
-0.334350633 -0.352909567 -0.053602291 0.089288983
0.202215092 -0.168274902 0.132544442 -0.001460473 -
0.130917463 -0.138537808 -0.193959841 0.268470255 0.49654943
-0.228363319 0.068873584 -0.108735431 -0.310903652
0.206244358 -0.11594345 0.366204926 -0.190695718 -
0.164771001 0.036401728 -0.230386998 -0.385797467 -0.151533034
0.27642501 -0.186628211 0.141741337 0.397570787 -
0.051883338 0.185530666 0.446330115 -0.085969568 -0.249358385

0.020457998 0.282358305 0.176202651 0.296400814
0.294715935 0.176379014 -0.316927548 0.188082942
0.030352281 -0.352557587 0.044447033 0.158214142 -
0.157437471 -0.248234174 -0.0540794 0.22886468 -0.359151695
0.416632908 0.021059723 0.453687037 0.233581565
0.461776827 -0.15659406 -0.287682406 -0.355799649
0.286486814 0.394996768 0.012200187 0.243109207 -0.49311
0.558863024 0.166988908 0.284558836 0.351130603 -
0.363366415 0.103034561 -0.207647756 -0.243302399 0.112814461
-0.306034527 -0.021620534 0.081080857 0.428118043
0.260263321 -0.068915765 0.199080561 -0.00610373 -
0.367798801 0.046348325 -0.251794114 -0.11568546 0.041785214
-0.045339372 0.409280059 0.300473258 0.182911907 -
0.099547575 0.181007571 -0.033735815 -0.182698325 0.013929353
-0.378218539 -0.328206604 0.425693645 0.069348115 -
0.147056416 -0.00565844 -0.047484913 0.217841533 -0.229517121
-0.263357765 -0.305095105 -0.212850316 0.255510777 -
0.018387403 -0.024868764 0.002643411 -0.28366908 -0.638548631
-0.072909779 -0.474508733 -0.114512406 -0.410701545
0.45997254 -0.216923949 -0.424073531 0.565698108
0.197822946 -0.415409067 0.2659824 0.099324699 -0.187068296
-0.184372963 -0.154253756 0.298962314 0.417671557

0.237789231 -0.339672242 -0.100480257 0.156858039 -
0.470367142 -0.295872257 -0.264041091 -0.197624178 0.337097014
0.194749811 0.327632751 -0.059168582 0.106454093
0.306502072 0.445876723 0.355121096 0.561851004
0.319772043 -0.096500294 -0.244785393 0.052392194
0.026453207 -0.186135845 0.360248102 0.40140678
0.491115598 -0.354944617 -0.267926922 0.244255941
0.21847177 0.353445741 0.429646562 -0.041101305
0.020207501 -0.465195606 -0.623765422 -0.484948392
0.185128145 0.510605136 0.168487352 -0.085413003 -
0.488717282 -0.448983499 0.474984932 0.429546497 -0.070105624
-0.353462394 0.320294786 0.190163652 0.436162783 -
0.206955192 -0.267632641 0.035064631 0.133421377 -0.093540002
0.05541678 -0.456570487 -0.262513659 0.068374948
0.011064295 0.142929565 -0.089459045 -0.330281673 -
0.304984653 0.413134247 0.073624288 0.39217349 0.093243654
0.231883141 -0.254307546 -0.248627175 -0.284639799
0.320581519 -0.177705397 -0.080021935 -0.314762936 -
0.329418304 0.01967397 0.085500722 -0.284224934 0.187303551
0.258037897 -0.52407823 0.011583756 0.486566865 -
0.064404199 -0.20081658 -0.201888323 -0.149065592 0.47912899
0.368086261 -0.384109031 -0.215676154 0.317465242 -

0.051390565 0.199585329 -0.474225616 0.590984984 -0.423990018
0.101399137 0.039019855 -0.211360427 -0.162450907
0.239834344 0.502630247 -0.110723505 -0.379431401
0.24208628 -0.147132882 0.050730274 -0.171858754 -
0.251950721 -0.309586465 -0.468330785 -0.354822027 -0.019953358
-0.209834805 0.420394553 0.414607652 0.237602791
0.120397139 -0.273348242 -0.118814383 -0.412126274 -
0.286808493 0.062636083 -0.180832735 -0.033138951 0.527614966
0.119655750.081957987 0.198224349 0.119050436 0.040085275
0.031177251 -0.188066442 0.105800862 0.332871626
0.129484305 0.068242603 -0.610137415 0.203034358 -
0.455820998 0.340224319 -0.031060824 0.537308664 0.248414379
-0.350776245 -0.109780819 -0.232651405 -0.347075357 -
0.079089695 0.068578065 -0.470984139 -0.492102328 0.335093694
-0.206938345 0.027779888 -0.145143038 0.126511025 -
0.117820393 0.137149946 0.404341373 -0.053053429 -0.273773808
-0.228771405 -0.205840789 -0.009526358 -0.401842114
0.023903136 0.368546236 -0.038906842 0.604680426 -
0.484252175 -0.014967389 0.056998242 0.460956494 -0.071925802
0.085778329 0.04971623 -0.1862659-0.189368222 -0.386571347
-0.070941636 0.343636695 -0.24931565 0.052096692
0.026323875 0.258324234 0.450792423 0.047388576 -

0.464807814 0.072093509 -0.034664712 -0.297859751 -0.091750958
0.346151996 0.309221922 0.239290705 -0.227587922 -
0.047856358 0.594320888 -0.222468362 0.336596812 0.270717192
-0.028378016 -0.329506443 -0.507227713 -0.01213211
0.036344844 -0.294042226 -0.089164514 0.093018451 -
0.15149006 -0.002821482 -0.130635332 0.08441014 -0.11949569
-0.045878202 -0.454033257 -0.298602603 -0.220634557 -
0.392827932 0.374522163 -0.189421534 0.45346787 -0.394356823
0.472357319 0.330135221 0.430906074 -0.348332295
0.218018737 -0.203016034 0.003260432 0.410023881
0.072990685 0.436541579 0.363962808 -0.18656446 -
0.379183121 -0.054671982 -0.014110059 0.535795719 0.290180721
-0.264100059 0.060015525 -0.143945814 -0.30835162
0.559898288 0.176659131 -0.263785679 0.550748276
0.114249532 -0.243917634 0.256955369 0.162383476
0.29468418 0.195106327 0.349503226 0.284851368 -
0.359898548 -0.305886304 -0.170051302 -0.04151394 -0.216535798
0.402724946 0.125042698 -0.416983575 -0.007331987 -
0.49159808 -0.351321181 0.047767953 0.270115151 -0.277811991
-0.351043599 -0.038682506 -0.169130262 0.454218888
0.081480508 -0.121944371 -0.297977179 -0.327361676
0.418185027 0.298987787 0.29565558 -0.187134512 -

0.016891752 0.512964912 -0.135608538 -0.041964529 -0.231675218
0.259910544 -0.209365805 -0.138607667 0.179923247
0.274713117 0.450697133 -0.375733887 -0.343673438
0.034967714 0.201392451 0.26243704 -0.009772974
0.012060602 -0.270910678 0.501086706 -0.195232699 -
0.144477272 0.277271348 -0.202475863 0.487286732 0.134605484
0.131522267 -0.379736313 -0.08845731 -0.455316278 -
0.425122617 -0.055434782 0.324380143 -0.034153876 0.094078111
0.012934471 0.084683373 0.298782044 0.116416909 -
0.116300097 0.07520155 -0.362368555 0.252632068 -0.477118326
-0.091168164 0.170389659 -0.141829589 -0.413162407 -
0.441113332 -0.070614686 0.269862044 -0.145046836 -0.050269421
0.035136463 -0.07471994 -0.172844876 -0.44477126
0.06020283 0.172736791 0.135477074 -0.266965063
0.518419226 -0.292839322 -0.288225009 0.247557715
0.07696956 0.181859845 0.099636779 0.000877758 -
0.380249824 0.206821919 0.078845718 0.060018184 -0.543562661
0.35424243 -0.047233023 -0.019381655 0.175130517
0.226832404 -0.082948229 0.210849197 -0.192991817
0.475340926 0.469315144 0.267167603 0.420944752 -
0.16218395 -0.323200557 -0.399063421 -0.041885662 -0.193431151
-0.198476363 -0.235157674 -0.244300284 -0.036279787

0.006582686 0.020113845 -0.033136154 -0.264551613 -
0.411489184 -0.164075176 0.273815378 0.111977557 0.424687641
0.176402535 0.238503142 0.225279931 0.097125213 -
0.223749203 -0.055046295 0.527747547 -0.01864886 0.22275711
0.130024555 -0.548021749 0.160769734 0.222069399 -
0.124106004 -0.439198144 0.143967585 -0.163000647 0.444169825
0.232528991 -0.141522894 0.235033746 0.391605576 -
0.235905881 -0.374544403 -0.048685733 0.030904339 0.348549629
0.086022165 -0.020102586 -0.197786567 0.244694241
0.214932875 -0.450715489 0.480786404 0.275435626 -
0.155937359 0.079905427 -0.022817549 -0.123086972 0.075734279
-0.117385614 -0.361453618 0.408324296
KEGG_RIBOFLAVIN_METABOLISM 0.228871779 0.338051345
0.14237649 -0.251024162 -0.083743766 -6.42E-05 -0.346892709
0.20297537 -0.003677363 -0.173662927 -0.105688462 -
0.034804812 -0.06216212 -0.338033258 -0.105001839 -0.004789345
0.012941264 0.022920001 -0.266375872 0.212568013 -
0.16904101 0.399788576 0.212738043 -0.322177705 -0.081019787
0.081460533 0.149148666 -0.07088511 0.363502161 -
0.220502647 0.078982338 0.241873176 0.156367254 -0.044097876
-0.412389722 -0.256055729 -0.363856039 0.184332659 -
0.442128598 -0.312499205 -0.121018235 -0.136772128 0.035495885

0.290967177 -0.20808565 0.263434951 -0.039389652
0.131675858 -0.311129957 -0.27237013 -0.379849337 -
0.081004075 0.185901254 -0.205012842 0.120999381 0.413225528
-0.350204451 -0.277739885 -0.274911275 -0.193859653 -
0.340259968 -0.496344263 0.237189176 -0.163726605 -0.251066228
-0.170189512 -0.022695207 0.178548443 -0.282525145 -
0.243790482 -0.137132204 -0.135757387 -0.38407468 0.279089361
0.203902837 -0.210761471 0.22134071 0.34067859 -
0.281230013 0.470874988 -0.216813256 -0.247529312 0.304711734
0.269717356 0.140847321 -0.200870469 -0.446696234
0.148054309 -0.106654952 0.244075614 0.193930197 -
0.271985573 0.023096041 0.063769087 0.372615924 -0.129326063
-0.294723243 -0.180971137 -0.022119383 0.324525005
0.401296935 -0.184299604 0.204689364 0.376511983 -
0.116159629 0.127478205 0.315471962 0.456449484 -0.439252181
-0.426356196 -0.499105768 0.095970184 0.159602371 -
0.240240834 0.086535096 0.245267085 0.327797497 0.048436475
-0.210387677 0.277452649 -0.165048534 0.126763006
0.177201191 -0.138435559 -0.307758936 -0.113565509
0.229697547 0.141997119 0.119742036 0.159665176
0.190823059 -0.107934811 0.284424643 0.098131351 -
0.040281286 -0.384013191 -0.309628697 -0.150903071 -0.072408748

-0.256888837 -0.293671624 0.363534919 -0.349844063 -
0.5991549 0.288581015 -0.263665664 -0.275752202 0.283793377 -
0.158888508 -0.177450786 -0.041460672 0.007221163 -0.120646162
-0.48734468 -0.174106592 -0.360024608 -0.333316155
0.35882289 0.124609058 0.181333225 0.296466175
0.209078856 0.144303222 0.028205747 -0.190333971 -
0.412538333 -0.181180137 -0.108719193 -0.204810809 -0.184707404
0.008386861 -0.075272032 -0.353909425 0.056990131 -
0.074176353 -0.369578451 -0.123078542 0.187756092 -0.134399011
-0.161903009 0.207564252 -0.295436103 -0.194970104
0.342042272 -0.346189338 -0.48727082 -0.161491172
0.097246587 -0.367373715 0.090773696 0.291007664
0.195915097 -0.004882544 -0.182579673 0.30034652 -
0.189643131 0.083511057 0.115608139 -0.064443716 -0.103850344
-0.13372486 0.352267029 -0.234522872 0.030617962
0.16592047 0.316654406 0.20815022 0.35107407 -
0.314085308 0.17973906 0.23004766 0.075248548 0.355159485
0.311351491 -0.400587045 0.306271312 0.430662412
0.137299922 0.190032747 0.048196038 -0.044634401 -
0.220915237 -0.383334532 0.008453898 -0.313655301 0.463314417
0.093446569 -0.267731985 -0.228625929 0.429352014
0.285750606 0.028347942 0.258608322 -0.054521471

7028 0.069965049 -0.325285265 0.018055073 -0.396029341

7029 0.087689912 0.108027356 -0.080870594 -0.083187698

7030 0.407873698 0.133877291 -0.270329894 0.246925787 -

7031 0.044516927 -0.158084742 0.36010941 -0.048093266 -0.012225841

7032 -0.157676829 -0.276307471 -0.381260909 0.138317602 -

7033 0.264167205 0.117950247 -0.154542519 0.022002544 -0.292051082

7034 0.197831416 0.384233056 0.242979025 -0.408273559 -

7035 0.052537358 -0.283190236 -0.2834574 0.12937899 -0.253861953 -

7036 0.291056057 0.245947972 0.107763076 0.035060219 0.388582814

7037 -0.011245294 0.519990429 0.252968434 0.21961476 -

7038 0.136198018 -0.18734671 -0.024733291 0.090887384 0.351590497

7039 -0.107766247 -0.256832345 0.32439332 -0.279797482

7040 0.278830695 -0.104916792 -0.445958838 -0.055634135 -

7041 0.067123283 0.166051772 -0.04706704 -0.096784347 -0.242461295

7042 -0.095586708 -0.176520781 0.118044666 0.150622013

7043 0.103488738 0.011046684 -0.069905039 0.097589257

7044 0.230455162 0.027096534 0.186769289 -0.242280324 -

7045 0.130466709 -0.405720338 -0.279546901 -0.033563623 -0.140947486

7046 -0.042796424 0.220262055 0.170962675 -0.223879331

7047 0.170440978 0.065160146 0.355314757 -0.243710809 -

7048 0.040258692 0.169464766 -0.39996739 0.257593343 0.146175749

7049 0.089495495 0.07770049 0.088348218 -0.344391848 -

0.068713394 -0.264728193 -0.112348809 0.311048283 -0.378532173

-0.228271862 -0.114845324 -0.267301663 -0.065388439 -

0.298639017 0.4118355 0.304837494 -0.09554558 -0.155226207

0.202609579 0.346115613 -0.091920576 0.02875291 -

0.129284078 0.013528732 0.130799359 -0.029057421 -0.037224843

0.547484329 -0.174817912 -0.056983714 -0.254087991

0.448251776 0.228701469 0.085524746 -0.041843764 -

0.459274186 -0.217501519 0.037857875 0.514352592 0.134583999

0.005069261 -0.205085058 -0.045091902 -0.196517957 -

0.019420032 -0.526748236 0.006060333 0.004861061 0.348032305

-0.178966765 -0.187866702 0.273952276 -0.289778223 -

0.125201594 -0.052630881 -0.148002515 -0.128376962 0.086624756

-0.24294072 -0.242892167 0.130891733 -0.268192178 -

0.341256631 0.181618121 0.307408698 0.147014405 0.137018863

0.359657765 -0.084732221 -0.175327118 0.092696747 -

0.0784309 -0.40137114 -0.278190602 0.059110416 0.140955235 -

0.039260084 0.127518948 -0.37372834 0.305279388 -0.056138001

-0.128757656 0.078424869 -0.087208916 0.102731647 -

0.121174455 -0.068352862 -0.189165166 -0.014720314 -0.34840297

0.379141417 0.539959029 0.007504796 0.100758761 -

0.080061755 0.014962742 0.031762627 -0.118089479 0.044541732

0.220725686 0.198400169 -0.078638676 -0.171713324

0.537254261 0.003508284 0.312158019 -0.174668601 -
0.171829928 0.12304633 -0.342941696 0.300876761 -0.401760871
-0.071492495 -0.350120256 -0.233138154 -0.222883271
0.59699229 -0.533777333 -0.347503765 -0.071670126 -
0.012313303 -0.472502534 -0.2312829 -0.083536381 0.035967678 -
0.176293094 0.081327976 -0.453561204 0.518025323 -0.203528611
0.097451312 -0.507670964 0.168092244 0.206239414 -
0.110923358 -0.162566081 -0.384557291 -0.124571421 -0.241497758
-0.209945822 0.328495256 0.263902715 -0.237652059 -
0.091574871 0.118965984 0.356258896 -0.002348215 -0.324264214
-0.122057142 -0.358154522 -0.063872014 -0.113620464
0.137040085 0.047457629 0.016666523 -0.09997066
0.230412097 -0.028108965 0.021125152 0.314272432
0.134904779 0.269696992 -0.169351008 -0.320008335 -
0.197884868 -0.20408478 0.001103078 -0.229799175 0.037088588
-0.217988806 0.281684191 -0.134749974 0.475454043
0.347324825 0.020266865 -0.192895059 0.129853054 -
0.514540089 -0.509287035 -0.053226886 0.060657741 0.045729207
0.007687789 0.302504399 -0.28518182 -0.428720603
0.006882561 -0.358054354 -0.002418291 0.484339334
0.214570464 0.144196118 0.096396927 0.066463154
0.356570204 -0.400112456 0.397435238 0.152043893

0.247970103 -0.074208174 0.29851766 0.079569568 -
0.097668006 0.154956189 0.17928699 0.060331348 0.191882262
0.038936486 -0.163369406 -0.335574453 -0.027841413
0.290660119 -0.050036856 -0.435332642 0.289472605 -
0.152430495 -0.078083463 -0.137114345 0.057996195 0.341680387
-0.115423962 -0.164726614 -0.337012807 0.034782958
0.11732006-0.077169668 -0.515356926 -0.157655088 -0.048062634
-0.279096046 0.206308434 0.041763455 -0.175294482 -
0.524028965 -0.077467421 0.38599816 -0.434924536 0.14645159
-0.120996067 -0.234540698 0.288966581 0.053899155
0.11963547
KEGG_NICOTINATE_AND_NICOTINAMIDE_METABOLISM 0.243598768
0.249994035 -2.81E-01 -0.033113321 0.084824454 0.180217888
0.187777899 0.116825017 -0.023146786 -0.197044847 -
0.006219673 -0.183177155 -0.452445956 -0.252138394 -0.11101189
-0.101496329 -0.241196015 -0.16152416 -0.316474965 -
0.00981939 0.019061584 0.130506623 -0.355916592 -0.259934348
-0.142970321 -0.053817355 0.01913818 0.273781762
0.043766184 0.028764031 0.34053224 0.340936203
0.020214381 0.088911331 -0.219981455 -0.170899781 -
0.354396544 0.208143129 -0.341262866 0.008300658 -0.259011032
0.358007275 -0.180417456 -0.190573305 -0.354613245

0.330636755 -0.088159866 -0.086050881 0.170849764 -
0.13502222 -0.265087448 -0.379296052 0.019556259 -0.213275382
0.089325856 0.266748193 -0.228449696 0.06765492 -
0.158605534 0.024867811 -0.350720862 0.327570096 -0.220917333
-0.138563608 -0.216167972 0.153303959 -0.249749549
0.246464175 -0.195617523 -0.158888954 -0.213589907 -
0.139631372 0.026274598 0.02036391 -0.089652118 0.264552861
0.058011030.328305114 0.209445678 0.3794097 -0.052645408
0.071979014 0.339101122 0.046179552 0.048234124
0.081167939 -0.394233266 0.123343554 -0.011351901
0.248320767 0.003871186 -0.192040218 -0.386373343
0.237452062 0.283967188 -0.037543421 -0.085446183 -
0.322289862 0.175434943 0.307601164 0.120554264 -0.165456553
-0.136267493 0.278523496 -0.152599747 0.248022568
0.018264404 0.278926272 -0.364141484 -0.290435259 -
0.129808695 0.252207456 0.050013563 -0.487351084 0.000667925
0.19196464 -0.099859373 0.208876705 -0.161113529
0.217368714 -0.093560533 0.426875653 -0.179497696
0.069836616 -0.230028133 0.030824647 0.015980328
0.047089465 0.082180887 -0.004936245 -0.105306881 -
0.116011353 -0.184490429 0.113842422 -0.19189992 -0.165846862
-0.299353264 0.247162951 -0.103873632 -0.286926003 -

0.167314439 0.323288485 -0.172587819 -0.143878933 -0.133435242
-0.249949017 -8.16E-05 0.252764471 0.070118403 -0.161946035
-0.006423771 0.220155599 -0.09392071 0.011955388 -
0.319202075 0.078756479 -0.212844888 0.332538683 0.239790601
-0.080895645 -0.351058559 0.167250041 0.105957959 -
0.332864797 -0.015916617 -0.387763223 -0.05942702 -0.076621545
0.105935631 0.006979681 0.174746892 0.193987715 -
0.272530403 -0.13018093 -0.128937712 -0.294033662 0.096912488
0.229932275 -0.320861892 -0.10402042 0.298492309 -
0.023759474 -0.09699395 -0.113115334 -0.20994561 0.291804349
-0.113163198 -0.148391249 -0.145410833 0.098965647 -
0.038754819 0.438053741 0.35809024 -0.252953684 0.515705582
-0.30066266 -0.056047388 0.442184 0.05385226 -0.214139256
0.050251514 0.247722302 -0.179839501 0.161206444 -
0.065048846 0.485621513 -0.01200674 0.511155049 -0.147790506
0.331283831 0.223783104 0.334627433 0.297740537
0.038339045 0.153952051 -0.004624092 0.170369621
0.171361516 0.257672127 -0.03447472 -0.065713599
0.132746499 -0.006880576 -0.161712256 -0.36856854 -
0.068416445 0.030358615 0.025878453 -0.063479834 0.427549434
0.127520828 -0.072865166 -0.216036654 -0.000736824
0.16798726 0.031000718 0.058043186 -0.260288975

0.064520677 -0.031020596 0.433393873 -0.270449023
0.112827633 -0.161457594 -0.285192603 0.004613026
0.176119854 -0.252060805 0.129041869 -0.00236202
0.013353836 -0.002955621 -0.104724973 -0.140747065
0.217784284 -0.22790093 0.328895053 -0.129969183
0.565310878 -0.350705653 -0.180820783 0.043729642
0.125001509 0.314473743 0.076900559 -0.337359721 -
0.417089263 0.000487835 0.006204418 0.139490028 0.224259273
-0.335998377 0.16422355 0.224103484 -0.20809655 -
0.009320027 0.404313049 0.329796151 -0.247672938 -0.052350827
-0.042702851 0.120990644 0.117778214 -0.155865103 -
0.071314933 0.158634601 0.02113302-0.028871554 0.134271575 -
0.45682539 -0.064967022 0.350080391 0.040173536 -0.039351923
-0.2255827-0.307891998 0.056741886 0.178322701 -0.370719756
-0.138207479 -0.015783453 0.277117231 -0.071660673
0.053897673 0.044282313 0.00196633 -0.247745216 -
0.014275674 -0.243386307 -0.27819489 -0.161345909 -0.091948094
-0.026907722 -0.077078532 0.259843954 -0.142808451 -
0.338919681 0.243493494 0.060697754 0.170106909 -0.001227363
0.162598336 0.249788705 0.177169861 -0.013012407
0.046300568 -0.030709379 0.149993401 0.234716448
0.209633416 0.003013341 -0.106105902 0.050567336

7182 0.311828479 -0.478083194 -0.216737676 0.148946564 -
7183 0.240063161 0.072243588 -0.271914774 0.319375315 0.317343547
7184 -0.294178125 -0.098219938 0.34671707 0.088648528 -
7185 0.309719897 0.270193976 0.199515616 -0.200509626 -0.019549503
7186 -0.096350605 -0.438120165 0.053494074 -0.204909069
7187 0.139694812 0.272772659 0.267077548 0.118399491
7188 0.338167196 -0.025586993 0.050311206 0.021251615
7189 0.047869447 -0.077839921 -0.190220227 -0.074633138 -
7190 0.345899145 -0.292386447 -0.248347105 0.013805347 -0.47930396
7191 -0.231932018 -0.107038447 -0.000381669 0.048987151 -
7192 0.310403151 0.393920817 -0.248580678 0.324564879 -0.146960972
7193 -0.295625673 0.40790462 -0.274618689 0.132106739 -
7194 0.226953517 -0.124149709 -0.203348947 -0.066099908 0.121514559
7195 0.144670711 0.288220165 0.101674727 0.32569248 -
7196 0.118045150.026020492 -0.016055085 -0.202168305 -0.454518139 -
7197 0.067127407 0.145678727 -0.061812343 -0.414360448 0.329123351
7198 0.246557878 0.22765291 -0.013867575 -0.162562147 -
7199 0.054136853 0.107146616 0.165930392 -0.055529056 0.225304319
7200 -0.24962328 -0.002932549 -0.177330479 0.164182843
7201 0.424641364 0.136873188 -0.294955856 -0.238667277 -
7202 0.0806305 -0.029805784 0.060875417 -0.03661684 0.220654293
7203 0.315802921 0.060009937 -0.151204505 0.153475769

7204 0.309561486 -0.182954392 -0.141325204 0.080356759

7205 0.182692008 -0.423186901 0.057419269 -0.243955022 -

7206 0.342709948 -0.083773483 -0.045632274 -0.179006234 0.084066424

7207 -0.107685693 -0.170938727 -0.206883354 0.046342337 -

7208 0.210784218 -0.240670425 -0.146997451 0.190023841 -0.142577914

7209 -0.020777934 -0.248759416 0.179749574 -0.325472838 -

7210 0.409107811 -0.403188098 -0.070812496 0.433229948 0.181194866

7211 -0.057691343 0.217006156 -0.004853883 -0.297516078

7212 0.185389333 0.094953416 -0.083512322 -0.152615329 -

7213 0.347336392 0.268899854 0.555184659 0.217210956 -0.320156137

7214 -0.169580461 0.021442522 -0.051351006 -0.029608725 -

7215 0.028750304 0.24135037 -0.193657547 -0.04180477 0.230087522

7216 -0.159688595 -0.127722022 0.336820366 -0.10317372

7217 0.161918764 0.137866527 -0.48013075 -0.160141543 -

7218 0.121080909 0.161606486 0.134438484 -0.004876688 0.081733232

7219 0.136772868 -0.042761156 0.185729171 0.166707262

7220 0.11497706-0.211209466 0.067255358 -0.186353604 -0.28143183

7221 0.045752265 0.05878036 -0.050736572 0.000650708 -

7222 0.280468253 0.105797778 0.077690611 -0.182642233 -0.28046428

7223 -0.029933813 0.025707341 0.034785429 0.134365071 -

7224 0.077928935 -0.062295633 0.182758513 -0.269039289 0.267754849

7225 0.307723601 0.279029548 -0.310738472 -0.068724127

0.230326074 -0.340788908 -0.515452476 0.054491022 -
0.009303041 0.169122257 0.036313334 -0.089348302 0.075365004
-0.284224498 0.284876936 0.015753174 -0.31020984
0.090502956 -0.114254293 -0.095099535 0.060386026 -
0.076998256 0.036843463 -0.30039452 0.141819768 -0.376018788
0.252057495 0.0992545 -0.198138827 -0.039619601 -0.087642348
-0.068255132 -0.084657382 0.219628439 -0.152611793
0.226608895 -0.272120395 -0.121359599 0.022573951 -
0.391337651 -0.305334516 -0.146000724 -0.133996196 -0.162380172
0.278576717 0.387190356
KEGG_PANTOTHENATE_AND_COA_BIOSYNTHESIS 0.308200861
0.532136349 -5.08E-01 0.146954758 0.0351236 0.108219959
0.187128625 0.021214273 -0.240043372 -0.069776506
0.043733514 -0.107762316 -0.302174481 -0.381034608 -
0.269710944 -0.100673959 -0.182524988 0.351185191 -0.323381932
0.31039699 0.162792186 0.278696136 -0.356554425 -
0.079688928 -0.201316116 0.0248798 0.038733354 0.097274283 -
0.013946972 -0.506910498 -0.048589102 0.313668493 0.28662645
0.207912357 -0.152359333 -0.171809956 -0.012324138
0.172274933 -0.328718079 0.295531876 -0.390960431
0.348052043 -0.057683331 0.339892422 -0.159534912
0.205926243 0.258177638 -0.149721908 -0.170555981 -

0.313737669 -0.192510589 -0.301956737 -0.217515884 -0.240336162
0.494828597 0.071780735 -0.301064328 0.161453328
0.302344157 -0.015470839 -0.561453189 -0.201378599
0.255326215 -0.094863876 -0.270722059 0.087493844
0.204640765 0.012484205 -0.057016762 -0.18672904 -
0.171521449 -0.11340026 -0.419979456 0.041090779 -0.180517401
0.048517110.415466011 0.409380402 0.126549835 0.412708184
-0.293222267 0.074293198 0.187441191 -0.125963197 0.3559917
-0.339605355 -0.379343912 -0.171135788 0.015744476
0.283538431 -0.449140586 -0.37919091 0.153488356
0.473584646 0.46747669 0.224792738 -0.045685852 -
0.04056427 0.06653848 0.078230327 0.371273199 -0.325550249
-0.035792937 0.241258957 -0.178602631 -0.125383799
0.243899009 0.171537146 -0.004987308 -0.427311337 -
0.279105045 -0.127527908 0.177195442 -0.252252565 0.173285437
0.424311799 0.084575172 -0.285871323 -0.253006702 -
0.122396692 -0.325031402 0.29357566 0.219390491 0.139660236
0.103329985 -0.25080722 -0.323832225 0.357959331
0.231890626 0.258271256 0.021789281 -0.393909876 -
0.217573972 -0.185165312 -0.242403146 -0.168268775 -0.440877702
0.250454259 -0.098735948 -0.071124069 0.093133012
0.261820986 0.179367725 -0.164450922 -0.074319506 -

0.289663722 0.137702968 0.362925326 -0.390691966 0.336093418
-0.006878684 0.152004176 -0.141638007 0.099789279
0.013522855 -0.192164401 -0.458434685 0.268108334
0.096667789 0.048809386 -0.126673022 0.108676495
0.12939744 -0.47035136 0.44410278 -0.111833697
0.055281387 0.19387252 -0.183545613 -0.029040742 -
0.072504771 -0.021609881 -0.13170887 0.180148579 0.035681037
-0.303292768 -0.073778521 -0.269679253 0.020939243
0.017048703 0.263772522 -0.141350528 0.093827885 -
0.079659789 0.142573575 0.255637332 0.04566277 -0.138571947
-0.245943639 -0.006002713 -0.131775487 0.44214235
0.238125938 -0.293466214 0.374838883 -0.014022393 -
0.277055227 0.162697388 -0.324959532 0.077018872 -0.277897856
0.452592834 -0.298760526 0.192308999 0.151780859 -
0.081982274 -0.256293247 0.498622162 -0.3282986 -0.030408548
0.246900605 -0.117651572 0.014845721 0.20242402 -
0.037905731 0.091388319 0.357944316 0.22268288 -0.052306594
0.14792001 -0.161166179 0.421792386 -0.020302458 -
0.140248247 -0.377165105 0.028788423 -0.025402604 -0.210182406
0.034489685 0.445569397 -0.041952925 0.330935322
0.099132765 -0.000376741 0.248887496 0.212318727 -
0.180682039 0.034436297 0.021652563 0.182685139 0.39101934

0.025432044 0.150982663 -0.380108839 -0.337011515
0.096141971 0.164374698 -0.066901274 0.156621798 -
0.323410338 -0.304822279 -0.071198786 -0.175761313 -0.315819021
0.108069043 -0.328563818 0.255409727 -0.100600168
0.36216659 -0.393370441 -0.437988604 0.189316208
0.091770585 -0.204482356 0.236872938 -0.406184995 -
0.388036142 -0.025222042 0.282419904 -0.262809933 0.309048808
-0.22339436 -0.21042541 0.03359593 -0.210886255
0.423580365 0.398465644 0.227854312 -0.261929468
0.024311564 -0.17691277 0.21875947 -0.097119187 -
0.379521792 -0.208928297 0.098645293 -0.26777049 -0.062189743
0.112719304 -0.126916874 -0.399196654 0.162171092 -
0.117698201 0.154170127 -0.41506393 0.165180073 0.26758505
-0.026544485 -0.252973127 0.174640662 0.257925952
0.12256416 -0.189496993 -0.048621031 0.234108043 -
0.368019431 0.081450757 0.214088161 -0.430794572 -0.309383052
-0.322285488 -0.248350819 -0.255037148 0.132326747
0.447803083 -0.360031661 -0.135409006 0.153917061
0.153389529 0.457473166 -0.297122398 0.274394671
0.33658896 -0.210036643 0.206880292 0.125014156
0.096152914 -0.034918905 0.262412759 -0.16949193 -
0.179095058 -0.269171709 -0.417156115 0.551775026 -0.205393399

0.094325931 0.225026302 -0.182037952 0.078955769
0.34047174 0.082385293 0.222959691 -0.018298637 -
0.203521716 0.241344283 0.271971425 -0.354257458 0.000157327
-0.268461059 0.035886367 -0.023166461 0.001629699 -
0.011894554 0.395192175 0.323244802 -0.383527271 -0.26457269
0.416682643 0.059165429 0.003697895 -0.468575955 -
0.370349825 0.052996864 -0.081343592 0.304056974 0.005712559
0.088274479 -0.103911532 -0.073494571 -0.066979217
0.007158751 -0.302461188 0.260760296 0.057400224
0.33366409 -0.151861077 -0.535663248 0.277097938 -
0.098006604 0.262288967 -0.392224767 -0.145817025 -0.42045009
-0.231014959 0.010162368 -0.044951937 0.003769658 -
0.06002231 0.097849046 0.485727397 0.09244808 0.185540441
-0.051714083 0.306699739 -0.201846996 0.059284246
0.186443203 -0.331276081 -0.441219938 0.075568584
0.061637768 0.373465941 -0.257012461 0.15994612
0.046086999 0.174286991 -0.261392426 -0.173602173 -
0.039082731 0.083655266 0.485663419 0.169512414 0.139282526
-0.281106125 -0.198716807 -0.201203352 0.500286011
0.494670183 0.16816656 0.044333793 -0.204999185 -
0.175925615 -0.036944874 0.244048642 -0.102489002 -0.137318715
0.051744368 0.084853319 -0.391893667 0.274391436 -

0.134339955 0.166020759 -0.430179385 -0.319394495 0.08529417

-0.362411392 0.472597934 -0.195727126 -0.171150065 -

0.419083713 -0.052108072 -0.069877259 0.30378384 -0.443764436

-0.243881649 0.382372823 -0.078165108 -0.323503233 -

0.16860564 0.186251283 -0.095746927 -0.073390922 -0.184082924

-0.275575846 0.345996634 -0.058273657 -0.129329353 -

0.430444069 -0.288914491 -0.234341951 -0.007513164 0.132135832

-0.358486565 -0.016537022 -0.011565259 0.110931217

0.042594387 0.086950391 -0.137256013 -0.050100358 -

0.223669883 0.396309208 0.378855255 -0.321999252 -0.428520113

0.089732012 0.228050918 0.019085234 0.068345239 -

0.112372044 -0.1372938 -0.008580755 0.209483546 -0.168968405

0.56315208 0.263370408 0.145161354 0.292640194 -

0.198749632 -0.358806524 -0.226276674 -0.44854172 0.177104893

0.092312163 -0.193302652 -0.160290023 0.059386899

0.304692247 -0.133023488 0.123059311 0.421661214

0.310242028 -0.080906713 -0.175709557 -0.132712281

0.122366314 0.057642232 0.120971285 -0.028529514

0.173022838 0.023967196 -0.47669539 -0.330222025 -

0.333726416 0.399452419 0.54052959 0.249518102 0.157018869

-0.092701362 -0.031546078 0.311533144 -0.362325202

0.145635872 -0.002854703 -0.121206683 -0.30148239

0.477960869 0.264938278 -0.16006754 0.108711479 -
0.260450916 -0.220977241 0.43408369 -0.181255784 -0.283926889
0.2885369 -0.17834651 -0.228315473 -0.251996128 -0.208399147
0.242338751 -0.007848699 -0.384889909 -0.400348928 -
0.199529609 0.28084575 -0.306884515 -0.029041819 -0.066374598
0.268058232 -0.241443882 -0.338221019 -0.026597554 -
0.013539265 0.125930232 -0.298171017 0.417777308 -0.064279569
0.357507983 -0.192134209 0.296599628 -0.368957423
0.062125563 -0.066633664 -0.130687992 0.317841906 -
0.268011346 -0.404627173 0.254599887
KEGG_AMINOACYL_TRNA_BIOSYNTHESIS 0.482898469 0.54870281
-1.04E-01 -0.013336264 -0.544543783 -0.265536372 0.365739641
-0.492475052 0.242994901 0.029676852 0.270123243
0.645430516 -0.586418652 0.294473591 -0.406548143 -
0.328936069 0.292870251 0.633069863 -0.71396014 0.067749305
0.053280525 0.004894861 -0.765503183 0.313182221 -
0.450760561 -0.366797013 -0.174763349 0.309795837 0.041108619
-0.601263931 0.315996255 -0.183776748 0.299148861 -
0.415780361 -0.357543693 -0.623871877 -0.292243318 -0.352057287
-0.546953582 0.175649748 0.548545219 -0.134716237
0.043266865 0.319897447 0.085260256 0.557603511
0.05163556 0.260826994 -0.046305381 -0.417926707

0.531059934 -0.320604952 0.328143326 -0.46424808
0.518466798 0.623967467 -0.542430909 0.418735717
0.382729332 -0.074806074 -0.309097219 -0.034937099
0.070326425 0.27742361 -0.179849196 -0.336598559 -
0.107422783 0.571125704 -0.538542987 -0.539304173 0.147148873
-0.216550734 0.540363491 -0.085053532 0.359466078 -
0.59012899 -0.230808087 0.481895724 0.429707101 0.494900873
-0.474561196 -0.293072702 0.276423556 -0.30311593
0.370510024 -0.114887462 0.370308214 0.368252008 -
0.585096702 0.364545757 0.031942951 -0.538872268 -0.08810394
0.355179255 0.596286835 -0.284327823 0.247858289 -
0.562558943 -0.16206899 0.538283696 0.092575267 -0.622923949
0.26103181 0.092352321 -0.408383827 0.20552483
0.108604939 0.63392798 -0.274834019 -0.705168147 -
0.622673483 0.278598272 0.445600515 -0.273583951 0.189885713
0.284949197 -0.019030041 -0.497340632 -0.52164423
0.53403018 -0.641774892 0.157121986 0.437000021
0.398039385 -0.289020752 -0.574120009 0.450249802
0.064179649 0.079822921 0.446690297 0.055522407 -
0.623327506 -0.063585403 -0.089863922 0.377508522 -0.636274913
-0.299368287 0.314431336 0.522203326 -0.259084757 -
0.173078611 -0.418990617 0.502649919 0.013733536 0.130206441

7402 0.476234163 -0.228179392 0.350495907 -0.555427411 -

7403 0.25192192 -0.675590454 0.46119244-0.359198415 -0.570696849

7404 0.440307388 -0.621536582 -0.2485486 0.288638509 -0.062045074

7405 -0.49612629 -0.042039186 -0.256572492 0.261108466 -

7406 0.596633894 0.502174123 -0.554448934 -0.042958923 -0.38419204

7407 -0.668140014 -0.046111682 0.51819225 -0.338473267 -

7408 0.602284135 -0.362894743 -0.15231204 0.196468046 0.310871534

7409 0.372570512 -0.154258729 0.014687574 -0.375363249

7410 0.353271638 0.109254863 -0.31902857 -0.352547718

7411 0.35002701 0.365471572 -0.056068637 -0.731158091 -

7412 0.157039733 0.373885646 -0.178012694 0.239393273 -0.576967258

7413 0.238851881 0.094493865 0.23126457 0.498649471 -

7414 0.257689943 0.267391302 -0.531893352 0.54932658 -0.442130446

7415 -0.046267653 0.244496296 0.37141305 -0.333484547

7416 0.465441914 -0.150948927 0.67419955 0.447140318 -

7417 0.091334589 0.673905864 0.146981744 -0.487239869 0.541248966

7418 0.391521895 -0.270441856 0.260405193 0.00400421 -

7419 0.573509241 0.708501071 -0.685019339 0.29240994 -0.386290401

7420 -0.533052051 -0.018587396 -0.391490117 0.535073063 -

7421 0.246153374 -0.280508557 0.21371924 0.12474056 -0.5734435

7422 0.190786659 -0.035062203 -0.562765496 0.211548326 -

7423 0.097242139 -0.529858757 0.674985694 0.41804183 0.196873852

-0.121221116 0.379416828 0.092857079 -0.056201109 -
0.470629323 -0.272927579 -0.426766561 0.359379728 0.314990069
-0.605127116 -0.349894257 -0.358695677 -0.394400339
0.248206052 -0.716566054 0.31567546 -0.627595833 -
0.136527048 0.48704074 -0.202237038 -0.364628468 0.475791684
-0.333124352 -0.410927635 0.061165121 -0.29522941 -
0.707087382 0.214049675 0.321674442 0.477859805 0.039443376
0.398102924 0.460245287 0.360269184 -0.398668782 -
0.41041026 -0.562180319 -0.681679985 0.167526669 -0.364106566
-0.619328766 -0.581582715 -0.247297098 -0.695883405
0.460018417 -0.196415454 -0.548097811 -0.08739475
0.006228475 0.196324498 -0.259322369 -0.549281635 -
0.608203402 0.210206371 0.192496457 -0.431801943 -0.489127044
0.044158058 0.603091463 -0.566772242 -0.338962412
0.507888831 0.357273401 -0.292165367 0.393308 -0.648948201
-0.509726596 -0.595125426 -0.475982592 -0.680824443
0.454356994 0.686658988 0.661848225 -0.641827513
0.455349009 0.600801995 0.594444512 -0.049372731
0.764734125 0.537267772 -0.590763966 0.10064838 -
0.488002104 -0.180548691 -0.286250634 -0.249041655 0.21123873
0.169518079 0.239738801 -0.393172071 0.078854899 -
0.086106245 0.482581752 0.486122287 -0.361425092 0.152738467

0.195207738 0.34957271 0.170000733 -0.597747657
0.093627908 0.527515205 0.139078186 0.320865294
0.531394853 0.049199375 -0.149635513 -0.302827012 -
0.072103759 0.183817832 0.337010616 0.442437184 -0.620622376
-0.408302075 0.597434005 0.246624021 -0.134620215 -
0.016081471 0.492973194 0.477799114 -0.456885434 0.035434249
-0.293540622 0.269479074 0.300907456 -0.631246885 -
0.114556279 0.396258189 0.135297031 -0.004103466 -0.024336623
0.144979668 -0.413104855 -0.450612291 0.340194585 -
0.433631434 0.561076617 -0.484512901 -0.511773044 -0.044764217
-0.399910736 0.004387673 0.210886471 0.033081864 -
0.040401752 0.378198732 0.272770421 0.238265357 0.245496147
-0.32641343 0.363449471 -0.390374996 0.124263909 -
0.227778383 -0.072608407 -0.389199758 0.095259031 -0.239022292
-0.135880782 -0.029225138 0.601217521 0.052805929
0.390594644 0.077736445 -0.140258163 0.232245167
0.463353175 0.478362375 -0.254836849 0.163210266 -
0.516927552 -0.348626281 -0.079936749 0.619029493 0.690627977
-0.396412302 0.031907171 -0.158099393 0.187782266
0.647227922 0.402836402 -0.434909459 0.560257479 -
0.45555559 0.012348273 0.261095507 0.576241698 0.213405752
0.559150054 -0.411663647 -0.577330353 0.519099893 -

0.562012518 0.515742593 0.670167954 0.115556533 0.219514937
0.283004609 0.003033405 0.417318707 -0.560593265 -
0.279863216 0.444133476 0.197059265 -0.685290157 -0.402565582
0.331576339 0.05944852 -0.658295291 0.096838515 -
0.439796461 0.004396571 0.506361297 -0.317488897 -0.506562039
0.080432266 0.328743096 -0.192079753 -0.308482331 -
0.735273633 -0.211688676 0.041416299 0.030200938 0.304919822
-0.34309574 0.399231827 -0.353838897 -0.031541348
0.555803587 0.347247581 -0.287173665 -0.405476157 -
0.190032565 -0.582019995 -0.393983694 -0.179991633 -0.061839996
0.071357272 -0.382698678 0.253756907 0.194258966
0.225379112 0.831531299 0.269907242 0.396203435
0.307978641 0.120119644 -0.48297833 -0.183749989
0.278140183 0.274249056 0.178527373 -0.282937882
0.121773763 -0.436486052 0.620367432 0.159302411
0.55979207 0.193486242 -0.07944225 -0.424319802 -
0.372429312 0.197229981 -0.357228229 -0.014692743 -0.548903946
0.125626311 0.126124284 0.199107404 -0.476439723 -
0.120509514 0.373454259 0.387960995 0.053169268 0.404438642
0.267215525 -0.094082004 0.687253502 -0.631271311
0.352795089 0.578085058 0.32593716 -0.273187949
0.671980947 -0.056900324 0.243832059 0.077827126 -

0.008496517 0.316982203 0.292331576 0.003224152 -0.102089843
-0.064188197 -0.039984571 0.448530005 -0.372139035 -
0.403821612 0.505051379 -0.054997282 0.120188497 -0.25918691
-0.366382547 -0.333954188 -0.214499471 0.150158516 -
0.391037602 0.550212529 -0.374492593 -0.318353614 0.034274536
-0.189336767 -0.051235759 -0.058360299 0.303113911 -
0.362173572 0.319069999 -0.091624477 0.130967109 0.212856589
-0.416053747 -0.283260046 -0.09292841 -0.223367537
0.180083847 0.045652325 -0.158095021
KEGG_BASAL_TRANSCRIPTION_FACTORS 0.177906053 -0.223932556
3.97E-01 0.132189669 -0.297086904 -0.306358193 0.05998158
-0.235847917 0.387952079 -0.235304635 -0.172678542
0.363789451 -0.180867347 0.1437866 -0.411171974 -0.314895617
0.314373206 -0.289259335 -0.336138071 0.378132405
0.249602772 0.350119945 -0.012331083 0.405695244 -
0.243423465 0.179199075 0.146084437 -0.24885366 -0.038931572
-0.277007493 0.300494961 -0.300565699 0.145839573 -
0.421516393 0.237141061 -0.043709734 0.044100738 -0.472800684
0.043781362 0.072668021 -0.015185257 -0.32709534
0.409320798 -0.032776249 0.186948346 0.0737642 0.378892224
-0.043214545 0.034125068 -0.205550177 0.004210608
0.477275253 0.140511088 0.252692113 0.059126505

0.174356678 -0.436541619 -0.319389284 0.182009574 -
0.271371216 0.379989374 -0.229001846 0.262721483 0.151346733
0.064452247 -0.21035841 -0.213826415 0.190548851 -
0.228732715 -0.294023223 0.341536856 -0.036205519 0.00365729
0.045866925 -0.261458808 -0.510461777 -0.188347959
0.129062169 -0.343337238 0.116144559 -0.438523365
0.047283754 0.111061440.193598492 -0.081081934 0.3384752
0.401181015 -0.127117774 -0.230026299 0.362400142 -
0.16991445 -0.121709046 0.273108405 0.079786044 -0.075979999
-0.210451896 -0.078202934 -0.23894387 -0.085255028 -
0.141569364 0.153188184 -0.28278711 0.318006198 -0.391914217
-0.346074049 0.149124047 -0.148634559 0.28543247
0.267224846 -0.34034871 -0.497591261 -0.265193334
0.193949075 0.177531815 -0.351215631 0.483977963 -
0.184250865 -0.165684589 -0.279815632 0.282964597 -0.412638737
-0.360388324 0.223150125 -0.290236828 -0.067501692 -
0.597058545 0.201836387 0.12612386 0.407606297 0.318942033
0.227000755 -0.227825893 0.035455063 0.089442179 -
0.019459838 0.141518866 0.053416018 -0.098404941 0.239426572
0.392755589 -0.303704711 -0.351226803 0.236466838
0.308540644 0.39991902 0.114069193 -0.07250479 -
0.221499592 -0.427479886 -0.263647173 -0.461176512 0.267672777

-0.103907216 -0.43896116 0.258366423 -0.340550825
0.152544156 -0.21005692 -0.095173734 -0.30137576
0.324781557 -0.188911432 0.328509965 -0.083277496 -
0.18653489 0.126091086 -0.363851866 -0.186500116 -0.522251817
-0.231711597 0.422245772 -0.205396934 -0.240353949 -
0.02865215 0.061277237 0.135146432 0.329630619 0.125133117
0.264855412 -0.168283037 -0.388717785 -0.075446763
0.203775007 -0.165629768 0.1863359 -0.308078072 0.311761374
0.352732698 -0.408764676 -0.221471687 0.417375418 -
0.163657851 -0.008191108 -0.159434805 0.400693477 -0.461614342
-0.00064826 0.235920541 0.138659467 0.257420036 -
0.236143088 0.184148394 -0.118480849 -0.426595847 -0.025157583
-0.273439389 -0.22435049 0.247199781 0.328951133 -
0.014809179 0.114456487 0.251174987 -0.06731433 0.287937531
-0.514245982 0.259993365 -0.094220302 0.214174662
0.168636512 0.119512851 -0.346341015 -0.003544017 -
0.379044426 0.490107151 -0.041951451 -0.128987617 0.066467355
-0.337065909 0.466154537 -0.482141933 0.005386145
0.289022197 0.131701212 -0.321730693 0.024513367
0.102867723 -0.345276474 0.416889327 0.043846381 -
0.097953643 -0.02700766 0.298269015 0.249053652 -0.000969289
0.483292123 0.271817381 -0.237523098 -0.132819972 -

0.075964597 -0.27112778 -0.014982072 -0.24926102 -0.377133177
0.256003529 -0.519079772 -0.3269803 -0.247006846 -0.36081154
0.198433689 0.060326222 0.103172275 -0.025298408
0.244502582 -0.491639182 0.234082861 0.258406593
0.029364758 -0.179107244 0.383109694 -0.592519417
0.125416619 0.508079338 -0.0697435 0.154925737 0.386310009
0.121459329 0.338648422 -0.327794235 0.178505452 -
0.279650949 -0.253987976 -0.224312858 0.256565283 -0.448799481
-0.334640632 -0.450280266 -0.346645489 0.332587032
0.034087816 -0.192665793 0.25433871 -0.541315936
0.326929447 0.037399648 -0.418915921 -0.270259018
0.084855275 -0.170167948 0.081718603 -0.375706062
0.379036208 -0.357042813 -0.385686967 -0.319511343 -
0.299900534 -0.071221701 0.193148417 -0.046442749 -0.108351352
-0.307878405 -0.255013004 -0.282895093 -0.495808107
0.43792079 0.376555113 0.191342512 -0.363144184
0.024077287 0.331395937 0.219771739 -0.368977093 -
0.121872081 0.117371747 -0.529641232 0.080900867 -0.299870479
0.139102306 -0.401435553 0.158563151 0.159283366
0.066066505 0.307437712 -0.032998512 -0.39321877
0.285068636 -0.208044276 0.317819687 -0.019162079
0.211147603 0.129453964 0.039082571 0.375737799 -

0.262251392 0.421775574 0.179187894 -0.278920112 0.164201339
-0.122419359 -0.438394894 0.26918314 0.192087626
0.277137168 0.2494918 0.338553956 0.213026891 -0.380996411
-0.400751612 0.228885859 0.209853301 -0.140060061 -
0.017202255 -0.153667074 0.341373675 -0.286847847 0.19591954
-0.112591825 0.002595942 0.275432576 0.240873085
0.261769239 -0.066765677 0.064048888 0.240062254 -
0.050048165 0.429646527 -0.150706612 -0.158100163 -0.166375574
0.12498622 0.210712766 -0.023190987 -0.191399414 -
0.389007112 -0.237376935 -0.052007694 0.140320173 0.163540483
0.086922578 0.268575326 0.411877347 -0.080572635 -
0.022702995 -0.057001356 -0.372008153 -0.291780763 -0.020839117
0.145185998 -0.15018707 0.210825016 0.134259626
0.014429311 -0.131698112 0.397688733 -0.047912926 -
0.410450643 0.127155603 -0.004396795 0.261600665 -0.002089058
0.477484613 0.362673123 0.179478616 -0.346180681 -
0.070446025 -0.216678731 0.182586715 -0.165355659 0.458582672
-0.378049869 0.118452495 0.111744689 0.29739736
0.086802561 0.082784269 -0.339868564 0.062571938 -
0.229177023 0.028809236 0.199962072 0.322553392 -0.329314887
-0.270083458 -0.108753267 -0.403484322 -0.212570006
0.295384873 -0.045304986 0.221478147 0.565621532 -

0.138028551 -0.045301677 0.342325937 -0.026790918 -0.350666719
-0.363990342 0.081251812 -0.333211883 -0.204495039 -
0.042797071 -0.276426688 0.38504331 -0.166438907 -0.108457575
-0.47974836 -0.126981926 0.204926136 0.000555674 -
0.016112792 0.531832973 0.075183291 -0.135475295 -0.113004469
-0.405384828 -0.137668002 0.37645666 -0.432643798
0.125309789 0.024871096 0.127393371 0.426930667
0.411254202 -0.330311195 -0.200440234 0.27600907
0.028452923 0.100409853 -0.161284019 -0.121638698 -
0.180032303 0.371644107 0.328052087 -0.359845103 0.148439725
-0.381090313 0.034520006 0.342603894 0.104617648
0.233970656 -0.074709932 0.196334619 -0.364585018 -
0.019610872 0.251472608 0.195978643 0.224624003 0.094071284
-0.383434352 -0.320149474 0.378658378 -0.303049125 -
0.156348097 0.219217475 -0.15806385 0.205418949 -0.059264044
-0.279102686 0.191524559 -0.084111427 -0.392377615
0.448402315 -0.187958862 -0.185669173 0.024007373 -
0.290091485 -0.027261172 0.128844437 -0.024507031 0.179299959
0.021228135 -0.211769582 0.158731101 -0.122819577
0.322721236 -0.072463857 0.270160357 0.32377622 -
0.101432314 0.338057859 0.127344269 0.334753664 -0.272488734
0.116066956 0.163094088 0.200774584 -0.058352253 -

0.134595784 -0.12686207 0.012925209 -0.507333357 -0.20535468
0.332293423 0.159965753 0.274765005 -0.17551222 -
0.372719284 -0.391558087 -0.189652589 -0.015411788 -0.26951144
0.11011254 0.166817566 -0.080171287 -0.183874653 0.085283591
-0.291077426 -0.179096215 0.39997094 0.053465205 -
0.068207348 -0.221679716 0.320016901 -0.21862523 0.024981387
-0.223168947 0.157650585 -0.073514764 -0.138826058 -
0.059852973 -0.029299773
KEGG_NON_HOMOLOGOUS_END_JOINING 0.089547052 0.526535133
-2.91E-01 0.16575246 -0.269713817 -0.209872346 0.091850583
-0.409714362 0.192666262 -0.026646564 0.298382657
0.283560943 -0.268836807 0.091018204 -0.317793985 -
0.342288534 0.202975353 0.172229397 -0.521297509 0.275042547
-0.05890973 -0.138242758 -0.528482379 0.360541699 -
0.438095844 -0.152769637 -0.24821322 0.574269796 0.316883625
-0.415534634 -0.316528131 0.408993324 0.167128026 -
0.193453932 -0.356838671 -0.242705461 -0.316016806 -0.348476968
-0.542983316 0.351397062 -0.095417012 -0.154523419
0.077415204 0.311120103 0.14057302 0.270473521
0.298884585 0.011282435 -0.044110141 -0.372948858 -
0.219806967 -0.215471011 0.098181226 -0.30972247 -0.063947803
0.375126752 -0.431768288 -0.248839284 0.250400266

0.178803982 0.196331801 -0.10263707 0.189409399
0.260564974 0.154006784 -0.104878841 0.17566254
0.307089956 -0.515179767 -0.322206577 0.397254271
0.12360952 0.39844206 -0.108212264 0.150566178 -
0.158754409 0.201882579 0.249951689 -0.168239345 0.212566727
-0.394981903 0.044294513 0.253172706 -0.105045558 -
0.050581565 0.568042677 -0.036122509 0.297601285 0.043499901
0.432796279 0.253487511 -0.484283541 -0.36794249
0.223555418 0.430296885 0.458639521 -0.09922387
0.004200323 -0.127113479 0.081937072 0.501191161 -
0.455123335 0.226408411 -0.542353421 -0.153741791 0.256315083
-0.070970725 0.0291687 0.054189865 -0.593893858 -0.47953906
-0.28294704 -0.191510588 -0.190841364 0.072744881
0.505910152 0.105504925 -0.344370412 -0.281371378
0.534828723 -0.416964841 0.301774753 0.233234725 -
0.349198711 0.253493849 -0.414890396 0.232522106 -0.240357262
0.260719223 0.487914237 0.339733825 -0.043136993
0.009671171 0.382966415 0.212137035 -0.368173446 -
0.28816816 0.435676351 0.489083492 -0.034576193 -0.019608038
-0.054551288 0.087924971 -0.264351737 0.289894763 -
0.073464406 0.128772599 0.594443037 -0.610185634 -0.176093388
-0.44108814 0.543329646 0.13499614 -0.372385202

7666 0.321901157 -0.339515696 0.162456618 0.12478824

7667 0.570309168 -0.045850228 0.12885984 0.342629951

7668 0.044205393 -0.5274865 0.042491075 -0.281888158 -0.082821853

7669 0.109158858 -0.411818474 0.254026799 0.386193593 -

7670 0.208171521 -0.517007362 -0.292457862 0.223329426 0.089566099

7671 0.53348986 0.388594941 -0.236158187 0.229210956 -

7672 0.251371526 -0.181700291 -0.186065101 -0.363976523 -0.203480648

7673 0.090240424 0.012052115 0.223365331 -0.306058518

7674 0.05019242 0.561558897 -0.224035513 -0.082710668 -

7675 0.393922528 0.193173339 -0.27425793 0.424385825 0.115233093

7676 0.128214536 0.352607204 -0.667471166 0.510279198 -

7677 0.480321764 -0.413900459 0.195512723 0.340381337 -0.270213767

7678 0.572948789 0.081361545 0.509072256 0.453563493

7679 0.188346848 0.315662828 0.345363237 -0.402105666

7680 0.379286592 0.490443839 0.074329132 0.114337622

7681 0.444010776 -0.397045249 0.322000157 -0.491643665

7682 0.369653604 -0.366218152 -0.535904516 0.075293707 -

7683 0.286544926 0.177222858 -0.298573249 0.125138364 0.200898702

7684 -0.423351742 -0.016203716 -0.119418738 0.084930355 -

7685 0.35096786 0.305175592 0.224745478 -0.225505428 -0.242266868

7686 0.219914439 0.240027048 0.124364663 0.203629781

7687 0.49415789 0.182405164 -0.297176423 0.320665725 -

0.273491243 0.03854009 0.125548119 -0.430590441 0.175760113
0.092698812 -0.35826102 0.191734406 -0.49454987
0.325881749 -0.421230946 0.03987509 0.294129735
0.038547062 -0.22010551 -0.164078334 -0.154235053 -
0.602994777 0.292126797 0.129648935 -0.401307745 0.177923379
0.302420449 0.116109994 0.069308026 0.325389918
0.054586753 0.356483299 -0.381147269 -0.195380883 -
0.429034739 -0.449712515 -0.29666874 0.188540193 -0.558303722
-0.461157187 -0.118790248 -0.518573036 0.547085552
0.108009705 -0.541388991 0.057810668 -0.183319689
0.231999197 -0.03172626 -0.526456085 -0.356237668
0.052074952 0.410514601 0.058623847 -0.274589291
0.20628592 -0.058452555 -0.356284708 -0.402455298
0.084372515 -0.328098029 0.111312677 0.219886954 -
0.423771075 -0.282452589 -0.347872297 -0.401073899 -0.324373485
0.304272924 0.284250632 0.28446831 -0.590903274
0.340731935 0.193967821 0.368011244 -0.313256843
0.294051056 0.37084971 -0.592337032 -0.083149085 -
0.319264886 -0.220465613 -0.079490772 -0.187163843 -0.071605596
0.280475988 0.26102072 -0.117575768 -0.125076625
0.22667479 0.086288188 0.447340279 -0.288383589
0.189712674 0.131043781 0.240127714 0.306593555 -

0.341723399 -0.229277532 0.463600252 -0.253946026 0.141249968
0.101354105 -0.1552874 0.057744698 0.066276699 0.588823158
0.056030599 0.287830175 0.071615813 -0.591158395 -
0.287004017 0.476393086 0.146350789 -0.140196182 0.204641239
0.114209049 0.549377886 -0.404246927 0.390248261 -
0.030700469 0.217499795 0.330528374 -0.569062195 -0.184943111
0.119420527 -0.140577063 -0.107114169 -0.015005143
0.259302505 -0.449920372 -0.299113416 -0.225203014 -
0.217314253 0.361686624 -0.395338347 -0.249753237 0.106406355
-0.223791813 0.326312197 -0.112451155 0.035478412
0.119397689 0.249875736 0.527571878 0.084954294
0.284354738 0.052374655 0.060468952 -0.355203181
0.300838865 -0.232730348 -0.395004837 -0.071886562 -
0.060008792 0.282901466 0.211683996 -0.039304556 0.484006374
0.144726482 0.253866696 0.204428336 0.056329954
0.256359705 0.411242261 -0.01114957 0.090464862
0.175634656 -0.48942041 -0.21068365 -0.378777943 -
0.023418269 0.284790192 -0.125960728 0.006417151 0.359512412
0.353589558 0.236991815 0.139247885 -0.580595588 -
0.078612438 0.023503779 0.447029968 0.088438719 0.424355732
0.309465741 -0.137086034 -0.479559768 -0.434747825
0.45358923 0.086289177 0.428808366 -0.071885657

0.141871917 -0.065723916 0.050621923 -0.245285711
0.139846666 -0.577658732 0.084215892 0.252333739 -
0.419009517 -0.176198832 -0.361995726 0.004643768 0.197725088
-0.49300864 -0.30730447 -0.400671678 0.183892456 -
0.10917412 0.087999211 -0.336039852 0.254559512 0.436084457
0.183629957 0.146195374 -0.317331925 -0.369417384
0.137250691 0.015703537 0.410267945 0.085103969
0.424953633 -0.350633685 0.169640818 -0.199809507
0.215091941 -0.158470975 0.230966062 -0.130817993 -
0.498858562 0.106196566 0.37646324 0.217974316 0.467663327
-0.05286388 -0.250021149 -0.369838519 -0.008611452
0.496667175 0.221814089 0.154794087 0.082362561
0.102138048 -0.333351038 0.022425098 0.401435178
0.424920842 0.244841551 -0.253263602 -0.140230841 -
0.155187573 0.099952075 0.041632625 0.452296422 -0.098706042
-0.417084226 -0.484252893 -0.241798687 0.461822246
0.003063884 0.173768931 -0.370789207 0.230808306 -
0.076071513 0.090073564 -0.326602187 -0.434099832 0.350637858
0.194084813 -0.101039095 0.203935003 -0.053415435
0.18276572 -0.107670817 -0.213244873 0.096082205 -
0.021272719 0.623213403 -0.406390793 0.106505084 -0.352673456
0.214953889 0.084162408 -0.198795066 -0.388084145

0.310613577 0.031309093 -0.160470947 -0.331503168 -
0.010180861 -0.127581905 -0.453969901 0.099058714 0.191627983
0.409637912 -0.307438004 -0.095764944 -0.023536578 -
0.48009875 0.067540603 0.28615873 -0.367067621 0.178110049
-0.350823315 -0.394940111 0.071977178 0.330553382 -
0.415117296 0.353574388 0.410328721 -0.070908324 0.546311089
0.177604337 0.489259101 -0.088808611 -0.068076453 -
0.004029042 -0.272965181 0.317804598 -0.512941291 -0.494464931
0.148390433
KEGG_SNARE_INTERACTIONS_IN_VESICULAR_TRANSPORT
0.268722616 0.23507566 -3.68E-03 0.098756028 -0.395102055
-0.293570457 -0.039453586 -0.146635494 0.323253022
0.043769204 -0.19091784 0.200224526 -0.532865846 -
0.167135351 -0.349938837 -0.434291709 0.36789085 -0.025518059
-0.503281262 0.447108628 0.06346697 -0.001321065
0.044405171 0.244195859 -0.11558878 0.005737373
0.081871898 -0.081228222 0.25327495 -0.08270729 -
0.119244418 0.277847746 0.331388 -0.204048499 -0.182445109
0.039009269 -0.358259719 -0.028088842 -0.196049657
0.352651227 -0.105090616 -0.392290585 0.069961633
0.195809163 -0.005061171 -0.052422092 0.012515964 -
0.204083539 -0.41261733 -0.158422441 0.106897409 0.266143095

-0.210538617 4.74E-05 -0.192559685 -0.028730255 -0.465234068
-0.041629368 0.173606102 0.279949714 0.200345777 -
0.247413363 0.211321496 0.152263244 0.10932904 -0.060660787
0.024072074 -0.142495292 -0.533092395 -0.297380646
0.368154745 0.383833603 -0.181547548 0.229017828
0.289369846 -0.20360631 -0.037094631 0.39775639 -
0.219773842 0.213313971 -0.283830358 0.029452052 0.232193512
-0.084892526 0.292478574 0.253803666 -0.057519508 -
0.002259371 -0.155095107 0.292801526 -0.262651019 -0.461424518
0.165209548 0.230141744 0.585625824 0.131710212 -
0.02639001 0.098225773 0.119413998 -0.221029918 0.211343298
-0.254890237 0.055489494 -0.028287957 -0.205090579 -
0.039734553 0.150515574 -0.057441099 0.028688501 -0.342223361
-0.51372051 -0.033298808 -0.225854889 -0.206505518
0.143065224 0.445454319 0.165330158 -0.431263079 -
0.369971453 0.285426453 -0.328256142 0.191420911 0.087639992
-0.342968703 0.200789343 -0.483104278 0.054995655
0.217118603 0.22545801 0.252693069 0.075833213 -
0.005171604 0.30779634 0.330295834 0.205922474 -0.018082436
0.179374209 0.15165342 0.138421451 0.115993371 -
0.259939098 0.151640618 0.234604149 0.021225655 0.108201029
-0.09266155 0.101783008 0.195265522 -0.486983691 -

0.147177171 -0.116544277 0.03465352 -0.142120712 -0.449294763
0.269624284 -0.382809254 0.222561809 0.142404648
0.146388255 0.186369644 0.027628457 0.152606038
0.212549934 -0.182261346 0.214221422 -0.070033982 -
0.056885412 0.010400225 -0.471893658 -0.320560611 0.153056305
-0.242529807 -0.254471547 -0.179088948 0.021913221 -
0.092972711 0.374527076 0.321293982 0.302343191 -0.036492381
0.264789949 -0.117620016 0.214787454 -0.142496844
0.078046358 -0.158032207 -0.127445051 0.079884186 -
0.333654178 0.096795845 0.297186799 0.06106325 -0.123396907
-0.285036846 0.296414792 -0.444738846 0.173617061
0.276873452 -0.058185509 0.422783888 -0.345078603
0.315938124 -0.2801177 -0.415798565 -0.01033547 -0.047954924
0.043574256 0.446247264 -0.079962149 0.475785617
0.236649423 0.138782304 0.235346492 0.228854333 -
0.446825381 0.465678779 0.524627215 -0.14272609 -0.059361171
0.337485271 -0.119599041 -0.008728806 -0.352364776
0.295719388 -0.102431693 -0.089925414 0.075803175 -
0.168492094 0.221367302 -0.115848732 0.041286185 -0.029967769
0.075266 -0.345918352 0.154795153 -0.329970309 -0.19212101
0.320269873 0.108174151 0.056117616 -0.014874956
0.023081976 -0.035276892 0.053610552 -0.049648745

0.153910547 0.038738623 0.059327797 -0.066676413 -
0.219185987 0.035197411 -0.126854695 -0.418201134 0.254670958
-0.108485693 -0.432773147 -0.181239716 -0.251829571
0.48185044 -0.141755279 0.147748326 0.269239236 -
0.100400049 -0.463200327 0.169120833 0.106326193 -0.22758614
-0.0821873 0.405443634 -0.43679575 0.176403636 0.071720681
0.20569303 0.030888568 0.141010328 0.287098028
0.136591052 -0.285406692 -0.271125689 -0.14335635 -
0.215021301 -0.04176345 0.089982991 -0.590743444 -0.300539212
-0.147356402 -0.251902158 0.49112118 -0.318914635 -0.294490492
-0.052642184 -0.095551248 0.339885005 0.293722185 -
0.444373654 -0.117642713 0.334052126 0.167224105 0.173328968
-0.143437915 0.297182073 -0.1515119 -0.288292709 -0.408776318
-0.271152769 -0.275921489 0.044833672 0.181547765 -
0.328729607 -0.380357257 -0.295535115 -0.200576472 -0.37193778
0.332129146 0.092055399 0.245256348 -0.492835302
0.231737003 0.057649401 0.288350337 -0.465817514
0.14386478 -0.105941634 -0.514466009 0.136586221 -
0.187532405 0.224389237 -0.358498606 -0.252794881 -0.336401988
-0.065595093 0.027668404 -0.192595977 -0.217734554
0.049249359 -0.039733548 0.22255019 0.042662242
0.297282641 -0.019373303 0.297531514 0.209610585 -

0.039320529 0.034185302 0.130280378 0.232506517 0.260337873
0.148198862 -0.274984295 0.172956901 0.185129182
0.282235124 0.232422511 0.302313764 0.208273572 -
0.229287187 -0.336351949 0.202507194 0.341859539 -0.094409308
0.094356798 -0.275852819 0.406270337 -0.29073153
0.340087221 -0.194573273 0.132510833 0.322373167 -
0.044386256 0.153898378 -0.062398087 0.139208657 0.316526649
0.243663353 0.53835415 -0.109933853 -0.220452374 -
0.507705635 0.191641172 -0.079358031 -0.245849328 -0.09857412
-0.170149797 -0.109862495 -0.165727254 0.065028138 -
0.017315985 0.10058707 -0.177272144 0.088634404 0.146966776
0.133745064 -0.265802905 -0.143728701 -0.136439645 -
0.058846154 -0.067466564 -0.221195832 -0.003942335 -0.099541296
-0.053693829 0.237779047 0.254612243 -0.118224329 -
0.26803586 0.182269924 -0.035875982 -0.081989239 0.094984695
0.339256144 0.389779482 0.21377064 -0.245324178
0.131840272 -0.314996466 -0.038003837 0.007316819
0.169891247 -0.273212859 0.012660903 0.279300241
0.193326663 0.045856548 -0.063040893 -0.493021206
0.084829682 -0.30199236 0.013064312 -0.158176766
0.118432307 -0.187721352 0.30918953 0.033509948 -
0.387202132 -0.333382665 -0.212897822 0.13521062 0.068438102

0.306770907 -0.258766918 0.110915984 -0.016964008
0.231247852 -0.55854275 0.136045453 0.322629797 -
0.311889151 -0.281994087 -0.141929035 -0.025578823 0.387889583
-0.180509172 0.175685521 -0.346171004 0.092336145 -
0.211691658 0.063488031 -0.196135164 0.258921529 0.089386259
-0.209677486 0.092779706 -0.129388368 -0.043114239
0.176917236 -0.067482157 0.301984368 -0.308884697
0.243925089 0.087314983 0.051287016 -0.202928227
0.023290467 0.153762327 -0.047435233 0.131650892 -
0.145134298 -0.225281124 0.123793755 0.346706151 0.276511119
-0.109619105 0.274651317 -0.349068006 -0.149561774
0.346203854 0.223859248 -0.07524005 0.295073732 -
0.079768838 -0.214969314 -0.320664192 0.025175979 -0.082278132
0.049679408 -0.225954795 -0.261239617 -0.04838116
0.280792344 -0.157823994 0.04748051 0.208048174 -
0.197692963 -0.075354084 -0.159887751 -0.282548054 -0.360671805
-0.048890391 -0.4054147 0.156845826 -0.160693515 -0.01542637
-0.181868095 -0.385494576 0.03133771 0.128613041 -
0.037206235 0.126938297 0.428888412 0.03541764 -0.090106688
-0.376028766 0.133731593 -0.009077963 0.450534037
0.160904639 0.139508674 0.230803009 0.200726517
0.317244378 -0.401091342 -0.113279468 0.243537721 -

0.017969836 -0.116205293 -0.261602651 -0.469751516 0.023610601
-0.014041113 -0.192229708 -0.185550566 0.214155348 -
0.256347524 0.218469855 -0.118785056 -0.327241048 -0.028993589
0.316006855 0.03317682 0.13663801 -0.245105646 -
0.162458549 0.008982024 0.184407151 0.171098394 -0.344801098
0.457979691 0.173977395 0.236507314 -0.040192514
0.313015492 -0.336396642 -0.197749039 -0.302668316 -
0.289692541 -0.133345405 -0.193643752 -0.369201358 0.31983704
KEGG_LYSOSOME 0.288684934 0.443551092 -2.07E-01 -
0.180394044 -0.114039224 -0.343462403 -0.404176472 0.237007187
0.28445413 -0.049343292 -0.392846964 0.253798684 -
0.439334021 -0.141249627 0.028375056 0.027441719 0.015945379
0.519292139 -0.451962634 0.371735803 -0.288659863
0.29516141 -0.3513196 -0.254665305 -0.00332141 -0.272920294
-0.354561243 0.192982968 0.057147965 -0.37560773 -
0.123601751 0.523046281 0.30822144 0.234933921 0.270396644
-0.224878668 -0.521482751 0.442784093 -0.509984192
0.076467678 0.042713327 0.356586833 -0.086780147
0.162585537 0.078409257 0.390635499 -0.042406604 -
0.14484545 0.170364468 -0.401109963 0.190780484 -0.323594652
0.142064598 -0.529589548 0.354854154 0.320993603 -
0.364357238 0.489293645 0.331289954 -0.024333692 -0.188052907

0.040135855 -0.068173447 -0.130142183 -0.079304992 -
0.151150230.254287885 0.341132315 -0.072908561 -0.43834345
0.412995254 0.201664504 -0.027436247 0.488839736
0.035909585 0.212196025 -0.138551402 0.109176275
0.485341284 0.367694313 -0.182952015 0.089377817
0.23290067 -0.069682523 0.303979115 0.187148396 -
0.262262761 -0.204029923 -0.293123673 0.075112359 -0.235860337
-0.461501027 -0.226245934 0.486305607 0.193920282 -
0.165896142 0.106461974 -0.090522518 0.260271386 0.503509383
0.138550983 -0.478173896 -0.220935727 0.524107535
0.03827465 0.074235075 0.031443204 0.322668625 -
0.236518961 -0.459580583 -0.534266536 0.123296677 -0.154952017
-0.239965847 -0.166893056 0.371657854 0.18199097 -
0.405681707 -0.540582176 0.48126715 -0.371006614 -0.107929568
0.357738175 -0.19115824 -0.370954908 -0.402484136 -
0.07197703 0.129833747 0.145616857 0.459879542 -0.005131996
-0.326099354 0.004676548 0.098805499 0.171359103 -
0.029530587 -0.255804127 0.088795131 0.210083348 0.020736078
-0.036869195 -0.031396904 -0.063096514 -0.06380258
0.260774704 0.028657175 0.002418753 0.324131722 -
0.294861047 0.184955023 0.12698739 0.272581411 0.385555131
0.16170059 -0.334268441 -0.458991606 -0.126627332

0.42700866 -0.210414963 0.309327815 -0.282029538

0.306197029 -0.422924225 -0.43242365 0.285564385 -

0.13241494 0.243095512 0.480384705 -0.302826644 -0.177077068

-0.22105613 -0.416026437 -0.383689028 0.232357377

0.329456292 0.134316153 -0.203164457 -0.155098526 -

0.198613226 0.446132497 0.483490613 0.067079955 -0.244889127

-0.025219145 -0.289494711 0.370858539 -0.262042197 -

0.295852002 -0.307094872 0.288654072 0.237462327 -0.011445587

-0.28243969 -0.463849708 -0.031664812 -0.115758726

0.157974798 0.060908861 -0.361265143 -0.16607009 -

0.00718461 0.522787002 -0.305187597 0.016011391 0.419403977

0.25664444 -0.258182436 0.071446585 -0.317025189

0.292326803 0.17328068 -0.321465618 -0.03861078

0.007147345 -0.137666682 0.291558483 0.445436441

0.072443343 -0.247140975 0.048626887 0.36731357

0.193043767 -0.362150088 -0.104946997 -0.11302043 -

0.221067774 0.171322882 -0.368237451 -0.290635927 0.256959016

0.071080059 0.434853708 -0.439360289 -0.052835911 -

0.193399703 -0.230449744 -0.299169658 -0.311980794 -0.225682011

-0.186003205 0.311076157 -0.088712615 -0.308310231

0.455489337 -0.116279437 0.13969391 0.19792439 -

0.164028258 -0.018208968 -0.48795062 -0.451497482 -0.044192478

-0.265165451 -0.264615346 0.119187804 -0.319818308 -
0.035660442 -0.361344933 0.322317233 -0.49956718 -0.307948638
0.368198299 -0.115634891 -0.36670064 0.227825943 -
0.429608164 -0.136656598 -0.06761596 0.163213999 -0.099145835
-0.021324127 -0.01179714 0.485720808 0.23364423
0.028245866 0.303335689 -0.056097825 0.11315727-0.526070343
0.156342702 -0.369427246 0.466171348 0.00633137 -
0.504078053 -0.411556952 -0.100903192 -0.521490746 0.137828968
0.15655128 -0.520224324 -0.347432023 0.337916572 -
0.321396598 0.221387901 -0.286585974 -0.359061935 -0.058551688
-0.116886065 -0.069406962 -0.277966356 -0.077710507
0.330171613 -0.005261025 -0.278217663 0.252136853
0.10778012 -0.153389 -0.159576288 -0.121001668 -0.45117706
-0.402965886 -0.040705334 -0.025096384 -0.099122251
0.452737687 0.195350665 -0.533378358 0.256937852 -
0.195563153 0.243449317 -0.157059502 0.311093816 0.403100692
-0.300040021 0.297608656 0.344210064 -0.092444772 -
0.436590806 0.063398107 -0.148362947 0.013888652 0.054212196
-0.236681865 0.444996999 -0.22095251 0.256582748
0.222406512 0.295754255 -0.118769304 -0.138521256
0.070294313 -0.152546052 -0.411695776 -0.407219881
0.187020894 0.202696922 -0.310190849 0.177479094 -

0.338820744 -0.199962444 -0.370000596 0.074311425 -0.130883957
-0.177337093 0.089284994 -0.370779917 -0.340359267
0.213254342 0.302586143 0.128030477 -0.322937774 -
0.070188017 -0.235708205 0.376053215 0.475887527 0.17063945
-0.173373075 -0.017271754 -0.33700687 -0.259465923 0.2935816
-0.107664066 0.09663258 0.492066724 -0.029873613 -
0.307809635 -0.428635057 0.120063143 0.23161471 0.066577086
-0.511733763 -0.112212172 -0.203981355 -0.428818695
0.177935974 -0.067202324 -0.210638279 0.250979279 -
0.092994197 0.156420059 0.158851534 0.227374676 -0.099557522
0.366462967 0.065486756 0.023029031 -0.200812015 -
0.187737713 -0.463983008 -0.004010234 -0.424241102 0.35745887
-0.248061722 -0.026957495 0.361580005 -0.119843116 -
0.254617004 -0.036976249 0.200376007 -0.014708278 0.102491947
-0.227883266 -0.332804308 0.010383019 -0.045087059
0.016411904 0.052232119 0.409957484 0.496366612
0.07409773 -0.152368858 0.058554445 0.257894743
0.309606388 -0.098587875 0.393834642 -0.075119979
0.204788442 -0.413745004 0.201567039 0.332270363 -
0.028760683 0.109846343 -0.444846769 0.017018499 -0.245295987
0.327306844 0.259967685 -0.167290153 -0.155523856 -
0.269921215 -0.101768435 0.168384886 -0.555640334 0.125886032

7996 0.139787308 0.344114567 -0.434619311 -0.104572069 -
7997 0.033636749 0.149184445 -0.423964282 -0.334354157 -0.113373285
7998 0.236193 -0.286307432 -0.187114735 -0.425297615 -0.358160138
7999 0.307748428 -0.019799052 -0.188618077 -0.111473057 -
8000 0.294593035 0.075719002 0.183654404 0.112581183 0.153647108
8001 -0.181408258 -0.424926596 -0.483876199 0.582157443
8002 0.510086794 -0.37811657 -0.247204333 0.124504234 -
8003 0.388377528 -0.194741633 0.006248319 0.185273578 0.020348684
8004 -0.113367913 0.000592778 0.520352909 -0.085919137
8005 0.059923996 -0.007509839 0.489574506 0.093418511
8006 0.003558705 -0.07797683 -0.541839501 0.081317581 -
8007 0.336159427 -0.305649782 -0.524674401 0.4094592 -0.010333678
8008 0.492685586 0.329211598 0.414352562 -0.154031939
8009 0.232777775 -0.22899233 -0.060290245 0.326734249 -
8010 0.200911624 0.18391655 0.039461331 -0.258945471 0.116612113
8011 0.15731654 -0.091922549 -0.438074061 -0.175060164
8012 0.36801043 0.111910773 0.273966781 -0.167404552 -
8013 0.292683573 -0.031557135 -0.51064432 0.119047126 0.386434713
8014 0.450767031 -0.010606479 0.411431340.212972648 -0.0509931 -
8015 0.200345452 -0.232079665 0.078072031 0.384320062 -0.293605665
8016 0.021365783 -0.361696547 -0.47203486 0.28129868
8017 0.006035023 -0.195500956 0.051269063 0.236431789 -

0.023635687 0.334518093 0.322325547 0.046850271 0.145393319
0.016384257 0.063619596 0.124761719 0.194048615 -
0.333490913 -0.069914981 0.207001852 0.193625496 -0.199206294
0.242419731 -0.316250396 -0.169626106 -0.070463925
0.138463876 -0.131885062 -0.378635138 -0.301067965 -
0.302417198 -0.028674914 -0.35414043 0.090086124 0.284497397
KEGG_CARDIAC_MUSCLE_CONTRACTION 0.007419319 -1.90E-01 -
1.40E-01 -0.311658564 -0.139593098 -0.286824135 -0.030380089
0.037555635 -0.370387851 -0.067354322 -0.247359331
0.099089582 0.255118463 0.112340488 0.409891181
0.464452333 -0.076302415 0.113265092 0.101438838 -
0.115180858 -0.267611105 -0.103659733 0.003863914 -0.39086135
0.258652067 -0.065328559 0.346271573 -0.127312587 -
0.293125791 0.126134504 -0.011861674 -0.41881112 -0.26409671
0.197114204 0.091227236 -0.364980233 0.34699412
0.359431166 0.036620574 -0.463007717 0.317516652 -
0.063510758 -0.393343791 0.09583771 0.161640846 0.360594789
-0.239717893 0.325558105 -0.079821172 0.241659007
0.486475522 -0.216651853 -0.021431973 -0.11653044 -
0.02024033 0.051390595 0.220337106 -0.098189041 -0.257483855
-0.127197317 -0.174428665 -0.201390791 -0.098464639 -
0.344442212 -0.002447777 -0.317064766 -0.38786181 -0.011090643

8040 0.295190313 0.17575651 -0.33587762 -0.149380271

8041 0.371543269 -0.084349606 -0.220580385 0.212829684

8042 0.283615378 -0.131246977 0.076285417 0.202621266

8043 0.007830335 -0.368073797 0.057976852 0.300192706

8044 0.015434368 -0.149050465 -0.001068583 -0.070933548 -

8045 0.229410863 -0.158844222 0.206408656 0.534343543 -0.188513646

8046 -0.026425638 -0.202360732 -0.280812977 0.34821657 -

8047 0.166922623 0.192257277 0.218309466 -0.008110419 0.19269237

8048 -0.35164675 0.431715349 0.187464398 -0.363762854 -

8049 0.185612323 0.308555085 -0.353104762 0.411219246 0.209734043

8050 0.122981679 0.203140538 0.190377988 -0.499428561 -

8051 0.433562648 0.259003259 0.181979999 0.113826099 -0.160754421

8052 0.212776434 -0.094134042 -0.155418736 0.468710418 -

8053 0.324203369 0.416768618 0.442403345 -0.447098128 -0.296995083

8054 -0.356328884 0.124189254 -0.061744202 -0.064880054 -

8055 0.400414472 0.228301789 -0.258211844 -0.069262997 -0.196014036

8056 -0.360123607 -0.239153519 0.190297157 0.280739212 -

8057 0.390871751 -0.085772687 -0.115966438 0.339207227 -0.191204943

8058 -0.14722104 0.154145209 0.310632893 0.281617212 -

8059 0.297004248 -0.144681328 0.367513079 -0.391754213 0.346714766

8060 -0.344507336 0.24881069 -0.362080554 -0.001740756

8061 0.213398563 -0.177031774 0.032271703 0.088251463

0.229403204 -0.323079282 0.143143829 -0.354039032
0.246733309 -0.323114081 -0.171315787 0.255745185
0.18191092 0.239186698 -0.353539757 0.208364112 -
0.406835876 -0.176778738 -0.063790955 -0.209895367 0.350680961
0.378415949 -0.09625217 0.059680653 -0.213500513 -
0.230687729 -0.184775533 -0.298059978 0.281307442 -0.29851966
-0.154008872 0.422779137 -0.208050677 0.292327769 0.1907509
0.232914627 -0.367358326 -0.074782524 -0.060018913 -
0.253489718 0.315688986 -0.374565646 0.44150481 0.449786966
-0.1249519 -0.090702884 -0.156718362 -0.17335169 -0.337610986
-0.273116151 -0.23017118 -0.360122099 -0.309177815 -
0.027153913 0.305643033 -0.216450012 0.194818961 -0.356391083
-0.396777939 -0.43339279 -0.020072001 0.05804138
0.24269396 -0.195756155 -0.104381423 0.303285008
0.353693775 0.289514888 -0.326805798 0.457452406
0.298721168 -0.166261138 -0.156311394 -0.257014999 -
0.330646319 -0.043438621 0.064942725 -0.442582271 -0.276306529
0.277801589 0.034436038 -0.457648109 -0.086563764 -
0.091583237 -0.040442524 -0.256916758 -0.206316091 -0.122003283
-0.086374389 0.189478893 0.166570958 -0.003221489
0.276213695 -0.197916146 0.434710984 -0.012110523
0.190282855 0.448504691 -0.231987426 0.067648738 -

8084 0.021131005 0.105840772 -0.319284246 0.3501098 0.031681376 -
8085 0.270407186 0.239425391 0.400209124 -0.431089885 0.257098448
8086 0.197796666 -0.034902354 0.088201978 0.33060674
8087 0.086949336 0.125467544 -0.165232427 0.157627584
8088 0.092351278 0.227027381 0.101006796 0.329675956 -
8089 0.218639115 0.281530761 0.054605025 0.44374643 -0.025390424
8090 -0.387679841 -0.043853305 0.155058833 -0.214747267
8091 0.298593099 -0.271721659 -0.355021965 0.144089849
8092 0.296465005 -0.461164333 -0.39328977 -0.190274097 -
8093 0.234467778 -0.500052723 0.085408205 0.098196595 0.360292426
8094 0.300450378 -0.120948258 -0.23350188 -0.374342714
8095 0.146535187 0.241293793 -0.069689667 0.092488001
8096 0.135853995 -0.449059259 0.083278436 0.065757332 0.3816331
8097 -0.371246282 -0.08071244 -0.262448679 0.499122436 0.4450793
8098 -0.083633289 0.450832668 0.055361466 0.298587166
8099 0.217839801 0.376325287 -0.437118172 -0.138627328 -
8100 0.261252339 -0.062877508 0.036343668 0.394833552 -0.250152025
8101 -0.190501574 -0.341720362 -0.21651738 -0.453867299 -
8102 0.272296935 0.209606584 -0.035390567 0.005718405 -0.019169898
8103 -0.365208653 0.213371803 -0.400244163 -0.226304431
8104 0.193054867 -0.355785297 -0.166946192 -0.227610454 -
8105 0.265743118 0.149123722 -0.247997712 0.267435543 0.394845599

8106 -0.222938325 -0.080252757 0.47004202 0.149746149

8107 0.454117763 -0.437661319 -0.090329299 -0.090642273 -

8108 0.302658067 0.157707693 -0.219762878 0.130367488 -0.392092031

8109 -0.2255689 -0.265792173 -0.328435897 -0.291762027 -0.39366359

8110 0.142664163 0.456237558 0.238460917 -0.10139329 -

8111 0.436904303 0.323772129 0.229403035 0.018760332 0.215316011

8112 -0.231745705 -0.025501888 -0.289187034 -0.043728758 -

8113 0.183468744 -0.307520741 -0.053412166 0.0908968 -0.107905678

8114 0.157596277 -0.038229866 -0.058723855 0.01396242

8115 0.219898476 -0.260828227 -0.298343907 -0.349050465 -

8116 0.259911993 -0.370051162 -0.082652731 0.143338848 -0.151640821

8117 -0.288273278 -0.238061314 0.206776713 -0.450973002

8118 0.030377021 -0.32551098 0.178314139 -0.116258677

8119 0.336173471 -0.321355322 -0.288294924 0.25596213

8120 0.068364128 0.246384193 -0.293264807 0.034858324

8121 0.19260775 -0.326527723 0.422364253 0.244093261

8122 0.15078798 -0.253167577 0.214142759 -0.261521907

8123 0.212516769 0.076226625 0.284328824 0.255895969

8124 0.081730312 -0.201827065 -0.19091308 0.360600127 -

8125 0.139285423 0.144523816 -0.257432376 0.158926042 -0.330809836

8126 0.242921502 -0.072061317 -0.491217716 0.516516027

8127 0.014451048 0.353584322 -0.017189447 -0.226015837

0.059821731 -0.338199738 0.479631728 0.229463443 -
0.261659618 -0.260502362 0.3884965 -0.094150886 -0.285159014
0.13529053 -0.345895929 0.167459439 0.252226564 -
0.294458137 0.158054531 -0.351217586 0.267248066 -0.306113392
0.088545146 -0.212969256 0.150223794 -0.227278338 -
0.337885983 0.119527372 -0.267273531 0.315492696 0.263362303
-0.553753531 -0.320055741 -0.295503689 0.238163407 -
0.257392735 0.519474463 0.361799699 -0.34930745 -0.343074923
0.129564169 -0.161715248 -0.003008623 0.433946821 -
0.037721407 -0.257237114 -0.397403108 -0.019052239 0.136896133
0.257438969 0.308756567 0.180476002 0.313688682 -
0.217532854 0.170606832 0.161584509 0.020352234 0.088516086
0.022963579 0.191410429 -0.026545524 0.24604911-0.424446696
-0.382346898 0.265163946 -0.036039301 0.279075383
0.061183333 0.164975455 -0.14186855 -0.236139729 -
0.334197101 0.202554123 0.054989532 0.298048425 -0.115115096
0.432622968 -0.145182457 0.13561747 -0.217758046
0.027360198 -0.10205324 -0.196914118 0.368339024
0.023302206 -0.031993372 -0.265832667 0.223097017 -
0.416666115 0.179205135 -0.08724439 -0.041914459 -0.071787521
-0.005966604 -0.039841567 0.356343375 -0.078858608 -
0.127311054 -0.183166327 -0.090443025 0.163959726 -0.181704929

0.02244007 0.193501687 -0.098606958 0.07194777
0.081845015 0.299809465 0.2728365 -0.171509473 -0.003188393
-0.070471697 -0.176445222 0.048667223 0.202352818 -
0.039810138 0.13256212 0.286481131 -0.064621969 0.208433724
0.149307329 -0.256688016
KEGG_RENIN_ANGIOTENSIN_SYSTEM 0.21922619 0.356507732 -
5.15E-01 -0.012554738 0.127464447 0.360250631 0.365229953 -
0.198805573 -0.206889343 -0.393051626 0.01314305 -0.614341272
0.237816722 -0.425632734 -0.298156943 0.068225975 -
0.41324106 0.016373971 0.048076405 -0.490148419 -0.147497178
0.282266303 -0.435194728 -0.193097746 -0.070879161
0.035902893 -0.074126094 -0.013464346 0.158809557 -
0.186145071 0.455123131 -0.213594747 0.016761374 -0.024326346
-0.078612421 -0.136083354 0.261192126 0.34482763 -
0.312172279 -0.160099738 -0.420678349 -0.004737245 -0.235971943
0.607256885 -0.567520329 0.023553909 0.236688917 -
0.328127273 0.463502936 0.062604818 -0.464141739 -0.21196213
-0.067410018 -0.4158249 0.239948972 0.454549405 0.16157042
-0.115872893 0.39307661 0.07127583 -0.739508453
0.06301637 -0.215303901 -0.080597721 0.116009932 -
0.356795612 -0.402748379 0.269407304 0.124611441 0.122767565
-0.561881968 -0.500674992 -0.131097391 -0.325442653 0.4379403

0.288515247 -0.283449909 0.065222958 0.537979183
0.347438631 0.165139968 0.393032846 0.281421757
0.154102912 -0.037251993 -0.157654128 -0.25227113 -
0.506919701 -0.283490618 0.110285077 -0.43420734 0.269367467
0.236260931 0.260448832 -0.335520992 0.131773841
0.263314705 -0.441914118 0.500895839 0.027765264
0.079214329 0.032552647 0.108852552 0.064068025
0.371393761 -0.311042081 0.549851132 0.08648746 -
0.471657947 0.220983537 0.394546247 0.184136537 0.383095244
-0.22663343 0.183172354 -0.036824338 0.116802451
0.236396793 0.063743021 -0.520406233 0.38626881
0.522741156 0.295433688 -0.017590981 0.06285359
0.567289874 -0.394772796 -0.313094004 0.371576859
0.021601633 -0.485718811 -0.481056082 0.12204352 -
0.07839936 0.002441733 0.062814959 -0.523248582 0.425142002
-0.097093263 0.072393524 0.268614806 0.225304691
0.360908705 0.294382811 -0.341425939 -0.230039954
0.534320934 0.202520631 0.393962161 -0.246022345
0.02103297 -0.039026405 -0.324572217 0.141990081
0.218377866 0.389184611 -0.280853662 0.03334892
0.001143111 -0.331180803 -0.440351837 0.13868271
0.104059236 0.12745754 0.542646684 0.041363214

0.496941124 0.361364933 0.18384027 -0.224355061
0.290251203 0.467322885 0.324910003 0.551588489
0.027561221 -0.07819016 -0.154145951 0.304080396
0.381791272 -0.563935989 0.167675332 0.200971707
0.496885067 0.029806973 -0.002512403 0.374889198 -
0.479459092 0.105097676 0.020823037 0.494733247 -0.476043195
0.415268814 0.214679572 -0.336521147 -0.034733802
0.353749085 0.510174921 0.392798278 0.406181567
0.366406666 -0.348855841 0.28685453 -0.035277545
0.223452777 -0.244466713 -0.14113321 -0.004118896
0.137252764 0.37983017 -0.299205801 0.573709598 -
0.312625522 0.334602536 -0.296684517 0.372356312 -0.351545619
-0.06044121 -0.287937572 0.386542583 -0.208904908 -
0.321156785 0.090765472 0.460653882 -0.475946701 -0.229248686
0.211725944 -0.528602738 -0.111046089 0.467784188
0.460286892 -0.169958006 0.346125065 0.151563612
0.211854253 -0.068537575 0.405033412 -0.343459076
0.051158018 -0.137955658 -0.038311007 -0.38084989 -
0.313070608 0.001718417 0.038678406 -0.481957685 0.569940833
-0.04958454 -0.131752591 -0.190096935 0.53652762 0.0966674
-0.037943186 0.115160184 -0.610095342 0.180305073 -
0.037009704 0.582411785 0.053547469 0.451550799 0.013365618

-0.508813924 0.402418632 0.068818164 0.16039266 -
0.01500278 -0.271960146 0.392018147 0.344385971 0.200380622
0.42431821 0.311344421 -0.561670927 -0.091811293
0.084855928 0.150354302 -0.242394559 0.051554638
0.421936503 0.031613235 0.120930971 -0.379512647 -
0.053760397 -0.169685242 0.255202368 -0.437935341 0.196988846
0.071827743 -0.096483198 -0.089263162 0.004618932
0.407487111 0.336318617 0.09732749 0.130930961
0.112567186 -0.23496852 0.551205639 0.346929727 -
0.101488824 0.190414889 -0.080827938 0.079748532 0.278002036
0.071046349 0.487725648 -0.093713364 0.058576837
0.533534035 -0.457634588 0.031725394 0.064929277 -
0.212363423 0.370826305 0.282324467 -0.294026389 -0.449171139
0.047594382 -0.161292693 -0.012853039 0.520823918 -
0.185127455 0.288185301 0.156809624 0.392116434 -0.295948659
0.43304168 -0.078697911 0.140134265 0.338651348
0.412508534 -0.565713093 0.537144129 -0.267194414
0.600519719 0.319102199 0.591197988 0.274685681
0.050316502 0.38526989 0.237853681 0.393575041
0.100134637 0.027777778 0.100535367 0.413014647
0.228574948 -0.270320821 0.343384023 0.291715373 -
0.539374712 -0.625124177 -0.046587567 -0.108828236 -0.261621165

0.263528589 -0.025761126 0.149023511 0.056841903

0.368984084 -0.053427611 -0.207432913 -0.307647368

0.020799309 0.024759763 -0.097019709 -0.129517037 -

0.456487715 0.054254986 -0.338681015 -0.206412152 -0.289452745

-0.304779271 0.39093101 0.509244662 -0.391174053 -

0.283181597 -0.217217982 0.113420373 -0.5718884 -0.267949914 -

0.334278241 -0.144917243 -0.497264984 -0.21528688 -0.147915229

0.535746635 0.176033268 -0.17935184 0.042903648

0.053500134 -0.428159942 -0.074752868 -0.138787288

0.148119309 0.256228314 0.465695765 0.14457764 -

0.427023761 0.070147064 0.464795818 0.219293501 0.121763372

0.007030933 0.636488014 -0.180496985 0.200455066

0.272733552 0.590107279 -0.360975473 0.263406353

0.417489062 0.217501173 0.19408276 -0.185640307

0.104373269 -0.438331076 0.398587581 0.021567222

0.174440063 0.283605083 -0.494360759 0.229786865

0.093891144 0.033225974 0.307410066 0.098299146

0.591369072 0.047049495 -0.451188097 0.156960181 -

0.141732409 0.439684563 -0.05720425 0.20250707 0.003672434

0.109513339 0.1955833 -0.401919234 -0.542714524 -0.393632366

0.420393522 -0.496894992 0.139637716 -0.025682644 -

0.19964299 0.169678474 -0.208158432 -0.456307085 -0.502364532

8260 -0.091196487 -0.641460555 0.513292047 0.189873489

8261 0.29460187 -0.200523947 -0.404761578 -0.130970241 -

8262 0.456115316 -0.509638296 -0.023065021 0.524546341 -0.14615372

8263 -0.414019582 0.261684417 -0.295787786 0.157486711 -

8264 0.230241009 -0.250893143 0.383118578 -0.255429755 0.076431313

8265 0.202234093 0.356289789 -0.130418205 -0.549830834

8266 0.058744587 0.107161421 0.08135365 0.447731757 -

8267 0.056446198 -0.201081587 -0.151517869 -0.165929868 0.093090497

8268 0.332952915 0.256421264 0.218776544 -0.149783904 -

8269 0.102335696 -0.349645243 -0.185268206 0.007505417 -0.008927485

8270 -0.329788176 -0.163980106 -0.159431097 0.238802422 -

8271 0.221968001 0.115266314 0.282504796 0.100422535 0.436221554

8272 0.636847052 0.388420306 0.0724268 -0.277770055 0.058110939

8273 0.441473459 0.298021464 -0.045614883 0.066426177 -

8274 0.250834626 0.17386417 -0.04196994 0.359528514 -0.012370704

8275 -0.126552711 0.126209546 0.049386978 0.477772934

8276 0.397413558 -0.325328111 -0.01754094 -0.40586918

8277 0.189546866 -0.383094201 -0.191167552 -0.141191711

8278 0.137163663 -0.244320835 0.408837958 -0.009249955 -

8279 0.180936246 -0.244479214 -0.441927338 0.286709954 0.290321418

8280 0.243957694 -0.1942552 -0.38486662 -0.157486969 -0.25519501

8281 0.057747523 -0.348162674 -0.164291458 0.175722203 -

0.149150806 -0.388970054 -0.275679668 0.161603015 0.469282208
-0.161493831 0.045427261 -0.357645001 0.210968113
0.255757132 -0.020387015 -0.351460395 0.34268811-0.158850031
-0.40660583 0.412669267 0.086343696 0.428373577
0.011717054 0.294642627 0.179323608 0.166106241 -
0.060340548
KEGG_CIRCADIAN_RHYTHM_MAMMAL 0.14038908 0.116348394 -
1.11E-02 0.409267926 -0.024352427 -0.361888012 -0.214676154 -
0.599831826 -0.52118402 -0.604356083 -0.329884321 -0.166942435
-0.126423677 0.137900568 -0.562772027 -0.43403787
0.588079265 -0.408895429 -0.23989808 -0.079583774
0.332164323 0.526394957 0.252633613 0.499581685 -
0.588652477 0.422903562 -0.305383059 -0.20363931 0.058685618
0.243226397 0.51289659 0.141757285 0.575779181 -
0.169400748 -0.4066838 0.291083596 -0.339785348 -0.557897114
0.297268818 0.654269777 -0.324450563 -0.155276351
0.46239346 -0.276416567 -0.33247857 -0.121737665
0.419410645 -0.314763804 0.41793806 0.092054248 -
0.059098233 0.309051686 0.124628647 0.329954455 -0.021960598
-0.134075756 -0.490842994 -0.003440958 0.36235533
0.009894675 0.206188924 -0.586566362 0.182244975
0.521526027 0.119855711 0.421977726 -0.111028179 -

0.276995588 -0.608069691 -0.177052919 -0.542673869 -0.210955759
-0.170629805 -0.14719027 0.225188582 -0.261909452 -
0.33633597 0.632072575 -0.609204099 0.247086983 0.466711625
0.541982558 -0.302045489 -0.370359587 -0.433885492 -
0.157838446 0.432995822 0.098121305 0.004308529 0.147621366
-0.489401063 -0.510622564 0.437146515 -0.262375919 -
0.292928272 0.418413106 -0.171616112 -0.217475794 0.234100465
-0.027581714 0.020074731 0.194070685 -0.141329752 -
0.185595517 -0.082987489 0.022812636 0.178826646 -0.214401372
-0.094406304 -0.34551059 -0.088940537 -0.034360557 -
0.147734743 -0.113531064 0.343343078 -0.355017602 -0.287093588
-0.429516518 -0.220818124 0.267334598 0.315158882
0.060320154 0.2695129 -0.464547579 0.256967203 -0.207125268
-0.516509044 0.141002541 0.737828172 -0.077488406 -
0.328491818 -0.342045868 0.238071646 0.355131184 -0.089399954
0.263341163 -0.187882645 0.016811709 -0.002278046
0.339501169 0.12841554 0.389386863 0.422604153
0.340974018 -0.263571284 0.091686127 0.245041146
0.312460702 -0.016966198 -0.463562544 -0.371404885 -
0.611355541 -0.503021439 -0.441931154 -0.153212972 -0.281022733
0.260893473 -0.277519825 0.466461715 -0.347885236 -
0.070523515 -0.294459125 0.430475612 0.477936371 -0.128842135

0.442652394 -0.158334364 -0.300559368 -0.357698475 -
0.316445408 0.584325557 0.314253348 0.134252253 0.256569603
0.143250056 -0.024319156 0.275704128 0.66993919
0.456817027 -0.538691923 -0.375376053 -0.226900249 -
0.066900822 0.016104343 0.092404091 -0.206546204 0.595478667
-0.221908722 -0.211927 0.363864712 -0.381371702 -0.532708463
0.29633775 0.334553707 -0.339452641 -0.379253794
0.19147864 -0.375770915 0.494585094 0.641608038
0.097820191 -0.135134245 -0.153143251 -0.53104354 -
0.469357171 -0.306835636 -0.471303743 0.097587495 0.339133489
-0.395269133 0.390333733 0.409172672 0.458323805 -
0.200724681 -0.462290313 0.085909549 -0.177396603 -0.301648841
0.214008575 0.598079609 -0.629876474 0.474584194
0.312300558 0.043292292 0.269229485 0.064738172 -
0.674568789 -0.447491235 0.473952249 -0.430374551 -0.315031668
-0.069587822 0.299715797 0.318378701 0.733545068 -
0.069917985 -0.377694139 0.345433535 0.349907374 0.006702927
-0.171734329 0.398157948 0.013262825 0.241186772 -
0.440984943 0.201654301 0.00156027 0.36517322 0.173771636
-0.015140264 0.465925046 -0.238173814 -0.319205376
0.006220247 -0.53751133 -0.247129452 0.016289272
0.281209207 0.443042236 0.498488148 -0.419426699 -

0.376263435 -0.080250276 -0.125510366 -0.371166232 -0.133072074
0.255939836 -0.348786631 0.367512979 -0.663444727 -
0.390964774 -0.151822141 0.024909578 -0.644103363 0.133163201
-0.309046853 0.189908157 -0.020136211 0.302401015 -
0.041078583 0.061816665 -0.558305168 -0.442973482 -0.072752583
-0.472029038 -0.220725786 0.14356683 -0.004305711 -
0.011032057 0.297716563 0.635280894 -0.482919376 0.509083649
0.056787628 -0.179633806 -0.188816831 0.268889833
0.346230466 -0.382177316 -0.040784203 0.357277452 -
0.089768574 -0.05433341 0.034227126 0.040668964 0.375112516
0.071792167 0.109978014 -0.172808863 -0.2950913 0.391722667
0.252381176 -0.521228318 0.609741398 -0.502472348 -
0.026304836 0.049224748 0.439577687 0.058412273 0.095140884
-0.47049845 0.360462043 -0.031629512 -0.42287555
0.001046497 -0.365482894 0.195465833 -0.149454349
0.343635148 -0.342834943 -0.381813654 0.512171366 -
0.00560327 -0.004542457 0.443342192 0.231248522 0.528586293
-0.2127829 0.28963664 0.084678003 -0.506257606 0.337065137
0.149564056 0.297343371 0.119017004 -0.505141151
0.596071036 0.376548969 0.025046014 0.411689292 -
0.322256147 -0.234686482 0.358168143 -0.154580098 0.370632462
0.235154854 -0.076858891 0.185418966 0.164482637 -

0.517385477 0.353094321 -0.10720679 0.322179618 -0.369614858
-0.235481257 -0.128507583 -0.534133872 0.199939087 -
0.063517812 0.395081701 -0.538735747 0.061176643 0.628823795
-0.09773603 0.443893253 -0.294357031 0.162563407 -
0.514624911 0.135021785 -0.239755363 -0.071197483 -0.33740474
0.4414191 -0.465861483 -0.513392136 0.339993743 -0.341027802
-0.2483531 0.115672660.048897243 -0.120777844 -0.353238715
0.136776922 -0.057453443 0.044199257 0.161582052
0.301864406 -0.473353301 0.483474724 0.507396435
0.056965191 0.462008023 -0.081153869 0.042653812 -
0.440615933 0.106775143 0.435767249 -0.148875001 -0.075582034
0.58682636 0.569465253 0.389593118 -0.480853118 -
0.306929921 -0.101677092 0.369428591 -0.028244551 0.126462234
-0.1260653 -0.389816382 -0.542629135 0.081011774 0.020269232
-0.298301058 -0.561229771 -0.014890257 -0.285355028
0.050184959 -0.161269864 0.21129964-0.296314783 -0.076911354
-0.222376107 0.404030577 0.299237937 0.148568389
0.246793005 0.283413048 0.241267809 0.286142514
0.376328272 0.241804858 0.193458059 -0.066240544 -
0.114404799 0.033715882 -0.572485021 -0.316789868 0.606570923
-0.171749281 -0.493204956 0.403059102 0.703125079 -
0.424282117 -0.520914756 0.285543551 -0.041807151 0.030866676

0.311744055 -0.289356526 -0.336113594 -0.099701473 -
0.540373757 -0.073013455 0.238332479 0.436386458 -0.01177053
-0.634577425 0.273247269 0.418597379 0.123543375
0.101119748 -0.176084946 0.453649403 0.189952195
0.464261432 0.07887139 -0.141988424 0.267766282 -
0.288557027 0.396819715 -0.271794721 0.296061119 -0.154305727
-0.574419906 0.08531154-0.009115989 -0.170579265 0.273432986
0.600590595 -0.182626097 -0.079419738 0.131825521 -
0.327228783 -0.372744819 0.183595212 -0.349422343 -0.354820872
0.102851091 -0.459983689 -0.240474913 0.204259023
0.28528256 0.567379098 -0.485917765 -0.409612989 -
0.610809283 -0.536262563 -0.539121291 0.500799145 0.173251691
-0.281498752 0.341886577 -0.501539652 0.046543676 -
0.35019873 -0.111583266 0.316361674 0.656743111 -0.005206793
0.13013305 0.10925689 -0.147336801 0.282316726
0.193073328 -0.155642974 -0.19918245 -0.3899793 0.350124291
0.317761099 -0.090789834 -0.208501376 -0.448866494
0.446300626 -0.542480603 0.123366613 0.088226907
0.49046852 -0.063062939 -0.020963935 0.281392648 -
0.517571447 -0.322124959 -0.048925474 0.448545713 0.323325525
0.008844186 -0.498177671 -0.310409954 0.372218836
0.200778586 0.240429215 0.060590519 0.002944304 -

0.036194408 -0.313063228 0.470171593 -0.370869156 -0.258909449 -0.036099779 -0.279543635 0.358417352 0.384323028 -0.409510158 -0.100404933 -0.136332847 0.121285392 -0.039973278 -0.384802841

KEGG_TASTE_TRANSDUCTION -0.507211163 -0.473340189 -7.97E-02 0.086795706 0.38419837 0.27367598 -0.231531614 0.448542422 -0.027297458 0.308711221 0.537139177 0.121256058 0.153447283 0.221344803 0.414325728 0.374976813 -0.283048542 -0.133987162 0.490543735 -0.067060024 0.028510528 -0.220844816 0.213740862 -0.211820727 0.240948412 0.151634277 0.015085447 -0.145395303 0.260648579 0.496570663 -0.19201033 0.110980018 -0.167816688 0.301728848 0.011995713 0.002925356 0.087887823 0.178251688 0.447911754 0.130552697 0.366939601 0.04873878 0.041663602 -0.390830364 -0.201512116 -0.28331993 -0.042209305 0.349427653 0.397417005 0.377791487 0.134262442 -0.086304027 -0.299226926 0.284799824 -0.284991678 -0.309407656 0.368888885 -0.188869001 -0.394634742 -0.15030805 -0.26130461 0.013685891 -0.242495483 -0.134768646 0.432846213 0.219675485 0.081194812 -0.412802024 0.376891567 0.406692678 -0.204162737 -0.216009488 0.388889472 -0.224724033 0.318310239 0.433325936

0.301796342 -0.019514014 -0.037861233 -0.364548229
0.419453359 -0.302067347 -0.30497551 -0.091593874 -
0.183247414 -0.062806394 -0.106798965 0.236544462 0.285441504
-0.279568491 0.366621628 0.411163537 -0.065091667 -
0.489512038 -0.326582862 0.019299923 0.225803943 0.51073837
-0.396940843 0.093798144 -0.144863184 0.440841582 -
0.108911198 -0.15637405 0.123776308 0.201442356 -0.014738085
-0.086239928 -0.110521668 0.300648836 0.484792274
0.037198929 -0.161264988 -0.124368217 -0.167956566 -
0.456830164 -0.311440246 0.44494336 0.47856351 -0.344893396
0.529537894 -0.002898255 0.02948735 0.443534396
0.141448681 0.492848476 0.232424264 0.178877431 -
0.027625874 -0.42120876 0.092695003 0.527526018 0.196966357
-0.258411469 -0.174361809 0.098460944 -0.167660964 -
0.293808136 -0.001127107 0.155659871 0.35209442 0.221447164
-0.186785156 -0.133365741 -0.472336356 -0.184890298 -
0.411409248 0.201460782 0.567787318 0.165344463 0.309250991
-0.544145636 0.127587989 0.04657181 -0.193341487
0.391310421 0.161550249 0.394917293 -0.192920445
0.097009619 -0.236473084 0.066088996 0.058997867
0.322099503 -0.418736764 0.196248879 -0.214959784
0.16290935 0.457285264 0.561554969 -0.479293284

0.36358237 0.412256273 -0.107552923 -0.424152461 -
0.182008696 -0.105516092 -0.174268915 -0.212833502 0.014761893
-0.035884808 0.186003757 0.192327699 -0.084938929 -
0.116197517 -0.273748167 0.114443376 -0.031600963 0.51448306
-0.140655879 -0.268021677 -0.078029559 0.256762141
0.428500335 -0.374466366 0.239438181 0.125098909 -
0.37833313 0.338224483 -0.170028931 0.264064886 -0.317098976
0.153269956 0.272168954 -0.188561212 0.130299232
0.364960618 -0.282220523 -0.234745422 -0.464985051 -
0.550631193 -0.080913039 -0.156753166 -0.218202931 0.505774793
-0.095454197 -0.360415541 0.129261119 0.197262032 -
0.171651508 0.345774518 -0.281040798 0.28271306 -0.137069463
0.348060938 -0.110246076 0.132258392 0.424834849 -
0.363447306 0.092368716 0.047213408 -0.342455909 0.066009548
0.201989915 0.258638043 -0.113856914 0.394715963 -
0.217148166 0.153960573 0.282811352 -0.351877349 -0.129948977
0.099113679 -0.283994494 0.123693783 -0.283663077
0.359191538 0.42376182 0.305265749 0.323183478
0.243105478 -0.133703068 0.33380778 -0.013849693
0.04269597 0.268728041 -0.215097325 0.407454299 -
0.454278299 0.245736444 0.214709962 -0.325867954 -0.244906348
0.398935122 0.094893897 0.270005019 0.261039046

0.315893444 -0.302795326 0.543231197 -0.327251755
0.052392129 -0.329679859 -0.117572224 -0.232127039 -
0.223258548 -0.461042064 0.072113530.42222515 -0.07359768
0.516794827 -0.185678276 0.084144309 0.509346725
0.395379474 0.226962213 0.506713557 0.023661278 -
0.132291736 0.501175825 0.205957033 -0.109881212 -0.16484696
-0.420487548 0.508844498 0.390006564 -0.149310092 -
0.205887224 -0.122929141 0.170756035 -0.272691041 0.19493515
0.419015226 0.269140051 0.269360925 0.16609134 -
0.100566845 -0.372854707 0.122045792 0.28210256 0.104016445
0.087612463 0.069751489 -0.345810111 -0.206823293 -
0.096623027 0.410794667 -0.0116683 -0.440006434 -0.390451051
0.40421597 -0.251372501 -0.466366396 0.527345805
0.032517767 -0.118164345 -0.181269105 0.365127295 -
0.225773993 0.227289943 0.342386914 -0.134589827 0.347234515
0.06692386 -0.253748078 -0.365832832 -0.506595 -0.029202352
0.110890035 -0.39538478 -0.463852361 -0.34741939
0.550939312 -0.137402605 -0.499998785 -0.066552127
0.145919274 -0.323553485 0.281473065 -0.035616495 -
0.035444401 -0.298538637 -0.344757303 -0.243890437 -0.392751748
0.376787131 0.275616132 -0.223331208 -0.1645959 0.346635574
-0.228260528 0.434898973 -0.257269121 -0.093798671 -

0.235008247 -0.086670681 0.199474987 -0.189767027 0.302157629
0.221089537 0.184947521 -0.051819124 -0.412439024 -
0.297401061 -0.303307519 0.419918073 0.356473645 -0.143209105
-0.195787198 -0.226949545 0.291266528 0.375705296
0.478297459 0.339123709 -0.330461284 -0.073142738 -
0.040201874 -0.263431738 -0.387590887 -0.377092903 -0.258812686
-0.410128973 0.236447397 -0.05543151 0.048650869 -
0.291506542 0.176966415 0.056049588 0.422940448 -0.270115247
0.61705746 0.043201113 -0.370697708 0.021216405
0.353713825 0.103115634 0.20864407 0.002655453 -
0.076702556 -0.345844974 -0.370583324 0.049613518 0.280235474
0.363923282 0.319916264 0.200181953 -0.023744132 -
0.214612513 -0.24679826 -0.04517774 0.061537481 -0.33164584
-0.045305312 -0.245679621 0.273386101 -0.254424728
0.270049362 -0.534452974 0.137819707 -0.17034885 0.305131
-0.219774782 0.352605659 0.429472039 0.179041665
0.047032593 -0.419241179 0.236581667 -0.005841929
0.155146995 0.113284263 0.060863852 -0.049826637
0.535080095 0.263209124 -0.039382627 0.227814585
0.387478887 0.363711589 0.384802038 -0.17939217
0.328760686 -0.034293662 0.337408644 -0.22500195
0.15779316 0.170151888 0.307795929 0.113408616 -

0.368930871 -0.106308776 0.216773807 0.451963524 -0.29024165 0.010997094 0.018668515 -0.129097312 0.293537447 -0.329995847 0.250669752 0.253123153 0.20027401 -0.326494798 0.219968508 0.450282618 -0.048089778 0.236555366 -0.1294808 0.040895927 -0.120201175 -0.17817539 0.400590455 -0.028973374 0.347035824 -0.20976066 -0.375970708 -0.343794979 -0.24327767 0.196350973 -0.130564887 0.457466862 0.471939434 -0.182695877 -0.083418182 -0.115746429 0.298611377 0.243125275 -0.194005043 -0.075653008 0.176279589 -0.107877717 -0.225724747 -0.214802724 -0.062857345 -0.160199107 -0.09031495 -0.035218736 -0.239538908 0.404475991 0.005202715 0.328996028 0.223451024 0.151770745 0.22384611-0.035425159 -0.312623592 -0.168056871 -0.256653264 -0.125820133 0.249055337 -0.327077602 0.426374737 -0.368426141 0.030949856 -0.470571674 -0.131381546 -0.344353043 0.115049134 -0.263122387 -0.090629924 0.153449009 0.053546356 -0.407436596 -0.100808755 0.294103467 0.458120177 0.187289075 -0.164110891 0.502314241 -0.006543289 0.059265377 -0.182104251 0.268097621 -0.270812823 0.206422096 0.325408826 -0.152724349 0.003792448 0.36413288 -0.191101406 0.025319771 -0.008408433 -0.129691948 0.060238261 0.187971308

0.250207277 -0.502094156 0.029764758 -0.150822812 -
0.048586662 -0.414808497 -0.039699421 0.271559194 0.103388279
0.298949713 -0.110746805 -0.085760452 0.159651697 -
0.169024661
KEGG_PROXIMAL_TUBULE_BICARBONATE_RECLAMATION
0.544930012 0.428603815 -3.26E-01 0.322769861 -0.220056746
0.201091096 0.086754989 -0.414181992 -0.545697167 -
0.307745481 -0.296513055 0.116712302 -0.106290287 -0.186945944
-0.228873506 0.207663639 -0.371996942 0.245829113 -
0.427789947 -0.093649415 -0.238781096 0.22390207 -0.27068832
-0.100023386 -0.485792099 0.161656346 0.289878949
0.002680613 -0.00859857 -0.360787978 0.532724325 -
0.472380584 0.235659326 0.346491055 -0.093950524 -0.264464992
0.082523341 0.252162142 -0.374514529 0.084325744
0.382131521 0.346361391 -0.181002847 0.538960814 -
0.375317094 0.166558547 -0.043106151 0.141124186 -0.313946744
0.307110144 0.513021366 0.058481969 0.250551158 -
0.282180071 0.021654761 0.266452227 -0.346665932 0.305581534
0.308240117 -0.370668947 -0.368126838 -0.442875067 -
0.200629681 -0.132026183 -0.266975304 -0.37910812 -0.124564491
0.262877228 0.170324702 -0.053754529 -0.541189751 -
0.445245237 0.44776391 -0.246705438 -0.05832855 -0.161036264

0.369865137 -0.017981564 0.120646244 0.388841815 -
0.007868206 -0.083893937 0.117695252 0.052561374 0.408809759
-0.475197366 -0.013210454 0.045010379 -0.20389668
0.218777583 -0.235027257 0.209128423 0.253730967
0.404569524 0.210112244 -0.212141115 0.358038332 -
0.405031947 0.59569855 0.425517731 0.330376963 0.021985691
0.045119604 0.095728117 -0.212343376 -0.349985993
0.280168048 0.212906985 -0.343671667 0.289818768
0.36669777 0.279569189 0.489016712 -0.208100572 -
0.035012205 -0.296707664 0.097516722 0.22122036 -0.081518777
0.040099266 0.224045899 0.517477374 -0.165091539
0.292106849 -0.420110336 0.369103786 0.514341753 -
0.089636866 0.119424360.189364539 0.118104517 -0.415026142
0.145839233 -0.148602789 0.283255867 -0.567906295 -
0.521770086 0.212160797 -0.055426474 -0.147167401 -0.052151651
0.326955847 -0.142173498 -0.042234811 -0.541280078 -
0.167622286 0.466314365 0.265429124 -0.345144531 0.153139026
-0.342493265 -0.198774694 -0.504562192 -0.052852611 -
0.10177359 0.124692717 -0.540469181 0.521848474 0.261158833
-0.194409065 -0.26282385 -0.062819057 -0.103077155 -
0.212149464 0.453167367 -0.383405216 0.623795759 -0.114847466
-0.106513681 -0.339401844 0.234546807 0.403081945

0.27322512 0.418338359 -0.414437161 -0.146132244 -
0.332769911 0.445886084 -0.024028147 -0.512641583 -0.180374962
0.317894929 0.231421457 -0.023980926 -0.106263109 -
0.033697072 -0.318045549 -0.090330867 -0.407989425 0.556647648
-0.466619814 0.519422859 0.371489176 -0.222765153
0.097608026 0.136203759 0.023699071 0.420367016
0.147153624 0.265854248 -0.284248076 0.031650056 -
0.033093403 0.444064348 -0.469967377 -0.108304337 -0.410397414
0.157045099 -0.054081111 -0.352380379 0.513432036 -
0.155228722 0.199740421 -0.355674753 -0.024553225 -0.327747998
-0.209328101 -0.187898799 0.037799499 -0.316882965 -
0.498605862 0.470146026 0.397573789 -0.567273542 -0.41924727
-0.035967944 -0.400286351 0.15192271 0.289559488
0.514450817 -0.026144866 -0.287429678 0.132929312 -
0.069402209 0.15294866 0.390086262 -0.229164342 -0.252384808
-0.021204462 0.060672408 0.016559635 -0.449809844
0.00382038 -0.246815525 -0.441396666 0.112947242 -
0.271375638 -0.071828084 -0.012947249 -0.065117633 0.369122653
-0.012350376 -0.206009145 -0.530325766 -0.158390021 -
0.059567843 0.397406474 0.279390876 0.399525124 0.332211248
-0.376409731 0.202585846 0.277834413 0.091962287
0.302526999 -0.261544503 0.156084409 -0.089432368 -

0.013984896 -0.046278458 0.521169729 -0.557956186 0.186127102
-0.269927583 0.019129982 0.118372680.172410161 0.286146653
-0.056800292 -0.366819016 -0.135160992 -0.173065933 -
0.443253608 -0.023779385 -0.424256044 0.19632033 -0.098452563
-0.269893344 -0.419035864 0.076490737 -0.024358506
0.407460345 -0.211773193 -0.289170156 -0.246618125
0.302806461 0.271850146 0.067755839 -0.321901416
0.255494375 -0.138984341 0.36246945 0.211284989 -
0.09137653 0.438884231 -0.147574379 -0.509364636 0.426007745
-0.431831897 -0.079115911 -0.29176943 -0.228847106 -
0.498266151 -0.112641222 0.343527007 -0.077312981 -0.265345518
0.011638058 0.301520972 0.109036755 0.374484597
0.627043544 0.355904271 0.280372284 -0.405717759
0.374684863 0.085279479 0.483359774 0.405919174
0.09216012 -0.425033957 0.394567678 -0.012229586
0.54816323 0.029297471 0.322892279 0.412499878 -
0.32678533 -0.034219575 0.221330097 0.43944729 -0.16218353
-0.105842248 -0.100270761 0.203867834 0.050750704 -
0.427875905 0.448496603 0.339103456 -0.545234555 -0.493322823
-0.250962429 -0.25930089 0.315587855 0.192855274
0.085479659 0.190313293 -0.432820228 0.145367978
0.602256981 0.194367802 0.558265458 0.315416726 -

0.473231006 -0.385966871 -0.209462556 -0.035803746 -0.271443811
-0.353370581 -0.265952458 -0.495563594 -0.120818658
0.068003727 0.134883657 -0.053377338 -0.406264332 -
0.126539304 0.25799103 -0.382299375 -0.349620355 -0.126992503
-0.2824574 0.002497462 0.089015274 -0.401016873 0.575666109
0.066707982 -0.409645628 0.436645698 -0.369787959
0.108784029 0.305284426 -0.278240876 0.363128721 -
0.065010234 0.421939459 0.179504246 -0.327487791 0.110813645
0.282499252 -0.057657322 -0.12347968 -0.059027587
0.550451902 -0.42038335 0.325116503 0.146738178
0.325433364 -0.156054447 0.087293384 0.324065006
0.331443899 0.396531628 -0.546122288 0.10666971 -
0.340907049 -0.087347053 0.457971374 0.101230041 -0.155521814
-0.364820654 0.195319797 0.453795369 -0.373786579 -
0.098848725 0.180830129 0.409424763 -0.17480125 -0.299542306
-0.00563977 0.257319287 0.308910585 0.250537904
0.183953254 0.421580092 -0.431426724 0.293452123
0.286870247 -0.557461365 0.188292921 0.088446805
0.019116535 -0.107773908 -0.16038489 -0.441006014 -
0.326350763 0.428230099 -0.52610948 -0.096362124 -0.121211195
-0.590796044 0.114497106 0.187741144 0.264034219
0.136784437 -0.342339547 -0.347958756 -0.358434653

0.076485375 -0.032491852 0.254977969 -0.083513023 -
0.463146488 -0.259874462 -0.338973935 0.376087258 -0.267112828
0.023808741 0.19107791 -0.052318938 0.173848126
0.413871863 0.035059608 -0.480133134 -0.56019951
0.05996939 0.397604644 0.226719511 -0.012192114 -
0.432062233 0.032469554 -0.070959203 0.349687108 0.442147176
0.389993788 0.226529939 0.281256852 0.223588001 -
0.366817335 -0.044047574 -0.097812309 -0.12428779 -0.303462823
-0.042821908 -0.17593267 -0.106890962 0.321873413
0.343602064 -0.154620096 0.386864437 -0.204148731
0.400666843 0.384984474 0.192359798 -0.176199961 -
0.276299769 -0.312398071 -0.22233861 0.11969897-0.299601924
0.008996101 0.410211770.165282213 -0.487557984 0.357617742
0.041191713 -0.275971166 -0.084501052 -0.091680167
0.306824541 0.453578598 -0.147903175 0.085608366
0.109335424 -0.252977634 -0.564902998 -0.344868272 -
0.125106766 0.221062664 -0.313209345 0.472833819 0.072691356
-0.309335901 -0.321175034 0.008048464 0.001095984
0.177174796 0.026187711 -0.528081805 -0.392932183
0.203454865 -0.327733082 0.024706109 -0.049071075 -
0.193224317 -0.174602922 -0.125685106 -0.441392441 0.012512352
0.325976913 0.319264939 -0.232518533 -0.348360157 -

0.433030842 0.295466733 0.3935879 0.352450876 -0.439597738
0.182407704 -0.217233844 -0.0216415 0.436693616 -0.025911642
-0.192978184 0.073614757 -0.231464091 0.138120191
0.229725095 0.353472719
KEGG_PATHOGENIC_ESCHERICHIA_COLI_INFECTION -0.117945767
0.126412271 -0.138791721 -0.217863895 -0.279417296 -
0.36457392 -0.238709072 -0.08400602 0.465138278 -0.171687016
-0.162099994 -0.184295834 0.071206417 -0.176691062 -
0.298967663 -0.366927264 0.272267308 0.205230241 -0.048372181
-0.051587456 0.216001679 0.45206763 0.162470383
0.304039311 0.438960714 -0.303670468 -0.450945278 -
0.091601761 -0.262036959 -0.229536043 -0.423571565 -0.108961781
-0.367590274 -0.192976954 0.459645808 0.150776964 -
0.038466208 -0.071584182 -0.37859177 -0.166027904 -0.229212079
0.139079109 0.078033408 0.1399007 0.234237379 0.145828358
-0.182466452 -0.418462541 -0.322020153 -0.260337689 -
0.299775984 0.098001569 0.471348429 -0.013675134 0.332909383
-0.021495392 -0.178789703 0.417155939 -0.148033845
0.388565124 0.233582252 0.158260271 0.171172522
0.136818838 -0.208235056 -0.327086211 0.221416349
0.277996507 0.093857151 -0.257891158 0.49431685
0.353255605 -0.4547954 0.017309361 -0.524161804 -0.060143751

-0.045681176 -0.370736752 0.057532472 -0.11197292 -
0.389150024 0.352603996 0.344980526 0.299222704 0.282901375
0.302421052 -0.049401196 -0.341750052 -0.068908091 -
0.347168535 0.322485531 0.020740406 0.030807415 0.263396722
0.033046098 -0.131251121 -0.224812997 0.154971523 -
0.049166501 0.227965396 0.233023772 -0.464892954 0.021639797
0.408859536 0.045600167 -0.390510831 -0.319349603 -
0.258606797 0.253173889 0.103632388 -0.201877504 -0.238671044
0.186721785 0.320960562 -0.277939309 0.324704498
0.017110703 -0.373086432 -0.486651026 -0.018919248 -
0.469008644 -0.470794498 0.106618213 -0.347988856 -0.264367464
-0.363602261 -0.309969484 0.122486792 0.112708054
0.234173586 0.167325957 -0.29754176 -0.357494883
0.095843508 0.145200195 0.369196273 0.328305986
0.133743792 0.166222902 0.284893388 -0.177130533 -
0.454300879 -0.294632478 -0.066373759 0.261844476 0.018923265
0.009952634 -0.103729311 -0.326280607 0.387109555
0.360937572 0.444334033 0.42170394 0.349319028 -
0.27880263 0.024601685 0.250591863 0.041057692 -0.268287982
0.019914374 0.455136718 0.248269982 -0.095627584 -
0.365864554 -0.082253974 0.147445888 0.436754441 0.226235179
-0.212324813 -0.260582708 -0.175001558 -0.456526833 -

0.311880859 0.095956484 0.330748378 0.345933803 -0.261927462
-0.405612442 0.387095907 0.467574802 0.109957058 -
0.251270623 -0.403065917 0.174942313 0.240278187 -0.173247716
-0.013481357 0.033008462 0.252030247 -0.056160177
0.328425085 0.08095644 -0.233701396 -0.520940933
0.099153149 -0.113450078 -0.372811082 0.208183305 -
0.530813818 -0.324552206 0.361669287 -0.127303402 0.113604538
-0.199159032 0.256811868 -0.426529716 -0.381811991 -
0.297065693 0.167768645 -0.052479774 -0.279011546 -0.250801684
-0.077976421 0.374117137 -0.050559868 0.083918291
0.147353654 0.135146185 -0.340481366 -0.086275505
0.291045499 -0.085701763 -0.14045006 0.446104254 -
0.151784452 0.210151983 0.343254453 -0.257354711 0.189601383
0.060929345 -0.215455278 0.252095974 -0.493730348 -
0.02397338 -0.491495009 0.211239637 0.031575439 -0.039628801
-0.082620008 0.114584281 0.012869065 -0.263397099 -
0.443125341 0.468959828 0.047753395 0.109009679 0.287363557
-0.115433056 -0.281690501 -0.50328256 -0.287317161
0.006679573 -0.03190707 0.282536922 0.019398705
0.295968119 0.068566194 -0.227414331 0.168776943 -
0.189176988 0.10380332 0.052597223 -0.308687993 -0.210469792
0.444641736 0.196106731 0.407932923 0.044929377

0.20726565 -0.246788843 -0.178023904 0.334630737
0.492609298 0.360712859 -0.057892731 0.144693237
0.148720396 -0.19005694 -0.403102192 0.04441106-0.222470625
0.393230145 0.205097128 -0.150240604 -0.007817441 -
0.185005863 -0.330380021 -0.109295543 0.434262044 -0.180562997
-0.091756489 0.272230606 -0.133126128 0.430671382 -
0.202098279 -0.113027807 -0.20280989 -0.337121346 0.177750585
-0.33729078 0.024984666 -0.340422393 -0.144916371 -
0.211413744 0.056624386 -0.16611009 0.470625165 0.004648759
0.357837908 -0.267822492 -0.315786921 -0.150677964
0.365163113 -0.044107009 -0.03571342 -0.222957153
0.026475239 -0.260388198 -0.023802421 0.245041958 -
0.310637563 -0.25053477 0.16708528 -0.220105563 0.111517196
0.295518888 -0.007298547 -0.459750972 0.207281915
0.016821029 0.040674658 -0.051331711 -0.073627276
0.200382416 0.198722881 -0.003107911 0.186652743
0.43375782 -0.110520845 0.234575697 0.305348281 -
0.066483984 -0.501402767 -0.063010525 0.044606299 -0.198059287
-0.434991274 0.091255394 -0.203234028 -0.283776704
0.088367206 0.454792826 0.202169235 -0.190693018 -
0.122345062 -0.397380459 -0.194062164 -0.01702949 -0.316652153
-0.267243485 -0.3862796-0.349661283 0.209225997 0.448141114

8766 0.372325235 0.079013178 -0.115204922 0.177952311 -
8767 0.019769972 0.066476523 0.165864608 0.308669453 0.131902832
8768 0.307893852 0.22643152 -0.269067343 -0.227053896 -
8769 0.280753267 0.280427514 0.116320578 -0.36088598 0.043562884
8770 -0.374298449 -0.234427458 0.334979101 0.039465019
8771 0.106496732 0.534387088 0.238872916 0.200305362 -
8772 0.050185792 0.256458475 -0.009905947 0.272944662 0.380261442
8773 0.059475055 0.270680689 -0.040410902 -0.058282632
8774 0.075994106 -0.415193679 0.252932437 0.360968279 -
8775 0.429246421 0.32991698 -0.368833821 -0.509997983 0.231845853
8776 0.315826841 -0.216049021 -0.096571665 -0.02708536 -
8777 0.300990478 0.04625094 -0.113140032 0.178720908 0.095147215
8778 -0.211808543 0.485463527 0.363581478 0.285018414 -
8779 0.040688199 -0.23831141 0.483242574 0.150339534 0.353608504
8780 -0.313235377 0.3941406 0.360705583 0.06479719 0.039469526
8781 0.302630543 0.209318825 -0.522355055 -0.217478452
8782 0.157851523 0.393447881 -0.111052651 0.00025197 -
8783 0.097739167 -0.34078082 0.094281154 -0.219027812 -0.050263129
8784 -0.149676025 -0.145007022 0.134765781 -0.144170919
8785 0.217318724 -0.290604141 0.403802493 -0.480213817 -
8786 0.189929756 0.23823041 0.215156586 -0.007233675 -0.09582959
8787 0.176801442 0.008379675 0.261008677 0.316927664 -

0.140410942 0.015994989 0.032296044 0.204339493 -0.299736036
-0.256645086 -0.008954203 -0.051110479 -0.117114381 -
0.260405294 0.20514841 0.408751109 0.062751285 -0.1868723 -
0.099979273 0.010007382 -0.139138551 -0.34643075 0.385175399
0.106245066 -0.159138055 -0.182178992 0.013659108
0.13609562 -0.15086309 0.314138844 0.254817566 -
0.39579414 0.196096184 -0.296003584 -0.302691355 0.319889102
-0.066612741 0.07690175 -0.438989426 -0.140836416
0.261485335 0.260136579 0.094709624 0.190310623 -
0.125389439 0.151209315 0.376972712 0.456941805 0.43307621
0.075065864 0.376412469 0.149235828 -0.443080056 -
0.203594103 -0.229438928 0.287682735 -0.295951023 -0.311569041
0.284885584 0.131212742 0.044887991 -0.037549996 -
0.374524993 -0.334994423 -0.469504832 0.303579487 -0.117708006
0.004113491 0.377527995 0.287758987 0.330097921
0.351603915 0.237274531 -0.420495926 -0.170414003 -
0.060907455 -0.283228536 -0.026506543 -0.289805869 -0.296634814
0.130969269 -0.117164835 0.043812086 -0.356849411
0.404219369 0.08292605 -0.059233242 0.130598606 -
0.312152997 0.285010217 0.243975984 -0.136377054 0.065119123
-0.018349356 0.073517632 0.463089111 0.34825675 -
0.014814377 -0.225232896 -0.094996117 0.197024523 -0.109506084

0.153127312 0.294656101 -0.358565727 -0.304436945 -
0.046138652 -0.00865 0.148313539 -0.064194159 0.123569537 -
0.39687324
KEGG_SYSTEMIC_LUPUS_ERYTHEMATOSUS -0.600909084
0.456688179 -5.81E-01 -0.309955715 0.166676776 0.07949214
0.028470669 0.303665156 0.552766139 0.514529142
0.169731719 -0.588427092 0.413842265 -0.64976048
0.487885912 -0.298732289 -0.194957849 0.539173898
0.273318287 0.525174192 -0.502609438 0.466764601
0.281663709 -0.602572996 0.576053802 -0.146549528 -
0.290134977 0.337554117 -0.120143335 -0.168871906 -0.592370749
0.492737946 0.101975644 0.524093091 0.506371827 -
0.284374405 -0.292942115 0.412616915 -0.5318854 0.007408437 -
0.440526318 0.377224693 -0.397788932 0.3334959 -0.0217016
0.223364356 -0.493834617 -0.40377615 -0.130182452
0.318474565 -0.424319244 -0.414205145 0.263775197 -
0.599587503 -0.284078276 -0.33531993 0.143947817 0.198028161
-0.148110704 -0.214644525 0.100546379 0.359770618 -
0.164820366 -0.482780849 -0.353662615 0.124878201 0.473225292
-0.45334986 0.381821931 0.481297187 0.539563373
0.560603012 -0.529437283 0.521135095 -0.463964147
0.433773641 0.402610044 -0.41704339 0.461175275 -

0.446679606 -0.395498377 0.210068809 0.620252413 0.341037118
0.507039775 0.354588712 -0.619211383 -0.278121959
0.564572385 -0.180934989 -0.165500412 -0.057137475 -
0.273149039 0.294300185 0.331333777 0.446212128 -0.410632935
0.623909548 -0.024970153 -0.214121232 0.498001425
0.160957379 0.010724534 0.641503417 0.233236495
0.083553263 -0.152063756 -0.357872808 -0.36014926
0.418919015 0.068805139 0.335549155 -0.13842086 -
0.487031994 -0.039737258 0.398705631 0.342478593 -0.05043196
-0.032030226 -0.429444409 -0.041621328 -0.207779291 -
0.182217767 -0.569826167 0.054407041 0.272610907 -0.546555602
0.153685565 -0.31221695 0.435494449 0.400109746
0.378437744 -0.580565879 0.239108706 0.373515904
0.087592227 -0.048049875 -0.433474354 0.145320223 -
0.311241996 -0.176547188 0.05075616 -0.468722862 0.046323305
0.457380741 -0.537632318 0.398682079 0.326945028
0.387669003 0.44154185 0.607233289 0.404604733
0.57302824 0.59408253 -0.245407527 -0.260505343
0.107193976 0.444997295 -0.433164716 0.573591965 -
0.274137964 0.37988277 0.070979025 0.118412387 -0.287081741
-0.220690675 0.148584405 0.392468827 0.506083478 -
0.036734442 -0.544544459 -0.472175456 -0.165565098 -0.39702867

0.169527662 -0.552504024 -0.14902661 -0.445656161 -
0.266432084 0.654693073 0.489322925 -0.491618623 0.390861095
0.24388733 -0.556045651 0.113268228 -0.567286199 -
0.155013916 0.598222937 0.115731824 0.206584404 0.208015464
-0.403597514 -0.458822262 0.278244784 -0.038669669
0.334210644 0.276179162 -0.557689131 -0.388813704
0.355992839 0.365921112 0.0202899 0.21474986 0.252985949
0.208805938 -0.190760613 -0.179107243 -0.551724196
0.217478698 0.099777254 -0.51167846 -0.379978633
0.156072518 0.436391028 -0.265552577 0.289342803
0.348133034 -0.008676298 0.370448267 0.634615317 -
0.34080496 -0.363187779 0.064977625 0.132110843 0.316466359
0.415031492 0.167225966 -0.490605349 0.482481554 -
0.0880858 0.576832448 0.043540258 0.452040582 -0.162698606 -
0.439078432 0.432295958 -0.311468201 0.148418122 0.27441568
-0.102813956 -0.157352149 -0.482726538 0.200058485 -
0.588453096 -0.296609415 0.004679229 -0.255623847 0.372663248
0.084821717 -0.478862944 0.093577832 0.261335423 -
0.122311095 0.244566142 0.242362328 -0.237769303 -0.067965284
-0.097318273 -0.496719065 0.156866106 0.5030135 -0.089325078
-0.312579971 0.537317341 -0.379612493 0.065637575 -
0.094290711 0.431838529 0.524144452 0.136612194 -0.375632427

0.354983818 0.377394033 -0.156694462 0.502167371 -
0.312708135 0.439201089 -0.633797386 0.403411725 0.271818834
0.528536017 0.33825659 -0.024631304 0.377248587
0.184523917 0.170065717 0.416911102 0.491161357 -
0.472794989 -0.52708488 0.499131664 -0.52697468 0.316129868
0.41077633 0.501154368 -0.428365104 0.258260086
0.337537893 0.599785661 0.309844395 -0.412158926
0.397348171 0.248669284 -0.081650485 -0.086895527
0.365539797 -0.432279732 0.27676188 0.365649524
0.152816728 0.254913144 0.539949367 -0.576679795
0.191349658 -0.48208046 -0.041497522 -0.541259197 -
0.424395521 0.075079699 -0.476718953 -0.414915909 -0.193652459
0.358249628 0.450950356 0.334402544 -0.377839071 -
0.344158524 -0.041985079 -0.406363621 -0.158006641 -0.585974212
0.271733912 0.34986467 -0.443779033 -0.181516268 -
0.242452364 0.237221746 0.30983982 0.199701206 0.496192003
-0.31749212 0.416595372 -0.55477454 -0.234076935
0.445672951 -0.31711458 -0.383710247 -0.458410105
0.017093543 -0.174281476 0.508131047 -0.451701308
0.159818088 -0.647561131 0.310203937 -0.204823841
0.298442986 -0.140804333 -0.134541115 -0.410080333 -
0.622137402 -0.401906329 0.509420754 0.502013967 -0.05568692

0.205427416 -0.424819446 0.118991167 -0.517839668
0.310445574 -0.449751706 -0.509817268 0.337978463 -
0.353201529 -0.366675574 -0.099401764 0.423065077 0.415266642
0.388661697 -0.447039384 0.377119721 0.003137729
0.424635319 -0.047707109 -0.345573094 0.050223471
0.618860614 0.249937319 -0.341210776 0.420768273
0.578011438 0.217214573 0.394055607 0.126617899 -
0.3278498 -0.347766902 -0.116813131 -0.581301952 -0.259604908
0.328428475 0.430641489 -0.325609201 -0.434214235
0.413028112 0.067021058 -0.313525064 -0.26229844
0.225623553 -0.238274293 -0.181344532 -0.412358949 -
0.008217123 0.246789734 0.209783368 0.376358579 0.344709829
-0.226753641 0.527305906 0.162715128 0.546428945 -
0.368519165 -0.584672203 0.286177895 0.444613625 -0.205375282
0.203001039 0.517110025 0.050702609 -0.54849835
0.131528635 0.070453271 -0.185363892 -0.258479301 -
0.444893231 0.380834438 0.112249829 -0.601040886 -0.397227486
-0.663608341 -0.319114476 -0.60192852 0.340888246
0.213987873 0.380235219 -0.031613855 0.339541278
0.46566353 -0.531433357 0.032983211 0.348960425 -
0.537473876 -0.600259656 0.395654363 0.525334899 -0.242884378
0.032225939 -0.402173482 -0.572239519 -0.229162267

0.250117527 0.509503877 0.49969018 -0.17064206 -
0.046193303 0.408216778 -0.08553584 0.49412848 -0.577899786
-0.281152832 -0.306514656 0.071171353 0.124766522 -
0.39027623 -0.469092602 -0.173380472 -0.174681873 -0.322631837
0.339198867 0.077883012 -0.450866619 0.160072747
0.16933576 0.223124837 0.501157067 -0.113533624 -
0.381112219 0.185274281 -0.19163212 -0.656522962 -0.283682547
-0.395611409 -0.28555825 0.319904689 -0.282515402 -
0.39668846 -0.206665132 0.597455516 -0.288678216 0.527957822
0.396709148 -0.540116264 0.134017586 -0.331113497 -
0.273543087 0.39844093 0.630214727 0.020753378 0.581617596
-0.23294708 0.210094572 -0.203178276 0.189398234 -
0.055207929 -0.543362697 0.18917508 0.043016955 -0.261902514
-0.165535843 -0.068361251 -0.403729711 -0.546255426
0.268747305 -0.268323205 -0.170060697 -0.041824197
0.286642527 0.2264561 -0.238596361 -0.429325058 -0.132298124
-0.459510062 0.404402366 -0.457288242 -0.323923891
0.043658735 -0.454242397 -0.330328835 0.158825701 -
0.15653151 -0.410570629 0.183904489 -0.076924124 0.410918177
0.2381286 0.094501722 -0.064790482 0.056889236 0.079236227
-0.322738316 0.087548981 -0.044347545 -0.298088419
0.476385738 0.040525068 -0.198668441 -0.236261764

0.314657805 -0.264712618 0.519286636 -0.230041957 -
0.216846898 -0.157544808 0.319876581 0.164030525 -0.134208364
0.012404986 0.216731763 0.129379496
KEGG_PRIMARY_IMMUNODEFICIENCY -0.547782929 0.372614819 -
5.70E-01 -0.035766066 0.071310046 0.054941834 -0.372667497
0.085807407 0.417016023 0.712807574 0.300890091 -
0.522608033 0.397704508 -0.658857593 0.411172430.08255938 -
0.546792252 0.500065175 0.394274456 0.585039547 -0.476013627
0.379556545 0.458108227 -0.570789965 0.607447456 -
0.415856443 -0.020942374 0.334686987 0.124062708 -0.09839462
-0.566893193 0.493623544 0.290620579 0.560573908
0.301368717 -0.405866073 -0.362077955 0.468938156 -
0.455788584 0.22431509 -0.433430801 0.276094248 -0.099166541
0.163087588 0.008793519 -0.457958487 -0.03505515 -
0.476197645 -0.40103564 0.421387545 -0.546991926 -0.050718378
-0.342585532 -0.211367513 -0.404739251 -0.485493088
0.17905181 -0.108912716 -0.11372256 -0.628063767 0.1190578
0.530286202 -0.46826388 -0.43850324 -0.175486362
0.26649216 -0.165365378 -0.615351553 0.260443307
0.579484365 0.554319055 0.656216478 -0.417297922
0.374081352 -0.409599667 0.423708694 0.544846965 -
0.368524133 0.477826747 -0.460977883 -0.364467888 0.30127629

0.459205937 -0.024163011 0.593809079 0.540599173 -
0.584802592 -0.214144572 0.635648754 0.086857752 -0.324209154
-0.251111854 -0.170870183 0.167338032 0.521493263
0.671114776 -0.336377162 0.632169549 -0.31751307 -
0.487041483 0.607481127 0.335572772 0.023643622 0.457178171
0.5320595 0.377066642 -0.426227359 -0.172800449 -0.339059817
0.494261556 0.318018306 0.31418049 -0.488933343 -
0.555918423 -0.050855975 0.40307965 0.398096409 0.324520523
0.23044672 -0.335845115 -0.010668619 -0.097947854 -
0.512867936 -0.527098894 0.55976328 0.052210741 -0.475681899
0.186777289 -0.509697281 0.59274906 0.430640921
0.588989164 -0.25635848 0.461403253 0.227066436
0.191398899 0.353176623 -0.637796711 -0.225480214 -
0.330800476 -0.108385761 0.123602856 -0.585401944 0.068891433
0.046548518 -0.660660739 0.262814747 -0.040869111
0.180392407 -0.377162679 0.562126258 0.402815254
0.635245191 0.512248147 0.31747488 -0.156530016 -
0.018256466 0.146991553 -0.080056233 0.73086433 -0.399309791
-0.030482339 0.411245550.205975822 -0.428411055 -0.440287969
0.27916126 -0.304008482 0.517564264 0.00697159 -
0.561723155 -0.476094746 0.309923704 -0.523172374 0.222094046
-0.658831856 0.225850246 -0.409783245 -0.203905457

8986 0.625499555 0.227824177 -0.441205574 0.44054729 -

8987 0.354836821 0.032065538 -0.259340932 -0.616871558 -0.052628456

8988 0.699797743 -0.494858043 -0.29464961 0.02923753 -

8989 0.524035883 -0.367700756 0.161077293 -0.411587679 0.447998654

8990 -0.106067363 -0.30649406 -0.346193138 -0.161744797

8991 0.320377515 -0.172960545 -0.405270429 0.511981330.47255577

8992 0.18232933 -0.196548257 -0.550541454 -0.002627951

8993 0.113707281 0.0687221 -0.373639742 0.031076302 0.13923954

8994 -0.051464991 -0.11852156 0.545225194 -0.021308061

8995 0.490515647 0.611310133 -0.227311045 -0.370048636

8996 0.297781223 0.213703748 -0.319073742 0.350574921

8997 0.368805688 -0.57305489 0.295274278 -0.081703652

8998 0.537689189 0.381388951 0.586957228 0.26472698 -

8999 0.381847206 0.696703248 -0.417267574 0.520705565 0.35845912

9000 0.200905501 -0.203731837 -0.212748453 -0.113730575 -

9001 0.479651489 -0.05907744 -0.073098264 0.044127339 0.496712733

9002 0.380587026 -0.364309079 -0.165645437 0.267560469

9003 0.103998978 0.033424897 0.021335254 -0.420534172

9004 0.205505889 -0.231482289 -0.132249537 0.474341299

9005 0.543717405 0.459697264 0.069122068 0.019763405 -

9006 0.426576949 -0.48045846 -0.269156414 0.521512675 0.550509299

9007 0.033619453 -0.203273005 0.321823228 0.183256 -0.390113279

9008 0.565326317 -0.216768742 0.509809005 -0.432087716

9009 0.48849079 0.427276855 0.454499545 0.462266988 -

9010 0.040445605 0.569134226 0.030348189 0.575382083 0.502385859

9011 0.296159278 -0.251777035 -0.504384671 0.532884466 -

9012 0.595514853 0.144351605 0.382024145 0.493031653 -0.306051781

9013 0.397167071 0.272474468 0.604774855 0.238542528 -

9014 0.188071475 0.288883432 -0.235419247 -0.513728841 -0.300625652

9015 0.296099213 -0.373240627 0.171992098 0.581065384

9016 0.041301023 0.29195689 0.677216977 -0.496675305 -

9017 0.005840544 -0.346530452 -0.141086267 -0.3928408 -0.165775453 -

9018 0.21711851-0.518885027 -0.594802485 -0.423339741 0.26960843

9019 0.181056341 0.026764353 -0.398276859 -0.15116031 -

9020 0.022721676 -0.411723343 0.195641905 -0.588402902 -0.252799492

9021 0.03124711-0.464194845 -0.192586492 -0.520786916 0.293639324

9022 0.54402747 0.208816937 0.469959352 -0.524107756

9023 0.577512099 -0.4040535-0.122960428 0.610647383 -0.019922167

9024 -0.463590292 0.26387779 -0.335913336 -0.126946392

9025 0.359279248 -0.443649148 0.51400277 -0.704746271

9026 0.214149974 0.165385762 0.300753037 -0.459685928 -

9027 0.275054416 0.06637605 -0.450595132 -0.317811504 0.596599821

9028 0.240520872 0.33481626 0.411957104 -0.391612085

9029 0.12487067 -0.36243679 0.545104677 -0.526771373 -

0.60875158 0.051005899 -0.23020132 -0.048188454 -0.34571571
0.014083164 0.381665271 0.583264932 -0.432145802
0.201219085 -0.038675097 0.463979207 -0.450017468 -
0.260275239 -0.041896254 0.488702123 0.062535346 -0.512400077
0.469593075 0.4442811 -0.367315336 0.112161204 -0.32390885
-0.209312747 -0.493639049 -0.414684974 -0.370415459 -
0.297200627 0.441097882 -0.002426643 -0.25224013 -0.482208358
-0.109432359 0.296318516 -0.413958485 -0.017362153 -
0.107801724 -0.333164541 -0.470580331 -0.119718414 0.184088909
0.363952635 -0.015703614 -0.17072156 0.171503195 -
0.469284666 0.36004974 -0.496997858 0.70897945 -0.473151167
-0.502813907 -0.391399372 0.474696159 -0.461961505
0.034496144 0.564477407 0.381374546 -0.662433633
0.178838027 0.127784335 -0.121062823 0.140472319 -
0.463331155 0.326912005 -0.002157414 -0.573740309 -0.464472946
-0.622737877 0.16919933 -0.632376582 0.481904575 -
0.030661108 0.197981191 0.242584638 -0.130110588 0.566919177
-0.471574927 0.24957317 0.215734775 -0.471433072 -
0.632261098 -0.140497728 0.607795333 -0.462818756 -0.330708573
-0.56795576 -0.351762003 -0.556266676 0.262137103
0.665493708 0.593959234 -0.031049826 0.020429596
0.440464795 -0.214121697 0.352336822 -0.579947366 -

0.303704536 0.0041876 -0.246235715 -0.560772104 -0.112417199 -
0.466179911 -0.159074516 -0.032416286 -0.450624235 0.611990742
0.012107164 -0.362832807 0.254396669 0.2048132 -0.019788465
0.371869963 -0.259208844 -0.434760582 -0.09014237 -
0.157162828 -0.634323714 -0.400471818 -0.160678406 -0.082788837
0.565433536 -0.378954089 0.017046278 -0.405334518
0.475556546 -0.44943994 0.668516612 0.243844575 -
0.432697976 -0.227355891 -0.345831208 0.032549764 -0.191558039
0.532785207 0.067414681 0.439331223 -0.157202215
0.38217288 -0.322211624 0.461910026 -0.206612276 -
0.493696738 0.303051095 0.010037959 -0.557039774 0.472347798
0.36519908 -0.321427296 -0.189076029 0.006778305 -
0.244644183 -0.476641708 -0.394593349 0.128336247 -0.164128406
-0.317414103 -0.041031524 0.105806745 -0.578585865
0.315445696 0.095104525 -0.493483097 -0.03002178 -
0.213297503 -0.477571104 0.001867803 0.253139844 -0.093884866
0.160058529 0.1401461 0.327243134 0.289614131 0.401453268
0.136633166 0.048511743 0.182328405 -0.485909547
0.321854902 0.202298345 -0.269953253 0.212460044 -
0.069904184 0.174529933 -0.527240562 -0.0015213 0.096301944
0.312563605 -0.3529661 -0.153064031 -0.068212103 0.384323495
0.141058544 -0.412912038 0.081881605 0.271784363

0.271803118
KEGG_HYPERTROPHIC_CARDIOMYOPATHY_HCM -0.37927317 -
0.18458921 0.031090183 0.075805844 -0.169396037 -0.094763758
-0.023556849 -0.07080461 0.043519201 0.087286011
0.107434192 -0.318476747 0.343972891 0.033575431
0.038836015 -0.099298143 -0.020125148 0.011276405
0.366448055 -0.387263973 0.074769706 -0.119799006
0.153625802 -0.074724231 0.21398358 0.019576425 -
0.22089681 -0.219036704 0.153779556 0.182874411 -0.160045317
-0.208949408 -0.274743509 0.016298984 0.348566026
0.137575141 0.367072866 -0.079104905 0.198923313 -
0.311739072 -0.07952062 -0.01252223 -0.038250916 -0.261618429
-0.100254931 0.257379919 -0.225770845 0.014098741 -
0.035378773 -0.048467503 -0.073345262 0.206248187 -0.085568145
0.221727492 0.131130735 0.004788356 0.296329166
0.053090613 -0.314479152 0.182721266 -0.012887674
0.401574489 -0.150960466 -0.161632498 -0.004710129
0.143961188 -0.217873597 -0.022308494 0.291747989 -
0.158672751 -0.180543963 0.373775388 0.063655113 0.120540268
-0.051574315 0.194321142 0.02695441 -0.136175061 -
0.123770163 -0.322313938 -0.104799547 0.231059111 0.063382375
0.290815786 -0.179542642 0.002147373 0.137429618

9096 0.277194841 0.162562066 -0.261220065 0.028901901

9097 0.297357897 -0.255176617 -0.255520663 -0.07567465

9098 0.126243852 -0.008505991 0.154196182 -0.219312085 -

9099 0.148303153 0.078270911 0.266801197 -0.241170153 0.160408864

9100 0.351226673 -0.224201845 0.013561439 -0.452580745

9101 0.266424796 0.16392017 0.308834081 -0.281266924 -

9102 0.25189889 0.24327575 -0.084734062 -0.27539162 -0.289247186

9103 0.025634332 0.209249264 -0.366101594 0.079457859 -

9104 0.337091784 -0.265402346 0.161323037 0.09938742 0.11092383-

9105 0.110193038 0.13936865 -0.246908649 -0.280080366 -0.037498391

9106 0.240170888 -0.223012235 0.082724418 -0.222294433

9107 0.389244338 0.24087093 -0.196109013 -0.128898393

9108 0.098497865 0.077021063 -0.131227049 -0.178199598

9109 0.005667461 0.062865983 0.30083723 -0.152919489 -

9110 0.183255847 0.283393103 0.077663471 0.239347429 0.118532527

9111 0.358060183 0.378493802 -0.224884804 0.256376885

9112 0.298405351 -0.280916785 -0.244778508 0.061177109

9113 0.300322057 0.010184528 -0.044094278 0.17209331 -

9114 0.220079949 0.177811443 0.118210704 0.238316062 0.208746087

9115 0.122862934 -0.146370699 -0.058165033 -0.181012316 -

9116 0.048735713 0.239168135 0.351680145 0.129385378 -0.317774618

9117 -0.027245646 0.01474329 0.0568167 0.002234564 -0.177596265

-0.019843287 0.033850806 -0.072334902 -0.061572613 -
0.061619246 0.382518205 -0.011326248 -0.181095693 -0.078322523
-0.019419877 0.0081319 -0.374950058 0.126879305 0.051865312
-0.136391083 0.060067514 -0.0708283 0.136982345 -0.245842846
0.26181728 0.029902678 0.016716142 -0.252075161
0.189669052 -0.288797789 0.167511138 -0.358835008 -
0.403499244 -0.217773078 0.040607026 0.272147669 0.139626926
-0.227441811 -0.104044089 -0.129881824 -0.06429487 -
0.106193125 0.325471483 -0.216098216 0.03113895-0.242214849
0.257494436 0.201541608 0.235802493 0.073240886
0.019252738 0.30412435 -0.290256413 -0.216682537 -
0.295007639 0.22253598 -0.094331829 0.230031947 0.18200719
-0.049649158 -0.060917568 -0.196149709 0.063674965 -
0.225108087 -0.209368512 0.198963892 -0.170414849 0.066562129
-0.070362141 -0.06609524 -0.133988589 -0.043591764
0.19598987 -0.054529679 0.255853282 0.295308167
0.058488258 0.341965182 0.090894427 0.199872824 -
0.108663256 0.124671449 -0.05710126 -0.358599358 -0.217482446
0.288259787 -0.01744109 0.250368685 0.345453279
0.103830377 -0.404421657 0.073198099 -0.332349791
0.060992309 -0.085432142 0.228779225 -0.318400954 -
0.325230695 -0.226772804 -0.177103084 -0.0103801 0.137173557

9140 0.439780294 0.266505396 -0.093115425 0.228376125

9141 0.323468236 -0.290022565 0.144317546 -0.211141193

9142 0.33335857 0.229798917 -0.149890564 0.078029879 -

9143 0.056935936 0.251211212 0.348346392 0.047218196 -0.30174387

9144 -0.047740226 -0.107503784 -0.221340523 -0.128010401 -

9145 0.289697656 0.029468641 0.1749912 -0.273709025 0.277665097

9146 0.317192334 -0.141433722 0.391856345 -0.058769439

9147 0.209671412 -0.229999294 0.397836081 -0.034828424 -

9148 0.289696362 -0.283398413 0.309893926 -0.141841724 -0.177197728

9149 -0.25244495 0.221899964 -0.200159016 -0.031718613

9150 0.353763917 -0.231021833 0.321442874 -0.013520664 -

9151 0.108008475 -0.226325463 0.228516909 0.228005887 -0.163776677

9152 0.274049122 0.127455847 0.143216204 -0.109399127 -

9153 0.1748253 0.113932594 -0.146997726 -0.146285201 -0.320689074 -

9154 0.114433291 0.02580073 0.107736286 -0.231285988 -0.016090523

9155 0.08545239 -0.044351951 0.186767015 -0.044843019

9156 0.337499081 -0.099907091 0.084696853 -0.328670132 -

9157 0.303402245 0.021314088 0.397658558 -0.148722865 -0.403345697

9158 -0.022796641 -0.262990595 0.102965826 -0.219314125

9159 0.315693419 -0.095779598 0.210575816 -0.324760706 -

9160 0.020497865 -0.212349332 -0.002906308 0.272411052 0.041166646

9161 0.047556457 0.15524949 -0.31320064 -0.097329981

0.242850114 -0.261300237 0.206674612 -0.155251808 -
0.041252477 0.173706162 -0.028596379 -0.139377415 0.031670728
-0.263819074 0.006309889 0.394403762 -0.070068075
0.053562975 -0.386884799 -0.09515739 0.276236953
0.063047272 0.306558156 0.162572947 -0.034115892 -
0.176235041 0.277732543 -0.002851095 -0.091532475 0.156599863
0.101281995 -0.139976909 0.073408327 -0.063093908
0.13638895 -0.044190123 0.079396259 -0.185039063 -
0.373556038 0.101524776 -0.206337344 0.228188505 0.270987863
-0.135347921 -0.004194368 -0.295706318 0.381158472 -
0.044742189 -0.079983194 0.017488211 -0.335770166 0.122510245
0.218183535 -0.09360597 -0.131795177 0.237533543
0.151114798 -0.110600992 -0.105069747 -0.178188647
0.305566566 0.029645648 -0.084249966 0.299332913 -
0.079522847 -0.113905488 -0.180206159 0.05461881 -0.048219877
0.382638274 -0.283638341 0.368801863 -0.136384851 -
0.222355435 0.317008237 0.19359283 0.274141172 0.211386063
0.052775316 0.11871472-0.215956858 0.108763469 -0.336372549
0.135094481 0.285168614 0.349205563 0.199289123 -
0.06174847 -0.128609423 -0.006556668 0.131333348 0.370291398
-0.036941659 -0.127661293 -0.336011418 0.313670059 -
0.033206626 -0.063266594 -0.167540023 -0.001692548 0.120037884

0.235383651 0.009722222 -0.197576076 0.049568176 -
0.044277882 -0.39376084 0.127904403 -0.141999803 -0.092299662
-0.305197804 0.176090903 -0.313749276 -0.270114093 -
0.093967331 -0.378302032 -0.169931173 0.018141197 -0.002386214
0.250506777 0.27090589 -0.065551031 0.141468459 -
0.07136237 0.095708741 -0.014999426 -0.120601886 0.05177455
-0.053143815 -0.133609489 -0.197290145 0.301326919
0.41751478 0.252759876 0.021809781 0.198475468
0.241533432 -0.183878504 0.052494064 -0.187101661
0.356987804 0.287794573 -0.076681986 -0.135514761 -
0.054992567 -0.117924039 -0.259356988 -0.113040863 -0.364401874
0.089699673 0.074260395 -0.180444834 -0.253905326
0.057561967 -0.10004347 -0.254679835 0.209357028
0.108223862 0.125715203 -0.223462296 -0.166473222 -
0.126148441 -0.026355228 -0.129904655 0.20248583 -0.048237717
0.24250137 0.037547805 -0.318758439 0.101180096
0.15908277 0.026263441 -0.151284363 -0.210980919 -
0.057426307 0.11585119-0.045988879 -0.199084417 0.111340782
0.082766451 0.258190218 0.154851476 -0.041281027 -
0.077915748 -0.212474025 0.211515363 -0.156193961 0.176206018
0.170369824 0.212993157 0.005861095 0.075657154
0.114337993 -0.090898555 0.112595366 0.196624244 -

0.256079098
KEGG_ARRHYTHMOGENIC_RIGHT_VENTRICULAR_CARDIOMYOPATHY
_ARVC -0.328513713 -0.21778552 0.046663057 0.162798078 -
0.227766765 0.000934653 -0.037444958 -0.222200312 -0.065588872
-0.152731083 0.085392506 -0.197551833 0.405001791
0.138640608 -0.055480243 -0.278195353 0.131409858
0.077040531 0.387744 -0.471316597 0.234673571 -0.026898468
0.180629896 0.066719317 0.112565141 -0.008585969 -
0.350510822 -0.073931773 0.011204226 0.25327785 -0.086945393
-0.248415722 -0.286471609 -0.112323705 0.318119635
0.281187809 0.41871356 -0.085893113 0.150583791 -
0.315564264 -0.024492909 0.126307872 0.077636916 -0.161482653
-0.176761762 0.314502971 -0.094516934 0.012910802
0.11760093-0.017516202 -0.067825967 0.167938599 -0.01943679
0.118255378 0.200865852 0.16619621 0.297613126
0.173291007 -0.125529392 0.32652794 0.044842311
0.299807378 -0.155993821 -0.151267863 -0.054415901
0.130313508 -0.138887162 0.081136633 0.310101818 -
0.202580171 -0.232219585 0.207665991 0.116125118 -0.011003087
-0.124107322 0.060124659 0.054524173 -0.212161493 -
0.193183601 -0.320464271 -0.276515717 0.387793108 -0.14313732
0.331514095 -0.128074603 -0.066460545 0.175483135

0.197038213 -0.008092752 -0.24897757 0.033356533
0.329895903 -0.121744345 -0.230180815 -0.28325581
0.003155228 0.063015137 0.002118793 -0.249811435 -
0.112480974 0.000473351 0.120595142 -0.277607229 0.188936565
0.305543068 -0.241239063 0.124915543 -0.426082921
0.337119173 0.098991027 0.379557751 -0.275495343 -
0.249562064 0.332243113 -0.129853277 -0.250146341 -0.332791983
-0.11976406 0.037366887 -0.409446215 0.019653934 -
0.250739427 -0.354911874 0.093696681 0.187416729 0.085476115
-0.246042124 0.189131704 -0.143000662 -0.219055749 -
0.230226347 -0.032866394 -0.275814382 -0.13621534 -0.260785454
0.361651688 0.296759641 -0.098088316 -0.035728966
0.150400155 -0.007625533 -0.241786964 -0.218336477 -
0.023099969 0.01469236 0.319693371 -0.074284148 -0.29966206
0.288357408 0.047207751 0.097034313 0.242839882
0.332543478 0.441248218 -0.299286368 0.261410227
0.232148524 -0.328121939 -0.324836219 0.037202911
0.360524047 -0.020774825 -0.057101562 0.106958489 -
0.019956696 0.261819051 0.241188651 0.218878175 0.194539903
0.236215804 0.000814467 -0.189286263 -0.172851582
0.056869347 0.29302821 0.404494256 0.093333013 -
0.309325925 -0.011941875 0.054613743 -0.119164624 0.090316878

-0.368021229 0.115402088 0.172271707 -0.076956063
0.184784364 -0.02874439 0.350396484 0.038928363 -
0.234305208 -0.180395985 -0.026394155 0.017245662 -0.442960014
0.099504906 -0.037723657 -0.165983566 -0.038102959 -
0.099850914 0.126894612 -0.170802628 0.276610093 -0.005743682
0.084738924 -0.228514043 0.035584778 -0.308392372
0.331668842 -0.410807766 -0.314499001 -0.21082924
0.107611836 0.215101817 0.095141533 -0.337100241 -
0.286244459 -0.009726685 0.021926525 -0.127631916 0.248029956
0.052008562 0.171005181 -0.249073625 0.150960378
0.227699827 0.193041836 -0.02895873 0.168436017
0.245721167 -0.353940807 -0.189811951 -0.303626824
0.276414314 -0.075494908 0.314323277 0.099182736 -
0.056805976 -0.103728204 -0.289219929 0.039155952 -0.1149154 -
0.066152467 0.311641434 -0.180321077 0.257571357 -0.133964327
-0.017002972 -0.318730304 -0.176619727 0.167498965
0.236461826 0.219698706 0.367374902 0.131146114
0.414280907 0.131716152 0.129422407 -0.085491329
0.05602028 -0.186705149 -0.405096152 -0.164206445
0.32803084 -0.11724455 0.176836137 0.37025503
0.170927306 -0.38846992 -0.001131094 -0.499377371 -
0.048444518 -0.065718535 0.103610767 -0.365298144 -0.27429548

0.026582668 -0.156505767 0.017924196 0.064397796
0.282943085 0.14665533 -0.090924349 0.27923326
0.235802448 -0.305403219 0.020232401 -0.379343577
0.366308461 0.273356346 -0.166475704 0.1424208 -0.110258109
0.340395152 0.297114446 -0.122243519 -0.253177632 -
0.156551306 -0.049369662 -0.339264107 -0.093595858 -0.311618812
-0.072080349 0.257885762 -0.251697108 0.317754256
0.290326946 -0.065088774 0.351001584 -0.093896477
0.345882717 -0.250763465 0.344269583 0.211631895 -
0.039104693 -0.266991777 0.318781658 -0.225529049 0.035025785
-0.057653347 0.183392217 -0.024056649 0.137126576
0.342123129 -0.305701487 0.243294354 -0.133673989 -
0.112221877 -0.105501486 0.342718848 0.227235838 0.00938837
0.216837178 0.067537315 0.363475148 0.054917244 -
0.02416379 0.223676031 -0.324475027 -0.001325078 -0.360602134
-0.111746628 -0.150744106 0.127890721 -0.170011923 -
0.108892548 0.046394775 -0.02451857 0.233557763 -0.100884282
0.414716265 -0.036320819 0.201787461 -0.422862429 -
0.031480914 -0.016093673 0.205780459 -0.118757373 -0.417590662
-0.117761909 -0.310052092 0.159824301 -0.18697846
0.441822899 -0.05020421 0.180269271 -0.3205291 0.217313355
-0.188717646 0.127004158 0.110516336 0.263958159

0.24897497 0.139114716 -0.295519608 -0.104305973
0.094840662 -0.085171482 0.168538277 -0.140530511 -
0.046501119 -0.077428892 0.046067711 -0.211656796 0.265114139
-0.172984166 0.037391627 0.429128274 0.005608526
0.16203034 -0.353863509 -0.142547207 0.206253509
0.063580284 0.38882039 0.213207092 -0.05992155 -
0.036971055 0.366991714 0.232140267 -0.207676431 0.307696183
0.197246615 -0.290422499 0.032619995 -0.336031724
0.029178366 0.121397113 0.020514065 -0.068966394 -
0.396505594 0.221242223 -0.231857725 0.19700359 0.233702039
-0.024014064 0.022244288 -0.330845087 0.39949291 -
0.124075378 -0.175921264 -0.004796807 -0.321011343 0.260148341
0.329070089 -0.067288173 -0.074927199 0.310183131
0.05782911-0.070549506 -0.269481612 -0.089861682 0.318197399
-0.06751975 -0.207844887 0.32858821 0.109550685 -
0.037073891 -0.280913924 0.202014968 -0.048171573 0.33881987
-0.334007677 0.377828953 -0.006013209 -0.296447368
0.311760247 0.054960425 0.257493857 0.1894794 -0.100506507
-0.007940196 -0.244611929 0.152182156 -0.37089419
0.071545205 0.261178204 0.370729438 0.224927614 -
0.131809153 -0.003519202 -0.12731163 -0.023096978 0.435508975
0.025678534 -0.116379641 -0.282786389 0.269371587 0.1926646

0.042435849 -0.221199504 -0.2135016 0.014083893 0.384757728
-0.009121298 -0.167743725 0.154647476 -0.105309411 -
0.256839979 0.160243461 0.097880013 -0.151086072 -0.273342001
0.117730453 -0.31167201 -0.281515753 -0.006881155 -
0.360365429 -0.106323484 0.225757398 -0.158877304 0.321843323
0.298911933 -0.078452861 0.050485832 -0.096072445 -
0.004165782 0.144733105 -0.261383884 -0.155356843 0.073994141
-0.055147352 -0.042090129 0.387225905 0.451418747
0.319989891 0.09057849 0.149470117 0.273993864 -
0.132466358 0.063246943 -0.255316296 0.398682949 0.286553779
-0.157357386 -0.114108242 0.140585506 -0.13364165 -
0.331287072 -0.313228678 -0.378081629 0.062958946 0.316555014
-0.265593618 -0.270316888 0.029491455 0.08593262 -
0.294901861 0.364469347 0.29227705 0.016965095 -0.152591562
-0.232894066 -0.268954984 0.066623163 -0.130252608
0.158032614 0.10111036 0.232346946 0.137708028 -0.282627504
0.026763972 0.129443064 -0.004315326 -0.132916353 -
0.220372947 0.014507041 0.002280364 0.180745126 -0.231322398
-0.015922288 0.110766743 0.251963214 0.096391554
0.002569562 -0.063382063 -0.182640267 0.116337979 -
0.198263924 0.135644467 0.126154216 0.062427076 -0.018305233
-0.002335121 -0.062343611 0.160853586 0.227625538

0.103567858 -0.164369643

KEGG_DILATED_CARDIOMYOPATHY -0.411079934 -0.218785822 - 0.005266508 0.179006053 -0.227217383 -0.067136717 -0.117097766 -0.071018929 -0.00108231 0.018265403 0.067769275 - 0.362837783 0.353476633 0.061284286 0.134300155 -0.095756416 -0.087895378 0.047764912 0.394764634 -0.369523155 0.177369289 -0.148898968 0.260109708 -0.033720548 0.203417813 0.058568841 -0.298493288 -0.235759066 0.094981488 0.223958768 -0.200651726 -0.127305922 - 0.361737613 0.051017834 0.389299523 0.093932904 0.390220336 -0.033689547 0.286628076 -0.285511103 -0.13296572 - 0.016326187 -0.030668834 -0.269323255 -0.165666477 0.219520803 -0.188983191 -0.022431933 -0.032571665 0.002099426 - 0.154416429 0.164748074 -0.037199734 0.267354516 0.109075168 0.009074639 0.324744997 0.187096322 -0.287511135 0.290713442 0.023353462 0.442699601 -0.210776458 - 0.078717138 0.026084107 0.183524991 -0.185119145 0.00987729 0.281152979 -0.120051731 -0.17508256 0.395397129 0.116501538 -0.012845297 -0.072552794 0.09581881 0.043160784 -0.26571838 -0.177903197 -0.332657041 - 0.085174068 0.196723133 0.016760834 0.283786301 -0.223004695 -0.002375997 0.182294196 0.310367839 0.236253491 -

0.365930286 0.056133598 0.306876443 -0.245966535 -0.240820533
-0.042286077 0.166523532 0.082664062 0.195093069 -
0.219249339 -0.188374866 0.138823016 0.276416045 -0.308657395
0.07315799 0.337197575 -0.191750348 -0.047411324 -
0.450712834 0.33386307 0.231595249 0.405861163 -0.246558225
-0.276823925 0.290992984 -0.14628587 -0.229764707 -
0.302738495 0.03315481 0.155181321 -0.326278125 0.101402216
-0.321222935 -0.221378692 0.117364392 0.127944444
0.23292935 -0.174057149 0.152264002 -0.190281394 -
0.237761905 -0.017887279 0.262678013 -0.293154933 -0.053851927
-0.222317632 0.439731398 0.297321639 -0.108178485 -
0.107744673 0.16572256 0.151176318 -0.196000908 -0.213251988
-0.059463114 -0.07061372 0.297168602 -0.151279724 -
0.157498715 0.275758919 0.125713153 0.30970585 0.100676814
0.390272219 0.419813045 -0.281845713 0.267425123
0.283245859 -0.310204314 -0.214290262 0.128094821
0.260901655 -0.057136215 -0.157591272 0.16448745 -
0.263536469 0.161119769 0.136439779 0.284874036 0.24215674
0.206070415 -0.204065442 -0.100365552 -0.066097483
0.01880239 0.3106953 0.410877155 0.108170799 -0.321513727
-0.022804162 0.006529233 0.104964166 0.053971555 -
0.158885887 -0.071456602 0.078625948 -0.053804898 -0.029073989

-0.152885654 0.426301813 -0.009080707 -0.267596496 -
0.163208179 -0.057931991 0.045888185 -0.436606104 0.172340463
-0.058238669 -0.150007095 0.053185978 -0.085711071
0.234494161 -0.269132748 0.300484326 -0.053806825 -
0.05603717 -0.254621532 0.143680599 -0.31285333 0.182078253
-0.446909403 -0.404971699 -0.299036229 0.034986749
0.278847057 0.170012113 -0.161679983 -0.278376997 -
0.131133238 -0.16138717 -0.027469262 0.349781983 -0.182734567
0.001670951 -0.239912885 0.258970064 0.162227983
0.157355762 0.044103469 -0.025406561 0.315972357 -
0.360022345 -0.215012671 -0.319680976 0.20107332 -0.131689617
0.270181835 0.201741426 -0.022080376 -0.090506711 -
0.150695514 0.150680242 -0.217291002 -0.302426788 0.227007784
-0.188432374 0.081871813 -0.157518143 0.070003189 -
0.095470801 -0.005846831 0.240663698 -0.048045676 0.281924447
0.398855772 0.059035183 0.38333665 0.077612315
0.280303383 -0.214564823 0.145892941 -0.163576034 -
0.333933083 -0.259915169 0.317014022 0.06601327 0.274056259
0.380641921 0.087915744 -0.349526047 0.147927224 -
0.433172951 0.031662239 -0.030253413 0.146562988 -0.28141743
-0.267186633 -0.23470011 -0.211546182 -0.003161867
0.163906697 0.459611773 0.200510373 -0.145989637

0.32380557 0.379568653 -0.231509577 0.176411873 -
0.210618685 0.310569079 0.298802609 -0.192848092 0.239713011
-0.008056453 0.322969623 0.315284735 0.069422537 -
0.151839256 0.081142937 -0.100963731 -0.21065987 -0.218117334
-0.244349117 0.075830822 0.148852288 -0.196882245
0.253303783 0.255910374 -0.106307253 0.434306452
0.015113898 0.178655952 -0.214832973 0.409983235 -
0.042296356 -0.294552073 -0.304323055 0.343148921 -0.13998634
-0.122195393 -0.218070483 0.161730362 -0.156489161 -
0.024750753 0.403459554 -0.310522412 0.343979785 -0.069322963
-0.107332231 -0.106148697 0.156154162 0.167122384 -
0.10893202 0.257115497 0.135742293 0.190203495 -0.090954656
-0.129889531 0.114425544 -0.122341245 -0.112308801 -
0.310875577 -0.184286769 0.029041959 0.142259218 -0.260316728
-0.1188889 0.073550348 -0.109833567 0.266846978 -0.1005731
0.373208897 -0.117528211 0.121605212 -0.397978618 -
0.265220469 -0.029526551 0.340950287 -0.198977759 -0.379780063
-0.100340976 -0.280808887 0.13537407 -0.260185718
0.343229825 -0.24541664 0.278092606 -0.31695793
0.057012324 -0.234780511 0.047460047 0.284431999
0.174981595 0.040170227 0.208409412 -0.297032719 -
0.110283781 0.22447863 -0.222324207 0.24026534 -0.061327589

-0.029998277 0.132752129 0.017277594 -0.039837803
0.119175529 -0.244283678 -0.100900205 0.365463732
0.001701334 0.015741016 -0.319114943 -0.153933925
0.235306757 0.113562158 0.347947626 0.263952241 -
0.006015288 -0.12155511 0.337527486 0.011701281 -0.090859869
0.231234987 0.059033286 -0.192305375 0.09331214 -
0.158109427 0.100377246 -0.065221438 0.020732028 -0.232537716
-0.431853215 0.140826886 -0.176971669 0.284536387
0.319939027 -0.086515406 -0.016182184 -0.338604574
0.392154426 -0.04385502 -0.057112192 -0.104351594 -
0.335502123 0.205572657 0.295407616 -0.039214488 -0.18044022
0.261314598 0.10385398 -0.162278024 -0.101345315 -
0.134308804 0.302586548 0.074342892 -0.022807755 0.285216229
0.011926137 -0.122263764 -0.198308403 0.13419553 -
0.002950319 0.366396528 -0.345065377 0.418728437 -0.066334023
-0.151057679 0.318985466 0.277476676 0.210453222
0.150348974 -0.027753769 0.121254892 -0.235363173
0.146062222 -0.371464496 0.196005967 0.366962114
0.370895205 0.185633835 -0.140401828 -0.13765714
0.04139479 0.240137245 0.405640585 0.013854419 -
0.114901796 -0.303446625 0.31609552 0.031689533 -0.002122582
-0.232976035 -0.057381174 0.04027993 0.240833792 -

0.006415925 -0.074878485 0.141462217 -0.039659254 -0.373437702
0.079897262 -0.041421433 -0.031542494 -0.340112605
0.151780588 -0.226476456 -0.366359056 -0.090031061 -
0.388098512 -0.141815321 0.095506835 -0.066266602 0.260965195
0.182663738 0.050520164 0.077059606 -0.108190982
0.039771178 0.013349499 -0.187892026 0.050162244 -
0.083313687 -0.123806887 -0.132984258 0.379114615 0.452765427
0.305436108 0.150145802 0.246336505 0.165922435 -
0.195576677 -0.02834726 -0.235856771 0.415573432 0.292201741
0.043457334 -0.090117856 -0.011596295 -0.051101689 -
0.270272728 -0.163459928 -0.385681728 0.144164302 0.125546185
-0.146485867 -0.269492912 0.048115977 -0.140817777 -
0.240645134 0.199807999 0.143258149 0.11994516-0.27746437 -
0.230328275 -0.071571047 0.050567553 -0.013055437 0.193575541
-0.027968162 0.269685684 0.1382883 -0.291831758 0.16981871
0.064931624 0.161320225 -0.177877857 -0.253320005 -
0.104108763 0.157219357 0.033244326 -0.177763637 0.02637637
0.024869086 0.122621729 0.159732505 -0.002017159 -
0.004839819 -0.236646283 0.129336633 -0.248577183 0.122612392
0.104777774 0.14462433 0.114558557 0.099798439
0.024501689 -0.021303556 0.144639698 0.113490027 -
0.189550412

KEGG_GLYCOLYSIS_GLUCONEOGENESIS 0.460093145 0.286795276
-0.305616668 -0.211753865 0.167087867 0.310098236
0.159784329 0.056328267 -0.191995965 -0.260986167 -
0.246280348 0.016111213 -0.365922596 -0.215113606 0.066312191
-0.011284178 -0.146116591 0.293997971 -0.487335188
0.056518513 -0.324396242 0.367365157 -0.446414946 -
0.246164052 0.04507373 -0.217316567 0.125293488 0.095549043
-0.317842149 -0.350179254 0.484258458 -0.115218181
0.302684441 0.372729447 -0.343442969 -0.258069306
0.061518928 0.288237086 -0.439189346 0.198601627 -
0.006593451 0.364695094 -0.45774379 0.322811233 -0.143532809
0.167666401 0.13656007 -0.058589847 0.041336408 -
0.076667967 0.080655616 -0.413491407 0.302841715 -0.458356231
0.234684382 0.211870983 -0.222903674 0.344269919
0.422768016 -0.205527133 -0.478023596 -0.281052679
0.255474113 -0.220849592 -0.119703903 -0.419026779
0.073107651 -0.011619284 0.000146868 -0.285834715 -
0.402644288 -0.408919881 -0.000579668 -0.121665973 0.101945222
0.023779012 0.296292664 0.231747305 0.413531773
0.431778186 0.242438985 -0.192775146 0.413485673
0.014008796 -0.007122528 0.009896141 -0.412469092 -
0.332385621 -0.255158428 0.125204168 -0.225705844 0.067631256

-0.12573141 0.414268095 0.031943444 -0.147480968
0.149972897 -0.505020484 0.480253847 0.321048657
0.085368012 -0.240243042 0.018352501 0.446204055 -
0.012725618 -0.223326959 0.383446668 0.326129294 -0.511720428
-0.048230212 0.087088904 0.481064848 0.434644336 -
0.443845607 -0.125341674 -0.0720409 0.246227877 -0.107907754 -
0.220546117 -0.015398878 0.143069778 0.522879811 0.197249152
0.104969448 -0.172460972 0.290242063 0.00920098 -
0.508218509 0.517103149 0.11974052-0.025715798 -0.433689046 -
0.033235619 -0.106744388 0.053981055 -0.583327082 -0.486008597
0.41654206 -0.085637223 -0.438604931 0.118470373
0.429555368 -0.211712827 0.011274789 -0.256568457 -
0.1546421 0.214816651 0.531650961 -0.027537722 0.259491027 -
0.093893233 -0.23843581 -0.397709011 -0.043078925 -0.295334317
0.270459119 -0.424004941 0.510602117 -0.138091169 -
0.113457082 -0.245007786 0.342279352 -0.141548253 -0.344745926
0.439823715 -0.311269325 0.37323351 0.020411181
0.039598001 -0.440682447 0.016478492 0.318312771
0.056804638 0.284210075 -0.39193577 -0.093282543 -
0.425207037 0.081662816 -0.069675377 -0.219362497 0.293188424
0.167873335 0.376716855 0.129248361 -0.296353296
0.285926255 -0.030218951 -0.321848139 -0.436869759

0.394215322 -0.0068254 0.473507494 0.279957633 -0.240360326
-0.138909613 0.399382151 0.112190924 -0.051140436 -
0.067309202 -0.104083842 0.010988548 0.211457441 -0.306154329
0.383168785 0.003023251 0.322772573 -0.19518094
0.267582031 -0.385481801 0.180211942 0.281068696 -
0.46894014 0.104970724 -0.425178422 0.383389034 -0.160888952
0.122522824 -0.182517877 0.358404886 -0.443755518 -
0.428362452 0.521928266 0.403933597 -0.388813369 -0.44167915
0.27239124 0.156304561 -0.103509932 0.010684918
0.452922652 0.226391231 -0.339555772 0.058786367 -
0.109354606 0.181835425 0.375289065 -0.29930729 -0.316644917
-0.143047954 0.284130753 0.136141492 -0.159261793 -
0.045396338 0.107639519 -0.316596758 0.02679334 0.31688608
-0.049303221 -0.108744327 -0.21560533 -0.052972621
0.113586234 -0.318579146 -0.423044363 0.160378307 -
0.497277532 0.332964673 -0.100578417 0.401353164 0.20416315
-0.464596185 0.277864209 0.33297307 0.040161001
0.25392015 -0.457217891 -0.298420429 0.272977681
0.308094211 0.260234878 0.30944374 -0.368902603
0.014473343 -0.006408621 0.301445264 0.309502559
0.377944886 0.242944939 -0.281532345 -0.243358816 -
0.403300541 0.192538885 -0.296790758 -0.161289221 -0.361084609

0.430025846 -0.283554384 -0.316704704 -0.312441154 -
0.109885814 -0.216691084 0.339213768 0.127312625 -0.306633568
-0.156764119 -0.231666509 0.335745298 -0.015913322 -
0.303104428 0.012768261 0.218833602 0.300377228 0.210253956
0.086844005 0.604280578 -0.010064598 -0.355405336
0.209790467 -0.49603675 -0.166309967 -0.248576411
0.349142142 -0.417781031 -0.079799262 0.325786627
0.323840232 -0.071354477 0.202867601 0.145094883
0.235209331 0.055692907 0.465565314 0.279411031
0.194784558 0.190223157 0.293785377 -0.020505357
0.361710229 0.45185204 0.37500038 -0.055502617
0.412574874 -0.26337859 0.584321051 -0.236149367
0.34392328 0.213887526 -0.276210874 -0.305134377
0.027975616 0.154761801 0.191342729 -0.336012002 -
0.321531301 0.111011263 0.041101 -0.402867165 0.336337582
0.086268711 -0.316659066 -0.364153271 0.179898433 -
0.410055966 0.310429961 0.233055046 0.139174969 -0.038888789
0.077216031 0.492507395 0.285549238 -0.090983071
0.02595345 0.134471119 -0.379861098 0.191110831 -
0.196205929 0.214141308 -0.173012037 -0.215523334 -0.489728637
-0.313939328 -0.447791207 0.19051051 0.273852959 -
0.234566926 -0.18577207 -0.374233232 0.423390033 -0.305959821

-0.291117963 -0.33096196 -0.164773538 0.320425011 -
0.328023478 -0.131042121 0.196470219 -0.142295236 -0.334318819
0.077567665 -0.113886292 0.176270164 -0.043220343 -
0.243560129 0.427582522 -0.244938322 0.204678103 0.344102764
-0.131475316 -0.411400158 -0.031971993 -0.126803632
0.018481464 -0.266690548 0.470842281 0.012704009
0.214512998 0.203752349 0.124342545 0.17729656
0.164207679 0.429654177 -0.130414045 0.345884797 -
0.477611789 0.187892024 -0.298079994 0.234104683 0.51047654
0.26737663 -0.331977229 -0.493736522 0.343148631
0.214501436 -0.206456207 -0.195748451 0.09336354
0.505860287 -0.268393475 -0.260918097 0.135371961
0.028390578 -0.016804529 -0.094477868 0.206599694
0.068921898 -0.497680621 0.173282976 -0.116160287 -
0.332208335 0.141377886 0.019556111 -0.285728042 0.179423399
-0.197220944 -0.185916321 -0.178060979 0.024157199 -
0.474813528 -0.318629975 0.371529455 -0.399477086 0.035573171
0.062163152 0.232530385 0.001130798 -0.250555163 -
0.355890186 -0.437424307 -0.334883837 -0.073921814 0.435281444
-0.256441391 -0.424285976 -0.247938767 -0.239355506
0.365011383 -0.095359677 -0.275359541 0.104457989 -
0.3678567 -0.415800756 0.42474207 0.480966337 -0.495210418 -

0.229405584 0.121245284 0.104904752 0.415441502 0.004792458 -0.242721706 -0.422837205 0.306578592 0.272271249 0.314953532 0.38646278 0.394012023 -0.173103142 0.300035093 -0.11005847 -0.211289191 -0.153693495 -0.198146369 0.194500325 -0.543127062 -0.142440369 0.123506789 0.520740853 0.038228938 -0.181995021 0.364790806 0.25708987 0.29973607 0.455828425 -0.202793567 -0.363372211 0.233893875 0.181733282 -0.01610102 0.377075209 -0.262302006 0.260526349 0.095170721 -0.267777156 -0.358470504 0.422885109 0.267305 0.096287149 0.035338514 0.122028767 -0.085962702 0.428144387 -0.298777015 -0.052047822 0.249164031 0.283503979 -0.377467962 0.166557472 0.293130082 -0.224176803 -0.491068987 0.008969285 0.329421888 -0.203656428 0.008935655 0.21322668 -0.008521962 0.060790901 0.259303733 0.18977486 -0.329016439 -0.024576384 -0.056978624 -0.08191472 -0.149144333 -0.074162674 -0.305699966 0.065721421 0.000438389 -0.105078195 0.348645682 0.196402005 -0.232703002 0.142902937 -0.139126292 0.216253494 -0.323465868 0.242108539 -0.429587632 0.19584975 -0.337971171 -0.0246478 0.087166746 0.337161468 0.061715496 -0.28183993 0.062714949 -0.155212829 -0.063202443 0.272308058

KEGG_CITRATE_CYCLE_TCA_CYCLE 0.649999344 0.646041588 -
0.39940351 -0.380847088 -0.55289888 0.037111918 0.435002704
-0.599416574 -0.133660838 -0.520979452 -0.402792393
0.670307756 -0.579458085 0.014059588 -0.50968854 -
0.278978665 0.304657865 0.522956064 -0.697591644 -0.269771983
-0.24416239 0.304091161 -0.609836047 0.044811896 -
0.454727805 -0.481958251 -0.044822731 0.154986915 -0.227015132
-0.558217427 0.630051899 -0.192686394 0.096955902 -
0.06401804 -0.133952873 -0.544552392 -0.18728459 -0.06930564
-0.600293527 0.293438346 0.470354745 0.478279438 -
0.373233392 0.618578964 0.282568765 0.60840407 -0.00815429
0.403951229 0.411296516 -0.474996857 0.706823794 -
0.387128917 0.504028731 -0.385793735 0.611218623 0.686862834
-0.603145026 0.357080985 0.565338243 -0.213647483 -
0.582973649 -0.204050398 0.230173422 0.005008858 -0.4550399 -
0.49393842 -0.218495635 0.57805526 -0.300949329 -0.638349668
0.032721996 -0.500501505 0.691135363 -0.219100646 -
0.033622718 -0.505385227 0.197895261 0.43434595 0.421069742
0.626354567 -0.226516324 -0.065359773 0.528019488 -
0.079419789 0.162076052 -0.140347713 -0.090491458 0.059825013
-0.574259065 0.314761988 -0.379721666 -0.43738382 -
0.133328032 0.553898412 0.546847191 -0.284582118 0.578237019

-0.736220227 0.542652011 0.584585377 0.272669174 -
0.700439358 0.188576437 0.644412192 -0.361113776 -0.3850462
0.490926091 0.770380196 -0.587293941 -0.575391062 -
0.462552564 0.312545358 0.629548974 -0.44754119 -0.277411206
-0.109426638 0.052656426 -0.540650833 -0.653386832
0.569687844 -0.377122175 0.642393507 -0.074391967
0.571060752 -0.501094257 -0.287560908 0.594467034 -
0.432163438 0.623882053 0.390861849 0.098222779 -0.694036293
-0.396643769 0.208496019 0.549991014 -0.582078784 -
0.59190147 0.694284956 -0.199067067 -0.501768977 0.072881641
0.444779817 0.282696185 -0.04247331 -0.289091508
0.031461579 0.186103405 0.687313942 -0.546165038 -
0.193063595 -0.580560221 -0.090961351 -0.49803915 -0.229409875
-0.038391328 -0.398632123 -0.528075086 0.548987696
0.322554921 -0.558474695 -0.294423337 0.139541527 -
0.106563869 -0.667398867 0.672943402 -0.494524988 0.382739243
0.13298317 -0.483990337 -0.61532386 0.388363179
0.390400643 -0.329923169 0.245895557 -0.301605727
0.065746988 -0.586872213 0.565734429 0.022287189 -
0.315877134 -0.028914237 0.546361816 -0.021764697 0.083177157
-0.314009923 0.473963147 -0.003650365 -0.276099073 -
0.605388383 0.524509278 0.169329693 0.587660443 0.375116853

-0.530478383 0.250121659 0.032858503 -0.026862318
0.285713832 -0.385596636 0.346702541 -0.372156997
0.26738946 0.095469378 0.546827616 -0.043714939
0.029574578 -0.438896189 0.238513291 -0.31251312
0.129390485 0.696590896 -0.245934497 0.553017395 -
0.432600675 -0.400763041 0.051194565 0.511674896 0.004369806
0.238717214 -0.481144436 -0.550113014 0.726346488 -
0.260241459 -0.151991898 -0.654574727 -0.395609644 -0.075105476
-0.496586855 0.2661784 0.523250377 -0.109683003 -0.472239599
-0.044036019 -0.308434294 -0.013909951 0.425967404 -
0.519479744 -0.12389399 -0.299871356 0.145556724 0.512378373
-0.173951348 0.092834538 0.13486603 -0.470350062
0.010506382 -0.318420588 -0.461886632 -0.192761662 -
0.559671562 0.234999823 0.251479695 -0.599276387 -0.593888387
-0.320312819 -0.424677201 0.646827338 -0.532082495
0.623319772 -0.307035501 -0.646251708 0.480314605 -
0.167984884 -0.285725897 0.572654679 -0.551052007 -0.504671162
0.308303608 -0.085953947 -0.504256593 0.479731129 -
0.107530667 0.378212032 0.299159633 0.450927584 0.381486145
0.370666617 0.212948746 -0.434494792 -0.516501973 -
0.72145576 0.095078198 -0.420424963 -0.657119467 -0.806137428
0.215101306 -0.688955578 -0.16232126 -0.292947205 -

0.516357063 -0.444094294 0.335756781 0.080219878 -0.379809415
-0.532372755 -0.53014275 0.354367254 -0.134705137 -
0.503849821 -0.370361828 0.192398753 0.637130553 -0.178591028
-0.212993158 0.698381945 -0.076727738 -0.490304882
0.429604301 -0.646328241 -0.635223854 -0.471714976 -
0.086130594 -0.679985701 0.067498166 0.505483662 0.674571555
-0.547642959 0.457272007 0.380554393 0.41601432
0.538412349 0.73022938 0.515095574 -0.500724165
0.266623859 0.209728086 -0.015144793 0.252115984
0.245714159 0.022675383 -0.494453142 0.50006199 -
0.43757916 0.74694031 -0.133940526 0.540422917 0.545982232
-0.509460433 -0.238030521 0.084922201 0.521369488
0.183717318 -0.68557075 -0.306787693 0.573492095
0.163822629 -0.39939341 0.610013275 0.075393909 -
0.605953867 -0.567783462 0.001528532 -0.234904871 0.461006805
0.4922805 -0.503372741 0.046733374 0.207893667 0.456146923
0.562117723 0.075018008 0.742300639 0.451457669 -
0.447779414 0.147104183 -0.319312967 -0.272856519 0.12784893
-0.623469898 -0.504065925 -0.538844525 -0.258180484
0.170913831 0.278584347 0.181771626 -0.636550786 -
0.581134192 0.613622869 -0.427700253 0.136630246 -0.532668539
-0.708119633 0.529390851 -0.473802996 -0.18926024

0.509310434 -0.253069555 -0.279422318 0.434105722 -
0.345804907 -0.08984658 0.364300783 -0.004701841 0.554869897
-0.308267009 0.37999908 0.465007003 -0.532688246 -
0.509829026 0.061536663 -0.355346546 0.178062708 -0.227858441
0.65028719 0.140404247 0.346605399 -0.128782842
0.056806923 0.286157323 0.395665954 0.604817135 -
0.163453072 0.297553756 -0.648238978 -0.225107815 -0.57763914
0.242866227 0.707396016 0.306249136 -0.285106713 -
0.551664946 0.337116796 0.631198461 0.2728563 -0.462480972
0.446401442 0.292776178 -0.076645326 -0.301942019
0.47895599 0.221074523 0.535169086 -0.44311386 -
0.334467167 0.5700136 -0.641228237 0.483063816 0.686611215 -
0.124025498 0.312763747 0.198445217 -0.228329736 -0.022405343
-0.62840955 -0.595131516 -0.163702331 0.647527005 -
0.726727156 -0.398677991 0.480230973 -0.56312672 -0.512314573
0.083040435 -0.178313963 0.296332123 0.04135457 -
0.523688672 -0.56403963 -0.248323918 -0.07443592 0.515610265
-0.479879185 -0.748687417 -0.389370644 -0.379184171
0.111681346 0.056456085 -0.355056537 0.275588416 -
0.488633921 -0.123568255 0.670552269 0.509312192 -0.594992784
-0.601441595 -0.02809752 -0.34597145 0.244176159 -
0.290281373 -0.219964807 0.015561473 -0.291918034 0.483180881

0.576249413 0.514749714 0.651296447 0.291848174
0.529004612 -0.393457677 -0.022576601 -0.150820539 -
0.527164795 0.234778924 -0.434363236 -0.16468706 -0.414678191
0.410671032 -0.31398159 0.4343638 0.394959323 0.519388286
0.538317799 0.374373677 -0.380100619 -0.383129635
0.149913993 0.050114771 0.103923719 -0.202225483 -
0.293423639 -0.308134006 0.391460499 -0.483503719 -0.589223086
0.58472903 0.540576627 0.00568955 0.277322349
0.278960653 -0.17354059 0.554433495 -0.557549285
0.190070895 0.713539219 0.56476118-0.281370617 0.287417376
-0.051000825 0.253567532 -0.557589067 -0.098351229
0.424203499 0.260904605 -0.143796109 0.175997476 -
0.349750013 -0.150202038 0.407844369 -0.49921577 -0.367494024
0.397051001 -0.358366531 0.006445742 -0.368687031 -
0.079513627 -0.166691724 -0.113134515 -0.039340785 -0.556516144
0.557139175 0.017911542 -0.362047937 -0.058345964 -
0.514129437 0.232252094 0.093568888 0.506813798 -0.443920815
0.531420367 -0.325739215 0.071281314 0.244290638 -
0.257185614 -0.207930476 -0.370085692 0.33232105 0.084971212
0.118958243 0.002018734
KEGG_PENTOSE_PHOSPHATE_PATHWAY 0.418434869 0.494924332
-0.360089721 -0.527664255 0.005398909 -0.026538256

0.003918269 0.22684054 0.137930138 -0.35763716 -
0.358170987 -0.083468898 -0.274263577 -0.165123273 -0.162401329
-0.033404055 0.10477858 0.451939573 -0.374029808
0.132538823 -0.21365176 0.292257775 -0.440678001 -
0.075930238 0.198648222 -0.481159794 0.035078305 0.405241487
-0.327466081 -0.191632993 0.210220439 -0.27243533
0.197717883 0.249529788 -0.169228613 -0.328230274 -
0.13229674 0.2398172 -0.605296144 0.135281338 -0.092548989
0.527111978 -0.526070508 0.157611343 -0.277689991
0.332942473 0.165734713 -0.309720927 0.385657248 -
0.078632681 0.049659317 -0.574194203 0.335786548 -0.490656568
0.282650284 0.364224108 -0.368422671 0.51787182
0.41856285 -0.014688033 -0.002302477 0.115542952
0.354210507 0.127977565 -0.299369234 -0.320308394
0.160312152 0.119630326 -0.118129259 -0.370116148 -
0.097455376 -0.282057701 -0.192183051 0.074923983 0.013108135
0.209010941 -0.133385251 0.112447638 0.396769748
0.192208573 -0.290336644 -0.06616566 0.362200474
0.027080709 0.081749265 -0.022114043 -0.308761288 -
0.373506736 -0.371212905 -0.402298926 0.074538953 -0.148120276
-0.18681241 0.347020234 -0.135364603 -0.360438828 -
0.154383576 -0.414505813 0.42894402 0.155737334 -0.087117879

-0.538582504 -0.319598614 0.545815098 -0.0712179 -0.281378603
0.290525399 0.227333826 -0.438616711 -0.315915646 -
0.195620795 0.502216816 0.37889937 -0.17629674 -0.295751013
-0.012052854 0.377323196 -0.49639602 -0.536980811 -
0.226590512 -0.204454164 0.42591427 0.289584557 -0.288694841
-0.234229192 -0.08601458 -0.044373409 -0.408515387
0.529023346 0.176994778 0.011968409 -0.385141596
0.045961785 -0.265647788 0.110604821 -0.42649731
0.057066565 0.232883134 0.302165076 -0.440111908
0.009417844 0.233369945 -0.324966918 -0.032960149
0.116297399 -0.14956283 -0.094340798 0.226359511 -
0.13801743 0.369577402 0.083376772 0.083247037 -0.193407625
0.398909582 -0.429668001 0.069358731 -0.500294566
0.396614415 -0.206848058 -0.137552052 -0.101283682
0.566067506 0.06739514 -0.380358184 0.509916784 -
0.044847759 0.313458139 0.170376586 -0.006385173 -0.475838649
0.216889545 -0.164118451 -0.10240057 0.327088494 -
0.268100442 0.178820127 -0.235623064 -0.092693612 0.199068266
0.136202939 0.178818711 -0.099367212 -0.087897747
0.35805568 -0.2470256 0.380937159 -0.190616311 -0.429333544
-0.382224675 0.140651464 0.413365842 0.297055442 -
0.108918846 -0.440336262 -0.153416572 0.396400284 0.092033902

-0.147529176 -0.45159225 -0.329737775 0.271714202
0.401332611 0.025564234 0.230532768 0.322680829
0.413517336 0.033323541 0.150090921 -0.251996717
0.233036675 0.266795678 -0.300557061 -0.165239305 -
0.167802294 0.275399659 0.042004338 0.407369166 0.244412874
-0.135335624 -0.484302835 -0.188985548 0.44851021 -
0.021035991 0.034506159 -0.354482879 0.042683869 0.368887213
0.000634795 0.167959372 0.464284407 0.217128545 -
0.099080895 -0.209634958 0.070912735 -0.011343319 -0.073716764
0.048862079 -0.49493522 -0.374640727 0.102570969
0.263567451 0.045118625 -0.07638543 0.367190914 -
0.093486281 -0.213366437 0.257844818 -0.223881985 -0.125500146
-0.390625594 -0.243427938 0.247713249 -0.422503932 -
0.285816117 0.012088868 -0.450442659 0.103983836 -0.470310043
0.448108203 -0.228032688 -0.4468382 0.404193248 0.260870152
0.296421955 0.362882119 -0.486005603 -0.07799773
0.409347333 -0.053320109 0.114931327 0.254473442 -
0.179770083 0.248954133 0.110732249 0.280954376 0.402685869
0.299151451 0.119293662 -0.517841634 -0.094328094 -
0.437452549 0.416864638 0.112230619 -0.309387445 -0.516721337
0.191830514 -0.378869607 -0.514018414 0.038533946 -
0.218401098 -0.284915595 0.4507994 -0.307453394 0.093434399 -

0.341002177 -0.324986508 0.119699017 -0.459384631 -0.004439242
-0.244913551 -0.198891524 0.117110663 -0.007797659
0.327428842 0.416229671 0.018061026 -0.007906772
0.295642791 -0.27690544 -0.359296749 -0.097284098
0.343146712 -0.42838776 0.158594497 0.346029379
0.355862819 -0.407093846 0.351285049 -0.000317395
0.202547818 -0.057644886 0.47427513 0.200708697 -
0.07368021 0.476866784 0.139684358 -0.005701188 0.131940287
0.317442872 0.482387544 -0.147529758 0.159076149
0.234798742 0.536942823 -0.05867195 0.467849141
0.195401562 -0.147236663 -0.228365771 -0.040190178
0.122794648 0.002354482 -0.585290299 -0.459898035
0.073770913 0.051866972 -0.378744004 0.272623993
0.107513842 -0.33863631 -0.342098309 0.30137546 -
0.264171722 -0.153646445 0.201610913 -0.218925678 -0.15066831
0.362080316 0.427468026 0.181377481 -0.332824296 -
0.317618429 0.19485633 0.340439931 0.262814811 -0.275416005
0.340083712 -0.097926507 -0.089020497 -0.597958269 -
0.549606889 -0.234000969 0.105332548 0.286693244 -0.131369879
-0.039472547 -0.290208823 0.379345782 -0.086828886
0.035859882 -0.382183723 -0.220590132 0.080708872 -
0.355635021 0.325854837 -0.312724822 -0.23612127 -0.139109391

0.165719297 -0.056645186 0.300938038 -0.251808917 -
0.231184372 0.415013323 -0.062147504 0.062659411 0.099344969
0.306410582 -0.516942227 -0.045299363 -0.414196978
0.019558082 -0.297332033 0.337980856 0.183939533 -
0.285174953 -0.18010287 -0.168139388 0.045020948 0.01557614
0.411384478 -0.202317671 -0.067300017 -0.256119721 -
0.032680285 -0.092670148 -0.258653825 0.332963384 0.511415861
0.108483478 -0.227636032 0.255527969 0.110241799
0.28083846 -0.045847773 0.215779905 0.379747371
0.064075591 0.165741149 0.078051704 0.029099532
0.188766894 0.199574891 -0.253477804 -0.311230048 -
0.321464933 0.226342513 0.018197282 -0.049074777 0.030485754
-0.259395109 -0.242441793 -0.130779167 -0.379882467
0.081125146 -0.255221981 -0.023946687 -0.536687322 -
0.346795986 -0.032101584 -0.047672142 -0.227589212 -0.205074763
0.20807403 0.03047756 0.042964618 -0.356258067 -
0.38255014 -0.402301717 0.295833673 0.286391192 -0.266962435
-0.379295325 0.18982129 0.060387916 0.27544204
0.034534377 -0.329226608 -0.055483642 -0.390994135 -
0.531413992 0.310852916 0.471560164 -0.480574124 0.051958613
0.191457664 0.066572186 0.343352425 -0.211809217
0.010101004 -0.405052883 0.346175637 -0.103186544

0.252320084 0.504359607 0.206669234 -0.223998807
0.197812277 0.066088476 0.117057406 -0.361937543
0.028938289 0.274368447 -0.579966307 -0.097367577 -
0.074620456 0.34686981 0.261226648 -0.262563105 0.141858824
0.161858133 0.191728374 0.539520946 0.01525136 -
0.165753439 0.544218751 0.037490076 -0.000355683 0.511148193
-0.098005906 0.22782083 -0.126170556 -0.140214122 -
0.509330533 0.367830837 0.369747086 0.322538341 0.098902152
-0.21705047 -0.137697803 0.405644223 -0.058008209
0.270996379 0.185176636 0.133347083 -0.165937994
0.138162713 0.322005774 -0.216643716 -0.41361208 -
0.438760171 0.358397043 0.034489726 -0.257065849 0.254844014
-0.174086394 -0.329045613 0.399555648 0.277784563 -
0.168931655 -0.118583414 0.128564007 -0.331187272 -0.238813954
-0.018248793 -0.254177637 0.377629764 0.202142763
0.073283235 0.408417741 -0.246445406 -0.252051712
0.317451713 -0.045372805 0.397440071 -0.229018257
0.12119133-0.461555215 -0.13147497 -0.013792018 0.29261392
-0.36476951 -0.034480616 0.112347114 -0.455849493
0.240840326 -0.300354192 -0.314051898 0.17631482
KEGG_PENTOSE_AND_GLUCURONATE_INTERCONVERSIONS
0.271020915 0.363362974 -0.32137022 -0.369469129

0.38686465 -0.00294037 0.030514628 0.192474562 -
0.386782283 -0.009052398 0.104088582 -0.311870022 -0.233160675
-0.335049881 -0.269240253 0.353002491 -0.07230369
0.291603538 -0.261959239 0.054595591 -0.278914052
0.521806675 -0.554243961 -0.071407625 -0.345153695
0.370980128 0.327182055 0.059863831 0.295122831
0.055790922 0.571085115 0.24455495 0.301305571
0.228690968 -0.216701133 -0.22544234 -0.363869154
0.365562407 -0.564561907 -0.202955685 -0.186907577
0.495563722 -0.512773134 0.459059534 -0.564781349
0.014321309 -0.495626279 -0.208160207 0.420389626
0.161207044 -0.099003512 -0.234735166 0.163314666 -
0.650678171 0.2344221 0.14332525 -0.306819077 0.277036324
0.413134535 -0.514296466 -0.502132032 -0.054518166
0.251117441 -0.042535498 -0.114706542 -0.124077982
0.310666822 0.307634128 0.517469768 0.130807429 -
0.323049858 -0.169145009 0.081334885 0.436173747 0.302453132
0.153844543 -0.191640131 0.437941466 0.57668772
0.447238232 -0.22241749 -0.159751982 0.468538197 -
0.124310207 0.32836975 -0.312920513 -0.655406437 0.196072326
-0.509997329 0.476119762 -0.168664188 0.250885062 -
0.108688478 0.518330732 -0.135060657 -0.207537301 0.133944391

-0.248128396 0.423942629 0.576189893 -0.276197614
0.151858396 0.163695482 0.472862218 0.134501337 -
0.140777927 0.487914764 0.474312119 -0.57610761 -0.249512073
0.174753083 0.448926856 0.438764476 -0.535501179
0.193401777 0.048183063 0.389430558 0.176221864
0.081300197 0.105534707 0.035238396 0.504023815 -
0.328295486 0.061041481 -0.281264362 0.373558115 0.301130761
-0.421856575 0.2898966 -0.280933274 0.165283912 -0.205379637
-0.128378241 0.033622978 0.38260242 -0.618632962 -
0.463433741 0.282684348 -0.179229387 -0.531167907 0.334197819
0.500843669 -0.033003425 0.172484003 -0.370240311 -
0.267566807 0.302138032 0.432133923 0.349725684 0.36477913
-0.063657629 -0.240366418 -0.417225763 0.520797732 -
0.288407593 0.472783523 -0.452742046 0.445141303 -0.419235178
0.127115362 -0.098688666 0.43025539 0.093288938 -
0.35690077 0.454990442 -0.095343775 0.462160784 -0.143052924
0.468544382 0.091230243 -0.127043079 0.373235007
0.077732234 0.286104348 -0.510311073 -0.468307826 -
0.097327308 0.042631231 -0.273050899 -0.006700393 0.125758102
0.039408585 -0.149930965 0.243384835 -0.567038627
0.278649134 -0.067787841 0.222978577 -0.466056575
0.256720442 0.578402549 0.48480251 0.253918855 -

0.413091881 0.260069234 0.412347154 0.1669118 0.24982867
0.353244353 0.05658456 -0.220870108 0.419132227
0.16705342 0.595000354 0.119497109 0.154066934 -
0.229308933 0.537788276 -0.334899183 0.39393464 0.130827498
-0.535594831 -0.158175421 -0.155129642 0.396313675 -
0.363997129 0.268831489 -0.185140669 0.434104235 -0.370977566
-0.236715863 0.184043779 0.195566111 -0.539501932 -
0.214465791 -0.102141636 -0.011131006 0.228947135 0.160544258
0.325727464 0.269141295 0.083304439 0.135662484
0.257586397 0.161788114 0.197798422 -0.347679914 -
0.45595992 -0.056417389 0.227253177 0.006741356 -0.384031162
-0.375576427 0.311717627 -0.156198865 -0.106139087
0.458936384 0.343882524 0.210761063 -0.047407467 -
0.147158236 -0.16367936 -0.30820774 -0.500819471 0.100791096
-0.569582065 0.316376323 -0.067131953 0.074691764 -
0.347730114 -0.543517424 0.301582787 -0.410155771 0.172305654
-0.055178869 -0.291815133 -0.075236031 0.557732101 -
0.045704112 0.244510835 0.490154815 -0.384952303 0.375374555
0.284060077 -0.017087039 0.291054082 0.15897802
0.402090571 -0.189335085 -0.266906627 -0.561265871
0.389728736 -0.004254424 0.323714031 -0.493714432
0.25155901 -0.066119059 0.005656466 0.068289637 -

0.083179753 0.012183528 0.31429223 -0.203188534 0.120262989
0.094667364 0.263733027 -0.014384299 -0.088347098 -
0.284597725 -0.112091813 -0.292545164 0.31059463 0.067102656
0.279714662 0.505355615 0.369898351 -0.283574626
0.053209522 -0.375410839 0.083893266 0.004510075
0.249316872 -0.502769588 -0.114217361 0.273132347 -
0.459973821 -0.128167531 0.49794293 -0.400749773 -0.045196996
-0.059805162 0.182191273 0.131634473 0.280073186
0.208632953 0.570476262 -0.216270299 0.314016138
0.053414673 0.099446731 -0.465950325 0.211876833 -
0.100957055 0.61951426 -0.49125777 0.19518217 -0.045482262
-0.313405666 -0.462781557 -0.324116366 0.175963545
0.024352536 -0.218854957 -0.030000364 0.406207185
0.278477136 -0.243673672 0.289491853 0.398826169 -
0.254631324 -0.594097712 0.290779024 -0.531665653 0.534663367
0.107833214 0.070185416 -0.305104367 0.388361466
0.356728231 0.413447941 0.267294242 -0.068537952
0.139534106 -0.451301729 0.428520541 -0.070106666 -
0.010630499 -0.285779976 -0.305582974 -0.507033806 -0.597694955
-0.735831775 0.058655691 0.427301301 0.093282553 -
0.276603123 -0.218456785 0.455841542 -0.540985243 -0.071885925
-0.248966567 -0.170929855 -0.003734482 -0.289692731 -

0.452536607 0.097919408 0.16175623 -0.318267313 -0.123251494
0.053529662 0.320188736 0.184714978 -0.087628406
0.268886165 0.212067769 0.039602737 0.007595458 -
0.025130166 -0.279865475 -0.312924035 0.02771261 0.230576943
-0.52566397 0.56153357 0.059331584 0.449864533
0.119961153 0.216776828 -0.272442575 0.137383293
0.347793983 -0.566850611 0.302031677 -0.399356998 -
0.27195407 -0.194635666 0.152932228 0.400581764 -0.014092492
0.187280148 -0.43024419 0.16027531 0.119098941 -
0.157607159 -0.256746364 0.304297942 0.548957399 -0.557262378
-0.435715405 0.148754989 0.314446804 0.149253511
0.17538018 0.027006948 0.445945409 -0.378447997
0.305981685 -0.080928176 -0.302065411 -0.14677256 -
0.235652602 -0.61985707 0.399727228 -0.124865714 -0.310832361
-0.16006382 0.015913583 -0.505789285 -0.157109134
0.163773498 -0.463263784 -0.083673652 -0.083976228
0.348103897 0.386953716 -0.26658901 -0.586809554 -
0.479820041 -0.22166263 -0.03450772 0.302638177 0.038093857
-0.26248554 0.211575011 -0.35345528 0.377585941 -
0.067114946 0.182119427 -0.148674314 0.049048223 -0.184150388
0.427658505 0.495066912 -0.586753654 -0.086048907 -
0.176164443 0.318351293 0.449515374 0.147337013 -0.407049377

-0.419901345 -0.141141803 -0.149662318 -0.050144546
0.563667527 0.195572534 0.288738229 0.479169604
0.170332597 -0.412974111 -0.234855622 0.254557536
0.262053152 -0.329822234 -0.113495529 0.25566032
0.468343539 0.12735033 0.448684503 0.474286168
0.093465524 0.129065837 0.433612735 0.280413147 -
0.698056606 -0.167573475 -0.13928698 0.082216566 0.51034719
-0.330753776 -0.043618842 0.140274644 -0.320241291
0.173417807 0.138876945 0.55767596 0.047393005
0.06630214 0.339141389 -0.215097402 0.573741348 -
0.361360838 -0.123251189 0.438238413 0.08981716 -0.413624415
0.314759705 0.260326572 -0.146958407 -0.463237765 -
0.058602105 0.322363404 0.110279585 -0.142204269 0.290247291
0.320775874 -0.087231694 0.11304801-0.088949154 -0.202915459
0.51598734 -0.519614103 -0.269953649 0.428655522
0.155261385 0.125565214 -0.029281964 -0.313206652
0.404074776 0.65928967 0.396013455 -0.528941091
0.058693873 -0.162659152 -0.238808593 0.111587138
0.60955194 -0.221256565 0.481558835 -0.498740787
0.246352042 0.217127986 -0.032553908 0.526020376 -
0.267448442 -0.285725766 -0.308559154 0.05522972 0.27895186
KEGG_FRUCTOSE_AND_MANNOSE_METABOLISM 0.402731992

9998 0.321406125 -0.354099333 -0.383979252 0.279203973

9999 0.067329218 0.061278089 0.141496055 -0.03407572 -

10000 0.220128211 -0.10731273 -0.28350732 -0.489263865 -0.244466412

10001 -0.149247801 0.074320782 -0.012012896 0.325175976 -

10002 0.371215574 0.155112962 -0.245864026 0.155942833 -0.439371208

10003 -0.12921875 0.009453245 -0.452778165 -0.104501553

10004 0.171915582 0.070363511 -0.201931435 0.271580267

10005 0.247607961 0.392718836 0.215594847 -0.391560889 -

10006 0.147807991 -0.431497105 0.290899792 -0.400143243 0.36792468

10007 -0.300650297 0.503659208 -0.26799624 0.34536294 -

10008 0.219856401 0.219796473 0.031448176 -0.37830502 0.104437681

10009 -0.231198852 -0.242184871 -0.561001624 0.400385969 -

10010 0.263336399 0.10690091 0.358260104 -0.263029691 0.254140518

10011 0.208196567 -0.159167829 -0.440119833 -0.174818286

10012 0.334342505 0.068427417 -0.338988104 -0.243144683

10013 0.100486761 -0.271380389 -0.315249066 -0.204995527 -

10014 0.281054461 -0.065904688 -0.410435782 -0.100815995 0.266224659

10015 0.189934682 0.171507859 0.377164989 0.195400493

10016 0.301759141 0.069737206 -0.211346817 0.499073637 -

10017 0.130144668 -0.021874768 0.216390321 -0.408539005 -0.23647504

10018 -0.173808223 -0.091843285 -0.210924395 -0.319922847 -

10019 0.213774191 0.330294235 0.417379746 0.057983 -0.147850958 -

0.202094799 0.457481304 -0.043066386 0.05347764 -0.229585541
-0.229531246 0.49249154 0.008875692 -0.235288468
0.279075339 0.108868783 -0.440736316 -0.260135278 -
0.332726211 0.398507588 0.221080532 -0.378604391 -0.201644927
0.253089558 0.287592144 -0.337761684 -0.470119209 -
0.143306171 0.078425348 0.302725258 0.400246288 -0.351650039
-0.218546259 -0.120040749 -0.298708521 -0.398397297
0.586017661 0.22605989 -0.021310404 -0.277559342 -
0.0985652 0.222142383 0.017755508 -0.478453962 -0.337936145
0.463181975 0.201673965 -0.310948442 0.165013229
0.506933287 -0.29658454 -0.091430616 -0.254226698 -
0.274706433 0.035126077 0.534199533 -0.034937898 0.195076315
-0.10062953 -0.10289968 -0.187152888 -0.231062425 -
0.197468377 -0.033612748 -0.399690803 0.477927461 -0.168560222
-0.110869479 -0.260144312 0.512980961 0.261939121 -
0.433290246 0.428331361 -0.01893517 0.200755281 0.119456073
0.081375923 -0.334414004 0.072595524 0.083764028 -
0.31345824 -0.009510775 -0.312634977 -0.002469192 -0.255175441
-0.041587839 0.142849541 0.052251042 0.395888522 -
0.195276838 0.196435924 0.356527914 -0.339051444 0.284679708
0.1672351 -0.263923718 -0.343184436 0.10206715 0.316318046
0.291905326 0.065608877 -0.225471262 -0.220135006

10042 0.239842216 0.221172685 -0.31747673 -0.413459249 -
10043 0.105510867 0.098846213 0.363837398 -0.291289417 0.044927343
10044 0.227189911 0.372933578 -0.056524781 0.381403826 -
10045 0.367952113 0.374194284 0.144837954 -0.130923344 -0.0107579 -
10046 0.084146452 0.245829617 0.330065005 0.445263214 -0.092372771
10047 0.078929199 -0.102046483 -0.366452608 0.459508847
10048 0.04701162-0.379832131 -0.250363248 0.12690895 -0.038555039
10049 -0.14031574 0.0278133 0.443774066 0.275478005 -0.186995596
10050 -0.020714552 0.194825596 0.282006721 -0.026820033 -
10051 0.104581703 -0.258849987 -0.07337282 0.373415027 0.241198716
10052 -0.313963477 -0.217715297 0.042618282 -0.266943949
10053 0.079436952 0.474923211 -0.153157993 0.027440017 -
10054 0.062473582 0.085880361 -0.087907269 -0.25059446 -0.283869818
10055 0.077365239 -0.493166464 0.163401279 -0.02002838
10056 0.41851203 -0.088352203 -0.218594975 0.383913691
10057 0.235783963 0.193969044 -0.013219847 -0.462540487 -
10058 0.246537124 0.24401251 0.352511442 -0.195614262 0.148618393
10059 -0.206973589 0.193193983 -0.274592329 0.416113169
10060 0.428983475 0.21930898 0.11607059-0.416214655 -0.331874934
10061 -0.349408131 0.135263763 0.202514988 -0.211812074 -
10062 0.476433564 0.122381178 -0.259226085 -0.148136286 0.021132355
10063 -0.173317937 -0.285297368 0.381246795 0.19336525 -

10064 0.134181775 -0.065277811 0.017400134 0.342039083 0.097362395
10065 0.228763436 0.066306734 0.217965904 0.014233848
10066 0.049727226 0.017752161 0.430039911 -0.187351114 -
10067 0.010687261 0.098425111 -0.407050872 -0.195570439 0.01053647
10068 0.387804315 -0.389332 0.06764217 0.279829691 0.401721568
10069 -0.104438275 0.324484241 -0.006573439 0.42739318 -
10070 0.389011792 0.284977501 0.063014975 -0.209922912 0.382378711
10071 -0.025702591 -0.078497101 0.230866022 0.172087906
10072 0.110669264 -0.133919179 0.227265262 -0.176332706
10073 0.588528088 -0.252298193 -0.036727364 0.196896531 -
10074 0.05287984 -0.131617978 -0.013475648 -0.058594488 0.248032643
10075 -0.436952992 -0.420371688 -0.000723215 0.263625543 -
10076 0.292552028 0.390405392 0.012727024 -0.145441756 -0.260053418
10077 0.244209001 -0.313253472 0.290715674 0.093533378 -
10078 0.136031166 -0.150402216 0.444595183 0.400402196 -0.106315616
10079 -0.161113233 -0.453743004 -0.056925957 -0.219008884
10080 0.315142688 -0.13777387 0.140828891 -0.125922786 -
10081 0.233266331 -0.530206545 -0.342731842 -0.278669993 0.182791893
10082 0.164765472 -0.092683404 -0.07351339 -0.298343035
10083 0.309261177 -0.222772321 -0.098584982 -0.472792645
10084 0.01771249 0.365651387 -0.275368464 -0.021868686 -
10085 0.171652299 -0.222772603 -0.128161349 0.103675432 0.08702936

0.329258953 -0.234559588 -0.081699322 0.417237432 -
0.317467076 0.110932491 0.152970194 0.031167521 -0.471380512
-0.167177819 -0.144335607 0.257112351 -0.286704564
0.44887658 0.147921499 -0.118900773 -0.054496635 -
0.170837086 0.223322435 0.015110685 0.176127716 0.001212855
0.179865782 -0.351699948 0.161503186 -0.441220006
0.190417563 0.517839503 0.184967663 0.023394786 -
0.121434842 0.107372651 -0.032876282 -0.233924401 -0.363874263
0.075421025 0.231940369 -0.149496312 -0.232118654
0.043792187 -0.092754092 0.238152559 0.019379554 -
0.09521072 -0.302425545 -0.356082215 0.514773602 -0.411983095
-0.28161936 0.051332004 -0.241025264 -0.28598506
0.309717687 -0.419218565 0.100599231 0.261536543 -
0.378502548 -0.390625191 -0.11167037 0.121198162 -0.163753236
-0.046404753 0.075049219 -0.081630239 -0.083369113
0.222333025 -0.168873535 -0.479011625 -0.159095622 -
0.055416709 0.278669805 -0.175218858 -0.435131072 -0.150272455
-0.021829628 0.201729445 0.060034941 -0.238236073
0.014154801 -0.352776252 -0.228890645 0.22172522
0.403436986 -0.377421729 -0.046194379 0.242938781 -
0.187608562 0.255869085 0.01874913 -0.24896577 -0.405184644
0.305311847 0.068567881 -0.188180588 0.126659337

0.143136859 -0.035823115 0.107315729 0.078609381 -
0.108302012 -0.373361086 -0.308454052 -0.005353589 -0.527058301
-0.21361076 -0.049947841 0.359879615 0.12929053 -
0.383098661 0.378862066 0.329771183 0.183104841 0.352041898
-0.441760797 -0.489974402 0.318967612 -0.236624597 -
0.219353712 0.299345991 -0.112724378 -0.072606559 0.090899608
-0.13085742 -0.380833268 0.399399386 0.448654907
0.346585413 0.37197158 0.402983628 0.026236385
0.42937988 -0.249536471 -0.211800054 -0.071255915
0.378500503 -0.369742633 0.260420596 0.090002803 -
0.248527116 -0.405710186 -0.067846555 0.04704853 0.0639827 -
0.29201202 0.360598541 0.043904011 -0.098172182 -0.08692606
0.195930202 -0.446973746 -0.029249958 0.162413797 -
0.131418715 -0.163277766 -0.002614997 -0.189307327 0.13390126
0.053711542 -0.26269048 0.367881555 0.191202431 -
0.441795192 0.346048939 -0.212121828 0.195232265 -0.387923478
0.426758187 -0.50759282 0.295359186 -0.46810216
0.227539483 -0.0882449 0.095402671 0.295750822 -0.278662506
0.028277233 -0.333254915 -0.147184807 0.303147217
KEGG_GALACTOSE_METABOLISM 0.232150113 0.425063801 -
0.320385305 -0.44786005 0.24519018 0.018125808 -0.043714615
0.424878187 0.177737915 -0.22842783 0.01381966 -

0.328918494 -0.393017037 0.124506003 0.004788423 0.298482998
0.155168006 0.435643991 -0.221605884 -0.000986501 -
0.223703044 0.201298686 -0.214761676 -0.106640283 0.17872356
-0.228807831 -0.083684557 0.263891819 -0.210690134
0.029805645 0.415943835 0.424969179 0.189519047
0.155309329 -0.303810785 -0.157084851 -0.372760116
0.367785691 -0.354383647 0.141711668 -0.084350229
0.498958255 -0.191388594 0.139247105 -0.095765775
0.166555113 -0.208690133 -0.450395401 0.343648315 -
0.247198725 -0.076980421 -0.465447811 0.327536916 -0.414916847
0.232539211 0.343221562 -0.206762438 0.514064244
0.386347374 -0.189118587 -0.05313307 -0.248151483
0.108962226 -0.033197576 -0.016626217 0.020104033
0.129431516 -0.174091411 0.08555747 -0.34675642 -
0.107582686 -0.14428662 -0.25972449 -0.128868431 0.230172155
0.36371931 0.009689184 -0.039282375 0.377062326
0.224863353 0.206416021 -0.244498746 0.362547472 -
0.069273327 0.316627745 0.332089295 -0.222802056 -0.117928535
-0.334906864 -0.232030005 -0.132472839 -0.212793721 -
0.274521572 0.225480485 0.096629873 -0.162070079 -0.286610317
-0.286447912 0.434950667 0.221993927 -0.020570813 -
0.465620721 -0.193309061 0.519232979 0.346758706 0.033696431

10152 0.34344174 0.109947937 -0.32080076 -0.214230009 -

10153 0.363622471 0.481667717 0.142887213 -0.10425421 -0.218803344

10154 -0.197509607 0.221256306 -0.091538017 -0.344278586 -

10155 0.021198078 -0.146127455 0.18774021 0.25448017 -0.414679306

10156 -0.340116119 -0.075271622 0.082632031 -0.369793662

10157 0.321057348 0.08709649 -0.197884596 -0.359186836 -

10158 0.21671286 0.116169425 0.082183381 -0.226064436 -0.331913472

10159 0.433736716 0.119495375 -0.232344645 0.138726258

10160 0.434533666 -0.201456244 -0.339333751 -0.097874217

10161 0.011834391 -0.077672457 0.408664305 0.115375869

10162 0.283528122 0.136421163 -0.025989486 0.097125198

10163 0.129240497 -0.366768051 -0.237764089 -0.358242091

10164 0.380650774 -0.132613512 -0.08588044 -0.143397382

10165 0.504750322 -0.262957029 -0.289721391 0.453958772

10166 0.003614414 0.257409256 0.201378522 -0.199103837 -

10167 0.293111729 -0.038208999 0.068292884 -0.284543738 0.240738448

10168 -0.099691656 0.316986748 -0.420751504 -0.233764647

10169 0.035719539 0.169408121 0.450293613 -0.228028932 -

10170 0.157133281 0.108924663 -0.212807502 0.407872421 0.14047896

10171 -0.406125827 -0.337734 0.053685621 0.212282717 0.344871359

10172 -0.209168068 -0.0856643 -0.189669039 0.249226728 -0.159576048

10173 -0.093752949 -0.101063945 -0.278201677 0.289572166

0.40969414 -0.198585211 0.229872 0.289696264 0.398360454
-0.353214305 0.152188715 -0.20782739 0.004399099 -
0.083704604 -0.451346196 0.155995357 -0.191131962 0.425032664
-0.029282347 0.340157179 -0.047721506 -0.189432297 -
0.219824045 -0.2602321 0.481226482 -0.072475514 -0.035523224 -
0.05311974 -0.271649783 0.2591768 0.244818444 -0.188020862
0.492098887 0.190164805 -0.08629489 -0.438828715
0.385146318 0.287017133 -0.115777622 -0.285707404 -
0.499028199 -0.40398256 0.265564027 0.287689366 -0.1462796 -
0.418416609 0.204599461 -0.263953162 -0.030315741 0.365478924
-0.05916 -0.002048858 -0.418476494 -0.042980588 -0.164446331
-0.009304829 -0.1617368 -0.154458192 -0.324339143 0.229671666
-0.117480135 0.33567235 -0.207218526 0.025466735
0.065292524 -0.254984092 0.145719912 0.071056419 -
0.578314039 0.077779716 -0.003123876 -0.061179563 0.247921007
-0.185172697 0.112281282 -0.098679962 -0.065396691
0.279500971 0.404049957 0.026872559 0.043918476 -
0.442392265 -0.040000512 -0.346387355 0.392986648 0.190872024
-0.345573568 -0.313901127 -0.147204347 -0.338523865 -
0.304943269 0.100112494 -0.353714714 -0.315388826 0.383639826
-0.050976099 -0.169242872 -0.213699328 -0.150189143 -
0.092821611 0.06294659 0.266331565 -0.25331197 -0.05239693

10196 0.170586118 0.090129068 0.161065648 0.469204023

10197 0.006975135 0.077019114 0.011860722 -0.302657912 -

10198 0.379811933 0.008178203 0.223440976 -0.353037698 -0.2986751

10199 0.523494895 0.300453459 -0.320107715 0.180135987 -

10200 0.065156633 0.319460109 -0.234900304 0.234010444 0.411703608

10201 0.330927806 0.513219128 0.307449644 0.066439386

10202 0.10192466 0.125963085 0.222740449 0.161450639

10203 0.303788596 0.060452423 0.70432765 -0.16286094

10204 0.17320504 0.122408012 0.290574601 -0.422428158 -

10205 0.263943662 0.262824252 -0.178089516 -0.517794569 -0.306212188

10206 0.045864783 -0.004014142 -0.403970007 0.285528565 -

10207 0.087453119 0.241698288 -0.302195091 0.325284587 -0.119993806

10208 -0.104110037 0.110551926 0.02582738 -0.166008948 -

10209 0.045910341 0.430167347 0.218459835 -0.236296474 -0.264080325

10210 -0.199416353 0.032677906 0.293938431 -0.190752125

10211 0.125986432 0.046518226 -0.315752296 -0.257849328 -

10212 0.044662907 -0.109427299 0.143640864 0.334727901 -0.392947605

10213 0.121986652 0.073945301 0.46315802 0.15695618 -

10214 0.245841302 -0.345765196 0.093915309 0.130976243 -0.385405469

10215 0.189848889 -0.106282576 -0.279791991 -0.023473494

10216 0.069321636 0.203484774 0.03054714 -0.119114048 -

10217 0.226654514 0.373314993 0.131382209 0.091091906 5.71E-05

10218 0.138638218 -0.410928252 0.138926003 -0.217087948
10219 0.070264302 -0.357794393 0.07524016 0.256399838 -
10220 0.024248901 -0.012300623 -0.209448483 0.230938317 -0.015778716
10221 -0.137565892 -0.270716522 -0.070209116 -0.269127539
10222 0.020934312 -0.02989098 0.088312886 0.410164442
10223 0.347801938 0.149265465 -0.207177655 -0.304947238
10224 0.096312448 0.075806471 -0.244174087 0.138632532
10225 0.323206177 -0.241997096 -0.176158286 0.374755152
10226 0.13709044 0.073132805 0.191514011 -0.018140887 -
10227 0.287445224 -0.421038652 0.398730075 -0.142470667 -0.184198437
10228 0.269539173 -0.24744882 -0.05371651 -0.239306551 -
10229 0.371229953 0.056981382 -0.07049854 -0.068743581 -0.403007653
10230 0.227299082 0.126766216 0.002853367 -0.140795543
10231 0.198095946 0.213130627 -0.105242295 -0.146750197 -
10232 0.263159401 -0.373582524 -0.393519793 0.174920498 0.091283796
10233 -0.382307311 -0.207043335 -0.431739435 0.169115570.283236772
10234 0.165588596 -0.133781487 0.020918388 -0.427600123 -
10235 0.400311047 0.275081845 0.585036675 -0.430914593 0.169334912
10236 0.188625421 -0.215465496 0.091300369 -0.174422256 -
10237 0.23641531 -0.069610779 0.398908645 -0.170437628 0.165830978
10238 0.022042302 0.285708749 -0.184778513 0.182027257
10239 0.359025071 0.142909087 -0.323583512 -0.26348372

0.423141755 -0.447209543 -0.355383812 -0.217237178
0.414324532 0.067442667 -0.011592312 0.268456036
0.347365981 -0.319317632 0.438115822 -0.214214911 -
0.220454032 0.295803234 -0.186418288 -0.046512356 0.422178588
-0.251309337 0.08677646 0.01872377 0.117633547 -
0.313311888 0.22796508 -0.006157669 0.352244473 0.331636454
-0.059992519 -0.250484637 0.322251097 -0.454033739 -
0.106460842 0.239215236 0.092475631 -0.18369917 0.15446584
0.421998446 -0.10357092 -0.485893013 -0.211167621
0.485889018 0.378516655 -0.487172828 0.445756693 -
0.033355303 -0.264711275 0.081487606 0.055174588 -0.037144468
-0.372911828 0.148589897 0.040820799 -0.030904099 -
0.134180509 -0.317892794 0.079204426 0.326392999 -0.121501355
0.252966803 0.139008797 -0.550498009 0.268537926 -
0.137832696 0.453331307 -0.150214745 0.202068018 -0.435525506
0.034321187 -0.149530013 0.417875634 -0.177927162
0.035966963 -0.154641022 -0.412380094 -0.067048859 -
0.457706793 -0.083023989 0.324286744
KEGG_ASCORBATE_AND_ALDARATE_METABOLISM 0.521280919
0.487693229 -0.558407654 -0.265376534 0.333086723
0.156330772 0.162161254 -0.251429409 -0.606981489
0.094761116 0.212380048 -0.361731999 -0.218164448 -

0.528034156 -0.153409258 -0.076347389 -0.068159075 0.064851979

-0.217208972 0.101666765 -0.121917675 0.66503469 -

0.655104477 -0.088556391 -0.394728726 0.299709109 0.275395939

-0.010607688 0.274376273 -0.31092298 0.681375609

0.079424265 0.476284783 0.320402963 -0.357299004 -

0.346874811 -0.019838788 0.398170837 -0.44212904 -0.182765059

-0.286082642 0.477769773 -0.454786289 0.524892759 -

0.583222885 0.056521774 -0.21688945 -0.207111963 0.013113555

0.241144319 -0.165661423 -0.320472187 0.028845836 -

0.704313579 0.363269654 0.42262175 -0.554574702 0.144107891

0.503402972 -0.42916584 -0.594810207 -0.312166015

0.318667916 -0.268016296 -0.037456718 -0.276942762

0.112761795 0.280318232 0.265709717 0.25274485 -

0.270104817 -0.231497801 0.038718534 0.227286203 -0.051005272

0.107090293 0.436374873 0.517659015 0.537738805

0.65329095 -0.092766341 -0.001171828 0.432442091 -

0.080978933 -0.077059323 -0.537491056 -0.705530068 0.140819267

-0.447321214 0.607163258 -0.210463364 0.397240012

0.152604383 0.571366211 -0.137676908 -0.105322746

0.122887926 -0.401549658 0.469124974 0.529652425 -

0.047596404 0.252120597 0.32209342 0.402197198 -0.126576062

-0.474813275 0.493496965 0.452829004 -0.562771839 -

0.117918921 0.493514474 0.335163002 0.568901134 -0.586213135
0.387447679 0.427196754 0.412020134 0.177231128
0.112995805 0.167219861 0.317624608 0.68200814 -
0.190237632 0.214475713 -0.149244351 0.515746492 0.107344127
-0.593983122 0.480844493 -0.055364246 0.30668922 -
0.206300722 0.118237795 0.287775366 0.109712329 -0.62368954
-0.496551976 0.453847413 0.035876361 -0.552170705
0.379689225 0.514935921 0.033100262 0.240933203 -
0.512065246 -0.346277348 0.188826039 0.579801785 0.277060406
0.178035911 -0.034410166 -0.561703379 -0.493780378
0.484174989 -0.200394438 0.432915824 -0.567147627
0.579927257 -0.258765952 0.101111493 -0.117529883
0.123830082 -0.246976826 -0.372172521 0.556790435 -
0.202229081 0.554163589 -0.114071384 0.491408113 -0.055081369
-0.03821568 0.460764071 0.31625566 0.397114972 -
0.538099376 -0.520134842 -0.114419252 0.45790669 -0.298556935
-0.145038723 0.083168443 -0.016042565 -0.004790323
0.312069668 -0.434568456 -0.05095175 -0.160571757
0.325813731 -0.526418043 0.412478738 0.069193474
0.560143248 0.540377303 -0.416841165 0.153645333
0.166214241 0.327104108 0.103438382 0.371995334
0.243382595 -0.298700406 0.315748847 -0.093353795

0.64969967 -0.207372328 -0.187178482 -0.399904505
0.429564868 -0.2831056 0.153225424 0.417027738 -0.562502978
-0.001348594 -0.072331988 0.450367478 -0.225775696
0.110743662 -0.29691237 0.566349089 -0.377184309 -
0.308572296 0.227694385 0.424239472 -0.604039621 -0.400383206
0.203180004 -0.316881133 0.035337756 0.315740617
0.407020744 0.3430396 -0.254683967 0.479788098 0.110401301
0.285264416 0.308243368 -0.424450623 -0.276269745
0.397345186 0.457382275 -0.119314606 -0.263980744
0.130500239 0.124604941 -0.394845463 0.337878079
0.439142928 0.268095365 0.200624033 0.273200823 -
0.047395151 -0.061171468 -0.515433876 -0.575545053 0.118970825
-0.554752515 0.527207425 0.22400825 0.095406267
0.093582661 -0.669517334 0.386125726 -0.080316111 -
0.120609378 0.196272328 -0.227331394 -0.330490216 0.306592502
-0.110526132 0.273077499 0.462614976 -0.461571 0.366537238
-0.16850433 -0.282925546 0.496423631 0.368690473
0.423391584 -0.185872266 -0.284491258 -0.439367493
0.058548584 -0.302563972 0.344229427 -0.413282241
0.52718712 -0.279824704 0.109233857 -0.342499755
0.319335974 0.220446321 0.344512215 -0.060003378 -
0.064916161 0.21796961 0.229515615 0.131374801 -0.065592166

10328 -0.421423642 0.226095002 -0.213824001 0.40750802

10329 0.31284184 0.106523754 0.662129402 0.091784928 -

10330 0.320458728 0.280693542 -0.395982894 0.187684539 -0.186764022

10331 -0.004764697 -0.390684786 0.151251785 -0.23914561 -

10332 0.28832651 0.057826298 0.424463349 -0.058719028 -0.057097714

10333 -0.123726567 0.280246176 0.135958812 0.397261132 -

10334 0.199047807 0.4649834 -0.375099099 0.441052033 0.415496644 -

10335 0.02178556 -0.671808503 0.359852864 -0.490720694 0.658578813

10336 -0.416469869 0.342343645 0.06671687 -0.554384232 -

10337 0.328395887 0.047011790.174224963 0.09113814-0.097048976 -

10338 0.237150665 0.483509367 -0.109152029 -0.377948038 0.29730921

10339 0.263903333 -0.390505205 -0.641012397 0.358961529 -

10340 0.609525298 0.613668496 0.410909583 0.308605978 -0.097819878

10341 0.612536542 0.250971190.122659462 0.369178754 0.032969943

10342 0.489451659 -0.581750621 0.188726522 -0.184194762 -

10343 0.214737497 -0.27388554 -0.314140155 -0.515916443 -0.650917115

10344 -0.719935936 0.209099815 0.392165919 0.283682867 -

10345 0.457882193 -0.199683396 0.358121818 -0.646796905 -0.241487769

10346 -0.334863072 -0.274920732 -0.038990067 0.072647796 -

10347 0.572639346 0.415564788 0.21018439 -0.356467846 -0.308209264

10348 -0.243083945 0.35344443 0.35041314 -0.32112305

10349 0.399517073 0.24405761 0.094723469 0.463433586 -

0.479548743 -0.075716568 0.165665632 -0.048233397 0.273545397
-0.344536618 0.589649284 -0.199363241 0.494376507
0.02367688 0.444328778 -0.37389854 0.242337159
0.502757253 -0.375964732 0.561550438 -0.432883794 -
0.046035544 -0.179646596 0.308025331 0.443884126 -0.168093473
0.209611828 -0.54489388 0.379151676 0.034908807 -
0.115111521 0.122395944 0.289414533 0.684209248 -0.617752721
-0.408501955 0.114757487 0.178281508 0.290876429 -
0.010360521 0.318818357 0.501533716 -0.341458952 0.245496681
-0.211019634 -0.338479542 -0.234533409 -0.322903688 -
0.600226764 0.585342171 0.139541275 -0.56464738 0.060382675
-0.049581093 -0.511930967 -0.529432613 0.213448096 -
0.525570577 0.253542308 0.107055934 0.22399722 0.452787358
-0.36479668 -0.535597794 -0.52608396 -0.187794373 -
0.336746882 0.416045717 0.169427647 -0.338539477 0.24069635
-0.42834115 0.409519774 -0.47711986 0.082066482 -
0.139387097 0.252677351 -0.086002885 0.383924181 0.354923267
-0.489061361 -0.339344109 -0.249990354 0.346314263
0.542815079 0.488216465 -0.524148462 -0.449652902 -
0.042518803 0.244223539 -0.158617306 0.706650999 0.446641056
0.449000622 0.485373016 -0.047042615 -0.62293955 -
0.288284596 0.256053679 0.044443116 -0.243755688 -0.214346267

0.395422251 0.369462114 0.428454858 0.433355995
0.519651977 -0.231476424 0.448989134 0.528733642
0.194872262 -0.690875423 -0.3353449 0.032694674 -0.212557815
0.50664228 -0.234683892 0.036809149 -0.145952703 -
0.435420671 0.162963339 0.321326999 0.499311366 0.338283462
-0.239735386 0.408861077 0.066928327 0.552671538 -
0.3325711 -0.373819018 0.547055327 -0.200931023 -0.401332967
0.421756039 -0.124679988 0.245381575 -0.353794996
0.045268258 0.032558592 0.052731667 0.03255093
0.057909546 0.483879592 0.118918773 0.2010068 -0.258581572
-0.443020924 0.651874173 -0.530805723 -0.426673484
0.529467307 0.020038733 0.265094687 -0.054983445 -
0.561950981 0.515608134 0.65786599 0.384314373 -0.611412309
-0.044879629 -0.222017926 -0.184706961 -0.190840444
0.561253574 -0.113052886 0.571600373 -0.573792737 -
0.139762189 0.26353974 0.249146623 0.600529233 -0.256979589
-0.217579257 0.124856336 -0.160711886 0.325567343
KEGG_FATTY_ACID_METABOLISM 0.576973514 0.583985694 -
0.564912099 0.087110643 -0.001354562 0.117655610.304545767 -
0.366844925 -0.444356301 -0.405394415 -0.064216518 0.340378743
-0.480923178 -0.348757826 -0.201413815 -0.389725676
0.041885854 0.292178399 -0.613123293 0.018294188 -

10394 0.327266505 0.303335687 -0.673273688 -0.153640686 -0.366614021
10395 0.065620613 0.347187553 0.334200321 -0.361974497 -
10396 0.37803297 0.54144333 -0.196148861 0.409930153 0.34152251
10397 -0.401208859 -0.362830274 0.099053932 -0.031386741 -
10398 0.402772166 0.488137012 0.262638529 0.281565359 -0.323670286
10399 0.637093732 -0.227023019 0.221232885 0.257827299
10400 0.242961356 -0.224552176 -0.111389848 0.326249307 -
10401 0.280599945 0.219334162 -0.363752883 0.347176086 0.381094592
10402 -0.416022699 0.272756756 0.507907808 0.160631135 -
10403 0.611501687 0.008875025 0.236929153 0.273741682 -0.193537165
10404 -0.342158911 -0.23175185 0.431178124 -0.285092267 -
10405 0.282407827 -0.304221119 -0.440109281 0.439064783 -0.392685753
10406 -0.178965796 -0.229830176 0.423992484 0.390378203
10407 0.155512301 0.653540259 0.012205498 -0.182468256
10408 0.378355676 -0.132508184 -0.220527116 -0.452589465 -
10409 0.508116903 -0.306304421 -0.35993416 0.577946119 -0.488923375
10410 -0.279376488 0.267264543 0.398802033 0.49533883
10411 0.246082110.30839881 -0.57404138 0.45179931 0.25767324
10412 0.310251993 -0.243469467 0.197509638 0.27197901 -
10413 0.378729633 -0.430950813 0.28460581 0.4286044 -0.43417907 -
10414 0.090695235 0.040374203 -0.173322755 0.461902912 -0.558794143
10415 0.265456321 0.19489238 0.237373167 -0.144133284 -

10416 0.196435122 0.202148248 0.053912581 0.539296583 -0.027321814
10417 0.237517583 0.045993725 -0.110788402 0.272119975 -
10418 0.473508396 0.454668928 0.342422313 0.276705749 -0.441492703
10419 -0.050721762 0.335420559 0.041623638 -0.524941801 -
10420 0.563519618 0.540484936 0.251837431 -0.48934407 0.055995156
10421 0.287699498 0.347944991 0.140434627 -0.498854167 -
10422 0.333271484 0.413764824 0.596375438 -0.420468245 0.016158674
10423 -0.391942248 -0.355915454 -0.49929839 -0.214814929
10424 0.262568305 -0.025309667 -0.517117335 0.489726454
10425 0.330332824 -0.39077305 -0.144553512 0.141349631 -
10426 0.21444267 -0.523088938 0.529335501 -0.561206356 0.335093228
10427 -0.112846879 -0.101661157 -0.347834628 0.198718132
10428 0.403033253 0.178048515 0.286900962 -0.36264315 -
10429 0.341279221 -0.315440251 0.576294885 -0.153676634 -0.389180299
10430 0.124873633 0.274084078 0.54225757 0.084164151 -
10431 0.091612525 0.222238916 -0.193636865 -0.049806292 -0.542143256
10432 0.440828688 -0.474860317 0.553899264 0.602315472 -
10433 0.244547527 0.256303085 0.173967221 0.056972352 0.158110776
10434 -0.102450856 0.28831168-0.341335331 0.326928497 -0.152904028
10435 0.574203836 -0.190270313 -0.014679285 -0.452161771
10436 0.163487016 -0.266571985 0.309553557 0.676870517 -
10437 0.254983701 0.258080788 -0.484045732 0.035679771 0.06858421

0.15097009 -0.195415035 0.344103535 -0.415861017 -
0.477606137 0.647920921 0.073040019 -0.36367564 -0.475779729
-0.054741543 -0.377488281 -0.059308778 0.204441749
0.430903939 0.033825322 -0.384092241 0.404297172 -
0.357071472 0.266394896 0.453003548 -0.551575982 0.235990827
0.380412486 -0.019239934 0.259847269 -0.145026457
0.116154587 -0.303040866 -0.381409913 0.099215019
0.105787153 -0.187433336 0.234349852 -0.022819973
0.145806166 0.096326393 -0.411510484 -0.603820557
0.133924441 -0.4245573 0.55870203 -0.160836552 0.510629282
0.264090639 -0.581944065 0.334529484 0.215957418 -
0.267525423 0.375497633 -0.471039627 -0.54143733 -0.178435154
0.371484444 -0.169776695 0.555522413 -0.468843485
0.14704557 -0.156269566 0.229214599 0.482976596
0.324621384 0.19280632 -0.072226908 -0.268581489 -
0.514380861 -0.257433181 -0.534455967 -0.363862204 -0.50791152
0.262338158 -0.528645326 -0.08757325 -0.392507289
0.217137623 0.303281617 0.383997559 0.29398734 -
0.39815837 -0.517411047 -0.365968997 0.419252232 0.461210294
-0.465866673 0.23337201 0.370273735 0.345072069
0.073896985 -0.235352684 0.573434634 -0.353371672 -
0.31267863 0.557177466 -0.593121162 -0.26599129 -0.464216363

10460 -0.201822726 -0.412233129 0.329440768 0.022624692

10461 0.326173647 -0.307205234 0.265701604 0.567313944

10462 0.422310681 0.203430684 0.520908419 0.409873274 -

10463 0.154764695 -0.378797925 -0.066389972 0.206004503 0.336687055

10464 0.449552466 0.075994027 -0.49686321 0.499674229 -

10465 0.579828384 0.565816928 -0.194867244 0.449446063 0.471693913

10466 -0.542883854 0.255541497 0.406208514 0.46301424

10467 0.23338847 -0.259272898 -0.13292203 0.532050196

10468 0.179811072 -0.346217319 0.471733327 -0.071227704 -

10469 0.523111871 -0.411762511 -0.293485785 -0.370575957 0.5900674

10470 0.409649313 -0.077922 -0.034573159 0.463447903 0.401841371

10471 0.216929394 0.315263339 0.358158862 0.515768783 -

10472 0.502847029 0.045273112 -0.123566393 -0.275065936 -0.036657008

10473 -0.298524476 -0.439238316 -0.382129997 -0.4823405 0.334111951

10474 0.14161427 0.434937308 -0.4796411 -0.590429581 0.282960594

10475 -0.457012093 0.177540134 -0.373370976 -0.52997284

10476 0.236039405 -0.247899395 -0.272644965 0.508428467 -

10477 0.026833673 -0.229771208 -0.082921267 -0.26565228 0.394949686

10478 0.248641459 -0.330400679 0.464748432 -0.33769959

10479 0.336313265 0.466073988 -0.466167404 -0.395209182

10480 0.344130342 -0.157049572 0.04668074 -0.113442618

10481 0.568701933 -0.325727578 0.443144213 0.090803151

10482 0.029591121 -0.286554566 0.174385826 0.588707377 -

10483 0.038190107 0.490947775 -0.504797885 -0.024658583 -0.1592939

10484 0.385022439 0.488607298 0.030442515 -0.458388581 -

10485 0.467584836 0.363863914 0.310527273 -0.22463171 -0.140901611

10486 0.034700705 0.452391516 0.024973782 -0.564228455

10487 0.007719916 -0.023434041 0.461574248 -0.499936306

10488 0.251662327 0.141017522 -0.516470251 0.269836493

10489 0.431521352 -0.347448623 -0.141723856 0.124883164 -

10490 0.393273158 0.242372445 -0.32210877 -0.481668897 0.283427733

10491 0.327854063 -0.585482174 -0.465543163 0.431110039 -

10492 0.454708899 0.017896328 0.236292901 0.259862511 0.359914268

10493 -0.371017472 -0.445553681 -0.569751301 0.131212087 -

10494 0.377186427 0.4972291 -0.139472337 -0.522169804 -0.033080824 -

10495 0.252182588 0.027709513 -0.325364773 -0.363750857 0.273006821

10496 -0.221256397 0.001112369 0.377752563 0.229957474 -

10497 0.442225378 -0.518528735 0.113596976 0.294116688 0.420783226

10498 0.125801599 -0.371739611 -0.222747343 -0.180035989 0.4955611

10499 0.372342379 0.371638391 0.520559854 0.054910226

10500 0.121311129 -0.339963259 -0.389422073 -0.241226544 -

10501 0.262110447 0.095254067 -0.294821683 -0.000313448 -0.087305708

10502 0.159980797 0.147980535 0.14599064 0.443097574

10503 0.057138399 0.517526867 0.493429623 -0.281352194 -

0.366097592 -0.190372689 0.127583779 0.01736981 0.36474843
0.034215232 0.113072149 -0.154903268 -0.464342801 -
0.393152742 0.614258299 0.342881968 0.072577775 -0.181689055
0.30702175 0.300842804 0.467119265 -0.439587371 -
0.123030457 0.493562153 -0.194686604 -0.483882578 -0.067469427
-0.213565409 0.181070306 -0.408274296 0.148731623
0.121180176 -0.017883029 0.166176787 -0.181251006
0.117548972 -0.258144606 0.30439083 -0.339409379 -
0.491399635 0.41569853 -0.300031042 -0.336620858 0.264416138
-0.262381367 0.27890363 -0.133201462 -0.277855619
0.182773648 0.419961991 -0.046336696 -0.088806758 -
0.057034379 -0.363322062 0.140898843 -0.311435417 0.358150533
-0.425908052 0.320142009 -0.421883528 -0.01322049 -
0.075688483 0.261695523 0.137207014 -0.4118234 0.243297304
0.170511493 -0.360952615 0.365686589
KEGG_STEROID_BIOSYNTHESIS 0.31680541 0.149033674 -
0.188871976 -0.534375015 -0.12320951 -0.0665517 -0.070268849
0.180006282 0.140706083 -0.311784124 -0.479304702
0.597285142 -0.694477889 -0.330412625 -0.333304829
0.341385977 -0.065126104 0.453130893 -0.448796563 -
0.408257594 -0.262450306 -0.288732147 -0.373809299 -0.128879754
-0.028951148 -0.034752398 -0.170096966 -0.081302889

0.142242751 -0.074843642 0.032257003 -0.148077357
0.059186142 -0.210744373 -0.06333583 -0.450722479 -
0.259821559 0.114219148 -0.432886743 0.118840346 0.615734899
-0.032463405 -0.255508525 -0.029148697 0.490729096 -
0.09353772 -0.293230011 0.572283394 0.165322846 -0.187920777
0.653250915 -0.40470107 0.365733157 -0.434575752
0.169813231 0.054672867 0.181023774 0.075976661
0.117640755 -0.509643152 -0.590225481 0.486049276 -
0.157057157 0.262197099 0.073652596 -0.000698942 -0.601976802
0.391544937 0.069674025 -0.020507545 -0.021974153 -
0.421847705 0.248151671 0.164880325 0.510982469 -0.181295677
-0.484676923 0.331166790.347188076 0.38202899 0.089115074
-0.219912714 0.137484805 -0.214957874 0.391268865 -
0.007210186 -0.245198272 -0.169509794 -0.496355507 -0.017261509
0.514277753 -0.488494433 -0.127838881 -0.028943551
0.181349866 -0.28435054 0.132406893 -0.327101542
0.320082025 0.499911032 -0.420275662 -0.364150323 -
0.367138335 0.271778408 0.158139631 0.282719792 -0.181976676
0.239887408 -0.536722864 0.089325755 -0.384711196
0.357552951 0.547490449 -0.159472886 -0.339130336 -
0.159902862 0.266842162 -0.147471559 -0.408052496 0.123310064
-0.281855773 0.339587345 0.177134481 0.536341505 -

0.352286091 0.144413641 0.357274867 -0.161358054 -0.087117651
0.030090005 0.310953937 -0.414430138 -0.101773861 -
0.207761164 -0.302109273 0.15031492 -0.279033495 0.33757295
-0.59844186 -0.040363524 0.143033986 0.3204937 0.139468053
-0.315697139 0.011717348 0.134276285 -0.100717317
0.114222561 -0.258674934 0.222550983 -0.036922566 -
0.234560333 -0.104948014 0.168183188 -0.352822245 -0.260234213
-0.127179022 -0.1778022 0.273783437 -0.147191727 -0.210824171
-0.076546926 -0.024316568 -0.459173527 -0.010896994
0.067816607 0.04583223 -0.054531373 0.045243642 -
0.421610997 -0.066792092 0.071917507 -0.120511779 0.151024368
0.048108667 -0.061212693 -0.283998569 -0.48618274
0.052005284 -0.026352709 0.197553273 0.188239173 -
0.153877169 -0.239980872 -0.483356365 0.123890203 -0.494780488
-0.429729644 -0.328669609 0.14808502 -0.071920891 -
0.055581334 -0.13016309 -0.275401001 -0.081336795 -0.047336567
0.088790199 -0.244487049 -0.157384544 -0.12343813
0.126638335 0.115496197 -0.249546594 0.680666885 -
0.042516755 0.11846554 0.246252675 0.327623349 -0.490472085 -
0.042198955 0.282076837 0.112296428 -0.053817135 -0.485916965
0.389203815 0.098617577 -0.067490729 0.055896317
0.225625646 -0.3842331 -0.249581449 0.266715022 -0.158000075

0.198316237 -0.489388067 -0.045685859 0.181895767 -
0.349325826 -0.026857488 -0.104342713 0.05688973 -0.131764378
-0.428147843 0.031689219 -0.061710379 0.123852677
0.231592771 -0.443088936 -0.509925843 0.019637119
0.185289433 -0.409717208 -0.20174803 0.226319852
0.536287186 0.000530611 0.364857651 -0.096273336 -
0.402250153 -0.016331413 -0.072173038 -0.320451424 0.090403025
-0.583969127 0.298261954 -0.306539824 0.0200143 -0.0865005
0.082091381 -0.058455448 -0.250707037 0.255324997 -
0.296034703 -0.033580272 0.145695565 -0.29237796 -0.286411879
0.146868467 0.008221697 0.187242405 -0.021625421
0.273590386 -0.211067844 0.072659634 0.215292423 -
0.236524384 -0.148807313 0.067396128 -0.528251307 -0.240482297
-0.360209932 0.37439699 0.389271798 -0.327000088 -
0.203224747 0.22947266 -0.078556651 -0.282301449 -0.245098104
-0.311020376 -0.239542618 0.244731128 -0.37496584 -
0.025669666 -0.446707026 -0.487546174 -0.189877779 -0.161641921
-0.255282697 -0.143166004 -0.293517458 0.410607574 -
0.150202115 0.329617078 -0.044274701 0.080112242 -0.199119335
0.148909188 -0.175260775 0.093734891 0.101441655
0.244104322 -0.258571405 -0.483814856 0.494659819
0.523559388 -0.326836993 0.416725717 0.2168555 -0.266634768

0.47999381 -0.219500992 0.429079779 -0.057293476
0.010615938 0.334884141 -0.005941181 -0.170320951 -
0.217651522 0.039091499 -0.346862368 0.08621939 0.009118644
0.269612115 -0.593275325 -0.048298523 0.064899178
0.205279132 -0.15222497 -0.367086541 0.093444564 -
0.194785072 -0.503908468 -0.254074345 0.077158603 0.042438977
-0.216262447 0.293305864 0.154199816 -0.05045488 -
0.284777607 -0.06329676 -0.324787723 0.231533941 0.259368954
-0.227943485 -0.464756157 -0.307919152 0.182590109
0.666809864 -0.134549105 0.215488254 -0.238308228 -
0.201030941 0.069336468 0.078513553 -0.293280947 -0.370634775
-0.388294298 -0.394456461 0.011548716 -0.07234452 -
0.401081575 0.291782578 0.199780139 -0.014296813 -0.127018463
0.379246124 -0.302059802 -0.459160436 -0.464050274
0.012857566 -0.228344988 -0.396409272 0.256365081
0.070033114 -0.110865214 -0.198931934 -0.153029948 -
0.119154275 0.237965111 0.011480887 0.219524497 -0.095323871
-0.234449976 0.229469276 0.194184695 0.119653164 -
0.248436254 -0.194026551 -0.447787179 -0.299265426 -0.492439929
0.471266138 0.202552372 -0.029017171 0.024413237 -
0.182630612 -0.037542199 -0.169081221 0.001880703 -0.262844083
0.019422542 -0.153605658 -0.401451586 -0.72051573 -

0.086608871 0.338416928 -0.04748611 -0.237406764 -0.287426108
-0.061517458 0.417687809 -0.172991907 -0.428154147
0.235618563 0.110571473 -0.396898865 0.07031931
0.00543985 -0.06708782 -0.014693873 0.106842756 -
0.169223549 0.305765974 -0.318016071 -0.140725247 0.571438384
-0.074822143 -0.070668671 -0.194872622 0.01634591
0.073409247 -0.443953169 0.277796538 -0.398257002
0.561860448 -0.335687676 -0.208015024 -0.141754518 -
0.222997399 -0.186189066 0.203331163 0.145870082 0.128432901
-0.186406679 0.083364732 -0.304045395 -0.290482807
0.208272686 0.230675302 -0.291275194 -0.2460447 -0.1283926 -
0.364017212 0.20184733 -0.204001996 -0.295167464 -0.037184059
-0.424098529 -0.275407777 0.273997232 0.340671752 -
0.464970301 0.057938253 0.107138592 -0.306178731 0.232298976
0.001004056 -0.199623068 0.006159002 0.312391755
0.103521807 0.766094287 -0.327754031 0.10645607
0.045009487 0.295432146 0.048586769 -0.179584871
0.305547929 -0.484492968 -0.080622453 -0.255277739 -
0.331906665 0.205060861 0.246216785 -0.306140177 0.325475011
0.248982791 0.512166929 0.137509613 0.114073938 -
0.329618238 0.057542785 -0.11313234 0.502695418 0.153320054
0.28231096 -0.31124016 -0.428917572 0.124470584

0.139543129 0.112186083 0.190755536 0.187601543
0.064648782 0.152904602 -0.176370848 -0.208704972
0.32090808 -0.127036079 0.014838139 -0.124726264
0.459250921 0.192416737 -0.032464032 0.104728594 -
0.092977456 -0.481209597 -0.129585286 0.392807984 0.073933284
-0.203392942 0.464327971 -0.460648104 -0.15262188
0.101982523 0.04524372 0.300802882 -0.028067919
0.400127904 -0.124701155 0.084114609 -0.070276673 -
0.437785281 -0.136465312 0.410321901 -0.303930287 0.10456424
0.077352307 0.302374862 0.258972004 -0.013216971
0.304008596 0.423045345 0.048659881 0.080973701 -
0.399171484 -0.062592959 0.292258621 -0.452216336 0.158529796
-0.455368922 -0.023991597 0.025250273 -0.101163666
0.061113191 0.053227329
KEGG_PRIMARY_BILE_ACID_BIOSYNTHESIS 0.489038962
0.266390609 -0.32123746 -0.211824895 0.593527075
0.226868726 0.410295283 0.255670184 -0.218211933 -
0.093399372 0.087437862 -0.10025568 0.030035094 -0.291483399
0.116211603 -0.074364661 -0.163792251 0.090885178 -
0.167458852 -0.423292673 -0.401354948 0.194562684 -0.622666301
-0.428016497 0.07471242 -0.194734091 0.078856277
0.515123468 -0.233044164 0.289913799 -0.040819358 -

10658 0.030760604 0.134278529 0.199651786 -0.098421575 0.061957635

10659 0.48157246 0.138809688 -0.165290772 0.043111144 -

10660 0.057823196 0.449815644 -0.38732942 0.344876143 -0.338413089

10661 0.083836691 -0.157655798 -0.147477479 0.480050011

10662 0.249555937 0.107800133 -0.487164671 0.16937886 -

10663 0.381152538 0.007352591 0.248713346 0.088862778 0.380525356

10664 0.18863218 0.155896804 -0.31140627 0.123696507 -

10665 0.23585926 -0.219320049 -0.134532843 -0.267021677 -0.29147356

10666 0.31895323 0.148783674 0.157430541 -0.59602774 -

10667 0.44372628 -0.091293191 0.140616463 0.088348909 0.505532144

10668 -0.178060673 -0.132006545 0.083417664 0.603437054

10669 0.034030794 0.206510156 0.001524058 0.151360462

10670 0.058890233 -0.49805533 -0.424897224 -0.540649206 -

10671 0.340181014 0.172655383 -0.447818245 0.069481091 0.281219124

10672 -0.008489641 -0.020201649 -0.273765716 -0.144717191 -

10673 0.094935492 0.18195286 0.341905112 -0.020995087 0.165276899

10674 0.087886046 0.283082567 -0.035695471 -0.285608607 -

10675 0.038795042 0.518923484 -0.245471996 0.220931981 0.312423544

10676 0.449135466 0.314177877 -0.423818912 0.156045649 -

10677 0.3076276 -0.054819739 0.165785156 -0.054167895 0.094052942

10678 0.32106054 0.436668693 -0.045498131 0.181584841 -

10679 0.104088175 0.423296958 -0.039593685 -0.338726745 -0.13773007

10680	0.098431514	-0.220115313	-0.374790763	0.165301313	-
10681	0.284787152	-0.237419617	-0.308839208	-0.124091754	0.173538479
10682	-0.353975061	-0.080133064	0.383162251	0.425895413	-
10683	0.210104186	-0.190172397	-0.478097725	-0.536952042	-0.041095698
10684	0.152864972	0.299662978	0.249933154	0.31626055	-
10685	0.314372929	-0.494037724	0.439802965	-0.004903098	0.107085306
10686	-0.081732332	0.09605294	0.134533477	-0.022776775	-
10687	0.440544806	0.225360947	-0.581764056	-0.166189435	0.314174897
10688	-0.285601068	0.415493435	0.367854065	0.055053701	
10689	0.003099963	-0.232473918	0.191578166	0.266096541	
10690	0.415731954	-0.068320537	-0.533254807	-0.446447093	
10691	0.261416612	-0.093100646	-0.389470048	0.480523868	-
10692	0.090073421	-0.11501538	0.354940665	-0.371706802	0.441490793
10693	-0.489577734	-0.015009214	-0.175630849	0.04323593	-
10694	0.238566324	0.377765613	0.015926059	0.040455033	0.070202469
10695	0.288695274	0.081430511	-0.146009757	-0.252246132	-
10696	0.105246746	0.220700895	0.241311751	0.202800964	0.79966667
10697	0.172042598	0.272839699	0.323043435	-0.116091031	-
10698	0.18288407	0.411925849	0.581030177	-0.155926766	0.220830468
10699	-0.459789544	0.513783419	-0.362830976	0.00695275	
10700	0.228013513	-0.428864594	-0.397328596	0.161727978	
10701	0.49509584	0.401501771	-0.501334404	0.075062727	

0.150636891 0.053053596 0.273086523 0.005449621
0.25242663 0.539716805 0.094124453 0.218555172
0.267697275 -0.006381159 0.134193757 -0.470209584 -
0.183996181 -0.447383697 -0.013591416 -0.051619865 -0.533655019
-0.134616559 -0.01934831 -0.524909168 0.276467938
0.398087561 -0.067883156 0.04373333 0.061462228 -
0.103340657 -0.060695474 0.086211811 -0.529426117 -0.04047722
-0.254336437 0.262833525 0.179353331 0.138753573 -
0.119843268 -0.51188725 0.055703626 0.124686701 0.129401341
0.362824892 -0.469100336 -0.232082775 0.312578647
0.186772254 0.419480141 0.348860114 -0.557020481
0.031279164 0.149225316 0.018603901 -0.030415931 -
0.014992492 0.295592229 -0.378707059 -0.208841432 -0.202187455
0.077505962 -0.609043543 0.154935891 -0.012462614 -
0.05933676 -0.210040432 -0.30665232 -0.145947264 -0.041663824
-0.026300565 0.33672888 -0.302689623 -0.116503734
0.296825544 -0.236955347 -0.004397824 -0.234719307 -
0.384027568 0.297721568 -0.136277119 0.368550778 0.3042605
0.361109164 0.203806642 0.127125141 -0.322951446
0.348703793 -0.080655307 0.205118868 -0.419612321
0.440361419 -0.188713316 -0.287194311 0.571540185
0.259055437 -0.033676803 0.181937199 -0.083341048 -

10724 0.034553482 0.092121756 -0.085447446 0.105157109 0.370000644

10725 0.09265725 0.230616808 -0.182552825 0.280105202

10726 0.099001219 0.108782256 -0.140023281 0.144950914 -

10727 0.238547461 0.325646778 -0.491045567 0.188107110.025214874 -

10728 0.355003137 -0.435896273 0.189068787 0.140742964 -0.052442791

10729 -0.142032628 -0.365403749 0.028472811 0.241707501 -

10730 0.206431442 0.4583209 -0.010040786 -0.355818425 -0.136897333 -

10731 0.297545636 -0.25847506 0.541902805 0.404969934 0.161359611

10732 0.225162716 0.322075191 0.02738857 0.270035075

10733 0.088922513 -0.024609989 0.109715448 -0.205024894

10734 0.018062878 -0.220083896 -0.342060755 -0.190031423 -

10735 0.235446017 -0.295042352 -0.44885501 -0.529990668 -0.27854203

10736 0.006195591 -0.368178054 -0.111455088 -0.220725986

10737 0.101437794 -0.4453512 0.176829477 -0.184548924 0.116474074

10738 0.043432839 -0.467366532 -0.067353075 0.074236033

10739 0.181222679 0.042618122 -0.399156813 -0.414388886

10740 0.271415775 0.287592996 0.212547634 0.16023531 -

10741 0.461862549 -0.099837656 0.16462512 -0.065606631 -0.143105345

10742 0.160036201 -0.21449714 0.061296646 -0.453254087

10743 0.246390532 0.502455009 -0.252338358 -0.156323951 -

10744 0.048388999 -0.418508976 -0.147770045 0.434800607 -0.286878679

10745 0.483741607 0.15525091 0.136002403 -0.37127213

0.110256593 -0.058735773 0.03072792 0.164608522 -
0.414560027 0.293201140.266174918 0.03457761 0.396096004
0.136236313 0.295165062 -0.214750161 -0.480859426 -
0.384376699 0.359150962 0.293463705 -0.136471434 0.394654717
0.394370857 -0.419279624 -0.127654963 -0.033082584 -
0.488610228 -0.337683 -0.418640772 -0.266189301 0.415020266
0.002039672 -0.058701819 0.069589542 -0.043945066 -
0.395698591 -0.436587725 -0.061853555 -0.42470078 -0.103359688
0.468342181 0.468010605 0.443414294 -0.125000715 -
0.371405926 -0.298102679 0.190946727 -0.086555441 0.266944315
-0.421848464 -0.229989308 0.125311858 -0.118675933
0.216109148 -0.136067209 -0.263382751 0.198029062 -
0.480823324 0.017121232 0.344096414 0.367189711 -0.436962275
-0.42577227 0.003756582 0.126991965 0.432788781 -
0.390644427 -0.188394102 -0.444749882 -0.048234763 -0.02392059
0.038437905 0.18677247 0.28524199 0.074883411
0.299230314 -0.288728563 -0.471332237 -0.140924448
0.027037457 0.039204885 -0.428124284 -0.017821893 -
0.089564663 0.600473062 -0.003498072 -0.328361316 0.284372767
0.314450093 -0.077278009 0.442309547 -0.191472403 -
0.454265309 -0.02531833 0.081751668 -0.267576844 0.503329261
-0.475418695 0.071945998 0.284555202 -0.359455612 -

0.170874372 0.025871248 0.466978107 0.498000555 -0.041551136
-0.017726337 0.010389747 0.483868439 -0.486836943 -
0.073470327 0.390521896 -0.058356949 -0.313987444 -0.121569344
0.233771326 -0.059001613 -0.232507234 0.483917919
0.243922617 -0.119688839 -0.498562405 -0.189111352
0.135884684 0.030916168 0.078545944 -0.425768432 -
0.543908817 0.326533267 -0.246967239 0.385540298 0.015387086
-0.542702171 0.449958373 -0.407399641 -0.480783056
0.014368668 0.432487847 0.303162449 -0.01331183
0.125903412 -0.371085435 -0.38967574 -0.223670235 -
0.068043214 -0.430866819 -0.146775673 -0.100657279 0.070213315
0.305638283 -0.357104005 -0.070966222 0.016098474
0.202461914 0.013159103 0.322721866 0.513983543
KEGG_STEROID_HORMONE_BIOSYNTHESIS -0.116113696 -
0.290128549 -0.192799268 -0.394742485 0.440468049 0.308622422
0.168499749 0.331774499 -0.463073483 0.261453547
0.207305628 0.10580141 0.268568487 -0.292579035
0.254947967 0.365751058 -0.380360584 -0.1647587 0.259727446
-0.289035713 -0.321741758 0.127331693 -0.490510269 -
0.521564167 -0.028223859 0.382743414 0.41728733 0.003675375
0.190164347 0.44163519 0.227748458 -0.043674474
0.168602796 0.194973484 -0.289030294 0.069699749

10790 0.272834983 0.288460802 -0.077470527 -0.120527146

10791 0.275403237 0.2106623 -0.323588355 0.286967348 -0.401683814

10792 -0.190901928 -0.31727565 0.211430716 0.19328482

10793 0.24495564 0.119190684 -0.191373844 -0.357203525 -

10794 0.336934736 -0.230684822 0.056872022 0.252837663 0.070183529

10795 0.122496967 -0.33903007 -0.507283016 -0.17295251 -

10796 0.152068182 -0.458273065 0.132048555 -0.104016335 -0.081641459

10797 -0.10107624 0.48049761 0.341292703 -0.516074099 -

10798 0.327240565 0.263080677 0.268690871 0.280110614 0.408779187

10799 0.198880049 0.042999105 0.249926774 0.331091046

10800 0.238946404 -0.384853197 0.062815534 0.142776621 -

10801 0.068413074 -0.366287931 -0.511012276 -0.08858828 -0.154228951

10802 0.263599374 -0.130205558 0.268350145 0.007021564 -

10803 0.087457353 -0.357600455 -0.302556467 0.101402338 0.059310024

10804 0.189837796 0.322357988 -0.055998603 0.458024123

10805 0.091676507 0.063520948 0.172340929 -0.303025644

10806 0.259651264 0.475628879 -0.490152494 0.324512016

10807 0.413738411 0.340071975 0.25596708 -0.332005459

10808 0.173965527 -0.303927 0.250266507 0.367407138 0.426637446

10809 -0.011960255 0.438938197 0.321318534 -0.105215187

10810 0.321150767 0.186021746 0.558658719 0.247691898 -

10811 0.410144753 -0.302500898 -0.378850389 -0.041714749 0.123105973

-0.029860961 -0.154338382 -0.017370647 -0.491658803 -
0.421059371 -0.055003654 -0.370224978 -0.412309157 0.536194449
0.361165169 -0.330577464 0.198510032 -0.512671172 -
0.257858079 0.079650622 0.359861663 0.393685485 0.368588597
0.160026511 -0.482193786 -0.365171648 0.319687536 -
0.202359516 0.501179403 -0.271605159 0.326617393 -0.322857942
0.119542321 -0.249359961 0.370078504 -0.018263926 -
0.069642438 0.120260674 -0.029533635 0.342264222 -0.023927447
0.538649959 0.174960618 -0.453996429 0.342533031
0.334561695 0.205970525 -0.416786101 -0.498064716 -
0.264610577 0.019293752 -0.341263633 -0.276765226 0.194013889
0.11286025-0.146566893 -0.124049949 -0.429785654 0.040337083
-0.449492691 0.153796556 0.032471726 0.180155639
0.079506667 0.444038614 0.270299968 0.198468302 -
0.028131675 0.198539393 0.185500362 -0.203499181 0.365433504
-0.106962162 -0.026559433 0.078238064 0.14827237 0.648903
-0.01505599 0.064136014 0.158881941 -0.066649382 -
0.255118428 0.17956466 0.114693669 -0.320024309 0.000658712
-0.06760833 0.521655497 -0.513840161 -0.154747848 -
0.0538368 0.238783821 -0.33557321 -0.043854532 -0.187489275
0.331228119 -0.480138562 -0.128798282 0.363740954
0.03111196 0.366021191 -0.265554611 0.253448291 0.415990375

-0.362451928 0.10873778 0.119790653 0.150904436 -
0.082183668 -0.04253217 -0.315935705 -0.126496636 -0.021887072
-0.084802264 -0.35133069 -0.063280132 -0.001456372 -
0.134000022 -0.020789863 0.302496647 0.228717795 0.264178062
0.328226683 -0.199962038 -0.295492042 0.181370941 -
0.393767788 0.278808618 -0.304245232 0.158615763 0.434620541
-0.175045834 0.076723462 -0.217743383 -0.019738272
0.099316899 0.370361011 0.138166132 -0.212576065 -
0.138672025 0.446385965 -0.302142122 0.548176951 0.326520537
-0.413455893 -0.091665984 0.258318991 -0.234086956 -
0.065822626 0.06686026 0.158207516 0.010392725 -0.176984994
0.21908501 0.189018728 -0.292863941 0.388858149
0.155652352 0.384889948 0.263427556 -0.037049781
0.006358039 0.409262009 -0.022855405 0.34883821 -
0.231113626 -0.189564433 0.339549791 0.187391957 -0.127814691
-0.096892207 -0.40238358 0.300073146 -0.413072626
0.240087827 0.239626034 0.339641735 0.347369404
0.011587364 -0.173611871 -0.175017755 -0.206544091
0.244570445 -0.073664386 0.143165786 -0.045882145 -
0.29810081 0.270766808 -0.272087853 0.31620079 0.041040263
-0.280071426 -0.363514491 0.411971684 -0.185374742 -
0.284382353 0.489644768 -0.002077804 0.24800101 -0.298998932

0.446914772 0.118866373 0.165803598 -0.18565941 -
0.120099131 -0.241901382 0.269260803 -0.485594324 -0.172125333
-0.413251324 -0.396139556 -0.402428482 -0.33725171 -
0.044925585 -0.20702133 0.193875509 -0.090804814 0.10237821
0.445953094 -0.316638879 -0.017727734 0.315033036 -
0.314957038 -0.35205733 -0.014780755 -0.512842957 0.560630114
-0.01036148 0.251797945 0.381233129 0.173135831
0.107076975 0.495065834 0.00030386 0.180396863
0.046954388 -0.041877341 0.003634369 0.094901062 -
0.06979716 -0.406258192 -0.145133529 -0.3796292 -0.241954075 -
0.648799033 -0.262174734 -0.011386836 -0.016097948 0.165582066
0.2029675 0.235697031 -0.422653225 -0.191469106 0.006785798
0.286681894 0.025674041 -0.099626902 -0.460034698 -
0.102701343 0.175386169 -0.150251107 -0.450390339 0.015495137
0.13817123 -0.095552836 0.281416516 0.192476298 -
0.209323053 -0.195188493 0.161417454 -0.114169063 0.085536955
-0.177181651 0.1797895 -0.199315214 -0.469152999 0.396411704
0.272459813 0.186975136 -0.032413855 -0.045361484 -
0.095752288 -0.150211742 0.289923616 -0.372616103 0.485152002
-0.016589823 0.183849015 -0.24584054 0.189216312
0.284459787 -0.206334627 -0.044182934 -0.342526415
0.184865873 -0.135828112 -0.454768276 0.199811262

10878 0.07854857 0.537749774 -0.509938185 -0.35321706 -
10879 0.247944571 0.193524602 -0.075947907 -0.003785607 0.457685808
10880 0.365853448 -0.345315373 -0.186740322 0.104774976 -
10881 0.316465588 -0.272626957 -0.332223321 -0.510224449 0.182220487
10882 0.412886311 0.094452181 -0.073189336 0.212564316
10883 0.043844826 -0.161626128 0.183652953 -0.370182114
10884 0.262700039 0.285184497 0.336071231 0.352948936 -
10885 0.165143441 -0.392545749 -0.251593646 -0.036697386 -0.194019161
10886 -0.000545263 -0.063448723 0.049292504 0.131630588 -
10887 0.386996571 0.192036514 -0.203166819 0.255793944 -0.170808004
10888 0.022302378 0.258250158 0.293459452 0.138155643 -
10889 0.357882229 0.029230887 -0.209583254 0.287936728 0.350604022
10890 -0.03204539 -0.18453129 -0.455462562 0.193810502 -
10891 0.024359436 0.340549684 0.478264747 -0.073965033 -0.005729332
10892 0.381873727 0.183661485 -0.460397661 0.265901764
10893 0.448373523 -0.085500467 -0.377507412 -0.074559176
10894 0.290737007 0.464924589 0.077962072 0.324277947
10895 0.229232542 -0.092586525 -0.07124032 0.130316674 -
10896 0.086969645 -0.489953508 -0.167955116 0.143700466 -0.193303333
10897 0.523696668 -0.226034563 0.220820584 0.066672852 -
10898 0.397209469 0.392386813 -0.104898555 0.379175739 0.045059011
10899 -0.230669972 0.007267123 0.211561021 0.207160254

0.207220809 -0.428638043 0.363418947 -0.176835122 -
0.385940248 -0.067431641 0.161826547 -0.289464945 -0.356680473
0.126465013 0.036593797 -0.232610996 -0.226561058
0.185797218 0.414083152 0.342126721 -0.014297379
0.04484565 -0.182565092 0.407497542 -0.136933159
0.084304917 0.013250493 -0.166692021 0.105648141 -
0.259913811 -0.288840951 0.402543793 0.415278744 0.381056102
-0.482787749 0.000251829 -0.133227487 -0.214602304
0.284833654 -0.10180155 -0.064086881 0.078554774 -
0.083789838 0.023822099 0.266127876 -0.081475664 0.238338824
0.115198286 -0.2023962 0.151970651 0.326042076 0.183231588
KEGG_OXIDATIVE_PHOSPHORYLATION 0.531465597 0.365742143 -
0.321655674 -0.544833434 -0.091382716 -0.355380013 -0.090582915
0.045856435 -0.420137239 -0.463513937 -0.540464816
0.635233538 -0.355787447 0.168015135 0.289759585
0.482397907 0.257703368 0.170091614 -0.553346171
0.248608674 -0.518995011 0.288400607 -0.18360605 -
0.472516585 0.061617058 -0.309073102 0.54938525 0.188548876
-0.492996949 -0.381563805 0.28598894 -0.543845152 -
0.310483058 0.232866579 -0.222068065 -0.61784022 0.115862175
0.480952661 -0.391885926 -0.402773785 0.602137711 -
0.033926832 -0.491226256 0.434163459 0.403540232 0.439909398

-0.047245562 0.490030915 -0.366189404 0.03099994
0.674044896 -0.336879665 0.394690084 -0.588056169
0.300624149 0.2427629 -0.383307159 0.052914806 0.374373052
-0.413144527 -0.193472516 -0.574696242 0.3226728 -0.349774426
-0.34613091 -0.670400998 -0.293393717 0.336311883 -
0.140467645 -0.001432782 -0.192278402 -0.488425648 0.552720307
-0.034937883 -0.341374575 -0.029715703 0.191785864 -
0.175926613 0.3809008 0.652739484 -0.265821047 -0.578987157
0.454740305 0.203875286 0.128341563 -0.381147013 -
0.16851092 -0.351915403 -0.570976092 -0.144513319 0.033073472
0.337359261 0.27641355 0.505423003 0.268019886 -
0.595096836 0.462232225 -0.644571323 0.550652198 0.425230673
0.158892912 -0.383093194 -0.294473862 0.564622919 -
0.413272024 -0.564844715 -0.161720966 0.682555434 -0.629691881
0.095752634 -0.373066861 0.219837454 0.574888324
0.110616314 -0.602244543 -0.318723793 0.615218963
0.093860042 -0.428135249 0.438906464 -0.040510053
0.289793018 0.40038538 0.585595878 -0.646202642
0.207206282 0.715921908 -0.601082513 0.135595235 -
0.216936547 0.416016871 -0.395814593 0.334121802 -0.415199914
0.3807718 -0.566366776 -0.435741631 0.264554882 -0.247866953
-0.560220064 -0.112256821 0.398657862 -0.35465501 -

0.322085747 -0.059852163 0.433117781 -0.31207213 0.126682749
-0.482846805 0.413625343 0.189023676 -0.366858046 -
0.558498065 -0.090170543 -0.417782895 -0.095924647 -0.582941661
0.395187104 -0.326333025 -0.420547554 -0.004734445
0.041426573 -0.198815836 -0.472075685 0.657405356 -
0.607601843 0.31045268 -0.388933521 -0.360637335 -0.662374024
0.169023195 0.361196613 0.304790054 0.486619914 -
0.507007593 0.123463932 -0.599434357 0.019301407 0.238030504
-0.387682771 0.514342804 0.514369933 -0.152471717
0.352002894 -0.39921473 -0.232696104 0.001872279 -
0.381263969 -0.446612496 -0.191556016 0.261756962 0.728284152
-0.167184887 -0.098814074 0.6086267 0.300124822 -0.533492715
0.205203694 -0.415443817 -0.274406521 0.51615076 -
0.058269452 0.353742794 0.588891318 -0.31127428 -0.180579413
-0.548124062 0.246069795 -0.414342106 0.349472937
0.342215894 -0.38047777 -0.232265933 -0.130534395
0.157842401 0.285800716 0.52214408 -0.579406801 -
0.4861335 -0.586480055 -0.550308538 0.499861516 0.067199804
0.127971759 -0.524061662 -0.164685088 0.451503794
0.288372793 -0.40697827 0.554307573 0.547250692 -
0.272042147 0.168798099 -0.585145299 -0.592406821 -0.236450651
-0.495885262 -0.515213452 -0.401013257 0.548302769

10966 0.395411265 -0.46404448 0.039437913 -0.183073712 -
10967 0.225475771 -0.340002172 -0.362487487 -0.328881247 -0.049837522
10968 -0.136014094 -0.349802769 0.030835924 -0.069215811 -
10969 0.535156043 0.513931138 -0.485563768 0.343982344 0.327584335
10970 0.190538777 -0.211991015 -0.240797055 0.581244574 -
10971 0.270424401 -0.117119055 0.373473517 -0.505996456 0.081643722
10972 0.512440831 0.089828749 -0.074631812 0.598607751 -
10973 0.2191872 0.393644716 0.309012948 0.597826628 0.436524985
10974 0.08234126 0.231957388 -0.395680636 0.01324624 -
10975 0.498265559 0.417521518 -0.365787031 -0.187179994 -0.557287581
10976 0.625871727 -0.547076381 -0.328395101 -0.453681247 -
10977 0.362173794 -0.259128878 0.396590621 -0.172987011 -0.589012714
10978 -0.482066446 -0.101914027 -0.226623636 -0.492914666 -
10979 0.165468973 -0.396215157 -0.470345212 0.442000966 0.069839081
10980 -0.116193542 0.624631479 -0.542043899 -0.600302297 -
10981 0.277938328 -0.438651472 -0.105784637 -0.550673478 0.216530732
10982 -0.3444691 -0.442997582 0.491485307 0.495969443 -0.237372358
10983 -0.349528446 0.232536845 0.191816912 0.608460503
10984 0.654454893 0.36920347 0.1251972 0.324728956 0.202843732
10985 0.523636402 0.446416936 -0.169313556 -0.385854994 -
10986 0.602979822 0.210318853 -0.561817594 0.51774217 -0.469977145
10987 0.045881915 -0.13919948 -0.368080126 -0.526816719 -

0.302504024 0.576565084 -0.104012099 -0.385648657 -0.358410403
0.045695856 0.329066061 -0.641538418 0.131093129 -
0.296591583 -0.527349781 -0.588524082 -0.293324846 -0.426220945
0.553760368 0.302333641 0.05106339 0.251448204
0.045032994 0.510461513 0.794188256 0.453204675
0.616929401 -0.239344781 -0.507725055 0.231307779 -
0.670175714 0.298050281 -0.25732481 0.287689148 -0.630724881
-0.53067071 -0.312290678 -0.383717497 -0.278852859 -
0.069752094 -0.060665044 -0.01532786 0.476558929 -0.555420556
-0.487057101 0.327701791 -0.03917881 0.185296207
0.092059183 -0.180327784 0.372395361 -0.382090268 -
0.488912535 -0.15812402 -0.431679909 0.375989574 0.432401475
-0.459795694 0.236064422 -0.458584653 -0.178561474
0.319565208 0.237328084 -0.587539686 -0.247078523 -
0.58308668 -0.438310309 -0.338018659 -0.056865385 0.104876021
-0.263968875 -0.591226149 -0.436690185 0.245903242 -
0.513838397 0.6060053 -0.584749022 0.388933979 -0.437864303
0.413722973 -0.403641737 -0.449400384 0.60984357 -
0.173316549 0.486698799 -0.523302124 0.223372355 0.644309742
-0.358265476 0.015445961 0.501278634 0.363313793 -
0.376548333 0.113576103 -0.100741337 0.176051767 0.399972428
-0.16425318 0.097791266 0.06703283 -0.581093401

0.07097882 0.684557 -0.012628621 0.248217915 -0.500564301
-0.197126121 -0.507435398 -0.365439595 -0.187548123 -
0.481044253 0.639146033 -0.442269221 0.250384409 -0.42121945
-0.472472527 -0.30125645 -0.387707754 0.505359892
0.637181797 -0.446525815 -0.481837433 0.271422978 -
0.141949441 -0.248817947 0.481081971 -0.546183428 -0.150231345
0.0345961 -0.236209614 0.118128701 -0.393365811 -0.195727662
-0.228083549 -0.161413881 -0.308286521 0.417270771
0.049926881 -0.641989904 -0.044443845 0.279963026
0.328443784 0.466959329 -0.535740362 -0.516061035 -
0.212035398 0.425682807 0.087157857 0.576165694 0.636275793
-0.066599191 -0.344957779 0.510279417 -0.392478132
0.100316698 0.560386266 -0.547761246 -0.542308856 -
0.577628651 -0.166997261 -0.263538821 0.444005788 0.445100067
0.512035825 0.392243264 -0.229136861 0.480168078
0.435112816 -0.315597625 -0.291752407 -0.278925422
0.087986525 0.096531412 -0.097184411 -0.454302984 -
0.606819173 0.52464079 -0.561147001 -0.081702522 0.349013734
0.572445167 0.184968574 0.135605912 -0.137505207
0.443007683 0.531265737 -0.099585198 -0.059367458
0.670411914 0.345701302 -0.121331145 0.010048239
0.26070545 -0.312130912 -0.374227939 0.1486989 0.409560186

0.185458 -0.280493906 0.403833993 -0.543357332 -0.19445657
0.335981323 -0.427084847 -0.371773997 0.205579146 -
0.078513697 0.21262595 -0.062928873 0.046090588 -0.018285981
0.214611074 0.33741926 -0.342344886 0.351598579
0.108648015 -0.268231868 0.232334441 -0.317995865
0.424111328 -0.155167786 0.222286808 -0.373892066
0.020095135 -0.301902594 -0.038644815 0.000918272
0.089779828 -0.025001132 -0.121067687 0.235705289
0.080987041 0.028116678 -0.163417486
KEGG_PURINE_METABOLISM 0.240395862 0.109923402 -
0.014434007 -0.14200796 -0.117865639 -0.029959827 -0.045022947
-0.04031215 0.142939784 -0.022722905 -0.186103432
0.045663755 -0.115905124 0.070366905 -0.081760281
0.222201434 0.090011910.142999437 -0.118130303 -0.015474515
0.076852471 -0.158721118 -0.004319828 0.064331724
0.047276729 -0.140575499 0.109030281 0.121844778 -
0.067116398 -0.056936854 -0.036402389 -0.149248594 -0.099503358
-0.126814831 -0.042332817 -0.021175311 0.109694379
0.027649559 -0.1090855-0.027092589 -0.04895719 0.079797561
-0.030445831 0.006735244 -0.096336329 0.253929286 -
0.072936384 -0.138134206 -0.098173959 -0.207125029 -0.11613318
-0.18838284 0.176220137 0.035105223 0.1081328 0.185577268

11054 -0.168475234 -0.025355929 0.092585709 -0.014169485

11055 0.067785116 -0.087357036 0.080951765 0.168209876 -

11056 0.098830419 -0.080639887 0.017229276 0.014513186 -0.161166095

11057 -0.310238207 -0.011111836 -0.094842663 0.01012525

11058 0.033760617 0.018654748 -0.11560962 -0.102952858 -

11059 0.024084186 -0.013011723 0.130036802 -0.113713639 -0.175600121

11060 0.12447873 0.180586764 0.129066385 0.108594402

11061 0.016249524 -0.077680936 -0.146898156 -0.022586736

11062 0.205477888 -0.051842752 -0.087356201 0.213376938

11063 0.036676746 -0.186030068 -0.073052018 -0.159197644 -

11064 0.034301378 -0.081963069 0.039643039 -0.24799482 -0.144958735

11065 0.112265311 0.13402802 -0.080382497 0.093544083

11066 0.064515051 -0.13491761 -0.177472789 -0.18562824

11067 0.06472219 0.104256171 0.170446404 -0.22571108

11068 0.073977291 0.049074639 -0.212053694 -0.226637386

11069 0.062826955 -0.269068446 0.057636239 0.021964176 -

11070 0.167932983 -0.134323455 -0.105421784 0.253297014 0.026970552

11071 0.080038383 -0.128462025 -0.018383108 -0.18929796

11072 0.062546883 -0.179075095 0.164762998 -0.04723904

11073 0.115738177 0.181874521 0.078902798 0.000226417 -

11074 0.16155992 0.136499898 -0.051746095 -0.073177635 0.01557532

11075 0.032279377 -0.052861088 -0.101018445 -0.188704357

11076 0.124933815 -0.04489652 0.14429525 -0.067546078 -
11077 0.076700373 -0.175685789 -0.069538529 -0.030343723 -0.105163037
11078 0.024002201 0.016542399 0.121143958 0.200858856
11079 0.060696905 -0.26835996 0.150132611 0.090492336
11080 0.03687168 -0.104093482 -0.10124322 -0.167436616
11081 0.265232783 -0.079460375 -0.144202829 0.025948093
11082 0.038131953 0.178501508 -0.106665911 -0.082727322
11083 0.090193737 -0.00909615 0.043250628 -0.124770893 -
11084 0.187855231 0.161269318 7.17E-05 -0.095182505 0.139565894 -
11085 0.209776372 -0.134876162 -0.170113025 0.195250934 0.16825512
11086 -0.231819215 -0.07866346 0.109997953 0.031237067 -
11087 0.211518511 0.197192295 -0.010700582 -0.13645914 0.144513413
11088 0.057413292 0.000999689 0.061741083 0.054931173 -
11089 0.081011288 -0.146327211 0.196831048 -0.034105154 0.017848605
11090 -0.024740166 -0.057994691 0.02581439 0.13592648 -
11091 0.021436727 0.146198569 0.193226056 -0.13859311 -0.027617055
11092 -0.153387346 -0.111268121 0.148254189 -0.325279886
11093 0.225823187 -0.241608801 -0.091187783 0.13423387
11094 0.017228918 0.020784742 0.045657199 0.104337375 -
11095 0.000268596 -0.196180149 -0.08074812 -0.185803232 0.073034774
11096 -0.17899802 -0.185522492 -0.213038554 0.037797311
11097 0.123353925 -0.127879987 -0.143322142 0.105015987

11098 0.107337196 0.011855191 0.031038717 -0.166390903 -

11099 0.27568996 -0.228781953 0.022746746 0.002043182 -0.182867652

11100 0.090550753 0.008118865 0.039975491 0.038741633 -

11101 0.11481652-0.019843974 -0.258458795 0.078262961 -0.049714025 -

11102 0.005366345 0.006614592 0.128932062 -0.006738599 0.011977216

11103 0.12339016 -0.203597024 -0.196136725 0.118675272

11104 0.143046573 0.081374345 -0.011587766 0.264064613

11105 0.053733259 -0.119613739 0.032421438 -0.173773524 -

11106 0.149268286 -0.153911163 0.201069293 0.122929745 -0.293755585

11107 -0.23036186 0.024825277 -0.25618472 -0.106701698

11108 0.086031494 -0.141613597 -0.095310736 0.026499127

11109 0.158118132 -0.028890267 -0.147653166 -0.176329604

11110 0.038790124 -0.065589551 0.056432115 -0.202277131 -

11111 0.247195817 -0.035419799 -0.218428139 0.044170517 0.066529485

11112 -0.078002333 0.042137925 -0.032011396 0.06417813 -

11113 0.258451522 -0.092229459 -0.010569623 -0.202055182 -0.105097185

11114 0.148484799 -0.007529111 -0.040224587 0.032869021

11115 0.124332305 0.105181502 -0.113268881 0.091484691

11116 0.221014762 -0.0538453 0.228945504 0.056192867 0.13256834

11117 -0.078392617 0.093266001 0.168219263 0.064223939

11118 0.088783464 0.035671126 0.02764103 0.045387578

11119 0.107488748 0.089924285 0.047565303 -0.109750791 -

0.053335825 0.239070647 0.04373509 -0.264199201 -0.126732035
0.087728797 -0.036228328 -0.06327855 -0.077146038
0.098522626 0.080864362 0.166284251 0.00943308
0.071271472 0.033795909 -0.055978354 -0.166131881
0.026841046 0.005430139 0.239539895 0.110331816 -
0.020872914 -0.033764406 -0.096619556 -0.04154208 0.120659294
-0.250464956 0.0884396 0.110757085 -0.043977788 -0.067659026
-0.03763205 -0.048877841 -0.052271198 0.140168334 -
0.15282874 0.008143305 0.030949624 0.296091799 -0.004850799
-0.099407371 0.002300667 -0.011480952 0.188060842 -
0.113546192 0.080594662 0.024879007 -0.148281844 0.022873623
0.166004693 0.109763237 0.014109110.092266843 0.021199285
-0.050562285 0.095693604 -0.026910496 -0.16349421
0.073787635 -0.134708467 -0.118737205 -0.171975535
0.104874684 -0.114148527 0.003775631 -0.003559203 -
0.19927688 -0.121660477 -0.013692305 0.0671809 -0.130278257
0.072065638 -0.074916814 -0.112628895 -0.038590101 -
0.101153021 -0.120738134 -0.037747729 0.169163355 -0.081480208
0.180452448 -0.068251522 -0.12794634 -0.028682928
0.093226931 -0.153703369 0.138139148 -0.089585185
0.033238442 0.237961208 0.186489715 -0.024839665
0.093677411 0.015708376 -0.133817414 0.052196164 -

0.164630426 0.063861553 0.044130843 0.095887624 0.134259874
0.028966985 -0.003147024 -0.052453773 -0.203743493
0.056631745 -0.09296235 -0.068337767 -0.204404089
0.014598446 -0.152774202 0.177546622 -0.229516556 -
0.071237131 -0.019887987 0.028120022 0.130720007 0.06595904
0.031938193 -0.125351961 0.168642483 -0.074530613 -
0.254110561 -0.108272221 -0.092154514 0.125172298 -0.004374059
0.018415614 -0.266462457 -0.078346709 -0.195026118 -
0.152310141 0.027908325 -0.041722156 -0.084745667 -0.020844477
0.0947384 -0.060176151 0.06252009 -0.102978758 -0.04272731
0.091689853 0.028898133 -0.05603131 -0.144629731
0.103758376 0.030440185 0.038984299 0.13986539
0.003525189 0.172705062 -0.212415098 -0.054843882
0.093981788 -0.080231229 0.066384688 -0.176453049
0.191181729 0.008047809 0.100083684 -0.000810395
0.239489159 -0.059798107 0.091628036 -0.017382314 -
0.04058522 0.195615515 0.064670559 0.116865715 0.021315022
-0.029938747 -0.225957801 -0.077831962 -0.100016639
0.05380756 0.02901826 0.013826042 0.198724311
0.060196113 -0.225902064 -0.148090325 -0.00820397 -
0.097679315 0.205822915 0.006706005 0.15727696 0.042170727
0.207357339 0.250449239 0.014543151 -0.08442681 -

0.148904015 0.096089962 0.139572151 -0.061721389 0.172662632
0.012603521 -0.153763671 0.113056035 0.101637896
0.087236066 -0.157342875 0.148401966 -0.110882569 -
0.216546885 -0.105371162 -0.102552766 -0.104626112 0.169756677
-0.051116629 0.186735573 -0.336592333 0.028521711
0.077592226 -0.159686016 0.032063652 0.036114010.050266016
0.017705158 0.066193026 0.228451257 0.119639241 -
0.089232968 -0.151990923 -0.156832644 -0.078829459 0.106918708
-0.166411962 -0.037003195 -0.183552391
KEGG_PYRIMIDINE_METABOLISM 0.327472223 0.069044372 -
0.073804288 -0.274729334 -0.206332938 -0.168761696 0.019603048
-0.148582695 0.261992795 -0.029792938 -0.312689345
0.273345963 -0.347488257 0.08772681 -0.234926577
0.284616559 0.160258279 0.062048539 -0.314166483
0.251268105 -0.009431749 -0.01609951 -0.166051775
0.175196054 -0.002683271 -0.237676643 0.161612282
0.208318309 -0.03163974 -0.253189999 0.050885244 -
0.193746069 0.067364516 -0.13534771 -0.256977048 0.045283254
-0.155525882 -0.057850897 -0.30067651 -0.055158576 -
0.105214886 -0.049386361 -0.083209334 0.046721321 0.013605133
0.277708654 0.153174844 -0.310511977 -0.157353749 -
0.270197247 -0.164771585 -0.291443706 0.162805293 -0.021097695

11186 0.276469412 0.204419626 -0.341010984 -0.019304344

11187 0.158151096 -0.206353366 0.143754749 -0.29113211

11188 0.202441092 0.253341371 -0.089105294 -0.220471645

11189 0.021421049 -0.012539541 -0.313854413 -0.234306457

11190 0.249253488 -0.109101766 -0.176619755 -0.007429101

11191 0.092032194 -0.115883625 -0.013375856 0.21089739

11192 0.020819828 0.220404096 -0.202771932 -0.285099365

11193 0.12813428 0.029637311 0.07306149 0.335599223 -

11194 0.014524831 -0.227589239 -0.157928109 0.176946115 0.326392601

11195 -0.165482668 0.104935346 0.260835118 0.227812674 -

11196 0.308503841 -0.203447637 -0.284807603 0.205287198 -0.137523793

11197 0.065899043 -0.407262038 -0.211565932 0.146704125

11198 0.000347434 -0.030205151 0.114528835 0.037367706 -

11199 0.272070224 -0.367162687 -0.469570721 0.177741266 0.393109768

11200 0.062988027 -0.208186296 0.087395548 0.250971804 -

11201 0.191838142 -0.330721181 0.207249037 -0.326026812 0.167114653

11202 0.29860488 -0.188158571 -0.256793885 -0.195859981

11203 0.272298801 0.026065961 0.273811401 -0.093864041

11204 0.073221262 -0.270798122 0.26811194-0.144999108 0.192150739

11205 -0.238004031 -0.040746256 0.256547222 0.165423671 -

11206 0.16092074 -0.1649955 0.000473324 0.023796983 0.079133893

11207 0.017156027 -0.071595256 -0.047249207 -0.264535696 -

0.315041829 0.15341901 -0.145314939 0.176125173 -0.264759973
-0.328926435 -0.215998556 -0.144801416 -0.146636496 -
0.063020675 0.09267361 -0.274525824 0.146455063 0.367832729
0.266022297 -0.388842751 0.25616847 -0.077407591 -
0.003224145 -0.27085423 -0.160757073 -0.31747028 0.396106518
-0.135872537 -0.269894971 0.048868087 -0.118512572
0.108387942 -0.079950887 0.008041296 0.221883932
0.121963677 0.008085619 -0.238691098 -0.175259775
0.147351019 -0.068355363 -0.04231925 0.221862216 -
0.303134315 -0.242661758 -0.116893007 0.375982062 0.212592309
-0.218958253 -0.276830621 0.372845069 -0.060766416 -
0.238247766 0.239060969 -0.12138141 -0.258861927 -0.068554678
-0.009987798 -0.267836438 0.074289306 0.282765869
0.146428645 -0.202419821 0.419853939 0.063159038
0.133633677 0.096813667 0.253741407 0.056480429
0.327139228 -0.13215045 0.317188424 0.359688243 -
0.298315532 -0.071545663 -0.207495213 -0.3565993 0.180882527 -
0.354439588 0.516686282 -0.311415496 -0.219193109 0.291029187
0.074714623 0.01602617 0.024421402 0.348557774 -
0.076270592 -0.159118042 -0.271314768 -0.165337464 -0.106233728
-0.248818633 -0.254320338 -0.31462687 -0.007970305
0.178163151 -0.080297536 -0.127140902 0.129847776

0.278782103 -0.051646751 0.052114483 -0.258061364 -
0.103149092 -0.303843256 -0.143250305 -0.040344608 -0.299514205
0.157083191 0.092839022 -0.19247631 0.018361607 -
0.135289831 0.263979388 -0.381090639 0.279547077 0.212274164
0.11497192-0.01185214 0.077948582 -0.227935675 -0.087593928
0.095094525 0.038603998 -0.377984848 0.285315929
0.385787217 0.242433665 -0.093174499 0.417469401
0.234022874 -0.027460262 0.219196862 -0.226638702 -
0.129625808 -0.353810496 0.270491168 0.160989403 -0.402576292
-0.40302139 0.1537399 -0.332694911 -0.036803754 0.049097879
-0.316743108 0.003655225 -0.181975048 0.177273211 -
0.120226398 -0.277208075 -0.282350689 0.02894215 -0.110311193
0.191942401 -0.297143965 -0.208144431 -0.093388924 -
0.318043088 -0.057581001 0.15089722 -0.255602737 -0.094184655
0.09598754 -0.186325639 -0.29588539 -0.137079918
0.156058098 -0.251389637 0.007178154 0.010910183
0.147539971 -0.34340788 0.048628141 0.257526304
0.124474057 -0.282338797 0.179376227 0.216912525 -
0.269932295 0.386296737 -0.30202928 0.311181231 -0.127787392
0.029858026 0.20668457 0.091838326 0.175846051 -
0.073394852 -0.031232826 -0.106747521 0.110984467 0.083797653
0.09116443-0.031521268 -0.215384779 0.291896969 0.252618815

-0.350257968 -0.191351616 0.116197633 0.014505309 -
0.259589467 0.050935286 0.206235742 0.205194793 0.09406384
0.144055911 -0.072201916 0.264859967 0.130293343 -
0.228433853 -0.027964834 -0.014282808 0.402455784 0.043712629
0.162240373 -0.209800812 -0.078581291 -0.052980826
0.211507096 -0.446867195 0.357912456 0.072373543
0.094124882 -0.19899275 -0.114930863 -0.125310914 -
0.180160554 -0.002655968 0.064347217 0.119946796 -0.014332153
0.342070967 -0.246396892 -0.076893163 0.014782942 -
0.088398722 0.01904539 -0.11078104 0.033727476 -0.072358884
-0.296683766 -0.171035645 0.220650005 0.257383925
0.175593149 0.125800669 -0.163839721 -0.119987083 -
0.14389918 -0.170060591 -0.074044214 0.153174838 -0.30786365
-0.391598037 -0.317881254 -0.099155212 -0.180884285
0.171498396 0.059729078 -0.046111846 -0.145653109 -
0.052179973 0.152571856 -0.141453558 0.310283861 -0.219515861
-0.121140565 -0.196920352 -0.023841519 -0.138867729 -
0.047892093 0.261271143 -0.206838443 0.245098172 0.031961881
-0.035048947 0.102742891 0.063805064 -0.25578735
0.309883096 -0.092427904 0.006905587 0.16204954
0.158367407 -0.111585725 0.123380092 -0.140148129 -
0.284646802 -0.009461153 -0.25155968 0.175513495 -0.017418126

0.374809432 -0.07867371 0.10224061 -0.164383765 -
0.17055343 -0.403485696 0.145814907 -0.126212384 -0.255842048
-0.290620478 -0.118076076 -0.048828012 0.292712253 -
0.314603046 0.048449541 -0.191719212 0.206535149 0.115207772
0.088361736 -0.117972323 -0.284614534 0.105859945 -
0.1510797 -0.233636581 -0.165054971 -0.128352735 0.148518149 -
0.045811351 0.155622478 -0.388709449 -0.029259803 -0.228820958
-0.113019715 -0.030884328 0.029358557 -0.196021061
0.128703994 0.324176956 -0.094293554 0.084203913 -
0.12063292 -0.193458415 0.214688166 0.071194569 0.141130276
-0.281106731 0.212800367 0.082536112 0.024917656
0.34105925 0.058030962 0.062737502 -0.191279399 -
0.291644604 0.007091495 -0.023800473 -0.112956773 0.002307206
0.192613796 0.023084127 0.295925336 -0.070086709
0.318804561 0.110246040.046114747 -0.284602083 -0.234905742
0.164501007 0.057219681 0.208568612 -0.02526035 -
0.092380853 -0.224758406 -0.076479884 -0.186913951 -0.03018751
0.069216289 0.090114642 0.210939958 0.245204936 -
0.326458572 -0.134225958 0.225590636 -0.062977679 0.09615479
-0.051198129 0.371371376 0.173359863 0.203595663
0.34002197 -0.0697425-0.069533776 -0.342791714 0.063460781
0.284006476 -0.100672949 0.273343404 -0.156865384 -

0.322569245 0.183023297 0.154598329 -0.015649592 -0.277236754
0.30185157 -0.156395498 -0.26437318 -0.005722909 -
0.282033101 -0.020335079 0.293140177 -0.047004127 0.385472181
-0.441376018 -0.147080647 0.036496188 -0.272273863
0.167074685 -0.160769818 0.217854149 0.07536194
0.272939254 0.281672296 0.07600245 -0.293339857 -
0.186687456 -0.166148949 -0.20504903 0.26765065 -0.197371873
-0.209759993 -0.093688301
KEGG_ALANINE_ASPARTATE_AND_GLUTAMATE_METABOLISM
0.433861291 0.28704889 -0.454250819 -0.125386188
0.349901297 0.243756078 0.011659381 0.144843437
0.011658368 -0.032938912 -0.124002111 0.038147887 -
0.391233723 -0.414800911 -0.072927243 0.271577539 -0.297150516
0.492125852 -0.429440883 -0.026806158 -0.340260729
0.271965724 -0.636879159 -0.370478397 -0.318390654 -
0.284636666 0.366433703 0.429016651 -0.040676293 -0.096915399
0.516536097 0.056299448 0.262397601 0.29241843 -
0.416713512 -0.423487125 -0.287351309 0.218485792 -0.559154198
0.031376855 -0.067613652 0.392805781 -0.439067185
0.300383694 -0.400691494 0.448418878 -0.234652922 -
0.392671646 0.203363685 0.089013803 0.029674462 -0.41296901
0.097780041 -0.507794349 0.245839317 0.469785044 -

0.350079286 0.132220294 0.355680035 -0.442208481 -0.442151711
-0.19815378 -0.110320464 -0.193195557 -0.300447692 -
0.318951802 0.213986062 0.310090834 -0.25631046 -0.079151165
-0.157810937 -0.495136709 0.087842602 0.039330544
0.295555617 0.140587728 0.390629338 0.208224955
0.441089898 0.397625588 -0.009215129 0.001173791
0.391456072 -0.377479597 0.31484761 -0.099214139 -
0.506089207 0.239810475 -0.268742047 0.310344079 0.077908839
-0.28629958 -0.164483485 0.493702666 0.312993482 -
0.382277154 0.160157536 -0.397151679 0.465871699 0.395012436
0.301859992 -0.240031156 0.138656235 0.51174608-0.155796324
-0.129999082 0.231569043 0.5227793 -0.623273419 -0.419804474
-0.244363664 0.253866818 0.400468806 -0.633131096
0.018458623 -0.148490925 0.027600793 0.176775365 -
0.040655555 0.076440539 0.166837583 0.470462489 -0.223593974
-0.027351571 -0.293329591 0.234736009 0.163409361 -
0.389198784 0.396591002 0.099585899 -0.020512464 -0.256279284
-0.143663628 -0.001630836 0.045881754 -0.557088627 -
0.567941885 0.402317945 -0.0837877 -0.518351311 -0.045223379
0.492358255 -0.014963446 -0.332238238 -0.518433889 -
0.42458717 0.016454612 0.509948634 -0.120855068 0.241449517
-0.215572337 -0.281397451 -0.430349264 -0.202718483 -

0.115133788 -0.261335193 -0.356351296 0.599282878 0.377473794
-0.10273178 -0.352071111 0.481568285 0.033051974 -
0.243747908 0.507976192 -0.3806954 0.207551624 -0.2383694 -
0.018436149 -0.293403194 0.044676341 0.344849571 -0.087150598
-0.189213843 -0.420766758 -0.570963758 -0.334691063
0.439659383 -0.034030235 -0.169764751 0.159644935
0.045397515 0.263106052 0.39051375 -0.429011712
0.260098629 -0.274413757 -0.213821389 -0.293463956
0.529196679 0.145293172 0.424054314 0.349731095
0.118908089 0.033323547 0.188790167 0.204559548
0.292946157 -0.142043923 -0.132109471 0.153894592
0.286023886 -0.15420609 0.478241515 0.113688268
0.284593402 -0.103519559 0.424239137 -0.356121276
0.482199784 0.479185337 -0.205427812 0.429055106 -
0.225794063 0.13229197 0.093221386 0.410829646 -0.041097287
0.141786332 -0.390634725 -0.508740062 0.509846717 -
0.083463292 -0.223863888 -0.291805747 -0.329768633 -0.139635416
0.096401134 0.149977109 0.483155329 0.271821887 -
0.223478607 -0.112964104 -0.200380825 0.09406647 0.050026066
-0.211449133 -0.170652434 0.148122356 0.036942396
0.202190841 -0.256125664 0.182220667 -0.290515482 -
0.192176753 -0.273415005 0.478632811 -0.432420259 0.23069285

-0.051051856 0.170836323 0.065502976 -0.272383273 -
0.587911253 0.009862304 -0.438207552 0.233775656 -0.069119792
0.399885039 -0.220533733 -0.293088758 0.202600615 -
0.194040873 0.226449162 0.100980759 -0.575909456 -0.298183321
0.265325717 0.005340715 0.191938806 0.290907001 -
0.311488008 -0.065861052 -0.122003376 -0.003442757 0.083091452
-0.045170078 0.12898444 0.005728011 -0.389542961 -
0.394758213 0.03113876-0.449150431 -0.351226644 -0.462367972
0.428654431 0.047314788 -0.220359699 -0.092306473 -
0.184406861 0.025883452 0.500344592 -0.136942566 -0.385865057
-0.200887466 0.147135447 0.101711948 0.007178431 -
0.341288156 0.124256572 0.036981942 0.383181648 0.106840494
-0.103217721 0.494100708 0.079520194 -0.397659478
0.303879088 -0.511485305 -0.112158152 -0.24674417
0.215247567 -0.448290345 0.009345608 0.437158312
0.053483988 -0.212944198 0.263870355 0.329289611
0.15507171 -0.151846827 0.496800114 0.050663941
0.072529069 0.098585119 -0.095394361 -0.108506728
0.507230294 0.061794198 0.112577445 -0.019269392
0.032458405 -0.359109562 0.556656247 -0.431036521
0.306544833 0.231208551 -0.422629787 -0.174933791 -
0.053005203 0.317904332 0.018177901 -0.259023929 -0.357551238

11384 0.385970049 0.069493942 -0.332959603 0.253486745

11385 0.244360025 -0.375848002 -0.450823713 -0.14687392 -

11386 0.504413477 0.505410551 0.058372862 0.238140573 0.136762605

11387 0.366323628 0.252308304 0.04185536 0.280304662

11388 0.105176861 0.116487486 -0.49637183 -0.215776439 -

11389 0.435974845 0.17959138 -0.256798965 -0.440335145 -0.396280187

11390 -0.20614055 -0.446576668 -0.058755318 -0.223708578

11391 0.099637604 0.018117505 -0.386816823 0.281833456 -

11392 0.294261495 0.01650618 -0.238688865 -0.268472599 0.479700944

11393 -0.066672021 0.041909333 0.361852651 0.172219048 -

11394 0.315144695 0.07389539 -0.136133087 0.456538501 0.250241367

11395 0.08125008 0.546130705 -0.096081157 -0.056005559

11396 0.268000695 0.028826927 -0.35989644 -0.227497749 -

11397 0.008939526 0.18705523 -0.373338788 0.50361014 -0.173564761

11398 0.267948704 -0.024405268 -0.183074876 0.111953431

11399 0.175214555 0.466363469 -0.268192897 0.622810522 -

11400 0.621194527 0.170171256 -0.427499092 0.15346359 0.474130457

11401 -0.224341855 -0.221151045 -0.225006083 0.303941146

11402 0.405687954 -0.400511605 -0.267448736 0.060514366

11403 0.524641589 -0.267644276 -0.430124538 0.103092032

11404 0.287786225 0.136891156 -0.163298537 0.146996836

11405 0.345457856 -0.495732374 -0.017153203 0.091183432 -

0.43414793 -0.303432221 -0.158160791 -0.441595274 0.095886477
-0.256531717 -0.065691355 -0.01701819 -0.194098274 -
0.48991697 -0.43490798 0.266712021 -0.3922708 0.051550866
0.367450334 -0.072598875 0.338996656 -0.258054162 -
0.495876061 -0.579504654 -0.355256511 -0.108930721 0.124365799
0.052042129 -0.231773587 -0.380322526 -0.527538454
0.425213085 0.061392414 0.064640001 -0.20861044 -
0.29159617 0.117400437 0.420368116 0.188394052 -0.489736345
-0.154765858 0.11825586-0.184299689 0.31961831 -0.209460637
-0.405284055 -0.300818764 0.069976097 0.306048508
0.017415626 0.233868481 0.373973047 0.035064348
0.38289002 0.203322582 -0.543614221 -0.234855453 -
0.310658494 -0.443929265 0.093012675 0.052165428 -0.019743764
0.399376928 0.251255859 0.043540001 0.398120117
0.339505979 0.138056262 0.299486516 -0.515810118 -
0.604410558 -0.1902735 0.012716558 -0.198404257 0.192390022 -
0.177904166 -0.001463586 0.295860306 -0.470404179 -0.204132229
0.453789797 0.402758204 -0.308037233 0.142798167
0.15946841 -0.104830559 0.560839059 -0.323976143 -
0.200411095 0.374063843 0.38442635 -0.440532224 0.165847065
-0.006018819 -0.129541106 -0.571636949 0.179619873
0.005707557 0.2493259 -0.252534179 0.217440589 0.219032423

0.259883531 0.283524759 -0.171660797 -0.425424876
0.142830094 -0.233630805 -0.014776523 -0.074803168 -
0.236265823 -0.092140423 0.052601224 0.048154074 -0.266969717
0.427578805 0.150463613 -0.443789922 0.003423036 -
0.376127752 0.122448136 -0.322411738 0.342040927 -0.155376254
0.360575186 -0.068254938 0.08769552 0.208178661 -
0.319612504 -0.103349347 -0.005487885 -0.059209053 -0.29923369
0.070935428 0.372991839
KEGG_GLYCINE_SERINE_AND_THREONINE_METABOLISM
0.406536512 0.418969061 -0.299029961 -0.36364037
0.391529297 0.122722646 0.159566481 0.214728324 -
0.069372769 -0.066308284 -0.295576533 -0.166562329 -0.468742042
-0.317288999 0.016047925 0.269396041 -0.299633305
0.33856918 -0.270531268 0.109539727 -0.28431142
0.436164982 -0.652794636 -0.103954523 -0.373505785 -
0.183916421 0.231109650.144620391 -0.355343077 0.320165174
0.468527565 -0.321753721 0.090216965 0.353900873 -
0.183747962 -0.315834797 0.019126977 0.295003059 -0.468996382
0.076154135 -0.307510953 0.34989781 -0.201026419
0.365681945 -0.581170339 0.493107298 -0.060428858 -
0.465003288 0.409368206 0.245102403 -0.017229229 -0.497935102
0.07471049 -0.220822396 0.173936218 0.368710022 -

0.109414738 -0.00286866 0.352618682 -0.131511007 -0.585845375
-0.113345573 -0.216697282 -0.012360107 -0.146989178 -
0.258851851 0.145803601 0.129129435 -0.117086848 0.016489867
-0.438210609 -0.467517402 -0.323400712 -0.190572934
0.229860115 0.244031205 0.01429141 0.104043082
0.377463827 0.163805768 0.182736259 0.233847943
0.188601454 -0.18765344 -0.134962392 -0.355487745 -
0.573186395 0.028841346 -0.194829748 0.29682234 -0.065724909
-0.001552179 -0.02184697 0.451892082 0.033810326 -
0.239946563 0.123847913 -0.490511814 0.509156021 0.305118531
-0.282166234 -0.108207376 -0.139236417 0.474080216 -
0.091840861 0.174945701 0.393626374 0.457132069 -0.472508784
0.057058968 -0.006586172 0.422290115 0.58856981 -
0.613535882 0.271747809 -0.437035493 0.173833431 0.210108217
0.304613229 0.034153489 0.256650802 0.490105523 -
0.155263671 0.108234863 -0.321641482 0.413728817 0.056554763
-0.466552125 0.374952856 -0.224234344 0.196076352 -
0.298410107 -0.089240991 0.015068864 0.365458885 -0.438730696
-0.610161941 0.339050626 0.106042347 -0.282422418
0.126101494 0.428043112 -0.061396705 0.206270051 -
0.480191137 -0.326427889 0.219821263 0.409918732 0.316541886
0.132477881 -0.225117678 -0.490340157 -0.465651437

0.059440282 -0.034568752 0.185048548 -0.539181118
0.483205839 0.163851218 -0.105279217 -0.429480021
0.481493649 -0.144753963 -0.179748838 0.412502704 -
0.3556899 0.224869519 -0.214233979 0.362383367 -0.452228711 -
0.069002677 0.419431038 0.000258357 0.196187434 -0.399487016
-0.351964515 -0.482273997 0.458094005 -0.130086566 -
0.427860163 0.238614356 -0.141948716 0.365561716 0.272074798
-0.073536801 0.540852352 -0.00379529 -0.285685208 -
0.316449315 0.168244444 -0.30561934 0.394391427 0.379159112
0.020252274 0.262728304 0.461376684 0.312613432
0.442799428 0.049148432 -0.162022349 -0.284959506
0.24395429 0.129513438 0.595210308 0.05531586
0.454898387 0.02725872 0.081144209 -0.243040512
0.445541359 0.209715653 0.069130341 -0.024420956 -
0.383847488 0.304696315 -0.233606553 0.260581623 -0.126401924
0.153640151 -0.455253333 -0.285447546 0.359426341
0.254237765 -0.611540938 -0.323471126 0.073071074 -
0.08633267 0.072923338 -0.02523647 0.490185163 0.388143493
-0.237394107 0.051240921 -0.221063277 0.112324765
0.448279471 -0.376472736 -0.269625711 -0.145796095 -
0.011731404 0.213481243 -0.17125179 0.162702048 -0.085399404
-0.475848708 -0.300514465 0.395959897 -0.400078713 -

0.14878956 0.011359495 -0.198793927 -0.078867599 -0.06456608
-0.661120597 0.129849064 -0.445113147 0.263461525
0.254616828 0.332150894 -0.005835888 -0.456409163
0.046355531 0.289321378 0.408859027 -0.217439438 -
0.461940512 -0.36400883 0.308262564 -0.148683419 0.286799628
0.309880947 -0.296005199 0.046068021 -0.214936503
0.174018334 0.015975023 -0.011923904 0.332022211 -
0.178810903 -0.032676595 -0.254802226 0.057379293 -0.461923304
0.054784313 -0.244533498 0.134745349 -0.111232517 -
0.190383235 -0.120003591 -0.229609797 -0.295620888 0.165154351
-0.164912888 -0.502191478 -0.212085526 0.243211126
0.041604528 -0.270790209 -0.363603038 -0.053362355 -
0.161186384 0.515627594 0.217642211 0.299420281 0.560227519
0.201004945 -0.389405971 0.146852206 -0.582706884 -
0.094584633 -0.261095062 0.470907306 -0.101275464 -0.185617216
0.189224597 0.101511187 0.036177564 0.463537039 -
0.022941302 -0.179978335 -0.129185076 0.299535733 0.307605945
0.257030172 -0.042655386 0.338776758 0.05583774
0.462059675 0.297330476 0.359159487 -0.02780347
0.301605063 -0.296948281 0.528043895 -0.345070037
0.130759507 0.2162893 -0.61153308 -0.191215063 -0.310096681
-0.102581688 -0.039448988 -0.217020329 -0.251275229

0.191503412 -0.219619014 -0.355035688 0.443290886
0.486899834 -0.409055204 -0.498036899 -0.286485787 -
0.387031041 0.348808851 0.130270594 0.126898441 0.327513332
0.083126852 0.39929152 0.075526305 -0.013354345 -
0.136000671 0.310215467 -0.347564973 -0.087103631 -0.339070014
0.017398788 -0.282195167 -0.137128342 -0.343956125 -
0.023012851 -0.460990397 -0.203537511 -0.121060848 -0.038643579
-0.115902316 -0.483073123 0.363441431 -0.52825574 -
0.1780045 -0.071282597 -0.255449507 0.371199741 -0.413520728 -
0.320419428 0.398771603 -0.157988726 -0.446607063 -0.261397941
-0.273726932 0.191366173 0.226929992 -0.129479277
0.266804017 -0.228297566 0.319203069 0.444396398 -
0.306143902 -0.283232829 -0.140694018 0.01640716 -0.153303449
-0.384197513 0.378425079 0.184243285 0.318806278 -
0.041594884 0.158540715 -0.390572758 0.179127018 0.2983653 -
0.307646643 0.514014088 -0.37692783 0.140247841 -0.158714115
0.014969587 0.209310883 -0.149020675 -0.337276977 -
0.515421992 0.142894121 0.21562985 -0.010043502 0.173927968
0.232031552 0.501746597 -0.246985329 -0.371863505 -
0.037538885 4.65E-05 0.16822484 -0.304836402 0.30691357
0.535400015 -0.54640409 -0.199102718 -0.293110273 -
0.416361291 0.002872001 0.052840462 -0.485346989 0.171549313

0.056739874 -0.297060343 -0.1568833 -0.311469518 -0.383733077
-0.467189324 0.430670421 -0.498788105 -0.01244344
0.388075932 0.330090198 0.414565993 -0.424566649 -
0.521068856 -0.402905854 -0.363475295 0.021484868 0.308751135
-0.079916914 -0.301020001 -0.130773989 -0.439927881
0.453306451 0.091496434 0.037596671 -0.034794302 -
0.250386499 -0.027727885 0.509157399 0.134007546 -0.371925045
-0.401172367 0.154262276 0.116555023 0.556150945
0.001235618 -0.378039227 -0.195753414 0.332830896
0.208052627 -0.109981901 0.230496566 -0.022003539 -
0.226354675 0.378241319 0.206632802 -0.393504435 -0.280007944
-0.134053443 -0.330356943 -0.214159513 -0.206974583
0.310424602 0.521753975 -0.043193145 0.037301498
0.339510273 0.102398299 0.076099757 0.074471818 -
0.39389217 -0.528260058 0.053631338 0.3331236 -0.23539909
0.45328582 -0.249209733 0.143682915 0.15453194 -
0.477764339 -0.068893445 0.480767131 0.270302134 -0.052742625
0.088515201 -0.090268803 -0.158761861 0.656576674 -
0.086366688 -0.298776391 0.473849973 0.075780252 -0.486293263
-0.103451039 0.147761707 -0.051526021 -0.471308592
0.44099992 0.244261124 -0.085402177 -0.28402428
0.105466737 0.454584943 -0.00769268 0.273734544 -

0.23421282 -0.491359503 0.508849518 -0.351055192 -0.116698259
-0.188421006 -0.121994018 0.299004761 0.070252325 -
0.251616751 -0.070306636 0.303308723 0.310673677 -0.441207227
-0.237007666 -0.357216708 0.234200429 -0.028474687
0.231472871 -0.326675637 0.00083996 -0.386876465 -
0.314773949 0.474328572 -0.115260964 0.082438805 -0.30197521
0.167897615 -0.141332268 -0.136986098 0.15503034
KEGG_CYSTEINE_AND_METHIONINE_METABOLISM 0.212557976
0.221509964 -0.14328962 0.166319213 0.183468611 -
0.038700272 -0.183351853 0.140875954 0.275429325 -0.130072004
-0.357418699 -0.009027116 -0.165513943 -0.155201798
0.031246617 0.196535725 -0.280017739 0.006274751 -
0.29823851 -0.024306686 -0.306035321 0.231185281 -0.265867112
-0.124200467 -0.091462839 -0.23534659 -0.058553389
0.214031696 -0.268986085 -0.013656361 0.380811543
0.081578428 0.15505472 0.209163843 -0.2480216 -0.324354512
-0.051842657 -0.02147063 -0.418753712 0.063300142
0.023880786 0.043909412 -0.296284019 0.159859657 -
0.105957939 -0.008859294 -0.101516619 -0.149593115 -0.023391095
0.078390302 0.135308691 -0.202347139 0.028725853 -
0.06467104 0.014021197 0.162071511 -0.252554175 0.136839572
0.084401682 -0.183488505 -0.297266146 -0.333021652 -

11582 0.052043973 -0.159427601 -0.413977258 -0.110413319 0.02201735

11583 0.092436163 0.103363766 -0.290386469 0.005989119 -

11584 0.267620912 0.025260096 0.026670137 0.236400213 0.033664902

11585 0.130868932 0.226609906 0.414200853 0.130173615 -

11586 0.006811686 -0.115648461 0.024349469 -0.1737609 0.234505362

11587 0.132580891 -0.207189601 -0.112867332 -0.283441716

11588 0.438616886 0.115477972 -0.065240428 -0.017026277

11589 0.369319537 0.094609132 -0.192116568 0.049475895 -

11590 0.225194999 0.127064273 0.516046843 0.014257216 -0.176924559

11591 0.186442932 0.29760842 0.040911678 -0.001136721

11592 0.207551808 0.37713933 -0.421306385 -0.273987324 -

11593 0.211129585 0.405532934 0.303130223 -0.268998044 0.106109266

11594 -0.03451405 -0.20433497 -0.011021387 -0.054679332

11595 0.174946157 -0.012674243 0.182209396 0.005489314

11596 0.035832737 -0.259879523 0.058802971 0.025381227 -

11597 0.330569344 0.22507319 0.064601771 0.009218681 -0.270699517

11598 -0.338938648 -0.01297569 0.002883977 -0.500141599 -

11599 0.43257709 -0.060261312 -0.190905182 -0.3824535 0.117561874

11600 0.436622927 0.125172341 -0.069475321 -0.159735898 -

11601 0.271500205 0.300560906 0.226016153 -0.100637296 0.157262554

11602 -0.147623492 -0.265780758 -0.208352367 -0.220113555 -

11603 0.033367876 0.072621655 -0.239854142 0.365595187 -0.020659001

11604 0.322271948 -0.183693285 0.37770205 0.153235474 -
11605 0.170513823 0.210167323 -0.026881584 -0.288792445 -0.011451457
11606 -0.018495869 -0.317063648 0.038919476 0.283944072
11607 0.090202078 -0.039269883 -0.422449648 0.041575018 -
11608 0.394546782 0.137878548 -0.081695099 0.026572201 0.293897998
11609 0.025340356 0.157079423 0.345974181 -0.320741118 -
11610 0.083844707 0.067444942 -0.015216042 0.014312339 0.214682918
11611 0.140398583 0.149708369 0.271785921 -0.059868555
11612 0.106529706 0.083357365 -0.107385626 0.141549561 -
11613 0.127140356 0.055082863 0.132821056 0.124485896 0.047596759
11614 0.346957265 0.315315513 0.154401553 0.112736753
11615 0.092954364 -0.292305393 0.250162923 0.161211775 -
11616 0.066918086 0.117621311 -0.264306706 0.235556348 -0.293465115
11617 0.175436852 0.111455345 0.091525299 -0.324711781 -
11618 0.141421423 0.191129788 0.05720084 0.014488475 -0.405574199
11619 0.050385184 0.393279692 -0.187897134 0.277231856 -
11620 0.139026412 0.234108217 -0.136071783 -0.131657258 0.10897333
11621 -0.044380386 0.057179915 -0.138700816 -0.055037988
11622 0.034527188 0.058937201 0.150913328 -0.05625638 -
11623 0.171929394 0.04470293 0.158032158 -0.25258594 0.433198813
11624 -0.495441397 -0.282842778 -0.200850445 0.168649446
11625 0.00181033 -0.26532297 -0.302618315 -0.210971413 -

0.396478166 -0.031706354 -0.25589292 0.240296998 0.231477233 -0.408336295 0.000840943 0.034100199 -0.025359016 - 0.076315696 -0.355633464 -0.140692531 0.013717938 0.06901304 0.131544511 0.155441269 0.171755696 -0.136143787 0.040957743 -0.031468627 0.128130505 0.259765324 0.207668404 -0.091760785 -0.282285793 -0.374753625 0.201555593 -0.135541595 -0.278017203 -0.195851707 - 0.026285458 -0.137855643 -0.093441572 -0.115662717 -0.203131338 0.124306372 -0.059304123 -0.112633703 -0.362603735 0.093976925 -0.1429868 0.074390605 -0.315934601 -0.199495271 -0.08025808 0.288032882 0.469403673 -0.13057933 0.161685102 0.454111514 -0.032889584 -0.028422784 - 0.066897585 -0.225112381 -0.290037237 -0.155093226 0.339204953 -0.360508645 0.012028218 0.067242528 0.239866906 - 0.121499351 0.113840585 0.028974863 -0.05768999 -0.03174756 -0.016077922 -0.320816877 -0.28873408 0.31739302 - 0.019837701 -0.315809086 0.205417664 0.032510011 0.190902746 0.06919629 0.003032448 -0.13230965 0.309782554 - 0.206191573 0.254280909 0.004781442 -0.000451455 -0.047711748 -0.242312729 0.077531316 -0.018262917 -0.220833318 - 0.373357249 0.144172931 -0.081823399 -0.173295254 0.124117061 -0.00919265 -0.393514497 -0.314257989 0.015661071 -

0.408781058 0.185942245 -0.09897698 -0.032681039 -0.075094075
0.144713889 0.073449593 0.096438512 -0.057922665
0.079028812 -0.053389545 -0.332986419 0.077622455 -
0.259463127 0.067533099 -0.290743457 -0.267452656 -0.190699794
0.166812884 -0.272290571 0.065368355 -0.149489036 -
0.019139008 -0.075806058 0.082308991 0.298053387 -0.283656957
-0.28591653 -0.323489847 0.165371435 -0.135755672 -
0.339306475 -0.082254876 0.064770245 0.394610794 -0.195163863
0.047372937 0.093785835 -0.120674876 0.045465895
0.184194932 0.377849598 -0.158171132 0.069837856
0.357104449 -0.174497109 -0.231252373 -0.258369061 -
0.095990398 0.041739546 -0.313003156 0.199205113 0.216309225
0.21631445 0.256003383 -0.030033074 0.060551887
0.143441156 0.194981132 -0.25241135 0.337049572 -
0.258373755 -0.120134673 -0.064906878 0.310813215 0.13737605
-0.120864681 -0.099725306 -0.374077783 0.104105436
0.114347286 -0.198310618 -0.011611913 0.083262974
0.351248632 -0.292970221 0.043792074 0.131504015
0.137776407 0.155202317 -0.062131234 -0.114257486
0.367316192 -0.264392019 -0.270196568 0.183361657
0.124345657 0.055221617 -0.003128842 -0.089614027
0.292105906 -0.118247153 -0.033656384 0.016764227 -

0.031137108 -0.28318893 0.018628284 0.164559029 -0.266193296
-0.095613577 0.276196286 -0.184467476 0.110843802
0.148354767 -0.079756323 -0.338314777 -0.057233424
0.056935615 0.137323654 -0.128178331 -0.284595742 -
0.160353181 -0.39939577 0.306015449 0.125016136 -0.361411777
-0.033537508 0.048537967 0.168177246 0.223486928
0.24783405 -0.289657113 0.145612313 -0.080484152 -
0.304566552 0.196366207 -0.190281167 -0.290836831 -0.182310513
0.034579011 0.306607535 0.035066622 0.342639281
0.256917156 -0.188043149 0.436287599 0.101510546 -
0.085444871 -0.25669642 -0.083521253 -0.251960567 -0.236682908
-0.199395852 0.093626365 0.409955691 -0.061186719
0.047339543 0.001015364 0.395560567 0.098359426
0.248492494 -0.412441556 -0.369377069 -0.243721873 -
0.197157077 -0.264397376 0.30193789 -0.197945449 -0.249727451
0.043448446 -0.174525092 -0.150649295 0.149329253
0.184566717 -0.151561317 -0.27817097 -0.095422329 -
0.206151389 0.20739652 -0.313731419 -0.018462972 0.347147345
0.14771943 -0.019703657 0.399564888 0.175386958 -
0.042755724 -0.325722654 0.044468655 0.168679239 0.327169229
-0.299151194 0.188663645 -0.199987309 -0.097680905
0.042611634 0.02122466 -0.265366545 0.059723722

0.074779342 0.11003795-0.183085838 -0.138506214 -0.138191027
0.088122586 0.154251973 -0.174732326 0.388354852
0.264777263 -0.152647425 0.18326186 -0.028861264
0.053448894 -0.297729993 0.233939451 -0.220532086
0.218824148 -0.079449994 -0.017661727 0.162598499
0.004340295 0.050043996 -0.172264739 0.264181443 -
0.36257949 0.190427406 -0.068803318
KEGG_VALINE_LEUCINE_AND_ISOLEUCINE_DEGRADATION
0.585028797 0.665004776 -0.446871745 0.146058449 -
0.15421548 0.23674374 0.419802375 -0.470891509 -0.41755393
-0.592576928 -0.050794744 0.476608272 -0.574507511 -
0.341716156 -0.48615102 -0.503648614 0.202899049 0.354313025
-0.670087116 0.180730945 -0.386983647 0.382703898 -
0.717927762 0.088107256 -0.637057881 -0.084154419 0.393328231
0.333299555 -0.075691167 -0.54020795 0.629099394 -
0.092423511 0.529515506 0.344337716 -0.405089333 -0.458698091
-0.033434747 -0.215624466 -0.554445106 0.49808577
0.289165082 0.351685796 -0.398410691 0.6286948 -0.193949905
0.444128549 0.018587355 0.276744322 0.053626618 -
0.165098845 0.534495129 -0.431683483 0.01312817 -0.578582507
0.555535937 0.506064551 -0.618845529 0.328996818
0.580229883 -0.047728231 -0.636187698 -0.001843862

11714 0.224425396 0.311335597 -0.376090891 -0.263796538 -
11715 0.031218703 0.517092784 -0.445272069 -0.383033852 -0.404307977
11716 -0.514612939 0.514908199 -0.443984099 0.06652696 -
11717 0.393185062 0.397177982 0.543378129 0.20061432 0.625732459
11718 -0.097604724 -0.049195429 0.492790849 -0.242034567 -
11719 0.33202156 -0.565324495 -0.380722515 -0.284546575 -0.561817294
11720 0.555659582 -0.444712963 -0.314883809 0.098417451
11721 0.628640153 0.476128091 0.106631882 0.360500376 -
11722 0.601589384 0.506665109 0.477684485 0.078394392 -0.4543423
11723 0.402086113 0.443538179 -0.48324399 -0.238303352
11724 0.437988099 0.657566133 -0.540133686 -0.497269802 -
11725 0.383009263 0.129632098 0.656531051 -0.526361557 0.331678438
11726 0.280218737 0.109046939 -0.203838888 -0.312820749
11727 0.423353759 -0.254046194 0.591777779 -0.180021086
11728 0.44083519 -0.310131597 -0.242080896 0.422928049 -
11729 0.457459352 0.586009861 0.417509095 0.230800376 -0.579284576
11730 -0.197745319 0.24634353 0.155909337 -0.568089626 -
11731 0.677287404 0.592306601 0.26342001 -0.549807345 0.111002389
11732 0.422117238 0.511940627 0.315076023 -0.478081278 -
11733 0.145066673 0.399674105 0.530993007 -0.402769379 -0.183273364
11734 -0.624232968 -0.399663241 -0.520450688 -0.36387059
11735 0.243374016 -0.282940328 -0.588651732 0.56533981

11736 0.384796997 -0.47855681 -0.356374725 -0.084635216 -
11737 0.293112088 -0.56878906 0.681197221 -0.650469958 0.306496817
11738 -0.025935514 -0.495662165 -0.435587889 0.34678869
11739 0.44157666 -0.098027983 0.135852635 -0.487874535 -
11740 0.35734903 -0.162971905 0.613520275 -0.242497912 -0.402562079
11741 -0.061649354 0.366241737 0.429789539 0.317949344 -
11742 0.106947398 0.279569153 -0.147127473 0.057356331 -0.54149306
11743 0.511308173 -0.44530486 0.536377093 0.625470351 -
11744 0.268125859 0.412363814 0.32155046 0.125546381 0.541598951
11745 0.052025644 0.420081005 -0.491632556 0.334187242 -
11746 0.246943826 0.548747326 -0.208971698 0.401178872 -0.530048077
11747 0.397998178 -0.275430718 0.262369725 0.692918405 -
11748 0.195833331 0.349072971 -0.372498286 -0.28738507 0.115097617
11749 0.377676117 -0.269742791 0.403505825 -0.408236255 -
11750 0.582682259 0.684206317 -0.045002145 -0.443955259 -0.540145441
11751 -0.061737098 -0.43854606 -0.460163716 0.287006936
11752 0.351990649 0.204809319 -0.203720482 0.435629813 -
11753 0.333597235 0.510378912 0.418803722 -0.574585865 0.198225129
11754 0.265137657 -0.081498439 0.394052858 0.097421114
11755 0.545105211 -0.422718761 -0.394413212 0.312791558 -
11756 0.054270029 -0.369623906 -0.010213429 -0.304462575 0.110345307
11757 -0.079217858 -0.61151957 -0.683998011 -0.189075299 -

0.489106486 0.536165525 -0.423778382 0.612216584 -0.286399322
-0.62201972 0.437299434 0.290287148 -0.359915995
0.414410768 -0.475338054 -0.595647018 -0.225017048 -
0.100810122 -0.42429521 0.554936219 -0.399750656 0.185734428
-0.194019396 0.113998456 0.358648957 0.543179843
0.200945061 -0.22419755 -0.45582031 -0.624527556 -
0.184905625 -0.479296403 -0.55671347 -0.660332835 0.133303468
-0.645538084 -0.13601743 -0.353158444 -0.284299761
0.080295197 0.334976219 0.301363316 -0.384720894 -
0.559110416 -0.523306381 0.493816966 0.271118188 -0.550050047
-0.109432795 0.340290997 0.548763049 -0.255882117 -
0.326590806 0.692104646 0.07596515 -0.557376012 0.626777957
-0.674213898 -0.434084704 -0.484695148 -0.155577615 -
0.552123817 0.416083427 0.263930508 0.612701893 -0.475111539
0.549144116 0.570643252 0.51413949 0.266873084
0.616980886 0.53710778 -0.272152841 -0.249965313 -
0.020368984 -0.064719393 0.237923632 0.420069012 0.00780411-
0.558043389 0.374453352 -0.573878039 0.631607291 -0.159414845
0.603749663 0.559308473 -0.616835446 0.110078465
0.405408039 0.407063146 0.272065027 -0.328023277 -
0.332183974 0.580705649 -0.096536611 -0.287504303 0.612542975
0.151424293 -0.410673726 -0.582076658 -0.214304124 -

0.378999805 0.556039822 0.514007278 -0.301075895 -0.249960292
0.463687525 0.497775219 0.244637661 0.163738031
0.556489482 0.641604135 -0.569786588 0.263415166 -
0.358551262 -0.379400766 0.076898828 -0.445697247 -0.406672003
-0.605946907 -0.402521569 0.355834721 -0.133390109
0.386140578 -0.492725157 -0.590049416 0.249898718 -
0.501159395 0.17982773 -0.471867609 -0.644159402 0.402877719
-0.487649285 -0.254305165 0.627448874 -0.032304829 -
0.524903203 0.118227494 -0.112519579 0.302444997 0.427580393
-0.302388037 0.398005154 -0.020002899 0.401694356
0.457117562 -0.515624928 -0.328376128 0.21050961 -
0.195803881 0.245237438 -0.159079197 0.757225709 -0.490361454
0.528249079 0.245590879 0.376626607 -0.124608138
0.430286149 0.576785008 0.221634665 0.296559395 -
0.677939211 -0.30985026 -0.216844552 0.356796543 0.531768907
-0.096535536 -0.414929245 -0.517527987 0.594502057
0.382823239 -0.075372915 -0.373802455 0.429226902
0.552495323 -0.130741497 -0.544655027 0.448209452
0.275997342 0.386056814 -0.481026364 -0.051412468
0.346401378 -0.616944494 0.287402628 0.611252691 -
0.253637179 0.011528920.320491413 -0.40489955 0.318854124 -
0.508250635 -0.624898613 0.217371882 0.296104385 -0.635236682

-0.500339681 0.212243429 -0.562614307 -0.288075854
0.198389012 -0.225772528 0.279572262 -0.372794174 -
0.502828294 -0.617013015 0.027860896 -0.20127182 0.365684285
-0.138787047 -0.656179165 -0.122271511 -0.383985662
0.200202711 -0.432080217 -0.399663197 0.248033138 -
0.403458364 0.09381913 0.599206072 0.445601342 -0.53175968
-0.618494101 0.187334574 0.045332307 0.371478492
0.232913183 -0.371695012 -0.325932962 -0.371077837
0.473472969 0.255221130.428327502 0.615835986 0.020192378
0.215253123 -0.311021894 -0.32727032 -0.238485473 -
0.415963718 -0.184787671 -0.19023435 0.095890455 -0.063706757
0.120823746 -0.232280844 0.307431234 0.281569825
0.442032646 0.587915816 0.524213437 -0.48409718 -
0.461531875 -0.264832583 -0.060551996 -0.173025408 -0.195325187
0.058580642 0.245099338 -0.024010021 -0.59861641 -
0.427351497 0.582874148 0.505112862 0.014016856 0.093766356
0.331688018 0.264397952 0.640775475 -0.456187959
0.055578974 0.565942754 0.299358096 -0.396896819
0.229454784 -0.44216719 0.250283254 -0.292787338
0.035385684 0.309234122 0.251124926 -0.001068738 -
0.087110517 0.122435767 -0.257208326 0.398768673 -0.500710643
-0.587751296 0.513993493 -0.500547738 -0.295643269 -

0.090319503 0.108180012 0.304314657 -0.049902144 -0.285123259
0.265271979 0.579301475 -0.115035727 -0.039537328 -
0.129820125 -0.586499471 0.094224772 -0.307322194 0.566730762
-0.438428613 0.485313766 -0.414901197 -0.221059628
0.008373723 0.171331122 -0.139734823 -0.366179786
0.053380372 0.057482651 -0.388336143 0.447752198
KEGG_LYSINE_DEGRADATION 0.491621159 0.494474772 -
0.049119794 0.269168763 -0.327944776 -0.057746209 0.300929903
-0.351752373 -0.132113615 -0.339298055 -0.040254242
0.208628092 -0.478743528 -0.046539258 -0.38987123 -
0.394240153 0.135120016 0.342885178 -0.557764167 0.158188527
0.232128575 0.250307897 -0.433130054 0.403922827 -
0.385583097 -0.35745579 -0.32760461 0.084636852 -0.225855154
-0.459735322 0.473753851 0.075877382 0.382329613
0.06886172 -0.249200906 -0.137433545 0.049511598 -
0.104539773 -0.395954593 0.322490695 -0.068049828 0.41062663
0.290743653 0.293515145 -0.008871585 0.415065998
0.309850215 -0.05784789 0.254338372 -0.341148719 0.1083031
-0.301190884 0.101055444 -0.205725086 0.465170211
0.401930103 -0.526298046 0.32921526 0.354249983
0.14323641 -0.389356543 0.115663581 0.265128174
0.328724518 -0.406281594 0.014817851 0.136268717

0.340679004 -0.416069435 -0.399567231 0.011684655 -
0.231496238 0.168605904 -0.354842108 0.191600479 -0.397743922
0.004544066 0.393314714 0.278807482 0.449877155 -
0.038560867 0.111400138 0.153623784 -0.154232351 -0.283301005
-0.10924134 -0.094288685 -0.163843107 -0.312086155
0.307457252 -0.341138699 -0.469984509 -0.012089194
0.329491137 0.279559743 0.202058267 0.090843929 -
0.458336836 0.034866259 0.382161256 0.284934178 -0.423569777
0.0230088 0.076858214 -0.232851801 -0.022806604 0.251968156
0.411377616 0.07466084 -0.483719711 -0.429763786 -
0.038014037 0.385665384 -0.470126288 0.209999505 0.146299032
-0.191445631 -0.401588643 -0.536219789 0.361145556 -
0.18825853 0.417555378 0.119139112 0.079130191 -0.123926749
-0.441483356 -0.16658618 -0.158569955 0.564672396
0.47435243 0.109290229 -0.467445992 -0.052250327
0.106261147 -0.104609522 -0.215807217 -0.336026039
0.604970885 0.439692793 -0.078394882 -0.012160478 -
0.035015986 0.468424329 0.170620509 -0.31443843 -0.13073777
0.304284558 0.421600561 -0.388845885 -0.062092487 -
0.502886441 0.083508308 -0.356238204 -0.31670107 0.21812002
-0.430105311 -0.276825263 0.214716098 0.399661597 -
0.316783253 -0.081933724 0.058368068 -0.179742751 -0.46934995

0.320890028 -0.349862928 0.167381742 0.264605731 -
0.276683803 -0.032251746 0.361492745 0.096536302 -0.115645805
0.087137995 0.104132037 -0.196785954 0.03704509
0.355833422 -0.038619469 -0.197171586 -0.239661724
0.030062464 0.109886791 0.011776862 0.226944133
0.381868636 0.076885023 -0.143183796 -0.572871261
0.537740098 -0.117732126 -0.051421106 0.422569859 -
0.287801173 -0.154067162 0.124429518 -0.071786324 0.192689189
-0.136720084 0.225178393 -0.237533112 0.324162371 -
0.307385673 0.310506278 -0.166005389 0.129720794 -0.303225383
0.149810249 0.058033398 0.077301034 0.541691466 -
0.230038629 0.35944898 -0.085891493 -0.162678689 0.174304607
0.108803557 -0.011839094 0.336939807 0.040810208 -
0.421188607 0.498773889 -0.363475923 -0.169788464 -0.502151153
-0.18623185 -0.261741545 -0.477950833 0.41890026 -
0.007667038 -0.268972003 -0.126883513 0.019023364 -0.259060267
0.370545521 0.383558593 -0.489264965 0.224505811
0.095420831 -0.260628217 0.268536226 0.217259593
0.084517757 -0.252049712 -0.180831975 0.283537102 -
0.146688174 -0.219492963 -0.05684834 -0.288678681 -0.106545892
0.250325007 -0.480766735 -0.193664542 -0.340480935 -
0.209318496 0.449704631 -0.451030731 0.547281507 -0.177790026

11890 -0.447290517 0.259376168 0.261872503 -0.34326925

11891 0.419733322 -0.354702645 -0.364270694 -0.262775705 -

11892 0.036142625 -0.442922113 0.086245042 -0.069337293 0.095474596

11893 -0.17038322 -0.056126354 0.304255247 0.504589824

11894 0.021030064 -0.044869402 -0.291121874 -0.486714336 -

11895 0.238544812 -0.142445928 -0.487382593 -0.573990495 -0.013163431

11896 -0.475085752 -0.04704269 -0.256450613 -0.324678612

11897 0.079955633 0.041882741 0.314654986 -0.0010674 -0.463529077

11898 -0.375489103 0.462744864 0.23355348 -0.311949676 -

11899 0.178040292 0.278842238 0.362999398 -0.139317001 -0.172850114

11900 0.503580901 0.214279219 -0.304591124 0.534556217 -

11901 0.483871718 -0.423759389 -0.319866126 -0.14730545 -0.430862222

11902 0.467662148 0.158748059 0.344318367 -0.491420089

11903 0.432902562 0.389326918 0.427081341 -0.203206963

11904 0.561764602 0.517002243 -0.427894044 -0.232511443 -

11905 0.106058272 -0.268675275 0.07743395 0.463679916 0.34526965

11906 -0.118409691 0.429057429 -0.435309324 0.378950171

11907 0.247561179 0.467683963 0.527236613 -0.254595972

11908 0.262735646 0.393208031 -0.081102828 0.101552413 -

11909 0.378206618 -0.157999509 0.529904178 -0.476748313 -0.042999998

11910 0.381788927 0.07177556 -0.174400433 -0.101706542 -

11911 0.239338504 0.072980478 0.120474774 0.539489875 -0.450952563

-0.279433867 0.404613945 0.364106918 -0.22916099 -
0.067378545 0.221034153 0.515489784 -0.377892141 0.087945201
-0.17832504 -0.137299459 0.226969591 -0.372453952
0.08367308 -0.252283445 0.125053085 0.358725389
0.111404121 0.204373841 -0.383571658 -0.517894904
0.173832706 -0.347185419 0.398901931 -0.338774921 -
0.469973194 0.204865395 -0.308376116 0.003112528 0.424109781
-0.161826122 -0.120427358 0.379583621 0.198442367
0.08659285 0.222685149 -0.121818035 0.312198089
0.201668304 0.441791068 0.168488365 -0.395698465 -
0.151611119 0.091851778 -0.127825533 0.294852982 0.048530626
0.542370157 -0.357812404 0.38699637 0.076351768
0.336078697 -0.025299072 0.517357322 0.413801528
0.299528802 0.164990941 -0.313887747 -0.180160187 -
0.247562285 0.256812152 0.452028066 0.35576894 -0.250463204
-0.416535705 0.314240084 0.263829731 0.402947444 -
0.40933956 0.04221682 0.197616896 0.27324675 -0.395036327
0.406703475 -0.023507929 0.069186776 -0.302333855 -
0.137584134 0.093345596 -0.185795001 0.311110151 0.26210742
0.170842721 0.22579427 0.452313277 -0.274296988
0.24201053 -0.345785762 -0.272452626 0.277096535 -
0.165426407 -0.619701824 -0.534626798 0.244359937 -0.280640947

-0.302974048 0.073921753 -0.419481819 -0.205094518 -
0.073021409 -0.126051009 -0.400919952 -0.231001106 0.103892779
0.285349429 0.047466197 -0.49041156 -0.299166147
0.162531779 -0.12755539 0.002549303 -0.366553219
0.446197246 -0.214127085 -0.181412168 0.341969756
0.40635026 -0.129900657 -0.443438128 0.275263531 -
0.18508908 0.229209175 0.093883528 -0.285239395 0.116928486
-0.243281377 0.268117478 -0.155175674 0.071278219
0.408351039 0.345315279 0.235045936 -0.129465821
0.174535486 -0.434842578 -0.340131938 0.275973533
0.185562408 -0.104347208 -0.252998987 0.078891961 -
0.104300835 0.071688735 -0.182170642 0.279280081 0.509765758
0.288201322 -0.273649975 -0.18784182 -0.141558265 -
0.019889017 -0.061442038 -0.263626701 0.289795424 0.25744633
-0.085322192 -0.217819341 -0.461262936 0.511299284
0.325611858 -0.047960071 0.355470149 -0.008409399 -
0.091931463 0.454380192 -0.41088451 0.085605769 0.143839714
0.165182003 -0.335894413 0.24452788 -0.083300742
0.241197206 -0.009381986 0.081951843 -0.167778031
0.227571195 0.028637473 -0.021169467 0.189790702 -
0.071761532 0.215202258 -0.290265588 -0.409438262 0.276825601
-0.333273784 -0.251557808 -0.1321838 -0.260767353 0.230643808

-0.24343853 -0.148692989 -0.061337874 0.301699258
0.034847252 -0.214097855 -0.005097771 -0.379037383 -
0.173628558 -0.324582074 0.477089773 -0.362858672 0.356317255
-0.292304622 -0.056060074 -0.021794402 0.257805915
0.120872238 -0.286048007 0.205148045 -0.192253179 -
0.175606333 0.347409409
KEGG_ARGININE_AND_PROLINE_METABOLISM 0.484091297
0.214568996 -0.271680112 -0.083000939 0.072540041
0.235178205 0.103031925 0.284282362 -0.179452548 -
0.150993291 -0.27276176 -0.097373893 -0.403765934 -0.333643001
-0.192975356 0.327160655 -0.073380352 0.209445121 -
0.398464294 -0.340191178 -0.239569666 0.363983035 -0.463474924
-0.168277652 -0.148271869 -0.026697318 0.237058592 -
0.025643018 0.026491249 -0.016955906 0.603216418 -0.153788898
0.271660895 0.355390685 -0.280173338 -0.308354634
0.130204496 0.361081773 -0.423196053 -0.0147657 -0.051834472
0.322733146 -0.261004903 0.436094438 -0.306287707
0.253477233 -0.018843572 -0.244196146 -0.191340927
0.191589966 0.169574958 -0.287910223 0.193015841 -
0.3999579 0.199748233 0.337162909 -0.1988288 0.153369723
0.409413772 -0.352046081 -0.505884291 -0.243095526
0.181814065 -0.239279949 0.027046931 -0.399388139

0.230473194 0.16902444 -0.027470134 -0.276432281 -
0.396699574 -0.462927403 0.033627929 -0.129791197 0.006442561
0.059051431 0.421091055 0.142907167 0.256930778
0.434934401 0.087735148 0.098904287 0.112895918 -
0.012028256 0.343038389 -0.18742826 -0.31064089 -0.184561329
-0.493283308 0.247003842 -0.09841179 0.09166475 0.0494559
0.57423335 0.16860027 -0.350601745 0.040077932 -
0.48564109 0.54434815 0.264354987 -0.030258997 -0.205112248
-0.036673759 0.379491593 0.056297933 -0.175666826
0.178791406 0.494338356 -0.506737338 -0.114631853 -
0.09638853 0.089799624 0.410611591 -0.463949341 -0.07740269
-0.156892761 0.085516749 -0.061365843 -0.15437141
0.223138475 0.021223819 0.513067636 -0.145784225
0.083731651 -0.288118365 0.123355975 0.198271741 -
0.504744586 0.37172872 0.003798436 -0.026138905 -0.451272601
-0.073240541 0.07755458 0.299704287 -0.480339235 -
0.447154747 0.271065047 -0.175473777 -0.427544125 -0.205197576
0.457048113 0.030586022 -0.098376536 -0.283351691 -
0.166891334 0.229601391 0.465781642 -0.20998645 0.140662451
-0.183864406 -0.371642072 -0.342280224 -0.090799335 -
0.070145507 0.193584638 -0.453446798 0.347993875 -0.017842627
0.066838166 -0.198314317 0.35264429 -0.174542348 -

12000 0.320295519 0.564293 -0.246957468 0.324114782 -0.055729057

12001 0.119078372 -0.376452234 0.130585565 0.130874512

12002 0.011803420.359252291 -0.323379856 -0.332789402 -0.242379008

12003 0.24086402 -0.012284702 -0.200864136 0.228107814 -

12004 0.052416528 0.215999396 0.258184226 -0.240765168 0.250882911

12005 -0.178799517 -0.225479232 -0.305000483 0.440554349 -

12006 0.161170955 0.437670066 0.248456347 -0.079110728 0.112105703

12007 0.189057110.10402889 0.128683789 -0.01027163 0.007239771

12008 0.193083154 0.015553345 0.015225847 0.406699911

12009 0.075592183 0.158599607 -0.0927648 0.374277004 -0.163017582

12010 0.151206313 0.361214058 -0.261965586 0.250202765 -

12011 0.447864714 0.26412696 -0.252819441 0.28092705 -0.052372562

12012 0.070128277 -0.521617356 -0.412925751 0.424490492

12013 0.252868253 -0.363493508 -0.479855132 0.014637197

12014 0.057500329 -0.138576123 0.325088725 0.371205731

12015 0.10622704 -0.367743338 0.109719478 -0.021061941 -

12016 0.156242511 0.300388848 -0.437784409 -0.179066391 0.116656286

12017 0.28466963 0.211279819 -0.248164137 0.161122888

12018 0.03224185 -0.230836722 0.013003805 0.258398524 -

12019 0.383189191 -0.313699342 -0.049523677 0.209355668 0.305817742

12020 -0.176874895 -0.563796907 0.174709302 -0.311671823

12021 0.264812985 0.015075836 0.364570093 0.130833822 -

12022 0.477177577 0.141185223 0.042695436 0.066495717 0.042587754

12023 -0.356872823 -0.090349095 0.310230077 0.095962281

12024 0.139350506 0.355126481 -0.431820218 -0.105027673 -

12025 0.261374085 0.076521982 0.179069304 -0.186087664 0.285656478

12026 -0.181198418 -0.199052331 -0.456763393 0.258723444 -

12027 0.351681021 -0.227249645 -0.438787801 0.217185011 -0.210765258

12028 -0.253084023 -0.205078297 -0.175515873 0.02241212

12029 0.269626402 0.19419627 -0.279386637 -0.127469765 -

12030 0.058056724 0.109278358 -0.118032674 -0.192752327 0.038634487

12031 -0.05139257 0.31883819 0.084897968 -0.070993998

12032 0.425360799 0.141467911 -0.35524225 0.348962064 -

12033 0.460346668 -0.265543889 -0.029826503 0.142847941 -0.342517854

12034 0.053592814 0.186904488 0.180585728 -0.101411303

12035 0.038684358 0.14696286 0.200188849 -0.128369072

12036 0.240307242 0.200850667 0.166622395 0.113605665

12037 0.268794812 0.025650045 0.200329519 0.321997126

12038 0.322405513 -0.322983689 0.359295943 -0.192577796

12039 0.517780404 -0.284576649 0.391154971 0.131922034 -

12040 0.235538925 -0.280139941 -0.010753148 0.369968537 0.126987749

12041 -0.403429042 -0.268330428 0.309742101 -0.049715479 -

12042 0.326555342 0.253578685 0.023001061 -0.328184308 -0.470203555

12043 -0.212610459 -0.342801206 0.364096246 0.135185207

12044 0.155785366 -0.0588629 0.152495321 0.388077707 0.255576548

12045 0.227164996 0.100531699 0.133547139 -0.298550199

12046 0.178058127 -0.397901639 -0.155367271 -0.15671374 -

12047 0.096016924 -0.455034079 -0.285599296 -0.285038128 0.222924912

12048 -0.029031039 0.149521721 -0.178014985 -0.295378382

12049 0.332172163 -0.40280959 -0.267055608 -0.152416289 -

12050 0.37412146 0.030831869 -0.23158932 0.097741145 0.455760372

12051 0.081056095 -0.377146319 0.132281243 -0.259170425

12052 0.122387545 0.300902133 -0.197522412 0.284364128

12053 0.071691827 0.241665407 0.300329834 -0.249660383 -

12054 0.189788157 0.122914426 -0.192648963 -0.056991224 -0.273428395

12055 0.304650154 -0.051794625 0.243665384 0.159288347

12056 0.035092321 -0.000831267 0.097422151 0.451811031 -

12057 0.281093675 0.312536572 -0.528097254 0.189905048 -0.422847525

12058 0.210824231 0.201161281 -0.085209208 0.008148311 -

12059 0.450109514 0.277158153 0.313608613 -0.199159806 0.004771286

12060 0.141678529 0.463081502 -0.358556093 -0.121757647

12061 0.112272005 0.060815465 0.267101796 -0.027589928

12062 0.102009338 0.28918217 -0.47806236 0.081413191

12063 0.101442552 -0.28908031 0.06151817 0.106479713 -

12064 0.33347084 0.090819709 -0.179733373 -0.050195492 -0.180646284

12065 -0.125551322 -0.49823509 -0.309237517 0.17683403 -

0.430621122 0.127675582 0.36472347 0.243999211 0.341240467
-0.260440391 -0.371126467 -0.340162344 -0.15076605
0.000749873 0.374893845 -0.294045481 -0.265527441
0.046059349 -0.396444526 0.454488879 0.001076944 -
0.278920468 -0.099585559 -0.142866992 0.103900205 0.290787772
0.218843141 -0.426972327 -0.301605213 0.308263288
0.034042535 0.418774153 -0.214112651 -0.300635186 -
0.206396426 -0.023937983 0.377889821 0.086349581 0.104058642
0.308082987 -0.194379536 0.265917477 0.010591206 -
0.331172925 -0.171635741 -0.292447231 -0.02059417 -0.198786873
-0.19628037 0.068624214 0.546205498 0.04564697 -
0.19431818 0.2674596 0.249751871 0.466759439 0.421465864 -
0.153083841 -0.42290759 -0.050326446 -0.089076165 -0.138686436
0.412433113 -0.220463382 0.002607016 0.14726804 -
0.298926876 -0.283028518 0.412798007 0.336283696 -0.109984461
-0.04917739 0.110096487 -0.123131573 0.474752323 -
0.304565309 0.009549231 0.274259962 0.194938476 -0.272126145
0.074764122 0.150897069 0.197207499 -0.282285538
0.108038526 0.115839375 0.145228209 -0.317687004
0.244439639 -0.056376049 0.029325301 0.199713307 -
0.055148616 -0.249326659 0.102170149 -0.40707432 -0.035404691
-0.062744996 -0.085598696 0.102489031 0.061942331 -

0.077789192 0.138509938 0.342469938 0.135813692 -0.409618088
0.133824365 -0.400132132 0.405406631 -0.211045517
0.27789881 -0.364932934 0.179639313 -0.301023075
0.043033379 0.169402966 -0.378737885 0.138163733 -
0.265385127 -0.001344992 -0.03065929 -0.11099324 0.188896021
KEGG_HISTIDINE_METABOLISM 0.41646851 0.0597817 -0.29936243
-0.134981497 0.252365148 0.091979969 0.444362611 -
0.225761166 -0.134658459 -0.281231924 0.04955428 -0.317423229
-0.404181341 -0.385379069 -0.058228422 -0.170397546 -
0.091472846 -0.347298261 -0.517172668 0.211800124 -0.191264779
0.483760016 -0.583626369 -0.180710103 -0.445236075
0.270806271 0.30406473 0.056530169 -0.149941994 -
0.065393381 0.419120071 -0.194301978 0.171138759 0.488041076
-0.511677447 -0.278931907 0.086695328 0.015308692 -
0.203991786 0.444477503 -0.317702495 0.021424715 -0.094985755
0.288817418 -0.449737827 -0.137732855 0.258063455 -
0.347989287 0.188658787 0.336982935 -0.326851023 -0.187955264
-0.152639317 -0.385463723 0.312579471 0.223714525 -
0.208852052 0.054107466 0.517085244 0.102089675 -0.46871973
-0.428891495 -0.03555225 0.061447435 0.474553701 -
0.301575229 -0.088010677 0.013574557 -0.282680295 0.199876726
-0.485739674 -0.507461557 -0.137911791 -0.268879734

12110 0.047097025 0.178300992 0.216322288 0.145755877

12111 0.118115582 0.37795506 0.308520019 -0.107960225 -

12112 0.013228964 -0.071993262 -0.393828385 -0.58820984 -0.534027647

12113 -0.158963375 -0.169258505 0.451101297 -0.374391764 -

12114 0.040263692 0.225713776 0.354058821 -0.042401508 0.389091472

12115 0.02017681 -0.464816303 0.336225856 0.340732629 -

12116 0.140602814 0.003368688 0.177650354 0.317495118 -0.202388264

12117 0.12027879 0.172212492 0.381165603 -0.468930049

12118 0.083314762 -0.060356491 0.395311344 0.286726645 -

12119 0.472125618 0.436964909 0.31451583 0.226228383 0.32868093

12120 0.16906083 0.238944894 0.226259022 0.479430787

12121 0.184904119 -0.203878251 0.049503958 0.267730736

12122 0.015785948 -0.310361424 0.338528603 0.18604498

12123 0.167589682 -0.111379319 0.33852867 0.312324727

12124 0.094853125 -0.526344504 -0.515739829 0.23477473

12125 0.147805802 -0.230235584 0.220738788 0.14751543

12126 0.060903608 0.316498912 -0.364527107 -0.236515714

12127 0.082486324 0.296003705 0.10084175 -0.1400174 -0.06391301

12128 -0.451171595 -0.432896866 -0.03229749 -0.079289278

12129 0.055681084 -0.350180964 0.370711340.473890187 -0.378077332

12130 -0.329859528 0.268419399 -0.117030523 -0.079695207

12131 0.204982613 -0.390506136 0.054432342 0.05879777 -

0.131760669 -0.349306369 0.063298754 0.42719139 0.301761428
0.167709283 -0.349835019 -0.413840518 -0.116637007
0.546193923 -0.21805189 -0.480669238 0.112654678 -
0.192727555 0.352879887 0.027402538 -0.359808336 -0.001182552
-0.045662959 -0.019057158 -0.366155662 0.423415728 -
0.319570314 0.377389552 0.349588108 0.184828352 0.507018123
0.229166162 0.462534116 0.311124025 0.38366907
0.235596317 -0.093973478 0.285617602 -0.315573597
0.438184003 -0.110049922 0.361196135 0.009611286
0.326501708 -0.180457316 0.444042526 0.321677353
0.070788726 0.189532713 -0.309816072 0.14696602
0.092530263 0.20313133 0.083430192 0.490264919 -
0.068290422 -0.437930157 0.234218783 0.272519183 -0.516831333
-0.057980118 0.059496436 -0.265948674 -0.261975404
0.093211014 0.232068517 0.382860404 -0.366206684 0.2302134
-0.514766939 0.286501524 0.034433416 -0.41799614
0.021726489 0.205058377 0.310381198 -0.397757461
0.220756168 0.323410787 -0.114460029 -0.457687116
0.295251178 0.006895764 0.075139169 0.28169254
0.391569162 -0.024252465 -0.156362374 -0.22823846 -
0.619112160.280172216 -0.456596797 0.465181833 0.069391937
0.326489802 0.143933647 -0.258588121 0.149504388

0.397640435 -0.114179916 0.09448667 -0.431827629 -0.182406204 0.073173302 0.381042917 -0.013863877 0.254144244

-0.507328111 -0.064286016 -0.121488421 0.004390939

0.130464927 0.31353853 0.223397635 0.115619778

0.209472954 -0.287758217 -0.092909584 -0.521358668 -0.079622723 -0.355936685 0.310410859 -0.12303821 0.02166493

-0.377347976 0.317318223 0.115803657 0.299779812

0.185232186 -0.490472534 -0.437713781 0.019666038

0.171616411 0.236414708 -0.383214657 0.314093758

0.051779137 0.475465221 0.338618606 -0.115271688

0.475143991 -0.152281413 -0.40018208 0.286672537 -0.498851356 0.252041499 -0.189199344 0.345658783 -0.205968921

0.116676290.016011050.208661027 0.05717738 0.386727757

0.292274838 -0.092962316 -0.372242548 -0.115243542

0.102410123 0.025916734 -0.027258544 -0.082700846

0.098574643 0.445732749 0.328241486 0.074472858 -0.181600974 0.44732143 -0.454773225 0.321176534 -0.498554935

0.123988108 0.222362005 -0.587230963 -0.094422997 -0.02980858 0.380287459 0.274562326 -0.015258328 -0.17063195

0.490126493 0.010793926 0.025864786 0.386660816

0.132522936 -0.204751868 -0.461630477 -0.156566932 -0.353285151 0.552720717 0.479403906 0.345625471 0.212482858

0.217307958 0.4983979 -0.161081837 0.363802525 -0.181995766
0.32502079 -0.502670559 0.149879391 -0.106394962 -
0.239766514 -0.118881455 -0.06642005 -0.417619356 -0.223840525
-0.491352453 -0.061934351 -0.094944956 0.368288158 -
0.22458719 -0.303049669 0.229797644 -0.349225005 0.022331621
-0.285472383 -0.248962086 0.365025504 -0.091483821 -
0.081586113 0.318100119 -0.031246723 -0.566860523 -0.397526126
-0.238794253 0.256184142 0.243922732 -0.07476199 -
0.05464571 -0.340807935 0.03218957 0.455594443 -0.371846335
-0.245577555 0.068935042 -0.015041495 0.050559553 -
0.281766084 0.552715036 -0.052102818 0.296599337 0.224328695
-0.033057403 -0.552112858 0.310106984 0.552098147 -
0.128594628 0.473889788 -0.127646369 -0.025706071 -0.234114271
-0.092193985 0.276195118 -0.186652279 -0.371963803 -
0.468697658 0.336014868 0.31369265 -0.313137936 0.081870447
0.219481114 0.488923597 -0.365469326 -0.515023619
0.280048332 0.005431391 0.219138232 -0.523163577
0.367659309 0.003397635 -0.366205602 0.018126992 -
0.386873101 -0.392498266 -0.32097832 -0.199604899 -0.263394786
0.441177952 -0.147377847 -0.250791139 0.093502505 -
0.403339581 -0.479086637 -0.515266946 0.136630811 -0.431179959
0.167757041 0.454709621 0.25164499 0.354239018 -

0.455051051 -0.364749254 -0.376217085 0.10234976 -0.082982362
0.305083076 -0.004962057 -0.334467312 0.087872105 -
0.292453584 0.401367437 -0.088419271 -0.161857455 0.185872161
0.173780574 0.211922073 0.342735704 -0.10074559 -
0.456232314 -0.33647907 -0.162575953 0.226935825 0.135709018
0.253880415 -0.041206804 0.027837626 0.213201547
0.455889821 -0.200108318 0.342081262 0.335713611
0.022106416 0.306807788 0.032848172 -0.447437988 -
0.281999823 -0.282959717 -0.146442111 0.172118288 -0.223071112
0.382496318 0.243541375 0.132512461 0.399637975
0.360371190.009057678 0.213110617 0.05860806 -0.446463376
-0.533816592 -0.086011214 0.123511663 -0.316846322
0.031768568 -0.042974711 0.018802159 0.155755247 -
0.260673988 0.130949437 0.38150705 0.199226788 0.273023863
0.203767547 -0.003277045 0.212069509 0.56464986 -
0.206605809 -0.343976059 0.257931506 0.306730809 -0.565697885
-0.121509213 0.025263502 -0.110960664 -0.221340635
0.516371582 0.095604715 -0.251754745 -0.161581183 -
0.016333038 0.178401319 0.185347117 0.320724578 -0.439832083
-0.475967881 0.427514327 -0.515723658 -0.048754121 -
0.094853733 -0.239205256 0.292883335 -0.15183217 -0.404135112
-0.085320243 0.344865746 0.353491235 -0.235607939 -

0.25348794	-0.504312033	0.14493827	-0.230625119	0.324066527
-0.31165684	0.001836239	-0.449634065	-0.218962939
0.46231158-0.104111903	0.134142379	0.228334691	-0.339543985
0.045837017	0.035268847	0.273609209
KEGG_TYROSINE_METABOLISM 0.243123674	-0.231142937	-
0.376512629	-0.368490327	0.338813831	0.105801179	0.242313707
0.314100352	0.12439085	-0.232322774	-0.105875948
0.061365218	0.044428606	-0.139049151	0.122846133
0.34968828	-0.245696161	-0.383131061	-0.173247001	-
0.210467015	-0.449994814	0.322859631	-0.354436676	-0.342952973
0.134164414	0.101880688	0.345910325	-0.033075785	-
0.259247095	0.143615446	0.552545467	-0.276765486	-0.348612026
0.310580625	-0.181712841	-0.200636694	0.116407273
0.251325212	-0.14979105	-0.097430833	0.25350552
0.214266205	-0.437613002	0.253125415	-0.189030271
0.021389539	-0.155429966	0.14359921	-0.001369195
0.262091071	0.139189066	-0.059981723	-0.021655397	-
0.292952735	0.069584057	0.071683853	0.050480525	0.25812094
0.147980633	-0.111797395	-0.391398544	-0.158099579
0.035098489	-0.37988066	0.023687202	-0.284470984	-
0.012472163	0.304610975	0.154681699	0.209771866	-0.444400023
-0.488000601	0.137469784	-0.082078646	-0.168474449

0.19609745 0.132901558 -0.032620157 0.241131617
0.217736823 0.271775867 -0.316409096 -0.158582452
0.142960574 -0.078677086 -0.216249719 -0.389298203 -0.138737941 -0.041296368 0.278522033 -0.034776486 0.215296865
0.282195704 0.069226236 -0.192131097 -0.282333613
0.194463786 -0.268126432 0.434173146 0.280584132 -0.113545278 -0.057314055 -0.19949753 0.258032325 0.114219692
-0.160616044 0.19090327 0.38199161 -0.440043328
0.15928735 0.116877718 0.339241841 0.437171467 -0.265284653 0.019318876 -0.277926349 0.207101553 0.277490463
0.185612637 0.112018522 0.255542913 0.217853912
0.055652703 0.287464995 0.057399244 0.33642519
0.287850331 -0.389089203 0.267900525 -0.32803759
0.350298495 -0.08997271 0.04540614 -0.286108395
0.056883902 -0.441845736 -0.200050408 0.029742304 -0.217606453 -0.324439067 0.160944475 0.214542647 -0.351974766
0.184371328 -0.369539176 0.112645178 -0.04840786
0.057424829 0.13419983 0.233669603 -0.040921222 -0.401100002 -0.485993392 0.074299341 -0.360380303 0.318538854
-0.441851482 0.386855244 -0.271235041 -0.066685482 -0.1311007 0.430996237 0.003296869 -0.111069078 0.263281925 -0.16248499 0.131428214 -0.275981945 0.334412069 -0.296247519

-0.125726452 0.312011433 0.302722232 0.095637604 -
0.430461799 -0.252200775 -0.489934046 0.205593616 -0.087933214
-0.261064298 0.311805251 0.242980667 0.206152254
0.19908193 -0.360389448 -0.001828249 0.075953264 -
0.321123326 0.017232473 0.222673047 0.172006462 0.412286162
0.172560696 0.091407497 0.228218667 0.257342884
0.281956005 -0.004233875 -0.106156672 -0.412413091
0.099601968 -0.193043931 0.033268045 0.490064554
0.121785033 0.09684375 -0.225658875 0.101457969 -
0.266831327 0.303599161 0.034874527 -0.233276626 -0.348378246
-0.27018513 0.282934284 -0.390499086 -0.011672003
0.046655596 0.203676893 -0.427904483 -0.209628641
0.133351109 0.291334161 -0.241481574 -0.243775513
0.139957016 0.212039031 0.283850754 -0.179659006
0.285601319 0.445480764 -0.27522712 0.096858239 -
0.248118651 -0.077526175 0.125445386 -0.366683546 -0.431887319
-0.182568956 0.216705615 -0.05794451 -0.137687394 -
0.0775829 -0.155314596 -0.281297896 -0.397621014 0.301594329 -
0.197061592 -0.029178503 0.361402609 -0.312325622 -0.09048095
-0.052863087 -0.460675898 0.376921017 -0.388373935 -
0.018818818 0.238998167 0.109907258 0.120320389 -0.230249428
0.05904597 0.030979314 0.251635374 -0.059867684 -

0.363583225 -0.113409737 0.322841482 0.483520956 0.2851573
0.105648239 -0.284522562 0.099179199 0.034496241
0.117083123 -0.028276762 0.224653541 0.349605668 -
0.025463719 -0.110642939 -0.134977143 0.177557489 -0.37077643
0.026995939 -0.030344446 0.364214164 -0.001270587 -
0.370770715 0.027046455 0.271557908 -0.020734139 0.273219565
0.135026731 -0.436038477 -0.059123111 -0.024783193
0.102727275 -0.156259158 -0.255119552 0.127356867
0.012729149 0.478702761 0.238585658 0.207861 0.435008222
-0.028751894 -0.248912324 0.079999817 -0.178272397
0.299863193 0.041877182 0.382027831 -0.084500054 -
0.374158785 0.129759956 0.017265403 0.300664202 0.090415936
-0.049306366 -0.232223806 0.347275754 0.051139496 -
0.01448536 0.194268635 0.241801823 0.125423588 -0.113949344
0.336636587 0.176585665 0.120024005 -0.103044601
0.000198029 -0.422485644 0.277178728 -0.385404398
0.006569442 -0.131845379 -0.37017242 -0.327997376 -
0.336339641 0.02857753 -0.211182081 -0.016892734 -0.154011661
0.085022843 0.123883007 -0.461622403 0.035819942
0.199382506 -0.347074467 -0.221446347 -0.096306614 -
0.427286914 0.445567241 -0.193206335 0.230783686 0.413242804
-0.150124053 0.305321071 0.408701246 0.200014634

0.151372582 -0.060627411 -0.22246252 0.022668743 -
0.040478508 0.030667104 -0.29145129 0.025224822 -0.515242291
-0.005644598 -0.481403297 -0.296780766 0.274247383
0.032985341 0.047105487 -0.034619788 0.14636336 -
0.322901283 -0.184889557 0.031641006 -0.056501775 0.039462842
-0.168762681 -0.270172658 0.102711913 -0.161603435 -
0.440315335 -0.337247038 -0.054733613 0.170700787 -0.090720078
0.005430464 0.117271187 -0.150318299 0.105994665
0.348259572 -0.032444016 -0.253758437 -0.218491731 -
0.018875826 -0.219235632 -0.452267921 0.273111764 0.093710678
0.037963848 -0.063750284 -0.284500987 -0.141016422 -
0.244002909 0.227019517 -0.365934012 0.459352467 -0.204547146
0.364855174 -0.154092486 0.02704761 0.154411547 -
0.054569004 -0.103079429 -0.376342543 0.221198815 0.352705741
-0.347026586 0.158465071 0.437782961 0.431479956 -
0.134536866 -0.227765391 -0.291710696 0.064236633 -0.123373948
-0.347871231 0.306965064 0.281191169 -0.182584052 -
0.306027211 0.062582519 -0.367523622 -0.096935497 -0.252691631
0.070946053 -0.061241271 0.204061356 -0.1179741 -0.383506349
0.212721696 -0.190982524 -0.023723162 0.213725462 -
0.375535253 0.093376571 0.114018053 0.344253092 0.16311571-
0.283406469 -0.302057232 -0.101672573 -0.04052996 -0.312197673

0.159515339 -0.118174517 -0.040784177 0.119485708 -
0.381544739 0.199118589 0.046573942 0.005034448 -0.233417031
-0.133264728 -0.081754503 0.354646821 -0.070234806 -
0.305720239 -0.032849414 -0.076241933 0.250205049 0.483053468
-0.254917862 -0.068294678 -0.392396949 0.308610843
0.110328966 0.383183396 0.361983734 -0.119637078 -
0.393396292 0.322751 -0.002284941 -0.330390892 0.377880384
0.20575424 -0.193189643 -0.325516995 -0.029231825 0.2814819
0.509844158 0.199969893 0.170927966 -0.072339911
0.030250604 0.110946344 0.135292491 -0.282309576 -
0.065077871 -0.112104211 -0.02999138 -0.098856732 0.330655703
-0.380459439 -0.020349533 0.257932052 -0.203968386
0.109252225 0.211466703 0.175544698 0.012903498 -
0.102883174 -0.173377348 -0.085190304 0.359274762 0.084537879
-0.405642086 0.556487193 0.109470041 -0.419652212 -
0.174146684 0.393102984 -0.262429407 -0.296361745 0.25226344
0.219853994 -0.109821583 -0.364193089 0.007321623
0.112913522 0.292872816 0.176183143 0.039072185 -
0.166522257 0.243374384 -0.090502659 0.213501629 -0.420849411
-0.266209512 0.011438724 -0.15268756 0.267178651 -
0.015062289 0.298377558 0.451823023 -0.402372413 -0.020812431
-0.12599445 0.169990042 -0.39521817 -0.16013879

0.046513659 -0.127647164 -0.100097896 -0.046073777
0.282031144 0.139534826 -0.003299261 0.240219068
0.193014181 -0.104097511 0.151943199 -0.015065502
KEGG_PHENYLALANINE_METABOLISM 0.32028123 -0.19404385 -
0.412271361 -0.44731036 0.53736636 0.086408149 0.218349
0.41254389 -0.134776643 -0.30308337 -0.541655762 -
0.065634021 0.056402797 -0.287414943 0.264623 0.546212326 -
0.11649598-0.239442931 -0.138967734 -0.043962519 -0.426346859
0.482007744 -0.587264599 -0.431479325 0.178515642 -
0.088520918 0.077948843 0.086797894 -0.467754496 0.10440008
0.606585414 -0.343202515 -0.148879404 0.373743409 -
0.182612188 -0.364403596 -0.199232781 0.495691129 -0.32925024
-0.091004253 0.138762601 0.499277519 -0.529203784
0.203860395 -0.363440141 0.297781468 -0.092020169 -
0.065997472 -0.170597518 0.419952531 0.036197449 -0.395650287
0.109555684 -0.42382276 0.305865027 0.146038765 -
0.002190798 0.352024625 0.333005002 -0.095563912 -0.473580075
-0.494692091 0.022551007 -0.474999967 -0.316247246 -
0.439077346 0.504071579 0.14441487 0.25769905 -0.000926053
-0.527372019 -0.557498449 -0.036373409 -0.004438113 -
0.214647171 0.345003251 0.280636945 -0.186343186 0.56599132
0.490839735 0.256649588 -0.448139684 -0.188041524 -

0.058525234 -0.019029694 -0.528672943 -0.55907799 -0.135030498
-0.211554664 0.134876421 0.066535563 0.365437528
0.277852856 0.339146321 -0.512689082 -0.357970817
0.015285263 -0.484806167 0.588240712 0.193539394
0.207634788 -0.086378335 -0.348511846 0.437656894 -
0.081629822 -0.397275059 0.172966823 0.477019073 -0.502986675
0.141495722 0.168092753 0.323306543 0.459784377 -
0.412060765 -0.013316536 -0.283152643 0.136221296 0.311265999
0.093106177 0.287257554 0.370497707 0.403972606 -
0.009737869 0.246846084 -0.031056147 0.478839831 -0.020115075
-0.518440842 0.404094161 -0.268717733 0.186471304 -
0.244416333 -0.015550415 -0.143560895 0.066800904 -0.584197484
-0.193119831 -0.176613164 -0.059526813 -0.379141622
0.066341803 0.39650431 -0.424713646 0.202503837 -
0.481786296 0.100703329 -0.080735899 0.188677604 0.129934481
0.349584254 0.007137165 -0.465323492 -0.539566712
0.216949595 -0.496642895 0.362772175 -0.45139026
0.522465335 -0.365505989 0.216127815 -0.10540577
0.590368881 -0.077350943 -0.048709812 0.450052459 -
0.248126615 0.310792576 -0.200050056 0.383457432 -0.478512606
-0.090734022 0.168125428 0.217679554 0.239701034 -
0.361592339 -0.398660997 -0.559066842 0.069837233 -0.247390882

-0.072139032 0.379153596 0.067063684 0.067445115
0.289898532 -0.318668533 -0.110363527 0.209981498 -
0.335647672 -0.095999568 0.257555623 -0.121098296 0.560383081
0.263049829 0.077139387 0.274244655 0.522270284
0.238042996 -0.220609882 -0.190768077 -0.509260563
0.350511651 0.006398506 0.011983613 0.507796193
0.298136229 0.290640328 -0.304760205 -0.320226632 -
0.187362046 0.602621087 0.003968956 -0.180122848 -0.28550795
-0.413825012 0.494635705 -0.486183308 -0.040510694
0.290999025 0.222153441 -0.5535688 -0.122548269 0.278968373
0.356552191 -0.481563092 -0.464645707 0.484672457
0.466420245 0.144049614 -0.027331765 0.438090487
0.646220119 -0.134268885 -0.122215972 -0.219367068 -
0.200503154 0.089346881 -0.198179708 -0.671050373 -0.044924066
0.248309121 -0.213466462 -0.320288241 0.242763042
0.062674502 -0.381590893 -0.369259293 0.322020979 -
0.294644128 -0.149842105 0.157150498 -0.370495036 0.103976954
-0.166301063 -0.626286517 0.202969443 -0.439737185
0.107683392 0.18651559 0.072480764 -0.071478379 -
0.591538584 0.331749246 0.395735863 0.326592325 0.139579686
-0.416998262 0.149113507 0.360466829 0.465622885
0.437346921 -0.141451822 -0.513159954 -0.176008133

0.06012164 -0.27386959 -0.009008111 0.29467386
0.363672277 -0.353125193 0.010341874 -0.359899969
0.281104402 -0.654486621 -0.022390159 -0.221657247
0.520269291 0.100853932 -0.448583242 -0.041578148
0.026362147 -0.02760845 0.403496895 0.010261908 -
0.401137485 -0.260262243 0.0530405 -0.249604115 -0.57721661 -
0.265865902 -0.09186756 -0.104064888 0.547538672 0.255491472
-0.018274621 0.666259323 0.228715705 -0.450851139
0.147065039 -0.217157199 0.173180786 0.185236488
0.335250623 -0.099314534 -0.476241007 0.308782723
0.244172389 0.330812476 0.089459176 0.035619242 -
0.236795301 0.250027629 0.278984099 0.27470203 0.435126296
0.157459698 0.334358722 -0.219307953 0.540845753
0.231030902 0.305147387 -0.345989267 0.050277171 -
0.546048386 0.670520886 -0.490264978 0.187179653 -0.31648554
-0.329053795 -0.347234168 -0.158068826 -0.195312501 -
0.195174619 -0.162459468 -0.158491291 0.209282279 -0.095338492
-0.567646502 0.145715299 0.333191922 -0.300444967 -
0.514766644 -0.028845318 -0.492633363 0.306031963 -0.006785566
0.424766955 0.343391204 0.017013563 0.482887388
0.392397122 0.048873651 0.011254293 -0.151993457 -
0.257787387 0.252226405 -0.306503665 -0.199010962 -0.273980873

0.053123678 -0.563965697 -0.019049978 -0.551065101 -
0.079725125 0.251882492 -0.100356082 0.099774347 -0.246947673
0.219001041 -0.201791784 -0.548208358 -0.060852436
0.044219183 0.177527365 -0.114131023 -0.089927353
0.083105577 0.021171642 -0.371989125 0.101451678 -
0.066446809 0.195951594 0.332408696 -0.090036627 0.416901167
-0.139705099 -0.014737097 0.354552494 0.056461431 -
0.32301317 -0.139712232 -0.376457452 0.00175812 -0.427789093
-0.209164439 0.305431581 -0.170992684 -0.308517027 -
0.039835321 -0.022052663 -0.087730168 0.283817396 -0.484061359
0.574855887 -0.071530311 0.400610631 -0.257133363 -
0.079205785 0.383842833 -0.092004314 0.11678067-0.46600328
0.39021291 0.214610751 -0.355623817 0.306604083
0.264996022 0.628414666 -0.247933902 -0.035788726 -
0.064028119 0.126292252 -0.058398602 -0.243285814 0.156263471
0.177822032 -0.299987867 -0.154693736 -0.01891158 -
0.496033569 -0.116045786 -0.383586954 -0.177098344 0.268336173
0.197379652 -0.259636508 -0.424524127 0.142659964 -
0.216747005 -0.019413897 0.248413149 -0.581845146 -0.044571776
0.229198998 0.331638851 0.156667283 -0.429798719 -
0.426495547 -0.070074756 -0.320331751 -0.210436433 0.413196133
-0.038599037 0.055594917 -0.004081296 -0.515534418

0.383185605 -0.030740181 0.024943358 -0.321473976 -
0.221445609 -0.271216734 0.364752801 -0.234115526 -0.284394669
-0.132876094 0.004258617 0.229687953 0.537368828 -
0.3288272 -0.034364353 -0.419309693 0.347554348 0.061231665
0.327654110.394422118 -0.121200401 -0.414868874 0.369293063
0.131690162 -0.389219575 0.324165009 -0.052737437 -
0.368307984 -0.555478205 -0.181869958 0.377959545 0.567035964
0.472110369 -0.265122828 -0.015995452 0.30508776
0.049892637 0.388256637 -0.174254772 -0.379135121
0.041766295 0.256843444 -0.293480524 0.555301833 -
0.456701548 0.176012279 0.052228752 -0.30494985 -0.10278657
0.156565305 0.256500569 0.233350888 -0.035261218 -
0.113389221 -0.360636869 0.423922375 -0.098009589 -0.182107473
0.469486259 0.261866009 -0.537152482 0.311383208
0.337197226 -0.191514936 -0.443643188 0.293427099
0.229677272 -0.031898137 -0.223306141 0.201489945 -
0.103977279 0.012768296 0.243626693 -0.299850809 -0.429553976
0.319655994 -0.324674678 -0.062721455 -0.215822409 -
0.28976382 0.09781974 -0.258647787 0.075420511 0.10230161
0.339913689 0.378804937 -0.40493812 0.098462733 -
0.318535425 0.125288956 -0.135293309 -0.116399477 -0.13613292
-0.03800012 -0.048544674 -0.026230622 0.356061362

0.143239626 0.121026208 0.113492873 0.246623656 -
0.431535043 -0.079659657 0.241047406
KEGG_TRYPTOPHAN_METABOLISM 0.451644702 0.486223086 -
0.370166144 0.067776109 0.310849086 0.283245328 0.155620677
-0.081785845 -0.165052752 -0.176619631 -0.128820343 -
0.097165528 -0.216033571 -0.431712081 -0.10992097 -0.04790763
-0.128961147 0.091981894 -0.321572318 -0.097422324 -
0.402830865 0.332168275 -0.553998367 -0.264306655 -0.30159046
0.061178831 0.329326739 0.299368869 -0.198548142 -
0.289214751 0.366854953 0.006271561 0.349285403 0.335995991
-0.407472366 -0.395799103 0.111198799 0.248304884 -
0.203834584 0.301147614 -0.09472461 0.424734029 -0.212645437
0.553872799 -0.358931824 0.412830606 0.124288201 -
0.110113305 -0.114509951 0.092300805 -0.00594867 -0.355217985
-0.07844973 -0.289756283 0.197047782 0.357878286 -
0.289430951 0.262891724 0.384803685 -0.13852807 -0.516735977
-0.176148529 0.065318389 -0.204712468 0.002475775 -
0.200361194 0.143667929 0.059703901 -0.250147964 -0.014132302
-0.386425406 -0.3674011 0.02243165 -0.188624167 -0.013457951
0.097412074 0.011856624 0.226895318 0.316935829
0.57864781 0.193342205 -0.180532582 0.242928119 -
0.102936937 -0.130267166 -0.407627817 -0.512007852 -0.334143917

-0.287075749 0.447074957 -0.32963216 0.035665613

0.231849996 0.371788181 0.324741666 0.039435799

0.203283846 -0.385908069 0.501057736 0.191506094

0.213415584 -0.068369482 0.163249679 0.404362155 -

0.206257594 -0.121895544 0.317462805 0.404484588 -0.48424158

0.001232564 0.036866985 0.237049584 0.389251295 -

0.51209297 0.246224905 -0.124590213 0.225750914 -0.055840485

-0.109031417 0.280979866 0.267630923 0.462722543 -

0.085217232 0.143508172 -0.172187927 0.222565415 0.066507452

-0.466192537 0.453378167 0.210107467 -0.025593028 -

0.289973083 -0.228492554 0.209915021 0.078299503 -0.336089537

-0.49790022 0.391722508 0.19681231 -0.465893358 -

0.099439683 0.349670373 0.015823312 0.164287978 -0.338031205

-0.352953154 0.293791784 0.410980575 -0.013443142

0.086599519 -0.193735283 -0.298718322 -0.446744756 -

0.195662365 -0.047344609 0.085753621 -0.45918732 0.498357271

0.33859984 -0.043340368 -0.13997046 0.25457501 -

0.05408347 -0.354362167 0.497237313 -0.440984149 0.271316461

0.025929153 -0.01519327 -0.336442562 0.00503502

0.430574983 0.194614286 0.197011904 -0.341280636 -

0.529851509 -0.303908444 0.608755416 -0.329863699 -0.351613333

0.254987992 0.081888989 0.392933803 0.201199326 -

0.287219641 0.188617352 -0.439373422 -0.137173934 -0.386900203
0.497297261 -0.325918241 0.548891438 0.480328596 -
0.048427744 0.281894398 0.145062383 0.189471068 0.31601312
-0.072433846 -0.039180959 -0.064700997 0.252861134 -
0.112850199 0.542020685 -0.191961483 0.189198521 -0.277941066
0.114542829 -0.3330414 0.267583949 0.49515172 -0.252120858
0.253381093 -0.359948597 0.36466595 -0.12149263
0.222238912 -0.095365139 0.404387316 -0.368613746 -
0.364799076 0.487998391 0.04579657 -0.501689943 -0.545849489
0.171179892 0.00365646 -0.063823442 -0.017288379
0.422888803 0.333349018 -0.151414152 0.228126169 -
0.163419731 0.135211018 0.104855056 -0.439335521 -0.224401512
0.142330451 0.093895549 0.088509898 -0.32601557
0.266813862 -0.294141661 -0.517738042 0.099297949
0.129763688 -0.165873264 -0.071045974 0.129196277
0.147505478 0.107433327 -0.281497023 -0.568465695
0.08055104 -0.383525522 0.500289396 0.074758775
0.476323618 0.256393464 -0.332793583 0.321320874
0.438818498 -0.031097806 0.099811952 -0.432831359 -
0.354554407 -0.03759718 0.123183978 0.177305803 0.459883388
-0.594156664 -0.132038304 -0.031527155 0.031859314
0.275387025 0.368347201 0.177311144 0.025736767 -

0.158074801 -0.396523691 0.043280619 -0.489085241 -0.108177708
-0.317706834 0.32469868 -0.296189305 -0.103815146 -
0.20864417 0.005445325 -0.081472164 0.350199645 0.205363573
-0.300808977 -0.367608847 -0.12047218 0.255528241
0.131598306 -0.364936512 0.282156583 0.167471785
0.412143481 0.230074492 -0.042858423 0.541354177
0.016546793 -0.344795137 0.309236343 -0.426985884 -
0.067613193 -0.19352919 -0.010855349 -0.1071218 -0.158188037
0.297449254 0.183991728 -0.253771157 0.06864316
0.134796996 0.248564144 0.060110286 0.260530554
0.442039711 0.182734998 -0.067121178 0.0465816 -0.123412252
0.393502708 0.511090426 0.121097674 -0.271469991
0.297342638 -0.436387055 0.54184352 -0.33811081
0.206529128 0.192809557 -0.480375875 0.006346285
0.206797086 0.455251239 0.075678802 -0.169104036 -
0.292151605 0.373848678 0.104180059 -0.37460683 0.418666885
0.270983839 -0.38241399 -0.351266084 -0.163389039 -
0.394236026 0.514640554 0.234793859 0.067238295 0.178745871
0.375524842 0.422881134 0.154212837 0.267261272
0.087423133 0.212951548 -0.306038615 0.067444293 -
0.286872712 -0.313755662 -0.238712898 -0.308457854 -0.477056688
-0.221105265 -0.50527841 0.182271333 0.164692199

12572 0.295546694 -0.304688112 -0.41013584 0.26223386 -
12573 0.387755386 -0.233713766 -0.338971169 -0.269276928 0.191878205
12574 -0.05245128 -0.304963656 0.459093056 0.019379558 -
12575 0.358861679 -0.035423313 -0.371673112 0.082148149 0.52485911-
12576 0.092434979 0.405941728 0.007268297 0.060028994 0.45268117-
12577 0.423285507 -0.201000261 -0.068367534 -0.117334423 0.240691507
12578 -0.212263595 0.446073306 0.071428433 0.209028855 -
12579 0.025565605 0.134061841 -0.182327139 0.10027601 0.402210226
12580 -0.131842424 0.496125073 -0.440684254 0.143625728 -
12581 0.177177945 0.331505662 0.476365386 0.204968695 -0.205665413
12582 -0.406677441 0.213930431 0.258295645 -0.274015477
12583 0.189923071 0.05104785 0.587443604 -0.128700137 -
12584 0.442097046 0.094615479 0.071166785 0.315192509 -0.430478048
12585 0.338647726 0.165767175 -0.345550245 -0.007580862
12586 0.003238098 -0.449974401 -0.454122383 -0.060290157 -
12587 0.458779928 0.27725799 -0.009461795 -0.283345133 0.01492079
12588 0.069675637 -0.493201793 -0.494499582 0.104536048 -
12589 0.459157015 -0.009068468 0.325128534 0.124578449 0.42254151
12590 -0.364117 -0.301115905 -0.365099471 0.034687202 -0.044033597
12591 0.473017527 -0.133782741 -0.211815733 -0.032737642 -
12592 0.429189325 0.176986786 0.005124018 -0.22230256 -0.007704371
12593 -0.029293749 0.076070129 0.350708273 0.208591592 -

0.540798951 -0.49745905 0.199987509 0.197791579 0.286692605
0.216973764 -0.301232691 -0.219039568 -0.004150146
0.406162424 0.201027473 0.186671681 0.289604521
0.102715993 0.292740464 -0.193050204 -0.451625559 -
0.290220305 -0.101646566 -0.267651324 -0.261580026 -0.004570892
0.089803034 0.396433661 0.287007869 -0.025875655
0.390513042 0.265253127 0.377558031 0.356952428 -
0.417468345 -0.404241452 -0.177866447 0.096480522 -0.099393388
0.3223196 0.003739478 0.150166007 -0.058499118 -0.417275904
-0.083351522 0.445313396 0.240507159 -0.105672868 -
0.210568237 0.06274522 0.21003203 0.470526081 -0.358205869
-0.13528836 0.423020832 0.117047866 -0.478824457
0.170090867 0.086637221 0.062663697 -0.495847937
0.261996755 0.233487119 0.280489331 -0.321680793
0.137245007 0.06831091 -0.165156742 0.298034638 -
0.389843925 -0.486088486 0.276069032 -0.339243786 -0.085967922
0.117876686 -0.308071721 0.373214638 -0.342882536 -
0.049460059 0.254780912 0.537260309 0.067310802 -0.200782592
-0.233382941 -0.477674126 0.052716879 -0.177349202
0.23219329 -0.371527165 0.294190431 -0.37450547 -
0.2533682 0.219561985 -0.03465234 0.201915943 -0.285129337 -
0.162443849 0.047295249 -0.295051148 0.386339177

KEGG_BETA_ALANINE_METABOLISM 0.692921628 0.604849809 -
0.390269788 0.02432225 0.289903263 0.295500823 0.154912433
-0.204125262 -0.383623293 -0.448512611 -0.32879106 -
0.101948798 -0.322546205 -0.551120141 -0.210964576 -0.191115016
-0.220544644 0.280716892 -0.504336345 0.061763028 -
0.338805566 0.489302325 -0.612380352 -0.118320238 -0.375044473
-0.292547165 0.160988979 0.382688037 -0.555924661 -
0.430601639 0.516575762 -0.321856542 0.430642164 0.470368025
-0.419301805 -0.291085754 0.176944317 0.276176629 -
0.405511448 0.391971674 -0.26894365 0.258904624 -0.153172598
0.5344593 -0.476459152 0.357221931 0.312637476 -0.346700703
-0.281903474 0.206786655 -0.190984532 -0.370900802
0.394974109 -0.176564022 0.346615454 0.496633148 -
0.414997092 0.369718986 0.425082494 -0.119824454 -0.552725008
-0.18142249 -0.120669271 0.108659516 -0.276006285 -
0.385101232 -0.038910513 0.112389375 -0.42820542 -0.196740482
-0.451895193 -0.478295408 0.021188704 -0.264738112
0.001242857 0.016508272 0.393930686 0.359689438
0.119718655 0.606206038 0.140060688 0.191488055
0.161808749 -0.18118205 -0.276412208 -0.401666461 -
0.610502685 -0.380548325 -0.477396773 0.479676585 -0.28776603
0.005488366 0.211406540.420623283 0.216160233 -0.006610612

-0.12186487 -0.461724453 0.579485974 0.203169097
0.14735736 -0.21356184 0.32455746 0.509056593 -
0.29266055 -0.480387755 0.420978025 0.391285631 -0.326457488
-0.170283528 -0.077360852 0.003329799 0.496275027 -
0.593385751 0.080564076 0.042608624 0.047345743 -0.41172629
-0.296432393 0.069296145 0.152390531 0.437827462 -
0.19851976 0.05135286 -0.144085188 0.247817899 0.01325789
-0.445424374 0.540626668 0.091706053 0.286073192 -
0.377014387 -0.075638453 0.290075334 0.192941365 -0.527679006
-0.545873239 0.506601117 0.144321814 -0.371117521
0.135085902 0.26945244 0.249549939 0.30789675 -
0.31674971 -0.43989049 0.420221822 0.496342248 -0.264025001
0.166076011 -0.227679494 -0.347381521 -0.482496639
0.148523712 0.028507786 -0.001687232 -0.587983124
0.460572228 0.399906517 -0.24563142 -0.395604193
0.288420129 -0.285771682 -0.381992038 0.646856711 -
0.494526317 0.351441192 0.192946028 0.048046086 -0.41053143
0.396593324 0.537298501 0.277912322 0.164761599 -
0.270155875 -0.464958146 -0.500139156 0.541417489 -0.152407589
-0.434043353 0.167665228 -0.209886783 0.521530524
0.341502326 -0.157752788 0.100427493 -0.211933156
0.034054785 -0.555286292 0.480905782 -0.17148594

0.509178723 0.560669918 0.138604341 0.114397389
0.332212939 0.335000553 0.254600291 -0.195267392
0.130287298 -0.270597738 0.400213381 0.04542673
0.522677514 -0.17212764 0.054214454 -0.385141711
0.282494874 -0.143902182 0.362297754 0.587625239 -
0.036468571 0.21595735 -0.345036097 0.272723901 -0.176364618
0.380938677 0.20762993 0.25258831 -0.49705763 -
0.350878572 0.539616497 0.329043491 -0.462667488 -0.412739435
-0.019890845 -0.12461639 -0.190138937 0.290496244
0.423291934 0.381402743 -0.410035827 0.479357985 -
0.14089778 0.179787035 0.361750928 -0.329785994 -0.018213421
0.216964404 0.248311842 0.090220521 -0.11431951
0.340854427 -0.315384678 -0.53289208 0.084449261
0.219043764 -0.380011314 -0.009915655 -0.046830476
0.134381302 0.188778977 -0.297298554 -0.65587346
0.11314759-0.459720462 0.471240384 0.055270952 0.512370931
0.187341246 -0.561317993 0.299916244 0.312015136 -
0.069823529 0.320497903 -0.49978758 -0.143316185 -0.11474129
0.250598809 -0.182770612 0.441208172 -0.467043239 -
0.12172341 -0.091799951 0.184096825 0.379205692 0.209777628
0.167546799 -0.271861354 -0.046374512 -0.353964556 -
0.163190268 -0.500118367 -0.283560998 -0.566568513 0.198766005

-0.296709127 -0.042988581 -0.288078263 0.138497033
0.393447986 0.457846365 0.325968906 -0.376562826 -
0.458124591 0.025868899 0.436468177 0.129913285 -0.417863662
0.250738007 0.183879894 0.359637724 0.448735589 -
0.042198239 0.651253334 -0.160317739 -0.432268093 0.427317167
-0.537615095 -0.325189037 -0.190107159 0.1768297 -0.312213583
0.305159452 -0.116536042 0.343002702 -0.123499745
0.351922279 0.581153779 0.258810505 -0.278801431
0.399433334 0.433770154 0.066100308 -0.15560304 -
0.013330935 0.042693983 0.194216112 0.618283219 0.198895295
-0.37393803 0.470647397 -0.322504166 0.620637888 -
0.148027457 0.421101528 0.397414825 -0.429119822 -0.013179373
0.453146214 0.3923426 0.14470507 -0.357443118 -0.215194378
0.307986954 -0.111708827 -0.467985122 0.590125332 -
0.07549946 -0.443626289 -0.52582876 -0.183653767 -0.400505984
0.441000752 0.427124406 0.064118898 0.076890732
0.467602447 0.30571873 -0.338949473 0.307738459
0.163130525 0.493187546 -0.431007572 0.127791314 -
0.265371237 -0.140579949 0.004865431 -0.012874749 -0.309418313
-0.549215403 -0.546119037 0.343876515 -0.152959237
0.373663624 -0.256225758 -0.506921469 0.285183032 -
0.27170833 -0.13503897 -0.251058164 -0.290189462 0.097159916

12704 -0.340780461 -0.268486824 0.627808095 0.070722277 -
12705 0.382941841 0.266099178 -0.286066093 0.094655175 0.404646366
12706 -0.101405734 0.346768873 -0.208182831 0.069360945
12707 0.59891961 -0.269868342 -0.419261882 0.213379851 -
12708 0.101769611 0.041469782 -0.177957644 0.512383172 -0.113337465
12709 0.302333421 -0.006690995 0.022445357 -0.298654641
12710 0.236164957 0.497616110.014634018 0.533645182 -0.483811719
12711 0.111778053 -0.258096532 0.266211406 0.33153991
12712 0.139035087 -0.246554538 -0.546103541 0.225140716
12713 0.237631733 -0.171162211 0.186662089 0.043031153
12714 0.520111669 -0.133679576 -0.642236833 0.204082911 -
12715 0.279767623 0.387479767 -0.404539632 0.34303821 -0.125471148
12716 -0.516108865 0.203079477 0.203585159 -0.391730978 -
12717 0.339899869 0.157728982 -0.504227316 0.321994309 -0.116331004
12718 -0.284294069 0.107206165 -0.294508002 -0.553455805 -
12719 0.449086068 0.378290103 -0.562715145 0.020957497 0.267360239
12720 0.09401563 0.349618347 -0.106267848 -0.393148405 -
12721 0.376585094 -0.240224887 -0.282936894 0.5758816 -0.213169151 -
12722 0.439474801 0.058754861 -0.198059494 0.268899928 -0.229794318
12723 -0.176932477 0.025826906 -0.229384883 0.14282139
12724 0.123836716 0.386934979 -0.391972755 -0.430539142
12725 0.020999953 0.209483071 0.397243066 -0.109683582 -

0.507372337 -0.066972942 -0.184810624 0.439682651 -0.364541999
0.459532273 0.457410577 -0.105726322 0.237062347 -
0.059856823 -0.496094246 -0.474133393 -0.285093636 -0.18285684
-0.302183569 -0.172071479 -0.011559447 0.234433526
0.270858825 -0.38678472 0.46947739 0.244320604
0.560358618 0.550131018 -0.094828059 -0.511802463
0.029407169 0.262405096 -0.186726134 0.361520239
0.043325548 -0.0874121 -0.223370894 -0.497183061 -0.335282925
0.679069635 0.388853786 -0.035377477 0.006836688 -
0.040161121 0.377290892 0.510234829 -0.424208022 -0.109241313
0.41377089 0.174250014 -0.401015898 0.207565071 -
0.140667137 0.368446629 -0.529677245 -0.051713225 0.124786327
0.393756972 0.011167817 0.201936193 0.203859298 -
0.089314995 0.365065463 -0.335620178 -0.472572857 0.442593398
-0.581907134 -0.262540306 0.057528457 -0.326278767
0.210627343 0.114793127 -0.063985491 0.063530912
0.564798044 0.012875175 -0.25271107 -0.077886297 -
0.437733799 0.025473636 -0.333224583 0.397326861 -0.15811758
0.220411535 -0.455105765 -0.200074624 0.089140073
0.155815203 0.017141813 -0.274862749 0.05688486
0.022168014 -0.297205305 0.503634071
KEGG_SELENOAMINO_ACID_METABOLISM 0.451011380.366663967 -

0.213695251 -0.128541403 -0.031421057 -0.210204354 0.051682374
-0.306344613 0.379531559 -0.004045128 -0.286610691 -
0.02483126 -0.437282307 -0.029752051 -0.289088525 0.072991005
-0.152901003 -0.020144497 -0.505193699 -0.306270124 -
0.125290119 0.009392919 -0.567314546 0.301332816 -0.390741806
-0.134339673 0.209870343 0.296990639 -0.142574449 -
0.211439080.367550941 0.165742994 -0.196692021 -0.221368893 -
0.316791838 -0.371816154 0.007991202 -0.096111678 -0.429155996
0.357401488 -0.125176168 -0.161822635 -0.060075102
0.280336537 0.029461866 0.013902208 -0.032787742 -
0.176198156 0.413465587 -0.015496684 0.129280125 -0.026435666
-0.013749969 -0.080820866 -0.103592375 0.424039831 -
0.434039541 0.236966816 0.03771099 0.078395115 -0.444081484
-0.098713804 0.189361905 0.341285822 0.009130328 -
0.318213994 -0.015685511 0.451604738 -0.208727109 -0.50026711
0.223054583 -0.293165808 0.078241929 -0.013563314
0.328966139 -0.214450733 -0.266251084 0.330356078
0.18197417 0.409207599 -0.026413971 0.055234043
0.29176765 -0.068437799 0.189376481 -0.134387264 -
0.035256051 0.178656911 -0.509343352 0.41905182 0.223180502
-0.310640023 0.078134721 0.427736622 0.245766709
0.138619574 0.397032464 -0.305120033 0.15800945

0.602797045 -0.190615058 -0.401428037 0.194876662 -
0.062131743 -0.29253578 -0.002406977 0.049779204 0.452927264
-0.313609446 -0.415111975 -0.508911718 0.194418475
0.061614452 -0.124478979 0.172534562 0.278010642 -
0.271441769 -0.230417199 -0.390738604 0.305409323 -0.445689439
0.108848689 0.182956146 0.098122954 -0.433191073 -
0.37817444 0.364647735 -0.231167504 0.146894244 0.207487599
0.127054843 -0.259835252 -0.075311384 0.254475813
0.270937757 -0.434324532 -0.401226244 0.374157738
0.002650284 -0.090051448 0.161019169 0.092195389
0.152967417 -0.074553599 -0.42985143 0.148103768
0.193328577 0.01623779 -0.352403053 -0.10240771 -
0.456733947 -0.161858979 -0.215415218 -0.352030304 0.056640102
-0.410012333 -0.393679006 0.082241097 0.365963811 -
0.149084474 -0.078548382 0.226308963 -0.108671277 -0.395135313
0.455859851 -0.347132716 0.013727578 0.316060486 -
0.418639424 -0.339565297 0.145372111 0.181956339 -0.000600891
0.068430908 -0.195708241 0.272334928 -0.030171228
0.43190287 -0.067444529 -0.272897111 0.174143448
0.37716632 0.044261303 0.170463031 -0.40074592
0.09233045 0.036024649 -0.049596494 -0.532589192
0.134419226 0.203617243 0.265921557 0.097435261 -

0.205530254 0.116677622 -0.001506186 0.278532537 0.377642569
0.048234419 0.311758496 -0.213809599 0.308804273
0.038331408 0.313167543 0.196015253 0.281563192
0.186890085 0.497394179 -0.167959285 0.583093393
0.439025421 -0.261116599 0.18097096 -0.115803762 -
0.338038317 0.163764431 0.380413389 -0.081892337 -0.233656154
-0.139641842 -0.365808974 0.415022226 -0.339821241
0.062948593 -0.321448691 -0.329733125 -0.186470795 -
0.28777757 0.258021253 -0.229906792 -0.026289949 0.017543005
-0.215555844 -0.350611396 -0.050237296 0.153152195 -
0.556752643 0.087625397 -0.073399228 -0.002043337 0.316705139
0.048050626 -0.312291168 0.100792549 0.146525558 -
0.246366962 0.313502709 -0.292138536 -0.277994789 -0.075679859
0.195958453 -0.067844218 -0.552826364 -0.406938028 -
0.036480414 -0.420437217 0.308689786 -0.282707426 0.448229928
-0.232411983 -0.308963237 0.276626404 -0.202164522 -
0.015424214 0.145892902 -0.222876079 -0.223516239 0.085369996
0.065802634 -0.371367977 0.442028466 0.201348636
0.528776154 -0.220167364 0.285965664 -0.020850843 -
0.150293792 -0.140502578 -0.250219939 -0.372838745 -0.453778279
-0.005598831 -0.090467457 -0.401183095 -0.408770244 -
0.386608413 -0.423218903 0.198764903 -0.226928386 -0.166804814

0.118591149 -0.025307045 0.185285988 -0.180544188 -
0.180762226 -0.135689474 0.356867058 -0.060636482 -0.420238788
-0.184426852 -0.013298452 0.58668013 -0.332066498 -
0.227889368 0.194106363 -0.058187581 -0.200458301 0.41884886
-0.371130221 -0.416271741 -0.153720067 -0.060664906 -
0.346241079 0.143096637 0.2340632 0.520442847 -0.322640924
0.291825381 0.331042259 -0.00249393 -0.187364942
0.344528378 0.040932652 -0.39436888 0.249089764 -
0.035013995 0.212260515 0.08435826 -0.037505248 -0.221080119
0.202186604 0.349721851 -0.251274798 -0.012587782 -
0.1123122 0.373599789 0.257056152 -0.369154581 0.11663192-
0.201963804 0.386826285 0.117039973 -0.331846436 -0.018536079
0.358832021 0.008713121 0.116210922 0.381480013 -
0.142281824 -0.463941634 -0.371603132 -0.253649295 -0.232334699
0.555925559 0.363082136 -0.268567441 -0.247704522
0.314066305 0.111758555 -0.025736214 0.224301692
0.07172179 0.385270955 -0.537091849 0.205989255 -
0.166329327 0.152095785 -0.085461984 -0.512200534 -0.32099966
0.116124019 0.099929002 0.143991396 0.109150881
0.254658438 -0.459466135 -0.35346857 -0.264825578 -
0.475078719 0.038298172 -0.347910917 -0.084648242 0.096453417
-0.510743872 -0.184157194 -0.013238238 0.259306605 -

0.195685663 0.023702861 0.143981301 0.128182865 -0.014034888
-0.012279546 -0.168420737 -0.229876103 0.208769861
0.288249445 -0.224853264 -0.17011275 -0.098967385 -
0.114327189 0.300036119 -0.081760273 0.5093268 0.010471161
0.292546784 0.168528219 -0.155681709 -0.123451682
0.323400806 0.433591632 -0.29687384 0.328583397 -
0.462064368 -0.344641207 -0.397372769 -0.080369359 0.160877655
-0.169994747 0.122458929 -0.144843088 0.253807069
0.510462077 -0.01505131 -0.468331789 0.638103111 -
0.027254794 -0.185171292 0.162769616 0.379750033 0.245775416
0.047669029 -0.362086642 -0.349621484 0.405874929 -
0.240482645 0.007522518 0.203358064 0.210815653 0.087943136
0.250782675 0.39812726 -0.216540601 -0.501771518
0.002289276 -0.049505501 -0.249081398 -0.49685436 -
0.38947784 -0.020704902 -0.03570257 -0.317634289 0.046144516
-0.210422659 -0.144199464 -0.247787971 -0.486387225 -
0.261508805 0.139974238 0.364752482 -0.079976801 -0.257044342
-0.389577404 -0.430787399 -0.228876889 -0.035630499
0.323472138 -0.442891195 0.227909233 -0.146057765
0.035665844 0.445915087 0.284398788 -0.327617868 -
0.064064991 -0.129734079 -0.35249781 0.250339451 -0.251097576
-0.152424257 0.039782563 0.007811208 0.142058412

0.119359707 -0.334869585 0.39837518 0.191703414
0.392381035 0.39931543 0.044777274 -0.260436101 -
0.349194733 -0.073673619 0.23944687 0.102394051 -0.15125944
0.170635888 -0.377684654 0.571722216 -0.010207397
0.322534923 0.377817692 -0.003150453 -0.381299564 -
0.031347312 -0.047330366 -0.464569876 0.127696592 -0.330458029
-0.218473547 -0.387092404 0.191809456 -0.223588707 -
0.160255875 0.444261227 0.192757221 0.067166508 0.041493831
0.263786693 0.068680492 0.486579614 -0.41609726 -
0.092250693 0.295061144 0.160904016 -0.277060837 0.360659075
0.10698691 0.047000801 -0.097407476 0.179712539
0.385255851 -0.069660229 -0.246982745 -0.022062125 -
0.223846284 -0.013238129 -0.10500811 -0.408640674 -0.405338504
0.284169662 -0.274493021 0.115945415 -0.151272241
0.071146065 0.500333119 0.054526882 0.114187336 -
0.177517847 0.438981988 0.243753438 -0.213947711 -0.139454272
0.033332239 0.131651939 0.120047825 0.156857438 -
0.238466311 0.013105549 -0.34729378 -0.046536554 0.233596142
-0.309443615 0.057319156 -0.01844165 0.244353084 -
0.401055261 0.02890056 0.012293906
KEGG_GLUTATHIONE_METABOLISM0.346220527 0.266858487 -
0.366456268 -0.231701528 0.235546001 0.035146554 0.099218709

0.381357884 -0.042269324 -0.097393224 -0.398163174 -
0.024847825 -0.258418357 -0.376197311 0.063220699 0.365963834
-0.325362698 0.156657647 -0.403581359 0.04207081 -
0.428301476 0.168322144 -0.387989702 -0.412186443 0.171731242
0.021683727 0.310242993 0.069031436 -0.43872273 -
0.142688287 0.126577413 -0.116109818 0.161145145 0.284514043
-0.0786691 -0.353054903 0.046627618 0.264946808 -0.401057798
-0.06890881 -0.053773883 0.230190469 -0.393243184
0.368855994 -0.254972201 0.199598368 -0.139391351 -
0.25611723-0.063425066 0.138293977 0.01342978 -0.317801362
0.24726726 -0.339798957 0.160402772 0.426581837 -
0.196946185 0.396003149 0.164428033 -0.264800317 -0.449913257
-0.385414299 0.266231684 -0.194717736 -0.377114675 -
0.289390599 0.063617779 0.260156138 0.013258474 0.111726019
-0.159451643 -0.312271847 -0.123248395 -0.161947083 -
0.0338898 0.020846722 -0.009495574 -0.218087965 0.243375205
0.419721363 -0.138428442 -0.439680013 0.193939893
0.334225714 0.088379534 -0.146826739 -0.420686052 -
0.427827049 -0.382495524 -0.141314808 0.165964889 0.300846677
-0.032948252 0.421583485 0.112488819 -0.188276154 -
0.074234817 -0.232474107 0.439203431 0.342446218 0.177858544
-0.226073937 -0.105670174 0.468405943 0.044080723 -

0.378729306 -0.146757206 0.348310085 -0.404541748 -0.069899296
0.063797845 0.420621865 0.383374159 -0.220242135 -
0.246180954 -0.211801378 0.367075989 0.027502112 -0.375633869
0.002541026 0.090806312 0.330612124 0.068857294 -
0.008308634 -0.586301521 0.069808391 0.394130646 -0.407483092
0.131430525 -0.03390168 -0.036807251 -0.381869386 -
0.215163339 -0.330890473 -0.165997911 -0.48117982 -0.395063164
-0.029636959 -0.035660626 -0.367384117 0.114441351
0.254287021 -0.076704214 -0.016587408 -0.158455939 -
0.268666429 0.146480737 0.323892807 -0.130478887 0.330163459
0.20240187 -0.374854183 -0.197821228 0.383504746 -
0.294483074 0.20986819 -0.600266194 0.318825256 -0.41026551
0.042313014 0.006033856 0.231737403 0.106424674 -
0.33746425 0.594436452 -0.210240532 0.389325938 -0.058369794
-0.001191889 -0.439916975 -0.195666401 0.15160979
0.120263938 0.239382898 -0.303874509 -0.117106914 -
0.501743101 0.034876666 -0.042385038 0.153071142 0.404127749
-0.111649886 0.015486942 0.152902675 -0.339104215
0.213581929 -0.191839773 -0.228760977 -0.14140164
0.001004709 -0.0933975 0.567325985 -0.041016644 -0.142740651
0.344074197 0.486066478 -0.192548057 0.06958432 -
0.113792164 -0.364932152 0.210651332 0.271489798 0.160836664

12924 0.532768643 0.217492429 0.299217282 -0.327790976

12925 0.420211083 -0.398552274 0.114508228 0.336442086 -

12926 0.255626714 -0.263290561 -0.413517404 0.327954527 -0.036518907

12927 0.314107367 -0.1378012 0.021137671 -0.520035561 -0.035398972

12928 0.152478569 0.1371336 -0.155454852 -0.443339917 0.074876022

12929 0.271630801 0.24941598 -0.224623689 0.451722778

12930 0.513711105 -0.118400132 -0.114366691 -0.149891242 -

12931 0.406026331 -0.284015087 -0.06769631 -0.474940079 -0.445566393

12932 0.098616606 0.155235156 -0.429654648 -0.016955976

12933 0.258050266 0.042680345 -0.283432275 0.076866199 -

12934 0.213952084 0.135179833 -0.289402242 -0.114206923 0.363235553

12935 -0.131977613 -0.503650514 0.372873197 -0.21444 0.21162012

12936 0.224953456 0.062597703 -0.217899956 -0.378920681

12937 0.386724996 0.05852671 0.309085379 0.380112193 -

12938 0.401062384 -0.089333524 0.407854103 0.269940348 0.296446479

12939 0.563582318 -0.300410552 0.152040054 0.15247031

12940 0.463743011 0.287054906 -0.082404101 0.371818001 -

12941 0.244170255 -0.051920474 -0.251512556 0.356499951 -0.170890041

12942 -0.066676978 -0.349559868 0.447754454 -0.389389143 -

12943 0.18702622 0.012023994 0.027842271 -0.094729284 0.384194663

12944 -0.209443422 -0.312408921 -0.050974511 -0.155613718 -

12945 0.048142038 -0.302504537 0.093812975 0.026650037 -0.229822022

0.311579265 0.215983508 0.245665743 0.526431702 -
0.22506778 -0.288717443 0.361442396 -0.241266076 0.111974655
-0.208741144 0.271886074 -0.208743664 -0.305924385
0.360696871 0.334762913 -0.21244981 -0.073316225 -
0.256698643 0.002809992 0.121279518 0.102712195 0.217809078
0.228961045 0.243194446 0.107434066 0.097957249
0.249003745 0.330590822 0.302901197 -0.368858137
0.056569545 -0.126254134 0.404480719 -0.450634107
0.218398266 -0.101819441 -0.03343882 -0.378953434 -
0.408347835 0.352075061 0.002997647 -0.304116339 -0.50730939
0.268068599 0.251765344 -0.482053839 0.118653873 -
0.168302054 -0.314600544 -0.383021944 0.048573663 -0.428610322
0.211362253 0.157557865 -0.084165307 -0.0104899 0.337518547
0.123055132 0.455891174 0.016622063 -0.088146994
0.227351751 -0.162410451 0.120409136 -0.29433963
0.047202268 -0.362527349 -0.027401836 -0.396222833 -
0.12709119-0.421302973 -0.184024286 0.145521878 0.133924871
0.05276627 -0.238866087 0.48172728 -0.314909099 -
0.138919852 -0.197905344 0.176990792 0.036308932 -0.188111908
0.126913034 0.072258358 -0.189516279 -0.315208986
0.155992886 0.063672522 0.374979107 0.104103786 -
0.120973354 0.377422656 0.036932831 -0.296599105 0.342483666

-0.128488749 -0.354437817 -0.277760558 -0.457639009 -
0.059881531 -0.195412462 0.027706742 0.046345784 0.059494568
-0.345551229 -0.143936575 -0.021157256 -0.247498209
0.265331125 -0.332520657 0.417952528 -0.178121658
0.219564006 -0.271214103 -0.004966617 0.303375749 -
0.076077241 0.234502146 -0.364864606 0.241960177 0.250888773
-0.009560816 0.260868341 0.232620914 0.542850743 -
0.355469733 -0.212625157 0.041744617 0.194899755 0.111416008
-0.299680237 0.03013193 -0.040912424 -0.546679696
0.136454736 0.002667354 -0.070994285 -0.055473617 -
0.203984344 -0.268287012 0.149336588 0.108474319 -0.002305284
-0.262760642 0.146142157 -0.370077648 -0.230079519 -
0.079386795 -0.398258454 -0.215391668 -0.145650924 0.479356995
0.436808813 -0.33850429 -0.036614181 -0.062178476 -
0.32585915 -0.128209215 0.284589386 -0.178279465 -0.041365126
-0.213179229 -0.255445704 0.315859268 -0.202949952 -
0.034303889 -0.365596599 -0.31272375 -0.239550256 0.270323835
0.325634848 -0.432099821 -0.278285795 -0.010196858
0.157689748 0.273432231 -0.318571351 -0.012417273 -
0.278889268 0.188946702 0.267811302 0.352215111 0.346384649
-0.040582872 -0.25264833 0.465591731 -0.084392061 -
0.186428691 0.003165914 0.011794848 0.006203254 -0.455176671

12990 0.18417178 0.058438721 0.463258066 0.158620652

12991 0.168200741 0.298656983 0.169089659 0.011730212

12992 0.356657995 -0.314286731 -0.382027239 0.216769927

12993 0.220451097 -0.089003695 0.436798882 -0.324722191

12994 0.000349922 0.055781783 -0.349198688 -0.148696947

12995 0.104870284 0.375233258 0.012875093 -0.047476317 -

12996 0.103512443 0.182936346 0.391735257 -0.016828921 -0.169192643

12997 0.481429463 0.01021202 -0.10025995 0.322507858

12998 0.136030726 -0.153090324 -0.378139772 0.048121183

12999 0.263933435 0.07846598 -0.20008403 0.080672907

13000 0.009996138 -0.269594418 0.067181486 -0.273007659 -

13001 0.250441193 0.033006549 -0.09091517 -0.275616751 0.139898633

13002 -0.264796959 0.21504022 -0.054227816 0.001992682

13003 0.217226395 0.327537794 0.026262383 -0.08958821

13004 0.331273248 -0.387307411 0.178230593 0.03015147

13005 0.354378374 -0.365420343 0.300596577 0.083797983

13006 0.089188036 0.201057796 0.026216254 0.009180613 -

13007 0.356584899 -0.015930659 -0.203416697 -0.193292818 0.226406598

13008 KEGG_STARCH_AND_SUCROSE_METABOLISM 0.290246163

13009 0.300564002 -0.157560534 -0.257583191 0.366963532

13010 0.043263929 -0.037709144 0.263233904 -0.086583796 -

13011 0.071860226 0.06355837 -0.381963991 -0.105949587 -0.238653445

-0.20510255 0.293882638 -0.086868481 0.2937034 -0.197033609
-0.216364743 -0.262894516 0.441644455 -0.312882973
0.051230205 0.058330635 0.141528371 -0.124310541
0.168651121 0.051662827 0.094479894 0.432572315
0.345932151 0.207992224 0.18410067 -0.162092999
0.086739608 -0.225045737 0.468122437 -0.401680958 -
0.108811279 -0.109429953 0.494744175 -0.278550706 0.215068688
-0.347090321 0.250231651 -0.329596838 -0.16720111
0.405763634 -0.14449522 -0.105513282 -0.292932078
0.140565375 -0.546396216 0.116179167 0.285800452 -
0.259795823 0.393161927 0.282389946 -0.272490581 -0.298921153
-0.190730081 0.250736683 -0.220942688 -0.091126333
0.016850779 0.319694524 -0.057949523 0.443455944 -
0.187104804 -0.277755529 -0.047787708 -0.088603682 0.128405794
0.291146665 0.185172633 0.117030952 0.209124292
0.448671851 0.275541392 -0.132935324 -0.019291477
0.390449123 -0.105457116 0.108278184 0.018304673 -
0.453425233 0.215277618 -0.352587114 0.00474949 -0.235196418
0.052342435 -0.224451614 0.32445393 -0.156553292 -
0.179746479 0.137163579 -0.244985211 0.300366492 0.300206183
-0.086611758 -0.002914796 0.124169638 0.421298327
0.185964767 -0.042654109 0.475448807 0.310584885 -

13034 0.393810948 -0.222052491 0.113511686 0.39331802 0.195573845

13035 -0.245268161 0.074918747 0.174582098 0.098334249 -

13036 0.126841715 -0.112888224 -0.211495212 0.008074635 0.311689805

13037 0.009227019 -0.078411283 -0.153923248 0.16201173-0.150299245

13038 -0.394706311 0.342040411 0.063740578 -0.249374004 -

13039 0.253781612 -0.111128399 0.134549084 0.111815119 -0.383273931

13040 -0.375028377 0.3422445 -0.097631118 -0.330141278 0.117482861

13041 0.438845126 -0.246172189 -0.034322968 -0.223148572 -

13042 0.160098347 -0.005791446 0.446409508 0.243557501 0.256802384

13043 0.107052872 -0.196910815 -0.126789102 0.378926851 -

13044 0.300060524 0.146198811 -0.361034933 0.34147421 -0.297238352

13045 0.106937188 -0.094501448 0.395776677 -0.080935134 -

13046 0.109569779 0.305750555 0.075911103 0.363971876 0.04304176

13047 0.320237429 -0.200031738 -0.208981908 0.011174381 -

13048 0.016986995 0.268356781 -0.175298631 -0.042940034 -0.10898568

13049 -0.211783969 -0.059174974 0.06620036 0.257758187

13050 0.073595497 -0.121633982 0.248768768 -0.206323954

13051 0.272807259 -0.026779311 0.096875404 -0.43552823

13052 0.150588483 0.274033777 0.260846110.010852372 -0.20947842

13053 -0.251915414 0.397323639 0.208087223 -0.078117507

13054 0.141224677 -0.035828019 -0.088885848 0.399395382 -

13055 0.000579886 0.334469425 0.21556584 0.197816614 -0.15251366

13056 0.155446214 -0.121641872 -0.100970403 0.002741067 -
13057 0.4269454 0.124524124 -0.222634438 0.362309325 -0.260013906
13058 0.198832372 0.111709690.210309575 -0.317040869 -0.213962018
13059 0.257430765 0.136679043 -0.479821666 -0.279828518
13060 0.022975924 0.223034136 0.068710045 0.113667455
13061 0.300999978 0.076244527 0.069932557 -0.069313939
13062 0.419740141 0.260361704 0.135015879 -0.19252054 -
13063 0.409823222 -0.11646976 0.071108377 0.055687422 -0.186549482
13064 -0.25249625 0.358145436 -0.30096848 0.203497652
13065 0.345050475 0.139283006 -0.116963384 -0.115465783 -
13066 0.117073355 0.038232621 -0.211993657 -0.310125763 0.066621284
13067 -0.35572316 0.246951548 -0.117868271 0.09919362 -
13068 0.262469107 -0.261649151 0.264581673 -0.267901939 0.215471202
13069 -0.007276344 -0.25252674 -0.01191351 0.361854659 -
13070 0.088215487 0.202371161 -0.106144194 -0.230350819 -0.075631631
13071 0.099018343 -0.063434794 0.188635244 0.172448468
13072 0.179263989 -0.328652129 -0.209008233 -0.286534265
13073 0.306637985 0.068161206 0.079707384 -0.3951665 0.195525004
13074 -0.092246254 -0.148128374 0.077897365 -0.205341998 -
13075 0.105092042 0.113748367 -0.113054846 0.020847835 0.198800006
13076 -0.060397578 0.187688814 -0.014410847 -0.327625594 -
13077 0.22136208 -0.125164234 0.130659624 -0.032529016 0.27251007

0.366160533 0.379912296 0.030626377 0.10423954 -
0.294997547 -0.253916017 0.056520477 -0.012435456 -0.28997768
0.165120431 0.444099764 -0.208628934 -0.141321138
0.435137906 -0.251411117 0.251376921 -0.200358603
0.297602355 0.258318033 0.217611135 0.183812457
0.539946899 -0.257924835 0.245656778 0.278862969
0.20446972 0.119932968 0.211017067 0.028219716
0.524050763 -0.106363102 0.273830449 0.233295705 -
0.111346531 -0.381010543 -0.10503864 -0.088341557 0.035705447
-0.39519917 -0.178598917 0.157842605 -0.279130008 -
0.298882193 0.254328066 0.087449079 -0.181917474 -0.34922812
0.303498291 -0.367442145 0.179123281 -0.125275071 -
0.058267533 -0.273044943 0.205189273 0.29376552 0.11080547-
0.29653785 -0.171218915 0.104313226 -0.17119043 0.358374363
-0.154015464 -0.206009528 -0.034936563 -0.323866669 -
0.314992747 -0.196133244 -0.448579197 0.286415655 0.378260348
-0.239046117 -0.193055484 -0.035520627 0.097308029 -
0.296473396 -0.220993757 -0.242390167 -0.119327187 -0.052333722
-0.249657266 -0.297233449 -0.088888712 0.257108222 -
0.159369614 -0.128573195 0.29643032 -0.051413706 -0.20057498
-0.029172247 0.372097147 0.375563776 0.042918848 -
0.008385646 -0.094317871 -0.085270705 0.074201393 -0.071397994

0.357304535 -0.266793323 0.352759209 0.088160307
0.280304547 0.101720331 0.34921798 0.172302772
0.236117565 0.157971375 -0.199256117 0.058596044 -
0.411217212 0.11609525-0.063312576 0.111033938 0.371376661
0.267265939 0.302469959 -0.344404675 0.234038351 -
0.148919734 0.096065854 -0.192236146 -0.084369723 0.47820364
-0.301000086 -0.276746278 0.32633858 0.11074267-0.017852368
0.315966557 -0.037841263 0.020288046 -0.179273892
0.307094155 -0.289640937 -0.132486768 0.064391849 -
0.1742556 -0.317028729 0.382188694 -0.047953839 -0.201482269 -
0.054610161 -0.016566479 -0.393192098 -0.118441106 0.177021641
-0.364280438 0.024544346 0.054874414 0.087533867 -
0.178205504 -0.133638998 -0.303385014 -0.343740779 -0.229085182
0.024866406 0.213820329 -0.023234346 -0.286180291 -
0.220220376 -0.197660052 0.399849391 0.064588338 0.059890071
-0.059153614 -0.153947963 -0.242134398 0.303238066
0.51534832 -0.268061232 -0.034452319 0.031632524 -
0.032025635 0.322838219 0.22156638 -0.204380772 -0.395285921
0.061681953 -0.182667813 -0.060607098 0.29307549
0.270043449 0.18128546 0.073065101 0.213546844 -
0.23312302 -0.131456094 0.225209286 0.326983092 -0.452581508
-0.105886945 -0.03096777 0.449477095 0.034930435

0.173299042	0.296733924	0.337528451	-0.00706989
0.405384239	-0.062691943	-0.452424555	0.021562733	-
0.108702276	0.114063560.471434161	-0.288117016	0.308987894	-
0.152279518	-0.103802445	-0.070861809	0.26984037	0.266301663
0.244692455	-0.079350979	0.193570419	-0.323741142
0.299294766	-0.325489964	-0.036222356	0.040362383	-
0.028838268	-0.334454552	0.207063401	0.279280971	0.002266155
-0.29006149	0.015590604	0.255124167	0.156752918	-
0.128760449	0.201550745	0.412006916	0.115740302	0.107025198
0.224180257	-0.091589905	0.215483449	-0.373874668	-
0.336301202	0.199237635	0.075176626	-0.201939518	-0.033949755
-0.117122982	0.12108969	0.235551925	0.257303862	-
0.381464916	0.106387355	0.022866722	-0.073427761	-0.230248816
0.477338666	-0.180747405	0.118675064	-0.403931605
0.142614516	0.210865589	0.04759062	0.395135315	-
0.283935476	-0.155451818	-0.13057029	-0.131633332	0.353564436
KEGG_AMINO_SUGAR_AND_NUCLEOTIDE_SUGAR_METABOLISM
0.486221684	0.474503199	-0.330567539	-0.217708035
0.041474947	-0.104296028	0.277441484	0.329413084
0.303408241	0.118211563	-0.161788578	-0.053619054	-
0.490885235	-0.335382126	-0.286749575	-0.020063374	0.064693665
0.279447824	-0.578236435	0.280761669	-0.287334245

0.157901169 -0.358305046 -0.176491404 0.181845524 -
0.320931841 -0.08659896 -0.03410354 -0.089522618 -0.235800538
0.141548033 0.468656197 0.321493057 0.06585839 -
0.265257469 -0.114484956 -0.594566899 0.15574262 -0.518116701
0.104935354 -0.154235851 0.524078177 -0.262230576
0.313446065 0.011696145 0.339587938 -0.035128812 -
0.282156633 0.171184595 -0.443132776 0.105997294 -0.322257447
0.177905934 -0.410260627 0.073646249 0.342422482 -
0.426719212 0.366756019 0.335262301 -0.262989799 -0.243819349
-0.134839954 0.173318965 0.027870445 -0.222372529 -
0.224678184 0.446309379 -0.071265392 -0.118494308 -0.511988634
0.059671063 -0.144553455 -0.324257773 0.119631244
0.182133217 0.001843266 0.313290843 0.268375714
0.177737662 0.459455096 -0.162727792 -0.078720258
0.368247133 0.010361834 0.404967796 0.386646351 -
0.452088787 -0.155366768 -0.377677652 -0.022667594 -0.039931547
-0.430825888 -0.325261483 0.376559782 0.157422097 -
0.250114266 -0.05358463 -0.144771871 0.427811944 0.224639122
0.240002927 -0.47091008 -0.009028542 0.514120816
0.478073453 0.038953906 0.205621933 0.28938614 -
0.43224561 -0.466341233 -0.541288132 0.38331598 0.001120167
-0.284026648 -0.191138841 0.329021957 0.217207902 -

0.406497385 -0.621249958 0.191786928 -0.394205523 0.232448171
0.226997046 -0.379596736 -0.290714875 -0.339680613 -
0.031571585 -0.376486782 0.420141509 0.270661405 -0.229673189
-0.44195107 -0.25569481 0.001104897 0.115416626 -
0.25743432 -0.39511713 0.28386207 0.190038373 -0.18596493
-0.268488885 0.465104866 -0.050440095 -0.151805697
0.280563689 -0.365122891 0.073112150.538089895 -0.273203919
0.408355347 -0.101282349 0.181914987 0.215397967 -
0.028070508 -0.168701722 -0.362300574 -0.191293101 0.372189562
-0.12499277 0.111074444 0.013605439 0.343810154
0.069412973 -0.506890298 0.424444394 0.021558724
0.027218317 0.208023828 -0.125101373 -0.265610243
0.113267352 -0.205419151 -0.432109235 0.191217204 -
0.090929515 0.020818904 -0.393034845 -0.079601261 0.093149619
0.41493791 0.4608624 -0.080105128 -0.083841366 0.340491386
-0.240630739 0.252374322 -0.05933325 -0.153522247 -
0.327507369 0.237874394 0.461324014 0.314308196 -0.112729695
-0.485614172 0.048247572 -0.205990445 0.116404149
0.084818901 -0.346658514 -0.127267254 0.253827635
0.493152619 -0.194058686 0.020088969 0.376684635
0.249218257 -0.151684459 0.445749167 -0.264404339
0.366192136 0.248761849 -0.411199751 0.289502842 -

0.020636292 -0.033512744 0.327597105 0.449318116 -0.095544578
-0.134037545 -0.239333112 -0.065908857 0.335186658 -
0.333971023 0.220617567 -0.249361399 0.02069486 0.279548648
-0.188447453 -0.018498367 0.344429018 0.139348438 -
0.025086511 -0.349572588 0.116335809 -0.059043959 -0.192856977
-0.322967501 -0.255977176 -0.420483369 0.15415208
0.417819201 -0.266096511 -0.182615095 0.22093592 -
0.15412618 0.211329982 0.310808157 -0.309103663 -0.192188121
-0.505423425 0.048690721 -0.051968716 -0.387354843 -
0.246754099 -0.067919686 -0.401749746 0.070937425 -0.386755485
0.407618883 -0.33405169 -0.229625873 0.352472178
0.048461464 -0.265626163 0.213086736 -0.417930979 -
0.212487032 0.272020598 0.298058967 -0.332360756 0.210150004
0.296081938 0.075765624 0.05723764 0.234859206
0.45986814 0.194180612 0.104645499 -0.522964198 -
0.142089659 -0.367391722 0.322855774 0.343836573 -0.548724915
-0.477615243 -0.054841282 -0.425010575 -0.112406534
0.095664198 -0.481637296 -0.26461103 0.18031386 -
0.021917107 -0.212435395 -0.426310387 0.060038354 0.148463595
-0.10172968 0.096242607 -0.06906479 -0.017675914
0.368217498 -0.090489053 -0.058190729 0.39861327 -
0.079568013 0.291100414 0.084231942 -0.266526387 -0.552515523

13210 -0.016508386 0.181063535 -0.488873546 -0.126069677

13211 0.397374279 0.492410212 -0.422003287 0.192479927 -

13212 0.180650114 0.399666485 -0.375376313 0.287340137 0.148398011

13213 -0.225967209 0.537640565 0.17888983 0.168492437 -

13214 0.091495473 0.26639236 0.015793758 -0.079949951 0.096787838

13215 -0.034976308 0.565787407 -0.35954287 0.054675738

13216 0.097964005 0.459559256 -0.412459139 0.212213822

13217 0.349042121 0.232885936 -0.486284445 -0.423743961

13218 0.219025631 0.183642262 -0.301808207 0.383982637 -

13219 0.405205816 -0.034936696 -0.286388921 0.31078833 -0.227591608

13220 0.338063357 0.117950143 -0.370961231 -0.296490581

13221 0.343837279 0.367214797 -0.025372384 -0.057786876 -

13222 0.33422968 -0.107033876 -0.002722871 0.434091439 -0.284058876

13223 0.086886779 -0.250370578 -0.324860603 -0.360505793 -

13224 0.100888535 -0.328218543 0.1602753 0.420868976 0.03256033 -

13225 0.270519338 -0.252925259 0.09470201 0.282260113 -0.063680373

13226 -0.399047705 0.020637495 -0.137052552 -0.366301663

13227 0.114883422 0.055883208 -0.08467339 0.115488373 -

13228 0.242558282 0.286882512 0.161906329 0.072845316 -0.019942115

13229 0.316012751 -0.128886289 -0.317328881 0.015055591

13230 0.026956563 -0.485896059 -0.220828469 -0.372948374

13231 0.240558034 -0.215709349 0.073221652 0.362050997

0.074042514 -0.237276571 0.090748556 0.34258052
0.113267731 0.421988521 -0.279046695 0.234403468 -
0.239699324 -0.191724515 -0.370190354 0.283280795 0.510612094
0.261900167 0.191256297 0.20459096 -0.085631828
0.281606072 -0.097067899 -0.3137975 0.236236232 0.11262488-
0.06910914 -0.236848821 0.324688208 0.122210777 0.209716135
0.106306221 -0.435870704 -0.192516789 -0.348490981
0.214812812 0.144908643 0.138521513 -0.319013504 -
0.129117611 -0.273174818 0.235486178 -0.505685486 -0.078771548
0.022270577 0.044457874 -0.457215713 -0.359122188 -
0.215890033 0.161473631 -0.356315026 0.038798655 -0.147145002
0.237970426 -0.017729897 -0.282570122 -0.516345461 -
0.375527321 0.199222046 0.010003341 -0.258884266 -0.435787648
-0.252880648 0.009339811 0.090073688 0.259923443 -
0.157064303 -0.044654388 -0.404970362 -0.35502287 0.174835978
0.472567604 -0.292447247 -0.047361322 0.108941337 -
0.374114153 0.123532468 -0.179233688 -0.041020478 -0.157548646
0.327013874 -0.074611788 0.040275163 0.329379522
0.287097936 -0.158191856 0.356393309 0.141667721 -
0.26613925 -0.305010379 -0.388346894 0.28617503 -0.480937612
-0.145247498 -0.393292657 0.3077633 -0.051614966 0.140701985
0.249003347 0.443698512 -0.044177739 0.304884331 -

0.444281104 -0.421525411 0.183488817 -0.435241684 -0.103831059 0.228744412 -0.205568473 -0.264741631 -0.081412164 -0.266674285 -0.457735379 0.061992782 0.44176578 -0.053849747 0.154762606 0.020406719 -0.321818042 0.262522394 -0.507235321 -0.096588164 0.184872909 0.47846602 0.314311909 0.506381236 0.351568137 0.028646369 -0.374747251 -0.440386427 0.426076248 0.493436294 -0.359478354 0.345050209 -0.126844304 -0.413431746 0.236448958 0.015865457 -0.230885377 -0.191944546 0.209257496 -0.230725613 -0.317984532 -0.099772179 -0.278497795 -0.355760917 0.366298927 -0.001566862 0.356627812 0.077478661 -0.366630643 0.216358627 -0.041065632 0.055456443 -0.299757718 0.463126828 -0.305476487 0.340333913 -0.07253266 0.430702909 -0.037395182 -0.124562515 -0.127923698 -0.237268282 0.161615718 -0.33844563 -0.240924305 0.150246386

KEGG_GLYCOSAMINOGLYCAN_BIOSYNTHESIS_CHONDROITIN_SULFATE -0.358832751 -0.188127748 -0.295393939 -0.149311374 -0.093290323 -0.472520518 -0.155759065 0.268584827 -0.251016676 0.127148122 -0.248511391 -0.33833623 0.038622937 0.247594009 0.384542503 0.416540643 0.002341499 -0.099802017 0.384604509 -0.334022964 0.029606657 -0.149088242 0.33667333 -0.352101161 0.61312505 -0.165319915 -

0.440290788	0.053266039	-0.434025276	-0.022367	-0.293611057	-
0.153212686	-0.416856265	0.043112224	0.097311165	0.309769349
0.263639005	0.607376053	-0.08404679	-0.330415829	-
0.506964109	0.259105442	0.034053729	-0.184926812	-0.183983841
0.525954094	0.097267264	-0.268672692	-0.350753925
0.173259292	-0.36266834	-0.414806957	-0.062937042
0.085927766	0.215106432	-0.105538542	0.106925614
0.365792047	0.004734328	0.238323781	0.419360303
0.35460243	-0.534570645	0.444523256	-0.222563812	-
0.377199564	0.179333095	-0.295933822	0.183141253	-0.179540747
-0.057740322	0.188988172	-0.37141644	-0.226081968	-
0.034591633	0.40584251	0.031717085	-0.329939663	0.525601932
-0.1248433	0.149855438	0.141158142	-0.301328315	-0.065912765
0.180005153	-0.18750536	0.218146123	-0.013144883
0.210525894	-0.468107514	-0.245211681	-0.104759892
0.27223977	0.260023289	-0.096225724	-0.371941966	-
0.359832131	0.378540662	0.120751183	-0.104343886	-0.136058026
-0.366363367	-0.521586294	0.302932046	0.446024456	-
0.109282694	-0.56887294	-0.374405721	-0.168344639	0.302106524
-0.370317591	-0.259366014	-0.036386554	0.20415665	-
0.400109993	-0.382715222	-0.154075476	-0.199957765	-0.295477552
-0.079788845	-0.161341881	-0.568012057	0.316341117	-

13298 0.292588295 -0.132915411 0.263613045 -0.335826851 0.077908119

13299 -0.281367241 -0.380484947 -0.40217768 -0.135364983

13300 0.01866719 -0.305863801 -0.169501623 0.515993314

13301 0.488794934 -0.426145865 -0.331636165 -0.250665591 -

13302 0.457029748 -0.328757204 -0.321804655 -0.313847861 -0.324309314

13303 0.127823253 -0.141670762 -0.293012047 0.4546487 0.511418205

13304 0.462170039 0.159015435 -0.095971072 0.263788837 -

13305 0.496881165 0.079183042 -0.08356636 -0.015608643 -0.133334188

13306 0.437253842 -0.206318835 -0.042321398 -0.116310568 -

13307 0.047592141 0.291153894 0.282134536 -0.294431063 -0.241731119

13308 0.393622848 -0.116128242 -0.430717792 -0.353972114

13309 0.114348648 0.042487032 0.127742138 0.057498673 -

13310 0.145138529 -0.545023122 0.297665465 0.280329961 0.561603722

13311 -0.332366953 -0.369417301 0.17497299 -0.009497972 -

13312 0.098210727 -0.240734426 -0.548244946 0.453576053 -0.022733127

13313 -0.2775035 -0.14122655 -0.536403919 0.317477554 -0.511724788

13314 0.310863993 -0.37212359 -0.096470863 -0.49497057 -

13315 0.414665041 0.609393917 -0.531960064 -0.009454326 -0.342322503

13316 0.151888926 0.031968051 -0.098872777 -0.494030096

13317 0.177264863 -0.084093798 -0.463110229 0.032211506 -

13318 0.193167217 -0.014285978 0.184993076 0.009696322 -0.107728864

13319 -0.158021427 -0.451716774 -0.295811798 0.278361068 -

0.189576066 -0.160231877 0.198735646 -0.101721989 -0.384667396
0.234714449 0.42960283 -0.185854823 0.298461857 -
0.381370541 0.179591611 0.157875176 0.194637471 -0.101251415
-0.59117353 0.082385546 -0.250727601 0.25680124
0.188705693 0.615744509 0.505634872 -0.622534101 -
0.384062302 0.054012459 -0.40375826 -0.104582994 -0.076378221
-0.00909612 0.136037613 -0.026602884 0.422179527
0.261239652 0.210033387 0.566532456 0.271084845 -
0.068337415 0.346525313 -0.042231761 -0.16162873 -0.001680263
0.012579649 -0.34519545 0.64435958 0.411481309 -
0.411360958 0.26396359 0.382159458 0.171252193 0.2555422 -
0.653598946 0.35191912 0.294102251 -0.102839077 -0.132175056
0.261573743 -0.444335564 0.063910318 -0.288021688
0.428633799 -0.102025103 0.318138713 0.343260722
0.145207665 0.189781536 0.2248882 -0.022092099 -0.29820681
0.39933477 0.042991325 -0.265973361 0.459575122 -
0.184696873 0.032009052 0.3831979 0.270310631 -0.186356105 -
0.345049067 0.366556913 0.01826959 -0.489296089 -0.139651828
0.157715262 0.509580096 -0.05979474 0.211415175 -
0.008934285 0.108452072 0.514171596 -0.12599192 -0.201465738
0.174438110.468660765 -0.453445505 0.081650777 0.17214084
0.426370624 -0.125154795 0.350858966 0.262880176 -

0.29964177 0.133200951 0.279372777 0.279420737 0.244118543
0.209807903 0.350704164 -0.091859129 -0.277475233 -
0.072308889 -0.213199821 -0.272419936 0.084303401 0.258640396
0.149294372 -0.152741097 -0.396885572 0.47858814 -
0.228465031 0.237516863 0.140792112 -0.515194416 -0.276775873
0.021112881 -0.104223629 -0.325394132 -0.217845893 -
0.212244141 -0.065197326 -0.243184379 0.490707035 -0.378505966
0.284324705 -0.490321511 -0.123019453 0.045331291
0.44679964 -0.160365313 -0.251485417 -0.433278369
0.187478919 -0.401441493 -0.445530785 0.424082347 -
0.453980217 0.290735548 0.084119076 -0.053741239 0.248632982
-0.313486514 0.354644669 0.230528833 -0.105414937 -
0.283598777 -0.355975454 0.373022979 0.148505335 0.194289331
0.45959464 0.14078417 0.332273293 0.423511201
0.126655166 0.264567113 0.36612343 -0.024976039 -
0.400888294 0.148092858 0.038371096 -0.14797125 0.323959456
0.037611903 -0.337375734 0.384196337 0.188907944 -
0.249855431 -0.305909325 0.374760976 -0.167623074 0.235456687
-0.431114203 0.313365765 -0.423979055 -0.591448184
0.330810463 -0.620374311 -0.376456987 -0.372173044
0.454535405 -0.714258673 -0.407354864 -0.482355337 -
0.083506406 0.202186318 0.543337188 0.342812721 -0.482947646

-0.382061439 0.117951374 0.667750781 0.193419689 -
0.233266886 0.27555965 0.399252738 0.417692371 0.30513588
-0.427130714 0.228829564 0.153219518 -0.340360345 -
0.431007618 0.193421385 0.527972653 -0.212191208 -0.281621398
0.06385573 0.2263693 -0.499295992 0.077430541 0.04968896
-0.097307294 0.324715291 -0.386574029 0.378717785
0.381578527 -0.073341348 -0.34780651 0.387156152 -
0.170355908 -0.220527417 0.108831074 -0.2273273 -0.353997409
0.231115702 -0.210099808 0.312850172 -0.052943323
0.366739958 -0.230634771 0.125947315 -0.021410271 -
0.311412721 0.365171997 0.184354508 0.190930779 0.02337145
-0.039887684 0.072513272 -0.116507148 -0.156821549 -
0.529956222 0.266486294 -0.252166881 0.218686292 0.472377396
0.352873795 0.240022626 0.154617241 -0.450252415 -
0.021452865 0.454408557 0.425322972 -0.196309016 0.0385721
0.029095492 -0.407069295 -0.39007282 -0.223030072
0.402262071 0.229640141 -0.085725775 -0.487562371 -
0.14067925 0.038958995 -0.381547695 -0.348930312 0.328281439
0.514531288 0.002208768 -0.317652545 0.205577933 -
0.084689697 0.19021389 0.145245236 0.358627082 0.131937342
0.222818409 -0.156966555 0.02555804 -0.231757812
0.042893344 -0.041977532 -0.374857955 -0.169984588 -

0.274223061 -0.150647229 0.222374995 0.26364348 -0.133392635
-0.028432963 -0.252637148 0.138088847 0.061236503
0.509962144 -0.470135235 0.423389699 0.21829061
0.444268989 -0.165151205 0.27905375 -0.167115569 -
0.160644502 0.392536267 -0.16123126 -0.392098436 -0.238161709
-0.088150301 0.06207402 0.200512591 0.185282602 -
0.471739036 0.305424002 -0.112410035 -0.091822857 -0.087196971
-0.384123087 -0.084968171 0.296560694 -0.109685875 -
0.373158896 -0.091162674 0.048515118 0.320115945 0.004681563
0.368148682 -0.229085227 0.306902917 0.107147114 -
0.087657028 0.120927101 0.20794335 -0.148055487 -0.074188441
-0.050325235 0.032332337 0.122904078 -0.224762181
0.281838197 -0.240144645
KEGG_GLYCOSAMINOGLYCAN_BIOSYNTHESIS_HEPARAN_SULFATE -
0.194686447 -0.255326769 0.064794081 0.191988708 0.054869252
-0.10676645 -0.313420891 -0.099906179 -0.409847986 -
0.21906066 0.046256422 -0.103681652 -0.11192949 0.138328516
0.089906335 0.147712299 0.112828087 -0.079615939
0.236529405 -0.268544802 0.109199912 -0.043637542
0.167608618 -0.06468857 0.245590401 0.212442276 -
0.298711732 -0.140568693 -0.044892008 -0.084865199 -0.107379819
0.336212717 -0.156055862 -0.140267092 0.380464082

13408 0.080773897 0.179225413 0.368425217 0.139581951 -

13409 0.10289976 -0.137634667 0.352336354 -0.152212578 -0.073536484

13410 0.329653347 0.267393379 4.93E-05 -0.017212917 -0.35248523

13411 -0.286577739 -0.198874178 0.07222776 0.223756083

13412 0.205895794 0.043525201 0.018889436 0.177285294

13413 0.153533451 -0.160167215 0.061810087 0.046308513

13414 0.372310799 -0.247289286 -0.077754808 -0.263396717 -

13415 0.090417914 -0.154991607 0.336979267 -0.045129497 -0.247958073

13416 -0.149586415 -0.16690535 0.181893992 0.002559207 -

13417 0.1360832 0.180658321 0.092595478 -0.292915427 0.136442769 -

13418 0.110298433 0.099304785 0.024658391 0.251905243 -0.05242708

13419 -0.157468298 0.030291364 0.093305623 0.165498201

13420 0.093461035 -0.458034477 -0.295839576 -0.084088357

13421 0.197356486 0.042882269 -0.176672668 -0.396208504 -

13422 0.114977043 -0.130468639 0.290347655 0.332969699 -0.112829331

13423 -0.194157227 -0.167615268 0.119176623 -0.022513875 -

13424 0.139801224 0.004375639 -0.090744278 0.107024604 -0.054491697

13425 -0.088983697 -0.376011139 0.142140714 0.320550971

13426 0.065685522 -0.16652786 -0.268098284 -0.324236654 -

13427 0.205110017 -0.055231358 -0.0781643 -0.201744595 -0.033703369

13428 0.042779887 -0.145016484 0.015286148 -0.136576611

13429 0.242604879 -0.21319442 -0.283520032 0.065294251 -

0.084776562 -0.164725706 -0.09473987 -0.243974377 0.36262423
0.422602544 -0.283606644 0.140359874 0.061378278 -
0.236592091 -0.351092359 0.015650132 -0.199450773 -0.132464152
0.145922881 -0.041253433 -0.00784657 0.055718491
0.190265946 0.286976242 -0.04584836 0.100877976
0.180148067 -0.12958366 -0.03257916 -0.008789703
0.024202522 -0.075908781 -0.0517515 0.256935529 -0.221228138
-0.206578207 -0.25818703 0.072398565 0.35977153 -
0.067898186 0.077241603 0.057559711 0.249360024 -0.395063332
-0.280864228 -0.037253557 0.227799393 0.205075519
0.399698249 -0.118644939 -0.409504316 0.2383082 0.062193293
0.472141625 0.003922938 -0.280338428 0.184515138 -
0.013851776 -0.103968963 0.182556218 -0.126076809 0.087073284
0.274488621 -0.208819 -0.054556299 -0.208737877 -0.025099034
-0.299608645 0.227924569 -0.345397915 -0.186371656 -
0.090756653 -0.053538333 0.175048223 -0.252669055 0.339532051
-0.049592095 0.072933812 -0.11753408 -0.15676706 -
0.074338828 0.160646348 -0.297510977 -0.228048468 -0.280972168
-0.272852162 -0.002512695 -0.048513098 -0.069127383 -
0.092003081 0.002896117 -0.227738061 -0.190055602 0.29726139
-0.15273944 -0.190842936 0.037504914 -0.151828368 -
0.226094106 -0.080801492 0.053459395 0.06506915 0.392776579

-0.283219305 -0.059495559 -0.146636017 0.031008688 -
0.330487888 0.015694524 -0.111884707 0.156600263 -0.185739 -
0.093327166 0.485562056 0.297130806 -0.466723359 0.142229634
0.215274335 -0.189663098 -0.192625858 -0.230265993
0.213519199 0.052827815 -0.017836352 0.009578842
0.054303047 0.370447077 0.345446029 0.399097152
0.025896923 0.229247991 -0.056887195 -0.344620833 -
0.151586464 0.074739649 -0.181763561 0.275751031 0.171024949
0.024061465 0.284335142 -0.14157411 0.062826293 -
0.084363829 -0.250620736 0.362389187 0.326671506 0.129541897
-0.1431072 -0.151482861 -0.309014091 -0.249624895 -0.186623801
-0.084348041 0.08852743 0.203096503 0.350274045
0.350798101 0.178625109 0.003418378 -0.09624559 -
0.300651478 0.198300906 -0.024190342 -0.062439492 -0.100670307
0.020048646 0.058712311 0.269938487 0.084252176
0.123498064 -0.149534916 0.0951505 -0.170432274 -0.339703989
-0.041450116 0.053501898 0.268264217 -0.019801939 -
0.04551202 0.407035944 0.016747025 0.489934992 0.107196164
-0.133281222 -0.067003864 0.20614822 -0.040539604 -
0.160403324 0.055143406 0.059806562 -0.128809219 -0.125092119
0.252786476 -0.052407124 -0.022498913 0.237836631
0.046449523 0.076394715 0.271929697 0.176414185 -

0.146304432 0.16231126-0.105988758 -0.203015211 -0.351415389
0.264756153 0.340578756 0.34085804 -0.227098626 -
0.13443686 0.175118007 0.243172508 0.316476222 -0.221198583
-0.305194196 -0.090339844 0.301905424 -0.334993462 -
0.026356041 -0.078602746 -0.008602797 -0.240436687 -0.282009361
0.328901643 -0.057957271 0.152838145 -0.389328982 -
0.118323001 -0.14102221 0.12725512 -0.241106282 -0.116570645
-0.305550466 -0.409283662 0.066615549 -0.136564578
0.205795408 -0.167063603 0.377979513 -0.174051269
0.19018352 0.064784353 -0.065658154 0.302115005
0.364631672 0.268320197 0.162259401 -0.175031765 -
0.019976043 -0.027593026 -0.008252414 0.327723835 -0.179454336
-0.199264359 0.179590259 0.06036664 -0.128520781
0.203451606 -0.088467157 -0.103745963 0.292634993
0.132523163 -0.026389841 -0.180802501 -0.35858472 -
0.058658962 0.056757872 0.146159145 0.231792896 -0.112245983
0.250916464 0.126441007 0.354458042 0.05015585
0.088628371 0.190697126 -0.371331745 0.209057592 -
0.402888072 -0.080354533 0.073166989 0.367541907 -0.195219871
-0.341329083 0.002953511 -0.182289226 0.027149303
0.308355004 0.02716154 -0.222785386 -0.209458758
0.348698408 0.371641655 -0.239312736 -0.063303327

0.328479137 0.088823341 -0.093096513 0.344240196 -
0.154072637 0.197097896 0.176608304 0.00475776 -0.0707773 -
0.003426407 0.288812106 -0.300414033 0.035573444 0.051136334
0.113002045 -0.082177619 0.281431649 0.444808013
0.011457856 0.24306124 -0.325365969 0.215946182
0.167343198 0.022142478 -0.101857835 -0.023850577 -
0.043295976 -0.11238467 -0.109047257 -0.190420419 -0.015369486
0.155080037 -0.42434655 0.282963874 0.28809236
0.102075456 -0.291159617 0.326543526 -0.049298835 -
0.292642171 -0.040581657 0.087761667 0.173026352 0.022111854
-0.076092643 0.052776343 -0.006549289 0.012064636 -
0.246269438 0.260890652 0.236971586 0.24079704 0.087046795
0.150129338 0.14159744 0.42131945 -0.120394114 -
0.02305923 0.038823025 0.132315806 -0.210511913 0.145822258
-0.188108716 -0.265216738 -0.039323942 -0.261876809 -
0.009154402 0.122302886 -0.008378232 -0.148804714 0.066808953
-0.358053012 -0.06818285 -0.153430955 0.125906306 -
0.255343231 0.058966645 0.037229326 -0.002138835 0.017373091
0.160123256 0.335785613 0.432521086 0.140857863 -
0.066118301 0.268139854 -0.1314648 -0.033231462 -0.033786132
0.061479022 0.078153938 0.05025726 0.226141449 -
0.104157048 -0.233365285 -0.047195084 -0.128591757 -0.058764067

-0.125808202 -0.087247523 -0.051963996 0.379639653 -
0.230548284 0.448973995 0.050269747 0.166063428 0.116299915
0.179138607 -0.03542061 0.09472896 0.018821911 -
0.305609238 0.081635259 0.12560436 0.299854804 0.04292028
0.231375332 0.19277367 -0.090476217 0.036739556 0.1416535
-0.156101946 -0.044315037 -0.145308491 -0.054841068 0.3466488
0.098973241 -0.216863378 -0.059461081 0.203013064
0.351794803 -0.010366626 0.214182968 -0.369295528
0.146347581 -0.067188504 -0.33400438 -0.139226094 -
0.064225657 0.009972908 -0.129826609 -0.175143531 0.02736695
0.359600134 0.048153169 0.13085587 -0.104651752
KEGG_INOSITOL_PHOSPHATE_METABOLISM -0.013663461
0.27334152 0.12060393 0.436189898 -0.173948629
0.063987341 -0.155885227 -0.319621597 -0.219765125 -
0.278440611 0.240418667 0.045654294 -0.100660987 0.08491154-
0.314443299 -0.335726318 0.202366767 0.156815569 -0.222383157
-0.073203171 0.41082759 -0.003832616 0.049357405
0.367248698 -0.313119767 -0.300932591 -0.425708375 -
0.002991582 0.072435413 -0.236821519 -0.111739297 0.03200552
0.084678176 -0.220816241 0.222540965 0.241574529
0.111651133 -0.310172143 0.241459912 0.256735702
0.13891125-0.153279554 0.432231347 -0.077954983 -0.002562133

-0.103539726 0.345716102 0.156923275 0.021806588 -
0.41246822 -0.126266378 0.136924382 0.104722763 0.264893086
0.438125429 0.012820752 -0.206829945 0.301299134
0.100824032 0.316493545 -0.326565929 0.300551979 -
0.219822242 0.353442792 -0.061941211 0.369581976 -0.215399702
0.245553656 -0.195140041 -0.197507014 0.104277065
0.093526765 0.096800558 -0.255618327 -0.155588879 -
0.244604197 -0.180426339 -0.008929159 -0.327232464 0.023480709
0.086802399 0.32790831 -0.184162091 -0.18080304 -
0.176861725 -0.198635984 0.36039636 0.172208735 0.129862796
-0.023515111 -0.073593719 -0.306090878 0.256760511
0.061953396 0.306293446 0.329747377 0.287619861
0.057368624 -0.076032971 0.142548087 -0.014629236 -
0.142222663 0.027073645 -0.254981113 -0.278741982 -0.212491388
0.092731555 -0.038241525 0.358000206 -0.090593502 -
0.01859007 -0.368463843 -0.248907452 0.067880573 0.152230977
0.273205527 -0.339693286 -0.21462186 -0.26315776
0.062762145 -0.129630406 -0.02764661 -0.197853538
0.067286574 0.256760634 -0.343353653 -0.271776175
0.241675221 0.110442276 0.320758343 0.003364601 -
0.236172447 -0.072467217 0.315155934 -0.152346911 0.318411003
0.105815118 0.183256372 0.129841937 0.340496389

0.032511166 -0.247098 0.254477853 -0.036778993 -0.305486489
0.0494758 0.288295708 -0.126780425 -0.315254156 -0.298753844
-0.273307792 0.231063459 -0.010044991 -0.05898026
0.330403382 -0.232388116 0.032377365 -0.031718755
0.203583084 -0.082147879 0.003874535 -0.383158462 -
0.214242299 -0.077320187 -0.219630654 -0.058793332 0.243560395
0.339569137 -0.409031876 0.066775448 0.145035666 -
0.047697836 -0.142774147 0.032831867 0.451733086 0.227223945
0.266964071 0.177177948 0.038886576 0.032502419 -
0.331251006 0.317121934 -0.047825832 -0.191723991 0.432399838
0.240971611 -0.083782777 0.059362804 -0.187215969
0.095554242 -0.363778748 -0.423452202 0.272737324 -
0.078048677 -0.394206652 -0.217237834 0.088693533 0.087385391
-0.06151724 0.254054742 -0.21750997 0.089347054 -
0.120644107 -0.230887943 -0.016991862 -0.380150055 -0.296428917
-0.10501333 0.379427728 -0.170534206 0.229410804
0.168310249 0.262003339 -0.171835355 -0.402848421
0.185021561 -0.229298929 0.235615878 0.024550704
0.327176748 -0.163213513 0.364001503 0.159792516
0.005439739 0.114053428 -0.222605985 -0.400569363 -
0.294928061 0.14385337 -0.176326797 -0.389548479 -0.055997691
0.106916734 -0.030417194 0.157304899 0.374540079 -

0.053075861 0.361392812 0.149066247 -0.176043797 0.259014697
0.026673308 -0.09532059 0.124181688 -0.205562392
0.293019025 -0.080319077 0.157842179 0.087562333 -
0.249129326 0.187298733 -0.035642032 -0.092224182 0.084302902
-0.324705302 0.200821186 0.120388559 -0.103966891
0.322436935 0.26104963 -0.231777411 -0.00134571
0.034658186 -0.291512242 0.146714136 0.251471361 -
0.107134404 -0.265391967 0.170898073 -0.379295142 -0.256859593
0.018960808 -0.068792549 -0.109482577 -0.017955872 -
0.009918856 0.202841742 -0.06699541 -0.008017855 -0.15602066
0.051193166 -0.217551918 -0.421863936 -0.320531205 -
0.080005648 -0.392011364 -0.024668143 0.08896274 -0.263341853
0.144661397 -0.114843166 0.095327135 0.155843809
0.347064675 -0.361797833 -0.376569958 0.185717164
0.373401896 -0.094560686 -0.144348514 0.354560086
0.189156051 -0.109001141 -0.349785458 -0.278904605
0.047478623 -0.130671641 0.232048752 -0.00178777 -
0.218623075 -0.118491113 -0.380020952 -0.016117573 0.419983873
-0.026734917 0.234884036 -0.11845156 0.219408006
0.205432225 0.295560845 0.081424017 0.196557246
0.366961413 -0.233578431 -0.466576668 0.120515653 -
0.144973664 -0.298786082 0.240662094 -0.000791541 -0.274556123

13606 0.333965276 -0.303969173 0.142195855 0.351478366 -

13607 0.117392211 0.402492081 -0.189836093 0.251613199 0.298788266

13608 -0.160057496 -0.043864004 0.031096016 0.238562402

13609 0.146963929 -0.031349162 0.259883851 0.034418941 -

13610 0.243511848 -0.025950504 0.193934893 -0.139680237 0.409503516

13611 -0.255341996 0.254863007 -0.328142734 0.010995353

13612 0.135836368 -0.148265375 -0.173536797 -0.111020676

13613 0.183635871 0.354660606 -0.001522978 -0.32516962

13614 0.384304412 -0.193319799 0.365246841 -0.149122274

13615 0.505271613 -0.032105097 0.338695707 0.422546513

13616 0.149461418 0.149469838 -0.166077289 -0.299271819 -

13617 0.061876422 0.108423484 0.399985381 -0.317421189 -0.242176393

13618 0.126451975 -0.06091891 -0.06101769 -0.004788546 -

13619 0.077051035 0.202330595 0.215232611 0.133625423 -0.131085764

13620 0.036737626 -0.153769154 0.127219598 0.151162295

13621 0.44104582 0.080267057 -0.413177599 0.29916186

13622 0.366295504 0.020609377 0.253819929 0.318431186 -

13623 0.16879596 -0.059718503 -0.194483858 -0.022045355 0.080713777

13624 -0.091180967 0.161539095 -0.088478216 0.441714814 -

13625 0.312786421 0.198501537 -0.23771852 0.013183385 0.067460548

13626 0.070488578 0.046076358 -0.372780713 0.113020898 -

13627 0.200207142 0.147091359 0.326254198 -0.233236772 -0.315903702

-0.297310333 0.400506192 -0.084448825 0.152338527 -
0.092514316 0.161776669 -0.185759525 -0.086920765 0.057983471
0.181446963 0.413246708 0.086023411 -0.244624707
0.260021056 0.252366104 0.271977059 0.177347961 -
0.1447733 -0.1661783 0.365080912 -0.152646694 -0.171598681 -
0.193465102 0.281354949 0.032998781 0.024049627 -0.086026274
-0.261272553 -0.329042411 0.160110538 0.215498237
0.026609869 0.311873625 -0.021933292 0.126526616
0.183013272 -0.305413045 -0.003799979 0.354667904 -
0.270774388 -0.097086037 -0.099541469 0.376433176 0.003454946
-0.08927164 0.010894245 0.157989101 0.316889388 -
0.263424935 0.113380938 -0.038705829 -0.103879787 0.071544617
0.031169547 0.310824415 -0.262872925 0.147461404 -
0.096220133 -0.322170528 -0.05713066 0.299550647 -0.030410595
-0.079935174 0.285818935 -0.045305365 -0.108310056
0.161779073 0.307310749 -0.095167336 -0.325324343 -
0.251899475 -0.066245058 -0.272378169 -0.240193559 -0.130302866
0.24112821-0.045222596 0.297264009 0.286677568 0.150266282
-0.112142312 0.145361496 -0.385226172 0.160328583
0.233576183 -0.224631736 0.293331231 -0.3578866 0.056567552
0.026295684 0.262472575 0.266167074 0.314546526 -
0.071737283 -0.286083344 0.013091471 0.150806791 0.024945641

-0.107446477 -0.35300535 0.050011502 -0.165684414
0.202732517 0.346299053 0.116106999 -0.117768824 -
0.244471097 0.176633558 -0.375476333 -0.053151599 0.230946364
0.049953144 -0.318865629 -0.113278821 0.118348798 -
0.197042985 -0.093696156 0.296645052 -0.177780298 -0.086587686
0.14063038 -0.371323589 -0.040974561 -0.185863268
0.192804585 0.178153387 0.110791433 -0.199641878 -
0.067719491 -0.105627601 0.221682328 -0.319256212 -0.052214697
-0.364320224 -0.000501303 0.24004193 -0.10298614 -
0.249257521 -0.113342267 0.034316243 -0.129277311 0.145379637
0.019131449
KEGG_PYRUVATE_METABOLISM0.300504103 0.255340723 -
0.234762798 -0.026354652 -0.095442383 0.156123703 0.336148984
-0.108328495 -0.291666814 -0.314768163 -0.304309247
0.545577778 -0.513524962 -0.169329644 -0.206711876 -
0.031260426 -0.248231066 0.151637443 -0.591659565 0.141371248
-0.293080547 0.393319153 -0.568101832 -0.218279891 -
0.395348236 -0.064466025 0.202342683 0.003103867 -0.233816702
-0.339104568 0.42263573 0.023473325 0.177770718
0.386187813 -0.238244647 -0.372765291 0.024493792
0.161432684 -0.337611225 0.260484881 0.299242669
0.180070232 -0.320834867 0.44455141 0.160315205

0.318715119 0.253127768 0.355870324 -0.014044288 -
0.189132249 0.537296241 -0.267322819 0.177744547 -0.175890545
0.304845371 0.326648486 -0.390340518 0.280670494
0.421604453 -0.293205973 -0.55826874 -0.05205136 -
0.112273007 -0.247201928 -0.254650589 -0.397340226 -0.19043433
0.244224709 -0.168145511 -0.391325887 -0.244817801 -
0.449814844 0.537044431 -0.231599848 0.098470879 0.021274999
0.284345892 0.358370678 0.380909672 0.466830381 -
0.061957343 -0.04939074 0.275303063 -0.227226912 -0.196543041
-0.364665216 -0.341684736 -0.078136798 -0.495982434
0.433189993 -0.082171857 -0.133059168 0.050030344
0.541962179 0.331454829 -0.230268738 0.384890023 -
0.548490032 0.369734418 0.587480257 0.087928599 -0.309544052
0.222343252 0.237580021 -0.351368694 -0.143928011
0.285485098 0.60924798 -0.473595103 -0.423085394 -
0.364282746 0.175138565 0.426031824 -0.457723501 0.022095948
0.073129855 0.126439998 -0.035290727 -0.303410244
0.460999604 -0.054910132 0.280024613 -0.035674343
0.389017076 -0.0952639 -0.014707565 0.546062266 -0.46714436
0.450905477 0.285261358 0.17730205 -0.525480125 -
0.015309229 -0.078956547 0.033008548 -0.496423751 -0.567359763
0.370328516 -0.246044815 -0.408830755 0.034586666

0.280815324 0.278058411 0.037703746 -0.157007079 -
0.234431223 0.161610419 0.471082496 -0.41564931 0.077685518
-0.176274516 -0.314537159 -0.327341321 -0.270648309 -
0.139821372 -0.206435228 -0.435198906 0.546589187 0.07586425
-0.08137649 -0.334549249 0.118540528 -0.259912677 -
0.434020334 0.476408773 -0.473783178 0.199882872 0.013140361
-0.183517197 -0.390742688 0.096814291 0.334258567 -
0.061980527 0.149892523 -0.390173866 -0.247175389 -0.316968261
0.372095659 -0.10576493 -0.38538545 0.340803744
0.388632022 0.168498094 0.026376722 -0.314833056
0.137824945 0.070265767 -0.130135851 -0.331924656
0.340727849 -0.131178668 0.485336103 0.298361179 -
0.132423123 0.236836972 0.259529909 0.030944887 0.428073128
-0.039098702 0.112139744 -0.100863495 0.138540684 -
0.214223262 0.557752885 -0.272648778 0.306905586 -0.270684086
0.214952815 -0.305631486 0.359221615 0.382051491 -
0.304753897 0.397816631 -0.433667375 0.076172267 -0.17845886
0.136294974 -0.160523963 0.226423179 -0.462260947 -
0.404913612 0.502814683 0.133575124 -0.216974935 -0.454643769
0.00658751 0.100049086 -0.361873574 0.104433174
0.366952276 0.234924122 -0.34437987 0.072196703 -
0.325979003 0.247862228 0.157149702 -0.456832136 -0.173874842

0.053578573 0.181579321 0.443676347 -0.21415031
0.012626755 -0.183333544 -0.147246527 0.071030144 -
0.006371674 -0.320103615 0.017029482 -0.317441307 0.071061443
0.125292311 -0.394556545 -0.576511723 -0.056092696 -
0.475021482 0.28698678 -0.151533535 0.419155394 -0.07680203
-0.573505666 0.180076219 0.172062595 -0.193879327
0.236022086 -0.496785026 -0.44367153 0.037036551 -
0.048576131 -0.182914278 0.429506025 -0.330606744 0.158055465
0.052428867 0.142747418 0.163911171 0.152910442
0.442628147 -0.104021703 -0.221774408 -0.444467443 -
0.006648229 -0.420072132 -0.456651853 -0.516144943 0.330069252
-0.433204091 -0.130332142 -0.417172782 -0.207337711 -
0.057291474 0.372573821 -0.159442732 -0.42921689 -0.249596897
-0.391222505 0.029012806 -0.063034223 -0.281475105 -
0.081898661 0.271537975 0.490035869 0.037851754 -0.193172518
0.641753438 -0.166261149 -0.495078347 0.231171957 -
0.48932765 -0.364937603 -0.436697949 0.093217146 -0.48038202
-0.022237331 0.158586489 0.442236889 -0.347807114
0.269854007 0.293419128 0.370684266 0.34330231
0.466755188 0.305963718 -0.273675801 -0.017065199
0.276822962 -0.205257551 0.292310091 0.302343173
0.072457674 -0.310055202 0.090151601 -0.42121144

13738 0.502442246 -0.33203818 0.114835333 0.214822017 -
13739 0.433635044 -0.167625879 0.063905044 0.24057338 0.044230487
13740 -0.373819341 -0.337407662 0.315915619 0.126156747 -
13741 0.316499719 0.261346392 0.106798796 -0.420674691 -0.43490397
13742 -0.201872558 -0.373102794 0.415649116 0.182583332 -
13743 0.164947957 0.026890597 0.235659783 0.415538051 0.368870775
13744 0.123994548 0.544249911 0.29911765-0.487277318 0.032299812
13745 -0.341612059 -0.104446367 -0.245513054 -0.210894117 -
13746 0.491785109 -0.445183212 -0.363158979 0.245561317 0.105080775
13747 0.150197659 -0.375829478 -0.347396556 0.461308504 -
13748 0.426537364 -0.10054979 -0.301628071 -0.424077786 0.230721075
13749 -0.34319579 -0.077527798 0.246737458 0.020893967 -
13750 0.364208666 -0.037946219 -0.244167753 0.099006065 0.354698179
13751 -0.143009088 0.293657998 -0.430562157 0.184128516
13752 0.223409505 -0.454067494 -0.301100393 -0.153450399 -
13753 0.060867411 0.139161635 -0.273547851 0.487277379 -0.096251685
13754 0.360310094 0.120137717 0.113464868 -0.026023059
13755 0.212060028 0.426266417 -0.009105771 0.234018021 -
13756 0.443949484 -0.072610145 -0.369458901 0.192344126 0.59602228
13757 -0.110688495 -0.323536149 -0.593353976 0.382439194
13758 0.432564828 -0.171075395 -0.237899701 0.196740228
13759 0.406725069 -0.330955812 -0.429607462 0.154894173

0.391132507 0.281887912 -0.487564927 -0.101911079
0.387809977 -0.549575352 0.197876223 0.395990942 -
0.163768651 0.024175864 0.074714619 -0.319383499 0.232424701
-0.439078185 -0.300245344 -0.017620563 0.324723871 -
0.514488465 -0.365109804 0.256011332 -0.466882591 -0.108164708
0.223804222 -0.086753118 0.20551439 -0.166931948 -
0.320134619 -0.405435773 0.033206829 -0.170829195 0.335958148
-0.446704046 -0.514302561 -0.194832093 -0.312966737 0.1011566
-0.152207524 -0.338585469 -0.025848109 -0.262433189
0.033183103 0.519182926 0.324350023 -0.48747305 -
0.388635671 0.196457365 -0.07607499 0.263317968 -0.13946989
-0.033903974 -0.120664084 -0.10427106 0.50560053
0.36164365 0.373254427 0.476240802 -0.019776926
0.421657056 -0.143470698 -0.209369597 0.010342812 -
0.291756505 -0.076976069 -0.403153411 -0.188685598 -0.08093582
0.391492113 -0.233032599 0.237382137 0.363558245
0.252378402 0.340122228 0.268716653 -0.39114899 -
0.443341873 -0.115945092 -0.199891055 -0.274214526 0.212288809
-0.263125773 0.035129324 0.245609418 -0.46435102 -
0.388587636 0.495990194 0.270100611 -0.123501235 -0.146178787
0.022800575 -0.229855107 0.562709593 -0.401731698
0.123975803 0.570854437 0.265232605 -0.347942639

0.103898913 0.066275929 -0.076803983 -0.398792494
0.060555073 0.39579456 0.019128573 -0.069147033
0.289750796 -0.31082822 -0.166965707 0.247645342 -
0.123095347 -0.43908223 0.180307842 -0.304928921 0.095703283
-0.179046992 -0.195390275 -0.148913408 -0.161464744 -
0.119555822 -0.14442705 0.374481545 0.107563673 -0.099507928
0.05170969 -0.400925052 -0.052609555 -0.148224799
0.391234149 -0.356633507 0.52953334 -0.303316416
0.163872387 0.158029518 -0.034995564 -0.09226292 -
0.278268645 0.103004054 -0.060704233 -0.023523049 0.209688251
KEGG_GLYOXYLATE_AND_DICARBOXYLATE_METABOLISM
0.428463012 0.396844373 -0.386749171 -0.165965251 -
0.495235464 0.237192552 0.320569454 -0.503985371 0.061459242
-0.18537613 -0.377429405 0.201693171 -0.342225609 -
0.195101925 -0.347274592 0.200165594 -0.209367242 0.189761804
-0.581856367 0.188405361 -0.146464133 0.093892138 -
0.581952722 -0.012291263 -0.257444304 -0.041858707 0.261651711
-0.009251867 -0.274204233 0.039950992 0.700035987 -
0.158852602 0.445605714 0.184589532 -0.19816923 -0.378820062
-0.119464759 -0.284465002 -0.364028021 0.121668512
0.159270537 0.451139182 -0.252929956 0.573725747 -
0.168900346 0.433446293 0.136935321 -0.32420113 -0.00309853

-0.334103952 0.147902017 -0.299209022 0.384022343 -
0.24801837 0.555946795 0.422293529 -0.453438055 0.031866212
0.232771976 -0.544682129 -0.613055548 -0.383810789
0.076805143 0.187961888 0.036382853 -0.149439379
0.045499038 0.319318516 -0.088334381 -0.111587638 -
0.152548864 -0.459015266 0.350860637 0.021670646 -0.007623618
-0.23293322 0.218091985 0.197249704 0.506168661
0.346590744 -0.141605613 -0.320731108 0.293131596 -
0.147937067 -0.130433283 0.114304911 -0.399616134 0.136882392
-0.237978339 0.469817015 0.217297184 -0.336548481
0.149693827 0.481803484 0.258602497 0.053407779
0.257176671 -0.529044235 0.523748979 0.561197120.042046182
-0.372079778 -0.232677701 0.555179859 -0.14646415 -
0.196789173 0.415039637 0.568855217 -0.553117532 -0.467990654
-0.406645825 0.185068110.537959875 -0.288013035 -0.163444849
-0.36027301 0.320415257 -0.194353608 -0.410839738
0.354344454 -0.168927308 0.401778426 0.071725677
0.31640053 -0.294260998 -0.224932638 0.477563848 -
0.252427765 0.558921140.168307219 0.038247813 -0.557807376 -
0.067096137 -0.030566158 -0.025058651 -0.45211743 -0.615539236
0.343304506 -0.085942215 -0.455559598 -0.196091309
0.436866929 0.21826061 0.122529895 -0.320683501 -

0.412397531 0.10994635 0.573122022 -0.442137925 -0.114301614

-0.508722212 -0.177458703 -0.347032071 -0.44780379 -

0.25578305 -0.107282964 -0.478111817 0.312439887 0.077441213

-0.472200808 -0.201205366 0.341801645 0.240237778 -

0.372008022 0.572739097 -0.097247385 0.245467577 -0.353474487

-0.231114441 -0.579136249 0.340612385 0.189707561 -

0.201955762 0.282438711 -0.520404226 -0.281476005 -0.478938251

0.44798385 0.196317731 -0.146343591 0.036632315 0.2866461

0.23116033-0.159471905 -0.256174435 0.269002335 -0.000808053

-0.394472664 -0.345994182 0.29969886 0.270318179

0.475078329 0.281381339 -0.195300319 0.436849951

0.223119626 -0.030445563 0.382039487 -0.147382916

0.140402436 -0.248316535 0.023480325 0.046782617

0.642862504 -0.182164053 0.029166462 -0.242793744

0.317994544 -0.312246769 0.062020713 0.449974588 -

0.351911347 0.414460949 -0.064610834 -0.450144672 0.011131636

0.326171666 -0.305420959 0.457583329 -0.35943215 -

0.485309722 0.605751885 -0.127270058 0.292983886 -0.452481839

0.03806722 -0.246206037 -0.168005496 0.212953706

0.41397318 0.058705157 -0.484960866 0.078027803 -

0.355048208 0.052032004 0.277521432 -0.397458227 -0.25875626

-0.395795247 0.262928021 0.273891774 -0.239819929 -

0.024917849 0.081733172 -0.049618334 -0.264803584 0.153170167
-0.177885379 0.109461606 -0.312274014 0.090309079
0.087807649 -0.419256233 -0.362394063 -0.209017137 -
0.379114478 0.311255446 -0.387641549 0.418968032 -0.183350702
-0.248179077 0.489244053 -0.051353704 -0.035510048
0.217444376 -0.575776565 -0.218673034 0.275593206
0.137916993 -0.39671606 0.562388034 0.192940858
0.065933334 -0.202608701 0.300380059 0.241197286
0.218370993 0.218464103 -0.088550437 -0.353252875 -
0.504389788 0.023653318 -0.127599323 -0.635470743 -0.537533672
0.070238477 -0.470394342 -0.191384625 -0.342485812 -
0.179230605 0.005566237 0.184894907 0.109282115 -0.461066869
-0.494926198 -0.196799106 0.174869605 0.218727436 -
0.397341512 -0.210139199 -0.078680662 0.448960509 -0.128162112
0.293157252 0.396361709 -0.105879437 -0.233306776
0.148456689 -0.508933898 -0.234575792 -0.125846543
0.056484635 -0.473643062 -0.051549546 0.055898906
0.425961738 -0.1536709 0.062904179 0.261341821 -0.042357735
0.008383175 0.543200467 0.317643916 -0.448765616
0.259721252 -0.13838111 0.035504993 0.163104273
0.343409902 0.027156527 0.064479533 0.107871786 -
0.495257539 0.568069821 -0.293821049 0.249391279 0.208525732

-0.281109624 0.044750798 -0.173351465 0.386480788
0.192540192 -0.496385767 -0.149032798 0.516445495
0.152583364 -0.381670816 0.435303895 0.413283827 -
0.037958428 -0.402126512 0.330067523 -0.411545387 0.581173589
0.054734407 -0.396222021 0.254023797 -0.145332511
0.457583587 0.158293116 0.117111393 0.401694778
0.280976547 -0.386932924 -0.123491833 -0.411628633
0.357530255 -0.242990767 -0.333850226 -0.323651383 -
0.330091325 -0.526833761 -0.083034078 0.098223542 0.312522966
-0.333199552 -0.390972873 0.487380253 -0.434502738 -
0.013970197 -0.171147108 -0.386640048 0.169794581 -0.257332442
-0.160780275 0.202917486 -0.408271422 -0.29257983
0.179288412 -0.285142891 -0.364139183 0.189117816
0.225937719 0.373186059 -0.303630212 0.250087605
0.516960556 -0.374580381 -0.263941637 -0.249746627 -
0.341951948 -0.126578142 -0.372741438 0.5515674 0.170809572
0.359691805 0.210108308 0.050999961 -0.098642613
0.155552784 0.544662121 0.086147007 0.330503269 -
0.565748279 -0.118682873 -0.320552953 0.271076463 0.420760835
0.170827159 -0.114602334 -0.313384496 -0.166176105
0.433032529 0.035976948 -0.471091178 0.146971483
0.293296118 -0.356165988 -0.167173494 0.166681901

0.105206553 0.266332459 -0.537105632 -0.201827062
0.489998348 -0.51804062 0.342373949 0.329130085
0.034189612 0.024594255 0.100817142 -0.217070768 -
0.160066182 -0.555084096 -0.493686003 -0.142270906 0.234547162
-0.638465824 -0.213383114 0.451016538 -0.246632957 -
0.241796495 0.389365171 -0.220847694 0.084989146 0.197964736
-0.430966234 -0.637018719 -0.315116078 0.004164015
0.059946302 -0.014555953 -0.543591564 -0.53251827 -
0.195976667 0.282736986 0.210173329 -0.322828313 0.101273972
-0.102297553 -0.179482042 0.517579431 0.253725873 -
0.492625815 -0.181485507 0.103630222 -0.180829085 -0.026053509
0.409946972 -0.269895394 -0.191317591 -0.122586107
0.351045123 0.375999667 0.271655859 0.472410001 -
0.278430682 0.377885742 -0.067008451 -0.432773939 -0.379036412
-0.27836022 -0.083985699 -0.171483439 -0.278446355 -
0.069771301 0.252520644 -0.223987905 0.19421677 0.425233446
0.32877068 -0.014787437 0.444650752 -0.398259433 -0.570061
0.061554774 -0.176961208 -0.140932155 0.065884523 -
0.257493935 0.164554616 0.280754823 -0.263402876 -0.50285471
0.517806121 0.201820496 -0.129677795 0.34496678
0.38631638 -0.093475622 0.546709341 -0.469062621 -
0.075559173 0.55904987 0.219045896 -0.285772598 0.213045054

0.26568063 -0.185013056 -0.509652929 -0.298149308 -
0.023158666 0.166926317 -0.379105865 -0.082762828 0.038547528
-0.041314296 0.408713755 -0.322785047 -0.38335198
0.225954702 -0.12069513 -0.203927506 -0.300164035 -
0.045527378 -0.081346578 0.068596209 0.29983844 -0.287178626
0.267389745 -0.125282245 -0.463425937 0.019991276 -
0.415757583 0.379514571 0.047823755 0.36506371 -0.1329942
0.543178927 -0.296622483 0.301770092 -0.115381449
0.004677184 -0.159667855 -0.144375737 0.296455898
0.00819559 -0.210196782 0.014639017
KEGG_PROPANOATE_METABOLISM 0.550380423 0.562527493 -
0.337607419 0.238098958 -0.194679412 0.263100651 0.433928933
-0.410937927 -0.313879118 -0.562502015 -0.164018592
0.493666916 -0.556571994 -0.232238245 -0.423209476 -
0.436566563 0.120155043 0.261206026 -0.666847869 -0.008470296
-0.166871594 0.442523157 -0.728483562 0.116317375 -
0.601128867 -0.155284562 0.252698863 0.167569252 -0.359306371
-0.552300714 0.525166571 -0.066846688 0.582407023
0.295421772 -0.413183946 -0.335529115 0.044298832 -
0.230195333 -0.447635231 0.48456985 0.252387957 0.083204768
-0.323392174 0.415740472 -0.148332658 0.389982473
0.338294413 0.34319372 -0.16675035 -0.250688554

13936 0.433670373 -0.351840645 0.27489564 -0.32093359

13937 0.529568471 0.505788345 -0.587919274 0.272681977

13938 0.444992233 0.027736684 -0.571316817 -0.106356251

13939 0.371915425 0.103306554 -0.295649749 -0.20401744 -

13940 0.105730741 0.462316719 -0.265456206 -0.464666849 -0.326978183

13941 -0.536954985 0.618355979 -0.34499427 0.148280643 -

13942 0.292232439 0.43159339 0.560051939 0.163632494 0.574726739

13943 0.088113295 -0.058976154 0.346107238 -0.320752057 -

13944 0.442831292 -0.561480227 -0.347931923 -0.289251849 -0.530494934

13945 0.596968704 -0.290160008 -0.154036839 -0.083348149

13946 0.554722541 0.479431282 0.04422643 0.190037833 -

13947 0.623699861 0.317528023 0.524957975 0.241761894 -0.3294819

13948 0.413730536 0.291026624 -0.406743674 -0.2522884 0.437424163

13949 0.586561346 -0.443366016 -0.491876153 -0.361894539 -

13950 0.077988599 0.526391614 -0.554942929 0.357555852 0.13392949

13951 -0.075418481 -0.24147605 -0.381300968 0.517295416 -

13952 0.198019523 0.544814154 -0.205857275 0.439300496 -0.011513456

13953 -0.236750749 0.398912842 -0.469238659 0.597201107

13954 0.465573027 0.113229624 -0.578068101 -0.206270334

13955 0.434075583 0.044992346 -0.549598226 -0.611024962

13956 0.619333717 0.315945613 -0.530341835 0.154805328

13957 0.441615538 0.549474343 0.204691525 -0.426862614 -

0.200177753 0.519415514 0.587387575 -0.470842552 0.082186545
-0.635923946 -0.420362377 -0.503141259 -0.398094957
0.235651501 -0.168555255 -0.49576072 0.561001348
0.446135085 -0.256073653 -0.309963015 0.12052931 -
0.195287351 -0.501996104 0.533902254 -0.400643182 0.118089657
0.133693401 -0.397794392 -0.412572647 0.323762684 0.5626622
-0.015139263 0.019756345 -0.444203972 -0.228373594 -
0.212090005 0.587210189 -0.4085978 -0.549038303 0.045177436
0.205542333 0.353279005 0.317027113 -0.127793467
0.08431686 0.078149512 0.06673657 -0.487594822
0.57693914 -0.237207921 0.421550552 0.605634735 -
0.140016744 0.272811668 0.157502548 0.113357499 0.470607672
-0.089234574 0.445785149 -0.430700928 0.213390229 -
0.230937821 0.484937444 -0.218803466 0.18558572 -0.415444123
0.37048008 -0.260516472 0.373587356 0.643408355 -
0.238069175 0.418072794 -0.498666859 -0.091557516 -0.205409836
0.302491089 -0.130439518 0.54024163 -0.36941831 -
0.577830452 0.67545296 0.079563515 -0.47860042 -0.379782837
0.038699925 -0.235178867 -0.463918887 0.379140201
0.327205927 0.236783595 -0.45761121 0.428411458 -
0.428132563 0.436925046 0.50915717 -0.600196696 0.254285535
0.254732971 0.138062308 0.334407891 -0.09199865

13980 0.467686304 -0.438539066 -0.229906186 0.357361658

13981 0.033964393 -0.226758975 -0.003011747 -0.406698584

13982 0.053867569 -0.070524054 -0.58907699 -0.630620928 -

13983 0.150283837 -0.480822343 0.616024044 -0.439400346 0.552460625

13984 0.070827574 -0.639758443 0.156401677 0.242570076 -

13985 0.332028294 0.312974704 -0.491377508 -0.581534797 -0.105548415

13986 -0.194480367 -0.460985064 0.453727467 -0.442499723

13987 0.025460377 -0.00928349 0.144855412 0.211692620.553264048

13988 0.323059269 -0.022863574 -0.379205466 -0.567407149 -

13989 0.27732086 -0.531115422 -0.54475871 -0.668158448 0.235760727

13990 -0.565447384 -0.141550511 -0.429621692 -0.137391888

13991 0.034653623 0.210992768 0.187005683 -0.431390103 -

13992 0.482444322 -0.446358454 0.464357497 0.157048098 -0.535416702

13993 -0.163921975 0.358343225 0.504200583 0.080257181 -

13994 0.299114806 0.730330252 0.171271841 -0.529457432 0.443845427

13995 -0.65319836 -0.476533016 -0.399894441 0.043545139 -

13996 0.573084588 0.457238558 -0.053962824 0.525393824 -0.508827556

13997 0.452842671 0.531661063 0.46431597 0.176929974

13998 0.623805897 0.357347665 -0.359224075 -0.325821205

13999 0.031739245 -0.111215793 0.240249584 0.405205199

14000 0.159932169 -0.40608911 0.334353331 -0.46869147

14001 0.62996936 -0.064826326 0.38731545 0.481524499 -

14002 0.613188838 0.078211792 0.422846345 0.370715366 0.404209211

14003 -0.361997371 -0.407314921 0.507128791 -0.10968042 -

14004 0.274288394 0.479376816 0.047215489 -0.47492097 -0.56014151

14005 -0.209854126 -0.272381263 0.47172438 0.451224215 -

14006 0.195299199 0.099849897 0.484626092 0.388493492 0.142537838

14007 0.162896813 0.570866084 0.602398325 -0.59539032

14008 0.193084144 -0.262514301 -0.280278975 0.010639168 -

14009 0.379135339 -0.384925762 -0.531701382 -0.407019449 0.462847169

14010 -0.140623385 0.357026026 -0.509924022 -0.553951543

14011 0.305230352 -0.504235521 0.018410252 -0.487562916 -

14012 0.495919864 0.313702644 -0.443924157 0.005292725 0.428548329

14013 0.151524131 -0.423956693 0.051029567 -0.13378172 -

14014 0.088747288 0.361407382 -0.030807404 0.459555826 -0.22791771

14015 0.476280453 0.5182248 -0.516708268 -0.382882574 0.21912316

14016 -0.040191022 0.413587434 -0.073513178 0.660311912 -

14017 0.310715003 0.476437373 0.098501526 0.253936748 -0.276715999

14018 0.43368904 0.644997276 0.086446697 0.332620852 -

14019 0.571238905 -0.156233395 -0.218551287 0.28921552 0.670118941

14020 -0.149930779 -0.507676673 -0.619296333 0.509386029

14021 0.357093361 -0.197704995 -0.404170465 0.279456255 0.587342

14022 -0.210641719 -0.518275032 0.36082156 0.333749715

14023 0.456688017 -0.577706702 -0.038031772 0.505676484 -

14024 0.561343692 0.343788274 0.602393282 -0.256125105 0.091698863

14025 0.334054407 -0.33634469 0.508987358 -0.518254484 -

14026 0.524093118 0.202412327 0.136486317 -0.674454877 -0.450957915

14027 0.347858985 -0.537198888 -0.146905567 0.179086099 -

14028 0.164258519 0.053906782 -0.101360599 -0.393639658 -0.602924087

14029 0.137630379 -0.130990922 0.319663058 -0.298329469 -

14030 0.645383503 -0.293146203 -0.297683917 0.139672862 -0.234237126

14031 -0.490957652 0.161337526 -0.342589485 0.268111854

14032 0.508873648 0.469372589 -0.455490969 -0.579056175

14033 0.087725892 0.002633474 0.284805064 -0.023114868 -

14034 0.304968524 -0.30874376 -0.38017486 0.609291103 0.074887252

14035 0.46604998 0.612459365 -0.024675542 0.358536137 -

14036 0.025022563 -0.15203401 -0.346539742 -0.418453404 -0.17176358

14037 -0.400743343 0.014868778 -0.006006315 0.358706407 -

14038 0.372545734 0.181048287 0.154691038 0.434908722 0.570572886

14039 0.48347072 -0.446328148 -0.468368952 -0.047721843 -

14040 0.228079011 -0.254369204 -0.112463053 0.095518364 0.164141138

14041 -0.059115646 -0.510562758 -0.377144317 0.66513912

14042 0.399523386 -0.283564529 0.015355825 0.312563883

14043 0.116171784 0.57092556 -0.490035623 0.055242058

14044 0.492926639 0.441424009 -0.4753137 0.271384023 -0.323417722

14045 0.189248109 -0.293288569 0.133591459 0.336545959

0.040279084 -0.05284729 -0.024079711 -0.108699337
0.010271828 0.331255682 -0.431854899 -0.612992007
0.413447015 -0.518912714 -0.156644051 -0.120483603 -
0.018720693 0.254357086 -0.115214034 -0.369767676 0.169705412
0.481073548 0.112133223 -0.178216212 -0.091896006 -
0.549764374 0.211317841 -0.285497685 0.48406393 -0.483363069
0.542962541 -0.436612984 -0.136385866 0.17844833
0.029872486 -0.068303787 -0.252025273 0.087966857 -
0.011778506 -0.181630284 0.478352633
KEGG_BUTANOATE_METABOLISM 0.535197681 0.334051508 -
0.319070992 -0.226738575 0.134461341 0.436723223 0.261790496
-0.333491724 -0.334844186 -0.367822956 -0.197418087
0.47398837 -0.496799052 -0.210890134 -0.197624994 -
0.261645296 0.059671233 0.299530519 -0.590945211 0.074384822
-0.485619442 0.121800891 -0.600110567 -0.294516523 -
0.470011278 -0.073117516 0.306232745 0.303249748 -0.233749584
-0.333851512 0.558732676 -0.248895342 0.18345781
0.340233611 -0.481074766 -0.472769052 0.328621641
0.264440294 -0.51363649 0.097189341 0.272680291
0.373564645 -0.294729598 0.503430616 -0.011736086
0.206938351 0.015647443 0.226621177 0.033600883
0.057891681 0.545106922 -0.453342977 0.301280664 -

14068 0.446718108 0.417777984 0.458925267 -0.407816397 0.260672946

14069 0.398263308 -0.292240423 -0.51718645 0.162679353

14070 0.057831768 -0.035034241 -0.371158502 -0.312537027

14071 0.047982835 0.457890189 -0.441027713 -0.110308188 -

14072 0.359356772 -0.548797791 0.483878708 -0.447846881 -0.066849 -

14073 0.279393187 0.371899027 0.325321437 0.394469865 0.580985634

14074 0.131896235 0.033235486 0.43909956 -0.111987812 -

14075 0.138656491 -0.549744333 -0.354021255 -0.24310192 -0.524839326

14076 0.21500912 -0.031482038 -0.002639219 -0.090800224

14077 0.427002457 0.294280476 -0.064064366 0.376019668 -

14078 0.61515091 0.50452055 0.518017464 0.008254359 -0.32749711

14079 0.318815619 0.54545454 -0.332730518 -0.454434062

14080 0.306573539 0.622137092 -0.561531503 -0.283103747 -

14081 0.09042852 0.225539921 0.523702952 -0.510041649 0.257591609

14082 0.099892393 0.315342349 0.012391652 -0.252080119

14083 0.446829942 0.072286766 0.455141381 -0.191086104

14084 0.378407439 -0.172016234 0.13479446 0.372730046 -

14085 0.595158516 0.43003 0.123807438 0.309790518 -0.505178639

14086 0.060069868 0.122052271 0.235416232 -0.536343473 -

14087 0.483191581 0.506419769 0.169680863 -0.509678355 0.179636976

14088 0.378536728 0.288521433 0.018822643 -0.352540066 -

14089 0.095982996 0.233542284 0.57490382 -0.44215804 0.067784331

14090 -0.468636479 -0.506913087 -0.509175477 -0.024525374 -

14091 0.039115762 -0.166520292 -0.577987122 0.510559704 0.387593711

14092 -0.329471453 -0.246091808 -0.011729464 -0.291398453 -

14093 0.374004543 0.596209837 -0.51969833 0.283571544 0.145380383

14094 -0.000440724 -0.328732787 -0.027913973 0.267056638

14095 0.12296544 0.113941373 -0.428842998 -0.39942181 -

14096 0.316367745 0.514159476 -0.296135288 -0.436692797 0.203250816

14097 0.406973942 0.367291189 0.257452829 -0.384853391

14098 0.310223154 0.079559045 -0.168997413 -0.561809759

14099 0.42351949 -0.410062196 0.593013508 0.451454817

14100 0.001598608 0.050412099 0.23033659 0.0372668 0.27468319

14101 0.054005413 0.130531783 -0.302860741 0.265180394 -

14102 0.043383253 0.622418675 -0.291592261 0.097175987 -0.453264035

14103 0.110831249 -0.255848798 0.266792844 0.602272468 -

14104 0.226067859 0.272017009 -0.417447994 0.327394023 -0.281871241

14105 0.307852518 -0.003064018 0.363139898 -0.493224825 -

14106 0.516549586 0.500527926 0.05320277 -0.385155375 -0.456557555

14107 -0.057931646 -0.155889504 -0.262057463 -0.018300995

14108 0.36504007 0.191943617 -0.382182289 0.032744428 -

14109 0.301258934 0.027339573 0.282278029 -0.567782114 -0.263662796

14110 0.119023390.166672995 0.299480735 -0.101871409 0.399177686

14111 -0.293524902 -0.286629707 -0.032246333 0.097307846 -

14112 0.360859271 0.168708702 -0.062939643 0.030658585 0.025205487

14113 -0.450298991 -0.549931481 0.162787559 -0.339506072

14114 0.448300662 -0.248316956 0.313909806 -0.193265616 -

14115 0.525056847 0.284587549 0.215915846 -0.077034464 0.337120321

14116 -0.459532964 -0.554225659 -0.224941713 -0.262437745 -

14117 0.028040639 0.407092278 -0.407280411 0.1920077 -0.020559683

14118 0.237717742 0.26929361 0.341141475 0.248550899 -

14119 0.049841031 -0.380120732 -0.561526091 -0.076114857 -0.488975752

14120 -0.281924772 -0.598399927 0.318231035 -0.485977955 -

14121 0.142580568 -0.340450632 -0.103430801 -0.151016098 0.274462146

14122 0.184455635 -0.565990709 -0.374476648 -0.46164471

14123 0.201226731 0.011434211 -0.576040689 -0.088535959 0.1696084

14124 0.541778866 0.069127677 -0.043085457 0.699584335

14125 0.02963504 -0.40957891 0.38405378 -0.597897564 -

14126 0.168579612 -0.350384609 -0.0820427 -0.419280579 0.340744556

14127 0.007332087 0.618093197 -0.263706521 0.398428532

14128 0.215022911 0.333132904 0.318205536 0.6255015 0.419707641

14129 0.05758021 -0.32323611 0.323891853 -0.12557863

14130 0.442925183 0.369491612 0.241196492 -0.360799974

14131 0.424927881 -0.399613415 0.602964891 -0.351770925

14132 0.509840456 0.280347171 -0.464539528 -0.153239415

14133 0.215229756 0.384661091 0.081351492 -0.328182476 -

0.323696101 0.472856381 -0.199384775 -0.348316794 0.351767775
0.109932957 -0.199532828 -0.564020324 -0.181816721 -
0.480941407 0.464967366 0.396093596 0.189980278 0.200774224
0.450797165 0.514413747 0.196264037 0.059729532
0.479157095 0.425848853 -0.568084847 0.122170774 -
0.414847961 -0.092288654 0.062797855 -0.385445405 -0.255906599
-0.632620513 -0.395119914 0.122888387 -0.296448134 -
0.018202041 -0.366322663 -0.507800856 0.458878036 -0.497647393
0.026849474 -0.082818651 -0.406412478 0.470153082 -
0.199755398 -0.012885463 0.516177864 0.009029061 -0.36699725
0.136653015 -0.254102421 0.343116775 0.347975788 -
0.220657022 0.197329006 -0.036993316 0.083758 0.268220354 -
0.069131213 -0.330068498 0.001863685 -0.234621144 0.135360999
-0.144067405 0.664875595 -0.478370421 0.215725247
0.076405957 0.274857401 -0.298664713 0.203550767
0.46385132 -0.117716393 0.392382129 -0.644452839
0.011633726 -0.188381178 0.257616926 0.49697108 -
0.016952221 -0.433018189 -0.584852566 0.452344506 0.528438971
-0.055115679 -0.174090462 0.390434969 0.60221671 -
0.333525318 -0.54682256 0.333801752 0.416559011 0.153706984
-0.376238626 0.228932329 0.410893087 -0.571952992 -
0.122935961 0.584522786 -0.403703925 -0.080313183 -0.047863719

14156	-0.434548672	0.189720248	-0.300370532	-0.39076772	-
14157	0.179469754	0.266391314	-0.591536423	-0.437408313	0.20167148
14158	-0.546256736	-0.228936908	0.308858623	-0.011677251	
14159	0.336286294	-0.153834547	-0.39801645	-0.430306506	-
14160	0.279567454	-0.262054898	0.389347134	-0.044688738	-0.475516451
14161	-0.142998729	-0.461734203	0.216997353	-0.329502692	-
14162	0.250668677	0.002942263	-0.509516823	0.081717848	0.626378787
14163	0.264481507	-0.620041594	-0.453560105	-0.079614422	
14164	0.058342071	0.509507529	-0.255024499	-0.361114588	-
14165	0.347299635	-0.181904471	0.336528266	0.254613617	0.457989333
14166	0.564358883	-0.115326005	0.402612464	-0.30597057	-
14167	0.279351378	-0.080938931	-0.293630265	-0.017627961	-0.342911956
14168	-0.07676777	-0.065857099	0.264957806	-0.070011611	
14169	0.381828084	0.507175862	0.047263735	0.485465514	
14170	0.405834722	-0.44183965	-0.495809169	-0.187444081	
14171	0.176819256	-0.148092114	0.117921519	-0.161345027	
14172	0.056736288	0.21902014	-0.517213371	-0.346553612	
14173	0.542564856	0.565615231	-0.144093546	-0.156766628	
14174	0.116830729	0.193245549	0.643325424	-0.251991782	
14175	0.184455756	0.610952665	0.063943902	-0.247679403	
14176	0.12291655	-0.427876282	0.0991267	-0.295231749	0.152946691
14177	0.330316928	-0.037945928	0.198207394	0.104419868	-

0.025813564	-0.005499402	0.448336125	-0.271610651	-0.465384036
0.279805358	-0.312634901	-0.139125534	-0.163890374	-
0.048650993	0.05700818	0.078146798	-0.094748738	0.200761911
0.572229815	0.143150528	-0.226010583	0.082033073	-
0.44467391	-0.018784939	-0.217640707	0.370612712	-0.362911499
0.419715241	-0.335623654	-0.187924262	0.100207279
0.057644558	-0.321066	-0.298391317	0.277378935	0.036627726
-0.128508081	0.465354854
KEGG_ONE_CARBON_POOL_BY_FOLATE	0.378693618	0.215389457
-0.226309167	-0.029362865	-0.38722982	0.182781386
0.346108245	-0.23729733	0.023077279	-0.358992998
0.028344929	-0.080733957	-0.479981499	-0.372577197	-
0.483277726	0.286651002	0.332975928	-0.013250327	-0.590356059
0.107727137	0.173896101	0.236934039	-0.473728196
0.256086364	-0.521240949	0.46470625	0.385166681	-
0.241732343	-0.067568603	-0.138699129	0.43314679	0.1607865
0.1293974	0.162801538	-0.273379602	-0.160061695	-0.321200028
-0.293902266	-0.317322193	0.147534488	-0.317347169
0.111843027	0.057050571	0.238727446	-0.402334809
0.445001315	0.202243935	-0.453358619	0.076694648	-
0.277655984	-0.12630388	-0.104824942	0.419879655	-0.104848394
0.526531646	0.429834711	-0.579617489	0.405157821

14200 0.390599406 -0.408519515 -0.484575871 -0.222576688

14201 0.153568966 0.328722497 0.034642883 -0.032219601

14202 0.219043765 -0.023360601 -0.402142527 -0.202504281 -

14203 0.105961774 -0.359176926 -0.183788414 -0.201881403 -0.03707409

14204 -0.187186415 0.031063281 0.489531687 0.401440702

14205 0.150046515 -0.13961544 -0.355931159 0.451942295 -

14206 0.214586801 -0.128254534 -0.135709047 -0.326282679 0.138395461

14207 -0.421745545 0.289245497 0.091472222 -0.179117004 -

14208 0.157826936 0.270289062 0.357242294 0.09357844 0.304113014

14209 -0.492349236 0.330446515 0.327857734 0.023842846 -

14210 0.257170334 0.157138436 0.274665023 -0.05001107 0.24582325

14211 0.460274709 0.265056101 -0.404384399 -0.43434409 -

14212 0.443966258 0.169169826 0.390673676 -0.299163518 0.29612874

14213 0.392408552 0.068930477 -0.140379935 -0.23363778

14214 0.248072398 -0.173622511 0.390890976 0.101858762 -

14215 0.36452371 -0.048402191 -0.234911668 -0.080661392 0.154835735

14216 0.655635517 0.260295961 0.358973842 -0.369873747 -

14217 0.38857399 0.298446614 0.305662077 -0.35709754 -0.611183206

14218 0.467359266 0.247728637 -0.188061285 0.023716251

14219 0.343681807 0.099584046 0.162192636 -0.106928927 -

14220 0.368985821 0.064702693 0.242229754 -0.46373122 -0.449595946

14221 -0.492909147 -0.28065911 -0.124891002 -0.308944966 -

0.19607266 -0.284369129 -0.283669546 0.338501435 0.150894505
-0.235928361 -0.388283742 0.220212869 0.285540401 -
0.280557119 0.20339534 -0.143469317 0.187717968 0.059626262
-0.40039306 -0.240821772 0.38070347 -0.017291434 -
0.404638005 -0.048812334 -0.10752293 -0.448789091 -0.01931226
0.169324619 0.034846753 -0.288600598 -0.263613584
0.153350875 0.040910759 0.019360192 -0.259861475
0.238621463 0.079441279 -0.047291219 -0.32922512
0.275629109 0.001702401 -0.008002081 0.391535051
0.128095648 0.084237921 -0.064309199 0.123400934
0.298137375 0.225650091 0.307693017 -0.019037841
0.229809984 -0.392110471 0.121178666 -0.238927353
0.370756934 -0.445136948 0.386543666 0.004667678
0.314833011 0.152466382 0.183188866 0.497299087
0.039792658 -0.446470765 0.317591908 0.401271598 -
0.232311231 0.430991084 0.060665951 -0.35690898 0.456717854
-0.359237861 -0.017364463 -0.220656915 -0.272620135 -
0.387012795 -0.386823686 0.328263866 -0.063818645 -0.179291259
-0.115779127 0.075666278 -0.245889191 0.037748896
0.176661016 -0.492599524 -0.085833312 0.014618895
0.171294788 0.104226159 0.336319844 -0.108897899
0.394685998 0.233046661 0.105384786 0.178850915

0.026738963 0.222838712 -0.143147355 0.228029819
0.185353666 -0.492273153 -0.408524439 -0.089921568 -
0.444116185 0.427547234 -0.261411635 0.391191464 -0.130711104
-0.449903953 0.277727353 0.191431581 -0.274732082
0.27271656 -0.339802394 -0.378758213 0.191601738
0.167445362 -0.42617862 0.346571985 0.31494859 -
0.109244512 -0.377630698 0.116475818 -0.231337364 0.276590639
0.120305924 -0.055632925 -0.291668682 -0.448713775
0.085344436 -0.222707108 -0.502463778 -0.545313678 -
0.163611016 -0.193254641 0.220425186 -0.071746796 -0.062300432
0.297354142 0.116709840.328338254 -0.192080881 -0.378004116
-0.12867908 0.255873771 0.143496937 -0.18779435 -
0.155654259 -0.206031209 0.336922586 -0.212661782 0.042970484
0.294516072 0.480937791 0.019224433 0.579379077 -
0.509867257 -0.276030536 -0.161219525 0.240920792 -0.534083014
0.328620279 0.278753938 0.187404804 -0.399332456
0.560874399 0.028295974 0.164048404 -0.554947552
0.271898768 0.436352488 -0.453424757 0.164609833 -
0.398497429 0.225511075 0.033558089 0.347489237 -0.023755181
-0.253180962 0.219349567 -0.502827789 0.489352055 -
0.226366573 0.267480731 0.502054145 -0.372426315 0.038049266
-0.011073054 0.296437423 0.372651398 -0.217964916

0.046108147 0.497176338 0.033633092 0.068756997
0.463624066 0.312032332 0.025003735 -0.518152463
0.272554565 -0.178029582 0.271228773 0.281768133 -
0.32257444 -0.074151864 0.174877008 0.380701383 -0.313193292
-0.296838566 -0.135858583 0.332988535 -0.390296 0.009576241
-0.131065321 0.24056975 0.107820861 -0.306839568 -
0.021917229 -0.384223542 -0.437248026 0.064625235 0.280574704
0.385606669 -0.399146416 -0.550484481 0.481454153 -
0.230298456 0.328615102 -0.298974479 -0.525234061 0.07102797
-0.33124403 -0.20541082 0.072805838 -0.237320876 -
0.18494728 0.418248558 0.279723583 0.032718974 -0.179183461
0.016815163 0.201330054 -0.157752303 0.237530552
0.39977529 -0.463266823 0.023872496 -0.384245459
0.181067199 0.44064563 -0.109877071 0.602371801 -
0.317643005 0.354851596 0.437845057 0.290492816 -0.240062359
0.340235824 0.372517484 0.209692827 0.323809487 -
0.513998573 -0.371356175 0.171764541 0.159120843 0.373422779
-0.124879683 -0.261830801 -0.237128258 0.118078282
0.312266787 0.081956282 -0.570307143 -0.069573023
0.201150907 -0.21376224 -0.316265844 0.296981379
0.044925447 0.415716283 -0.503084622 -0.314046653
0.442794871 -0.188374491 0.26830264 0.004181159

0.240205295 -0.263518985 0.255951203 -0.279801844
0.118824755 -0.37227629 -0.325241309 0.238871052 -
0.22136087 -0.508713565 -0.225484258 0.274457468 -0.198724004
-0.293892526 0.534539147 -0.318361962 0.100502702
0.296638939 -0.232423448 -0.505833293 -0.2134964 -0.185144569
0.099524666 -0.109629206 -0.52200197 -0.373816859 -
0.255968003 0.45153453 0.124210962 -0.259226862 0.411003252
-0.258002732 0.275166416 0.010141762 0.449617382 -
0.018957076 -0.267227744 0.268919511 -0.093329039 0.169620727
0.511742289 0.162184299 -0.116323389 -0.084000277
0.39126399 -0.262582443 0.198557163 0.586738244
0.091681939 -0.079182582 -0.042763141 -0.507688241 -
0.676245954 -0.074787826 -0.168077419 0.192638074 0.200311005
0.018552619 0.159690942 -0.430554897 0.092585347
0.353486125 0.254520141 -0.102231967 0.355565888 -
0.467137757 -0.583407408 -0.013992581 -0.210673327 -0.104449168
0.075865922 0.23548596 0.028598682 -0.150549276
0.055392872 -0.109373579 0.459837427 0.072310228
0.200669825 0.368381839 0.529079577 -0.105373781
0.498921121 -0.421857317 -0.02009608 0.041738205
0.25787698 -0.451873178 -0.013397294 -0.14552522
0.130119425 -0.354941998 0.005246179 -0.088365467

0.031864398 0.059079495 0.131559635 -0.012857685 -
0.173703684 -0.105397666 -0.343205675 -0.4162202 0.130190983 -
0.211088335 -0.235865771 -0.332317435 0.141006536 0.035888723
0.378366738 0.073933737 -0.265985047 0.358125709 -
0.176499763 -0.231301026 -0.289162847 -0.414090616 0.074569806
0.126384817 0.391762129 -0.454049983 0.389388268 -
0.251377941 -0.015354636 0.136169861 -0.280186732 0.413511335
-0.339223137 0.305448848 -0.231699019 -0.128456881
0.174892911
KEGG_FOLATE_BIOSYNTHESIS 0.613494061 0.565106173 -
0.455441196 0.220837428 0.318909316 -0.129182072 -0.048836134
0.334449697 -0.021230222 -0.111175927 -0.462184794
0.227166688 -0.36359105 -0.696798326 -0.360275077 -
0.075130595 -0.242590239 -0.213400057 -0.181796716 0.054137014
-0.562425319 0.355385234 -0.530984011 -0.321679346
0.548738194 -0.335275298 0.293479841 0.179906083 -
0.39079857 -0.646604141 0.066839498 0.392614663 -0.0803663
0.599258639 -0.580225274 -0.497347412 -0.186446886
0.484697913 -0.515991383 -0.074657061 -0.101449563
0.303038607 -0.521111308 0.766500303 -0.025456618
0.462396671 0.165457875 -0.154722892 0.020596407
0.349727019 -0.050982934 -0.324237149 0.243361909 -

0.311399174 -0.296900788 0.369613227 -0.33095025 -0.381471572

0.528662529 -0.411581941 -0.465876248 -0.453832489

0.098070335 0.315916511 -0.371490737 -0.641183041

0.356539555 0.123539406 0.19715514 0.111708239 -

0.145839014 -0.164065803 -0.309197857 -0.454407316 -0.539068305

0.200453847 0.309741029 0.4319645 0.022641877 0.549569995

-0.125221062 -0.189817328 0.567037805 -0.529245432

0.189275206 0.069137056 -0.702691071 -0.290852173 -

0.048183565 0.04252383 0.263315357 -0.022937363 0.181699906

0.547268074 0.527311726 -0.477275353 -0.280288488 -

0.353167931 0.571936566 0.182989478 0.281821315 -0.20748221

0.255492749 0.4263772 0.342256877 -0.273533349 -0.246079877

0.433660094 -0.557808371 0.180094666 -0.365155776 -

0.181021627 0.548981324 -0.424033166 0.229172025 0.024443413

0.472502073 -0.050196126 -0.182829074 0.117001280.138028681

0.476863042 -0.271497405 0.144954626 -0.23368351

0.412523738 0.307543818 -0.600315326 0.472007936

0.103746579 0.244690644 -0.255942189 0.218668483

0.41848726 0.340795856 -0.559453391 -0.395709223

0.427945095 -0.476565802 -0.627150407 -0.202597665

0.27974417 -0.132023028 -0.155576598 -0.6125918-0.508663951

0.495284975 0.472330389 -0.326091609 0.504504595

14354 0.105236036 -0.385249171 -0.383835115 -0.081495397

14355 0.229543922 -0.210123744 -0.702197802 0.412546075

14356 0.606948262 -0.129183575 -0.343074016 0.338947954

14357 0.026865222 -0.373474324 0.57257508 -0.15027743 -

14358 0.095130069 -0.322908273 -0.025306035 -0.629450549 -0.161508414

14359 0.691501832 0.44487803 0.422651675 -0.438155918 -

14360 0.647592699 -0.525718584 0.539938311 0.098390858 -0.014404506

14361 0.386885065 -0.213787354 0.529983445 0.382896282 -

14362 0.209079722 0.034270406 -0.473173639 -0.14727497 -0.263504605

14363 0.381138312 0.320622544 0.691028376 0.271444708

14364 0.080697129 0.307884463 -0.549141243 -0.129504216

14365 0.571501263 -0.135961036 -0.160867083 0.321361216 -

14366 0.452274475 -0.081850459 0.567796356 -0.397769097 0.303679629

14367 -0.054792549 -0.175166803 -0.437250988 0.174358758

14368 0.457865499 0.086107338 0.16970556 -0.631579971 -

14369 0.123349336 -0.025247663 0.521350367 -0.064082443 -0.155266819

14370 -0.544487649 -0.43459592 0.349054832 0.0731907 0.345683948

14371 -0.66524285 -0.20213914 0.370180225 0.260437495

14372 0.377900808 0.385286578 0.420802913 -0.339874 0.103494544

14373 -0.314732747 -0.478679875 -0.459057648 -0.341430142 -

14374 0.291700094 -0.232920787 0.585009243 0.314938584 -0.107452581

14375 -0.536860333 -0.259883914 0.228899119 -0.222750832

0.120879365 -0.432719834 0.149979352 0.294050053
0.097088237 -0.402978574 -0.048744872 -0.580011244
0.113941632 -0.456054877 0.442160687 0.142643101
0.484744531 0.466649035 -0.126654209 0.264174416
0.248902993 0.02907392 0.201481995 -0.644017728
0.218442167 0.292754767 0.433431088 -0.193217396
0.604750726 0.421604347 -0.057260585 -0.031621921
0.39789184 0.412597941 0.0717979 0.400663103 -0.296554468
-0.233553114 -0.743558563 -0.097759785 -0.051390016 -
0.172573836 -0.247585323 0.318231266 -0.503125898 -0.147005865
-0.526553802 -0.205356284 0.489094037 0.331836523
0.080679189 -0.540412824 -0.615247188 0.150607672
0.330470953 0.215044554 0.456021591 0.430796221
0.002622542 0.280798828 0.428148634 0.249731015
0.565643958 -0.544291498 -0.243159195 0.365754294 -
0.405392979 0.058624237 -0.446455657 0.651135531 -0.105662216
-0.579107867 0.30803957 0.174172271 -0.207579102
0.463578833 -0.03355151 0.172502821 0.047855148 -
0.189674548 -0.289477775 -0.251116299 0.403939657 -0.054602486
0.412436548 0.25744688 0.295473325 -0.498236682 -
0.570763517 0.287282893 -0.522383973 0.586166949 -0.630011679
0.158048907 0.087890949 0.036018135 0.222426102

14398 0.184021578 0.733005021 0.200581608 -0.312772679

14399 0.356659413 0.371617561 0.384245851 -0.525861897

14400 0.704566142 0.385396045 -0.712080743 -0.61523519

14401 0.021393038 -0.499934876 0.357907478 0.485820878 -

14402 0.177176846 -0.246494905 0.352839279 0.346809741 0.415653475

14403 0.498969824 -0.163932688 0.342039219 -0.513500693 -

14404 0.46263214 -0.467786312 0.094007399 -0.516368132 -0.306164711

14405 -0.607545788 -0.284093407 -0.590811894 0.264753202 -

14406 0.322135274 0.2395515 -0.138500008 0.396846607 0.454098346 -

14407 0.321035048 -0.073911861 -0.283663379 -0.120051698 0.190541557

14408 0.229327136 -0.32107319 0.113297731 0.091963644 -

14409 0.215853092 -0.065011243 -0.457905305 0.608781937 0.466882442

14410 -0.130103231 0.463404267 -0.375429231 -0.199315409 0.5554478

14411 -0.398489016 -0.690944259 -0.353792238 -0.342241426 -

14412 0.562512218 -0.274266976 0.348800113 -0.285359181 0.486922247

14413 -0.13260671 -0.446043357 -0.332251572 -0.369518515

14414 0.622508904 -0.537557486 0.6539266 -0.677024608 0.136240544

14415 -0.55874121 -0.236721876 0.274802696 -0.129734798

14416 0.208295807 0.000268498 -0.049386047 0.027054409 -

14417 0.440055552 -0.173986804 -0.171140072 0.276653314 0.316960541

14418 0.342730741 0.256103971 0.035027399 0.675485421 -

14419 0.263405937 0.395506818 0.129402112 -0.489326425 -0.079638333

0.006742937 0.104625439 -0.620877508 -0.03284055 -
0.642572675 -0.221402599 -0.496216805 -0.38469386 -0.144848784
-0.053785151 -0.367889855 -0.284811246 -0.270866636 -
0.271910288 0.081870287 0.607139955 0.060400723 0.743702752
-0.093008558 -0.588245643 -0.545552165 -0.144860555 -
0.267840274 0.409889665 -0.184759603 -0.410777563 -0.249888228
0.000663306 -0.105124889 -0.36812316 0.087875613 -
0.351300757 0.075482562 0.293719389 0.106520147 0.474809205
-0.74207953 -0.117723004 -0.130247225 0.36682016
0.298000892 -0.152687613 -0.544428394 -0.08097117 -
0.143984072 0.505410405 0.082832811 0.401151077 0.36649426
0.101444736 0.566601202 -0.476678317 -0.630460882 -
0.093504991 -0.536260795 0.094490281 -0.233971275 -0.348941657
0.051082896 0.129277906 0.35240241 -0.024825251
0.721334982 -0.01067403 0.290124726 0.513281484 -
0.481724842 -0.670286827 -0.114507064 -0.216960456 -0.078593683
0.262618407 -0.442268316 -0.434599946 0.022968007 -
0.483946015 -0.421001356 0.228197785 0.342779425 -0.377087796
-0.019064979 -0.15566479 0.299635329 0.409406124 -
0.258958723 -0.198195478 0.267059881 0.598974359 0.013992674
-0.487486954 0.592192254 0.231110862 -0.567888307 -
0.045859808 0.022356445 0.057517676 -0.401159058 0.578532923

0.195978733 -0.049600058 0.00376307 0.088331844
0.061257216 -0.383916515 -0.36598454 -0.007303802 -
0.353334093 0.017019149 0.191184946 0.172355043 0.479887792
-0.423861943 0.354747096 0.160427981 -0.095318789 -
0.007194841 -0.38990742 0.100744257 -0.152407426 0.162747752
-0.108404441 0.634689066 -0.145714286 -0.369889421
0.012516676 -0.317396316 0.129534119 -0.186903515
0.469723349 -0.168851955 0.100848706 0.07347369
KEGG_RETINOL_METABOLISM 0.17796111-0.122709749 -0.334267421
-0.380097736 0.472127229 0.290624602 0.137963867
0.332001978 -0.472355937 0.046972474 0.095580498
0.180906103 0.136676721 -0.227309314 0.297341701
0.227088359 -0.350259896 -0.091948047 0.071728075 -
0.166234766 -0.394124901 0.206280545 -0.354340827 -0.514763602
-0.083183083 0.314110918 0.339264666 -0.057813375 -
0.045493117 0.323174869 0.451302748 -0.146621594 -0.068924701
0.242482187 -0.297030705 -0.083086412 0.316500015
0.330437077 -0.113875909 -0.010734847 0.296469545
0.131893128 -0.436436926 0.271237517 -0.220493899 -
0.411360803 -0.139180171 0.345913869 -0.056797012 0.306153024
0.159418199 -0.216781955 -0.218093167 -0.238978028 -
0.171523122 -0.09172144 0.255522782 0.058270553 0.094988292

-0.392192013 -0.469510141 -0.113708545 0.00647005 -
0.274581025 0.090283895 -0.235529248 -0.138445344 -0.064543582
0.460576589 0.420389223 -0.453247519 -0.360777516
0.169921613 0.293470182 0.210932519 0.32143545
0.344297012 0.154052513 0.370339424 0.358932195
0.329869472 -0.456390116 -0.047125295 0.184291044 -
0.159268534 -0.35367707 -0.576683204 -0.179567524 -0.007611981
0.481736112 -0.176502095 0.362052795 0.394364574 -
0.081291916 -0.405490651 0.084985248 0.313117277 -0.077465827
0.36224748 0.349171726 -0.038597755 0.419696254
0.154004226 0.225493132 0.021438103 -0.180893614
0.285204821 0.408593705 -0.486255415 0.410215318
0.547550232 0.222118416 0.400490633 -0.455125987
0.411688748 -0.248437143 0.364552698 0.39620744
0.372923087 0.115225350.519832185 0.376670622 -0.080594136
0.420025655 0.057268605 0.545806457 0.323103615 -
0.432791217 -0.059815766 -0.324487755 0.027434756 -0.025221273
-0.14374241 -0.019886877 0.066186554 -0.491244243 -
0.529821718 -0.03708224 -0.234770449 -0.529786656 0.459493523
0.341740515 -0.236251811 0.217288418 -0.489337554 -
0.38568616 -0.071459904 0.469262437 0.391530041 0.290227432
0.265837486 -0.46708405 -0.441690568 -0.004068521 -

0.225497834 0.489329073 -0.345064903 0.452759635 -0.181499478
-0.162645097 -0.320578344 0.318736559 -0.166778253
0.099911442 0.185741005 -0.321380957 0.34768808 -
0.166030249 0.527450324 0.065181457 -0.349728031 0.487420579
0.473805201 0.419290381 -0.434767663 -0.508583595 -
0.396084276 0.200595747 -0.228806976 -0.226022652 0.17426334
0.318406719 0.267773331 -0.097873157 -0.355530534
0.065857897 -0.348781429 0.040618506 -0.042110517
0.263118976 -0.186813762 0.437132867 0.352472675
0.164995221 -0.091738371 0.266679266 0.103081995 -
0.042354119 0.208623068 -0.121345458 0.028702024 -0.024821436
0.001593825 0.641445403 0.00047186 -0.106774078 -
0.055269049 0.032906262 -0.374594498 -0.080065932 0.083617551
-0.228393406 -0.177170685 -0.181655127 0.50658659 -
0.418392267 -0.136846408 -0.156049181 0.303934873 -0.365849166
-0.18552541 0.114413587 0.451768484 -0.530251808 -
0.074529021 0.128594117 -0.16294154 0.384570601 -0.173755685
0.367277133 0.470959999 -0.30923866 0.271617382
0.066052110.170553337 0.144340165 -0.20073962 -0.070561751
0.059573867 0.028368428 -0.292119799 -0.438331973 -
0.205202631 -0.349904129 -0.112811862 -0.205586637 0.332947985
0.095042888 0.238281332 0.469492833 -0.32630743 -

14508 0.242738836 0.043710309 -0.526285752 0.203571592 -0.346672931

14509 0.340288494 0.291526827 -0.152018497 0.327964729 -

14510 0.359539812 -0.047357934 0.17811198 0.374440331 0.026324974 -

14511 0.194027854 -0.163734728 0.302827231 -0.007280452 0.496080123

14512 0.43053198 -0.480040843 0.02055827 0.065940701

14513 0.058740736 0.128779881 0.162396401 0.201353047

14514 0.104068623 -0.328746527 0.029857433 0.237309487 -

14515 0.39467425 0.428364942 0.105942288 0.412973555 0.138232227

14516 -0.230040168 -0.221342782 0.377497316 0.216702468

14517 0.341031034 -0.256023452 -0.295624674 0.228918839

14518 0.337916966 -0.019933559 0.150553775 -0.341815541

14519 0.391786587 -0.123370562 0.350184493 0.319193767

14520 0.344735624 0.526041155 0.016407387 -0.347219547 -

14521 0.128858659 -0.35250686 0.388825103 0.039148115 0.21220766

14522 0.028231711 -0.285881294 -0.099998709 -0.138765284

14523 0.288504812 0.247723255 -0.192551418 -0.386268074

14524 0.485150179 -0.265297645 -0.132164966 0.375621238 -

14525 0.17263045 0.16796658 -0.242859455 0.398667271 0.279587005

14526 0.333115587 -0.185592804 0.233887159 -0.296554203

14527 0.329997844 -0.460174913 0.042307365 -0.219147921 -

14528 0.472880333 -0.386021376 -0.13684338 0.073769563 0.045080382

14529 0.23803583 -0.045956827 0.115043389 0.356993996 -

14530 0.566896784 0.142479774 0.362294107 -0.3864463 -0.402656689

14531 0.031870665 -0.515411671 0.528976816 0.105364306

14532 0.337350916 0.047385488 0.032588501 0.268611320.488257002

14533 0.099318972 0.149625686 0.116862619 -0.319955929 -

14534 0.177975475 0.045510083 0.259357081 -0.439830341 -0.163423273

14535 -0.396953352 -0.32506879 -0.659818313 -0.109372194

14536 0.095826234 -0.036913468 -0.033206618 -0.165085168

14537 0.260469586 -0.55939349 -0.37203709 0.142217495

14538 0.089375877 0.004316163 0.140966887 -0.473070511

14539 0.219550427 0.133333509 -0.376973538 -0.481143103 -

14540 0.25412179 0.038710877 -0.092780029 0.000846296 -0.06502304

14541 -0.185419981 -0.075491801 0.336778108 0.09344115-0.159839023

14542 -0.063987619 0.20183305 -0.360971829 -0.491138028

14543 0.369905489 0.08057922 0.364231633 -0.086087361

14544 0.038326521 -0.192923245 -0.331053906 0.419737804 -

14545 0.381695382 0.567407608 -0.27583531 0.191665815 -0.165343506

14546 0.052598716 0.354796214 0.11410455-0.327593216 -0.265783958

14547 0.25449701 0.105060105 -0.5042983 0.144522327 0.21006098

14548 0.594009587 -0.416628903 -0.345265639 -0.254838172

14549 0.108179633 0.276137536 0.294530596 0.546960531

14550 0.372529437 -0.356627347 -0.244957546 0.12701737 -

14551 0.306544267 -0.331978893 -0.248985634 -0.449740755 0.15801915

0.39311845-0.047085155 -0.0629168 0.313628373 0.005109869 -
0.228185278 0.269671327 -0.469215293 0.455347351 0.197677648
0.367541127 0.404287627 -0.077470501 -0.43944733 -
0.349108794 0.108214532 -0.21426488 0.40266716 0.164800563
-0.005334143 0.046485846 -0.382973367 0.099325871 -
0.295425911 0.235492212 -0.210957727 0.026147825 0.213990442
0.369831299 0.038006625 -0.446749558 0.030248587 -
0.28073106 0.369971247 0.433970573 0.294450244 -0.357270566
-0.437649187 0.152447119 0.114738117 0.426955853
0.536493404 -0.082634103 -0.090312539 0.475359418 -
0.141797419 -0.503256522 0.456403006 0.32499609 -0.13208606
-0.390425566 -0.21054279 0.437391876 0.435061967
0.244156477 0.385933384 0.474602377 -0.304917013
0.095255472 0.182343323 -0.105620057 -0.52853407 -
0.217494575 0.116616423 -0.255772144 0.49512452 -0.373393411
0.224755134 0.155363463 -0.44990893 0.324928759
0.059429697 0.294404868 0.00088568 -0.238964511
0.200448364 0.348850501 0.240317793 0.04338512 -
0.507082524 0.524966638 -0.358075025 -0.351775426 -0.175640023
0.22318866 -0.297587021 -0.504054801 0.337565428
0.203848532 -0.20332953 -0.11402525 0.211307286
0.339165499 0.242936721 0.121376898 0.029543498 -

0.071230847	0.515761453	-0.260493676	0.015409507	0.165478565
-0.100548872	-0.010705764	-0.153178164	-0.208072153
0.298533456	0.423244315	0.157375243	-0.471864122	-
0.084157285	-0.004795014	-0.089205846	0.041638654	-0.045920225
0.131733891	0.067964084	-0.27238331	-0.227729865
0.14709768	-0.025379055	0.221092352	-0.047762247
0.074305939	0.193785403	0.026084096	0.225876454
KEGG_PORPHYRIN_AND_CHLOROPHYLL_METABOLISM	0.407874796
0.319300746	-0.273247036	-0.434827936	0.045434256	-
0.224605695	-0.059149719	0.068366209	-0.356828568	0.131316656
-0.218777054	0.058326102	-0.349146177	-0.387192797	-
0.175530097	0.367774165	-0.000431881	0.415188143	-0.406038214
0.259089303	-0.302481251	0.45988426	-0.453542619	-
0.287806474	-0.072586176	-0.077960979	0.32441582	0.267299171
-0.012616391	-0.003707501	0.42459191	0.024758131	-
0.05211137	0.197182662	-0.033574191	-0.184337852	-0.32248646
0.349348169	-0.549955915	-0.221235338	-0.003312166
0.138170976	-0.51212392	0.455236614	-0.329965121
0.093987124	-0.300824122	-0.250527313	0.21604706
0.062111086	0.11748151	-0.330829277	-0.04138038	-0.634541317
0.370355731	0.361843377	-0.2889891	0.487788091	0.269047273
-0.3953337	-0.249713951	-0.227044302	0.086496533	-0.208248974

-0.408446267 -0.266169516 0.022999703 0.161689572 -
0.066882174 0.006766103 -0.175933184 -0.079907627 0.053130538
0.303742252 0.223853703 0.156747284 0.20983327
0.209636808 0.572106055 0.466831759 -0.281438263 -
0.275691506 0.481223718 0.024299304 0.274607849 -0.171370948
-0.565574806 -0.063915491 -0.380908455 0.240133928 -
0.105509694 -0.226142319 0.102753741 0.383910101 0.116822765
-0.39824275 -0.025179763 -0.374801554 0.384227895
0.262235512 -0.042962947 -0.105152493 -0.232273528
0.450778882 -0.056635241 -0.29312329 0.168003966
0.457967494 -0.575820572 -0.268284405 -0.216117619
0.289086143 0.510375407 -0.459316404 -0.141173048
0.339454304 0.432525422 0.028353937 -0.206555717
0.042728448 -0.159631617 0.362718097 0.280916696
0.14127751 -0.382538687 0.209351428 0.362418743 -
0.347466231 0.320967521 0.101576633 0.174983184 -0.271148979
0.166934651 -0.025892793 0.175856913 -0.414709355 -
0.387104815 0.213967736 0.114296642 -0.2627631 0.37645445
0.502830414 0.03144025 0.086749128 -0.19563151 -
0.189170434 0.035461282 0.321217086 0.159224102 0.307459778
-0.000685947 -0.19124843 -0.218909862 0.138813405 -
0.239334344 0.154082119 -0.442704592 0.481146801 -0.389130098

-0.16869569 -0.162753036 0.406054386 0.021687921 -
0.462379402 0.524802817 -0.14662049 0.418933024 -0.345163054
0.180808318 -0.247211863 -0.022940675 -0.125523205
0.027632846 0.336446395 -0.423831238 -0.372584563 -
0.232591463 -0.059236159 0.237661591 0.245224022 0.372050632
-0.051637673 -0.072114503 0.164773674 -0.346393562
0.265282074 -0.289993314 -0.113574745 -0.297508678
0.078574751 0.201945489 0.463090887 0.028782298 -
0.455657395 0.383141318 0.299526053 0.044398543 0.028882554
-0.020162688 -0.009041926 -0.002116207 0.443631433 -
0.337644085 0.528043597 0.3948633 0.296525148 -0.20980244
0.459306354 -0.331486179 0.478457773 0.34853678 -
0.073595018 0.055284511 0.103009897 0.316974298 0.125974917
0.486097413 -0.316958871 0.235336468 -0.438294165 -
0.113032315 0.315571019 -0.077581337 -0.309854218 -0.300564541
-0.200889028 0.345261298 0.242441302 -0.125360488
0.383422586 0.385540376 0.178501197 0.058672674 -
0.184353717 -0.115635716 -0.109537356 -0.352699856 -0.353857769
0.108091102 0.290695838 0.314543729 -0.409731916
0.10422025 0.037090725 -0.115376752 -0.176809971
0.439128352 -0.231240788 -0.141069057 -0.094335982 -
0.324230852 -0.0876229 -0.345048086 -0.541535334 0.102269826 -

14640 0.475221159 0.191637982 -0.198476596 0.1490479 -0.264541869 -

14641 0.352908531 0.431469793 -0.166759726 0.114666019 0.31404605

14642 -0.356478915 -0.35915115 0.462879782 -0.052945612

14643 0.13492394 0.431351755 -0.262298241 0.47382972 -

14644 0.001891362 0.181699165 0.340417706 0.022389997 0.185284727

14645 -0.420851391 -0.300087185 -0.519263329 0.378532907

14646 0.248493492 -0.153136742 -0.443339323 0.30995296 -

14647 0.29768649 -0.13458466 0.000681668 -0.307782315 0.004256472

14648 0.4163887 -0.222210887 -0.19803402 0.015585819 -0.147028109

14649 0.012645916 -0.118508566 -0.102180422 -0.131646485 -

14650 0.25873339 0.209065596 -0.024902196 0.244748954 0.527670722

14651 -0.062977542 -0.240708321 0.058774105 -0.420877249 -

14652 0.034411289 -0.200311289 0.127854902 -0.34928686 -0.143883439

14653 0.358087502 -0.067715648 -0.344723121 0.111803342 -

14654 0.031250611 0.20998382 0.006693554 0.265093096 0.201747135

14655 0.033395073 0.362038838 0.294555584 -0.119680333

14656 0.345887524 0.094519559 0.31770933 -0.229162028

14657 0.070475224 -0.389200869 0.535727984 -0.523263753

14658 0.244477158 0.014912569 -0.316583501 -0.469001152 -

14659 0.218221792 0.290679354 -0.011914856 -0.17142918 -0.438146849

14660 0.123624768 0.332360529 -0.508014259 0.086066868

14661 0.046646546 -0.231369844 -0.492310043 0.161012705 -

0.364465092 0.525495336 0.119425522 0.050269458 -0.222662717
0.446166061 0.321374761 0.456423059 0.217021604 -
0.058747464 0.28187053 -0.275340196 0.340487978 -0.33439823
0.07536738 -0.385333005 -0.11513693 -0.4972555 -0.303980536
-0.530256286 -0.044289418 0.248812349 0.159935037 -
0.254533703 -0.439995267 0.335743475 -0.410504674 -0.088585712
-0.283172812 0.018740836 0.183899337 -0.170259692 -
0.291866356 0.139866284 -0.252573326 -0.286102779 -0.145912124
-0.085842586 0.462701561 0.259017243 -0.391950046
0.347850523 0.006646308 -0.260372261 0.269204405
0.002486715 -0.406269516 -0.400269265 -0.387166378
0.078899424 -0.494110459 0.411436933 0.281696791
0.217163231 -0.402415384 0.017039392 0.019553528 -
0.08436247 0.411234668 -0.447023473 0.43856283 -0.240994475
-0.261354589 -0.070375314 0.329465287 0.484515394
0.012700021 0.464080598 -0.338880597 0.270788861
0.252446382 -0.003540417 -0.044088498 0.451270449
0.444571557 -0.313923314 -0.11874087 -0.044522709
0.261970419 0.100075682 -0.019573666 -0.170893128
0.054695018 -0.47829345 0.354139767 0.031152396 -
0.00754595 -0.110306278 -0.15157812 -0.465710656 0.284854932
-0.284955777 0.012050854 -0.057312369 0.033766104 -

0.463475973 -0.393936178 -0.000975801 -0.25816882 -0.183539315
-0.332407376 0.256949534 0.430970181 -0.368241222 -
0.467412364 -0.440768523 -0.397978172 -0.019311432 0.11421409-
0.055078392 -0.203150491 0.055513876 -0.121000916 0.370842133
-0.046894781 0.032530586 -0.110869641 -0.016436652 -
0.05883432 0.448509778 0.342405558 -0.547142009 -0.037989223
0.193696621 -0.111189468 0.356064968 -0.026042057 -
0.282309955 -0.49772484 0.223596226 0.008997604 0.111444077
0.54124777 0.333297185 0.239116982 0.442600974
0.298783272 -0.32882303 0.014154073 -0.151382876
0.04110989-0.318827358 -0.099509972 0.024732221 0.476935626
0.334385506 0.451155226 0.370604294 0.329059993
0.271961729 0.432902482 -0.056531515 -0.569130956 -
0.033895413 0.174785239 -0.125283943 0.520204077 -0.309634508
-0.169291503 0.281450159 -0.446130917 -0.04075982
0.026556162 0.549885745 0.247602705 -0.051206753
0.190101305 -0.112487441 0.534391393 -0.302154436 -
0.092884824 0.41408947 0.147222322 -0.334069881 0.391588854
0.300725537 -0.305723613 -0.362160623 0.058936216
0.025708805 0.246252835 -0.105601832 0.114174319
0.179326632 0.079236397 -0.041276097 -0.122622891 -
0.328986751 0.391958214 -0.237214776 -0.055939658 0.352334005

0.053313733 0.117101687 -0.093050069 -0.109256335
0.226088116 0.427949105 0.403043606 -0.5263328 -0.002691367
-0.020533331 -0.289370442 -0.298772427 0.403594595
0.007032247 0.321096775 -0.165834532 0.20502384
0.156908916 -0.108623273 0.152008249 -0.168529134
0.059578526 -0.467299399 0.149735013 0.107078359
KEGG_TERPENOID_BACKBONE_BIOSYNTHESIS 0.452825953
0.570317578 -0.298034866 0.178685762 -0.298279818
0.227110769 0.226255856 -0.377956518 -0.20807205 -
0.253746839 -0.253538186 0.311137011 -0.661967074 -0.219347746
-0.388275607 -0.32221978 0.275759052 -0.029901063 -
0.543271519 -0.170564818 0.170617265 -0.11057341 -0.63025608
0.440173104 -0.356378661 0.090071699 0.144584889 -
0.283686765 0.11695132-0.416926303 0.586968757 -0.274155925
0.122991036 -0.287450616 -0.542914977 -0.318706506
0.110427468 -0.211841318 -0.384588504 0.475135734
0.061129914 0.016081327 -0.07267855 0.603231362 -
0.126146731 0.138556366 0.29014713 0.206398233 -0.427276487
-0.327420765 0.576462376 0.118502080.123821217 0.114161135
0.076117204 0.187063592 -0.396048669 0.036474117
0.548055412 -0.276522129 -0.455837257 0.484875895
0.32860356 0.012678891 -0.408083069 -0.477878701 -

14728 0.2946455 0.247546475 -0.566879078 -0.323140983 -0.47399339 -

14729 0.429128058 0.102467123 -0.214626615 -0.104390608 -0.38190886

14730 0.369272 0.346855436 -0.065804674 0.592469348 -0.405881798

14731 0.099924314 0.546962691 -0.141302143 -0.158766052 -

14732 0.351909383 -0.091269172 -0.027584896 -0.391043984 0.585663954

14733 -0.143029673 -0.408371056 0.309925867 0.610545925

14734 0.430390397 0.079264384 0.165415734 -0.52648648

14735 0.40682409 -0.013336245 0.009578762 -0.436692173

14736 0.129982679 0.033369076 -0.362504671 -0.492928812

14737 0.292506753 0.401316458 -0.431508944 -0.200436729 -

14738 0.393353203 -0.043574105 0.533390468 -0.215828371 0.307248966

14739 0.062973524 0.382154107 -0.417920986 -0.263784679

14740 0.214766672 -0.330520599 0.314192357 0.094520096

14741 0.323629433 -0.266422343 -0.306249198 0.207561066 -

14742 0.522510799 0.590105725 0.246649886 -0.06401331 -0.616892512

14743 -0.260038784 0.182621526 0.432410355 0.300107049 -

14744 0.569110524 0.696637362 -0.152059351 -0.156267227 -0.087987294

14745 0.255107511 0.250854571 0.157413138 -0.039255988 -

14746 0.055795975 0.038918696 0.479146165 -0.707852982 0.025345258

14747 -0.391188427 -0.288201095 -0.452538593 -0.225614745

14748 0.377379395 -0.041892977 -0.181085213 0.078432215

14749 0.295818554 -0.560766541 0.098616665 -0.017517171 -

0.168455704 -0.58176951 0.655560004 -0.397980785 0.104482585
-0.026383291 -0.513757425 -0.526832636 0.549368862
0.060070102 0.005533913 0.457524309 -0.242624229
0.082816687 -0.346005696 0.506789293 0.106496488 -
0.256351796 0.028228606 -0.037599605 0.03568251 0.128265762
0.309396579 0.048915414 0.021246947 -0.0146748 -0.500835398
0.564749679 -0.065363302 0.438298149 0.46939745 -
0.477494331 0.332568968 -0.058606875 0.067403134 0.102689532
0.004662701 0.232947138 -0.313288931 0.193399191 -
0.05592578 0.645802476 -0.387259855 0.213349295 -0.365754185
0.314594286 0.180572889 0.340467785 0.580852969
0.412721044 0.415996865 -0.531393722 -0.212028019 -
0.044904446 0.522296677 -0.385424691 0.198765294 -0.45403783
-0.530628835 0.491443541 0.089574233 0.172297856 -
0.556956518 -0.073003192 -0.141061424 -0.294774672 0.164256132
0.366747693 0.115871826 -0.052637702 0.262977097 -
0.283270919 -0.019539026 0.138751021 -0.729918943 0.449047353
0.062802254 -0.023517777 0.301699871 -0.182131083
0.116360482 0.27595771 0.07237091 0.055535739 -
0.057852673 -0.482491196 0.001624654 -0.137449405 0.202102121
-0.025220667 -0.405030489 -0.42435995 0.048532351 -
0.519960473 0.285123482 -0.361904069 0.555565585 0.392052824

-0.563668956 0.403955139 -0.018120375 -0.270657881
0.200153901 -0.083325301 -0.426284536 -0.049069969
0.132700048 -0.265965293 0.464726906 -0.312892766 -
0.009148573 -0.002349421 0.368586539 0.174467384 0.463039485
0.013018392 -0.1411103 -0.38423816 -0.621504904 -0.161108063
-0.158079704 -0.45315223 -0.554281585 -0.03257541 -
0.510929572 -0.147106707 -0.167141194 -0.134238587 -0.174002027
-0.084027497 0.556531416 -0.389622489 -0.58917034 -
0.310876719 0.246376421 0.111122235 -0.51304969 -0.086275034
0.530642242 0.258355608 -0.372745698 -0.637689416
0.506982718 -0.470036448 0.049731928 0.089903008 -
0.507553129 -0.121106769 -0.434762003 -0.15872983 -0.628226732
0.343201012 0.412197684 0.439488851 -0.596249155
0.228910863 0.457133554 0.117441044 0.180867198
0.450280523 0.533046361 -0.35985218 -0.196200158 -
0.000293148 0.192184516 0.280877966 0.408478599 -0.000812199
-0.431455352 0.403287467 -0.53134649 0.510424684 -
0.318330139 0.406578944 0.508254485 -0.656972239 0.014445912
0.491133682 0.242221728 0.355590378 -0.561039844 -
0.023431874 0.52408253 -0.02671795 -0.044422685 0.328733074
0.026974066 -0.448135969 -0.075114572 -0.171799444
0.070968594 0.627095946 0.199566929 -0.249109219 -

0.035525643 0.017837231 0.258134142 0.584046918 0.058837613
-0.088005794 0.665401465 -0.55417073 0.320523223 -
0.460336008 -0.285625503 -0.036748323 -0.201783869 -0.255637687
-0.448619608 0.202945254 0.370550391 -0.191766619
0.580202102 -0.516817328 -0.309988651 0.103799588 -
0.427753626 -0.273380915 -0.253819848 -0.265487255 0.001612604
-0.35313842 -0.209766669 0.488403291 -0.012804152 -
0.281080697 0.150378737 0.380921764 0.377783948 0.167051172
-0.157807011 0.170797525 -0.385969688 0.129711781
0.551320414 -0.545428491 -0.268778888 -0.012345475 -
0.179904425 0.163536383 0.186880927 0.534978753 -0.234805569
0.435551943 -0.04508771 0.185375583 0.068319417
0.206598615 0.590414694 -0.163454651 0.159050943 -
0.527036136 -0.27193238 -0.47249704 0.316935528 0.437212398
-0.083649421 -0.108016456 -0.127889135 0.401513942
0.090582631 -0.1541181 -0.450845951 0.196343088 0.102372073
-0.069516249 -0.190116227 0.130177209 -0.308511444 -
0.057056048 -0.466890018 -0.055720496 -0.166849122 -0.237908585
0.072756181 0.566568578 0.296204298 -0.090216196 -
0.068703336 -0.079483936 0.282434649 -0.540134546 -0.385306802
-0.285892268 -0.099031395 -0.429981788 -0.182430796
0.087506183 -0.298873847 -0.056621183 0.441932077 -

0.158762735 0.449957642 0.165681073 -0.245286131 -0.538249765
-0.070011685 0.002309785 0.382122208 -0.149866754 -
0.638889615 -0.010896808 0.036996711 -0.046008852 -0.061561618
-0.348250611 0.037212467 -0.127033658 0.273909488
0.075977457 0.341567176 -0.28750715 -0.548917471 -
0.149715741 -0.22837889 0.254294759 -0.021237178 -0.225432862
0.445714199 -0.128517858 0.580280363 -0.080644792
0.160777346 0.573166295 -0.069532578 0.131845081 -
0.198437718 -0.08558904 -0.186864045 -0.515732771 -0.0234141 -
0.189708874 0.235283224 -0.101849044 0.11149065 -0.223354521
0.10320227 -0.028460916 0.217821405 0.30131014
0.321775952 -0.322131187 -0.413507828 -0.259651506 -
0.317304436 0.117188765 -0.244856659 0.26462948 -0.179541463
-0.320385244 -0.340571062 -0.288645065 0.368448733
0.430823302 -0.174613367 0.128321269 0.063087296
0.076769978 0.657328153 -0.344724915 0.266692758
0.385494064 0.413118701 -0.037479012 0.135594921 -
0.207782876 0.452504578 -0.180373371 -0.09394625 0.039560387
-0.000383288 -0.319433402 0.069708356 0.097024167 -
0.097598442 0.3446398 -0.174207269 -0.270354761 0.290027441
0.207480716 -0.161800984 -0.376239428 0.152216929 -
0.380167894 -0.397748295 0.14822032 0.028924709 0.577245764

-0.097788881 0.149694981 0.349862105 -0.440351924
0.042746729 -0.138628462 0.299755318 -0.225366953
0.24472643 -0.233774315 0.371046856 -0.273947706 -
0.334271693 -0.112298329 -0.328792055 -0.17855196 -0.139671329
-0.150558116 -0.213056771
KEGG_NITROGEN_METABOLISM 0.223099024 -0.148070643
0.275252333 0.192298994 0.39257451 0.355310741 -
0.123220661 -0.122285242 -0.285305333 -0.309496161 0.325625534
0.409223575 -0.175991374 0.171461757 0.166239702 -
0.101723941 -0.077754558 -0.168168478 0.052923499 -0.206347959
-0.013910275 -0.048987414 -0.279823736 -0.106803121 -
0.37018031 0.344572407 0.432090157 0.064078415 0.284231231
0.397738335 0.428136721 0.022981626 -0.032477219 -
0.269651045 -0.254301379 -0.136109393 -0.386544627 -0.098978072
-0.204534536 0.079803489 0.235948711 0.130811748 -
0.025885608 -0.064536172 -0.135453636 -0.327038196 -0.184707613
0.252234101 0.260355102 0.092827271 0.462239217
0.220997756 0.228810586 -0.297274243 -0.182011146
0.05600533 0.054582683 0.180960268 0.187152819 -
0.500972178 -0.163828693 -0.178273864 -0.003351269 -0.199298881
0.101598578 0.159060168 -0.271402953 0.028470404
0.199142199 0.171336597 -0.430542111 -0.333324501

0.473823972 0.035497133 -0.085654342 0.214396769 -
0.244991865 0.044542333 -0.124590676 0.289523021 0.215103042
-0.107826191 0.038738924 -0.217643616 -0.053667313 -
0.264433202 -0.022349146 -0.242138365 -0.404164112 0.22181944
-0.082819724 -0.108415426 -0.191157359 0.19400368 -
0.139142833 -0.116099586 0.342144478 -0.241408514 0.199097437
0.08810856 -0.166530379 0.381154421 0.27881498
0.031312592 -0.368833837 -0.015789379 0.057363906
0.376293703 -0.255198031 -0.175508462 -0.071331763
0.181340642 -0.091418728 -0.361430113 0.262494038 -
0.087134732 -0.080195466 0.153663247 0.296034703 -0.016975769
0.166311771 0.205173597 -0.274431537 0.105967631
0.077959989 0.120652671 0.409325524 -0.072891998
0.057810682 -0.066738122 0.160425955 -0.060836072 -
0.074602715 -0.212630477 -0.162940505 -0.245919715 -0.543302498
-0.154695592 -0.22870024 -0.085420648 0.380155777
0.235863763 -0.213361786 0.173201582 -0.087604554
0.075744442 -0.134290464 0.327819493 0.040203862 -
0.093687355 -0.372885991 -0.52670264 -0.095367889 -0.174483164
0.163517527 -0.258929122 0.155804924 0.341854639 -
0.035427986 -0.214723384 -0.258901338 0.20106672 -0.138983253
0.431188947 0.011810159 0.17050173 0.010597695

0.318472338 0.094374804 0.235536552 -0.022811789
0.188108621 -0.039506862 -0.02201702 -0.131669512 -
0.238404541 0.029216583 0.068478107 0.091068219 -0.269174087
-0.166300284 0.4400646 0.390951119 -0.018282897 -0.133559455
0.169952735 0.063664003 0.101928577 -0.359005907
0.133522795 -0.312398615 0.098839139 0.307253662
0.229804158 0.204085224 0.059997387 0.033579818
0.049793523 0.28230584 0.296673341 -0.1344172 0.351055835
-0.228895726 0.470473647 -0.172878506 0.115957257 -
0.196741475 -0.063149948 -0.272991055 0.103872238 -0.042322028
0.237527698 0.215229228 -0.078988303 0.150406738
0.081929816 0.211725641 -0.036323033 0.407380578 -
0.027903907 -0.343251134 -0.137985781 0.247324572 -0.510345981
0.323575284 -0.007308473 -0.108609073 0.064636953 -
0.292383402 0.344011755 0.072056841 -0.313813326 0.205498497
0.285004369 0.076264634 0.079926437 -0.148086869 -
0.032689796 -0.175463872 0.081240356 -0.154846288 -0.325654469
0.148495324 -0.315273291 -0.214764049 -0.163831553
0.161392006 0.128423914 0.080245274 0.189348575
0.129606264 -0.265224115 -0.017250964 -0.252327692 -
0.320648829 -0.277555755 -0.253533984 -0.020742575 0.204463609
0.182162895 -0.033568681 -0.218589656 0.068049802

0.037857162 0.077427444 -0.157427439 -0.007539444
0.304702983 -0.330228737 0.121200748 0.297552176 -
0.414292099 -0.283074015 -0.272037523 0.009733387 -0.308591563
-0.040786514 -0.345564691 0.161897176 -0.43312484 -
0.0775981 -0.019634631 -0.22877225 -0.034528424 -0.074844112
0.100591451 0.213239332 0.231076975 -0.25961683 -
0.011381766 -0.045451057 0.320985009 0.193166072 -0.265346639
0.171921979 -0.084496966 0.089745459 0.201156733
0.061858056 0.042148338 -0.088867903 0.113525432
0.238801514 -0.09625164 0.127704159 0.23856256 -
0.355314863 0.194174117 -0.349708586 0.263649838 0.324344828
-0.221685309 -0.452714603 0.151883472 0.206520045 -
0.501215964 -0.278820484 0.071002007 -0.038090395 -0.113892518
0.305986873 -0.086111997 0.07258528 0.030631051 -
0.123498024 -0.009897049 -0.067658582 0.306522705 -0.236425768
0.065158977 -0.115324434 0.209816837 0.283190313
0.124017525 -0.269411288 0.32849434 -0.113074772 -
0.151961255 -0.027064122 -0.184025965 -0.072378061 0.120999961
0.012577855 0.14654356 -0.080936764 -0.038960095
0.009865182 0.052421618 -0.026675159 0.149838962 -
0.4504795 -0.175071941 0.009150921 0.453676975 -0.239510907
0.282297565 -0.283458823 -0.234619455 0.178710918

0.379862197 -0.229346416 0.477672325 -0.170023087 -
0.544678539 0.201831749 -0.357643315 -0.312728074 0.165177777
-0.152882009 0.070671208 -0.302369974 -0.232314408
0.066654439 0.144928464 -0.050170384 -0.233516221
0.067915932 0.246806615 -0.254970085 -0.237115666
0.122219685 0.092058975 0.032421235 0.060850102 -
0.049375101 0.197752682 0.272892017 -0.448029426 0.133527925
0.114405541 0.188623319 -0.120883007 -0.077955766 -
0.229836005 -0.142166708 -0.005855303 0.044350792 0.003797081
0.321312216 0.085501421 0.355480008 -0.149834749 -
0.21645922 0.220066215 0.169497072 0.229496324 0.309020537
0.368034275 -0.065958289 0.264189607 0.200147038 -
0.264904367 0.485819087 -0.23610619 -0.021916002 -0.137651243
-0.241804398 0.095132565 -0.412909068 -0.190684162 -
0.341098742 0.164189912 0.097938057 -0.06569933 -0.532442032
-0.074966765 0.360416035 -0.516395817 -0.093899286
0.043035099 0.036577796 0.004083605 0.212921243
0.229165156 0.265797978 -0.150224575 -0.016332184
0.46759067 -0.397182689 0.087079449 0.110687189 -
0.243087796 -0.014184628 -0.16712343 0.152768424 0.03693712
0.229914266 0.056447374 -0.017480624 -0.118906605 -
0.152925441 0.397060949 0.431477386 0.16718546 -0.158175023

-0.296953867 0.004933462 -0.390287437 0.136087538 -
0.003149384 -0.191858776 -0.331062546 -0.001062043 -0.496229923
-0.272716214 0.277490505 -0.083775036 0.200781142
0.167963803 0.031579266 0.272335565 0.138508025
0.10684082 -0.228371026 0.055213346 0.076172829
0.035287305 0.257893264 -0.018817577 -0.233421902
0.060536093 -0.023548102 -0.012274904 0.380310775 -
0.175319372 0.371393862 0.208592602 -0.15492071 0.128501347
-0.034100606 0.307562735 -0.013317116 -0.048407193 -
0.271159115 0.053637361 0.364026503 0.213080256 -0.034813678
-0.130650399 0.168974138 0.158949244 0.000389481 -
0.048484881 -0.03169586 -0.424557579 -0.345210466 -0.256262056
-0.458756201 -0.143856569 -0.041795164 -0.126252689
0.22303651 0.184564883 0.20326716 0.204452005 -
0.09040234 0.134064762 0.34968272 0.454863166 -0.014782083
0.289187719 0.02776014 -0.375499158 -0.205262054 -
0.176046568 -0.494099704 -0.474288648 -0.246950175 -0.383554197
-0.09090303 -0.037371803 -0.133641123 -0.056989999 -
0.097117893 0.256635214 0.499714103 0.034550086 0.160321821
-0.020088747 0.059528184 0.266357193 -0.208868111 -
0.264008912 0.173486101 -0.049582382 0.178199912 -0.070609714
-0.221844075 -0.230435764 -0.007006028 -0.118882115

0.10294067 0.344851784 -0.186067964 -0.077758513 -
0.053987238 0.298866808 0.178458934 0.023757797 0.142042045
0.41924861 0.034004552 -0.011096473 -0.082144379 -
0.09188141 -0.174840774 -0.113427883 -0.180779747 0.221828952
KEGG_SULFUR_METABOLISM -0.253096163 -0.213582257 -
0.451006315 -0.44205701 0.392291367 -0.306202728 -0.011237833
0.202622332 0.07271538 0.543182219 -0.219997807 -
0.264076582 -0.067836359 -0.373393128 0.296754173 0.508689883
-0.475874431 0.072999421 -0.308249672 -0.290516231 -
0.350403122 -0.431915596 -0.266183142 -0.499319315 0.305773781
-0.173891742 -0.092769362 0.251688456 -0.351980817
0.280507644 0.137010471 0.391256454 -0.105928763
0.269981215 0.008456885 0.024795364 0.27144045
0.431290854 -0.036942674 -0.002166481 -0.334927862
0.288682659 -0.303747048 -0.164535319 -0.358208211
0.074420609 -0.101010157 -0.458773444 0.100809752
0.190612283 -0.17896568 -0.437681375 -0.307006554 -
0.372475122 0.318957922 -0.067811417 0.29441296 0.508696839
-0.262163634 -0.46469521 -0.065677981 0.115176511 -
0.398488606 -0.000112616 -0.231525881 -0.152368791 0.065763842
0.133254135 0.220456978 0.076755265 -0.220955887 -
0.179469563 -0.305583499 0.174385049 -0.205901166 0.500481141

14992 0.229223474 -0.431008758 0.388672129 0.189997736

14993 0.126996193 -0.38003577 -0.384272548 0.492067769

14994 0.244010392 -0.159570011 -0.332283598 -0.313567349

14995 0.299191246 -0.299718721 0.467414459 -0.083635568 -

14996 0.299905885 -0.02120598 -0.225353803 -0.549725801 -0.217589349

14997 -0.180349856 -0.190961241 0.390804875 0.096245493 -

14998 0.438640351 -0.230153865 0.28437489 0.384666768 0.045467183

14999 -0.332140106 0.516050315 -0.06980722 -0.329493114 -

15000 0.011123847 0.089323661 -0.175452166 -0.28610473 -0.212012332

15001 -0.333210994 -0.017704353 0.176038912 0.078553275

15002 0.044742464 0.006799969 -0.022637022 0.085722036 -

15003 0.199892766 -0.118197095 0.150181927 -0.320070345 -0.063945045

15004 -0.301491513 -0.044155573 0.140623993 -0.066864502

15005 0.245484951 0.272516323 0.182351677 -0.352395636 -

15006 0.129596528 -0.299121263 0.303236204 -0.396098565 0.246524972

15007 0.047910293 -0.085803145 0.023963831 -0.298357338 -

15008 0.342847215 -0.096329367 0.016040167 0.27191896 0.362448668

15009 0.227075958 -0.134897296 -0.158182979 0.160617345 -

15010 0.364305449 0.055218771 -0.254241511 0.345046668 -0.469763062

15011 0.058651271 -0.428282253 0.627672678 -0.215935594 -

15012 0.151491219 0.42563751 -0.287637078 0.369218 0.069864405

15013 0.346492097 -0.181798197 -0.469456542 0.092580471 -

15014 0.097227454 -0.004840716 -0.140345154 -0.28538335 -0.483649174

15015 0.135999368 -0.259055255 -0.138363676 0.49863919 -

15016 0.133115282 -0.061320734 0.081791919 0.135775434 -0.107003706

15017 -0.480039691 -0.301971683 0.466120224 -0.521500916 -

15018 0.274117013 0.32309189 -0.320965403 0.102342346 -0.429905293

15019 0.399758959 0.086395262 -0.138297343 0.074955726 -

15020 0.1843732 0.359807116 0.081407042 -0.441798304 0.11685089

15021 0.437929545 -0.170164235 0.285681945 -0.185682214 -

15022 0.215470512 0.549849864 -0.0221848 -0.537648823 -0.152902387

15023 0.122297722 0.581564913 -0.343968805 0.043415324

15024 0.143617002 0.083648062 -0.497485082 -0.030249549

15025 0.056886447 0.039218087 -0.275625937 0.112372992 -

15026 0.344780104 0.382718867 0.381172957 -0.419547603 0.100060081

15027 0.316655091 0.015646146 -0.330370341 -0.167480591 -

15028 0.315694006 -0.480648577 0.133063425 -0.633089043 0.230057959

15029 0.281565655 0.348205824 -0.344105368 -0.440645556 -

15030 0.065567905 0.379631505 -0.36819028 0.368548491 0.106237587

15031 0.250698722 0.008101071 -0.205519266 -0.092076352

15032 0.332623578 -0.193998607 0.347266452 -0.116491305 -

15033 0.008526053 0.193563403 0.05645072 -0.083819947 -0.200482952

15034 0.146074054 -0.404503185 0.405983612 0.184945375 -

15035 0.278900118 -0.441909246 0.529813859 -0.509202013 0.427627031

15036 -0.028679523 -0.304156032 0.033739438 -0.150763601

15037 0.05727397 0.33059508 0.059904496 0.088097949 -0.170209

15038 0.284233719 0.127105785 0.494837593 -0.082127792

15039 0.248333533 0.192612006 0.183362527 -0.001715615 -

15040 0.327511535 0.030022412 0.334949983 -0.32595052 0.371962134

15041 -0.389564095 -0.202537831 0.300862116 -0.050986285 -

15042 0.274991736 -0.576498141 0.096736575 0.015569282 -0.325484192

15043 0.073942451 -0.02214269 0.390120373 0.295551979

15044 0.254912284 0.169983256 -0.174137706 0.247926371

15045 0.182785941 0.061045025 0.12037004 0.13551107-0.288000585

15046 0.515952814 0.443342806 0.397760265 -0.322123886

15047 0.219406727 0.138118207 -0.16436393 0.039129727 -

15048 0.135910705 0.518922347 0.347463522 0.20979815 0.141999608

15049 0.377363326 -0.009089553 0.453211790.230381538 -0.160183618

15050 -0.434746696 -0.060241478 -0.313889614 0.195552759 -

15051 0.167314182 -0.005243813 -0.225656197 -0.026258698 0.057990405

15052 -0.359475978 -0.341363956 -0.515926088 -0.086752304

15053 0.258734407 -0.214746895 0.281336747 -0.115111397 -

15054 0.116550703 0.248993776 -0.206410886 0.208600225 -0.158677193

15055 0.02647704 0.253512053 0.254033899 0.079998686 -

15056 0.140726333 -0.115529175 0.356664301 -0.20896448 0.168844575

15057 0.254466227 -0.252844149 0.116376278 0.376210944 -

0.384347501 -0.144092022 -0.131466426 0.495502175 -0.374311157

-0.066836372 -0.359558796 -0.532559511 0.371223056 -

0.17901936 0.188767062 -0.193051843 0.001488338 0.165382363

0.638432879 -0.166766664 0.157961874 0.160754289 -

0.306725907 0.104375208 0.051584902 0.027576855 0.157339543

0.081936047 0.36331682 0.043665026 0.308217474 -

0.179644631 -0.283471681 -0.201326699 0.132395115 -0.037996686

0.070922255 -0.410353137 -0.075274028 -0.354401361

0.01046589 0.317351336 -0.296460034 -0.252258876 -

0.49540304 -0.037604808 -0.460096102 -0.193047186 -0.570645893

0.377473008 0.19243844 0.300168771 -0.040478781 -

0.167883952 0.407229484 0.091152054 0.284503124 -0.123844554

0.069934622 0.314940715 -0.277888619 0.484929548

0.192727378 0.430621078 -0.320722034 -0.103997059

0.367196946 0.531969961 0.39450441 -0.40158411

0.191975801 -0.350661522 -0.685324608 -0.296344733 -

0.422593704 -0.036940409 -0.302612528 0.116092732 -0.467701716

0.092387331 0.534522086 0.343019059 0.115386587 -

0.215811916 0.022074602 -0.347394619 0.342515189 0.226028481

-0.016234945 -0.291615879 0.367187489 0.390627254 -

0.324924269 -0.184973319 -0.095540359 0.114566781 -0.025307874

-0.155769246 -0.229024057 0.236009995 0.125888474 -

0.224993307 0.399080763 0.284025631 0.321123742 -0.628407727
-0.176781604 -0.437484162 0.543818822 0.031379764
0.126276883 -0.13917213 0.08863909 -0.062974075
0.062915533 -0.295947943 0.006794899 -0.239457298
0.350729035 -0.300547752 0.147752173 0.053894218 -
0.207528075 0.094379717 0.448186712 0.583289584 -0.369266263
0.067103045 -0.360225866 0.046469904 0.285761172 -
0.034185673 0.09413549 0.705379524 0.346058399 0.258686509
0.030293646 0.317334965 -0.134396868 0.246641578 -
0.230495578 -0.167288934 0.215254123 0.561461092 0.104572357
0.480523118 -0.433951768 0.179797325 -0.352184096 -
0.694880306 0.464393651 -0.393404598 0.286578454 0.055967912
-0.139730183 -0.086154308 -0.104641883 -0.10268967 -
0.186211068 -0.372261119 0.438774395 -0.009382819 -0.256494373
0.135661668 0.599177251 -0.293142657 0.303694985
0.019475031 0.223959151 0.295273698 -0.282393218 -
0.237642563 0.11490672-0.034879205 -0.319558634 0.038166283 -
0.33120394 0.511450582 0.068659 0.220606853 -0.088504639
0.011693313 0.679609975 -0.297388666 0.143515448 -
0.032165724 -0.301751073 0.218013962 -0.131364523 0.434177444
-0.019214605 0.339385585 0.455022714 0.036474994
0.29935568 0.154570845 0.194598213 0.295354286 -

0.118878206 -0.540362219 -0.477367279 0.215606487 -0.078370978
-0.480974599 0.319435182 -0.08136818
KEGG_METABOLISM_OF_XENOBIOTICS_BY_CYTOCHROME_P450
0.201303807 -0.011497696 -0.184510931 -0.299358168
0.511424796 0.330921272 0.107151567 0.362939504 -
0.437926254 0.151116665 0.12070573 -0.145795356 0.122758832
-0.242359613 0.299502539 0.377822151 -0.450494019 -
0.146382499 -0.11490593 -0.15384402 -0.507062567 0.302033995
-0.475615473 -0.577506486 0.217625203 0.36827166
0.423734534 0.128179873 -0.191955376 0.213487422
0.235327602 -0.17791146 0.064856341 0.345501323 -
0.239418703 -0.289458568 0.334232521 0.353664259 -0.067254587
-0.123930934 0.068772441 0.041820963 -0.361596103
0.361411062 -0.351148203 -0.262662865 -0.220940039
0.014370897 0.062010924 0.291503537 -0.070290754 -
0.193553655 -0.096006064 -0.355677 -0.161928689 0.066426365
0.335203954 0.045984324 0.086437941 -0.431255764 -
0.529862747 -0.210772414 0.039613065 -0.328973268 -0.050085883
-0.282159859 -0.157466973 0.033803237 0.407025656
0.431885274 -0.478306387 -0.371745322 0.082449774
0.151073648 -0.043734491 0.465958864 0.186848656 -
0.203049731 0.194359029 0.40588516 0.173726154 -0.48192862

15124 0.103560686 0.34793234 -0.063529077 -0.433137416 -

15125 0.549951045 -0.241998615 -0.0658151 0.243106357 0.087484019

15126 0.362888638 0.269096837 0.061766783 -0.240105037 -

15127 0.172770328 -0.050646083 -0.201720905 0.417991238 0.319791111

15128 0.033832709 0.373498136 0.060552643 0.328087766 -

15129 0.040657333 -0.344747346 0.062329552 0.489779504 -0.462246398

15130 0.423153511 0.559620023 0.437993749 0.267775397 -

15131 0.435897117 0.15769091 -0.30867617 0.370187547 0.425927894

15132 0.412383683 -0.150548819 0.519192944 0.398237527 -

15133 0.09302485 0.28905383 -0.017019638 0.566828654 0.277818224

15134 -0.491515368 -0.202384062 -0.291909006 0.084088667 -

15135 0.035115755 -0.085905733 -0.139712006 -0.059823543 -0.535060699

15136 -0.481730933 -0.146851048 -0.224259972 -0.454835647

15137 0.391938095 0.378117695 -0.329627031 0.144303399 -

15138 0.438251928 -0.230272488 -0.078737748 0.313936635 0.412707806

15139 0.322478767 0.326309396 -0.439473658 -0.452999933

15140 0.314022364 -0.263132309 0.455470303 -0.40787432

15141 0.421039727 -0.369954066 0.006759971 -0.225899136

15142 0.270647569 0.059060856 0.027177318 0.352734092 -

15143 0.281435077 0.416482093 -0.14092427 0.483342327 -0.104866608

15144 -0.406321862 0.397231347 0.430329413 0.265469 -0.421022839

15145 -0.402949428 -0.437337319 0.224604364 -0.320697755 -

0.158257526 0.254479018 -0.001974566 0.143323045 -0.192913939
-0.457620491 0.066870427 -0.122966134 -0.002086702 -
0.166906567 0.079176743 -0.176495039 0.570081795 0.267735049
0.034095786 0.083513983 0.4311316 0.067442325 -0.105610166
0.34029131 -0.266900398 0.005141831 0.062802765
0.013292344 0.649590145 -0.051145126 0.011339523 -
0.133292264 0.172372992 -0.287926955 0.143753627 0.178599428
-0.263870836 -0.128920785 -0.174243416 0.61111219 -0.338171646
0.052453285 -0.340012738 0.23090544 -0.372191105 -
0.065019128 0.068806402 0.463241534 -0.519501195 -0.116407904
0.313554469 0.029709876 0.437575516 -0.335966296
0.42842288 0.587970993 -0.160617206 0.038667326 -
0.251265423 -0.011493217 -0.010276542 -0.13065636 -0.365750637
-0.284599186 0.121168871 -0.124938155 -0.457883082 -
0.109089763 -0.042069315 -0.223514693 -0.249999388 0.188917498
0.165270641 0.310529838 0.370128076 -0.185424116 -
0.214765141 0.211730724 -0.542062762 0.303737101 -0.424417859
0.257409664 0.466004689 -0.252221038 0.052231645 -
0.403328931 0.160174291 0.245442337 0.392514548 0.179312163
-0.312559612 -0.05019033 0.404454283 0.232227134
0.524292632 0.414418477 -0.504063275 0.061844809
0.230150857 0.243998896 0.054672952 0.053337989

0.316088626 -0.037846985 -0.115504647 0.083613438

0.213792956 -0.370589118 0.498181778 0.179669244

0.53429383 0.224987758 -0.181006281 -0.322970049

0.458309288 -0.069301714 0.411876695 -0.165267383 -

0.269742548 0.112903842 0.206073458 -0.163004334 -0.034834039

-0.200349744 0.261491593 -0.328607249 0.237188929

0.384645258 0.28698765 0.535179697 -0.282656897 -

0.402455265 -0.00741208 -0.250366356 0.461555504 -0.10953118

0.229750998 0.019009109 -0.325420184 0.309788907 -

0.09870087 0.292548666 -0.028928043 -0.408057248 -0.310840721

0.32950022 -0.248302284 0.083532325 0.492988522 -

0.223644839 0.237226256 -0.133655183 0.533073966 0.114151188

0.237436847 -0.220320048 0.139045472 -0.314301257

0.33074609 -0.532214367 -0.118756213 -0.219606594 -

0.429180233 -0.466104254 -0.342195647 0.178065874 -0.227639195

0.180240863 -0.272147226 0.234319741 0.393558888 -

0.532671773 -0.136869276 0.215917737 -0.252818492 -0.41372078

-0.179456869 -0.548256102 0.510579875 0.10305986

0.363818471 0.274887385 0.269540964 0.23153153

0.488447873 0.266242538 -0.128760546 0.108885571 -

0.319768927 -0.070390391 -0.07095149 0.163666434 -0.409941516

-0.057626915 -0.41492395 -0.232529427 -0.654241126 -

0.267351646 0.122806411 -0.062451599 0.142044128 -0.182064236
0.331165452 -0.45378409 -0.308693835 0.03731323
0.243951939 0.024995066 0.104917182 -0.237004748
0.089610626 -0.077925824 -0.385478038 -0.339907637 -
0.170345957 0.392124108 0.191918238 -0.032687742 0.112603925
-0.174946446 -0.152584355 0.302679363 -0.200137849 -
0.27357873 -0.167846182 -0.134709206 -0.2670185 -0.37753873
0.333450994 0.130175897 0.195999197 -0.077913802 -
0.044443722 -0.10601948 -0.244095646 0.334072278 -0.4611506
0.597544278 -0.101113737 0.327363234 -0.175512138 -
0.03615237 0.392032615 -0.169541584 -0.309948751 -0.331463208
0.391585472 0.096872245 -0.293041634 0.180743668
0.168857412 0.611832418 -0.4127912 -0.362612947 -0.174859491
0.308727778 -0.180777841 -0.236397727 0.491897401
0.208232911 -0.56367874 -0.238575201 -0.196965004 -
0.366676765 -0.144616421 -0.368351719 -0.486736051 0.194989553
0.436895169 -0.189168221 -0.17029847 0.197722619 -
0.062750387 -0.101390446 0.120762069 -0.48182536 0.219773075
0.096735047 0.525970583 0.453306229 -0.435455048 -
0.343443678 -0.149668158 0.096404006 -0.195399258 0.321521564
0.00571636 0.274537563 -0.000669246 -0.214018154
0.283163434 -0.37575847 0.252352643 -0.305277358 -

0.049351811 0.10769186 0.248295465 0.124242938 -0.450588649
-0.082622443 -0.329765381 0.376010935 0.402986087 -
0.048308966 -0.29984972 -0.43549703 0.170883226 -0.034984521
0.406192566 0.50622521 -0.018099161 -0.06153343
0.463962772 -0.113002059 -0.4924171 0.331989845 0.34760541
-0.028803639 -0.340414704 0.027704648 0.322080783
0.512862454 0.264244978 0.363062162 0.364197217 -
0.304053122 0.029914008 0.234186163 -0.160872476 -0.476488115
-0.107900823 0.158308687 -0.196640664 0.538137203 -
0.282708997 0.146777779 0.261155146 -0.340709247 0.335760211
0.034616002 0.363670631 -0.041910317 -0.187072157 -
0.150768639 0.361859986 0.320368226 -0.002214523 -0.458655042
0.541235511 -0.34320658 -0.384075695 -0.021216175
0.16023721 -0.340687707 -0.399545468 0.390460525
0.216976104 -0.341241791 0.005874074 0.247382897
0.358481734 0.278871788 0.261135538 -0.076838898 -
0.208504289 0.410704017 -0.404053161 -0.046334244 0.240587445
-0.116183468 0.156049381 -0.237808104 -0.244358265
0.257963682 0.424401150.232557308 -0.395317096 0.094065317
-0.195288 -0.109925988 0.098419521 0.017099088 -0.14561993
0.174907171 -0.203162523 -0.066026959 0.321064191
0.089083114 0.198479496 -0.001492681 -0.207022907

0.094534676 0.116140123 0.276136076
KEGG_DRUG_METABOLISM_CYTOCHROME_P450 0.250534119
0.007884238 -0.15208625 -0.285597809 0.486958286
0.345691190.197745765 0.271301314 -0.474218239 -0.02358132
0.184951324 -0.173478371 0.112747989 -0.194639463
0.312843958 0.276149784 -0.410600383 -0.168515366 -
0.145406686 0.00406022 -0.500510833 0.247306195 -0.420514516
-0.493073824 0.217919363 0.36291477 0.404451931
0.112133066 -0.164543762 0.251113515 0.36452814 -
0.197109225 0.110732449 0.424021747 -0.269592859 -0.269444913
0.336422562 0.283062722 -0.059124979 -0.038339433
0.160445723 0.00536305 -0.355655737 0.337631752 -
0.406845084 -0.181505132 -0.205667702 0.073082634 0.208219665
0.228786308 -0.028124397 -0.221024236 -0.188872308 -
0.307456661 -0.102179391 0.032430538 0.292016519 0.051746394
0.081176226 -0.351057531 -0.515712259 -0.287972933 -
0.006405289 -0.336104718 -0.052029442 -0.139549197 -0.173332676
0.090073085 0.406126054 0.49070417 -0.539147151 -
0.380320932 0.050672658 0.273232317 0.082212448 0.429733531
0.169596475 -0.141453376 0.2120313 0.435278884 0.203860257
-0.407918993 0.029983493 0.367092133 -0.18768407 -
0.468650635 -0.557534083 -0.187059958 -0.039346202 0.273015653

-0.205437572 0.411250089 0.287546672 0.057957389 -
0.223626446 -0.090974221 0.062764396 -0.152393625 0.386436996
0.284467025 -0.065526204 0.370367329 0.083263824
0.266142981 -0.01469026 -0.171433814 0.142209815
0.42828828 -0.444727544 0.376403014 0.530931143
0.442877496 0.264339702 -0.476749881 0.259774277 -
0.281304329 0.245460365 0.448275276 0.425364676 -0.168844499
0.518315539 0.416654228 -0.248132799 0.307444388
0.00749407 0.530024984 0.252289424 -0.420231798 -
0.098292593 -0.272888977 -0.063503572 0.037740668 -0.050948616
-0.122894561 -0.09627815 -0.521354486 -0.449286232 -
0.118344879 -0.128555746 -0.452413998 0.421705233 0.362502934
-0.222415861 0.171684751 -0.360026849 -0.27134134 -
0.091521327 0.296316622 0.467497294 0.258204037 0.306024963
-0.466549235 -0.442696766 0.133322139 -0.262118389
0.477313454 -0.371290286 0.365341012 -0.267554866 -
0.147957354 -0.179905496 0.185178702 -0.105905796 0.091583577
0.29017198 -0.25530436 0.307043386 -0.205889071
0.481901717 -0.05867358 -0.321128758 0.408824725
0.415299542 0.191620639 -0.469365235 -0.422352634 -
0.389702322 0.290000875 -0.333808405 -0.309056946 0.178403226
0.080318081 0.107963294 -0.199422309 -0.431116456

0.131518719 -0.104093083 0.106244063 -0.006530907
0.031333331 -0.274929288 0.500792842 0.21431045
0.135214609 0.038747144 0.445957599 0.136458395 -
0.1114837 0.335029471 -0.283896967 -0.030986992 0.155636833
0.024582734 0.653034474 -0.091615512 0.014568596 -
0.058311427 0.25951434 -0.311679699 0.014589596 0.091205622
-0.150672772 -0.031497704 -0.181166962 0.575839396 -
0.346189128 0.141646461 -0.275027422 0.357756728 -0.350750463
-0.127989823 0.056097303 0.437205086 -0.522668715 -
0.068548554 0.320096649 0.032125088 0.420249661 -0.306163226
0.428095791 0.526360546 -0.212329593 0.126831625 -
0.08374595 0.125533452 0.016043872 -0.188059377 -0.266647082
-0.134337398 0.068863129 -0.254180508 -0.390912202
0.005014925 -0.212811383 -0.375755218 -0.231165351
0.236975824 0.069855669 0.376058748 0.36935238 -
0.121895167 -0.145129743 0.202378559 -0.542119014 0.261455177
-0.368164731 0.236519774 0.436578534 -0.09980896
0.148261068 -0.384558871 0.122692122 0.221158590.395273344
0.09443037 -0.314967564 -0.166761483 0.376803596
0.218777399 0.518053498 0.400622451 -0.519258599 -
0.123168644 0.219021026 0.224334912 0.059562236 0.047151258
0.295549113 0.037915203 -0.246521969 0.101686786

15300 0.192631459 -0.37319765 0.482283985 0.142773321

15301 0.485037284 0.242925158 -0.14911914 -0.248596511

15302 0.46744914 0.05101381 0.328392358 -0.032944978 -

15303 0.331351826 0.181525092 0.222334123 -0.006399415 0.048222703

15304 -0.298257947 0.308785836 -0.261808308 0.266970128

15305 0.347418747 0.218797721 0.521186317 -0.088834841 -

15306 0.41604532 -0.024202898 -0.293463996 0.47913241 0.051700036

15307 0.309427765 0.048355293 -0.291841326 0.204895812 -

15308 0.119046143 0.291793919 0.031384621 -0.414650744 -0.240590595

15309 0.333212479 -0.24298508 -0.012688185 0.500304543 -

15310 0.271586784 0.167909725 -0.230606066 0.51377536 0.186106095

15311 0.293228808 -0.227949442 0.135740504 -0.289058853

15312 0.33776388 -0.547609479 -0.162260762 -0.120261779 -

15313 0.440989601 -0.461614389 -0.294838818 0.055325532 -0.186520218

15314 0.167361268 -0.263843816 0.142370881 0.243350234 -

15315 0.439823627 -0.026029451 0.295480211 -0.264409066 -0.376515777

15316 -0.167594259 -0.512618593 0.536766631 0.041419657

15317 0.386174935 0.284138311 0.171279944 0.226470988

15318 0.462159416 0.289633228 -0.10243996 0.144672669 -

15319 0.383437095 0.012808439 -0.173338928 -0.030011118 -0.464297056

15320 -0.040499344 -0.389760828 -0.252914408 -0.623973771 -

15321 0.202848516 0.03462421 -0.073003996 0.087249337 -0.071048312

0.333734667 -0.436751213 -0.292539298 0.132437079

0.155655829 0.101307614 0.139924945 -0.354759918

0.189715758 0.018485357 -0.439531246 -0.348616868 -

0.195441391 0.306071434 0.123910359 0.039118710.152339273 -

0.099451025 -0.139775552 0.413715258 -0.226595522 -0.255037679

-0.293241304 0.005969989 -0.181604818 -0.369877978

0.320599419 0.07833425 0.211135027 -0.034467184 -

0.002029313 -0.271522444 -0.219672611 0.314356788 -0.288438696

0.591572663 -0.122028557 0.351624095 -0.03049123 -

0.036447685 0.3506903 -0.210868194 -0.28116113 -0.373128393

0.370436677 0.04875844 -0.323943901 0.281504283

0.124995273 0.641283943 -0.450599612 -0.432009598 -

0.089359364 0.264317936 -0.138414962 -0.15571314 0.478696821

0.204303739 -0.482412481 -0.245968965 -0.169878148 -

0.386080898 -0.136139966 -0.200950407 -0.43653367 0.256153066

0.403412588 -0.198973421 -0.107779348 0.136769126 -

0.086281684 -0.11396729 0.090147704 -0.547103935 0.290428084

0.068699885 0.492108106 0.420974807 -0.420663297 -

0.345326603 -0.147461041 0.069435677 -0.188857674 0.310631743

0.091286019 0.1710091 0.040290788 -0.237062404 0.327307906

-0.162244565 0.243292889 -0.298705428 0.011768261

0.227733485 0.236609513 0.137309413 -0.382427937 -

0.092093955 -0.239664132 0.347298582 0.372522814 0.010550509
-0.196938716 -0.432747261 0.034845828 -0.001799566
0.227453684 0.388955897 0.05846654 -0.065954393
0.398225749 -0.034486097 -0.485830202 0.320217841
0.380929814 -0.023590889 -0.325243149 0.094192764
0.327664905 0.506563095 0.214380648 0.349590838
0.373912632 -0.246864144 0.06769879 0.165628683 -
0.203766701 -0.503451237 -0.187874243 0.085222938 -0.180489516
0.502819344 -0.228701937 0.362623844 0.264052858 -
0.383566646 0.264514161 0.083792938 0.311946149 -0.010001316
-0.156312312 -0.162581915 0.340359903 0.292307312
0.009069129 -0.474255676 0.506937934 -0.268932658 -
0.42969858 -0.097377454 0.103038936 -0.390225306 -0.423916011
0.39869238 0.136294057 -0.301948388 -0.056296922
0.196966882 0.313489677 0.256414424 0.228041424 -
0.149803971 -0.319157362 0.46101291 -0.36772744 -0.012584681
0.256256047 -0.239587161 0.206697239 -0.278956694 -
0.202020357 0.206114151 0.413546785 0.262106799 -0.383287496
0.083250011 -0.163737977 -0.220861302 -0.012233184
0.10620259 -0.107847878 0.091391031 -0.268666043 -
0.089290858 0.339127604 0.122116543 0.050600891 0.049683202
-0.218390988 0.064624632 0.094341825 0.217644968

KEGG_DRUG_METABOLISM_OTHER_ENZYMES -0.004180788 0.040841996 -0.251050128 -0.479399767 0.335043425 - 0.14741122-0.188329037 0.028994967 -0.203651591 0.109294204 0.054933191 -0.045529999 -0.084747462 -0.259580356 0.108342305 0.223904536 -0.129049686 0.001660166 0.006250911 0.1344266 -0.200108787 0.454335444 -0.304136978 -0.033304442 0.046725502 0.242526738 0.290239112 0.172328317 -0.09915297 0.094904807 0.310282162 - 0.173979964 0.045869387 0.219327311 -0.1270909 -0.185426349 0.061636125 0.345175792 -0.192923531 -0.064841736 0.026901093 0.139649315 -0.368944333 0.157927811 - 0.266966314 0.173655658 -0.176694108 -0.016492446 0.286379228 0.178417452 0.132142586 -0.390361646 -0.076349078 - 0.378003253 -0.145167652 0.207804976 -0.002502294 0.231486522 0.145069105 -0.341622111 -0.203284708 -0.354608964 0.128782126 -0.248658744 0.220949283 -0.219476484 - 0.114449775 -0.007949175 0.300911169 0.188853966 -0.264889409 -0.185913083 -0.015314059 0.42935541 0.058627565 0.299487222 0.187433059 0.241136895 0.337256624 0.346944018 -0.090284106 -0.302938671 0.149941496 0.168400989 -0.034204477 -0.200697419 -0.484094009 - 0.074585248 0.047172475 0.084313666 0.020482678 0.162146473

15388 0.07245714 -0.051330861 -0.152695256 -0.263176746

15389 0.011450993 -0.219065675 0.323359883 0.214590802

15390 0.076960999 0.014209811 -0.12544119 0.278007357 -

15391 0.056825303 -0.060455928 0.208155599 0.279809179 -0.411354189

15392 0.011699187 0.097991395 0.207528903 0.39538422 -

15393 0.385060703 0.049543113 -0.055358468 0.191395596 0.261384053

15394 0.145003433 -0.207919551 0.138312968 0.239973805

15395 0.143670848 0.20276635 -0.072921233 0.242581348

15396 0.226624665 -0.186706248 0.088358362 -0.158656771

15397 0.022630725 0.028256288 -0.098697491 0.120470868

15398 0.19649927 -0.396317631 -0.200285005 0.18246348

15399 0.00084465 -0.38931205 0.206069901 0.234000825 -

15400 0.227563428 0.067449364 -0.303127139 -0.42001975 0.028868268

15401 0.211451301 0.330089241 0.370145289 0.23606459 -

15402 0.203005082 -0.321130201 0.153150039 -0.316225676 0.209483099

15403 -0.230051172 0.243866283 -0.033736618 -0.219696429 -

15404 0.237974694 0.44787743 -0.111648103 -0.256668382 0.283235149

15405 -0.081329619 0.280818139 -0.154152827 0.365627784 -

15406 0.022411015 -0.24463342 0.284822121 0.103569782 0.225834978

15407 -0.404010882 -0.399717991 -0.295792561 0.109371749 -

15408 0.046250664 -0.177517626 0.079942203 0.083653079 -0.186034505

15409 -0.061741187 -0.299576972 0.086245616 -0.067374194 -

0.046078806 -0.124838614 0.19647127 0.296983874 0.364449463
0.075953806 0.037884066 0.019123377 0.13974429 -
0.046917781 -0.122256742 0.093992487 -0.209048258 -0.013000084
0.154127942 -0.026512443 0.52900301 0.06914014 -
0.025586112 -0.0517187 0.074607243 -0.235743877 -0.16813354
0.053875385 -0.317211489 0.185444405 0.288842055
0.389633797 -0.131214438 0.081847637 -0.039273985
0.094443573 -0.255095757 -0.137298103 -0.047393744
0.069630721 -0.192817101 -0.063168679 -0.198282642
0.34033324 0.320996944 -0.089836237 0.354559662
0.456750346 -0.250879241 -0.054808956 0.008004944
0.112411485 -0.081553036 -0.135047289 -0.16551469 -
0.087676686 0.080564696 -0.283629436 -0.337953308 -0.206666337
-0.135336368 -0.24824761 -0.196823826 0.272384413
0.097619663 0.240410178 0.119518941 -0.269032955 -
0.334395214 -0.088769979 -0.344998778 0.001472702 -0.441228868
0.30347943 0.169812795 0.00847865 -0.173068496 -
0.248066226 -0.035745921 -0.103392115 0.309909452 0.046979135
-0.388208497 -0.022515936 0.461822566 -0.09321679
0.229267078 0.250785383 -0.250296155 0.27186476
0.215264568 -0.087410128 0.125343598 0.144886906
0.133849735 0.038289955 -0.270665737 -0.141220401

0.27138944 -0.14098092 0.207941162 -0.152663341
0.32516772 0.10606602 -0.091492474 0.085754356
0.15943946 0.032565998 0.149651214 -0.174223118 -
0.154078421 0.169110155 0.076244438 -0.069646885 -0.067520461
-0.268175277 -7.75E-05 -0.406166958 0.177319419 0.242542084
0.115137696 0.474263995 -0.03263357 -0.14623406 -
0.078093171 -0.163052725 0.209471135 -0.185466308 0.317623838
0.070202535 -0.052523872 0.132880084 -0.330532543
0.063280233 0.242264078 -0.08695704 -0.326500186
0.213793245 -0.16161957 0.076231676 0.224222961
0.141822554 0.224631739 -0.231239496 0.293988384
0.13934227 0.135912935 0.072948487 0.089450129 -
0.332733763 0.438640152 -0.446497593 -0.136136253 -0.176653254
-0.268991218 -0.326340048 -0.207195364 0.22266587 -
0.191147492 -0.112124031 -0.095135902 -0.027489397 0.223264337
-0.494276819 0.08633909 0.297685878 -0.234527556 -
0.357741203 0.098233656 -0.43837654 0.427571601 -0.13492765
0.118551831 0.124436406 0.104969259 0.182720796
0.320361505 0.109660745 -0.14449157 0.064434529 -
0.202479102 0.054860988 -0.223890369 0.192475158 -0.286275252
-0.192999584 -0.404076654 -0.363417435 -0.558671907 -
0.230974404 -0.080312823 -0.053753513 -0.016223815 -0.001802134

0.086633816 -0.311056016 -0.197372895 -0.03059151
0.078550917 0.163212376 0.060394828 -0.32312668 -
0.069802636 -0.163139048 -0.243098732 -0.359822193 -0.117660638
0.139865592 0.08634822 0.097707266 0.278960271 -
0.038658082 -0.231600668 0.160629052 0.207244325 -0.2847897 -
0.321079448 -0.073053812 -0.115774077 -0.453243233 0.27459932
0.126819404 0.161608988 -0.086885999 0.01928471 -
0.141132579 -0.141638599 0.239453199 -0.279328512 0.486330146
-0.077801074 0.190967874 -0.156590473 -0.047080551
0.231722224 -0.110065863 0.006770617 -0.281188636
0.046640634 -0.084577891 -0.142850029 0.058947822
0.270721905 0.323104068 -0.370913911 -0.169611104
0.146557896 0.18013675 -0.099073412 0.14653038
0.197477482 0.216922094 -0.353696076 0.073489344 -
0.195992435 -0.20556974 -0.16354193 -0.152584882 -0.462425019
0.111016351 0.142566652 -0.215587606 -0.083086495
0.024854528 -0.175308521 -0.021088642 0.244210351 -
0.254226984 0.048238066 0.040687488 0.257085642 0.3630308 -
0.286060821 -0.466858602 -0.306721079 -0.092814589 -0.061138961
0.153940603 -0.068979682 0.180586958 -0.089908971 -
0.172884305 0.186897185 -0.072726365 0.37731161-0.183369818 -
0.038255389 -0.017458332 0.314998738 0.09841673 -0.327213182

0.084215104 -0.073301545 0.252307888 0.396752395
0.306148598 -0.289277618 -0.27002693 0.083806722 -
0.211960608 0.184390307 0.393102967 -0.024200413 0.006571695
0.301452645 0.095597692 -0.413504912 0.217808278
0.023913141 0.110326931 -0.2450258 -0.259959778 0.207211658
0.448116584 0.286510807 0.295715638 0.371345458 -
0.000165943 -0.138296901 0.088648155 -0.15259011 -0.534184329
-0.060917741 0.129409165 -0.085525775 0.420246729 -
0.366606135 0.177155867 -0.011218028 -0.441929798 0.316904385
0.117339587 0.119573091 0.105433882 0.053952792 -
0.005494838 -0.038666845 0.208258049 -0.185686597 -0.371002527
0.429254387 -0.238804549 -0.298693191 0.080898955
0.353452162 -0.327127909 -0.205619678 -0.215781575
0.094627951 0.067908251 -0.096715751 0.248441471
0.26324905 -0.016707442 0.134628311 0.029857177 -
0.284080543 0.373853138 0.025331702 0.031683714 0.23346433
-0.200935148 -0.076296869 -0.027716948 -0.124740671
0.036046043 0.561870629 0.085285635 -0.564564293
0.105722019 0.131793434 -0.327059062 -0.004097568
0.305218942 0.047169773 0.291105009 -0.300009817
0.219520261 -0.122385282 -0.147130826 0.158496736 -
0.053354013 0.106986442 -0.231516493 0.098367648 0.276772682

KEGG_BIOSYNTHESIS_OF_UNSATURATED_FATTY_ACIDS 0.582109127
0.383018881 -0.484913759 -0.46308609 0.256016979
0.10382086 0.39366809 0.000953957 -0.022227945 -
0.377716403 -0.287166638 -0.234412917 -0.440907 -0.388500678 -
0.375858462 -0.210661243 -0.202698553 0.408620877 -0.479782692
-0.050544436 -0.056622399 0.241990774 -0.627964265 -
0.101342775 -0.102657626 -0.14026213 -0.018932067 0.264278254
-0.35053501 -0.387908705 0.216991489 -0.090426708
0.125725627 0.031116269 -0.328131105 0.060939118 -
0.103406992 0.281201304 -0.344995704 0.053792184 -0.144264452
0.364256955 -0.530286528 0.588248832 -0.155225245
0.356194128 -0.156976567 -0.175258632 -0.110182347
0.011389359 0.148707127 -0.381733433 0.397864947 -
0.307304792 0.145242859 0.418763694 -0.199021582 0.06936654
0.417358789 0.27268387 -0.556494993 0.015348551
0.243028466 0.293296954 -0.311711761 -0.333935037
0.181895154 0.229027661 -0.053544266 -0.430146163 -
0.249086948 -0.495678877 0.167027558 -0.189631804 -0.25369507
-0.372754526 -0.266548735 0.215918504 -0.232904218
0.433764607 -0.166686687 -0.035714211 0.356826146 -
0.329560122 0.332579905 -0.19332584 -0.247746934 -0.193615125
-0.23201884 0.297696128 -0.394958721 -0.189108472

0.220556102 0.448797399 0.066153573 0.171819164 -
0.063079319 -0.435574189 0.403828741 0.21537746 0.049807689
-0.446045251 0.295899683 0.231473256 0.001205287
0.035636561 0.007090197 0.074111055 -0.402940284 -
0.247443327 0.175985426 -0.018084512 0.160199681 -0.308157334
-0.032371066 -0.015692688 0.07472953 -0.385461052 -
0.465426462 -0.100860559 -0.324147343 0.485100959 -0.141444026
-0.140795317 -0.016654335 -0.109596708 -0.102520257 -
0.493333612 0.054522134 0.109464108 -0.205239732 -0.587218582
-0.229583813 0.057766747 -0.212877994 -0.296837236 -
0.509399584 0.649440722 -0.318290728 -0.315764041 -0.270505051
0.035236339 0.256511612 0.093361784 -0.486580495 -
0.211932889 0.379354523 0.413512001 -0.451857736 0.267160073
-0.109950518 -0.147085316 -0.419590879 0.011811804
0.036914829 0.322499549 -0.159287812 0.422454453
0.277610022 0.240002172 0.000301928 0.119844874
0.19529517 -0.418960949 0.536964411 -0.153744707
0.232450611 0.061839278 -0.379079801 -0.352312989
0.319483978 0.063019011 -0.069734939 0.257957529 -
0.281560953 -0.092822973 -0.388152409 0.116238217 -0.210823804
0.087212082 0.148750002 -0.136966907 0.211140584
0.512935909 -0.08261252 0.042635057 -0.250541766

0.010514015 -0.608238377 0.317884563 -0.146234118
0.489350207 0.230182221 0.012534819 0.164005399 -
0.015092097 -0.33730982 0.053829792 -0.069152572 0.11663598-
0.122988986 0.356750897 0.099710181 0.653316143 0.37233765
-0.206629304 -0.216060412 -0.035135684 0.08798958
0.365363006 0.497127497 -0.030057302 0.346311879 -
0.440809533 0.158809188 -0.261667031 0.277400621 0.250881547
-0.000195829 -0.432483397 -0.231919346 0.332895733
0.016957154 -0.153262817 -0.411693046 0.209278498
0.091589817 -0.203246579 0.297001932 0.42099797 -
0.197980491 -0.081616359 0.168050751 -0.047417726 -0.116602209
0.3173121 -0.444971667 -0.05416078 -0.098688674 -0.042813959
0.106962956 -0.430074693 -0.006210176 -0.030411994
0.019674324 0.251134080.335308538 -0.13142723 -0.347388053
-0.121554476 0.219747004 0.139596893 -0.45607459 -
0.538185793 -0.380439575 -0.54773523 0.332089975 -0.264909218
0.456325111 0.222767655 -0.482864399 0.261712205
0.101177715 0.275732208 0.354419574 -0.281516505 -
0.287811025 0.370196149 -0.182645864 -0.145136058 0.33055457
-0.107102761 -0.074068057 0.303480488 -0.036740221
0.142736905 0.081733739 0.214586633 -0.095513854 -
0.154775153 -0.481585705 -0.231699707 -0.233301533 -0.226165907

-0.624626405 0.14175778 -0.435457263 -0.3575631 0.0816815
0.202934682 0.253470878 0.184032886 -0.235753994 -
0.254212071 -0.441240318 -0.473753909 0.180762898 0.204710576
-0.445949613 -0.124078767 0.386944427 0.370293141
0.086041596 -0.150933315 0.38578291 -0.155686198 -
0.094079912 0.487469544 -0.261087353 -0.141704243 -0.235398999
0.100204644 -0.39765834 0.1911317 0.471037639 0.372793428
-0.180342433 0.255719395 0.121880489 0.439803262 -
0.052369204 0.466452654 -0.017301321 -0.019288337 0.00651225
0.221123465 -0.213870347 0.089889413 0.075893273 -
0.068262217 -0.245981027 0.176174021 -0.197340554 0.4524693
0.042491499 0.386722085 0.384165703 -0.277987097
0.172933178 0.515682154 0.296779004 0.209461479 -
0.342019138 -0.07096102 0.291926899 0.053978736 -0.090537282
0.572224729 0.075345929 -0.526180717 0.059087161 -
0.123718821 -0.320464947 0.389170091 0.23033934 -0.40688977
0.010624938 0.158198291 -0.079125255 -0.045629018
0.520511898 0.338690623 0.085834619 -0.500868503 -
0.108638489 0.417991079 -0.10319095 -0.18080594 -0.448427523
-0.186237187 -0.107724717 -0.329940509 0.203557169
0.057348175 0.279554051 -0.388641777 -0.308193127
0.097566339 -0.364327405 0.485041046 -0.366378671

0.01064987 0.290166817 -0.520975428 -0.023853634
0.506271197 0.137489728 0.104692826 0.081131301 -
0.159796214 0.069415797 0.252393642 0.163781623 0.528786347
-0.448158449 0.202848103 0.454554471 -0.286943977 -
0.237692526 -0.216205544 -0.18361809 0.298590077 0.063243226
0.412340485 -0.226541732 0.18229079 -0.142374735 -
0.048600852 -0.026087538 0.168047722 0.392850549 0.017409766
0.378227883 -0.432258864 -0.163679096 -0.519980075
0.085259233 0.187116155 0.186849985 -0.033023253 -
0.161593966 0.442251417 0.249502774 0.028468572 -0.368928248
-0.025509703 0.121251475 -0.210712257 -0.20268754
0.071262327 -0.101753515 0.243448425 -0.46703768 -
0.111607593 0.113743582 -0.480501941 0.166302022 -0.139276806
-0.066835264 -0.293522561 0.073089613 -0.403300309
0.231814005 -0.158678909 -0.370418408 -0.14166435
0.032756231 -0.515229932 -0.417840077 -0.043472534 -
0.025251912 -0.305050015 0.02179444 -0.199514749 0.178642979
-0.269946531 -0.410769664 -0.486662439 0.208212169 -
0.001379299 0.601992536 -0.230155535 -0.290895515 0.107835286
-0.273048115 0.273949685 -0.240362058 -0.589885345 -
0.1541301 -0.152775485 0.093911524 0.036606336 0.424637515 -
0.30790796 -0.398630082 -0.185938152 0.283546519 0.42170305

-0.132753366 -0.206156186 -0.119575108 -0.18527982
0.263902141 0.106230152 0.083710456 0.134099518
0.103962837 0.077481628 0.037157518 -0.236960407 -
0.211377428 -0.192212474 0.018858815 -0.208394155 0.419276266
-0.211308601 0.208664396 -0.145111369 -0.087030782
0.421311439 0.275683385 0.181484461 0.355145318 -
0.26182593 -0.274697247 0.228227514 -0.024164048 0.060831506
0.303543535 -0.047359849 0.360474173 -0.412714858 -
0.459097906 -0.215693206 0.294416975 0.412849158 -0.005892371
-0.314941295 0.244706496 0.135819642 0.167114384 -
0.400556259 0.333193071 -0.199850888 0.068652101 -0.147693539
0.507473109 0.14434276 0.301665406 -0.378916916 -
0.007137867 0.056387314 0.154630986 -0.454529485 0.248211571
-0.199803063 -0.229118476 0.370177656 -0.283951399 -
0.484580395 0.070209664 -0.146071258 0.189675586 -0.336051784
-0.339100351 0.231006154 0.207411270.143239544 -0.26236087
0.408254441 0.248863803 -0.114697173 0.224666999 -
0.431591528 -0.068421786 -0.179511518 0.510437147 -0.341966062
0.196734844 -0.313719576 0.439574693 -0.276407457 -
0.067637429 -0.189478395 -0.167894101 0.365345705 0.02379662
0.012824368 0.400065581
KEGG_ABC_TRANSPORTERS -0.168472792 -0.075089488 -

0.34949206 0.070071662 0.319989336 0.152500088 0.076490753

0.127440505 -0.065047817 0.114553670.286715448 -0.243076333

-0.20183369 -0.370856284 0.22684656 0.002925831 -

0.471863725 0.291320361 0.113071188 0.200767122 0.047512369

-0.063156574 -0.028731266 -0.210184003 -0.032395135

0.021883119 0.100980985 0.115910459 0.158059244

0.231512539 -0.184640786 0.308648883 0.051875572

0.31833404 -0.356520828 0.067309108 -0.25354976

0.100351615 -0.05488068 0.280233728 -0.011008631

0.104723817 -0.263983798 -0.082655372 -0.327001743

0.082017261 0.166131432 0.021787664 -0.006858595

0.169900466 -0.28102559 -0.264171741 -0.374604009

0.201360606 0.00592766 0.111328743 0.221834607

0.105814916 0.093234411 0.124582616 -0.290770701 -

0.06192445 -0.188746351 -0.063052129 -0.176970205 0.330438085

0.061048948 0.125573673 0.018357152 0.378191381 -

0.059500106 0.012306365 0.069366899 0.02979378 0.114092224

0.27659142 0.061485992 0.205259531 0.164801537 -

0.266127382 0.163219567 0.190041022 -0.237379296 -0.438110975

-0.066136787 -0.003372874 -0.450838506 0.227084922

0.185118534 0.195094095 -0.218235669 -0.249872444

0.153177658 -0.118622344 0.061405561 0.036149199 -

0.116788054 0.024340402 -0.029678675 0.217058817 0.318005232
0.333560227 -0.128305784 0.022158931 0.114230106
0.294439827 0.016748703 0.35172088 -0.094030885 -
0.061250908 0.117109168 -0.024902798 -0.126505713 -0.474226198
0.335606702 -0.011021971 -0.221098387 0.286084781
0.377859631 0.133146238 0.389211649 0.249592453
0.01983756 0.074347137 0.339442827 0.090960149 -
0.200303481 0.078899939 0.308136144 0.231177634 -0.09401348
0.243614776 -0.111539178 0.208756827 -0.127396289 -
0.285831606 -0.30445717 -0.031692799 0.141715237 -0.049421126
0.163577899 -0.019561451 0.02367524 0.06854954 -
0.417875465 -0.438221215 -0.212997056 0.330828724 0.266600012
-0.035579551 -0.027770706 0.045068258 -0.167816162 -
0.357965308 0.040582922 -0.295993838 0.273096109 0.263601641
0.187441314 0.191268992 -0.39133355 0.144648789 -
0.049137415 0.123824371 -0.294169869 -0.079681358 0.112463313
0.019692544 0.053541048 0.17173886 -0.317649738
0.463548605 -0.168771017 -0.108817103 -0.22595449 -
0.340129922 -0.099450915 0.070939096 0.042169068 0.139417124
0.120177123 -0.135515691 0.34241776 -0.02706931 -
0.108935746 -0.138598161 -0.260039112 -0.056696558 -0.004239558
0.126120274 -0.337852762 -0.090797222 0.194038691

0.086140263 -0.356101129 -0.273531171 0.333461906 -
0.220530784 0.278931448 0.083335884 -0.349839166 0.116311795
-0.28363808 0.379062237 0.218649148 -0.089644054
0.237746068 -0.082994207 -0.139726974 0.073440318
0.163375592 0.019416219 0.323948884 -0.031532021
0.153982029 -0.004790133 -0.318165392 -0.196193682
0.34063692 0.120868307 -0.06928345 0.137624279
0.194209065 -0.370774488 0.089677478 -0.268315841 -
0.275974525 0.183831381 -0.037881388 0.09726371 0.321638939
-0.326385431 -0.021471245 -0.092050459 0.414364347
0.077281009 0.159941108 -0.169868159 0.105634389 -
0.293092788 0.170183622 -0.19128335 0.219237551 -0.24353584
-0.082737761 0.144169257 0.205072259 0.029342277
0.297575328 0.376334011 0.254924407 0.074484498
0.229878856 -0.028814694 -0.060924025 -0.263522367 -
0.013896582 -0.113666358 0.177961858 -0.008668339 -0.210721418
-0.109248564 0.112650481 0.286505076 -0.397483148 -
0.030742634 -0.35036962 -0.072623099 0.04643902 0.184004659
-0.259057953 -0.420704271 0.141711114 -0.289677708 -
0.1468433 -0.027556849 -0.019798077 -0.02480273 0.100638261 -
0.018071262 0.139966857 -0.32756363 -0.219369506 0.121748571
0.242868888 0.104253901 0.435250184 0.109264162 -

0.252117163 0.231730732 0.047958609 -0.093245918 -0.274803305
-0.071440748 -0.005090994 0.070989244 0.088524683
0.250459457 -0.156988645 0.351277582 0.258096456
0.234775881 0.026924816 0.238565566 0.155599988
0.055384966 0.006170879 -0.003663908 -0.251947633
0.379737788 0.162267528 0.307783434 0.177321509
0.17265099 0.20809732 -0.258173962 0.249133275
0.161999319 0.038789159 0.1027123 0.034178948 -0.103362236
-0.335191684 0.163024431 -0.141981856 -0.140127767 -
0.196678417 0.284052691 0.041532469 0.08301086 0.153837327
-0.340542112 -0.13637746 0.267363017 0.0896726 -0.234353852
-0.086865691 -0.279306966 0.290813378 -0.119245477 -
0.219518933 -0.085796948 0.367664264 0.173615613 -0.113733638
0.271095883 0.045446887 0.132977402 -0.107547452 -
0.096957725 -0.070115844 -0.129256945 -0.220291543 0.264240678
-0.158695486 0.091675111 -0.007314595 0.157329742
0.025628146 0.034043586 -0.192178947 0.036455486 -
0.16549786 -0.074163876 -0.171247888 0.233028902 0.002034673
-0.307283658 -0.126620667 0.264105551 0.204201901 -
0.484610989 -0.221333621 0.100119141 -0.021866659 0.150136424
0.137319879 -0.044378872 -0.263338976 -0.147212309 -
0.115925282 -0.040328688 0.143219049 0.231751825 -0.230253643

-0.194550195 -0.003062981 -0.006565432 -0.39448894
0.067636293 -0.001618108 -0.282510057 0.288281302
0.074874417 -0.157195365 0.012565452 -0.024327366 -
0.195551715 0.236790776 -0.158517497 0.347783382 0.165474043
-0.330935141 0.351654514 0.044124247 0.324141716
0.391704521 -0.022990362 -0.195912034 0.18184613 -
0.072660863 0.237208504 0.28369581 -0.03387722 0.399672286
-0.044656368 0.330703794 0.079125875 0.091968598 -
0.372964798 0.030290716 -0.216989203 -0.050457837 -0.339769362
-0.024358202 -0.09458118 0.151583998 0.100538632 -
0.363082898 -0.147194319 0.406863023 -0.155793825 0.060096336
0.324419927 0.162041102 -0.100395908 0.125162113 -
0.078241326 -0.213218136 -0.330635752 0.122672086 -0.336769593
0.16382986 0.072700219 -0.12272598 0.411782332 -
0.124335049 0.031372208 -0.245506265 0.242953391 0.028597959
0.317592994 0.107687719 -0.115414489 -0.332305824
0.17402202 -0.182312171 -0.351924749 0.256938318 -
0.145571381 -0.090159589 0.339468461 0.133833991 -0.257405598
0.019803869 -0.021093487 0.178187629 0.19141107-0.191764059
0.1002222 -0.005768818 0.308998195 -0.077992369 -0.009674561
0.107473995 0.21654992 -0.062272852 0.027057668
0.348572217 -0.092537176 0.034145594 -0.054825246

0.233264026 -0.008594907 -0.140511389 -0.071670289 -
0.052191956 0.268578528 0.072582162 -0.397571112 -0.090221093
0.049114906 -0.301767793 -0.237335918 -0.307985902
0.373861274 0.020660042 -0.096057827 -0.077755381
0.131001691 0.059881335 -0.292838446 -0.186370785 -
0.074951123 -0.348429468 0.026309199 0.148229073 -0.273422859
0.056701165 0.338129633 0.248065636 -0.106662292 -
0.075857478 0.227355249 0.203617736 -0.008560607 -0.175128903
-0.049011007 -0.126891591 0.226795009 -0.178450026 0.3207025
-0.192426844 -0.052087043 -0.024101676 -0.342690958 -
0.085223252 0.212308572 -0.084279323 -0.31443194 0.18016143
0.120082313 0.042197426 -0.19214783 -0.21119624
0.134272828 -0.075630898 0.160891149 -0.088085028 -
0.06062462 0.31588892 -0.092652655 0.120133434 -0.068572272
-0.012767453 0.237238149 -0.083044963 0.026331871 -
0.114892320.377869305 0.114654369 -0.06658567 0.028816417 -
0.245098833 0.008211587 0.143814675 0.203539056 0.012415913
0.127925768 0.014859 0.123023355 0.095133748 -0.215098868
0.052713128 0.129038062 0.210559355 0.00835996 -
0.19625824 -0.268543841
KEGG_RIBOSOME 0.309121383 -0.629649737 0.481476929 -
0.692756647 -0.446730142 0.568397738 0.520487988 0.085621352

-0.079554925 0.069059459 -0.799373419 -0.250789666
0.669947414 0.503341951 0.295952593 0.287345926
0.680436065 0.06561505 -0.266614125 -0.015785769 -
0.010094642 0.281485659 0.217590725 -0.028878824 0.45527112
0.457832363 0.818028977 0.264079296 -0.70950438
0.14815047 0.074556137 -0.888083598 0.341645529 -
0.492064535 0.351016516 -0.166674835 0.654257402 0.297608364
0.229760207 -0.761723387 -0.310410425 -0.657193618 -
0.646884359 0.106486406 -0.440764507 0.096744715 -0.334837061
-0.661858707 0.196919611 -0.358304464 0.119203507 -
0.252124605 0.633275151 0.111133358 -0.570916106 -0.236257459
-0.317181507 -0.470450307 0.318400422 0.101842221
0.590528711 -0.477634334 0.80898003 -0.449694786
0.597150173 -0.28661959 -0.631053407 -0.549288121
0.387320106 -0.218645683 -0.337411345 -0.399332582 -
0.607007148 0.356529556 -0.008272785 -0.271970949 -0.717209213
-0.327971128 0.679730655 -0.350049366 -0.229313284 -
0.677931357 0.437385487 0.849541394 0.132477497 -0.185242942
0.318214525 -0.780669134 -0.572542169 0.428240493
0.642788688 0.815157988 -0.418766933 -0.025255996 -
0.568524927 -0.447983797 -0.300500411 -0.381011204 0.687602261
-0.532785572 -0.709145166 -0.314465458 -0.63755289

15784 0.582791752 -0.421020952 -0.103965998 0.666001012 -

15785 0.833280834 -0.553437132 0.303793382 0.031990332 0.558179648

15786 0.35289969 0.662130994 -0.825507784 -0.633161316

15787 0.830706083 -0.314290012 0.259668242 -0.80157628 -

15788 0.416796856 0.243806009 0.468528425 -0.506928777 -0.357252886

15789 0.448698804 0.773038195 -0.417198813 0.236558001 -

15790 0.595660998 -0.513053607 -0.591606693 0.312596601 -0.684023286

15791 0.718695494 -0.502532012 0.575064035 0.146172249

15792 0.353358707 -0.686538518 -0.837424494 -0.04135733 -

15793 0.693799146 0.369089196 0.864596719 0.655181583 0.564464735

15794 -0.662942835 0.084031198 0.493802169 0.582915103

15795 0.101993465 0.462536786 0.506875988 -0.490661808

15796 0.756033588 -0.439616547 -0.697025973 -0.573706739 -

15797 0.690911198 0.731331869 0.445100342 0.570625598 0.618998104

15798 0.555341783 0.407497682 -0.123482629 -0.625639188

15799 0.233312623 -0.771440354 0.253225523 -0.258422132

15800 0.378149044 0.92312188 -0.506418535 0.43847256

15801 0.263105622 -0.474259454 0.596825646 -0.448296686 -

15802 0.387840351 -0.410982608 -0.772797309 0.661441045 -0.413467909

15803 0.452098265 -0.155486336 -0.665746759 -0.350736844 -

15804 0.777014363 0.799236193 0.749049215 -0.870592812 0.246236572

15805 0.799269834 0.440552347 0.291039417 0.79576888 -

0.134749357 -0.750793891 0.713612428 -0.558892919 0.722783154
-0.11354129 0.355724785 -0.11843094 0.384991215
0.690839853 0.547433254 -0.510765505 -0.355119126
0.145811378 -0.580126093 0.320589871 -0.074948779 -
0.371072641 0.543455459 -0.599772571 0.486639323 -0.736160973
-0.452976093 -0.337550097 -0.304770135 0.550386807 -
0.191706352 0.725705628 0.724557275 0.47064257 -0.599259974
0.141136806 0.305388899 0.110122857 -0.479016538 -
0.628746844 -0.50965155 0.108508543 -0.311312496 -0.609683718
-0.735728416 0.56745794 -0.542955538 0.13166701
0.663894246 0.590855089 0.577982184 -0.405062269 -
0.65160844 -0.247000924 -0.814117491 -0.566633274 -0.71380243
0.776649594 0.072057641 -0.435971806 0.718011494
0.451505515 0.59651489 0.619057344 -0.275281806 -
0.374143829 0.54216659 0.134777236 0.547445675 0.253927471
0.17396918 0.234382137 0.215493994 0.881395846 -
0.299085087 0.146465402 0.854817227 0.043393013 -0.56395796
0.601376717 0.708673057 0.486749811 -0.193288333
0.704565169 0.379580631 0.204189366 -0.719967242
0.766121276 0.303008281 -0.02487126 -0.334385107
0.524261511 -0.675685141 -0.703831368 0.507365681 -
0.522715034 -0.146686783 -0.457147843 0.457664922 -0.561722372

15828 -0.339108105 0.052142879 -0.445048538 -0.765469645 -
15829 0.130367023 -0.5786035 -0.780239139 -0.758418451 -0.477148121
15830 0.441691251 -0.512320398 0.519577642 0.356646585 -
15831 0.499348892 -0.16277403 -0.295797357 0.245872876 0.465821736
15832 0.291944135 -0.62922403 -0.478919525 -0.651024296 -
15833 0.244908015 -0.714980524 -0.431155606 -0.590052802 -0.584410177
15834 -0.501775813 0.513387512 -0.260341333 0.411862571 -
15835 0.544808717 0.641228847 -0.574960118 0.685228609 0.735806301
15836 0.471789829 -0.106178907 0.441743395 -0.479127266
15837 0.09584475 0.503612811 -0.545325529 -0.155196009 -
15838 0.801534653 -0.059983446 0.596086709 0.743161852 -0.21225323
15839 -0.385596161 0.100184068 0.566073827 -0.560190789 -
15840 0.78336552 0.092086472 0.493442245 -0.02771584 0.706649492
15841 -0.40069055 0.315411146 -0.249294977 -0.004899804
15842 0.298244366 -0.480735083 0.550220136 0.671841297 -
15843 0.310393816 -0.516671626 -0.459151303 0.511311543 0.717729399
15844 -0.750217758 0.648840366 0.21664635 0.856090902 -
15845 0.673650998 -0.78180588 -0.270855847 -0.679706666 -0.18596348
15846 -0.466421805 0.52890276 0.624763226 0.809915978
15847 0.142755071 -0.699721739 0.772997029 -0.173167059 -
15848 0.093533125 -0.502508575 0.659878777 0.605875655 -0.699576619
15849 -0.494782356 0.308773058 0.179941508 -0.452035011

0.240455235 -0.376648706 -0.63359194 0.325921473 -
0.656930238 0.38980352 0.685475014 -0.648163115 -0.540673684
-0.571071604 -0.38147265 0.109686628 0.363293404
0.037519949 -0.727378872 -0.509112005 0.447958392 -
0.321973645 -0.178370285 0.699090631 -0.67684776 0.030510873
0.067219088 -0.156141071 0.243272991 -0.683558556 -
0.51923699 0.303526476 0.639421737 -0.584880957 0.560724151
0.118138603 -0.137419571 0.577733164 0.081490662 -
0.752920823 -0.600928398 0.525483573 -0.264678248 -0.613103289
-0.564651322 -0.504838673 -0.629240586 -0.668619137 -
0.053360336 -0.526019178 -0.305379691 -0.026143121 -0.125420335
0.197116648 -0.326409665 -0.43837369 -0.467954826
0.391016625 -0.846671536 0.311599801 0.186992252
0.459601679 -0.72895762 -0.513977634 0.125787672 -
0.605765184 0.676507154 0.752349073 -0.343505832 0.602381242
0.65684572 -0.777963108 0.696256976 0.600110112 -
0.707859573 0.332643445 0.460525779 0.437759563 -0.482472969
0.630663922 -0.749344888 -0.339834798 -0.344527122 -
0.363296498 -0.784682614 0.033853328 -0.627540742 0.522759312
-0.185542687 0.627708969 0.363510545 -0.444587719
0.407364709 -0.436670937 0.616834627 0.511389154 -
0.375387595 0.457088458 -0.236792862 -0.570232447 -0.101869439

0.159014264 0.415107571 -0.301708468 0.125113828 -
0.251248163 -0.214622068 0.48065933 0.110553635 0.005915212
0.384676826 -0.090716487 -0.516271734 -0.387467689
0.67037058 0.053716486 0.068604603 -0.03893683 0.7919808
0.282497977 0.768052212 0.442378466 0.468379459 -
0.288588947 0.606877004 0.047699842 0.181776573 -0.498070388
-0.534064707 0.373467417 0.305009121 -0.692657441
0.237379796 0.054184944 0.664100427 0.583573573 -
0.539208244 0.149997709 0.585996974 0.15638735 -0.146403938
-0.387039012 -0.582933604 0.041294551 0.340725938 -
0.185700433 9.75E-05 -0.391271524 0.229778523 0.264270192
0.520716122 -0.009576204 0.281187996 0.603867363 -
0.400627586 -0.152178232 -0.170734169 -0.395002227 -0.517579272
-0.278064961 0.107106465 -0.629805507 0.067925838 -
0.433374817 -0.468955708 -0.545564188 -0.286370444 -0.126405712
0.388264248 -0.305899327 0.421894292 -0.114347984
0.508993184 0.212047854 0.141533725 -0.497579067
0.392865228 0.400829907 -0.445026357 0.634885031 -
0.448676859 0.279513589
KEGG_RNA_DEGRADATION 0.362793002 0.095628767 0.392066704
0.010050966 -0.485226729 -0.050447596 -0.05334599 -
0.341007882 0.384504941 -0.145701525 0.113270917 0.274293568

15894 -0.086336381 0.320536491 -0.423950457 -0.101642524

15895 0.476749037 -0.067553361 -0.462277861 0.402597534

15896 0.315991996 0.005168452 0.214174235 0.391383422 -

15897 0.201536511 -0.094805396 0.190652129 0.036130913 -0.127302823

15898 -0.36106794 -0.001291476 -0.226525237 0.195701323 -

15899 0.392778483 -0.172982179 -0.290215334 -0.002333394 -0.413071653

15900 -0.112214817 0.14356332 0.144509525 -0.254710552

15901 0.243247404 0.061915908 0.451776715 0.143746735

15902 0.344074887 0.159156854 -0.118567246 -0.297012097

15903 0.169272096 0.259971792 0.105264239 0.165675766 -

15904 0.281085553 0.059326256 -0.518459333 -0.272804462 0.166574874

15905 -0.111145781 0.38349378 -0.291558514 0.378566583

15906 0.277317516 0.196672902 -0.231507008 0.100485761

15907 0.03097407 -0.431662395 -0.428589288 0.496700992

15908 0.223385165 -0.038988084 -0.151790117 0.074628965 -

15909 0.488025705 -0.26825256 0.272440283 -0.286364283 0.045195065

15910 -0.389771779 -0.252125837 0.23098701 0.067950613

15911 0.069812252 0.434681229 0.410888063 -0.084254924 -

15912 0.33915599 0.237432664 0.255126696 -0.270192469 0.182157382

15913 0.293966234 0.100562214 -0.093934643 0.101786539 -

15914 0.068432045 -0.200584786 -0.059749214 0.164291372 -0.367152874

15915 0.227712486 -0.191922841 -0.290446462 -0.007476776 -

0.039284161 -0.095487335 0.17101268 -0.26653238 -0.573803894
-0.139859213 0.019277128 0.3860331 -0.366041812 0.290307166
0.108601278 -0.377651857 -0.371809128 0.18342144 -
0.544381319 -0.075719182 0.305968746 -0.173633519 -0.113489935
-0.479025119 0.286847995 0.006628564 0.202420706
0.071541925 0.339540772 -0.141071378 0.036965107
0.157795241 0.400111887 0.068253364 0.051925384
0.386648665 0.207824565 0.173705257 -0.380439602 -
0.179993366 0.274437257 0.120388771 0.433471872 0.299408995
-0.154391519 -0.297244665 -0.504441716 -0.10220603 -
0.457785816 0.400138396 -0.163736684 -0.381906574 0.239672356
-0.387954274 0.059898808 -0.332788361 0.027054488 -
0.243480499 0.320727315 -0.180083796 0.182071836 -0.327060827
0.098440709 -0.171001027 -0.131344146 -0.201690498 -
0.473330396 -0.242894531 0.42224251 -0.198855312 -0.248639078
-0.114223869 0.093919332 0.252124596 0.362802586
0.054897226 0.299431114 -0.054240354 -0.315133075
0.092341608 -0.113950965 0.041673597 0.039193873 -
0.281250842 0.370642701 0.193033038 -0.442660135 -0.292550431
0.528302727 -0.117130037 -0.36325072 -0.190483925
0.288682874 -0.395067021 -0.017402395 0.122801789
0.043495035 0.227240926 -0.1763549 0.099478476 -0.213567611

-0.41624578 0.005073735 -0.006605136 -0.286572375
0.303885274 0.292967237 0.157843474 -0.023092011 -
0.217113784 0.052183763 0.460798044 -0.511822232 0.3232466
0.257339896 -0.034930683 -0.048312336 0.303763806 -
0.332800942 0.021221313 -0.459868076 0.535279715 0.04006206
-0.023522531 0.248159085 -0.265434996 0.27289112-0.510579503
0.006272314 0.074148957 0.03476935 -0.409044534 -
0.074948878 0.011040769 -0.387038908 0.292965296 0.10493853
0.033932773 0.024921911 0.138045081 0.273526281
0.229031965 0.385439124 0.073291115 -0.328826019
0.008003405 -0.264554878 -0.375990694 -0.167136063
0.034936544 -0.415787001 0.374170042 -0.111085925 -
0.247660544 0.142616249 -0.327223854 0.186124848 -0.322410355
0.340081795 0.356867409 0.183029681 -0.42762485 -
0.029117150.237921056 -0.112790237 0.068574138 0.16911816-
0.526202177 0.136078567 0.458669634 -0.031303108 0.109543115
0.379287188 0.269476694 0.092153498 -0.226273908 -
0.105810244 -0.185930341 -0.376423084 -0.204011825 0.30850626
-0.552622805 -0.311729017 -0.326817434 -0.38718538
0.334385446 -0.15685634 -0.285798416 0.230513461 -
0.415771433 0.207770735 -0.048858973 -0.425034951 -0.357448155
0.033451245 -0.038405158 0.31124827-0.346585588 0.040867113

-0.280333288 -0.416628023 -0.32000383 -0.172270062 -
0.28042267 0.005080172 0.13642187 -0.156773003 -0.408804817
-0.123140082 -0.001153664 -0.379940305 0.334258215
0.142441744 0.269248778 -0.456854936 0.016837683
0.348025873 0.316774666 -0.303931985 -0.006463708
0.158405223 -0.527563601 0.252483748 -0.468246348
0.214983847 -0.431838867 -0.055891478 -0.214058009 -
0.083347005 0.308833832 -0.190969921 -0.457314093 0.27243038
0.199296167 0.333871356 -0.011520244 0.285171946
0.184967267 0.336252508 0.247647188 -0.259675959
0.175225768 0.055584039 -0.093150081 0.084419657 -
0.023349519 -0.244273471 0.350083639 0.17469472 0.23508599
0.220833091 0.359848716 0.306984139 -0.379765891 -
0.378713341 0.040233018 0.227999059 -0.055993052 0.04505063
-0.108663521 0.210992591 -0.306949178 0.305783685 -
0.069890201 0.274383931 0.430574174 0.09771266 0.143322888
-0.040701069 0.30608353 0.089486962 -0.089211085
0.362094583 0.027302657 -0.287323322 -0.224856088
0.016356324 0.281381211 -0.095903516 -0.228210081 -
0.09923412 -0.099413287 0.092341848 0.08221685 0.062112289
0.132660952 0.214676223 0.149221861 -0.095586693
0.138548515 -0.087686122 -0.346963432 -0.178004647

15982 0.022909334 -0.156941285 0.162183703 -0.133229422 -
15983 0.087960827 -0.003251162 -0.128809247 0.344535138 0.149876811
15984 -0.288673677 0.027975973 -0.355313366 0.183587866
15985 0.057309346 0.249740742 0.277721366 0.025464289 -
15986 0.463240372 -0.236669769 -0.398911784 0.048379102 -0.331262172
15987 0.281442807 -0.345214054 0.12108533 0.317818868
15988 0.226711041 0.125663021 0.226833621 -0.362012559
15989 0.124008217 -0.392461615 0.036083279 0.24698624
15990 0.300162724 -0.272902427 0.071013839 -0.267331289 -
15991 0.427687174 -0.205974448 0.080626464 0.066331841 0.266648094
15992 0.509306096 0.08485726 0.05627825 0.304235044
15993 0.04840576 -0.53026383 -0.123229379 0.058942881 -
15994 0.224282149 -0.322500818 0.098388091 -0.349598938 0.409863438
15995 -0.30966716 -0.255016042 -0.304220318 0.011832115 -
15996 0.049083651 -0.150780105 0.053533444 0.010081937 0.09104747
15997 -0.195507312 -0.074247979 -0.385191214 -0.307872352
15998 0.261994328 -0.347701484 0.287514002 -0.36980833
15999 0.288334201 0.206353646 -0.169330237 -0.402543201 -
16000 0.132877855 0.136083203 0.227971978 -0.109318903 -0.308351868
16001 0.07877359 0.018370525 0.236984572 0.261226962 -
16002 0.244997992 0.000868518 -0.250433625 0.016588367 0.359741197
16003 0.180690046 0.167976406 -0.009939065 0.346473274 -

0.299251863 -0.129196334 0.211095086 0.257678486 0.157150781
-0.305842025 -0.341662543 -0.061666743 0.318755645 -
0.123872365 -0.224423996 0.207794817 -0.221446485 -0.061162455
-0.074047733 0.0267653 -0.250758994 0.209022516 -0.503607978
0.339205709 -0.383011952 0.106674538 -0.108131429 -
0.243658826 -0.084558223 -0.153684829 -0.059748602 -0.022671738
-0.009051781 0.064930597 0.046755018 -0.168198518
0.192785729 -0.24547356 0.394264183 0.251850034
0.150996655 0.16135345 0.297782814 0.251249012 -
0.328955641 0.252711849 0.106385469 0.20726325 -0.21661445
-0.182887381 -0.369217036 0.065740427 -0.213861343 -
0.060750222 0.003462027 -0.057679752 -0.123526918 -0.250494631
0.053826564 0.018038065 0.237837519 0.112417115 -
0.036171189 -0.12986118 -0.193182223 -0.205302115 -0.178112834
0.066589564 0.108373542 0.088380021 0.340026647 -
0.17175137 -0.166856623 0.13194816 -0.237661177 -0.291767042
0.080698694 0.16485122 0.082032508 -0.163855134 -
0.043502267 -0.159648934 -0.328502921
KEGG_RNA_POLYMERASE 0.437117198 0.208503394 0.080842913
-0.157089354 -0.334416646 -0.065864521 0.249277223 -
0.186606231 0.03936296 -0.223205229 -0.418484506 0.286030829
-0.258559628 0.131034265 -0.302682039 0.166957271

0.412380436 0.318965157 -0.537770279 0.236249119
0.272919919 0.022242708 -0.081272205 0.460246393 -
0.051184311 -0.331760281 0.091370996 0.160262327 0.100788244
-0.216842937 0.212604734 -0.440136041 0.338570765 -
0.393716089 -0.267135789 -0.009375171 0.135914749 -0.181348229
-0.295081853 -0.052913932 -0.073709913 -0.327298757 -
0.114931167 0.14348778 -0.023791611 0.245073186 0.191800541
-0.379287295 -0.384133136 -0.154950145 0.044913498 -
0.226638303 0.170979561 -0.032330383 0.473930216 0.190679439
-0.500444636 -0.1566314 0.168436315 -0.186641676 0.339626466
-0.450716133 0.32580003 0.308907682 -0.008602474 -
0.297874517 -0.08753364 -0.049604128 -0.432988533 -0.175183062
0.247782323 -0.225843264 -0.308229229 -0.025379576
0.192431473 -0.295319545 -0.413473228 0.225844638 -
0.035350413 0.382122069 -0.339852431 -0.254680232 0.122876523
0.400845844 0.177613349 0.167359265 0.272056253 -
0.223011913 -0.367016005 0.391093053 0.193475985 -0.17218716
0.279989763 0.297408484 0.146800166 -0.324856134 -
0.176532355 -0.256309717 0.190982141 -0.256318281 -0.196656256
-0.402658116 -0.191736245 -0.016005878 -0.359108113 -
0.309955821 0.261704249 -0.042459105 -0.242882445 -0.118726233
-0.46429315 0.224134856 0.440467177 0.138976978 -

0.374767692 0.099603427 0.41315958 -0.282943884 -0.393033392
0.244955972 -0.423115652 0.195654666 0.362590793 -
0.29289716 -0.291174505 -0.238289416 0.29467928 -0.416927201
0.511130358 -0.054863985 0.311444715 -0.38731804
0.393310365 -0.183227873 0.377157997 -0.231683571
0.085009695 0.313941140.168205628 -0.186886914 0.088312948
-0.079349073 0.172663031 0.103256772 0.476856352
0.254195061 -0.058134979 -0.420210128 -0.418406209
0.245433002 -0.335867896 0.244008264 -0.376074914 -
0.455805865 -0.22622532 0.020688192 0.104309499 0.020986822
-0.226901094 -0.403339202 0.416154997 0.166321714
0.286758401 -0.375014348 0.396135948 -0.185429709 -
0.063788564 -0.328087965 -0.106606109 -0.459671786 0.536618679
-0.217181684 -0.120590296 0.158980524 -0.01374777
0.071123967 0.121301202 -0.220626529 0.349724216 -
0.077018998 0.019005653 -0.257257704 -0.206820922 0.236427572
0.140834739 0.013770602 0.080090895 -0.184040719 -
0.446363018 -0.325953976 0.449099275 0.129144064 -0.199795886
-0.381921601 0.454755286 0.079106227 -0.104730048
0.237847717 -0.162689092 -0.209878779 0.069022225
0.174056365 -0.201387543 -0.241739852 0.213437928
0.348307124 -0.332942238 0.547791946 0.240336378

0.268770732 0.356351278 0.328823575 0.020555923
0.244652011 -0.169626248 0.293013679 0.427867474 -
0.393767366 0.07063746 -0.162164792 -0.506094829 0.349145578
-0.265588143 0.548199766 -0.143691949 0.027156151
0.244069808 0.030187494 0.330281558 -0.178837744
0.299009567 0.211775619 -0.135132872 -0.460200607 -
0.083270063 -0.010943259 -0.437926358 -0.01442072 -0.273767355
0.031299877 -0.034485468 0.020793998 0.15103454
0.235707963 0.267359588 0.264719308 -0.159681447 -
0.079257065 -0.463613659 -0.360771289 -0.050599841 0.405453276
-0.468726786 0.015009164 0.22294737 -0.010146929 -
0.000935675 -0.137057414 0.304030289 -0.172974441 0.07233071
0.462715382 0.319250294 -0.355401249 0.092640363 -
0.117184586 0.397554754 0.061149165 0.218444917 -0.395581723
0.34503438 0.48338739 0.112526899 -0.103405319
0.542453213 0.365234656 0.2436709 0.257181043 0.001253621
-0.346738559 -0.458918383 0.128823064 0.28472916 -
0.467840709 -0.538020205 0.244405995 -0.433189134 0.039147869
0.197642994 -0.301369172 0.21565523 -0.145031811
0.167211834 -0.135029814 -0.237315015 -0.313697793 -
0.056534319 -0.245625982 0.164700693 -0.052443774 -0.135845499
-0.299956805 -0.405085555 -0.24103914 0.081395598 -

0.209887213 -0.234844092 0.084934233 -0.202157394 -0.218539632
-0.336471283 0.120042017 -0.414351529 0.23292509
0.279000934 0.166291671 -0.410175588 -0.167878177
0.315001103 0.233449383 -0.310737114 0.195296394
0.331100617 -0.376456645 0.425121176 -0.356259803
0.405899158 -0.201360025 0.185613448 0.158804704 -
0.229745509 0.390041496 -0.122439639 -0.15251816 0.354408923
0.180770239 0.243436479 0.118788914 0.027823293 -
0.28181877 0.36332133 0.427728963 -0.377565209 -0.205824467
0.207306161 0.126873128 0.009551889 -0.054994924
0.030815613 0.257767533 -0.053237722 0.037405311
0.036826017 0.356935062 0.274382615 -0.128112889 -
0.107861053 0.233063294 0.444461196 -0.07148845 0.317769953
-0.363791457 0.170811916 -0.194663287 0.335652009 -
0.506953654 0.256877398 0.269918823 0.384343417 -0.236011361
-0.318580253 0.179572122 -0.055465347 0.095924746
0.213475581 0.07277813 0.061794267 0.184213383 -
0.260394068 -0.358639382 0.087917703 -0.288114822 -0.141896347
-0.087114915 -0.030313613 0.215774132 -0.330747805 -
0.407509458 0.278769866 0.315066001 0.346377592 0.051815168
-0.2796126 -0.305509291 0.090690203 -0.06683799 0.204183127
0.164943325 -0.240662762 -0.361653035 -0.435651037

0.144430202 0.200904441 0.004546512 -0.1589472 -0.187470512
-0.244319523 0.023924477 0.165635666 0.118435443
0.340254362 -0.297223905 -0.340271902 -0.030937916 -
0.23462895 -0.069379986 -0.059740338 0.139694422 -0.378674352
0.523376474 -0.315160712 0.24872716 0.072921034 -
0.106063847 -0.08164284 0.367134543 -0.134553466 0.03913592
0.175860565 0.203634036 -0.410271047 0.060155388 -
0.258997789 -0.283186256 -0.18444936 -0.229961836 0.257851162
0.188278277 0.57918055 0.111124142 0.037601356
0.029247326 -0.169182752 -0.435511135 0.097203314 -
0.086250782 -0.366517594 -0.376218428 -0.166687919 -0.178321999
0.072622285 -0.316375897 -0.092231571 -0.153181495
0.416575134 0.06302104 0.002983294 0.016122681 -
0.277604362 0.084899391 0.291886834 -0.455871542 -0.465831504
0.361220385 0.245070834 -0.176467033 0.120287889 -
0.610471261 0.160278571 0.007324768 -0.068983745 -0.385708189
-0.20327228 -0.026592176 0.02402144 0.277609662 -
0.148840106 0.396179663 -0.137399551 -0.17101311 0.374747856
-0.001869597 0.46056163 -0.421598217 0.333457717
0.25842995 0.144839304 0.251930153 0.000228664
0.138814129 -0.294049454 -0.152867072 -0.066839483 -
0.150832777 0.056885779 -0.143548746 0.04894423 0.124495693

0.2527741 -0.36570505 0.114307044 0.394813634 0.219895045
0.047464593 -0.203392172 -0.034447416 -0.069251671
0.224755206 -0.123319347 0.182664137 -0.291052853 -
0.030888002 -0.16192332 -0.066801177 0.016816502 0.463953399
0.293321807 -0.127813843 -0.493037824 0.10550872
0.271307619 -0.118656283 0.293333792 -0.143100199
0.476412256 0.247836174 0.272347177 0.126582974
0.113131537 0.165112784 -0.32407931 0.130581096
0.189785922 -0.012573451 0.101792569 -0.044909688 -
0.2701171 0.152244118 0.020363193 0.052426055 -0.217371133
0.261673971 -0.134694171 -0.213878313 0.227961812 -
0.141729028 -0.011033484 0.049595029 0.114034931 0.357015892
-0.509529706 -0.052374688 0.08114817-0.451186927 0.254929182
-0.260939588 0.189677547 0.132537028 0.276974239
0.077431349 0.014166655 -0.34764705 -0.024628115 -
0.122595391 -0.285351268 0.014783861 -0.075097589 -0.114600518
-0.149384954
KEGG_DNA_REPLICATION 0.339117266 -0.279501368 -0.024618185
-0.46583162 -0.542134452 0.058915022 -0.106145417 -
0.441876594 0.751427102 0.233476514 -0.158887174 0.288253285
-0.320006788 0.478342003 -0.306927867 0.434469044
0.158608289 0.150329707 -0.474749606 0.479636637 -

0.23240793 -0.321739811 0.063213007 0.372233535 0.107397372
-0.325824361 0.236293172 0.541147588 -0.341646085 -
0.41643644 0.148556121 0.000157344 -0.320761911 -0.09248052
-0.245470213 0.458316521 -0.420834236 -0.328397085 -
0.539298709 -0.335040135 -0.314336987 0.181118443 0.076145323
0.168150791 0.408081648 0.408189554 -0.2777956 -0.46834524
0.418073483 -0.304161875 -0.303072435 -0.123792082
0.554564113 0.139206511 0.085279325 0.40417429 -
0.452922341 -0.480864727 0.089306854 -0.192887551 0.51694911-
0.240810632 0.456218988 0.276416026 -0.303542649 -0.083875994
0.183003672 -0.390065858 -0.472877101 -0.355160277
0.627603405 0.485737477 -0.419667421 -0.428725325 -
0.035641638 -0.315609962 0.345666633 0.341513789 -0.15044559
-0.448978977 -0.436504572 -0.269371215 -0.059698558
0.059595388 0.34915312 0.715877855 0.258345388 -
0.373875173 -0.086862387 0.081831893 0.520717143 -0.409293349
-0.111093072 0.356471669 0.056971527 -0.0916271 -0.499437599
0.008688576 0.11076361 -0.57109607 0.199642443 -0.468774435
0.004040589 0.135851303 0.440696027 0.332332125 -
0.162317446 -0.353080697 -0.283964904 -0.445004317 -0.622653336
0.258542 0.518182911 0.122450714 0.154463275 0.424720013
0.551751602 -0.407488224 -0.100845197 0.117180172 -

0.573385926 0.290003027 0.559641019 -0.563710608 -0.455574434
-0.430196437 0.042951385 0.217637568 0.073947074
0.177413518 0.497361974 -0.112878987 0.167739812 -
0.051957449 0.245829218 -0.526890583 0.230034053 0.495589634
0.360710428 -0.447548819 0.098089115 -0.414146793 -
0.014604081 0.323897212 0.146099455 0.018370831 0.070365183
-0.342405426 -0.478537412 0.276215617 -0.359653401
0.498745138 -0.025623164 -0.383615622 0.032708248 -
0.157658904 -0.367547814 -0.111993769 0.675472504 -0.005209524
0.138431406 0.456593035 0.214226415 -0.464209785
0.347127625 0.143354818 0.019572342 0.211205544 -
0.392168069 -0.327807051 0.552627185 -0.316096895 -0.513266446
-0.112233867 -0.252243327 0.406519335 -0.223638877 -
0.09701775 0.316822617 0.423560125 -0.255601994 -0.507343357
-0.114325138 0.178155943 0.179208564 0.330577431
0.404904052 -0.297828454 0.264443336 -0.283112037
0.571444742 -0.082580402 -0.467493208 -0.282060957
0.352776949 -0.244966616 -0.379464067 0.051751509
0.019754273 -0.502968382 -0.390373422 0.311004229 -
0.448462092 0.155352047 0.418761597 0.432789438 -0.400345979
0.359177868 -0.255028348 -0.31930146 -0.006522799
0.678870984 -0.419914761 0.547130579 -0.306519276

16202 0.525506181 0.325568494 -0.292268889 0.304777802

16203 0.051525341 -0.321452188 0.263682181 -0.653714066

16204 0.768893726 -0.481623441 -0.181844095 0.594911477

16205 0.188571157 -0.36973695 0.065696176 0.502685712

16206 0.054268396 -0.459297455 0.070709931 -0.247108003 -

16207 0.262637174 0.156951802 -0.383996623 -0.00483353 0.000722818

16208 -0.418023081 0.206613318 -0.019954379 0.114057324

16209 0.640138734 0.101336433 0.164255685 -0.175408181

16210 0.23937164 -0.543012712 -0.432317664 -0.034103006 -

16211 0.401726804 0.406836836 0.128911901 -0.335836324 0.278365663

16212 -0.484840125 0.255516438 -0.528857196 0.557347634

16213 0.005177299 0.354355603 -0.281369228 0.293613865 -

16214 0.510192749 -0.389879822 0.107032482 -0.361130103 -0.547752985

16215 0.083475164 0.701264776 0.297148101 -0.099712145

16216 0.186462325 -0.210415985 0.160318611 0.310875252 -

16217 0.014862185 0.205114395 -0.497343349 0.312156804 0.632520493

16218 -0.571127502 -0.543920659 -0.442897418 -0.551188175 -

16219 0.082530995 0.354636636 -0.630641615 -0.244264834 -0.383725257

16220 -0.077555684 -0.050444159 -0.483537324 -0.418517114

16221 0.058945385 0.085223168 0.481124904 -0.525874947 -

16222 0.326755885 -0.630251698 -0.584071187 -0.190888742 0.092575585

16223 -0.17562031 0.189383217 0.152611776 -0.010148767 -

0.350327228 -0.196565762 0.183919552 -0.488439371 0.236460467
0.040146299 0.249197877 -0.493086365 0.361135446 -
0.141215014 0.330752827 -0.534294674 -0.154152146 0.396902454
-0.456017147 0.594902256 -0.457989266 0.171934079 -
0.027835464 0.010373444 0.434823582 0.334295019 0.168587704
0.1235382 -0.287946718 0.19535907 0.491399187 0.414295645
0.430069433 0.237484979 -0.29831556 0.380735312
0.335288349 -0.443988767 -0.493504951 0.530433998
0.250188199 -0.091536247 -0.348414229 0.220974313
0.20950979 0.179117859 0.361065243 0.104702215 -
0.201026439 0.375781521 -0.477273043 -0.335602326 -0.355219653
0.506433741 -0.39504549 -0.343554072 -0.62871973
0.002641426 0.125399132 0.447724387 -0.28407487
0.550580954 0.307121516 -0.162854618 -0.180396108
0.263257689 -0.161155779 -0.176177608 -0.105956815 -
0.213220899 0.116341602 -0.225568072 0.635168064 -0.277746271
0.366452284 -0.307231254 -0.087121879 0.364968009
0.041891688 0.182494673 -0.38489898 -0.432807305
0.325676829 0.565456879 0.445247038 0.436826031 -
0.07958697 -0.326909317 -0.348149658 -0.269499018 -0.338201629
-0.591684557 0.522296346 -0.489528694 -0.450762004 -
0.22569533 -0.0712247 0.130851039 0.213475737 -0.059598596

16246 0.196439794 -0.041934315 -0.045158865 0.083132892

16247 0.029761232 0.015724645 -0.327538313 -0.467176786 -

16248 0.491545099 -0.314703128 -0.324145876 -0.110795184 -0.013925615

16249 -0.271011357 0.254505525 0.485698987 0.18146723 -

16250 0.236303021 0.240635027 -0.439568605 0.066620337 -0.334964423

16251 0.163702079 0.466854243 0.551330493 -0.511728808 -

16252 0.297613507 -0.292573611 -0.560918623 -0.458664874 0.063312867

16253 -0.265660718 -0.199337699 0.733286966 -0.1554126 0.230951221

16254 -0.402590472 -0.087131698 -0.620663643 -0.085013672 -

16255 0.191686829 -0.519536849 -0.408047701 -0.415708091 -0.108044842

16256 0.575335049 -0.637979174 0.284690228 -0.222560394

16257 0.123584663 -0.192862733 0.493272597 -0.377784054 -

16258 0.558076684 0.163505117 0.34789794 0.098988133 -0.103067962

16259 -0.333449965 0.243745153 -0.04614941 -0.1397852 0.218827334

16260 0.037065413 -0.550010926 0.33817696 -0.346501934 -

16261 0.167069519 -0.261295545 0.20542623 0.271792187 -0.037308259

16262 0.258892406 0.073783918 -0.360516906 0.335848371

16263 0.031104526 0.317493206 -0.471415503 0.371494502

16264 0.031602809 -0.1985198 0.518181273 0.113687489 0.50637499

16265 -0.603855862 -0.444568172 0.497590128 0.272594312 -

16266 0.172722269 -0.083946009 -0.21858952 0.092297483 0.218681161

16267 -0.240274448 0.602988348 -0.209724639 -0.358692953 -

0.623130914 -0.069607809 0.31547536 0.478440408 0.057600725
-0.165851323 -0.085903475 -0.223286937 0.039669411 -
0.192082803 -0.080172708 -0.526764435 0.385682307 0.616600126
0.381737619 -0.53363055 -0.339804554 0.291370992 -
0.005385803 0.38707871 -0.576289902 0.306563312 0.401355156
0.192748356 0.302951327 -0.283309348 0.04754373 -
0.342537983 -0.146239474 0.255657586 -0.17260111 0.333810323
-0.379978294 -0.351262658 0.162329256 0.343148571
0.450459483 -0.372786869 0.415640983 -0.236404903 -
0.533906359 0.172205268 -0.52391649 0.322602962 0.357555676
-0.159271656 0.279788485 -0.498967137 -0.207980033 -
0.118274316 -0.507057283 0.218725883 0.382303188 0.039737522
0.225037616 -0.048375108 0.500859611 -0.387746484 -
0.361841903 0.009696124 0.318928343 -0.081886303 0.308321492
-0.421130191 -0.006804938 -0.424364779
KEGG_SPLICEOSOME 0.410670332 0.154397246 0.264025737 -
0.075794144 -0.501154965 -0.319125809 0.186975172 -0.355334589
0.330753899 -0.182053541 -0.4101486 0.204729877 -0.238409422
0.366684234 -0.323407577 -0.102147129 0.542520537 -
0.240972038 -0.513873918 0.372113013 0.274834914 0.204197366
0.36179554 0.465995332 -0.069833104 -0.328261023 -
0.077207075 0.010083083 -0.39942647 -0.380167339 0.214546275

16290 -0.464236045 -0.04224012 -0.417135997 0.073570121 -
16291 0.278386895 0.343512348 -0.343446624 0.147874879 0.041052307
16292 -0.219735477 -0.307771599 0.374439355 -0.084521255
16293 0.423670324 0.017532543 0.458450968 -0.369951298 -
16294 0.185194945 -0.077493438 0.141019265 0.271859008 0.471393959
16295 0.282819023 -0.288807782 -0.144534094 -0.456443982 -
16296 0.203104465 0.136977023 0.046695442 0.478045805 -0.300248412
16297 0.464882314 0.407377977 0.054598777 -0.374922447
16298 0.080912512 -0.089725903 -0.516077417 -0.278424575
16299 0.391942015 0.243555953 -0.393511674 -0.236782246
16300 0.027192076 -0.498069179 -0.415473609 0.249259012 -
16301 0.342019976 0.280305916 -0.286315691 -0.044300724 0.03744182
16302 0.177799964 -0.021208431 0.431745924 0.469277917 -
16303 0.320896965 -0.289428088 0.286132259 0.198900788 -0.254530054
16304 0.458410948 0.152251696 0.239999326 -0.016333785 -
16305 0.190529089 -0.285656226 0.186542152 -0.411874783 -0.026836409
16306 -0.400629057 0.074254285 -0.293922732 -0.379240089 -
16307 0.20088267 -0.069587685 -0.197509519 0.10790481 -0.016347825
16308 -0.51979243 -0.036336978 0.29764414 0.375951864 -
16309 0.406639205 0.286567964 0.386383092 -0.352401447 -0.31825745
16310 0.299762709 -0.396046964 -0.163804344 0.352538026 -
16311 0.376112452 -0.158451048 -0.487676874 -0.135062982 -0.034972921

0.354945899	0.257624803	0.45498174	-0.155438676
0.337288631	-0.005010383	0.475801391	0.150090110.102631345
0.332708393	0.263260965	0.285862796	-0.311831235	-
0.429387832	0.258040913	0.191530045	0.45853564	0.50936106
-0.124490667	-0.372636356	-0.43468223	-0.062992808	-
0.440177739	0.321063257	-0.243333623	-0.367069996	0.140859702
-0.257897526	-0.052902707	-0.341365056	-0.04422391	-
0.426331922	0.320698834	0.031027255	0.299489339	-0.038184887
0.216802482	0.060049399	-0.240154982	-0.325932354	-
0.424788124	-0.489550429	0.605006872	-0.202571863	0.16152727
0.139035423	0.142788619	0.307566842	0.269459226
0.205023321	0.589361247	0.234883391	-0.219914619	-
0.201090947	-0.261318806	-0.235318782	0.254294979	-0.197189695
0.462880198	-0.052474329	-0.372877743	-0.346620435
0.541218091	-0.150132605	-0.297263826	-0.178285227
0.242728204	-0.250658929	-0.074986706	0.183776664	-
0.203074818	0.170482756	0.12037629	-0.095520062	-0.185383221
-0.448633693	-0.246820295	0.009989388	-0.351223544
0.091404257	0.237191353	0.060133798	0.244481953
0.304886299	-0.159622325	0.266273012	-0.37043717
0.277138972	0.266319738	-0.343013927	-0.107315987
0.111683786	-0.393324211	-0.015274193	-0.274699472

0.529966274 -0.159127147 -0.07722983 0.208736491 -
0.223332944 0.400259969 -0.560733668 0.013322513 0.307118498
0.008083522 -0.448119393 -0.201713464 0.017854088 -
0.396950172 0.286170939 -0.094313973 0.141352526 -0.14336071
0.404499461 0.224514523 0.108456484 0.416452047
0.175955728 -0.4737063 0.234113675 -0.303793098 -0.410501445
-0.194532109 0.250219595 -0.295711056 0.300012555
0.077503625 -0.235274748 -0.054646548 -0.106041035
0.261426568 0.128512937 0.205765861 0.352965643
0.251852139 -0.419423374 0.209131953 0.239365294
0.315180864 -0.122949724 0.362171954 -0.521205258 -
0.084739866 0.503382189 0.145100821 -0.03278501 0.499409824
0.278410388 0.287142488 -0.041287276 0.1144468 0.04874376
-0.418358782 -0.090548283 0.239259016 -0.522214036 -
0.378832971 -0.130715572 -0.472842835 0.214286503 0.006864711
-0.04675108 0.28455339 -0.398293069 0.486475179
0.074084425 -0.470967235 -0.264181196 0.130929215 -
0.221708451 0.301782553 -0.270583673 0.096203314 -0.300370313
-0.346425046 -0.346861354 -0.029716989 -0.513219393 -
0.076044759 0.199078284 -0.051442682 -0.298309656 -0.193638066
-0.142444444 -0.404643804 0.333403498 -0.135896587
0.243981646 -0.393192963 0.059590394 0.445436724 0.1493112

-0.422408793 0.05470408 0.174271233 -0.536108159
0.295073646 -0.384596222 0.439149843 -0.331385601
0.134967716 -0.19823492 -0.110203063 0.473577124 -
0.203181606 -0.486910479 0.391202641 0.195739968 0.23791018
0.091062597 0.203963776 0.178894046 0.287152315
0.417974551 -0.2002081 0.209504768 0.151818076 -0.345818353
0.148510105 -0.027187953 -0.225770656 0.321622805
0.161650812 0.169196037 0.152305721 0.221972376
0.413582881 -0.300585858 -0.323189425 -0.164192234
0.42867024 -0.267291645 0.325794666 -0.439001291
0.270327901 -0.291097629 0.422567359 -0.425828539
0.291906326 0.398462997 0.313734243 -0.016757251 -
0.248651119 0.390839648 0.167086666 -0.034419721 0.291657953
0.033635886 -0.212846871 -0.070592758 -0.014789738
0.131981179 0.15063357 -0.211925641 -0.105778743 -
0.0875845 0.133253947 0.220868318 -0.228353762 0.089789074
0.412238443 0.123442205 0.231816349 0.118867062 -
0.320650639 -0.557556663 -0.166773674 0.021146307 0.048176151
0.328557095 -0.171438322 0.125101581 -0.439216227 -
0.182035733 0.398042411 -0.145580899 -0.339034662 -0.271964675
-0.371010206 0.14815007 -0.217367866 0.335880101
0.250847266 -0.167672453 -0.403102353 -0.079688339 -

0.357142214 0.009177642 -0.399730269 -0.080912879 -0.188312903
0.34868493 0.125863568 0.184263828 0.227828553
0.281261643 -0.133075841 0.262581095 -0.383068859
0.123953749 0.372517619 0.151070427 -0.502365056
0.015404383 -0.18083148 -0.320732482 -0.448929348
0.299998839 0.120348016 0.113486311 0.515496079
0.290652727 -0.193941522 0.219552902 -0.229308336 -
0.436893362 -0.086489055 -0.229245436 -0.339831889 -0.327635389
0.321471201 -0.386808642 0.327516766 -0.220548923 -
0.141774437 -0.276344613 0.270544118 -0.139871568 0.069596461
0.248673594 0.030551677 -0.038857337 0.210251524 -
0.041627939 -0.308513041 -0.03432928 0.448204062 -0.479240172
0.318491069 -0.466503456 0.356699871 0.257755262 -
0.01949628 -0.550795544 -0.334613955 0.142586915 0.119060154
0.276129167 -0.277375843 0.182313327 -0.009847998 -
0.082468237 0.455790369 -0.196446963 0.250966177 -0.471647173
0.305948379 0.19841567 0.226507573 0.247413725 -
0.138982615 0.397427251 -0.437102067 -0.365816138 0.071647836
0.226077423 -0.000747388 -0.076323149 -0.399585322
0.023328119 0.263671557 -0.367018399 0.0102798 0.398346986
-0.002280928 0.222922187 0.211138709 -0.213826406
0.058850497 0.032934244 -0.378016824 0.401994537 -

0.413075179 0.057185256 0.044507359 -0.41656213 -0.013835101
0.079759898 0.182724742 0.126156061 -0.299096459
0.063485232 0.135533945 -0.004364655 0.303814156 -
0.138864465 0.272834485 0.366921485 0.004592625 0.145705981
0.352733105 0.223128578 -0.434648329 -0.059590028
0.275255434 0.146078479 -0.260026687 -0.181507474 -
0.37270272 0.156081506 -0.208332663 0.001290996 0.108451494
0.128836447 -0.25251785 -0.093566366 0.04903854 -
0.218826405 0.318357389 0.060220903 -0.057511823 0.042206546
-0.340961538 -0.143311597 -0.081490104 0.188166118
0.243010302 -0.007820395 0.403428008 -0.127307623 -
0.049537983 0.013928442 0.062694597 -0.393646511 -0.154531295
-0.097068505 -0.247242421 -0.219845655 -0.108969708 -
0.177315153 -0.191118012
KEGG_PROTEASOME 0.367623268 0.418743077 -0.429334731 -
0.566294682 -0.452457172 -0.54225483 -0.252682091 0.483996938
0.536521208 0.172690918 -0.582029535 0.29548614 -
0.115115103 -0.559308612 -0.344381388 0.351092572 0.49085952
0.036536576 -0.651309434 0.484306511 -0.337474671
0.576283798 0.500272582 0.111720481 0.685649955 -
0.318143772 0.208945597 0.263333687 -0.534957407 -0.501043971
-0.335554048 -0.418090658 -0.325699418 0.208860772

0.151849225 -0.467712154 0.087642156 0.037306808 -
0.450113818 -0.206796391 -0.479754199 -0.042338531 -0.528161505
0.588601148 0.502625902 0.186443367 0.218382345 -
0.501906211 -0.587575326 0.224862393 0.017531058 0.295096385
0.537394514 -0.377738832 -0.255951145 0.075036446 -
0.512919069 0.439438613 0.201492939 -0.220681814 0.471359354
-0.406156343 0.521813297 -0.194706655 -0.532723159 -
0.628074748 0.377783917 0.046216674 -0.340123594 -0.288074726
0.62074217 0.4513781 -0.506186443 0.196891253 -0.613728536
-0.411300509 -0.163930819 0.272143032 -0.092205358
0.47822818 -0.712785622 0.011552945 0.600738766
0.080524156 0.550099582 0.444575546 -0.376087354 -
0.503276534 -0.269670108 0.059010092 0.037014389 0.004905267
0.543341776 0.701968522 0.517198519 -0.51151324 -
0.406274254 -0.140480262 0.473676423 -0.44155345 0.393201843
-0.507384402 -0.144839564 0.563217876 0.009585058 -
0.497675525 -0.40038406 0.300554593 -0.469042771 0.13864301
-0.602337104 -0.001076098 0.574522711 0.045522983 -
0.654235817 0.571175037 0.629640229 -0.490812072 -0.554230774
0.296767685 -0.625658869 -0.181157258 -0.010829893 -
0.470842373 -0.494850025 -0.573845121 0.231436678 -0.489408292
0.251223114 0.328612063 0.457251465 -0.031006439

16444 0.045981644 0.167450323 0.520522036 -0.285042387

16445 0.320791607 0.267537733 -0.300444377 -0.347421446 -

16446 0.635396741 -0.531651654 -0.463364938 -0.118260646 0.447415359

16447 -0.01180892 0.120396915 -0.090336293 -0.510641112

16448 0.360539009 0.280762691 0.244922618 0.200665533 -

16449 0.033718705 -0.004258074 -0.342351395 -0.30426991 -0.374957752

16450 -0.474108942 0.353675209 0.447497328 0.274488328

16451 0.458043767 -0.442599024 0.677556497 -0.484140457

16452 0.309601012 -0.551608267 -0.406365452 -0.64149333

16453 0.38379461 -0.31097658 0.024485636 0.356890194 -

16454 0.043063877 -0.172561081 -0.323365921 -0.190141296 0.504064091

16455 0.63568805 0.437583774 -0.439924143 0.07364277

16456 0.322216088 0.05695019 -0.039941409 -0.344624932 -

16457 0.203789999 -0.083785094 -0.281473123 0.621787457 0.501209892

16458 -0.387416558 -0.520520216 0.609026249 -0.367063425 -

16459 0.434048519 0.363758394 -0.604621046 -0.385172194 0.463813081

16460 0.165040098 0.107770575 0.089098072 -0.35795953

16461 0.10434431 -0.508096602 -0.014180826 0.062519107

16462 0.560974821 0.411963798 -0.27715808 -0.288222454

16463 0.31315778 0.161937748 0.049316097 0.503633739 -

16464 0.020761912 -0.41166585 -0.511044466 0.038230847 0.342104497

16465 -0.439437229 0.704665003 -0.521391777 -0.103111521

0.788826169 0.282524856 0.007880457 -0.052456975
0.267993425 0.140443249 -0.045704758 -0.492395413 -
0.662994569 -0.387112808 0.100252014 -0.072818147 0.152153989
0.502218132 0.090786102 -0.328277224 -0.431649074
0.233789243 0.498613704 -0.334927677 -0.327872434 -
0.393969773 -0.413473809 -0.478482183 -0.379748032 0.406036406
-0.393805807 0.451568792 0.309203736 -0.538833855 -
0.050108106 -0.17967954 0.475437208 -0.233456989 0.387815252
0.472926249 0.275470777 -0.219848238 0.655082205 -
0.328333395 0.525297626 0.451561205 0.573144248 -0.3712057
0.414734356 0.478294641 0.560929311 0.498901898
0.549468554 0.441261113 -0.161393346 -0.017997314 -
0.515940774 0.056794961 -0.567402695 0.580854023 0.344401461
-0.561399581 -0.448706884 0.24047136 -0.653144846
0.163218149 0.094058006 -0.482358214 0.031089732
0.135936823 -0.20601993 -0.086212741 -0.610032226
0.007609592 -0.292759199 -0.398452209 0.678694131 -
0.238401418 0.300409577 -0.075310464 -0.511758752 -0.470599016
0.174042723 -0.656505657 -0.148735332 -0.152611736
0.199252181 -0.303261795 -0.696379723 0.411259717 -
0.171848751 -0.031787119 0.242289348 0.296333 -0.526829438 -
0.417011713 0.405784555 0.500352534 -0.469123865 -0.262191647

16488 0.15753974 -0.551398577 0.584045142 0.187029693

16489 0.536805049 -0.222867723 -0.226183315 0.342700952 -

16490 0.30533389 -0.047207979 -0.307720953 -0.289488066 -0.030157057

16491 -0.331083995 0.019773328 0.551622296 0.109178653

16492 0.397532211 0.673809877 0.164907626 -0.284378481 -

16493 0.265335272 0.087107767 0.001661199 -0.520491156 0.062982942

16494 -0.478397823 -0.507582936 0.102472 0.237623564 -0.244613256

16495 0.583205178 -0.143863857 -0.218730759 -0.576578918

16496 0.167035995 0.265806652 0.284624548 0.363698046 -

16497 0.47975926 0.47954221 -0.2447679 0.251523233 -0.575229525

16498 0.420677985 0.294051294 0.185349866 -0.518636425

16499 0.127231051 -0.255319167 -0.064104648 0.057576514

16500 0.470279444 0.26359197 -0.200711561 -0.244572227

16501 0.066161307 0.168056461 -0.347166407 -0.129815663 -

16502 0.355923335 0.224183411 0.168550127 0.220427774 -0.22916892

16503 0.471910764 0.210044584 -0.298570253 0.313378139

16504 0.574871352 -0.631860923 -0.365283363 -0.275202784 -

16505 0.350400342 0.122365676 0.072216014 -0.601788085 -0.405770958

16506 -0.37973915 -0.107280586 0.280905385 -0.464103192 -

16507 0.059298191 -0.203292504 -0.70720164 -0.450198866 0.197449543

16508 0.011261456 0.475429968 -0.56765183 -0.130382126

16509 0.166399191 -0.45301825 -0.354555664 0.01673514

16510 0.114208156 -0.138042515 0.610996661 0.374676226

16511 0.410496015 0.146952104 0.309996742 -0.192026496

16512 0.555290588 -0.261907231 0.122241994 0.522504154 -

16513 0.300926108 -0.601135965 0.430113951 0.330673866 -0.545280906

16514 -0.5045655 0.246301529 0.361095407 0.099124112 0.56727139

16515 -0.402700692 -0.527766759 -0.367096079 -0.32931933 -

16516 0.557912624 0.131401732 -0.459168673 -0.513009023 -0.063766132

16517 0.038173426 -0.521943623 0.421099077 -0.629849874 -

16518 0.568799786 0.156392676 0.610489614 -0.434948932 0.017454507

16519 0.048197806 -0.13076101 -0.382706543 0.407851327 -

16520 0.269191557 0.055817242 -0.100212943 0.099909092 -0.368739403

16521 0.164989616 -0.426964486 -0.301797443 0.063837159 -

16522 0.004984206 -0.368406691 -0.291948879 -0.33223592 -0.184939439

16523 -0.177420269 0.072254658 0.244222264 -0.539435479 -

16524 0.241318845 0.191139898 0.269445854 0.465888469 -0.515511846

16525 0.614929367 0.26601008 -0.281214215 0.625994013 -

16526 0.057204603 -0.325676479 -0.628580118 -0.405599002 0.296386933

16527 -0.090623445 0.063369552 -0.395218111 0.136169221

16528 0.428930334 0.362034457 0.16052759 0.221905819

16529 0.176897739 0.413354386 0.007322044 -0.211540207 -

16530 0.329233381 0.447082348 0.258991794 0.088375175 -0.025244863

16531 -0.616224451 0.150458774 -0.240302716 -0.37168128 -

0.089118855 0.385649627 0.260181425 -0.471448987 -0.086383242
0.128998144 0.195658805 -0.433933152 0.454881097 -
0.164500604 0.506180236 0.461288894 0.352234515 0.676666846
0.549823795 0.06802386 -0.614476773 0.201579521
0.542205516 0.004105806 -0.231034302 -0.668760965 -
0.573776033 -0.196353826 0.276046706 0.28715113-0.617708627
0.513412452 0.235377154 -0.004792481 -0.355871989 -
0.546774777 0.397683826 0.307088856 -0.309652527 0.399975597
-0.140557282 -0.000827931 0.08968797 0.563863844 -
0.142578021 -0.304437019 0.179116056 0.271817585 0.549876621
0.544311173 0.284711741 -0.529048746 -0.21835054 -
0.226544453 -0.17588936 0.255801295 -0.369804978 0.184006105
-0.221897886
KEGG_PROTEIN_EXPORT 0.544401415 0.326681199 0.074655773
-0.091063909 -0.332629039 -0.405356351 0.468162475
0.016458638 0.371819101 0.030053881 -0.385762587
0.495943346 -0.20522101 0.010888279 -0.606978566 -
0.199933503 0.449223546 -0.284001893 -0.691672523 0.15991329
-0.247986749 0.350782802 -0.437625283 0.299993736
0.129457628 -0.093788594 0.097138301 0.222019784 -
0.361671134 -0.396412049 0.039166706 -0.243257829 -0.162725745
-0.35462064 0.030772381 -0.332886751 0.052067104

0.094692587 -0.367851136 0.031565668 -0.11790899
0.031281195 -0.174252554 0.38241259 0.503242834
0.260715749 0.021988497 0.028964471 -0.312579201 -
0.370013229 0.44372359 0.324553083 0.330013201 -0.230929983
-0.310119732 0.581546445 -0.720612273 0.168596202 -
0.121299769 -0.210503741 0.193854071 -0.316594727 0.39123138
0.065444924 -0.196828588 -0.54385231 0.403623628
0.020109582 0.30647791 -0.614011187 0.157139581 -
0.319139739 -0.251470602 0.510513951 -0.419873528 -0.203420003
0.171164963 0.3842706 -0.24773377 0.493458002 -0.438892436
-0.464737703 0.517113986 0.149456473 0.538363383 -
0.07230461 0.011749582 -0.377208218 -0.669368722 0.15655489
-0.332403823 0.025900632 0.173025863 0.582972285
0.485161102 -0.32333801 0.185756395 -0.367335247
0.242647262 -0.143868878 -0.133597868 -0.348520264
0.565588198 0.18903176 0.022230448 -0.275744302 -
0.079818448 0.40444759 -0.511878006 -0.378299555 -0.623307014
0.202510928 0.202946194 0.366217638 -0.400829315
0.474011581 0.463147316 -0.559350788 -0.699694428
0.396011731 -0.622767754 -0.446096849 0.073058168 -
0.224944018 -0.342948626 -0.555864928 0.307839478 -0.570033479
0.118279547 0.168138407 -0.194218462 -0.431039835

0.257050572 0.023251507 0.225708458 -0.458294449
0.004834286 0.198732489 -0.314264147 -0.172527763 -
0.535564745 -0.054085121 -0.154705482 0.174573015 0.649124153
0.442292346 -0.022251035 0.131905281 -0.569895584
0.448771597 -0.463906846 0.208126558 0.019878668 -
0.367722682 0.173732094 -0.400363437 0.304450359 -0.231989016
-0.013956564 0.078960434 0.452762774 -0.095061062
0.237169395 -0.390032762 0.502008371 0.228695487 -
0.371179319 -0.492908352 -0.095678386 -0.507139765 0.459168187
-0.279625912 -0.256579974 0.208252621 0.144154009
0.038562045 -0.034017615 0.103454122 0.436370828 -
0.03529043 0.172143494 0.182953067 -0.174402895 0.470226504
-0.191960726 -0.060311434 -0.07982063 0.503875594 -
0.410385409 0.317940032 0.50440333 0.373395251 -0.123229222
-0.44096047 0.605379939 -0.244265641 -0.289877191
0.548749683 -0.350032116 0.179196169 0.356984 0.308293873
0.224118555 0.151874009 0.022046788 -0.418499944 -
0.279730341 0.42872288 0.020190776 0.562660848 0.290219279
-0.286956165 0.282965995 0.187487928 -0.390500758
0.022940763 0.638492983 0.118626212 -0.51592718 -
0.476441002 -0.320181141 0.051967961 -0.324085786 0.208701085
-0.516292317 -0.053646963 0.373927453 -0.002381321

0.608308286 -0.355189858 0.045416147 0.434679754
0.407024023 0.303214225 -0.445558057 -0.329432451 -
0.483643555 0.33623034 -0.047014529 0.242632907 0.378052807
-0.309305754 -0.429720569 0.134166023 0.171032358
0.265191845 0.105386496 -0.20940228 -0.587896718 -
0.380868672 -0.079113576 0.4388683 -0.639749964 -0.096419219 -
0.098020666 -0.497081632 0.089004887 -0.347579665 0.543407156
-0.043118027 -0.407404959 0.496967526 0.152929469 -
0.588742933 0.572482967 -0.058574233 0.285131712 0.429975868
0.45204513 -0.588069681 0.428580691 0.485056852
0.084005166 0.321993507 0.398323963 0.282201853 -
0.271011976 0.07644473 -0.178848026 -0.395734046 -0.75522091
0.239935761 0.418886953 -0.648854315 -0.575192615
0.025365053 -0.659989264 -0.126731424 -0.068448819 -
0.361481128 -0.023861379 -0.395922356 0.414900607 -0.282614154
-0.45615127 -0.104089564 -0.107024928 -0.323986539
0.425992624 -0.382284269 0.355067323 -0.301199288 -
0.609471525 -0.480269342 0.140986635 -0.452008968 -0.143896044
0.098668588 -0.091352097 -0.619373229 -0.441323316
0.10753114-0.671632376 -0.009329177 0.015114810.49536333 -
0.488661975 -0.18120384 0.398178743 0.601621919 -0.115085088
-0.186529736 -0.019186546 -0.606303732 0.422375345 -

0.214868674 0.462080417 -0.537431903 0.023747909 -0.393182518
-0.339815269 0.27731931 -0.08924116 -0.085633056 -
0.183822525 -0.087593731 0.202031187 0.289148663 -0.29878541
0.4145195 0.437176991 0.598491285 -0.542717626 -0.012886115
0.26292733 0.249206334 -0.332444698 0.539191347 -
0.483082002 -0.395056569 -0.234068329 -0.204924938 -0.096481846
0.575686429 0.288087078 -0.478887111 -0.539381466
0.429463526 0.057846619 0.298884241 0.427693009 -
0.279270145 0.206069936 -0.536676222 0.384991345 -0.586178733
-0.037460597 0.174716739 -0.020678562 -0.271050478 -
0.39946296 -0.145359925 0.382469885 -0.01840846 0.609613612
-0.358344441 0.016242659 -0.333251373 -0.301594892 -
0.204327238 -0.180807051 0.055939286 -0.456490538 -0.371119324
0.141926012 0.288446824 0.505026332 0.310318545 -
0.051963731 0.149700559 0.050401674 0.392005726 -0.067849391
-0.33197468 -0.146484451 -0.315045746 0.379741076 -
0.141471392 -0.564380439 0.157796208 -0.47049103 -0.053446691
0.435763979 -0.020351648 -0.277403054 -0.177687806 -
0.504256943 -0.271872761 0.279627942 -0.046120656 0.58120886
-0.549176447 0.003557783 -0.253022722 -0.389733732 -
0.325364828 0.088591979 0.452752424 -0.535109267 0.546634095
0.326526871 0.45690239 0.298755701 0.103512636 -

0.451453024 0.365864204 -0.329841605 -0.501832456 0.26996403
0.317760675 -0.458225034 0.422403656 -0.070488022 -
0.39167366 -0.320728022 -0.192647169 0.183445341 0.201291107
0.397857514 -0.052268536 -0.431112639 0.094518269
0.147211766 -0.590664897 0.103209744 -0.313634099
0.106480641 -0.167468029 0.324876903 -0.441541554
0.386409948 -0.51876489 0.123131926 -0.074629403
0.603755467 0.348887626 0.05290506 -0.250353189
0.298813381 0.145595848 0.057043787 -0.597776982 -
0.670423071 0.024029682 -0.283949965 -0.246451249 0.050696723
-0.441212546 0.083204513 0.108247681 0.445005707 -
0.323266962 -0.053538677 -0.332522886 -0.01412584 0.013916748
-0.004658484 0.125849264 -0.52738956 0.025377743
0.219810586 0.039199246 0.562620964 -0.351470837
0.535727442 0.465439217 0.266571186 0.479172061 -
0.096122726 0.023971476 -0.394850564 -0.365726588 0.401273367
-0.349614016 0.392398464 -0.35974213 -0.078548999 -
0.104749327 0.30947811-0.035184563 0.144021003 0.455839081
0.312608792 -0.323045839 -0.278320402 -0.343495817 -
0.256683982 0.08371764 -0.524557379 -0.219535145 -0.488710253
0.191373179 -0.481536519 -0.57630266 0.142943279
0.389647411 -0.158253054 -0.269074564 0.36621634

0.331784494 0.4669806 -0.435869496 0.142812327 0.232410433
0.58150247 0.448907686 0.586413654 0.388466946
0.347966854 -0.011739394 -0.400764841 0.515511480.4229055 -
0.54274517 0.049078872 -0.456523236 -0.337819909 -0.325509586
-0.125729046 -0.021253821 -0.102986406 0.041281862
0.087994033 -0.081568761 -0.217643321 -0.383362781 -
0.128591366 0.260694113 -0.216199876 0.014799397 0.092484096
0.171071047 0.148099257 -0.130264058 -0.063291613 -
0.311230813 0.528076695 -0.330601958 0.22860879 0.46016522
0.293718039 -0.164159179 0.29965266 -0.00966024 -
0.29187129 0.065825617 0.175537974 0.002224169 -0.348990826
KEGG_PPAR_SIGNALING_PATHWAY 0.363666697 0.307768776 -
0.340783931 0.011272757 0.310359236 0.159493857 0.320789145
-0.132528603 -0.381758687 -0.287820496 -0.267707856 -
0.071124027 -0.090034485 -0.279559863 -0.051728466 -0.190846058
-0.071859946 0.066296421 -0.307326787 -0.027925414 -
0.191150255 0.300624121 -0.392006448 -0.251481273 -0.086011293
0.124089852 -0.031546139 0.167925236 -0.21831037 -
0.039155681 0.381194826 -0.138428215 0.129126839 0.315782244
-0.240816702 -0.094936625 0.251169516 0.217878528 -
0.124143325 0.342233237 0.141703146 0.251206784 -0.268438526
0.330587104 -0.364138277 0.165076482 -0.11887745

0.015528113 0.019640302 0.155090554 0.231358561 -
0.297284488 0.318064239 0.147900685 0.119579478 0.17424669
-0.100406406 0.057191375 0.349754704 0.166693651 -
0.436642215 -0.007049044 0.046120453 0.094867502 0.023368131
-0.214131562 -0.074863249 -0.105293026 0.074665018 -
0.084179866 -0.409316474 -0.362286015 0.089496386 -0.124274252
-0.019788361 0.114018833 0.246037373 0.077685723
0.263614892 0.397896113 0.144621291 0.085250769
0.08357681 -0.001392578 0.045933654 -0.309320691 -
0.288999888 -0.286762859 -0.155982209 0.341529707 -0.359616861
0.046718413 0.162786798 0.26642108 0.194788659 -
0.114179646 0.24099504 -0.369387889 0.432458955 0.098859353
0.109044713 0.03812453 0.037482031 0.201540551 -
0.183129096 -0.262194281 0.156325686 0.142383542 -0.330550747
0.06217589 0.117183547 0.09309595 0.261942735 -
0.389252106 0.296457395 -0.159816606 0.181374592 0.071625041
0.156757117 -0.146484418 0.256880144 0.21360511-0.029650738
0.039266944 0.050342154 0.335560855 0.069431639 -
0.468856607 0.367258994 0.204826482 -0.076558628 -0.24822941
0.015983973 0.249946343 0.041532115 -0.293495295 -
0.404351836 0.291243748 -0.055929903 -0.171097192 -0.117873548
0.262854017 0.053570303 -0.026151369 -0.323392389 -

0.331727553 0.304711166 0.379561352 0.146806432 0.255783781
0.187779539 -0.305842737 -0.261326294 0.081627902
0.031190722 0.210905043 -0.452440814 0.503185206
0.211201537 -0.1853995 -0.321643151 0.374746277 -0.171803235
-0.093974542 0.299549735 -0.095107876 0.303321165
0.01338184 0.146638485 -0.282655926 0.098750223
0.334494087 0.245128957 0.320551141 -0.100789601 -
0.362945423 -0.345505274 0.2262752 -0.023339795 -0.220234406
0.303633264 0.188800678 0.171003422 0.268755492 -
0.010135282 0.201505325 -0.348235531 -0.219126319 -0.343756542
0.310347065 -0.300688247 0.381054661 0.240133749
0.036726824 0.182437153 0.14985818 0.125585761
0.121383761 0.006475129 0.162816765 -0.100243696
0.105630748 0.134034452 0.556248993 -0.181050717
0.152732733 -0.192837128 0.06496914 -0.350842026
0.141666815 0.396528154 -0.045149307 0.10505758 -
0.329050279 0.263229597 -0.012598254 -0.030905693 -0.111065487
0.107216755 -0.407302721 -0.058700371 0.274974101
0.251855709 -0.364609983 -0.362085619 0.137198902 -
0.259255403 0.004288576 0.17870926 0.412447446 0.065828622
-0.139723845 0.111477843 0.065220125 -0.002174145
0.294610427 -0.345829849 -0.128599424 -0.019722137

0.001412099 -0.055735104 -0.32248414 -0.053956605 -
0.130934231 -0.450123085 -0.012851374 0.063476109 -0.149456382
0.109586589 0.217037045 0.172015889 0.044449922 -
0.0923418 -0.4822857 0.043331304 -0.211316117 0.439286524
0.25622243 0.223184744 0.282102698 -0.42416827
0.183595147 0.131280943 0.146532565 0.086614971 -
0.207218243 -0.10675551 0.024482993 0.030598691 0.201886425
0.427327945 -0.465965987 -0.325594956 0.002198341 -
0.078568233 0.281236592 0.129351503 0.354579174 0.071468876
0.027091972 -0.156502919 -0.178211815 -0.261110051
0.097232497 -0.248096949 0.207425866 0.027050368 -
0.113171363 0.038915802 0.326855098 0.231248319 0.322083702
-0.164669019 -0.435528214 -0.135497773 0.081957287
0.100784071 0.096560699 -0.367587148 0.276541149 -
0.146008636 0.173695657 0.351107998 0.096151836 0.42182771
-0.122808326 -0.15203188 0.448003417 -0.232804814 -
0.143793088 -0.267341928 0.159104011 -0.328041097 -0.136979352
0.218276015 -0.00362267 0.021637431 0.214690796
0.229699567 -0.002219413 0.226704666 0.297272729
0.179618454 0.217095669 -0.144900332 0.17080207
0.046342994 0.183218426 0.24380361 0.104716199 -
0.266393788 0.21872961 -0.176742383 0.454207688 0.056009688

0.416840927 0.301458489 -0.190791696 -0.136120917
0.319589304 0.135187598 0.009923668 -0.166426128 -
0.127168102 0.235698481 0.204407745 -0.312295371 0.351231989
0.268783551 -0.409707505 -0.30803375 -0.260883397 -
0.274660765 0.403135705 0.153600908 0.062963153 0.216133455
0.195159365 0.198258048 0.230930514 0.125833268
0.216596655 0.307185784 -0.128330697 0.236756118 -
0.105534045 -0.204822018 -0.297732193 -0.045723297 -0.209095274
-0.306579629 -0.435138757 0.158910378 0.258031065
0.26499425 -0.190639031 -0.316969145 0.215912587 -
0.168377224 -0.157926961 -0.169901677 -0.223909499 0.166553362
-0.300292504 -0.239470107 0.312549914 -0.069156502 -
0.333047781 -0.056788977 -0.158845668 0.266330119 -0.078478161
0.123649655 0.332938007 -0.110148029 -0.075828257
0.321874437 -0.367449249 -0.303059998 0.278678456 -
0.006482617 0.147827986 -0.066359053 0.28440703 -0.03312064
0.140357421 0.146417938 0.235928722 -0.409519912 -
0.024368045 0.368321004 0.022646058 0.350950202 -0.138222316
0.034012595 -0.134350029 0.009987782 0.270434251
0.182787197 -0.314284816 -0.384492904 0.07207936 -
0.113305674 -0.340609115 0.079630442 -0.337518094 0.315471843
-0.255184392 -0.427302773 -0.094081861 -0.00707241

0.244465604 -0.436727392 0.314337651 0.087455988 -
0.381924482 0.164017445 0.103832066 -0.338817253 -0.235623814
0.154493539 -0.368762967 -0.086022248 0.105939873 -
0.322562113 -0.040145147 0.243866623 -0.435549757 -0.232861781
0.105307078 -0.408664369 0.286802757 0.32684417
0.318268145 0.294915022 -0.149456335 -0.398192486 -
0.382038562 0.063642277 -0.140005119 0.367104806 -0.216667638
-0.190723405 -0.134613073 0.041246017 0.119255042 -
0.257397638 -0.116433931 -0.14308411 -0.085427891 0.032150502
0.134828749 0.03314231 -0.11585907 -0.395437155 -
0.070402378 0.121059954 0.410545514 0.069697834 -0.258228876
-0.187159725 -0.144659275 0.296275924 0.336813566
0.222155899 0.150408837 0.054508257 0.013508102 -
0.314114032 -0.357938339 -0.012946007 -0.115585791 -0.068665265
-0.349525379 -0.251407474 -0.099132852 0.269106705
0.054850224 -0.302442199 0.434453295 -0.230814238
0.247805297 0.413986328 0.005183518 -0.355729466 -
0.035731158 0.009587629 -0.099875298 0.318591199 -0.327233366
0.068897227 -0.009586117 -0.302831151 -0.222165712
0.448689689 0.131187698 -0.135339395 -0.025597298
0.181380829 0.216322092 0.145126876 -0.311226118 -
0.07657816 0.063098829 -0.010106547 -0.380050308 -0.267631717

0.024634671 0.163208689 -0.293655867 0.251680202
0.052526238 0.054357226 -0.050294433 -0.00963921
0.290876374 -0.085980366 0.193076664 -0.253044074 -
0.392977165 0.117054412 -0.248663996 -0.135300145 0.201804714
-0.07472439 0.333401943 0.207398857 -0.353419631 -
0.063818592 0.239873059 0.13789577 -0.016293865 0.140616449
-0.147588638 -0.161929036 0.042267362 0.289303122 -
0.145752921 0.153941915 -0.280488932 0.006403546 0.13357901
-0.290803889 0.06855326 -0.142592623 0.045254883
0.012268567 -0.200311687 0.2962372
KEGG_BASE_EXCISION_REPAIR 0.324925438 -0.274028214
0.122385769 -0.239601108 -0.437033595 -0.265032062 -
0.060961549 -0.191957937 0.45745699 0.360656536 -0.297230064
0.361148946 -0.31665527 0.380086727 -0.267755265
0.36155693 0.158912407 0.113613973 -0.24920197
0.535768351 -0.120796555 -0.442083311 0.264193497
0.176949364 0.038818286 -0.274116677 0.213213917
0.36372806 -0.54167214 -0.425849028 -0.148774398 -
0.043234015 -0.10315832 -0.125333702 -0.314203807 0.077384514
-0.076710183 -0.052822453 -0.281121818 0.140496671 -
0.201323968 0.179774924 0.359294065 -0.110000453 0.357568515
-0.072439783 0.242008621 -0.47844119 0.180355461 -

0.385521047 -0.366685756 -0.128954865 0.203244158 0.327700596
-0.187128922 -0.003970435 -0.248368172 -0.146055382
0.018613265 -0.227811922 0.4500951 -0.11493797 0.194720358
0.379070319 -0.060426021 0.050280156 0.262302865 -
0.164527453 -0.560282253 -0.205540818 0.434416248 0.307198632
-0.438429746 -0.476103483 0.064960374 -0.431820799 -
0.201775828 0.125616442 -0.253663627 -0.180607647 -0.009240485
-0.374246139 -0.130806981 -0.147673782 0.18953961
0.584240683 0.140341758 -0.086926137 0.058894492
0.226072882 0.472879659 -0.47301495 0.125242637
0.034194515 0.216127073 0.185840362 -0.388366758
0.026544275 -0.01288971 -0.36824208 0.097024271 -
0.498737898 -0.34274671 0.240809005 0.200264546 0.351569951
-0.20545925 0.086384957 -0.10359925 -0.350487424 -
0.656710987 0.364223064 0.398282218 0.151931214 -0.275862876
0.113844304 0.417288033 -0.366821727 -0.21595492
0.310136495 -0.356016347 -0.013813085 0.485822046 -
0.30550161 -0.242838751 -0.507671649 0.189409319 0.224765883
-0.131502304 0.056758491 0.335500996 -0.312582264
0.318836961 -0.005387131 -0.019161059 -0.27104058
0.293955553 0.358420467 0.272756007 -0.162996691 -
0.284845658 -0.27603125 0.24155102 0.472857023 -0.152711895

-0.048966201 -0.158473433 -0.112126642 -0.456801693
0.038165953 -0.391119152 0.321260935 -0.285864434 -
0.329479634 0.080124743 -0.370577543 -0.215644077 -0.140770522
0.321305456 -0.115184858 0.199866256 0.191462514
0.215417817 -0.274208905 0.161931586 -0.170195947 -
0.286972525 -0.311590903 -0.38557209 -0.500462669 0.409271848
-0.215659477 -0.158484603 -0.111886835 -0.105335975
0.12708489 -0.362030961 0.217581065 0.35544447
0.269495207 -0.027396792 -0.365759509 0.158432052 -
0.013745143 0.073203517 -0.090858832 0.309820859 -0.42024718
-0.188634996 -0.41512114 0.285408924 -0.098915375 -
0.369938853 0.055212436 0.427855245 -0.336859184 -0.364387398
0.131189169 -0.106142798 -0.284741997 -0.30766691
0.097856699 -0.364791226 -0.193424648 0.294107143
0.133737938 0.093578545 0.341041859 -0.18394437 -
0.04982187 0.095705964 0.374580338 -0.134929444 0.283477348
-0.294870682 0.505039461 -0.054096425 -0.375029393 -
0.010154362 0.036333124 -0.407161431 0.330872228 -0.493607984
0.63914981 -0.359446112 -0.345944175 0.311347295
0.020726839 -0.055844744 -0.259379202 0.402089426 -
0.208216836 -0.283418288 -0.270963188 -0.257729334 -0.266593426
-0.190091509 -0.226453779 -0.254620431 0.158160437

0.350805869 0.244724458 0.053531249 -0.026664994
0.559252178 -0.366167371 0.140552602 -0.144457644
0.227066923 -0.323810019 -0.465920446 -0.067446722 -
0.082160633 0.295646307 0.030160399 -0.413383442 -0.100713962
-0.248402432 0.129851919 -0.33072332 0.56505505
0.057566835 0.356306776 0.01660022 0.150189154 -
0.228696674 -0.4461541 -0.019866062 -0.083708586 -0.527151996
0.129183174 0.520150308 0.265875036 -0.443451641
0.504017743 0.210683575 0.133469561 0.318766110.222568476
0.131672893 -0.362183727 0.127518825 0.389089641 -
0.593492456 -0.440305028 -0.327926769 -0.343709289 0.020812263
-0.095303873 -0.328416114 0.028628115 -0.402476927
0.233520915 -0.251180847 -0.502909457 -0.362347596
0.057141005 0.147243837 0.532815658 -0.42565868 -
0.036539691 -0.211746854 -0.38323306 -0.062141748 -0.047728177
-0.307031153 -0.10159947 0.177354509 -0.12198084 -
0.268729809 0.169354579 0.423667472 -0.313330072 0.087029594
-0.053189562 0.274684479 -0.367164898 0.125589753
0.375668804 0.181752416 -0.433241005 0.171199512
0.040799427 -0.42193238 0.508357697 -0.450030978
0.241035985 0.004971403 -0.046323384 0.358662683
0.318244246 0.185480789 0.004524012 -0.326165963 -

16884 0.046002845 0.126915595 0.19473072 0.260529503 0.208735625

16885 -0.101074707 -0.037437891 0.079152712 -0.138927332 -

16886 0.220765322 0.244844665 0.171621293 -0.154019017 -0.173461704

16887 -0.08587451 0.470330615 0.363559409 -0.118618048

16888 0.341774015 0.173182339 0.268295179 -0.333309278

16889 0.015156044 -0.301122188 0.485142093 -0.162403599

16890 0.018769501 -0.524732832 0.04233589 -0.179604987 -

16891 0.063852354 -0.259864545 0.596718539 0.369633254 0.035439505

16892 0.018923706 0.252769321 0.124492275 -0.202712621 -

16893 0.216543023 0.060264245 0.33777691 0.12653787 0.47953506

16894 -0.205012232 0.287685961 -0.019014592 0.164736202

16895 0.305980066 0.135474743 0.189182301 -0.3800044 -0.422008611

16896 -0.089572066 0.413946069 -0.010370463 0.307797269 -

16897 0.038133767 -0.101865771 -0.454727362 -0.316824292 -0.266508478

16898 -0.268697659 0.460005656 -0.410964142 -0.32032059 -

16899 0.290868601 -0.411108257 -0.141535858 0.196606732 -0.233300117

16900 0.224918282 -0.127252271 -0.111265165 -0.077027755 -

16901 0.171153683 0.327244253 -0.093050561 0.039909231 -0.052408035

16902 -0.113132998 -0.132570685 -0.188889779 0.236358575 -

16903 0.267088728 0.013757264 0.358082975 -0.171389485 0.128266935

16904 0.162428497 -0.48016252 0.281109381 -0.342964743 -

16905 0.073942405 0.292901941 0.074889145 -0.121875176 -0.291875684

-0.396914003 -0.378691095 -0.24022097 0.196119252 -
0.048390928 -0.145317458 0.503187354 -0.062020751 0.228435099
-0.104155542 0.151442412 -0.521637625 0.169381423 -
0.061328628 -0.511046871 -0.21950595 -0.127634955 0.111000104
0.386749678 -0.365614546 -0.129763826 -0.401479448
0.106275747 0.254675713 0.298204313 -0.029590046 -
0.336015379 -0.020007847 0.017181985 0.283236772 -0.00527837
-0.389097724 0.430295101 -0.183846134 0.315327638 -
0.29063879 0.025907125 -0.189385432 -0.231542048 -0.320058778
-0.094692411 -0.075906181 0.314131882 0.45553014 -
0.181278266 -0.113091155 0.209946555 -0.367247424 0.333667945
0.271742813 -0.003071208 -0.387894069 -0.248397907 -
0.192132821 -0.258397276 0.267375965 0.002802883 0.144940739
-0.252878437 -0.498731139 0.210403023 0.341422838 -
0.453226453 0.24465227 -0.070853517 -0.051430548 0.420535778
-0.254415762 0.392021477 -0.10506273 -0.230409302 -
0.409770425 -0.186562146 0.059062236 0.378928512 0.132813999
-0.252339074 0.0919786 -0.153856954 0.044269956 -0.251780726
-0.232207708 0.123910235 -0.117921738 0.1456004 0.518828623
-0.184092501 0.134650703 -0.015220423 0.153290102
0.08716661 -0.175624735 0.218948271 0.255686585
0.207448433 0.213896821 -0.405570861 -0.151573052 -

0.414303343 0.152240517 0.307422267 -0.271075282 0.336475061
-0.375500659 -0.401004562 0.070737702 0.184205808
0.14485586 -0.366606426 0.323178491 -0.29082034 -
0.449174885 0.035330267 -0.278188175 0.302305482 0.331510868
0.323908803 0.128502239 -0.470539416 -0.134815614
0.247661481 -0.352694086 0.324433985 0.354638744 -
0.133284709 0.105258473 -0.050013676 0.079290024 0.023180677
-0.470317568 -0.12815296 0.316589839 -0.250826219
0.319975535 -0.233571425 -0.355484419 -0.058957249
KEGG_NUCLEOTIDE_EXCISION_REPAIR 0.306249241 -0.300064771
0.125367987 -0.247510716 -0.403046323 0.252166647
0.096830968 -0.414084735 0.469819428 -0.221140369 -
0.014272733 0.227177862 -0.20822227 0.389607947 -0.360414089
0.009859675 0.198276881 -0.215801835 -0.51748854
0.394998503 -0.03768659 -0.314763272 -0.062629647
0.365789546 -0.26138656 0.01469409 0.29250646
0.234311893 -0.045951295 -0.409606452 0.090752223 -
0.254955899 0.134480274 -0.366552334 -0.212172489 0.253932916
-0.28282249 -0.550154292 -0.255265404 -0.030639287 -
0.291477984 -0.260454831 0.208724898 0.067386861 0.177824866
0.029201235 -0.022071694 -0.196540535 0.29087383 -
0.397750373 -0.132034566 0.246583337 0.306721609 -0.111731663

16950 0.155563556 0.240759254 -0.395603389 -0.147034405

16951 0.207700111 -0.217167815 0.457596798 -0.268392574

16952 0.509581903 0.222544285 -0.007763532 0.160115517 -

16953 0.05429795 -0.29781857 -0.386370471 -0.261791771 0.351259572

16954 0.080952165 -0.302299277 -0.078083759 0.101187772 -

16955 0.450179142 0.094300265 0.472034592 -0.390403766 -0.077333138

16956 -0.294234161 -0.194665452 0.156632577 0.090181883

16957 0.122951734 0.445997878 0.272061217 -0.39740185 -

16958 0.261042815 0.255327552 0.221659989 -0.278496817 0.02805332

16959 0.225531467 0.032977924 -0.099029075 -0.332475783 -

16960 0.227839929 0.0944271 -0.359015332 -0.04353986 -0.334964103

16961 0.242072525 -0.036076307 0.149874956 0.190633167 -

16962 0.098510236 -0.165726102 -0.169416964 -0.48868767 -0.583425496

16963 0.164371278 0.273476322 0.156193798 0.033672625

16964 0.265085686 0.38704461 -0.203165405 -0.179348259

16965 0.201994168 -0.470656834 0.235407338 0.305742736 -

16966 0.427931918 -0.194696215 -0.523756121 0.220180745 0.12072451

16967 0.115858327 0.199158274 0.187816934 -0.385981978

16968 0.343028889 -0.018945454 0.128615375 -0.291915273

16969 0.079275305 0.397911456 0.447844248 -0.223460956 -

16970 0.065135664 -0.220484159 0.294426865 0.382937809 0.283165962

16971 0.234844873 -0.083362933 -0.052112163 -0.466006542 -

0.116168274 -0.450241868 0.15924757 -0.240662066 -0.469523816
0.242169477 -0.334294502 0.025123829 -0.182864891
0.421643467 -0.36808128 0.249339917 0.189957982
0.236760118 -0.204236019 0.289427569 -0.053189558 -
0.184092129 -0.151352953 -0.478168528 -0.224196872 0.444915334
-0.220421314 -0.361994632 0.020190296 -0.330875674
0.326192845 0.199932819 0.294385944 0.309523112 -
0.017212001 -0.375925981 -0.354461078 0.051264299 -0.036169001
-0.028501438 0.06064597 0.330537864 0.091635928 -
0.181562248 -0.093373559 0.427214268 -0.019583467 -0.238272809
-0.157301995 0.426906456 -0.316475009 0.123827595
0.327618862 0.137635575 -0.075142818 -0.366235288
0.089367057 -0.404491559 -0.204491705 0.097240166
0.085041013 -0.10877747 0.459818056 -0.051790719
0.064700748 0.277431092 0.427980829 -0.193564473
0.361855447 -0.426190974 0.370389093 0.284670971 -
0.1927734 0.35698488 0.056365403 -0.486887609 0.127239722 -
0.426702761 0.588006221 -0.164040097 -6.19E-05 0.272843376 -
0.215074701 -0.036462049 -0.433669012 0.332546743 0.156152348
0.207346681 -0.181649269 -0.29111053 -0.193910789 -
0.21286248 0.027661289 0.007501697 0.008064406 -0.36891749
0.331844296 0.325592645 0.211209184 0.468393583

16994 0.22025864 0.003129213 -0.074267684 0.075776476 -

16995 0.391168463 -0.142912519 0.036006781 -0.32803547 0.3136946 -

16996 0.090275006 -0.412101275 0.146393563 -0.42429991 0.30714026

16997 -0.356921467 0.354769508 -0.004011319 0.400040867 -

16998 0.392461027 0.198686452 0.069076014 -0.426115123 0.084275687

16999 -0.149187352 -0.496522242 0.305360126 0.551864293 -

17000 0.072507952 0.160470006 0.450595717 -0.279787588 0.350319734

17001 -0.136795021 0.059158104 0.040625642 -0.416824675 -

17002 0.009405766 0.270507704 -0.58608611 -0.474963523 -0.308226687

17003 -0.490583824 0.110533182 0.009428605 -0.28817459

17004 0.005335155 -0.48388566 0.246470161 -0.068481354 -

17005 0.464147431 -0.53818806 0.172955639 -0.071741739 0.205697415

17006 -0.489098113 0.129173746 -0.478674129 -0.568859927 -

17007 0.269985783 -0.150707202 -0.07390465 -0.08043134 0.208187835

17008 -0.317112535 -0.309488345 0.073264755 0.061688086 -

17009 0.498385572 0.370101874 0.063935072 0.273351968 -0.448489463

17010 0.032487911 0.299556738 0.263050531 -0.471900518 -

17011 0.049120428 0.281242976 -0.574070467 0.297411317 -0.449193755

17012 0.223950066 -0.279578972 0.155208016 0.454465343

17013 0.121540739 0.28189183 0.151755537 -0.371207497

17014 0.161514134 0.29280222 0.34583252 0.001062869

17015 0.111349717 -0.163790428 0.291899541 0.442035548 -

17016 0.330115189 -0.160095272 0.321101376 0.1393174 0.132565617 -

17017 0.273945116 -0.286081933 0.421934422 0.07882299 0.376140515

17018 0.220058434 0.272370344 0.289680075 -0.440320996 -

17019 0.400715672 0.022206872 0.466387612 -0.251792638 -0.023263921

17020 -0.423764722 0.339728447 -0.119024589 0.409339009 -

17021 0.283010424 0.310165717 0.308689017 0.183582611 0.007533402

17022 -0.044934774 -0.037944533 -0.068247938 -0.054734606

17023 0.201204224 0.061400021 -0.137317094 0.372123619 -

17024 0.228523494 0.350029442 -0.293677558 -0.173646704 0.040440336

17025 -0.244098087 0.277936808 -0.085763516 0.007639347

17026 0.153562684 0.399496047 0.450058692 0.112230532

17027 0.031732729 -0.286017578 -0.477514805 -0.134795719 -

17028 0.275664356 -0.34067196 0.231786603 -0.122225837 -0.157017368

17029 -0.10122541 -0.153632038 0.285687963 0.301605315 -

17030 0.165283623 0.138657854 -0.049459664 0.202523878 0.096786974

17031 0.340539125 0.468983158 0.018440544 -0.405275324 -

17032 0.361941724 -0.221091086 -0.044437662 -0.003521877 0.270849875

17033 -0.290493921 0.213654251 0.25701327 0.350668697 -

17034 0.027835267 0.084818914 -0.302138882 0.111714377 -0.223633482

17035 -0.019913785 -0.012480418 0.433220891 -0.376495 -0.255894573

17036 -0.30430747 -0.444779676 -0.321858605 0.105597467 -

17037 0.067746566 -0.120255721 0.527517866 -0.115098157 0.031479563

-0.058542337 0.162924324 -0.533187616 -0.117379054 -
0.030467543 -0.448934639 -0.391200537 -0.246219225 -0.35195062
0.458254454 -0.363765184 0.126857524 -0.354872176
0.006568992 -0.061731677 0.30347796 -0.24720906 -
0.100479565 0.098491854 0.112216356 0.150961747 -0.448430702
-0.045561241 0.348565022 -0.193534912 0.324025492 -
0.116873434 0.00541868 -0.186338886 0.269164943 -0.301029241
0.005973823 -0.139141713 0.212983129 0.18393546
0.009324321 0.034639117 -0.006877205 0.205685401
0.443430934 -0.166014597 0.172863972 -0.518997425
0.19377347 0.336465836 -0.046967139 0.286331295
0.145410927 0.369507623 -0.517127127 -0.036545382
0.363985468 0.22889044 0.106921228 0.105997089 -
0.303698458 -0.290484716 0.349759461 -0.434231942 0.302840664
0.124052382 -0.222974718 -0.376008242 -0.218642225
0.162248747 0.020866377 -0.141116234 -0.302736969
0.410429893 -0.082577882 0.112328079 -0.314608693 -
0.269334132 -0.242262128 0.207178214 0.291233636 0.298987838
-0.247958269 0.040581762 0.363416324 0.044419144
0.371625236 -0.327094892 0.476989394 0.171926159
0.206018295 0.177153918 -0.186543152 0.175260605 -
0.281595148 0.149476007 0.239679822 -0.062014581 0.223623905

-0.214170521 -0.220779991 0.278805995 0.023605541 -
0.046132414 -0.172263223 0.095241589 -0.20522181 -0.37034349
-0.219695391 -0.491319888 0.13883235 0.236577037
0.003402006 0.209192589 -0.22576347 -0.114854501
0.019506926 -0.388366157 0.116128519 0.058546006
0.19206546 0.168061015 -0.001680823 0.21233867 -
0.066114721 -0.230958243 -0.030161437 -0.010967311 0.032137892
0.111701132 -0.114776545 -0.19409418 -0.293387182
KEGG_MISMATCH_REPAIR 0.292316726 -0.003297766 0.127404863
-0.383407495 -0.493624645 0.17350534 0.163920602 -
0.348843488 0.716162733 0.145465065 0.067086611 0.185855863
-0.494156634 0.437173567 -0.378431208 0.306179312
0.160453011 0.203598154 -0.600663016 0.480201778 -
0.122989308 -0.241070281 -0.307154253 0.414120112 -0.231286088
-0.346015642 0.242905769 0.585166883 0.118438754 -
0.376308352 0.262398364 -0.063055292 -0.155662994 -0.296699522
-0.15736146 0.217187238 -0.305343955 -0.468724593 -
0.451193615 -0.216259076 -0.329887412 -0.080759105 -0.108885726
0.122414428 0.273394383 0.433625894 -0.165463142 -
0.401179144 0.486767311 -0.421871327 -0.281881616 0.12903817
0.367247944 -0.190151684 -0.013974291 0.551513819 -
0.541498165 -0.370780084 0.05036143 -0.113123957 0.530319915

-0.308583344 0.390683998 0.333986016 -0.239159522

0.094051898 0.103310267 -0.284859021 -0.42519735 -

0.3900809 0.504035622 0.358972938 -0.286757707 -0.017214244

0.158974313 -0.241732435 0.299684669 0.506971103 -

0.128129831 -0.309744042 -0.432163108 -0.264808262 -0.029346123

0.324076295 0.387049283 0.675723183 0.349440749 -

0.159357593 -0.252528951 0.25892865 0.304515472 -0.294136283

-0.325845678 0.380292928 0.141459716 -0.161049743 -

0.365487598 -0.138520202 -0.034418465 -0.470475149 0.316708965

-0.360074131 0.325248636 0.013630493 0.352626669

0.509088474 -0.152136399 -0.141654058 -0.271404375 -

0.610434965 -0.641885039 0.434385669 0.312748125 -0.017878303

0.126562548 0.451975371 0.303529961 -0.370325552 -

0.176832437 0.23828958 -0.587677915 0.366766529 0.364809437

-0.517297668 -0.307719805 -0.55501979 0.120814751

0.280254074 0.027325938 0.259621181 0.377860262 -

0.232475648 -0.095619816 0.13083689 0.293744373 -0.537167684

0.065866406 0.400585098 0.551993025 -0.44074102 -

0.055724836 -0.211513553 0.26196871 0.258368616 0.326839166

0.061685261 0.00120157 0.039133598 -0.42140742 -

0.05094693 -0.425209074 0.574005499 -0.220086702 -0.454239876

0.174402775 -0.130693419 -0.253030128 0.020853622

17104 0.566887447 -0.28355935 0.137466871 0.511328372 -

17105 0.00396913 -0.48709654 0.393917371 -0.045917461 -0.223461312

17106 -0.206916501 -0.388118828 0.101923628 0.532828945 -

17107 0.252226296 -0.56853264 -0.258579968 -0.232703835 0.392393009

17108 0.187469995 0.108859952 0.263736692 0.34441148-0.456584373

17109 -0.354723354 -0.150794028 0.200750248 -0.159391163

17110 0.283195199 0.35726537 0.080263896 -0.240127645 -

17111 0.291760787 0.583119968 -0.149318032 -0.371478896 -0.251494309

17112 0.399435935 -0.067394218 -0.047044271 0.211708519

17113 0.124290323 -0.260402093 -0.507898854 0.501338868 -

17114 0.460232705 -0.140740943 0.362990307 0.364558177 -0.152624757

17115 0.551934705 -0.232014762 0.036806676 0.329839523

17116 0.536136644 -0.366316373 0.536000388 -0.293590782

17117 0.354556914 0.408503787 -0.18885828 0.333307855

17118 0.236695812 -0.398929077 0.189899939 -0.68300053

17119 0.600578436 -0.414685959 -0.22721426 0.371752763

17120 0.221460687 -0.153456843 -0.194292449 0.341547368

17121 0.262903088 -0.367328949 -0.064927482 0.151885575 -

17122 0.19727767 -0.125534999 -0.221732764 0.057572534 -0.35950018

17123 -0.400283897 0.161834101 0.299081896 -0.210735266

17124 0.450793406 0.302922362 0.180879042 -0.236398172

17125 0.080729395 -0.51782924 -0.332960159 0.073236732 -

0.396796026 0.3284307 -0.223746213 -0.393422641 0.175024189 -
0.554140703 0.357513125 -0.518402647 0.497122984 0.151108034
0.261771202 -0.274423602 0.033292329 -0.415386521 -
0.484896327 0.325096234 -0.414139847 -0.455339014 0.22574986
0.641358689 0.250671029 0.03470451 0.247764993 -
0.082249766 0.281218225 0.120777298 -0.140521012 -0.04798811
-0.49051738 0.268101547 0.591598787 -0.632895351 -
0.568546456 -0.433374529 -0.513438106 0.058961821 0.175135963
-0.633577767 -0.171311068 -0.433142814 -0.132128269 -
0.147091031 -0.485558104 -0.580324025 -0.059885358 0.081479331
0.208580625 -0.475048092 -0.030871893 -0.458361873 -
0.628456711 -0.322397104 -0.030484227 0.02558768 0.203300225
0.115114791 -0.255394337 -0.371849367 -0.222285719
0.052107608 -0.555839555 0.323436823 0.232948054
0.357314716 -0.606779254 0.318004936 0.09396865
0.409185597 -0.56071224 0.095278391 0.244882076 -
0.576484506 0.479586724 -0.471738013 0.120842665 0.025695742
-0.116291453 0.538100545 0.390599018 0.220886158
0.309753413 -0.262706536 0.182275757 0.399373799
0.507333225 0.197367614 0.140144195 -0.249097178
0.352762029 0.350499618 -0.326733098 -0.423938711
0.479297718 0.021318251 0.303893707 -0.187545308 -

0.040745058 0.308426195 0.14399358 0.499789583 0.068903674
-0.068256467 0.182505069 -0.584193852 -0.458940844 -
0.024004939 0.376187031 -0.375619007 -0.194271998 -0.532770829
0.168020522 -0.025616441 0.510118186 -0.268696548
0.416812763 0.336050191 -0.252911097 -0.195248449
0.288014162 -0.134913901 -0.218116431 -0.148246378 -
0.101928577 -0.090288575 -0.348648434 0.476062023 -0.214806362
0.299889441 -0.316396982 -0.228659643 0.269681457 -
0.155400068 0.193011416 -0.351465515 -0.085972168 0.190477724
0.506911582 0.543067893 0.254926354 0.166926261 -
0.202438944 -0.21872069 -0.32059159 -0.26994381 -0.612235342
0.449682706 -0.436830408 -0.234882971 0.045906621
0.093130921 0.105596255 0.431672786 -0.072059884
0.374102452 0.087610422 0.170043125 0.309587235
0.283289477 0.332902723 -0.181705365 -0.311639722 -
0.395128978 -0.383254853 -0.269212346 0.105434013 0.32123566
-0.364437101 0.093102232 0.462588936 0.187234878 -
0.125268707 0.207382279 -0.471222477 0.306755998 -0.203767498
-0.014299333 0.274417853 0.57324322 -0.430596137 -
0.172862236 -0.214217956 -0.526442604 -0.345604939 -0.012266799
-0.098378066 -0.162972658 0.696154914 0.037381733
0.191785436 -0.354144874 -0.030616191 -0.664737321 -

0.032480547 0.073964986 -0.562741786 -0.53344032 -0.43813989
-0.120705242 0.593892133 -0.580836192 0.039389122 -
0.293661604 -0.115626444 -0.338840397 0.371604171 -0.421858958
-0.407462821 0.429139193 0.113470109 0.158822468 -
0.291369037 -0.318304032 0.166986508 -0.001174443 0.240306887
0.200820052 0.158819878 -0.473498217 0.349346312 -
0.237009637 -0.173284418 -0.279749544 0.194327327 0.085583025
-0.13221138 0.146353906 -0.047224463 0.055454336
0.229930392 -0.281652731 0.267180692 -0.542758431
0.273231321 0.263042219 -0.023325798 0.298209074
0.422130443 0.452844438 -0.627041157 -0.143170524
0.479040445 0.220154327 0.084620371 -0.024937511 -
0.06882864 -0.176150738 0.37402281 -0.346428834 0.506613041
-0.088074481 -0.427966762 -0.560624115 -0.271871981
0.308253365 0.271949871 -0.25861344 -0.11686977
0.193783684 0.258192371 0.038518354 -0.361516095 -
0.009901877 -0.351406331 0.333733416 0.530490795 0.200033729
-0.43008343 -0.310479892 0.37379711-0.046396747 0.368905534
-0.444898569 0.374586437 0.161065671 0.363581626
0.244166477 -0.210253048 0.110465847 -0.429835355 -
0.030628622 0.345234761 -0.12206699 0.288490478 -0.279036097
-0.368313352 0.329382601 0.245386468 0.444173871 -

0.152239003 0.214014866 -0.288065526 -0.449019256 0.256838772
-0.532457762 0.167533171 0.294032094 0.161331491
0.195838308 -0.492412662 -0.262297383 -0.136836352 -
0.492011936 0.338439923 0.408732069 0.319498814 0.188662588
-0.045039667 0.362995047 -0.21137529 -0.472549936 -
0.176381348 0.214028149 -0.300895323 0.125770608 -0.213746397
-0.259855951 -0.455225365
KEGG_HOMOLOGOUS_RECOMBINATION -0.145517464 -0.278687649
0.287609494 -0.279647242 -0.262094652 -0.2213161 -0.132236888
-0.031295411 0.634892111 0.431377775 0.209576691
0.18985327 -0.391494801 0.171547886 0.261390672
0.442090152 -0.416682035 0.043186337 -0.375866253
0.54151272 -0.102177347 -0.187665945 -0.051485856
0.276428836 -0.067455145 -0.045486495 0.251971238
0.326119330.2240559 0.042883306 -0.18750279 0.232120869 -
0.315820743 0.333799825 -0.364387929 0.303131025 -0.444221876
-0.299728894 -0.192413045 0.1142025 -0.3497605 -0.385920238 -
0.211232884 -0.244016038 0.322218223 0.106982721 0.090063013
-0.384501495 0.349236771 -0.267801583 -0.451918639 -
0.109358385 -0.033325757 0.251840395 -0.20172725 0.115613086
-0.180179934 -0.466425431 -0.125715143 -0.306336633
0.458500998 -0.271490086 -0.309143584 0.325407354 -

0.08369293 0.134608445 0.064552085 -0.60176668 -0.579258838
0.16930755 0.53085551 0.447546913 -0.301789384 -
0.1742898 0.297192617 0.175722702 0.355360616 0.187382138 -
0.153528776 -0.350061835 -0.19141352 -0.370106684 -0.34697886
-0.252007166 0.378501782 0.672840643 -0.043425909
0.325957695 0.341630938 0.189841911 0.426770691 -
0.450717674 -0.188114385 -0.189767114 0.408172501 0.130948108
-0.499475068 0.39386973 -0.358653878 -0.66249845
0.382353682 -0.060840466 0.03611136-0.017901456 0.447170016
0.522576759 -0.359640635 -0.300657017 -0.152201687 -
0.363220481 -0.571143001 0.2959366 -0.013266771 -0.337215812
0.125073603 0.438322347 0.228992096 0.215546796
0.205728399 -0.044552647 -0.152774563 0.082751085
0.469107766 -0.41836324 -0.211121034 -0.2429252 0.004679917
0.564803909 0.125452546 0.041771264 0.470587302
0.401265695 -0.072300298 0.16195555 0.021550787 -
0.358974757 -0.109584009 -0.067559018 0.440044002 -0.089719081
0.414529181 -0.141832067 -0.042047311 0.236445072
0.021077293 -0.344826924 -0.129026609 0.085852524 -
0.06631774 -0.053992066 -0.207489011 0.445881194 -0.066040564
-0.419017628 -0.2024431-0.342391788 0.150925989 0.110693359
0.539554018 0.027972373 -0.379629377 0.359420758

0.361658504 -0.349103526 -0.270262513 0.070959423 -
0.513400512 -0.310529941 -0.17603645 0.226719512 0.29154664
0.08810443 -0.521222281 -0.536087558 -0.377143161 -
0.06920131 -0.097961508 0.030076432 0.243936932 0.26604318
-0.347198074 -0.413289513 0.125609248 -0.259136119 -
0.427767719 -0.074468425 0.189012566 -0.083332975 0.313942338
-0.361921138 0.466805186 -0.190082751 -0.438454552
0.22527299 0.25291714 -0.411795475 -0.112585302 -
0.188603714 0.333750418 -0.036988584 -0.364486565 0.314197537
-0.500113782 -0.235524441 0.325656811 0.336836344
0.013949241 0.553636015 -0.433596219 -0.035017795 -
0.343379136 0.595198165 0.16314238 0.604501606 -0.068419066
0.664548483 0.17756787 -0.239705588 0.276483249
0.371953368 -0.17530647 -0.096904711 -0.596742379
0.616299901 -0.145275008 -0.455102489 0.377727805
0.292411446 -0.230704798 -0.20463631 0.38906046 -
0.031785869 -0.372566108 -0.035222648 0.379308435 -0.451833597
0.23086126 -0.179277309 0.198121369 0.004128937 -
0.287364228 -0.032261453 0.415739383 -0.554591644 0.507255282
-0.0214415 0.353576934 0.017905402 0.372960069 -0.135546823
-0.096651681 -0.304571734 -0.025205436 0.383125297 -
0.257598202 -0.39556025 -0.284987198 -0.104540232 0.162877199

-0.270027688 0.627999949 -0.340266311 0.178765871 -
0.028861387 -0.406396779 -0.228278555 -0.533045251 0.007233019
-0.31167212 -0.180324511 -0.128786025 0.62938953 -
0.275250606 -0.421528418 0.119010465 0.281954426 0.069197132
0.02350902 0.16195002 0.19043337 0.096803412 -
0.079802965 0.560630675 -0.47635394 -0.217848621 -0.119630496
-0.033039246 0.333388392 0.137151244 -0.39498629
0.346793425 -0.495129283 0.126898139 -0.290599056 -
0.133504801 -0.098500966 -0.342124013 0.288091727 0.39282524
0.210008888 -0.10835936 -0.40320287 -0.219225649 -
0.214028842 -0.055269217 -0.186748489 0.343432453 -0.084762811
0.121606516 0.102785239 0.052362132 0.311497108 -
0.401602744 0.231789654 0.006424819 -0.056268565 -0.257512249
0.463027346 -0.098175347 -0.044497472 -0.450862878 -
0.205929749 -0.202379808 -0.390662515 0.5252077 -0.554123514
0.022718437 0.388425193 -0.362624457 0.122620473
0.41602218 -0.334621642 0.199594569 -0.187574708 -
0.281285984 -0.265795838 0.268559323 0.307037379 0.344448982
-0.284988923 0.053842627 -0.255918429 0.120683198 -
0.041914358 0.293778127 0.170210183 0.320714022 -0.407729606
-0.045546132 0.362103144 0.401344047 0.253725284
0.11151135 0.198143798 -0.007721694 -0.226241871 -0.077202165

-0.419084312 0.199780895 -0.421159504 -0.098020104 -
0.526557393 -0.233449753 -0.369938161 -0.017306459 -0.286485808
0.518756871 -0.311177307 -0.324192423 0.13309819
0.51899398 -0.40990349 -0.521065256 -0.463694444
0.046096799 0.34066871 -0.100950598 0.275204181 -
0.140281242 0.24462008 -0.195566896 0.276234077 0.487784824
0.353442839 -0.15788662 -0.554141627 -0.143855018
0.019818124 0.1539772 0.338886565 0.222155087 -0.226904617
0.282679503 -0.282240322 -0.459500377 -0.5260672 -0.427407564
0.321608972 -0.15921812 -0.487314602 0.324150823
0.074100263 -0.401897402 0.458843018 0.193621276
0.418845505 0.397235676 -0.227082717 0.081254925 -
0.012132059 -0.135385698 -0.029551922 -0.11138476 0.089079324
-0.247258823 -0.069594269 0.075665471 0.229936988 -
0.457991173 -0.071565062 0.570903718 -0.398210076 -0.439879042
-0.343366232 -0.507862294 -0.183094912 -0.112714393 -
0.02177125 0.386798878 0.405170763 -0.291114483 -0.391553237
-0.306392997 -0.212207671 -0.076659969 0.045912999 -
0.259250587 -0.132762778 0.643368854 -0.322551615 0.295146321
-0.466036851 0.134573887 -0.441718563 -0.032840947
0.441769014 -0.580821476 -0.075219248 -0.333353679
0.141735413 0.654617707 -0.223342594 0.115630738 -

0.524009068 -0.364933751 0.172389091 0.353538639 -0.393538092
-0.195614849 0.080246981 -0.451916204 0.484682995
0.21362068 -0.430253678 0.063741299 0.348180571
0.113860280.260180843 -0.196969844 -0.213509238 0.406446121
-0.262823778 -0.502320841 0.099304012 0.437112863
0.153929045 -0.205468723 0.049932635 0.397541237
0.071074022 0.2624106 -0.100436856 0.108020216 -0.454642202
0.186927848 -0.018466261 -0.077331518 0.381478506
0.378972713 -0.059824292 -0.577446034 -0.409362232
0.349287825 0.224131845 -0.231140548 0.303957097 -
0.105685737 -0.272282825 0.326569018 0.210896364 0.477230731
-0.487173815 -0.521411742 -0.643830008 -0.482009681 -
0.111042956 0.212633561 -0.377377773 -0.259168363 0.041153792
0.271569418 -0.132325059 -0.027936244 0.040848627 -
0.335225722 0.107622655 0.097058568 0.314587246 -0.432057842
-0.253413413 0.121237608 0.078818971 -0.275786214 -
0.543059498 0.186548917 0.071693326 0.238195457 0.405710907
-0.532751522 -0.089850668 -0.216023194 -0.109079088
0.218014724 -0.153351802 0.448281735 -0.19348173 -
0.244210854 -0.117898684 0.254723198 0.298311761 -0.159940929
0.261405319 0.16360311-0.355898343 0.172225853 -0.436008264
-0.031257133 0.380537083 0.318537983 0.101356168 -

0.038090387 -0.417220987 0.018840264 -0.227335597 0.065670515
0.235433385 -0.023884898 0.33371528 0.013347046 -
0.024119727 -0.202009297 -0.226886918 -0.075451492 0.051952727
0.225458934 0.158228731 -0.373908723 0.352283717 -
0.449288318
KEGG_MAPK_SIGNALING_PATHWAY -0.049971053 -0.005407805
0.031353322 0.202667225 -0.125570451 -0.091827173 -
0.088405141 0.034567907 0.017461904 0.03542684 -0.083112805
-0.140111968 -0.003640284 -0.185056925 -0.133038433 -
0.14376742 0.048205961 -0.027731685 0.224358386 -0.116546919
0.16894523 0.15847523 0.211956426 0.211708495
0.077292618 -0.080597532 -0.239219925 -0.187670851
0.025225104 -0.086047214 -0.055543192 0.038426805 -
0.1053602 -0.146553353 0.224423494 0.161379082 -0.023488352 -
0.058042398 0.140072282 0.011982332 -0.111759033 0.046675639
0.123246938 -0.089773307 0.072838601 -0.066783377 -
0.055280415 -0.037300581 -0.149024398 0.092889459 -0.011592616
0.211970915 0.173822218 0.265060355 0.110068645 -
0.127797908 0.10550058 -0.121441299 -0.064014524 0.048339604
0.018094759 0.298320411 0.049564444 0.126663386 -
0.097069615 -0.055235009 0.039812081 -0.056729506 0.054834598
0.016772891 0.082233599 0.334757555 -0.076183267 -

0.019103876 -0.100991739 -0.168913635 -0.097933798 -0.058423596
-0.074183873 -0.226982649 -0.042668194 0.331859935
0.022637176 0.089671636 -0.052424933 0.098593729
0.094353985 -0.006171332 0.096489539 -0.14771292 -
0.166070559 0.029091069 0.157148855 -0.077836113 0.099598609
0.011132342 0.084077654 0.160549921 0.008228284 -
0.135450218 -0.014420602 0.102784963 -0.077793561 -0.136554454
0.034671965 -0.09685891 -0.003192153 -0.214490922
0.249988665 0.153270054 0.087255152 -0.245246086 -
0.027640046 0.052571131 0.155105608 -0.026291437 -0.205880524
-0.169850877 -0.047224318 -0.118309859 0.00101484 -
0.195600298 -0.127326891 -0.101254168 0.06276408 -0.077359794
-0.227154058 0.218201228 0.0412391 0.077205141 -0.04425857
0.055883374 -0.145280939 0.123786752 -0.153929566
0.317080974 0.225151282 -0.081209292 -0.086214573
0.270543987 -0.154363831 -0.015200032 0.026429992
0.036754264 0.042819769 -0.045297802 0.136624588 -
0.190841085 0.055763787 -0.047999961 0.143070958 0.104657498
0.22271538 0.140805897 0.013313411 -0.163606123
0.042882409 -0.088026322 0.091056615 0.108444229
0.047976855 -0.103468193 0.052191816 0.051547171 -
0.135660366 0.134029506 0.057444538 0.110677686 -0.046530292

-0.044107521 0.131899556 -0.186213396 -0.063768578
0.008164028 0.261082703 0.146628789 0.149696589 -
0.007281369 0.14782863 0.125599781 -0.083905239 0.103266038
0.029734901 0.002613452 0.133892765 0.055614308 0.0168888
-0.010291163 0.130853565 0.036809549 -0.083352862 -
0.269830976 0.243015198 -0.113837908 -0.19016037 -0.097787095
-0.102992786 -0.043415971 -0.13560693 0.133685636 -
0.00924946 -0.164101332 0.079747366 -0.130412847 -0.004444402
-0.161196163 -0.073324078 -0.116092381 0.10039715 -
0.09899369 -0.023682245 0.06252952 -0.081838605 0.019199864
-0.17316004 -0.022167237 -0.185341766 0.07863281 -
0.118214714 0.109044123 0.23599338 -0.146945218 -0.022253578
0.041081832 0.021308853 0.086376663 -0.053275914 -
0.115833010.155548399 0.006201496 -0.221829817 0.00826863 -
0.056764885 0.053426172 -0.023740105 0.151845927 0.152839942
0.243921534 0.177184838 -0.150920352 -0.038380755 -
0.070339768 -0.141589988 0.15653725 -0.133776438 0.029208596
-0.026824403 -0.134294921 -0.221069377 0.02304898 -
0.00731781 -0.024664745 0.064966326 0.138523516 -0.113455172
0.252580691 -0.148796951 0.09410301 0.097307848
0.22309174 -0.106447475 0.034711215 -0.073879871 -
0.101749951 -0.036019101 0.215546714 0.162519525 -0.258565033

17390 0.072841561 -0.101232605 -0.235508918 -0.00654877 -

17391 0.116232679 -0.022415416 -0.140602737 -0.101916491 -0.105645292

17392 0.014939521 0.006680912 0.061562498 0.172151023

17393 0.071620629 -0.023986215 0.125970794 0.243274775 -

17394 0.208149405 0.093106383 -0.024936249 0.094649761 0.02075618

17395 0.119569867 -0.039513254 0.174466912 0.242545055

17396 0.047548711 0.052088519 0.089883985 0.139161827

17397 0.051726811 -0.02845663 0.160868734 -0.144030075 -

17398 0.044534018 -0.031698713 -0.317877946 -0.019058148 0.208521636

17399 -0.012196141 0.234797317 -0.015798567 0.111764646 -

17400 0.205245725 0.060430944 0.106947409 -0.262319024 -0.109914572

17401 0.074111793 0.113997577 -0.055579278 -0.006648084 -

17402 0.064057193 -0.07880514 -0.067106047 0.005743313 -0.10949699

17403 0.143884717 -0.140662048 -0.225599482 0.046824477 -

17404 0.101593047 -0.131860426 -0.06571858 0.110484775 -0.039282087

17405 0.101903694 -0.055029911 0.086090021 0.177128217

17406 0.175874925 0.117954384 -0.112793891 -0.052668493 -

17407 0.0640542 0.284640961 -0.08369758 -0.123513685 0.155360494

17408 0.042641119 0.175916834 0.017560338 0.227598098

17409 0.038399423 0.061397779 -0.281484464 -0.090756516 -

17410 0.071525647 -0.000561788 -0.030265941 -0.271850863 -0.256136561

17411 -0.048559429 -0.021162724 0.019092286 0.258658453 -

0.122757085 0.267118313 -0.073748865 0.030155084 -0.060382799
0.111974911 0.152563506 0.202130605 0.096364819
0.171586768 0.033967242 -0.059160247 -0.049961079 -
0.226254582 0.272319161 0.059996801 -0.122635706 -0.023224747
-0.230005257 -0.091460926 -0.112801466 0.040325116 -
0.044527895 0.171736002 0.245836805 -0.061417902 -0.090494541
-0.114840159 -0.021727577 -0.016311505 0.12624388
0.123112468 0.05444906 -0.252519911 0.149640145
0.185446855 -0.004275722 0.044715524 0.159937956 -
0.210761442 -0.049050866 0.080143255 -0.006120622 -0.062304047
0.002415651 0.015172216 -0.199448209 0.152372973 -
0.268325539 0.040009712 -0.081297816 0.12902505 0.106536111
-0.248499278 0.204727056 -0.131748102 0.03063784 -
0.165157878 -0.212237857 0.146363841 0.057057749 -0.146911149
-0.30244175 0.216841981 0.016127057 -0.150909041 -
0.178373962 0.056273839 0.074942426 -0.072653249 -0.091548094
0.26833293 0.144111087 -0.054239959 -0.179759343
0.016723813 0.077633558 0.168255497 -0.072725785
0.095021353 -0.004237458 0.054383383 0.032333002
0.07369326 0.060837154 0.087800716 0.173997606
0.072574311 0.002667757 -0.123758391 -0.152800289
0.032774827 0.191781931 0.158033951 0.030341462

17434 0.004167656 0.002154373 0.143128187 0.006827391

17435 0.108083154 0.075778404 -0.06885873 -0.107281173 -

17436 0.021759012 -0.017401969 0.126127504 -0.053534209 -0.154934393

17437 0.028957996 0.2495529 -0.068489807 -0.005575343 -0.029599757

17438 -0.088522307 -0.032239369 0.084990919 0.191116549 -

17439 0.029646536 -0.120216725 0.074688663 -0.170557918 -0.170959278

17440 0.02359395 -0.173220804 -0.061806985 -0.007999997

17441 0.071114958 0.064408519 -0.015180699 0.012519716

17442 0.008737498 -0.07501931 -0.201172608 0.114256634 -

17443 0.166788602 -0.103173876 -0.08483986 0.097509254 -0.091806912

17444 0.264072645 0.210097669 0.056786705 0.004932589

17445 0.13956559 -0.034707103 0.000548786 -0.027936744 -

17446 0.211007402 0.328639619 -0.037752306 -0.089880552 -0.063628008

17447 -0.024433938 -0.058399057 0.144222295 -0.030984157 -

17448 0.253559099 -0.032319732 0.128207475 -0.06789374 -0.177992147

17449 0.086449917 0.024526906 -0.008497377 0.228613697

17450 0.125860857 -0.027605682 -0.245948455 -0.081471056 -

17451 0.004013906 -0.151024092 -0.058887378 0.034414213 -0.175742459

17452 -0.00928026 -0.005300339 -0.052630069 0.069877244 -

17453 0.078408063 0.199687532 0.002799804 -0.032279517 0.020019719

17454 0.016680201 0.070750046 -0.155808726 0.024057035

17455 0.001037221 0.067498935 0.147905727 0.053335591 -

0.006057725 -0.068231472 0.162742929 -0.129579953 0.022733213
0.017584543 0.010899671 0.089537447 -0.021813797
0.059855504 -0.020529028 -0.073261373 0.02287096 -
0.037089811
KEGG_ERBB_SIGNALING_PATHWAY 0.18073368 0.1453997
0.000988212 0.332761344 -0.197963785 -0.008955548
0.154694707 -0.184292693 -0.115855447 -0.207783872 -
0.143310745 0.008779088 -0.284912564 0.033450816 -0.402108354
-0.304207811 0.288046345 0.048112195 -0.099611271 -
0.173777323 0.292408911 0.224674714 0.052771567 0.194704914
-0.167258515 -0.199294628 -0.314622387 0.02752359
0.08844428 -0.220266896 -0.022691918 0.142583247
0.051296149 -0.352666342 0.099085857 0.279112371 -
0.11324017-0.126238364 -0.037941492 0.086045963 -0.080854423
0.096137166 0.195885582 0.147460443 -0.093150895
0.083905482 0.089713019 -0.00231183 0.0003391 -0.039838023
-0.098586606 -0.062717946 0.079133407 0.064645644
0.360193155 0.153358459 -0.19868577 0.095869716
0.210309739 0.314029989 -0.040963244 -0.045391502
0.134917242 0.098648055 -0.22204013 0.054346108 -
0.115297920.121101877 -0.074200497 -0.252011611 0.200458657
0.19250154 -0.019798557 -0.024001562 0.098237168 -

0.265061274 -0.109262703 0.219995337 -0.1097394 -0.217068958 -
0.166850132 0.280230546 -0.087231883 -0.090334017 -0.114894488
0.077407225 0.226545741 0.134853892 -0.194299768 -
0.196821289 -0.393214966 -0.250079757 0.182304408 0.19686458
0.092781104 0.007335299 0.108438967 0.009610547 -
0.086184647 -0.073191058 -0.09258615 -0.21360441 0.071114762
-0.256974951 -0.118971932 -0.177129571 0.162104734 -
0.149399722 0.215267788 -0.245457231 -0.23599566 -0.0976478
0.0620327 0.204927823 0.211568795 0.225078789 -0.254197296
-0.427393195 -0.327020999 0.067947638 -0.213793839
0.076814435 -0.040008424 -0.147711528 0.031781653 -
0.263185894 -0.230662664 0.252134451 0.085339293 0.215130137
-0.180262976 -0.299485962 0.053317671 0.11259806 -0.260824056
0.066810208 0.182464411 0.177950999 0.131745383
0.243632097 -0.050553984 0.058239122 0.183180768 -
0.055694396 0.113208083 0.027502331 0.154761567 -0.125723563
-0.213836472 -0.19389939 -0.143876512 0.285749431 -
0.078077807 -0.099704627 0.189675614 -0.26279809 0.013113589
-0.064211176 0.146616152 -0.127308315 0.177304759 -
0.16597068 0.127435607 -0.17010804 -0.078766203 0.235926042
0.211666148 0.063468632 -0.252568484 -0.047268586
0.115897688 -0.262163873 -0.195600206 0.165013238

17500 0.269416226 0.229517936 0.269296819 0.180315018

17501 0.097977672 0.126705017 -0.267378277 0.112232009 -

17502 0.144051553 -0.017817645 0.272505914 0.344145535 0.100081262

17503 0.083867893 -0.201620844 0.252686868 -0.139090828 -

17504 0.329443489 0.219365787 -0.118556637 -0.32098872 -0.150830782

17505 -0.256117741 -0.013433065 -0.260781788 0.288221115 -

17506 0.242777357 0.100804655 0.090291321 -0.267605431 0.209540444

17507 -0.19171578 -0.064612086 0.031593628 0.2129539 -0.004303278

17508 0.267094599 0.21646013 0.256934176 0.091652044 -

17509 0.329429423 0.102825399 -0.006400269 0.187570381 -0.058262146

17510 0.211637246 -0.034143923 0.192184671 -0.137462916

17511 0.104707288 -0.126339052 0.120583202 -0.139196575 -

17512 0.376361551 0.33455942 -0.070017122 -0.054712477 0.147478747

17513 -0.010028892 0.048523763 0.116102609 0.304504049

17514 0.107866584 0.204483278 0.245759754 -0.260733769

17515 0.157731776 0.048968918 0.101555548 0.077677805 -

17516 0.099942753 0.232196271 -0.028683239 -0.108829267 -0.163599946

17517 -0.170177061 -0.097498608 0.081142859 -0.22172276

17518 0.171874949 -0.181783223 0.133572432 0.16841079 -

17519 0.073195133 0.352739262 0.097486764 -0.223166665 0.17359709

17520 -0.121260824 -0.190330579 -0.086447492 0.175425092 -

17521 0.116401816 -0.062250869 0.228238874 -0.294806587 -0.315225903

-0.046827371 0.075464236 0.016904211 -0.290331538 -
0.135842143 0.167002772 -0.061759172 -0.049369022 -0.129724802
-0.234037612 -0.14191891 0.057634902 -0.137306057 -
0.090214519 -0.214996524 -0.275131689 0.014632073 0.140843375
-0.195183449 0.165464117 -0.060983862 0.15165347
0.218444065 -0.2635912 -0.30342187 0.165581707 0.193099322
-0.155893138 -0.240019893 0.253364635 -0.214417387 -
0.333316404 -0.212538202 -0.255125779 -0.014229883 0.207094506
0.199019866 0.011806654 -0.239444581 -0.131805886 -
0.227120543 -0.17132421 0.267682339 0.104679398 0.068815837
-0.336703867 0.253472888 0.00410694 0.288721653 -
0.201151647 0.240299429 0.262419167 -0.253927827 -0.184449357
0.074732435 -0.092780025 -0.297478032 0.120291888 -
0.015760271 -0.13630527 0.105166802 0.031296382 0.129252168
0.306127715 0.170098758 0.288563073 0.071611978
0.229508413 0.1361671 -0.247458062 0.221148224 -0.322633602
0.164512432 0.210195787 -0.203347599 0.13830216
0.120359288 0.181552217 0.250570665 0.089643226
0.179380341 0.167908836 -0.235779724 0.209639214 -
0.369465221 -0.216747396 0.119338178 -0.216686315 -0.262531665
-0.012068399 -0.063900496 0.157708259 0.171781376 -
0.017671306 0.290316313 -0.120776971 0.250625157 -0.17644175

0.123904142 0.066992188 0.306583742 0.325452944
0.098005399 0.194062762 -0.105920492 -0.167028766 -
0.190410036 0.111436206 0.059541517 -0.349035052 -0.34980369
-0.253990783 -0.287163375 0.13909128 -0.001922421
0.008123803 0.031944522 0.156942581 0.268251375 -
0.106488294 -0.061921996 -0.296071939 0.171887041 0.085327724
0.324712003 -0.033837945 -0.246712863 0.191612737
0.24704552 -0.174083904 0.27519474 0.154346861
0.035107811 -0.024296614 0.023560999 -0.162601916
0.156657114 -0.014023041 0.19123839 0.004326247
0.149843777 -0.338355388 -0.109516729 -0.293286001 -
0.021702032 0.258737717 -0.026989578 0.12925125 -0.064664949
-0.062976953 0.103306414 0.071697904 0.192769469 -
0.114311473 -0.021400595 -0.208132078 0.343680335 -0.054784519
0.046557653 -0.242295616 0.366577768 -0.102782654 -
0.259448134 -0.097101447 0.238147511 0.456881639 -0.024100472
-0.19475387 0.20822272 0.053758099 0.294019836 -
0.115684019 -0.216900194 -0.047052444 0.158376163 -0.086109046
-0.131106425 -0.087084679 0.207954971 0.03093799 -
0.057739478 0.195820127 -0.304927471 -0.278080166 0.194137448
-0.012820533 -0.024574772 0.175829585 0.20715173 -
0.04525864 -0.116774755 -0.244857546 0.166798144 0.111091172

0.070078206 0.037796309 -0.343997191 0.334804725
0.018224604 -0.15527522 0.005159423 0.253333292
0.334383131 -0.1668164 -0.004084157 0.017099638 -0.056669526
0.037991215 0.069250437 0.211437142 -0.031585978
0.115139446 -0.079180561 -0.154564231 0.073616835
0.292955337 -0.111507418 0.006346775 0.21918511 -0.048326964
-0.029594211 0.12266033 0.211807937 -0.007058273 -
0.152321221 -0.283123208 0.029519291 -0.131425311 -0.318971679
0.091232646 0.343666937 0.115288731 -0.029168571
0.193868565 0.206423958 -0.026205463 0.186703696 -
0.180414705 0.036507559 0.197975911 0.046515837 0.189548043
-0.227566885 0.045580919 0.042624114 0.244928897
0.22268351 0.305466155 -0.029006447 0.057197336 -
0.071276765 0.258200557 -0.147658667 0.077188192 -0.147892207
0.106965283 0.08793176 0.31330122 0.206185852
0.001838769 -0.187051165 0.131820232 -0.060927059 -
0.155818379 -0.082957748 0.212622203 -0.078892332 0.069170312
-0.214567476 -0.170725598 -0.023997913 0.012642897
0.157464866 -0.092029876 -0.263352301 -0.124292255 -
0.083775478 -0.069097829 -0.157741477 0.020399235 -0.133563304
0.156159558 -0.183424366 0.017876214 -0.068963351
0.023360256 -0.007808159 0.041302084 -0.261020478 -

0.121005568 0.039903804 -0.063722647 -0.187411011 -0.114356034
0.046581724 -0.157304069 -0.051299631 -0.165487018
KEGG_CALCIUM_SIGNALING_PATHWAY-0.249684692 -0.123088453 -
0.100550474 0.176668039 -0.007730428 0.15416081 0.01909929
0.032541113 -0.130086864 0.028184598 0.082911274 -
0.003952493 0.242789601 -0.097829936 0.150564643 0.100955409
-0.205207833 0.002381148 0.298422106 -0.290011035
0.291662973 -0.201040328 0.216570056 -0.18000204
0.137625847 -0.049534449 -0.226089302 -0.020033196
0.073656572 0.139470081 -0.139446337 0.000624822 -
0.33542024 -0.049176028 0.276125146 0.128677195 0.284287901
0.065064266 0.301427044 -0.124653217 0.206549563
0.125446819 0.148708662 -0.146114523 -0.019804342
0.052720851 -0.065402602 0.261279189 0.1088362 0.098046818
0.058066795 0.055036576 -0.137669028 0.225444951 -
0.023445931 -0.07169973 0.28721832 0.034440533 -0.231780098
0.177082105 -0.203188522 0.280753841 -0.294784083 -
0.043971553 0.068620419 0.229205458 -0.07425819 -0.074829735
0.251699347 0.030492836 -0.251135283 0.066954162
0.237519066 -0.050860091 -0.188374534 0.115158730.077341616
-0.377442715 -0.119822382 -0.296013545 0.075615846
0.147050774 -0.171338123 0.19409442 -0.029892071 -

0.062014953 0.035116550.231951629 0.239978037 -0.091880207
0.005551042 0.276695278 -0.092851344 -0.207495776 -
0.238989317 0.152707951 0.227219856 0.183193132 -0.157073443
-0.157954052 -0.032777929 0.19767294 -0.16379656
0.06931866 0.156413729 -0.0079714 0.129710293 -0.141939962
0.255168067 0.228598663 0.417620761 -0.155573412 -
0.129714769 0.111945374 -0.09121936 -0.242039115 -0.247862084
0.062682084 0.167964517 -0.261866337 0.132990816 -
0.127108217 -0.28072851 0.294006 0.091558287 0.214720997
0.022655797 0.054943257 -0.164183742 -0.21063075 -
0.137564113 0.084090143 -0.256317633 -0.040322004 -0.122367497
0.204936479 0.159001033 -0.002572341 -0.081707767
0.26104254 0.108703682 -0.035693427 -0.029047504 0.0529759
-0.058840789 0.009301612 0.008232944 -0.066543692
0.213299347 -0.129316376 0.272475954 0.037153622
0.229389865 0.324187104 -0.057699856 0.232931994
0.18249792 -0.092385161 -0.002619785 0.185003833 0.0446475
-0.053812888 -0.207074162 0.078242505 -0.221742544
0.05662814 0.097784013 0.223662099 0.244685255
0.262209855 -0.098005915 0.147032233 0.07804269
0.00775242 0.241724802 0.092661215 -0.019370537 -
0.215769664 -0.142396395 0.041046214 -0.026826217 0.196030152

-0.050373822 0.046424834 0.149484336 0.005905364 -
0.157886983 -0.047497936 0.297683267 -0.055995503 -0.223031606
-0.104128239 0.079036052 0.140230038 -0.251093627
0.069168344 -0.112937849 -0.092114852 0.116005232 -
0.056738052 0.057880169 -0.144641134 0.287217589 0.210361932
-0.080741467 -0.162987671 0.105866521 -0.324945605
0.151190105 -0.354086653 -0.223501234 -0.088531137
0.015283569 -0.140235538 0.174356956 -0.257785356 -
0.36652795 0.145410402 0.051023689 -0.057745177 0.32711518-
0.141335099 0.05983686 -0.228998086 0.071254 0.167346285 -
0.095284477 0.013554443 -0.230149801 0.227258077 -0.293672354
-0.142060165 -0.212818878 0.185332364 0.012819755
0.33423672 0.25570818 -0.038063725 -0.147222959 -
0.043002054 0.088181114 -0.091448361 -0.117597311 -0.010431708
-0.202705508 -0.020665824 -0.042769493 -0.00619357 -
0.095841916 0.098235717 0.145339312 0.118606683 0.291939056
0.133587087 0.069949385 0.329919173 0.088693641
0.175960433 -0.236890356 0.147775014 -0.087711074 -
0.332676499 -0.180952154 0.150938874 -0.114556643 0.246277474
0.140234676 0.007347656 -0.256906898 0.250909365 -
0.272223737 -0.117930584 -0.287970644 0.055983966 -0.171912239
-0.189843257 -0.231267738 -0.106299745 0.105148597

0.014310667 0.358418489 0.018423953 -0.099853399
0.232467089 0.26860895 -0.132757801 0.206624118 -
0.15784017 0.074415989 0.170251258 -0.25018039 0.016440068
-0.181190374 0.036138873 0.130969687 0.12868857 -
0.009219821 0.223988099 -0.123500143 -0.081717944 -0.15285522
-0.087923164 0.20339769 0.188978507 -0.123884716
0.161594091 0.062570987 -0.192812834 0.34463155
0.031092707 0.181562977 -0.143864305 0.256072386 -
0.160455336 -0.152922911 -0.181001744 0.291103585 0.007106835
-0.196889634 -0.127696127 0.294826011 -0.23208604
0.002688635 0.305474036 -0.180520148 0.258681483 -
0.161288382 -0.030489753 0.057050129 0.095117685 0.109447568
0.030724769 0.248038997 0.089365815 0.139058366 -
0.125510291 -0.088171319 0.049157126 -0.16686038 0.145602927
-0.277236039 -0.071106496 0.132268453 0.072688788 -
0.260631767 0.057207248 -0.001260144 -0.205413165 0.122979991
-0.119719354 0.254145879 -0.10469936 0.133489787 -
0.354027073 -0.206150106 -0.070325464 0.281278066 -0.224809904
-0.318853654 0.139998415 -0.306437748 0.28676543 -
0.213583116 0.271450209 -0.218609612 0.287342508 -0.285099099
-0.038214168 -0.159464194 0.217293375 0.224352004
0.061625878 0.000285615 0.137043571 -0.26674544 -

0.073016837 0.065143707 -0.083855366 0.131107073 -0.071238889
0.186066252 0.008835991 0.091193181 0.144226165
0.205608143 -0.046706055 0.007542186 0.175389462 -
0.074793906 -0.075619213 -0.336688345 -0.209258368 0.262946501
0.129848058 0.207947456 0.22302509 -0.039465505 -
0.213389281 0.233223101 0.079193459 0.183483106 0.060030406
-0.035933592 -0.077589375 0.186948786 -0.129353652
0.037618043 0.028038072 -0.020199113 -0.235515085 -
0.334640521 0.183656604 0.00397364 0.298493852 0.126809113
-0.137799866 0.11804698-0.138443612 0.192398011 -0.239093757
-0.060408567 -0.291617652 -0.153166367 0.025648246
0.19767273 -0.272313915 -0.022130595 0.176163092
0.041117985 -0.214693607 -0.038975098 -0.049167933
0.196607236 0.139421913 0.120432477 0.173493987 -
0.041707121 0.053905328 -0.224665084 0.115215010.076833153
0.133563286 -0.073908957 0.35062453 -0.041792677 -
0.108798608 0.360160236 0.247438444 -0.080874784 0.252155927
0.074386213 0.09110137-0.024816722 0.188017587 -0.239416433
0.202968561 0.082045791 0.146719266 0.030081261 -
0.096920489 0.055930406 0.041645587 0.174445824 0.112149893
0.026493625 -0.146282696 -0.168307553 0.215816918 -
0.015829413 -0.113303798 -0.084403863 -0.063040892 -0.087411619

17698 0.235868086 -0.110160044 -0.174436545 0.009311423 -

17699 0.108494328 0.028947254 0.098501968 -0.08492079 0.049347687

17700 -0.151372314 0.292923276 -0.245532614 -0.250525608 -

17701 0.032278742 -0.233639019 -0.093537129 0.042064634 0.162629295

17702 0.163859594 -0.110595176 -0.054169881 -0.047141772 -

17703 0.063710819 0.038768329 -0.047568724 -0.311247976 0.076344802

17704 -0.124096506 -0.092914255 0.003370529 0.145077348

17705 0.288209238 0.225022326 0.271363683 0.153809467

17706 0.151189033 -0.124398167 0.095634136 -0.150309029

17707 0.250800562 0.224497387 -0.013585865 -0.151079733

17708 0.058234674 -0.110422011 -0.144065655 -0.060907219 -

17709 0.358433489 0.140476736 0.067271396 -0.084387613 -0.302569474

17710 -0.127107305 -0.162486853 -0.080399716 -0.022207753 -

17711 0.000805628 0.164309537 -0.143985293 -0.126045888 -0.058734571

17712 0.166862306 0.068506763 0.17816591 -0.085732832

17713 0.271116193 0.256395874 -0.24334619 0.064441039

17714 0.125887305 0.099566363 -0.201990945 0.075752836 -

17715 0.081531987 0.090907872 -0.003424089 -0.191215673 -0.0244195

17716 0.075321752 0.005987246 0.065648738 -0.02007603 -

17717 0.037426843 -0.339596968 0.183452666 -0.253013853 0.006642888

17718 -0.099951002 0.045185544 0.147748299 -0.05567737

17719 0.137564446 0.191375502 -0.054568388 0.10936195 -

0.065355918
KEGG_CYTOKINE_CYTOKINE_RECEPTOR_INTERACTION -0.457784119
-0.032114639 -0.304482124 -0.05636772 0.222060608
0.197225362 -0.133993643 0.357891044 0.174402179
0.344623265 0.109141357 -0.427249018 0.388096433 -
0.523430979 0.343282004 0.056622249 -0.2864758 0.117510693
0.352027335 0.157462476 -0.37304008 0.393615268
0.11650506-0.347337209 0.450780131 -0.115569649 -0.031101526
0.136270198 0.159906146 0.109623309 -0.222407164
0.280641659 0.123522654 0.334448914 0.234608797
0.133898575 -0.094327721 0.366203137 -0.196270735 -
0.215031814 -0.306971538 0.154777407 -0.211681262 0.128598635
-0.189898171 -0.312938207 -0.334902395 -0.230221257 -
0.211057212 0.282198338 -0.328733742 -0.04277227 0.085676088
-0.183647156 -0.08905695 -0.333300621 0.253457953
0.011038312 -0.148347193 -0.296073122 -0.067298294
0.285466954 -0.079736372 -0.344432995 -0.240346971 -
0.029142794 0.235222579 -0.374676031 0.473531607 0.436659138
0.223333978 0.309035258 -0.289909045 0.415520241 -
0.280739393 0.433005793 0.239820208 -0.341791502 0.263814881
-0.333683082 -0.034063081 0.319483195 0.362510833
0.116219064 0.339905703 0.161121226 -0.434911298 -

0.222788343 0.405862126 -0.166555112 -0.19384911 0.256284017
-0.096721703 -0.142482136 -0.10199201 0.184071037 -
0.260734088 0.291114549 0.047883455 -0.259254778 0.243365351
0.35506582 -0.008691934 0.319733565 0.34486329
0.002250215 -0.201252865 -0.298329452 -0.26056729
0.41921974 0.392259866 0.188768988 -0.146405368 -
0.305125113 0.108475966 0.107388221 0.129098108 0.267293724
0.239543153 -0.413114196 0.267870392 0.016143413 -
0.284808596 -0.273418692 0.171169059 0.372336309 -0.318561988
0.055249529 -0.37349444 0.137599045 0.027523159
0.361274951 -0.219233864 0.076180584 -0.178891872
0.167085269 0.174457225 -0.431729826 -0.108099296 -
0.154592189 0.012137881 0.173848284 -0.385440879 0.014321087
0.14840107 -0.343228246 0.310494688 0.04842312
0.422665723 0.022931007 0.434126073 0.136257252
0.440214333 0.412069192 -0.200189672 0.137930857
0.066407718 0.084526387 -0.210047154 0.436173092 -
0.106227554 0.143047058 0.17680089 0.224084997 -0.260265896
0.012999306 0.109162372 0.071512642 0.394658996 -
0.012773811 -0.431653296 -0.206572736 0.060957892 -0.117062247
0.123959008 -0.342222076 0.10235916 -0.406504257 -
0.012776848 0.341541501 0.283160372 -0.273657443 0.09104086

0.128575224 -0.175758773 -0.078775471 -0.418496826
0.13769943 0.486973809 -0.173426596 -0.151621558
0.226965235 0.103627255 -0.305273771 0.190457562
0.143579046 0.280192133 -0.199665392 -0.177039908 -
0.330250636 0.1871583 0.184979817 0.106753973 -0.003758039
0.195687377 0.016302055 0.049453509 -0.106007508 -
0.297290306 0.14256073 -0.181880732 -0.303401093 -0.34816003
0.022441084 0.303721337 -0.223200411 -0.192703143
0.358337322 -0.067339927 -0.020716813 0.462274664 -
0.218068896 0.053948566 -0.130563485 0.129029437 0.213135162
0.276425776 0.323716977 -0.196801418 0.328218544 -
0.019785737 0.386200429 0.229914296 0.348768288 -0.051853751
-0.269644594 0.46625442 -0.130052753 0.237246343
0.086456052 -0.105897161 -0.227614056 -0.279517315
0.114926616 -0.462570186 -0.019075169 0.053596803 -
0.260947489 0.104375859 0.254586632 -0.219482894 0.047127866
0.293145286 -0.097049298 0.029180262 0.241440552 -
0.373933668 0.296713274 -0.225319531 0.056890461 0.100542518
0.149998275 0.159732553 0.08667928 0.04854654 -
0.040051517 0.256455294 -0.277009322 0.115186497 0.490057638
0.148004879 -0.260339287 0.148282533 0.16465334 -
0.31790998 0.193685892 -0.246256186 0.411929875 -0.249416637

17786	0.343091509	0.345775863	0.355284391	0.120934125	
17787	0.390475852	0.497266504	0.14124598	0.396288744	
17788	0.027456723	0.318093439	-0.083860235	-0.237511627	
17789	0.310964961	-0.27634125	0.058236881	0.436425476	
17790	0.410401871	-0.22289043	-0.119875866	0.173992477	
17791	0.355638646	0.167635038	-0.296185836	0.331299442	
17792	0.059121384	-0.195080152	-0.068834456	0.337351896	-
17793	0.278742337	0.331692345	0.363734835	-0.050864315	0.154775784
17794	0.381864569	-0.383161062	-0.177840556	-0.468899203	
17795	0.258420603	-0.3191328	-0.215625602	-0.123220829	-0.134738658
17796	-0.421237055	-0.288029593	0.447260809	0.058146616	
17797	0.266118757	-0.290191253	0.01989484	0.043477388	-
17798	0.158465717	0.066993448	-0.398151802	0.309653102	0.180296525
17799	-0.284926847	-0.217720102	-0.219721681	0.261460142	
17800	0.016281122	0.153888982	0.195161097	-0.214615369	
17801	0.278732185	-0.079948899	-0.309292057	0.418867238	-
17802	0.147059151	-0.147793925	0.164927493	-0.284843099	-0.128901657
17803	0.386560414	-0.343167039	0.016215478	-0.504141247	
17804	0.335066594	0.057656151	0.126129655	-0.399087464	-
17805	0.12303274	-0.130308708	-0.325981283	-0.22806234	0.436724585
17806	0.238434269	0.250839948	0.028994187	-0.296354264	
17807	0.106420409	-0.258556082	0.144719895	-0.350836447	-

0.342340549 0.12267387 -0.119286013 -0.008801396 -0.108516936
0.03929664 0.458589336 0.065422764 -0.133030565
0.227863267 -0.196119843 0.210645221 -0.364033658 -
0.136787402 0.091776016 0.2862861 0.075818783 -0.182185653
0.189179919 0.157034982 0.068053948 0.294152566 -
0.012100888 -0.293742256 0.055504254 -0.263292506 -0.210170809
-0.015545851 0.248631051 0.0480133 0.030197879 -0.377069958
0.20643494 -0.127039747 -0.107444471 -0.142790281
0.055828512 -0.191883454 -0.352210825 -0.085411596
0.149433285 0.163717625 0.210440788 0.035542576
0.220833687 -0.377959472 0.354659505 -0.073729961
0.325740641 -0.188698149 -0.460651688 -0.089599562
0.410914502 -0.215439383 0.09881063 0.183860888
0.126189811 -0.400523799 0.140171663 -0.010772803
0.28577299 0.191606951 -0.25743935 0.36953658 -
0.06718691 -0.474017762 -0.421149118 -0.433563202 -0.313360068
-0.353535553 0.26497915 0.355335899 0.06709117-0.008036057
0.131756281 0.428634032 -0.220119066 0.018281771
0.246702964 -0.199006076 -0.25141616 0.265431895
0.334318338 -0.29878706 -0.011819732 -0.292211998 -
0.18960849 -0.174168911 0.169770753 0.284205315 0.396980453
-0.004562146 -0.216154693 0.237728907 -0.266972503

0.32213547 -0.422343427 0.002111885 -0.096412445
0.078842165 -0.082751625 -0.102910136 -0.297281256 -
0.064498059 0.085521455 -0.224155384 0.195786774 -0.081902764
-0.34099062 0.154322425 0.089898619 0.156640458
0.211823348 -0.252214092 -0.198616778 -0.161578525 -
0.217057591 -0.482613605 -0.07211653 0.204268033 -0.139869506
0.021103567 -0.059706278 0.071916005 -0.055006832
0.352049556 -0.237777642 0.441515806 0.089184145 -
0.316214992 0.018573742 0.066749378 -0.079555931 -0.082513123
0.288927352 0.152354002 0.39053568 -0.057445208
0.129543936 -0.229350169 0.343691676 -0.056649724 -
0.368785886 0.151857364 -0.05885563 -0.409754818 0.166092029
-0.046539436 -0.238478581 -0.152979986 0.089772452 -
0.225287356 -0.247268236 -0.218027186 0.169004133 -0.132051354
-0.111061214 -0.004413988 0.144122794 -0.227878373 -
0.086629026 -0.039235029 -0.20811631 0.053361964 -0.005350348
-0.138023293 -0.165215789 -0.156575399 -0.076201896
0.062890167 0.030986264 0.213871277 0.128502346
0.198000045 0.059376529 0.018814211 0.244337817 -
0.223190575 0.160991353 0.19103559 0.044742227 0.11922069
0.025086512 0.085268788 -0.213744859 0.147081279 -
0.115782623 0.176369013 -0.089925627 0.03445207 -0.010216938

0.261633058 0.130472729 -0.219879657 0.150946831
0.115382777 0.004578819
KEGG_CHEMOKINE_SIGNALING_PATHWAY -0.314508737 0.267264456
-0.157748873 0.255178254 -0.063006611 0.058949879 -
0.254481653 0.175925176 0.196456249 0.254920378 0.030796968
-0.424197345 0.271209791 -0.43944635 0.029342803 -
0.136453398 -0.162706028 0.28977301 0.156293742 0.213583452
-0.042823282 0.377057493 0.299224428 0.069895875
0.337827397 -0.294806855 -0.344786104 -0.024597235
0.049326539 -0.213197105 -0.344865883 0.337159793
0.161954829 0.222659115 0.296177012 0.008847236 -
0.080622292 0.206837881 -0.235528759 0.0883261 -0.348685156
0.279180254 0.10820252 0.159526999 -0.241137923 -
0.162288649 -0.135317381 -0.275206224 -0.314806681 0.125446602
-0.353397957 -0.079932304 0.047114812 -0.078900987
0.149774387 -0.188495263 -0.065340895 0.284697623 -
0.064556233 -0.048077003 -0.17538894 0.380434457 -0.006940124
0.016235002 -0.266648419 0.119368896 0.169154377 -
0.262247801 0.169273923 0.212212979 0.361636509 0.487573474
-0.30755113 0.151541682 -0.216019561 0.07804208
0.098191328 -0.355615297 0.238987761 -0.34310065 -
0.191168112 0.377383129 0.258355516 0.045859335 0.251337862

0.259058044 -0.240765671 -0.017188719 0.356557735 -
0.245481294 -0.329538679 0.022756458 0.124157114 0.082543178
0.136394112 0.233539315 -0.204813495 0.389174073 -
0.030981576 -0.187915529 0.324315932 0.059053481 -0.018709302
0.340455843 0.236006903 0.000363675 -0.073736674 -
0.294697097 0.133050326 0.257376958 0.141998552 0.137983602
-0.211613358 -0.131666215 0.086929527 0.272917054 -
0.089207744 -0.044565881 -0.244140143 -0.27993857 -0.146727301
-0.127183365 -0.219210289 -0.342435957 0.211357511 -
0.11686287-0.436562624 0.169792452 -0.030087979 0.27098295
0.14787531 0.230937279 -0.36344504 0.198336047 -
0.052674674 0.338978192 0.163699669 -0.178709027 0.10424015
0.160430142 -0.16444652 -0.144056486 -0.18080582 -
0.037037882 0.187476028 -0.284075363 0.295067408 -0.076698376
0.081873662 0.032016956 0.345386098 0.292911132
0.435858859 0.36604899 -0.063751639 -0.17404238 -
0.026696113 0.070856499 -0.036985177 0.379550665 0.074175628
-0.106129953 0.145404152 -0.120564096 -0.254406138 -
0.127427499 0.133798767 0.220880231 0.117043926 -0.070873114
-0.313606948 -0.330823524 -0.091539554 -0.072847543
0.338774995 -0.135692281 0.085118283 -0.385483837
0.070483235 0.380337603 0.025840204 -0.265507074

17896 0.124074744 0.024579881 0.167152757 0.009066647 -
17897 0.349344152 0.038790415 0.381412713 -0.057641833 -0.250997833
17898 -0.089424347 -0.068367491 -0.344703104 0.056818465 -
17899 0.004317835 -0.008364524 -0.022714329 -0.296870376 -0.131249449
17900 0.078273597 0.177892855 0.135106921 -0.274048143
17901 0.029747962 -0.058582787 -0.24047755 -0.053128954 -
17902 0.072220217 -0.124222966 0.030070844 -0.27080934 -0.27047498
17903 0.139883757 -0.059105029 0.062318495 -0.116394652
17904 0.380997736 -0.047963613 0.207114768 0.42115882-0.114362105
17905 -0.216982401 0.164257637 -0.004826347 0.108859257
17906 0.181186605 -0.162294452 -0.048350391 0.232416013 -
17907 0.222863031 0.28700298 0.115494259 0.255600419 -0.126404208
17908 -0.046304654 0.388909143 0.087090265 0.275363876 -
17909 0.003054447 0.121545148 -0.000683994 -0.334107989 0.198571479
17910 -0.416048716 0.185198468 -0.106827667 -0.191321124
17911 0.028388372 -0.013579038 -0.256204014 0.085438032 -
17912 0.009162244 0.204635034 -0.013748071 0.299493051 -0.225413622
17913 0.048515304 0.100922184 -0.081043451 0.005756803
17914 0.238179142 -0.037958057 -0.210293067 0.183554382
17915 0.180985788 0.157882713 -0.324630567 0.373277722
17916 0.124269343 -0.128081891 -0.240276547 0.268724695
17917 0.114414035 -0.218337166 0.274851689 -0.071377327

0.167802403 -0.323821864 0.34657515 0.202359687
0.267588701 0.198810027 0.0463739 0.336512526 -0.123128264
0.087682269 0.142770611 0.24008849 -0.159840657 -
0.270401354 0.324447846 -0.132464361 0.384558247 0.171136226
0.262002059 0.083512422 0.124995682 0.129007615
0.255048575 0.257479373 -0.245038489 0.064708184 -
0.214237522 -0.208662462 -0.02916485 0.280899978 -0.099923414
0.301909147 0.116314188 -0.026958492 -0.096212232
0.334072772 -0.105139453 -0.118574874 -0.178529502 -
0.070136513 -0.13969745 -0.118018925 0.129858024 -0.342830775
-0.162568975 0.035708751 0.193745044 -0.028545023
0.236847837 -0.248920973 -0.32187908 0.253787213 -
0.325395766 -0.082841704 -0.265345112 0.018322317 0.237269521
0.016923748 -0.080955134 0.119378156 0.341952274
0.270414216 0.256928368 0.144712345 -0.354702882
0.132681742 0.064322478 -0.200582732 0.236260578 -
0.084534453 -0.005407066 0.078371278 -0.216178808 0.054798776
0.2729722 -0.006520971 -0.106612942 -0.319304637 -0.035684187
-0.161699926 0.242513232 -0.297752336 -0.349240679 -
0.324123506 -0.34018955 0.028203435 0.417802951 0.224144327
0.267991485 -0.035220622 -0.008408377 0.056206182 -
0.001940263 0.101182393 0.181304281 -0.060632759 0.22230101

17940 0.075359235 -0.267218049 -0.351564961 -0.210101697

17941 0.434117080.228529011 -0.280250099 0.017664702 -0.287555485

17942 0.079775528 -0.263372135 -0.063847432 0.01956341

17943 0.298420393 0.318760193 -0.065090917 0.208902279

17944 0.108864086 -0.03019001 0.187575132 0.210439882

17945 0.108819485 -0.061534701 -0.335151284 -0.078814521

17946 0.039443846 0.086099922 0.214329046 0.223782166 -

17947 0.369712603 0.124212485 -0.187133363 -0.242801608 0.066409409

17948 0.114378331 -0.190661019 -0.335982471 0.146052777 -

17949 0.221535679 0.207508663 -0.05198078 0.210289774 0.243263829

17950 -0.265893623 0.360532743 -0.091676272 0.378346335 -

17951 0.27636797 -0.353292112 0.288599231 0.225189949 -0.22366467

17952 -0.200714155 0.419511410.133569432 -0.269077859 -0.132247813

17953 0.176614033 0.063051048 -0.152471068 -0.347545 0.371606124

17954 0.360863727 -0.383054389 -0.389094323 -0.287657057 -

17955 0.023530657 -0.242169554 0.257507309 0.148955992 -0.087383769

17956 0.233842179 0.094582394 0.267567966 -0.302350874

17957 0.106409883 0.257339782 -0.231842477 -0.366173094

17958 0.094446484 0.136010528 -0.023545509 0.10648289 -

17959 0.185169231 -0.221827311 -0.170214088 0.12304219 0.263162391

17960 0.273337571 0.009732326 0.092392067 0.03485201 -

17961 0.190620724 0.198518265 -0.180038207 -0.115817796 -0.299021166

-0.031439782 -0.04980763 0.161909079 -0.359081586

0.034462362 0.019179232 -0.209997269 0.327661159

0.091061507 -0.056290732 -0.107805312 0.028047709

0.005862903 0.105549093 -0.275463504 0.016805627 -

0.23584064 -0.251981265 -0.313793431 -0.297827816 -0.062397592

-0.032573826 0.112074184 -0.113912878 -0.270735602 -

0.336845878 0.267974332 -0.251915473 0.374965009 0.179373179

-0.157629217 0.045201604 0.165024631 0.231210833

0.119091077 0.198342431 0.272862969 0.141672242 -

0.017100915 0.148297875 -0.326436121 0.305560604 -0.233969785

-0.289405515 0.193149157 0.082601574 -0.243989458

0.15610706 -0.009531499 -0.359998052 -0.22117725

0.210090745 -0.248224012 -0.275220751 -0.210555117

0.28147581 -0.126942366 0.24157949 0.209218448 -

0.057737877 -0.357823752 0.152279054 -0.00766409 -0.373177342

-0.067535634 -0.167973461 -0.229327089 -0.198982171 -

0.126634407 -0.051181738 0.244446174 -0.033163887 0.338137782

0.123061541 0.002616901 0.007236603 -0.02499855

0.129519954 -0.307994426 0.040616492 0.157288806 -

0.109258002 0.253487164 -0.105722383 -0.053692574 0.010265358

0.080691398 -0.223886622 0.102099922 -0.157604354 -

0.047158799 0.094764585 0.281600564 0.002243057 -0.208973117

0.090199971 0.049758993 -0.027353413
KEGG_PHOSPHATIDYLINOSITOL_SIGNALING_SYSTEM -0.193890997
0.12206596 0.062999348 0.357880167 -0.168055821 -
0.052709194 -0.022954961 -0.325141416 -0.241578931 -0.102325501
0.320579182 -0.05538718 -0.080149979 0.05409436 -
0.207594779 -0.169001873 0.050175373 0.117151733 -0.045477545
-0.033518881 0.401097318 -0.06062326 0.167756142
0.331840639 -0.139221213 -0.154124967 -0.440505287
0.064913122 0.156340891 -0.08146762 -0.286840866
0.144566972 -0.093067521 -0.092444612 0.175848664
0.231763785 0.013267203 -0.301266829 0.294896138
0.19696336 0.130089982 -0.163224335 0.451294524 -
0.10502182 0.061476026 0.010216707 0.332489436 0.207508472
0.010389276 -0.292998271 -0.119027505 0.01576318 -
0.090687009 0.356982023 0.326054502 -0.120316516 0.033573578
0.218406461 0.027044706 0.315661961 -0.264824725
0.313908259 -0.276970701 0.21462462 -0.01063132
0.413515781 -0.322685687 0.083238752 -0.193760935 -
0.058694841 0.146632858 0.196983104 0.097942072 -0.092497419
-0.200511762 -0.064346069 -0.127081574 -0.176013802 -
0.233808335 -0.239102836 0.025210483 0.358300237 -0.193793627
-0.171344078 -0.196799916 -0.025452629 0.309627491

18006 0.349073122 0.184485035 -0.000545093 -0.148877593 -

18007 0.203263901 0.334620571 -0.024342689 0.229843854 0.310147136

18008 0.200484421 0.16253258 -0.179776701 -0.028311717

18009 0.012232675 -0.072453113 -0.078615095 -0.171534904 -

18010 0.197976404 -0.061891456 -0.006199714 -0.102400999 0.327675016

18011 -0.052391322 0.051453749 -0.314516695 -0.334985868

18012 0.105643357 0.179080651 0.214857122 -0.318564657 -

18013 0.215673223 -0.17056836 -0.000109495 -0.124375525 -0.157479724

18014 -0.18151331 0.058650861 0.328075676 -0.299939148 -

18015 0.340256547 0.303917329 -0.010164923 0.279563059 0.073058592

18016 -0.006417383 -0.037371089 0.243332543 -0.233369448

18017 0.31252465 0.156350821 0.070384336 -0.062477092

18018 0.404778068 -0.053405652 -0.259523461 0.183199785

18019 0.012073634 -0.279246065 -0.045555364 0.089463419 -0.149592

18020 -0.150200218 -0.323732057 -0.089149823 0.112098794

18021 0.078173454 -0.012112982 0.23702121 -0.234899118

18022 0.200451409 -0.094377644 0.218086151 0.13877859

18023 0.003015851 -0.372103676 -0.11329787 0.08300372 -

18024 0.332890618 -0.012152789 0.187972741 0.312474174 -0.217510569

18025 0.204580396 -0.002205632 -0.0068934 -0.224165539 -0.156583698

18026 0.388189642 0.172893126 0.198476698 0.021988477

18027 0.068931522 0.201973097 -0.264063529 0.237777562 -

18028 0.087876123 -0.206816994 0.352761023 0.226617492 -0.000768656

18029 0.075238059 0.058710786 0.100126715 -0.405260681 -

18030 0.412660818 0.187266922 0.041563715 -0.375584049 -0.194967836

18031 0.011128703 0.074403066 -0.023764675 0.260965595 -

18032 0.240732149 0.156945344 -0.04929091 -0.280019757 -0.009459242

18033 -0.268219173 -0.10730424 -0.187617542 0.351608427 -

18034 0.170514843 0.048119704 0.014884189 0.298102008 -0.031586762

18035 -0.298789969 0.114256169 -0.343774662 0.290940091

18036 0.045161418 0.336692543 0.081041279 0.165362908 -

18037 0.029622926 0.01335393 0.244628461 -0.209798497 -0.388105066

18038 -0.358305919 0.086098291 -0.13852684 -0.330702878 -

18039 0.092337437 -0.027014249 0.096924502 0.193875172 0.305304908

18040 0.116861508 0.317609291 0.19526296 -0.160664575

18041 0.201343872 0.058539167 -0.10754234 0.039494808 -

18042 0.315832404 0.272917027 -0.083101176 0.211175468 0.201477174

18043 -0.126734376 0.277985133 0.118950093 0.086211437

18044 0.205707506 -0.284340947 0.299023256 0.008978278

18045 0.005766518 0.213775055 0.212804507 -0.167946537

18046 0.001238181 -0.071323888 -0.106754251 0.005731701

18047 0.301985124 -0.018064561 -0.25868736 0.136447277 -

18048 0.286872795 -0.422849194 0.027696671 0.017260532 -0.108887604

18049 -0.172290633 -0.063130241 0.05863957 -0.085280633

18050 0.125713014 -0.006540351 0.232342483 -0.290737777 -

18051 0.27305517 -0.126467097 0.168180457 -0.359817606 0.13396574

18052 0.057135259 -0.214308912 0.172784011 -0.091520266

18053 0.237140253 -0.003082949 0.260888782 -0.252072213 -

18054 0.238376883 -0.001074617 0.399827208 -0.104933482 -0.008476385

18055 0.27708627 0.035411589 -0.108147111 -0.25986413 -

18056 0.370678397 0.048008097 0.028120532 0.136808809 0.156085793

18057 -0.072335276 0.097802775 -0.274544281 0.057567853

18058 0.346261496 -0.041037274 0.17445632 0.069011994

18059 0.09197198 0.109720373 0.169930882 0.106083685

18060 0.031000588 0.245764295 -0.154922451 -0.284770967

18061 0.077442914 -0.189510275 -0.324133052 -0.045390764 -

18062 0.158141411 -0.191283185 0.097862708 -0.2261828 0.054676516

18063 0.269244718 -0.229748277 0.222179314 -0.06565235

18064 0.303606885 0.250674771 -0.188868775 -0.112293811

18065 0.074726958 0.296837549 0.061529841 -0.130704024

18066 0.29059916 0.038366652 -0.231405495 0.142704944

18067 0.107283386 -0.281247223 0.290892681 -0.301941549

18068 0.046615214 -0.310403872 -0.005914749 0.094661452 -

18069 0.303253614 -0.183981175 -0.187121724 0.236255553 0.219635191

18070 0.155835836 -0.326806824 0.374723003 -0.23992208

18071 0.197686556 -0.13893088 0.470017586 0.095748585

0.354868441 0.345154881 -0.008941506 0.190793227 -
0.155824103 -0.198351051 -0.201502391 0.135673083 0.375989879
-0.23372792 -0.232746736 -0.014414885 0.035399974
0.090403534 -0.113650414 -0.076242335 0.168400674
0.144090704 0.0639567 -0.214269166 -0.032328751 -0.015502544
0.049175772 0.255275455 0.327347141 0.004788463 -
0.397733014 0.301566659 0.239094054 0.153755482 0.263605867
0.15246938 -0.21470578 -0.158799992 -0.20826685 -
0.021173268 0.063764582 -0.182290357 0.150476976 -0.347051222
0.380103128 -0.310837645 0.28247663 -0.23012725
0.082258103 0.020937544 -0.028195001 0.001893517 -
0.344857279 0.030528383 -0.309954271 0.07297745 0.299636738
-0.13107114 -0.228356744 -0.2990849 0.323448662 -0.090421988
-0.062299244 -0.038219029 0.203228624 -0.189233429 -
0.091665212 -0.062355675 0.180709605 0.385125226 0.087678383
-0.224741894 0.281754845 0.13748642 0.155058697
0.147381667 -0.06369368 -0.096492794 0.390210842
0.015997818 0.08374825 -0.043271215 0.300837793
0.085261789 -0.005196813 0.005470236 -0.25260267 -
0.379137037 0.196570624 0.208419603 0.079026446 0.304152595
-0.030688608 0.000394765 0.24098578 -0.065073136 -
0.063064056 0.345567247 -0.220814977 0.087802827 0.035821793

0.294168942 0.158766263 -0.168615375 0.040213829 0.0370169
0.353148422 -0.154697818 0.020366705 0.070443548 -
0.150842915 0.088253338 0.212061659 0.290525418 -0.241866994
0.031665993 -0.021982473 -0.339384269 -0.023111014
0.25793576 -0.245029349 -0.139588471 0.247175153
0.088570576 -0.097282801 0.129757235 0.339137858 -
0.193101803 -0.218915503 -0.285919303 -0.049375271 -0.30884225
-0.078897011 0.015852274 0.116015875 -0.212105791
0.187811236 0.348177187 0.079706122 -0.078829084 -
0.072991978 -0.26960495 0.030487651 0.307895299 -0.278939972
0.284658377 -0.13421203 0.054429781 -0.061306614
0.254760914 0.18563021 0.235514541 -0.089059834 -
0.261344515 -0.097010647 0.096346146 -0.074246575 -0.226580225
-0.368840848 -0.002756479 -0.135410424 0.178749078
0.309511824 0.099118929 -0.167149834 -0.157080175
0.085249371 -0.250782207 -0.073666027 0.252478906 0.036002
-0.175995206 -0.024782116 -0.043152249 -0.026123275 -
0.019722844 0.170974362 -0.254961329 -0.095468434 0.168290873
-0.191812707 -0.214579977 -0.198883687 0.24167283
0.183882739 -0.015333781 -0.076196189 -0.105455494 -
0.118587763 0.116317329 -0.192052029 -0.092751069 -0.354618338
-0.015808548 0.248455533 -0.146357817 -0.115345676

0.012115070.093465537 -0.079083629 0.146743202 -0.066673482
KEGG_NEUROACTIVE_LIGAND_RECEPTOR_INTERACTION -
0.25237951 -0.30486198 -0.133036585 0.00593817 0.356947346
0.380196206 -0.03793588 0.353625286 -0.291366045
0.207483704 0.15577948 -0.217587348 0.338568432 -
0.283948793 0.348927947 0.294461587 -0.29987513 -0.142394333
0.480469999 -0.320352438 -0.145557008 -0.165640255
0.038166803 -0.357501636 0.254469698 0.157979523
0.116188016 -0.174905389 0.116889507 0.400001059
0.183541219 -0.041975093 -0.268767907 0.121155892
0.119523989 0.257663393 0.295574112 0.295953986
0.285222726 -0.241697932 0.129113623 0.227950725 -
0.239321988 -0.013591259 -0.260098382 -0.142679014 -0.274798989
0.257101265 0.153987095 0.311236512 0.039639034
0.101300189 -0.155990958 0.089333391 -0.304806183 -
0.249270711 0.466942966 -0.077309227 -0.290500688 -0.035572237
-0.314401083 0.131231366 -0.293514393 -0.290705516
0.123662334 0.075154061 -0.035657709 -0.301716214
0.482917281 0.370072563 -0.227664497 -0.09379145
0.174860718 -0.022131666 -0.150171523 0.440352981
0.203157287 -0.355143005 0.093886187 -0.305898174
0.256132871 0.075403044 -0.128406996 0.069351271

18138 0.056290269 -0.12985357 -0.22487975 -0.021065077

18139 0.286925093 -0.156901925 0.112437180.457469668 -0.21898039

18140 -0.202625129 -0.306701149 -0.031455762 0.131951202

18141 0.279305189 0.012843848 -0.07302197 -0.190367903

18142 0.454827708 -0.095170637 0.084661653 0.216866096 -

18143 0.090881751 0.208408339 0.06043102 -0.087012057 0.464109155

18144 0.57385196 -0.071775348 -0.139327185 -0.088898189

18145 0.078265954 -0.398952481 -0.018549329 0.440796467

18146 0.469660229 -0.361566509 0.461049861 -0.049907623 -

18147 0.328016849 0.289794466 0.06469761 0.553808442 0.193289778

18148 -0.081532282 -0.181794727 -0.305913058 -0.219227184

18149 0.372881285 -0.17133945 -0.163552202 -0.003914161 -

18150 0.006399421 0.003564331 -0.167675426 -0.178498157 -0.10530744

18151 0.297310136 0.127093288 -0.31402564 0.007061532 -

18152 0.135335642 -0.119904149 0.09442035 0.011770766 0.52312078

18153 0.006782907 0.418514421 -0.265511237 0.281843864

18154 0.350218308 -0.168029375 0.458807862 0.095329659

18155 0.043547284 -0.095996111 0.293174452 -0.079182808

18156 0.170702217 -0.112717479 0.397679921 -0.158650654

18157 0.220316938 0.221977477 0.197187185 0.538857904

18158 0.239294244 -0.255290137 0.292353608 0.251554071

18159 0.188564216 -0.190652334 -0.179699957 -0.173747098 -

0.183762804 -0.331657324 -0.021316381 0.178156377 0.114914379
0.105674729 0.057766203 -0.0625338 -0.080771269 -0.33625671
0.003176175 0.444622353 0.122888554 -0.271464405
0.250189906 0.278351099 0.250301508 -0.156907762
0.289810162 0.056366074 -0.075911139 0.215189903 -
0.186390715 0.028868231 -0.246056092 0.335191104 0.36664207
-0.17788435 -0.029832421 0.166227264 -0.306697726
0.005690109 -0.367197712 -0.20354672 -0.049111211 -
0.217256977 -0.251278949 0.461069254 -0.40622159 -0.288680935
0.208734915 0.15044064 -0.204541312 0.346123611 -
0.275551073 0.347397143 -0.308209159 0.212475659 0.317418891
0.068183482 0.39357345 -0.270477086 0.357454064 -
0.088709273 -0.099558722 0.032365081 0.37124602 0.047792957
0.041086046 0.388248935 -0.2510611 -0.033605936 0.138208687
-0.23181308 -0.155036523 -0.064507152 0.029273644 -
0.133753605 -0.287436426 0.055337942 -0.116841732 -0.064619813
0.380101615 0.236310919 -0.030206459 0.415849511 -
0.108081569 0.325654211 0.339059818 0.055316702 0.369309914
-0.352345172 0.305732859 -0.086518749 -0.364416962 -
0.131245632 0.368561733 -0.24406547 0.05231733 0.270743179
0.000149696 -0.206519319 0.534837981 -0.010272174 -
0.183625652 -0.187950085 0.274781 -0.291775795 -0.239475389 -

0.316439898 0.108523249 0.233295773 -0.103274146 0.415275439
0.165199687 -0.003738196 0.585288867 0.45376666
0.297435998 0.43992295 -0.240619299 0.20517159
0.327091516 -0.096777691 0.144092242 -0.284176278 -
0.167738724 0.464341184 0.409942504 -0.124688164 0.055037246
-0.215994338 0.249181397 -0.230829231 -0.008165026
0.394028399 0.407554857 0.123869613 0.255807869
0.101251636 -0.22739284 0.302992609 0.360196105
0.179880646 -0.058942341 0.313606264 -0.359349735 -
0.080098203 -0.193538439 0.4442365 -0.146963916 -0.273040637 -
0.320475707 0.415655493 -0.319915358 -0.202848655 0.529964049
-0.153129092 0.376482905 -0.066261332 0.288677081 -
0.082622678 0.208391492 0.047601995 -0.161759199 0.352816244
0.220681967 -0.1241185 -0.210263476 -0.290073812 -0.006373378
-0.258832262 -0.081604304 -0.162090669 -0.153238103
0.343311865 -0.079518402 -0.293491351 0.299672817 -
0.103378746 -0.293079234 0.235209475 -0.289563074 -0.073730305
0.0949992 -0.223263293 -0.234752094 -0.335236588 0.438352458
0.354828395 -0.283445225 -0.199548915 0.335627223 -
0.274644788 0.228998274 -0.341915757 0.266986879 -0.015103083
0.130085059 -0.120479863 -0.234673553 0.046914735 -
0.192173887 0.00952005 -0.250362446 -0.254105727 0.111629046

18204 -0.329217666 0.140086988 0.293966379 0.101638187

18205 0.05798589 -0.374781395 0.322415977 0.208233764

18206 0.099405352 0.238635512 -0.155582149 -0.065566014

18207 0.078732627 0.058566019 -0.283701301 -0.155036316 -

18208 0.141189205 -0.246879984 0.249604415 0.126483137 0.067296449

18209 -0.079917467 0.116393572 -0.021274933 0.148618034 -

18210 0.114448334 0.298699927 -0.114550981 -0.213197234 -0.077202081

18211 0.162194371 0.007575973 0.288011925 -0.03826515 -

18212 0.016920491 -0.285108617 -0.307165798 -0.192438646 0.289007386

18213 0.101728717 0.375459041 -0.010339827 0.121877281 -

18214 0.281421568 0.170109241 -0.188675771 -0.03313127 -0.06253653

18215 -0.117961094 -0.323067207 0.296362931 -0.172858763

18216 0.347000331 -0.03781409 0.137572391 -0.353278162

18217 0.216377952 -0.104241433 0.335848141 0.418901265

18218 0.268750134 0.127530067 -0.332456632 -0.16667686 -

18219 0.20271563 -0.149449437 -0.179072501 -0.130151118 -0.059817532

18220 0.494533958 -0.031231552 -0.263473043 0.342049896

18221 0.393508323 0.082369665 0.121868415 -0.146086683

18222 0.344477888 0.151911578 0.424970915 -0.044730034 -

18223 0.068427195 -0.070993673 0.17783822 -0.056760184 -0.09746427

18224 0.035518514 0.036795686 0.398861952 0.006299747 -

18225 0.354712477 -0.047350016 -0.352355723 0.351690572 -0.300559876

-0.030404484 0.156091535 0.093160484 -0.173366823
0.013013811 0.029890715 -0.19654901 0.119183814
0.154935903 0.087185741 -0.207910556 -0.360608515
0.171855917 -0.045230267 0.366850385 -4.54E-05 -0.277368307
-0.296814543 -0.122417412 -0.092341568 -0.314024843
0.354881459 0.367032399 -0.281952571 -0.145810162 -
0.021408055 0.265882097 0.217353319 0.107153995 -0.276608058
0.27365021 -0.257707527 -0.193698153 0.115914170.077091095
0.062716069 0.092223398 0.18147313 0.037466001
0.455795342 -0.185350657 0.072272714 -0.00796494
0.02734905 0.441845162 -0.039059342 -0.14090992 -
0.035292278 -0.33271926 -0.176853498 -0.046023669 -0.155019922
0.23546382 -0.11313987 -0.034182708 -0.352231561
0.032737742 -0.341718718 -0.170742294 -0.089931066 -
0.213386751 0.27476882 -0.011533252 -0.195127148 -0.138799502
0.165880134 0.094017103 0.121124147 -0.128422812
0.348327434 0.208914419 -0.170093712 0.033012199
0.171249209 0.017618993 0.044311863 0.25796295 -
0.038269061 0.096836517 0.090254716 -0.020214566 0.026471889
-0.015872347 -0.017936081 0.040111975 -0.051251628 -
0.03299321 -0.436434698 0.299118421 -0.110621411 0.224507667
-0.171554365 0.050221474 0.220488349 0.033104774

0.184493452 -0.045307238 0.017203548 0.183538207
0.005226675
KEGG_CELL_CYCLE 0.043255849 -0.045484816 0.047664995 -
0.202202309 -0.343706824 -0.172255008 -0.260083609 -0.364367316
0.442467392 0.183502304 -0.041471126 0.249356526
0.079105641 0.137196426 -0.384858772 0.062520137
0.004618253 -0.000625505 -0.085483897 0.274723469 -
0.122120968 -0.04995042 0.253444456 0.384387301 0.195040303
-0.172374002 -0.216392516 0.166243198 -0.170531741 -
0.367131366 -0.16672927 0.163299716 -0.347277037 -0.141334003
0.212628073 0.300167304 -0.254184247 -0.359215751 -
0.310288273 -0.163546309 -0.243425078 0.21099858 0.222943234
0.074739626 0.410883557 0.24128255 -0.168340707 -
0.205238036 0.155331973 -0.170537868 -0.148318353 -0.013114436
0.37390601 0.132801903 0.075458082 0.367071154 -
0.213650224 -0.288189022 -0.049680979 -0.112625145 0.276479388
-0.099725114 0.174513474 0.119125994 -0.267826726 -
0.27913597 0.218819752 -0.106507636 -0.283746609 -0.290343683
0.53997876 0.399327548 -0.231624265 -0.27356195 -
0.31878133 -0.306224512 0.026810471 0.17585375 -0.066065107
-0.392312348 -0.358499708 -0.00778675 0.132904441 -
0.17464071 0.2779639 0.549087395 0.142953625 -0.040551316

0.050315473 -0.234153798 0.347279276 -0.329903869 -
0.037472694 0.08750496 0.308492888 -0.035309064 -0.326965516
0.120469838 -0.148995204 -0.345528551 0.257328954 -
0.337807109 0.340065467 -0.307368098 0.247845518 0.078980033
-0.052456026 -0.282395325 0.086860819 -0.277327807 -
0.368569739 -0.131267003 0.316392983 0.183103641 0.006453512
0.371232222 -0.009839229 -0.222515321 -0.160807331 -
0.178448138 -0.488507492 -0.278126442 0.124306381 -0.247102151
-0.240275045 -0.414991952 -0.1954572 0.107935859 0.10584769
0.24378952 0.304607184 0.037471197 -0.135355025
0.124056774 0.258279509 -0.100509072 0.249273762
0.255751488 -0.044096426 0.087030586 0.076355196 -
0.332821252 -0.00283139 0.041020812 0.040357925 -0.080228767
-0.156937836 -0.110084418 -0.362831115 0.120938915 -
0.212304103 0.396659324 0.227313549 -0.058301511 0.101148146
-0.079470779 -0.039713298 -0.230785167 0.330536256
0.155724916 0.202184076 0.285746136 0.325951936 -
0.255300818 -0.098266918 0.196408062 -0.066864802 -0.011697347
-0.301461164 -0.19582779 0.357820621 -0.400264975 -
0.382530653 -0.165285952 0.17443982 0.233347286 -0.16467841
-0.171366447 0.300149319 0.375904397 -0.281823398 -
0.305479719 -0.145226548 0.138270937 0.299491534 -0.033379842

18292 0.369754744 0.156624343 0.211898058 -0.072032294

18293 0.316622397 -0.293786346 -0.348793028 -0.204482095

18294 0.093489441 -0.409809409 -0.352990451 -0.26113931 -

18295 0.199959372 -0.304370861 -0.05801118 -0.051948375 -0.144327447

18296 -0.099324925 0.237622622 -0.138549982 -0.096916674 -

18297 0.012470657 0.027401968 -0.273810614 -0.157945316 0.308721119

18298 0.017416383 0.376411595 -0.283121461 0.193363195

18299 0.252528234 0.261125475 -0.249556382 0.14036934

18300 0.196292041 -0.135590495 -0.423661091 0.63373452 -

18301 0.365124594 -0.3941257 0.400556135 -0.154316765 0.015482778 -

18302 0.263007638 0.164665049 -0.027028907 -0.344693115 0.154409232

18303 -0.287340888 -0.205866442 0.304063857 -0.110192739

18304 0.220234036 -0.028459468 0.022914321 0.076916251 0.0226779

18305 0.050470676 0.439850285 0.134187968 0.2019923 -0.292978044

18306 0.12176275 -0.341672323 -0.225263903 -0.216265095 -

18307 0.243098248 0.45768643 -0.355449226 -0.04967274 -0.101426028

18308 -0.263895935 0.008225127 -0.222924305 0.380642165

18309 0.013035482 -0.037573229 -0.053502535 0.182367957 -

18310 0.163777511 -0.328544033 -0.133811992 -0.131922111 -0.412929565

18311 -0.143502424 0.503114037 0.081952473 0.119681652 -

18312 0.098753544 -0.220595596 0.283204656 -0.207075345 0.065950995

18313 0.003076949 -0.291379071 0.000682091 0.43776849 -

18314 0.360543599 -0.242713271 -0.332199291 -0.352716565 0.087059631
18315 0.2905219 -0.39485738 0.232088341 -0.24586248 -0.000428754
18316 0.165984996 -0.310113764 -0.147162445 -0.068100301 -
18317 0.019684845 0.274076759 -0.336656676 -0.017568146 -0.383325271
18318 -0.347165348 -0.020715519 -0.263292475 -0.211096065
18319 0.427234946 0.187194811 0.284777071 -0.206036436
18320 0.001009487 -0.201703577 -0.07349929 0.144369384
18321 0.049293839 0.350444996 -0.242427427 0.124630616 -
18322 0.212315193 0.35834558 -0.345213398 -0.235019916 -0.037171391
18323 -0.393122357 0.246761366 -0.230114848 -0.21375904 -
18324 0.316846608 0.017679487 0.279315633 0.181497586 -0.07105689
18325 0.01975792 -0.271288094 0.15634427 0.131664673
18326 0.300239537 0.366714295 0.207557093 0.071421096
18327 0.134598734 0.120523801 -0.300118724 -0.112838572
18328 0.182719499 -0.107345672 0.005135089 -0.181342542 -
18329 0.112200455 -0.061388121 0.250862213 0.397452188 0.122060016
18330 -0.178448717 -0.041923412 -0.465346648 -0.301228208 -
18331 0.231433197 -0.282706868 -0.373468648 -0.25109603 -0.25196494
18332 0.282849076 0.086544447 -0.041934908 0.012355009
18333 0.258274452 0.16431046 -0.184890046 0.171214073
18334 0.274815831 -0.010173381 0.169911961 -0.245096518
18335 0.043089894 0.067641437 -0.02324 0.247879809 0.065321353

18336 0.33312588 -0.266166098 -0.044221173 0.157320693
18337 0.120815134 -0.122823047 -0.151924995 0.132618329
18338 0.433986935 0.344417181 0.398345406 0.127778558 -
18339 0.05453232 -0.106142213 0.036994829 -0.178616524 -0.079921318
18340 -0.24253203 0.182630217 -0.185826463 -0.117833593 -
18341 0.019001783 -0.0319119 0.221670759 0.050715366 -0.089365991
18342 0.052182201 -0.204021923 0.051952058 0.289404984
18343 0.169672985 -0.236139919 0.026214323 -0.234588708 -
18344 0.142581041 -0.35217398 -0.133371648 0.153045421 -0.10613306
18345 -0.205136694 0.244867771 0.413435355 -0.110023631 -
18346 0.106681025 0.267419126 -0.375521604 -0.174539995 -0.361938682
18347 0.26993514 0.439430515 0.367262676 -0.413145789 -
18348 0.218905315 0.067177948 -0.482165106 -0.0598827 0.414682281
18349 0.106756812 -0.231679889 0.429363381 -0.025231041 -
18350 0.098250007 -0.157087183 -0.117308073 -0.322018112 0.084154678
18351 -0.232706325 -0.388260379 -0.03025974 -0.229756982 -
18352 0.083044942 0.410153677 -0.421014956 0.027744822 -0.337046282
18353 0.050430522 0.265858892 0.182559373 -0.154689298 -
18354 0.248551197 0.084125529 -0.100401702 0.147648119 -0.053735542
18355 -0.036353601 0.187364531 0.145850495 -0.166699299 -
18356 0.000125413 -0.071552864 -0.290079509 0.28519592 -0.328212766
18357 0.114021964 0.075251643 0.230258351 -0.058118174 -

0.109488086 0.117005597 -0.03119164 0.169231111 0.283998072
-0.063431661 -0.138036883 -0.348134562 0.275583091
0.114414518 0.312012818 0.244243031 -0.134586069
0.199580694 -0.411947781 -0.090988086 0.464863081
0.218941677 0.089365058 -0.213577857 -0.152293154
0.001182758 -0.129173892 0.080585471 0.374912272 -
0.22648778 -0.269960672 -0.305047364 -0.028669186 0.099001902
0.240952852 0.003623867 -0.098819676 -0.036895232 -
0.309774418 -0.316284605 0.250950725 -0.187673764 -0.271334875
0.109947629 0.082269679 0.056845689 -0.252532887 -
0.202311439 0.093697026 -0.01277099 -0.032605434 -0.428448631
0.028731657 0.368922654 0.089381102 0.28366788
0.216091756 0.243309485 -0.427168972 -0.04054991
0.205990063 -0.015989942 0.087283515 -0.409306976 -
0.327612769 0.054858831 0.278693921 0.297348236 -0.339391321
0.318790629 -0.059595793 -0.32114526 0.098112696 -
0.36051895 0.252089222 0.326556021 -0.105760331 0.000105506
-0.314010654 -0.052944646 0.182211123 -0.136839732
0.16187675 0.244644265 0.060904766 0.123915967
0.046281822 0.232545702 0.10919768 -0.367099927
0.061944885 0.131756793 -0.166642747 0.141230147 -
0.140627708 -0.078417947 -0.443250762

KEGG_OOCYTE_MEIOSIS 0.101122982 0.062862581 0.178064773
0.030815472 -0.193558629 -0.230079125 -0.259683983 -
0.320846999 0.168590085 -0.152014635 -0.014798734 0.276380984
0.022133432 -0.120362334 -0.33521349 -0.004474907
0.07533579 0.044722587 -0.151736946 0.118954889
0.103629922 0.09867822 0.098141922 0.351973382
0.009719459 -0.061427802 -0.095642029 0.221568685 -
0.102848302 -0.300690419 -0.242135427 0.058586855 -0.216451849
-0.218963646 0.261894622 -0.008939404 -0.080124245 -0.223769
-0.14248265 0.028461601 0.046811268 0.136293143
0.162613665 0.217864224 0.315675162 0.259057128 -
0.10134788 0.142481481 0.098865142 -0.063618425 0.06738346
0.148551085 0.159422548 0.008559023 0.005766368
0.326046801 -0.212653627 -0.040011409 -0.026753993
0.224479383 -0.04692211 -0.098218329 0.237555144
0.029653414 0.041390657 -0.222493323 0.089729712
0.154056707 -0.233866356 -0.34005474 0.300190944
0.290896087 0.066880227 -0.216417968 -0.181927995 -
0.318853141 -0.004319438 -0.072535889 -0.218160042 -0.241453724
-0.242382891 -0.03047971 0.142013733 -0.123952979
0.024182485 0.359301655 0.091834592 0.137287645 -
0.105401015 -0.184595874 0.063190305 -0.075325178 -0.131648546

0.045139911 0.295895472 0.024289512 0.141754369 -
0.048590218 -0.167826638 -0.109178102 0.24345227 -0.255380831
0.259494461 -0.044847815 0.085698401 -0.110820569
0.095825262 -0.076115483 0.303244484 -0.158445775 -
0.193109067 -0.128206972 0.110308131 0.258429583 -0.086297222
0.262977733 -0.089590711 -0.220346298 -0.251674546
0.067415045 -0.335417281 -0.118166992 -0.062920932
0.161021279 -0.107431895 -0.391813476 0.109785566
0.086443175 0.141686451 0.271488077 0.145589021 -
0.036861455 -0.317058834 0.139234169 0.109157721 0.039971373
0.252618893 0.268089218 -0.072411182 0.343625254
0.113117852 -0.19869514 -0.070951145 -0.024886816 -
0.057649651 -0.066280569 -0.11298082 0.037783132 -0.356810581
0.06174524 -0.038744694 0.180199641 0.191693141
0.021599578 0.131151499 -0.137464437 -0.010256928 -
0.089764828 0.26155336 0.060511709 0.272803904 0.108004216
0.128480609 -0.330772205 0.006192083 -0.001691755
0.108329471 0.199426962 -0.332423521 -0.080716846
0.092421484 -0.231913885 -0.216089614 -0.124346155
0.244590982 0.231865454 -0.14338985 -0.026230987
0.281327054 0.204937099 -0.285027436 0.1778454 0.05042009
0.187395969 0.197810845 -0.030202465 0.069724692

0.223358921 0.050777592 0.02695806 0.217173933 -
0.088092805 -0.197344892 -0.179208892 -0.010229754 -0.297170302
-0.336469411 -0.287020124 -0.158382833 0.046846434
0.013147535 0.034670794 0.300447531 0.142573268
0.016193559 -0.305244265 -0.107797708 0.072913138
0.142779712 -0.216102055 0.006141779 0.01724264
0.203800391 0.242619227 -0.239756409 -0.004753647
0.079590838 0.286533732 -0.134596263 0.119413027
0.112432426 -0.033788237 -0.357816666 0.392144194 -
0.298943962 -0.27455286 0.18540971 -0.161656798 0.111152818
0.060336048 -0.069672015 -0.122042811 -0.333464125 -
0.039454135 -0.197180427 0.178076942 0.021517964 0.01949129
0.088696803 0.008563345 0.067181335 -0.081592906 -
0.121524661 0.227723787 0.131748909 0.113984588 0.135035112
-0.122997161 -0.106161138 -0.339097173 -0.062566264 -
0.262569082 -0.290595582 0.370153034 -0.288826515 -0.063028392
0.125973135 -0.215331237 -0.008669177 -0.129169469
0.024769256 -0.015570837 -0.126416326 -0.091848407
0.233186981 -0.023344904 -0.259427233 0.052863698 -
0.101522612 -0.332606559 -0.097178207 0.323612314 -0.017301391
0.218985844 0.049723702 -0.087550828 0.191917541 -
0.370885783 -0.186226186 -0.178969331 -0.183913557 0.027300994

18446 0.210049609 -0.224975759 -0.230859542 -0.255290593 -
18447 0.315484949 0.02459355 0.010977896 -0.292359026 0.077428646
18448 -0.140612435 0.113115381 0.187405395 -0.241850228 -
18449 0.140640591 0.203087206 0.127589864 0.10185633 -0.278081808
18450 0.141059401 -0.20066429 -0.272856236 -0.149335039 -
18451 0.235867284 -0.072051953 0.240954866 0.126688605 0.227040834
18452 -0.304957082 -0.063719997 -0.22729793 -0.082691676
18453 0.122175627 0.08984402 0.286623204 -0.243216177 -
18454 0.010195041 -0.150945281 0.18801482 -0.049230528 0.050700196
18455 0.117861788 -0.216068878 0.017666152 -0.098684386 -
18456 0.202977196 -0.148376764 0.186450199 0.061627465 0.033007081
18457 0.199529526 -0.084000141 -0.011143433 0.181820499
18458 0.09144208 0.318670614 0.128549382 0.094448399
18459 0.223005009 0.006169154 -0.072769459 -0.191361589
18460 0.140736041 0.111327926 -0.150214593 -0.063355611 -
18461 0.03673755 -0.245084885 -0.075408372 0.194484853 0.205365655
18462 0.254151218 -0.043756116 0.141296555 -0.396231014 -
18463 0.203539743 0.001292933 -0.222060489 -0.025112245 -0.260324832
18464 0.197431703 0.163902749 -0.193378093 -0.004112676
18465 0.179791772 -0.0266716 0.22664206 -0.272606313 0.217392961
18466 0.139417315 0.153247368 0.15902483 -0.014258943
18467 0.137373176 -0.161928895 -0.146149156 -0.056826981 -

0.03468381 0.143721553 -0.205384987 -0.12376713 0.173178442

-0.009711584 0.042026626 0.002672535 0.168562881

0.275535177 0.197924893 0.230965296 -0.102891646 -

0.054916091 -0.001682089 0.261930449 -0.058071974 0.188682189

-0.042472064 -0.043964163 0.016268655 0.027905459 -

0.033098651 0.135411391 0.272026222 0.052510465 -0.212978985

-0.015465619 -0.07330732 0.123599315 0.229239799

0.254266915 -0.099780158 0.157319293 -0.18453247

0.058914815 -0.351457737 -0.063956063 0.052139921

0.146785313 0.123018585 0.079401577 0.211805808 -

0.077779485 0.030260742 0.174608288 -0.373881224 -0.249796624

-0.267225143 0.069057652 0.232213726 0.228990562 -

0.204074158 0.032122739 0.196892856 -0.327256145 0.028463693

0.270825281 0.197235153 0.176248745 0.322533658

0.113801111 -0.046260457 -0.037857802 -0.168358417 -

0.265231684 0.041728568 -0.090303556 -0.049834634 -0.120583353

-0.09587521 0.050785768 0.229811598 -0.275760519 -

0.063467195 -0.278415686 -0.139121225 0.141133151 0.119902805

-0.092130646 -0.124532665 0.134792013 -0.164946405 -

0.011467767 -0.112029271 0.018843315 0.183935452 -0.042389401

-0.038882648 -0.040204784 0.14999794 -0.082074805

0.130342752 -0.113330052 0.194287736 0.013330966 -

0.130907516 -0.133450815 -0.033911215 0.10157038 0.010693763
0.22004162 0.072295919 -0.03959465 -0.095224986 -
0.042025863 0.038037597 -0.00205823 0.304833038 0.059788703
-0.158892232 0.155079038 -0.090280209 -0.001671062
0.270290717 -0.06314174 0.142730328 -0.176077734 -
0.149038629 -0.018882848 0.016848295 0.141922659 0.266850161
-0.034915539 -0.039100501 -0.123610247 0.218808062
0.196091305 0.199929327 0.263619833 -0.090956368 -
0.115840109 -0.083977284 -0.337227866 0.219476006 -0.148519722
0.079228326 0.166789201 0.087512019 0.028512808 -
0.017670091 -0.145990801 -0.151180886 -0.170881298 -0.072895684
-0.290254429 -0.009186134 0.2096391 -0.004835114 0.111957216
0.186490438 0.140359979 -0.199602269 0.096152933
0.09710876 0.035942452 0.019999841 -0.280450661 -
0.165113796 0.109089233 0.219798631 0.114491534 -0.253204036
0.223995606 0.133033111 -0.141406153 -0.18204439 -
0.295421226 0.094142356 0.015843978 -0.039364854 -0.07788599
-0.048074603 0.053146888 -0.033617348 -0.064587779
0.126114767 -0.052822992 -0.024506534 -0.032450435 -
0.171327997 0.026896829 -0.042782229 -0.031804868 0.02561332
0.04754867 0.024447863 0.091091984 -0.026637952
0.188510648 -0.278451858

KEGG_P53_SIGNALING_PATHWAY -0.184092165 -0.366080064 -0.256738128 -0.239552136 0.044083008 0.255141441 -0.138441222 0.185538051 0.122465448 0.306037889 0.101523038 -0.085892969 -0.210337422 0.022711389 -0.113396231 0.213715361 0.008854879 0.146504084 -0.027366888 0.345668313 -0.107714781 -0.179845184 0.143088421 0.27159494 0.309194876 -0.181306811 0.070162208 0.338175419 -0.156134139 -0.273602247 -0.11704913 0.379921534 -0.328206176 -0.10118636 0.209665876 0.318202102 -0.392453324 -3.04E-05 -0.350203943 -0.124718163 -0.147623503 0.389648486 -0.09961437 -0.069058944 0.152341393 0.044186038 -0.374780616 -0.159612625 0.054334721 -0.262658022 -0.189480458 -0.065674475 0.270088882 -0.055130919 0.049906621 0.327562819 -0.043494554 0.082708157 -0.219952655 -0.155806689 0.315247763 0.121431469 0.214378425 -0.209079003 -0.283556926 0.055996769 0.453965677 -0.248802735 0.172869181 -0.311723208 0.359680839 0.276375993 -0.306657355 0.233860005 -0.391699601 0.046899945 0.201652344 -0.017409384 0.061298224 -0.35121028 -0.257488941 0.208605368 0.303337014 0.060848392 0.339159469 0.367139151 0.225348338 0.162758494 0.005878078 -0.161981248 0.079353818 -0.148562465 -0.328909709 0.004853582

0.069889679 -0.358873819 -0.055765065 -0.103586085 -
0.184016271 0.026549485 0.260386646 -0.1678952 0.278452182 -
0.018834918 0.382502227 0.189866977 -0.129070189 -0.125297053
-0.290310357 -0.282841598 -0.412890554 0.111286538
0.00213265 0.223774038 0.083360758 0.176382566 -
0.149828828 -0.178438695 -0.296034051 -0.189750153 -0.387240101
-0.203907675 -0.059151775 -0.272320221 -0.131153909 -
0.306930842 -0.150980159 0.21370725 -0.008251839 0.168349745
0.206546958 0.01807768 -0.195241882 0.223079492 -
0.141241249 -0.039057157 0.338250182 -0.192420655 0.00546358
-0.259121357 -0.070268305 0.146767574 -0.056648874 -
0.073694621 0.321178533 -0.100416427 -0.057119386 0.186507936
-0.228219026 -0.08433994 -0.040585918 0.334831007
0.345712366 0.014281841 -0.061540768 -0.233918963 -
0.168913404 0.030073865 0.102996458 0.351397811 0.062704302
0.215564464 0.177872993 -0.00856435 -0.115724906 0.2034104
0.017240577 -0.03554554 -0.105457936 -0.045613822
0.02479144 -0.443190233 -0.400230306 -0.140485876 -
0.304752166 0.215564406 -0.065217934 -0.145419177 0.281281981
0.307559532 0.154296135 -0.031761937 -0.250672451
0.383097342 -0.033826936 0.14472544 0.267861994
0.161577542 0.081548577 -0.134781118 0.123708402 -

0.053226354 -0.299361216 0.019962503 0.058085257 -0.357175283
-0.00280128 -0.128844321 -0.105218872 -0.331807902 -
0.02997276 0.249415693 -0.170916431 0.002612671 0.380285602
-0.244359591 0.009987557 0.080329495 -0.079318119 -
0.042320665 -0.275361465 0.094545091 -0.181014385 0.434816859
-0.086577286 -0.037506468 0.251040419 0.21152286-0.282608991
-0.247156136 0.108297475 -0.118248421 -0.30348983
0.370693013 -0.092383012 -0.301396849 0.376906479 -
0.063438936 -0.181614574 0.055871942 0.235383833 0.183179987
-0.345384244 0.357857726 -0.32110878 -0.330293889
0.191050147 -0.243968128 0.120406348 -0.020475165
0.097607817 -0.09875137 -0.138525483 0.160560689
0.172752939 -0.076620584 0.378557844 -0.34697778
0.308077574 -0.385551934 -0.277098029 -0.226010192 -
0.036024107 0.324903279 -0.386124353 -0.076822785 -0.268637743
-0.21551188 0.021377797 -0.180760836 0.372814363
0.093955758 -0.400296856 0.012252926 0.014386269 -
0.162950106 -0.286405142 0.097584529 -0.096659293 -0.178690193
-0.07563335 0.08044204 0.160314646 0.113734578 -
0.133974304 -0.142255556 -0.1416117 0.035870684 -0.234642631
0.003113707 -0.192002782 0.240369417 0.108508956 -
0.173541127 -0.072439681 -0.141484716 -0.246081236 0.067251305

0.28768588 -0.40857316 -0.010107535 0.054160371
0.092097521 0.020877047 -0.118495677 0.222763857 -
0.25564401 -0.226558354 0.233477491 -0.30036355 0.021468216
-0.262640894 -0.113986612 0.252761228 -0.22094875
0.011321266 0.481707526 -0.040636105 0.157896516 -
0.276447266 0.134593473 0.11535407-0.14087884 -0.06296135
0.137124881 0.122216056 0.006076378 -0.139850332 -
0.268671845 0.076875404 -0.264488162 -0.164730465 -0.175553748
-0.249158787 -0.01840261 0.109885831 -0.192017484 -
0.309005039 -0.012231796 0.25647751 0.264981413 -0.277593442
0.329005784 0.189185011 -0.233317498 -0.049519561
0.111050551 0.386169917 -0.028487103 -0.220473093 -
0.055036426 0.084174642 -0.334362539 -0.121796392 -0.23062902
0.087836487 0.043967325 -0.083964218 -0.111011182
0.329904336 0.089705504 0.404724851 -0.191872154 -
0.163748221 -0.390783049 -0.3696406 -0.053221936 -0.213789423 -
0.294575242 -0.258754875 -0.030515535 -0.323558781 0.108264758
0.169101463 0.124196478 -0.04683093 0.205842529
0.02221229 -0.191500085 0.135609614 0.128901173 -
0.046558255 -0.127309144 -0.076660348 -0.032818867 0.293970372
0.044837245 0.199909111 0.011680241 0.241495529 -
0.365689488 0.155825875 -0.211861901 0.113548003 0.096359413

18600 -0.2326276 0.291772538 0.383653307 0.314497253 0.428538381

18601 -0.08434319 -0.024248649 0.091606515 0.141649591

18602 0.132837836 -0.341555401 -0.160581155 0.009537412 -

18603 0.137572745 -0.049538551 -0.083065928 0.265943117 0.00368951

18604 -0.22456835 0.118128605 -0.050062948 -0.285188692

18605 0.022441525 0.369886027 0.076406719 -0.256227753

18606 0.087804316 -0.173380595 -0.217544401 -0.046647274 -

18607 0.067079711 0.087665839 0.218718502 -0.018435389 0.168064897

18608 0.407436298 0.028098124 -0.033235148 0.198839117 -

18609 0.101061487 0.037393464 -0.25867803 -0.131890071 0.11555614

18610 0.109424457 -0.22672245 -0.083911403 0.119110133 -

18611 0.515053276 -0.020980422 0.163441479 0.014248469 -0.331644712

18612 0.067451599 -0.040611387 -0.217219775 0.176744324 -

18613 0.043261255 -0.282487982 0.287105061 -0.230907229 -0.310267767

18614 0.166272591 -0.162784156 -0.044478988 0.424269164 -

18615 0.38010456 0.234667641 -0.258173431 0.065522631 -0.177885169

18616 0.171122606 -0.291062331 -0.259250229 0.263168439 -

18617 0.387151454 0.089839868 0.136936916 -0.315828678 -0.17329375

18618 0.419195263 0.132688804 0.030976412 -0.371541149 -

18619 0.417538161 0.099529263 0.055552152 0.130529301 0.067177177

18620 0.448488977 0.061691127 -0.059796725 -0.113954201 -

18621 0.224921693 0.320693216 -0.010599924 0.229069556 -0.129657347

-0.103772247	0.251550601	-0.062379992	0.06128624
0.129033954	0.161415316	0.126557162	-0.146002408	-
0.024635518	0.457078929	0.090656183	0.166444131	-0.230012496
0.296170736	-0.008793954	0.008950831	0.065234415
0.410250757	-0.224037924	-0.315077341	-0.157258438	-
0.268280506	0.080799053	-0.198870076	-0.206845408	0.182876278
-0.241424448	-0.215194921	-0.282748869	0.373545902	-
0.195099488	-0.361125535	-0.239620961	-0.203778767	-0.159656639
-0.056479017	-0.273487333	0.111636185	-0.082879505	-
0.019241339	-0.322286514	0.127956738	0.154393738	0.266365627
0.15888761	-0.112718043	0.181948184	-0.322964387
0.002297691	0.169979734	-0.210451044	0.185033581	-
0.31851991	-0.085316096	0.047231793	0.262147129	-0.03050278
-0.35692099	0.285149831	-0.141070481	-0.186056921
0.111439345	-0.292739363	0.250554273	0.122779407
0.086595293	0.068387872	-0.175866449	-0.192824711
0.203636545	0.011615480.139312219	0.345303009	0.257937657
0.079657339	-0.095938993	0.272351456	-0.144339502	-
0.118079106	-0.195719379	-0.133053477	-0.095351915	0.169885873
-0.265125626	0.013517806	-0.196568455
KEGG_UBIQUITIN_MEDIATED_PROTEOLYSIS	0.35066514
0.340480212	0.115907380.114775787	-0.333123329	-0.251844983

18644 0.006304382 -0.330564798 0.181305881 -0.149292657

18645 0.023565536 0.291496556 -0.328015733 0.156682625 -

18646 0.492961201 -0.299182691 0.333765594 0.197406121 -0.42754503

18647 0.114653845 0.313242458 0.120069927 0.000529295

18648 0.432475169 -0.239515945 -0.278951474 -0.250009576

18649 0.162766159 -0.003405947 -0.426014403 -0.192019662

18650 0.146279454 0.067344058 -0.411957694 0.068358314 -

18651 0.003834361 -0.240295109 -0.338728681 -0.216531891 0.334727568

18652 0.003171955 0.089501491 0.262633003 0.045621732

18653 0.241477841 0.216504479 0.151336091 0.15041235

18654 0.071499534 -0.320727111 0.208336199 0.149707259

18655 0.108367166 0.139942978 0.216045164 0.392317412 -

18656 0.462192274 0.118369667 0.113536125 0.20509537 0.303277294

18657 0.006905695 0.353102526 0.279811821 0.009621093 -

18658 0.126485865 0.111057007 0.353857321 -0.374166392 -0.415516729

18659 0.439789854 0.223037879 0.172933827 -0.074668881

18660 0.006292858 -0.412585631 -0.251274328 0.150501602 -

18661 0.315657206 0.110761504 -0.32886275 0.090621649 0.110247952

18662 -0.047442707 0.095755333 0.220837014 0.344407448

18663 0.11541021-0.295639473 0.031212149 -0.155611311 -0.354881533

18664 0.014002464 0.218540276 0.369899629 0.080009841

18665 0.210069528 -0.038137974 -0.253080215 -0.036559669

0.192600374 -0.45081674 0.284163608 -0.257858492 -
0.022724524 0.031801227 -0.044506303 0.076437222 0.328854353
-0.458179722 -0.582447989 -0.176837286 0.005846822
0.30543862 0.069437925 0.38375061 -0.207004905 -
0.450946214 -0.515594601 0.263120795 -0.48444348 -0.132090381
0.199832851 -0.189377483 -0.049781563 -0.55232079
0.003144201 0.231678431 0.293269101 0.439261847
0.144620558 -0.28538613 -0.066754693 0.147250309
0.118730660.189740082 0.260810049 0.282160236 0.246350015
0.257263729 -0.274538067 -0.182112995 0.303407992 -
0.019454305 0.262949538 0.230246214 0.034987215 -0.085792313
-0.534785161 -0.111562357 -0.387283055 0.31789843 -
0.056672581 -0.329944971 0.33095622 -0.410839427 0.117063183
-0.143112883 0.201622896 -0.1929383 0.316668398 -0.014378764
0.037041203 -0.366964763 -0.005978666 0.003529515 -
0.042134138 -0.012399286 -0.471933886 -0.032194806 0.368805672
-0.344895273 -0.390267865 -0.093445022 0.280143565
0.289596815 0.190461576 0.147840818 0.194869806
0.183723579 -0.318475363 0.209329664 -0.221179604
0.192704679 0.293155887 0.07478718 0.275947792
0.232507896 -0.327843174 0.058862887 0.311670159 -
0.216406097 -0.137204169 -0.258388099 0.096693989 -0.378003186

18688 -0.265293671 -0.070988648 -0.212338644 0.242510984 -

18689 0.141696325 0.309262267 -0.02155239 -0.236826657 0.030960268

18690 -0.17527915 -0.159653183 0.322510319 0.332794294

18691 0.269751656 0.292610895 0.232675286 0.171027756 0.2791449

18692 -0.491438122 0.22402217 0.304447094 0.28529532 -

18693 0.209495126 0.324368017 -0.268203453 0.25890848 -0.427042914

18694 0.399937341 -0.170160167 -0.239439121 -0.015810058 -

18695 0.342610626 0.427681357 -0.416466793 -0.119283692 0.0734433 -

18696 0.069512066 -0.108697713 -0.226452543 0.133244273 -0.340965877

18697 0.290867779 0.23379848 -0.222500997 0.090888096

18698 0.08602689 -0.106476988 0.115295329 0.150565678

18699 0.277020002 -0.096594025 -0.13450958 -0.188002969 -

18700 0.410520712 0.020435998 0.051685763 -0.429078928 0.30270361

18701 -0.232865174 -0.143123566 0.048520631 -0.416657624

18702 0.41289304 -0.314550956 0.162246841 0.173925376 -

18703 0.118149885 -0.413447729 0.267938476 0.157405995 -0.218812988

18704 -0.036127094 0.139141612 -0.534754698 -0.040841807

18705 0.319454551 -0.004201854 0.068931347 0.114185330.091435424

18706 0.205911851 -0.295614834 -0.218213674 -0.25950904 -0.373148

18707 -0.168408467 0.234254336 -0.485910774 -0.4305459-0.339670099

18708 -0.422412856 0.206819521 -0.146438817 -0.376014823

18709 0.140393536 -0.314625638 0.276435807 0.229163205 -

0.454335847 -0.393316136 0.284372817 0.082366465 0.06988909
-0.399017526 0.197788476 -0.218897531 -0.417018939 -
0.309225349 -0.313362939 -0.056755508 0.106402305 0.216565181
-0.081219827 -0.454253437 -0.137738523 -0.293772685 -
0.340487984 0.375191311 0.055797155 0.373840467 -0.39476336
0.126594716 0.248795064 0.441084681 -0.308553778
0.245371188 0.165575518 -0.500729028 0.01165271-0.297520383
-0.065386561 -0.411111217 -0.019971169 -0.051235334 -
0.104019961 0.218722197 -0.29749815 -0.171269827 0.346467011
0.094469145 0.385563985 -0.027850051 0.284519707
0.364299953 0.044527318 0.227719755 -0.392254322
0.154481686 0.35177051 -0.248511078 0.230063801
0.223470364 -0.309914994 0.171105488 0.145056476
0.090971868 0.187454894 0.112380281 0.329102179 -
0.479843581 -0.423902339 0.221198958 -0.063552543 -0.163493169
0.120695075 0.09585786 0.29512066 -0.232632665 -
0.038764116 0.257457811 0.036656776 0.385731448 -0.228262596
0.298327385 -0.024019487 0.42291346 0.283889715
0.062855296 0.338219085 -0.180015017 -0.191252794 -
0.341284648 0.122656891 0.347479834 -0.350529279 -0.252413388
-0.064728228 -0.251736724 0.160445179 -0.029871432
0.182027222 0.227407734 0.280560688 0.31308534

0.04286216 0.124600818 -0.261330493 0.127888385 -
0.077926012 0.250797445 -0.178824109 -0.198768783 0.069623433
0.174847259 -0.034515907 0.160985792 0.351520099 -
0.070067575 -0.240154825 -0.113181426 -0.204735524 0.148649298
0.26247725 0.312101293 0.124554161 0.212567497 -
0.436696959 -0.042134207 -0.419172903 0.006883502 0.077523563
0.308200085 -0.152613503 0.237372207 0.249440127
0.062882535 0.209736231 0.335493422 -0.425356525 -
0.096912854 -0.439438466 0.229074527 -0.033283462 0.360212317
-0.305494855 0.294769907 -0.079250527 -0.451927473 -
0.108107857 0.208984495 0.425952527 0.244266804 0.39395786
0.262008247 0.059686426 0.314429601 -0.069412393 -
0.456598237 -0.077500965 0.179697883 -0.30295312 -0.339625492
-0.062414183 -0.278452573 0.298792984 -0.365078492
0.06799496 -0.430032249 -0.141210498 0.245644933 -
0.047272219 -0.078834828 0.161824268 0.286071536 -0.150656704
-0.169561222 -0.375398557 -0.163409703 0.32804129 -
0.269418893 0.299851971 -0.371449103 0.337984004 -0.067823762
0.06340263 -0.254269012 0.219461906 0.188246791 -
0.032630071 -0.114148929 -0.280320474 -0.154593823 -0.232684053
0.216661603 0.196762719 -0.088911847 0.100413378 -
0.22610415 -0.088065688 0.345774696 0.390065518 0.17191125

0.006529902 0.285387828 -0.273884573 -0.089497682
0.325470141 0.249234535 0.255932756 -0.309550124 -
0.35186034 -0.098984527 0.060603171 -0.257627011 0.15610538
0.137219139 -0.226231245 -0.075380575 0.271789921
0.105741526 -0.126936789 0.098874275 -0.387180552
0.185461484 -0.295847967 0.070995894 0.024922349 -
0.385630206 0.131940094 0.136265325 0.1759531 0.109706685
0.15552004 -0.116573195 0.085247967 -0.283433535
0.065716679 -0.175305092 0.302308392 0.090601929
0.333046032 0.14255449 0.278706445 0.40903416 -
0.27666847 0.062600757 0.163507097 -0.042702786 -0.095196242
-0.148502901 -0.247241505 -0.042072274 -0.143580907
0.008336486 -0.207561087 0.122074824 -0.094622418 -
0.067329872 -0.16629365 -0.216452512 0.009353774 -0.043229419
0.020199576 0.004856342 -0.156354203 0.012912254 -
0.037433726 0.009914945 -0.0354235 -0.062229053 0.296852318 -
0.197723176 0.017392202 0.059506874 0.057321687 -0.175573598
-0.183760376 -0.000907117 -0.201823498 0.104775561 -
0.16401478 -0.17678502 -0.289732486
KEGG_REGULATION_OF_AUTOPHAGY 0.096516979 0.251580364 -
0.146617777 0.001830817 -0.065764663 0.125073372 0.033790264
-0.278857721 -0.089282148 -0.139008877 0.167227593

0.408454482 -0.314882956 -0.119685777 -0.197979446 -
0.194093492 0.30417253 0.026517292 -0.28041798 0.298939339
-0.114444909 0.214974672 -0.264721034 0.238422758 -
0.376584056 -0.053134385 0.260721824 0.026535113 0.173066801
-0.131102769 -0.19368078 0.249425061 0.299028365 -
0.192617964 -0.085684095 -0.389622522 -0.221254179 0.000355911
-0.064274693 0.179391541 0.366411811 -0.141632043
0.217681038 0.242740349 0.259916406 0.070940243 -
0.109644496 0.267155896 -0.020965271 -0.301647867 0.328321469
0.467770088 -0.280178335 -0.11683735 -0.045044917
0.256676543 -0.383306921 0.118994525 0.153033665 -
0.057010999 -0.163898627 -0.209544222 0.286595817 0.112176732
0.182564441 -0.262594513 -0.083628764 -0.086946524
0.008794578 0.053675479 0.218214429 -0.093460886
0.425280467 -0.036842608 -0.137800167 0.129350675
0.18657551 0.354286935 -0.196970526 0.3478414 -0.165985177
-0.073691555 0.188561098 -0.15963732 -0.168269041
0.000736027 0.122704734 -0.193301737 -0.171588006
0.283364421 -0.246429427 -0.031387313 0.150666628
0.100943309 0.359907037 0.146504292 0.278058108 -
0.21684634 -0.184499991 0.165971304 0.112904409 0.105445551
0.174608947 -0.166898846 -0.423283928 -0.271974182

0.051445858 0.414794295 0.057706197 -0.193978072 -
0.066964827 -0.118321014 -0.226352216 -0.079774839 0.354448063
0.423246778 0.126794301 0.13906536 -0.116355568
0.376421217 0.018125936 0.136256437 0.065577635
0.417580714 -0.07029364 -0.174931868 0.516368922
0.027071332 0.224814795 0.413817956 -0.314878426
0.157841207 -0.036598813 0.339763343 0.135303893 -
0.245345472 -0.133520632 0.318304697 0.284285729 0.156902889
0.403638583 0.250805121 0.066056849 0.174393048
0.212244465 0.118207947 0.069986192 0.124942813
0.009709955 -0.328279128 -0.154283648 -0.194532957
0.005620996 -0.218264914 0.114237953 -0.024659574
0.031166060.322467625 -0.054504967 -0.148208074 0.040234312
0.203112665 -0.174789626 0.06757757 0.174587775
0.034035282 -0.047240293 0.11092941-0.022594809 -0.159035017
-0.02011287 0.124485464 0.00222293 -0.045009122
0.019248868 -0.148787383 0.189789707 0.303762512
0.237858135 -0.034094954 0.033180638 0.334159982
0.157420616 -0.144866803 -0.021480182 -0.274148398
0.08895144 0.223821926 -0.010694628 -0.07909784
0.113525673 0.201056016 0.068315463 -0.123923741
0.394373277 -0.103189195 -0.023299009 -0.07481372

0.303448008 0.120915817 -0.358583895 0.163419095 -
0.119832198 0.132190021 -0.099171729 -0.107098129 -0.038676761
0.020170785 -0.01937661 0.200985439 0.302590398
0.078801321 0.160731026 -0.167997132 -0.137570619 -
0.087369027 0.483371672 0.03247473 0.100039474 0.383533049
-0.174699396 0.23792954 0.219271462 0.125411078
0.198665361 -0.12149783 -0.09391641 0.133542394 -
0.059432433 0.091310753 -0.056955645 0.085945628 0.292109015
-0.036882335 0.341861704 -0.187030028 -0.161055517
0.15697447 0.068599344 0.10816026 0.029275172
0.066063266 0.387900532 0.005571338 -0.170849339
0.233023401 -0.020575349 0.255946184 0.355734202 -
0.096342212 -0.004975353 0.052561538 -0.347006418 -0.185793546
-0.271698723 -0.116059538 0.200866569 -0.195308068
0.112117033 -0.034007355 0.080357819 0.245399276 -
0.249498587 -0.251176085 0.07260429 0.217403565 -0.333056373
0.033234015 -0.020355712 -0.085680934 0.197397842 -
0.172126476 0.025522631 0.178187283 0.066153498 -0.058632871
0.052279655 -0.347767346 -0.071134168 -0.147879573 -
0.235603766 0.070579386 -0.171600336 -0.022791271 -0.205629296
-0.134799809 -0.028402775 0.424679007 -0.408207493 -
0.006535821 -0.167254661 0.003586298 0.074572967 0.253219526

0.074096389 -0.059046043 -0.08333776 -0.170095175 -
0.103944432 0.185910096 0.364144284 0.117313478 -0.086350025
-0.297941281 0.132881585 -0.20932172 -0.039642125 -
0.06038042 0.042251416 -0.140401139 -0.312307608 -0.364327091
-0.177018281 0.472129503 0.023769349 0.213552249 -
0.257878425 -0.001420689 0.122208301 0.292782925 0.434187011
0.075152635 -0.160814451 -0.239773338 -0.013618872 -
0.161564038 0.061193620.01092876 0.188871914 -0.153852822 -
0.322668473 0.196281714 -0.358003168 0.082440535 0.075800306
-0.107258547 0.034413531 -0.246187187 0.136997235
0.165674373 0.103726651 0.444874925 -0.060386389 -
0.206717233 0.249752835 0.13647591 0.059289256 0.130070622
-0.362066698 -0.19292509 -0.321237265 0.022447115 -
0.136629201 0.38860885 0.281007835 0.056519743 -0.108384378
0.292879571 0.185337801 0.482770448 0.157886004
0.419760011 0.39866234 -0.355489051 0.04432049
0.195767211 -0.131059413 0.32135572 -0.006784448 -
0.041527834 -0.22893505 -0.202900091 0.187386484 -0.067625626
0.408287987 -0.448505352 -0.245674466 -0.24424448
0.177019544 -0.195923112 -0.113637349 -0.344953896 -
0.131005658 -0.166491799 -0.203678058 -0.036551086 0.121313887
-0.005291345 -0.128369641 -0.030783355 -0.155090832

18864 0.152029379 -0.274156791 -0.04880409 -0.263347293 -

18865 0.228862613 0.187256809 -0.282093979 0.332567948 0.197014606

18866 0.171722502 0.015137584 0.078969692 0.082904815 -

18867 0.219719756 0.275928775 -0.157203882 0.118736003 0.03396427

18868 0.133052964 0.306253668 0.046259989 -0.094105552

18869 0.14993282 -0.245538156 0.190617805 0.046325542

18870 0.270422034 -0.015628332 0.113095653 -0.047290975

18871 0.327886847 0.131500389 -0.122745499 -0.310026015 -

18872 0.114555429 0.26034048 -0.01719506 -0.330157893 0.073726004

18873 0.188990528 0.337969946 0.013179998 -0.030797578 -

18874 0.045153975 -0.168665392 -0.016013022 0.526799765 -0.208880042

18875 -0.080610253 -0.292422164 -0.015874225 -0.145277648 -

18876 0.080738879 -0.045611893 0.193929261 0.292128394 -0.296107704

18877 0.178851364 0.136879477 0.073951954 0.076780576 -

18878 0.217711955 0.033883106 0.121886633 -0.354194945 -0.179605809

18879 -0.273859981 0.413008974 -0.281503482 -0.146334161

18880 0.121323696 -0.338068911 -0.313417464 0.104510702 -

18881 0.147270288 -0.049740125 -0.026980319 0.062367143 0.074836179

18882 0.076009138 0.112099496 0.110314389 -0.02222469 -

18883 0.223518063 -0.027929586 -0.131216217 -0.424810081 -0.048412302

18884 0.243312683 0.016051838 -0.058017759 0.189356059

18885 0.290863183 -0.002562796 0.325108047 0.343318946 -

0.107877126 0.039976233 0.056958223 0.162523884 0.076850756
-0.136721641 0.036449592 0.033193467 0.194950427 -
0.290982256 -0.018305668 0.330383939 -0.077609747 -0.122956879
0.284444606 -0.059535983 -0.22210465 -0.172730368 -
0.228403838 0.096134786 -0.037855626 -0.209825206 0.283664764
0.087465591 0.217248308 -0.247851666 -0.264638116 -
0.207272389 -0.223661299 0.30308502 -0.205988662 0.306528377
0.285233205 0.302375421 -0.055432983 -0.17253758
0.195184084 0.124943754 -0.168811022 -0.244781916 -
0.180105236 0.050489768 0.335401734 0.066946925 0.272717219
0.027498082 -0.108571574 0.115824891 -0.016396546 -
0.240892349 -0.037747247 -0.037274443 -0.232180356 0.052550774
0.094663121 -0.205751953 -0.033494826 0.160242247
0.06214368 -0.073394086 -0.252206054 0.38582139
0.29889789 -0.183891602 -0.239470251 0.102321404
0.157381897 -0.007818244 0.055076111 -0.297904811
0.157360908 -0.035057965 -0.060119786 -0.026289962
0.134692343 0.201152248 -0.00423139 -0.319033214 -
0.006868273 0.107234551 -0.043270239 0.27783845
KEGG_ENDOCYTOSIS 0.254336577 0.251439998 -0.119572327
0.115945464 -0.266794622 -0.205001063 -0.025488934 -
0.131358626 -0.158424862 -0.278119188 -0.157640484 0.041065068

18908 -0.27088738 -0.027821584 -0.259814856 -0.295475718

18909 0.203814658 0.214602417 -0.281840682 0.017922284

18910 0.294489081 0.284914074 0.034973491 0.208715255 -

18911 0.06014586 -0.206359705 -0.28886823 -0.034295667 0.004347211

18912 -0.340899074 -0.097178678 -0.049835247 0.041827688 -

18913 0.191982676 0.05592773 0.056472745 0.029509568 0.004746211

18914 -0.143210273 0.161072362 -0.020887687 0.093694463

18915 0.198792161 0.129186545 -0.031534135 0.020389035

18916 0.183578402 -0.144326393 -0.190502667 -0.107982081

18917 0.004752883 0.015845302 0.240369098 0.024927025

18918 0.331135238 0.110918119 -0.195209867 0.27258681

18919 0.257422494 0.306837725 0.082597573 0.057230558

18920 0.135673106 0.249084357 -0.109868047 -0.053320666 -

18921 0.135890696 0.236571745 -0.284962631 -0.232369563 0.173736433

18922 0.15845226 0.03603934 -0.214762181 -0.049182562 -

18923 0.259695983 -0.171109527 -0.047690374 -0.019633939 0.060426194

18924 -0.141823338 0.312120377 0.016130004 -0.150713425 -

18925 0.10299223 0.076772995 0.137508076 -0.069762503 -0.070070192

18926 -0.040694738 -0.250013276 -0.282197038 0.191346679

18927 0.223746926 0.225062584 0.155851566 0.006467367 -

18928 0.024415959 0.119825523 -0.105543969 0.065005115 -0.322898193

18929 -0.056487862 0.035606408 -0.191721283 -0.231650543

18930 0.015298472 -0.26288111 0.265236103 -0.000835938 -

18931 0.291046397 -0.121855497 0.163814614 0.181881217 0.07805648

18932 0.219835287 -0.015207095 -0.333853971 -0.40935831

18933 0.070743726 -0.303876429 0.0352876 0.134348695 -0.112172496

18934 0.111316406 -0.323567447 -0.062766368 0.127152392

18935 0.124607703 0.276434303 0.166325677 -0.341369911 -

18936 0.062202065 0.164364715 0.082872543 0.141661002 0.14852115

18937 0.289133624 0.12749559 0.192528907 -0.108627529 -

18938 0.080940263 0.126966512 -0.06382572 0.002018621 0.139710887

18939 0.079676045 -0.005232492 -0.315211579 -0.107671782 -

18940 0.14786401 0.130098263 0.046649506 -0.090615736 0.195055717

18941 -0.153278411 0.109483574 -0.025926518 0.007728135 -

18942 0.194526803 0.142549455 -0.205736762 -0.058987289 -0.252652087

18943 0.076399502 0.001240038 0.2198321 0.198306621 -0.328036511

18944 -0.234874164 0.092530825 -0.212252275 -0.084911693

18945 0.173293289 0.287584825 0.189108605 0.121501468 -

18946 0.04227357 0.20530242 0.172663803 -0.062392435 -0.011049852

18947 -0.037871319 0.018214861 0.231857786 0.177300952

18948 0.057702781 -0.02689309 -0.077955845 0.178178946 -

18949 0.070000008 -0.105677589 0.02746796 -0.314824394 -0.137074553

18950 -0.101361334 -0.11770353 -0.086736495 -0.251960398

18951 0.164422578 0.005443199 0.104507927 -0.048693581 -

0.194035633 0.103404158 0.058944232 -0.226073827 -0.139892569
0.193903049 0.145903847 0.255135953 -0.027475587
0.088178937 0.087299109 -0.257788471 0.195299855
0.036180885 0.026186626 -0.138125526 0.199567891 -
0.131568859 0.295325545 -0.143647751 0.045303275 -0.159543995
-0.19987016 -0.160134809 -0.326509577 0.201679621
0.014744181 -0.148989073 -0.142091125 -0.039934586 -
0.04799566 -0.052173406 0.183806341 -0.145864035 0.255915041
0.075783548 -0.171857443 0.19992699 0.109008272 -
0.051589453 0.20457337 -0.140136264 0.301800329 -0.021002404
0.095534532 -0.102168958 -0.229414419 -0.02017721
0.125636746 -0.236145891 0.148011877 0.036171909 -
0.018453981 0.153269114 -0.225043606 0.306171793 -0.034816354
-0.190595645 0.250431615 0.030335969 -0.259709331
0.175495932 0.103536721 0.104490515 -0.147196522
0.299544631 -0.315205025 -0.222143844 -0.221356618
0.284853583 -0.042789597 -0.037251226 0.141909888
0.121132114 -0.013872186 -0.238105746 -0.073751441 -
0.254607444 -0.11527226 -0.067508253 -0.230878015 -0.168422473
-0.127132739 -0.279940352 0.041927501 0.07763924 -
0.2305724 -0.023129981 0.122932141 0.05961716 0.331776595 -
0.277381885 -0.281207881 0.293508193 0.147618847 -0.048808967

18974 -0.157768121 0.097405489 -0.175387782 -0.21215783 -

18975 0.323802333 -0.026681223 -0.021705354 0.066729268 0.220914246

18976 -0.050218935 -0.239596228 -0.188268401 -0.110409506 -

18977 0.053036703 0.28613958 0.030893835 0.157462753 -0.272324998

18978 -0.002861844 0.270928273 0.243981053 -0.185901676

18979 0.102914247 0.287252082 -0.265122226 -0.240190643

18980 0.061659887 0.049202942 -0.31529286 0.17156769 -

18981 0.138761868 -0.254457921 0.190495962 -0.184642058 0.148751072

18982 0.248416713 0.209932991 0.188896464 0.148570508

18983 0.173862758 0.257740793 0.148469357 -0.042897509 -

18984 0.271446507 0.061281915 0.304526652 -0.155403197 -0.037384246

18985 0.158601972 -0.018720491 -0.070799342 0.082746276 -

18986 0.032760888 0.161101465 -0.087322318 0.170053589 -0.238493987

18987 -0.273665224 0.158050169 0.02247751 -0.124232142

18988 0.004328486 0.013231672 0.208524228 0.038193554

18989 0.019475159 0.238125087 -0.123874782 0.242905156 -

18990 0.136764291 0.069930119 -0.119108359 0.330630442 0.251856161

18991 0.241702917 0.174547039 -0.114989762 -0.31061843 -

18992 0.168931425 0.094439655 0.177074729 -0.328298394 -0.195993422

18993 -0.177680959 -0.135510219 0.230142204 0.14530509 -

18994 0.100365506 0.121095419 0.29103928 -0.027304868 0.131502288

18995 0.060827821 -0.31232134 0.078417739 0.107664846

18996 0.300183576 0.041714349 -0.196937863 0.018507913

18997 0.224160697 -0.245300057 0.209893107 0.247351571 -

18998 0.126672112 0.01506067 -0.179295668 -0.254664939 0.149727608

18999 -0.064034655 -0.019668339 -0.020980078 0.17760879 -

19000 0.210776652 -0.12625403 -0.122693607 -0.071990821 0.078247686

19001 0.11411591 0.224541558 0.026045876 -0.033585444 -0.058139903

19002 0.09173691 0.285762603 -0.093100035 0.137513662 -

19003 0.162734583 0.321449631 -0.099654788 0.039530305 -0.303247025

19004 0.244864256 0.001390446 -0.306049309 -0.227141261 -

19005 0.015294823 0.428431235 0.053309791 -0.233604798 0.11849359

19006 0.101683775 0.175287564 -0.121590004 -0.330314435 -

19007 0.07570075 0.188696572 -0.083888342 -0.219676292 -0.041179035

19008 -0.052981224 -0.008046723 -0.226521416 0.023061183 -

19009 0.105201553 -0.091109408 -0.001543228 0.126998082 -0.080738899

19010 0.094475045 -0.035057243 0.274691645 -0.05774433 -

19011 0.275770419 0.092879363 0.275952541 -0.119464621 -0.080108594

19012 -0.275614032 0.245068365 -0.103131993 -0.236693601

19013 0.064771056 0.181579868 0.082187139 -0.252879533

19014 0.152846555 -0.006596019 0.015513442 -0.010656072 -

19015 0.008424806 0.308769305 -0.010990926 0.036014364 -0.078908335

19016 -0.113244936 0.114455093 0.25954838 -0.096169386 -

19017 0.151699471 0.153177852 -0.053658411 -0.202562664 0.05405923

0.131311307 -0.091401996 -0.254479519 -0.126312973
0.080785501 0.025694459 -0.131524152 0.107999092
0.275613253 0.145607732 0.18522252 0.219067344
0.07339352 -0.158551848 0.254084391 -0.217817975
0.003130795 0.00314428 -0.050033738 0.106397724 -
0.277910441 0.112253058 0.261727558 0.283560305 0.154167944
0.210876916 0.087896959 0.020635005 -0.15827675
0.202913115 -0.055556427 0.172641577 -0.120253472
0.155786968 -0.028629619 0.271541685 0.167250946 -
0.141247123 -0.17016408 -0.071377396 0.126132472 -0.230909749
-0.089517207 -0.107811193 -0.03371243 -0.179638644 -
0.205495367 -0.027437934 -0.001864933 -0.025994344 0.181252438
-0.150617754 -0.177829901 0.087143734 -0.193595532 -
0.096010518 -0.049332495 0.097337026 0.101253156 0.158591895
0.127962876 0.094929257 -0.212413472 0.227961903 -
0.193602261 -0.015797251 -0.187325537 -0.01250827 -0.089851715
-0.179843009 0.006060858 -0.098910618 -0.05446999 0.036155
-0.121079332 0.06551331
KEGG_PEROXISOME 0.577886584 0.59448833 -0.333688588 -
0.256662625 0.145866218 -0.017848101 0.256432529 -0.377496617
-0.299272451 -0.300679009 -0.261170695 0.173111649 -
0.463577455 -0.284778493 -0.315729109 -0.29025724 -0.03427549

19040 0.373972225 -0.632423401 0.191757133 -0.261605287

19041 0.226524917 -0.597636725 -0.234410217 -0.33218621 -

19042 0.235363391 0.313193157 0.386048321 -0.180810097 -0.283878639

19043 0.428010413 -0.217642006 0.351857071 0.255829332 -

19044 0.416996016 -0.425760434 -0.205425223 0.054582422 -0.490893913

19045 0.316727998 0.078616693 0.337798007 -0.277606958

19046 0.591658644 -0.265387406 0.362891222 0.322399725 -

19047 0.158369555 0.251485564 -0.079676794 0.157337329 -0.442429601

19048 0.229180525 -0.443168561 0.297124102 0.518899813 -

19049 0.465848101 0.314034065 0.496688763 -0.213733524 -0.345787173

19050 -0.230266693 0.194623639 0.26530543 -0.30931457 -

19051 0.318428289 -0.335922243 0.351109551 -0.363321645 -0.261490831

19052 -0.32801015 -0.369069986 -0.04638498 -0.11611884 -

19053 0.009108187 -0.098905124 0.055442706 0.380709138 0.121857028

19054 0.543323185 -0.167665693 -0.260069977 0.415393837 -

19055 0.05213508 -0.11898932 -0.472023683 -0.379772758 -0.303704506

19056 -0.436267827 0.368508917 -0.403205729 -0.274938319

19057 0.063551384 0.478144653 0.45769264 -0.109226408

19058 0.000606872 -0.497205774 0.401662731 0.271285048

19059 0.321187497 -0.449519125 0.173341087 0.389171013 -

19060 0.194339625 -0.08528651 0.301150001 0.544145642 -0.447209419

19061 -0.111949862 -0.341813247 0.344935615 0.478514563 -

19062 0.421050796 0.025114856 0.108064375 0.246951847 -0.16104249

19063 -0.383385659 0.281284809 -0.079477499 0.442570258

19064 0.029597208 0.054370448 -0.283836857 -0.095236341

19065 0.185458603 -0.287220549 0.312696775 0.318279555

19066 0.182602758 -0.553504922 0.047281007 0.019503746

19067 0.196319382 -0.55765634 -0.504093829 0.564814576 -

19068 0.035971067 -0.475791773 0.191565174 0.109715713 0.241779947

19069 -0.159495253 -0.378203048 -0.229863702 0.224343199

19070 0.469405581 -0.314053805 0.234547027 -0.183309746 -

19071 0.265761327 -0.505173138 -0.151695305 0.215968166 -0.077589015

19072 -0.443671292 0.426811437 0.258948175 -0.446573493 -

19073 0.341817161 0.376906235 -0.397099902 -0.541282077 0.516239718

19074 -0.552761594 0.378628319 -0.15564756 -0.375972873 -

19075 0.350276965 0.245920152 0.344225134 0.200749484 0.280603948

19076 -0.424562305 -0.276723522 -0.328959295 0.39518859 -

19077 0.145661252 -0.26486852 0.230780908 -0.01109015 0.316331726

19078 0.361779438 -0.181408551 0.410405231 -0.259934785 -

19079 0.096347388 -0.485035564 0.277656221 -0.069456958 0.525898437

19080 0.160072267 -0.207534666 0.349717948 0.130766731

19081 0.14065734 0.356830255 -0.05502561 0.067554137 -

19082 0.048658879 0.409516355 -0.197499008 0.497562228 0.098707614

19083 0.322954792 -0.067823969 0.384664736 -0.290324315

19084 0.508002219 0.603106631 -0.155273312 0.109787485 -

19085 0.245333322 0.095634091 0.169262992 0.351350669 -0.168149876

19086 -0.064710962 -0.315199671 -0.477337755 0.595847657

19087 0.004514321 -0.343911228 -0.320471498 -0.124160788 -

19088 0.130477039 0.166783033 0.12634151 0.449891063 0.410841526

19089 -0.173027164 0.312409448 -0.26401044 0.005554333

19090 0.082385975 -0.519268978 -0.07560689 -0.188509154

19091 0.248743645 0.20767943 -0.111893403 0.141740145 -

19092 0.10946706 -0.461475888 0.177361027 0.23869401 -0.075091837

19093 0.243510379 -0.073186675 -0.269654132 -0.115202596 -

19094 0.469406318 -0.609467946 -0.082360421 -0.581907203 0.503933651

19095 -0.240777397 0.458162119 -0.052388945 -0.493185625

19096 0.431778371 0.250155352 0.120668633 0.449835026 -

19097 0.481608344 -0.453191325 0.299310505 0.303880697 -0.164008367

19098 0.528905149 -0.375337643 0.32264294 -0.017344763

19099 0.348011032 0.381703244 0.33863986 0.207380931 -

19100 0.279320553 -0.333072852 -0.579515884 0.007321309 -0.412508359

19101 -0.421839375 -0.554648255 0.27786882 -0.49980005 -

19102 0.024839893 -0.373048582 -0.308097352 -0.162139929 0.418382584

19103 0.08970393 -0.243306704 -0.423559447 -0.466347152

19104 0.410945781 0.084500037 -0.349356474 -0.052685088

19105 0.094033421 0.441132377 0.034240931 -0.213223133

0.577850601 -0.195285002 -0.483633856 0.478725802 -
0.612041773 -0.303120662 -0.447024581 0.334721046 -0.382806676
0.167699797 0.384889722 0.334244932 -0.44224535
0.388840393 0.417038666 0.204353276 0.010916404
0.462300359 0.408577831 -0.0330195 0.069345538 -0.15341463
0.226774847 0.29530961 0.402138473 0.17985125 -
0.356217759 0.462998938 -0.539857936 0.508241333 -0.379958199
0.484055446 0.320466929 -0.484769605 -0.207528292
0.27179707 0.51092716 0.157932697 -0.350549684 -
0.453795726 0.445887131 0.222171558 -0.362268515 0.495709797
0.228854239 -0.302285271 -0.425588386 -0.331698383 -
0.305284947 0.585325124 0.43975616 -0.178855641 -0.127195373
0.277448781 0.434700357 0.26287583 0.304717117 -
0.118108560.398474401 -0.446108712 -0.010655067 -0.31729705 -
0.100216573 -0.052938451 -0.256231104 -0.386110287 -0.472049461
-0.400514323 -0.00137458 0.109905883 0.303527546 -
0.262003199 -0.448035698 0.289942045 -0.562378292 0.26024294
-0.448324765 -0.219582516 0.314661897 -0.319455453
0.045847226 0.491808379 -0.068953878 -0.349748308 -
0.016016565 -0.265029037 0.430191924 0.351236033 -0.18311505
0.387054741 -0.319358694 0.24389977 0.368293512 -
0.159383045 -0.391893683 0.118972279 -0.312820123 -0.148701642

-0.143704913 0.530233803 0.001177553 0.311064669 -
0.212291748 0.032640916 -0.318839371 -0.014446801 0.599581019
-0.195387618 0.408793317 -0.372087558 0.102281049 -
0.371104644 0.033992541 0.469482974 0.101604571 -0.232431903
-0.32351895 0.354321454 0.41336246 -0.216668958
0.085881118 0.356258486 0.355215523 -0.043432359 -
0.483384764 0.239092516 -0.008683297 0.362463929 -0.384701485
0.136505948 -0.038669185 -0.616242268 0.313030488
0.214061447 -0.186391264 -0.095026066 0.03497957 -
0.430610591 0.100486042 -0.486174532 -0.224547407 0.191341563
0.082679438 -0.594891136 -0.463616296 0.178942993 -
0.441776637 -0.130659504 0.200650817 0.233348943 0.45485471
-0.280743739 -0.428901861 -0.459635939 -0.00762529 -
0.082627705 0.523419268 -0.174982897 -0.525215479 -0.000303414
-0.062510444 0.066923051 -0.056646118 -0.256332487
0.167256978 -0.32213084 -0.132275256 0.4262341 0.361378792
-0.543497007 -0.519335988 0.127055038 -0.038968792
0.376009094 -0.162827741 -0.334925135 -0.06381896
0.199122459 0.403598271 -0.123708132 0.353989598
0.352211327 0.188612352 0.415032338 0.102263915 -
0.272199868 -0.265387627 -0.365178633 0.042115198 -0.156545823
0.019124992 -0.121829508 0.321576156 0.18343539

0.282572356 0.229126732 0.314763047 0.375982178
0.483236861 -0.379659041 -0.435094507 0.030251935
0.078770545 0.053519151 0.306535044 -0.1613638 0.04036473
0.045066007 -0.532285654 -0.438082472 0.485396242
0.524728042 0.336350826 0.23438515 -0.080149917
0.316893877 0.479724138 -0.391851559 -0.161268868
0.48418148 0.306769862 -0.386065404 0.161597576
0.252831748 -0.199253913 -0.272067687 0.012911549
0.220830918 0.223019223 -0.231665137 -0.010887548
0.129760797 -0.197301089 0.325427567 -0.351301937 -
0.524717735 0.324808789 -0.232508009 -0.183321412 0.020433028
-0.359237745 0.215784045 -0.093359441 -0.09652513 -
0.034870536 0.440240476 0.103992254 -0.15904333 0.071279409
-0.468229336 0.115247359 -0.179477719 0.495255747 -
0.455547231 0.264742536 -0.354519916 0.284652916 0.12229492
-0.234963613 0.01091405 -0.298500388 0.096138348 -
0.031732771 -0.167072137 0.397823735
KEGG_MTOR_SIGNALING_PATHWAY 0.036110077 0.127728039
0.118819845 0.270230161 -0.244908567 -0.002575315
0.061045083 -0.129366153 -0.174410596 -0.211530299 -
0.014733625 0.020492155 -0.216781165 0.102828722 -0.380191683
-0.337685687 0.306268334 0.070721204 -0.254497145

19172 0.064871705 0.266853392 0.21306244 0.035861945

19173 0.417385649 -0.323677109 -0.022586739 -0.180679953

19174 0.005448807 0.135725054 -0.264695053 -0.302842036

19175 0.259676477 0.392196871 -0.117126441 -0.113436993

19176 0.271656023 -0.402413173 -0.226166852 -0.104743027

19177 0.215318192 0.157654518 0.16073377 0.257230884

19178 0.150890966 0.001471713 0.137314707 0.23368391

19179 0.147360711 0.172893256 -0.316026541 0.025100277

19180 0.078710849 -0.149322307 0.075727686 0.340816725

19181 0.180047826 -0.270450186 0.159833205 0.169438121

19182 0.294199134 -0.22558772 -0.083724611 0.25202631

19183 0.243485735 -0.146349494 0.225763856 0.032785989 -

19184 0.044569444 -0.297679894 -0.24335993 0.064600649 0.066662063

19185 0.104675772 -0.006682643 0.145927809 -0.127196062 -

19186 0.131663268 0.29447298 -0.166595827 -0.228127423 0.045441357

19187 0.157993586 -0.018219556 -0.024144915 -0.274651968 -

19188 0.123067434 0.17729608 0.059088463 -0.068639557 0.110514013

19189 -0.390898083 -0.105474437 0.201069302 0.330465864

19190 0.032959189 0.08456621 0.269616037 0.203441299 -

19191 0.057377386 0.09594691 -0.115866244 -0.285637027 -0.000344485

19192 -0.139372486 -0.032676915 0.022242735 0.211442957 -

19193 0.170488575 0.304278974 -0.346234414 -0.301221796 -0.143806171

-0.220816062 0.177182852 0.272976786 0.253038454 -
0.207541608 -0.231666876 -0.297920072 0.066812996 -0.191052934
0.083494959 -0.042686896 0.055161294 0.094464711 -
0.307835048 -0.074317879 0.334935873 0.26615366 0.188057942
-0.212962252 -0.150067439 -0.146306863 0.190546583 -
0.231112316 -0.015912169 -0.016171173 0.167780092 0.230352155
0.309109787 -0.135292539 -0.003025981 0.280164435 -
0.060577674 0.141242057 -0.05954082 0.355866423 -0.014948251
-0.230626415 -0.155562833 -0.115735097 0.106247171 -
0.04156229 -0.174506767 0.160988397 -0.148552847 0.14502393
-0.148371796 0.014394309 -0.117774498 0.085439832 -
0.280370534 0.176453414 -0.109731563 0.052035902 0.29247176
0.079747194 0.127600172 -0.229039542 -0.128776065
0.042331275 -0.189988491 -0.295285092 0.021644502
0.112656971 0.072113672 0.285234769 0.011596548
0.064836939 0.06954593 -0.186264877 0.179524268 -
0.033183289 0.089114723 0.180774799 0.096892921 0.025351836
0.035756031 -0.252353897 0.114596093 -0.194493084 -
0.207800148 0.239320788 -0.113978149 -0.065677555 -0.068499303
-0.080693564 0.12493174 0.041451921 0.361495838 -
0.278034125 0.062987157 0.015632126 -0.378326304 0.026715755
-0.070826569 -0.197721036 0.221934131 0.240599889

19216 0.005487822 0.168788962 -0.034104948 0.074636269

19217 0.242539135 -0.270583635 0.280964913 0.239108507

19218 0.022181572 0.116771099 0.217705441 -0.034485597

19219 0.249340637 -0.141473553 -0.063798653 0.040721473

19220 0.062390989 -0.260454593 -0.406495581 0.272406173 -

19221 0.075076456 -0.244824853 0.049829834 0.238802187 -0.230836007

19222 0.127277892 0.053623584 -0.20305152 0.152574092

19223 0.192298178 -0.244970011 0.072317989 0.372629316

19224 0.071945894 0.169488373 -0.357800293 0.349645434 -

19225 0.03328429 0.067997702 -0.134287787 -0.229271303 0.006749824

19226 0.113318555 -0.222397195 -0.061129002 -0.094456639

19227 0.00620757 0.178002026 -0.295133453 0.359290655 -

19228 0.07942977 -0.162691678 0.207874969 -0.073150331 -0.453577536

19229 -0.086284541 0.186458483 -0.16949854 -0.162679931

19230 0.313113074 -0.400527923 -0.180745988 -0.12551423 -

19231 0.207368962 -0.058676777 -0.139950579 0.002382865 0.059504468

19232 0.037959882 -0.200293172 -0.14042111 -0.322126074

19233 0.057754039 0.004764349 -0.215122462 -0.251810901 -

19234 0.358820149 -0.26587544 0.113481618 -0.110158545 -0.16902336

19235 -0.018474346 -0.286611717 0.110610484 0.200559649 -

19236 0.242658477 -0.151049815 0.12640337 -0.024285931 -0.011659312

19237 -0.07747922 0.177276259 -0.140293747 -0.271526184 -

0.326180307 -0.285887272 0.288610472 0.125481987 0.137225225

-0.145001461 -0.313492331 -0.126545908 -0.194087311 -

0.200246336 0.376539879 -0.033845765 0.148742823 -0.376806661

0.333642655 0.023746723 0.275711312 -0.172657264

0.265522625 0.118133366 -0.31853286 -0.070771884 -

0.153329461 -0.014794727 -0.395367717 -0.05830597 -0.223204962

-0.154675803 0.145444627 -0.06740349 0.199080006

0.279003192 0.236601391 0.269342695 -0.004076172

0.236081674 0.155967855 -0.114226805 0.121108784 -

0.105509817 0.092947122 0.247901736 0.078131012 0.294119716

0.108954691 -0.146515292 0.246384802 -0.164881701

0.05788844 0.186568232 -0.12242389 0.182832644 -

0.288998387 -0.198824978 0.291271332 -0.188972189 -0.097296865

-0.187693237 0.172287652 0.24991361 0.016791977

0.008718277 0.318917349 -0.038244382 0.210371738

0.049484434 0.260933694 -0.12335725 0.345028222

0.380805677 0.225661626 0.358101505 -0.328035144 -

0.317835254 -0.259680003 0.095947917 0.181556911 -0.199188719

-0.327049925 -0.039460854 -0.329901744 -0.122038927

0.00177498 0.025580859 -0.089652254 0.032312639

0.350503517 -0.08689986 -0.144445638 -0.197848208 -

0.050460494 0.19298918 0.173385355 -0.062122745 -0.355763896

0.236777878 0.192502693 -0.095411652 0.426506417
0.183863658 0.107861246 -0.222018458 -0.080098884
0.102797153 0.168444757 0.128559454 0.13217577
0.024122869 0.276592251 -0.445485407 -0.089335465 -
0.234260142 0.342510428 0.227987972 0.139136822 0.029986504
-0.038083897 -0.060876987 0.213087876 0.161303217
0.212993568 -0.194475354 -0.292857476 -0.356784651
0.246143331 -0.239061113 0.193065313 -0.212659305
0.179260935 -0.38615203 -0.266697681 -0.05947246
0.190442942 0.428672349 0.163785939 -0.15739049 -
0.061145044 0.349065767 0.194838215 0.110892208 -0.278256701
-0.216384813 0.23870995 -0.069179107 -0.247608269 -
0.045602908 0.289215022 -0.036382062 -0.154176056 -0.249694199
-0.136446858 -0.306721169 0.283266033 0.112255850.096412802
0.004987523 0.113466556 -0.105363149 -0.056922754 -
0.262798215 0.112585926 0.219565583 0.00305636 0.303199036
-0.316981896 0.309719262 0.056441201 -0.276736731 -
0.015396313 0.335906548 0.287145301 -0.300735664 0.059958691
-0.031686129 -0.090525879 0.114768985 0.198918386
0.275741686 -0.220720281 0.163577141 0.000572511 -
0.233767867 0.252316084 0.132616166 -0.154056282 -0.033610452
0.158306606 0.178489235 -0.119681959 -0.139567217 -

0.036491942 -0.075220837 -0.150573114 -0.257222258 -0.14086103
-0.030383388 -0.253637008 0.083204437 0.324019135
0.035870256 -0.011703311 0.059256054 0.187562727 -
0.2331551 0.216778969 -0.177463602 0.206842128 0.259900293 -
0.237429789 -0.059535482 -0.36736527 0.096521951 0.098478594
0.193742598 0.219194238 0.213922746 0.013760813 -
0.101538857 -0.115742443 0.224269397 -0.049648851 0.130098985
-0.230227479 0.326664573 -0.203265364 0.200854409
0.13428302 0.163898086 0.097502492 0.243474811
0.015496056 -0.237690071 0.014752507 0.032478128
0.098585507 -0.078741883 0.054413972 -0.036902428 -
0.20311393-0.100424788 -0.051750279 0.229507459 -0.227064634 -
0.027019326 -0.290899828 0.025689638 -0.202857812 0.018457581
-0.196116231 -0.040698998 -0.065915726 -0.164795214 -
0.265344058 0.34924711-0.258727184 -0.090179332 -0.243746208 -
0.070901776 0.220066185 0.082668582 -0.204844186 -0.095494247
-0.216387984 -0.090612999 -0.141378753 -0.111128467
KEGG_APOPTOSIS -0.147599453 0.07588699 -0.078956367
0.145676368 -0.196926734 -0.125447474 -0.044026431 -
0.014823024 0.131560356 0.101450703 0.190968032 -0.322093622
-0.336785712 -0.225859362 -0.257264576 -0.31115587
0.27991403 0.321807908 -0.170783925 0.357220956

0.288258317 0.367254389 0.067104222 0.368975899
0.121053989 -0.232960432 -0.269350192 0.198061022
0.130769563 -0.257110264 -0.299247256 0.357856925
0.167088862 -0.104597094 0.148884716 0.086154512 -
0.390559951 -0.10552668 -0.226713195 0.187002705 -0.319886844
0.146156537 0.200925986 0.15904247 -0.055303914 -
0.051910894 0.076753946 -0.248148942 -0.019220286 -0.206509648
-0.334784391 0.028717779 0.311133997 0.041153882
0.334865007 0.004893908 -0.372536513 0.140062766
0.073842852 0.176467245 0.093864507 0.17024869
0.317633552 0.193361525 -0.125642195 0.019960469
0.271819561 0.029544576 -0.193516941 -0.156228414
0.469481232 0.466040062 -0.302859798 0.059780629 -
0.166097282 -0.173969178 -0.132871282 0.001065385 -0.285574803
-0.158190952 -0.193824221 0.237074923 0.236498444 -
0.038045146 0.21834849 0.078436929 0.106622538 0.107190796
0.254028556 0.016246088 -0.228895387 -0.303604957
0.367852712 0.175767424 0.287418488 0.206491247 -
0.171165801 0.114602517 -0.103941192 -0.109476977 0.346829492
-0.315556786 0.0028139 -0.0438129 -0.002776399 0.070919264 -
0.239218984 -0.290160075 0.176065358 -0.193310689 -0.269235882
-0.070154386 0.055508652 0.221593385 0.151433609

19326 0.390068127 -0.174208788 -0.33355252 -0.434702867

19327 0.081522616 -0.354114733 -0.06636148 0.208879717 -

19328 0.326256431 0.083090288 -0.367959946 -0.358063634 0.293014676

19329 0.089825277 0.410538132 0.185347828 -0.096722884 -

19330 0.196135384 0.247868959 -0.448072002 0.348838297 0.349432746

19331 0.078244361 0.027909776 0.235971824 -0.109307737 -

19332 0.131041449 0.049635447 -0.006687786 0.129637988 -0.210440166

19333 0.254270949 0.175740084 -0.326796881 -0.10080732 -

19334 0.108151827 0.283357602 0.326756018 0.058576085 0.096534672

19335 -0.299622004 0.103818492 0.064302819 0.13968203

19336 0.186038325 0.119670575 -0.057425321 0.203061349 -

19337 0.341719165 -0.27599569 -0.001900667 0.219074758 0.11798964-

19338 0.327042919 0.127427883 0.146825161 -0.377029238 -0.265328617

19339 -0.149991355 0.331917131 0.12561367 0.042500629 -

19340 0.153557155 0.169274296 0.367953681 0.011769727 -0.202021218

19341 -0.066651281 0.138163818 0.225784106 0.113124 0.031491364

19342 0.056894977 0.04400997 -0.10218256 -0.078883445 -

19343 0.260553799 0.124106363 -0.395266263 0.156378259 -0.319272341

19344 -0.184496126 -0.059488294 -0.22090142 0.128676078 -

19345 0.053213034 0.31588533 -0.206886913 -0.358562608 0.276471618

19346 -0.118834759 -0.152862557 0.243839871 0.094958816

19347 0.178295768 0.102520999 -0.103651323 -0.053722316

0.314216954 -0.338797929 0.381265786 -0.204794323
0.220185272 -0.131132504 0.338759895 0.099463279
0.153354894 -0.143882753 0.294388705 0.131615339 -
0.258460502 0.083171407 -0.211932863 0.280389106 0.020174058
-0.213202288 0.300851581 -0.034166189 -0.03768493 -
0.120942404 0.120314748 -0.032339777 0.20413534 0.239891888
-0.137018691 0.127120807 0.082861933 -0.392143549
0.078008446 -0.36694906 0.244592357 0.078852955 -
0.111810593 0.151388176 -0.338612547 -0.176492886 -0.226258706
-0.324602676 0.231946424 -0.371553779 0.06713981 -
0.296135535 -0.18893245 0.322897877 -0.072720102 0.205063533
0.272038688 -0.105219882 -0.301088148 0.245109362
0.125051845 0.161828286 -0.277254163 0.426568634 -
0.264702122 -0.072749479 -0.012863766 0.299144639 -0.114742616
0.053510418 0.235005417 0.224709496 0.129819745 -
0.266633582 -0.060910655 -0.19940668 0.040125516 0.070826197
-0.295540707 -0.06375269 -0.146994775 -0.175464873
0.297842731 0.124047264 -0.164008497 0.19640521
0.124160269 0.112913 0.378524491 -0.135310804 -0.059503335
0.07887864 -0.033628915 0.169487357 -0.12890502
0.224639686 -0.180111453 -0.155110688 -0.247655494 -
0.095324132 -0.12420961 0.246492717 0.17355002 0.058373698

19370 -0.218590415 -0.158199803 -0.132624765 -0.010227837

19371 0.296948974 0.099921481 -0.04075558 -0.219970424

19372 0.183199719 0.207376402 0.260466089 -0.412675161

19373 0.21487675 -0.049596821 -0.291203075 -0.061437585 -

19374 0.033822991 -0.228646762 -0.303909781 0.166585011 -0.233421352

19375 -0.075894443 -0.151657584 0.076026296 0.135846942

19376 0.169477795 0.039278973 0.337730604 0.232171222

19377 0.363856995 0.241820522 0.168014242 -0.212565214 -

19378 0.23137954 0.204355874 0.10140156 0.083012439 -0.097410157

19379 0.364956902 -0.004398668 0.126073458 0.120269336

19380 0.275899315 0.146127273 -0.147410838 -0.122593086 -

19381 0.34319629 -0.315734382 0.251201896 -0.148981766 -0.349758967

19382 -0.149821247 -0.396362451 0.138183237 0.133681901

19383 0.00189002 0.329961725 0.040297281 0.194314735 -

19384 0.259233061 0.229862114 0.148356517 0.269398007 0.199677117

19385 0.209773473 0.353817353 -0.172101025 -0.322682687 -

19386 0.216812703 0.276853392 0.368923078 -0.440625854 -0.315393521

19387 -0.373360973 -0.133162656 0.140952067 -0.151013058

19388 0.049025299 0.253635776 0.189905246 0.244891913

19389 0.180045133 0.174749494 -0.018966685 0.20609059

19390 0.200440569 0.112423034 0.169324795 -0.30693808 -

19391 0.074107618 0.193052281 -0.0435538 0.25372323 0.195597505 -

19392 0.204119181 -0.126915382 0.049149148 -0.206739584 -0.006281459

19393 0.228548629 0.094051294 0.007535489 0.193074118 -

19394 0.323128698 0.053554765 -0.17814576 0.294134438 0.324798369

19395 0.118335213 0.14496577 -0.104656396 0.369335703 -

19396 0.174284614 -0.248858005 0.329325921 -0.006136196 0.016306631

19397 -0.355992196 0.374497262 0.032041052 0.054301024 -

19398 0.147912135 0.195048441 -0.042697296 -0.433508508 -0.19342332

19399 0.3075159 0.412056698 -0.287419471 -0.281247358 -0.055821603

19400 0.130456164 0.085856607 0.163994667 -0.321260288 -

19401 0.156895753 0.467795393 -0.270779608 -0.06897952 -0.001912982

19402 0.240720563 0.365218096 -0.377368825 -0.163778789 -

19403 0.208883442 -0.072770972 0.04776571 0.105461975 -0.32752304

19404 0.033361425 0.23106129 -0.072155498 0.346072796 -

19405 0.147508054 -0.078782081 0.279687022 0.020527408 0.141665193

19406 -0.252527241 0.060884539 0.079884698 -0.283619074 -

19407 0.032717785 0.144336368 0.275952795 -0.220005726 0.183900397

19408 -0.179962142 -0.345576879 0.179864525 0.100645795

19409 0.281864918 -0.071889573 0.024700828 -0.340107463 -

19410 0.003265436 0.017650729 0.203399051 -0.171023978 -0.104969043

19411 -0.015400464 -0.323137425 -0.227467973 0.232766479

19412 0.352120838 -0.135214587 -0.287101033 -0.290447039

19413 0.166253737 0.16060798 0.06254261 0.257726289

0.007979639 -0.128494305 0.258180541 0.098796548
0.111634671 -0.039798287 0.098974073 -0.189311367 -
0.156412218 0.016826528 -0.317001755 0.199404035 -0.400295247
-0.166718113 0.127394367 0.168305037 0.062418257
0.317465814 -0.194152101 -0.21476051 -0.363078233
0.363205193 -0.199606758 0.017921127 -0.081786022
0.391739509 0.134231791 0.20181588 0.211233195 -
0.151770916 -0.371640027 0.194566003 0.020472666 -0.267970501
-0.066369798 -0.105435509 -0.26539326 -0.131681857 -
0.010356892 -0.094035719 0.122935442 -0.090383942 0.08699933
-0.030473012 -0.003742059 0.136696896 -0.107557658
0.112764211 -0.189912746 0.02190781 -0.11891415
0.010271409 0.210222465 0.144221755 -0.082151743
0.171823305 -0.151864072 -0.220798286 -0.15147375 -
0.023264128 -0.062158359 -0.081814183 0.088078878 -0.035725958
-0.09481497 -0.191166586 -0.169180227 -0.138458333
KEGG_VASCULAR_SMOOTH_MUSCLE_CONTRACTION -0.097007133
0.057789372 0.007192853 0.309693227 -0.367378335
0.200149997 -0.005858504 -0.2000612 -0.135885713 -0.114374062
-0.104598985 -0.151842403 0.219342514 -0.136574255 -
0.085979515 -0.038630431 -0.01268286 -0.101532888 0.201354124
-0.343618707 0.377965116 -0.150789494 0.22494702

0.219372356 0.176301424 0.116901558 -0.274478029 -
0.069829041 0.083810646 0.104666663 -0.061266522 -0.182928371
-0.253770336 -0.120350731 0.333432431 -0.103578923
0.412268835 -0.185676203 0.344974218 0.019284364 -
0.013284793 0.032832702 0.223870693 -0.06895582 -0.190392524
0.052500298 0.108901069 0.128561621 -0.021135889 -
0.077842909 -0.09164507 0.00885506 0.078649967 0.340370181
0.262824256 -0.007386936 0.110837563 0.021459675 -
0.133574751 0.357038182 -0.195859704 0.346369356 -0.213332043
0.125768577 0.203262361 0.058489335 -0.289778467 -
0.12568957 0.061204434 -0.17073943 -0.284008315 0.152115086
0.115652584 -0.044046008 -0.02980692 -0.193807483 -
0.008771462 -0.276562324 -0.138371684 -0.183250943 -0.112822865
0.347651296 0.116435624 0.248853905 -0.296843468 -
0.194492395 0.055196864 0.146490363 0.081394556 -0.122245902
-0.143058468 0.327506391 0.163737659 -0.063436752
0.140009935 0.215885688 0.185309531 -0.024658336 -
0.127557429 -0.294528032 -0.207520095 -0.059471657 -0.173188752
-0.127942284 0.255574733 -0.111542157 0.217499096 -
0.371165598 0.419592888 0.204494442 0.326809807 -0.271040561
-0.168511837 0.241930804 -0.211216729 0.048498814 -
0.334782505 -0.183562261 -0.083035039 -0.330700497 -0.055517212

19458 -0.162885954 -0.214207956 0.127944314 -0.002646216

19459 0.054593218 -0.125422608 0.288980442 -0.000434192 -

19460 0.191484211 -0.203384274 -0.03304879 -0.289089153 0.112364418

19461 -0.050623038 0.32849383 0.23391001 0.249527411 -

19462 0.096118257 0.489931614 -0.221674684 -0.242857319 -0.014442188

19463 -0.021299563 0.000110958 0.117017293 -0.04356892 -

19464 0.140224769 0.207816235 -0.12126493 0.301732694 0.017219309

19465 0.184250126 0.301514268 0.10994038 0.085742382

19466 0.16222146 -0.258094291 0.016997509 0.079996232

19467 0.198795588 -0.218254943 -0.241430509 0.086710794 -

19468 0.137895082 0.196616089 0.161391718 0.269635358 -0.068747202

19469 0.18152699 -0.023202827 0.101756367 0.100245692

19470 0.107499392 0.408884748 0.091519199 0.058365916 -

19471 0.125748468 0.197590326 -0.166701929 -0.076602796 0.127735318

19472 -0.067194027 -0.001021701 0.271137078 0.299418784 -

19473 0.159058303 -0.028937976 0.095845465 0.114530675 -0.308984521

19474 -0.15082213 -0.050898804 0.154617588 -0.249449301 -

19475 0.013679831 -0.099922464 0.025802627 0.017653977 0.008551545

19476 0.182370387 -0.227679562 0.327785677 -0.081583537 -

19477 0.156520318 -0.253086705 -0.033411756 -0.230641998 0.245271134

19478 -0.326145526 -0.118383929 -0.192594328 0.220343962

19479 0.019152471 -0.045059844 -0.059811657 -0.25237226 -

0.032579003 -0.184699587 -0.099977869 0.329857888 -0.015966794
-0.103543568 -0.315384299 0.005437035 0.128591906
0.037399995 -0.176604146 0.052303887 0.273402733 -
0.363054811 -0.262045437 -0.130601537 0.221811528 0.063902084
0.326758438 -0.089730749 0.196011529 -0.112569005
0.013531309 0.072271543 -0.13346304 -0.057230465
0.241123184 -0.350366604 0.340449195 -0.234034985
0.196585743 -0.270365195 -0.076456278 0.090235492
0.168824834 0.156267703 0.22652663 -0.078610054
0.276419996 0.324895031 0.256232245 -0.104793856
0.106209737 -0.186448763 -0.321647285 -0.184358433
0.171071439 -0.003705365 0.285401107 0.170185152
0.087367476 -0.235781966 -0.010718732 -0.222830953 -
0.29013227 -0.374367824 0.021567168 -0.142504905 -0.262659973
-0.151264242 -0.193949553 0.035287253 0.214622184
0.359587866 0.016089293 -0.270332781 0.195748681
0.108327513 -0.293583253 -0.050804288 -0.200001192
0.119920008 0.191789614 -0.191334599 0.155264343
0.115771512 0.254372998 0.04695664 -0.170375478
0.197794499 0.300621121 0.054517487 -0.19983577 -
0.162317611 -0.258468972 0.077752055 0.132223212 -0.250426969
0.02353785 0.258275236 0.036565543 0.41874411-0.190911997

19502 0.252172987 -0.257183764 0.269038748 0.237480055 -

19503 0.256812778 -0.279682808 0.320491714 -0.018891336 -0.020808584

19504 0.119886865 0.194500051 -0.159187886 0.084883566

19505 0.255953667 -0.304166735 0.120736796 -0.029663828 -

19506 0.188108332 0.2243507 0.111355164 0.021276665 0.238730461 -

19507 0.032490114 -0.031209997 0.276701717 0.110696366 0.160376329

19508 0.057091723 -0.091572732 0.231640237 -0.238573084 -

19509 0.05007729 -0.134420644 0.336473441 0.064340722 -0.341645492

19510 0.116001073 -0.037433143 0.328252736 -0.138225072

19511 0.302328803 -0.260594759 0.178647623 -0.335473382

19512 0.055806833 -0.2005131 0.183426529 -0.189373764 -0.363337486

19513 -0.106515294 -0.098625932 0.197040832 0.160640795

19514 0.309511365 -0.244090801 0.208596318 -0.341415829

19515 0.245743951 -0.106623408 0.278063144 0.091185922

19516 0.27193769 0.218401247 0.170198452 -0.039294835 -

19517 0.09368312 -0.091939312 -0.23935913 0.190343997 -0.08839662

19518 0.021550639 -0.1073944 0.09871987 -0.145634858 0.26301323

19519 0.155898321 -0.017940563 0.279638959 0.245713879 -

19520 0.06681169-0.325228839 -0.146713655 0.113916646 -0.007155326

19521 0.373089979 0.34793233 0.094826112 -0.191073427

19522 0.338257828 0.295101655 -0.059302654 0.120498552

19523 0.241513854 -0.142605846 0.052426802 -0.182145623 -

0.076616537 0.257561399 -0.269280814 -0.064033399 -0.265769914
0.348258961 -0.211982394 0.337349424 0.091363679 -
0.063876309 -0.053755035 -0.244484984 0.201934722 0.116417868
-0.21239072 -0.230462027 -0.310978128 0.16207907
0.204232279 -0.345456678 -0.147117159 0.132205307 -
0.156795488 -0.00427774 -0.404568927 0.275914899 0.071817342
-0.013287925 -0.19040089 0.125848777 0.164389836
0.004313112 -0.290496751 0.135448934 0.098915865
0.28267104 -0.239145906 0.275987463 0.20276811-0.004706803
0.277201949 0.069750224 0.015126358 0.152253186 -
0.200804001 0.143909127 -0.075110605 0.078049115 -0.343140722
0.069646828 0.134162368 0.301734526 -0.060326265 -
0.141655711 0.121739758 -0.024347696 0.027910421 0.345424794
0.20576527 -0.231861547 0.04600395 0.067112536
0.245954854 0.029908335 -0.278477123 -0.315513873
0.077300028 0.362952318 -0.342948859 -0.149859904
0.175899211 -0.112936522 0.022778552 0.30888855
0.204267252 -0.115640436 -0.219767892 -0.027021269 -
0.241580876 -0.31076739 0.277958001 -0.349270818 -0.065995028
0.061778585 -0.076245731 0.17968816 0.112653978 -
0.001991687 0.069879643 -0.196832917 -0.117293125 -0.008720738
-0.401103908 0.013539983 -0.088945956 -0.041309909 -

0.017250135 0.408586596 0.39702441 0.121664924 0.233901217
0.269056931 0.065546714 4.41E-05 -0.135131931 -0.283359711
0.423025011 -0.034626034 0.183661347 -0.289723645
0.110412445 0.171771043 -0.073982769 0.162623767 -
0.377445775 0.099616236 0.063211691 -0.322396604 -0.158283036
-0.191073393 -0.103199932 -0.030686434 0.261273913
0.306259565 -0.110470757 -0.233132017 -0.164951594
0.075036754 -0.142151904 0.148723278 -0.006170696
0.01935965 0.013738865 -0.10103765 0.0898596 0.190051178
-0.116952816 0.182296683 -0.312333074 0.006769099
0.050842837 -0.1133984 -0.06245057 -0.293737954 -0.123536523
0.246894655 0.013818191 0.092283893 -0.191342284 -
0.097974583 -0.033917399 -0.005144818 -0.28585772 -0.081813617
0.211549511 0.107370123 0.112221208 0.063413953
0.048836195 0.10716425 0.103790122 -0.070019524 -
0.111501495
KEGG_WNT_SIGNALING_PATHWAY -0.083806884 0.028067106
0.240076377 0.27171893 -0.088137351 -0.018025701 -
0.103281067 -0.150972968 0.016480729 -0.170333393 0.061108786
0.009882454 0.105634985 0.0650098 -0.238467339 -0.103789362
0.128270889 -0.006474521 0.023251002 -0.21877724
0.319510078 0.0473844 0.306871943 0.127936628 -0.063063403

19568 -0.073928423 -0.322497815 -0.102938865 -0.0512353 -0.232439114

19569 -0.091417717 -0.23802845 -0.188591773 -0.271452203

19570 0.24900073 0.043208844 0.236607032 -0.116992462

19571 0.08343977 -0.125796456 0.001424761 0.04803812

19572 0.196942505 -0.113161464 0.127304665 -0.009946345

19573 0.099911055 0.105423669 -0.044815214 -0.105108049 -

19574 0.142897295 0.307664118 0.012679468 0.195054876 0.011891002

19575 0.011244282 -0.052496087 0.073893609 -0.120891702

19576 0.295626209 0.128117465 0.408850725 -0.036325049

19577 0.064643425 0.106615133 -0.028799263 0.153141753

19578 0.257821544 -0.04321994 -0.212732485 0.113058474

19579 0.034633687 0.115520101 -0.154540455 -0.057472815 -

19580 0.210098524 -0.281240083 -0.317451162 -0.143277836 -0.227807341

19581 -0.149627645 0.233659505 -0.056318949 0.112275866 -

19582 0.101507915 0.004779665 0.223275597 0.23263739 -0.115732248

19583 -0.259651451 -0.007988588 0.031600758 0.161145289 -

19584 0.083651621 -0.113778031 -0.001621007 0.101583728 0.032414413

19585 -0.204977891 0.061902708 -0.11756527 -0.128727805

19586 0.119904624 -0.229888519 -0.14508865 -0.130181831

19587 0.015167092 -0.129821842 0.34381699 0.060727989

19588 0.169203919 -0.212412408 -0.052899547 0.381397662

19589 0.018109082 -0.174331789 -0.239272118 -0.253386369 -

19590 0.148796867 -0.068339228 -0.20354465 -0.208060921 -0.202308993

19591 0.100926205 0.066400744 -0.261546985 -0.231647506

19592 0.27217934 -0.067738334 0.056901213 0.178991986 -

19593 0.078214432 -0.230821733 0.000655112 -0.160306686 0.323645808

19594 0.345270389 0.059117569 0.012975394 0.367341885 -

19595 0.160998714 -0.251946807 0.194219222 -0.126190532 -0.059613367

19596 0.293955682 0.032229513 -0.221520144 -0.21870702

19597 0.012076084 0.060274671 0.249066393 0.125112690.203301259

19598 0.076709289 -0.120975014 0.167569443 -0.361143965 -

19599 0.094721261 0.079824844 0.220565499 -0.260477299 -0.064491811

19600 -0.183222953 -0.072401841 0.089347287 0.134769306

19601 0.202592222 -0.162035511 0.136174152 0.031509321 -

19602 0.231254629 -0.082320889 0.117627684 0.355293261 0.305959776

19603 0.147796189 -0.037630927 0.053208106 -0.085369702 -

19604 0.122066337 0.117397709 -0.223307403 0.027401498 0.208799619

19605 0.015556169 0.160813247 0.136070652 -0.083132247

19606 0.114404007 -0.046202069 -0.276943447 -0.024924005

19607 0.002186555 -0.327299758 -0.089792074 -0.121605627

19608 0.070960189 -0.155109527 0.134756489 0.127673999 -

19609 0.242902003 0.236103473 -0.209071146 -0.090094245 -0.297000545

19610 -0.134550714 -0.21775805 0.286255244 -0.177232364 -

19611 0.092804985 -0.015899761 -0.013224884 -0.094944071 -0.258456331

-0.252577829 -0.280954086 0.147259325 -0.031310652
0.001493696 0.162541635 0.078715472 -0.013664867
0.042032495 -0.043324642 0.147504027 -0.248706189 -
0.281789869 0.131741167 -0.110397122 -0.305008573 -0.004318008
-0.103649001 0.093574643 -0.166279456 0.307520644 0.0593423
0.107596633 0.082647916 -0.229310755 0.21736869
0.083443268 -0.039675583 0.167922141 0.158074677
0.100586936 -0.259711408 -0.074526438 -0.158112298 -
0.190836855 -0.066263867 0.047814911 -0.136274197 0.310879534
-0.076620304 0.374434714 0.115373041 -0.019145843 -
0.06506187 0.024988963 -0.252870842 -0.120271384 -0.12012581
-0.013920628 0.060269397 0.211520576 0.158260911 -
0.154343176 -0.024709175 -0.289760765 -0.272431329 0.150744882
0.086386894 -0.029497149 -0.277796086 -0.161016088
0.042516956 -0.160711281 -0.071776963 0.024594415
0.034250134 -0.088826477 0.115088976 0.025372732 -
0.008741341 -0.273701406 -0.148947798 -0.234791937 0.197810269
0.037235315 -0.103614939 -0.136225446 0.105452734
0.337795831 -0.061575404 -0.204686377 0.164020601 0.0966275
-0.024824244 -0.159754114 -0.023687499 -0.169813349 -
0.082687699 -0.074188643 -0.322445986 0.14911194 0.141140025
0.05786307 0.225969707 -0.239248109 -0.124195276 -

0.194094791 0.082627491 0.23796432 -0.185218805 0.026848848
0.015353475 -0.126683311 -0.101618356 0.048178255
0.012722559 0.089135312 0.19525943 -0.107106746 -
0.277941107 0.099211573 0.062066275 -0.297634242 0.228438762
0.132553124 -0.019468069 0.14678218 0.07939291 -
0.120914297 0.291945421 -0.065173352 0.238915081 0.197165958
0.077997884 0.213676288 -0.166496566 -0.189875676 -
0.179574131 0.291871681 -0.046680423 -0.27630802 0.112483588
-0.215405323 -0.064098913 0.035795785 0.307480098
0.04274829 0.221776708 -0.322528701 0.039022056 -
0.226055444 -0.102054774 -0.095333483 -0.236574744 -0.226119016
-0.093943612 0.085638311 -0.027066757 0.198459382 -
0.287435116 0.416735503 -0.192282335 0.234357462 -0.09190016
0.229275152 0.147522853 0.447102479 0.207061249
0.21665753 -0.059351276 -0.149631727 -0.214315492 -
0.269946433 0.016341817 0.044240609 -0.044191357 -0.094919442
0.021687806 -0.169599804 0.150473356 0.072844776
0.162373654 0.162016135 0.146440773 0.008793869 -
0.279167853 -0.123508308 -0.023600083 0.118506416 0.307936167
0.397076698 -0.171717892 -0.136964052 0.312565809
0.186639846 -0.038510958 0.081868889 0.337115621 -
0.206216451 -0.020588036 -0.241039642 -0.119923943 0.027896498

0.129846207 -0.01811703 -0.230644557 0.22821428 -
0.261722703 0.017348982 -0.100639403 -0.022716359 -0.040596341
-0.206542587 0.232472065 -0.170018408 -0.057004989
0.105844722 0.147417278 0.290569683 -0.128203229
0.101399202 -0.235014401 0.326162056 0.214545422 -
0.003169911 -0.097646274 -0.022722714 -0.045137214 -0.135327881
-0.017528001 0.250618502 0.162254384 -0.100150128
0.042760787 0.326523963 0.105081711 0.392506663 -
0.215622527 0.107775877 -0.030514331 -0.03685088 0.11247945-
0.073649634 0.080495577 -0.101047997 -0.044773247 -0.128426422
-0.013239282 -0.013574578 -0.304786663 0.164056541
0.141359916 0.306024981 0.09346583 0.045011511
0.091968954 -0.069671351 -0.065835354 0.312801084 -
0.032346513 -0.123247674 -0.087469096 -0.030250795 0.214280288
-0.00814345 -0.065768923 -0.054699205 0.031417805
0.286464758 0.009470205 -0.070131253 0.034321458
0.044564379 -0.123547335 0.113727105 0.059553643 -
0.143173696 -0.077994392 -0.131171898 -0.221661882 -0.240226485
0.181294147 -0.25306929 -0.05643559 0.187262856 -
0.084925133 0.155538275 0.117606750.028659949 0.086740958 -
0.215523521 -0.201806491 -0.033225473 -0.048230504 -0.204564335
-0.127524308 0.036714652 0.202353161 0.275076425

0.455928536 0.139299508 -0.158901037 0.32170288 -
0.155752735 -0.069404248 -0.050156582 -0.170429297 0.397765046
-0.081197034 0.001057019 -0.045833811 0.042495273
0.020353175 -0.008885218 -0.070538192 -0.194068047
0.045968967 0.076070205 -0.064797667 -0.148454286
0.27187486 0.027157295 -0.109249852 0.297982110.291932106
0.102937709 -0.089863993 -0.123475332 0.269709804 -
0.157509088 0.128926229 0.196276878 0.004538337 0.011823999
-0.022879243 -0.064174296 -0.048388431 0.094535114
0.173355843 -0.18002615 -0.197001726 -0.052369879
0.048623313 0.021065339 -0.214337377 0.087799271
0.105388253 0.07529726 0.007267982 -0.159068644 -
0.20752735 -0.011469751 0.004827117 -0.242469709 -0.165348055
-0.047505281 0.038373911 0.202638276 -0.189679859
0.172231537 -0.026229009 -0.069073984 0.092792148 -
0.020843033
KEGG_DORSO_VENTRAL_AXIS_FORMATION 0.010180968
0.283101543 0.076703925 0.312380059 -0.27813869
0.403984636 -0.079865524 -0.134968139 0.205714977 -
0.145629325 0.055764272 -0.399787131 0.26498067 -0.142234138
-0.437240386 0.15693608 0.395622432 0.018830043
0.34571005 -0.323200688 0.212711469 0.212290914

0.021925881 0.270387126 0.025137007 0.110736019 -
0.249944125 -0.069125095 0.180927258 -0.184824503 0.150650685
0.131207235 -0.072886526 -0.240113016 0.294835239
0.307962712 0.155869665 -0.205698701 0.201812614
0.044499583 -0.077905429 0.194235789 0.161128576 -
0.060646881 -0.364590815 0.22776834 0.016668312 0.055194676
0.122027598 0.062764782 -0.315839359 0.146473159
0.146083649 0.220921084 0.122337957 0.238454335 -
0.111929836 -0.098599368 0.013081659 0.409148918 -0.16542624
0.171440522 -0.004598972 0.098029197 -0.048681123
0.338544346 0.148714739 -0.152531499 0.160892027 -
0.198502233 0.040726003 0.108716017 -0.111560524 -0.154755247
-0.074812446 -0.157948469 -0.355533645 0.258391423 -
0.240285406 -0.320910839 0.134415433 0.433720244 0.166503466
0.095452364 0.173107617 0.206595596 0.102902943
0.192376327 -0.090081667 -0.013778818 -0.270345784 -
0.136135363 0.037370536 0.050094778 0.032917087 0.037569096
0.080764249 0.180042854 -0.092142078 -0.433913419 -
0.125342364 0.088032247 0.213237596 -0.033072121 0.024874137
0.110916623 0.255654819 -0.39522471 0.283988665 -
0.309023268 -0.033507948 -0.345351681 -0.19425297 0.071978843
0.182429981 0.137329922 -0.341570929 -0.235501266

0.105818546 -0.350736658 -0.126727013 -0.178725294 -
0.261215348 -0.240297814 0.283238553 -0.174859299 -0.406877934
0.452425786 0.128735961 -0.12364054 -0.16297632
0.007751565 -0.161256387 0.325089525 0.279745475
0.152422539 0.28310101 0.029827506 -0.028573003
0.335663907 0.036004986 0.128671661 0.007496318
0.166564564 0.076996407 0.075586824 0.151341688 -
0.199708017 -0.012417809 -0.433854562 -0.255513505 0.218720423
0.194348191 0.100228516 0.257703977 0.079699002
0.084268298 -0.12728033 0.233326336 0.019674019 -
0.051180815 -0.064354063 0.168104248 0.243548193 0.161782796
0.174617369 0.159812518 0.312957928 -0.153885518
0.166821579 0.34196991 0.091289105 -0.122889789 -
0.208550742 0.301287656 0.142790362 0.290901959 0.088438371
-0.122706011 -0.071034614 -0.315937809 -0.074766646
0.007373152 0.096772921 0.23175831 0.099378486 -
0.085271245 0.197410704 0.270841 0.184851193 0.136587993 -
0.43145098 0.196571104 -0.020881544 -0.50755518 0.124574366
0.223340805 0.199537199 0.162987408 0.330411284 -
0.219771319 -0.108919546 0.415273179 -0.247023761 -0.216870382
0.004249506 -0.071448525 -0.079592982 0.211651133 -
0.321878227 -0.077696568 0.063158898 0.136307514 0.327999328

19744 -0.258353166 -0.244890228 0.070583788 0.4514265 -0.114906661

19745 0.1985792 0.011733266 0.23558487 -0.150465515 -0.217661433

19746 0.254062612 0.117834732 -0.169896559 -0.201342926

19747 0.259764781 -0.143439302 -0.299325736 0.018693897 -

19748 0.044678148 0.336539761 0.206029097 0.204314079 0.315996981

19749 0.162147654 0.137860216 -0.415561705 -0.269350079

19750 0.131313839 0.165984891 0.29522641 -0.266460984

19751 0.368656594 -0.032252842 -0.006587692 -0.406091922 -

19752 0.052970588 0.29192617 0.0861545 -0.081814828 0.287380395 -

19753 0.248690155 0.330706789 0.01624443 -0.301958641 0.134064271

19754 -0.061565015 -0.07775364 -0.257278004 0.213852087 -

19755 0.123683838 -0.319669728 0.275497411 0.29320951 -0.156191859

19756 0.184733445 -0.25023142 -0.398989916 -0.213232188 -

19757 0.141860056 0.090663003 -0.317473227 -0.332568216 0.041766946

19758 -0.142782077 0.129553618 -0.323120814 0.130796161 -

19759 0.150615364 0.118722741 -0.007293828 -0.226703995 -0.387360623

19760 -0.13267258 0.086700578 0.434025372 -0.309799274

19761 0.279673418 -0.032714002 0.091861852 0.177817473

19762 0.127154066 -0.172382555 0.10617701 0.149598164 -

19763 0.035299789 -0.248148594 0.350082881 -0.455282282 -0.166737674

19764 0.122823077 -0.367647279 0.34279724 0.347435989 -

19765 0.041079727 0.143899349 -0.159035795 0.370143659 -0.213630937

0.103330805 0.33139356 0.025449964 -0.247113702

0.195668573 -0.077622424 0.07337562 0.246075988 -

0.229345255 -0.214914049 0.07498222 -0.074045061 -0.258744573

0.331265227 -0.043259389 -0.200933674 -0.160964154

0.101565875 -0.150268777 -0.109319878 0.010621568

0.042568636 0.426571528 -0.119680252 0.24277774

0.114669794 0.174943233 -0.037441974 -0.137542384

0.115902403 0.054308117 0.284259956 0.138429554 -

0.105797146 0.459990492 0.042028329 0.213755098 0.114671101

0.41890785 -0.156330093 0.165342415 -0.452847131

0.069394673 -0.087486087 0.008831373 -0.022616077 -

0.343075904 -0.325526394 -0.381233027 -0.002045874 0.354014234

0.490624235 0.195277686 0.444665848 -0.153225647

0.145432891 -0.136889741 0.205050089 -0.024063315

0.411481307 0.44785643 0.350576106 -0.213032984 -

0.028858659 -0.172811832 0.092719237 0.369767352 0.132904603

-0.115222177 -0.237210629 0.180904513 -0.246668976 -

0.07413654 0.061109262 0.149885828 0.440003076 0.236053213

-0.017177426 -0.198898923 -0.198858987 0.068051296

0.317131824 0.384911052 0.193034967 -0.11122642 -

0.460479558 0.406632332 0.098846659 0.151715435 0.428362173

0.341859679 0.05416685 -0.057838035 0.009572887

19788 0.206021676 0.331937986 0.156118681 0.389228884 -
19789 0.37635121 0.25690871 -0.2595721 0.354383228 -0.276619068
19790 0.01668815 0.315448393 -0.211001259 -0.061904799 -
19791 0.049130164 -0.291545448 0.041388815 -0.411565963 0.086802623
19792 -0.187662374 -0.470231718 -0.36018577 0.136803664
19793 0.011624914 0.119317329 -0.357338833 0.131395017
19794 0.274780246 -0.125951106 -0.102392318 0.158571296
19795 0.153277767 -0.338981821 -0.448695613 0.181827468
19796 0.380078941 0.342270495 -0.018905914 -0.231328648 -
19797 0.060016433 -0.061705978 -0.080334753 -0.138243874 -0.061577233
19798 0.258610401 0.330040119 -0.006057109 0.263803967 -
19799 0.221687986 -0.3566214 0.15634803 0.173857499 -0.06242665
19800 0.134553443 -0.017154542 0.238029134 -0.150338697 -
19801 0.239544204 0.273178848 0.030496379 -0.210687201 0.272426398
19802 0.086990496 0.227374619 0.084761539 -0.0568559 -0.178598277
19803 -0.111455635 0.535963641 -0.342330428 -0.181939886 -
19804 0.102319601 -0.061489698 0.045678321 -0.011832746 0.333623976
19805 -0.23645712 0.069726321 -0.173374428 -0.368425558
19806 0.148288439 0.251184191 -0.155946179 0.074737964
19807 0.137829471 -0.352977343 0.325507676 0.277354963 -
19808 0.038547001 0.239431625 -0.113054032 -0.001976565 -0.027197734
19809 -0.458301332 -0.424071755 0.203917618 0.161493699 -

0.064551592 0.31957711 0.210084005 0.247505921 -0.227150661
0.285122967 -0.201432315 0.281401293 0.100773786 -
0.198259554 0.402650682 -0.236860927 -0.1413263 -0.243155703
0.069752274 0.1357485 -0.091449878 -0.255229513 -0.336664607
-0.230420837 0.18949608 -0.444574175 0.146991945
0.044643379 0.172544555 0.073276829 0.419041627
0.270182914 0.006076863 -0.00094627 0.034647562
0.044103269 -0.178855192 0.15224976 0.084633205
0.001390064 0.023249991 -0.275509533 0.145797439
0.038930922 0.137768805 0.193234298 -0.162299496 -
0.022344689 -0.069821184 -0.413135519 -0.10495042 -0.07373771
0.102893308 0.119850849 0.022892459 -0.063581444 -
0.26942894 -0.074722261 0.011887221 -0.316395305 -0.102673006
-0.085679914 -0.162151495 -0.014570748 0.261339621
0.283286034 0.130072763 0.228218058 -0.130083934
0.207880062 0.295917049
KEGG_NOTCH_SIGNALING_PATHWAY 0.031075181 0.099105268
0.398507462 0.348372559 -0.259748959 -0.135467672 -
0.256272785 -0.109874203 -0.077816156 -0.236781482 -0.414447733
-0.395393717 0.10426233 0.283685271 -0.109746343 -
0.047265873 0.427400756 -0.085425927 0.024597953 -0.134790013
0.393367348 0.154153123 0.383168152 0.444222771 -

0.037645711 -0.200820777 -0.495362003 -0.22691479 -0.190836029
-0.125699417 0.367788053 -0.143215506 0.182594056 -
0.263712604 0.256993702 0.362330276 0.338150219 0.149108532
0.394882638 0.18051408 -0.436178423 0.171664444
0.543599023 -0.285058973 -0.321220439 -0.010407846
0.426150549 -0.45514934 -0.021812627 -0.015191571 -
0.451260522 0.003245259 0.302975652 0.370535529 0.217808278
-0.377588793 0.142008689 0.164620692 0.17345269
0.359926749 0.29496532 0.155170511 0.09262703
0.451313861 -0.118569741 0.225521505 0.220279218 -
0.2495939 -0.305101913 -0.179287862 -0.00404167 0.101914009 -
0.433227028 -0.297350226 0.031479473 -0.314045302 -0.461882501
0.033176073 -0.196810639 -0.104434361 0.2799323 0.338473122
-0.425288066 0.192499287 -0.20766584 -0.034508294
0.396418936 -0.142093996 0.079161853 -0.143638838
0.137120795 -0.116088052 0.487821262 -0.144376291 -
0.141002924 0.239787547 -0.303126437 -0.167161814 0.03858449
-0.134544998 -0.274776324 -0.199507071 -0.308097433 -
0.322610085 -0.319317586 -0.063092809 0.145373331 -0.510837445
0.463588673 0.006492897 0.076829882 -0.235312332 -
0.068886136 0.313661082 0.106440947 -0.225709679 -0.033089363
-0.175747249 -0.148735473 -0.175191599 0.201712925

0.031681306 0.155022474 -0.357207116 0.108283943 -
0.320403722 -0.586073196 0.353961963 0.199950245 -0.225479231
-0.41368445 -0.336986477 0.160102632 -0.071215914
0.133872492 0.336064625 0.390931307 -0.002866049
0.059328998 0.415320795 -0.240895124 -0.456283086
0.437339584 -0.018598288 -0.166273573 0.177265818
0.168462157 -0.332238166 0.118086434 -0.075463628 -
0.122190639 0.348207167 -0.017334678 0.34426794 0.303228364
-0.107845622 -0.071648137 -0.415235536 -0.212001406 -
0.300092539 0.196198165 -0.178859634 -0.127655974 0.163855528
0.139706258 0.270436705 0.126704867 0.291689249 -
0.233530798 -0.302173364 0.464774018 -0.151518141 0.309142076
0.368884629 0.516392977 0.25225067 0.074332204 -
0.154739549 0.337296638 0.065914241 -0.290846117 -0.29242412
-0.108809629 -0.207933615 0.50684012 0.080211342
0.253168118 -0.346569665 -0.124148008 0.084760272 -
0.209436496 -0.467401372 -0.196198773 0.048167879 -0.34461295
0.043990617 -0.302113029 -0.15356493 -0.06239286
0.341852252 0.093714042 -0.372247571 -0.15768662 -
0.567938005 0.040799176 -0.168481509 -0.210703716 -0.365367596
0.3903365 -0.446462188 -0.017486159 0.340960173 -0.311294415
0.050299212 -0.366257262 0.025670254 -0.285192264 -

19876 0.068857228 -0.196993736 0.178617815 -0.042581969 0.128522302

19877 0.345594376 -0.083023775 0.144004894 -0.088028517 -

19878 0.326044587 -0.320270074 0.288925236 -0.535145662 -0.461531204

19879 0.102517156 0.099089897 0.002661346 0.179876605

19880 0.082673444 -0.076577505 0.310750809 0.051805544 -

19881 0.145194936 0.000909131 0.385213411 0.072218366 0.177490278

19882 -0.068611663 0.216760923 -0.448065123 0.1444975 -0.367297448

19883 -0.205185627 -0.104748081 0.280438234 0.198269081

19884 0.400052977 0.014485063 0.33483611-0.252483267 0.122154939

19885 0.106941585 0.241352694 0.051944906 -0.064900685

19886 0.284681727 -0.227201483 0.190498454 0.387795196

19887 0.341476736 -0.206098094 0.270987579 -0.302789433 -

19888 0.419029243 -0.073251078 -0.210834489 -0.414420864 0.066071913

19889 -0.1682312 0.363851339 0.140146284 0.110279312 0.195385071

19890 0.165915288 -0.005249844 0.015708974 0.08581335 -

19891 0.170542486 -0.193134207 -0.282212662 -0.253431188 -0.041807118

19892 0.178935981 0.271816187 -0.103895089 0.113068972

19893 0.068263477 -0.183332857 -0.269791256 0.45338794

19894 0.068003569 0.311729074 -0.142820626 -0.043360378 -

19895 0.382058439 0.355531807 0.109948744 -0.27496918 0.154221817

19896 -0.04304085 0.237305011 0.31335463 -0.24734208

19897 0.076148635 -0.238098075 0.081968993 0.402971386 -

0.195647222 -0.077556091 0.183208412 0.222091285 0.345232544

0.094067242 -0.354409688 0.110200965 0.418663314 -

0.238699453 -0.208623752 -0.000131827 -0.002665888 -0.247642497

0.267447321 0.128262625 0.06847576 0.337885088 -

0.021360163 -0.323596363 0.539171286 0.407212374 0.376135454

0.391744522 0.214316475 0.220211177 -0.322854755 -

0.07730391 0.099197957 0.182874248 -0.070515421 -0.423413726

0.349811234 -0.059297184 -0.039528557 0.317514961

0.44617066 -0.272147518 0.521900709 -0.527391762

0.296324588 0.002697255 -0.150193646 -0.131968566

0.104486673 -0.519444856 -0.07548473 -0.355451557 0.3086151

0.464771206 0.112796215 0.281863078 -0.071161396

0.517911922 0.273950237 0.325678602 -0.086018788

0.549113107 0.511988387 0.288558156 0.042820331 -

0.008819682 -0.10000153 0.011969633 0.354714418 -0.124073614

0.274678031 -0.000788177 0.289180638 -0.003116011

0.079367016 0.320631085 -0.350900399 0.181684932

0.236884765 -0.111934378 -0.078403807 -0.237469966 -

0.278856653 -0.258937887 0.277071847 0.178174664 -0.200083444

0.064294109 0.213310907 0.426737711 -0.377035493 -0.158702

0.285992282 -0.355474023 -0.308526597 -0.125673229 -

0.03964176 0.168537345 -0.185247368 -0.184548811 -0.032332124

19920 0.403760226 -0.398447494 0.240315055 0.121933487

19921 0.409571984 -0.282728592 -0.518738855 0.175174663

19922 0.154397568 -0.106550624 -0.023621133 -0.230787087

19923 0.546859495 0.03949408 -0.108589772 -0.461260507

19924 0.235601787 0.040162305 -0.050913863 -0.446571326

19925 0.04046585 0.286882533 0.013567013 -0.31058145

19926 0.183088307 0.278770473 -0.347212185 -0.146545884

19927 0.292387821 0.35786246 0.315889086 -0.193019523

19928 0.136461581 -0.101731799 -0.149226076 -0.402543652

19929 0.015322307 0.024445899 0.170237377 0.020850309

19930 0.284306437 0.106741165 -0.106248249 -0.372083184

19931 0.388949392 0.071172544 0.274846211 -0.04442648

19932 0.023328918 0.102595 -0.13349392 -0.18250677 0.147147296

19933 0.483869867 -0.441286951 0.017072053 -0.144841292

19934 0.360424193 0.220481269 -0.356315175 -0.34087514 -

19935 0.123681708 0.381996691 0.051888295 0.313627954 0.261925262

19936 0.084492261 0.246181273 -0.153509667 0.501743899 -

19937 0.150099225 0.185924328 -0.274924567 -0.499832382 -0.252378026

19938 0.06461805 -0.402310514 -0.120915045 0.432336125 -

19939 0.301437019 -0.110452307 0.189955798 0.146605809 -0.371273688

19940 -0.237845972 -0.368909586 0.091432398 -0.25064614 -

19941 0.566150066 -0.153715846 0.410058751 0.28443379 0.49045057

0.427543828	0.058304119	0.049204939	0.074568429	-
0.355188934	0.434341951	-0.002728429	-0.296910112	0.220780275
-0.332986941	-0.062914796	-0.311997151	0.156778134
0.475839418	-0.008472051	0.097455373	-0.271029511
0.165583196	0.206728349	-0.221625983	-0.133660919
0.261998691	0.071375153	-0.02241973	0.400722523
0.442396257	0.092429876	-0.244643255	-0.262675542	-
0.050984832	-0.439245915	0.146589489	0.241931256	0.059957792
-0.050841223	-0.104639928	0.187462092	-0.156692754	-
0.127027591	0.120673612	0.192699079	0.153756021	0.229217723
-0.363490626	0.032690149	-0.348870984	-0.149168406
0.155858595	0.042138592	-0.077042057	-0.201642171	-
0.223970484	-0.024656856	-0.11571298	-0.29013916	-0.512297294
-0.060946577	0.05319517	0.250250631	-0.133762881
0.183007482	-0.13118701	0.048941901	-0.254615841	-
0.207720866
KEGG_HEDGEHOG_SIGNALING_PATHWAY	-0.209816929	-0.330548303
0.140592719	0.095988149	0.111022123	-0.013587787	-
0.234223328	0.225018118	0.024224689	0.191814497	0.129707961
-0.2794302	0.094586298	-0.151231751	0.330224031	0.135344294
-0.136481583	-0.21449303	0.393527127	-0.163928588	-
0.017858062	-0.25458506	0.272603959	-0.340585443	0.101587248

0.07163346 -0.193450129 -0.085143014 0.138320742
0.099630392 -0.172828954 -0.213166283 -0.329941626 -
0.042835957 -0.014438469 0.186789874 0.232199667 0.207060854
0.142110472 -0.289198717 -0.293188671 0.239086367 -
0.136576539 -0.290045348 -0.023653889 0.126566604 -0.221383674
-0.099329519 -0.050169389 -0.016514278 -0.368966763
0.289685216 -0.40398453 0.134132515 -0.115213463
0.018072868 0.31820087 0.084803928 -0.345046325
0.335457224 0.154466007 0.477006873 -0.301078419 -
0.10781933 0.198748822 -0.067864634 0.318206725 0.275858752
0.120576598 -0.074685252 -0.021096444 0.056678524
0.270643932 -0.115212644 0.039718205 0.059542387
0.182471209 -0.35345107 0.250196812 -0.312642977
0.116283983 0.343517617 -0.018986176 -0.21137063 -
0.12276895 0.163600255 -0.165986174 0.356633321 0.30353948
-0.339036891 0.0180095 0.005716633 -0.118957248 -0.116786232
-0.204030303 -0.006765391 -0.135004898 0.242143348 -
0.218903796 -0.028053479 0.036712457 0.252688286 -0.20323243
-0.219338293 0.134825987 0.029021644 -0.166417669 -
0.249266769 0.142669781 0.118431571 0.291994991 -0.244648297
-0.275619301 0.067696792 0.031700631 -0.410361288 -
0.31773368 0.122521794 0.191206315 0.12165197 0.020555876

19986	-0.271968854	-0.234607626	0.232488717	0.19121224	
19987	0.137996305	-0.139922569	0.17774685	-0.335980275	-
19988	0.135915315	0.227014787	0.20641082	-0.158421578	-0.168053956
19989	-0.14346081	0.305623691	0.314706374	-0.207877985	-
19990	0.080729055	0.159953295	0.185843158	-0.004649655	-0.145538881
19991	0.112573523	-0.300927552	0.015162644	-0.136302925	-
19992	0.066909471	0.156321903	0.208512604	0.263225279	0.103333281
19993	0.271759588	0.161073487	-0.125763526	0.1754069	0.190966441
19994	-0.045760925	-0.098196304	0.227469442	-0.049754226	-
19995	0.00377362	0.033894183	-0.05292912	-0.134730521	0.139779516
19996	-0.129060785	0.217481602	0.245039849	0.149116339	-
19997	0.426633719	0.087401889	0.017537808	0.125656059	0.195975143
19998	0.149300013	0.021187941	0.08647722	-0.134348161	-
19999	0.049169751	0.192501266	0.011172908	-0.194899422	0.009908525
20000	-0.107534669	-0.168901924	-0.186794977	-0.165926785	
20001	0.334274784	0.095556599	-0.052719497	-0.2231055	-0.069340861
20002	0.168839362	-0.446124205	0.149085496	-0.142472943	
20003	0.059887263	-0.042875805	-0.225814546	0.14724155	-
20004	0.240467861	0.319285223	-0.124107983	-0.079081268	-0.247462868
20005	0.330784287	-0.402072634	0.160235081	-0.224843498	-
20006	0.289167049	0.109461726	-0.13552139	0.034956133	0.065418289
20007	-0.300701296	-0.37860634	-0.101176798	-0.060225013	-

20008 0.019500457 0.35153944 -0.078867495 0.065545107 -0.267409536

20009 -0.034002775 -0.008310362 -0.247414576 0.124918552 -

20010 0.23385746 0.232319765 -0.056151359 -0.188273388 -0.125855354

20011 -0.116659536 -0.11563429 0.008752102 0.355485825 -

20012 0.166813439 -0.040891151 -0.143137701 0.235545108 0.190242913

20013 -0.051240123 0.080096747 0.159426509 -0.05376239 -

20014 0.100634855 -0.171757022 -0.010906049 -0.153510771 -0.014468851

20015 -0.057001507 0.378439421 0.361318967 0.029190913

20016 0.314850258 -0.029235496 0.222432991 -0.201152639

20017 0.072897053 -0.050568271 -0.366981902 -0.195427769

20018 0.370126783 -0.222237012 0.156671454 -0.005564006 -

20019 0.115021318 -0.157160889 0.10197133 -0.418121941 0.156938411

20020 0.201200347 0.116357509 -0.384717671 -0.185317834 -

20021 0.043269369 -0.18768407 0.008880923 0.170600264 0.386579967

20022 -0.056396582 -0.115309873 0.234872726 0.327832211 -

20023 0.140396969 0.187626367 -0.149552743 0.356822035 0.159985125

20024 -0.266828718 0.08439517 -0.108650741 0.192988135

20025 0.335772698 0.309641284 -0.068743654 0.088307158

20026 0.007099886 0.0419045 -0.102980178 0.093989171 0.256551108

20027 0.146920351 0.014802873 0.09264389 0.244009774 -

20028 0.195215988 0.428614216 0.016492303 0.052668881 -0.036376741

20029 0.409036256 -0.100133242 -0.302815687 0.037022115

20030 0.192114565 -0.26224324 -0.259531959 -0.217613588

20031 0.181744574 0.064514934 -0.099543802 0.1865239 -0.28070979

20032 0.264490696 -0.0703996 0.099083284 -0.216036304 0.196942099

20033 0.312682711 -0.139599383 0.262737093 0.105760636

20034 0.001415813 -0.242232057 0.031436998 -0.017807281 -

20035 0.216150741 -0.004624903 -0.229681381 -0.207202985 0.064259402

20036 0.043489281 -0.34551866 -0.071273211 0.013982376 -

20037 0.223845402 -0.042033715 -0.174282389 0.37971944 0.100250018

20038 0.045598101 -0.374438005 -0.155480622 -0.03615023

20039 0.345318686 -0.235231796 -0.276954714 -0.188402776 -

20040 0.0837546 0.144254321 -0.407989388 0.261664414 -0.385994668

20041 0.436479461 -0.1068964 -0.070870663 -0.186670099 -0.221972761

20042 0.428647365 0.090552426 -0.111163398 0.296316876 -

20043 0.315982383 0.069505326 -0.156304022 -0.255439207 0.129314329

20044 -0.079911527 0.051299646 0.135415842 0.175557337

20045 0.049725694 0.097220684 -0.21332903 0.034614177 -

20046 0.159691953 -0.285820619 -0.180960416 -0.393070237 -0.188097917

20047 0.096063579 0.254848494 0.077241933 0.438759978 -

20048 0.293689469 0.190487665 0.392122077 -0.111927401 0.021258417

20049 -0.181317737 0.102393868 -0.259439136 0.159515766 -

20050 0.365648548 0.06771811-0.233837896 0.27941103-0.187173926 -

20051 0.294899347 -0.128227219 0.116281628 -0.000892799 0.288379521

-0.190355104 -0.107790888 -0.086372472 0.335864987 -
0.254214953 -0.017291939 0.023839182 0.039963593 0.0673125
0.076349966 0.261529594 0.011125064 0.395095889
0.22228007 -0.264226292 0.209093507 -0.090672615
0.248800704 0.115587298 0.326298158 0.285096582 -
0.164580987 -0.330847599 -0.142294035 0.1845468 -0.055250745
0.257649601 -0.313438549 0.39933437 -0.173741826 -
0.234358592 -0.006142279 0.255120559 0.059583292 0.034063536
-0.109718529 -0.061388963 -0.130253408 -0.058791469 -
0.350858196 0.296863946 0.135535524 0.33369265 0.320674314
-0.042295091 -0.205031908 0.127754656 0.222165073
0.399079413 -0.144751046 -0.021832975 -0.185856515
0.337017768 0.088977916 -0.177563747 -0.113401905
0.154198866 -0.020608325 0.143808926 0.255423144 -
0.135696969 -0.099832609 -0.074664634 -0.205129893 -0.057730244
-0.211127997 0.258874123 -0.055009968 -0.11640285 -
0.026623386 -0.298109496 -0.070641946 -0.139612925 -0.009706105
-0.262368716 0.099840515 -0.078785568 -0.067490046 -
0.146590939 -0.014378501 0.068160717 0.230571556 0.204449829
0.223514103 0.137126918 -0.278713247 -0.242801595
0.107727715 -0.130693533 0.418341301 0.003720371 -
0.038078446 0.123975832 0.169254038 -0.184558826 0.097807222

0.082256271 0.443202279 0.139876827 0.140628355 -
0.133391737 -0.22815505 -0.032303514 -0.032496249 -0.099117575
-0.137625863 0.375127348 0.122519891 0.271893638 -
0.143203352 0.237566783 -0.144196778 -0.277302564 0.00625611-
0.044115141 0.082116041 0.009165159 -0.290369151 0.283145918
0.145771307 0.193166765 0.033866783 0.127687733
0.238080421 0.166719244 -0.096300575 0.056218107
0.070313093 0.044623241 0.039248312 -0.256192344
0.102993004 0.278908579 0.039989166 -0.069022624
0.065191767 -0.008928954 0.218826277 0.23574414
0.032290507 -0.077988079 -0.18349963 0.042221172
0.026088506 -0.033199117 0.017888371 -0.110277628
0.289935189 -0.254608903 0.171515511 0.15003618 -
0.183813551 0.120280413 0.09053176
KEGG_TGF_BETA_SIGNALING_PATHWAY -0.014115221 -0.172526363
0.359560913 0.28340392 -0.354952855 0.065074816
0.051524341 -0.141329448 0.055426949 -0.233138145
0.017101873 -0.244256749 0.40268531 0.063115123 -
0.237195374 -0.310257129 0.30359196 -0.302013357 0.155715163
-0.377018578 0.217410583 -0.100478885 0.23522026
0.301846018 -0.099584726 0.080373251 -0.398825149 -
0.282326739 0.19485183 -0.217205843 0.008775012 -0.341055722

20096 -0.291878467 -0.386802773 0.213242662 0.143514409

20097 0.344263654 -0.286791916 0.259805859 -0.131890853 -

20098 0.193226379 -0.046400303 0.259925901 0.031210248 -0.092624351

20099 0.132755226 -0.113985716 0.059647513 0.064137022 -

20100 0.184211266 -0.259811371 0.41965981 -0.05634913 0.294504222

20101 0.207985574 0.223598562 -0.101912102 -0.273659236 -

20102 0.202282606 0.296766776 -0.04225897 0.258334638 0.23459267

20103 -0.032688255 -0.018703599 -0.14271795 0.291230524

20104 0.15384525 0.242463542 -0.391402827 -0.101925216 -

20105 0.042930443 -0.008763483 -0.264459006 0.032110287 -0.301252895

20106 0.0986816 -0.025033602 -0.216532933 -0.268751938 0.000903483

20107 0.367771264 0.016220679 0.145800541 -0.229437448

20108 0.037019034 0.219890277 0.294623653 -0.143862081 -

20109 0.189663356 -0.130823391 0.190853212 0.20428652 0.075283885

20110 -0.284244214 0.110712056 0.159581394 -0.152567975 -

20111 0.249053128 -0.239330629 -0.205453974 -0.036788372 0.209674126

20112 -0.255443252 0.016351414 -0.067942484 0.141481853 -

20113 0.415537795 0.314205902 0.102676645 0.06761537 -0.394264576

20114 -0.236909137 0.354822477 0.286286724 -0.053928955 -

20115 0.253940859 -0.030691487 -0.081391843 -0.326347825 -0.105518057

20116 -0.209553608 -0.228457614 -0.142786021 0.125838978 -

20117 0.254917953 -0.205358592 0.14536483 -0.121075264 -0.188014807

20118 -0.171146605 -0.074647565 -0.205275488 0.003216597

20119 0.020414971 0.373089355 0.30213195 0.094361483

20120 0.114590153 0.270683797 0.040902613 -0.232608612

20121 0.236615694 0.190263381 0.086458958 0.240507804

20122 0.148948121 -0.226877663 -0.139021895 0.061749157

20123 0.076812781 0.24243151 0.171893675 0.17639959

20124 0.267636502 0.066308215 0.146145805 -0.278579588 -

20125 0.009157951 -0.258700517 0.396371544 -0.161741994 0.089477873

20126 0.017442379 0.10018998 0.215977159 -0.047000165

20127 0.372841417 -0.255996148 -0.115855694 0.207361399 -

20128 0.091600153 0.058535042 0.099774724 0.306902096 0.255670959

20129 0.335566239 -0.159412485 0.000207445 -0.204426138 -

20130 0.144208655 0.135518825 -0.374184041 0.314842825 0.25355296

20131 0.209396455 0.145991754 0.295885946 0.226575133

20132 0.115475873 0.150482123 -0.266937119 0.081341876 -

20133 0.160205868 -0.295590944 -0.005821761 -0.00387144 -0.054545751

20134 -0.08298475 0.176443647 0.018157655 -0.230376008

20135 0.212176427 -0.261098534 0.094242513 -0.245055831

20136 0.104134145 -0.232449076 0.369018481 -0.337142904 -

20137 0.201221317 0.128365677 -0.183118165 0.214780191 -0.303790647

20138 -0.205591944 -0.145589889 0.143093333 -0.00207831 -

20139 0.093805104 0.270596501 0.011324053 -0.030369998 -0.044632116

20140 0.046989113 0.364144883 0.017906668 -0.224061565

20141 0.203635493 0.104555791 -0.345427248 -0.145717373 -

20142 0.005664618 0.111455745 -0.235568752 0.236195118 0.03431844

20143 0.282945377 0.025364481 -0.128908681 -0.054002238

20144 0.127499511 -0.009575546 0.320411528 -0.15885072

20145 0.385956952 -0.258743224 -0.125206001 -0.327820243 -

20146 0.120422449 -0.122995873 0.187684465 -0.073365125 0.236608861

20147 -0.2584422 0.385557475 0.240018447 0.124972642 -0.093447931

20148 0.077431051 -0.084195286 -0.325151624 0.080174003 -

20149 0.100390367 -0.063625943 0.301558392 0.310480375 -0.216287499

20150 -0.140047372 -0.354925908 -0.285335651 -0.04734437 -

20151 0.129489344 0.251910531 -0.272114029 -0.376838435 0.073333991

20152 -0.210148962 0.065646905 0.011916832 0.251341933

20153 0.086022985 -0.111763625 0.210440639 0.034839376 -

20154 0.406096481 -0.242937474 -0.24817779 0.304975056 0.140517025

20155 0.077598822 -0.345307189 0.213463144 0.164558524

20156 0.102970022 -0.070257081 0.201312605 -0.07027925 -

20157 0.02826512 -0.339462787 0.193058873 -0.346335331 -0.124838331

20158 -0.236419215 -0.252983899 0.253110990.362167532 -0.029567516

20159 0.340329724 -0.339165407 0.029758885 -0.258665453

20160 0.178345579 0.355089805 -0.336455 -0.18503133 0.120025252

20161 -0.217152663 0.093090194 0.02267507 -0.069174576

20162 0.096053624 0.096430101 -0.095495308 -0.29786392

20163 0.214995187 -0.060985144 -0.267615932 0.180488463

20164 0.152659009 0.065789192 -0.011567627 0.087299154 -

20165 0.190174273 0.31984228 0.135319352 0.165211796 0.269762837

20166 -0.171426122 0.341323345 -0.145955989 0.085269239 -

20167 0.28386254 0.229100236 -0.13437611 -0.018555847 0.188953885

20168 -0.053986992 0.014267938 -0.081396774 0.358190325

20169 0.014831124 0.242763107 -0.363442057 0.094438127 -

20170 0.208577546 0.01302696 -0.144706927 -0.336993713 -0.299351502

20171 -0.214819851 0.124096906 -0.022870085 0.227412627 -

20172 0.083844104 0.288573562 -0.217066173 0.30218357 -0.037165397

20173 0.265676149 0.187570307 0.302584635 0.278188014

20174 0.310385851 0.062658265 -0.258250325 -0.242648052 -

20175 0.365018497 0.190727589 -0.033075607 0.039769162 -0.119256709

20176 -0.184701043 -0.367264237 -0.115472814 -0.056742321

20177 0.270845257 0.217399835 0.104250645 0.22952131 -

20178 0.341157397 -0.18350112 0.014203295 0.160578912 0.27536115

20179 0.273501681 -0.011154312 -0.19648539 0.324983016

20180 0.211661031 -0.097693657 0.033522306 0.468203993 -

20181 0.129658786 0.082952295 -0.17872531 0.199899122 0.193309295

20182 0.017673087 0.135493785 -0.153247432 0.235033026 -

20183 0.248973559 0.090816483 -0.16203242 -0.174947905 0.002672695

20184 -0.293689836 0.226855823 0.034628482 -0.098816279

20185 0.328568459 -0.167193711 0.2892075 0.062175189 -0.126280238

20186 -0.409785524 0.346372813 0.108891794 0.098199044 -

20187 0.418686823 0.088145457 0.113248682 -0.216906784 -0.061268174

20188 0.369214992 -0.2092799 -0.235293118 0.077061187 0.077997802

20189 0.155520928 0.364734063 -0.158985413 0.134501055 -

20190 0.227097717 -0.163437469 -0.076971804 -0.255446293 0.067662599

20191 -0.212091749 -0.125465123 -0.115933253 0.07390808 -

20192 0.028982602 -0.300203793 0.160507066 0.18766206 0.344560465

20193 0.174523842 0.268657107 0.159318774 -0.096859043 -

20194 0.301443543 0.437426122 -0.005016028 -0.295024057 0.162169928

20195 0.162190165 0.295622075 -0.166546889 0.066446685 -

20196 0.263121948 0.299307676 0.380364724 -0.181237478 -0.094319907

20197 0.145402403 -0.021178316 -0.140638846 0.031442719

20198 0.300215185 -0.309799449 0.06280318 -0.2398247 -0.285822102

20199 -0.065172861 0.159061088 -0.275530709 -0.275973018

20200 0.108440049 -0.268089131 0.113984188 0.184079451 -

20201 0.07597607 0.182212186 -0.17097133 -0.081039501 -0.030805142

20202 -0.165238603 -0.267775457 -0.179591355 0.188084095 -

20203 0.066809846 0.322085692 0.399121265 0.072180687 -0.145572513

20204 0.424211461 -0.307146916 0.243291593 -0.06573636 -

20205 0.295267462 0.456026325 -0.257387863 -0.068122041 0.133663382

-0.083069885 0.142026473 0.085823103 0.030704849 -
0.264473975 0.03914156 0.300177036 -0.301568389 -0.088839548
0.296166008 0.176055315 -0.182860388 0.326774579
0.375476316 0.018832187 0.016221182 -0.090298282
0.216615622 -0.319444537 0.043464474 0.134398568
0.111592557 0.015346769 0.065503229 0.016158344 -
0.158444984 -0.038589165 0.273545259 0.059240618 -0.289361502
0.055007112 -0.194458446 0.094586983 -0.291600127 -
0.149214806 0.249362543 0.073141785 -0.024494304 -0.105175028
-0.092346883 0.096394246 -0.053506069 -0.282007208
0.084378809 0.077512956 0.232501364 0.173702848 -
0.133169305 0.009618076 -0.22819884 0.202714203 -0.057244716
-0.196300887
KEGG_AXON_GUIDANCE -0.236225644 0.020224551 -0.076865253
0.190206762 -0.277322178 0.061691709 -0.053241338 -
0.1004508 -0.057346112 -0.178440015 -0.064904365 -0.342615254
0.097081262 0.073028386 -0.239773547 -0.157080872
0.173142006 0.14739517 0.067923819 -0.197047282
0.244414883 0.157753247 0.116870947 0.225098946
0.197473762 -0.21130117 -0.347392416 -0.070879017
0.044117154 -0.050848371 -0.082466835 -0.093393191 -
0.115068304 -0.14609173 0.294593783 0.278766357 0.162913317

20228 0.07023275 0.082524492 -0.175003006 -0.223629297

20229 0.195857818 -0.040944364 0.04189774 -0.088380469

20230 0.208567203 0.0123182 -0.190919998 -0.05765258 -0.153414471

20231 -0.300468671 -0.0672272 0.178265674 0.154075443 0.335311723

20232 0.019625239 0.140192456 0.107306681 0.016976704

20233 0.342687056 0.173581777 0.156343122 0.027398986

20234 0.093058712 -0.177028644 0.174737866 0.043917226

20235 0.183147866 0.198815293 -0.201253146 0.083261635

20236 0.124716071 -0.222454686 -0.167674982 -0.177092706 -

20237 0.029790516 -0.149890855 -0.251656652 -0.04921527 -0.303554335

20238 0.047294623 0.274648109 -0.175406196 0.298172609 -

20239 0.072231589 0.034187238 0.210356492 -0.037878316 0.136723321

20240 -0.288488636 -0.152141116 0.075973166 0.049292337

20241 0.078546121 -0.122661281 -0.035915859 -0.155798565

20242 0.092648463 0.018163378 -0.041730543 -0.07705466 -

20243 0.13506656 -0.129096401 0.002911441 0.05991892 -0.084010178

20244 0.089885703 -0.427805637 0.321707153 0.08686147

20245 0.191798928 -0.213698977 -0.08158485 0.310278161

20246 0.011270085 -0.048044078 -0.253339315 -0.157030638 -

20247 0.183941478 -0.29226592 -0.132138782 -0.037468928 -0.125056147

20248 -0.203209664 0.13714987 -0.059946371 -0.408246734

20249 0.255459598 -0.00235742 -0.069219566 -0.158030958 -

0.232088578 -0.146845095 -0.040956188 -0.153382278 0.269369387
0.333116975 0.047247621 0.022575358 0.044356988 -
0.152411811 -0.202367152 0.095568946 -0.20755921 -0.05348335
0.109143573 0.208578469 -0.186292308 0.134202149
0.158208397 0.061413529 0.334167527 0.239042142
0.273184473 0.065823444 0.127274379 0.0545647 -0.247420619
-0.112196725 -0.086504747 0.198594668 -0.117958282 -
0.295460498 0.002402634 0.00452236 0.226748554 0.28109297
0.086750067 0.083848608 -0.044657185 0.086830677 -
0.16378968 -0.074543302 0.164319188 0.308560552 0.242640201
0.023390543 -0.265130386 0.048106147 0.041915893 -
0.269714745 -0.144032569 -0.239514468 0.214376715 0.375787028
0.083451815 0.0715186 -0.074467563 0.23717084 0.029324797
-0.114583287 -0.26496575 -0.111255029 -0.016437224 -
0.347658621 0.321519767 -0.118895833 -0.071083014 -0.166915038
0.01346066 0.115402961 -0.222042381 0.188418918 -
0.15604273 0.072563151 -0.304020739 -0.057239343 -0.273242442
0.173439707 -0.367408237 0.011342488 -0.039557431
0.043402642 0.086203511 -0.059516939 -0.202221099 -
0.259687101 0.162161951 -0.092346042 -0.028325344 0.134274123
0.155471606 -0.032537764 -0.059893211 0.081283402
0.070105598 -0.111466918 -0.239983142 0.178240545

0.114265487 -0.303599767 0.204917988 -0.196386249
0.13325624 -0.059981482 0.307255364 0.162492113
0.121871917 -0.069698581 -0.225072721 0.036947307
0.172095468 -0.088746612 0.191573688 -0.091421597
0.289667359 -0.101267664 -0.057771846 -0.31771187 -
0.156395411 -0.097088503 0.184394384 0.109061227 0.283801786
-0.059779933 0.390084868 0.121711159 -0.019195267 -
0.000827921 0.054364678 -0.111354141 -0.075318056 -0.210614494
-0.023101438 0.161952523 0.174100014 0.288586092
0.014765323 -0.169630046 -0.140056346 -0.276144974 -
0.149224332 0.037520367 -0.054812626 -0.322078701 -0.057457527
0.060508799 0.005622217 -0.153685674 -0.116554502 -
0.027722487 0.049689586 0.086507206 0.219508417 0.139782541
-0.182633572 -0.230831326 -0.250290753 0.327265887 -
0.002345314 -0.152748115 0.100915215 -0.100912038 0.306465113
0.172094404 -0.111639715 0.06189495 0.00961743 -
0.021797546 -0.206848267 -0.038839835 -0.288708702 -0.048182011
0.070277683 -0.126700391 0.256067702 0.33062397
0.075166758 0.305784626 -0.20446716 0.036395701 -
0.234613367 0.248938798 0.135486518 -0.156152508 -0.263574687
0.100932267 0.006839758 0.161217172 0.082685599 -
0.124142463 -0.005808357 0.274909296 0.153138436 -0.264697349

0.253239431 -0.103332585 -0.326317883 0.051376648 0.2977579
-0.042683381 -0.037928442 0.241205859 0.205141211
0.350170506 0.148849603 0.148392891 0.28297148 -
0.013331561 0.251652255 -0.135167628 -0.082454802 -0.182809033
0.024839294 -0.092545758 -0.216798022 0.063359444 -
0.02504607 0.075907774 -0.028770291 0.315590932 0.195693002
0.267410748 -0.37668629 0.043552764 -0.122555235 -
0.094211068 -0.000398718 -0.265603807 -0.326710711 -0.238109205
-0.216363974 -0.03450568 0.442398114 -0.006765428
0.315955989 -0.267581958 0.20011936-0.04611148 0.221824239
0.065960634 0.372681965 0.281516853 0.175641718 -
0.163810618 -0.073314532 -0.164793541 -0.062414225 0.269996866
-0.023127147 -0.028989996 0.04188166 0.098652042 -
0.191629382 0.197844206 0.13125785 -0.03385572 0.376592342
0.147123995 0.088280425 -0.069021963 -0.131093285
0.105212755 0.217393522 0.411584003 0.31567179 -
0.239275377 -0.013813132 0.209054863 0.266543182 -0.241703787
0.338441816 0.268261596 -0.192443308 0.086366242 -
0.217048174 -0.07587292 0.152881133 0.206868625 -0.027476476
-0.235729838 0.186108549 -0.167650683 0.137153552 -
0.007462274 0.225190364 0.186932207 -0.287943465 0.258633445
0.107429805 -0.054172279 -0.084570752 -0.073865399

20316 0.363492168 0.172288869 -0.04578967 -0.188050397

20317 0.386398812 0.123564928 -0.010724967 -0.275413337

20318 0.192955110.015293446 -0.19707986 -0.130250256 0.186940901

20319 0.243102758 -0.34460662 -0.217895215 0.213745389

20320 0.177355407 0.211770522 -0.203313088 0.186383177 -

20321 0.030879051 -0.082309855 -0.053235919 0.047312643 0.084107106

20322 0.018022619 0.012054015 -0.048656155 -0.118131936

20323 0.19687187 -0.285320954 0.225205865 0.12806577

20324 0.189263835 -0.220733833 0.096486397 0.287485669 -

20325 0.007654393 -0.105298685 0.270015378 0.191535219 -0.146607362

20326 -0.234081499 0.020236669 0.175285481 -0.214847339 -

20327 0.227966275 -0.094233717 0.088342758 0.341785701 -0.091706205

20328 -0.153685662 0.224925806 0.052591383 -0.090913929

20329 0.099459292 0.134472348 -0.01124866 -0.230001057 -

20330 0.127747202 -0.283354323 -0.297490378 0.207129911 -0.324587592

20331 -0.254607136 0.257643182 -0.205326853 -0.071984858

20332 0.239626706 0.031292846 0.031625838 -0.24198863 -

20333 0.117414466 0.104587209 -0.254360162 -0.232015512 0.15025041

20334 0.147982914 0.13635873 0.227385829 0.399688909

20335 0.307737301 0.136065586 0.131770525 0.204872776

20336 0.022399473 0.117148141 -0.202244972 0.14199831 -

20337 0.0973495 -0.069628279 -0.035299728 0.210787755 -0.011806384 -

0.073185465 -0.170633398 -0.150642254 -0.039924566 0.171262948
-0.275672301 -0.22216052 0.131902711 0.153686526 -
0.06686734 0.263589917 0.351150622 -0.00209447 -0.209528843
-0.111078582 0.152107473 -0.256022577 0.05579963
0.116880802 0.128620325 0.031890027 0.010046392 -
0.066329103 0.035079538 -0.014095453 0.139875249 0.026530169
-0.182132415 -0.113168135 -0.157390404 -0.054722502 -
0.127113937 0.030785929 0.135235607 0.195364369 0.015140392
-0.224477265 -0.267518115 0.031063688 -0.055407333 -
0.155671696 -0.090634502 0.074575413 0.005404143 0.206544379
-0.063646182 0.057444752 -0.055135097 0.132754007 -
0.071838645 -0.146480334
KEGG_VEGF_SIGNALING_PATHWAY -0.036923707 0.141905256 -
0.126858278 0.237605574 -0.114261028 0.047848815 -0.12893864
0.043874904 -0.054871513 0.023369162 -0.187861534 -
0.052558559 -0.091947782 -0.255596109 -0.18094153 -0.034737062
0.14056406 0.174795875 0.149337783 -0.031223753
0.202240766 0.251823967 0.179156797 0.155376322
0.202659148 -0.163819006 -0.244534545 -0.10744347 -
0.034359409 -0.044586427 -0.200948262 0.197585644 0.058705151
0.047282699 -0.040984487 -0.045535438 -0.098690836
0.027866683 0.046804935 0.018728451 -0.028029758

0.075130962 0.099437135 0.175314329 0.111008574

0.088156741 0.121443307 0.003855221 -0.293071648 -

0.002613052 -0.074804512 0.032587582 -0.08860267 0.142767763

0.266357815 0.032816941 0.081345884 -0.044990297

0.078681851 0.075700778 -0.141873226 0.107869312

0.029395181 0.185641366 -0.160389664 0.138405145 -

0.013753814 -0.132953963 -0.125155731 0.041719321 0.151549636

0.204303049 -0.025761552 -0.129445776 -0.240806547 -

0.04219097 -0.006770065 0.029501802 -0.01093203 -0.14109589

-0.116454449 0.241163292 -0.050839356 0.023360853 -

0.063491867 0.162672436 -0.194664853 -0.05123001 0.157678433

-0.130925425 -0.234899154 0.056389022 0.291622477

0.194977975 0.169278919 0.101990911 0.009254634

0.21958477 0.089735524 -0.107801878 0.008254542 -

0.047516557 -0.180261893 0.083487381 0.063083899 -0.111318942

-0.063956571 -0.125129693 0.239587451 0.09084103 -

0.132754496 -0.265736487 -0.16748375 0.103310056 0.065875286

0.003698094 -0.144291008 -0.16316964 -0.139139378 -

0.237623616 0.014552366 0.029844511 0.013838277 -0.014104199

0.074868884 -0.065911485 -0.209765773 0.027288544

0.021782465 0.020703273 -0.138233095 -0.084739749 -

0.080548897 0.163069978 -0.10631475 0.199807728 0.260985379

0.095449153 -0.115481 0.149631462 -0.215405674 -0.050088808
0.042141829 -0.175490939 -0.01770931 -0.297050323
0.232698447 -0.086307104 0.030019773 0.032046003
0.295575495 0.070228054 0.019769288 0.077606395
0.168825591 -0.17083153 -0.13537918 -0.071615312 -
0.100865389 0.203097225 0.076881583 -0.06324099 -0.04354666
0.037095449 -0.047053659 0.163141825 0.235375472
0.006600677 0.005194814 -0.172563344 -0.007394869 -
0.243890311 0.005536268 0.082981785 0.096049171 -0.047272564
0.067682299 -0.149478793 0.130263628 0.219728225 -
0.075036205 0.064141771 0.159379307 0.073680434 0.172260424
0.014995256 -0.089691036 -0.146846557 0.180064782 -
0.049141568 -0.077529545 -0.124739631 0.01954528 -0.188300006
-0.150225407 -0.07890398 -0.273082903 -0.008379709 -
0.266159718 0.190215432 0.099664616 -0.09481848 0.20496665
-0.082946449 0.018670046 -0.149947678 -0.167840348 -
0.069910976 0.078749032 -0.198179436 0.042250137 -0.054427992
-0.098188119 -0.063294058 0.005833699 0.169197625 -
0.085603736 -0.033408974 -0.183225401 0.145737141 0.183815018
0.014852079 -0.018865622 0.074416499 -0.043682816
0.21197155-0.023445704 -0.05894067 0.190149736 0.174787289
-0.097993218 -0.032254724 0.004009385 0.070000227

20404 0.100655032 0.138076231 -0.000418798 0.047887463

20405 0.280384142 -0.105251663 0.027592639 0.120467591 -

20406 0.091156263 0.047276696 -0.31336881 0.135204158 0.098413023

20407 -0.133105326 -0.210805108 0.031340118 -0.103440539

20408 0.118243816 0.070965292 0.138846582 -0.058415895

20409 0.146096822 -0.008384145 -0.029215429 0.115543369

20410 0.040593005 -0.170687082 0.18373665 -0.101325879

20411 0.053518931 -0.114163811 0.07561568 0.175605369 -

20412 0.086623395 0.171398665 -0.101352936 -0.055401679 -0.166258604

20413 -0.051185939 -0.165631962 -0.230006307 0.181151526 -

20414 0.035503009 0.049710191 -0.143340222 0.015557324 0.00127062

20415 0.128133411 0.126356694 0.003271907 0.180831545 -

20416 0.0689816 -0.033149301 -0.020678317 0.125347233 -0.096469054 -

20417 0.082974832 0.044836791 0.011661068 0.113202713 -0.126719587

20418 0.006858071 0.146453979 0.045827475 0.058381064 -

20419 0.047827851 -0.067837684 -0.300422725 -0.10331964 0.012290147

20420 -0.261541403 0.054976428 0.249155802 0.215168958

20421 0.246670707 -0.064131508 0.113286124 -0.134394652

20422 0.055038647 0.048701945 -0.031184208 -0.11626385

20423 0.02988873 0.072257282 -0.008031498 0.139287083 -

20424 0.10690667 0.119116975 -0.114030204 0.013929342 0.025994036

20425 0.143363428 -0.132264713 -0.102890479 0.266886681 -

0.080143144 -0.301830288 -0.179111036 -0.04880563 0.062000805
0.15437658 0.096812418 0.02833998 0.207659495
0.378374634 0.243481385 0.031242558 -0.141049986 -
0.055590437 0.206334351 -0.040705319 -0.233392898 -0.021656553
0.103631252 0.272348324 -0.168490768 0.152979958
0.000148343 0.093266791 -0.221804496 0.134288278 -
0.076533528 0.06227004 -0.136182419 -0.14510804 -0.10942993
-0.112025575 -0.147585998 0.112562307 0.306298608 -
0.182170656 0.211260245 0.062134288 0.040972112 -0.048288438
0.100497659 0.082813686 0.183518028 0.229123713 -
0.021462336 0.237104902 -0.053548755 -0.056663256 -0.273895664
0.1792031 -0.029029543 -0.155162635 -0.046349916 -0.248908894
-0.107754707 -0.11327742 0.182122491 -0.163744022
0.148275041 -0.007329002 -0.038660396 -0.006755793 -
0.03694001 -0.139925552 0.046072304 0.136005229 0.134482338
-0.078774858 -0.244927024 -0.028655502 0.145561564 -
0.087355372 0.120021188 0.096775405 -0.144460804 -0.034460555
0.111669425 -0.221501328 0.013382841 0.031135704 -
0.103658861 -0.082513878 0.163474597 -0.267838672 0.054774068
-0.11971603 0.030276484 0.220729033 -0.150680677
0.080313407 0.06776826 0.108138722 -0.261071974 -
0.275882013 0.179061407 0.091669566 -0.125782015 -0.332966763

0.139945004 0.033632866 -0.280336113 -0.274285775
0.358658101 -0.103671804 -0.03410668 -0.339297941
0.099601312 0.224439029 0.029348356 -0.205711266 -
0.127878735 0.175153596 0.075661535 0.151468028 0.069798449
0.094871758 0.086926691 0.126016067 0.165586891 -
0.061780631 0.255729057 0.136465016 0.114735456 0.014495921
0.008080993 -0.116489931 0.067571787 -0.192911506
0.014624684 -0.25845265 -0.1138325 -0.034655505 0.218988211
0.028312586 0.038918796 0.102813787 -0.056680927 -
0.145365566 -0.019224593 0.077339394 0.128970088 -0.351183841
-0.067866589 -0.073621178 0.243333843 -0.032299832
0.009583998 -0.041343108 -0.036573365 0.088078543 -
0.012784623 0.152056674 0.006062458 -0.124265784 0.020944028
-0.10422013 -0.259195193 -0.025253497 -0.181842075 -
0.073246868 -0.020482919 0.147131221 -0.153456305 -0.120077106
0.014390682 -0.08734451 -0.099151025 -0.199982779
0.208391762 -0.294058499 -0.082092329 0.099425166
0.013829513 0.078373287 0.067617904 0.140671664
0.172304577 0.040510181 0.103211021 0.014241553 -
0.070274808 0.093759226 -0.307481346 -0.071477083 -0.098358336
-0.033825135 -0.043615859 -0.120235777 0.165430065
0.186985175 0.130281679 -0.189643747 -0.151347994

0.09553544 -0.157601449 -0.18356183 0.051072336
0.240268068 0.119659557 0.21873135 0.210845285
0.027466502 -0.367100952 -0.122603895 0.06967973 -
0.248873231 0.000318606 -0.040721407 -0.170141242 -0.073501395
0.086211944 -0.091068921 0.088307624 0.021386827
0.119631493 0.024207906 0.007845962 -0.022876647 -
0.10039937 0.077021039 -0.172368197 0.073397219 -0.038709726
0.150382833 0.027663182 -0.147179956 -0.209607473
0.213610226 -0.072212973 0.092424101 -0.11505432
0.152614548 0.01204454 0.133372769 0.107112744
0.121204578 0.094841132 -0.225179743 -0.006322945 -
0.061191981
KEGG_FOCAL_ADHESION -0.21383955 0.015603853 -0.075420355
0.216340346 -0.372378179 0.061222528 0.032699897 -
0.117329304 -0.012853047 -0.225812985 -0.114840072 -0.305822954
0.250922995 -0.014648805 -0.260013404 -0.287268977
0.258483743 0.057827121 0.100037424 -0.431602816
0.344894449 0.084177838 0.258089946 0.30255996
0.238985632 -0.141920445 -0.425297829 -0.084850007 -
0.117665901 -0.150991468 -0.003743148 -0.091884438 -0.28786462
-0.255277475 0.391200744 0.330509537 0.299955699 -
0.02417359 0.014996689 -0.246963285 -0.159817484 0.195591355

0.088898329 -0.048991166 -0.08491709 0.307359069 -
0.071027136 -0.135902724 0.016001777 -0.260273828 -0.303769243
-0.03373543 0.08480977 0.10174665 0.34931865
0.169849627 0.025169352 0.353463784 -0.078683076
0.429788084 0.149118527 0.281968261 0.062785479
0.151861925 -0.180984426 0.090582153 0.148395711
0.118213515 0.127736238 -0.350057403 0.043501792
0.166252754 -0.026321556 0.036355981 -0.233369128 -
0.07932417 -0.190698333 -0.277550441 -0.034431117 -0.296146655
-0.172546564 0.389110497 -0.091453869 0.334603317 -
0.148360626 -0.026686993 0.21271674 0.169239032 -0.037951499
-0.274142322 -0.192190232 0.189741424 0.131791447
0.024952652 -0.11847167 -0.068503368 -0.125629066 -
0.027171899 -0.085983413 -0.072276888 -0.169530382 -0.203051358
-0.187785487 0.058397734 0.195770649 -0.165469227
0.249406122 -0.391468811 0.325293818 -0.043845365
0.151091382 -0.316421531 0.031507704 0.300249874
0.025828518 0.046058853 -0.304195916 -0.325226728 -
0.249667903 -0.300500447 -0.199175732 -0.212453463 -0.113777906
-0.23489781 0.078757433 -0.163007448 -0.390425988
0.265570325 0.092336443 -0.172831673 -0.273818205 -
0.132456955 -0.250324158 -0.13192493 -0.25389019 0.419692624

20514 0.369515198 0.077909395 -0.074695213 0.25861748 -

20515 0.277823838 -0.335271508 0.094512759 -0.119013129 0.218167236

20516 0.196942717 0.161413225 -0.281871832 0.10457077 0.189929

20517 0.245880048 0.343121719 0.309419233 0.390364886 -

20518 0.132008086 0.172113262 0.257521504 -0.410286535 -0.163263807

20519 0.083521592 0.388775575 -0.087481717 -0.146676707 -

20520 0.105539403 0.17668128 0.187936376 0.322108969 0.295184288

20521 -0.043007509 0.031196350.136125585 -0.309958727 -0.115784175

20522 0.172096731 0.383773499 0.321821284 0.129011781 -

20523 0.321262461 0.178754039 0.084148745 -0.075803127 -0.17340602

20524 -0.38218327 0.165553665 0.319209415 0.23644206

20525 0.015904891 -0.053561345 0.209308325 0.251859447 -

20526 0.141546232 -0.285324779 -0.064821769 -0.176312136 -0.418169683

20527 0.264394356 -0.262100066 -0.020021598 -0.197123038 -

20528 0.14401422 0.21968933 -0.155767555 0.346467387 -0.264879169

20529 0.081159495 -0.339802348 -0.100173931 -0.208486912

20530 0.327829228 -0.34694342 -0.167394888 -0.12748368

20531 0.137573454 0.17102341 -0.097751534 -0.126101337 -

20532 0.148153631 -0.009200562 -0.196092144 -0.090635147 0.244450349

20533 0.020866768 -0.103194915 -0.051190587 0.06648564

20534 0.157088756 0.160253796 -0.235781988 0.31083978

20535 0.150018832 -0.410814152 0.032009186 -0.284686565

20536 0.275534801 -0.147391291 0.338061909 0.032545486

20537 0.170377921 -0.084363309 -0.288449225 0.096237791

20538 0.085203157 -0.1482891 0.372784464 -0.285372433 0.345360139

20539 -0.158966114 -0.158427123 -0.411812857 -0.298674449 -

20540 0.031316482 0.322808662 0.008616402 0.416832045 -0.078365227

20541 0.439138142 0.160237271 -0.024533797 0.075995914 -

20542 0.017712479 -0.230164562 -0.217433562 -0.093403195 0.050602992

20543 0.249089229 0.216108138 0.395002293 0.067489495 -

20544 0.177021555 -0.216762742 -0.360126887 0.088918576 0.012382676

20545 0.070998602 -0.380847539 -0.325492574 -0.008207353 -

20546 0.123861009 -0.099512211 0.066903835 0.111072488 0.231493785

20547 -0.062885467 0.085000854 0.007849923 -0.328338029 -

20548 0.252552831 -0.308693943 0.386696537 -0.035997127 -0.14573137

20549 0.117310566 0.023827517 0.40077089 0.092134972 -

20550 0.181062312 0.094943703 -0.108175204 0.08153943 -0.325204381

20551 -0.148865829 -0.388361757 -0.087381349 0.179596153 -

20552 0.254787016 0.29035321 0.277476412 0.072120399 0.320012966

20553 -0.312620448 0.148712851 -0.288657663 0.267789078

20554 0.195995134 -0.053733192 -0.208593543 0.141178306 -

20555 0.00890388 0.046273052 0.275910892 -0.126348313 0.09495186

20556 0.303450143 0.025779543 -0.294476951 0.38737508 -

20557 0.144590313 -0.340086729 0.220168466 0.279382678 -0.024545548

20558 -0.031995119 0.172838907 -0.00830509 0.47088936

20559 0.239527111 0.200359327 0.377675586 -0.213446089

20560 0.18293849 -0.328398535 -0.162407872 -0.347505655

20561 0.221509247 0.056647804 -0.351185612 0.061991882

20562 0.048502101 0.144568221 0.091456232 0.345809821 -

20563 0.109814119 0.291829484 -0.443234109 -0.003044259 -0.249843149

20564 0.044471618 -0.066827772 -0.404302419 -0.360872232 -

20565 0.304887306 -0.051556053 0.10124077 0.433595628 0.092975799

20566 0.313615903 -0.274603694 0.251454061 -0.185993355

20567 0.23406921 0.153118245 0.376732069 0.322954982

20568 0.303217051 -0.114489531 -0.239611064 -0.212523226 -

20569 0.219447142 0.316701571 -0.019086698 -0.115391389 -0.043394902

20570 -0.176597945 -0.187398768 0.197695297 0.088419402

20571 0.01310897 0.398792487 0.241897937 0.183076797 -

20572 0.318575105 -0.183874112 0.074685254 0.242030556 0.435750624

20573 0.341157053 0.008708723 -0.182176931 0.263333424

20574 0.253744484 -0.295794872 0.347477975 0.297158641 -

20575 0.25990819 0.082926018 -0.333428742 -0.112748507 0.281368602

20576 0.158655744 3.48E-05 -0.30032154 0.310158063 -0.348610481

20577 0.239510606 0.051486964 -0.046892249 0.144740889 -

20578 0.362682975 0.372393733 0.205726847 -0.183517027 -0.016086569

20579 -0.277238523 0.408883741 0.203660496 -0.109204166 -

20580 0.276524876 0.376393458 0.037951743 0.172076343 -0.357936598

20581 0.108422068 0.318802204 -0.291765609 -0.258613883

20582 0.280452833 0.321349948 -0.192397054 -0.031227062

20583 0.227470602 0.095105352 0.275033843 -0.31079942

20584 0.235637805 -0.061301248 -0.194360142 0.221820795 -

20585 0.056452564 0.088400393 0.116312342 0.078355862 -0.114401752

20586 -0.085918555 -0.005885245 -0.390033879 0.235804024

20587 0.15966133 0.272648112 -0.021441316 0.146257139

20588 0.018209287 -0.27799734 -0.110773439 0.340141838

20589 0.195741798 -0.119010459 -0.077104258 -0.045312303

20590 0.226959091 -0.240927664 -0.283057292 -0.161610924

20591 0.176321368 0.361231371 -0.15982108 0.148943781

20592 0.180305618 -0.079972277 -0.234038981 0.189169094

20593 0.150142333 -0.167560405 -0.291540098 -0.183579099 -

20594 0.259428249 -0.268292846 0.255976107 -0.372734285 -0.171940466

20595 0.206141741 -0.291732377 0.14782381 0.326365755 -

20596 0.040916402 0.05356272 -0.40072955 0.029960543 0.074034814

20597 -0.318906141 -0.25563483 0.110728073 0.091626193

20598 0.078873822 0.365781862 0.452737726 0.311557666

20599 0.042811205 0.337608091 0.160562491 -0.007606582

20600 0.15552577 -0.33518515 0.25854306 -0.207873636 -

20601 0.089676042 -0.041943975 0.122210809 0.081960281 -0.161385065

-0.284066067 -0.190985148 -0.105045591 0.295393032 -
0.26437454 -0.130258396 0.111615546 0.161954924 0.074812947
0.333772146 0.377267026 -0.212284877 -0.147115094 -
0.055121821 -0.155799942 -0.224844678 -0.012039486 0.055679326
0.052363831 0.146291634 0.083551107 -0.235093181
0.124667737 -0.066270209 0.098786092 -0.146408564 -
0.193681941 -0.081504625 -0.040952345 0.041942985 -0.327881618
-0.11412962 0.209019241 0.278392017 0.047042901 -
0.247939981 -0.215451358 0.214468364 0.115428817 -0.212461008
0.032889493 0.142301395 0.028780205 0.07598326 -
0.107895574 0.006986967 0.088208573 0.034679198 -0.021031249
-0.276184643
KEGG_ECM_RECEPTOR_INTERACTION -0.404351217 -0.261817678 -
0.229340127 0.048910631 -0.320892418 0.126277626 0.109933584
-0.020533564 0.237360251 -0.015831764 -0.239726883 -
0.374398068 0.433750784 0.169785052 -0.061001437 -0.283939098
0.030332353 0.035749803 0.317648711 -0.570803391 0.2075266
-0.265869195 0.215307305 0.251667489 0.389012559 -
0.248510321 -0.471448757 -0.096551244 -0.217644931 0.166924308
0.005431395 -0.065662394 -0.461500166 -0.223582866
0.449497615 0.403478776 0.395082463 0.155813509 -
0.028813732 -0.438427545 -0.080690426 0.24062252 -0.197521518

-0.291848297 0.02938158 0.46485188 -0.202241409 -
0.111393039 0.208300348 -0.441695155 -0.309488705 -0.162530362
0.070538621 0.093641558 0.138542064 0.285728019
0.255335284 0.425053774 -0.217553024 0.466971956
0.210373058 0.391630881 -0.19820454 0.064579526 -
0.161195172 0.149137801 0.203101949 0.086370304 0.410926972
-0.373320706 -0.020039938 0.012805413 0.173602518
0.08646437 -0.214285685 0.266826288 -0.167853774 -
0.457840048 -0.022398386 -0.377956629 0.020823649 0.426523177
-0.048115749 0.352191888 -0.256714208 -0.113595466
0.271606597 0.17086467 0.143681188 -0.359082333
0.031096561 0.287919471 0.058000241 -0.239052155 -
0.293977384 -0.20449006 -0.158861471 0.050891567 -0.233384499
-0.148200762 -0.176830699 0.056621292 -0.307447888
0.226567234 0.326728562 -0.220679056 0.393495355 -
0.423180659 0.240094162 -0.036763911 0.422887675 -0.410740979
-0.100959942 0.241013907 -0.130417383 -0.083017818 -
0.367455826 -0.107018285 0.087425455 -0.433031126 -0.102985026
-0.360454792 -0.193121676 0.079651858 0.132646528
0.182632864 -0.459684987 0.342793221 -0.083458369 -
0.351172005 -0.286202974 0.075550052 -0.284044894 -0.296087139
-0.228768474 0.465733749 0.426859233 -0.12277589 -

0.216430336 0.173691178 -0.177564811 -0.334702479 0.025200791
-0.072686963 0.089075314 0.222575868 0.0587483 -0.280063774
0.459802211 0.28767942 0.337767776 0.347047614
0.450349644 0.524466225 -0.297483224 0.295310575
0.406183656 -0.4355274 -0.323106602 0.219594663 0.371982799
-0.00630802 -0.280074374 0.020683392 0.128196574
0.285812855 0.25841065 0.297907313 0.29164666
0.239718108 0.115240803 -0.113089354 -0.109148937
0.037575333 0.273403948 0.366764442 -0.102902811 -
0.470031929 0.126096665 0.001913809 0.068186367 -0.21475863
-0.496132797 0.357391023 0.231127679 0.213286865
0.106461549 -0.231806777 0.38634072 0.330412381 -
0.080419803 -0.254225736 -0.120268159 -0.085080004 -0.542044021
0.370685876 -0.058715683 -0.118202938 0.000346955 -
0.409966041 0.322764145 -0.257360314 0.367333328 0.110433441
0.008419909 -0.419357685 0.194186152 -0.355344927
0.346889264 -0.428496826 -0.373094671 -0.102271582
0.125049167 0.061274812 0.25791658 -0.335411482 -
0.215411342 0.007907425 -0.304113641 -0.317872601 0.443778804
-0.278116377 -0.205152511 -0.307571939 0.099312435
0.039819266 0.262376292 0.062321401 0.079097452
0.316639884 -0.484281505 0.068936632 -0.364249228

0.454872109 -0.178687384 0.360964616 0.16158163 -
0.082532673 -0.363912075 -0.334556171 0.244861713 0.123289446
-0.113585761 0.339938833 -0.193659353 0.336052872 -
0.165911414 -0.217024194 -0.367887618 -0.257901301 -0.022953946
0.235704914 0.262439538 0.407388871 0.095324903
0.509524217 0.0837374 0.114042418 -0.223075483 -0.193031735
-0.237766837 -0.433524218 -0.021065613 0.383640658
0.324734539 0.054567302 0.502206602 0.17030108 -
0.435663435 -0.104442372 -0.424110075 0.029710803 -0.024403905
0.204264632 -0.439339024 -0.453137415 -0.314828047 -
0.268503375 -0.096432533 0.203455011 0.341400886 0.226777981
-0.158396015 0.350595835 0.229649893 -0.354001983 -
0.099444176 -0.38043129 0.532060753 0.10903593 -0.24556442
0.169553884 -0.07766969 0.30750228 0.334451334
0.066112002 -0.08422597 -0.288720533 0.079064916 -
0.367578544 -0.31092327 -0.439953303 0.16316179 0.380034188
-0.168300488 0.356739519 0.419942031 -0.117298098
0.442102015 -0.230027017 0.230732891 -0.284790112
0.379564355 -0.037332804 -0.095053951 -0.27796375
0.35531891 -0.170421207 -0.144317202 0.026049508
0.209181135 -0.146379306 0.2436919 0.311190317 -0.324162905
0.522375452 -0.208885751 -0.188321833 0.166142565

20690 0.377906148 0.198277834 -0.252493669 0.322390153 -

20691 0.025128496 0.484701807 0.198736155 0.014854074 0.410760726

20692 -0.406168146 0.077752518 -0.454584073 -0.306827649 -

20693 0.18124173 0.183253165 -0.097059212 -0.363669649 -0.189354187

20694 -0.081801532 0.234944603 0.017398153 0.406953051 -

20695 0.104985529 0.246798008 -0.561337661 -0.142013381 0.006052847

20696 0.321854957 -0.181669309 -0.500156642 -0.336620022 -

20697 0.466217588 0.180453837 -0.143567917 0.522462651 0.137320153

20698 0.25470414 -0.429527602 0.157436444 -0.267314748

20699 0.114829178 0.305991218 0.305957623 0.154955629

20700 0.246526835 -0.41927247 -0.163291445 -0.132254908 -

20701 0.091882226 0.432916134 -0.231693636 -0.039839566 0.201599863

20702 0.010712901 -0.171796333 0.279460938 -0.033775183 -

20703 0.033498383 0.487776156 0.103203098 0.072688226 -0.482977841

20704 -0.181319811 0.336825981 0.336216644 0.451634199

20705 0.283904273 -0.045020883 0.069014958 0.335537838

20706 0.297636533 -0.298329928 0.356901743 0.242160852 -

20707 0.330148862 0.387152059 -0.45094491 -0.055610899 0.373071012

20708 0.181118706 -0.213574759 -0.453780075 0.317135027 -

20709 0.266409746 0.349398242 0.315622085 -0.0002901 -0.067655189 -

20710 0.441359721 0.454421371 0.249510946 -0.379752559 -0.172312848

20711 -0.445221776 0.445752121 0.254778368 -0.02477134 -

0.229917133 0.345206093 0.210199739 0.24655843 -0.188253203
-0.087998945 0.458464675 -0.299677613 -0.033081021
0.273765114 -0.016361586 -0.235155242 -0.016914385
0.259198602 -0.101863963 0.279730634 -0.378148356
0.483790953 -0.015722588 -0.367672574 0.395230971
0.091985676 0.254588846 0.047134448 0.104596087 -
0.104558846 -0.139113471 0.035625504 -0.536927166 0.29976402
0.139144775 0.310708979 -0.239465335 0.199104816 -
0.18546025 -0.399340054 0.095973548 0.345662074 0.136672267
-0.06159379 -0.265542115 0.176787775 0.037863426 -
0.4244728 -0.212906388 -0.062142063 0.132207044 0.393969666 -
0.090154993 0.075211571 0.27066237 -0.011977778 -0.394631992
0.196417383 -0.02650592 -0.227127118 -0.437085958
0.093679882 -0.332684053 -0.432162638 0.134727078 -
0.438301568 -0.132627928 0.208979411 -0.274688726 0.24310303
0.367534414 -0.23359767 0.036635537 -0.440476759
0.357851295 0.052811588 -0.396274496 -0.13905211
0.134763795 -0.210644433 0.075059777 0.414330446
0.507355772 0.404646523 0.127762288 0.335683029
0.332982581 -0.057774094 0.199848751 -0.315052603
0.256193061 0.078823297 -0.165096621 -0.309120485 -
0.006956128 -0.004297019 -0.410404633 -0.395496991 -0.337303377

0.075484802 0.324075597 -0.357165009 -0.211490637
0.117622351 0.040266446 0.139630419 0.267538064
0.308423809 -0.080786226 0.184172017 0.009326319 -
0.313903042 -0.034126354 -0.116254914 0.192146709 0.142946913
0.328696863 0.249383979 -0.274115503 0.184746733
0.081277351 0.053369966 -0.014354942 -0.168268998 -
0.048621959 0.038039091 0.262048022 -0.33517843 -0.169218257
0.233767663 0.388695979 0.135750577 -0.205907503 -
0.071862912 -0.126509171 0.121861038 -0.309477829 -0.02520001
0.271006913 0.102358811 -0.097128403 -0.128706713
0.09935663 0.206390493 0.194356067 0.110375391 -
0.364469233
KEGG_CELL_ADHESION_MOLECULES_CAMS -0.381436052
0.358494101 -0.45693732 -0.046695538 -0.036779847 -
0.060355105 -0.211471786 0.028028453 0.0581976 0.2074434
0.153815783 -0.445261463 0.236724237 -0.332760636
0.17514385 -0.270492129 -0.227033764 0.440419046
0.206516625 0.270731631 -0.327788257 0.429882673
0.042943557 -0.330481267 0.334872862 -0.075322882 -
0.239147545 0.170198398 0.078475669 -0.184958012 -0.304616225
0.174601674 0.268426952 0.412727206 0.393271255 -
0.081588153 -0.117775906 0.301203285 -0.44000264 0.024877163

-0.263307912 0.235875653 -0.111163306 0.389973425 -
0.162481711 0.10574008 -0.241785813 -0.331106521 -0.151456383
0.269456422 -0.347831211 -0.106482216 0.279765371 -
0.39310761 0.030741237 -0.26811546 -0.024723156 0.318660694
0.093564512 0.075680413 0.03386906 0.402387147 -
0.114975347 -0.269356632 -0.240543418 -0.014947816 0.088505393
-0.269979519 0.338615771 0.335904928 0.257617623
0.458265048 -0.348283246 0.243550346 -0.227348438
0.386857969 0.336970572 -0.385637783 0.366145097 -
0.377661951 -0.208965481 0.312097192 0.430232348 -0.012004381
0.152877637 0.052574539 -0.308406015 -0.065338047
0.408428218 -0.169127393 -0.368705082 0.084938323
0.075490068 0.282683469 0.245363073 0.328678186 -
0.134415228 0.308679779 0.20124034 -0.20501118 0.300511928
0.220783438 -0.049161916 0.441662275 0.181347027
0.163775223 -0.194444468 -0.420609205 -0.174796088
0.399909603 0.243172189 0.201173471 -0.16703569 -
0.296537293 -0.009584227 0.363774481 0.195376186 0.163346641
-0.075036678 -0.433482049 -0.023187032 -0.01122116 -
0.260004782 -0.237185668 0.083838334 0.231654757 -0.450659274
0.263784913 -0.219648905 0.393672919 0.314323014
0.293069503 -0.298262315 0.290160048 0.186553021

0.048733123 0.06981835 -0.325633434 -0.130760119 -
0.140007422 -0.106723319 -0.054714876 -0.384677279 -0.125506361
-0.036188788 -0.221010556 0.293545964 0.260917339
0.233440742 0.124302512 0.443280599 0.332367428
0.432245252 0.491751064 -0.111928621 0.116427464 -
0.049038156 0.17402026 -0.27841064 0.333678631 -0.151362825
-0.031891853 -0.064692327 0.006073029 -0.037355238 -
0.318476076 0.337981674 0.246891437 0.288578885 0.093751589
-0.420891726 -0.34165051 0.157391318 -0.165580249
0.215911807 -0.194620098 0.002238489 -0.388806748 -
0.190523104 0.273811916 0.184502188 -0.224187879 0.233272617
0.138233637 -0.148164004 0.154685023 -0.245915265
0.019522855 0.420508533 0.195410327 -0.266947347
0.198039834 -0.047918523 -0.423284664 0.101160769
0.07396131 0.270549579 0.253448902 -0.202941373 -
0.282271401 0.159632681 0.126603595 0.119406261 -0.129194944
0.169553246 0.245473998 -0.282278911 -0.127192839 -
0.239415313 -0.062560274 0.082720328 -0.310482458 -0.284210565
0.037528403 0.13137936 -0.09095452 -0.065216984
0.356570642 -0.107165344 0.247076908 0.405942647 -
0.02267629 0.057316289 -0.290850071 0.188111673 0.251217627
0.153725008 -0.027619436 -0.331151065 0.427747014 -

20800 0.248158118 0.395013541 0.105949762 0.417160528 0.185363049

20801 -0.0438256 0.453972933 -0.211987552 0.352608841 0.237015739

20802 0.199869255 -0.020175653 -0.269104318 0.31988986 -

20803 0.467259422 0.201811821 0.014133284 0.011453987 0.142777086

20804 0.085131458 -0.244230703 0.082281635 0.060105853

20805 0.033764793 0.169466173 0.047535378 -0.136946301

20806 0.186616941 0.216022093 -0.226501138 -0.208994018

20807 0.369465078 0.096121671 -0.205142398 0.184913051 -

20808 0.204327795 0.224607543 -0.196801023 0.199708794 0.402023948

20809 0.083675749 -0.450696987 0.459194421 0.214505374 -

20810 0.365361532 0.371407719 -0.14929192 0.327252223 -0.423375009

20811 0.367165881 0.255710705 0.370331348 -0.060826574

20812 0.255019494 0.358184626 0.057096923 0.146689251

20813 0.264738508 0.288553659 -0.273157562 -0.299525624

20814 0.474282226 -0.448860418 0.377372754 0.269853019

20815 0.356082098 -0.263079863 0.271586969 0.059043647

20816 0.347659091 0.261732076 -0.344235865 0.233891949 -

20817 0.127774629 -0.020461311 0.017728925 0.023080759 -0.117161067

20818 0.172529333 0.306086475 -0.203039787 -0.08924821

20819 0.521401507 -0.261584508 -0.036562554 -0.438416363 -

20820 0.077049183 -0.375352216 -0.006543181 -0.042640323 -0.295118394

20821 -0.166467119 0.023395371 0.404355283 -0.111874299

0.205819463 -0.120064647 -0.287757116 0.074378793 -
0.205442957 -0.183307211 -0.311768329 0.083804081 0.307152632
-0.156736564 0.013555192 -0.064390499 0.234148164
0.210685727 0.288047545 0.348966382 -0.321727846
0.275365105 -0.12980334 -0.114582796 0.271521552 -
0.196853226 0.025436739 0.032405619 -0.344918723 -0.249889663
0.246311477 -0.192498555 0.008767836 -0.392420394
0.22650444 -0.262742908 0.086032442 -0.181343558 -
0.096503699 -0.265407984 -0.446114603 -0.009105114 0.436727045
0.301901735 0.267516541 -0.082330746 -0.049105913 -
0.024369291 -0.298783176 0.097197328 -0.166440873 -0.241839591
0.320214338 -0.082504324 -0.342771032 -0.319456048
0.104405592 0.252997078 0.211856553 -0.33422973
0.074366553 0.030237389 0.144793163 -0.106659338 -
0.016457517 -0.031434649 0.404894069 0.279454398 -0.228968474
0.296828854 0.371838766 -0.251774441 0.373268522
0.190780985 0.045233516 -0.184830727 -0.214301289 -
0.238382914 0.133221151 0.239050581 0.237482691 0.011750479
-0.323893743 0.121188046 -0.023884828 -0.280064029 -
0.090929189 -0.133562086 -0.208680785 -0.288695285 -0.055043355
-0.158171689 0.085164446 0.195794553 0.266378337
0.196360724 -0.318012874 0.386036733 -0.149197513

20844 0.226366265 -0.05770126 -0.474857869 0.185994641

20845 0.336143788 -0.063977353 0.088813721 0.422170112 -

20846 0.193114474 -0.353286142 0.025420192 0.338330469 -0.003970182

20847 -0.081125482 -0.354753597 0.031949864 0.17347216 -

20848 0.383604984 -0.569100364 -0.29144635 -0.034775621 -0.404084363

20849 0.237188959 0.127324416 0.078896697 0.135809341

20850 0.233952282 0.280981823 -0.270221788 0.006656619

20851 0.031881573 -0.414197341 -0.416833537 0.339853631

20852 0.340415648 -0.235004575 -0.187832365 -0.287492484 -

20853 0.264895871 -0.285728528 0.274407797 0.382719486 0.310220985

20854 0.232179949 -0.024835581 0.330201698 -0.264088034

20855 0.384431773 -0.251493177 -0.281558065 -0.235737714

20856 0.103241337 -0.018101674 -0.074813515 -0.460383541 -

20857 0.218560848 0.178923217 -0.087590587 0.288342481 0.073697643

20858 -0.293325058 -0.095718326 0.05757028 0.1754285 0.268790232

20859 -0.040828208 -0.183319905 -0.163198144 -0.386065275 -

20860 0.233658141 -0.154633527 -0.234335308 0.142303654 0.297075429

20861 -0.260010687 -0.331319447 -0.173432443 0.403450678 -

20862 0.221792671 0.39790428 0.163987983 -0.161247049 0.126787617

20863 0.173906083 0.133086987 -0.03055518 0.414491243

20864 0.180807194 0.402868392 -0.129480343 0.211096139 -

20865 0.023972282 0.362889928 -0.202101108 -0.31481639 0.253671688

0.168642536 -0.194885996 0.179135658 0.019385915 -
0.161949107 -0.178421612 0.251700322 -0.292857749 -0.17084278
-0.275130491 0.149869042 -0.098643502 0.122298751
0.147723207 0.166915755 -0.401371608 -0.173575206 -
0.205140441 -0.349505685 -0.044180979 -0.125631433 -0.274119089
-0.136037171 -0.219667983 -0.225569915 -0.145695716
0.001559168 0.287559304 0.110416450.185677275 0.039024756
-0.259710976 0.17157498 -0.297919157 0.240503793
0.260899698 -0.191261063 0.160939232 0.022398948 -
0.138245186 -0.204495016 0.049784805 -0.12766479 0.006906103
-0.268136255 -0.096370487 -0.10361491 0.373479031
0.19818997 -0.099138033 0.273648228 0.049305388
0.090349791
KEGG_ADHERENS_JUNCTION 0.061115391 0.308193058
0.145630178 0.26266905 -0.418186276 -0.152974288 -
0.043947767 -0.30195429 -0.067889228 -0.469726518 0.007088001
0.101970639 -0.006297493 0.042485128 -0.481999626 -
0.440524529 0.320602871 0.174540861 -0.275215115 -0.218399052
0.419821018 0.389046031 -0.032925964 0.407889873 -
0.14807334 -0.051973374 -0.530126989 0.003045042 -0.037281348
-0.338235929 -0.081382456 -0.114945362 -0.131169139 -
0.360398928 0.221793562 0.049951657 0.04899762 -0.229173465

20888 -0.092069577 0.17258838 -0.0462587 0.149009093 0.35525779
20889 0.245208871 -0.059059287 0.23478579 0.05847604 -
20890 0.003119542 0.067120342 -0.273422179 -0.170455483 0.215074427
20891 0.345530032 -0.012340888 0.397982956 0.317167577 -
20892 0.350196144 0.313604589 0.187980172 0.347176057 0.100360763
20893 0.230723502 0.213929089 0.129519085 0.053562598 -
20894 0.081261236 0.107679595 0.382856919 -0.212705077 -0.44343152
20895 0.171030475 0.066615622 -0.000467993 -0.218190594 -
20896 0.064081981 -0.39592005 -0.244231798 -0.016093464 -0.191445469
20897 -0.20347129 -0.28245643 0.39451741 -0.033124 0.155757116
20898 -0.093268241 0.050148823 0.237220456 0.15173424 -
20899 0.196048527 0.046292054 -0.080683952 -0.183401789 -0.026936956
20900 0.147682664 0.010392332 0.240595607 0.181620518 -
20901 0.058355563 -0.118083647 0.184822296 -0.007131798 -0.429927821
20902 0.146773642 -0.133656267 -0.029035789 -0.156726028
20903 0.197254339 -0.392056328 0.405060357 -0.084863311 -
20904 0.192116349 -0.274566871 0.046986256 0.330470441 0.088533442
20905 0.261378449 -0.375695639 -0.479432071 -0.475929489 -
20906 0.100600055 -0.355439312 0.003524845 -0.186192907 -0.138273322
20907 0.04297779 -0.433297387 -0.375528266 0.258660067
20908 0.339088918 0.235138194 -0.103041972 -0.38958507 -
20909 0.356087907 0.104204624 0.22050692 0.233110869 0.196367889

0.407844463 0.210178064 0.314040169 -0.1794074 -0.288912339
0.209030433 -0.149408726 0.048030629 0.31222594
0.062873493 -0.066471607 -0.340382876 0.020621067 -
0.140631364 0.368542413 0.046302808 -0.015364743 0.202129106
-0.02287751 0.125712283 -0.218699249 -0.143257468 -
0.218409183 0.340988237 -0.004314994 -0.061396003 -0.333461579
-0.013609948 0.05977393 0.280810708 0.339989949 -
0.319225438 -0.051240672 0.224030942 -0.323331233 -0.235407731
0.173846912 0.387672804 0.413799201 0.352731808
0.067030041 0.100681224 0.003237256 -0.390978209
0.079950118 -0.228318096 0.042984409 0.323789004
0.358103016 0.239764484 0.179166027 -0.08915669
0.344636352 0.169062621 -0.358239556 0.100131209 -
0.285113666 -0.458863877 0.08054481 -0.016278558 0.031885024
-0.276097986 0.221927071 0.007451497 0.153620328
0.15278893 -0.362547675 0.155470119 -0.124892182 -
0.314816728 -0.092486424 0.295009601 -0.13048947 0.143926906
-0.249551168 0.315878227 0.196291915 -0.344759637 -
0.168153593 -0.094971846 0.260207491 0.002183237 0.04250959
-0.093037714 0.380491254 -0.188794518 0.077317688 -
0.110973748 -0.041956573 -0.267717753 -0.43377418 0.500916337
-0.121685405 -0.357883735 0.095568791 -0.173377421

20932 0.155979201 -0.036298091 0.393466108 -0.213707817

20933 0.315295982 0.121637682 -0.37994217 -0.162371545

20934 0.11052371-0.141300801 0.335399005 -0.10189399 0.450757462

20935 -0.079791654 0.101105677 -0.446625328 -0.44665957

20936 0.127971696 0.393157376 -0.379637129 0.276810287 -

20937 0.300231815 0.342028708 0.360111087 -0.309472121 0.329608381

20938 -0.04355819 -0.324110206 -0.108888785 -0.115593376 -

20939 0.113866803 0.118485625 0.049463733 0.221389251 -0.062131284

20940 0.18562063 -0.329109825 -0.378291561 -0.093459051

20941 0.149186409 -0.135084701 -0.36828692 -0.140616942

20942 0.326501128 -0.334782834 -0.235841758 -0.260437234 -

20943 0.1368361 -0.081670356 -0.052461947 -0.261379106 -0.307110479 -

20944 0.376854105 -0.39443989 -0.098264208 0.332315935 -0.096667469

20945 0.024817452 0.02728765 0.213166175 0.459280483 -

20946 0.264995625 -0.47416656 0.323031238 0.168878977 -0.194814819

20947 -0.292860326 0.105658332 -0.167589697 -0.336108041 -

20948 0.286283765 -0.152586269 0.311144921 0.246432159 0.291925584

20949 0.077595111 -0.39269043 0.120281325 -0.316530314 -

20950 0.09473959 0.431128472 -0.026922532 0.027077694 -0.349809103

20951 0.106146959 0.101084429 0.435961587 -0.176732693

20952 0.165433888 0.357945216 -0.308390794 -0.436976791 -

20953 0.04453659 -0.195991832 -0.446629332 0.352650814 0.2001783 -

0.118157295 0.250400839 -0.118808673 0.091961985 0.473873961
0.278620457 0.351458927 0.082541749 0.085001371
0.333202119 -0.177580168 -0.128800296 -0.471734864
0.235383755 0.29894632 -0.250977357 0.16260508
0.128826866 0.109993965 0.035497202 0.178403763
0.132063849 0.295298853 -0.260379989 0.307337762 -
0.369937338 -0.290910002 0.262552622 -0.375100045 -0.457108636
-0.165487045 -0.019253756 0.329480095 0.232151952
0.131467079 0.396546634 -0.214594175 0.377053304 -
0.186194616 0.40465109 -0.11078647 0.45567872 0.54860599
0.341423947 0.251928461 -0.357759717 -0.376334894 -
0.250856837 0.051113401 0.185930047 -0.394229461 -0.370333095
-0.025936972 -0.362308059 0.304143842 0.084466074
0.206307015 0.318194284 0.370895593 0.427607795 -
0.103683088 -0.108619666 -0.280944568 0.190986597 0.372408317
0.382500373 0.065030076 -0.218425156 0.258959556
0.305914158 -0.201584187 0.419459844 0.381016627 -
0.012006184 -0.217017644 -0.271249153 0.010228945 0.348730024
0.088975091 0.339733999 -0.09215975 0.394647114 -
0.348688068 -0.038554841 -0.262919839 -0.141772328 0.261943864
-0.017506277 0.383929198 -0.190467977 -0.112105037
0.118079338 0.007545366 0.421589262 -0.085617606

20976 0.04000105 -0.181870164 0.380468081 0.0274354 0.298821237

20977 -0.340055059 0.081941782 0.073871749 -0.426112631 -

20978 0.132953011 0.166598552 0.449500539 0.18848663 -0.270069503

20979 0.315247468 0.314050126 0.28719901 -0.194263374 -

20980 0.236588714 -0.211549655 -0.021373559 -0.022789619 -0.358334684

20981 0.192428487 -0.037324301 0.024086425 -0.279979041

20982 0.04209348 -0.111851857 -0.329060343 0.146597925

20983 0.142331474 0.066975783 0.088801525 0.132243503

20984 0.321586036 -0.04902407 -0.318568716 0.2729262 0.119700092

20985 -0.116412093 0.181662611 -0.187877621 0.434592308 -

20986 0.065796943 -0.24045773 -0.125663335 0.232033589 0.330447361

20987 -0.23899912 -0.111739792 0.021175842 -0.020804341

20988 0.065453459 0.249045613 0.283264396 -0.281851177 -

20989 0.055492577 -0.087323951 -0.318427159 0.127323232 0.32620204

20990 -0.194760034 -0.172402239 0.378902706 -0.345967995

20991 0.182044605 0.432272294 0.189665433 -0.025268351 -

20992 0.335390236 -0.268752175 0.07574725 -0.182306991 -0.318286561

20993 0.230909701 0.187274358 0.169858825 0.328170647

20994 0.418388208 0.286995622 -0.338762019 0.305810056 -

20995 0.093367336 0.017188052 0.110631697 -0.211896886 0.357950497

20996 -0.342546635 -0.199614435 0.194974789 0.234103026

20997 0.175657295 0.117716309 -0.221410147 -0.109988398 -

0.350315033 0.162879921 -0.221833179 0.067886315 -0.040887989
0.182010956 -0.169005804 0.473562239 0.476263482 -
0.235022702 -0.142032314 -0.249160111 -0.126951586 -0.34340422
0.054711538 -0.117079348 0.279301032 -0.183703313 -
0.088750712 0.170608541 0.008658789 -0.133284243 0.110206054
-0.161554978 -0.195596218 -0.000400174 -0.216791732
0.028327532 -0.282497501 -0.240633233 0.069890799
0.09009975 0.193221219 -0.167248618 -0.219121336
0.320344741 -0.130571088 -0.195628067 -0.028440128
0.133188487 -0.201144029 -0.031885181 -0.069093277 -
0.143605046 0.200100735 -0.062975994 -0.180982121 -0.004275949
KEGG_TIGHT_JUNCTION 0.189297724 0.088702609 -0.074968671 -
0.046533119 -0.193842661 -0.15429945 -0.105015341 -0.194269209
-0.262032062 -0.28714931 -0.042219588 -0.02881183 -
0.09063713 0.267915232 -0.327677116 -0.167182852 0.161312683
0.061738198 -0.107709489 -0.254341279 0.170851755
0.201553589 -0.102669116 0.089099895 -0.069583981 -
0.088855905 -0.201366938 0.034197524 -0.18351687 -0.160585306
-0.084695041 -0.284311413 -0.083796148 -0.054885277
0.169517139 -0.103927333 0.129854082 -0.136739848 -
0.020576045 -0.039565954 0.086160356 -0.011634441 -0.031483688
0.079982328 0.000985484 -0.07673337 -0.036541791

0.035284167 -0.021602853 0.031301213 0.14400414
0.066732421 0.280963725 -0.00607553 0.264459763
0.062341207 -0.067677278 0.237005389 0.006520228
0.352242943 -0.090658041 0.000558513 0.059538265
0.066364701 0.006723379 -0.236637188 -0.196350756
0.424774699 -0.015300951 -0.144370607 -0.050544361
0.008107035 -0.015876465 -0.265619046 0.018264007 -
0.073658847 -0.150640069 -0.112193705 -0.064354001 0.136307949
-0.12768657 0.242002465 0.099787844 0.12100632 -
0.144443296 -0.146578112 0.276587066 -0.058190768 -0.162848156
-0.158625039 0.08441045 0.029022157 0.057492055
0.140638857 -0.098613052 0.025124027 0.155568828 -
0.258613897 0.193692622 0.240703267 -0.09045274 -0.138245048
-0.122460387 0.031598736 -0.149806709 -0.256018341 -
0.015046218 0.075191223 0.264165618 0.118842673 0.176945704
-0.191008379 0.239800633 0.339422861 -0.151826277 -
0.106621272 -0.053525817 -0.260276642 -0.192608944 0.024560813
0.063891423 -0.031479703 0.173362614 0.16927137 -
0.191465757 0.002499002 -0.186016824 -0.024453658 0.18459065
-0.031644173 0.093447981 -0.214210409 -0.056956441 -
0.127020921 -0.130539394 0.0554091 0.014080058 0.316813108
0.03938312 0.333296754 -0.05364604 -0.075343981 -

0.122172768 -0.168851643 -0.064479333 0.347048414 -0.038143895
-0.04289844 -0.107009055 -0.026330136 -0.066586703 -
0.04430423 -0.103577581 0.082867841 -0.070146791 0.08310638
-0.20449914 -0.15518416 0.015417261 -0.2629141 0.091695344
-0.246151721 -0.055657313 -0.120635641 0.111517961 -
0.102815043 0.224812431 0.121431847 -0.203430601 0.016597754
-0.010160676 -0.091761043 -0.026971389 0.131276646
0.198299052 0.343150262 0.014871417 -0.031820821
0.028512388 -0.193439379 -0.107572501 0.217784521 -
0.211357769 0.039741378 0.16299216 0.068050191 0.222867756
-0.091103687 -0.083866477 0.165150612 -0.057623836 -
0.059290913 0.210254557 -0.060536219 -0.20818681 0.197554121
-0.258783163 -0.029357832 -0.195880006 0.011658227
0.093389177 -0.097529467 0.159711405 0.044241134 -
0.008355916 -0.012287413 -0.223746759 -0.057046074 0.070277672
-0.159210201 0.046132221 -0.099520235 -0.067472389 -
0.017964599 -0.067797667 0.0509251 -0.178938969 -0.078122723 -
0.131240084 -0.09938495 -0.088694012 0.343470745 0.161191268
-0.042858009 -0.016661779 -0.01487835 -0.076536649 -
0.244440802 0.204092632 0.085397535 -0.122515159 -0.089437578
-0.109511303 -0.139235421 -0.087462649 0.287185607 -
0.163908227 0.014528343 -0.049851564 0.050713121 0.189748986

-0.157762664 0.017284682 0.273252884 0.147025945
0.172460751 -0.041419959 0.218962057 -0.265005279 -
0.194017326 -0.068744932 -0.051779714 -0.119425452 -0.030175037
0.102606369 0.03523046 0.356407806 0.157016076
0.18716191 0.148052942 -0.30811336 -0.04570881 -
0.157732063 -0.14117793 0.069270637 -0.067150711 0.172409937
-0.040479075 0.023977725 -0.162032655 -0.195866538 -
0.0766794 0.295431339 -0.103890252 0.01366589 0.028825896
0.205458444 0.056219042 -0.133597312 -0.033415222 -
0.041120805 -0.127841105 -0.167123203 0.158452892 -0.048570542
0.048114352 -0.036644713 -0.187131074 0.210591986
0.187415547 0.024928792 0.222508574 -0.010107488
0.219804784 -0.06576296 -0.111768133 0.114943026 -
0.034116085 -0.213942723 -0.205917909 -0.125691046 -0.041938512
-0.106063911 -0.211638045 0.140004218 -0.02291091 -
0.018987738 0.126023107 -0.031423338 -0.118721481 -0.044040611
-0.018338685 0.003320035 0.031298502 0.05564286 -
0.028491212 -0.040052706 -0.063821892 0.178731465 -0.061147355
0.035713599 0.120681629 0.282785365 0.135067037 -
0.330824976 0.003372496 0.16147991 -0.09496295 0.12930326
0.196183076 -0.20430927 0.290159307 -0.126810747
0.012706574 0.188385995 0.08594667 0.16857759 -

0.059877159 -0.067395405 0.044026291 -0.071176685 -0.169523264
-0.268059777 0.272991545 0.094007067 -0.254425899 -
0.222655359 0.245193657 0.138993378 -0.088993227 -0.044369293
-0.072176407 0.072267749 -0.080714001 0.12137851
0.090068671 -0.098499301 0.116518544 -0.127079327 -
0.145721609 0.047267618 0.01768237 0.217467742 0.063793402
-0.103296522 0.09321528 -0.109636893 0.264939577 -
0.070170622 0.028510647 -0.238675623 0.25135341 0.152275495
0.111461679 0.109494324 -0.158254525 -0.26056487
0.017629907 -0.047492402 -0.105277998 -0.230865635 -
0.277228983 0.027986858 -0.169374099 0.162747065 0.171461712
-0.032056785 0.037881032 0.204278226 0.172193385
0.086487425 -0.024251593 -0.284832771 0.056400423
0.218241568 0.330478387 0.185637346 0.009644244
0.052304827 0.245277874 -0.285522826 0.069371872
0.120072187 -0.045306545 -0.210602989 -0.188468259 -
0.207059923 0.044559685 -0.165756301 0.024125409 -0.027812934
-0.020951524 -0.18784564 0.063131713 0.086763388
0.018546926 -0.070059007 -0.049752808 0.172935962 -
0.11703762-0.303579544 0.147001130.151939449 0.08121672
0.142965876 0.329155297 0.067647465 0.129712638
0.138798254 0.123206353 0.017894375 0.146328157 -

0.000482144 -0.027195227 -0.09870956 -0.110881221 0.311652539 0.061457461 -0.136101192 0.349430353 -0.068962425 0.30014666 -0.291542943 0.066528232 -0.18819336 0.022320934 0.08827273 -0.117157122 0.353814773 -0.152573708 -0.240205201 -0.026379285 0.127396157 0.147422881 -0.156591844 -0.115666375 -0.163597863 0.274241665 0.236527995 -0.093867582 0.281294235 -0.082019882 -0.273247923 0.208031047 0.149954922 -0.051543639 -0.183932718 0.023437462 0.321623114 -0.060578859 -0.137177761 0.162537426 0.149118876 0.012389313 -0.167548856 0.058828122 0.082198708 0.138889052 -0.214222844 -0.030093065 0.16967038 -0.140309535 -0.207437467 0.082146947 -0.190059938 -0.129850762 0.199041335 -0.012127949 -0.190761123 0.382329642 0.066319411 0.003223648 0.167881999 0.095909333 0.107438184 -0.061584743 -0.107593714 0.074810255 0.088640659 -0.168623342 -0.073760776 0.176255976 0.19320642 0.256145506 0.369080219 0.078906829 -0.060800473 0.2410569 -0.053915717 -0.048401987 -0.008087142 -0.019186003 0.363413791 0.031052085 0.276612369 0.242743244 0.282868224 0.082753424 0.289333301 0.083260617 0.091039994 -0.016788516 -0.023945093 0.049834156 -0.011853581 -0.122589951 -0.115301609

-0.181579525 0.208432514 0.164329115 0.03779057 -
0.136891409 -0.291227517 0.033051583 -0.184339761 -0.062805129
0.218145894 0.153719465 -0.177225892 -0.042807308
0.046085882 0.055670262 0.09746824 0.037951573 -
0.175569078 -0.250328882 -0.133448815 -0.250535095 -0.098023351
-0.126303286 0.026146352 0.171360673 0.142846046
0.075058204 -0.016014299 -0.12379287 0.060471922 -
0.127291606 -0.143096014 -0.289246116 0.000906586 0.171710261
-0.049605981 -0.096796396 0.046427909 0.212486315
0.168295525 0.130408956 -0.082819901
KEGG_GAP_JUNCTION -0.193179668 0.003122292 0.082161738
0.262093671 -0.263120041 0.157561789 -0.145797823 -
0.10740918 0.011517672 -0.26115597 -0.051362194 -0.039757361
0.016577546 -0.080433501 -0.25767327 -0.051912179
0.03746622 -0.020533143 0.085499771 -0.389787839
0.371779765 0.139203704 0.247044912 0.318319062
0.075656408 -0.04320156 -0.4240368 -0.344312824 0.102218783
-0.003275696 0.02020056 -0.060392562 -0.377188328 -
0.335248784 0.292887676 0.324910843 0.259100752 -0.217646864
0.004263977 -0.209415617 0.07425959 0.007096215
0.27814803 -0.032762769 -0.092596881 0.177646911 -
0.001864862 0.024558526 0.0912893 -0.092101816 -0.044321807

0.10292659 0.271380051 0.24006696 0.268986789
0.17392878 0.034989728 0.334709084 0.047980819
0.437865811 -0.063139114 0.286466526 0.024143174
0.191554177 -0.142301614 0.017901344 0.022962076
0.224337128 0.016636167 -0.207209756 -0.093971845
0.132303424 0.072474211 -0.140337315 -0.131643058 -
0.249135058 -0.254997755 -0.235323791 -0.23651793 -0.218909622
-0.119551072 0.377229656 -0.045744319 0.074021951 -
0.106522384 0.03884315 0.133253072 0.083585857 -0.180346753
-0.297235267 -0.156642531 0.079856854 0.049008237
0.032154818 -0.178479191 -0.142143391 0.289646872 -
0.031365104 -0.017274976 -0.048222768 -0.131277445 -0.210508243
-0.119116615 -0.277593427 0.127961253 -0.134991534
0.281619622 -0.217595898 0.370636407 -0.010741648
0.268569544 -0.32291207 -0.061294244 0.245797731 -
0.028034682 -0.003418534 -0.348620743 -0.204938224 -0.153580994
-0.141391516 -0.156975421 -0.272162564 -0.00779244
0.010136226 0.132964414 0.033761909 -0.217474184
0.110840126 0.134252724 -0.112459102 -0.165632056 -
0.162757525 -0.227373618 -0.099183848 0.049544709 0.422225969
0.310318997 0.284582246 -0.067896883 0.370800105 -
0.009676359 -0.230485542 -0.07940666 -0.046445888 0.034887324

0.092224508 0.063452296 -0.124030494 -0.160628523 -
0.006800716 0.060968939 0.057077601 0.238642127 0.177060003
-0.084233479 0.006534091 0.265501861 -0.305962601 -
0.045954885 -0.073065054 0.305785965 -0.170838229 -0.06725993
-0.074263916 -0.04859027 0.246936779 0.268190386
0.345869998 -0.173164411 0.103667419 0.109345924 -
0.113622741 -0.112063465 0.164542189 0.37784185 0.343784121
0.172290319 -0.151281564 0.002341863 0.091749913 -
0.090642199 0.237536763 -0.244411014 0.03738806 0.287696046
0.049951957 -0.073648681 0.067682045 0.014523437
0.116346523 -0.014151508 -0.328947319 0.037521087 -
0.156007997 -0.396289157 0.087215967 -0.209469762 0.051405559
-0.102693621 0.012138904 0.1529099 -0.229999918 0.319698868
-0.048751237 0.071191579 -0.249341543 -0.071899427 -
0.278534645 0.301462663 -0.39679648 -0.103216635 -0.057428292
0.140873075 0.259266704 -0.119222168 -0.03720999 -
0.223558014 0.031458658 0.010103825 -0.057301274 0.124791031
-0.031926928 -0.015534939 0.033349864 -0.08072187
0.003645282 -0.156328989 -0.325736742 0.231049203
0.039385874 -0.342138072 -0.183568581 -0.288499868
0.111032232 -0.169314171 0.380147081 -0.074920912
0.156767449 -0.124793622 -0.303244314 0.045432504

21196 0.10027652 -0.254941733 0.375057534 -0.228669762

21197 0.329591122 -0.117529211 -0.056049257 -0.236976108 -

21198 0.176358429 0.163330903 0.200814684 0.119280741 0.398929848

21199 -0.162459964 0.336857408 0.121680265 0.025202241

21200 0.037329083 0.063666536 -0.151207243 -0.208572751 -

21201 0.075403591 -0.081103295 0.165951795 0.341942108 0.245232105

21202 0.069563078 -0.057335026 -0.232615531 -0.429297627

21203 0.088147791 0.027897432 -0.024679484 -0.177752341 -

21204 0.294957217 -0.03525212 -0.171570859 -0.103149588 -0.125095824

21205 0.112278838 -0.063970151 0.097401194 0.095856655

21206 0.041089534 -0.319553894 -0.122188644 -0.08141847

21207 0.29547206 0.051575021 -0.06901312 0.032592798

21208 0.10603754 0.368361132 -0.080352636 -0.211782376

21209 0.335897367 0.079338097 -0.070729189 -0.306789074 -

21210 0.104462663 -0.160894445 -0.138461917 0.014434768 -0.162824632

21211 0.210493916 0.334233978 0.083955431 0.327323044 -

21212 0.197823513 0.010482771 -0.270348433 0.083822909 0.247131857

21213 -0.114813771 -0.07211881 0.119159073 0.010564768 -

21214 0.040129144 0.173934521 0.016429294 0.019386598 0.262880181

21215 -0.034813134 -0.230332818 0.346793631 -0.029631902 -

21216 0.302618565 0.25088683 0.08882307 -0.108487236 0.11253717

21217 0.027094425 0.042585317 0.366250811 0.109108093

0.104754224 0.209230988 -0.185454057 0.145479171 -
0.243754155 -0.02938994 -0.227326149 0.128645075 0.032118921
-0.331247944 0.17707399 0.016333411 0.133642698 -
0.057748765 0.259875561 -0.15171107 0.239975204 -0.370349012
0.13159782 -0.282719752 -0.066657816 -0.097715582 -
0.181167893 -0.235799339 -0.335382537 0.170846863 0.127354197
0.302960865 -0.086310437 0.379894219 -0.228210832
0.255961252 -0.089316486 0.268347349 0.033122806
0.319502396 0.19202189 0.347818138 -0.008811785 -
0.146678331 -0.097341415 -0.211989763 0.133810363 -0.017658173
-0.098425935 -0.228158636 -0.112298826 -0.237595772
0.227338204 0.135846913 0.026367657 0.302392889
0.200010053 0.211917674 -0.17122482 -0.329063462
0.006181325 0.102197735 0.354871037 0.378602605
0.104904046 -0.205725743 0.209697783 0.219748605 -
0.143745809 0.258810163 0.313693281 -0.247555123 0.04067244
-0.211053282 0.026866432 0.180641907 0.069471542
0.099285541 -0.261991476 0.180230378 -0.34654793
0.209568829 0.032041513 -0.005483708 0.196230919 -
0.282327712 0.28216551 0.219515394 -0.197311589 -0.156475896
0.065417717 0.361648593 -0.083234612 0.002392852 -
0.261700785 0.193090027 0.01377791 0.118757456 -0.124414968

21240 0.132549059 0.229664947 -0.167239032 0.023634524

21241 0.201767557 0.320199485 0.144760213 -0.00574687

21242 0.287846123 0.075152219 0.157839866 -0.282765684

21243 0.193215376 -0.056325695 -0.086475325 0.186336628 -

21244 0.056086788 0.087704206 0.005391098 -0.001222933 -0.014321711

21245 0.183466208 0.065700833 -0.382681248 0.08593823

21246 0.357090085 0.13397978 0.03268225 0.006032969

21247 0.155163605 -0.153235024 -0.157403606 0.272955611

21248 0.168284974 -0.181953944 -0.104645398 -0.103201145

21249 0.24385067 -0.116765673 -0.156666462 0.006908702

21250 0.197160009 0.32587222 -0.110336993 0.030529561

21251 0.066555081 0.000397393 -0.115639318 0.050464014

21252 0.304059897 -0.087141871 -0.147146092 -0.054701951 -

21253 0.156656423 -0.126804182 0.221905142 -0.251658242 -0.036734242

21254 0.216433906 -0.078202327 0.208414676 0.231988769 -

21255 0.090436773 0.038643246 -0.324235402 -0.042289885 -0.161564268

21256 -0.18814843 -0.238349449 0.051087135 0.16139321

21257 0.282831778 0.268180234 0.411496728 0.352755296 -

21258 0.17814202 0.306584435 -0.122993057 -0.028942069 -0.074167554

21259 -0.241300229 0.364783146 -0.198600858 0.025384764

21260 0.055907762 0.035420656 -0.029561316 -0.071897061 -

21261 0.151219424 -0.249012599 -0.109857637 0.297006765 -0.179177546

-0.169912964 0.125487627 0.073924237 0.155474615
0.310240537 0.161770172 -0.140806359 -0.146364008 -
0.277473031 0.089407298 0.031620535 0.028702171 0.156427262
0.17919293 0.105242812 0.17678913 -0.201015879
0.248199585 -0.090358263 0.095076157 -0.293621855 -
0.335039973 0.00725178 0.053626133 -0.253651935 -0.036414017
-0.027093282 0.10682337 0.118295257 -0.01206186 -
0.193031679 -0.201677225 0.054756724 -0.076329646 -0.113697085
-0.160831071 0.026557999 0.027081716 0.072909646 -
0.081099344 -0.02315555 0.171910503 -0.042289852 0.100514447
-0.251426191
KEGG_COMPLEMENT_AND_COAGULATION_CASCADES -0.383137061
0.027020415 -0.334821383 -0.410760282 0.341261538
0.098722408 0.251891053 0.519612267 0.207644573
0.307508316 -0.084286262 -0.320367734 0.26879666 -
0.178930501 0.315451277 0.210425362 -0.126124699 0.147327691
0.369736135 -0.220656683 -0.280711952 0.294297673 -
0.086187044 -0.40136701 0.441664479 -0.022967477 -0.334878088
0.203842132 -0.321823846 0.381436928 -0.155588149
0.34800102 -0.169951951 0.255216005 0.483292028
0.301664488 0.036165828 0.284832463 -0.172141214 -
0.373187604 -0.281055483 0.531695811 -0.32994298 0.005262321

21284 -0.268043315 0.215147644 -0.298317729 -0.175157379

21285 0.178144812 -0.004632638 -0.156159423 -0.245991789

21286 0.33090849 -0.325234313 -0.115927958 -0.199628608

21287 0.353910833 0.338243133 -0.167731521 -0.004894506 -

21288 0.136709637 0.103707315 -0.227948448 -0.487876414 0.108160045

21289 -0.118579625 0.400831748 0.041190439 0.65819206 -

21290 0.026128676 -0.007216977 0.039890058 -0.143466373 0.384118471

21291 -0.271520647 0.582362497 0.110004512 -0.409021498

21292 0.453098282 -0.356505668 0.239221721 0.187965972

21293 0.191846945 0.449695792 0.271187340.058845348 -0.46167459

21294 -0.232111017 0.262841423 0.118307227 -0.233787773

21295 0.335413973 0.099524898 -0.171578219 -0.390832756 -

21296 0.277412814 -0.12187912 0.123355425 0.163278593 0.005452906

21297 0.271222665 0.227560105 -0.102971362 0.412045549

21298 0.392156213 -0.325794702 0.013181773 -0.107273474 -

21299 0.269571338 0.295833436 0.353486299 0.229938209 0.008650309

21300 -0.301806195 -0.317757846 0.028758368 -0.059900007

21301 0.145896072 0.260967 -0.440202979 0.227812237 -0.216651882

21302 -0.146858542 -0.10004017 -0.129030734 0.386313922 -

21303 0.298254124 -0.131824594 -0.242926649 -0.229063015 -0.035647508

21304 -0.086020714 -0.448348363 -0.259681896 0.19815685

21305 0.122361009 -0.195110913 -0.262058638 0.024412452 -

0.133641854 -0.029356436 0.30807865 -0.451206191 -0.153693759
0.202893755 -0.322295002 -0.077716463 0.126575678
0.505879133 0.38894979 0.388286664 0.014540929
0.312791038 0.388860353 -0.417569626 0.153329093
0.031299552 0.22989442 -0.55621877 0.343344254
0.155906928 0.523016674 -0.309704947 0.114684073
0.007135698 0.443197129 0.224158439 0.134917515
0.609406768 -0.015364868 -0.426357267 -0.156383552 -
0.165062212 0.098387757 0.152617564 -0.344632523 -0.394647466
-0.439969239 -0.308360073 0.337854908 0.370506823 -
0.187266335 -0.291179838 0.244894485 -0.221737339 0.175403266
-0.437142736 -0.079640461 0.403221515 0.057662549
0.231852609 0.287946835 -0.203172148 -0.270873494 -
0.226784045 0.437345227 0.307217444 -0.113946341 -0.412149381
-0.471058651 0.353736122 0.318117307 -0.023384336
0.655563821 0.40797141 -0.217383198 0.380731518 -
0.496588348 -0.200118736 0.132864893 -0.419558886 -0.557102603
-0.229598583 -0.308150081 0.573230222 -0.45490508 -
0.153883811 0.305946198 -0.264072387 -0.316071394 0.480174697
-0.183275461 -0.013850886 -0.366803269 -0.055530827
0.294490397 0.397697127 0.28422833 -0.345710734
0.415540357 0.100097258 0.343013643 -0.228135966

0.514726347 -0.320059867 0.030233507 0.335455912 -
0.456021253 -0.415390986 0.126261323 -0.078218228 -0.368501792
-0.356893035 0.356897093 -0.394990286 -0.333228858
0.412888089 -0.195909943 -0.176670251 0.054574436 -
0.39676728 0.270444382 0.44191121-0.307504132 0.267185472
0.336888798 -0.089017206 0.259518117 -0.389373637 -
0.277122554 -0.157430838 -0.046684424 -0.377724912 0.158462669
0.323728647 -0.337154171 0.329210093 0.254115873 -
0.108144888 0.582331284 -0.247267707 -0.350621293 -0.008357008
0.42640226 -0.333148 -0.139715033 -0.394323907 0.057170477
-0.415284828 0.003045563 0.178513009 0.43573037 -
0.131298673 0.469366156 0.393805508 0.210202347 0.246704712
-0.307921824 0.366371304 -0.22835227 -0.381912248
0.271580694 -0.426809181 -0.007705372 0.445055433
0.465604742 -0.440636693 -0.407390218 0.112032872
0.316172039 -0.352047957 -0.221270805 0.426267319
0.473483003 0.16061801 0.250422393 0.455431121 -
0.426707902 0.263450715 -0.123573933 0.311202881 0.266841072
0.417106213 -0.464164761 0.243225901 -0.356709827
0.459836805 -0.295440328 -0.462967691 -0.263106199 -
0.066777494 -0.415360592 -0.098630139 0.487964802 0.327458393
0.588805466 -0.307113299 -0.206717202 -0.063331742

0.07249917 0.127519886 -0.306397592 0.308207248
0.355223978 -0.415382395 -0.055127866 -0.326138683
0.191546652 -0.49581494 -0.212744895 -0.037906485 -
0.324818017 -0.021308516 -0.438619192 -0.357209417 0.281367964
-0.410191862 -0.096267338 -0.110119798 -0.167670667 -
0.149705307 0.336945243 -0.344006562 0.180452784 -0.413020133
0.262817997 0.018655178 0.320714967 -0.302975739
0.020585723 -0.330329307 -0.084006033 -0.269207574
0.372847274 0.249628586 -0.030114408 -0.299149625 -
0.287900478 -0.034420708 -0.401470219 0.250648856 -0.37335677
-0.270490509 0.056712883 -0.324180673 -0.085772244
0.077310177 0.268642718 0.181256626 -0.138880655 -
0.12683129 0.392149654 -0.29170237 -0.118551511 0.064382704
0.016850597 0.2633167 0.503069804 -0.112514523 0.031809851
-0.104143702 0.176407927 0.46826129 0.31446708
0.036010113 -0.102882543 -0.071827581 0.167682831 -
0.303323483 -0.046212234 0.051394555 0.364180348 -0.337453919
-0.38761859 0.510047305 -0.45899795 -0.340843762 -
0.133679454 0.338068896 -0.438768291 -0.268060663 -0.357965865
0.325331263 0.323121936 0.330578938 0.032005519 -
0.092588714 -0.192957855 0.48342552 0.38798269 0.055504331
-0.188107861 -0.383113585 0.253139894 0.373568589

0.043182763 0.297518726 0.027756485 -0.105703312 -
0.368966917 0.251864156 -0.218440576 0.332373288 0.059129753
-0.174632812 0.053141365 -0.111014511 -0.408757006 -
0.512859451 -0.277224006 -0.520903808 -0.382670591 -0.083175985
0.469847659 0.217897313 -0.406813051 0.334062712
0.277130202 0.008284659 0.029434043 0.248538668 -
0.296087489 -0.193907194 0.423755453 0.293603415 -0.036997025
-0.180627121 -0.135413161 -0.211587374 0.175673238
0.152582158 -0.156309069 0.323838418 -0.120873058 -
0.018740154 0.346928392 -0.112769102 0.274044457 -0.355480266
-0.364305194 -0.357899587 0.205214964 0.317478985 -
0.338751969 -0.253675615 -0.15699177 -0.064652569 0.192478863
-0.342625151 0.041495858 -0.303104138 0.243260915
0.083838012 0.18052236 0.069792832 -0.243208311 -
0.269805609 0.040684956 0.217944653 -0.449758416 -0.062763218
0.151438068 -0.016846247 -0.315950365 0.194363974 -
0.33177776 0.468426828 0.385494843 -0.252985094 0.196704165
0.321554128 -0.293319555 0.143618881 -0.101499409 -
0.284952056 0.453112118 0.359045205 -0.052194441 0.588551318
-0.423304201 0.018672499 -0.073613337 0.046524589
0.124325056 -0.229545856 -0.125007829 0.118252714 -
0.073524645 -0.069295275 -0.311442307 -0.228003711 -0.311020937

-0.113187413 0.217599391 -0.127887675 -0.214682524
0.161068247 0.45384189 -0.138918509 -0.250422801
0.113308202 0.084126368 0.157551903 -0.557256144
0.132925639 0.11228594-0.029736407 -0.164121867 0.219827815
-0.268245328 -0.193958753 0.27555604 -0.130903405
0.220331374 -0.068099249 0.154546204 0.02661227
0.078461134 0.087339312 -0.06173051 0.057596502
0.115858270.154421788 0.402988 -0.165364749 0.109912154 -
0.028768881 0.370490521 -0.107393271 0.181862125 0.205125752
0.057681619 -0.366101756 -0.00266179 0.051219476
0.352500247 -0.154286431 0.113289710.005018897
KEGG_ANTIGEN_PROCESSING_AND_PRESENTATION -0.249428035
0.501171517 -0.502495899 -0.037060357 -0.066720415 -
0.046908838 0.227623263 0.224571713 0.21468961 0.351793878
0.166378143 -0.341005515 0.092953867 -0.537164421
0.362752265 -0.35749505 -0.134552141 0.53355276
0.035004129 0.593913737 -0.405187532 0.535088861
0.400702404 -0.372246397 0.467982364 -0.125340176 -
0.16483317 0.168967117 -0.079124997 -0.358281184 -0.457049173
0.430452878 0.400908942 0.633976691 0.403340325 -
0.466485524 0.067615119 0.309205266 -0.55308509 0.088202232
-0.349765488 0.190699202 -0.248563958 0.533092511

0.106006473 -0.149606626 -0.242924248 -0.41593994 -
0.452414587 0.442815191 -0.214912779 -0.105032125 0.329790299
-0.480242995 -0.36959638 -0.356440348 -0.362579365
0.465951767 0.275295609 -0.318659417 -0.083551486
0.27669507 0.053680352 -0.285815136 -0.272086432 -
0.23919999 0.235995095 -0.552696078 0.365244022 0.333188236
0.505541057 0.574702236 -0.435339271 0.163806674 -
0.466879198 0.175511483 0.348027686 -0.299677525 0.386509843
-0.157371616 -0.387882407 0.228181059 0.641302224 -
0.245430234 0.43044713 0.306918288 -0.473935994 -0.280852896
0.510648123 0.097713114 -0.436515903 0.110366255
0.052959544 0.531643258 0.539105076 0.515793816 -
0.229984202 0.532496533 0.061668137 -0.255923546 0.470318131
0.119847177 0.190924933 0.426398464 -0.192098543 -
0.115021537 -0.447704068 -0.265266109 -0.399295987 0.489640511
0.095124646 -0.00569308 -0.231324937 -0.456425624
0.109333052 0.609074209 0.370971843 0.053507654 -
0.284272105 -0.280178357 -0.050453661 0.027725455 -0.250851729
-0.480512335 0.064570805 -0.094238928 -0.343723699
0.082207819 0.03136778 0.665758464 0.535437724
0.485929275 -0.368799076 0.549658383 0.468228812 -
0.202225579 -0.337403663 -0.34804771 -0.310103228 -0.287433257

21438 0.039607968 -0.190625245 -0.468372842 -0.227941018

21439 0.178311644 -0.473169994 0.50318196 0.45821554

21440 0.019759513 -0.00605978 0.462480332 0.224719175

21441 0.53702808 0.411430153 0.198520821 -0.098288926 -

21442 0.092774664 0.279483909 -0.494194854 0.536511989 -0.330536971

21443 0.113996911 0.020252001 -0.032770523 0.032506915 -

21444 0.276915175 0.432298058 0.148879316 0.146872028 -0.337391829

21445 -0.402327649 -0.333648838 0.310537783 -0.345213743

21446 0.035071077 -0.517863306 -0.071332812 -0.226341792 -

21447 0.220674703 0.584299859 0.477313798 -0.321704622 0.410123785

21448 0.216881468 -0.262288905 0.276309199 -0.411225699

21449 0.121493335 0.479959812 0.116744432 -0.056231004

21450 0.20287602 -0.256233488 -0.507342582 0.405688044 -

21451 0.204814242 0.129323331 0.333766994 -0.297520783 -0.223425026

21452 0.197548942 0.340276578 -0.008791705 -0.231914938

21453 0.01259915 0.36472082 -0.343441466 -0.354524052 -

21454 0.472817389 0.221253759 0.405350974 -0.543045719 -0.236192536

21455 0.001162926 0.150238272 -0.255504263 0.366088245

21456 0.359757296 -0.283699609 0.344909921 0.54008923 -

21457 0.108188083 0.082662741 0.225692637 0.194846925 0.060252514

21458 0.282113578 0.183696253 -0.307837243 0.331237983 -

21459 0.251300733 0.425296269 0.22544251 0.488636522 0.123443649

21460 -0.431362477 0.495708151 -0.23143614 0.37273121

21461 0.473537406 -0.026429835 -0.141492217 -0.324367397

21462 0.12700856 -0.351267042 0.033208516 -0.198491215

21463 0.086977855 0.399835213 0.038398759 -0.296557433

21464 0.28833493 -0.281945643 -0.076725082 0.029269986 -

21465 0.18270363 -0.229608447 -0.040871334 5.72E-05 -0.323491394

21466 0.060733799 0.644138735 0.124182233 -0.410013846

21467 0.426728936 -0.38129088 0.176700706 -0.255434535

21468 0.475544327 0.409699876 0.164196272 -0.28456683

21469 0.439660649 0.364820705 -0.193961139 0.559591613

21470 0.002043122 0.345722713 -0.598827985 0.159354215

21471 0.002277948 0.437751225 0.384360673 0.086242518

21472 0.251508714 0.173330532 -0.079355784 0.550896097

21473 0.008228503 -0.467927169 -0.382188377 0.6160037 -0.308874809

21474 0.46881552 0.104884041 0.462005394 -0.298810515

21475 0.331735669 0.16664447 0.592891527 0.396080363 -

21476 0.308291022 0.119581222 -0.295995753 -0.067745822 -0.382667924

21477 -0.278033287 -0.119571797 0.176048214 0.297656624 -

21478 0.41562702 -0.028303975 0.445710611 -0.363476585 0.097898179

21479 -0.294922043 -0.371854545 -0.454143089 0.050646579

21480 0.269371695 -0.34438471 -0.366571976 -0.306431482

21481 0.140028131 0.289266975 0.177324623 0.060352275 -

0.155728654 -0.017264489 -0.487946047 -0.345253262 -0.365031831
-0.207005324 0.011732019 -0.266153493 -0.131305113 -
0.288740446 0.361711054 0.498209446 0.294329601 0.560600286
-0.214783915 0.330999186 -0.258033556 0.161571433
0.361649427 -0.241320236 -0.010274912 -0.06336802 -
0.458848908 -0.474538166 0.32283717 -0.46431895 0.471948421
-0.397571876 0.332216839 -0.525567679 0.151297468
0.235283953 -0.035363435 0.233263819 -0.466483359
0.116530396 0.311334233 0.408861267 0.120650675
0.07774004 -0.167237488 -0.03596593 -0.41542378 -
0.041976782 -0.429121741 -0.410309626 0.28076463 0.320938529
-0.268189614 -0.377150962 0.091973138 0.39317738
0.443200366 -0.334369804 -0.162960961 0.154700967
0.399383674 -0.150276377 0.039309356 -0.163104945
0.529577052 0.447891828 -0.420293794 0.541914337
0.627571091 -0.363193734 0.22985447 -0.183937119 -
0.433591224 -0.177743553 -0.410887668 -0.354063758 -0.176136144
0.40552839 0.152495386 -0.14468747 -0.387859881
0.045296483 0.371756316 -0.430395184 -0.239836408 -
0.135039485 -0.10134673 0.064847735 -0.287084701 0.158848144
0.215352872 -0.142719179 0.182814347 0.052488777 -
0.307960582 0.308947501 -0.144438773 0.504917455 0.011160427

-0.369744581 -0.143710719 0.333046105 -0.317670942
0.083904757 0.441632756 -0.05894897 -0.447343306 -
0.007616157 0.512723594 -0.001726503 -0.024964826 -0.490148873
0.057089648 0.312278718 -0.165879746 -0.423572178 -
0.544380372 -0.090451113 -0.551315061 0.390711732 -0.253836461
0.19902805 -0.022211032 0.071818869 0.286123291 -
0.412310801 0.067945575 0.178228917 -0.38824732 -0.554809158
0.344509401 0.607338243 -0.419065289 -0.370107922 -
0.548255877 -0.331005883 -0.34008446 0.444475508 0.53859915
0.260135266 -0.009226659 -0.004590458 0.296541114 -
0.217296563 0.427787706 -0.474554696 -0.006867437 -0.231517185
-0.087103908 -0.305369222 -0.324340047 -0.492971321 -
0.103798267 0.087313166 -0.217499598 0.298221519 -0.0493868 -
0.152520651 -0.034976387 0.228947786 0.131742306 0.500731858
0.250136326 -0.186198772 0.231364189 -0.304304825 -
0.497238282 -0.252538602 -0.177199949 -0.113720112 0.476050884
-0.409762159 -0.294132118 -0.318601412 0.534692152 -
0.258575558 0.514176008 0.030115184 -0.198105752 0.224991034
0.207295525 -0.411358107 -0.266888278 0.515589563 -
0.116770490.340076575 -0.09987015 0.036771187 0.068853055
0.08856956 -0.387669698 -0.533878243 0.455973262 -
0.109109908 -0.418685483 0.209902402 0.394180277 -0.062837562

-0.36693792	-0.029644569	-0.335636346	-0.057527016	-
0.256864235	0.127089517	0.243639208	0.245355588	-0.042393483
-0.228084562	-0.364540773	0.302208892	-0.113980028	-
0.299317275	-0.263789568	-0.477068305	-0.442747427	-0.175377763
-0.400136952	-0.352584529	0.149711685	-0.177003736
0.425629502	0.300109924	0.102859254	0.062734685	-
0.275142151	0.246238074	-0.275390833	0.317951514	0.24320554
-0.309036642	0.477535222	0.186557863	-0.149715894	-
0.32694769	0.264070886	0.138888562	0.332285571	-0.286218807
-0.357515675	0.076471287	0.44026459	-0.15600102	-
0.216929131	0.230676079	0.205594941	0.244890322
KEGG_TOLL_LIKE_RECEPTOR_SIGNALING_PATHWAY -0.220692678
0.319875525	-0.152257392	0.172819962	0.084023352
0.004037915	-0.122081075	0.211004356	0.264939038
0.34655374	0.198803158	-0.328300572	-0.138036186	-
0.451688856	0.039054201	-0.175782123	-0.021725729	0.359863918
-0.151287829	0.400393636	-0.207322166	0.416284663
0.177598799	0.09020661	0.235305956	-0.343851943	-
0.385053651	0.056369314	0.149107753	-0.204967251	-0.269516107
0.441799812	0.338486657	0.249325416	0.225982095
0.035729127	-0.395060598	0.095985249	-0.3523746 0.288196186
-0.32879985	0.230509597	-0.001626691	0.206960418	-

21548 0.072186136 -0.084723419 -0.165896342 -0.28873411 -0.102584408

21549 0.01294479 -0.306868671 -0.092104983 0.20852937 -

21550 0.198482302 0.153395691 -0.225956863 -0.272041657 0.177991616

21551 0.042810157 -0.217691454 0.134907368 0.361353049

21552 0.130081126 0.083329442 -0.33653147 0.189584523

21553 0.302234222 -0.241704129 0.136581534 0.191730356

21554 0.424125471 0.450960321 -0.380635142 0.23242002 -

21555 0.261248127 0.234036941 0.154781244 -0.067344332 0.237754443

21556 -0.288192031 -0.226838015 0.319603101 0.308068089 -

21557 0.135856305 0.222753505 0.243747765 -0.222371039 0.079091318

21558 0.334484503 -0.115235338 -0.322071307 -0.265603926

21559 0.084496092 0.182456361 0.369170191 0.309042923 -

21560 0.164707406 0.456154087 -0.112582804 -0.245833975 0.33913372

21561 0.069046213 0.024552118 0.242265259 -0.096766697

21562 0.123054685 -0.159889757 -0.156547248 0.075706732

21563 0.007687114 -0.290599625 0.079515315 -0.06241964 -

21564 0.294116154 0.388414148 0.425225284 -0.131121015 -0.218438074

21565 -0.269473608 -0.05164375 -0.011401892 0.081869484

21566 0.034321686 -0.371163361 0.14966132 -0.132769723 -

21567 0.335709748 0.270675415 0.03057498 0.398629623 0.037863095

21568 0.237300062 -0.212852222 0.374683055 -0.312084288

21569 0.036283904 0.094566936 -0.187062211 0.238132981 -

0.004503462 -0.095271772 -0.039564228 -0.028773116 -0.115579996
0.220389227 -0.428768857 0.396144367 0.152225374 -
0.008095952 0.123794882 0.273214093 0.24405787 0.421259695
0.26287396 -0.034020657 -0.326757552 0.092870897
0.283540013 0.134309732 0.38236277 -0.257844669
0.017196865 0.128987578 -0.19443986 -0.280629849 -
0.047485127 0.164586096 0.278308621 0.053770061 0.018189919
-0.257660941 -0.362902119 -0.244764187 -0.224846637
0.31883494 -0.256373833 0.25779637 -0.200016064
0.113956413 0.422389706 0.035822185 -0.192011423
0.263386634 0.041037776 -0.087119594 0.203666411 -
0.235070944 0.051457984 0.293895764 -0.026067109 -0.057709578
-0.180138849 0.008528411 -0.411193015 0.279555332 -
0.219832577 -0.001390378 -0.034003743 -0.241986065 -0.016656832
-0.057582831 0.327977674 -0.12004586 -0.253349539
0.137360409 0.046931045 -0.120361449 0.145883797 -
0.18527837 0.357753288 0.17856558 -0.112203134 -0.235102252
0.103063 -0.046312999 0.210314386 0.095309804 0.371512985
-0.085215574 0.392421415 0.376274544 -0.103641726 -
0.209950734 0.154628577 0.018349647 -0.005210604 0.177958254
-0.061486374 0.050955653 0.105066747 -0.163454771
0.398892541 0.235924443 0.161875125 -0.018040903 -

0.157818252 0.356927964 0.129596405 0.379193335 -0.130972051
-0.079050632 0.077963046 -0.300202617 0.112619254 -
0.427092856 -0.105939044 0.211773884 -0.238138778 0.234520401
-0.208617472 -0.255352083 0.049379313 -0.120729036
0.107103315 -0.198128585 0.21291069 -0.327434527 -
0.101343161 0.263750945 -0.080987821 0.127668738 0.369810125
-0.068351224 -0.343604199 0.211534843 0.256404892 -
0.073295847 -0.341531745 0.367341269 0.085895935 0.119293306
-0.093097628 0.238481811 0.221091132 -0.060889803
0.350858345 -0.003819001 0.241523842 -0.35498089
0.031863057 -0.012231063 0.373061001 0.193852631 -
0.084885399 0.120396707 -0.173703701 0.134455023 0.408978912
0.010985406 -0.239854695 -0.084397177 0.210087562 -
0.128060579 0.322212985 0.182128833 0.133797882 -0.068502201
0.140748656 0.08774374 0.359609679 0.33533806 -
0.288631697 -0.027783727 -0.220434882 -0.226230696 0.099075167
0.245640495 -0.006313598 0.13030916 0.089145122 -
0.221320928 0.044981347 0.243992301 0.009210604 -0.02621425
-0.334029378 -0.246581635 0.052733555 0.011669197
0.135920216 -0.352236761 -0.076432283 -0.22186142 -
0.087783842 0.263698102 0.034464248 -0.354499776 -0.31904807
0.015602769 -0.276656639 0.008304176 -0.336879701

0.066181049 0.239952819 -0.174037427 -0.013391415
0.069424655 0.236045971 0.473895842 0.111750202
0.147842504 0.026498455 0.079570415 -0.152118444
0.099802734 0.241042459 0.055575639 0.22047138 -
0.065600513 0.066992373 -0.183709184 0.395317268 -0.188272126
-0.194389908 -0.339845084 -0.165008665 -0.281958065
0.231928505 -0.139460056 -0.347759198 -0.214898939 -
0.426045197 0.082272552 0.233475317 0.230684044 0.352553463
0.064028369 -0.038927103 -0.15883698 0.003435244
0.326101744 -0.012214169 -0.1693038 0.312355715 0.18295034
-0.300535667 -0.256507423 -0.10966681 0.348091443
0.406725984 -0.418386434 -0.091410854 -0.347078154
0.060310918 -0.228972595 -0.233264607 -0.012231662
0.349325592 0.150728954 0.092765445 0.288166882
0.307854729 -0.086719704 0.279692475 0.131697858 -
0.262053377 0.116480954 -0.408823606 -0.139709191 0.02523105
0.260424425 0.185187893 0.008144821 -0.314424802
0.110397712 0.311232325 -0.096246532 0.080160696
0.260803942 0.037708235 -0.139618284 0.191486013 -
0.279304961 0.057688778 -0.122149915 0.342749947 0.398848569
-0.032016452 0.289348137 -0.215959307 0.417394259 -
0.140951081 -0.38341656 0.21965604 0.024242526 -0.232376977

-0.279492736 0.395474712 -0.228924912 -0.290387352 0.093815814 0.213861756 0.098458802 -0.227187662 -0.305936046 0.363391779 0.300987075 -0.420411853 -0.238682147 -0.332173184 -0.034912647 -0.270710925 0.348791953 0.039573276 -0.034475562 0.396287532 -0.109422383 0.106250645 -0.300784772 0.326060336 0.400270489 -0.334017928 -0.253002762 -0.140251607 0.19626616 0.048794802 0.162299456 -0.403375339 -0.09996097 0.149781433 -0.035530618 0.450114632 0.124630928 -0.324257493 0.119178759 0.08502262 0.058704509 0.055058828 -0.147066224 0.000371272 -0.258759519 0.095454077 0.224934808 0.07439271 -0.243500437 0.161333056 -0.260931979 -0.368573549 0.308037221 0.132511288 -0.065579254 -0.074210866 0.304951624 -0.116354205 0.132959938 0.025572215 0.03907919 -0.050559698 -0.179590683 -0.248927652 -0.186426225 -0.058251278 -0.0757771 0.306945567 -0.079629047 -0.180530726 -0.318500773 0.283692108 -0.106580561 0.270452917 0.241312177 -0.272678648 -0.033876107 -0.117718854 -0.179728512 0.179582804 0.231637391 0.242944629 -0.061246561 -0.070231002 0.221912033 -0.150713433 0.163356335 -0.411407607 -0.318646252 0.196069529 0.029894802 -0.155440661 0.353615978 -0.051704195 -0.133347617 -0.338458306

0.331920855 -0.250246524 0.060969525 -0.232282327
0.314507595 -0.00647447 0.170823654 -0.053101415 -
0.004420613 -0.336154076 0.284855704 -0.167038528 -0.325980094
-0.157073312 -0.123525497 -0.271100062 -0.318980665 -
0.254550425 -0.153969672 0.154869946 -0.046392828 0.16347164
0.08320257 -0.106784348 -0.097795 -0.164670463 0.144671041
-0.186420901 0.189100304 -0.130037012 -0.060611919
0.275771273 0.020858338 -0.070733413 0.025963446
0.051474965 -0.124236994 0.02118808-0.076871123 0.082040748
-0.000159513 0.223685618 0.176471814 -0.127145838 -
0.110880069 0.121142884 0.126362655
KEGG_NOD_LIKE_RECEPTOR_SIGNALING_PATHWAY -0.329036533 -
0.049472493 -0.082428253 0.078357782 0.309021835 -0.251059671
-0.003856407 0.344967235 0.291348645 0.328827637 -
0.007929004 -0.283796525 0.113965197 -0.515158461 -0.07849232
-0.142766587 -0.106509812 0.011259873 0.062457833
0.263535857 -0.190035177 0.451344237 0.195255487
0.145398561 0.371006691 -0.375605982 -0.384453301
0.10398149 0.097692825 -0.177671663 -0.262314594
0.418370248 0.123959042 0.187883331 0.304211930.317893882
-0.304631621 0.077697 -0.179833031 0.172988188 -0.378708458
0.250066469 -0.066259903 0.078139828 0.040253222 -

0.259349403 -0.114542621 -0.313610298 -0.350279658 -0.074923021 -0.386791326 -0.003596764 0.324347146 -0.050110603 0.025984178 -0.292521548 -0.186484456 -0.012764286 -0.159914937 -0.366496867 0.18734969 0.161246438 0.286875777 -0.070486411 -0.31787361 -0.232664276 0.391997047 -0.266253522 0.386933921 0.108905026 0.500859412 0.526460307 -0.308798282 0.209628629 -0.285380812 0.029490183 0.081797373 -0.155770227 0.319067895 -0.172009443 -0.153183711 0.295687735 0.292266323 0.004474647 0.260756305 0.272784218 -0.210857462 -0.058614942 0.182835959 -0.184896918 -0.094751944 -0.179902658 0.134083247 0.097498005 0.056268213 -0.057370115 -0.308299062 0.251238703 0.117960717 -0.380550629 0.365755662 0.164017694 0.135478272 0.250719022 -0.065949841 -0.034820513 -0.199274971 -0.276885348 -0.028740696 0.051075882 -0.338389668 -0.006894095 0.040708066 -0.166318185 0.261412739 0.219161053 -0.088831359 -0.23785491 -0.268297956 -0.058749939 -0.03054396 -0.273584448 0.030755167 -0.367872033 0.062078567 -0.259497609 -0.406566516 0.039098975 0.042807737 0.210684772 -0.041759644 0.118416228 -0.189888304 0.343502333 -0.292401051 0.330057119 0.330845468 -0.163675271 0.219666285 -0.129081627 -0.340947146 -

0.054250883 -0.090516202 0.260586931 0.265000966 -0.311803019
0.339588585 -0.014288795 -0.0222284 0.289932074 0.102146305
0.154017764 0.459789896 0.294504104 0.005881773 -
0.355103666 -0.153791293 0.139969657 0.055853762 0.429036437
-0.06877266 0.17502097 0.284686715 -0.094078105 -
0.302814316 -0.032913346 -0.04185285 0.078958395 0.124693724
-0.062935466 -0.06358621 -0.385965447 -0.172689499 -
0.229157542 0.291886963 -0.222365112 0.293780052 -0.193273711
0.362553889 0.3831717 0.289399905 -0.326846616 0.159479431
0.269313731 0.141974344 -0.13851384 -0.268336273
0.023606524 0.289298057 -0.276739856 0.15937348 -
0.039819089 0.255785108 -0.342370204 0.367928219 0.004639492
0.051539804 -0.117782267 -0.363169893 0.078591119 -
0.030382226 0.156539498 -0.20836175 -0.364504951 0.005657918
-0.172263118 -0.271405287 0.086339267 -0.152763092
0.433426846 0.154437151 -0.090689374 -0.35485607
0.099113562 -0.235524223 0.054743481 -0.122451406
0.33720864 -0.171441657 0.055847836 0.305126081 -
0.193149115 -0.129771416 0.233389486 -0.199519164 0.077674631
0.35694636 -0.066813491 0.166421513 -0.209653005
0.080007557 0.320090883 0.088322736 0.016107946 -
0.184778004 -0.22022067 0.360791031 0.191588729 0.133959194

-0.133614873 -0.018189486 -0.245480888 -0.183046607
0.005140639 -0.060145274 -0.146730243 0.155721349 -
0.234693617 -0.073031939 -0.14173376 -0.352839985 -0.173066426
-0.095664325 0.228311553 -0.095960331 0.115241877 -
0.510191493 0.021255785 0.133383287 0.177920863 0.221908651
0.203200863 0.241401998 -0.37631875 0.192674554
0.175011434 0.212808527 -0.401975353 0.424435294 -
0.022713233 0.224458181 -0.11648644 0.152133358 0.106204774
0.074424535 0.189296729 0.021720023 0.36297754 -
0.319182212 0.016300912 -0.02916515 0.309691938 0.229238633
-0.072500874 0.23524116-0.13985593 0.025312683 0.047698684
0.255256898 -0.118350333 0.037926102 -0.029586419
0.230813512 0.211980693 -0.007787075 0.218587584 -
0.00853004 -0.189923234 0.268558846 0.182276217 0.435801328
-0.385394133 0.085997295 -0.102306975 -0.244432587 -
0.248431062 0.300426391 -0.0448506 0.088779608 0.022171618 -
0.168704552 -0.033973747 0.047485341 -0.097096098 -0.091651827
-0.313549132 -0.039869264 -0.052288755 0.006321067
0.100165789 -0.411374948 -0.257117819 0.047447471 -
0.094753914 0.177734857 0.275389451 -0.202722428 -0.281568835
0.163761706 -0.33167685 0.094304233 -0.28703131
0.184363875 -0.008133021 -0.206958418 -0.141509273

0.176899765 0.280926627 0.295342212 0.229505487
0.012574845 -0.110555992 0.167634281 0.221308428 -
0.168304223 0.225546063 -0.249565838 0.308942329 -0.203872913
0.093297694 0.09052513 0.33595298 -0.085014836 -
0.278585926 -0.360135443 -0.033318768 -0.155105255 0.330980617
-0.131942143 -0.343609974 -0.270403078 -0.408924406
0.092621899 0.242110168 0.329649914 0.153429702
0.168970155 -0.175388741 0.000351918 -0.017864209
0.245970731 0.016647937 -0.193489285 0.153135463
0.140963972 -0.061149715 -0.120781651 -0.229348006
0.480733931 0.287928859 -0.314196103 0.019276052 -
0.451940711 -0.004014322 -0.368862019 -0.205650848 0.041404035
0.118239019 -0.045982718 0.1027591 -0.029981174 0.204823035
-0.034503718 -0.091797951 0.137342019 -0.25933286
0.108539008 -0.299364364 -0.305962061 0.230576076
0.184836351 -0.000913568 0.235879933 -0.360310913
0.009374633 0.157717468 -0.178053954 -0.074051683
0.27266497 -0.082100884 -0.187375192 0.117274937 -
0.238592003 -0.181827155 -0.129401468 0.347564692 0.167150586
-0.139680978 0.241557398 -0.140903499 0.434191703 -
0.299775984 -0.497748172 0.054438667 0.132464063 -0.3410014 -
0.317381838 0.341524225 0.207792962 -0.246703231 0.078225547

0.181888288 0.235242156 -0.134787927 -0.420637699
0.370703096 0.194356797 -0.390873552 -0.194163667 -
0.415871309 -0.277849932 -0.157225025 0.35101073 0.076520012
-0.12864183 0.239879147 -0.277778203 0.114458015 -
0.270579913 0.112595853 0.557586059 -0.063943593 -0.291283419
-0.265239737 0.298182918 0.033201315 0.095874422 -
0.424441179 -0.041360187 -0.059359269 -0.148791902 0.195444841
0.076889908 -0.30019806 0.122994777 -0.027060757 -
0.176860016 0.072603106 -0.061012248 0.176557875 -0.119426034
-0.14360638 0.114053138 -0.056214139 -0.089456583
0.140660086 -0.303260439 -0.149605223 0.076824292
0.100713061 -0.068044519 -0.196164897 0.161005814 -
0.186740898 0.047874687 -0.080380463 -0.183966866 0.130273946
-0.240987363 -0.330774142 -0.25293174 -0.109400835
0.040745605 -0.001934224 -0.015157881 -0.16491098 -
0.314671115 0.224303413 -0.282859893 0.178245523 0.315417459
-0.031541183 0.054783742 0.024436821 -0.085685732
0.263830287 0.127625682 0.229352567 -0.165719748 -
0.077546271 -0.070723109 -0.213231179 0.279148538 -0.291809663
-0.269655954 0.263574919 -0.191114418 -0.224853575
0.367051939 -0.235277338 -0.239053822 -0.297174577
0.23982651 -0.212921663 -0.012198905 -0.235321701

0.261129591 -0.13770975 0.053480895 -0.081732437 -
0.197646158 -0.420174438 0.388966067 -0.208049522 -0.292096849
-0.253242049 -0.324998512 -0.370577427 -0.223666572 -
0.259238308 -0.100138824 0.390151762 -0.111309842 0.198501319
0.237830888 0.132122435 0.047250331 0.183873537
0.354193409 -0.166379805 -0.117857542 0.168102501
0.007549033 0.345784304 0.226583179 -0.103558151
0.005969703 0.278228978 -0.229540691 0.212893635
0.13173131 -0.291194281 -0.304496411 0.120235986
0.090696976 -0.090595394 -0.124625038 -0.056246901
0.008978391
KEGG_RIG_I_LIKE_RECEPTOR_SIGNALING_PATHWAY -0.101592035
0.138707861 -0.011386655 0.066061486 0.072065169 -
0.02223445 -0.085985705 0.109910344 0.037521237 0.214593129
0.276445507 -0.185372214 -0.212520881 -0.323824712 -
0.152842901 -0.245046749 0.169453765 0.034065744 -0.15043379
0.352538842 -0.001627187 0.343755892 0.198015138
0.199068888 0.091338266 -0.108987384 -0.017046682 -
0.018626607 0.055507282 -0.250264978 -0.22851597 0.203141316
0.297980693 0.218901773 -0.090125361 -0.265197924 -
0.261628324 -0.119427108 -0.065681047 0.291815142 -0.263093237
-0.106344948 0.30605373 0.184730035 0.065629159 -

0.071136611 0.217765744 -0.223036077 -0.174836752 0.051615508
-0.153545471 0.234056442 0.010337564 0.121225785
0.15810031 -0.205390156 -0.275020669 0.109391831
0.175973227 -0.146738698 0.244821146 0.225148533
0.06312983 0.297956284 -0.04418381 0.056457709 -
0.06257756 -0.14806047 -0.059151206 0.271938266 0.331047075
0.332353728 -0.199749341 -0.191628941 -0.317467887
0.056251201 0.134259036 0.109743261 -0.178193662 -
0.040871053 -0.080503614 0.110371897 0.027439278 -0.381234475
0.091276486 0.182074859 -0.023480913 -0.103564181
0.311267687 0.212247758 -0.200631136 -0.13594751
0.278038679 0.176300962 0.373279654 0.289750542 -
0.293758139 0.311782954 -0.025831308 -0.305041298 0.210742332
-0.013448659 0.054048733 -0.174482958 -0.258907295
0.036829989 -0.267632306 0.074163116 0.088866606 -
0.017350591 -0.162506678 -0.251578906 0.032597457 -0.155017233
0.102182498 0.324204813 0.015708075 -0.114263404 -
0.004355812 0.233529156 0.120900354 0.198838455 0.010858889
-0.235623822 0.311557952 -0.101536989 -0.119111767
0.056902649 0.079942683 0.30721606 -0.009139165
0.251603682 0.097279001 0.269663763 -0.345291017
0.192850138 0.239225685 -0.020730431 0.159426501

0.162965332 -0.064547346 -0.003359218 -0.042100976

0.123729741 -0.188495304 -0.388550861 0.311576155 -

0.063706718 -0.104014766 -0.015603443 0.079988167 0.042002769

0.238892555 -0.061943003 0.05811847-0.045664897 0.096182906

0.041436926 0.101268327 0.290402227 -0.228977408 -

0.022205223 0.246760334 -0.093445251 -0.115460981 -0.066643192

0.062059162 -0.037220747 -0.025276737 0.06730573 -

0.036096279 -0.097221793 -0.072453231 -0.253958064 0.077731302

-0.231437561 0.112814464 0.03019317 0.301702171

0.219705026 -0.124588844 -0.244291894 0.161305685 -

0.129310573 0.159729331 -0.075833529 0.116608433 0.077512474

0.182895381 -0.134168684 0.185701019 0.019314359

0.260043415 -0.282776064 0.288739553 -0.303005163 -

0.26824968 0.008248198 0.097065494 -0.070524705 -0.116234314

0.048538985 -0.13450092 -0.237625632 -0.370784367

0.029738945 -0.17096961 -0.099426344 0.069685605

0.374626829 0.197729201 -0.001689763 -0.111628475 -

0.065412976 -0.125124948 0.214864033 -0.094839159 0.197858333

0.080744357 0.396752583 0.089111607 0.102841861

0.018256979 0.340440642 -0.099502377 -0.066470971

0.016768514 0.035512424 0.077722239 -0.148815931

0.137786627 -0.001492105 0.303550915 -0.116383989

0.041971204 -0.083130767 0.021134056 0.079609089
0.302925788 0.047688597 0.017024894 0.172289742 -
0.002912775 -0.190630122 -0.236708351 -0.074375511 -0.181152769
0.184860775 0.307053731 -0.122815914 0.117082899 -
0.042832343 -0.163582366 0.100624026 -0.213295452 -0.081550791
-0.212746443 -0.035167643 0.293692602 0.144608189
0.259973996 0.273008982 0.187597257 -0.180564592
0.092570769 0.211012843 0.07217687 -0.274138913 0.3460475
0.044245831 -0.010554581 0.021729368 -0.079356069 -
0.124610201 -0.00748211 0.282931999 0.352545764 0.162419388
-0.091465551 -0.013495589 0.06717935 0.112266862
0.007432214 -0.030327616 0.08889114-0.125242668 0.203698298
0.407005174 -0.187344846 0.173479394 0.081407406
0.068528709 0.124204765 0.190460016 -0.108540435
0.060222046 0.088190526 0.061720214 0.24408959
0.326080276 0.281992031 -0.272127334 0.055538689 -
0.237988953 0.080538848 -0.251209022 0.161239724 0.04957432
0.045813838 0.108754763 -0.262561904 -0.025802642
0.080729588 0.262497945 -0.107709452 -0.295000319 -
0.176249136 0.032168965 0.232555417 -0.013287594 -0.199116161
-0.054792069 -0.219248257 -0.17809684 0.01733012 -
0.258405885 0.024779889 -0.037521896 0.002596313 -0.218580506

-0.26700174 0.032584311 -0.026914007 -0.073765987 -
0.210798424 0.096261573 -0.1409621 -0.061247817 0.558566894
0.200682106 0.089997635 0.169012642 0.215422439
0.119187550.206320947 -0.143489066 0.074790772 0.375546842
0.012099632 -0.258116596 -0.067423146 0.056775581 -
0.063550189 0.113734391 0.102744178 0.018302777 -0.23918793
0.023877065 0.127668271 -0.170625827 0.092896636 -
0.24378773 0.277786707 -0.125053761 -0.048352803 0.297934984
0.007076933 0.008163467 0.071313314 0.281495734 -
0.124747526 -0.018369252 -0.01997972 -0.022329824 0.261202562
-0.006675183 -0.095075796 -0.073364625 0.221951629
0.368053193 -0.316019509 -0.244019577 -0.281651212
0.243837303 -0.107293224 0.073037982 -0.03860115 -
0.010356034 0.019060425 -0.080906748 0.1986588 0.306287011 -
0.379458452 -0.054235119 -0.108236274 -0.317690999 0.241654755
-0.312377425 0.125639153 -0.050935805 0.284078398 -
0.138183025 0.244941443 -0.226707464 -0.327780844 0.262129945
-0.056920932 0.163473223 0.002560625 -0.075461706
0.168992919 0.272244666 -0.206387319 0.136351471 -
0.184714977 0.250986426 0.027259455 0.136269036 -0.066415758
-0.400358355 0.266805458 -0.048765006 -0.200273778
0.062170758 -0.0277301 -0.305245298 0.038619061 0.184383408

-0.189055384 -0.279654108 -0.109118341 0.154135367
0.082960003 0.084852296 -0.324736185 0.291382038
0.08987202 -0.234731728 -0.10792464 -0.261520946
0.106764602 -0.132904681 0.158039842 -0.116137866 -
0.173806565 0.435373029 -0.316857655 0.096557091 0.050684956
0.1272173 0.275170806 -0.129881704 -0.096163044 -0.254518815
0.248438083 -0.172424803 -0.185218597 -0.373909649
0.218473703 -0.085660103 0.220368677 0.476142276 -
0.139136668 -0.188889 0.091518542 -0.190271986 -0.019732158 -
0.148536317 -0.063675866 0.290645591 -0.176075989 -0.176126179
-0.012477185 0.168115962 -0.10001058 0.140025007 -
0.200183288 -0.329212319 0.356034852 0.020216125 0.277746186
-0.047701097 0.236838253 -0.352617947 0.028497911
0.107171183 0.097170848 -0.050675368 -0.284969406 -
0.217394923 -0.076871206 -0.005126724 0.008527476 0.411200669
-0.026870907 0.099121248 -0.319548906 0.166839838 -
0.037827536 0.109234691 -0.224046965 -0.119167008 -0.04088482
-0.021325891 -0.22699352 -0.239608907 0.066825142
0.038280976 -0.200610217 0.187007332 -0.015145825 -
0.042837167 0.138722743 -0.236539006 -0.192576228 0.174538722
0.197681776 -0.045678053 0.160335811 0.150204023 -
0.166481593 -0.052821685 0.237369872 -0.284287297 0.171285308

-0.167678396 -0.03765375 -0.095150467 0.171579485
0.129110144 -0.041051118 -0.239582347 0.128453678 -
0.075820744 -0.215772683 -0.211081305 -0.078711543 -0.2500344 -
0.072331077 -0.071247568 -0.136442974 0.131742669 -0.149333989
0.057936405 0.091225238 -0.193371551 0.168936737 -
0.260598252 0.207630718 -0.057836651 -0.020420935 0.061285873
-0.047299552 0.158139299 -0.063256164 -0.025193174 -
0.119330849 0.090145342 0.052693279 -0.154486834 -0.031033673
-0.045812611 -0.11710541 0.225187817 0.072767071 -
0.15670264 -0.104781879 0.082714959 0.232530422
KEGG_CYTOSOLIC_DNA_SENSING_PATHWAY -0.207118386
0.315572048 -0.195829306 -0.177308043 0.164488842 -
0.087261699 0.263397315 0.342739233 0.264077364 0.472320655
0.044965332 -0.271315185 0.024605052 -0.399810066
0.129056056 0.073251022 -0.016412481 0.262587651 -
0.037160968 0.514610548 -0.295931101 0.333812744 0.370998887
-0.037615097 0.414642957 -0.291682536 -0.210275707
0.056349108 0.06710776 -0.091343489 -0.29779121
0.233636684 0.320153742 0.256128646 0.265437158 -
0.176310224 -0.20518844 0.10700422 -0.304497852 0.06121437
-0.211496462 0.012236802 -0.110474977 0.175520733
0.182012254 -0.280768981 -0.1661245 -0.333605829 -0.403455136

0.061654092 -0.193088231 0.022431493 0.091194606
0.068172786 0.035838986 -0.401016159 -0.098769663 -
0.069977712 -0.044973988 -0.43217828 0.240372571 0.077960087
0.103557584 -0.05262559 -0.258952975 -0.080654098
0.20910728 -0.348234269 0.134665062 0.362889872
0.491062218 0.437099375 -0.282281692 0.170019432 -
0.341806034 0.148513339 0.122934646 -0.251892474 -0.021072286
-0.033352333 -0.184743153 0.195494726 0.266177668
0.045547328 0.395522118 0.341742448 -0.208234604 -
0.179252971 0.388478311 -0.068034139 0.109840867 -0.093647819
0.217586799 0.10621513 0.405900735 0.253234611 -
0.292762948 0.427056874 0.005968089 -0.343630658 0.403925447
0.031738144 -0.083155295 0.23326431 -0.171498527
0.036155642 -0.353989943 -0.144525839 -0.215377251
0.187357161 -0.064510437 0.140406024 -0.0197181 -0.290425776
0.049689656 0.347636894 0.210624732 -0.036128027 -
0.027026983 0.014207234 0.001088126 0.016150055 0.025792188
-0.327892188 0.061886076 -0.105962007 -0.108960848
0.013045962 0.050567167 0.399977307 0.299002858
0.309421464 -0.012861123 0.314845138 -0.046439437
0.16373221 0.110394748 -0.36712094 0.249324213 -
0.186639656 -0.216446923 -0.056652544 -0.23486771 -0.105257017

0.22937166 -0.313707177 0.3463896 0.04552896 0.083103567
0.162768117 0.213313334 0.172158003 0.402129798
0.02603078 -0.187453636 -0.124792686 -0.067071221
0.167337816 -0.326282404 0.387822933 -0.048117134
0.09851268 0.379723686 0.034719963 -0.04344786 -
0.184077457 -0.026414947 -0.210477824 0.362870469 -0.212102222
-0.088489514 -0.367664743 -0.080036351 -0.335951177
0.092094339 -0.296739562 0.100047601 -0.245416845
0.346107731 0.451296901 0.210337587 -0.271569529
0.17888143 -0.0341232 -0.059961855 -0.26544765 -0.293072436
-0.038729123 0.339635232 -0.358691461 0.155083395
0.129728653 0.112498358 -0.381683287 0.390609524 -
0.176640741 -0.039787624 -0.149810618 -0.09622337 -0.269378625
-0.116448033 0.135043984 -0.304895436 -0.160498146 -
0.036273684 0.183821833 -0.115320734 0.093373881 -0.355778255
0.444451905 0.260781453 0.093887764 -0.268944228 -
0.090283655 0.175322612 0.175649652 0.078069165 0.293760222
0.091687685 0.231868065 0.242019948 -0.026632078 -
0.076343232 0.44181886 0.070924218 -0.083276777 0.289158725
0.230764919 -0.015709116 -0.016447941 0.263508542
0.375291959 0.27772507 0.099763634 -0.022220039 -
0.312584531 0.262635975 0.02150287 0.339953949 -0.007634782

21988 0.001274361 -0.132185553 -0.04865681 -0.121271715 -
21989 0.205667967 -0.261005635 -0.008998517 -0.051231214 0.277850303
21990 0.164922524 -0.139506805 0.244685067 0.059019155
21991 0.014049449 0.048769713 0.096342667 -0.48662971
21992 0.174365161 0.142530164 0.117802705 0.389449616
21993 0.463918515 0.235238461 -0.234121543 0.134762627
21994 0.038067944 0.045525588 -0.181460283 0.405787996
21995 0.234170212 0.081340528 0.202391285 0.163325253
21996 0.088987118 0.135696995 0.493414592 -0.029215359
21997 0.362452592 -0.223905631 0.065046211 0.018523087
21998 0.273791213 0.262761348 0.093772136 0.297501631
21999 0.207566127 0.241778617 0.414264414 0.047157184 -
22000 0.029845861 -0.046340905 0.087359257 0.007274689 0.147321411
22001 0.153450248 0.303235567 -0.273521355 -0.044303325
22002 0.377478228 0.498290759 0.383970789 -0.286896069
22003 0.006199171 -0.224939965 -0.046896281 -0.329960724
22004 0.092424927 -0.165839323 0.177598024 0.321622403 -
22005 0.261385597 0.249457978 0.134221805 -0.081251392 -0.10355874
22006 -0.355903012 -0.240772704 -0.390198428 0.182058425 -
22007 0.045866803 -0.119035968 -0.314294225 -0.199555204 -0.035757433
22008 0.222051103 -0.123459829 -0.065320759 0.04642832 -
22009 0.072338095 -0.175318062 -0.255609406 -0.327198664 -0.019158667

22010 -0.228331358 -0.251339629 -0.213682742 -0.198738687

22011 0.257263562 0.497709915 0.010814183 0.273858852

22012 0.092516147 0.244725024 -0.108941888 0.044233005

22013 0.139049989 -0.047037299 0.109558088 -0.054038565

22014 0.081224886 -0.160706492 0.250086285 -0.31090499

22015 0.287000868 -0.355601993 0.189748579 -0.223189155

22016 0.166867144 0.001100395 -0.04918033 0.223326945 -

22017 0.407594502 0.277851651 0.026542095 0.156647674 0.019513366

22018 0.2741735 -0.118289705 0.151024001 -0.297957986 0.141137098

22019 -0.302813483 -0.201865238 -0.126234484 0.193216864 -

22020 0.006498929 -0.151091677 0.073418309 0.315052001 0.089107643

22021 -0.081208162 -0.135680509 -0.317055712 0.392075692 -

22022 0.164589665 -0.230821851 -0.094756292 0.177953509 0.081583942

22023 -0.110651953 0.434253455 0.351843962 -0.177485047 -

22024 0.239711468 -0.201014642 -0.405351645 0.127684151 -0.235393948

22025 -0.262541191 -0.129826369 0.28740597 -0.242107821 -

22026 0.054595416 -0.395694697 -0.108958844 0.18245248 -0.225978476

22027 -0.173072847 0.222457961 -0.010650655 -0.109309544 -

22028 0.224239397 -0.084099614 0.15190055 -0.037230391 0.32690908

22029 0.194576822 -0.112215004 -0.058318628 -0.038085654

22030 0.468819109 -0.121109541 -0.35755271 -0.166468256

22031 0.160900307 -0.222542865 0.001751325 0.266134748

22032 0.197816516 -0.430109391 0.090178639 0.020309908

22033 0.094680029 -0.061808995 -0.313699719 0.308083947

22034 0.048049834 -0.3327533 -0.012989166 -0.325732027 -0.284932009

22035 -0.234426366 0.281362112 0.046557324 -0.052283652

22036 0.178341733 -0.176211622 0.361638821 -0.140626063

22037 0.183289148 0.383821668 -0.307708372 -0.325230882 -

22038 0.034300472 0.428112617 -0.172425828 -0.052337739 -0.350531781

22039 0.07520932 -0.009664812 0.099943272 0.437150264

22040 0.139248327 -0.116762749 0.033527147 0.140374984 -

22041 0.035688153 -0.038429816 -0.369851317 0.140588698 -0.1277709 -

22042 0.250996326 -0.215975815 -0.079504946 -0.098689415 0.078873739

22043 -0.150235896 -0.342070801 0.236568477 -0.033588655

22044 0.032859292 0.112053199 0.146102782 -0.165329062

22045 0.149636478 0.012729838 -0.272174121 0.199629747 -

22046 0.199715891 -0.385469251 -0.085902302 0.124480281 0.011039595

22047 0.352945838 -0.053201091 0.219153218 -0.142359219

22048 0.377895848 -0.128361658 0.281899225 0.088761538 -

22049 0.116033665 -0.057703424 0.07316434 -0.297854364 -0.211524369

22050 0.361076949 -0.158785886 0.18688245 0.110913254 -

22051 0.118582725 -0.294241132 0.085788209 -0.170715794 -0.371559763

22052 0.250837537 0.158774575 -0.320965331 0.250604768

22053 0.200132761 -0.020836084 -0.047093269 0.182199603 -

0.232200764 0.210476139 -0.065795626 0.278948422 -0.048247532
0.106430745 0.025366442 -0.024468261 -0.407294474
0.259636668 0.007850064 -0.344855364 -0.229245214 -
0.193924616 -0.212418982 -0.261819423 -0.079663984 -0.140923781
0.283703263 -0.055626766 0.246220634 0.255386196
0.000867991 0.100429921 -0.158776903 -0.007428794 -
0.172749814 -0.039099781 -0.109873164 -0.046496164 0.355171413
0.012343174 -0.073952633 -0.164850019 0.136119276
0.132321884 0.136673484 -0.080670104 -0.079502129
0.053744473 0.146686185 -0.060228464 -0.185584121 -
0.019692941 0.142094915 0.241380495
KEGG_JAK_STAT_SIGNALING_PATHWAY -0.367356583 0.053221729
-0.177990847 0.115608462 0.062671573 0.219859076
0.123305099 0.156383216 0.044487571 0.280764096
0.254865518 -0.308605329 0.16226861 -0.413490993
0.072331438 -0.204539829 -0.004753635 0.143964467
0.286614981 0.159020157 0.01098383 0.373114782
0.054695809 -0.05850169 0.290710835 -0.057518558 -
0.264786334 0.199990503 0.189850691 0.040426054 -0.240328999
0.353653015 0.20768395 0.112434374 0.236056413
0.093792525 -0.25906026 0.079630309 0.099668346
0.075794006 -0.227709654 0.134876833 -0.06892754 -

22076 0.0498731 -0.080605693 -0.25454971 -0.134748262 -0.10067423 -

22077 0.028859959 0.23286062 -0.171987541 -0.091907422 0.121875924

22078 0.130927685 -0.166331655 -0.309016622 0.11172181-0.037904209

22079 -0.128928066 -0.219926727 -0.084221592 0.289991317

22080 0.070649446 -0.223820781 -0.084424863 0.225278413

22081 0.094537651 -0.328485392 0.347408868 0.276133904

22082 0.290296021 0.316051669 -0.145068428 0.305202721 -

22083 0.245241312 0.278711970.188189121 -0.143040208 -0.019435065 -

22084 0.29082861 0.066655718 0.36047754 0.281350201 -0.035851612

22085 0.186719673 0.163318035 -0.277856667 -0.185552765

22086 0.36875215 -0.147892366 -0.23783146 0.010623204

22087 0.082756146 -0.139891825 -0.061294963 0.315151187 -

22088 0.176536544 0.310304104 -0.132741595 -0.231546293 0.16083768

22089 0.29937974 0.135698959 0.040790757 0.181464634 -

22090 0.013247344 -0.188334892 -0.280529261 -0.032180251 0.246727578

22091 0.19391481 -0.082363025 -0.296417181 -0.274032659

22092 0.197143199 0.226745238 -0.180562356 -0.069130294

22093 0.026759971 -0.294082889 0.140849949 0.013311819 -

22094 0.258179167 -0.247136356 0.179763174 0.087618984 -0.315297583

22095 0.20732514 -0.20783320.316844628 -0.034472963 0.366475901

22096 -0.107372735 0.258028559 -0.169980684 0.314883883

22097 0.163269554 -0.214950578 -0.020635597 0.115481783

0.058018617 0.108521859 -0.209934251 -0.175855841
0.123249841 -0.363423692 0.263298937 0.147053925
0.224869394 -0.153297195 0.247550837 0.129671503
0.34052819 0.210017087 -0.004865916 -0.183511882
0.226117443 0.060768855 -0.103178006 0.296684692 -
0.089225924 0.122316942 0.214447151 0.223658837 -0.335147533
0.238430503 0.05552234 0.103257265 0.186045409
0.15656808 -0.255647355 -0.104836608 -0.094975483 -
0.090744532 0.212605067 -0.233852241 0.243944788 -0.215529343
0.02845571 0.282820063 0.07859771 -0.191079119
0.066995517 -0.136945889 -0.010035442 0.114689494 -
0.28092538 0.21956861 0.402214156 -0.050861631 -0.196083264
-0.212029184 0.23825529 -0.195396019 -0.075010646 -
0.065074676 0.118508139 -0.096922474 -0.090381169 -0.017245961
-0.036641857 0.113984981 0.031387913 -0.192913429
0.069157605 -0.035524352 0.050615044 -0.051142175 -
0.128682399 0.187276486 -0.148952325 -0.112847182 -0.09073141
-0.013225286 0.050541699 -0.121648305 -0.139421097
0.37069138 0.025859305 0.199156531 0.404889314 -
0.065956601 -0.020005794 -0.031008035 0.315035922 0.070689481
-0.028548279 0.056602307 0.041864438 0.11765879-0.170287929
0.320815475 0.233857572 0.339293247 0.151743886 -

0.12255943 0.376824743 0.073817062 0.391087043 -0.017023554
-0.149949337 -0.077235817 -0.158573535 0.080213954 -
0.384797813 0.090036106 0.111660963 -0.047732337 0.181010107
0.18593295 -0.03209674 0.021441757 0.096923541 -
0.017769453 -0.198683427 0.237884051 -0.260829288 0.096405722
0.057229951 0.033590317 0.149039577 0.131872566
0.025834284 0.022530259 -0.113540249 0.024828673
0.163689033 -0.238703704 0.162359021 0.275800144 -
0.118574803 -0.138194215 -0.075674292 0.127376192 -0.306556341
0.131965854 -0.123411223 0.231131734 -0.154272054
0.163025228 0.313802612 0.120981236 0.047644084
0.247896295 0.41440563 -0.170751429 0.356781557
0.159118520.169587136 -0.067181798 0.058384346 0.232184867
-0.151117471 0.214580276 0.343102274 0.299802474 -
0.164685757 0.081007311 0.053911835 0.341536447 0.239562612
-0.187480285 0.255552064 0.106035723 -0.357492822 -
0.038812523 0.352594223 -0.188704634 0.284850796 0.221037865
-0.1324312 -0.041587986 0.146525488 -0.09672495 -0.1732651 -
0.337998082 0.079417909 -0.269737786 -0.227342478 0.081425826
-0.236843571 -0.387590062 -0.289571403 0.209962649
0.011153441 0.103285887 -0.383883307 -0.173261957 -
0.137378112 -0.169786367 -0.125015686 -0.263760939 0.213048152

0.117406638 -0.159036441 -0.194248242 -0.05099012

0.164307819 0.294482512 0.206624924 0.019022616 -

0.021684931 0.225217846 0.067609691 -0.024016287 0.267849419

0.193808069 -0.008690935 0.084273486 -0.153520674 -

0.179728382 0.344934942 -0.258201294 -0.100403884 -0.364604364

0.156819225 0.041228578 0.06189046 -0.372910205 -

0.263965564 -0.136103238 -0.144357019 -0.102701984 0.328443956

0.198133304 0.392375158 -0.055053851 -0.151754181 -

0.094837681 -0.027916685 0.101046981 -0.093410825 -0.102239002

-0.045773538 -0.00050173 -0.066632105 0.000660687 -

0.098649158 0.464398908 0.227034629 -0.233968147 -0.030919667

-0.105233504 0.119701344 -0.209281411 -0.132705189

0.090093986 0.297149846 0.115368111 0.034627864

0.100828677 -0.048372103 0.101120953 0.226630807 -

0.188639097 -0.170661886 0.074986271 -0.38436231 0.016422171

0.16036318 0.312393293 0.172432847 -0.037415259 -

0.220451997 0.189445995 0.009274573 -0.062372723 -0.066753402

-0.010382418 0.081174303 -0.294194615 0.184192786 -

0.037131506 0.161344896 -0.117695172 0.18415413 0.100580082

-0.217178579 0.289908408 -0.178114658 0.344466565 -

0.195336245 -0.346847042 -0.057007259 0.229809939 -0.307143535

-0.101861733 0.153671749 0.003496399 -0.289240565

0.043053082 0.140357682 0.225561149 0.050241409 -
0.242586897 0.383477409 0.042628802 -0.221871667 -0.327491022
-0.366681678 -0.099719348 -0.221804125 0.164607814
0.274621104 -0.020871721 0.177543667 -0.039738201
0.370104527 -0.147460386 0.149378921 0.266411566 -
0.093211459 0.004225812 -0.053259436 0.074787974 -0.144962946
-0.168077764 -0.272917739 0.062800981 -0.114965941 -
0.087327395 0.275528003 0.187294115 -0.282971186 -0.038123563
0.085994551 0.017886437 0.129456505 -0.205795906
0.221937301 -0.033871806 -0.057963657 -0.041297956
0.145266337 -0.274167479 -0.15998621 -0.049625586 -
0.342735045 0.150087303 0.080269611 -0.214048028 -0.045604989
0.008946544 0.111317824 -0.075554948 -0.007693356 -
0.062263385 -0.254319504 -0.085377719 -0.350722309 -0.099794156
0.117883834 -0.022529809 0.148533227 -0.097166493
0.054392649 -0.239776408 0.256995303 -0.282117545
0.264563778 0.019325068 -0.204420634 -0.11472884
0.057767888 -0.088323221 -0.135451886 0.173202465 -
0.037558909 0.258064719 0.098482122 0.231011982 -0.134107428
0.365534607 -0.141146048 -0.298918578 -0.166808217 -
0.065509568 -0.211979995 0.286577135 0.003436689 -0.234241553
-0.203581795 -0.095794745 -0.248544116 -0.182582671 -

0.172841203 -0.019581926 -0.106821704 -0.070252669 0.202128046
0.038561813 -0.159322586 -0.063926964 -0.035739446 -
0.128568849 0.058419076 -0.076349532 -0.094579577 0.016259891
-0.1210757 -0.198299527 0.016641069 -0.118460081 0.19254041
0.189130677 0.115204635 -0.040521598 -0.077225773
0.192765161 -0.129778735 0.041490499 0.040220751
0.083488151 0.110804086 -0.012002621 0.127967267 -
0.265289067 0.152119653 -0.01243889 0.108208537 -0.018376094
-0.079320205 0.13104707 0.215674475 0.089892283 -
0.053894736 0.080494964 -0.012968595 -0.063161176
KEGG_HEMATOPOIETIC_CELL_LINEAGE -0.517560046 0.144459828
-0.425707658 -0.136693984 -0.073890806 0.026486115 -
0.116656193 0.20111617 0.257430417 0.429696925 0.170016944 -
0.348928922 0.410899275 -0.526424005 0.400313791 -0.094972001
-0.211431526 0.305936972 0.433063609 0.17115872-0.171552494
0.535978277 0.142105776 -0.359178044 0.441033987 -
0.12803807 -0.162209768 0.236046971 0.151341257 0.095375938
-0.461248271 0.392915583 0.203024578 0.345423253
0.484308066 0.018460842 -0.064186215 0.260038349 -
0.170298791 -0.010811674 -0.208921918 0.158448807 0.001594183
0.155419843 -0.149911388 -0.154639772 -0.150250286 -
0.243079858 -0.226189479 0.214718199 -0.330979489 -0.099826554

22208 0.214692217 -0.080314779 -0.270515612 -0.400548395

22209 0.129556567 -0.033958163 -0.221369931 -0.241149745 -

22210 0.103559567 0.41248319 -0.309545075 -0.359240336 0.06846173

22211 0.066757347 -0.077428939 -0.354501256 0.44732628

22212 0.336839038 0.269975531 0.355769375 -0.203109978

22213 0.467974285 -0.429502107 0.536947947 0.358175589 -

22214 0.494413409 0.388626613 -0.389517013 -0.017661985 0.32629477

22215 0.321441306 0.088629731 0.322738141 0.31825059 -

22216 0.460464963 -0.27141132 0.450001971 -0.125013943 -0.332316106

22217 0.123078274 -0.03456466 -0.059848655 0.005672051

22218 0.40589331 -0.212656922 0.260612595 -0.140051276 -

22219 0.262877741 0.399752614 0.410132273 -0.022578048 0.427108364

22220 0.327633361 -0.124502749 -0.260170574 -0.432481564 -

22221 0.051238955 0.462141772 0.439093467 0.077137314 -0.234133876

22222 -0.413270874 0.111567923 0.249243899 0.154981889

22223 0.163482928 0.18570563 -0.396996372 0.159867366 -

22224 0.203615733 -0.341172882 -0.280180493 0.271193254 0.250374484

22225 -0.322151692 0.31181065-0.489372858 0.201134647 0.208936032

22226 0.436423217 -0.317670563 0.24569108 0.051156296

22227 0.452217995 0.244858386 -0.435257984 -0.172979462

22228 0.010952642 0.088094305 -0.012620607 -0.442697889

22229 0.040221083 0.205689776 -0.437705454 0.393122511

22230 0.062634711 0.41791561 -0.013362802 0.449759014

22231 0.304771797 0.541248765 0.471331269 -0.0813974 0.031852493

22232 0.138664525 0.229722457 -0.196702463 0.413010002

22233 0.006968813 0.180311736 0.255172927 0.132156548 -

22234 0.392044061 0.017445893 0.27225363 0.339603279 0.502965212

22235 0.11235074 -0.4371799 -0.265435815 0.121195961 0.003626958

22236 0.288435252 -0.34562406 -0.006471922 -0.431841858

22237 0.043161548 0.420773979 0.061141531 -0.256133937 -

22238 0.026694288 -0.163073311 0.010389741 -0.019453655 -0.521801202

22239 -0.015829105 0.580199623 -0.175269035 -0.168717924

22240 0.193441379 0.041527505 -0.321108178 0.166533202 -

22241 0.036093441 0.304189726 -0.150724556 -0.175311922 -0.228231139

22242 0.124384272 0.14249273 0.091060787 0.007010583

22243 0.141330481 -0.103011168 -0.046950972 -0.305428331 -

22244 0.326741384 -0.036188667 -0.270711551 -0.117895623 -0.139320791

22245 0.22622955 0.212638674 -0.103928169 0.084094695

22246 0.326826077 -0.155789519 0.206102119 0.567016883 -

22247 0.294925862 -0.033422783 -0.085498154 0.280470209 0.222310136

22248 0.146801403 0.219594338 -0.308043907 0.415104338 -

22249 0.21020239 0.424040934 0.247954273 0.44218019 0.158078761

22250 -0.070680433 0.497822885 0.039580407 0.23482835

22251 0.184565053 -0.060357688 -0.139572089 -0.441700754 -

0.031313844 -0.550918657 0.266535009 -0.063209189 -0.135135331
0.110901487 0.219263871 -0.230360409 -0.111774559
0.191133668 0.016717958 0.102365126 0.306259042 -
0.308894988 0.357788065 -0.180559917 -0.089897533 0.177806901
0.202867341 0.185454724 0.02832908 0.198536179 -
0.153084858 0.320375149 -0.196299716 0.124696703 0.470833395
-0.069922423 -0.316593933 0.193401981 0.276479167 -
0.343180401 0.160084957 -0.27991847 0.365123562 -0.286975717
0.417517509 0.473893266 0.358481221 0.138328139
0.358005446 0.544733063 -0.022103634 0.352375994
0.208882992 0.335624911 -0.205910831 -0.390604061
0.409909032 -0.379837089 0.190453624 0.444389352
0.500548685 -0.330843468 0.005148039 0.190226887
0.347892813 0.010607832 -0.226703029 0.492961216
0.016005493 -0.330427319 -0.166806705 0.395024693 -
0.421161038 0.439751867 0.252664022 -0.106659518 -0.013133982
0.495475209 -0.425626553 -0.192701609 -0.379133604
0.294281508 -0.336730466 -0.368169766 -0.266593765 -
0.233176246 -0.543554513 -0.086551998 0.415812057 -0.030059892
0.367166546 -0.399500912 -0.316377248 0.028524001 -
0.327948418 -0.184428073 -0.497483187 0.167601821 0.272526001
-0.170917192 -0.124274776 -0.103553163 0.463661049

0.02998536 0.188822538 0.299203946 -0.35002574
0.293734447 -0.068736302 -0.326681045 0.496728842 -
0.164670526 -0.276829258 -0.042941975 -0.168627958 0.003689909
0.357420577 -0.251248171 -0.157130445 -0.51318954
0.220608186 0.105346275 0.22798656 -0.477818697 -
0.1580961 -0.35505639 -0.300808386 -0.355702327 0.491042405
0.3455753 0.406479144 -0.220211438 -0.294977005 0.012859279
-0.250373991 0.293949925 -0.400123543 -0.319926095
0.192423595 -0.108330937 -0.226038863 -0.181358686 -
0.082942786 0.571467104 0.27765384 -0.307289341 0.246749029
-0.204862426 0.213566012 -0.340861127 -0.227214226 -
0.067511896 0.403931528 0.13339203 -0.277655013 -0.018681231
0.203395863 0.079282208 0.334108884 -0.039371549
0.044158429 0.028646298 -0.330086342 -0.18149708
0.383182627 0.138741928 0.137788192 0.00899308 -
0.304581276 0.242458837 -0.106833615 -0.285697378 -0.133784244
-0.037912966 -0.23285396 -0.379287008 -0.05620574 -
0.031142237 0.371704048 0.188006223 0.210334471 -0.077242636
-0.445412096 0.460933005 -0.097114362 0.432504595 -
0.311615693 -0.547969574 0.107132734 0.395990158 -0.25525521
-0.250740832 0.436672448 0.112501124 -0.522875765
0.263582574 0.133349545 0.081504903 0.04917393 -

0.24171536 0.445100896 0.1131284 -0.447973803 -0.500434435 -
0.50599768 -0.173202673 -0.483857106 0.363152147 0.232277057
0.001947944 0.144286634 0.291170986 0.441619514 -
0.300001583 0.3894354 0.306578095 -0.273510154 -0.395052836
0.235713406 0.407564305 -0.338992411 -0.129938296 -
0.489265335 -0.322498924 -0.251298428 0.232157065 0.256438625
0.420683738 -0.107904118 0.071888981 0.109700126 -
0.354197559 0.374981732 -0.512840446 0.170990083 -0.05777972
0.094239595 -0.014415178 -0.078387686 -0.384584005 -
0.181999958 0.015176884 -0.344385822 0.327900791 0.073534633
-0.339283103 -0.106560737 -0.02266015 0.315931095
0.178563955 -0.245062463 -0.188912553 -0.125448675 -
0.321563201 -0.547132756 -0.172559062 -0.167252928 -0.041523021
0.074990764 -0.227697561 -0.201614134 -0.336173577
0.453174187 -0.364544844 0.479398644 0.26613802 -
0.388275489 -0.307161813 -0.089738438 0.124842429 0.212186822
0.475439125 0.297748676 0.404719434 -0.161441291
0.31703053 -0.271442161 0.346406648 0.085044629 -
0.346762074 0.066060814 0.157089322 -0.210902085 0.257070009
-0.135122902 -0.389613759 -0.392455538 0.134508858 -
0.148304677 -0.474471789 -0.263490944 0.020439771 -0.045156561
0.021700662 0.068254975 0.094584068 -0.32178015

0.065375079 -0.291027343 -0.290925978 -0.14609562 -
0.204616497 -0.110162684 -0.039518021 -0.052445062 0.080366762
0.121123335 -0.072845336 0.253333285 0.300252534
0.175954584 0.166497349 -0.021018859 0.233320225 -
0.307190539 0.089100323 0.038057436 -0.136493491 0.362775919
0.086607164 0.15006766 -0.307796518 0.249000529 -
0.128442353 0.355926932 -0.095046204 -0.001440658 -0.078833242
0.331759383 -0.000833708 -0.048049051 0.115762904 -
0.040747322 0.016171707
KEGG_NATURAL_KILLER_CELL_MEDIATED_CYTOTOXICITY -
0.342614469 0.393522103 -0.315386296 0.042713685 0.083873951
0.178954388 -0.03260041 0.131198525 0.192458609
0.363571975 0.282809929 -0.322511603 0.125169998 -
0.417718094 0.141399747 -0.124723387 -0.127385554 0.432840782
0.203399329 0.441438929 -0.103031664 0.374311002
0.417794589 0.117522971 0.433965818 -0.033930059 -
0.165471165 0.20281794 0.072894832 -0.189921177 -0.365230887
0.432997798 0.317617484 0.309946 0.257480731 -0.114365171
-0.194781221 0.248194944 -0.423077576 0.056202871 -
0.336412763 0.157344126 -0.037018363 0.380821606 0.065419729
-0.271735196 -0.27242519 -0.330503503 -0.33418518
0.203447741 -0.371706707 0.00161624 0.145894699 -

0.234082684 -0.10777454 -0.321918055 -0.198326613 0.343535456
0.206545284 -0.122257072 -0.041349524 0.190944599 -
0.030360038 -0.245209572 -0.185459255 0.27086761 0.086296917
-0.4466919 0.249500096 0.300368963 0.498847737 0.428299546
-0.371990383 0.030987921 -0.431057593 0.284743485
0.226635327 -0.36494686 0.063669404 -0.408377859 -
0.215442437 0.196369276 0.355005991 -0.085678055 0.4295062
0.292102771 -0.259887069 -0.236485414 0.454981313
0.044937228 -0.348854956 0.164370006 0.146861335
0.231576381 0.314522972 0.399263876 -0.172874925
0.455130597 -0.119127815 -0.31567589 0.433618958
0.029411194 0.095665274 0.257312663 0.005542174 -
0.000207646 -0.264131182 -0.326731339 0.157324226 0.352796306
0.285484029 -0.070318429 -0.389466765 -0.340682969
0.122111076 0.449746341 0.145928203 0.215939857 -
0.219934937 -0.420406532 -0.049978012 0.012454349 0.003065773
-0.410856137 0.349487504 -0.108378788 -0.301580911
0.199776775 -0.168535775 0.382931769 0.297926442
0.447692997 -0.246342785 0.450524822 0.008362468
0.125340931 0.091031642 -0.290879766 -0.022951124 -
0.092595589 -0.055222028 -0.245914875 -0.318471699 -0.363115329
0.194116799 -0.473618708 0.285234005 0.229982225

0.063384489 -0.10098967 0.374706226 0.264376683
0.462081235 0.288291686 0.183749593 -0.060915583 -
0.01803939 0.037423074 -0.299350869 0.498898007 -0.110404087
-0.137018039 0.111308975 -0.14519953 -0.326096088 -
0.090796837 0.380000973 0.149559362 0.128093037 -0.022834251
-0.343350249 -0.394551832 0.235700843 -0.180321899
0.134816335 -0.387919949 0.003021914 -0.320744734 -
0.069158562 0.432704808 0.152498691 -0.270574505 0.350691809
-0.097672148 0.201223669 -0.016170978 -0.251125202
0.190882477 0.417180127 -0.138985314 -0.23924816
0.04848259 -0.248723677 -0.349786495 0.163437583
0.112741296 -0.079237966 -0.007958745 -0.145235398 -
0.111091039 0.05772193 0.241475419 0.035770107 -0.319012634
0.062520829 0.026238803 -0.16969773 -0.147181029 -
0.216384733 -0.0682746 0.031332263 -0.2921722 -0.327904649
0.156480711 0.002527887 0.052499068 -0.128830581
0.383300676 -0.102573224 0.281271123 0.408318553 -
0.210823358 -0.037045251 0.198519418 0.323105072 0.001578064
0.088942153 0.135504326 -0.201128141 0.208758575 -
0.189946187 0.383403165 0.294246137 0.396553439 0.014984162
-0.214225124 0.41590761 0.029401190.361493419 0.022109967
-0.139686125 0.189729562 -0.363970187 0.085528146 -

0.419217074 0.257585737 0.031994169 0.047156376 0.304158454
0.048968355 -0.158898843 -0.024591567 -0.111865249
0.074663213 -0.17570844 0.238741252 -0.339346441 -
0.011867706 -0.108450134 -0.282694432 0.274994433 0.351743107
0.048916692 -0.339807426 0.17528929 -0.152824352
0.049497369 -0.343726482 0.38153879 0.379973358
0.178053887 -0.135217577 0.336678836 0.096386333 -
0.26884452 0.337176744 0.027752414 0.096869493 -0.460568453
0.063112127 0.089994667 0.275169296 0.343228097
0.150849863 0.377892333 -0.01737513 0.148279262
0.418650906 0.103072558 -0.170090599 -0.365470056
0.398262201 -0.115992635 0.283788433 0.161890631
0.296162307 -0.173096437 0.158006554 0.274624685
0.424988728 0.143584641 -0.345092215 0.088514226 -
0.248479507 -0.2627293 0.017037387 0.043292236 0.101285498
0.231289377 0.220704015 -0.170478376 -0.073733337
0.327667686 -0.105247087 0.000622344 -0.418715424 -
0.197857783 -0.097856752 0.028164929 0.28955925 -0.418247365
-0.323096572 0.004450239 0.091183863 0.07709779
0.25112341-0.175537922 -0.205823618 0.228056043 -0.309281988
-0.128563232 -0.362553475 -0.001565767 -0.055881292
0.102835894 -0.113867375 -0.101576969 0.366232563

0.457296839 0.195855868 0.310281406 -0.342944842
0.335535854 0.084807724 -0.057961704 0.343024833 -
0.11985113 -0.007327759 0.179499732 -0.197489643 -0.172846843
0.233982812 0.022726347 0.301514291 -0.307074102
0.043037761 -0.368536797 0.211080407 -0.017738168 -
0.246412026 -0.135070642 -0.350551846 0.185327872 0.303265853
0.242915641 0.32502138 -0.054785775 -0.069519386 -
0.031587817 0.084819045 0.14159622 -0.138202218 -0.066724325
0.050456689 0.295357307 -0.193585676 -0.322176022 -
0.1931509 0.413228783 0.333035362 -0.178863988 -0.091777169 -
0.078850154 0.293477533 -0.200986124 -0.056213849 0.022482071
0.410889891 -0.05883968 -0.123055664 0.396993936
0.229803294 -0.312484493 0.239896952 0.095978847 -
0.244713496 -0.07682321 -0.434881861 -0.142520956 -0.094185843
0.323476953 0.167624649 0.052251094 -0.319844732 -
0.03073668 0.087750995 -0.303524356 0.046797434 0.048882911
-0.055894726 -0.323646905 0.05601079 0.047074599
0.267360705 -0.10096081 0.280188111 0.201402391 -
0.254196908 0.107529179 -0.173750601 0.409879617 -0.251847234
-0.437744274 0.094153026 0.261098924 -0.369620839 -
0.048258785 0.388869004 0.164926157 -0.342601236 -0.104098252
0.370002939 0.083416585 -0.037288111 -0.417818839

0.23192713 0.203056192 -0.334301373 -0.472453858 -
0.406242247 0.096751101 -0.426407261 0.274372922 -0.065562
0.028222762 0.27336358 -0.012717906 0.373402719 -
0.355984247 0.06750244 0.302618111 -0.204976778 -0.293597087
0.235832613 0.270162854 -0.261648297 -0.227181052 -
0.397155474 -0.312093944 -0.288460478 0.247357004 0.471570654
0.276400219 -0.02298803 0.197566187 0.221800891 -
0.137293581 0.295864727 -0.172997043 -0.05691328 -0.259541756
-0.126286423 -0.266942813 0.049531223 -0.340066166
0.065199638 0.166711508 -0.323761766 0.314311239
0.141787006 0.059056689 -0.06069432 -0.056625766
0.03668708 0.181462532 -0.015241916 -0.155361747 -
0.291352499 -0.064732549 -0.397872625 -0.269844088 -0.015747575
0.065843806 0.374196016 -0.266487954 -0.209576827 -
0.294195654 0.391657887 -0.346219397 0.410732398 0.054639646
-0.26601693 0.158052676 0.27261985 -0.189402919 -
0.099656264 0.366024452 -0.083775219 0.268250446 -0.131666999
0.290035269 -0.312462867 0.190429565 -0.282391066 -
0.458819443 0.119286218 -0.016819049 -0.291312841 0.397616961
0.271487065 -0.346690597 -0.101759136 -0.031554013 -
0.439399865 -0.382254851 -0.201261265 0.100864645 0.227758072
0.014342667 0.154534196 -0.12992715 -0.454260802

0.212817426 0.070040374 -0.323776867 -0.290380185 -
0.257452303 -0.357740371 0.018401144 -0.252296603 -0.371845945
0.138571933 0.083826066 0.329712565 0.021026377
0.067749498 0.117427702 -0.063559211 0.165605176 -
0.328847671 0.207335709 0.08661659 -0.18537923 0.320357383
-0.103951974 -0.120679699 -0.129241344 0.219942237
0.024582527 0.225576888 -0.069473019 -0.166042969
0.01336636 0.381283044 0.136290882 -0.161931851
0.068222759 0.138159356 0.094799178
KEGG_T_CELL_RECEPTOR_SIGNALING_PATHWAY-0.284217585
0.332360745 -0.210328643 0.178439524 -0.208077599
0.179771253 -0.088869483 -0.220876621 0.172200294
0.283008239 0.210568297 -0.368824772 -0.155189768 -
0.370080496 -0.16894846 -0.240227193 -0.072578284 0.330979717
0.118062862 0.445281598 0.033694973 0.42140593
0.358437158 0.037535014 0.324696847 -0.297108298 -
0.313109043 0.101806633 0.265898831 -0.244602925 -0.343209163
0.363879493 0.282214031 0.224910278 0.130907706 -
0.020966252 -0.362150403 -0.060960243 -0.266193326 0.316364979
-0.2853368 0.116617962 0.189167247 0.267398537 0.228789104
-0.115787833 0.040662836 -0.20689744 -0.208064421
0.163137698 -0.318634381 -0.061131732 0.055001381

0.085914536 -0.098136287 -0.167899833 -0.180904343
0.104719935 0.104475908 -0.133468644 0.188069411
0.346510519 -0.02319385 -0.059515223 -0.294049547
0.169129649 0.095935157 -0.299134936 -0.012157309
0.149796876 0.441002423 0.508829881 -0.336497362
0.056922848 -0.242452809 -0.031336518 0.241924204 -
0.110825721 0.070118087 -0.38547248 -0.267521611 0.347665383
0.252715171 -0.209199947 0.260194786 0.31932809 -
0.223953235 0.023680836 0.432009488 -0.139095856 -0.395261424
-0.330979039 0.161278667 0.137411399 0.371791612
0.419928778 -0.084947717 0.412307784 -0.150795952 -
0.312884441 0.411656782 0.020735779 0.072747988 0.2617056
0.080325931 0.124422326 -0.304883171 -0.301604149
0.132396398 0.172774554 -0.198448627 -0.156906745 -
0.287734288 -0.235348052 0.285535622 0.31815049 -0.16525985
-0.069528482 -0.306891137 -0.175029857 -0.180737183
0.07511877-0.233646775 -0.386790213 0.314353476 -0.291054219
-0.453620547 0.330128153 -0.14941392 0.437975783
0.28270295 0.360180565 -0.242054336 0.453643644 -
0.079573352 0.278055207 0.277683889 -0.165128259 0.131391449
0.151420111 -0.067471401 -0.02902338 -0.034721291
0.013965497 0.149424144 -0.386348312 0.355346176

0.197143972 -0.16146927 -0.262727599 0.241078384
0.418672687 0.38455414 0.239198767 0.324315248 -
0.316089107 -0.021790615 0.205026654 0.158462902 0.30127535
-0.113280515 -0.136729171 0.186441598 -0.136104164 -
0.350143902 -0.068314027 0.239113447 0.102096554 -0.050635238
0.031115621 -0.261713994 -0.395739422 -0.068955434 -
0.26105953 0.257151056 -0.221415969 0.306371796 -0.108724634
0.080125934 0.384329993 -0.200980525 -0.128425864
0.287215691 -0.11976118 0.087296762 0.002990271 -
0.2494215 0.139812037 0.379578822 -0.189551906 -0.050582896 -
0.217874148 -0.099586531 -0.329085241 -0.122490363 -0.289782766
-0.017636117 -0.127623776 -0.34102051 0.235672394
0.032616451 0.199147949 -0.051350755 -0.419354551
0.087132265 0.000193071 -0.145420442 -0.080944089 -
0.071554331 0.006743934 0.224292456 -0.036058677 -0.029727807
0.099148683 -0.226327965 0.257156147 -0.01287525
0.456566465 -0.106380401 0.410956213 0.359689086
0.100619419 -0.282836929 0.279324293 0.10580682 -
0.091442834 0.00645438 -0.206003698 -0.018123744 0.129958492
-0.228789858 0.367401644 0.179173487 0.148652981
0.231510521 0.081166250.364844214 0.217954696 0.448394791
0.0840592 0.140128201 0.145997841 -0.289711176 0.010726848

-0.353664877 0.168437505 -0.008918661 -0.065358927
0.291479546 -0.012434439 -0.179043348 0.026558539 -
0.154382303 0.19711433-0.28283048 0.20870769 -0.22733638 -
0.199279448 0.29449005 -0.0385673 0.223057999 0.409530267
0.149153197 -0.132190544 0.037898574 -0.103253131 -
0.143197337 -0.23833812 0.442166748 0.038089902 -0.129692549
-0.293406593 0.282977719 0.057179877 -0.266634478
0.382660855 0.064913368 0.309041444 -0.316432302
0.070241728 0.125471804 0.095103773 0.23667527 -
0.221241894 0.320914043 -0.182004025 0.109941945 0.429948945
0.251392941 -0.25280381 -0.210750843 0.328354439 -
0.250991578 0.350808192 0.155193218 0.073513578 0.052243156
0.341285808 0.134347285 0.383833906 0.32524054 -
0.305926991 0.028423324 -0.265059463 -0.348175032 -0.164161901
0.303609534 0.128522237 0.206096091 0.025814329 -
0.104777136 -0.283122426 0.259766992 -0.018166607 -0.023309773
-0.29950887 -0.294645852 -0.152056735 0.065105883
0.12274545 -0.422902633 -0.254373911 -0.157663364 -
0.085376135 -0.112568412 0.092998303 -0.350849591 -0.36466504
0.162473361 -0.318301505 -0.173652755 -0.25795522 -
0.133714932 0.133782004 0.016326635 0.025522545 0.012513949
0.273641857 0.570784137 0.346295837 0.269141828 -

0.196905703 0.160464843 0.04616895 0.098832163 0.120510323
0.126830857 0.084084602 0.188480704 -0.005102893
0.051580842 0.348324746 0.104847019 0.134651571 -
0.234782419 -0.177878148 -0.243441746 0.22596619 -0.348918708
-0.403666375 -0.152294896 -0.386414721 0.104312122
0.335693928 0.132413862 0.431491817 0.024733496
0.016928214 -0.202086186 0.009390894 0.118641210.037980307
-0.10373655 0.117332805 0.160304685 -0.244270039 -
0.331684186 -0.288242632 0.39194552 0.466180656 -0.442440797
-0.179264901 -0.114242078 0.214779453 -0.232596086 -
0.052976138 0.01615454 0.377041627 0.143361456 -0.171734713
0.343652512 0.210950339 -0.313036695 0.271440133
0.038566324 0.068403907 -0.180743991 -0.373992293 -
0.124666762 0.165054467 0.308116761 0.201031108 0.083064304
-0.378642268 -0.049689355 0.317266905 -0.254367796
0.007729386 -0.121407412 0.038258926 -0.305956483
0.24893423 -0.325478585 0.08749236 -0.299306705
0.186073307 0.230401438 -0.140331053 0.348703344 -
0.311943580.458672605 -0.317924224 -0.402753717 0.145897397
0.03845332 -0.308839912 -0.369261696 0.461683798
0.14962264 -0.389273852 -0.171738141 0.423779202 -
0.093111712 -0.186916074 -0.432172347 0.292698368 0.405452857

-0.379241774 -0.389200734 -0.383452796 0.208144491 -
0.288247642 0.378892495 -0.099582797 -0.000875761 0.253752832
-0.047070751 0.16442357 -0.336300266 0.19017059
0.262483323 -0.340288823 -0.348141136 -0.244081286
0.063267209 -0.053641951 -0.098779229 -0.354917686 -
0.136441379 -0.211307602 0.0904452 0.487169325 0.099350672 -
0.22900473 0.185952611 -0.043403584 0.026986475 0.107956714
-0.115288168 0.013500364 -0.249248179 -0.19043455 -
0.072834995 0.112522121 -0.357328965 0.009914604 -0.08513706
-0.33987435 0.328467753 0.13131562 0.083408631 -
0.153966278 0.143322201 -0.16939654 -0.040409626 -0.004611135
0.023535276 -0.289619876 -0.181719711 -0.255771971 -0.343142
-0.20883365 0.083087703 0.375852774 -0.241845634 -
0.216907092 -0.488711296 0.399290217 -0.330198646 0.346623675
0.180826538 -0.022083353 -0.081060139 0.07523915
0.136409547 -0.013488338 0.269016833 0.21168969-0.108098752
0.172447369 0.106623645 -0.139244967 0.304481194 -
0.335843254 -0.317791338 0.101941371 0.16755854 -0.065956847
0.459961455 0.214269937 -0.167427363 -0.279319921
0.129279492 -0.288394616 -0.255602717 -0.238070806
0.121173077 -0.137039106 0.080539457 0.229756016 -
0.033683188 -0.453441387 0.249959774 0.122064402 -0.441190827

-0.148448719 -0.093468138 -0.246167288 -0.229022603 -
0.090642118 -0.179404118 0.14378605 0.144271953 0.269729266
0.032530505 0.022896631 0.036488184 -0.146572127 -
0.052919425 -0.235499094 0.198219261 -0.07430459 -0.130727805
0.185505923 -0.073382599 -0.045757552 0.091276439 -
0.079422309 0.034801204 -0.011631501 -0.170542175 -0.061737601
-0.028109469 0.206875419 0.080258616 -0.292135375
0.074792109 -0.005206708 0.167130232
KEGG_B_CELL_RECEPTOR_SIGNALING_PATHWAY -0.224077887
0.381935561 -0.245985581 0.251172673 -0.166131681 -0.198352
-0.313775827 -0.077212541 0.21337061 0.169259634
0.153981927 -0.308492206 -0.10573269 -0.318829808 -
0.13622677 -0.299773245 0.071904828 0.301752578 0.013828406
0.349977785 0.064018635 0.48364944 0.255795964
0.171794896 0.244186562 -0.313716914 -0.437830908
0.015323437 -0.11317783 -0.306362766 -0.302109453
0.411312538 0.328835819 0.138823838 0.347951317 -
0.097639726 -0.372772745 -0.034877792 -0.237886796 0.173177423
-0.188222511 0.290849119 0.168929657 0.280355334
0.048486934 -0.175396862 -0.099532129 -0.171057807 -
0.230630036 -0.130346813 -0.26484681 -0.021904904 0.302626835
-0.000667271 0.335077538 -0.242186382 -0.2138592 0.081465815

22604 0.086702027 0.063928544 -0.004538549 0.413871498

22605 0.17027252 0.050891648 -0.3010227 0.103396917 0.185031754

22606 -0.135805241 -0.071163569 0.01308074 0.372387815

22607 0.48309504 -0.273444869 0.047802952 -0.360786284

22608 0.110094505 0.019960151 -0.251987401 0.124184851 -

22609 0.315570628 -0.295983329 0.31132661 0.224811551 0.157714721

22610 0.067974269 0.260473606 -0.048703451 -0.08220151

22611 0.286211985 -0.176129093 -0.421493689 -0.170439431

22612 0.249177509 0.2538436 0.234858621 0.362545127 -0.157997415

22613 0.436959444 -0.036687294 -0.077669909 0.359184849 -

22614 0.108521294 -0.014022261 0.236323102 -0.150784191 -0.130940536

22615 -0.079942076 -0.225896647 0.28307291 0.063152296 -

22616 0.194031976 -0.071243303 -0.106526236 0.089214238 0.153012348

22617 0.307250068 -0.047069996 -0.354467934 -0.400414208 -

22618 0.097942774 -0.279637323 -0.17690454 -0.025672226 -0.302978722

22619 0.242517473 -0.323618856 -0.443223495 0.328116285

22620 0.002714227 0.419678778 0.203737612 0.236133556 -

22621 0.259984303 0.333685043 -0.279523316 0.36915704 0.340975921

22622 -0.209797086 0.179648 0.250298806 -0.244234925 -0.161747187

22623 0.131080058 -0.118066723 0.281132554 -0.208029372

22624 0.247873386 0.004617798 -0.04788231 -0.000547398

22625 0.234594012 0.501758888 0.480396838 0.248590878

0.074597743 -0.353817834 -0.080762103 0.043137099
0.06557473 0.350325443 -0.015600037 -0.212416079
0.051167675 -0.148725636 -0.262988177 0.039152501
0.165990014 0.21980785 -0.016535664 -0.095115438 -
0.090554154 -0.441246824 -0.106467858 -0.054308385 0.408184874
-0.023052788 0.273929289 -0.143892308 0.117068792
0.464772306 -0.223660498 -0.177387438 0.194314333 -
0.065204473 0.434369415 -0.063394149 -0.098560411 0.04759479
0.311958664 -0.116573366 -0.104915342 -0.349721831 -
0.10735918 -0.329896886 -0.062537772 -0.079310656 -0.116807731
0.033868516 -0.42114763 0.110915378 -0.116316081
0.226639695 -0.030014559 -0.402050084 0.147062067 -
0.171945274 -0.321470287 -0.127195848 -0.074923465 -0.128624221
0.174274063 0.043339948 -0.330518769 0.158213527 -
0.321251914 0.207752884 -0.037045951 0.390166317 0.033878478
0.352962145 0.410370495 0.072766272 -0.191260995 0.0396015
0.232463992 0.053608155 0.110042687 -0.240940816
0.034200365 0.064445956 -0.365679326 0.42694408
0.237395815 0.180985735 -0.127593435 0.109768412
0.269210642 0.199972226 0.335830373 -0.126210658
0.211090621 0.016722646 -0.269215935 0.151731536 -
0.40286322 0.119072571 -0.03200965 -0.196025495 0.083493265

-0.218458341 -0.302481017 -0.056370954 -0.18674327
0.093623816 -0.14266836 0.332874298 -0.215026382 -
0.075105717 0.302606318 -0.147427753 0.021265862 0.360578928
-0.057371505 -0.399154855 0.160356747 0.170526842
0.045415866 -0.333014169 0.387641407 -0.047468199 -
0.166410794 -0.218753424 0.263065394 0.053877074 -0.17437717
0.348636006 0.083101431 0.181475274 -0.401584467
0.028646406 -0.064133092 0.155497728 0.100134836 -
0.172874143 0.234575053 -0.27636066 -0.210573078 0.317416168
0.262082872 -0.261933287 -0.110786626 0.328964155 -
0.12906459 0.465684315 -0.043186491 0.047991578 0.032332408
0.212279449 0.052896088 0.285017778 0.263897971 -
0.28198618 0.001980489 -0.34674723 -0.316141978 -0.060456448
0.234356381 -0.010191728 0.201691604 -0.05217288 -
0.177294275 -0.20308098 0.302671937 0.021832217 -0.049527142
0.03178227 -0.24029149 -0.031058421 -0.154537966
0.283561042 -0.339306441 -0.119926588 0.213977242 -
0.192493977 -0.089476558 0.197799512 -0.246561395 -0.487229214
0.31525345 -0.293283288 -0.306917687 -0.256818749 -
0.112415951 0.213077435 0.05240856 0.040959386 0.218045663
0.319960325 0.475309715 0.308981128 0.240899464 -
0.181852239 -0.043032346 0.01550089 0.014861441 0.094622758

-0.142167852 -0.016924365 -0.201290596 0.129815765
0.002796155 0.339344184 0.179111194 -0.275923879 -
0.178346401 -0.239708246 -0.340919128 0.253792398 -0.316348414
-0.414708035 -0.312584111 -0.323078891 0.141682295
0.398478443 0.26586579 0.342397367 -0.101340326
0.038381442 -0.027193235 0.042073492 0.185526 0.37848343
0.092933935 0.262478441 0.170837054 -0.354811748 -
0.371640855 -0.28637686 0.411327415 0.40446206 -0.43249756
-0.121081973 -0.365150856 -0.031773271 -0.198914327 -
0.084112359 0.03201737 0.387427538 0.200173664 -0.078272329
0.288807839 0.134711113 -0.25490171 0.113232881
0.204199611 0.25612174 -0.079694653 -0.433513755 -
0.183297188 0.148406609 0.106694258 0.319247243 0.177514282
-0.31920971 0.074076502 0.10624686 -0.293631746
0.123677186 0.157840644 -0.001208249 -0.184680014
0.190871708 -0.409744068 0.16974981 -0.258915354
0.388831852 0.281590684 -0.073018978 0.371176733 -
0.147173427 0.439787252 -0.24201364 -0.232659748 0.335230911
0.121252229 -0.19583835 -0.377678118 0.538388239
0.090147329 -0.166856773 -0.15812181 0.303065666 -
0.235938489 -0.341980013 -0.379398692 0.396569907 0.420127061
-0.331161387 -0.372915411 -0.181284022 0.083844646 -

0.030314282 0.355859663 -0.210974228 -0.230050847 0.347910613
0.212571827 0.257312252 -0.122551154 0.203359103
0.22888537 -0.2956224 -0.258544957 0.057518212 0.138309784
0.157670526 0.075946711 -0.175925694 -0.21850505 -
0.114837161 0.017643596 0.375817682 0.07764283 -0.149415249
0.239517609 0.020102632 -0.099994905 0.073085353 -
0.136018663 0.074698838 -0.279090557 -0.145900246 0.147716843
0.121285079 -0.370731816 0.12539264 -0.17774655 -
0.227026887 0.29132014 0.105336136 0.104749243 -0.215914983
-0.031403157 0.041580962 -0.086558471 -0.197356566
0.079173469 -0.251706358 -0.351416983 -0.053299065 -
0.222614944 -0.224887441 0.0015138 0.202429224 -0.311791217 -
0.38188598 -0.444107299 0.320755538 -0.267965749 0.374006894
0.182508252 0.008739754 -0.012197337 0.177173187
0.29850538 0.256122824 0.149265023 0.287251025
0.148708073 -0.061197804 0.064873942 -0.310540817
0.172664824 -0.317291031 -0.336197593 0.162014942
0.239390425 -0.102234102 0.359817822 -0.198878718 -
0.226774266 -0.349076696 0.255602785 -0.223380124 -0.199443315
-0.228896313 0.284647737 -0.100976123 0.165867277
0.160738924 0.108611086 -0.401333818 0.196635715 -
0.106514559 -0.429842037 -0.040355234 -0.110794682 -0.360081503

-0.209208171 -0.17996239 -0.148642451 0.154678921
0.112462335 0.411411823 0.216719295 -0.06023518
0.006685237 -0.24849004 -0.001364738 -0.40986818
0.232724735 -0.025365936 -0.066739028 0.256343166 -
0.135096186 -0.214919549 0.179505899 -0.080520075 -0.237005898
-0.108822136 -0.15899782 0.054933529 0.152479986
0.250767356 0.113915344 -0.31363399 -0.102268933
0.229466698 0.085800933
KEGG_FC_EPSILON_RI_SIGNALING_PATHWAY -0.134607968
0.199934358 -0.088751065 0.142821656 -0.069420862
0.023793149 -0.197412803 -0.008255001 0.005590714
0.196430923 0.120736595 -0.033709471 -0.112035489 -
0.256316469 -0.01725437 -0.210821413 -0.146995309 0.235609304
0.067480872 0.217357061 0.119969685 0.342964277
0.072556073 0.127542227 0.071329281 -0.164608466 -
0.322122785 0.059534945 0.240565106 -0.021689202 -0.226978557
0.281121640.209701019 0.217633416 -0.017951478 -0.059936239
-0.25277627 -0.085860655 -0.049022453 0.21708953
0.032820598 -0.042381689 0.172472364 0.135236281
0.063539199 -0.082156287 -0.0583046 0.008612239 -0.018351959
0.03785131 -0.091645709 -0.06599206 -0.216894676
0.081438066 0.236902934 -0.06669412 -0.018506082 -

0.099515139 -0.055303569 -0.011515817 -0.280922544 0.247138737
-0.223853512 0.157192426 -0.051887329 0.213643987 -
0.152419124 -0.196529482 -0.205064644 0.102392404 0.222215425
0.216808588 -0.06873995 0.052373358 -0.192365453
0.14109372 0.186048843 -0.041308481 0.101708266 -
0.333783763 -0.153016129 0.233744182 0.036560368 -0.028561348
0.035727445 0.065507997 -0.13032697 0.125968513
0.225028393 0.108497548 -0.337528268 -0.117924344
0.118193761 0.02202834 0.276791304 0.215320262
0.087100851 0.26962913 -0.017487045 -0.116471221
0.206383966 0.016537102 -0.043457647 0.163247879 -
0.184866615 0.062787654 -0.002327491 -0.110847737 0.202484278
0.152393675 -0.070689169 -0.204299208 -0.306876609 -
0.107929427 0.190546007 0.237934712 -0.081080001 -0.127400897
-0.047412752 -0.177426763 0.011942816 0.163374462 -
0.022183706 -0.098483912 0.17431872 -0.03402767 -0.302189014
0.268560892 -0.033905713 0.200831578 -0.020079255
0.171057375 -0.157818899 0.258798523 -0.127648585
0.150189014 0.06144215 0.085731379 0.121968168
0.106995497 -0.042019995 -0.016198148 0.096291169 -
0.013095669 -0.001789944 -0.310381507 0.160043673 0.2087049 -
0.000647284 -0.092005726 0.227933659 0.168699679 0.12529752

22758 0.023361767 0.156653319 -0.268741414 0.092333963

22759 0.270152815 0.219355754 0.155801778 -0.216287026 -

22760 0.099397518 -0.042909649 -0.113585728 -0.291440323 -0.020109621

22761 0.147312718 0.212284319 9.34E-05 -0.013761087 -0.157635268

22762 -0.044610468 -0.041897398 -0.076245229 0.181503369 -

22763 0.156080047 0.174322337 -0.113018506 0.025558515 0.230342232

22764 -0.216492568 0.091758554 0.19606312 -0.223103127

22765 0.099012756 0.053033938 -0.157159013 0.006839866

22766 0.318902858 0.050129075 -0.212051147 -0.128878662 -

22767 0.009298712 -0.261945814 -0.034074894 -0.054907454 0.035961898

22768 0.17512686 -0.097023977 0.200021968 -0.168709124

22769 0.192953689 0.043320293 -0.063639542 -0.050577026 -

22770 0.036415234 -0.067104736 0.014737235 -0.0501859 0.025280382

22771 0.127077763 -0.056814363 -0.099274806 0.137908841

22772 0.031271138 0.358588165 -0.097065721 0.146088598 -

22773 0.002863732 0.21678825 0.281285485 -0.028974043 -0.093890604

22774 -0.050951132 0.10407355 0.03264496 -0.202580695 -

22775 0.12542711-0.017589977 0.233354108 -0.203976303 0.248421432

22776 0.003548271 -0.015925158 0.078709583 0.129087525

22777 0.089297974 0.120831821 0.225943515 -0.095165737

22778 0.069935804 0.175660992 -0.15800226 -0.029077486 -

22779 0.405002579 0.188579931 0.192296937 -0.224857585 0.027207034

0.027037215 0.014674527 -0.061982115 0.05693662
0.06329241 -0.120519811 0.151722056 -0.046001865 -
0.202706735 0.207679473 -0.122176463 -0.064761817 0.207094886
-0.010639253 -0.090778631 -0.041783453 0.204717743
0.000827929 -0.321162407 0.13670274 0.043701284 -
0.00479859 -0.218939393 0.152121439 -0.0484396 -0.197430162
0.248537313 0.080229669 0.064653581 -0.15502831
0.030041573 0.124217883 0.14164291 -0.066807634 -
0.121993211 0.257340562 -0.11405142 0.011007187 0.295957818
-0.075914769 -0.120678743 -0.163446463 0.237460717 -
0.101652941 0.161646643 0.094177626 0.062576986 0.049716182
0.209493874 -0.074540908 0.13465234 0.041175702 -
0.035611653 0.053178804 -0.210460582 -0.185149317 0.071767787
0.227951425 0.034632296 0.092125619 0.006558128 -
0.119629749 -0.187993637 0.089599524 0.173853555 -0.027084343
-0.119972223 -0.086949543 0.161765733 -0.164953769
0.044493887 -0.116176786 -0.038323908 -0.097933585 -
0.027145226 -0.052970924 0.03283576 -0.270414936 -0.16204
0.166704777 -0.211198146 -0.221728895 -0.189204127 -
0.14232123 0.302756378 -0.000713977 -0.015236377 0.052518766
0.036532692 0.392000766 0.182937948 -0.032365639 -
0.110728719 0.105735035 0.013694784 0.080377995 0.066933194

0.154516939 0.01001406 0.002583695 -0.050963505
0.009988749 0.277412242 0.036837992 -0.290777045 -
0.030120342 -0.12303363 -0.06421063 0.178214242 -0.179902031
-0.276283769 -0.260743518 -0.217794404 0.102907976
0.226064103 -0.064764741 0.284576966 0.081530008 -
0.074437656 -0.168725341 0.226850681 0.278380621 0.177930758
0.059843129 0.119970770.214520067 -0.182221895 -0.121279761
-0.294437913 0.181620536 0.161531649 -0.299465302 -
0.138152489 -0.213017557 -0.029978823 -0.268811732 0.069631102
-0.023204611 0.123425494 -0.189530491 0.076461009
0.073710491 0.052443113 -0.139168713 0.195726502
0.059056394 0.011092443 0.093488546 -0.324428952 -
0.002396497 0.017277511 0.143149448 0.202386351 0.136315691
0.012059193 0.029054324 0.252367638 0.08434046
0.003679235 -0.108487569 0.105539659 -0.143264226
0.356524995 -0.192373339 0.050729182 -0.059267854
0.103379598 0.249562652 -0.029029463 0.193576619 -
0.232601648 0.340333158 -0.300980187 -0.352403835 0.148477529
0.051550616 -0.062290051 -0.193003349 0.209279991
0.087007434 -0.279554089 -0.078260722 0.222744605 -
0.190967656 -0.02748088 -0.184386849 0.160739181 0.245246908
-0.0229612-0.370221717 -0.25554316 0.188736561 -0.09212981

22824 0.255542926 -0.033593849 0.024177878 0.445581531
22825 0.175239492 0.149942247 -0.213043868 0.390304038
22826 0.169587615 -0.036760919 -0.123002702 -0.083742595 -
22827 0.074291195 -0.023305425 -0.1514741 -0.247724167 -0.15644709 -
22828 0.101406811 0.066753603 0.263798725 0.037595579 -0.180184036
22829 0.01725032 -0.034626447 -0.130937923 0.144162469
22830 0.016273126 -0.018837111 -0.226711329 0.134080294
22831 0.09000576 0.235062803 -0.256176998 -0.00369627 -
22832 0.235754783 -0.267031656 0.301557896 0.146896658 0.013287817
22833 -0.079599068 0.054925999 0.115510211 0.07876032
22834 0.053860155 0.104678873 -0.178883854 -0.110296524 -
22835 0.139303355 0.082644454 -0.338563124 -0.117536462 0.185807781
22836 -0.156437211 -0.206948512 -0.332197392 0.171449964 -
22837 0.086182727 0.283410259 0.118613693 -0.1647847 -0.14033759 -
22838 0.064028429 0.116585470.145505577 0.223088504 0.164106509
22839 0.006648411 -0.045682095 0.186521596 -0.252887413
22840 0.055542592 -0.057625839 -0.154280532 0.084726778 -
22841 0.049760757 0.030966251 0.212372239 0.044262053 -0.17235344
22842 -0.30847888 -0.014344812 -0.248827299 -0.185100378 -
22843 0.258274997 0.039645522 -0.005735882 0.039791698 0.074490431
22844 0.167591864 -0.424560714 -0.023548051 0.084304787 -
22845 0.334041497 -0.048545995 0.020170756 -0.07630604 -0.256203303

-0.134396203 0.020712488 -0.017466469 0.062838768
0.253131092 0.020849219 0.008026464 -0.009232047 -
0.178818422 -0.105664941 -0.113299256 0.209652116 -0.153215384
-0.130897813 0.118351371 -0.09853782 -0.21071707
0.048891816 0.022110641 0.070882566 -0.004900486
0.171506293 0.203875763 -0.020680037 0.159397749
0.245371868 -0.152273298 -0.078805351 0.082316433
0.07839512
KEGG_FC_GAMMA_R_MEDIATED_PHAGOCYTOSIS -0.286583041
0.319648691 -0.221074914 0.161040569 -0.257382621 -
0.311612277 -0.385085667 -0.055325993 0.271539096 0.163868418
0.015231264 -0.084437051 0.092926696 -0.272137132 -
0.052163461 -0.376758693 0.142612428 0.296088634 -0.084036646
0.286982823 0.15159734 0.345538651 0.277418353
0.236425878 0.254201607 -0.327409058 -0.455061789 -
0.019553294 0.055689356 -0.186865891 -0.380526116 0.317217267
0.122818756 -0.020911376 0.370288785 0.153704335 -
0.131703598 -0.016830444 -0.104016443 0.159173306 -0.023605073
0.18354954 0.183560883 0.205662176 0.252050928
0.067352518 -0.099734897 -0.091252315 -0.061798497 -
0.295291649 -0.108883769 -0.044109728 0.054307922 0.110853307
0.189956828 0.002520188 -0.174827325 0.216907801 -

0.21706441 0.24590887 0.04738392 0.370661544 -0.045628958
0.142869132 -0.287536925 0.129307931 0.102981111
0.012636667 -0.202190723 -0.114822633 0.465733813
0.443941044 -0.178962152 0.065017233 -0.285276613
0.007329739 0.045540933 -0.185494864 -0.053920057 -
0.312866209 -0.329204616 0.264863284 0.074846322 0.103989638
0.067302594 0.265979971 -0.093112734 -0.020252681
0.236699575 -0.237102561 -0.082236735 -0.148355806
0.200446857 0.175810485 0.244256969 0.302499301 -
0.055185468 0.432609273 -0.228742334 0.057136557 0.226738759
-0.281633262 -0.112427916 0.241061823 -0.176574739 -
0.077281795 -0.208937301 -0.092164773 0.399165128 0.032219725
-0.234591095 -0.252157938 -0.26379544 0.03159573 -
0.014497912 0.318283137 -0.083560778 -0.310079807 -0.399725451
-0.042194298 -0.403044425 -0.332621901 -0.033735516 -
0.145316295 0.203224025 -0.406590474 -0.269922182 0.375258399
-0.264328983 0.398040636 0.130014808 -0.041202596 -
0.2716827 0.154577626 -0.144134022 0.315035729 0.289443918 -
0.022744517 0.23474405 0.257439146 -0.194712886 -0.369842547
-0.0505361 -0.105973387 0.263229493 -0.228002542 0.171692455
-0.11973661 -0.142816487 0.131153041 0.263616328
0.34127066 0.424146016 0.116014663 0.096276568 -

0.311767655 0.116788378 -0.007482346 -0.124248162 0.270932509 0.191292221 -0.098189104 -0.043501931 -0.247684146 - 0.276164197 -0.111304371 0.151560883 0.350219909 -0.106828402 -0.144887452 -0.160066519 -0.427425495 -0.110925714 - 0.111463008 0.385865408 0.027461668 0.067948103 -0.29417474 0.022173475 0.425793892 -0.153833568 -0.047755325 0.050990188 -0.174137332 0.332079251 -0.040542158 - 0.259554086 -0.050635715 0.326998871 0.030271999 -0.113747928 -0.316724063 -0.202329506 -0.283042098 -0.051163984 - 0.1149565 -0.078387796 0.022297924 -0.339023989 0.023218059 - 0.012543321 0.203316557 0.062712009 -0.365974456 0.11978199- 0.213749208 -0.25337288 -0.299952185 -0.149903791 -0.189206308 -0.105239427 -0.133845761 -0.124739731 0.336730387 - 0.202725855 0.265433705 0.00729335 0.245400334 -0.010942773 0.327563175 0.378502248 -0.109133444 -0.305794671 0.317523057 0.310383546 -0.059526776 0.079308583 - 0.356160494 -0.001693279 0.107340692 -0.32401896 0.411723911 -0.173978446 0.081994085 -0.162999614 0.08763026 0.138459193 0.142522551 0.302873911 -0.127328337 0.189179799 0.169300352 -0.379976147 0.140473883 - 0.205168263 0.096843448 0.059939708 -0.120190256 0.057935448 -0.286588276 -0.245369968 -0.122181585 -0.145733494

0.359982971 -0.161884984 0.301915188 -0.199496345 -
0.248193513 0.168065795 -0.184140161 0.052150949 0.235102273
-0.191101574 -0.368515133 0.194437761 0.260502181
0.038702509 -0.245931247 0.330077336 -0.191737899 -
0.196923427 0.249057344 0.321043894 0.109411618 -0.303660397
0.188997501 0.020100705 -0.234994194 -0.341883277
0.164919886 -0.05984376 0.269771606 0.141247562 -
0.197739598 0.0822271 -0.344794374 -0.195850335 0.302493322
0.216607207 -0.228621535 -0.21678964 0.235202858 -
0.230416756 0.355660515 0.069301283 0.021307085 -0.125500062
0.126993643 0.014799369 0.173500796 0.088023245 -
0.110630589 -0.224327421 -0.282906215 -0.33199851 -0.136963052
0.34940698 -0.070802805 0.353807932 -0.121637855 -
0.174136893 -0.380977917 0.28076372 0.048117589 -0.096834704
-0.052063763 -0.17459613 0.045833161 0.147409037
0.175027276 -0.221114347 -0.041070037 0.141952876 -
0.247166357 -0.003939386 0.25427455 -0.076302699 -0.445481938
0.052972586 -0.305302964 -0.03532359 -0.258666404 -
0.15388603 0.143805244 0.274431061 -0.039272996 0.091732969
0.384667601 0.363353255 0.210949123 0.170780462 -
0.345987264 -0.064964526 0.001947775 -0.065642562 0.024036073
0.063265254 -0.004612532 -0.217909692 0.068992945

0.205960312 0.379003044 0.349121209 -0.295829888 -
0.276914866 -0.366019265 -0.293006892 0.213113246 -0.32020158
-0.214797705 -0.316902767 -0.188281369 0.118744367
0.353240493 0.168564222 0.340198426 0.046449323
0.053089382 -0.055200002 0.203321671 0.33319644
0.321308435 0.13069276 0.159019838 0.209768946 -
0.319427694 -0.261456174 -0.393626963 0.396130511 0.367023567
-0.394106359 0.023597691 -0.307102472 -0.039791857 -
0.083642845 -0.145852206 -0.06753322 0.423688455 0.093471982
0.013759379 0.161869214 0.129551711 -0.204802871 -
0.011899427 0.283813477 0.200441925 -0.168996269 -0.267828072
-0.129152501 0.080381617 -0.20646043 0.215217528
0.262704417 -0.24135446 0.238956181 -0.154027763 -
0.318624062 -0.05074911 0.23351254 -0.100882798 -0.245662056
0.154511038 -0.423145727 0.254158606 -0.21938869
0.344923522 0.264959471 -0.182877069 0.374806892
0.099738593 0.343353617 -0.125338433 -0.228579347
0.331424366 0.015220515 0.076605117 -0.462439748
0.425254564 0.216679264 -0.095600348 -0.165707848
0.302930973 -0.012750714 -0.294025743 -0.252293919
0.277853561 0.345270187 -0.131407564 -0.104275 -0.174430751
-0.063783095 0.040474977 0.129458393 -0.191957595 -

0.026498297 0.318515107 0.254674412 0.077698436 -0.137271462
0.229628992 0.405768471 -0.363197566 -0.279150212
0.088801636 -0.06303733 0.11688075-0.042210567 -0.054104175
-0.072173329 0.034505987 -0.031062176 0.236617795
0.061046797 -0.13678259 0.331674941 -0.17514849
0.104503702 0.134884062 -0.077609567 -0.154957559 -
0.395768035 0.163793076 0.166157627 0.276683497 -0.275486113
0.104346339 -0.202328364 -0.321438667 0.174177553
0.216969513 0.231020873 -0.173700208 -0.154683911
0.220689407 -0.013475667 -0.123604049 0.184379409 -
0.123316582 -0.261088219 -0.077804012 -0.08525892 -0.421424326
0.052199042 0.27840337 -0.37523014 -0.435504216 -
0.321621542 0.226406109 0.052684404 0.077030026 0.256370035
-0.102920376 -0.107031865 0.156467925 0.351677651
0.220089587 0.153219971 0.283267812 -0.093919587 -
0.07745213 0.165682389 -0.317933551 0.062564491 -0.286013231
-0.204704718 0.173689815 0.000434874 0.032311611
0.233201294 0.001245887 -0.340629782 -0.318287371
0.185624052 -0.216914616 -0.275867141 0.229848882
0.317570456 0.171247782 0.258809216 0.307263072 -
0.137092895 -0.368913969 0.15976341 -0.060500135 -0.323716615
-0.063166685 -0.14158058 -0.245153437 -0.063514177

0.039442451 -0.24440293 0.221323816 0.112288181
0.358246552 0.220574275 -0.216440718 -0.001303534 -
0.125462117 -0.110012802 -0.362579444 0.069870376 -0.071131689
0.040057922 0.407379091 -0.172215921 -0.190212245 -
0.029482785 0.125386205 -0.161256764 0.218659216 -0.016586012
-0.152535431 -0.143150158 -0.045593468 0.062483667 -
0.137321714 -0.212007635 0.1202635 -0.026309998
KEGG_LEUKOCYTE_TRANSENDOTHELIAL_MIGRATION -0.26359703
0.160249515 -0.148258151 0.041793254 -0.063078399 -
0.052176405 -0.200275631 -0.083319806 -0.052003637 -0.019982345
0.022529928 -0.395175947 0.201820806 -0.235678996 -
0.055906174 -0.225855257 0.026108378 0.299085985 0.143220637
0.023250487 0.107012338 0.381625311 0.148507247
0.211335194 0.272620778 -0.203984234 -0.348476602
0.07077042 -0.203242736 -0.122600203 -0.13534576
0.06785981 0.097753778 0.289380768 0.393721942
0.027654309 0.095409649 0.128719908 -0.190228572
0.010990367 -0.215789451 0.20595863 0.053912518
0.262993468 -0.083267503 0.144706338 0.131161913 -
0.314233591 -0.202087166 -0.031328772 -0.330792737 -0.001252231
0.214217025 -0.0138169 0.279333478 -0.166897672 0.106808706
0.249828179 0.003396089 0.253689697 -0.151860881

23000 0.268790311 -0.015919006 0.001330563 -0.122118379 -

23001 0.055659831 0.226941913 0.082107543 0.196476916 0.011385479

23002 0.121000224 0.318180665 -0.322551947 0.020493372 -

23003 0.291433722 0.19873818 0.180990525 -0.342471318 0.192602437

23004 -0.298342559 -0.222242122 0.251015616 0.114797695

23005 0.174732928 0.051593488 0.032579989 -0.052068382 -

23006 0.060193596 0.143551344 -0.173655442 -0.212020018 0.267795449

23007 0.22241509 0.211296226 0.149699832 0.087000877 -

23008 0.157345471 0.096248263 0.038447295 -0.190048544 0.093615873

23009 -0.038245551 -0.119137655 0.30182236 0.056235919 -

23010 0.181543801 -0.097989731 -0.306169837 0.323195864 0.279514993

23011 0.250134512 -0.269715818 -0.09219716 0.105907539

23012 0.025023568 0.165635514 -0.077091397 -0.076729802 -

23013 0.185826256 -0.341261906 0.019119143 -0.266713043 -0.029329627

23014 -0.241902173 0.094172219 0.039726589 -0.422522629

23015 0.095937406 0.001363284 0.068113420.035694337 0.088933388

23016 -0.218231146 0.0730871 -0.238696113 0.19747355 0.245207751

23017 0.029919202 0.0059288 0.220503273 -0.170254822 -0.17718725

23018 -0.163275876 -0.022111235 0.06489208 -0.098400053

23019 0.165502728 -0.060358851 0.135937097 0.172929723

23020 0.352363273 0.255390492 0.347214117 0.39674756 -

23021 0.07581803 0.080528472 -0.182490604 -0.026815541 -0.271545171

23022 0.184352164 0.197064172 -0.119006151 -0.226758888 -
23023 0.108012211 -0.040891891 -0.065107996 0.345092701 0.288394105
23024 0.039246554 -0.024712581 -0.141637197 -0.310193484
23025 0.062908376 0.209150539 0.392076691 0.096020088 -
23026 0.053052796 -0.406306696 0.133111625 0.165571171 -0.081953125
23027 -0.183992508 0.014288058 -0.032643307 0.356684753
23028 0.026906998 -0.091944201 -0.055074864 0.322807258 0.1061067
23029 -0.170681209 0.057451397 -0.175924233 -0.257293963 -
23030 0.028210442 0.302342657 -0.220864042 0.099584787 -0.25219705
23031 -0.216767483 0.182900656 0.017623655 0.279651349 -
23032 0.236665724 0.090458672 -0.130840163 -0.276469063 -0.102756546
23033 0.060604392 -0.25049006 -0.143057796 -0.268219266 -
23034 0.298962111 0.118243357 0.038818075 0.041518917 -0.271570859
23035 0.107014091 -0.190527554 0.023358955 0.345921261
23036 0.13024025 0.134803091 -0.120959171 0.27772968
23037 0.129889912 0.152061818 -0.221634827 0.08964775
23038 0.194198784 -0.265216148 0.260023032 0.014299775
23039 0.207308727 -0.045347777 0.237740749 0.261481809
23040 0.067911791 0.071644898 0.085332182 0.002053315 -
23041 0.028518097 -0.269578959 0.26371684 -0.329718517 0.245907764
23042 -0.014925439 0.024798608 -0.128974927 -0.18094982 -
23043 0.202024585 0.062852407 0.106707206 0.163978782 0.148833425

0.255397806 -0.043726488 0.142151853 0.14312085 -
0.054601508 -0.189223244 0.086521919 -0.092949442 -0.257295364
0.189760791 0.138986658 0.273505068 -0.045897809
0.172014235 0.080052296 -0.122114391 -0.273026972
0.258618465 0.08018262 -0.252709599 0.13447999
0.163030478 0.137687495 -0.282784846 0.202205916
0.163895205 0.271335105 -0.053569721 0.264538564
0.285391677 -0.038145043 -0.05187764 -0.12646804
0.170477149 -0.076627417 -0.141827835 0.336755895 -
0.16705046 0.300800592 0.214467144 0.072092335 -0.124967802
-0.055485366 0.079698113 0.066797374 -0.127522293 -
0.361527933 0.143544853 0.059330261 -0.027265024 -0.082902849
0.152414895 0.120995019 0.210418389 -0.055956893 -
0.049345382 -0.184454688 0.36705964 0.000463041 -0.117335852
-0.344423223 0.124060812 -0.021349584 0.135690377
0.206073079 -0.285224249 -0.06436627 0.231559526
0.251959867 -0.193422946 0.275586343 -0.109977333 -
0.264382959 0.248299147 -0.064663543 -0.105731526 -0.07590987
0.034981185 0.212207053 0.237070124 0.143705768
0.158555518 0.211234154 0.063861488 0.248551193
0.05972549 -0.319454835 -0.034231195 0.134453856 -
0.096552633 -0.054293596 -0.195295792 0.064995787 0.113678547

23066 -0.180655372 0.099670537 0.138920356 0.192239331 -
23067 0.235273429 -0.191009592 0.051854762 -0.14324251 0.053418828
23068 -0.199058735 -0.279235355 -0.390305553 -0.354518226
23069 0.150845417 0.438283259 0.314693437 0.198065629 -
23070 0.246081106 0.179533351 0.095002165 0.194924189 0.081531873
23071 0.173183063 0.172943512 0.209822116 0.111405155 -
23072 0.242471649 -0.204684924 -0.230031091 0.321629 0.027523881 -
23073 0.166487671 -0.007982788 -0.160399547 -0.045824638 -0.013225035
23074 0.064261866 -0.040561186 0.377614721 0.087476486
23075 0.003928264 -0.001778334 0.045158849 -0.171856564
23076 0.200815628 0.377527249 0.187666725 0.053624037 -
23077 0.162365851 0.014215307 0.258569661 -0.185917842 0.137003106
23078 0.235572228 -0.333271684 0.079691803 -0.190629786 -
23079 0.265027281 0.246486925 -0.083619982 -0.194628339 -0.239827849
23080 0.155345597 -0.258913009 0.247987769 0.0252767 0.401868664
23081 0.138080688 -0.237623884 0.325159158 0.031021773
23082 0.057832477 -0.098118233 -0.350996276 0.250741777
23083 0.322816718 -0.033358912 -0.15395556 0.347506455
23084 0.090732042 -0.223471907 -0.062890073 0.278455215 -
23085 0.071928489 -0.172315172 -0.390328107 0.138427572 0.227297207
23086 -0.307570068 -0.295543582 -0.162700182 -0.033717572 -
23087 0.128939814 0.017392786 0.222361924 -0.064885668 0.117562011

0.129163845 0.183668737 -0.106218217 -0.020372274
0.112878693 -0.125042173 -0.221275186 0.292828725
0.041282299 -0.188988865 -0.281434562 0.091113308 -
0.132031779 -0.125373377 0.209196685 0.147227164 0.120061738
0.094042204 0.260287362 -0.07787192 -0.275655886
0.263192784 -0.004479483 -0.076481645 -0.335763909 -
0.076756484 0.071148525 0.202630848 -0.272805415 0.036123692
0.086664064 -0.048829106 0.104798189 0.172252178
0.054078832 -0.204221393 -0.203895816 0.067709769 -
0.001808513 -0.259231726 0.180896266 -0.22420705 -0.324900689
-0.016384286 -0.215420706 -0.197471299 0.105520641
0.029603754 -0.157280082 -0.368674099 -0.223435217
0.285803192 -0.236116573 0.226034978 0.141107054
0.008087368 0.1072186 0.297905144 0.343292818 0.196281621
0.277995586 0.331296541 0.212823505 -0.011264026
0.263614942 -0.237189448 0.330718158 -0.13580997 -
0.073267644 0.124660637 0.273381786 0.013480698 0.147031695
-0.211961772 -0.210869554 -0.233510862 0.104905469 -
0.303878156 -0.259142937 -0.114381387 0.1375921 -0.029289921
0.165631184 0.351635545 -0.199792984 -0.297769569 -
0.105917819 -0.217224237 -0.310367428 0.014713244 -0.096063115
-0.091416477 -0.046840269 -0.077488586 -0.079540275 -

0.006573985 -0.170606768 0.217120459 0.004055014 0.030221791
-0.014432521 -0.135417587 0.152247623 -0.388732122 -
0.085958243 0.267388625 0.09678209 0.143611179 -0.065619755
-0.132631438 0.150779495 0.200113672 -0.25338862 -
0.102076167 0.033898086 -0.136770374 0.011213460.277974837 -
0.029380476 0.007464338 0.214458095 -0.171288627 -0.132210898
KEGG_INTESTINAL_IMMUNE_NETWORK_FOR_IGA_PRODUCTION -
0.558721402 0.220053525 -0.616380079 -0.13240764 0.215118915
0.016594159 -0.227383014 0.18292312 0.418978602
0.483138868 0.288194799 -0.52158991 0.341843983 -
0.667495536 0.43907701 -0.282093575 -0.455712153 0.389710351
0.340931194 0.564447646 -0.6057933 0.516762657 0.299084391
-0.644192457 0.461714764 -0.140501232 0.144658529
0.194578037 0.264018448 0.140023604 -0.428614329
0.525209746 0.356072609 0.599868422 0.270994428 -
0.037515457 -0.363671759 0.446547886 -0.443276058 0.158867649
-0.394332997 0.231717921 -0.23952794 0.382469283 -
0.313198146 -0.273726051 -0.372793468 -0.380817488 -0.248296734
0.518139268 -0.440001353 -0.380285817 -0.146881663 -
0.52975738 -0.336520071 -0.556426528 0.065566377 -0.117061279
0.01410258 -0.449727895 0.038322953 0.487541451 -
0.215171575 -0.414387558 -0.36847644 0.025320027 0.206113905

23132 -0.680641292 0.404021263 0.577792583 0.512390906

23133 0.567637246 -0.452379056 0.575116444 -0.402597232

23134 0.560943909 0.563346204 -0.19061 0.467214467 -0.443853931

23135 -0.288358068 0.314266151 0.577670724 0.085566697

23136 0.472502048 0.347684214 -0.656264343 -0.357776184

23137 0.602204496 -0.03267876 -0.388151668 -0.093037573 -

23138 0.235640619 0.105874291 0.414240158 0.605963409 -0.431248251

23139 0.658914295 0.018815032 -0.341495145 0.523147667

23140 0.515151004 -0.02508687 0.532145032 0.444083365

23141 0.146674194 -0.336732007 -0.344321543 -0.459826647

23142 0.555084339 0.101491931 0.377932704 -0.353028991 -

23143 0.476428993 0.142635794 0.288941876 0.294193408 0.211047875

23144 0.208403836 -0.370112793 0.060745344 0.083434986 -

23145 0.297140994 -0.51792418 0.216496142 0.366591881 -0.432686665

23146 0.205563872 -0.311428898 0.594942938 0.43879138

23147 0.56281418 -0.304133283 0.408169495 0.214385115

23148 0.268328081 0.051185068 -0.508454045 -0.374946752 -

23149 0.296481495 -0.055333627 0.266210183 -0.545734659 0.076375294

23150 0.220346652 -0.589443853 0.471717361 0.330413825

23151 0.396667672 -0.026620478 0.646393246 0.292526451

23152 0.60276231 0.595545226 -0.142539301 -0.20606654

23153 0.239970625 0.446950426 -0.260089449 0.644487524 -

0.479340311 0.240677964 0.198205761 0.224506647 -0.343842784
-0.26726134 0.318134578 0.126317622 0.504998029
0.012291457 -0.508734193 -0.35332918 0.062615535 -
0.398080803 0.079221346 -0.665383492 0.19396378 -0.269422527
-0.136934635 0.56707236 0.36784941 -0.497440782
0.31942899 0.054172039 -0.43439578 -0.129042288 -
0.58989304 0.014230849 0.667555049 -0.137372086 -0.053707882
0.234996571 -0.257679463 -0.320588339 0.462814034 -
0.344312439 0.402626031 0.117969836 -0.25478387 -0.331639403
0.006745866 0.335214938 0.0660694 -0.24514052 0.329339253
0.315983678 -0.112940462 -0.111050187 -0.593727154
0.192987758 0.175827434 -0.385380636 -0.399213895
0.296000994 0.39479149 -0.181000589 0.213178331
0.454800076 -0.089819166 0.418307789 0.580901524 -
0.368238008 -0.311907996 -0.153892174 0.354280717 0.023740983
0.228725673 0.23581246 -0.585862848 0.469794698 -
0.210656359 0.518475014 0.183978602 0.45503028 0.328244321
-0.538265049 0.613367526 -0.443276188 0.549869937
0.415561356 0.011800802 -0.075930966 -0.225744713
0.012214778 -0.593769173 -0.169571778 0.084515008 -
0.190386936 0.457900342 0.400452333 -0.361068867 0.026612257
0.147874371 -0.023184773 -0.014388483 -0.101899886 -

0.408682395 0.086462906 -0.095216689 -0.345459518 0.254167198

0.526115959 0.023625968 -0.159220096 0.178709239 -

0.354589983 -0.170933021 -0.126581969 0.441971348 0.607622297

0.211267151 -0.455462407 0.388565871 0.337944507 -

0.382625483 0.552661667 -0.284212609 0.515260587 -0.433010498

0.503679541 0.418270502 0.497155788 0.386345232

0.073523297 0.464924578 0.208260179 0.388266786

0.494174728 0.282702183 -0.518155325 -0.446748911

0.469230412 -0.621540221 0.265742164 0.555352586

0.588759613 -0.449616237 0.312776524 0.144595368

0.633071699 0.365846499 -0.365576354 0.364934716

0.146578333 -0.31410768 -0.33133492 0.185124174 -

0.544393754 0.382532856 0.501761185 -0.01605336 0.070099988

0.5478781 -0.580565501 0.028253965 -0.475202924 -0.11533492

-0.408779632 -0.34107298 -0.265755785 -0.451100225 -

0.399949495 -0.340791359 0.44212808 0.282838882 0.242747986

-0.271225608 -0.099710729 -0.241082174 -0.421953155 -

0.00883745 -0.571173338 0.14062896 0.243590808 -0.490273338

-0.343131022 -0.31220255 0.154577211 0.425171735

0.218685513 0.541423323 -0.395001623 0.510800306 -

0.524602969 -0.348798214 0.536534255 -0.061828887 -0.381659348

-0.175223169 -0.204220353 -0.427466076 0.577927513 -

0.564867012 0.316114042 -0.633085274 0.440438663 -0.089255732
0.259838586 -0.193579334 -0.178331594 -0.177002182 -
0.541619603 -0.308615641 0.562181977 0.371807669 0.131534792
0.281653521 -0.562715164 0.072246016 -0.543342463
0.341179324 -0.551600397 -0.650395691 0.138231929 -
0.26770048 -0.109007712 -0.046375095 0.27719534 0.391348083
0.451874103 -0.348564867 0.299536768 0.037235519
0.464661621 -0.27529749 -0.161075615 0.025386317
0.530723604 0.292362033 -0.458985646 0.517489498
0.389997183 -0.357792649 0.262375336 -0.19341793 -
0.463304664 -0.370550833 -0.367013363 -0.532961957 -0.211026585
0.4868711 0.290110752 -0.430990409 -0.417854359 0.052911853
0.103877062 -0.311763562 -0.249715508 -0.093584563 -
0.182231073 -0.245755944 -0.253638366 0.046152815 0.34026885
0.095643038 0.165031224 0.281112255 -0.27888794
0.423864399 -0.252146237 0.618892381 -0.311245075 -
0.637826103 -0.184871728 0.377707092 -0.260487865 -0.09571554
0.486576494 -0.046371296 -0.695441925 0.20645156
0.231165559 -0.027861592 -0.017294427 -0.371084165
0.302069198 0.171143784 -0.529571282 -0.541714078 -
0.618407983 -0.334538168 -0.564150219 0.271440154 0.141443636
0.460517641 -0.001137983 0.197610225 0.497692286 -

0.50826001 0.275140517 0.2554846 -0.442881419 -0.519697327
0.267732292 0.564427794 -0.422163932 -0.214687844 -
0.516137045 -0.325985613 -0.449044606 0.248219779 0.600530174
0.613664167 -0.094163362 -0.138473412 0.447388809 -
0.236006765 0.48931675 -0.666984288 -0.138767981 -0.119844305
0.189248234 -0.283078386 -0.299613715 -0.439504104 -
0.268453435 -0.102849611 -0.292048705 0.532096551 0.041227842
-0.520512012 0.127754908 0.339276915 0.169715715
0.379602335 -0.034852955 -0.313260798 0.011380838 -
0.274899482 -0.700907029 -0.247809295 -0.373439132 -0.338849899
0.43923737 -0.402341783 -0.108525909 -0.351390648
0.587263212 -0.213844157 0.659815775 0.153326902 -
0.495263059 -0.197104687 -0.409115794 -0.302216721 -0.033733758
0.536124095 -0.090291067 0.563408444 0.064457028
0.268656709 -0.041475361 0.083472521 -0.128792204 -
0.488129139 0.135556467 -0.145800614 -0.425999853 0.266248021
0.184855028 -0.176594075 -0.383071404 0.156932542 -
0.357180113 -0.284368446 -0.328149097 0.071852215 -0.127249739
-0.2433141 -0.176164163 0.052255842 -0.488686874 0.113322295
-0.215890287 -0.475971822 0.309511479 -0.24697706 -
0.361282879 0.028287248 -0.244600793 -0.159568693 0.137167408
0.135411556 0.379998046 0.165659093 0.320642437

0.288709333 -0.008002464 -0.02971044 -0.372956955
0.327022983 0.04431641 -0.211972749 0.282937196
0.038514469 -0.022460671 -0.378450353 -0.011284451
0.133061197 0.251102101 -0.400279338 -0.093571968 -
0.060616348 0.397701206 0.044546359 -0.393891831 0.149658532
0.251442815 0.022531071
KEGG_LONG_TERM_POTENTIATION -0.057960543 -0.073517445
0.021934319 0.266823412 -0.210573454 0.115402955 -
0.149035385 -0.283706435 -0.069765741 -0.13211661 0.059061268
0.12241996 0.023011014 -0.135263212 -0.2859985 -0.085150805
0.210536412 0.123698031 0.014297157 -0.129812691
0.318020359 0.118607108 0.116208461 0.292267905 -
0.112574490.100046324 -0.119584222 0.223504816 0.053173563 -
0.072661141 -0.334305149 -0.008106015 -0.287603601 -0.322970713
0.394461885 0.094002679 0.108701163 -0.093778604
0.042858316 -0.179115119 0.198470792 -0.072463594
0.046336424 0.098214795 0.116331053 0.072861618 -
0.029143818 0.258669401 0.115054542 -0.161208163 0.062732215
0.303374864 0.060225463 0.190670284 0.006863773
0.107049073 -0.017938909 0.194167741 0.001685632
0.408769507 -0.159919746 0.22258475 0.107421445 -
0.057935245 -0.025538954 0.138970013 -0.172544024 0.209135832

-0.101927188 -0.265096765 0.061549318 0.219998881
0.091928793 0.01379585 -0.11154981 -0.195837111 -
0.177360882 -0.200125959 -0.313585595 -0.285359832 -0.147798103
0.272763976 0.032632046 0.042894635 -0.103446545
0.140168653 0.132144306 0.03546417 -0.080125628 -
0.217567827 -0.22129783 0.080001667 -0.039371891 -0.093106401
-0.088123273 0.035622937 0.318823964 0.172484675 -
0.193230471 0.034395344 0.026063393 -0.109803376 0.148770474
-0.034234342 0.060107742 0.059916287 0.386599329 -
0.116253887 0.409966567 -0.114175433 0.074948092 -0.067061315
-0.171067435 0.352930726 -0.017600197 0.109465356 -
0.278336714 -0.300269691 -0.190305828 0.018862679 -0.205416059
-0.155550319 -0.150423079 0.193276477 0.210692925 -
0.258114544 -0.021928916 0.283056518 0.069498557 0.123543787
-0.10026679 -0.175309678 -0.226949642 0.067689626 -
0.214331975 0.282349339 0.257869814 0.188203806 -0.027352439
0.384743439 0.08231842 -0.141338035 -0.004769841 -
0.17519157 0.051552043 0.131221284 0.045101736 -0.037363285
-0.189026395 -0.263944957 0.093431843 0.12003446
0.18267367 0.10884683 0.067090651 -0.165767694
0.189332067 -0.148392218 0.120881523 0.02435006
0.285989917 -0.1129069 -0.076564542 -0.091691844 -0.013019263

23286 0.164050088 0.272953584 0.254458137 -0.247568537

23287 0.10199236 0.02745302 -0.23186238 -0.086808492

23288 0.08054235 0.234407445 0.308667873 0.029784558

23289 0.020466123 0.03288208 0.124629088 -0.237691548

23290 0.323756045 -0.064640547 0.148666552 0.228397065

23291 0.204525048 -0.119004118 0.222780478 0.012887004

23292 0.131133992 0.005012662 -0.160253686 0.070687785 -

23293 0.021404544 -0.286678221 -0.077918493 -0.17223139 -0.082950257

23294 -0.14760595 0.092956687 -0.113554394 0.018860692

23295 0.327292696 -0.102527527 -0.02094731 -0.186935992 -

23296 0.027450227 -0.077546674 0.328529779 -0.370215563 0.015353009

23297 -0.182343432 0.129665967 0.056267335 -0.26103482 -

23298 0.25108191 -0.10899548 0.309155419 0.066996121 0.061825598

23299 0.216804553 0.063903681 -0.105414444 0.078761631 -

23300 0.041159965 -0.000450611 -0.13075476 -0.259013486 0.183636782

23301 0.15863651 -0.285218374 -0.011888017 -0.304913104

23302 0.100447925 0.062584003 0.413228968 0.033894035

23303 0.032016251 0.015819358 -0.205342221 -0.073826107 0.1687764

23304 -0.151288247 0.276788147 -0.29830798 0.247608154 -

23305 0.085147067 -0.090120072 -0.161722435 -0.250147884 -0.109578033

23306 0.058351395 -0.04985973 0.236317068 -0.131118694

23307 0.272520787 0.15896035 -0.073657995 0.041269935

0.020130204 -0.189102445 -0.043171625 -0.134820562 -
0.087440003 -0.012592198 0.280485748 0.053691208 0.126024954
-0.168101303 -0.233780229 -0.241409369 0.171680614 -
0.203490455 0.162664114 -0.197455266 -0.139404476 -0.048860014
-0.229423127 -0.256190726 -0.136624035 0.040151711
0.052123664 0.134832067 -0.000372514 0.004847615 -
0.300780044 -0.184977419 -0.155699691 0.066594452 -0.10050355
-0.161392091 -0.07471308 -0.002323802 0.29311283-0.107462515
-0.211092148 0.193733138 0.156635236 -0.24695044 -
0.284470486 0.10674812 -0.167349202 -0.266605742 0.068945797
-0.369977149 0.127983389 0.185505847 -0.093663105
0.219907087 -0.208098705 -0.012048428 -0.187328634
0.046734351 0.254267089 -0.02204235 -0.057879494 -
0.107907591 -0.052942038 -0.1974411 0.01524487 0.075521127 -
0.0706653 0.228191724 -0.159079956 -0.130369086 0.196704139 -
0.204585505 -0.327528376 0.195555476 -0.087023805 -0.103803119
0.061274194 -0.041861809 0.101068817 0.280828055 -
0.002574689 0.157594125 0.070103656 -0.037475687 0.177294142
-0.322999823 -0.053489362 -0.18887147 0.425841924 -
0.032976599 -0.123771361 0.0229095 -0.032309292 -0.056565711
0.107224711 0.30062572 0.043300489 0.178276776 -
0.150776677 0.115946569 -0.331287997 -0.075452015 -0.034382854

-0.209749946 0.152739614 -0.303122307 0.145190292 -
0.028261252 0.249712093 0.092206917 0.380965955 -0.227913093
0.163613614 -0.148010084 0.238363895 -0.072691666
0.411220697 0.232260268 0.176161659 -0.047639932 -
0.261595609 -0.062006268 -0.156955852 0.193516051 0.176564059
0.009677679 -0.083347063 0.04619761 -0.228300712
0.285008074 0.097395682 0.216095553 0.275201182
0.139362109 0.012615041 -0.212744348 -0.204213549
0.045766909 0.070047728 0.278517345 0.407040207 -
0.036997193 -0.287349063 0.194306169 0.115054466 -0.070066083
0.287253896 0.212663805 -0.127201318 -0.035953563 -
0.124296446 -0.075139665 0.114697356 -0.031582432 0.19264284
-0.211441607 0.173450892 -0.249445257 0.096352133 -
0.122282559 0.052375729 0.080533079 -0.076127702 0.156962901
-0.175696455 -0.067176953 0.114967463 0.06285077
0.213616969 -0.162134868 -0.219819126 -0.265562908
0.084159124 -0.00881875 0.014223214 -0.117242238
0.133153214 0.102552583 -0.283758473 -0.076501169
0.095927555 0.205150646 0.217091316 -0.005397384
0.276588778 -0.081434869 0.178912865 -0.113696602 -
0.110240634 0.074921743 -0.04671247 0.230343931 0.140890324
0.078743296 0.067651062 0.093172734 -0.020461998 -

0.085945322 -0.048611336 -0.271433412 -0.060538407 0.204199114
0.21083589 -0.208066963 0.056913937 -0.031362186
0.017513238 0.003785419 0.203849827 0.1685428 -0.027314913
0.199106377 -0.078718927 0.23131492 0.063031603 -
0.184139349 0.008426991 -0.000239879 0.241732987 -0.20531299
-0.076840912 0.173867749 -0.081612441 -0.060401637
0.292162214 0.111529053 0.02346576 -0.113988014
0.026458483 -0.257442357 -0.014812385 0.182039084 -
0.279584178 0.048386258 0.19351863 0.131153 0.28074008
0.154244217 -0.17226078 0.059717999 -0.149922816 -
0.149998572 -0.022814968 -0.168421933 -0.11028359 0.196151649
0.22357116-0.010908812 0.163337598 0.368084265 0.296813593
-0.034282211 0.349043195 -0.127980198 -0.08091428
0.072871538 -0.290872327 0.297578591 -0.116237023 -
0.002450332 0.000563212 0.172227086 -0.121151229 0.041560618
-0.097986617 -0.259783416 -0.062984394 0.166146174 -
0.313732498 -0.115040481 0.121392161 0.006759607 0.085616155
0.202151981 0.173511115 0.019880163 -0.174218806
0.026370866 0.201561932 -0.092968617 -0.04733667 -
0.008686289 -0.017528094 0.087175483 0.07703895 -0.129814473
0.251287377 0.073009589 0.178616882 -0.250018997 -
0.241985223 -0.003326923 0.025237101 -0.046139971 -0.106049375

-0.001261324 0.085136028 -0.076657999 -0.019854726 -
0.014570438 -0.24674859 0.151789007 0.057405513 -0.243387004
-0.223035849 -0.07955253 0.051165560.170199786 -0.014970725
0.049873202 -0.05450096 -0.141370985 0.048923101 -
0.002240805
KEGG_NEUROTROPHIN_SIGNALING_PATHWAY 0.17663922
0.206117810.053578234 0.325759268 -0.351924411 -0.167481747
-0.146345096 -0.186683136 -0.008596776 -0.23390848 -
0.171452105 -0.103775821 -0.141743435 -0.134530745 -0.420033086
-0.295986958 0.273621723 -0.026186401 -0.16801377
0.022998683 0.393714496 0.283417527 0.192738693
0.399632375 -0.065213873 -0.115565694 -0.414342634 -
0.06699443 -0.000118032 -0.31162657 -0.149589414 0.1460346
0.131305112 -0.240758396 0.250781124 0.13747546
0.01154185-0.10217284 -0.022057562 0.237867919 -0.107994507
0.127419361 0.288377319 0.183865064 -0.007376602
0.068590663 0.185957318 -0.08840591 -0.139198075 -
0.22750156 -0.126245874 0.195404317 0.172624121 0.188189561
0.265749608 0.138031969 -0.252772113 0.15204142
0.18319888 0.364658334 0.006496857 0.193708313
0.266075158 0.278969697 -0.056730843 0.015857252
0.06532935 0.245258812 -0.214921597 -0.33567606

0.33247584 0.325033684 -0.202058599 -0.133090909 -
0.187492297 -0.324479018 -0.149975355 0.036423538 -0.181298065
-0.152058106 -0.261553488 0.33083219 -0.035757841 -
0.125349805 -0.212879861 0.175865945 0.267823069 -0.00135879
-0.063951668 -0.133567703 -0.378763176 -0.166754572
0.303642652 0.223798145 0.222418314 0.10838766
0.004947609 0.017680244 0.031798927 -0.149574954
0.039051571 -0.361488898 0.011770137 -0.020706865 -
0.059865005 -0.095124522 0.194758574 -0.236430986 0.400928124
-0.163295202 -0.205308055 -0.272797228 0.066501507
0.236882573 0.200919542 0.182858526 -0.188621772 -
0.440738595 -0.395904436 0.02543535 -0.340209706 -0.117576398
0.050180663 -0.129308862 0.196100986 -0.373274173 -
0.343148923 0.237660264 0.221202878 0.261063699 -0.117498739
-0.298023193 -0.102543031 0.174330884 -0.07894652
0.360533748 0.362800631 0.266028585 0.085566455
0.370753359 -0.233907468 -0.240370356 0.172784866
0.044899833 0.079756217 0.008112086 0.218016783 -
0.278900187 -0.368460275 -0.193045124 -0.02553418 0.253098314
0.225388545 0.039706110.144089502 -0.223649429 -0.012900517
-0.14632323 0.10310468 0.041294328 0.195553863 -
0.150473731 0.178892437 -0.253011202 0.013574893 0.030566372

23418 0.245744295 0.219234846 -0.33434243 -0.241825238

23419 0.289242403 -0.318018706 -0.107141417 0.202655374

23420 0.366639477 0.285495677 0.20073397 0.043609902

23421 0.321216073 0.159880852 -0.192835802 -0.015020551 -

23422 0.145666569 0.07019648 0.376980327 0.120540573 0.008454194

23423 0.031234296 -0.128496741 0.178504816 -0.097537085 -

23424 0.357088383 0.147148228 -0.311070081 -0.155441189 -0.244094378

23425 -0.265256495 0.100431836 -0.239519831 0.233111117 -

23426 0.125938033 -0.041173997 0.190532929 -0.33659441 -0.00389049

23427 -0.31799206 -0.265039217 -0.002316726 0.365689143 -

23428 0.204162853 0.280196909 -0.019093173 0.101685568 0.227912053

23429 -0.326942378 0.204106268 -0.080873375 0.173593764 -

23430 0.113266058 0.211983165 0.18444723 0.105623371 -0.140591728

23431 0.194088056 -0.061965035 0.081983977 -0.079412579 -

23432 0.361456548 0.41630735 0.031431208 -0.333757506 0.04038248

23433 -0.057560543 -0.100283731 -0.211951938 0.288251862 -

23434 0.17141041 0.281582215 0.24469352 -0.261422846 0.046967547

23435 0.238034821 -0.261715858 0.326907912 -0.33353447

23436 0.17647149 -0.031833772 -0.065205228 -0.226807039 -

23437 0.312887961 -0.11964841 0.0356442 -0.27382955 0.285156833 -

23438 0.199071896 0.235038417 0.141156669 -0.114525235 0.349650491

23439 0.105244642 -0.120223848 0.147490756 -0.075859223 -

0.265504903 0.176415049 0.328353363 0.18738459 -0.153035588
0.308243394 -0.37305714 -0.23283004 0.173469957
0.098154507 0.059531613 -0.020717442 0.015377286
0.177169244 -0.061601791 -0.262470311 -0.112567487 -
0.231637121 0.004006387 0.014490881 -0.215830284 -0.125877673
-0.280222655 -0.347475999 0.054674234 0.158041002 -
0.096582476 0.094324336 -0.03131217 0.33566712 0.375317173
-0.286023125 -0.319881002 0.368904255 0.215427812 -
0.020740549 -0.228858843 0.299790851 -0.329245928 -0.305573872
-0.239302347 -0.222785435 0.00358591 0.187723597
0.301410807 0.196737929 -0.316618148 -0.126651559 -
0.257612948 -0.054050684 0.361991903 -0.09023185 -0.012466847
-0.245519046 0.271594998 0.183309737 0.318869033 -
0.28461361 0.198291921 0.250201365 -0.343145827 -0.196245165
0.119609065 -0.095763698 -0.407883849 0.299161198 -
0.070753639 -0.183371021 0.176660022 -0.22398756 0.105260142
0.420576645 0.2368786 0.407990959 0.164048455 0.389393186
0.254616875 -0.185269043 0.028102793 -0.340821167
0.353407911 0.180301427 -0.299006111 0.234279986
0.217796729 0.09068166 0.177614447 0.185252447
0.004750969 0.356459065 -0.272115211 0.28788511-0.382504644
-0.255790603 0.049235562 -0.136280626 -0.328650046 -

0.231890126 -0.176201165 0.194236894 0.229176945 -0.003309148
0.350100228 -0.097640321 0.3297981 -0.127971603 0.242408557
0.035995589 0.415764835 0.405395966 0.278399112
0.320828894 -0.267196547 -0.337751311 -0.302910135
0.262187196 0.193675875 -0.30476809 -0.296951207 -
0.345044464 -0.318682467 0.015729348 0.133480642 -0.018260489
0.209508738 0.348101299 0.144946683 -0.15651104 -
0.088340765 -0.27853374 0.064824547 0.269948172 0.323657806
0.102000569 -0.327029883 0.20029433 0.271886033 -
0.263241033 0.281352915 0.318130543 -0.15799448 -0.257616322
-0.087393343 -0.1576036 0.164175468 0.031267381 0.181047336
0.029818913 0.353110226 -0.417476776 0.018715204 -
0.285104976 0.271570796 0.113133935 -0.073629582 0.203232648
-0.039183748 0.014834996 -0.020701594 -0.050363467
0.325198503 -0.108964207 -0.097102615 -0.422585658
0.357658326 -0.110318221 0.078065235 -0.325078736
0.287363606 -0.013894037 -0.3447943 -0.277307485 0.253010081
0.445531662 -0.058919857 -0.04074075 0.21293968
0.245444032 0.252864439 -0.085808303 -0.242407781 -
0.154017076 0.25433443 -0.011184339 -0.100614039 -0.039926575
0.124202138 0.148132461 -0.11882798 -0.024623895 -
0.247735962 -0.294947587 0.109022747 0.164589972 0.03662429

0.03435044 0.095261682 0.034272582 -0.004745923 -
0.211207998 0.201893325 0.279079361 -0.169481375 0.131203764
-0.2824657 0.356350532 0.054765672 -0.289326484 -0.163547498
0.187634737 0.308862185 -0.214674915 0.187621924 -
0.003695439 -0.118115754 0.120707381 0.141674253 0.332791342
-0.181071086 0.086937917 -0.107727736 -0.258713427 -
0.046945731 0.306269381 -0.28318016 -0.191673224 0.210345641
-0.128874945 -0.10669615 0.101608422 0.106652611 -
0.024392371 -0.303257763 -0.311772492 0.089503871 -0.130622327
-0.150513198 0.131546152 0.367252323 0.163078658
0.278630649 0.311497043 0.179210257 -0.073002256
0.300130463 -0.240284923 0.074987841 0.127768448 -
0.284441788 0.2152124 -0.326623327 -0.000854824 -0.026642022
0.231842141 0.144650391 0.281381765 -0.052443407 -
0.219133627 -0.189520287 0.293876378 -0.245750144 -0.05676525
0.087456712 0.250599993 -0.060493222 0.399009499
0.299549633 -0.144195395 -0.278768674 0.166680302
0.065000185 -0.329171486 -0.153258634 -0.107974503 -
0.110340546 -0.148873133 -0.158783527 -0.11644525 0.125274278
-0.117601538 0.136841429 -0.032956598 -0.165300752
0.031580922 -0.082329075 -0.08474544 -0.208780149
0.020529977 0.006500097 0.077993505 0.059439538

0.072647663 -0.312749356 0.251256622 -0.045421855 -
0.194993701 -0.183026251 -0.00547609 -0.040302593 0.053394006
0.017342224 -0.112760342 0.045935598 -0.091126705 -
0.051373495 0.038837978
KEGG_LONG_TERM_DEPRESSION -0.173014795 0.050272753 -
0.108541583 0.207307227 -0.115320748 0.091936816 -0.036764611
-0.017584022 -0.077503465 -0.088802566 0.040067026
0.108389701 0.131677085 -0.180846283 -0.218499111 -
0.02813394 0.065692368 0.121122398 0.244520495 -0.270746045
0.295391145 0.182411345 -0.011268821 0.175253526
0.091510432 -0.007304586 -0.152946574 -0.037074029
0.114802332 -0.047236391 -0.10245751 -0.010996093 -
0.178555151 -0.18167007 0.136108971 0.005779497 0.164865263
-0.109070056 -0.040492765 -0.191356364 0.276732177 -
0.016113148 -0.016356058 -0.01873761 0.102392036 0.148053816
-0.082988967 0.308913284 0.133825087 -0.060811752
0.250952659 0.12640376 0.036088186 0.153008219
0.235682913 0.169314339 0.169967293 -0.157989561 -
0.133077131 0.264011821 -0.180763845 0.159611719 -0.01498483
-0.006585897 0.095252646 0.083419272 -0.297854973
0.057402372 0.11292482-0.075945029 -0.039418008 0.174350258
0.097431917 -0.108725084 -0.051645553 -0.080543291 -

0.060354551 -0.117295705 -0.164102303 -0.233589943 -0.212181054
0.337212572 0.149852301 0.093964389 -0.195165819
0.07083808 0.102322607 0.050617428 -0.098166709 -
0.264146933 -0.179868953 0.139232737 -0.029902131 0.029861393
-0.095783364 -0.102758261 0.330408559 0.182839229
0.055135609 0.08274106 -0.098103543 -0.006470796 -
0.01529976 -0.000784827 0.151694808 0.0575276 0.263430232 -
0.057779083 0.166160857 0.077803372 0.251508978 -0.302043038
-0.200055146 0.131019689 -0.045611111 0.016016622 -
0.263154249 -0.289621309 -0.009125517 -0.181710698 -0.064075441
-0.084028356 -0.135226078 0.174383556 -0.084392739
0.07078681 -0.046457859 0.07753304 0.022856857
0.091407602 -0.183530555 -0.246420105 -0.339074201
0.090724934 -0.087788559 0.118933752 0.139977502
0.156733067 0.104003886 0.345753002 -0.066773488
0.05187492 -0.162184355 -0.061213373 0.007739059
0.098112113 -0.040467061 0.137011225 0.124066126 -
0.024513978 0.251669541 0.035982273 0.096116674 0.197509656
0.104088373 -0.034405138 0.079510017 -0.039133521
0.219995123 0.075225737 0.063970093 -0.050275533 -
0.157670281 0.046700069 -0.101536022 0.25094988 0.038252383
0.122911927 -0.032415279 0.08807675 -0.057439018 -

0.105055761 -0.216474527 -0.032860174 0.135294776 0.102154443
0.118245563 -0.075046991 -0.03538054 0.119420831 -
0.067491647 0.32827885 -0.179093291 0.112684701 -0.016877234
0.189171081 -0.276956571 0.118466698 -0.015243295
0.076331251 0.258178299 -0.018299906 0.042271314 -
0.025694682 -0.344491659 -0.121279497 0.005744779 0.031310818
-0.122022517 0.146413511 0.262493246 -0.164365396
0.334795387 0.101134752 0.238954846 -0.158071364
0.016634571 -0.150042897 0.149438438 -0.261773617
0.000234335 -0.059697751 0.135693743 0.035065739
0.072535759 -0.096096147 -0.134696652 0.233263027
0.073444898 -0.035551284 0.119426626 0.103393309 -
0.100842477 0.010214528 -0.206497093 0.08191854 0.135070351
-0.116229304 0.073421884 0.309640473 -0.159943142 -
0.138578719 -0.129660056 0.205887614 0.102789996 0.255086517
0.054654051 0.01313507 -0.050642808 -0.12577859 -
0.004869426 -0.08364437 0.093800841 0.284861026 -0.312959418
0.20573282 -0.007424207 -0.202354633 -0.191727143 -
0.177574204 -0.033338747 0.232095952 0.114808513 -0.022295763
-0.076863606 0.278143772 0.278856141 -0.03001115
0.170847713 0.016613216 -0.225832121 -0.12243575 -
0.096422782 0.033765715 -0.111125882 0.149185026 0.067357421

-0.065857682 -0.124733526 -0.011562763 -0.273504022 -
0.136693748 -0.154577833 0.111409493 -0.302038958 0.090234611
-0.112144772 -0.024341186 -0.111532859 0.009045221
0.053281111 -0.075904695 0.123356902 0.112378266
0.008112377 -0.100967834 -0.058990756 -0.109080792
0.173944894 -0.111910488 -0.201539952 0.070908223
0.066691044 0.166355001 0.01129577-0.139692181 0.00766921
0.205000719 -0.225585244 -0.197697989 -0.014199755 -
0.156495926 -0.177428131 0.06928769 -0.296963623 0.033142715
0.264090238 -0.099911052 0.133404636 -0.095550911
0.176801471 -0.147637501 -0.028114723 0.07713724
0.08131843 -0.11658903 0.001315459 -0.099306001 -
0.236078086 -0.154195857 0.214606832 -0.216600132 -0.042995172
0.078252124 -0.125778798 0.152224789 -0.06061072 -
0.195287318 0.129538285 0.012441813 -0.071738246 0.002645214
0.127557405 0.181839955 0.116239847 -0.089776716
0.061445413 -0.108343963 -0.148503214 0.092697453 -
0.100209637 -0.074926713 -0.125688123 0.19419148 0.073250752
-0.073322223 0.193015036 -0.048149423 0.057614407 -
0.146717777 0.075517101 -0.039744734 -0.052472471 -0.284797879
0.043037758 -0.196355002 0.030959453 0.043212766 -
0.229019136 0.026911086 -0.249098946 0.121829174 -0.147437918

23594 0.296861135 -0.078383506 0.281118074 -0.183000432

23595 0.003691377 -0.081102418 0.097789333 0.010885817

23596 0.218170304 0.064438973 0.094678128 -0.031401605 -

23597 0.039016144 0.035500858 -0.047667552 0.138224877 -0.194131875

23598 -0.055238408 -0.143002737 -0.119229745 -0.139980536

23599 0.114014254 0.104996661 0.121112691 0.248010075

23600 0.033579077 0.140474382 -0.237447987 -0.174579623

23601 0.118290489 0.18464043 0.216525157 0.23931982 -

23602 0.060344272 -0.241748631 0.171625128 0.233458709 0.069554158

23603 0.138959339 0.180670864 -0.090872193 0.174302945 -

23604 0.064954198 0.006431524 0.02992259 0.042482825 -0.102767466

23605 -0.206507309 0.084147408 -0.198335176 0.076834952 -

23606 0.063396325 -0.096565832 0.187260917 -0.150936917 0.118720251

23607 -0.063912672 -0.108811013 -0.094498764 -0.060861491

23608 0.136688869 0.177862541 -0.139762947 -0.123586381

23609 0.139219054 0.113124698 -0.044465616 -0.222325491

23610 0.176918804 -0.034817182 -0.077057448 0.06007449

23611 0.082639894 0.310094316 0.266615997 -0.074353408 -

23612 0.007986191 -0.060001049 0.120533533 -0.078039003 0.093512594

23613 0.013193865 -0.046021793 0.318429935 0.029878639

23614 0.100225881 0.106549326 -0.030221084 -0.0091047 -0.036237279

23615 0.051460212 -0.231315925 -0.028295606 0.13228417

0.123457839 -0.169195648 0.070100488 0.026502958 -
0.143963867 -0.121694338 0.288118039 -0.102958814 -0.013466309
-0.136947716 0.040296248 0.071936227 -0.129614698 -
0.099529248 0.01452801 0.070653437 0.130370932 -0.064942498
-0.214981279 -0.065149086 0.025613995 -0.093459923 -
0.031912272 0.019198757 -0.097314766 -0.054014637 0.121539249
-0.100777754 0.04815937 0.036789519 -0.097898567 -
0.037537029 0.104246962 0.151056499 0.109612229 0.14177798
-0.273192695 0.048224591 -0.131890036 0.001230142
0.062557426 -0.323966408 0.051386774 0.199943623
0.001434241 -0.07731413 0.034126954 0.230190575
0.332307087 0.174425323 0.269128322 0.172529188 -
0.163919429 0.021993133 -0.332079463 0.170242266 0.17789219
-0.1289731 0.122318422 0.158009489 -0.052318997 0.030990602
-0.079894053 -0.38987259 -0.166140756 0.203885759 -
0.135574537 -0.032915193 0.059134109 -0.015264879 0.215970913
0.101981403 0.009780968 -0.034677939 -0.127878715 -
0.187697534 0.07298061 0.083840788 -0.052248621 -0.024058873
-0.218558139 0.094140814 0.104588838 -0.181642567
0.280434998 0.129869101 0.129795681 -0.060143664 -
0.060194065 0.138762429 0.174423405 -0.194160874 -0.025197733
-0.064062288 0.077910016 0.248193717 0.07384292 -

0.182394718 -0.12445455 -0.089127904 0.13418918 0.142766207
0.177517846 0.015747133 0.12313649 0.159870466 -
0.149344154 0.056891488 0.201734432 -0.225836624 0.093871847
-0.160140955
KEGG_OLFACTORY_TRANSDUCTION -0.257746529 -0.406131692 -
0.260827296 -0.297957871 0.505080416 0.156577958 -0.198607188
0.545609965 -0.343036822 0.307553728 0.452722675
0.220020116 0.275110948 -0.21253001 0.500275637
0.51275514 -0.35373118 -0.224583126 0.72799099 -
0.221390634 -0.241898636 -0.050807505 -0.154174182 -0.365589901
0.320739042 0.308986105 0.458962237 -0.195446122
0.239864557 0.588767234 0.31017669 0.147227961 -
0.30091027 0.385801342 -0.005267423 0.020192281 0.40787117
0.361631369 0.297032484 -0.528057699 0.500102704
0.10454241 -0.450772496 -0.038227302 -0.220227717 -
0.383323542 -0.471063341 0.484453562 0.368933229 0.52056998
0.38870169 0.09520251 -0.253123837 -0.151890165 -
0.446967006 -0.182649378 0.467626725 -0.292957946 -0.118521952
-0.378560593 -0.343017634 0.018706928 -0.179758965 -
0.469489643 0.349321278 -0.162425955 0.046787607 -0.406608968
0.672769531 0.524382494 -0.233809847 -0.171947159
0.454039818 -0.03849147 0.232046595 0.572654878

23660 0.43424648 -0.009985923 0.002524868 -0.231068751

23661 0.304335331 -0.196049082 -0.05197799 0.311765115

23662 0.189089548 -0.144215073 -0.42478526 -0.245523817

23663 0.377045492 -0.027761797 0.332857803 0.611951677 -

23664 0.396602837 -0.313936094 -0.412074708 -0.25552297 0.297055703

23665 0.248428329 0.216685387 0.212579841 -0.34014909

23666 0.533914953 0.038761944 -0.000789851 -0.003503068 -

23667 0.199287144 0.295997558 0.338394736 -0.418328014 0.525826929

23668 0.696263162 0.224203886 -0.278722488 -0.294944676 -

23669 0.325750559 -0.431677414 0.100010908 0.56637321 0.58078647

23670 -0.031876055 0.607978793 0.1101053 -0.360710943 0.562331192

23671 -0.264061292 0.668641336 0.517332149 -0.296620596 -

23672 0.228860508 -0.221194006 -0.360899307 0.514406002 0.116496605

23673 -0.426030054 0.044264632 -0.269613891 -0.198513644 -

23674 0.18948532 -0.300089567 -0.404292347 0.437200078 0.458864633

23675 -0.486173113 -0.176287466 -0.087863957 -0.210516008 -

23676 0.195347841 0.302110353 0.613464082 0.147697321 0.529784711

23677 -0.478524687 0.397473671 0.410143527 -0.320711185

23678 0.620848714 -0.1808272 0.37436242 -0.338880685 0.412766224

23679 -0.311310723 0.44615209 -0.186361694 0.454085508 -

23680 0.058683612 0.175025178 0.076586859 0.095110318 0.626023975

23681 0.475496769 -0.456601554 0.484994314 0.412251180.297014172

-0.476370431 -0.301628051 -0.392439381 -0.093195942 -
0.300415184 -0.070931294 0.349295549 0.25922505 0.1975148
0.033396876 -0.301296041 -0.137227871 -0.401160492
0.151310761 0.486384165 0.139109226 -0.06645522
0.438413529 0.318525406 0.171744465 -0.134464735
0.437174657 0.192583229 -0.035901004 0.362992499 -
0.270663496 0.262440517 -0.180818974 0.37893352 0.577432742
-0.082005428 0.317749287 0.267928235 0.118570961 -
0.362356222 -0.215479459 -0.334963357 -0.428340163 -0.134905888
-0.4288422 0.643602574 -0.52097246 -0.17457238 -0.051990815
0.049044434 -0.375256034 0.215186226 -0.352020027
0.490489039 -0.058266862 0.364184854 0.375604039
0.233240026 0.676626631 -0.457106251 0.533973151
0.294829293 -0.144837981 0.136196506 0.471349135
0.311403902 -0.459583035 0.495761964 -0.478453307
0.122692542 0.412455855 -0.573125023 -0.411187286 -
0.115258519 -0.237445555 -0.066214467 -0.351976526 0.218713687
0.214354292 0.262143106 0.431955135 0.517962256 -
0.252394466 0.492880879 -0.439825442 0.312094306 0.192144124
0.119705877 0.50669062 -0.43838795 0.349009361
0.211625665 -0.225513014 -0.233225551 0.507285951 -
0.314979159 -0.164229729 0.319169484 0.344337507 -0.359073892

23704 0.63670165 0.12900672 -0.151549029 -0.203798189

23705 0.197009863 -0.308750728 0.18393538 -0.321556783

23706 0.22777051 0.315467666 -0.008187026 0.516334186

23707 0.165504795 -0.123994455 0.678459151 0.518301198

23708 0.552358312 0.58942243 -0.254827284 0.033779187

23709 0.283144904 0.010576925 0.339688492 -0.382402908 -

23710 0.210272248 0.607665036 0.545456908 -0.408772189 -0.213275228

23711 -0.106688591 0.425032983 -0.292708028 0.262177812

23712 0.457139619 0.451161937 0.470902462 0.173782717 -

23713 0.052659456 -0.406310754 0.273831308 0.484844695 0.081339669

23714 0.231514466 0.160561186 -0.544947939 -0.068684081 -

23715 0.256114737 0.521129839 -0.332707022 -0.558858358 -0.515003377

23716 0.600012918 -0.339031652 -0.542110101 0.643869498 -

23717 0.040667828 0.314220031 -0.423214136 0.51309984 -0.097236852

23718 0.268123391 0.124452671 -0.161809578 0.384652663

23719 0.369238932 -0.303812482 -0.264639342 -0.503520713 -

23720 0.144235076 -0.405351722 -0.43924688 0.051738398 -0.157103283

23721 0.482827998 -0.194388244 -0.362516062 0.293266997 -

23722 0.237014947 -0.250109372 0.48018267 -0.427445844 -0.428683084

23723 0.065836061 -0.521025683 0.152293498 -0.364562522

23724 0.592726936 0.318815904 -0.325974186 -0.162711085

23725 0.552710788 0.070144213 0.536957438 -0.397580314

0.100910591 0.093032197 -0.342186033 0.23024071 -
0.436737987 0.307496729 -0.284598506 -0.386743742 -0.476829796
-0.332732869 -0.299938323 -0.52687436 0.216924616
0.469573657 0.290605612 -0.00128453 -0.429086771
0.485598288 0.358031366 0.305215631 0.406017753 -
0.331754826 0.312410519 0.322880284 -0.168084139 -0.492082801
-0.359810369 -0.096440593 -0.227948673 0.355415295
0.184018262 0.089334888 -0.580612523 0.220664892
0.225294665 0.195781728 -0.341429603 0.505103953 -
0.150986231 -0.454107082 0.244083603 0.16920453 0.230548493
0.195565319 0.002418787 0.169021749 -0.304807444
0.060590735 -0.375241712 0.45306383 -0.027576944
0.447587096 0.166208552 0.11204437-0.089590505 0.037109787
0.075867019 -0.001071188 -0.007725867 -0.054646317 -
0.47173244 0.42173046 -0.127575566 0.621128616 -0.522383501
0.184262315 -0.321962899 0.473538093 -0.122508164
0.461656755 0.550332026 0.475447837 -0.421295817 -
0.446231141 0.424133425 -0.005743015 -0.258424848 -0.241934266
-0.438501825 -0.199675275 0.574990968 0.044045607 -
0.32070862 0.519478682 0.49585618 0.298406818 0.181739742
-0.10905471 0.404834136 0.070334639 0.530444063
0.121291522 -0.22138947 -0.294588342 0.310659806 -

0.196195935 -0.345100223 -0.111407686 0.045140923 0.516742926
0.014030664 -0.52101139 0.213291526 -0.373863902
0.43841446 -0.575190619 0.185171108 0.236540938
0.357403267 -0.328040745 -0.28973538 0.344875057 -
0.406202526 0.258619868 0.173080085 -0.064871905 -0.240052167
-0.523171619 0.409469624 -0.178303912 0.581856135
0.174879898 -0.20469266 -0.324209379 0.099113151 -
0.169693332 -0.348781263 0.5962839 0.666430956 -0.310253387 -
0.343217595 -0.007907377 0.443075656 0.409939945 0.284174724
0.137347043 0.302271367 -0.166809945 -0.341223288
0.276378249 -0.068963445 -0.348910499 -0.267118684
0.088565276 -0.372042245 0.58611432-0.311949351 0.038346971
0.323792939 -0.07363198 0.544358731 -0.34844843 -
0.053196093 -0.264128244 -0.486009385 -0.172970681 -0.005657789
0.052804704 0.294231442 -0.397660532 0.029856434 -
0.300836557 0.137432128 -0.258816761 0.273181075 -0.333164028
-0.342790259 0.319934288 0.230421389 -0.495644498 -
0.071691327 0.136864516 0.290509918 0.188595066 -0.378123084
0.321922110.30847491 -0.260982468 -0.356804434 0.263181879
-0.030938098 0.094323651 0.107066981 -0.005348747 -
0.341268234 0.174517534 -0.196703771 0.15857458 -0.146071101
-0.244131699 -0.12179748 -0.013692671 0.255357083 -

0.667935837 0.33615305 -0.233481125 0.312801241 -0.467057454
0.185501348 -0.066713413 0.189020184 0.387878608 -
0.301784972 0.088970993 0.444582509 -0.128519006
KEGG_REGULATION_OF_ACTIN_CYTOSKELETON -0.234486925
0.098382011 -0.119416683 0.028744025 -0.262002926 -
0.139751883 -0.108914935 -0.076913514 0.121238906 -0.116280996
-0.098700489 -0.094397859 0.141020291 -0.070103124 -
0.161532713 -0.234546935 0.032020884 0.216551932 0.133264206
-0.165140197 0.168600561 0.172306239 0.114861769
0.233043498 0.157928991 -0.193739319 -0.293370519 -
0.014716369 -0.050543403 -0.093404473 -0.123154692 -0.032625726
-0.133018487 -0.085381723 0.391279196 0.10399135
0.108060827 0.026754451 -0.066270471 -0.201924772 -
0.05244627 0.173638707 -0.005252884 0.064264148 0.041662649
0.246563124 -0.117127154 -0.112885269 -0.066670032 -
0.068573359 -0.169847953 -0.006354027 0.134694461 0.050118371
0.26833174 0.094348623 -0.006180253 0.210431974 -
0.055641897 0.282743245 0.020619378 0.246381987 -0.027670098
0.088144689 -0.199441012 0.085238486 0.153879329
0.149064813 0.073399571 -0.207338729 0.235988482
0.282226978 -0.051437035 0.012142936 -0.17083904 -
0.086243902 -0.024969771 -0.211373663 0.050829415 -0.287827836

23792 -0.272211943 0.290201429 0.029699721 0.229621868

23793 0.123392574 0.162777196 0.02038021 0.097448513

23794 0.086326635 -0.255029239 -0.063432277 0.092897701

23795 0.038498395 0.055781234 0.042408899 0.059899132 -

23796 0.042657341 0.147382886 -0.084669616 0.082112016 0.031261392

23797 -0.166101027 -0.103743221 0.092115369 0.177149373 -

23798 0.016054682 0.043717552 -0.213024827 0.3244385 0.077990098

23799 0.049969208 -0.14210919 0.011512813 0.143920768 -

23800 0.062602153 0.063975308 -0.21757941 -0.229887521 -0.229068769

23801 -0.173582238 -0.179778751 -0.255449333 -0.178972201 -

23802 0.110563594 -0.073508546 -0.127946861 -0.195775147 0.252068941

23803 0.050732922 0.101647384 0.001910857 -0.058529793 -

23804 0.274115387 -0.031863425 -0.067202128 0.285093967 0.258337208

23805 0.084216169 0.084115035 0.166516396 -0.086842335 -

23806 0.277173687 -0.137858651 -0.173348159 0.105528765 0.068985302

23807 0.073051245 -0.14741897 0.063408267 0.234161187

23808 0.279548058 0.342145856 0.293193642 0.324672223 -

23809 0.157114621 0.036544388 0.102855288 -0.195984808 -0.157241491

23810 0.111223126 0.2373417 0.036227738 -0.073569989 -0.19820689

23811 0.008755178 -0.087511226 0.230398901 0.158613101 -

23812 0.041624554 -0.056094894 -0.044548049 -0.230830359 -0.151865407

23813 0.031161207 0.292258206 0.220413847 -0.055046087 -

0.288355559 -0.014245321 0.196838811 -0.117619121 -0.051023708
-0.20660773 0.095757549 0.280658807 0.152099347 -
0.064927698 0.034684048 0.24661562 0.099351782 -0.056608139
-0.161394158 -0.081221121 -0.230683727 -0.230898759
0.111349207 -0.161628661 0.057298027 -0.246802863 -
0.116816256 0.176657844 -0.002545342 0.183772259 -0.144417564
0.141410448 -0.135080255 -0.080681093 -0.150846838
0.130465578 -0.223502581 -0.098174446 -0.168200642 -
0.003706001 0.223724659 -0.084037283 -0.060614721 -0.114906681
0.130906712 -0.168923658 0.008912969 0.284770008
0.053269319 -0.080545857 0.139124455 -0.015713037
0.002952457 0.13310878 -0.124495705 0.147524024
0.286442951 -0.321350269 0.09244728 -0.287025605
0.123875541 -0.117451818 0.241921082 0.194349479
0.056208592 0.088142748 -0.103985143 0.007960431 -
0.084794766 -0.267727816 0.241276285 -0.092161452 0.216965147
-0.009759352 -0.063861044 -0.192260985 -0.161020086 -
0.047409928 0.095951685 -0.022634676 0.329829657 -0.018420119
0.353501774 0.085436413 -0.089641196 0.029426066
0.003189997 -0.236208404 0.059458634 -0.198913942 -
0.110549062 0.103213712 0.115687976 0.1544314 0.033567283 -
0.015864089 -0.077183607 -0.25797198 0.111698298 0.090425219

0.094933417 -0.340488497 -0.058358829 -0.023697964 -
0.141291211 -0.164320845 0.029600937 0.125813149 0.258650708
-0.034158611 0.086841864 0.13279098 -0.247865873 -
0.11416155-0.138361746 0.310704793 -0.138731899 -0.188836124
0.248824545 -0.086875393 0.325220728 0.116200239 -
0.091099481 0.003122415 -0.027890258 -0.052440023 -0.109005908
-0.213904453 -0.25469455 -0.105158948 -0.061594249 -
0.169671665 0.138999575 0.234552704 0.049629186 0.267160132
-0.217440898 0.016066097 -0.174245713 0.260564867
0.05057568 0.04124806 -0.086001331 0.050047679
0.028214236 -0.093875355 0.150238443 -0.099137478 -
0.004246511 0.154538878 0.083901188 -0.145044006 0.277623534
-0.163893021 -0.25910528 0.169599478 0.061802302
0.044354955 -0.099081937 -0.002391203 0.128303051
0.284929143 0.040644311 0.045787683 0.290586804 -
0.02433892 0.103244745 -0.145109325 -0.289132711 -0.221373051
0.081231887 0.027075574 -0.174581686 0.006735992 -
0.005808815 0.146708829 -0.114937602 0.240089359 0.192255011
0.156591205 -0.309964065 -0.075153079 -0.175096698 -
0.080486154 -0.053565456 -0.334490649 -0.184012436 -0.234643187
-0.064014549 0.086715386 0.372528344 -0.011323621
0.23675868 -0.071798211 0.164168062 -0.175980923

0.112598077 0.23027305 0.273851817 0.212559105

0.21640904 -0.061300568 -0.220610719 -0.148708484 -

0.269791229 0.182965513 0.103729468 -0.249611218 -0.033265819

-0.164636432 -0.139728648 0.094973317 -0.029127473

0.038295465 0.371476024 0.061634874 0.071328745 -

0.087236399 -0.046084297 0.093195417 0.255443425 0.32747949

0.206343787 -0.131919725 -0.141171913 0.037617838

0.101468584 -0.160450231 0.191082235 0.20172473 -

0.210156449 0.12402177 -0.137292558 -0.140228259 0.115367727

0.18803395 -0.153492588 -0.273059141 0.165418992 -

0.314441405 0.126188598 -0.005713031 0.083345016 0.156468499

-0.217438438 0.356688834 0.057795743 0.022668652 -

0.042442822 -0.160737889 0.29658283 0.127766281 -0.078718509

-0.261425169 0.382940296 0.073000424 0.028303007 -

0.213989122 0.116338114 0.229735381 -0.176112268 -0.153721338

0.118438289 0.252783503 -0.09042217 0.057986027

0.02167659 0.022145796 0.096066933 -0.114124634

0.132214933 -0.022693079 -0.079313319 0.18549258

0.007034128 -0.056351446 0.095420917 0.104909927 -

0.131693151 -0.224801421 0.094403187 -0.156275047 -0.001016255

0.047084249 0.204639512 -0.107857317 0.114613066

0.021863856 0.010157992 0.019583185 0.267182965

0.043703989 -0.033474624 -0.130261216 0.174195449 -
0.010422498 -0.130141773 -0.260787878 -0.046380869 0.196381291
0.286463577 -0.141871018 -0.047390455 0.002112266 -
0.121238083 -0.089600013 0.179456485 -0.000587029 -0.083894889
-0.268629704 -0.001416959 -0.071642327 -0.215096064
0.11028177-0.029034321 -0.109747507 0.109338599 -0.070805499
0.074727257 0.132309579 -0.023201259 -0.052578855 -
0.339235225 -0.065183206 0.192912175 -0.144531534 -0.029168064
0.118551385 -0.032193802 0.063119122 0.267134208
0.371234992 0.29927243 0.127830361 0.307989367
0.152159538 -0.126756344 0.067791875 -0.320215105
0.156281912 -0.156178793 -0.167667616 0.15492082
0.121711205 -0.015524249 -0.054552583 -0.177031297 -
0.259944358 -0.139588688 0.180925743 -0.134790191 -0.110602389
0.138791493 0.243822261 0.080859792 0.210222126
0.268113249 -0.15300097 -0.160812246 0.065925509 -
0.160768394 -0.094015151 -0.106061531 -0.080624478 -0.079612247
0.154930321 0.016080492 -0.173551698 0.214878436 -
0.024949777 0.188382889 -0.179723936 -0.189102717 0.068337149
0.132530256 0.048620545 -0.239092971 0.007686817 -
0.065937554 0.13532039 0.104042844 -0.055841257 -0.197656961
0.00230967 0.195381595 -0.17414175 0.112231757

0.14465692 -0.152055553 0.1416274 0.073557342 -0.091318699
0.006381718 0.023580701 0.015652627 -0.126023746
KEGG_INSULIN_SIGNALING_PATHWAY 0.221818996 0.246335127
0.181424537 0.171592544 -0.241266955 0.0482928 0.02259228
-0.06282157 -0.073088913 -0.207394239 -0.201152695
0.011004366 -0.237530307 -0.10521997 -0.34090825 -
0.133561719 0.215713749 0.110930455 -0.164542289 -0.163702991
0.302217679 0.21507307 -0.028634824 0.247495963 -
0.219114474 -0.049949932 -0.241422832 0.026653747 0.091995423
-0.254073047 -0.063501923 0.049102011 0.194821103 -
0.190907343 -0.053669018 0.10919455 -0.225455451 -0.048227176
0.073587923 0.255006347 0.02507175 0.22878344
0.197832785 0.015123564 0.005491916 0.108041074
0.021454978 0.009752222 0.114556307 -0.2109559 0.073600848
-0.076589593 0.070093472 0.200218547 0.245912969
0.174099281 -0.181323967 0.172134065 0.19413035
0.272188687 -0.291462618 -0.048583664 0.268441464
0.256904376 -0.076642447 -0.009013377 0.031826232
0.100541847 -0.138720029 -0.335227533 0.008348172 -
0.050933287 0.015899931 -0.148754548 0.221054732 -0.168672942
-0.211471023 0.147623698 -0.163034212 -0.112415251 -
0.017253651 0.142296136 -0.03430835 -0.082875409 -0.179091262

0.018727451 0.076195595 0.059873155 -0.16083159 -
0.006129679 -0.304016435 -0.217636782 0.072103663 0.197972506
0.102926217 0.120217261 0.050095942 -0.10202279
0.03759887 0.11053792-0.081067819 -0.287455885 0.011136984
0.024989856 -0.064673945 -0.013689801 0.325660735 -
0.040525887 0.208258881 -0.193817172 -0.153563015 -0.070384718
-0.030991017 0.079743405 0.051475456 0.119142452 -
0.226113359 -0.34784306 -0.265982161 -0.008931947 -0.15422874
0.152319268 -0.001074708 -0.010302171 0.069875416 -
0.240600131 -0.128789383 0.013910533 0.17046038 0.150199949
-0.164509634 -0.197807101 -0.007784903 0.121443408 -
0.095849511 0.102815939 -0.118668243 0.298213079 0.140324794
0.25070822 -0.086406685 0.028607938 0.139747661 -
0.124774509 0.002801508 -0.115748243 0.18047779 0.01586918
-0.219384629 -0.177431322 -0.04507175 0.067923401 -
0.131062009 -0.156749578 0.069277779 -0.085422163 -0.051649519
-0.031290488 0.192599735 -0.173893235 0.207921321
0.027743253 0.195516819 -0.194396753 -0.085085281
0.184422959 0.080163857 0.197934346 -0.2746988-0.21472264
0.114254064 -0.014651187 -0.116748034 0.06743649
0.244911189 0.067115818 0.098693714 0.088619021
0.108408418 -0.051999583 -0.139682139 0.086508702

23946 0.020550786 0.06091831 0.210449411 0.148633675 -

23947 0.020259661 -0.04708787 -0.278001493 0.087384188 -0.0816219 -

23948 0.194729876 0.186737252 -0.092272429 -0.18078476 -0.098912109

23949 -0.074734123 -0.005139813 -0.095687256 0.22216539 -

23950 0.209234025 0.044914156 -0.018149482 -0.124475111 0.061896556

23951 -0.008132493 -0.046236017 0.120274651 0.214587045 -

23952 0.168445058 0.048703932 0.029465299 0.184660535 -0.001007725

23953 -0.269759403 0.010738617 0.084843831 0.197632398

23954 0.027669574 0.055346193 -0.051213218 0.170550362 -

23955 0.07251787 -0.148803484 -0.154199548 0.149629035 -0.065189075

23956 -0.312062821 0.348350275 0.122314812 -0.081174053 -

23957 0.124130891 -0.092940209 -0.019607419 0.041176968 0.213902655

23958 -0.090994397 0.106879189 0.07636509 -0.232135892

23959 0.091722764 0.064816695 -0.052243654 0.160408867 -

23960 0.252498846 0.180821411 0.085053016 0.074247196 -0.162099832

23961 -0.158264467 -0.063408515 0.065472659 -0.222786031 -

23962 0.022628749 -0.182707314 0.007203038 0.217083392 -0.123746641

23963 0.338768831 0.00659518 -0.081452919 0.084343728 -

23964 0.13249369 -0.108415816 -0.083641375 0.012151578 0.005514542

23965 0.06767533 0.170712578 -0.227470147 -0.101527205 -

23966 0.135573278 -0.123233784 0.033156176 -0.149388184 -0.094542923

23967 0.165012117 -0.116097317 -0.116989194 -0.17896312 -

0.160100782 -0.014296114 0.133750226 -0.16718225 -0.237685991
-0.178431871 -0.18022344 -0.013390411 0.06890058 -
0.15803788 0.0617162 -0.106565975 0.214823929 0.052171805 -
0.161791306 -0.260395635 0.260815088 0.154100121 -0.055301408
-0.222025098 0.165828418 0.087241592 -0.173090726 -
0.078209477 -0.133990774 0.086795325 0.116470173 0.242594914
-0.102186129 -0.298463897 -0.065305683 -0.099447872 -
0.175157048 0.254829556 0.058796478 0.267069159 -0.247167072
0.255877161 -0.027203213 0.252740999 -0.147140549
0.200656662 0.144232064 -0.233099727 -0.027503198 -
0.106428628 -0.108598556 -0.236422853 0.185955148 0.006753108
-0.02438144 0.161631923 -0.035120394 0.253666401
0.228043913 0.204804534 0.255603857 -0.041486235
0.18819086 0.156124316 -0.052789629 0.079100196 -
0.19316767 0.291337956 0.169718775 -0.076150508 0.046103151
0.232108503 0.031102623 0.039386771 -0.065829349 -
0.003981527 0.152959189 -0.028711336 0.16335339 -0.311885811
-0.093392424 0.024525156 -0.03082574 -0.072388402 -
0.152253388 0.030829668 0.069273345 0.055961108 0.000470471
0.307127662 -0.091157201 0.217090219 -0.085904236
0.095843195 -0.003518135 0.181795433 0.302051582
0.176692398 0.169617566 -0.160960863 -0.123607262 -

0.227402779 0.061356292 -0.015019021 -0.232946132 -0.265532316

0.006648111 -0.323560732 0.018688529 0.108729969

0.03157807 -0.081657449 0.177193415 0.17866843 -

0.175504778 -0.186536242 -0.102370319 0.137303435 0.127065298

0.251423904 0.105010197 -0.208722645 0.068906002

0.146969066 -0.093298826 0.176234297 0.111224425

0.163380946 -0.097697609 0.001296865 0.095681663

0.174309037 0.098457995 0.109812662 0.005212527

0.221250203 -0.295330153 -0.084406208 -0.24130022 -

0.051423882 0.035118107 0.126512302 0.153055753 0.00860529

-0.156876073 0.089840839 0.110438626 0.146844144 -

0.209077028 -0.153954781 -0.212633505 0.112008836 -0.116508065

0.166060208 -0.221135301 0.169719626 -0.054627528 -

0.210508901 -0.016469963 0.06073323 0.402462915 0.120681287

-0.227433392 0.210264095 0.26980467 0.184524609 -

0.017324491 -0.225369809 -0.177408191 0.150103088 -0.054434267

-0.169766162 0.096110122 0.256326633 -0.084044121 -

0.065990068 0.063357294 -0.101864787 -0.337822161 0.141622421

0.001454646 0.030092289 0.015135407 0.120936052

0.074931842 -0.145285433 -0.253715278 -0.073979098

0.177680279 -0.06055983 0.167341058 -0.276452373

0.279230619 0.057307196 -0.233762548 0.039360703

24012 0.291423323 0.139930913 -0.122075278 0.018065979

24013 0.065020696 0.008767313 0.05879833 0.06066549

24014 0.149204787 0.033948762 0.17257581 0.013468434 -

24015 0.354924526 0.14975139 0.190282356 -0.167018348 0.040747305

24016 0.013160741 0.055423643 -0.10916341 0.059188218 -

24017 0.105836164 -0.015360083 -0.13067535 -0.026059089 -0.16616768

24018 -0.091250365 -0.130389459 0.118419555 0.208003946

24019 0.045089348 0.072593842 0.027136725 0.17249807 -

24020 0.183660297 0.186109011 -0.026386631 -0.063516458 0.116561871

24021 -0.04788987 0.066612738 -0.269301335 0.130253314 -

24022 0.044932878 0.1027174 0.1697221 0.233848147 0.029071414 -

24023 0.18260979 -0.11494354 0.070936715 -0.092938762 0.092402598

24024 -0.245964967 0.124360923 0.131379555 0.232668025

24025 0.014886883 -0.019111796 0.06721302 0.125777495 -

24026 0.103919641 -0.154410534 -0.04921399 0.056347338 0.13473437

24027 0.038140507 -0.231115878 0.02082699 0.018148772 -

24028 0.073605588 -0.098133993 -0.068484652 -0.311199929 -0.01284088

24029 -0.078307457 -0.193439551 -0.081621292 0.057102603 -

24030 0.098449141 0.114425604 -0.14962359 -0.00930581 -0.252947841

24031 0.171820961 -0.232536815 -0.073221946 -0.197250282

24032 0.12926084 0.159330792 -0.029167066 -0.057210661 -

24033 0.066203884 -0.036084401 -0.063098934 -0.027138322 -0.068969473

KEGG_GNRH_SIGNALING_PATHWAY -0.013788919 0.076874857
0.027283033 0.318063968 -0.128146023 0.043135675 -
0.134217469 -0.021560833 -0.016525143 -0.083577119 -0.05255952
0.034415652 -0.104132368 -0.09227396 -0.091233572 -
0.025629064 -0.01997427 -0.027651252 0.117039339 -0.04748096
0.268032879 0.083155606 0.127470624 0.153609712 -
0.024962275 -0.026065119 -0.29488357 -0.05281666 0.123003225
-0.015184751 -0.189724109 0.200274873 -0.086380222 -
0.114510662 0.009423806 0.13796651 0.122439401 -0.062102598
0.202481158 0.050294354 0.088971055 -0.039321933
0.262051175 -0.123992223 0.062237527 -0.02627508
0.217824994 0.071287058 0.064576855 0.059687814 -
0.004235789 0.035919999 -0.116741993 0.281093076 0.109386432
0.007840178 0.033659314 0.040564075 -0.040292188
0.236957829 -0.15390474 0.144928756 -0.18339023
0.229136706 0.092458538 0.0890207 -0.187650185 0.048189906
-0.146593234 -0.01289797 -0.110813024 0.171482265 -
0.020314569 -0.104670223 0.052298916 -0.164398791 0.042558798
-0.093112717 -0.150283777 -0.186829003 -0.060414297
0.25634728 -0.134617654 -0.103741327 -0.228393425 0.1249277
0.135978349 0.217483759 0.021249087 -0.088003983 -
0.095206551 -0.07205416 0.204004466 -0.170801311 0.115230827

24056 0.071539373 0.256468785 0.024187952 -0.070150736 -
24057 0.150866757 0.014164687 -0.02726935 -0.158878507 -0.185387367
24058 -0.035849582 -0.102807601 0.06763447 -0.143712265
24059 0.262931582 -0.024154757 0.081502082 -0.130727888 -
24060 0.179923876 0.050202547 0.051445775 0.039186808 -0.274122614
24061 -0.230761395 0.008538101 -0.030707326 0.002844105 -
24062 0.007630596 0.000922702 0.13378943 0.188609642 -0.058416494
24063 -0.201926751 0.186392612 -0.010004824 0.018792935 -
24064 0.097305294 -0.061862146 -0.066015374 0.042609012 -0.259533702
24065 0.204301636 0.160868818 0.174308387 0.003862074
24066 0.356453438 -0.001303479 0.045046232 -0.092737671
24067 0.056254089 -0.046411127 -0.086624142 -0.037397406
24068 0.095382427 0.00965409 -0.147233259 -0.016302038 -
24069 0.085915337 0.019358686 -0.019183185 0.069623537 -0.242375569
24070 0.159605952 0.140629394 0.212546193 0.017880051 -
24071 0.04213254 -0.135022581 0.040884799 -0.003638734 -0.192262525
24072 0.185811627 0.036859355 0.160682043 -0.086975543
24073 0.092638361 0.052670494 -0.037380905 -0.058657283 -
24074 0.062023094 0.215357469 0.129738673 0.154455354 0.01475746
24075 0.239144973 0.045781959 -0.075341001 0.250630022
24076 0.048051141 -0.223564674 0.134460076 0.203653176 -
24077 0.063717233 -0.091099526 0.035919263 -0.05005621 -0.132049885

-0.270507572 0.243603293 0.003201497 -0.287764986 -
0.069065593 -0.061862957 -0.022838856 -0.047777816 0.192862822
-0.01463003 0.02985008 0.113179924 -0.106517833
0.071215087 -0.122920914 0.021252534 -0.056765895
0.153642448 -0.197908551 0.066784142 0.01969148
0.18048918 -0.002363598 -0.067867988 0.125571385 -
0.218728761 0.004308282 -0.09051707 0.15758604 0.012160966
-0.062792512 0.080667737 0.013343121 0.049393474 -
0.120645135 -0.256755546 -0.109101582 0.117804568 0.09202416
-0.183664011 -0.060790587 -0.062418197 0.098378388
0.196937985 0.183806549 0.018939394 0.209982929 -
0.058652945 -0.153128805 0.109250717 0.062432428 -0.023008248
0.005135644 -0.203582793 0.130210432 0.092886315
0.094405592 -0.060180661 -0.107518592 0.106194082
0.064104781 0.12467443 0.268034744 -0.17799415
0.069145148 -0.051422114 0.025142109 0.058498273
0.099163735 -0.094988925 -0.094249885 -0.158184538 -
0.011252286 -0.201468888 0.266458374 -0.103556018 -0.193189146
-0.017484286 -0.001405521 -0.304288355 -0.13953107 -
0.140132601 -0.172334574 -0.061575919 0.028707007 0.066522212
-0.09500754 -0.03816825 0.035532184 0.209520503 -
0.169304696 -0.111017196 -0.083535992 0.12909951 -0.08410476

24100 0.017051379 0.078779234 0.002676864 0.054817954 -

24101 0.019690962 0.082723903 0.121712431 0.174016072 -0.106476205

24102 -0.107469221 0.170985259 0.217016692 0.008030427 -

24103 0.132964714 0.159123007 -0.044370504 -0.082752756 -0.035064115

24104 -0.149231294 -0.097277878 0.169172522 0.06491704

24105 0.151082109 -0.047020338 0.181504181 -0.172045492 -

24106 0.093311434 0.149736252 -0.058660676 -0.158192431 0.11076955

24107 0.118079712 0.098061442 -0.03009892 -0.011809589

24108 0.061317069 -0.061654395 0.027249466 -0.108379475

24109 0.024579189 -0.044184499 -0.067598608 0.015253726 -

24110 0.052890298 -0.062656038 0.113599794 0.022131228 0.08494746

24111 0.161881169 -0.010004438 0.175096042 -0.062151026

24112 0.124606481 0.051940069 -0.26777202 -0.067881685 -

24113 0.032024402 0.300832328 -0.000817187 -0.18369477 0.18699709

24114 0.008956177 0.1717085 0.073269446 0.245002994 -0.007526121

24115 0.149473933 -0.310671518 0.13468776 -0.20495091

24116 0.05846662 -0.02961042 -0.143669389 -0.141690539 -

24117 0.186580648 0.006469518 0.022374358 0.113408118 -0.164196905

24118 0.192740967 0.016294286 0.116004595 -0.083413908

24119 0.268348689 0.162192437 0.235105881 0.162492926

24120 0.116595011 0.032901877 -0.011134135 -0.063014406 -

24121 0.086660795 0.146319257 -0.002660373 -0.081927905 -0.153016735

-0.035416612 -0.05944333 0.10830075 -0.002289834 -
0.102544728 0.02481563 0.009052482 0.078880167 -0.214037398
-0.205478223 -0.114825276 0.005208874 0.086492649
0.276619872 0.042609617 -0.135196017 0.257873546
0.100265388 -0.038989204 0.025723021 0.030193201 -
0.040185093 -0.090360518 -0.069671194 0.05636934 -0.010505622
-0.09190244 0.038720545 -0.158816551 0.283759196 -
0.192578249 0.241761519 -0.026883479 0.010732244 0.132312287
0.072723983 -0.025320795 -0.079287664 -0.079202873 -
0.319231702 -0.086965854 0.146290976 -0.010471416 -0.119875014
-0.202439012 0.022256635 -0.167431886 -0.237836847 -
0.149752301 0.15634148 -0.068210998 0.042363155 -0.039832353
-0.008182704 0.16989469 0.15958477 -0.023146997
0.206722023 0.10362397 0.228199074 -0.088782287
0.12699535 0.13885174 0.128383521 0.056587192
0.131851173 0.101337185 0.23619451 0.121486356
0.042139209 0.0294031 -0.278901564 -0.346479749 0.076643844
0.031482124 0.065119835 0.074133252 -0.211596188 -
0.038439828 0.072776025 -0.068718072 0.07312728 0.249735572
-0.056295043 -0.083555198 -0.102816412 0.234558081
0.083778036 -0.181580928 -0.002102309 -0.083423585 0.2674102
-0.108730232 0.073711594 -0.087272311 -0.130735848

0.189507544 0.139474856 0.261311986 0.002341714 -
0.000655085 0.076760157 -0.119600707 -0.077203631 0.113434901
-0.273647913 0.061277117 0.100474557 0.118663204 -
0.046354169 0.043139454 0.000695485 0.005148154 -0.1228116 -
0.169137893 -0.026379473 -0.169714562 -0.122134017 0.116646417
0.016685014 -0.022828534 0.006727449 0.189886141
0.115202562 0.052378255 0.060848117 -0.04905121 -
0.052439795 0.085880471 -0.246745131 0.184071319 -0.060369618
0.077276784 -0.109837221 0.016999019 0.06657515
0.039225726 -0.018564489 -0.226656171 -0.05618655
0.012857043 -0.156589596 -0.039665469 -0.13025004 -
0.109739056 0.109955031 0.224385908 0.107412607 0.046075501
-0.292486941 -0.221980816 0.114199694 -0.12249802
0.068761041 0.164994685 -0.051204625 -0.001353672
0.012709329 0.040285227 0.080671989 -0.017288483
0.11943913-0.094503594 -0.165448867 -0.036037584 -0.124305116
-0.101436702 -0.050023097 0.080561111 0.030459929 -
0.05700338 -0.028930777 0.082725014 -0.108806936 -0.031975869
-0.111917295 -0.00967623 -0.128843063 0.022197838
0.166048725 0.092301598 0.013883106 0.122110944
0.082439669 -0.076215804 0.065901071 0.007336238
KEGG_PROGESTERONE_MEDIATED_OOCYTE_MATURATION

0.074205753 0.133732438 0.163389403 0.107303796 -
0.259216631 -0.021131842 -0.165139966 -0.182686212 0.113047015
-0.015252212 0.050237091 0.012041001 -0.006674422
0.04032003 -0.353294747 0.02900255 0.079971929
0.174372459 -0.125869838 0.001168407 0.106538877 -
0.059406065 0.112772649 0.410757168 -0.042879539 -0.190648099
-0.280496649 0.069272459 -0.010941079 -0.230609303 -
0.302461618 0.211852875 -0.086921666 -0.173911368 0.10398847
0.115155134 -0.157933769 -0.253555605 -0.177697383
0.202360533 -0.081922502 0.148699002 0.219928295
0.190508084 0.158083308 0.21492394 -0.021147293
0.037983578 0.263588865 -0.072856573 -0.019202349 -
0.060191204 0.113482866 0.147775709 0.158369679 0.331588208
-0.149090643 0.048794937 0.053524592 0.24655667 -
0.147221884 0.159407509 0.155541103 0.184283346 -0.001560095
0.024801951 -0.024612877 0.098409507 -0.306513751 -
0.289011535 0.28559405 0.310476149 -0.027002712 -0.215157387
-0.055687242 -0.269979238 0.004027679 -0.007238244 -
0.024710014 -0.373189126 -0.148841546 0.014787858 0.12288209
-0.197052732 -0.071794762 0.20158428 0.175144443
0.200557609 -0.02016549 -0.177701146 0.020592471 -
0.208807958 -0.123330755 0.098389012 0.300516357 0.148091788

24188 0.130447975 0.064615427 -0.218992516 -0.117626601
24189 0.120171435 -0.20370753 0.111239404 -0.200691896 -
24190 0.017286917 0.026733936 0.078804466 -0.0745376 0.344141172 -
24191 0.165051603 -0.225911343 -0.156884777 0.015363119 0.20524609
24192 0.114665788 0.282491751 -0.111900815 -0.235878748 -
24193 0.222412063 0.04351585 -0.306515071 -0.11880956 0.132069548
24194 0.098352626 -0.013024107 -0.281274794 -0.166411682
24195 0.24718461 0.051823328 0.148080499 0.051024777
24196 0.02406873 -0.215359477 0.169541631 0.089130415
24197 0.085919683 0.266856819 0.348760585 0.069237026
24198 0.362134953 0.173197005 -0.226783263 -0.011031966
24199 0.080781579 -0.059503955 -0.080598762 -0.00458912 -
24200 0.063146201 -0.2786334 0.027559946 -0.068427874 0.175386392
24201 0.211510846 0.054769026 0.231754524 -0.116166136
24202 0.045643581 -0.047810934 0.293080141 0.045530752
24203 0.153914913 -0.001960745 0.081917808 -0.264507298 -
24204 0.149118345 0.126640959 0.128101577 0.274384993 -0.31067027
24205 0.036871115 -2.72E-08 -0.198389337 -0.289749437 -0.133687906
24206 0.264133508 0.162543946 -0.018220768 -0.094904793
24207 0.122946113 0.171535098 -0.338716498 0.16164195
24208 0.047429444 -0.041183675 0.231541098 0.037904461
24209 0.075012837 0.040031170.116589369 0.107093715 0.032123153

-0.248170568 -0.187319011 -0.246057349 -0.136525387 -
0.210391806 -0.332365281 -0.116298405 -0.033079483 0.053089759
-0.10870026 0.096704286 0.231908837 0.002844258
0.067547754 -0.259727799 0.034642959 0.01358954
0.129402751 -0.225414858 0.075982355 0.107153239
0.119611803 0.288242109 -0.148100612 0.155062132
0.138415071 0.256529747 -0.041534629 0.249458938
0.164138888 -0.102413215 -0.309533616 0.29415042 -
0.238921421 -0.202997499 0.085598302 -0.262265395 0.111222842
-0.005604009 -0.216630382 -0.049958863 -0.286840919 -
0.096256194 -0.138962631 0.174508062 0.003388967 -0.033294889
0.178189536 -0.17534258 0.254055796 0.136595993 -
0.144279407 0.292341475 0.054541866 0.241125695 0.149916341
-0.081254535 -0.092236994 -0.26862063 -0.046171935 -
0.171547619 -0.188489345 0.323201134 -0.221525823 0.145490574
0.090165629 -0.261216927 -0.027252333 -0.184984452
0.022518921 -0.041926946 -0.190443941 -0.094691231
0.076910035 0.088690876 -0.266344889 -0.07000798
0.002035773 -0.328462004 -0.250443287 0.225509554
0.027052111 0.194076691 0.010379552 -0.111747998
0.025485675 -0.270459445 -0.052221825 -0.036902721 -
0.157369309 0.018134057 0.130781821 -0.204788987 -0.132286169

-0.298444095 -0.22708738 0.097178555 -0.017668119 -
0.290048185 -0.10850087 -0.087111836 0.07019645 0.231167141
-0.220135774 -0.133717406 0.112287069 0.16795686
0.102614096 -0.209998262 0.032325378 -0.17397733 -
0.248114898 -0.093203717 -0.251971373 -0.118182319 0.255909227
0.103836348 0.156004815 -0.226014131 0.03288824 -
0.302742531 -0.042476916 0.185268864 0.14893057 0.161867479
-0.253590771 0.269813727 -0.145763916 0.237155388 -
0.110873928 -0.015958965 0.143749202 -0.241563379 -0.054328409
-0.118789938 -0.189700468 -0.316257272 0.166501587
0.02645212 -0.015768392 0.056843714 -0.185747306
0.043017714 0.220286164 0.117167224 0.281879584
0.140390502 0.179991172 0.117729716 0.020949526 -
0.034417335 -0.120171879 -0.024011136 0.193913608 0.001202982
0.175495691 0.029665505 -0.030946321 0.128989051
0.20524132 0.175649187 0.218918593 -0.213492909
0.146798449 -0.425120681 -0.223655334 0.067453794 -
0.236269042 -0.165589881 -0.342938882 0.033095707 0.078021581
0.060429604 -0.017709406 0.337441256 -0.029022113
0.250247336 -0.215651965 0.258842294 0.19854666
0.228996263 0.084860694 0.186001150.168015427 -0.186734695
-0.18435162 -0.025298532 0.073981138 0.274499372 -

0.284670801 -0.208732265 0.0866159 -0.097660665 0.012250547 -
0.073337726 -0.040896534 0.227358722 0.138058523 0.26900819
-0.008108544 -0.197916345 0.007157359 0.21016516
0.040126327 0.188337857 -0.135366527 -0.10672308
0.061745335 0.089434647 -0.030408675 0.150764597
0.149711237 -0.016064414 -0.109973944 0.039568801
0.04317539 0.177185792 0.099242629 0.161754488 -
0.212168989 0.215743291 -0.335804872 0.154028923 -0.314542279
0.019737141 0.197672252 -0.008060223 0.151053897
0.060099706 0.199560364 -0.330309016 0.021078383
0.308844621 -0.367928279 -0.16090986 -0.429622731
0.223867913 0.178483279 0.164590313 -0.241940581
0.031454527 -0.053847066 -0.311612438 0.09163918
0.251345184 0.327181175 0.007934121 0.196726753
0.034330429 0.173870489 -0.013564425 -0.135386509 -
0.207403721 -0.029484015 0.103406431 -0.16249406 -0.137846072
-0.130447393 0.181592142 0.250509385 -0.245609718 -
0.152911391 -0.280735104 -0.197814859 0.242476318 0.187332093
-0.106204984 -0.136200664 0.081351879 -0.167687647
0.075488909 -0.147876798 -0.118411253 0.27188178 -
0.004914327 0.090896678 0.017587174 0.207159022 -0.212782534
-0.015973395 -0.034699055 0.264245814 0.168928395 -

0.177984233 -0.117578925 -0.167078502 -0.048938085 0.151613877
0.14458462 0.176917164 -0.055548388 -0.053424997 -
0.079612345 -0.078869346 0.062684375 0.337844055 0.09546043
-0.107206755 0.128994438 -0.136640183 -0.167642097
0.173242821 0.078953691 0.057002114 -0.280105036 -
0.191962468 -0.192658243 -0.024534467 0.10568764 0.169312704
-0.144745703 -0.129989983 -0.115221741 0.211450848
0.306556909 0.292233024 0.316701711 -0.170340726
0.001206489 0.084505917 -0.24349095 0.264175981 -
0.146766111 0.144040852 0.084302462 0.162726532 0.187993172
0.046202997 -0.123208549 -0.24335225 -0.160543145
0.058300274 -0.221550615 -0.10667009 0.114508231
0.078803442 0.045507394 0.148506743 0.176892779 -
0.066159928 -0.116067799 0.130491487 0.047728491 -0.008101143
-0.279517216 -0.054894768 0.118741634 0.161270288
0.068684901 -0.126808693 0.22044275 0.149469978 -
0.054039735 -0.10514076 -0.268215347 -0.159945057 0.056129521
0.017404878 -0.112292506 -0.018305613 -0.086999183 -
0.017763045 0.01204679 -0.123905598 -0.192081154 0.032642698
0.018972302 -0.031481268 0.024737507 0.03293432
0.055035256 0.137927202 0.00123005 0.034127759
0.113803077 -0.130955735 0.1072322 -0.246670928

KEGG_MELANOGENESIS -0.104080896 -0.086695932 0.026896146
0.197914691 -0.065924368 0.068120457 -0.124237923 -
0.102085181 -0.094169972 -0.097833315 -0.017431252 -0.095165389
0.204103059 0.020036514 0.056421412 0.10085722 -
0.12317919 -0.101352115 0.213918747 -0.178923256 0.193866576
-0.132906835 0.234060955 -0.024353036 0.018877361
0.065186356 -0.197215487 -0.049501555 0.034818069
0.032657635 -0.205326123 -0.226460097 -0.177920481 -
0.149279348 0.122817371 0.206304026 0.286039111 0.020516817
0.196555702 -0.213793067 0.075728227 0.114964197
0.101915394 -0.08820446 0.047806272 0.015034689
0.101827835 0.135468295 0.019521073 0.01426061 -
0.035883196 0.057004344 -0.135397632 0.137925651 -0.064438292
-0.064629268 0.143875854 0.100581508 -0.083569372
0.34215349 -0.087258554 0.314427331 -0.091048056 -
0.048306467 0.046706718 -0.046380455 -0.086581794 0.130336003
0.022009472 -0.099592829 -0.180574886 0.036675259
0.074058898 -0.200163601 -0.013279253 0.027517046 -
0.036466022 -0.286804165 -0.109234404 -0.228539036 -0.022049255
0.22056722 -0.206959668 -0.004100566 -0.15619194
0.003440909 0.152219228 0.250964376 0.086050157 -
0.263309093 -0.004909707 0.133007874 0.018001022 -0.026275072

-0.113184982 0.028223411 0.167736174 0.124255305 -
0.179185712 -0.03255948 -0.165748547 0.023257454 -0.101092312
-0.070833066 0.051394594 -0.134208475 0.064718694 -
0.150759576 0.241668237 0.154947036 0.326335506 -0.11413079
-0.215131712 0.202885047 -0.060844001 -0.20044969 -
0.239168459 0.001390065 0.099298187 -0.078716416 -0.034016917
-0.069166564 -0.16112834 0.239875341 0.213916828 -
0.003028471 -0.16421546 0.116829561 -0.091569192 -0.10257487
0.172079995 -0.05889 -0.110600949 -0.189060858 -0.108629674
0.276874148 0.307149774 0.05195945 -0.117612356
0.29649283 0.029707435 -0.169767198 -0.002406741 -
0.087577404 -0.154156317 0.16866528 -0.02555913 -0.196327092
0.012648435 0.038580205 0.248585314 0.062673099
0.133211985 0.228591139 -0.029350347 0.07194877
0.096258991 -0.225291304 -0.065840512 0.032751406
0.107424814 -0.239506567 0.01383254 0.077013774 -
0.158573557 0.112959851 0.085447945 0.18898 0.001674494
0.103402511 -0.1794584 0.025272779 0.10673566 0.154724569
0.213488428 0.213077308 0.008962314 -0.090268313
0.057292226 0.007492173 -0.12566283 0.20332886 -
0.04290796 -0.101314203 0.193396595 -0.039239994 -0.035081861
-0.053494212 0.182575318 0.012960952 -0.241668224 -

0.18651005 0.003975303 0.141799622 -0.401041279 0.026710634
-0.095935721 0.049485569 -0.03683707 -0.015319464
0.123262282 -0.222372372 0.23542562 -0.063508386
0.003430617 -0.233160779 -0.01350547 -0.195151731
0.137668591 -0.392953056 -0.136153088 -0.047706811 -
0.096474132 -0.047158126 -0.080845177 -0.232334765 -0.31977484
0.076289553 -0.052885804 -0.101345439 0.123513159
0.021674758 0.164356369 -0.128538497 0.069425047
0.124668519 -0.220095387 -0.120931765 -0.008640939
0.199077375 -0.212173715 -0.13521106 -0.113893295
0.141905756 -0.025097684 0.213429326 0.151130735 -
0.078762209 -0.053121278 -0.126897897 0.185921292 -0.008412335
-0.095603172 0.03058057 -0.051011006 0.088918929 -
0.060864027 0.024040912 -0.08991953 0.021272601 -0.071364311
-0.002566776 0.121527251 0.350186018 0.015643543
0.301366857 0.104120024 0.18023973 -0.03184095
0.04986407 -0.056735066 -0.160993142 -0.016278071
0.174553833 -0.114680871 0.103321693 0.067433839
0.079446418 -0.118629567 -0.010190966 -0.269432346
0.029313339 0.02793092 -0.009863364 -0.226446463 -
0.074168667 0.055490039 -0.047650106 -0.108722731 0.098979578
0.282862232 -0.141288042 0.036387099 0.159355411

0.09435282 -0.13696015 0.010628676 -0.191809486
0.172567267 0.119758912 -0.08002309 0.133082685 -
0.06968938 0.210886671 0.070619197 -0.072924791 0.098721133
0.128472907 -0.101280599 -0.05477707 -0.059432455 -
0.092650803 0.071638111 0.029621108 -0.172654879 0.005574805
0.069328476 -0.06739779 0.299354088 -0.048471909
0.085917131 -0.165787703 0.185238369 -0.024050533 -
0.204904436 -0.027051359 0.214230999 -0.193762941 -0.198956745
-0.077423121 0.153823478 0.014796956 0.045841705
0.210970878 -0.209468636 0.1116042 -0.012672195 -0.09889165
0.086891379 -0.011884186 0.029298858 -0.010534523
0.084583901 0.0590221 0.238256513 -0.118677977 0.02641375
0.058512815 -0.08994183 0.081575793 -0.190545566 -
0.163693835 0.0127025 0.269277079 -0.159732757 -0.10048156
0.008921022 -0.263447758 0.068445769 0.033182771
0.327401105 -0.010298469 0.124207008 -0.302656198
0.010134898 -0.10506037 0.127966152 -0.163971058 -
0.155782236 -0.017975273 -0.122295243 0.069550651 -0.188354975
0.253978885 -0.184917675 0.365208789 -0.171669785
0.066373047 0.051854196 0.07310473 0.150732797
0.275769504 0.104558744 0.1513218 -0.225847403 -0.073747114
-0.129213285 -0.135231497 0.019010146 -0.015830444

24386 0.100224472 0.088022023 -0.064986119 -0.041868246

24387 0.208034507 -0.080372077 0.101149035 -0.000340053

24388 0.038318062 -0.063460892 -0.263120877 -0.289660128 -

24389 0.042871218 0.091044695 0.214127183 0.35916898 -0.210098413

24390 0.067502683 0.272881846 0.108335329 -0.155603766 -

24391 0.03853623 0.156415317 -0.090203418 0.067503327 -0.151429593

24392 -0.050329537 0.022486921 0.015723352 -0.299501146 -

24393 0.185542992 0.066532896 -0.108642258 0.091182606 0.157702891

24394 -0.082539572 -0.094597796 -0.200392989 0.16481297 -

24395 0.238673544 -0.137215826 0.021558085 0.092309699 0.121409006

24396 0.083187722 0.041887698 -0.223732744 0.2435832 0.029720237

24397 -0.127357512 -0.010782302 0.085739348 0.050089877

24398 0.076385519 -0.013005138 0.074911693 0.11824538-0.020288829

24399 -0.250516079 0.189029406 0.092670747 0.174924408 -

24400 0.289209085 0.223948604 0.003128296 -0.158211896 0.217175266

24401 0.126961632 0.079038035 0.021098851 -0.193441888

24402 0.075123618 -0.101843445 0.198597272 -0.260915339

24403 0.128260211 0.159024363 0.318663267 0.000325479

24404 0.004467039 0.030025158 -0.087666042 0.068620741

24405 0.354647442 0.094968523 -0.108318247 -0.077254644

24406 0.119270655 0.129125012 -0.052829339 -0.214969066

24407 0.015517723 -0.100980554 0.204517236 0.082550059

0.006910129 0.217900584 0.048231861 0.01139136-0.025547953
0.092003879 0.162864294 -0.06206 0.014607971 -0.08840888
-0.394345989 0.029162738 -0.247884988 -0.039406731
0.05734693 0.090021938 -0.029841879 -0.076204294 -
0.125972826 0.04772424 -0.045223454 -0.062898409 0.035007753
-0.177476901 -0.113674758 -0.068833474 0.038985561
0.117777182 0.088845603 0.379469977 0.219381025 -
0.086121084 0.285540842 -0.041121059 -0.195162113 0.002981745
-0.083827193 0.299467526 0.031391775 0.12909829
0.011063945 0.042365358 0.001274644 -0.134008193
0.04401291 -0.224771038 0.221279642 0.127871213 -
0.127392041 -0.17005185 0.14487463 -0.07604482 -0.072457585
0.049237862 0.182788548 0.060233617 -0.088798674 -
0.149857341 0.170054628 -0.151093614 0.223475187 0.13628084
0.037272462 -0.0073764 0.071618495 0.003966197 0.060065462
0.075319203 0.065093843 -0.05295907 -0.262350735 -
0.055101553 0.092694716 -0.04259278 -0.190199958 0.141024579
0.215115582 0.028412869 0.022379538 -0.081113274 -
0.10994913 -0.042770587 -0.013223822 -0.261234644 -0.201362586
0.008571064 0.122020291 0.248927565 -0.228575827
0.129115080.101095769 -0.011828604 0.055122259 0.003546508
KEGG_ADIPOCYTOKINE_SIGNALING_PATHWAY 0.139316138

0.250170137 0.010659293 0.26752672 -0.100351337 -
0.086425793 0.137519396 -0.266697185 -0.222264074 -0.082014935
-0.006520863 0.143798596 -0.320441701 -0.082434997 -
0.298644837 -0.337652962 0.215440842 0.017853014 -0.197980738
0.053157681 0.228884577 0.23412365 -0.094851564
0.12113203-0.285624614 -0.01473868 -0.254542094 0.001211739
0.183484506 -0.186183004 0.129895804 -0.038468506
0.241296883 -0.046328967 0.078206391 -0.161964146 -
0.161938768 -0.110820303 0.008434283 0.330905476 0.172742692
0.04949913 0.174872969 0.191111866 -0.1099335 0.123171972
0.308187711 -0.025696624 -0.071629016 -0.246798629
0.356018555 -0.082670959 0.067123488 0.165459911
0.101175341 -0.095753372 -0.249789592 0.10191801
0.252394584 0.243541846 -0.37695203 0.057986628
0.231088358 0.279649769 -0.086187936 -0.124796571 -
0.091611286 -0.110559406 -0.109347304 -0.216163692 -0.065345549
0.079561117 0.193451568 -0.159110414 0.05039215 -
0.191687778 -0.184186195 0.149418734 -0.015407438 0.119291996
0.078381848 0.282588289 0.045273935 -0.279630372 -
0.264113741 -0.192400626 0.152704448 0.03371762 0.024047215
0.18873099 -0.335617359 -0.247692103 0.240271806
0.020341126 0.188991559 0.255582582 0.141020334 -

0.167057994 0.224852259 -0.122846255 -0.000222381 -0.20919408
-0.059883215 -0.076370474 -0.148363468 -0.107493624
0.302233859 -0.104523728 0.187957484 -0.115617595 -
0.085897595 -0.056401684 -0.016516152 -0.119844779 0.256029422
0.167468901 -0.158312298 -0.23710437 -0.218452067
0.031030863 -0.031035709 0.281417348 0.107807852 -
0.005254961 0.04210692 -0.244064397 -0.127343471 -0.014904778
0.235786171 0.25641762 -0.018910763 -0.116467493
0.063481927 0.270313667 -0.086499931 0.215927221 -
0.10542062 0.159524903 0.105389368 0.250343769 0.012152119
-0.123153751 0.24813323 -0.176013073 -0.21332706 -
0.229895531 0.265520098 0.102670846 -0.333252072 -0.302287112
-0.202526278 -0.295370264 -0.189894943 -0.114164825
0.097597376 -0.147666067 -0.133046534 0.187767375
0.183574816 -0.250570747 -0.024608499 0.040041828
0.024741385 -0.231306259 -0.042598363 -0.063734139
0.14731276 0.034809022 -0.32878063 -0.108558952
0.106439386 0.018938775 -0.010019869 0.080942809
0.261295363 -0.054788043 -0.029510087 0.314791831
0.130382137 -0.055686923 -0.075736445 0.161877227
0.129842489 -0.179053365 0.147354152 0.011233246
0.157847878 -0.076683653 -0.351316227 0.1081371 -0.258568331

-0.216578462 0.360901068 -0.077470344 -0.055551922 -
0.10148985 -0.081943007 -0.140976658 0.146773123 0.296930172
-0.382575187 0.02196477 -0.187066309 0.17106047 -
0.10043122 -0.0903558 -0.115475687 -0.127966068 0.035856221 -
0.090261671 0.285236458 0.24395342 0.07171547 -0.021164794
-0.312278805 0.069023844 0.096171766 0.05701459
0.025141369 0.217127339 -0.131938999 0.153930886
0.037147797 -0.139744416 -0.056821584 -0.030413724 -
0.13017851 -0.32215404 0.207400324 0.012564352 -0.170155932
-0.13328207 0.279220933 9.39E-05 0.211824873 0.141005225
-0.262942564 0.225328811 0.266900097 -0.27163382
0.013230843 0.213840481 0.008029722 0.125713014 -
0.403583753 0.045302852 -0.05152098 -0.002698432 0.088674172
0.010027222 0.05566073 -0.047936563 -0.276741948 -
0.170815321 -0.138118377 -0.123863807 0.185507375 -0.015346859
0.378568115 0.121268009 -0.063466168 0.048058611 -
0.169999405 -0.09519167 -0.07395106 0.091112925 -0.013698484
-0.136285131 0.368252801 -0.209664337 -0.029393361 -
0.133819287 -0.164706579 -0.192562671 -0.004317949 -0.018408792
0.223575933 -0.013913144 0.000114746 0.039673744 -
0.131767752 -0.073925147 -0.166944672 -0.225721506 -0.319164466
-0.09868729 -0.164225498 0.293964558 -0.075492571

24496 0.067882963 0.150615532 -0.066779483 0.263790337

24497 0.139111336 -0.220743733 -0.182653645 0.254088902

24498 0.246089901 0.016919844 0.088370108 0.193009487 -

24499 0.003054002 0.000646973 -0.206947922 -0.069152939 -0.080180068

24500 -0.040678288 0.316490525 -0.222673904 -0.246679452 -

24501 0.089723966 -0.189441078 -0.157364538 0.220019389 -0.180238054

24502 0.161595786 -0.331312714 0.236758078 0.243556061

24503 0.075164668 0.028614518 0.233508851 0.170834708 -

24504 0.232994732 -0.065740885 0.094903258 0.001270209 -0.210333815

24505 0.101316758 -0.284600185 -0.197200084 0.234348311 -

24506 0.239640906 0.195792957 0.178512236 0.280096703 0.253888723

24507 0.048950131 0.354255942 0.270625799 0.064592788 -

24508 0.001245454 -0.096370835 0.29720663 0.170392551 -0.081417017

24509 0.007420721 0.289488717 0.069752825 0.105076644 -

24510 0.131689559 -0.08530191 0.106151836 0.022136067 0.166234693

24511 -0.119985279 -0.017792119 0.093427269 0.165521837

24512 0.015025352 0.017284872 0.175772007 0.233466849 -

24513 0.029805231 0.1682231 0.205681499 -0.183336218 0.115829108 -

24514 0.09302817 0.099606814 -0.031760862 0.028603097 0.216958258

24515 0.269910205 0.082096767 -0.269790678 -0.164356303 -

24516 0.072949307 0.211559833 0.084022912 -0.262878372 -0.379474107

24517 0.022869863 -0.286432043 -0.0828341 0.195547589 -0.118153103

-0.043287631 0.170971782 0.094819709 0.100223155 -
0.059912857 -0.075451019 0.117970937 -0.091132062 0.211602036
0.204165734 -0.379348039 0.275352026 0.354825629
0.030552749 0.058401854 0.109145817 0.040842469 -
0.178046101 0.144884019 0.154757668 0.085181489 -0.16597668
0.159905411 0.054820514 0.296646374 -0.215958203 -
0.131573669 -0.188373533 0.070531539 -0.077596793 0.068039949
-0.00914714 -0.310769765 -0.075235946 0.013897909
0.012988503 0.024231207 -0.170234865 -0.361618684 -
0.173360769 0.209336155 -0.273905659 0.084549027 -0.132199596
0.104159452 -0.13147544 -0.077925399 -0.099096324
0.056314857 0.329269701 0.258323724 -0.194555376
0.168304141 0.248163353 0.122198959 -0.008210175 -
0.079504546 -0.308268024 0.266748248 0.179767435 -0.336871486
-0.052939547 0.27724061 -0.104353579 0.096228777
0.229866647 -0.230421439 -0.110249808 -0.052226989 -
0.054060658 -0.173868517 0.239593486 -0.10890278 0.198278521
0.074473792 -0.362183955 -0.100474889 0.146868667
0.029138267 0.057815489 -0.308808536 0.223508028
0.180960614 0.015322828 0.007000534 0.13491269
0.054601452 -0.171898687 0.14159315 -0.086463217
0.009549086 0.135268933 -0.004882475 0.348101201 -

0.26439392 0.104019116 0.221550237 -0.270957142 0.249951634
0.272107614 -0.258594901 -0.123307706 -0.077966966
0.123140433 -0.123417358 0.013760384 0.006039778 -
0.132988976 -0.154826935 -0.297678928 -0.092572479 -0.227517508
-0.00335559 -0.128338686 0.260735062 0.032569624
0.18246287 -0.061841406 0.053136516 -0.245685462
0.015736362 -0.066076949 0.160062521 0.043750163
0.007010903 0.235864888 -0.198235098 0.226176711
0.022165564 0.267342292 0.124201656 0.282045628 0.1988692
-0.07963117 -0.195402353 0.141642207 -0.028400414 -
0.07510799 -0.354785813 -0.070885438 -0.142660241 0.371509014
-0.049477368 0.196164573 -0.336082285 -0.106030776 8.71E-05
-0.360495766 0.021397645 0.110526697 -0.016374568 -
0.014059243 -0.182658914 0.042567354 -0.135255252 -0.049368598
0.169753931 0.171677982 0.18310309 -0.038298422 -
0.277470091 -0.119658958 -0.177188964 0.128638645 0.128956993
0.098637239 0.107379685 -0.175337902 -0.086710406 -
0.000670125 -0.151656506 -0.103020783 -0.188572023 0.051629255
0.186414931 -0.090404911 -0.031286712 0.012775005
0.02358155 -0.045794312 -0.000527493 -0.000958149
KEGG_TYPE_II_DIABETES_MELLITUS 0.049346663 0.059866229
0.054234197 0.146970629 0.0944158 0.003774969 0.182305937

0.080334615 -0.218358547 -0.015774791 0.013164704 -
0.223514166 -0.110408067 -0.252931297 -0.111081317 -0.043928311
-0.11356326 0.057961421 0.052147996 -0.185256479
0.308475284 0.369711604 -0.036594062 0.237254369 -
0.219291387 -0.028591593 -0.317584682 0.061883651 0.094462572
0.192705642 -0.042384561 0.205196305 -0.076929457
0.124672679 0.093768272 0.169012284 -0.209930295
0.196569752 0.193935269 0.211812708 0.034719991
0.190578494 0.060845448 -0.055899301 -0.057706523 -
0.077507128 0.15382558 0.20616603 0.089827869 0.164847441
0.137605912 -0.179921099 0.188922742 0.154683073
0.09467018 -0.067408126 0.037465107 0.123947817
0.078453747 0.002140869 -0.240022015 0.253667714
0.075237055 0.149872447 0.046919058 -0.070056434 -
0.002685074 -0.166955368 0.222851827 -0.026239266 -0.037873732
-0.027536657 0.032733296 -0.247302733 0.222821916
0.179628415 -0.007969279 -0.014622372 0.020827024 -
0.171972902 -0.140138328 0.245609263 -0.026695087 -0.012092881
-0.114216596 0.037051016 -0.002455667 0.103596639
0.084555935 -0.079887275 -0.225109001 0.148384994
0.100907891 0.048127501 -0.158439807 0.104826125
0.256241381 0.070258387 0.073952649 0.115707276 -

0.181839234 0.109318757 0.047159239 0.114007480.028523101
0.041507671 0.22706838 0.111638389 0.013827264
0.167172083 0.346753212 0.037395905 -0.063189943
0.073973974 0.082771443 0.039577848 -0.232998344 -
0.076886943 0.028541607 -0.199330558 0.090295956 0.014954542
-0.090530617 0.077241717 -0.041329325 0.040257138 -
0.238284942 0.011852530.201319962 -0.048750135 -0.256791183 -
0.118187166 0.010380291 0.144727244 -0.194522178 0.116302932
-0.265017281 0.18552452 -0.085904265 0.186092028
0.012705072 0.039400735 0.034422263 -0.214220098 -
0.249476432 -0.204115684 0.203734988 0.043265935 0.091133478
-0.018587788 0.061321014 -0.133129949 -0.031613147
0.031731896 -0.102085945 0.111266958 -0.254539205
0.034900315 0.280273622 0.165944547 -0.055568254 -
0.044552111 0.059536559 -0.057502936 -0.165000131 -0.014363278
0.024147367 0.210781993 0.088662913 0.153107765
0.110684166 0.129679015 -0.000444579 0.18331861
0.286505651 -0.007586053 0.015232982 -0.04646354
0.079034632 -0.07937991 -0.134477414 0.303702925
0.090629857 0.033724331 0.08839525 0.341286706
0.04782394 0.024010605 0.03798289 0.081627424 -
0.24778756 -0.203104669 0.272830033 0.024217635 -0.277558373

24606 0.214193463 -0.052392606 -0.012153253 0.014427777

24607 0.153475182 -0.159486901 -0.006563012 0.041031491

24608 0.105811635 -0.258097285 -0.053513623 -0.083254036 -

24609 0.064632293 0.119264383 -0.130762226 -0.007718348 -0.03188134

24610 0.085335501 -0.185229851 0.13860535 -0.15340298 -

24611 0.228730874 0.280584042 0.035282024 -0.010968318 0.183674119

24612 0.023389032 0.113809984 -0.420514226 -0.055864532

24613 0.175120305 -0.035665091 -0.175480942 0.229706284

24614 0.045162666 -0.147672997 0.010294953 -0.066848182

24615 0.279604761 -0.05354281 0.192603798 0.102558116

24616 0.066581859 0.235630952 -0.292294161 -0.089689605 -

24617 0.229326933 -0.186486979 -0.016574549 -0.321446984 0.076771196

24618 0.0785189 -0.027723926 -0.013115696 0.194783453 -0.01380294

24619 0.079111688 -0.017999138 -0.215596735 -0.178500223

24620 0.136111259 -0.036168978 0.251081523 0.280447147

24621 0.166138268 -0.119308102 -0.094220241 -0.135754881 -

24622 0.025156226 -0.199076714 0.135447928 0.272885906 -0.07277564

24623 0.151703954 0.194602903 -0.118184551 -0.215797865 -

24624 0.094050752 -0.093146592 -0.17072156 -0.153131272 0.0211818

24625 0.046807631 0.077131842 -0.207488017 0.038263956 -

24626 0.046817138 -0.173334834 0.342452181 0.071450148 -0.039034379

24627 0.242475961 -0.142345722 0.180256031 0.039740411

0.168634898	0.028934654	0.054643515	-0.216688848
0.215371029	0.201419734	-0.0025628	0.158443914	-0.153020508
-0.068124282	0.014767139	0.224009762	-0.016536285	-
0.047410258	-0.17512883	0.058022851	0.079305598	0.08114351-
0.048215034	-0.043966765	0.09541201	-0.30648633	0.044299064
0.023417408	0.078511945	0.225216309	0.088408296
0.328651527	-0.190287887	0.031122625	0.085097801
0.168152748	0.110648356	0.25074121	-0.140860396
0.174806516	-0.265746156	-0.129981915	0.051813426	-
0.261528982	0.013752091	0.107315535	0.110059878	0.154026458
0.193936508	-0.060203907	0.138964155	0.063640143
0.212732635	0.240380172	-0.126533066	-0.072323203	-
0.044179044	0.16718669	-0.07282409	-0.022059355	0.010626577
0.259492424	0.20258213	0.025437536	-0.13027949
0.078760237	-0.15093313	-0.302778134	-0.052117305	-
0.056923504	-0.121521713	-0.075678812	-0.185023835	0.10206991
-0.26683833	0.130193575	-0.089129267	-0.087189476	-
0.073598189	0.364206113	-0.260087455	0.110003695	-0.177368136
0.074841763	0.048977214	0.072317169	0.163394886
0.146627251	0.075651737	-0.193983547	0.078196952	-
0.1649985	0.008532267	-0.070237326	-0.122031667	-0.110868943
0.186475708	-0.146463537	-0.075751081	0.173541051

0.165162826 -0.032321406 0.005501712 0.278433 -0.082799935
-0.259857033 -0.020802165 0.216645252 0.22883834
0.003938025 0.255315675 -0.28303657 0.158637508
0.264274924 0.160343154 0.150972189 -0.006507992
0.319412599 0.070115188 0.067205582 0.15224531
0.167386515 -0.010653406 0.108908493 -0.1197608 0.076988919
-0.170983759 -0.114023942 -0.100683607 -0.040734194
0.081357705 -0.127224947 0.223053054 -0.04342596 -
0.125768373 -0.23449654 0.064880547 -0.048909053 0.030445596
-0.294959403 -0.013961252 0.147461809 -0.250045896 -
0.026327388 -0.065084473 -0.041531596 0.153020934 0.122975316
0.045669449 0.051931955 0.292385363 0.022219393 -
0.453333436 0.033053797 0.128060488 0.023848341 0.015174936
0.297446209 -0.182732024 0.106505394 0.09425673
0.037568013 -0.112459017 0.313517478 -0.176117337 -
0.050526803 0.215926118 0.161155432 -0.280464643 0.209003781
0.009542351 0.008820505 -0.156892274 -0.023017158
0.093044707 -0.043017841 -0.007698099 -0.131533291 -
0.111372628 0.12787357 0.056193844 0.033579608 0.143227745
0.102825789 -0.048595435 -0.043053142 0.27284608
0.131226695 -0.190669343 -0.22495496 -0.093714105 -
0.051963216 0.121112131 0.114112424 -0.099030564 -0.195750401

0.097232307 0.254677184 -0.189315997 0.076656956 -
0.030606687 -0.209952788 -0.031517583 0.029647669 0.300114139
-0.028312813 -0.189833656 0.003124069 -0.069628011 -
0.064089498 -0.104637526 -0.140882189 -0.439962449 0.062279467
0.216674635 -0.140801704 0.003041322 0.152455637
0.061739089 0.026998476 0.029130011 0.158234833
0.129927064 -0.262596246 0.045715751 0.018977085
0.183771449 0.158370557 -0.045573301 -0.071359402
0.092302088 -0.193382946 0.226490003 -0.102918958 -
0.185048136 -0.252949531 -0.01758631 0.064450084 -0.194067409
-0.375730859 -0.137442383 -0.045939575 0.249912732 -
0.13050235 0.375819068 -0.278293527 0.175461014 -0.193679686
0.065411557 0.211286929 0.208757082 -0.180080979
0.203088429 -0.172977773 -0.173083094 0.175608831
0.181851056 0.072833515 -0.106704379 0.080715272 -
0.109519381 0.052987368 0.0576632 -0.215381123 -0.048711919 -
0.285043594 0.162944645 0.152442254 -0.281008372 -0.347938391
-0.095379441 0.17473665 -0.031999038 -0.10671261
0.191484145 0.087904498 0.309366569 -0.164550136
0.201552962 0.03371006 -0.294378179 0.037007169
0.170320246
KEGG_TYPE_I_DIABETES_MELLITUS -0.470814611 0.541542445 -

0.605895406 -0.177205317 0.312447914 0.018906663 -0.225097034
0.281553603 0.404149771 0.440227083 0.320837937 -
0.460360059 0.267375215 -0.630308318 0.532335294 -0.312407057
-0.496226312 0.650930054 0.104434574 0.633576132 -
0.595486813 0.546562807 0.440936501 -0.647968313 0.569662121
-0.053208314 -0.042515676 0.326396467 0.142895236 -
0.335987652 -0.549550021 0.44537925 0.328424761 0.657951402
0.440874621 -0.376850531 -0.254590409 0.393472335 -
0.61072775 0.100538691 -0.396248777 0.362841068 -0.306188077
0.597961336 -0.130188438 -0.108394885 -0.39095983 -
0.463073614 -0.451359919 0.490288481 -0.32350834 -0.299244142
0.316947955 -0.506821472 -0.465754697 -0.511051922 -
0.288955557 0.227398324 0.114486386 -0.403897272 0.317553679
0.479522273 -0.020888218 -0.468526332 -0.489495257 -
0.082782872 0.286925049 -0.741839454 0.48584173 0.585220316
0.538206207 0.67407828 -0.447370119 0.45978031 -
0.503136362 0.47335493 0.466521847 -0.464992917 0.476254855
-0.413194442 -0.470502646 0.340648243 0.757784539 -
0.128990644 0.385056583 0.336887896 -0.626169663 -0.136378373
0.625402695 -0.267320294 -0.505419712 0.125415143 -
0.044632348 0.344665392 0.587376497 0.579765216 -0.314009617
0.600781479 0.290495463 -0.340756493 0.527902305

24716 0.436685278 0.158843397 0.585670332 -0.003302513

24717 0.159709838 -0.523470612 -0.307455123 -0.457782256

24718 0.62953558 0.246482705 0.233326835 -0.092496904 -

24719 0.583395361 -0.058619485 0.561074265 0.440055793 0.25961589

24720 0.028113144 -0.462065202 0.08962789 -0.161450223 -

24721 0.242004699 -0.602224388 0.202135526 0.202483714 -0.342365729

24722 0.308523225 -0.307908993 0.619677356 0.570604782

24723 0.553516131 -0.540034168 0.561724656 0.53673158 -

24724 0.157869943 -0.005847196 -0.483326265 -0.342354965 -0.457173225

24725 -0.184164119 -0.266958662 -0.625499014 -0.447601302

24726 0.276383403 -0.543550934 0.473451882 0.348232183

24727 0.261009211 0.090751649 0.605601170.24403269 0.647052679

24728 0.584723104 0.239276692 -0.365511299 0.136449522

24729 0.407267602 -0.24857021 0.584023828 -0.453151088

24730 0.077768125 -0.111340851 0.325833915 -0.272743682 -

24731 0.473318856 0.493406079 0.257493487 0.356662879 -0.189784323

24732 -0.608496286 -0.421511854 0.170815338 -0.482055794 -

24733 0.05093874 -0.676794773 0.12040271 -0.311007902 -0.276665483

24734 0.607916847 0.470982132 -0.388498018 0.539637888

24735 0.229828204 -0.425352864 0.214070392 -0.649312053

24736 0.128812364 0.601339434 -0.220028286 -0.043408031

24737 0.408502301 -0.237917532 -0.421900164 0.467574772 -

0.181427221 0.367283614 0.267267995 -0.219901422 -0.458918941
0.171523497 0.361411065 0.016343027 -0.285441897
0.074607493 0.395020145 -0.325831026 -0.359640556 -
0.622604544 0.397731305 0.471448915 -0.603808782 -0.394097841
-0.1252317 0.342313815 -0.084809955 0.264525858 0.518147493
-0.371673595 0.44680436 0.572267633 -0.286360924 -
0.029371964 0.03518716 0.302809346 0.130137502 0.332712175
0.183219789 -0.607046596 0.477718668 -0.366207539
0.535681279 0.313609827 0.557606895 0.136012804 -
0.588809189 0.593265168 -0.300667578 0.551918991 0.501251049
0.07825582 -0.20939261 -0.493736856 0.096264289 -
0.588194726 -0.086958414 -0.085627563 -0.172085302 0.447540302
0.321712641 -0.091482135 -0.045696628 -0.106942287 -
0.009736581 -0.011601439 -0.147384269 -0.345830542 0.247656357
-0.059289959 -0.218597046 0.376045959 0.629671119
0.271940755 -0.498107308 0.480410304 -0.343337662 -
0.019486094 -0.334993714 0.460705168 0.597392609 0.419718498
-0.603105579 0.532853954 0.453827025 -0.049090711
0.679524924 -0.281619619 0.47515493 -0.635012991
0.440602555 0.286347303 0.47037922 0.398649617
0.171726143 0.503607681 0.265019132 0.342877246
0.635799313 -0.133795574 -0.584758887 -0.520396844

0.690848911 -0.54651302 0.376451807 0.23658299
0.661329657 -0.391543839 0.457701001 0.2897866 0.716735827
0.568880384 -0.383878239 0.492057763 -0.094514128 -
0.100651358 -0.287093596 -0.137391414 -0.322399541 0.219517375
0.518764108 -0.217391247 -0.064775304 0.650199486 -
0.552504907 0.254102064 -0.424829005 -0.22748178 -0.541997317
-0.288121169 -0.106516746 -0.533412902 -0.530612221 -
0.383174772 0.497018173 0.196070365 0.349525749 -0.145854912
-0.269603876 -0.241594594 -0.584312932 -0.185440384 -
0.559977367 0.084116679 0.145405004 -0.512536869 -0.342689429
-0.220988594 0.375978138 0.542633244 0.4374388 0.695735105
-0.456959879 0.573966789 -0.460537344 -0.328083547 0.5319646
-0.279691503 -0.153874187 -0.183593742 -0.359894717 -
0.479324633 0.33355712 -0.616976438 0.40843245 -0.691634805
0.43621095 -0.411085329 0.363653246 -0.007231297
0.083961871 0.011151663 -0.512239309 -0.14015373
0.402910847 0.526560746 0.200097821 0.177153963 -
0.382133114 0.159922464 -0.536098426 0.031233533 -0.619175172
-0.616207124 0.289742803 -0.141951405 -0.316491498 -
0.352766145 0.398167977 0.509174643 0.533757415 -0.285826729
0.367815257 0.176605683 0.564579431 -0.317904543 -
0.278880115 -0.046146822 0.580951275 0.477042589 -0.497793695

0.606220114 0.602290378 -0.242615931 0.416773837 -
0.15231457 -0.618747576 -0.1961686 -0.355789509 -0.510431302 -
0.079344655 0.517516374 0.155430752 -0.12727062 -0.521639977
0.081295004 0.328134677 -0.457761747 -0.38924333 -
0.07371412 -0.16770688 -0.299006331 -0.364086986 0.090669368
0.032710469 0.205684009 0.280493994 0.180691914 -
0.377940129 0.451150693 -0.173255805 0.698946884 -0.3032881 -
0.619940115 -0.176017392 0.443283937 -0.399017242 -0.084454054
0.535160499 -0.188727554 -0.634023744 0.148121596
0.463780735 0.109335051 0.07211796-0.554108013 0.158807251
0.311561966 -0.503236221 -0.646877875 -0.700914078 -
0.349344635 -0.658632209 0.523788118 -0.131609188 0.460571312
0.129002501 0.227056741 0.517580822 -0.505801274 0.0647036
0.173553718 -0.511918545 -0.635878517 0.433336105
0.641874246 -0.313296794 -0.230421391 -0.595085024 -
0.419960433 -0.308221013 0.465378551 0.594632627 0.528941595
-0.282058437 0.084185947 0.348088851 -0.213280692
0.510366004 -0.615805339 -0.208169668 -0.182780987 -
0.142816195 -0.146992193 -0.385541137 -0.555228493 -0.193399236
0.160569147 -0.246241717 0.405118127 0.055057172 -
0.456684236 -0.083547305 0.17903859 0.170008501 0.510982458
0.227930563 -0.336219634 -0.057583141 -0.492289594 -

0.683195788 -0.240652152 -0.287437239 -0.112581362 0.530460424
-0.150583499 -0.40981751 -0.398007087 0.660332641 -
0.265355632 0.734190973 0.052719065 -0.530263032 0.173223274
-0.038932684 -0.475693003 -0.085604776 0.636842616 -
0.166618234 0.397561688 0.037112981 0.105584087 -0.002504222
0.140030339 -0.395001683 -0.62216514 0.23789243 -
0.244960037 -0.498958959 0.2708518 0.340697899 -0.095983601 -
0.465554974 0.187295821 -0.458094023 -0.494185693 -0.334147052
-0.030822368 0.210791914 -0.043835275 -0.249171033 -
0.100389955 -0.613000022 0.308923652 -0.126632685 -0.501905849
-0.060211707 -0.415389868 -0.314617409 -0.242058939 -
0.328868166 -0.245312758 -0.02181527 0.043485121 0.469664356
0.209464238 0.171045453 0.248543212 -0.223175608 -
0.04973015 -0.232819702 0.453101705 0.122750247 -0.434042672
0.544810085 -0.126680837 -0.067564617 -0.228795764
0.130926423 0.268625427 0.459059981 -0.403070662 -
0.125798143 -0.156478729 0.44962889 0.085362747 -0.344193846
0.226133495 0.360656998 0.188057583
KEGG_MATURITY_ONSET_DIABETES_OF_THE_YOUNG 0.170325966
-0.145178555 -0.003257925 -0.373257765 0.409109226
0.108789464 -0.188973919 0.41708314 -0.256830819
0.32008585 -0.020612876 -0.134546467 0.450424166 -

0.452191994 0.240779165 0.253181692 -0.157330746 0.18545572
0.448665788 -0.393160446 -0.311419225 0.219235709 -
0.078139173 -0.175715445 0.209269831 -0.155764485 0.211336876
-0.209523617 0.071545157 0.256793662 0.506967292 -
0.059210372 -0.228858956 0.073778973 0.129171569 0.11843029
0.342509081 0.246650433 0.092933216 -0.370919091 -
0.130514096 0.24370747 -0.210431015 0.228240064 -0.050153043
-0.130060231 -0.319853216 0.035333113 -0.11854879
0.191914418 -0.016192401 0.09829282 0.10625225 -
0.158034191 -0.114926373 -0.042613183 0.357512599 0.047792197
-0.230795504 -0.301651698 -0.159312624 -0.192319774 -
0.172756791 -0.401871628 -0.198636565 -0.401826743 0.16772808
-0.244714945 0.502026852 0.456143747 0.000181568 -
0.276636761 -0.20084981 -0.29694521 0.145555845 0.367443975
0.37823929 -0.13393472 0.211206158 0.124515528
0.221709359 0.159370916 0.108165841 -0.021393021
0.385798992 0.164732563 -0.396328421 -0.151201451
0.000888092 0.023440121 -0.065341419 0.471034048 -
0.013100429 0.134453965 -0.249559052 -0.197857155 0.312767217
-0.046495588 0.172291096 0.324197684 -0.267329312
0.404242721 0.18580645 0.274433113 -0.048602618 -
0.085322279 0.06500219 0.140434535 -0.382573304 0.574352933

24848 0.591798952 0.26060187 0.066043706 -0.217711863

24849 0.111317602 -0.279103512 0.213378953 0.427622398

24850 0.563695273 -0.370704094 0.511063554 0.180569443 -

24851 0.485285943 -0.049031656 -0.182582862 0.63269923 0.102939441

24852 -0.2096239 0.039737756 -0.23872183 -0.08184931 0.163433289

24853 0.100733138 -0.246543941 0.375155305 -0.165892985 -

24854 0.151313839 0.202569737 -0.424832233 -0.494721807 -0.020805169

24855 0.303501966 -0.237556329 -0.315431943 -0.06889541 -

24856 0.327704398 0.028084463 0.134472848 0.489744834 0.332579239

24857 0.347222911 -0.169671094 -0.031774213 0.203301994

24858 0.019081925 0.437940639 -0.296625449 0.01286865 -

24859 0.115006950.375680019 -0.168993616 0.292748256 -0.219998972

24860 0.267689913 0.468869284 0.318203941 0.423405268

24861 0.17844131 0.542294735 0.013215038 -0.197377504

24862 0.467924005 0.318477256 0.201392962 -0.409741419 -

24863 0.337047562 -0.438022068 0.13593323 -0.245851541 -0.131231744

24864 -0.154234041 0.281265499 0.264976776 0.309636554

24865 0.320874201 0.328262256 -0.04947599 0.200873186

24866 0.193252175 0.119101975 0.050993098 0.384175506

24867 0.351847217 0.176061691 -0.033979419 0.411680695

24868 0.261877531 -0.063929619 0.24763013 -0.173172469

24869 0.145874346 -0.164607179 0.293404727 0.652128923

0.099064852 0.031436775 0.311754574 -0.117129296 -
0.054041309 -0.056078667 -0.042387358 -0.147773278 -0.298711258
-0.491812475 0.483262704 -0.556708584 -0.106941497
0.231510741 -0.156963485 -0.200424479 -0.005178607
0.007717062 0.604146812 -0.156122823 -0.057555937
0.471980516 0.382379431 0.29996719 -0.1222965 0.295982812
-0.002857897 0.205634493 -0.072405127 0.605320333
0.042324749 -0.156669827 0.210553702 -0.228680446 -
0.054492317 0.130609969 -0.46210119 -0.525787121 -0.149752485
-0.210465798 -0.177912413 -0.044868881 0.045942302 -
0.195431978 0.031935512 0.320889622 -0.237639725 -0.135241349
0.28700189 -0.525474134 0.365757604 0.197348613 -
0.050247097 0.48330193 -0.348408986 0.303774684 -0.146290541
0.017843129 -0.131927239 -0.139104421 -0.334140603 -
0.111280727 0.381813458 0.159612022 -0.107664233 0.508040223
0.358085052 -0.087891825 -0.22260567 -0.092323657 -
0.042555813 -0.188191428 -0.058231714 0.187904103 0.294161463
-0.145244432 0.043039349 0.125137728 0.106496788
0.607849715 0.263368858 0.336774689 0.445013593 -
0.063979668 0.110759409 0.048698909 0.118660249 0.180744304
-0.247195609 -0.236402214 0.424033542 0.523232748 -
0.038954691 -0.278350771 -0.155387964 0.312170066 0.006804317

0.033754598 0.362520555 0.16512194 0.237065992
0.237381249 -0.103080279 -0.007293061 -0.178149678
0.215862927 0.045394941 0.285773966 0.314962286 -
0.269417911 0.115249125 0.217881593 0.52803615 -0.084838888
-0.1433373 -0.306159616 0.354667997 -0.190421252 0.079107737
0.508008818 -0.067049535 0.259595716 -0.234781757
0.188547604 0.155932522 0.265374723 0.268911124
0.293473376 0.182188196 0.196705117 -0.264607539
0.069950459 -0.251701898 0.213158844 -0.391628373 -
0.154909696 0.162645363 0.019080976 0.206964809 -0.342852978
-0.306030805 -0.038474768 -0.237404252 -0.018448533
0.366648325 -0.191894379 -0.395470773 -0.06681422 -
0.461923011 0.456919894 -0.17264925 0.543417371 0.170759518
-0.217890009 0.126219636 0.413786274 0.188075621
0.09471402 0.151222479 0.290493036 0.105335235 -
0.209554187 0.069085602 0.072449838 0.030364689 -0.336401077
-0.145799284 -0.274490029 0.318566734 -0.124827691 -
0.278731535 0.17365909 0.269783627 0.114824831 0.022069078
-0.469357047 0.195611302 0.305883053 0.135633138
0.079919411 -0.434740964 0.443780059 0.322620728 -
0.135216994 -0.061370843 -0.138668548 0.250520749 -0.163599007
0.145486682 0.161175328 -8.65E-05 -0.375619906 0.113595653

0.086010989 -0.281407269 -0.07236203 0.048753666 -
0.510891596 -0.33112417 0.332294521 -0.232357205 0.275502211
0.389970953 0.116265354 0.147777983 -0.219836555
0.160535493 -0.442818907 0.108459815 -0.414050998
0.346451293 -0.231113456 0.123854272 -0.311035071 0.0485278
0.36968162 -0.12326117 0.335097696 0.196624436 -
0.188813017 0.523673044 -0.257327921 0.254975454 -0.111493666
0.208360009 -0.098594819 -0.028566624 0.076878831
0.568968911 0.392737633 0.308500705 -0.313126489 -
0.491619096 -0.244485054 -0.225375175 -0.281628182 -0.33423795
-0.467906766 -0.102882328 0.416582304 -0.419999155 -
0.276571652 0.059021233 0.177477848 -0.185642879 0.11206662-
0.341521194 0.343453601 0.399950482 0.616881262 0.350223643
-0.231723893 -0.25330431 -0.060237169 -0.247307724
0.407099697 0.482866337 -0.089268023 -0.011325752
0.388590472 -0.318409683 0.108933417 -0.333198752
0.257809018 -0.054178886 -0.08683771 0.045905806
0.01441382 0.244786864 -0.347372879 -0.186258464 -
0.078470443 0.100379334 0.291296817 -0.379364483 -0.241277724
-0.253968146 0.108899371 0.136476723 -0.008747477
0.240051794 0.04636087 -0.152487272 -0.19911867
0.001458652 -0.296591443 0.361154585 0.543788259 -

0.090828453 -0.033983683 0.021222775 0.232736393 0.460604833
0.285030503 -0.364943182 0.218624299 -0.110654755 -
0.118441399 0.277335218 0.072757415 -0.295679568 -0.08427138
0.097681598 0.0977911 0.522425899 -0.227768742 -0.233843647
0.308432371 -0.490644622 0.493844935 -0.437933791
0.132648148 -0.178911153 -0.368725475 -0.181746724
0.001052957 0.326932429 -0.277918858 0.043827078
0.225799666 -0.012986941 0.014656627 -0.32587707
0.108459569 0.254888259 -0.273093126 0.185612784 -
0.078754736 0.115628339 -0.341098213 0.520750945 0.377249677
0.096739215 0.410985379 0.27289795 0.127321707 -
0.07293889 0.183700053 0.295831848 -0.338652807 0.189380564
-0.042233468 -0.071214897 0.402411846 0.431054071 -
0.092299121 -0.200307174 -0.294997392 0.162742723 0.246061023
-0.10902028 0.130494427 -0.366002809 0.396031905
0.163554328 0.000801017 -0.057025895 -0.211762628
0.533131125 -0.002837494 0.217528697 0.140766776 -
0.150053743 -0.114975089 0.182855054
KEGG_ALDOSTERONE_REGULATED_SODIUM_REABSORPTION -
0.121242651 0.012405926 -0.185536648 0.091047681 -0.228506427
0.104150904 0.042399167 -0.190747203 -0.269879659
0.014628284 0.059091934 0.141203241 0.19693401

0.065720063 -0.181204665 -0.129135745 0.024586108
0.197155065 0.037090973 -0.305179596 0.004863418
0.130414617 -0.072708854 0.080548828 -0.067723906
0.151049797 -0.079073999 0.117315828 -0.017843728
0.199506148 -0.142147477 0.018821814 0.145065073 -
0.116038860.250314791 8.44E-05 -0.140490827 -0.041218797 -
0.11017561-0.300298875 0.378255118 0.138090383 0.02550439
0.224612169 0.070582484 0.053700662 -0.14664915
0.329422889 0.050432471 0.100743855 0.319209819
0.097399891 0.034245142 -0.244986375 0.187103773
0.142670442 0.026186929 0.112389117 0.060551028
0.334372531 -0.253755324 -0.16367404 -0.100851879 -
0.351705013 0.063853424 -0.07655332 -0.165369309 0.247985177
0.310681789 -0.071800824 -0.05991824 -0.064388698
0.514937252 -0.109451078 -0.174183013 0.251789969 -
0.083269409 0.055381442 0.036857246 -0.175816631 -0.021123479
0.087885261 -0.067222309 0.229648665 0.032381033 -
0.16958464 0.130727638 -0.011663798 -0.012227285 -0.050347359
-0.194349262 0.303663554 -0.16358567 0.019842418 -
0.294771496 0.068250437 0.396919024 0.214922256 0.214536074
-0.112984317 -0.278811583 0.095584986 0.012920762
0.116480121 0.289168726 -0.055568086 0.070807445 -

24980 0.139680391 -0.057811558 0.271261133 0.196440124 0.07326044

24981 0.095502698 0.223789863 0.014548414 -0.022168581 -

24982 0.126077162 -0.091182432 -0.003678944 -0.213155159 -0.071780666

24983 0.128591855 -0.225744952 0.333260792 -0.02686048

24984 0.142883044 0.137574053 -0.091677045 -0.214010381 -

24985 0.00322169 0.212769644 -0.233598516 -0.221399633 -0.121169173

24986 0.006198792 -0.324837733 -0.220518118 -0.024711497

24987 0.017510725 -0.004751123 0.126374156 0.145031209 -

24988 0.312449899 -0.08110986 5.71E-06 0.236375728 0.080236843 -

24989 0.139816037 0.051953043 -0.143596622 0.112700468 -0.128575659

24990 0.107300617 0.266084325 -0.169257305 0.266679057 -

24991 0.342297509 0.063150022 0.010750036 0.040023697 0.040858706

24992 -0.143424284 -0.253245502 -0.003902661 0.175576232

24993 0.205558102 0.293071345 0.187944969 0.21616251 -

24994 0.013496312 -0.152373185 -0.003992661 -0.133072423 0.198367388

24995 0.046857971 0.156907705 0.11488528-0.33129607 -0.258841427

24996 0.055680819 0.001438645 0.442058356 -0.122476338

24997 0.116059871 -0.08415539 0.135508381 -0.157264185

24998 0.060468484 0.134690014 0.34034212 -0.433953366

24999 0.035996574 -0.081960458 -0.134666332 -0.365622133

25000 0.314690968 -0.027092541 -7.73E-05 -0.110679931 0.108559509

25001 -0.059046733 0.022734871 0.297174141 0.338852367 -

0.008444697 -0.281610216 0.063986101 -0.185754819 -0.025923392
-0.392901433 0.080536355 -0.267928031 0.000132113 -
0.113853106 0.167491508 -0.354163525 -0.141189817 0.273761031
-0.120069964 0.029904575 0.158992434 0.148337431
0.098927986 -0.275002846 -0.029061757 0.354565911
0.058225593 -0.09199368 0.14149485 0.141394927 -
0.22791037 0.096367116 -0.136249775 0.383390436 0.05167075
0.375289868 0.095538737 -0.245362761 0.315835261 -
0.226397509 -0.011824573 -0.216247365 -0.139821539 0.340441332
-0.448713059 0.241787946 0.078198457 0.071184928 -
0.181686701 -0.154670104 -0.069651672 0.389594586 -0.10230827
-0.228157748 0.215218264 0.267975366 0.24932681
0.086115742 0.160751463 -0.077125121 -0.154112109
0.214480335 0.024654145 0.143104523 0.031705206 -
0.057136845 0.284319518 0.243399853 -0.115267134 0.327656578
-0.221862238 -0.368082785 0.067782308 0.147504463 -
0.257160969 -0.16625836 -0.099384672 0.168045608 -0.28344446
-0.132184123 -0.048058439 -0.045005707 -0.232721357
0.271102752 0.103505525 -0.142353782 -0.015149218 -
0.212446747 0.003175748 -0.124786102 -0.314171152 0.088241717
-0.21419388 0.140654599 0.203344942 0.000797772 -
0.165373362 -0.027766684 -0.260084171 -0.052618356 -0.23111852

25024 -0.198791214 -0.194598303 -0.025928502 -0.312261254

25025 0.215857663 0.073511403 0.045930534 -0.12483032 -

25026 0.132960751 -0.070712717 -0.312233524 0.228938412 0.130359567

25027 -0.028379641 -0.190031035 -0.080998165 -0.218633557 -

25028 0.331328339 -0.015669466 0.426331209 0.379007034 0.307379101

25029 0.334748797 -0.185616047 0.422468984 0.001083562 -

25030 0.038581594 0.169949937 0.029613343 -0.254855822 -0.037584307

25031 0.077873432 0.293153335 0.096538005 0.059653115 -

25032 0.103351232 -0.119645629 0.000776776 -0.001518936 0.02365916

25033 -0.022524334 0.038062652 0.08241934 -0.015624177

25034 0.119891879 -0.031137704 0.103626722 0.249255188 -

25035 0.24190722 -0.394970073 -0.090921774 -0.222656866 -0.240660299

25036 -0.001158402 -0.095978969 0.084908942 -0.112374997 -

25037 0.382599783 0.25075831 -0.31420119 0.444849346 -0.099218205

25038 0.200325866 -0.017769052 0.229389384 -0.396946764

25039 0.084793801 -0.173339645 -0.06597956 -0.000442676

25040 0.255295416 -0.127154003 0.135700243 -0.208192629 -

25041 0.173300938 0.064509689 -0.157923404 -0.090443958 -0.1743885 -

25042 0.226732419 -0.141312632 -0.275460357 -0.248050761 -0.261614088

25043 0.03269273 0.167592502 0.062992469 -0.017375802 -

25044 0.035914777 -0.071138019 -0.139691336 -0.062313094 0.183299505

25045 0.288138399 0.393809404 -0.10157268 -0.304997193

0.080824077 0.196987646 -0.057218165 0.23660538
0.220800691 -0.066101497 0.058553906 -0.130777625
0.035856433 -0.006936952 -0.09697247 -0.030808897 -
0.18633609 -0.009054947 -0.221496477 -0.264017323 -0.187574322
-0.188029995 -0.032356792 -0.245510769 0.019484659
0.111441565 -0.120270757 0.172004468 0.209903503 -
0.022222381 0.345323007 -0.096161173 -0.13756605 0.181897281
0.083863616 -0.260331259 -0.097896461 0.237904047 -
0.005545261 -0.008623689 0.081327876 0.165724985 0.144764963
0.35214299 -0.480168706 0.205261627 -0.049534242
0.215839006 -0.047811081 0.121776546 -0.30212192 -
0.131731907 0.46990178 -0.107144199 0.057160196 -0.030242865
-0.352698738 -0.095490403 -0.148094446 0.315837968 -
0.045521292 -0.185843131 -0.071606514 0.173965983 -0.043221041
-0.010763451 0.22730282 -0.055894639 0.086426619
0.012217373 -0.192921869 0.144314871 0.025489023
0.162708265 0.288662391 -0.199949342 -0.220002197
0.045859183 -0.029419314 -0.167843774 -0.271945147 -
0.273070356 0.323712097 0.146219739 -0.020739849 -0.082745445
-0.176820439 0.118179748 -0.027425215 0.505561587 -
0.01895389 -0.104037162 -0.082161649 -0.104402576 -0.053810179
0.15294448 0.360659994 -0.069094432 -0.107987781 -

0.209326272 0.103764201 -0.198200722 0.074208688 0.191923143
-0.330122056 0.001803856 0.162617647 0.299852445 -
0.02103622 -0.004510702 0.383788226 0.278005321 0.077840616
0.235908913 0.167278126 -0.287483091 0.167098386 -
0.152082206 0.154971594 -0.070041256 0.142345764 0.249615957
-0.003750174 -0.335051023 0.123840903 -0.018451193 -
0.099726573 0.055592776 0.167657859 -0.072893782 -0.286361002
-0.393335275 0.107394904 -0.0848216 0.1631695 -0.003814741
0.128351272 0.010106487 -0.075545171 -0.123534839
0.005069779 0.392030403 0.083449689 0.086397305 -
0.27039634 0.046626362 0.041909263 0.017732483 -0.041221431
-0.019103828 -0.101427754 -0.106955387 -0.278949602 -
0.019001074 0.196768353 -0.131458985 -0.058781587 0.052979574
-0.071474483 0.112872182 -0.271284023 0.002874756
0.311579684 -0.022793864 0.085412482 -0.049921358
0.102166112 0.188191263 0.19218115-0.032354186 0.012235159
0.112659929 0.022764897 0.0239041 -0.069417586
KEGG_VASOPRESSIN_REGULATED_WATER_REABSORPTION
0.317018564 0.382470378 -0.053131638 0.209457495 -
0.387138579 -0.134644888 0.130163797 -0.414612644 -0.280371343
-0.485787542 -0.340341396 -0.003097977 0.080971912
0.001421708 -0.297211287 -0.413133033 0.530400233

0.151593783 -0.340309491 -0.216459077 0.401363859

0.069015319 -0.015389764 0.302150905 -0.315969308 -

0.118575432 -0.238136337 0.022227015 -0.130007201 -0.398012974

-0.005550747 -0.113346226 -0.068865235 -0.510367692

0.073849755 -0.124953295 0.325923533 -0.239832835

0.169722742 0.108746184 -0.103366541 0.015679211

0.170675691 0.280113952 -0.218567387 0.140856356

0.210954576 -0.209026518 -0.11869607 -0.13227999 -

0.223381988 0.201904127 0.223377943 0.169228518 0.491063364

0.311294731 -0.433835135 0.335106594 0.338747353

0.497767932 -0.004302489 0.189781362 0.349715594

0.279007499 0.133917688 -0.247071763 -0.216407745

0.365833575 -0.273028358 -0.498960743 0.061643192 -

0.114031836 0.045745209 -0.257216655 0.136253139 -0.393802909

-0.300165222 -0.196175645 -0.358979272 0.439216115 -

0.233388503 0.317103441 -0.083577271 0.186153294 -0.325167968

-0.28789096 0.287386458 -0.199637316 -0.438868695

0.119438848 -0.140192354 -0.146247756 0.174076333

0.347331644 0.112081705 0.149233265 0.042330815 -

0.322025579 0.22945466 -0.097695806 -0.101083949 -0.422734673

0.047311932 -0.04424381 -0.237369551 -0.336373812

0.310253332 0.030305593 0.244446286 -0.0010757 -0.012380923

-0.217619271 0.003286609 0.163347198 0.128798265
0.212449902 -0.048051387 -0.449075949 -0.389220717
0.248388549 -0.350506984 0.080553517 -0.049545846 -
0.196529988 -0.063762059 -0.235539735 -0.134870103 -0.179642985
0.326880552 0.225967651 0.083270935 -0.346073032 -
0.008411553 0.163897584 0.254281555 0.145753073 0.222211015
0.471884783 0.13212033 0.320661596 -0.210646507 -
0.152771818 0.240329378 -0.063576479 0.249562211 0.287880672
0.19293001 -0.199978981 -0.40255202 -0.027829771 -
0.24875163 0.003937593 0.047671269 -0.103599365 0.093387335
-0.046062815 0.02778926 -0.275735309 0.014452323 -
0.318909734 0.337018329 -0.053456298 -0.30108092 -0.331073397
0.353266003 0.013502621 0.377693012 0.349767733 -
0.408549144 -0.227024674 0.368847684 -0.173060098 0.031743459
0.350218881 0.336257497 0.332928661 0.150799443
0.258656581 0.077585954 -0.147173923 -0.032515197 -
0.08249392 -0.353233962 0.104185110.501343824 0.279316333
0.039065666 0.118146811 -0.297223328 0.309623375 -
0.057742215 -0.179042943 0.058395828 -0.212083715 -0.173567701
0.075318217 0.086041039 0.144985921 -0.216773492
0.196155675 0.035758116 0.168811901 0.37988393 -
0.376698034 0.089589447 -0.230536956 -0.228194998 -0.226253779

25134 0.266134269 0.081264097 0.306369348 -0.16909537

25135 0.246215111 0.170950957 -0.256689491 0.163845415

25136 0.212055947 -0.099117609 -0.005741778 0.031781436 -

25137 0.260245799 0.404408635 -0.059021778 -0.091754161 -0.095267026

25138 0.117577352 -0.15272141 -0.353344708 0.217728906 -

25139 0.054441262 -0.185480762 0.005569079 -0.122232544 -0.136311653

25140 -0.227795009 0.353315217 -0.417039844 0.329065829 -

25141 0.237299736 -0.140124321 0.129069743 0.069411641 0.016333853

25142 0.310947293 -0.295578927 0.437064218 -0.137869972 -

25143 0.063828792 -0.357948189 -0.430452954 -0.003205831 0.263010166

25144 -0.39997548 0.011374536 0.064075568 0.106253545

25145 0.439717484 -0.264384798 0.299028642 0.059683716 -

25146 0.305380801 0.169156368 -0.165307388 -0.201534493 0.106551576

25147 0.334992281 0.076867751 -0.008776653 -0.006367155 -

25148 0.279761352 -0.180760244 -0.120715513 0.3068336 0.04606958 -

25149 0.024632845 0.068297143 0.237908289 -0.28767042 -0.047056536

25150 -0.297388896 -0.42268221 -0.269831634 -0.224591892 -

25151 0.122764968 -0.561581049 -0.213764541 -0.505660185 0.096263358

25152 0.006683385 0.060035392 -0.17092731 -0.126138469

25153 0.264102188 0.367082921 -0.394723365 -0.421725728

25154 0.381391114 0.064111556 -0.159273336 -0.32546132

25155 0.008752462 0.250419299 -0.257686702 -0.299436935

0.063317502 0.133197391 -0.059765151 0.384161358
0.069594926 -0.444987786 -0.212682383 -0.282042959 -
0.208858964 0.352292444 0.014348158 0.215968051 -0.217897981
-0.072978711 0.254254491 0.334268751 -0.216405684
0.237371442 0.384856679 -0.15755093 -0.24974516
0.15379703 0.049457846 -0.311175299 0.316862977 -
0.077168615 -0.199930559 0.472756503 -0.165312819 -0.025870034
0.454573986 0.454794382 0.36547575 -0.168639114 -
0.200286688 0.305757445 0.159479797 0.21910436 -0.410847063
0.335127651 0.403887535 -0.138163811 0.07734608
0.272690837 0.171581925 -0.102020964 0.15535352 -
0.213984375 0.327081582 -0.071678338 0.409284856 -0.293538989
-0.274600547 0.266860112 -0.096045076 -0.242099199
0.132860079 -0.144974051 0.322357085 0.092203219
0.263301145 0.080498466 -0.091110473 0.591900253 -
0.147804549 0.255534644 -0.270181439 0.415768803 0.469262967
0.322239342 0.242437258 -0.361877497 -0.362610725 -
0.363575101 -0.209698486 -0.07200349 -0.381332586 -0.296145066
-0.201844201 -0.363762687 0.327136535 0.374446224
0.022306427 0.198545871 0.276360106 0.306315848
0.193704222 0.011257124 -0.33063187 -0.170141723
0.397535547 0.382567869 0.284074621 -0.059802808

0.327900365 0.453138991 -0.353685778 0.441011595
0.369238067 -0.125516886 -0.142385755 -0.29223594 -
0.188573632 0.332475284 -0.007150397 0.139719964 0.18063885
0.223102275 -0.312198909 -0.129388423 -0.329633419 -
0.241702524 -0.055640571 0.174633233 0.036655544 0.031432969
-0.22079215 0.316927039 0.19325961 0.218989782 -
0.023305622 0.089947995 -0.221634221 0.203128929 -0.304881085
0.295760839 -0.137766738 0.403481349 0.034451126 -
0.30568687 -0.237818903 -0.173986807 0.376214391 0.030108198
-0.188933822 0.29251513 0.026339591 0.161054947 -
0.32475568 -0.268512786 -0.360112536 0.094150943 -0.025118919
-0.502773816 -0.180790057 -0.016758455 -0.084172217 -
0.211598058 0.024343149 0.023256654 -0.152337817 -0.21320914
0.260154037 0.042769517 0.260817487 -0.00090027
0.385989266 -0.308457532 -0.466981796 0.24004728
0.332499844 -0.180341519 0.031742299 -0.282795125
0.388645012 -0.11197285 -0.271507459 0.035968865
0.208200339 0.319586164 -0.3308225 -0.086094533 0.119116334
0.194828313 -0.186244451 0.160501202 0.387170036 -
0.165287356 -0.071010189 -0.173833845 -0.058290975 0.100774758
0.378668988 0.053962322 -0.043062201 0.280930269 -
0.318508175 -0.08573982 0.134350611 -0.018219747 0.139327532

-0.317187449 -0.080321153 0.047207245 -0.056727366 -
0.267142386 -0.01066687 0.403884496 0.351974274 0.425600133
0.312251026 0.354125277 -0.222972829 0.323950308 -
0.266528601 0.091749144 -0.133392947 -0.099116218 0.011492004
-0.337550284 0.335343389 0.172137417 0.28215445
0.005579826 -0.038609401 -0.119802354 -0.004134261 -
0.098455459 0.332807591 -0.177322803 0.219249549 -0.026516783
0.258533953 -0.071803228 0.512829575 0.519284673 -
0.172818646 -0.249702169 -0.075176348 0.140472852 -0.061165942
0.102938197 -0.052437666 0.176143355 -0.273978948 -
0.157287091 -0.120657102 0.16661537 -0.18372791 0.21640549
-0.23366566 -0.086783649 -0.06842898 -0.051591864 -
0.02817969 0.044723233 -0.143112746 -0.046272656 0.101087937
0.044553348 0.178114909 -0.121935515 0.3175119 -0.368799175
-0.214767062 -0.203424657 0.188893508 -0.111518895
0.05045664 -0.057603617 -0.115374602 -0.153198872 -
0.191160045 -0.195650888 0.126869046
KEGG_ALZHEIMERS_DISEASE 0.290768567 0.226922171 -
0.264698052 -0.39128999 -0.15504347 -0.296923285 0.091877755
-0.108651298 -0.267626992 -0.242898216 -0.380669794
0.354260194 -0.252270213 -0.077125604 0.16105678
0.325807511 0.225137467 0.260436015 -0.416758639

0.167180815 -0.278675357 0.25896571 -0.084600306 -
0.205081532 0.132270651 -0.262811008 0.304832724 0.26670331
-0.358581992 -0.19944595 0.153440228 -0.373607702 -
0.246287763 0.117648572 -0.13614705 -0.367522088 0.07434944
0.383509957 -0.285031526 -0.302117788 0.39422957 -
0.049425554 -0.365889427 0.247433594 0.15808371 0.331074345
-0.05477269 0.226996237 -0.210186207 -0.068305572
0.463206207 -0.309784763 0.263323708 -0.381350849
0.269340118 0.154966405 -0.245905516 0.079617661 0.2221346
-0.15777588 0.008617916 -0.406348171 0.180810493 -
0.151889172 -0.311741485 -0.451843554 -0.201303948 0.142417666
-0.078566001 -0.079512124 -0.096632102 -0.325579424
0.229479201 0.097326085 -0.237869582 -0.018944089
0.156148219 -0.15753112 0.189213507 0.398142759 -
0.220606604 -0.345124313 0.3140634 0.211798141 0.214434742 -
0.196855742 -0.031249605 -0.304197878 -0.422257544 -0.129434088
0.030872898 0.275197358 0.205538248 0.382245127
0.190053083 -0.384518077 0.268215029 -0.440807868
0.288914187 0.274707318 0.053431325 -0.366489264 -
0.171876733 0.444734644 -0.077982557 -0.383769351 -0.080705042
0.420156411 -0.43354003 0.031493524 -0.23117702
0.275072909 0.392613574 0.096379974 -0.413010215 -

0.115239590.313003514 -0.081296173 -0.384867192 0.189204892 -
0.15336269 0.182036373 0.275070505 0.269926474 -0.448334706
0.026909597 0.469637145 -0.418907737 0.10337253
0.097304805 0.225098691 -0.342899233 0.10641548 -
0.302690607 0.223941275 -0.382886665 -0.153833824 0.167571853
-0.192691737 -0.398268336 -0.112170589 0.257624117 -
0.223629229 -0.168050203 0.100480867 0.185974721 -0.046330267
0.118434025 -0.349586471 0.308451568 0.140750409 -
0.158083913 -0.349438375 0.008384048 -0.326740582 -0.033033204
-0.332634278 0.240309288 -0.214683611 -0.271813892
0.066310281 0.016488603 -0.080391792 -0.340691665
0.462067615 -0.359283393 0.227087067 -0.29151941 -
0.305734154 -0.475780966 0.149543106 0.10697562 0.094995719
0.365660349 -0.184178397 0.103541797 -0.473724846 -
0.072318961 0.208486954 -0.201695112 0.320912896 0.301307844
-0.220490159 0.342124965 -0.154317872 0.087821715 -
0.094295747 -0.23743909 -0.279696887 -0.150406364 0.15004358
0.544532995 -0.273499816 -0.152012893 0.395842261
0.230287414 -0.418901815 0.129979052 -0.367399702 -
0.167027683 0.383452421 -0.029157466 0.253588794 0.376323834
-0.088890694 -0.164275675 -0.322325133 0.170435431 -
0.182403096 0.07272743 0.156835229 -0.258650354 -0.111964216

-0.047828674 0.048654685 0.161490799 0.329982421 -
0.400102531 -0.365358397 -0.415847577 -0.419040441 0.318769692
0.066168013 0.087126045 -0.407205689 -0.051633812
0.388320054 0.10204965 -0.115723555 0.443615022
0.332763704 -0.080471363 0.055513295 -0.288023068 -
0.427875656 -0.183221991 -0.363572973 -0.289767253 -0.235771192
0.356060187 0.199029085 -0.261753931 -0.015591471 -
0.097959097 -0.206877085 -0.077074683 -0.26220772 -0.208777637
-0.153215722 -0.294139576 -0.207461126 0.137198962 -
0.013710908 -0.282140253 0.218808092 -0.352645826 0.199692226
0.182190921 0.201943267 -0.160461571 -0.196583381
0.414072508 -0.192160581 -0.073057481 0.311092456 -
0.251691435 0.09068582 0.396235334 -0.007524693 -0.137020986
0.372769604 -0.144822076 0.241081342 0.223716396
0.368637795 0.320373597 0.131234619 0.270787426 -
0.30387788 -0.089326729 -0.35245903 0.337681861 -0.147914189
-0.161676855 -0.374070489 0.429504944 -0.43450169 -
0.233293065 -0.137136499 -0.257708072 -0.272509775 0.30796144
-0.155845582 -0.304473443 -0.349705895 -0.240769611 -
0.236092813 -0.455769742 -0.051733978 -0.36242545 -0.364559527
0.224361844 -0.141265562 0.107307117 0.407382604 -
0.263694155 -0.351237412 -0.021976689 -0.261279665 -0.135585265

-0.374654573 0.106655821 -0.300142763 -0.187975623
0.372027814 0.353962498 -0.023485004 -0.181041952
0.212491364 0.101686178 0.361312327 0.444697606
0.21604181 0.044079961 0.276811208 0.173538246
0.213426964 0.193088384 -0.088881166 -0.181884419 -
0.410023564 0.070848365 -0.26447326 0.401722863 -0.209424997
0.095996272 -0.011127414 -0.102123871 -0.38565222 -
0.119874846 0.41808371 0.045323475 -0.334446837 -0.189440363
-0.031367132 0.153518613 -0.408639757 0.116445161 -
0.175684048 -0.312827197 -0.334679702 -0.145384721 -0.189038414
0.313364006 0.110278278 -0.106258371 0.163760252 -
0.101203071 0.248223738 0.48558765 0.246706146 0.381324755
-0.071312911 -0.319058724 0.139330331 -0.461758301
0.191745382 -0.018368085 0.158527814 -0.368659181 -
0.336894295 -0.045047604 -0.048778668 -0.148261983 -0.043176008
-0.003759528 0.100399206 0.283548708 -0.375820138 -
0.341940642 0.2170061 -0.032581073 0.041901499 -0.089917343
0.023518063 0.269375397 -0.194096456 -0.172337249
0.013238606 -0.121057775 0.167243608 0.35868075 -
0.227674826 0.259487836 -0.115568613 -0.165411843 0.228243343
0.206094531 -0.446311268 -0.05252036 -0.454245116 -
0.185159187 -0.110512529 -0.147134934 0.240884746 -0.196286609

-0.424347444 -0.227156187 0.244877492 -0.331495886
0.358141492 -0.404587665 0.082002155 -0.269341695
0.267501987 -0.220249896 -0.179916661 0.440811444 -
0.092105864 0.363966844 -0.384473265 0.198856869 0.35534827
-0.000161941 0.118989168 0.277793816 0.15211343-0.132949116
0.071851838 -0.032555912 -0.027068454 0.321805608
0.017804555 -0.136522005 -0.051028256 -0.337578213
0.140877019 0.444020153 -0.080393336 0.155164762 -
0.363190338 -0.023300439 -0.283095153 -0.149347912 -0.075622188
-0.322429607 0.416117591 -0.241596659 0.116959295 -
0.140616464 -0.226950834 -0.276059959 -0.356240226 0.399087898
0.469733992 -0.315670519 -0.296895856 0.136826505 -
0.132583177 -0.051282744 0.361551955 -0.386805471 -0.127828422
0.174885131 -0.117961184 0.22518727 -0.041528185 -
0.181868067 -0.106402399 -0.113160051 -0.226739808 0.304644657
0.007609102 -0.471843545 -0.118308944 0.171616806
0.195346479 0.215603064 -0.435754762 -0.303740858 -
0.038381499 0.26930309 0.060997641 0.351544938 0.485457398
-0.025779409 -0.143080354 0.29897461 -0.172175959
0.104120664 0.326944681 -0.431887431 -0.19744957 -
0.40043002 -0.086694422 -0.197589458 0.278744914 0.320149239
0.233964211 0.196856405 0.157365786 0.277299215

0.353634142 -0.178756169 -0.064008932 -0.026281927
0.067632814 0.033130832 0.007855191 -0.349836703 -
0.391859773 0.246119269 -0.381500929 -0.131379439 -0.013061432
0.433061143 0.169083325 -0.024594252 -0.124037559
0.138301373 0.288358326 -0.118113381 0.220861167
0.372922287 0.292680298 0.010019606 0.212651252
0.308295282 -0.103340658 -0.125284223 0.02702282
0.186634834 0.104038988 -0.283259322 0.282135131 -
0.409016466 -0.101038104 0.07677108 -0.127228519 -0.207726977
-0.020944578 -0.086292679 0.182618764 -0.203394488 -
0.026964666 -0.069450843 0.201818802 0.20797721 -0.321573943
0.129848079 0.064522963 -0.127071204 0.201400126 -
0.262940088 0.318593407 -0.12724481 0.164081952 -0.237342324
-0.05711011 -0.288535662 0.040128995 -0.065560874
0.050119412 -0.06975789 -0.042082476 0.155337059
0.028279068 0.066796505 -0.310847804
KEGG_PARKINSONS_DISEASE 0.466557824 0.299072127 -
0.291016097 -0.540288085 -0.212563445 -0.373197873 0.174741092
-0.014763557 -0.481043004 -0.487612174 -0.499258497
0.531189791 -0.308429136 -0.057300218 0.306003467
0.392455797 0.330531617 0.23094425 -0.587363886
0.206686882 -0.448167342 0.258559615 -0.191547315 -

0.331793471 0.060402335 -0.218797032 0.559071203 0.244252883
-0.496311614 -0.350619985 0.347184251 -0.598569374 -
0.323211206 0.324352824 -0.314565623 -0.599640096 0.264270498
0.471319312 -0.339914988 -0.299297332 0.517804501 -
0.059202968 -0.526332339 0.44145703 0.23689 0.402739169 -
0.006421058 0.389389071 -0.347040695 0.046190026 0.659379919
-0.301493164 0.396688686 -0.592014086 0.29349381
0.300883565 -0.407388513 0.114947377 0.369587024 -
0.376676111 -0.057370286 -0.576828133 0.32594521 -0.259106839
-0.383245824 -0.606422081 -0.264658709 0.133513341 -
0.166249112 0.009009111 -0.172194471 -0.479660246 0.445196892
-0.008735529 -0.39720018 -0.095960432 0.251854862 -
0.210424342 0.364759278 0.586873543 -0.289513966 -0.532022294
0.46596573 0.285801966 0.099062451 -0.327786578 -
0.083687491 -0.375984224 -0.592216151 -0.143524328 -0.098833032
0.433748832 0.271780207 0.482065268 0.225452404 -
0.541062172 0.412577652 -0.592042931 0.526083136 0.391788943
0.042529888 -0.441381285 -0.25495149 0.603835853 -
0.312326644 -0.518226986 -0.092812952 0.602941496 -0.583870892
0.0490763 -0.280845673 0.39958154 0.537713958 0.07988744
-0.562420052 -0.210728663 0.600170931 0.082301935 -
0.418349209 0.30776563 -0.06981448 0.304452138 0.401256049

0.506130437 -0.643612351 0.232433423 0.689169772 -
0.577647175 0.209712202 -0.265213952 0.282523997 -0.392688371
0.29332858 -0.384545195 0.420943038 -0.562384919 -
0.32006046 0.26129885 -0.356254647 -0.531419941 -0.150117582
0.412880503 -0.255612019 -0.2424944 0.02669763 0.35735213
-0.13362312 0.141950838 -0.437058721 0.427840621
0.193598934 -0.419513349 -0.545906868 0.002650398 -
0.407148064 0.06583532 -0.518635439 0.364131341 -0.3669583 -
0.48687818 0.175596441 -0.057040093 -0.041535279 -0.440047971
0.634576455 -0.544549072 0.329275542 -0.489306476 -
0.323348601 -0.611862197 0.224167029 0.33880829 0.316776819
0.476746906 -0.448052388 0.116037931 -0.554177037 -
0.145924677 0.360117864 -0.385124654 0.423587148 0.462851883
-0.197808922 0.392079164 -0.270584126 -0.225715954 -
0.008475917 -0.338967846 -0.399709589 -0.392152536 0.217666343
0.722856809 -0.337226913 -0.184003845 0.634279289
0.368271114 -0.512558636 0.226980182 -0.330713509 -
0.26618057 0.464352385 -0.140654688 0.473663912 0.533422017
-0.328564319 -0.209580536 -0.492475553 0.291289514 -
0.357217993 0.298282679 0.305818656 -0.335614091 -0.2491781 -
0.084877978 0.219624998 0.254564242 0.500187615 -0.530509978
-0.431718907 -0.565766448 -0.562345051 0.429504034

25398 0.106281517 0.104585163 -0.520398701 0.028160331

25399 0.455333693 0.235911625 -0.359806043 0.573320295

25400 0.460668664 -0.271539166 0.224564311 -0.530953826 -

25401 0.566014095 -0.355099822 -0.492670157 -0.433361384 -0.350528874

25402 0.507329538 0.250465936 -0.302422623 0.151235696 -

25403 0.01280003 -0.258892232 -0.220851467 -0.397516757 -0.23987666

25404 -0.118273379 -0.14931203 -0.254893942 0.121171032 -

25405 0.09934721 -0.441915608 0.377000192 -0.472517135 0.346513808

25406 0.316012586 0.156994439 -0.136003944 -0.311355052

25407 0.516962788 -0.24779915 -0.187402931 0.419211935 -

25408 0.383210927 0.115034093 0.538458265 0.10184682 -0.088935094

25409 0.601879701 -0.164250657 0.315746579 0.279830116

25410 0.604483618 0.452577292 0.127668106 0.30386053 -

25411 0.327717995 -0.05389539 -0.511556239 0.420577301 -0.33114523

25412 -0.270115952 -0.576550832 0.603904092 -0.571491282 -

25413 0.327550867 -0.383770016 -0.313090965 -0.268804504 0.342996419

25414 -0.135576603 -0.550101314 -0.479298224 -0.022305159 -

25415 0.272421819 -0.556224178 -0.092085711 -0.371094412 -0.433876123

25416 0.341120661 -0.084224232 0.03240194 0.581673169 -

25417 0.384001276 -0.56692477 -0.17533814 -0.399573123 -0.079693371

25418 -0.570189122 0.164608144 -0.364178687 -0.375224035

25419 0.351192176 0.447908833 -0.173838565 -0.247471582

0.282083342 0.236190122 0.557529371 0.597342489
0.266864962 0.100362455 0.360446071 0.044294344
0.456423455 0.437457542 -0.017979287 -0.378716636 -
0.572715573 0.189215591 -0.460671482 0.482717775 -0.351224051
0.20164246 -0.150196241 -0.345733867 -0.522228193 -
0.178899216 0.593267405 0.026365885 -0.354749653 -0.320271446
0.138430545 0.324562933 -0.592418474 0.066149372 -
0.297819364 -0.478154304 -0.512677066 -0.234725434 -0.370471089
0.508757097 0.227380352 0.06660493 0.226340173 -
0.00316668 0.47633847 0.718220559 0.416807946 0.531388623
-0.089374451 -0.516920048 0.187717562 -0.664268831
0.336650387 -0.145972263 0.332058848 -0.614525244 -
0.547569461 -0.25096222 -0.305189104 -0.251422752 0.058185924
-0.106107606 -0.012411975 0.481537253 -0.517151647 -
0.463952003 0.330455917 -0.206022736 0.21777694 0.048262528
-0.145219224 0.384101851 -0.350718136 -0.414761308 -
0.188485745 -0.377218079 0.3141276 0.379084873 -0.404017063
0.308018112 -0.346411808 -0.314636791 0.33947216
0.260126956 -0.551758486 -0.267661343 -0.550358743 -
0.435190113 -0.254817029 -0.122658523 0.072556891 -0.283714934
-0.475773861 -0.317454353 0.280427357 -0.423886652
0.602527488 -0.544462842 0.303862039 -0.36580134

0.371605679 -0.294654896 -0.394454728 0.568986639 -
0.255725015 0.451207485 -0.502960745 0.319983383 0.573740431
-0.290676321 0.100744446 0.431729653 0.373929743 -
0.438506975 0.003611977 -0.055123595 -0.01502491 0.424326583
-0.146128007 0.020523039 -0.01809957 -0.50650813
0.109908354 0.65653942 -0.070979002 0.083306318 -
0.452091026 -0.13325373 -0.459117215 -0.291094922 -0.224517158
-0.466875209 0.552948668 -0.397904304 0.2106574 -0.371002276
-0.481510544 -0.345313018 -0.350677376 0.516078529
0.598693354 -0.37668292 -0.403623521 0.193031383 -
0.216485874 -0.195870172 0.460005804 -0.51786819 -0.185582141
0.203554653 -0.210011583 0.207842726 -0.21145192 -
0.237355548 -0.201917016 -0.060208308 -0.15680951 0.328162982
-0.00800435 -0.581234477 -0.135176903 0.318757477
0.284653919 0.422896438 -0.505279666 -0.494115298 -
0.137637114 0.324057166 0.149921736 0.483866818 0.688066031
0.044360393 -0.265257149 0.340773671 -0.302415603
0.049104993 0.497991468 -0.521891648 -0.483661908 -
0.551842482 -0.115790431 -0.180302041 0.362376992 0.419902035
0.46278288 0.433686209 -0.239000544 0.404285527
0.484187323 -0.307140655 -0.243719393 -0.189558087
0.224669871 0.14215967 -0.030128284 -0.363440424 -

0.528624877 0.52332377 -0.540642489 -0.175600043 0.220273037
0.574777972 0.228174809 0.001297416 -0.189599778
0.321474715 0.539934161 -0.03609975 0.168475212
0.624801317 0.313035519 -0.078036924 0.127732474
0.23757724 -0.185306039 -0.313638182 -0.206377369
0.344809722 0.309368404 -0.329835464 0.420918522 -
0.540560308 -0.254177145 0.249136769 -0.375586458 -0.313616272
0.024986711 -0.144273918 0.023624135 -0.205904998
0.01979808 0.220979652 0.208081636 0.314911226 -
0.390091281 0.292479735 -0.014571853 -0.137229312 0.15488165
-0.378123095 0.365063524 -0.003278336 0.30340445 -
0.28454747 0.240353447 -0.31002923 -0.028217029 -0.123300077
0.176488698 0.036956179 -0.172733561 0.168137821
0.213629989 -0.080079257 -0.320287352
KEGG_AMYOTROPHIC_LATERAL_SCLEROSIS_ALS -0.118526894 -
0.076820243 0.006635979 -0.029488759 -0.05974519 -0.008607667
-0.247675654 -0.007042015 0.224173714 0.174933191 -
0.290035701 -0.071538004 -0.073284668 -0.247117739 -0.14795188
0.145814864 0.306264392 0.234034798 0.032938279
0.239791625 0.113083223 0.205915163 0.210988221
0.163796697 0.213980075 -0.176827764 -0.107407896
0.028032987 -0.229149145 -0.177259983 -0.083915036

0.091685312 -0.322209858 0.092079838 0.241702935 -
0.03409784 -0.150444808 0.248376296 -0.174122864 -0.083189533
-0.117303794 0.035596866 -0.051531695 -0.034875157
0.078235608 0.153930936 -0.109117155 -0.223164023 -
0.218658887 0.057600642 -0.20562763 0.162731074 0.095536518
-0.065773307 0.068301176 0.031211093 0.092706584
0.111660407 -0.118279254 -0.046610559 0.082137037
0.019044905 0.308747563 0.020515866 -0.139949931 -
0.128954313 0.161107122 -0.023322948 -0.05377058 -0.185684558
0.200905451 0.222448322 -0.39213783 -0.04128355 -
0.339620489 -0.063073685 -0.011412678 -0.16620123 0.030266578
-0.110475867 -0.147197059 0.061386422 0.111005587
0.19884651 0.268385358 0.126643677 0.092206258 -
0.049395068 -0.02809802 -0.373973969 0.007451248 0.150535165
0.040566945 0.258509072 0.040382488 -0.230562191 -
0.189921205 -0.050229131 -0.006719233 -0.187935294 0.186831433
-0.300035813 -0.128455517 0.306964203 0.219749381 -
0.031245461 -0.238286935 0.231966831 0.061934261 -0.170010104
-0.137673162 0.019935095 0.187824384 0.222150282 -
0.173788077 -0.009866589 0.009344672 -0.180051073 -0.24506535
-0.051899633 -0.176303867 -0.189497288 -0.088603338 -
0.294182081 -0.181537041 -0.160392881 -0.11971232 0.07527391

-0.084550376 0.150844947 0.049127116 -0.190198967 -
0.097380987 -0.060920013 -0.205376747 0.240664524 0.336874789
-0.140638712 -0.020326778 -0.080612524 -0.246379497
0.057923652 0.074230625 -0.029717303 0.289418242
0.054262585 0.170049678 0.002116855 -0.218305656
0.078873965 0.33717499 0.090933861 0.213305593
0.199457835 -0.069251129 -0.203103505 -0.062373648 -
0.090063125 -0.254856593 0.295491192 0.27064962 0.123934822
-0.010900764 0.001835329 0.151936711 -0.034790447
0.210156666 -0.134597251 -0.146896549 -0.266290238
0.073143617 -0.203353443 -0.047100942 0.121744642
0.051368749 0.17816995 -0.15465762 -0.193756178
0.319458184 0.172363973 0.22567637 -0.224677328 -
0.022687989 0.280763631 0.21990574 -0.145609265 -0.150070347
-0.104442301 0.017470027 -0.009641275 0.012709775
0.083088475 -0.103372616 -0.165947437 0.117864899 -
0.061083004 -0.354407939 0.196652517 -0.26976263 -0.181306689
0.229753034 -0.028958198 0.12066575 0.080741637
0.040819245 -0.298698283 -0.101689808 -0.047377946
0.037666596 -0.117965203 -0.150852327 -0.278341481 -
0.2620287 0.057353668 0.017413033 -0.143308694 -0.114124757
0.016771254 -0.208151658 -0.044161691 0.035922553 -

0.085124995 -0.16061466 0.091886052 0.010350706 0.025177962
0.311925734 0.091642505 0.150098837 0.360651887
0.045203834 0.183638471 -0.126671311 -0.131090658 -
0.230813649 0.096250731 -0.019171134 -0.038404244 0.071926521
0.093406274 0.074792043 0.062135237 -0.215960074
0.216517637 -0.184120044 -0.241113757 0.053148287 -
0.335047714 -0.207795512 -0.19492553 -0.277903695 -0.067681183
0.096781135 -0.034572284 0.04870745 0.073407976 -
0.250446271 -0.071112467 -0.137452624 -0.045980206 -0.098492285
0.21100428-0.083032026 -0.135122133 0.334226064 0.208877602
0.202852584 -0.050045035 -0.043610268 -0.091501646
0.009601528 -0.138401126 0.077715459 0.091512692 -
0.021546867 0.123205005 -0.091517337 0.193558748 -0.249509546
0.047572798 -0.234740038 0.35618307 0.165401153 -
0.041097183 0.051465169 -0.007536868 -0.309245596 -0.236320567
0.197233589 -0.106727113 -0.046656327 0.073905611 -
0.037686904 0.02402646 -0.202432575 -0.075785245 -0.130613836
-0.364497425 0.248232243 -0.211398976 -0.224761679 -
0.307700528 -0.099086426 0.236564443 0.026850493 -0.094689214
0.179387246 0.067023447 0.220918728 -0.17918209 -
0.237892576 0.130042411 -0.011138228 0.073813128 0.088752945
-0.089231103 0.018085621 0.012444427 -0.114635014

0.125245657 -0.244956484 0.042334533 0.219355231 -
0.076228455 0.111476942 0.37672288 0.013768578 -0.086490527
0.16666563 -0.059343776 -0.151161452 -0.306986009
0.032319285 0.169558504 0.142100424 0.049181957 -
0.106930898 0.180582667 0.115306466 0.079045862 0.058896827
-0.027758758 -0.181647068 0.261299325 -0.240905176 -
0.149154068 -0.217297112 0.008374454 0.12557577 -0.004175038
0.229004408 0.249179767 0.062785136 -0.076913503
0.000742287 -0.181953178 -0.027542608 -0.278074046
0.099914632 -0.103866079 -0.067346948 -0.405272146 -
0.004430549 0.193296403 0.265387679 0.055983944 0.246466483
0.023065307 0.002519881 -0.066535749 0.15316956
0.209436347 0.093141007 0.164937331 0.070146088
0.042273451 0.005134777 -0.109231998 0.080611411 -
0.012719018 0.131630556 0.041262234 -0.335860635 -0.04815789
0.069367843 0.235740366 0.00487586 0.231340208
0.189455475 -0.060103737 0.078262822 0.185052818
0.013591005 0.103809712 0.219472741 -0.228482463 -
0.02598597 0.143213818 -0.256022146 0.024242465 -0.284984793
-0.000427053 0.150892162 -0.277608393 -0.053676306 -
0.089833617 -0.201318982 -0.066112209 0.129302043 -0.13134383
0.027230747 0.022893545 -0.218021541 -0.041892227

0.06165899 0.306205192 -0.011115523 -0.089103078
0.128560119 0.145465034 0.25033473 0.01347948 -
0.079264016 0.313790317 0.151118419 0.100301 -0.275757968
0.087529979 0.184168668 -0.155080949 -0.110393899
0.173229249 -0.098660039 -0.321702972 -0.215423733
0.105373256 0.024601336 -0.118478798 0.047302696 -
0.034091178 -0.055353574 0.157686725 -0.14613221 -0.025013273
0.020707948 -0.010416223 -0.021645389 0.063481891 -
0.070535543 -0.163655935 0.307873535 -0.155307535 -0.270830741
0.05426977 0.156827011 -0.164086832 -0.014208807
0.077229241 -0.251415114 0.057578453 0.053013992
0.121460729 0.126279037 0.0357327 0.058882515 0.250353385
-0.149582836 -0.0700745 -0.137943762 0.023292455 -0.212084059
0.173102508 -0.019519848 0.091593235 -0.003770057
0.08851276 0.012946957 -5.91E-05 -0.31215638 0.053514037
0.059675371 0.094422705 -0.246716568 -0.109237899
0.137396288 -0.095586465 0.028336902 -0.040633624 -
0.087489889 -0.041506503 -0.040147388 -0.162923313 0.171595588
0.070021117 -0.024180857 -0.078642325 0.095683197
0.328454567 -0.022811759 -0.021034397 0.268307768 -
0.031829757 0.2318472 0.176196381 0.235359372 0.165866067
0.112682406 0.104140165 0.030475433 -0.193631104 -

0.049519702 -0.280496613 -0.164435108 -0.071068924 -0.244669972
0.064097841 -0.155878718 -0.208783874 0.005894325 -
0.211776143 -0.024894252 -0.131699076 0.267234612 -0.033020091
-0.042073829 0.091441024 0.394974618 0.336657536
0.067482092 0.158094368 -0.095872706 -0.211182442
0.089445467 -0.24686065 0.025181138 -0.15715161 -
0.088186983 -0.250652638 0.128440158 0.037309593 0.025171505
0.257309857 0.109601812 0.089445934 -0.110338133
0.174398243 0.095905102 0.169433424 0.018053092 -
0.084643485 -0.018590667 -0.056031175 0.170474993 0.121491108
-0.02129722 -0.20486138 0.125961808 0.27017492 -
0.078121976 -0.102826437 0.087244171 -0.183009867 0.144139846
-0.054133657 0.054461403 -0.025732682 -0.237725943
0.058468452 -0.098000011
KEGG_HUNTINGTONS_DISEASE 0.344051444 0.188304308 -
0.255291997 -0.334480038 -0.211439016 -0.26601749 0.028067252
-0.152784726 -0.373327447 -0.355747799 -0.414078196
0.371561548 -0.235537006 0.10600947 0.134096789
0.252538518 0.34631349 0.157140275 -0.440301021
0.20058822 -0.254636725 0.171594965 -0.097315704 -
0.079355792 -0.002437903 -0.275299729 0.3720977 0.144918536 -
0.346064204 -0.266844958 0.266613327 -0.48381845 -0.207780841

0.045617162 -0.298613993 -0.389439797 0.194580462
0.28980938 -0.260517791 -0.225562478 0.376293551 -
0.075719318 -0.347349895 0.277443597 0.160673306 0.266952047
0.074275758 0.183711638 -0.197783698 0.009432243
0.482421487 -0.252876593 0.301657414 -0.369863052
0.279570122 0.172722436 -0.281344647 0.025248156
0.305807682 -0.140556817 0.138711139 -0.47537699
0.29990727 -0.07362454 -0.32158445 -0.458392968 -
0.242360299 0.176303644 -0.248234762 -0.045229667 -0.014443909
-0.348968689 0.255302707 -0.090739369 -0.171198154 -
0.143255624 0.050824207 -0.076129561 0.216647573 0.457147321
-0.206853073 -0.360363914 0.258468322 0.261491441
0.038278477 -0.156481602 0.110662799 -0.346601289 -
0.496334406 -0.077643153 0.011558973 0.149967077 0.255739237
0.387116363 0.142915635 -0.422353333 0.220040549 -
0.465607827 0.379221214 0.307633493 -0.101554453 -0.360308955
-0.206550328 0.369778057 -0.313292034 -0.406629581 -
0.017674926 0.411676268 -0.362154674 0.045435142 -0.278942608
0.243191057 0.418758471 0.007185406 -0.441835673 -
0.09878125 0.425205684 -0.021780217 -0.362433387 0.261735504
-0.081818698 0.244274019 0.304797506 0.327024711 -
0.433760666 0.066785119 0.469879091 -0.437998324 0.241278497

-0.056242328 0.175139002 -0.302762616 0.296134405 -
0.333303765 0.344572094 -0.432856957 -0.204435236 0.314451213
-0.150329273 -0.339650909 -0.027870934 0.24764919 -
0.052207614 -0.117707443 0.127998299 0.268270263 -0.145188108
-0.021142307 -0.382523504 0.300212406 0.039508347 -
0.277043594 -0.452380573 -0.185626826 -0.26778406 -0.042827511
-0.341470318 0.178694785 -0.212966995 -0.418308376
0.103861691 0.036405554 0.036556068 -0.307346819
0.512415879 -0.429487538 0.203682225 -0.393794888 -
0.332010352 -0.508013621 0.301951197 0.222116852 0.247696465
0.37413667 -0.25686002 0.121093887 -0.337979759 -
0.041423002 0.32567356 -0.220849725 0.237962412 0.253826591
-0.169516014 0.323127545 0.001816931 -0.137794748
0.093486505 -0.283334368 -0.338204741 -0.268714965
0.201271965 0.483477767 -0.227009417 -0.125942388
0.457468353 0.23666889 -0.342289517 0.23842641 -
0.248498707 -0.123126797 0.403068016 -0.178800409 0.278632499
0.347166381 -0.110654662 -0.12956587 -0.370190724
0.239794692 -0.170819961 0.146644091 0.340180761 -
0.145545513 -0.105325844 0.070303638 0.07972471 0.184900642
0.365695354 -0.442883068 -0.277208562 -0.371303799 -
0.487494701 0.392219917 0.143408991 0.196701661 -0.317945841

-0.055708505 0.37174311 0.122737612 -0.150042232 0.373891939
0.398696473 -0.186077064 0.188813078 -0.483462758 -
0.386689549 -0.238368624 -0.404768005 -0.291080895 -0.254364082
0.354587618 0.177229969 -0.246868206 0.240731079 -
0.131008752 -0.044257812 0.01971446 -0.31196283 -0.213132919
-0.199200275 -0.187802445 -0.221864218 0.16242455 -
0.070774697 -0.310905793 0.24092016 -0.372354282 0.236402855
0.181457167 0.253988863 -0.067811982 -0.182179517
0.429993753 -0.017733209 -0.149930462 0.278074551 -
0.282747811 0.050408292 0.419802302 0.055708578 -0.216528588
0.43322782 -0.012635017 0.260490005 0.007178634
0.482464716 0.265026587 0.159095586 0.268499183 -
0.206339226 -0.069070685 -0.401135516 0.284621929 -0.284117855
-0.198147 -0.497699294 0.422235569 -0.465884691 -0.250566839
-0.203204818 -0.240651727 -0.129795169 0.265795832 -
0.016357454 -0.359324522 -0.385287036 -0.196999336 -0.128662497
-0.45173017 -0.155228346 -0.312738893 -0.344361536
0.180406248 -0.148089482 0.04428127 0.461839825 -
0.353202467 -0.425910327 -0.047540945 -0.32282613 -0.076740005
-0.403938857 0.125699619 -0.337284978 -0.134461681
0.275012877 0.36597477 -0.12364208 -0.142619299
0.31645336 0.184091106 0.389619691 0.413658676

0.20677557 -0.06873685 0.271335746 -0.065868655
0.354081191 0.237924463 0.021828246 -0.223609294 -
0.389067013 0.208459824 -0.371021241 0.304773891 -0.093697511
0.141248789 0.00742024 -0.234588224 -0.397222312 -
0.205409726 0.432938035 0.112533686 -0.35090022 -0.238825604
0.087398897 0.172664324 -0.411299182 0.021528513 -
0.204365388 -0.261985999 -0.323266917 -0.151639458 -0.135271726
0.324948383 0.282286061 0.017609785 0.147373182 -
0.017948239 0.396560389 0.455731991 0.322409687 0.319861979
0.048980222 -0.321072553 0.215041441 -0.529145374
0.283833344 0.018483286 0.289236121 -0.394381127 -
0.449195437 -0.040001374 -0.122499992 -0.176712266 -0.05110861
-0.020764496 0.008942943 0.352777998 -0.412571767 -
0.352509658 0.23022712 -0.100997893 0.100505102 -0.011168572
-0.020684391 0.273804282 -0.301783599 -0.27211912 -
0.046773528 -0.192992774 0.216290339 0.260463868 -0.353956975
0.075127839 -0.223542026 -0.201954556 0.217159431
0.274148533 -0.428663995 -0.095325559 -0.4603881 -0.228792745
-0.162197187 -0.048616801 0.097184845 -0.206679707 -
0.375062895 -0.244513537 0.130968905 -0.279911251 0.472709897
-0.33689301 0.106971 -0.269668496 0.220405836 -0.179553331
-0.302853138 0.436510498 -0.271444579 0.376613676 -

0.411167637 0.269647031 0.438337264 -0.141377168 0.12127873
0.402977177 0.184954963 -0.270431382 0.081438786
0.03701008 -0.013126459 0.29200565 -0.139137493 -
0.060636921 -0.092817007 -0.422230505 0.115092557 0.464418721
0.006024174 0.069505415 -0.325794443 -0.026241768 -
0.323458113 -0.286007411 -0.084182738 -0.336787271 0.365382277
-0.337073388 0.128077498 -0.29633972 -0.273374968 -
0.147793186 -0.214057205 0.383636683 0.468706646 -0.293750938
-0.310630689 0.189688586 -0.1454391 -0.150455684 0.311639972
-0.428783162 -0.212813031 0.233237014 0.026237616
0.121769946 -0.057436315 -0.302691263 -0.038553779
0.019187818 -0.169154805 0.230789488 -0.125016765 -
0.415719144 0.02847993 0.287913751 0.247025859 0.319954743
-0.377164847 -0.358732459 0.095366969 0.228354931
0.226096057 0.294977607 0.518768683 -0.017848959 -
0.174043412 0.284596926 -0.126060064 0.197804623 0.299044944
-0.437511717 -0.306357401 -0.380684828 -0.237125185 -
0.099931014 0.341151682 0.315028553 0.34320411 0.02038358 -
0.042544198 0.387550061 0.372209731 -0.182306479 -0.170576039
-0.085639441 0.037446872 -0.098769685 -0.047226167 -
0.221798704 -0.406942308 0.374884787 -0.433405095 -0.124539991
0.160260075 0.449953995 0.179954942 0.079721268 -

0.167897529 0.261539115 0.40955175 -0.040224828 0.226857345
0.432201916 0.370606807 -0.128553083 0.028919108
0.253860458 -0.014197722 -0.067179893 -0.00434009
0.284463705 0.205196833 -0.27255179 0.317034053 -
0.410846738 -0.087130544 0.127472571 -0.247685532 -0.189274488
-0.041543687 -0.098805485 0.051008424 -0.217472479
0.016084363 0.064686444 0.062469954 0.262343205 -
0.17381211 0.220011203 -0.116662855 -0.147775997 0.08227765 -
0.325620651 0.33401255 -0.112977637 0.24513811 -0.249672768
0.136792603 -0.270510557 -0.043554328 -0.153128665
0.069445584 -0.084960868 -0.124626704 0.04533627
0.090022964 -0.107771753 -0.220365241
KEGG_PRION_DISEASES -0.040555177 0.163779672 -0.289852507 -
0.112789678 -0.023994938 -0.197190876 -0.021444433 0.375156055
0.227499368 0.376719931 -0.394613315 -0.311225782
0.194807241 -0.362578755 -0.002697679 0.039500198 -
0.12819491 0.096641525 0.280289648 0.045643339 -0.073280643
0.364143668 0.274615971 -0.045393575 0.511183288 -
0.194251411 -0.408154684 0.093923277 -0.092624141 -0.212321219
-0.145079042 0.086634992 -0.342827141 0.130665244
0.476097741 0.171195613 0.017538955 0.16696736 -
0.185436454 -0.403673841 -0.292360893 0.420106267 -0.13019322

0.077728166 0.260637262 0.076929075 -0.188133859 -
0.264548537 0.123882307 0.023980608 -0.124172827 -0.136099501
0.369696225 -0.125487383 0.073261154 -0.158391372
0.03320069 0.196594816 0.120013043 -0.182858153
0.131663501 0.2902432 0.10856181 -0.317155492 -0.146367955
-0.404730816 0.371705141 -0.07215195 0.448472677 -
0.041931456 0.248980612 0.201051258 -0.167218026 0.241038416
-0.262346235 0.163463057 -0.076394692 -0.369614399
0.311406713 -0.124826524 0.142079896 0.37852404
0.131704994 0.46302586 0.325214135 0.307330423 -
0.104806794 -0.230516348 -0.048321311 -0.183878243 -0.122963405
0.053338061 0.167458122 0.239487862 -0.016010321 -
0.261187206 0.180576379 0.053923907 0.248231327 -0.2635807
0.337560308 -0.023046759 -0.188283226 0.32683644
0.171822242 -0.131702523 -0.025466988 -0.21766551 -
0.145088049 0.302763819 -0.051587626 0.233790345 0.239636992
-0.130288624 -0.024036229 0.089886639 0.017095764 -
0.330365854 -0.21651715 -0.074738722 -0.18362238 -0.339444018
-0.055708829 -0.272974978 -0.10259234 0.049188819 -
0.33319851 -0.008686519 -0.031429985 0.178382252 0.293194179
-0.139671504 -0.204281126 -0.238843913 0.388664385
0.270611005 0.420050333 -0.104591088 -0.180809694

0.004341452 -0.2947093 0.224084277 -0.257016963 -0.29346067
0.308552693 -0.189286598 0.140948681 0.061286298
0.215980834 0.40591455 0.257324015 0.217835318
0.290246012 0.313757759 -0.236718187 0.001320001 -
0.150982648 -0.031659605 -0.046860937 0.262230328 0.241584943
0.377170378 -0.156477707 0.070253574 0.156458789
0.335673696 0.052707575 0.309956496 0.368654804 -
0.425697667 -0.214091021 -0.358697483 0.137002369 0.187534352
0.20849624 -0.263731817 -0.150233368 -0.383990462
0.034280766 0.438205226 0.209885299 0.113667459 -
0.019773095 0.40233536 0.069536597 0.136365748 -0.411431005
-0.278084925 0.280040418 0.154377706 0.151856747 -
0.128558558 -0.200418043 -0.385132392 -0.03177303 0.042068894
0.091140156 0.067161194 -0.545435074 -0.202939187
0.314782753 0.012228067 0.220200822 0.244897803
0.27023671 -0.244380944 0.116935935 -0.363969874 -
0.071284083 -0.079810122 -0.081552487 -0.334068677 -0.273620839
-0.146376522 0.048848445 -0.314282046 -0.005485189
0.253517233 -0.402654627 -0.386718395 0.363669554 -
0.106284818 0.101184690.154637731 -0.22110994 0.211750391
0.466304494 -0.066393008 -0.011085413 0.222141517 -
0.16199861 0.351780688 -0.252249653 0.329711357 -0.407815701

0.030965617 0.217126239 -0.04913837 -0.219608194
0.043690827 0.062641879 -0.062794231 -0.283813719
0.187006483 -0.259884624 -0.120708053 -0.00061758 -
0.385570331 -0.273256276 -0.178216022 -0.31776443 0.028878571
0.141657945 0.129852028 0.128110442 0.297810681 -
0.041680584 0.051478427 -0.113829356 -0.03641749 -0.091565459
0.359954271 -0.225504454 -0.212086835 0.410268763
0.090041325 0.451218718 0.066505017 0.184019014
0.162992723 -0.225363167 0.082926218 0.210847675
0.036933797 -0.132337122 0.007651743 -0.266362657
0.212925019 -0.479358468 0.105802734 -0.132302782
0.301357281 0.126459566 0.075394302 -0.018814185
0.138609269 -0.375997993 -0.000738987 0.338952395 -
0.286793127 -0.292906597 0.161869185 -0.140161146 0.022394718
0.115623716 0.275971328 -0.195062744 -0.298371575
0.237537692 0.128433107 -0.044066194 -0.153697037
0.020990157 0.297474101 0.047649291 -0.085566548
0.194938789 0.092190885 0.237629679 -0.05849318
0.204059467 0.165589371 0.012703988 -0.216354339 -
0.226821926 -0.062562481 0.239467511 -0.035169325 -0.107518988
0.097342847 -0.161865831 -0.2786369 0.049737262 0.190757874
0.22038563 0.425740794 -0.044973857 -0.413345103

0.156202097 -0.023762106 -0.21584666 -0.11015488

0.055828714 0.18888156 0.167093681 0.262692113

0.122658286 0.380018491 -0.355188271 0.045173372

0.245919716 -0.037875836 -0.296726572 -0.080248377 -

0.158820055 -0.065533485 -0.35188564 -0.041332745 0.063242976

-0.133440403 0.205942943 0.167711403 -0.069605785 -

0.108342274 -0.263379115 -0.090756654 -0.081586728 -0.016980414

-0.105564037 0.099993422 -0.199219399 -0.21202554 -

0.167670834 0.459276791 0.30793928 -0.197915521 -0.232056772

0.096387356 0.220634355 -0.308864525 -0.110636706 -

0.045837123 -0.05273811 0.346515299 -0.154447597 -0.167159743

-0.015017563 -0.020406715 0.292955186 0.026889705 -

0.15293065 0.174705596 -0.209335526 -0.131819373 -0.026318932

0.194757862 -0.147910527 0.4654054 0.348867511 -0.350015401

0.202153114 0.310196905 0.129613431 -0.115571238

0.267496798 -0.131544678 -0.20280745 0.007292327 -

0.264295755 0.175525094 -0.190719475 -0.088693787 0.047540894

-0.475590736 0.343209483 -0.246884042 -0.313621681 -

0.10158358 0.275957576 -0.224654176 -0.243069879 -0.409623813

-0.145739215 0.154151522 -0.050759257 0.051653281

0.134741066 -0.339645448 0.469915785 0.45480315

0.322372871 -0.038782683 -0.308075959 0.352492954

0.334811683 0.015959169 -0.04353876 0.255871309
0.169635182 -0.259078533 -0.078382192 0.196734512
0.224815137 -0.283753302 -0.273630163 0.235924314 -
0.008019968 -0.18090133 -0.248985051 -0.330650718 -0.281859092
-0.14539284 -0.254754657 -0.07153661 0.255058449 -
0.342736601 0.28824512 0.17225148 -0.438712508 -0.255418235
0.175636182 -0.23679508 -0.135720301 0.285248782
0.272948716 0.114982391 0.251153683 -0.099958968 -
0.463685104 0.20800942 0.229530029 -0.068572561 0.046969142
0.044655472 0.190269185 -0.128107795 -0.07803595 -
0.008657015 -0.253271726 -0.220514393 -0.387344685 -0.229716051
0.114879014 -0.10113364 -0.167523167 0.006754327
0.069009349 0.129200554 -0.340211307 -0.119866857
0.161805785 0.098784235 0.015049687 -0.066365899
0.231781257 -0.304540207 -0.089100616 0.355824515 -
0.133837936 -0.304812192 -0.126179243 -0.079746889 -0.002415995
-0.094875712 -0.103614021 -0.393424576 0.152778134
0.412221775 -0.078136776 -0.072400639 0.336943634
0.042000672 0.320909328 0.072483171 -7.73E-05 0.249459712
0.383143233 0.139847121 0.447119091 -0.398190292 -
0.34903156 0.143603787 0.268214909 -0.254228735 -0.287938827
0.252020664 -0.259947365 -0.201799813 -0.21773948 -

0.179732138 0.051361139 -0.372181737 0.347955118 0.210425358
0.124798349 0.150299764 0.290891516 0.416348275
0.112047867 0.026252582 -0.170977099 -0.269182213
0.339272493 -0.482495178 -0.053688188 -0.063210259 -
0.228265844 -0.097434003 0.199175546 -0.215195309 -0.343385066
0.203870379 -0.290477182 0.324181481 0.295773428 -
0.106646406 0.112904604 0.071362055 0.275191707 -0.237700631
-0.056065788 -0.109038313 -0.165974711 0.219612223
0.246686406 -0.082975097 0.179037707 0.064349418 -
0.05669163 0.12139142 0.008595962 -0.156652336 -0.226156008
0.338932139 0.155591013 0.017536594 0.003338562
0.052296077 -0.094129193
KEGG_VIBRIO_CHOLERAE_INFECTION 0.341294645 0.354312949 -
0.122210539 -0.197717443 -0.326851849 -0.217848609 -0.328984388
0.119557874 0.298281622 -0.159117103 -0.467855958
0.531673455 -0.181264983 -0.109112722 -0.271313487
0.090399698 0.105094928 0.067430804 -0.276071649 -
0.099049486 0.117162362 0.251866378 -0.018985416 -0.0921805
0.072447499 -0.315318865 -0.307454456 0.073689313 -
0.220146626 -0.417955454 -0.137873704 0.106117816 -0.215793866
-0.218594896 0.344276645 -0.061947258 -0.011491162
0.069416277 -0.15079514 -0.200267592 0.404825788

25882 0.220128416 -0.132647987 0.05615275 0.502775371

25883 0.315037002 -0.066536437 0.292840135 -0.04970477 -

25884 0.118496020.365924679 -0.03851964 0.260582043 -0.047061042

25885 0.258477471 0.386109711 -0.293818061 0.36885475

25886 0.015567791 0.338814839 -0.140972837 0.006371338

25887 0.022622693 -0.073969353 -0.054272823 -0.518340606 -

25888 0.039656362 0.478728825 0.000229585 -0.362688618 0.33010557

25889 -0.017622296 0.35312336 0.018790065 -0.221651712

25890 0.049262775 0.053167264 -0.159778114 0.062908615

25891 0.401293247 -0.186797119 0.01935571 0.017175844 -

25892 0.02129162 0.043313917 0.06831894 -0.031756662 0.073437814

25893 -0.284449948 -0.287908876 0.227035253 -0.157899054

25894 0.02743957 0.333988916 0.024246821 -0.191200765

25895 0.418448563 -0.140235649 0.310484358 0.198192195

25896 0.31392927 -0.254029723 -0.290036631 0.304002273 -

25897 0.035189067 -0.385017196 -0.214414509 0.126488712 -0.120935354

25898 -0.064843959 -0.317377189 -0.117573061 0.286792575

25899 0.272030064 -0.322058031 -0.035418437 -0.015539706 -

25900 0.39498093 -0.459413958 0.33133146 -0.312756007 -0.309500715

25901 0.10423529 0.36785962 -0.336535187 -0.307586935

25902 0.289671622 -0.343981452 0.013483359 0.002375266

25903 0.280429105 -0.337116865 -0.052886804 -0.079124065

0.196740391 -0.002476569 0.101738021 0.196808831
0.102023441 0.105984072 -0.256232784 -0.109632369 -
0.270893546 -0.359317837 -0.088798655 0.343339373 -0.301798251
0.108702792 -0.265246863 0.2434731 -0.101584672 0.118910193
0.201370499 -0.052125647 -0.297328391 -0.26018102 -
0.336848251 0.160450725 0.052124812 0.014621315 0.188286704
0.137802464 -0.322208336 -0.327125877 0.228014041 -
0.115931372 0.081014125 0.212180527 -0.109902394 -0.40077265
-0.143881962 -0.004626482 -0.193822764 0.213253703
0.006399213 0.307858733 -0.386873251 -0.083711246
0.005129177 0.330565447 0.335888734 0.444143132 -
0.234882078 0.134114479 0.173227375 0.055605562 -0.202784472
-0.268960345 -0.146318356 0.291538042 0.229706632
0.061201115 0.115555758 -0.115290436 -0.058425832 -
0.21410566 -0.357319824 -0.04512832 -0.36615989 -0.173450676
0.160028221 0.213920834 0.22538313 0.255897657
0.165548812 -0.176073784 -0.133623242 -0.160193845
0.002533943 -0.071047555 -0.036194381 -0.186285709 -
0.102465546 -0.048104657 -0.228104385 0.055023365 0.23930792
-0.150995845 -0.432125923 -0.298224906 0.152510737
0.348090213 -0.163287961 0.068093836 -0.233203879 -
0.272054177 0.048895035 -0.174988096 -0.043389878 0.159306771

0.015925384 0.064048746 -0.436155822 -0.066440217 -
0.336589275 0.353241699 -0.217826641 -0.28707463 -0.018429608
0.289752276 0.296980053 -0.411864536 -0.379202472
0.12298427 -0.057240778 -0.137695509 0.208764033 -
0.03310038 -0.344226705 -0.378420901 -0.261197698 0.062824798
-0.220693058 -0.039679041 0.213902966 0.09839687
0.289359634 -0.132203419 0.113894303 -0.117760793 -
0.225908366 0.20989523 -0.30088281 -0.178417413 0.145169071
-0.256201337 0.342314059 0.22684941 -0.052728395 -
0.172264005 -0.16287037 0.279062479 0.368566783 0.03762983
0.118712050.243997914 -0.041455009 -0.201118391 -0.391015557
-0.172398764 -0.303225171 0.102788889 0.167913632 -
0.305810832 -0.261449054 0.079082381 -0.307189599 -0.295164525
0.019947978 -0.219257798 -0.056541818 0.198138594 -
0.058021915 -0.157235353 -0.208660529 -0.033423403 0.031599402
-0.052133878 0.023199875 -0.324117546 -0.267869623
0.322982811 -0.069476466 -0.203396933 0.263023327 -
0.350811556 0.004320776 -0.175282897 0.187541303 -0.343153102
-0.294394707 -0.146018273 -0.102646216 -0.125310925
0.228773362 0.345716796 -0.153466157 -0.174184436 -
0.013093033 0.249805478 0.333636698 0.316519679 0.296059959
-0.073640182 0.198411381 0.312449615 0.20668829 -

0.307195797 0.100668681 -0.214958226 -0.197986109 0.243417322
-0.289621692 0.373940769 0.131308764 -0.034878269
0.065037758 0.23947022 -0.143088469 -0.059853368
0.004400554 -0.115646502 -0.384882937 0.034596606
0.019436568 -0.032526607 -0.437346264 0.31399386 -
0.182088742 -0.471794406 -0.141683628 -0.056523792 -0.00812919
0.010155986 0.185154946 -0.3400958 -0.140446464 0.142706272
-0.101608509 0.361225247 0.071968116 0.30217363 -
0.211190836 0.202921571 0.134133521 -0.029919703 -0.142908668
0.069802842 -0.274521487 -0.181758999 -0.021237378
0.162970552 0.062475968 0.13323636 0.04315004 -
0.203346041 -0.160893361 -0.300039301 -0.061102421 -0.279774675
-0.317994631 0.125609616 -0.056098427 -0.169009934
0.044118016 0.120373491 -0.165558471 0.285548791
0.074950551 -0.182187203 -0.0676045 0.07536752 -0.307318703
-0.01085406 -0.23376564 0.388317395 0.190811484 -
0.156520561 -0.236564899 0.169327211 -0.40046441 -0.003091527
0.062693337 -0.231870944 0.044986018 -0.35186557 -
0.480786693 -0.298031475 0.256498467 -0.36670166 0.124191493
-0.307605767 -0.121904479 -0.059060547 -0.173933456 -
0.307048427 -0.020937766 0.275988163 0.24242058 0.259186579
0.005741947 -0.160789494 0.422847078 0.002175869 -

25970	0.103468951	0.379378439	-0.366951813	-0.023673363	0.199641124
25971	0.046383521	0.035522965	0.36483234	0.13463505	-
25972	0.211936084	0.028328823	-0.266978233	0.266884539	0.466977899
25973	0.108070596	0.297188616	-0.350520387	0.081563213	-
25974	0.351764331	-0.416418063	-0.041564872	-0.138076585	0.499169719
25975	-0.241286695	0.015355606	-0.275878354	0.062649561	-
25976	0.35234578	-0.192316074	0.096403596	0.069314645	0.063535488
25977	-0.237751247	-0.057149528	0.092706078	0.169364346	
25978	0.257544805	-0.347576222	-0.184573437	-0.029717981	
25979	0.054063795	-0.239691897	-0.213017048	0.073433014	-
25980	0.043931495	-0.091031629	-0.299121689	0.197609763	0.052797363
25981	-0.242109658	0.072458844	-0.091122466	0.030974829	
25982	0.119866015	-0.346180827	-0.173186727	0.100149984	
25983	0.173490901	-0.197842232	0.448988336	0.109943787	-
25984	0.240438353	0.043844058	0.442536608	-0.203736812	0.269364568
25985	0.406687219	-0.396761825	-0.209669739	-0.330867395	-
25986	0.117700437	-0.402895204	0.240362336	0.144044908	0.234505148
25987	-0.071047232	0.218827745	0.132393192	0.280718875	-
25988	0.027444129	0.197172505	0.235707396	-0.237286431	0.106068318
25989	-0.178138326	-0.402080182	-0.413279833	0.260407687	-
25990	0.07044039	-0.205891251	0.185162456	0.315302486	0.017235925
25991	0.130665967	-0.097208943	-0.091253252	-0.071175824	-

0.361973026 -0.193649498 0.216324551 0.301504963 0.183968925
0.20526524 0.324013466 0.128898014 0.127364977
0.02996177 -0.149160208 -0.117555958 -0.231700193 -
0.018974727 -0.223696549 -0.08414412 0.014171238 -0.053990185
0.185758237 -0.129135332 0.310627887 0.124447677
0.23322461 0.170327171 -0.229362494 -0.109853698
0.111588760.041079736 -0.021242228 0.110631035 -0.180819087
0.182994533 0.236197942 0.279230211 0.008605435 -
0.091067165 -0.226373768 -0.243017698 -0.016290464 0.006766127
0.167810748 -0.001083402 -0.13683054 -0.059191157
0.222566606 -0.173511744 0.080328868 -0.061952123
KEGG_EPITHELIAL_CELL_SIGNALING_IN_HELICOBACTER_PYLORI_INF
ECTION 0.405971959 0.311499107 0.038651009 0.162547188 -
0.186139831 -0.239125184 -0.243809528 0.183907702 0.237552199
-0.129151963 -0.347912657 0.290549567 -0.312210005 -
0.181106994 -0.382199224 -0.25928029 0.2203384 0.150231944 -
0.31878641 0.215591246 0.152254301 0.369285141 -0.115999537
0.315582753 -0.063221083 -0.309408243 -0.348247058 -
0.141530402 -0.15925335 -0.41003507 -0.11679364 0.300865269
0.028823378 -0.150039756 0.3201434 -0.161187429 -0.271685615
0.049508941 -0.211639981 0.138433988 0.279357902
0.276503604 0.234510734 0.285929929 0.254479419

26014 0.265156774 0.187316379 0.072325939 -0.244697111 -

26015 0.298326449 0.162525477 0.148500708 0.201055677 -0.013673452

26016 0.329453746 0.305277871 -0.337365852 0.242473262

26017 0.159538945 0.114363924 -0.168641297 0.037306258

26018 0.284886776 0.159497499 -0.21369473 -0.356458521

26019 0.224238783 0.373622589 -0.083680176 -0.299978799

26020 0.278760185 0.220365453 0.134530472 -0.115304478 -

26021 0.262147376 -0.183854129 0.042485531 0.076179243 0.169974666

26022 0.29724027 -0.209199301 0.235873454 0.145378657 -

26023 0.105647109 -0.016100555 0.092642662 0.044070757 0.118263078

26024 -0.069980826 -0.039162743 -0.193971654 -0.195227775

26025 0.240882046 0.294623559 0.279538804 -0.105856418

26026 0.269299759 -0.131079526 0.191990804 0.116331216

26027 0.297035733 -0.252307272 -0.059205666 0.16088479 -

26028 0.235384988 -0.296114101 -0.163813794 0.155193083 0.055659425

26029 -0.201066551 -0.340303756 -0.283692959 0.252744799

26030 0.168038861 0.05267058 0.301855258 -0.10035117 -

26031 0.393453323 -0.511845753 0.28560905 -0.28370435 -0.103982499

26032 0.088218076 0.212948323 -0.185442911 -0.411363322

26033 0.040229538 -0.000973387 0.152241933 0.222535851

26034 0.255476284 -0.245755447 -0.067525115 0.231862053 -

26035 0.113108138 0.095774119 0.152560233 0.108442117 0.194475787

26036 0.081645993 -0.270257215 -0.027244223 0.037643434 -
26037 0.134622756 -0.124936201 0.151283789 0.217332221 0.092250421
26038 -0.312571509 0.065787615 -0.227215915 0.22508926
26039 0.156076138 -0.10513242 -0.121840713 -0.280898812 -
26040 0.402392005 0.170661208 0.029363923 0.166768607 0.100952314
26041 -0.07361166 -0.218600234 -0.408477716 0.116553759 -
26042 0.230477496 0.114655773 0.264104059 -0.337572896 -0.349672775
26043 0.126301391 -0.257763389 -0.18406402 0.088090345
26044 0.317579867 0.138440123 -0.068273708 0.039768918
26045 0.289698110.203755548 0.194425506 0.241806846 -0.00634289
26046 -0.102532638 0.221037203 0.057580054 -0.043442733 -
26047 0.075273487 -0.19940937 0.150206174 0.158687108 -0.01699935
26048 0.283771548 -0.363857247 0.022417697 -0.213457912 -
26049 0.291520532 -0.043823797 -0.337565255 0.080231181 -0.163003907
26050 0.222369801 0.019437906 -0.011411881 -0.019868809 -
26051 0.12310637 -0.345093785 0.046929154 -0.020397762 0.290155187
26052 0.154725186 -0.118413597 -0.26239135 0.032919568 -
26053 0.338124191 0.128967548 0.120943953 0.069915232 -0.257517305
26054 0.00099192 0.089432679 0.354244977 -0.136956912
26055 0.078042306 -0.263690479 -0.072776546 -0.129200424 -
26056 0.315007362 0.290547432 -0.012811178 -0.09160388 0.173546594
26057 -0.043563306 -0.104908239 -0.238141906 0.291423272 -

0.101703071 0.186757183 0.169563743 -0.152928047 0.277034715
-0.211721406 -0.315258504 0.204467149 0.013918655
0.127031661 0.130705724 -0.19496572 -0.083492571 -
0.33061917 -0.151421183 -0.081277019 -0.322688318 0.045766713
-0.060876109 -0.072701555 -0.035803895 -0.228300409
0.302106715 -0.072184289 -0.267351472 0.310206749 -
0.251347275 -0.388609066 0.108045562 0.073524567 0.073118381
-0.294110794 0.254852963 -0.354124541 0.008629504 -
0.111923792 0.354099633 -0.065807877 0.062639998 0.184216918
0.068793228 0.043742425 -0.362543544 -0.134774954 -
0.278914977 0.213044184 -0.066632616 -0.27635687 -0.137898419
-0.226888769 -0.33979833 0.001819173 -0.026685809 -
0.202939641 0.13856243 0.118682016 0.159414966 0.207158374
-0.352546015 -0.175634577 0.23250335 0.006747921 -
0.100887067 -0.102156685 0.160751436 0.018429563 -0.140964342
-0.310347147 0.160286836 -0.15470672 0.134606311
0.171650146 0.047898652 -0.328422647 -0.374835729 -
0.331261237 -0.120911911 0.164751505 0.170025592 0.013682559
-0.380090783 0.0902175 0.148811342 0.39301782 0.094347303
0.400862443 0.383077578 -0.299482216 -0.23671903
0.245324755 0.217252859 -0.32341686 0.055650826 -
0.312958626 -0.371181494 0.223006505 -0.227648253 0.347243712

0.251538506 0.162416702 0.272522073 0.227388501
0.306001619 0.121043471 0.004360243 -0.095563484 -
0.305368282 0.155843821 0.150305362 -0.062593932 -0.271366911
0.401576867 -0.191675943 -0.189113421 -0.262043006
0.010032881 0.082951407 -0.14968396 0.195176757 -
0.368869138 -0.270127572 0.349748616 -0.111989636 0.111523617
-0.141126 0.098843035 0.203306586 0.193185883 0.145414603
0.14465079 -0.127883296 0.187079939 -0.264913528
0.109134848 0.055061956 0.339444199 0.345498236
0.253120617 0.257194703 -0.342990781 -0.414866175 -
0.239650873 0.123924118 0.125131697 -0.389900109 -0.157337979
-0.180969197 -0.192483036 -0.173585528 0.08697044 -
0.034251127 0.211292225 0.197725464 0.124713366 0.003527747
0.100433812 -0.332871131 0.036121797 -0.002852502
0.303015617 0.143697085 -0.417429697 -0.084434193
0.259128188 -0.304590546 0.142815047 0.226596568 -
0.280871232 -0.166140793 -0.109878814 -0.341212004 0.037452915
0.074304416 -0.004802549 0.190083419 0.054852979 -
0.304021982 -0.169891539 -0.29838671 0.030684358 0.116096214
0.208932273 0.296402445 -0.093333451 0.024529335 -
0.143096297 0.235872777 0.25865762 -0.260565192 0.249685369
-0.300634715 0.344323431 -0.108222345 0.02992929 -

26102 0.121468412 0.353123311 0.027344177 -0.333800245 -0.148419585

26103 0.069426595 0.372469962 0.189070202 -0.096387278

26104 0.197589082 -0.035061362 0.123321799 -0.120173163 -

26105 0.277453155 -0.197820061 0.106563227 0.21799867 -0.361117457

26106 -0.034574961 -0.164222766 0.279323697 -0.288909389 -

26107 0.158121014 -0.245958768 -0.047168137 0.150595061 -0.089673651

26108 -0.298933713 0.278520264 0.003504074 0.177399871 -

26109 0.01448494 -0.330357885 -0.196938313 0.151532764 -0.095092069

26110 -0.314188344 -0.069865937 0.118726587 -0.073064695 -

26111 0.193174389 0.177001693 0.107717011 -0.061747795 -0.244982624

26112 0.172516971 -0.047312644 0.037737398 -0.112878262

26113 0.103495309 0.195035617 -0.208745295 0.10069233

26114 0.303466674 -0.077042078 -0.033675281 0.123627921

26115 0.303708209 -0.335354011 0.105539816 0.230967479 -

26116 0.279175757 0.14177793 -0.135234273 -0.123084374 -0.411062232

26117 -0.142347903 0.202074633 0.10585795 -0.022317628

26118 0.131577586 0.366160286 0.281275483 0.069464115

26119 0.098970244 0.239715429 -0.32454921 0.247933777 -

26120 0.230804725 -0.174175245 -0.215106695 -0.071462839 0.136634659

26121 -0.348357791 0.089465528 0.32338612 0.216011485

26122 0.095908746 0.25720522 -0.114084939 0.01054221 -

26123 0.407490581 0.014622062 -0.019469116 0.142770315 -0.11798603

0.274495107	0.060122968	0.274236967	0.264204377	-
0.137097107	-0.087085116	-0.031977397	-0.078234222	-0.284815591
-0.263630457	-0.227566569	0.1150935	-0.258462149	-0.069339797
0.034941405	0.139646502	0.107395983	0.271847739
0.114245585	0.047733936	0.153725183	-0.086094693
0.165373131	-0.02421733	-0.038327802	-0.165279584
0.282930734	-0.046830426	0.29325094	-0.191291005
0.16748437	-0.176452143	-0.241834779	-0.197741197	-
0.051560967	-0.104251069	0.007065802	-0.087414263	-0.066892747
-0.004065366	-0.161885435	-0.004947379	0.201749154
KEGG_LEISHMANIA_INFECTION	-0.397299795	0.403150868	-
0.475048819	0.102073817	0.180409252	-0.05408433	-0.102101784
0.331520764	0.521851727	0.35513824	0.201027251	-
0.46793583	0.07134004	-0.617740152	0.252848889	-0.405903596
-0.20792244	0.500847893	0.051398739	0.520436216	-
0.406358438	0.525057749	0.192205912	-0.290907729	0.453833667
-0.170404117	-0.394260652	0.042281024	0.145556597	-
0.318334404	-0.456892887	0.526764511	0.268518798	0.487427907
0.529553845	-0.140186382	-0.418571216	0.217295643	-
0.455257922	0.266346455	-0.465905785	0.329695637	-0.224246905
0.403757246	-0.141557872	0.094823275	-0.309286992	-
0.417685356	-0.307875787	0.085379131	-0.390850891	-0.317461332

0.351962219 -0.395444401 0.145964924 -0.321012835 -
0.305588526 0.094525372 0.040137323 -0.081445011 0.10323212
0.47154166 0.203383459 -0.285401805 -0.40639282
0.081755049 0.432945081 -0.429084149 0.25592842
0.297562741 0.524236457 0.679652739 -0.480163898
0.507478541 -0.435928406 0.293782845 0.355961069 -
0.324809059 0.403446037 -0.403342037 -0.401227457 0.399082056
0.49828075 0.124830743 0.381491603 0.34133209 -
0.434409599 -0.225215468 0.537720204 -0.252183916 -0.435527844
-0.177687497 -0.034113368 0.352755938 0.474563015
0.38775794 -0.293454657 0.51562372 0.003923013 -
0.3426183 0.413993964 0.03915708 -0.013767138 0.52313969
0.034418409 -0.050750077 -0.311838296 -0.463668832 -
0.057691479 0.444752112 -0.304923513 0.144711811 -0.013166666
-0.395150501 0.438408212 0.469706208 0.017856209 -
0.149102742 -0.294772763 -0.371364101 -0.17794296 -0.312781578
-0.061000903 -0.5550348 0.136896187 -0.200196235 -0.475989012
0.313011725 -0.147871402 0.495982514 0.341706014
0.391226056 -0.568749029 0.463532982 0.164357572
0.282984308 0.057180557 -0.255373559 -0.019565488 -
0.179751177 -0.191782396 -0.051723802 -0.306724949 -0.048412291
0.435106761 -0.509690519 0.527938417 0.210703873

26168 0.227279788 0.331301252 0.484798041 0.350246846

26169 0.603374923 0.508848796 -0.153444367 -0.363918006

26170 0.044012907 0.409656401 -0.156338301 0.514913494 -

26171 0.219682576 0.136740934 -0.047158514 -0.177388263 -0.251297346

26172 -0.095444743 0.389566306 0.433696573 0.1694746 -0.056942491

26173 -0.354626406 -0.533226487 -0.175561607 -0.285201534

26174 0.441557617 -0.37708554 0.208837955 -0.372911257

26175 0.068929033 0.563621708 0.318409092 -0.326955964

26176 0.32129445 0.218805158 -0.163918789 0.312598233 -

26177 0.583649608 -0.019448377 0.494186341 0.023694577 0.133973141

26178 -0.053639681 0.013128723 -0.501549692 0.441558553 -

26179 0.10256029 0.286899963 0.248787665 -0.433843483 -0.210080643

26180 0.119952898 0.271185161 -0.121812049 -0.427322961

26181 0.17554131 0.102900734 -0.387873046 -0.075636539 -

26182 0.462402974 0.252578216 0.268665199 -0.357729331 -0.084177444

26183 0.25497899 -0.164808198 0.260763191 0.223979934

26184 0.342589803 -0.249647865 0.429568116 0.545126541 -

26185 0.293850594 -0.327785966 0.191617565 0.270178781 0.172010834

26186 0.291401797 -0.204201804 -0.236743634 0.330251479 -

26187 0.356204954 0.52652059 0.074347906 0.354833841 0.061131967

26188 -0.316030332 0.464177305 0.051523554 0.404648087

26189 0.141282396 0.083646678 0.007077776 -0.501545114

0.173743469 -0.558662371 -0.077006634 0.105592623 -
0.322916708 0.241932152 -0.135096868 -0.273156757 -0.099777104
-0.13772471 0.048993915 -0.240838457 0.227165611 -
0.342123933 -0.188468121 0.166328466 -0.255792977 0.240426557
0.474135053 -0.082010455 -0.442463727 0.401578044
0.060493772 -0.014238927 -0.387168487 0.404081212
0.309554332 0.178238422 -0.201825224 0.444486555
0.347459256 -0.18930031 0.43753715 -0.234959884
0.364078254 -0.536760293 0.323056036 0.204718977
0.467223481 0.254932101 -0.221000901 0.315051059 -
0.220725375 -0.029194245 0.478411743 0.376785219 -0.450780398
-0.272176793 0.514861981 -0.342413311 0.446329326
0.309181387 0.382021138 -0.278468396 0.230874719
0.168264654 0.483498815 0.445832892 -0.492507116
0.167690654 0.047385144 -0.241968805 -0.180807209
0.284230221 -0.187876415 0.403614891 0.227308371 -
0.299619191 -0.231575196 0.438300493 -0.283919868 -0.100758505
-0.411138628 -0.168187768 -0.058018292 -0.265874827
0.193750469 -0.515598861 -0.226544008 -0.009506521
0.17589548 0.30735301 0.285184512 -0.28989701 -
0.528167928 -0.049527804 -0.46006868 0.044979434 -0.521331095
0.30305754 0.339417796 -0.242121559 -0.121775341

0.213907779 0.357721018 0.518371759 0.310835068
0.468023361 -0.307385196 0.269526181 -0.225424235 -
0.198994831 0.340327688 -0.178167436 0.038636853 -0.346349327
0.068124419 -0.184104637 0.472595312 -0.258769701 -
0.227738133 -0.504095973 -0.090202108 -0.341820816 0.145545338
-0.181928295 -0.38095632 -0.412757971 -0.518626935 -
0.119131077 0.509535339 0.509738489 0.244254646 -0.072248692
-0.220132984 -0.023242271 -0.323371612 0.325301289 -
0.188571122 -0.466348572 0.49048728 -0.050451797 -0.377521194
-0.255040984 -0.028224351 0.579330533 0.468636451 -
0.481905074 0.153275329 -0.224265695 0.123482499 -0.142417309
-0.288346825 -0.080399809 0.500204158 0.366483721 -
0.054023249 0.300535806 0.453439136 0.085912736 0.419007569
0.305453905 -0.284525302 -0.01818958 -0.366839318 -
0.295558167 -0.012331862 0.333772797 0.361776723 -0.033266918
-0.446311795 0.275503992 0.260586116 -0.184942685 -
0.092186139 0.270186231 0.011788186 -0.260504298 0.122984996
-0.3967301 0.110430151 -0.108574468 0.533379487 0.403249273
-0.107733315 0.535287155 -0.037698036 0.511243269 -
0.30937504 -0.615404249 0.341673486 0.259650201 -0.259423409
-0.366758262 0.531313239 -0.106083539 -0.528692987
0.05458798 0.419375685 -0.048169892 -0.367924314 -

0.47124735 0.457537531 0.441508989 -0.531823183 -0.384646064

-0.57681537 -0.239052663 -0.473812589 0.422374049 -

0.095537284 0.116533571 0.342344851 0.214360188 0.265599751

-0.562912804 0.236727403 0.518036596 -0.517421556 -0.428188

0.13582531 0.429743402 -0.126395057 0.047860301 -

0.442229802 -0.438758764 0.011230336 0.051812103 0.53304082

0.346451457 -0.233986352 0.188477975 0.041019926 -

0.205503704 0.386730505 -0.339416711 0.000946879 -0.38577482

0.055615555 0.226501946 -0.113036974 -0.502512061

0.088213944 -0.268547914 -0.312335323 0.347156267

0.186136503 -0.287007251 -0.247486036 0.378642927

0.131285326 0.226477991 0.041230506 -0.141540114 -

0.174460696 -0.330474861 -0.492191006 -0.37499419 -0.386361269

-0.01788626 0.403713744 -0.293605109 -0.474886567 -

0.51880955 0.472662608 -0.203807302 0.511013632 0.355527634

-0.390594755 0.022235767 -0.102360599 -0.10754488

0.357674064 0.466000249 0.278609472 0.314826689 -

0.102259608 0.274852373 -0.236146225 0.303062172 -0.428291478

-0.490645597 0.195228311 0.018921682 -0.115953233

0.189875187 -0.00855231 -0.367449315 -0.537858206

0.369586416 -0.427892018 -0.283158589 0.062821102

0.360390396 -0.083509789 0.109697433 -0.082936646 -

0.221481505 -0.535480306 0.294907714 -0.250067819 -0.425524975
-0.08790345 -0.481570641 -0.360107591 -0.242791739 -
0.349237866 -0.204794635 0.036165561 -0.082658718 0.406682782
0.337346574 0.144066395 0.008079165 -0.035683941
0.163538768 -0.386422909 0.157086384 -0.041572103 -
0.129253951 0.480912897 0.091224729 -0.231094294 -0.070232947
0.166635369 -0.303508141 0.388346034 -0.266585642 -
0.197877886 -0.059854946 0.424544706 0.011405873 -0.329169015
0.001929173 0.064750129 0.196129985
KEGG_PATHWAYS_IN_CANCER -0.125703065 -0.025463477
0.008622949 0.133236183 -0.159673674 0.006152804 -
0.092909751 -0.088379314 0.087732418 -0.040981585 -0.053126905
-0.183697234 0.020041548 -0.015790132 -0.171171323 -
0.145318006 0.14400796 -0.018873522 0.032650632 -0.109417885
0.158860223 0.104896129 0.194055793 0.210524868
0.081912399 -0.137814881 -0.347908214 -0.024279771
0.035127559 -0.137634881 -0.135768558 0.017071425 -
0.139834014 -0.140075311 0.241349039 0.245551102 0.078937918
-0.087923626 -0.045227071 -0.076506337 -0.126985464
0.214808553 0.140777961 -0.079750749 0.057558812
0.061900656 -0.011148416 -0.108947007 0.009038581 -
0.144227784 -0.173772537 0.112194764 0.184099023 0.102663165

0.119320755 0.004246586 -0.06227695 0.172606104 -
0.017441508 0.301664265 0.126051872 0.274851043 0.102008639
0.135378357 -0.148979019 -0.019089311 0.197044611
0.106474331 0.016383981 -0.189454914 0.204177119
0.256448581 -0.013405513 -0.043548409 -0.13985653 -
0.085129959 -0.112882884 -0.117551191 0.032929725 -0.278405094
-0.150517514 0.306824496 -0.000936321 0.002292684 -
0.04041862 0.072614973 0.170870574 0.122680599 0.036260991
-0.252599031 -0.08301917 -0.082515678 0.049655165
0.062139196 -0.055358665 0.010148537 -0.001492495
0.125906614 -0.099512604 -0.114384604 0.021982847 -
0.115096578 -0.029671644 -0.092591039 0.047327915 -0.003611146
-0.023844496 -0.325183857 0.221624156 -0.087419461
0.018777852 -0.220511524 0.039588943 0.213946922
0.081289762 0.006054989 -0.182139475 -0.264875765 -
0.181986939 -0.131129851 -0.1860105 -0.149602032 -0.032488511 -
0.081827819 0.109866621 -0.196500333 -0.301748793 0.256743312
-0.017242862 0.064110353 0.078582559 -0.068259005 -
0.154272996 0.046978735 -0.125433892 0.242582207 0.316572912
-0.00424169 0.012802247 0.223854296 -0.080151769 -
0.163653325 0.040423689 0.017628961 0.084324354 0.083773829
0.111146143 -0.190989899 -0.077710076 0.032751213

0.070881786 0.253628934 0.229578757 0.192088296 -
0.047474708 -0.083183465 0.161757584 -0.251737142 0.00074409
0.061201606 0.161954332 -0.163292578 0.123408943 -
0.043006711 -0.099048361 0.125439964 0.106045228 0.1876649 -
0.094187962 0.003073218 0.025053498 -0.207242735 -0.12142429
0.065522486 0.290098623 0.271128093 0.061719126 -
0.161637104 0.07730828 0.168848283 -0.135201937 -0.007896806
-0.172136314 0.097944874 0.185153185 0.084940013
0.112177994 0.022178382 0.126235129 0.104574756 -
0.094135405 -0.288759561 0.049797505 -0.169093175 -0.292929004
-0.054690311 -0.159325824 0.021719594 -0.125584741 -
0.048191682 0.087916192 -0.091596531 0.08720348 -0.176918775
0.077134888 -0.256143188 -0.045317951 -0.166895855
0.200721484 -0.16883505 -0.018343739 0.063472137 -
0.038789815 0.107514803 -0.154147093 -0.069342257 -0.173204824
0.096326656 -0.079469045 0.087522649 0.179108392
0.008076476 -0.069213551 0.036821501 -0.014719114 -
0.052068171 -0.035151613 -0.205302511 0.195712312 0.008249718
-0.24532483 0.01207964 -0.094743115 0.157553353 -
0.074394578 0.173637313 0.150909636 0.084054502 0.141348167
-0.177809326 0.135692131 0.119387656 -0.169935082
0.129720036 -0.008999872 0.182918333 -0.045077955 -

0.082122819 -0.153715793 -0.205626133 -0.043134945 0.068305017
-0.078745813 0.304441085 -0.129653169 0.287825894 -
0.031535112 -0.091550103 0.024800258 0.024995073 -0.05489101
-0.005112408 0.013630268 -0.01444167 0.058949399
0.13101851 0.157127467 -0.12211773 0.019645317 -
0.179780476 -0.287415285 0.070682877 0.120684128 -0.012546872
-0.255069327 -0.134872337 0.037155872 -0.014082908 -
0.135118976 0.093319835 0.039352214 0.040598598 0.009606947
-0.034354033 0.03731588 -0.253348992 -0.099345442 -
0.082723256 0.29362951 -0.043430857 -0.039071222 -0.028444211
0.098005306 0.297323641 0.03069254 -0.078748511
0.08761249 0.027035791 0.015313815 -0.126277403
0.069849268 -0.294795924 -0.10779109 -0.085890236 -
0.2545985 0.102210443 0.186464793 0.076366765 0.243687271 -
0.177216575 -0.009763121 -0.210355418 0.096669781 0.16692403
-0.133014886 -0.036793372 -0.024590145 -0.058418523 -
0.040083388 0.114040499 -0.069239136 0.026699434 0.08429292
-0.096162476 -0.176972411 0.168748917 -0.138094744 -
0.280767706 0.111934358 0.079790461 0.022720827 -0.069382933
0.083863725 0.002630769 0.321558501 -0.000402039
0.17584534 0.230204072 0.102912663 0.151331067 -
0.147415378 -0.12117148 -0.18720871 0.147115675 -0.082306922

-0.125417369 0.125607133 -0.040500866 0.038333444
0.071531305 0.207778639 0.05873174 0.145451683 -
0.359010939 -0.058126615 -0.237523505 -0.130477865 -0.084585759
-0.269405191 -0.267580488 -0.159228958 -0.075312919
0.007395237 0.254478969 -0.067017439 0.301360324 -
0.092328452 0.137739228 -0.13702134 0.144878513 0.170865574
0.308616448 0.220648435 0.226360248 -0.006047336 -
0.1653517 -0.155005437 -0.108297669 0.227168992 0.102577851 -
0.166407656 -0.061409933 -0.112173814 -0.143824877 0.063254482
-0.105227854 0.054007406 0.216474376 0.210551452
0.128214966 -0.19010457 -0.014353979 0.004163593
0.126489234 0.208982722 0.247217174 -0.060687504 -
0.119029653 0.192322983 0.170436768 -0.047211967 0.087903291
0.232049927 -0.19112024 0.018774833 -0.124666919 -
0.03327979 0.105250087 0.098573056 0.015238271 -0.204773941
0.131141601 -0.300501122 0.051700098 -0.074873931
0.075475164 0.056552481 -0.2309647 0.260049791 1.60E-05 -
0.011335588 -0.00550482 -0.13276136 0.307251919 -0.004663438
-0.004733973 -0.309216145 0.311170453 0.132814405
0.03660346 -0.203495342 0.051594186 0.092736758 -
0.21842577 -0.115000934 0.240375519 0.185460306 -0.149550662
-0.077565439 0.156880626 0.120887603 0.166280083 -

0.098942588 0.050755737 -0.056320005 -0.075313067 -0.040835088
-0.080296435 0.048325046 0.071993795 0.043593804 -
0.192438701 -0.065406252 -0.138172136 -0.220789983 0.172648634
0.082506357 0.082907444 0.037216051 0.115645018
0.092150984 -0.003998692 -0.060395697 0.183371609
0.119052836 0.01111656 -0.075491297 -0.069438436 0.115566895
-0.093815582 -0.1337744 -0.108660566 0.096641865 0.241249608
0.013611165 0.053608723 -0.047100512 -0.098428126 -
0.061566508 0.080663256 0.165791533 -0.062323939 -0.04991612
-0.079128452 -0.151604527 -0.199614737 0.077481502 -
0.094172039 -0.129258651 0.155920694 -0.075060815 0.015008794
0.21593975 0.023557019 0.005241702 -0.219325888 -
0.137893154 0.058243974 -0.097462946 -0.09680329 0.040892914
-0.00279325 0.003795733 0.169630118 0.331200018
0.22192878 -0.034146855 0.162160468 -0.057918178 -
0.039161872 0.065784415 -0.182398626 0.290938162 -0.170440568
-0.065392542 0.010279301 0.104172132 0.030566562 -
0.025109595 -0.137304504 -0.182081377 -0.086529027 0.238905732
-0.143439546 -0.059996162 0.130337041 0.141924998 -
0.055056832 0.283420652 0.174638993 -0.135391 -0.094607556 -
0.047603225 0.066916347 -0.173761216 0.009729132 -0.012123966
-0.023941424 -0.004520798 0.051990726 -0.075668576

0.108086901 -0.071173991 0.046388046 0.036716569 -
0.129850643 -0.008107877 0.011024937 0.119867546 -0.227582662
-0.092931797 0.087029524 0.204730717 0.040542787 -
0.042031993 -0.036203457 0.085638376 0.02333621 -0.114439151
-0.061271037 0.0621985 -0.099216346 0.09921055 -0.045239729
-0.096871285 0.044875339 -0.011632662 -0.112895658 -
0.070581753
KEGG_COLORECTAL_CANCER 0.130177442 0.271855904
0.228283444 0.261624483 -0.398686492 -0.004101347 -
0.04822786 -0.353105697 0.060262958 -0.195214734 -0.014367824
0.014142743 -0.178406438 0.046485623 -0.430853477 -
0.27687621 0.358476972 0.024508995 -0.166542758 -0.126568102
0.372322759 0.225863348 0.186906032 0.404201485 -
0.116308775 -0.229819688 -0.483107048 -0.038000083 0.03152933
-0.327436233 -0.063627669 0.142801853 0.035409255 -
0.311069035 0.219792527 0.024808061 -0.10005632 -0.330617076
-0.023138567 0.161262743 -0.163749924 0.174616195
0.302354392 0.175276512 0.047662176 0.217123669
0.077869001 0.012042678 0.202861898 -0.248379312 -
0.220213234 0.198702554 0.347481135 0.244618256 0.203203517
0.237363907 -0.351054973 0.10575696 0.191492169
0.271510009 -0.007348464 0.373624936 0.156908561

0.256426268 -0.225186982 -0.085598683 0.259383452
0.201350903 -0.173141525 -0.447026848 0.255932166
0.28375522 -0.175382878 -0.151774877 -0.161715535 -
0.303412385 -0.147133526 0.035313959 0.044063298 -0.329932999
-0.176107426 0.33038367 0.099161347 0.020283151 -
0.138637933 0.094330608 0.321169124 0.207328333 -0.19183063
-0.119572541 -0.277559685 -0.224706998 0.223951636
0.029324471 -0.068718668 0.147296054 0.16637837
0.063411641 -0.077999458 0.109293906 -0.106509847 -
0.324407689 0.126079878 -0.146223205 0.04356719 0.033737624
0.120047304 -0.183054088 0.2807066 -0.223337011 -0.368152249
-0.268726184 0.083358817 0.269617661 0.25287136
0.090110152 -0.292493566 -0.475546987 -0.49770041
0.017836181 -0.441094437 -0.10669817 0.01711526-0.248169279
-0.093048138 -0.39460429 -0.311392567 0.232949376
0.18453292 0.231750056 -0.087448022 -0.24577523 -
0.157849046 0.234206976 -0.11968008 0.346140659 0.403966348
0.375546254 0.096523336 0.298213148 -0.193744769 -
0.195902183 0.377041285 -0.083184729 0.298551975 0.154284848
0.211707898 -0.224581242 -0.327276193 -0.205384126 -
0.016843783 0.342896993 0.195956929 0.124261706 0.297096305
-0.293875864 0.073958735 -0.176153091 0.269839014 -

26432 0.074399587 0.307430916 -0.199091279 0.023018761 -0.238164096

26433 -0.078613935 0.191572911 0.077055866 0.253573547 -

26434 0.352479306 -0.047030864 0.258166855 -0.274092502 -0.284644213

26435 0.163694434 0.375925702 0.298122315 0.256348454 -

26436 0.056500236 0.242643232 0.227411961 -0.329333822 0.076177822

26437 -0.165988066 0.23047838 0.380297185 0.314378298

26438 0.236835111 0.121564438 -0.169205719 0.091173288 -

26439 0.018339775 -0.346164682 -0.093088135 -0.324775698 -0.264897518

26440 -0.244651808 -0.231317653 0.183117356 -0.320242122 0.2245769

26441 -0.071557792 -0.006815003 0.249697564 -0.381244433

26442 0.011912864 -0.246903133 -0.259342784 0.085399455

26443 0.342727317 -0.129240256 0.165414314 0.162812793

26444 0.098483796 0.121695429 -0.37098098 0.108970558

26445 0.088438347 0.282483507 -0.217544113 0.204570072

26446 0.140878233 0.093895176 -0.331955758 0.177654636 -

26447 0.034403932 -0.046214786 -0.122553923 -0.343373697 0.329369526

26448 0.021605142 -0.385385486 0.102922984 0.019032848

26449 0.161199992 -0.14370615 0.298525418 0.04138547

26450 0.27284737 0.245590783 -0.119352928 0.257120112

26451 0.150574591 -0.1135355 0.250627598 -0.254690005 0.269444605

26452 -0.142888925 -0.181819792 -0.224095228 -0.329323725 -

26453 0.096698446 0.05525924 -0.349570027 0.239982068 -0.327379564

0.3638786 0.178082271 -0.214385314 0.272891766 -0.007929503
-0.004805696 0.12241189-0.252982646 -0.195730812 0.09244743
0.171384315 0.073837087 -0.143608962 0.159984978 -
0.410304116 -0.196551071 0.129447763 0.021630106 -0.007884289
-0.177640925 -0.083395855 0.059397631 -0.098539527 -
0.251409517 -0.044848339 -0.192424356 0.033200372 0.09753948
-0.325659234 -0.139426466 -0.389979655 -0.367938142 -
0.109816343 0.158921089 -0.20003956 -0.059430858 -0.181536033
0.128574532 0.393190589 -0.215165588 -0.33919362
0.250048459 0.173883142 -0.097788562 -0.261853976
0.223832949 -0.284796898 -0.308526137 -0.073705097 -
0.418210671 0.152790906 0.30047037 0.290209646 0.258967486
-0.447924333 0.015050935 -0.351872122 -0.103422872
0.274097198 -0.071450658 0.149007376 -0.247422515
0.306155139 0.207558401 0.382154738 -0.214451002
0.137693291 0.309044833 -0.360687312 -0.158016754
0.036012724 -0.171015557 -0.454511159 0.317689315
0.063542012 -0.194881524 0.059254837 -0.05394292
0.01820335 0.335859714 0.059512758 0.407499491
0.239958677 0.306117857 0.325290655 -0.16415227 -
0.015612872 -0.409422793 0.152720263 0.196932648 -0.147811918
0.163865764 -0.005417776 0.136537479 0.028960549

26476 0.205292832 0.085861991 0.307634616 -0.358138874
26477 0.087655077 -0.459864301 -0.304307087 0.166673883 -
26478 0.273968473 -0.362420944 -0.218535228 -0.210585479 0.26253296
26479 0.268342474 0.001232732 0.384466465 -0.150539264
26480 0.408734789 -0.210190504 0.268370421 0.0054026 0.484598238
26481 0.422159812 0.216039664 0.242125058 -0.332103494 -
26482 0.340787239 -0.168085291 0.230085514 0.337095873 -0.384017271
26483 -0.339079816 -0.081939865 -0.347159812 0.067550116
26484 0.083093042 0.058748874 0.274002506 0.402181868
26485 0.276641036 -0.096592318 -0.125884917 -0.156952258
26486 0.127091519 0.213926629 0.332704025 -0.089669918 -
26487 0.309887835 0.16373495 0.282490391 -0.061089203 0.256658127
26488 0.329052213 -0.070596365 -0.138182542 0.12212976 -
26489 0.134527682 0.253661716 0.13821584 0.197396113 -0.010726656
26490 0.20773669 -0.504986271 0.015381278 -0.305441734
26491 0.037403499 0.176229867 -0.08014928 0.225898299 -
26492 0.005924713 0.174069854 0.045978245 0.055621551 0.519137084
26493 -0.237581534 -0.021143948 -0.543546429 0.440484651
26494 0.065945608 0.256496852 -0.373927519 0.143144725 -
26495 0.159732305 -0.43119217 -0.119541721 0.280371862 0.442675899
26496 -0.151807115 0.048705304 0.279623933 0.293811291
26497 0.272258301 -0.0539174 -0.226023926 -0.085874397 0.033806655

-0.034715718 -0.29719594 -0.050099268 -0.071053439
0.133567117 -0.27036124 0.082643065 -0.18292624 -
0.291935226 0.215743358 0.157495354 -0.016077385 -0.073515703
0.221781227 0.072374864 0.087043515 -0.256096467
0.055067067 0.276361353 -0.127882342 0.267138888 -
0.226418638 0.312653507 -0.25946283 -0.121494594 -0.22868768
0.265958383 0.343011038 -0.137655151 0.075466138 -
0.112909585 -0.042107283 -0.081518308 0.123252153 0.356944007
-0.252918797 0.043935775 -0.177600936 -0.271622365 -
0.035420219 0.242429424 -0.237629908 -0.107397077 0.267765721
-0.258397422 0.015872792 0.350028038 0.13929026 -
0.094394576 -0.357684904 -0.318473152 -0.071576384 -0.156892285
-0.123054189 0.071508055 0.22398868 0.041615415
0.214540877 0.374808297 0.216352542 -0.184819022
0.320551136 -0.357062411 0.098847225 -0.130836454 -
0.130603653 0.350560287 -0.292965969 -0.185962059 -0.030040036
0.165211396 0.211287621 0.209433409 -0.068635406 -
0.023228452 -0.354761014 0.19408025 -0.078892389 -0.009500243
0.127597623 0.130663271 0.035754779 0.367384923
0.35080329 -0.119654333 -0.184208323 0.12424971
0.034775732 -0.362502193 -0.169331455 0.050663358 -
0.093688995 -0.080166272 -0.039648558 -0.066434318 0.050297304

-0.045449817 0.142798017 -0.143257876 -0.267816323
0.067131517 -0.113030997 -0.081897033 -0.346438126 -
0.00166255 -0.026305638 0.082980237 -0.089290549 -0.175600355
-0.20677555 0.335674785 -0.168898951 -0.133299852 -
0.044901672 4.77E-05 -0.000899786 0.094133317 0.17912181 -
0.149244672 0.045190358 -0.174659437 0.047700839 -0.280973686
KEGG_RENAL_CELL_CARCINOMA 0.147980094 0.347348139
0.138206479 0.350021974 -0.437002345 0.092210818 -
0.010919705 -0.290923459 0.010497886 -0.340406672 -0.106098002
-0.222211466 -0.087047804 0.032196767 -0.457034025 -
0.364583556 0.337895664 0.119936709 -0.279401049 -0.13249643
0.428086403 0.35000275 0.080358676 0.477478983 -
0.064383717 -0.060012504 -0.396812227 0.019173347 0.024126656
-0.428735338 0.060031513 0.112964707 0.261968316 -
0.29637927 0.22037656 0.194180756 -0.143720525 -0.149512288
-0.081773489 0.252262158 -0.09002746 0.251483719
0.283769196 0.310862086 -0.053530646 0.108871206
0.016335637 -0.087850886 0.089723487 -0.261070218 -
0.142251139 0.17721564 0.280820159 0.144329537 0.389619962
0.286233501 -0.215938885 0.303767218 0.229209341
0.413977573 -0.012424638 0.197717403 0.325509686
0.289625924 -0.26422223 0.057900756 0.104296077

0.075228555 -0.160247116 -0.366321593 0.212550715
0.193649689 -0.232896067 0.072231862 -0.230181435 -
0.372127787 -0.284300749 0.209018419 -0.256377628 -0.019397044
-0.245611096 0.339495558 0.141755639 -0.007529814 -
0.067576079 0.102438244 0.327450685 -0.082311541 -0.229180806
-0.153682469 -0.478810695 -0.121079025 0.281005668
0.312374775 0.142979104 0.103552898 0.059005894
0.051406568 -0.087506263 -0.08271805 -0.236232035 -
0.358353959 0.056765771 -0.065765032 0.169351396 -0.00409336
0.227227274 -0.333731725 0.37806424 -0.288133266 -
0.239235808 -0.246180309 -0.032256878 0.311537520.293868752
0.179076584 -0.299115119 -0.479583663 -0.480130615 -
0.082535102 -0.391802295 -0.017830707 0.003552848 -0.268218815
-0.034512153 -0.372741717 -0.375225273 0.284913603
0.235339628 0.315419165 -0.28059805 -0.386035275 -
0.233100433 0.226971278 -0.050782896 0.000589442 0.233542542
0.34879304 0.04524599 0.373385195 -0.16618095 -
0.106240367 0.327322979 -0.12155364 0.328397919 0.063417509
0.310130884 -0.189169832 -0.371573788 -0.141699232 -
0.062910932 0.240307742 0.090084422 -0.074590476 0.242864885
-0.254273474 -0.04592967 -0.231691637 0.110676136 -
0.102402353 0.268245848 -0.220037227 0.068432563 -0.247788963

0.087756611 0.134939432 0.321191485 0.264315663 -
0.396795275 -0.185133639 0.248621626 -0.323440468 -0.266246178
0.224402066 0.433320155 0.286465706 0.193566782
0.05658274 0.114141497 0.273595742 -0.28738587
0.077252589 -0.206252531 0.228716608 0.364374177
0.360879293 0.081748139 0.139768511 -0.231302738
0.259425932 -0.049799307 -0.349543235 0.237883286 -
0.304352092 -0.313261823 -0.117989268 -0.287294723 0.10646218
-0.271111874 0.315288938 -0.0432936 -0.019949883 0.17477298
-0.415058937 0.166091794 -0.292022901 -0.214312096 0.0792204
0.409977605 -0.140617988 0.195964009 -0.026960284
0.113348744 0.2114874 -0.362557294 0.127768452 0.191423469
0.220342679 -0.192206527 0.236830479 -0.009839196
0.253086866 -0.140442126 -0.010099204 -0.16662265
0.130910454 -0.053646666 -0.430040242 0.461620609 -
0.128720929 -0.412581168 0.250742958 0.141855734 0.042608274
-0.073825755 0.260106325 -0.182516751 0.305981346
0.244087778 -0.176001403 0.082014452 0.238073455 -
0.165507513 0.250294785 -0.377108564 0.284275686 -0.040985576
-0.009109885 -0.349401224 -0.336114664 -0.028941144
0.226796223 -0.223305643 0.266420115 -0.183659364
0.229090554 0.158718164 -0.297765686 0.454669065

0.027261798 -0.17123337 0.30344084 -0.205566302 -
0.306688091 0.25756585 0.264226588 0.217581159 0.009514654
0.259807906 -0.422935165 -0.269332552 0.150223328 -
0.035763484 0.042237913 -0.289310155 -0.055619602 0.203554639
-0.01717551 -0.279400481 -0.308929353 -0.391571631
0.042168784 0.154290337 -0.239989939 -0.313516365 -
0.301329566 -0.433246071 -0.018548225 0.197407985 -0.198165262
0.062203446 -0.174826159 0.298605085 0.452722643 -
0.308306188 -0.366224819 0.301588874 0.057085632 -0.103950467
-0.247216789 0.263634022 -0.414065012 -0.355172394 -
0.170592718 -0.339281333 0.214224265 0.133604165 0.27978143
0.071277611 -0.409507958 -0.040350054 -0.282246849 -
0.249242476 0.458447981 0.021006836 0.096448829 -0.319887453
0.179323833 0.206322961 0.427007023 -0.279254034
0.191641624 0.256388385 -0.322441536 -0.054647476
0.144332479 -0.159285573 -0.520038488 0.270731609
0.014422007 -0.12401494 0.239465159 -0.02178605
0.071086368 0.407308589 0.310136812 0.44809827
0.337026045 0.348858273 0.375030182 -0.081041942
0.163879949 -0.39821984 0.190277823 0.203348472 -
0.210777514 0.318228824 0.340894611 0.056204225 0.171622402
0.062043809 0.012468985 0.325060566 -0.254126894

0.246390196 -0.45129436 -0.453103701 0.07901871 -
0.259096218 -0.397143386 -0.075908674 -0.216540719 0.280597277
0.240676273 0.080821226 0.403495636 -0.23947719
0.372328829 -0.218532471 0.264724316 -0.048988024
0.526848059 0.483258231 0.295050988 0.310223006 -
0.330405393 -0.342287279 -0.211508051 0.244936103 0.350181261
-0.349820585 -0.249636776 -0.158321966 -0.402402008
0.052176611 0.036770948 0.146308741 0.281610132
0.387768156 0.252607024 -0.074657787 -0.089195095 -
0.193057917 0.235115772 0.285916975 0.33796876 0.027889785
-0.277614806 0.26734992 0.312840061 -0.177536186
0.289888942 0.445479372 -0.177699441 -0.034121899
0.016054969 -0.215375013 0.277453106 0.162675683
0.191745999 0.056362912 0.265228423 -0.506273814 -
0.133154212 -0.317239208 0.219350011 0.200159177 -0.048672119
0.197302422 0.092971934 -0.039716287 0.204276612 -
0.168074554 0.496775539 -0.048607411 -0.262993156 -0.470867293
0.333366153 -0.172587509 0.225444856 -0.439791356
0.427255958 -0.057579263 -0.394624858 -0.339954088
0.233877191 0.49761424 -0.042541025 -0.079695181
0.300994472 0.283084131 0.295323553 0.065773856 -
0.310035413 -0.159647317 0.042334562 -0.093135213 -0.244603532

-0.10018152 0.208993461 -0.030845901 -0.251369977 -
0.043809067 -0.122666243 -0.271085775 0.302714704 0.031977499
0.09345794 0.024235437 0.257048066 0.099174256 -
0.233397492 -0.357101417 0.197073391 0.267496453 -0.075297245
0.26420709 -0.353027541 0.362933886 -0.026201566 -
0.270233947 -0.204649481 0.323299443 0.26035851 -0.141311142
0.170372636 -0.004410602 -0.145439546 -0.130434503
0.151148551 0.284606354 -0.245333677 0.086538291 -
0.209288489 -0.244069504 -0.001496255 0.294160639 -0.300156486
-0.166245434 0.319615025 -0.177180665 -0.022908236
0.353690773 0.021471657 0.040948459 -0.380509454 -
0.298860856 0.006313939 -0.275035919 -0.314126579 0.18579554
0.375904098 0.183501608 0.267358938 0.360411657
0.26753797 -0.157891731 0.272840026 -0.198264418 0.1775919
0.103155815 -0.14119036 0.113161831 -0.449482234 -
0.044417862 0.01707494 0.308921462 0.264380248 0.293114584
0.046375136 -0.132539052 -0.323104566 0.354686011 -
0.109454408 0.199119177 0.044449968 0.421610334 -0.037593936
0.406162391 0.292704071 -0.28273088 -0.079140057
0.182983449 0.02302877 -0.284220376 -0.03790226 -
0.15366525 -0.078101382 -0.235001036 -0.243142656 -0.227260872
0.00655142 -0.146798915 0.106578997 -0.063807743 -

0.335067249 -0.093500283 0.031704186 -0.060841974 -0.154412143
-0.098580786 -0.105904079 0.250980275 -0.048810227 -
0.099147783 -0.261181458 0.371737246 -0.131361234 -0.082668342
-0.130422156 0.083761853 0.044188644 0.204961346
0.125720838 -0.193333991 -0.027835972 -0.189272704 -
0.153372158 -0.168474969
KEGG_PANCREATIC_CANCER 0.130500393 0.2650178 0.140115178
0.257765544 -0.39871061 -0.01388096 -0.315919828 -
0.289671978 0.101916176 -0.193374651 -0.032666187 -0.084372058
-0.204280687 -0.02771066 -0.443942858 -0.29514976
0.278536915 0.17539947 -0.272930386 0.087530535
0.167424541 0.382808012 0.090504481 0.452107564 -
0.137413702 -0.284119361 -0.466338842 0.13470102 0.090205057
-0.439326947 -0.072715146 0.31500526 0.035881967 -
0.120005251 0.228565287 0.140782029 -0.250710214 -0.211625084
-0.215874647 0.210361135 -0.147898058 0.279752519
0.321027497 0.341637041 0.06109704 0.128551481 -
0.053314901 -0.048791452 0.077355731 -0.226277238 -0.205137011
0.216958055 0.304831249 0.020356045 0.432531097
0.250239898 -0.416687414 0.209053493 0.235476516
0.266704756 -0.108542104 0.168651776 0.282042819
0.235916658 -0.342543977 0.053696838 0.27338372 -

0.034081534 -0.160889122 -0.404964481 0.416848879 0.318598331
-0.17860481 -0.060310954 -0.186608796 -0.316341301 -
0.161161133 0.240902367 -0.124601741 -0.310250374 -0.214176955
0.320807492 0.214235581 -0.130286105 0.009103212
0.023622588 0.213624284 0.120075953 -0.085385741 -
0.077191705 -0.33555875 -0.264999259 0.257457502 0.385486043
0.270906744 0.210932319 -0.056679613 0.206897245 -
0.031205813 -0.144368286 -0.103278322 -0.395537082 0.089703831
-0.0910929 -0.041775567 0.086327617 0.171355835 -0.312227418
0.286340808 -0.233946435 -0.297865977 -0.179474711
0.125015453 0.151817311 0.305479747 0.321460998 -
0.167526525 -0.44425746 -0.500865372 -0.131690583 -0.476985881
0.151063351 0.04733262 -0.233834052 -0.070765219 -
0.412481282 -0.31760525 0.304796497 0.216287099 0.248542559
-0.043780327 -0.217542856 -0.084701301 0.194458415 -
0.164684263 0.112201456 0.291608867 0.291979402 0.112108786
0.223954077 -0.077877408 -0.209045995 0.300963562 -
0.022536569 0.219077223 -0.008010519 0.317628468 -0.072229799
-0.400199665 -0.158460795 -0.038546933 0.18866015
0.095032233 0.022912741 0.214395492 -0.241876755 -
0.01336146 -0.128910912 0.220206298 0.072138734 0.266476344
-0.096365677 0.209807247 -0.307244215 0.162639856

0.174840167 0.341259379 0.271411145 -0.43977956 -
0.029233772 0.281037418 -0.370397115 -0.259217855 0.177426533
0.386591479 0.282472404 0.181684513 -0.146161786
0.191746981 0.293399337 -0.320650805 -0.115714383 -
0.048412482 0.160620906 0.395906993 0.355499276 0.210097898
0.136732548 -0.164472779 0.224129951 -0.085979587 -
0.355275934 0.030988844 -0.395411931 -0.147976875 -0.05144493
-0.255724233 0.154005624 -0.210018257 0.184144164 -
0.173209978 0.216579742 0.06405716 -0.352447885 0.05320989
-0.090106451 -0.242791807 0.095912537 0.292836487 -
0.008981199 0.2423382 0.096596761 0.050700175 0.328177354 -
0.394572194 0.226233344 0.153466792 0.227405409 -0.059934096
0.20679883 0.072063132 0.168338837 -0.319592898
0.176733239 -0.244299286 -0.079678839 -0.007614221 -
0.376633146 0.383402351 -0.013821372 -0.325625371 0.203060866
-0.060441567 0.240041773 -0.192181282 0.133700686
0.00624273 0.195117285 0.38733806 -0.245676775
0.07496222 0.292718941 -0.277158141 0.275452664 -
0.241816628 0.389505166 0.118361432 -0.128109464 -0.092988898
-0.309673622 -0.087103019 0.013783398 -0.36638685
0.33299614 -0.292764546 0.216554982 0.058247109 -
0.328292916 0.338685404 -0.168148804 -0.094903328 0.254214254

-0.140089279 -0.296700551 0.083291081 0.06308853
0.104242506 -0.24038253 0.293029426 -0.397724022 -
0.17631043 0.143585187 0.092306903 0.055167347 -0.321477341
-0.121796054 0.244042534 -0.161124583 -0.240106474 -
0.174074349 -0.388592775 0.199178128 0.162732447 -0.300067618
-0.34260196 -0.388014284 -0.429028649 0.214719124
0.134285965 -0.290555508 -0.019816785 -0.15213357
0.010944388 0.441007523 -0.193425215 -0.317132977
0.263806113 0.02001989 0.009424194 -0.265129857
0.200513365 -0.431211477 -0.438072418 -0.158157507 -
0.32859877 0.232481837 0.23677864 0.329993057 0.156815498
-0.366177118 -0.160285682 -0.276363735 -0.125576422
0.376440101 -0.072616724 0.217815273 -0.362873638
0.304902416 0.078694848 0.487937767 -0.307906879
0.175123418 0.311182646 -0.366890606 -0.093681863
0.179078363 -0.225506093 -0.465803009 0.236553502
0.130662412 -0.206022112 0.056758652 -0.033229657
0.129004047 0.437228619 0.242777456 0.349984235
0.252235726 0.419241936 0.28684537 0.068337444
0.064866311 -0.38715263 0.07790465 0.283490447 -
0.170355724 0.306737536 0.117531264 0.156024111 -0.012611106
0.206445433 0.171883336 0.217736645 -0.308390826

0.154283648 -0.46199962 -0.43148535 0.166962017 -
0.203183619 -0.36421037 -0.166981996 -0.221504593 0.379587122
0.195140076 0.079370187 0.341798108 0.009279603
0.376302895 -0.25879726 0.215007155 0.020279763
0.351103504 0.497801893 0.262493796 0.34180587 -
0.388648122 -0.368847251 -0.142947889 0.100506509 0.363817271
-0.433362054 -0.323803554 -0.159061905 -0.360170391 -
0.004243392 0.125193661 -0.001530831 0.292762683 0.384952737
0.379086517 0.088842956 0.125648064 -0.276954192
0.150458738 0.249794616 0.222485526 -0.111514736 -
0.372907212 -0.012192877 0.092792433 -0.090348175 0.345135421
0.29540244 -0.01469335 -0.164092569 0.122038545 -
0.087489029 0.220980952 0.339471675 0.238224277 -0.012472287
0.315001217 -0.480587256 -0.053422866 -0.264830313
0.140173471 0.323019894 -0.050265793 0.134472878
0.043727834 0.138157415 0.137516889 -0.079888208
0.430802951 -0.309396499 -0.217982629 -0.35299285
0.33697152 -0.084275779 0.257309077 -0.434362682
0.267625813 -0.068739196 -0.470935335 -0.31517702
0.232738122 0.482805332 -0.140702313 0.045027489
0.20680648 0.209571555 0.103106508 -0.047029131 -
0.377427616 -0.044153748 0.234614008 -0.216099669 -0.381211563

26762 -0.187778958 0.18258007 0.182003688 -0.381667878

26763 0.024481699 -0.238727653 -0.267037388 0.202176038

26764 0.239615519 -0.201949974 -0.054241916 0.124782796

26765 0.172897373 0.13651606 -0.339277199 0.161588027

26766 0.267936416 0.180709318 0.11173331-0.147599207 0.29203914

26767 -0.223912105 -0.165032627 -0.147874791 0.321255792

26768 0.304937794 -0.239398724 -0.009037835 -0.14169668 -

26769 0.066255008 -0.041838452 0.176290374 0.428178705 -0.298894605

26770 0.020675477 -0.169827524 -0.151959148 0.135718574

26771 0.337225187 -0.161529555 -0.091800646 0.258362023 -

26772 0.181739919 -0.136115982 0.423936133 0.310485251 -0.099047853

26773 -0.37404326 -0.338066793 -0.164461499 -0.139901381 -

26774 0.136513524 0.191782632 0.132680138 0.116045749 0.113507155

26775 0.268815355 0.204897065 -0.163494508 0.335342507 -

26776 0.327123803 0.118309137 0.036412413 -0.186733756 0.210991697

26777 -0.400411122 -0.137576558 0.197215245 0.259299185

26778 0.116122807 0.280465586 -0.172588371 0.020728921 -

26779 0.341554784 0.270984079 -0.357720817 0.147947221 0.026827975

26780 0.34474525 -0.029725999 0.411833958 0.385755249 -

26781 0.202816688 -0.15067252 0.093850168 0.148798519 -0.3717636 -

26782 0.195892103 -0.00822842 0.01798554 0.039770843 -0.112040753

26783 -0.243571469 0.117659076 -0.185857464 0.026802948 -

0.031875935 -0.305729601 0.10351307 -0.041083808 -0.0501357 -
0.259181352 -0.138026532 -0.143661697 0.262376569 -0.194705168
-0.006486201 -0.176485919 0.356199505 -0.125684596 -
0.144905064 -0.114435018 0.088135794 -0.129751712 0.111342083
0.145271183 -0.149748319 -0.038886076 -0.156239164 -
0.053439946 -0.200347423
KEGG_ENDOMETRIAL_CANCER 0.112977126 0.3281456 -0.04989108
0.29826549 -0.381380827 -0.015636171 0.04693224 -
0.363702738 -0.131099964 -0.325814345 -0.033580804 0.157716581
-0.321498934 0.134026252 -0.449616199 -0.300785757
0.352626335 0.205171312 -0.172659258 -0.127194946
0.504327424 0.208798693 0.038573887 0.351058979 -
0.330136126 -0.276924147 -0.464975543 0.116368565 0.122624759
-0.303637224 -0.115233436 0.040410435 0.197683902 -
0.404153672 0.033272326 0.146000888 -0.220649108 -0.195516706
-0.136611948 0.209556267 -0.052533463 0.244822297
0.330224468 0.211254346 -0.090170854 0.2297701 0.197318271
0.044663057 0.195582428 -0.23250822 -0.105074371
0.059931391 0.240423326 0.063067248 0.414507748
0.190944494 -0.344087289 0.350604508 0.27064926
0.387655202 -0.128890741 0.211095226 0.134654957
0.314301975 -0.251574542 0.118329021 0.068588269

26806 0.227718545 -0.335185781 -0.395712307 0.098244249

26807 0.090516291 -0.04933668 -0.275535248 0.012113191 -

26808 0.360450288 -0.281284702 0.151779548 0.067229269 -0.267113349

26809 -0.251174102 0.420781749 -0.084505799 -0.142533481 -

26810 0.22025688 -0.066794098 0.292304862 0.12754306 -0.21978671

26811 0.062468107 -0.362368351 -0.293764192 0.193210153

26812 0.194305697 0.114764675 0.127893602 0.211755863

26813 0.056903473 -0.053654083 0.259005823 -0.141861951 -

26814 0.491538948 -0.029888665 -0.197527191 -0.077843401 0.009459579

26815 0.159022137 -0.227908312 0.349444368 -0.337602615 -

26816 0.236756864 -0.159605556 0.060840584 0.252473423 0.179862295

26817 0.185648667 -0.233813835 -0.511045003 -0.437052152

26818 0.094601947 -0.422491911 -0.017229395 0.110711884 -

26819 0.108560852 0.088312667 -0.340167755 -0.254322295 0.216698556

26820 0.165864925 0.242315537 -0.165584953 -0.34656332

26821 0.001669877 0.140436809 -0.19227052 0.230703110.251414275

26822 0.477529456 0.190949267 0.372200169 -0.073289757 -

26823 0.245501524 0.333636013 0.038872747 0.151365065 0.156514355

26824 0.191062582 -0.191326815 -0.364473394 -0.17117253 -

26825 0.166822942 0.186073435 -0.040533473 0.073950537 0.177890153

26826 -0.282990174 0.027860422 -0.185200917 0.166469138 -

26827 0.219033581 0.230512179 -0.164799496 -0.075359818 -0.284048458

0.162807144 0.094792857 0.295289957 0.250901441 -
0.328534028 0.013641334 0.261581699 -0.332567305 -0.227632854
0.15927748 0.356384003 0.272418721 0.285912114
0.07945774 0.142035978 0.109363118 -0.279052941
0.189619076 -0.32578992 0.168706011 0.437228784
0.295265752 0.133235136 0.002775554 -0.287888716
0.206148173 -0.056092596 -0.463735709 0.023688054 -
0.348959731 -0.292608449 -0.101068968 -0.240859157 0.164310784
-0.340927878 0.330972619 -0.269631447 0.032874665
0.068773029 -0.205818432 0.142299344 0.016983624 -
0.258181894 0.139399918 0.33029878 -0.096294491 0.325886189
0.192006714 0.099613311 0.20556731 -0.328942786
0.246025322 0.094726127 0.266872573 -0.09359411
0.20586637 -0.022193632 0.368988985 -0.253610197
0.094853814 -0.240387489 -0.090156316 -0.232294109 -
0.414484196 0.404564228 -0.031539389 -0.310599963 0.054291662
0.090497057 -0.164030176 0.015258773 0.286423177 -
0.192208329 0.222262074 0.176430525 -0.266503511 0.086331359
0.258638341 0.032433732 0.177190419 -0.272823615
0.364688093 0.109529997 -0.116702719 -0.372522633 -
0.292367527 -0.08053758 0.263617477 -0.29450968 0.1751633 -
0.223873835 0.325388441 0.262701329 -0.27597174 0.403807263

26850 -0.014875854 -0.1322505 0.277600951 0.028141683 -0.278722074

26851 -0.018863253 0.237507441 0.100085173 -0.070103979

26852 0.352188727 -0.373016154 -0.282045793 -0.0434355 0.126380213

26853 -0.13928704 -0.198489059 -0.055681276 0.279977847 -

26854 0.131902041 -0.126962614 -0.15997819 -0.27239659 -0.08714475

26855 0.136407729 -0.327847967 -0.302045152 -0.295361191 -

26856 0.386141279 -0.013918721 0.171404641 -0.220281906 -0.108810265

26857 -0.095609055 -0.037998843 0.378638824 -0.289990859 -

26858 0.41288106 0.33846062 0.157203506 -0.164793478 -0.32905371

26859 0.128741309 -0.132641265 -0.393059654 -0.24083368 -

26860 0.336083278 0.120658897 0.121957735 0.35257383 0.079721261

26861 -0.441044895 0.006200646 -0.168090224 -0.176649601

26862 0.375583835 0.058806871 0.272931196 -0.341136231

26863 0.361541299 0.295501695 0.373327043 -0.120410028

26864 0.346437605 0.319432583 -0.404673171 -0.172219287

26865 0.005354937 -0.091709988 -0.375819553 0.352560822

26866 0.089774994 -0.220437016 0.245772576 -0.168848951

26867 0.063252761 0.403141712 0.335735997 0.341177334

26868 0.047390642 0.333874473 0.291747336 -0.281186673

26869 0.146203133 -0.400465315 0.127460631 0.298051903 -

26870 0.293783393 0.306964372 0.13434746 0.227789508 0.077067818

26871 0.14636131 -0.12606605 0.291596109 -0.266222642

0.18626674 -0.383699123 -0.302499201 0.144609433 -
0.174276085 -0.26836895 -0.21179902 -0.01625553 0.293997601
0.262071313 -0.068091992 0.398865848 -0.114044011
0.33356117-0.264450691 0.254303109 -0.068115724 0.466219625
0.413718545 0.276651064 0.381078251 -0.280265638 -
0.366732682 -0.20400928 0.040407008 0.124113531 -0.427551908
-0.411389261 0.015387329 -0.386276274 0.334292823
0.079168938 -0.012376178 0.093649052 0.285800989
0.350813259 0.001264617 -0.157896255 -0.24576305
0.122522964 0.215085131 0.37504341 0.071907407 -
0.301922974 0.100255508 0.266855795 -0.241236355 0.366461445
0.281183197 0.048178355 -0.131569326 -0.11261903 -
0.08153152 0.295856528 -0.132014254 0.142878403 0.026048122
0.298296807 -0.430919254 -0.077033569 -0.283421868
0.008410462 0.191288159 -0.005520401 0.253769623 -
0.102938572 -0.052784319 0.17415885 0.151616646 0.422118145
-0.174844873 0.002701745 -0.365473491 0.40467828 -
0.151258619 0.09826832 -0.310465147 0.270348256 -0.210529508
-0.410888185 -0.141830804 0.105745482 0.554292697
0.108907483 -0.144475002 0.41252958 0.336415871
0.280958713 -0.086197705 -0.32166141 0.02376399
0.244093196 0.028175757 -0.376639475 -0.017422396

0.215207559 -0.073440978 -0.296587556 -0.021912407 -
0.219932911 -0.342674949 0.264199788 0.093166789 -0.001857228
0.009952634 0.149249649 0.189951183 0.05060849 -
0.31409463 0.228811939 0.279069994 -0.025867884 0.315043729
-0.336760614 0.409193493 -0.195872139 -0.299519046 -
0.076741253 0.200222666 0.316713384 -0.196406622 0.172775236
-0.158688677 0.06799066 0.108497656 0.004232318
0.433212785 -0.057631735 0.071033292 -0.073767792 -
0.240992546 -0.012279005 0.281167127 -0.176397338 0.083620718
0.370454687 -0.202244797 -0.108989276 0.161113687
0.183955246 -0.13885411 -0.309811109 -0.307973603 -
0.127969756 -0.14920468 -0.397006366 0.165250703 0.282962254
0.125541563 0.045473297 0.323829628 0.334626285 -
0.136058945 0.319332053 -0.291630009 0.117867247 0.03251965
-0.080782851 0.126889233 -0.353751311 0.08596552
0.146444365 0.331114113 0.206559018 0.317712188 -
0.045074589 0.064208004 -0.169232361 0.33319936 0.05509032
0.089328187 -0.190853321 0.361117668 0.027517047
0.389409161 0.327684385 0.001136732 -0.24161328
0.069701019 -0.044145482 -0.348440083 -0.099688656 -
0.009679042 0.238713738 -0.19189339 -0.20075659 -0.068146539
0.029681741 -0.033530991 0.070200684 -0.052660426 -

0.162646322 -0.069187983 -0.284104435 0.011880635 -0.240413633
0.071699917 -0.056656158 0.063594842 -0.107463251 -
0.096181644 -0.191565491 0.258821673 -0.178725734 -0.087800848
-0.297579829 -0.147682056 0.054916063 0.13562184 -
0.038609007 -0.222386829 0.177558324 -0.086572836 0.05898465
-0.120617556
KEGG_GLIOMA 0.061497328 0.048969572 0.088303112
0.285509393 -0.367711242 0.107268853 -0.262870614 -
0.110793628 -0.164626371 -0.063253756 -0.099009843 -0.144931694
-0.147647315 -0.086015012 -0.297920426 0.016589926
0.143075499 0.06020974 0.040113982 -0.138675763
0.271185496 0.061965223 0.165657465 0.366334249
0.090367489 -0.166620117 -0.391087515 0.068023954
0.100275275 -0.310089496 -0.068823441 0.229543654 -
0.156160011 -0.31367669 0.318616142 0.302022402 -0.093687061
0.014023798 -0.15455831 -0.021797896 -0.133606011
0.196634729 0.214102848 0.237342911 -0.068118151
0.109572534 -0.069213096 -0.046372885 0.060707582 -
0.086157709 -0.181778542 0.030551981 0.122834283 0.192961053
0.20039386 0.222796319 -0.1256869 0.135277747 -0.055868696
0.298234061 -0.102307544 0.253658317 0.088220395
0.176427136 -0.214738343 0.135091975 0.108572579 -

0.022533406 -0.044393008 -0.270262102 0.170118069 0.209052046
-0.188393362 -0.068219162 -0.197438909 -0.215174513 -
0.115410588 0.096371458 -0.149209946 -0.305921552 -0.264694756
0.33284257 0.107244645 -0.080762769 -0.047270347
0.145663257 0.033215886 0.239661933 -0.073760478 -
0.194115058 -0.326311958 -0.057480669 0.156936082 0.229177111
0.149814887 0.100055212 0.058901772 0.109833669 -
0.002517294 -0.203387576 -0.159342715 -0.243738492 0.021588641
-0.13053146 0.025085355 0.056074266 0.217802516 -
0.133036323 0.332936786 -0.205766317 -0.07388591 -0.3287623
0.051141562 0.122421528 0.235566613 0.123424513 -
0.154937578 -0.324336451 -0.18995519 -0.034096375 -0.314163648
-0.248224859 -0.113896457 -0.069398149 0.22886468 -
0.242570035 -0.273161424 0.297962071 0.201001681 0.118519169
0.002161041 -0.093014439 -0.097241219 0.174696307 -
0.168356053 0.205628005 0.279778062 0.187549831 -0.040622903
0.38090429 -0.058686311 -0.257753049 0.109533611
0.195312701 0.280856229 -0.002649894 0.13930028 -
0.025701178 -0.216173055 -0.272490504 0.076794631 0.256468654
0.223246622 0.077921931 0.086046682 -0.21145493
0.20214442 -0.175618939 0.240805419 0.089032807
0.235810617 -0.118742846 0.151986557 -0.166707919

0.081288488 0.190461678 0.345922954 0.320018321 -
0.221066318 -0.046238938 0.1332761 -0.186016438 -0.194832203
0.116521960.232431416 0.256122293 0.096816794 0.044994105
0.194256296 0.215299516 -0.166925303 0.054412397 -
0.136603988 0.099123288 0.251399176 0.155447347 -0.018414896
0.126258524 0.00515969 0.347841554 -0.226699075 -
0.297213861 -0.106288955 -0.23942071 -0.28207579 -0.158267334
-0.174273488 0.057694958 -0.197635177 0.153530118 -
0.121700147 0.016204097 0.160427101 -0.196697459 0.060422954
-0.1831181 -0.072693784 -0.135400603 0.282311559 -0.254926113
0.073911228 0.062017074 0.150111819 0.321494956 -
0.243739645 0.110654943 -0.059459128 0.244450309 -0.007657962
-0.021209526 0.190220546 -0.090808545 -0.277492365
0.061357971 -0.114996101 -0.124977306 0.070470599 -
0.378344894 0.145710756 0.193490686 -0.243952328 0.004739072
-0.175763778 0.156274559 -0.05890918 0.260326879
0.00135434 0.044474679 0.26879864 -0.187648373
0.108480548 0.066587954 -0.206483665 0.247092689 -
0.170298861 0.253246453 -0.037258589 -0.07100595 -0.1952683 -
0.197699552 -0.03514555 0.175546297 -0.120432596 0.375219343
-0.195244173 0.286303243 0.138883496 -0.066679955
0.104844373 0.044513176 -0.092535351 0.173615099 -

0.070428808 -0.076809074 -0.004509792 0.263135589 -0.021130716 -0.167375627 0.090397283 -0.276687414 -0.246479612 0.169583431 -0.031323059 0.149002582 -0.26887218 - 0.203524258 -0.005963184 -0.237895122 -0.092381817 -0.090047312 -0.035660814 0.05652841 0.093347002 -0.096568338 - 0.081554064 -0.254030394 -0.175743327 0.015391009 0.195420387 -0.259949432 -0.01147425 0.038286978 0.101079171 0.330038763 -0.108660418 -0.235643978 0.286613541 0.258668991 -0.101945099 -0.241715735 0.11443279-0.315912499 -0.312006196 -0.12320033 -0.306123511 0.067302595 0.364222394 0.254232869 0.313177419 -0.234574476 0.116337968 -0.214226636 0.001447417 0.344113089 - 0.008355718 0.150065875 -0.055939547 0.257365548 -0.209358577 0.276090806 -0.185670544 0.075572982 0.186665449 - 0.221573267 -0.131140903 0.311483882 -0.145266342 -0.323742851 0.249880413 0.123872787 0.010582249 -0.121359642 - 0.120728011 0.206433236 0.361125225 0.112703435 0.160185275 0.180697534 0.231539438 0.214254258 -0.247216463 0.142821588 -0.262741159 0.14871467 0.115592161 - 0.080728429 0.281492086 -0.026933037 0.195920562 0.159389684 0.236423336 -0.003957148 0.126792876 -0.321912333 - 0.062432043 -0.339966497 -0.147216088 -0.09105812 -0.219637772

27004 -0.197738631 -0.206591243 -0.107551941 0.216775479

27005 0.26088955 -0.031270132 0.388006065 -0.1340983 0.172556816

27006 -0.250592636 0.186709049 0.141484821 0.245381152

27007 0.303572353 0.244951167 0.1881703 -0.195058981 -0.140972311

27008 -0.233591522 0.064864805 0.125825285 -0.258739157 -

27009 0.236706574 -0.161591792 -0.198383952 0.097662982 0.060315772

27010 0.03828698 0.295515035 0.267242992 0.263223757 -

27011 0.208769869 -0.06867109 -0.08150766 0.17788343 0.179436676

27012 0.259812941 0.038513186 -0.382799556 0.179420633

27013 0.012540558 -0.153246878 0.302714112 0.184909977

27014 0.152914532 -0.136565389 0.018969785 -0.015681973

27015 0.163869933 0.216505214 0.193917392 -0.189494276

27016 0.242748181 -0.367219305 0.043289908 -0.173738279 -

27017 0.206202267 0.258090083 -0.144499237 0.252925024 0.137157346

27018 -0.012648526 0.197299617 -0.110212235 0.312468244 -

27019 0.241123903 -0.207189421 -0.372714149 0.148329465 -0.059010028

27020 0.106721612 -0.299903664 0.120425301 0.114331178 -

27021 0.235612499 -0.191496987 0.209353514 0.317505366 -0.146337268

27022 -0.159419198 0.243823632 0.115473830.151500866 -0.265374777

27023 -0.179790938 0.061346297 0.133734017 -0.042420873

27024 0.034780648 -0.185965151 0.345414155 0.122666011 -

27025 0.121469149 0.093225309 -0.214035234 -0.295729464 0.121337265

27026 0.298043932 -0.065127553 -0.035484191 0.235201141 -

27027 0.010975435 -0.063161988 -0.102733692 0.190059591 0.152740392

27028 -0.056005312 0.072961973 -0.138165249 0.19164488 -

27029 0.226986218 -0.166542656 -0.110392479 0.290880178 0.364091334

27030 -0.098861572 0.128158521 0.007729879 -0.043398228

27031 0.132682143 0.159714971 0.248620685 -0.023128032

27032 0.112592717 -0.166744721 -0.259719872 -0.037043789

27033 0.300487124 -0.245460889 -0.00794062 0.106842297 -

27034 0.105613319 -0.074136037 0.22461549 0.212138432 0.040332939

27035 -0.224461267 -0.217233697 -0.043444068 -0.22218691 -

27036 0.178985151 0.072418289 0.203094173 0.067227372 -0.035829196

27037 0.263349457 0.22958349 -0.137141307 0.244345968 -

27038 0.208986422 -0.135193933 0.140398985 -0.227049178 0.260985173

27039 -0.178772004 0.04148342 -0.052219091 -0.013059767

27040 0.108145651 0.2638864 -0.056874343 -0.075613751 -0.13868405

27041 0.132666961 -0.283587329 0.04759798 0.035264121

27042 0.216431213 -0.004766118 0.200744259 0.160187667 -

27043 0.10222539 -0.22465194 0.014587663 0.045397465 -0.198159705

27044 -0.081175609 0.04262194 0.171913398 0.108937532 -

27045 0.026918338 -0.18233434 0.187042498 -0.077898621 0.152333374

27046 -0.116012775 -0.342342661 0.054005849 0.009782749 -

27047 0.244510414 -0.117123204 -0.126185701 0.048218329 0.175741523

-0.165435835 -0.070753978 -0.121359529 0.180849572
0.088647936 -0.200639094 -0.194070711 0.076416863
0.104090557 -0.05847997 0.022114303 -0.113257486
0.237671833 -0.080196384 -0.057403213 -0.187386695
KEGG_PROSTATE_CANCER 0.051512782 0.096922311 -0.008312418
0.22866402 -0.340240519 0.05507663 0.056510904 -
0.212867213 -0.084539284 -0.16488219 -0.135386409 0.018600034
-0.143898623 -0.021888467 -0.423552356 -0.226499444
0.265013681 0.150820948 -0.200833374 -0.078951129
0.304815725 0.168059376 -0.002098865 0.423271618 -
0.15017887 -0.199437367 -0.321197472 0.165292293 -0.048070958
-0.346039241 0.024903276 0.044833774 0.032070272 -
0.350365449 0.123247716 0.254549539 -0.151106578 -0.131185079
-0.229918143 0.110104689 -0.057605238 0.17854518
0.288473783 0.173418467 -0.107945023 -0.034223846
0.074409093 -0.108675549 0.068327698 -0.121104609 -
0.102882986 0.06502612 0.327744629 0.121112217 0.32152533
0.231125849 -0.288968294 0.155542518 0.149193479
0.347028839 -0.111810454 0.227391975 0.199514023
0.170752528 -0.151299823 0.026085538 0.208332955
0.072258461 -0.211677888 -0.254989483 0.215150595
0.177029802 -0.054836583 -0.111007044 -0.050496666 -

0.304323988 -0.221878424 0.199045035 -0.028709536 -0.209107829
-0.238593226 0.397743434 0.014976441 -0.098398093 -
0.10580231 -0.085887406 0.162665916 0.145288913 -0.187831871
0.004952679 -0.276862778 -0.192175241 0.1852147 0.17661369
-0.003966374 0.177654291 0.160327704 0.01084471
0.056114765 0.018331852 -0.158806276 -0.341888121
0.056210389 -0.263936771 -0.143604368 -0.007720209
0.191655656 -0.30331296 0.218901353 -0.178875717 -
0.108643682 -0.167106102 0.097071997 0.103639973 0.240759635
0.214322371 -0.141941155 -0.415572522 -0.300552857 -
0.097860415 -0.328940993 -0.008565077 -0.066370831 -0.182092204
0.119850081 -0.275547742 -0.277814273 0.227075275
0.288281668 0.192133934 -0.041622033 -0.258648194 -
0.066587932 0.113166694 -0.243730642 0.159858337 0.169863383
0.355080642 0.092831161 0.282744989 -0.117334725 -
0.163758672 0.267772348 0.228911935 0.200921339 0.028816607
0.305808622 -0.168422672 -0.210441442 -0.110977378 -
0.065513706 0.154705689 0.13864597 -0.014358846 0.163863986
-0.212272874 0.012047911 -0.269290328 0.222514665 -
0.100082706 0.183415309 -0.141359941 0.12549507 -0.206230205
0.128085206 0.103241077 0.298449018 0.205378045 -
0.240843692 -0.119106919 0.289923946 -0.319013531 -0.257686584

27092 0.102269285 0.299663721 0.234093771 0.224900945

27093 0.057333585 0.192778065 0.025998621 -0.189309631

27094 0.157696322 -0.153819251 0.217283059 0.305853194

27095 0.299002999 0.153746121 0.138639862 -0.162922068

27096 0.179506961 -0.186219011 -0.393912683 0.051578106 -

27097 0.270360404 -0.180813814 -0.123600496 -0.125480321 0.056106774

27098 -0.158445695 0.137360109 -0.183622115 0.140374971

27099 0.115899263 -0.141385934 0.132437169 -0.112081347 -

27100 0.096433568 -0.009212509 0.264173007 -0.180927339 0.193582894

27101 0.233939479 0.140266007 0.237756599 -0.25076402

27102 0.126555057 -0.034862855 0.228127683 0.03565435

27103 0.084859243 0.019594065 0.199695505 -0.133474264 -

27104 0.008250586 -0.274093975 -0.091584157 -0.04673122 -0.322597243

27105 0.37190008 -0.055999693 -0.220251992 0.073994658 -

27106 0.05870148 0.106982415 -0.082781259 0.304116979 -0.016078851

27107 0.207602133 0.239256121 -0.296784659 -0.011703546

27108 0.101986228 -0.057739752 0.223763287 -0.190157835

27109 0.335568673 0.096526507 -0.168990319 -0.209903161 -

27110 0.239695774 -0.118564123 0.215493308 -0.242211765 0.128589055

27111 -0.176491279 0.154384211 0.170799401 -0.130833924

27112 0.26544589 0.075671367 -0.14420253 0.157757327

27113 0.10403798 -0.234350157 0.028204408 0.156882303 -

0.039358884 -0.136422158 0.228615821 -0.288667662 -0.213971642
0.101004799 0.03593222 -0.090828576 -0.183573366 -
0.153666028 0.185714974 -0.06798827 0.000565102 -0.142199933
-0.270401427 0.020324729 -0.061408201 -0.135201089 -
0.253507576 -0.263315241 -0.349951825 0.067174924 0.252929773
-0.210695668 0.094775481 -0.072031605 0.216978946
0.359964395 -0.22439035 -0.247221569 0.277540129
0.191958492 -0.141911505 -0.255577533 0.167315267 -
0.266716706 -0.334252336 -0.249446793 -0.249978037 0.066296475
0.227729132 0.293473396 0.103411127 -0.249408101 -
0.049114339 -0.256847257 -0.138994984 0.395630421 -0.09906306
0.172286608 -0.303417436 0.233182618 -0.013147549
0.328140123 -0.156531307 0.216711445 0.174404492 -
0.285376519 -0.18575503 0.021729035 -0.190804772 -0.340023069
0.309207361 0.208039574 -0.072795988 0.084949618
0.013051053 0.069953323 0.370002167 0.277440345
0.252850115 0.170598386 0.297185847 0.251147141 -
0.189952791 0.039409546 -0.344728929 0.200297034 0.335444095
-0.092880876 0.281025114 0.103729854 0.252738334
0.160319098 0.120101317 -0.062095932 0.167893232 -
0.285569138 0.139046059 -0.274273803 -0.286432073 0.032141548
-0.181071692 -0.304231762 -0.115307001 -0.06744774

0.296842221 0.17089193 0.001428829 0.263277436 -
0.055010814 0.310429202 -0.24866507 0.206521597 0.044350799
0.293869513 0.372093665 0.249755862 0.299734723 -
0.239441607 -0.279116859 -0.176256316 -0.012533906 0.201877013
-0.372161992 -0.297341655 -0.231199374 -0.358360157
0.134013093 0.09878806 0.044079697 0.168529894
0.357174316 0.326882631 0.013835365 -0.020090028 -
0.17217055 0.002328375 0.22049305 0.244319173 0.071449788
-0.335344358 0.124179864 0.181317113 -0.167825749
0.290046809 0.289883424 0.046534702 -0.104534913
0.057903944 0.026631552 0.281478342 -0.031088903
0.266067903 -0.032002672 0.287052929 -0.377043652
0.04879661 -0.309320662 -0.036161971 0.222390862 -
0.233469531 0.083798312 -0.038176887 -0.037487029 0.107564529
-0.033747004 0.354713602 -0.203054397 -0.17017415 -
0.3273586 0.178547391 -0.101714725 0.205897485 -0.333850931
0.131331644 -0.107597746 -0.334741541 -0.238113794
0.150906623 0.381420216 -0.07960158 -0.081150512
0.231854337 0.24758107 0.116228138 -0.137274684 -
0.138169391 -0.016667169 0.110009196 -0.196484355 -0.18958481
-0.091445328 0.248755849 0.035179654 -0.26051971
0.071724364 -0.253029669 -0.208208872 0.064348602

0.289931565 -0.063225242 -0.053006726 0.221402012
0.188329568 0.004406209 -0.233941942 0.206382336
0.121601461 0.07685206 0.084055982 -0.234722758
0.273402965 -0.147923656 -0.054895064 -0.199633198
0.197570848 0.26976701 -0.14004946 0.092216157 -
0.010017856 0.032360102 0.165691874 0.048496817 0.309100801
-0.10571546 0.079622748 -0.209522951 -0.170319397
0.035075067 0.271687095 -0.127843476 -0.034852945
0.199230888 -0.111503928 -0.004174658 0.30317251
0.213540176 -0.030701364 -0.308569315 -0.272002058 -
0.137615155 -0.175533499 -0.274379069 0.144577849 0.178683717
0.08303399 0.089331492 0.215850725 0.226609173 -
0.145876023 0.187733685 -0.16815886 0.01900367 0.064810388
-0.194516762 0.233101583 -0.26210375 0.037329617
0.127338918 0.110357057 0.104576078 0.287218685 -
0.140642055 0.042310765 -0.156966918 0.224224536 -0.304261345
0.068835647 -0.062268161 0.321428854 -0.106823491
0.364200894 0.266850862 0.003126921 -0.266870413
0.095197197 0.016518076 -0.259731655 0.119339495
0.014420869 0.085967558 -0.028625859 -0.186671321 -
0.033473604 -0.035375107 -0.139920083 0.064838521 -0.141180208
-0.13056003 -0.175147193 -0.103368302 0.136332778 -

0.213938411 -0.050628157 -0.074135923 0.004407941 -0.137302256
-0.115869692 -0.114789841 0.145549201 -0.024522512 -
0.193585501 -0.226224647 -0.161842511 0.046124443 0.190155524
-0.094440527 -0.034696706 -0.049080277 -0.015586242 -
0.158063687 0.055553473
KEGG_THYROID_CANCER 0.260618986 0.267758239 -0.145269246
0.191547314 -0.283812846 0.131139273 -0.16812154 -
0.29361202 -0.098381624 -0.336555567 -0.164838066 -0.121026554
-0.199720953 -0.033176162 -0.445868602 -0.207871205
0.278103852 -0.065749208 -0.104273574 -0.248163948
0.491028257 0.191096453 -0.082654378 0.355744281 -
0.1128401 -0.203640017 -0.42806858 -0.083121622 -0.074257017 -
0.305585429 -0.134186586 -0.258235369 -0.092380684 -0.361688179
0.184580854 -0.168440377 0.032886343 -0.282907726 -
0.136845665 0.067137579 0.055753998 0.299204761 0.311606227
0.344795746 -0.08795281 0.123166681 0.284309132
0.001375787 -0.087434075 -0.201989914 0.052598521
0.116053278 0.378673417 0.304965056 0.179672813
0.128408257 -0.147403338 0.205628806 0.128234615
0.184203562 0.05945385 0.056855912 0.299123659
0.348551002 -0.123659414 -0.145480979 0.092797685
0.278905078 -0.222908862 -0.406441008 -0.041289582

0.112057865 -0.063963844 -0.320097322 -0.015037197 -
0.391152752 -0.141937911 -0.038757318 -0.123538841 -0.012209918
-0.287700877 0.342223352 -0.062230532 0.160023694 -
0.147849305 0.020969941 0.306016561 -0.058805218 -0.009818179
0.045420925 -0.109169543 -0.221022394 0.247296602
0.022723003 0.104307576 0.097559037 0.137679929 -
0.076844609 0.150980212 0.03528916 0.003119588 -0.396985933
0.193381666 -0.252415946 -0.139553073 -0.298306072
0.187016882 -0.446282751 0.394739162 -0.007832125 -
0.171429703 -0.27463057 0.175863429 0.396917324 -0.17495473
0.17296075 -0.054441573 -0.475020117 -0.39500316 -
0.294390916 -0.306082389 -0.049165457 0.08276922 -0.099226684
-0.047423823 -0.387552311 -0.219841728 0.083649187
0.326486426 0.109441922 -0.166340578 -0.326604802 -
0.112537180.119989147 0.097305158 0.18588507 0.239882273
0.49297834 0.250219516 0.246958121 -0.322408514 -
0.209001396 0.396718649 -0.185784884 0.221293937 0.140326401
0.365042725 -0.09119857 -0.315200752 -0.03631755 -
0.146144978 0.348511375 0.065356116 -0.076043819 0.124608274
0.034898844 -0.067364988 -0.003351037 0.092991587 -
0.331723851 0.195702959 0.099754606 0.093364496 -0.217637572
0.122594607 0.113104721 0.072327389 -0.031045643 -

0.18999025 -0.310145661 0.500429448 -0.079189968 0.002157
0.272107055 0.374502029 0.278587456 0.162514885
0.217338229 0.34359855 0.297416393 -0.346672683
0.095388395 -0.093113592 0.208277818 0.36215412 -
0.01455313 0.241551411 -0.016413482 -0.241490655 0.214265732
0.119108684 -0.395584541 0.09953905 -0.336191176 -
0.080507698 -0.331010947 -0.130462856 0.102883961 -0.157441089
0.1125304 -0.07490811 -0.161481188 -0.083974268 -0.271455137
-0.239108865 -0.095292681 -0.224904318 0.039897555
0.344768257 -0.186731424 0.374653237 0.14928663
0.159172816 -0.06985891 -0.242811351 0.010731131
0.223045258 0.114702182 -0.058717129 -0.075998105 -
0.075125397 0.271029209 -0.197425659 0.116421034 -0.326195497
0.004245102 -0.213629381 -0.397522718 0.459912584 -
0.070611695 -0.274032422 -0.072633527 -0.052382246 -0.081881074
-0.142880191 0.314010682 -0.21469685 0.243285517
0.02186236 -0.135761836 -0.080625121 -0.047797598
0.156029041 0.239409542 -0.316875134 0.339599589
0.005586606 -0.090114557 -0.455175802 -0.072419685
0.180248582 0.271074667 -0.237749599 0.124340596 -
0.289384776 0.384983936 0.325956242 -0.225880715 0.303888579
0.110018669 -0.094919258 0.175472422 0.005485878 -

0.003765407 0.020916569 0.135303364 0.189469261 0.02107319
0.314864591 -0.299476866 -0.034055831 -0.343553148 -
0.23999128 -0.121146059 -0.125617863 -0.033632742 0.142601849
-0.138046215 -0.133039329 -0.086621229 -0.247075378 -
0.233587673 0.149360784 -0.260028951 -0.202891482 -0.188335331
-0.355389435 -0.173759617 0.136668989 -0.020979074
0.175365672 -0.199167727 0.173016036 0.237008743 -
0.406470119 -0.354922847 0.355278222 0.067327046 -0.101985382
-0.204285435 0.172060485 -0.313506163 -0.178856725 -
0.091014456 0.156576899 -0.099296302 0.226891954 0.273515944
0.023941577 -0.34110291 -0.119149119 -0.239531651 -
0.110473598 0.348683586 -0.272406214 0.005148483 -0.340529045
0.192135288 0.141302322 0.338864424 -0.124745312
0.234870152 0.332311434 -0.342906479 -0.303737501 -
0.197370194 0.043156963 -0.414507709 0.477183859 0.14290191
-0.209964893 0.246083075 -0.349048232 0.160959067
0.43765967 0.372918509 0.233027039 0.049840518
0.34145044 0.408821275 -0.196761852 -0.017764848 -
0.414743859 0.23255853 0.140338916 -0.372208679 0.09474869
-0.027884238 0.164231544 -0.084665266 0.204556953 -
0.122992067 0.268800626 -0.136114927 0.092994811 -0.289779697
-0.184874968 -0.098166514 -0.062122646 0.007992381 -

0.052621087 -0.00474535 0.395404049 0.090944298 -0.059506851
0.280592243 -0.131871625 0.193677074 -0.165952501
0.154834377 -0.242107503 0.453911902 0.420997657
0.235468126 0.222515873 -0.22879268 -0.148713122 -
0.112671499 -0.115732718 0.0925308 -0.442675658 -0.235001968
0.101357386 -0.355651446 0.258285512 0.318528104
0.091376614 0.262257087 0.408534938 0.079590713 -
0.113753811 -0.138400408 -0.184071335 0.163831150.068635903
0.320669988 0.213738392 -0.379093242 -0.049854657
0.37278165 -0.223842357 0.235732669 0.289917921
0.010591208 -0.099748232 -0.021015171 -0.190019943
0.44504379 -0.182694253 0.039430945 0.089084485
0.295750777 -0.089813845 -0.113872726 -0.214154233 -
0.129242302 0.013306529 -0.044476233 0.219313921 0.002371311
-0.055471067 0.138935798 -0.025080861 0.215699466 -
0.16105844 -0.21374282 -0.38243611 0.365111008 -0.066804907
0.224106665 -0.417729597 0.413492842 0.034367305 -
0.235785804 -0.177584908 0.184289092 0.508990938 0.172909003
-0.270399124 0.277663096 0.438223708 0.168444708 -
0.314537207 -0.205188221 -0.039650984 0.110861638 0.027246202
-0.353793224 0.20747695 -0.237070489 -0.229991383
0.021831091 0.15587682 -0.113222034 -0.230551399

0.267504527 -0.078143448 -0.091289321 0.048822078
0.24432892 0.321281627 -0.107694325 -0.361472261
0.125243093 0.319341291 -0.236691628 0.264285926 -
0.316518687 0.34762852 -0.066776777 -0.193181784 -0.398970678
-0.052095536 0.182938254 0.01254357 -0.005531746 -
0.045220876 0.282396241 0.233222642 0.079354092 0.28762285
0.076647201 0.007061116 -0.063496411 -0.184830567 -
0.13778777 0.327074087 -0.132449239 -0.147909615 0.383242952
-0.153692888 -0.009990994 0.307623668 0.219090303
0.076379923 -0.374245564 -0.352125039 0.145308162 -
0.312514736 -0.248911683 -0.002881129 0.37843006 0.307532045
0.290904083 0.36797895 0.217179619 -0.299447708
0.213689349 -0.312948398 0.257596197 -0.309959361
0.063676337 0.250297973 -0.284071707 0.099070446 -
0.183048045 -0.11116961 0.170867958 0.278986002 0.031713348
-0.136988583 -0.317484418 0.018329518 -0.155063968 -
0.140902127 0.04619769 0.059661626 -0.122262268 0.427330488
0.14601744 0.014520787 -0.08015382 0.070001251 -
0.283247421 -0.423009557 0.074423845 -0.086648055 0.014141755
-0.291283737 -0.137761676 0.049679733 0.122541756 -
0.108626033 -0.199229609 -0.25012764 -0.210993645 -0.187848659
-0.021474303 0.050921844 0.100025426 -0.1263768 0.007201895

0.158756409 -0.015870499 -0.12617845 -0.145273196
0.216686322 0.103165202 0.203100401 -0.19065391
0.264225049 0.026419571 -0.110839692 0.116311765 -
0.239628179 0.147952858 -0.103266034 -0.239731693 0.086432406
KEGG_BASAL_CELL_CARCINOMA -0.230348164 -0.302218007
0.175246156 0.107192115 0.101705277 0.106853831 -
0.121372966 0.095305622 0.07669347 0.118764477 -0.063913251
-0.179446784 0.188511410.068222317 0.306400351 0.218339669
-0.271436816 -0.321788538 0.3685979 -0.245141544 0.02198772
-0.406256027 0.416895176 -0.293845793 0.135057962 -
0.063940956 -0.30981186 -0.137109861 0.085600497 0.227344681
-0.250774264 -0.345654864 -0.194667998 -0.055833845 -
0.104145575 0.330106381 0.359739667 0.141862144 0.202060605
-0.341904713 -0.20160283 0.234712811 0.024377387 -
0.404988161 0.062300378 0.112593686 -0.028971196 -0.140640212
0.062958079 -0.056169859 -0.347748754 0.164888111 -
0.332464072 0.101137313 -0.21752479 -0.144328443 0.324919176
-0.009966321 -0.372854491 0.380298083 0.238746657
0.479082604 -0.314780504 0.07003338 0.078351166 -
0.083813903 0.2409726 0.251539348 -0.037378965 0.020057899 -
0.072324305 -0.023476794 0.156608883 -0.193842423 0.128421391
0.168726407 0.034974471 -0.327684987 0.25298479 -

0.329992012 0.073395877 0.17762913 -0.336853223 -0.058962047
-0.033001724 0.09404655 -0.006500177 0.450610688
0.313860717 -0.306060477 0.24784594 -0.03922399 -
0.117489558 -0.065970857 -0.306173909 -0.053132257 -0.125361443
0.271553233 -0.288605065 -0.098233567 -0.057252469
0.071809148 -0.240330376 -0.163703898 0.141404518
0.018966939 -0.367460168 -0.21474219 0.137036564
0.111066196 0.311819174 -0.173779347 -0.18775769
0.18937625 -0.17631989 -0.478975818 -0.25735552
0.180326104 0.325434956 -0.050639967 0.148771591 -
0.199518313 -0.169215004 0.19493881 0.289031245 0.154297832
-0.247384131 0.222615762 -0.428295591 -0.213537569
0.131077551 0.152786417 -0.039309382 -0.172834629 -
0.096753341 0.272448326 0.430156182 -0.299488808 0.021528369
0.160880261 0.085475983 -0.127115275 -0.006154685
0.170670089 -0.287971902 0.232529345 -0.113595222 -
0.243714147 0.182834388 0.318002929 0.31419685 0.262226737
0.103153385 0.256234578 -0.168985106 0.110971394
0.167494884 -0.212238089 -0.143800604 0.167358756 -
0.158161108 -0.24387368 -0.073430428 0.087345052 -0.228355179
-0.003786659 -0.102028512 0.204333479 0.237389396
0.086206057 -0.484394796 0.136316917 0.104571288

0.019539351 0.183017891 0.300495494 0.035758641 -
0.138141216 -0.225297753 0.009181702 0.147125887 -0.036661934
-0.203198246 -0.155170983 -0.007054882 -0.256379426 -
0.136977969 -0.249223498 0.298402137 -0.065278232 -0.103306984
-0.24660006 -0.17284151 0.222322107 -0.490201521
0.089755205 -0.059617238 0.186249526 -0.1100121 -0.157103134
0.230678156 -0.402387193 0.122017426 -0.186518198
0.110251973 -0.280681049 0.254912947 -0.295735813
0.108433738 -0.348478192 -0.255550249 0.188138196 -
0.309297894 -0.21220622 0.111353081 -0.307744355 -0.505061366
-0.060725431 -0.165056693 -0.077812961 0.180337091 -
0.065312509 0.140658037 -0.176486113 0.071005977 0.049989596
-0.314472784 0.101508907 -0.182566126 0.201448767 -
0.09967898 -0.217753007 -0.041158533 -0.062506822 -0.066487193
-0.129152979 0.379243646 -0.216225112 -0.030284036 -
0.090196604 0.338800645 0.003228187 -0.047920886 -0.039856024
0.338212632 -0.173577597 -0.017220798 -0.095184439 -
0.100500515 -0.066633435 -0.112653762 0.058477921 0.398424904
0.41503526 0.135286093 0.374567726 -0.077645069
0.26365121 -0.239957031 0.133284426 -0.006003247 -
0.370487426 0.066954765 0.390124213 -0.21614752 -0.051610615
-0.045536663 -0.216638195 -0.228081803 0.105617846 -

0.39131816 0.153840222 0.161698331 -0.126612528 -0.337388876
-0.118469673 0.04594315 0.127824938 0.073657819
0.362660783 0.443350702 -0.155410574 -0.036049288
0.243931269 0.298398765 -0.204589998 0.314355592 -
0.399514827 0.376415458 0.252987556 -0.275687181 0.07809859
-0.272041235 0.209462044 0.362220531 0.10307167 -
0.04248966 0.006892637 0.153705608 0.05256977 -0.142386306
0.060249177 0.333323197 0.118028253 -0.050552661
0.004144869 0.081902867 -0.207285112 0.399997915
0.021330709 0.046962536 -0.015557073 0.415905664 -
0.127555758 -0.133075692 0.074655927 0.348549789 -0.285025142
-0.144890498 -0.256799805 0.213173224 0.119172963 -
0.131687608 0.291402953 -0.254290236 0.104561108 0.05087596
0.237065669 -0.072731683 0.162538041 0.330833017 -
0.14993965 0.245714822 -0.04220038 0.166188729 -0.224904672
-0.124183749 0.054328299 -0.217422067 -0.050098088 -
0.215097939 -0.27593644 0.139161613 -0.002906725 -0.365319306
-0.0558861 0.089938677 -0.322358347 0.057125293 -0.012023388
0.343415512 -0.088797243 0.147040457 -0.421311132 -
0.076113844 -0.028697295 0.365440822 -0.387148941 -0.150559973
-0.196195079 0.059734044 0.047679963 -0.307521941
0.350367234 -0.427451613 0.520695059 -0.034712415 -

0.116166026 0.033867897 -0.115404136 0.402856156 0.259565433
-0.015254725 0.172124459 -0.391177259 0.037203259 -
0.214508835 -0.231802011 0.127778678 -0.122766452 0.20112086
0.272748259 0.038429122 0.100567301 0.187695919 -
0.238371231 -0.032145448 -0.237211838 -0.14399231 -0.169441132
-0.344658194 -0.162853131 0.061386922 0.060768107
0.061055905 0.418186 -0.343362478 0.347093721 0.276131639
-0.035840701 -0.157754266 -0.331833367 0.191049631 -
0.323220253 0.178411977 -0.273643272 0.082471157 -0.1026381
0.145187738 -0.425508771 -0.319225888 -0.118185957
0.066357142 -0.02711214 0.375954041 -0.187897331 -
0.181561952 -0.258996843 0.29970769 -0.252818646 -0.009069531
-0.082604089 0.07583903 0.163314671 0.199081406
0.260153035 -0.196284904 0.412659444 0.224404147 -
0.26507336 0.228847482 -0.163466026 0.195857054 0.227775136
0.204157722 0.077660888 -0.198870895 -0.400979321 -
0.044781836 0.308508963 0.181622632 0.386472643 -0.26691654
0.402132832 0.013075911 -0.38961497 0.109565043
0.106281882 0.159638462 0.083307549 -0.15273804 -
0.088405821 -0.236429714 0.14320147 -0.326062958 0.316814291
0.087860281 0.424599613 0.182465119 0.025668632 -
0.093779677 0.072215114 0.301416348 0.500636789 -0.074288381

0.055550944 -0.214366078 0.308465672 0.130854032 -
0.210739069 -0.241142992 0.097974549 -0.136037532 0.135020555
0.266895003 0.029443055 -0.11076136 -0.067676275 -
0.173128636 -0.316864912 -0.026555129 0.209833115 0.032035825
-0.124983894 -0.121053427 -0.533574042 -0.186700082 -
0.0762099 -0.038170649 -0.077564705 0.023953156 -0.203840786 -
0.183315744 0.050511603 -0.134395453 0.058626581 0.282208403
0.180585589 0.09768774 -0.063008755 -0.18186702 -
0.199311082 0.074062805 -0.025438193 0.467764293 0.093052534
-0.09941576 0.167324458 0.085230757 -0.198379529
0.111749247 0.120499401 0.357468381 0.231701392
0.033987449 -0.039067739 0.001459657 -0.017482452 -
0.216778983 -0.09591687 -0.250089902 0.396891797 0.200270395
0.234916306 -0.173485903 0.279270216 -0.107865239 -
0.036360193 -0.112923257 -0.055725201 0.075725163 -0.044483176
-0.223814256 0.305445099 0.075709059 0.205593195
0.051672596 0.003021459 0.147789514 0.282766699 -
0.046149077 0.015935448 0.176334728 0.114226897 0.046829392
-0.229757584 0.083696793 0.239533162 0.10115662-0.239411912
0.19842725 0.14655993 0.21756744 0.107153643 -
0.136829941 -0.099484157 -0.208818764 -0.053669051 -0.15532719
-0.010932305 0.062520419 0.028478303 0.362296162 -

0.426464435 0.09638995 0.215161111 -0.256530111 0.228113686
0.21909462
KEGG_MELANOMA -0.08520357 -0.069867348 0.049673848
0.166988659 -0.186185396 0.105235286 -0.213635568
0.036058577 -0.113201825 -0.172867873 -0.04135352 -
0.002726249 -0.053795269 0.033420391 -0.19482453 -0.038943393
0.044844117 -0.027730917 0.113671369 -0.262071434
0.057042171 0.019158416 -0.007464697 0.245067881 -
0.088079008 -0.031157885 -0.073002145 -0.085453357 0.156974494
-0.178600017 -0.003386211 0.021258764 -0.160130977 -
0.179633972 0.245914863 0.347125436 -0.03275373 0.107051255
-0.040733619 -0.296837206 0.064417073 0.187152835 -
0.05902269 0.167521888 -0.042100987 -0.043546619 -0.14813386
0.104290949 0.090741295 0.117570876 0.063037815
0.042740522 0.234177076 0.010250851 0.192937885
0.053317951 0.085791444 0.038569887 -0.059613023
0.179830719 -0.075974643 0.325484279 0.059250875
0.001359614 -0.208149791 0.087997597 0.152464395 -
0.060871912 0.188508488 -0.035348865 -0.016959442 0.099895237
0.034813539 -0.047532118 -0.113218951 -0.160148025 -
0.111437601 0.073030912 -0.020258894 -0.324434569 -0.261931885
0.270698126 0.064263713 0.136267166 -0.138513563 -

0.030540212 0.023203888 0.068597392 -0.04571865 -0.263311459
-0.243175304 0.207838398 -0.048864489 0.207338887 -
0.063533451 -0.062610599 0.190630224 0.098419643 0.110319058
0.001626688 -0.196884787 0.108801824 -0.021559622 -
0.212472567 -0.064972697 0.113118093 0.047413002 -0.183183894
0.17636224 -0.005482298 0.21831594 -0.177675032
0.139097646 0.156225110.170621998 -0.001605808 -0.061071183
-0.065731796 -0.012876906 -0.227623676 -0.019936974 -
0.18533142 -0.140735289 0.080064901 0.039918651 0.066990622
0.052460284 0.216991326 0.101575224 -0.017796102
0.058605445 -0.058937888 -0.051657512 -0.013001581 -
0.157951337 0.072230399 0.119893056 0.109497241 0.115340765
0.119628235 0.107050265 -0.119532993 -0.110639514
0.150516498 0.1079124 0.054078533 0.216597901 -0.02262284
0.0209601 0.021563103 0.187923743 0.169380515 0.123521225
0.116058303 -0.178014732 0.182221597 0.076243281 -
0.261245747 0.062972127 0.04109978 0.120910843 -0.26560697
0.166500141 0.038152707 0.23794319 0.030886655
0.361472611 0.21462425 0.050978782 -0.020340976
0.040394211 -0.051620561 -0.113318235 0.037436532
0.120143181 0.261891605 0.021033907 -0.215772532 -
0.150241431 -0.020864527 -0.074164115 0.222609612 -0.011141167

0.277808298 0.038917794 0.082607944 -0.010184468
0.171205526 0.098889401 0.287167854 -0.258826101 -
0.08239153 0.139134052 -0.21796474 -0.294940878 0.019724967
-0.063857802 0.014590271 0.050720131 -0.077643165 -
0.110355281 -0.086847873 0.194590437 0.007771624 0.051607486
-0.123172126 -0.055450745 -0.103754751 0.2008987 -0.261039215
0.017131801 0.055484228 -0.080109866 0.219083211 -
0.024556442 -0.046243701 -0.123006718 0.046747399 0.088567381
-0.153881407 0.113820397 -0.026812294 0.035579011 -
0.06813712 -0.113036931 0.033863079 0.141899055 -0.152391237
0.074858659 0.27870744 -0.151654535 -0.039355667 -
0.182436686 0.004119204 -0.146556659 0.192073534 0.075180975
0.024773502 0.181326603 -0.161275202 -0.16162751 -
0.125842887 -0.283207712 0.190365349 -0.096059383 0.160183857
0.000722974 -0.076195325 -0.182856842 0.072647392 -
0.0680636 0.09959771 -0.043462121 0.140054696 -0.047608612
0.282910133 0.058091595 0.044069631 -0.041217454
0.175611551 -0.258295109 0.130546388 -0.034801826 -
0.100263487 -0.082685724 0.247354522 0.135474312 -0.038481113
0.064409775 -0.033056557 -0.099960932 -0.037424645
0.119008532 0.142037644 -0.2574179 -0.313833644 -0.164315513
-0.066413328 -0.06532873 -0.107158949 0.041998017

27510 0.185457749 -0.129182972 0.190811971 0.075090746 -

27511 0.129317503 -0.03841402 -0.072346552 0.198556413 -0.159098348

27512 0.097612504 -0.01598329 0.022915110.27701265 0.132691369

27513 -0.036345176 0.119427770.157253541 -0.133737942 -0.109619755

27514 0.098343684 -0.307009627 -0.290090333 -0.180506091 -

27515 0.301270691 0.246167489 0.149386387 0.174094237 0.224361648

27516 -0.029817498 0.02892164 -0.12039115 -0.014138843

27517 0.113265582 -0.194876584 0.044854781 -0.041629879

27518 0.162942692 -0.324846324 0.104477344 0.118933147

27519 0.071512894 -0.036297141 0.124307295 -0.181620612

27520 0.276465325 -0.14109486 -0.168558066 0.170026566

27521 0.234645917 -0.081010621 -0.09594977 0.093465054

27522 0.159001648 0.223896445 0.011467541 -0.053802844

27523 0.144297014 0.014889241 0.007518175 -0.122851235

27524 0.01016758 -0.139602456 0.105958839 -0.101816999

27525 0.018366132 0.28694971 -0.093355516 0.218183037 -

27526 0.051115174 0.075809061 0.075647289 -0.13358873 -0.316324244

27527 -0.190423571 0.019205614 -0.038981696 -0.15648371 -

27528 0.250279187 0.002683867 -0.256872148 0.039388106 0.069524385

27529 0.17780779 -0.104801969 0.263719649 -0.001405801

27530 0.07737815 -0.128825668 0.002863252 0.122459019

27531 0.253195204 0.137170092 0.32320798 0.085414039 -

0.221392573 -0.069079216 -0.082611492 -0.082727137 -0.092764294
-0.224172706 -0.157392889 -0.178737252 -0.142133538 -
0.063516081 -0.040860612 0.126890311 0.111609361 0.138732205
0.190090983 -0.129240658 -0.121463423 0.080445778
0.186104011 0.254702678 0.069967345 0.063111971 -
0.190684436 0.115081653 0.027668736 0.0750123 0.1271177
0.106766383 0.056123642 0.138377993 0.133741214
0.181676233 0.18744606 0.163633985 -0.009342526 -
0.172992416 0.03707853 -0.353872105 -0.076696818 0.098010228
-0.100950958 0.191160256 -0.236951036 0.206532288
0.159551269 -0.19695436 0.239626959 -0.049286736
0.095108214 -0.021124435 0.043258614 -0.218439915
0.111756253 -0.013043587 0.014382831 -0.22706096
0.045957062 0.161383223 -0.020558096 0.011596817
0.077006671 0.142370487 0.088229703 -0.231713729
0.192933794 0.094045825 0.144902075 -0.211199719
0.074106576 -0.070011619 -0.020897256 0.128220885 -
0.058151497 0.031981242 0.215284244 -0.019954704 -0.052278731
-0.046047703 0.083633802 -0.133437061 0.03600359 0.3088884
0.109449995 -0.008122867 0.131473043 0.1395394 -0.011784521
-0.082100406 0.329436088 -0.144660997 0.124607439 -
0.201300501 0.016969131 -0.109659744 -0.100177441 0.015852536

-0.053750362 0.305098124 0.154970424 0.160322384
0.085242149 0.089381847 0.190809539 -0.009020336
0.040084385 0.055421121 -0.075471929 -0.104618999
0.060415927 -0.062340544 -0.156725395 0.044050943 -
0.060103604 0.048002779 0.130701113 0.23009567 0.191260027
-0.001929055 -0.08265731 0.101428205 -0.142860168 -
0.031155428 -0.122135726 0.001102276 -0.171148804 -0.105580984
0.042635618 0.116272155 0.126182178 0.208772595
0.286317171 -0.122619595 0.239938171 -0.03603955 -
0.083158154 0.100123074 -0.16967835 0.135989719 -0.008555996
-0.053559519 0.120581193 0.014534628 -0.062610342
0.17725512 0.037013731 -0.142922503 0.122382313
0.209580322 -0.09477708 -0.018948924 0.119829305
0.331091363 -0.183497666 0.172123591 0.044491618 -
0.070345518 0.110846389 -0.102542374 0.052436767 -0.068648945
-0.051151015 0.0935075 0.195405089 0.072169573 0.086055456
0.029142578 0.213973364 -0.22894494 0.106517333
0.068239796 -0.300284236 0.044525354 0.039659425
0.117086343 0.070158176 -0.043308333 -0.005609998
0.133747029 -0.190448633 0.020428774 0.101165638
0.118741788 0.055745556 -0.197079879 -0.046899259
0.046419924 -0.095093293 0.278334448 0.158870846 -

0.212459637 0.157631835 0.091715831 -0.109468428 -0.095278204
KEGG_BLADDER_CANCER 0.23712364 -0.187537803 -0.049301798
0.114012571 -0.170633373 0.16148714 -0.243575246 -
0.040545451 -0.039509371 -0.092630091 -0.341131186 -0.070531138
-0.247274845 -0.003167927 -0.317745067 -0.003109665
0.17987713 0.000788956 -0.102995155 -0.016049391
0.05304994 0.206376763 0.045110392 0.302215731
0.10139226 -0.169788966 -0.289138738 0.111350159
0.191247412 -0.172962459 0.19059879 0.077677031 -
0.052092134 -0.180758168 0.067720022 0.39477885 -0.093237651
0.125797142 -0.303371976 -0.117384317 -0.167242839
0.260091307 0.215669908 0.149443997 -0.091720437 -
0.053210078 -0.078232244 -0.243087521 0.136188489 -0.092754669
-0.255328336 0.135113628 0.145469674 -0.026736472
0.301910883 -0.044454101 -0.083670514 0.149416388
0.201145623 0.0736948 0.165067633 0.235694674 0.177605801
0.19461701 -0.23811574 0.024462298 0.187026326 -
0.291780649 -0.051138636 -0.338304303 0.075269233 0.002925002
-0.346829863 -0.091138388 0.090345271 -0.196219506 -
0.007208115 0.12134783 0.218393345 -0.419741815 -0.156063406
0.466711471 0.023563387 0.091054857 -0.053497625 -
0.021748438 0.16849104 -0.233453101 -0.230839252 -0.060572719

-0.183517665 -0.138456927 0.247858565 0.176021005 -
0.028535291 -0.190301997 -0.01054536 -0.071014028 0.366453937
-0.158670575 -0.286123865 -0.298493991 -0.149398964 -
0.154012974 0.170053919 0.061307483 0.120408135 -0.469461313
-0.037636816 -0.217227701 -0.192503572 -0.076902051
0.393855679 0.211008014 0.309037513 -0.164059207
0.004223406 -0.274431135 -0.257336062 -0.37820965 -
0.245660251 -0.067439439 0.217935276 -0.235866627 -0.147659315
-0.151829052 -0.248793236 0.154444905 0.223388146
0.078719791 -0.249829851 -0.220968508 0.18040129
0.057338404 -0.192486754 0.046425226 0.281827094
0.204668378 -0.185867059 0.130699043 -0.120428122 -
0.1230682 0.233342793 0.170260481 0.116657127 0.017567554
0.306158894 -0.129613499 -0.071165332 0.09521357
0.043064656 0.216585216 0.019109625 0.017609239 -
0.010787488 -0.155336917 -0.01524332 -0.048224723 0.101845738
-0.043800732 0.102008546 -0.256631375 0.180137444 -
0.071056068 0.316510713 0.253252774 0.322291981 0.059806391
-0.217468691 -0.235622051 0.378509921 -0.324292519
0.049587926 0.271120870.125905697 0.362310565 0.021088678
-0.040433116 0.428578833 0.110846093 0.00970128 -
0.065899433 -0.229548464 0.28794 0.459976216 0.383480159

0.304315609 -0.089507107 0.003378522 0.274314563 -
0.099194437 -0.250062711 -0.030255495 -0.273223953 -0.144191301
0.128762296 -0.062008998 -0.066839775 -0.183105898 -
0.008270711 -0.076338027 -0.18347309 -0.202588548 -0.228384828
0.179586696 -0.187614633 -0.057063124 -0.042027274
0.218245564 -0.091990256 0.256284989 0.233837258 -
0.004712103 0.161306654 -0.088538754 0.281790739 0.156431993
-0.095568898 -0.307716364 -0.356710846 -0.091482147
0.227872842 -0.117704644 0.089729219 -0.319379598 -
0.144548121 0.136525039 -0.379286078 0.300703959 -0.018053448
-0.093202255 0.051928862 -0.163538215 0.117969457 -
0.19104413 -0.127134378 -0.002214351 0.032686002 0.074060362
-0.080360753 -0.217120916 0.206386204 -0.145818013
0.338293409 -0.189073352 0.329442071 -0.006665213 -
0.158144133 -0.236644348 -0.194877041 -0.315331274 0.248184861
-0.005514757 -0.062916428 -0.09434522 0.269257134 -
0.037965578 -0.060889166 0.203482127 0.047879297 0.011761448
0.223632331 -0.181075028 -0.153080241 0.021130135 -
0.033745695 0.198162153 -0.046895633 0.25110728-0.249551471 -
0.138190302 0.013496195 -0.154163634 -0.17912924 -0.174485356
-0.006319758 -0.023634893 0.073576575 -0.156606744 -
0.131944694 -0.342430871 0.308895919 0.014839332 -0.00956498

-0.369559906 -0.198414829 -0.359119621 -0.099550013

0.325514976 -0.127271927 0.012551511 -0.14521581 -

0.036764368 0.079629786 0.000532364 -0.118666323 0.160727032

-0.356804901 0.003597957 -0.339992145 -0.064505079 -

0.431937546 -0.09071623 0.15729355 -0.277312361 0.096268897

0.245263807 0.26841675 0.046982556 -0.316110157

0.054875589 0.088536 -0.165005908 0.187555661 -0.132285987

0.097550942 -0.017336964 0.32838773 0.064119038

0.315271817 -0.216533617 -0.019088458 0.222661495 -

0.218404857 -0.091326793 0.384110213 0.113726406 -0.422650027

0.187970135 0.339610701 -0.115621731 0.129522909 -

0.068221514 0.18625596 0.29904659 0.284795366 0.147697069

0.249807499 0.07991824 0.088627954 -0.306063943

0.191438104 -0.420715792 0.035994635 -0.203371221 -

0.086122704 0.22638374 0.168296851 0.332029003 0.075808478

0.14846101 -0.084421708 -0.013009049 -0.293237729

0.029010346 -0.245129073 -0.342867345 -0.093987338 -

0.104085905 -0.244451107 -0.165693343 -0.38575328 0.467056832

0.34235703 0.03429816 0.206499313 0.01973966

0.171975903 -0.058586448 0.089601227 -0.026024461

0.15476225 0.466829251 0.362642503 0.275762316

0.010580563 -0.130611099 -0.000684554 -0.023475526

0.065623133 -0.296299724 -0.206492689 0.058698904 -
0.307452744 0.014395292 0.335298266 -0.179594843 0.187690285
0.355067421 0.246069194 -0.12002755 -0.2368563 -0.071771356
-0.016880301 0.2564129 -0.045164072 -0.068824552 -0.271901135
-0.003548361 0.104235947 -0.257990756 0.207652666
0.073953576 0.047100788 0.037378703 -0.063713094 -
0.141859497 0.204429267 -0.003339362 -0.00268005 0.019641218
0.186172994 -0.315325665 -0.06796594 -0.118676123
0.127476865 0.084451511 -0.239656557 0.168479218
0.235483783 -0.219373159 0.244878505 -0.150951086
0.319045532 0.022431915 -0.237748964 -0.388495249
0.071215043 -0.212083878 -0.057388845 -0.429750527
0.282743227 -0.126821317 -0.397899716 -0.29060645
0.176898103 0.334178806 -0.259587357 -0.084806999
0.247590927 0.002007941 -0.048206608 -0.372774144 -
0.202426682 0.224019281 -0.319332968 -0.185852502 -0.239786001
-0.06221935 0.254833231 -0.131482744 -0.04944742
0.320120272 -0.092816579 -0.074488163 0.077976173
0.298147425 -0.084599077 -0.076421515 0.058707548
0.272926167 -0.110783507 -0.333106624 0.068913298
0.399774009 0.232340731 -0.164042481 -0.323495134
0.002427528 -0.107629208 -0.050935413 -0.28356867

0.275655126 0.273768592 -0.12236276 0.166665749 -
0.016662033 0.269331212 -0.18673216 0.060797299 0.479572113
0.177084864 0.035031938 -0.063782274 -0.111252475 -
0.172791735 0.10130708 -0.126049154 0.056418662 0.351639316
-0.087590129 -0.068508758 0.29456179 0.053221999 -
0.146666115 -0.297122263 -0.050549857 -0.069103781 -0.411771947
-0.306258429 0.195549923 0.372118335 0.185094227
0.058266956 0.143993732 0.174372613 -0.212593862
0.016029016 -0.09852957 -0.04554502 -0.029316515 -
0.140205727 0.068797952 -0.20641655 -0.138401649 0.08044174
0.161157568 0.214222949 0.22925246 -0.00937457
0.082130153 -0.226338331 0.239081056 -0.362451158
0.158524842 0.075908943 0.291425605 0.00013132
0.38824467 0.15170035 -0.121842938 -0.056242244
0.043637649 -0.161942131 -0.258006444 -0.244691017 -
0.007064302 0.327484108 0.245971178 -0.283590146 0.003480055
0.298629978 -0.22343137 -0.081045199 0.093788315 -
0.196973748 0.111173027 0.05142444 -0.152381654 0.17855334
-0.275808477 -0.040914273 0.300723068 -0.177188886 -
0.040091129 -0.237862758 0.13663042 -0.193621935 -0.108037085
-0.151551499 -0.048205657 -0.171559076 -0.005655913 -
0.016992296 -0.244252019 0.21255272 -0.134115922 -0.150191881

-0.120460439
KEGG_CHRONIC_MYELOID_LEUKEMIA -0.019280153 0.162918673
0.056848117 0.303789466 -0.466747112 0.077368755 -
0.254841687 -0.247728949 0.032381743 -0.117115059 -0.168138926
-0.209309369 -0.318793859 0.048394866 -0.452404284 -
0.239611171 0.331402571 0.143493586 -0.123914185 -0.03174137
0.38994282 0.315123893 0.212522683 0.462152074 -
0.063129616 -0.251609617 -0.457968578 -0.044542194 0.011059748
-0.43680597 -0.12301112 0.262997795 0.065855462 -
0.320784549 0.178693451 0.231220345 -0.197384124 -0.139886508
-0.164329797 0.178628801 -0.211270422 0.211096823
0.345747448 0.09652014 -0.006699174 0.137291812
0.174190021 -0.229637935 0.044811745 -0.228391045 -
0.189895664 -0.00948542 0.372624738 0.287116231 0.387337485
0.253399688 -0.371367593 0.159781701 0.243748535
0.306102462 -0.058315568 0.216892191 0.317333566
0.294130885 -0.1993647 0.14512872 0.183705609 0.089222804
-0.104677313 -0.389924313 0.424370853 0.320674991 -
0.27887189 -0.038274251 -0.125737933 -0.385172744 -0.231118829
0.200078035 -0.163500752 -0.310268629 -0.226349097
0.388672372 0.14069394 -0.033339045 -0.118961403
0.216836715 0.234514528 0.103072615 -0.107294793 -

0.236173373 -0.406144697 -0.348744166 0.305624381 0.209500168
0.26151001 0.094048718 -0.069820901 0.062967545 -
0.067512754 -0.201024939 -0.064829152 -0.39764656 0.011477896
-0.20753773 -0.03896774 0.095757273 0.173789299 -
0.401544493 0.340817596 -0.359743049 -0.338184391 -0.262852283
0.064569176 0.267838585 0.219915481 0.290081157 -
0.263724663 -0.513336802 -0.501263157 -0.087807071 -0.443729187
-0.037127318 0.119166928 -0.292856494 0.098723075 -
0.405539647 -0.403788667 0.334741694 0.296570652 0.313387244
-0.100705289 -0.237638847 -0.149691662 0.179881442 -
0.191470603 0.393114834 0.335147711 0.329150851 0.186761645
0.337640546 -0.174884742 -0.185010781 0.346330199
0.035170865 0.337451673 0.075227328 0.258426132 -
0.089927769 -0.327039823 -0.231284588 -0.022683893 0.34307352
0.19600973 -0.077317627 0.11419611-0.300705322 0.132337398
-0.188200397 0.141389554 0.013110108 0.305274358 -
0.158282363 0.275971026 -0.179454599 -0.022862348 0.300073109
0.299765856 0.202904804 -0.372625241 -0.15949274
0.24894117-0.349758089 -0.319461075 0.093871639 0.420441144
0.346140656 0.113164467 -0.062872488 0.239025585
0.315030549 -0.180463575 -0.094347459 -0.264048114
0.191833704 0.414746711 0.298875988 0.200931354

0.043251856 -0.094542934 0.312134394 -0.154883373 -
0.422645431 -0.013170161 -0.394192431 -0.312313952 -0.318864333
-0.229213124 0.056614429 -0.28377505 0.200640463 -
0.068696181 0.005889475 -0.118266908 -0.450110294 0.13358278
-0.143749636 -0.222271439 0.051548227 0.401839602 -
0.148298019 0.273113295 0.116893799 0.136584248 0.319661335
-0.432001614 0.27503744 0.106033976 0.216057481 -
0.159693937 0.175108565 0.143249225 0.140774782 -0.280930556
0.312761771 -0.115482749 -0.058146251 0.062663096 -
0.430403456 0.410346713 0.066714298 -0.284648803 0.093249865
-0.066439385 0.021978206 -0.147916184 0.200458297 -
0.082188923 0.254114924 0.287076753 -0.199978888 0.163735889
0.286305822 -0.198621133 0.301737065 -0.204210851
0.307982757 -0.05033805 -0.053850924 -0.152739608 -
0.293650892 -0.172977243 0.097678682 -0.338648069 0.284878821
-0.268954912 0.289416632 0.105718994 -0.218926561
0.30400361 -0.086828667 -0.056241729 0.269399582 -
0.235934437 -0.300599591 0.199882244 0.247334243 0.21329193
-0.098230921 0.397004657 -0.44518042 -0.364798732
0.23143688 0.084253466 -0.02334502 -0.297741416 -
0.171701258 0.140518074 -0.072100958 -0.227522657 -0.139173411
-0.286969129 0.054729421 0.133831313 -0.28154016 -

0.257636707 -0.367964789 -0.414100884 0.082802996 0.245328918
-0.284686785 0.16583092 -0.073195078 0.224514742
0.457003167 -0.185042187 -0.308577368 0.305079258
0.168230528 0.013489349 -0.238925709 0.141247782 -
0.429198519 -0.355358463 -0.130592719 -0.337459912 0.137544679
0.372797267 0.339487387 0.255654363 -0.388180053 -
0.069402404 -0.122798991 -0.000963391 0.406110516 -0.223636825
0.07354586 -0.322795502 0.264105607 0.043539453
0.384287835 -0.371099761 0.150442872 0.194293023 -
0.416522788 -0.157003909 0.176378073 -0.196035654 -0.480230287
0.313259835 0.100044533 -0.076727851 0.104592822 -
0.150198389 0.182724071 0.441839318 0.208173617 0.324138627
0.219562821 0.41380366 0.306163917 -0.156511355
0.118078763 -0.417909093 0.221879822 0.272363994 -
0.286090891 0.257473649 0.112460279 0.211613179 0.155662658
0.163542824 0.109429521 0.241266187 -0.360617889
0.144639447 -0.422919242 -0.346481343 0.012881489 -
0.192420234 -0.481772214 -0.134214358 -0.245899512 0.35802395
0.306165257 0.021454731 0.394873174 -0.009182633
0.294055996 -0.25862151 0.256503495 0.095616803
0.445543255 0.39706322 0.21847303 0.357334736 -
0.263221648 -0.267113342 -0.221008567 0.233598675 0.318293295

27796 -0.471682542 -0.305019357 -0.30803559 -0.330347401

27797 0.094486756 0.050057501 -0.029863819 0.360340387

27798 0.367049374 0.301687792 -0.00299587 0.100099636 -

27799 0.188655118 0.124342638 0.33530258 0.256333227 0.080128531

27800 -0.376352345 0.146657525 0.096182689 -0.167458385

27801 0.294397705 0.310671573 -0.070157586 -0.095424624

27802 0.007067321 -0.179685189 0.244004577 0.280429479

27803 0.167284165 -0.003972396 0.322850113 -0.456052068 -

27804 0.022021572 -0.285833646 0.206925242 0.256809633 -0.081453815

27805 0.267319682 0.148408267 0.082112041 0.146282751 -

27806 0.084463115 0.459412871 -0.15612908 -0.184762891 -0.438393493

27807 0.425066132 0.078729979 0.252880234 -0.42078268

27808 0.299601705 -0.019356914 -0.495503694 -0.235495707

27809 0.276361476 0.523586283 -0.167295951 -0.09078916

27810 0.219679426 0.261561137 0.308905689 -0.132365387 -

27811 0.328539723 -0.109354081 0.201409909 -0.224827038 -0.25687048

27812 -0.064426416 0.229941051 0.239398187 -0.305338485 -

27813 0.008170481 -0.310292437 -0.317849852 0.310799555 0.237976165

27814 -0.02876073 0.06822025 0.191740479 -0.069604062

27815 0.018644491 -0.322051291 0.117594753 0.30531694 -

27816 0.098548757 0.116428161 -0.293508237 0.293583661 -0.060543274

27817 -0.258621655 -0.184997581 0.38262474 0.383500444 -

0.155560228 0.143619195 -0.060980459 -0.098845949 -0.03196701
0.15931737 0.437470957 -0.105955058 0.037052825 -
0.240552775 -0.277799529 0.012407556 0.371122141 -0.237732792
-0.034686998 0.243077486 -0.240107986 -0.184555534
0.359985784 0.296030635 -0.128536895 -0.345360146 -
0.33145943 -0.011480896 -0.290534492 -0.17359583 0.198326484
0.255397554 0.109624566 0.224112133 0.322075233
0.318300067 -0.183762254 0.269391284 -0.269595631
0.059195349 -0.040455761 -0.26036136 0.292286844 -
0.43056823 -0.137697012 -0.013374748 0.160978375 0.306743643
0.323934505 -0.068732705 -0.167725441 -0.267973902
0.211197295 -0.348257488 0.215350927 0.120340899
0.342067698 0.023612382 0.356243965 0.364611919 -
0.186898628 -0.202799527 0.207581408 -0.040026174 -0.275002451
-0.148133152 -0.063035587 -0.117297235 0.14190553 -
0.107794139 -0.092082705 0.079076837 -0.059176183 0.058413874
0.046444131 -0.202348224 0.148381238 0.03155881 -
0.102480551 -0.217162736 -0.195737736 -0.147872851 0.233273492
-0.162731032 -0.008912502 -0.214992482 0.196602243 -
0.068235924 -0.226565295 -0.17716748 -0.013894017 -0.134716131
-0.013440519 0.056424005 -0.165803621 0.071762337 -
0.183842021 -0.151469954 -0.22425437

KEGG_ACUTE_MYELOID_LEUKEMIA -0.203752453 0.299131969 -
0.002750749 0.229960573 -0.34540545 -0.079530238 -0.14535877
-0.083704008 -0.040847293 -0.008432285 -0.191256231 -
0.173467208 0.006870405 -0.237106246 -0.238084331 -0.240402238
0.426318142 0.256214261 0.01540335 0.192928123
0.321618073 0.411847371 0.242376235 0.25969754
0.079617798 -0.32797645 -0.448428398 0.037351102 -
0.159393565 -0.235947941 -0.171528313 0.148548951 0.24813274
-0.186832526 0.211668585 0.1888243 -0.278611528 0.02044722
-0.107244937 0.211398543 -0.301044418 0.29968188
0.21429753 0.011907819 -0.193200555 0.096314643
0.204209156 -0.126169779 -0.097373539 -0.058374668 -
0.291616084 -0.182990906 0.2479558 0.289310145 0.378584997 -
0.164848571 -0.261141814 0.143529424 0.238200053 0.179216258
0.038451592 0.258250522 0.295475204 0.265102198 -
0.320827517 0.144072417 0.166116441 -0.091443413 -0.231680725
-0.161029747 0.251959301 0.344649042 -0.252877132 -
0.225614644 -0.090770142 -0.129865109 -0.273406797 0.06681411-
0.019139354 -0.367920711 -0.201537993 0.235929036 -0.09142597
0.141133511 -0.085102317 0.031397847 0.116174181 -
0.083981572 0.163450525 -0.14061842 -0.395142786 -0.263543406
0.419549955 0.076756091 0.089828285 0.239493224 -

0.043073803 0.162296295 0.042850398 -0.106394909 0.079601107
-0.211589484 -0.103598338 -0.041837457 -0.103271243 -
0.089509594 0.134986164 -0.436998954 0.329247435 -0.046310782
-0.130351183 -0.100059635 0.050453386 0.106605559
0.049033073 0.206561112 -0.10234995 -0.471949741 -
0.435657268 -0.196325483 -0.249339338 -0.006288558 0.122804155
-0.343022673 0.110455756 -0.286096433 -0.350479257
0.287052677 0.101539436 0.276420384 -0.16564847 -
0.117041496 -0.056608882 0.056142438 -0.355416503 0.383886647
0.320071766 0.207639369 0.254668823 0.370248656 -
0.135275463 -0.133594111 0.359780976 -0.062881497 0.250968398
-0.112435726 0.303975137 -0.248311002 -0.15118583 -
0.052817573 0.020120083 0.404800636 0.261019912 0.070512916
0.017328017 -0.225245455 -0.00029084 -0.22249251 -
0.053567406 0.063742953 0.284289192 -0.175242842 0.318924604
-0.138898224 -0.154393736 0.167656081 0.094815459
0.183653197 -0.131107213 -0.17694745 0.125803232 -
0.340038816 -0.119011693 0.153156371 0.407326098 -0.013412626
0.207564468 -0.145270121 0.249194837 0.283273981 -
0.14510595 -0.137810772 -0.145448141 0.070301853 0.379026335
0.223165312 0.062819759 -0.17058703 0.001495147 -
0.089949305 -0.132650256 -0.418339621 0.195071168 -0.330346459

27884 -0.204458745 -0.147892441 -0.276399026 -0.080640538 -

27885 0.371944378 0.230488611 -0.019579731 0.120128254 -0.138340778

27886 -0.382451878 0.125931716 0.118756962 -0.2204576 -0.057119774

27887 0.092823993 -0.026776013 0.253299064 0.293264628 -

27888 0.121432421 0.262111641 -0.256023572 0.322115513 -0.140265111

27889 0.185298797 -0.068761468 0.222537613 0.242992704

27890 0.165798605 -0.065256082 0.069324569 -0.137680314 -

27891 0.003893332 -0.086481816 -0.325129117 0.399243593 0.074551483

27892 -0.242899546 0.245877827 0.198123647 0.029190717

27893 0.028987773 0.028492163 0.154077374 0.167806856

27894 0.22795569 -0.229679485 0.047827172 0.272926822 -

27895 0.028021088 0.178979716 -0.34725647 0.297257015 -0.021117673

27896 -0.262493025 -0.191132447 -0.147084737 -0.167622198

27897 0.072058647 -0.13918915 0.276142095 -0.095195239

27898 0.347946959 -0.061278456 -0.10593251 0.280086909

27899 0.139266916 0.016292913 0.288438949 0.084230813 -

27900 0.283058229 -0.017312589 0.152999919 0.274659109 -0.251502488

27901 0.492010333 -0.220485234 -0.317703572 -0.036500815 -

27902 0.007712402 -0.217350476 -0.111254594 0.208895886 0.273369546

27903 -0.045208627 -0.160618055 0.085067477 -0.090916105

27904 0.123054053 0.194106019 -0.20950661 -0.024776309 -

27905 0.282284292 -0.225185927 0.063803025 0.129426353 -0.133624687

0.088018678 0.026886063 -0.018419674 0.355021377 -
0.143878406 -0.142216131 0.233318899 -0.03245691 0.06125501
-0.018699629 0.193570635 -0.333838435 -0.178690386 -
0.291852427 -0.285962576 -0.028302808 0.1622421 0.171486996
0.178710441 -0.225576008 -0.110757166 -0.127271744 -
0.000807582 0.142542685 -0.096376393 -0.057995858 -0.357098395
0.118176224 0.211417301 0.191335571 -0.270579633
0.165925671 0.16750224 -0.322557767 -0.164077805 -
0.157400746 -0.178958846 -0.44863286 0.249974227 -0.13597903
-0.109876644 -0.03497241 -0.185912637 0.059339464
0.314170431 0.270839642 0.276393419 0.335686171
0.391069443 0.266530695 -0.209165409 -0.226893626 -
0.208992581 0.188903077 0.032800233 -0.151930643 0.056669725
0.084472938 0.057732471 0.228230063 0.124951391
0.154677802 0.34092025 -0.323901401 -0.053303704 -
0.196684206 -0.149691198 0.175814393 -0.217181931 -0.429075906
-0.256993881 -0.188771913 0.095494128 0.383803763
0.04521743 0.333798448 -0.04415378 0.15374258
0.073198669 0.183837698 0.097451592 0.411339029
0.255269115 0.307407887 0.340208858 -0.080573987 -
0.296841256 -0.086208301 0.216158079 0.154136135 -0.403679402
-0.230275683 -0.124357418 -0.242932631 0.162444029

0.02865797 -0.156716212 0.185651959 0.320863738
0.242665683 0.07980234 -0.068232671 -0.278204902
0.069800984 0.280383534 0.159860229 0.287720046 -
0.340443219 -0.151205488 0.294385841 -0.189624226 0.281262925
0.202006966 -0.26591374 -0.085351727 -0.172459307 -
0.237427719 0.182448373 -0.091993354 -0.025653576 -0.112550814
0.243461553 -0.445569242 0.154195198 -0.099245345
0.299104376 0.206883207 -0.184411901 0.298210485 -
0.100830235 0.207163125 0.046498018 -0.223586856 0.351883414
0.100745962 -0.185937688 -0.454713656 0.492318016 -
0.035871265 -0.161338778 -0.397275658 0.244795399 -0.167216656
-0.382349012 -0.391457693 0.373990313 0.507870265 -
0.119486422 -0.151420561 0.046022396 0.288920081 0.087005953
0.08019132 -0.180987705 0.057875699 0.211997749 -
0.106464333 -0.024239325 0.014202497 0.289397831 -0.046003194
-0.170631908 -0.13189618 -0.185742514 -0.126762043
0.29939477 0.133609688 0.028583317 -0.113933068
0.004022434 0.074555163 0.20079447 -0.187912713
0.085033566 0.343873042 -0.030678811 0.074421183 -
0.276544766 0.095249061 0.208696647 -0.326213645 -0.308610203
0.039994761 0.367535804 -0.151894935 0.297263935 -
0.171301965 -0.171768899 0.280551697 -0.062136762 0.346002993

0.020761232 0.024774957 -0.034692366 -0.16812827 -0.229899413 0.02835208 -0.039648213 -0.006728109 0.014616727 -0.087699252 -0.27733685 0.037102542 0.069890169 -0.233053226 -0.200283437 -0.386821446 0.317691936 -0.308961558 -0.231734574 0.216388036 0.178148293 0.24434045 0.256467829 0.244004009 0.208005736 0.0462929 0.270194234 -0.071609826 0.105717327 0.101683326 -0.201101387 0.226487441 -0.36528005 -0.158478811 0.025908668 0.076290987 0.099475419 0.363788471 -0.072197122 -0.303771219 -0.248621708 0.242327294 -0.261366908 -0.126211018 0.007622641 0.412647485 0.028205575 0.325007969 0.187322384 -0.059973837 -0.340797872 0.212115294 0.132274056 -0.383523037 -0.089499031 -0.012214703 -0.209125462 0.059019942 -0.120991963 -0.121344301 0.221049494 -0.093482706 0.187768372 0.00607377 -0.129638823 -0.068639398 -0.036872257 0.016508568 -0.386198939 -0.015690769 -0.058959135 0.010580886 0.2398461 -0.157709076 -0.287723124 0.127073686 0.07455148 -0.219841104 -0.151384105 0.016509182 -0.090657376 0.121704779 0.000563255 0.011891081 0.043220305 -0.211241352 -0.166341705 -0.151783748

KEGG_SMALL_CELL_LUNG_CANCER -0.212009979 -0.153015964

27972 0.031554444 0.251432533 -0.362493658 0.014179525 -
27973 0.05107386 -0.222550258 0.20549726 0.039872641 0.071130008
27974 -0.223780165 -0.04777768 0.164193898 -0.299931871 -
27975 0.229825835 0.127758781 0.157282541 -0.010874364 -0.002978476
27976 0.283679017 0.096788968 0.233339979 0.475044627
27977 0.085622768 -0.263084993 -0.408406392 -0.097456163
27978 0.067667017 -0.235296318 -0.151307149 0.252654132 -
27979 0.091213059 -0.1681127 0.247620236 0.316669213 -0.069224867 -
27980 0.147928267 -0.123757543 0.140992639 -0.182424587 0.213352185
27981 0.224511901 -0.127743655 0.008099198 0.217724318
27982 0.04952974 -0.116152288 0.031165695 -0.383873688 -
27983 0.203306214 0.1045932 0.331341335 0.203448386 0.184045604
27984 0.089462585 -0.323434133 0.190478947 0.007223959
27985 0.320182256 0.017343809 0.124925118 0.078741607
27986 0.323701752 -0.30615946 0.135177099 0.107179102
27987 0.071558016 -0.133299768 -0.342008755 0.303531176
27988 0.392764621 -0.017218527 0.009912936 -0.254006444 -
27989 0.255877587 -0.213601664 0.028943444 -0.10380225 -0.29050749
27990 -0.179416753 0.367897301 0.051410948 -0.153004004 -
27991 0.178401159 0.026314649 0.336594236 0.245633022 0.011392285
27992 -0.140428478 -0.053351206 -0.277150438 0.197992047
27993 0.115765825 0.109752239 -0.016903759 -0.062253156

27994 0.12802039 -0.115873463 -0.16082136 -0.037289091 -

27995 0.237603018 0.000579272 -0.234844182 0.032549515 0.067927517

27996 0.049472222 -0.25959431 0.314191938 -0.337354464 -

27997 0.220293788 -0.321895366 0.19207068 0.199148873 0.286718925

27998 0.278856157 -0.277790966 -0.387155703 -0.229630262 -

27999 0.070777882 -0.354747059 -0.123471732 0.086418183 -0.161992567

28000 0.234008338 -0.364520357 -0.412006809 0.264370313

28001 0.145485796 0.101822292 -0.020385424 -0.045715314 -

28002 0.076118774 0.167055027 -0.243141686 0.329051747 0.324907687

28003 0.001151065 0.057317632 0.258996955 -0.235257537 -

28004 0.289555503 0.091776809 0.192496735 0.015108828 0.015515895

28005 0.143281418 -0.118267147 -0.131488097 -0.158716249 -

28006 0.203640111 0.33438287 0.282088963 0.081495876 -0.02605806

28007 -0.259084802 0.258779134 -0.312253585 0.194452110.078458559

28008 0.249557312 -0.217141331 0.162126058 -0.081755305

28009 0.005979229 0.271080204 0.12999195 0.130739178 -

28010 0.293828084 0.134984273 0.245228017 -0.293071339 -0.346755974

28011 -0.034346154 0.363696012 0.376781927 0.066083561 -

28012 0.118656284 0.281633926 0.154733179 -0.188132421 -0.097761568

28013 -0.223913853 0.060544982 0.312536542 0.158376935

28014 0.351241556 0.04747399 0.043159462 0.16659229 -

28015 0.234288804 -0.436196579 0.077154078 -0.224332016 -0.21581288

28016 -0.241691516 -0.224669577 -0.041247007 -0.032555809

28017 0.089059658 -0.056014704 -0.040536946 -0.004267103 -

28018 0.267896637 0.027810697 -0.310843225 -0.134519705 0.01417556

28019 0.239104395 -0.224055311 0.032932458 0.212326736

28020 0.210204454 0.278067185 -0.240285979 0.234900875 -

28021 0.119265596 0.084783786 0.008944398 0.218793605 0.15918812

28022 0.040563062 -0.236786574 0.155261851 -0.053173865 -

28023 0.212567562 0.036172285 -0.327591979 0.3362081 -0.1886906 -

28024 0.301881167 0.010160209 -0.092149667 0.264826648 0.035508109

28025 0.26094528 0.062112395 0.181473553 0.228291385 -

28026 0.242184862 0.196115781 0.247765752 -0.038414492 0.148088302

28027 0.013864394 0.281384866 -0.035296642 -0.021029941 -

28028 0.097157966 -0.341916384 0.012852767 0.092073509 -0.287159166

28029 0.409171091 -0.312358337 0.191716031 -0.088809382 -

28030 0.198978687 0.092503057 0.001332738 0.038736132 -0.007007235

28031 0.163488805 -0.146252393 0.026694032 0.204229907

28032 0.217920049 -0.21897602 0.065264228 -0.39126496 -

28033 0.286883261 0.101771605 0.071219087 -0.029939013 -0.097716082

28034 -0.246546114 0.171052203 0.045334223 0.011584114 0.0119982

28035 -0.043374547 -0.051811923 0.061244262 -0.267104411 -

28036 0.106649162 -0.31373465 -0.168616074 0.065813856 0.299944181

28037 -0.124732982 0.147238101 -0.178629676 0.28734956

0.401784026 -0.190438052 -0.242255038 0.165893729
0.129840713 0.020177365 -0.250656225 0.189407749 -
0.353421463 -0.219478062 -0.10198128 -0.267840601 0.0042271
0.229075357 0.314326741 0.323030607 -0.319280005
0.016348303 -0.301219568 -0.015946263 0.383111924 -
0.019486878 -0.038547466 -0.008271821 0.160629578 0.085307393
0.355510222 -0.266935434 0.178280902 0.162815504 -
0.338462257 -0.172956522 0.14436962 -0.197044614 -0.332756889
0.125101244 0.152570576 -0.074525541 -0.143350815
0.015996435 -0.053302931 0.435488025 0.034953341
0.39791466 0.194789767 0.272176475 0.121511723 -
0.264895486 -0.037235912 -0.233485481 0.228392393 0.008175859
-0.209271173 0.247482366 0.199920823 0.092619584
0.237943292 0.326628627 -0.036642194 0.290877955 -
0.400692187 -0.066716491 -0.355960635 -0.232984446 -0.162407276
-0.249364822 -0.407574345 -0.29877315 -0.125146804
0.227132429 0.215135086 -0.086599463 0.175671692 -
0.094050704 0.201744383 -0.263193801 0.250714021 0.131653639
0.392751271 0.271561446 0.132011077 0.11183113 -0.212533002
-0.120947913 -0.057669201 0.254942738 0.203346633 -
0.29843378 -0.205065792 -0.091753852 -0.169029397 0.011364644
-0.228153119 -0.013601454 0.277756605 0.321318021

28060 0.218542953 -0.230316081 -0.0161939 0.038118025 -4.13E-05

28061 0.110235849 0.263590195 0.093048517 -0.284333637

28062 0.317568264 0.142532451 -0.038088496 0.235973148

28063 0.295093998 -0.084310259 -0.207312455 0.006229027

28064 0.100103093 0.14049486 0.030388171 0.315394643 -

28065 0.21769586 0.346242395 -0.435734862 0.024192397 -0.234842961

28066 0.209912747 0.226692226 -0.131016185 0.123242066

28067 0.03522953 0.032846864 -0.110738758 -0.201805445

28068 0.366880768 -0.22653705 -0.135331396 -0.476893958

28069 0.344090725 0.158774522 0.278698794 -0.274330146 -

28070 0.01375917 -0.044775565 -0.377281759 -0.085285751 0.319913303

28071 0.326946537 -0.159957098 -0.061439036 0.222962789

28072 0.204232098 0.123103782 0.084524825 -0.070465721 -

28073 0.247189813 0.17115007-0.176982557 -0.246976291 0.032854326

28074 0.19782822 0.154002845 -0.322988091 -0.05623011 -

28075 0.378575337 -0.352316021 0.302033582 0.165160979 -0.088724232

28076 0.054196011 0.147914453 -0.172872764 0.015360647 -

28077 0.082796211 0.022947098 0.298714642 0.029192998 -0.034768577

28078 -0.212955197 0.164839049 -0.154903596 -0.079293038 -

28079 0.092010603 0.071471403 0.330345075 -0.047004919 0.240384995

28080 -0.090356465 -0.08478484 0.14422404 0.130844029

28081 0.35044271 -0.326023304 -0.088687123 -0.219373378 -

0.229249838 -0.149881305 0.254406223 -0.103462104 -0.100484781
0.247792317 -0.291583787 -0.058780466 0.399475915
0.175626417 -0.07379608 -0.325058486 -0.255675915 -
0.162044178 -0.181474548 -0.061761267 0.109855707 0.131753021
-0.160087743 0.20069656 0.283303032 0.243187736 -
0.110583156 0.192170187 -0.295532248 0.179450019 0.058803747
-0.367505019 0.395118095 -0.301637496 -0.030275667 -
0.070926114 0.056731735 -0.001787597 -0.003410734 -0.240802597
-0.161826094 -0.187239138 0.283170956 -0.241667112
0.101540171 -0.072234256 0.24306178 -0.057713911
0.290268704 0.247336429 0.000173495 -0.128245955 -
0.081095376 0.028917928 -0.247349148 -0.097959575 0.146361867
0.000899354 -0.048447263 0.03092363 -0.160159243 -
0.104903083 0.004644289 -0.002716578 0.092157761 0.042946803
-0.052773258 -0.146271158 0.014762976 -0.288817551 -
0.036664029 0.090857547 0.132151754 -0.036412372 -0.206809594
0.026582867 0.085623313 -0.069950389 -0.194859866 -
0.214166853 -0.05476474 0.077760502 -0.124935432 -0.106886049
-0.007596956 0.049649661 -0.062873312 -0.110395841 -
0.257930086
KEGG_NON_SMALL_CELL_LUNG_CANCER 0.108730456 0.288168325
-0.095465012 0.247155492 -0.339902801 -0.02363861 -

28104 0.120600096 -0.349772855 -0.037654624 -0.005979663 -0.025257184

28105 0.085764416 -0.350725553 0.050232979 -0.32908033 -

28106 0.145465392 0.093113235 0.302962759 -0.065191896 0.115287993

28107 0.211822544 0.080459124 0.143510836 0.368343777 -

28108 0.101370052 -0.342401449 -0.384311809 0.024601776 0.162539908

28109 -0.254201942 -0.043270158 0.231984519 0.121798719 -

28110 0.199486094 0.078240094 0.199732468 -0.310492363 -0.068618556

28111 -0.266842318 0.296239199 -0.067395563 0.225889975

28112 0.303768727 0.278058449 0.019019873 0.060292543

28113 0.105579632 -0.12140068 0.001695197 -0.16732973 -

28114 0.125720593 -0.050602686 0.292256858 0.074139079 0.250945154

28115 0.118374449 -0.293533941 0.015398368 0.251594254

28116 0.299770003 -0.150177764 0.253026983 -0.092161832

28117 0.300929388 -0.3163607 0.109743222 -0.05147379 -0.026367924

28118 -0.309625669 -0.22635251 0.29960721 0.265217729 -

28119 0.113970859 -0.178213299 -0.15723298 -0.176289685 -0.266850791

28120 0.258736132 0.049275762 -0.300822949 -0.25670575

28121 0.338866336 0.03960501 -0.165363362 -0.089528528

28122 0.093663257 0.15123009 0.082063994 -0.042611086

28123 0.039236658 -0.314485213 -0.387907923 0.265217459

28124 0.329433309 0.22648955 0.241729653 0.061192469

28125 0.177148002 0.039822999 -0.084032319 -0.144073168 -

0.346139523 -0.08280485 -0.146920016 -0.044354987 0.135107098
0.113153125 -0.116868342 0.258079658 -0.237437648 -
0.332169692 -0.209089132 0.127504959 0.005864801 0.288969934
0.240907986 -0.073463275 -0.389004703 -0.278822956
0.022069893 -0.30492212 -0.017687863 0.187827732 -
0.223530656 0.182060387 -0.283535714 -0.231566977 0.312272962
0.174631656 0.279840954 0.035367172 -0.119404559
0.01731629 0.188304387 -0.118654 0.037602456 0.143615307
0.40118926-0.03755501 0.27446174 -0.093462424 -0.252100779
0.263208567 0.072164215 0.110884382 -0.125591018
0.229280672 0.08225868 -0.34695856 -0.257242395 -
0.06092885 0.190216238 0.040058946 -0.099069049 0.176875726
-0.376908644 0.010731121 -0.094232584 0.320830963 -
0.006561658 -0.115113116 -0.181536157 0.013316589 -0.211324001
0.067410091 0.060822993 0.365697725 0.220929157 -
0.286227081 -0.126814711 0.159050122 -0.280533817 -0.235507775
0.038486908 0.291193950.172908906 0.181970598 0.134764506
0.103580069 0.27371552 -0.141363054 0.017714674 -
0.009624893 0.059015347 0.365431412 0.416130987 0.065406554
-0.026617966 -0.167278817 0.189492126 -0.137449053 -
0.402791962 -0.086799552 -0.347008986 -0.270737348 -0.162191154
-0.198863724 0.2459849 -0.25764512 0.224035721 -0.291017671

28148 0.103043756 -0.145528277 -0.290630323 0.196200331 -

28149 0.009683376 -0.089563056 0.007584979 0.155913252 -0.127713123

28150 0.297577411 0.244466121 0.016692354 0.271132567 -

28151 0.304617403 0.290728646 0.025230343 0.143028608 0.025951879

28152 0.238447698 0.031152458 0.125174571 -0.252269499

28153 0.133190233 -0.13512453 -0.205344973 -0.041662968 -

28154 0.431499277 0.223161996 0.051747863 -0.089090065 0.067225595

28155 0.011454558 0.220369404 0.098518545 0.188836448 -

28156 0.018668101 0.026043546 0.318814007 -0.211606496 0.071545945

28157 0.269355332 -0.009280373 0.23155565 -0.185184323

28158 0.337493173 0.131839407 -0.007574605 -0.060136472 -

28159 0.139177218 -0.070765364 0.090939936 -0.193513515 0.193558865

28160 -0.122933511 0.257105103 0.133567349 -0.165264011

28161 0.157580067 -0.07695536 -0.112131182 0.367230139

28162 0.08226674 -0.181605184 0.064398926 0.184851403 -

28163 0.070391585 -0.20067408 0.349367425 -0.32096467 -0.190343188

28164 0.012776598 0.135480416 -0.176842024 -0.217595136 -

28165 0.031240018 0.221058507 -0.101206607 -0.050687931 -0.139863075

28166 -0.285556149 -0.004218811 0.191515593 -0.325015597 -

28167 0.257475972 -0.213100688 -0.248391132 0.199360107 0.115984195

28168 -0.336513014 -0.157394968 0.086003632 -0.049167601

28169 0.35802976 -0.228291079 -0.264828824 0.317471269

0.233838505 -0.067025338 -0.15154578 0.044525401 -
0.238812697 -0.354497527 -0.253914514 -0.28116899 -0.048761265
0.047553264 0.356679325 0.043281112 -0.275409475
0.003256521 -0.150443535 -0.032564992 0.342956142
0.024916392 0.240459955 -0.305867593 0.379704035
0.074586471 0.370825648 -0.286641552 0.221088741
0.288328565 -0.37635219 -0.001730856 0.094319904 -
0.056619504 -0.345210509 0.27463464 0.070411048 -0.141304905
-0.052503891 -0.267296859 0.084453199 0.336527132
0.197445662 0.218157945 0.029724727 0.436980332
0.177631211 -0.111483568 0.090611763 -0.166564418
0.008058777 0.26306085 -0.232224972 0.330593589
0.177318161 0.235185035 0.119316416 0.03379868 -
0.043550017 0.198323478 -0.245044777 0.141705334 -0.351841288
-0.311351734 0.02163548 -0.038755583 -0.248455872 -
0.140609035 -0.147864846 0.30684087 0.159679715 -0.069738
0.386183002 0.022059417 0.106997727 -0.23995122
0.108529798 0.088321635 0.346277172 0.360030244
0.28080961 0.237909202 -0.208927253 -0.18937581 -
0.1491837 -0.071608622 0.277463803 -0.412572569 -0.352540004 -
0.09542973 -0.312183242 0.180734447 0.028647398 -0.124983012
0.153150024 0.14432803 0.252612043 0.13309376 -

0.085581043 -0.27717557 0.030566756 0.059462262 0.298090753
0.055961458 -0.377032498 -0.017550154 0.066674844 -
0.139659292 0.306592177 0.192815265 0.131466106 -0.160968627
0.168339382 -0.075148245 0.135606323 -0.073913613
0.169485427 -0.10619244 0.265137554 -0.412019555 -
0.223637824 -0.252731463 0.013208476 0.259696892 -0.098162548
0.173865705 -0.046841617 0.16337162 0.093956924 -
0.029879851 0.321089599 -0.276447639 -0.120762586 -0.42333629
0.211069907 -0.086179384 0.089163272 -0.334215662
0.305692578 -0.319270368 -0.256084598 -0.265004674
0.136622023 0.461386317 -0.014956383 -0.197326281
0.138539682 0.267015004 -0.019448036 -0.019779427 -
0.324182486 0.053451229 0.359622236 -0.204220504 -0.213366842
-0.284822627 0.306360103 -0.012057809 -0.132486787
0.121418147 -0.14802227 -0.147505581 0.245060078
0.195452334 -0.155105762 -0.122219738 0.137666698
0.130479654 0.184975932 -0.210924148 0.02751351
0.289129816 0.039551782 0.141994046 -0.3564805 0.312822965
-0.246651259 -0.263860686 -0.010892805 0.213590062
0.191858207 -0.12048758 0.175233493 -0.227964048
0.000441281 0.253559479 0.005508534 0.33739347 -
0.090900207 -0.014402505 -0.161860646 -0.029706506 0.081373141

0.330755571 -0.167739464 0.043200971 0.155721686 -
0.14245453 -0.247852668 0.208560074 0.351382172 -0.150061249
-0.315250649 -0.320305043 -0.068487949 -0.239052408 -
0.160593471 0.129157849 0.181470843 -0.033943797 -0.101254596
0.155280787 0.149701713 -0.068312187 0.244306299 -
0.37552681 0.081455851 -0.004852166 -0.165745664 0.15901585
-0.365632291 0.037269079 0.102046496 0.081486129
0.199139954 0.420904096 0.054809413 -0.028368123 -
0.214494346 0.306484339 -0.15450515 0.099529339 -0.101555816
0.296022271 0.023247411 0.174757843 0.158553098 -
0.037507926 -0.2273141 0.017824864 0.081898142 -0.275660492 -
0.044996959 0.04393641 0.014689266 -0.057771294 -0.20947257
-0.035163347 0.17528354 -0.086974932 0.058874906 -
0.001145345 -0.210657054 0.023328404 -0.096136319 -0.189460862
-0.196962595 -0.116475243 -0.119460333 0.083226863 -
0.120521295 -0.129868189 -0.047865296 0.261135541 -0.142353914
-0.032910295 -0.215836666 0.050439937 0.030833913 -
0.095690445 0.069360645 -0.259471278 0.11913801-0.217448636 -
0.145686302 -0.076668437
KEGG_ASTHMA -0.659123043 0.22008889 -0.54864176 -
0.115066748 0.14202809 0.424897395 -0.245848421 0.332551235
0.388541813 0.493466556 0.391682456 -0.620875995

0.352411341 -0.645399926 0.656203119 -0.472507442 -
0.490739719 0.507746162 0.208093313 0.510345473 -0.571159895
0.445570207 0.233076241 -0.709738277 0.377791082
0.035480771 -0.004434324 0.114511888 0.38890777
0.04105215 -0.568520138 0.420834167 0.225510306
0.681119334 0.297585921 -0.109839522 -0.329258313
0.326650613 -0.329001884 0.297544645 -0.450160829 -
0.048679319 -0.434483349 0.473249704 -0.423600338 -0.242458821
-0.442936527 -0.386243859 -0.231652685 0.565146553 -
0.463242499 -0.302591766 -0.046950888 -0.468691108 -0.312450925
-0.5631451 0.042574066 -0.206003329 -0.071673736 -0.376413446
-0.108503843 0.541281528 -0.239493917 -0.434597378 -
0.401734325 0.137935158 0.16047337 -0.700211528 0.34841811
0.634177596 0.436112912 0.504149619 -0.55093916
0.560729383 -0.455853553 0.509039851 0.55888461 -
0.325039554 0.480650874 -0.554245498 -0.301318954 0.148605765
0.652496229 0.279430305 0.350166226 0.070050675 -
0.667090786 -0.268650152 0.649469711 0.010773378 -0.540613628
0.170076975 -0.16749222 0.054437635 0.417485469
0.667558752 -0.446337522 0.590064513 0.275298326 -
0.235313295 0.454577746 0.553629651 0.018265845 0.552703596
0.12032165 0.13426693 -0.410241392 -0.332878272 -

0.406630225 0.68894235 0.228836806 0.37226349 -0.322793989
-0.578948753 0.127441154 0.392695858 0.438356712
0.446321441 0.341962172 -0.374319314 0.312479924
0.048632024 -0.212664693 -0.703252559 0.243609653
0.376771403 -0.474246016 0.258936539 -0.294367395
0.669504558 0.34187774 0.650731257 -0.503277417
0.442779159 0.301941611 0.193039074 -0.2600772 -0.480194667
-0.248746256 -0.346396255 0.390514788 0.066001497 -
0.528120628 0.298300188 0.423639136 -0.567338939 0.438385805
0.443050861 0.508182223 0.105522995 0.689024137
0.081841523 0.617610251 0.601346306 -0.086758673 -
0.283836856 0.447188093 0.584978719 -0.286487258 0.404531831
-0.493923843 0.089715376 0.169642561 0.300046283 -
0.263650243 -0.48598592 0.260145536 0.463477969 0.556151286
0.053595803 -0.615830771 -0.117878846 0.339943385 -
0.411436849 0.048652105 -0.642276844 0.255904169 -0.258282333
-0.141763108 0.399035083 0.494027001 -0.499914267
0.600739902 0.177683243 -0.52466504 -0.215677155 -
0.637741474 0.119289686 0.654976966 0.094658336 -0.353148362
0.467938502 -0.156475769 -0.413116537 0.534309602 -
0.126478871 0.452407505 0.34683564 0.034451739 -0.213410729
0.056751843 0.379624131 0.003617514 -0.122834944 -

28280 0.008252664 0.442871785 -0.367033653 -0.170951741 -0.667427562

28281 0.127694206 0.260030856 -0.215239453 -0.20700836

28282 0.160887302 0.488754677 0.094131083 0.328710287

28283 0.121941963 0.263368789 0.449781072 0.582902105 -

28284 0.46187023 0.007662124 -0.278835797 0.318925795 0.34014105

28285 0.116558466 0.214696613 -0.701792898 0.549235056 -

28286 0.304132889 0.482539089 0.075927378 0.234753703 0.113603473

28287 -0.568603217 0.580812935 -0.164294651 0.391883652

28288 0.375518101 -0.093902925 -0.164961585 -0.42989849 -

28289 0.140610658 -0.665309446 0.002930074 -0.071947295 -0.493816055

28290 0.438636599 0.407105286 -0.021456391 0.086263394

28291 0.204970928 -0.151988326 0.125556007 -0.029069219 -

28292 0.38101287 0.058796845 -0.276392542 -0.22494794 0.192553745

28293 0.524816684 0.320687734 -0.362817502 0.393937147

28294 0.029611953 0.150687421 -0.360037632 0.384068213

28295 0.624424599 0.287844247 -0.513278007 0.514927524

28296 0.449881886 -0.022430167 0.495300958 -0.279433795

28297 0.419035172 -0.377361868 0.297212456 0.516278401

28298 0.473530553 0.277005329 0.053403756 0.520053983

28299 0.228794843 0.377852418 0.566694802 0.17707174 -

28300 0.520187782 -0.511978383 0.599450708 -0.598210716 0.24680641

28301 0.607815978 0.586640201 -0.277611667 0.48229597

28302 0.047916372 0.5972533 0.521861922 -0.210722984 0.509108379

28303 0.155195775 -0.243993627 -0.016646517 0.327125611 -

28304 0.572224001 0.426253704 0.476504374 -0.120251309 0.011296977

28305 0.64763903 -0.497129592 -0.122299658 -0.475477433 -

28306 0.116341524 -0.377424915 -0.430731057 -0.338359667 -0.504794198

28307 -0.48561272 -0.424566822 0.508829027 0.23238509

28308 0.243719817 -0.397741753 -0.217012357 0.036115899 -

28309 0.483872677 -0.231682706 -0.47314894 0.233487815 0.416265807

28310 -0.534799716 -0.374870691 -0.101701637 -0.172607308

28311 0.421452741 0.368248511 0.565886827 -0.179160865

28312 0.65474413 -0.581706297 -0.43393471 0.616761197

28313 0.01042817 -0.430405498 -0.5839928 -0.257879878 -0.390396698

28314 0.656216976 -0.516840858 0.267707003 -0.699183519

28315 0.568276964 -0.128417257 0.315213006 -0.276742489 -

28316 0.023767606 -0.425637859 -0.651026515 -0.274388603 0.409706556

28317 0.50487252 0.251765665 0.2215787 -0.518161629 0.014413648

28318 -0.527817395 0.232465818 -0.495790195 -0.644264351

28319 0.375697842 -0.141964828 -0.393412551 -0.001139437

28320 0.196432607 0.45805514 0.129500878 -0.332080162

28321 0.405120345 0.295219744 0.505631406 -0.404020721 -

28322 0.221867456 -0.075264085 0.441319014 0.180592651 -0.505527841

28323 0.384519802 0.569251293 0.352755412 0.315065744 -

0.147159046 -0.446486353 0.135749753 -0.302559574 -0.381652387
-0.254483874 0.61725652 0.546409918 -0.165166516 -
0.206105708 0.202242567 0.364427622 0.095263215 -0.354050901
-0.242668556 0.039221933 -0.204314887 -0.027111103 -
0.055859011 0.124974898 0.215416945 0.516685155 0.317181634
-0.290794163 0.562043118 -0.33155967 0.637052798 -
0.258034156 -0.537997635 -0.179245688 0.486877835 -0.098036679
0.211934425 0.408201803 -0.29600015 -0.766714326
0.293901083 0.295807292 -0.196376693 0.210982701 -
0.491969335 0.276786631 0.120044738 -0.610885985 -0.512812587
-0.695253644 -0.149699112 -0.625333657 0.299182223
0.137188616 0.466899478 0.38099835 0.365700684
0.477532491 -0.543094682 0.279749128 0.187472937 -
0.554042302 -0.597063993 0.46464185 0.612162784 -0.345095066
0.176620236 -0.54250294 -0.371443404 -0.461987486
0.34454651 0.609486169 0.585908717 -0.345469918 -
0.249109566 0.263776455 -0.419420956 0.630239442 -0.707759822
0.063730399 0.291357066 0.096684272 -0.118196823 -
0.147042058 -0.421647287 -0.050692854 -0.188823944 -0.355059053
0.599425695 -0.027147363 -0.623740787 -0.028187263
0.387440836 0.463101656 0.397385413 0.21765528 -
0.369167333 -0.019163798 -0.173259039 -0.667776938 -0.207971672

-0.419212041 -0.552675811 0.402595063 -0.405560846 -
0.185660921 -0.569974649 0.475988194 0.036387512 0.64348104
-0.040019854 -0.625611745 -0.163609531 -0.47195018 -
0.138506703 0.043401429 0.521701123 -0.144699358 0.491846677
0.093436738 0.221704202 -0.168572799 0.386766667
0.029291211 -0.550604594 0.142471859 -0.18975845 -
0.40640288 -0.019741333 0.360719433 -0.182713625 -0.568055726
0.222944329 -0.393436109 -0.450430381 -0.112612365 -
0.077832712 -0.341609647 -0.377393993 -0.162788054 0.394658591
-0.504362189 -0.210034407 0.004047602 -0.480056267
0.188351023 -0.228523745 -0.221837971 -0.287592024 -
0.377920909 -0.193473616 -0.287058226 -0.177289615 0.555767073
0.275028526 0.087717297 0.129249677 -0.237034626
0.231861246 -0.24989077 0.343937283 -0.003772236 -
0.37162485 0.393683655 0.230358304 -0.174491821 -0.404719016
0.027817094 -0.274056651 0.488129674 -0.421152751
0.15623111 0.193875987 0.412181904 0.203511744 -0.368798158
0.342498108 0.164953431 0.159430454
KEGG_AUTOIMMUNE_THYROID_DISEASE -0.485902552 0.474331678
-0.580188325 -0.153006481 0.267017489 0.216859286 -
0.127858423 0.337341106 0.212872939 0.431409624 0.505246529
-0.533578875 0.233126815 -0.597857763 0.518322054 -

0.243102716 -0.399304983 0.460023566 0.292809891 0.630065177
-0.572229697 0.591469647 0.398860741 -0.632718358
0.544888394 0.020034485 0.194718003 0.112223798
0.23194325 -0.090806493 -0.448206039 0.440352301
0.390106248 0.639551628 0.278423019 -0.370916599 -
0.196322168 0.432675887 -0.44556528 0.22404858 -0.402588522
0.228966101 -0.468713674 0.698139824 -0.275592151 -
0.321422643 -0.333468645 -0.430127632 -0.434705 0.574057582 -
0.358706069 -0.127067525 0.019606838 -0.430065673 -0.323006335
-0.600879298 -0.081980789 0.213624119 0.272795504 -
0.423854471 0.045317797 0.454958061 -0.110867308 -0.374165272
-0.331268924 0.013430084 0.181297868 -0.664132632
0.515081188 0.666166724 0.515266603 0.54427932 -
0.419727957 0.328825927 -0.489264305 0.559082575 0.442565503
-0.33304592 0.383740287 -0.384665813 -0.377025028
0.318503953 0.723180637 -0.272177173 0.336544304
0.269276465 -0.621491114 -0.328402659 0.669352351 -
0.284936553 -0.51635025 0.31150702-0.053993653 0.27607333
0.483015843 0.655027506 -0.393085626 0.672657651
0.239643388 -0.325193654 0.429745083 0.551331143
0.127641187 0.486472266 0.055439449 0.09557394 -
0.431878149 -0.306686572 -0.426187586 0.666836958 0.414326047

28390 0.215621067 -0.209311666 -0.547200779 0.146431732

28391 0.469283344 0.487931656 0.426976314 0.196340937 -

28392 0.460830414 0.365624836 0.048929165 -0.262045077 -0.584737548

28393 0.318968733 0.36509044 -0.30884799 0.157370738 -

28394 0.142044879 0.652141365 0.553556583 0.657563458 -0.401882087

28395 0.548305028 0.382610125 -0.005355563 -0.128233992 -

28396 0.485017387 -0.328424018 -0.310270856 0.082766755 -0.19594311

28397 -0.609484893 -0.286612697 0.083255255 -0.601575081

28398 0.495146467 0.437502437 0.358122527 -0.028307614

28399 0.595461146 -0.013185091 0.59745842 0.531872551

28400 0.055522222 -0.176935924 0.010017049 0.441437708 -

28401 0.476642491 0.570255936 -0.411194317 0.16013365 -0.018413194

28402 0.34330021 -0.265755993 -0.453250907 0.548331361

28403 0.232153712 0.433240765 -0.066686603 -0.572435659 -

28404 0.313199066 0.215428611 -0.422656528 -0.241929891 -0.643127458

28405 0.113615559 -0.249803423 -0.202108347 0.507066109

28406 0.44487413 -0.468943648 0.512182156 0.131638458 -

28407 0.308657048 0.028093676 -0.474776314 0.135873837 0.635793179

28408 -0.014421089 -0.254021286 0.403417611 -0.207084466 -

28409 0.377589003 0.463321121 -0.257734408 0.349389671 0.218379279

28410 0.013290329 -0.373058081 0.125947086 0.290097931

28411 0.073469288 -0.151733499 -0.11324636 0.443802243 -

0.429475167 -0.290101685 -0.568089079 0.275548379 0.422586611
-0.532710289 -0.430466487 -0.170206817 0.417161596 -
0.290037356 0.259307961 0.464239436 0.072404723 0.441221774
0.549168515 -0.298270333 0.112573801 0.066277075
0.361194856 0.108713963 0.132006764 0.361491467 -
0.622267013 0.530354442 -0.040575356 0.374944469 0.486840362
0.544051112 0.362770776 -0.533986211 0.531226147 -
0.381786354 0.475181044 0.464672805 -0.104616682 -0.065710111
-0.190899794 0.063692466 -0.606624565 -0.176729253 -
0.162758597 0.072459213 0.537323119 0.421289072 -0.066027781
0.072782397 0.054637862 -0.14296726 -0.011604848 -
0.151335203 -0.30179442 0.323530692 -0.071034971 -0.268739883
0.302950281 0.64979987 0.083261854 -0.432071373
0.369448585 -0.356775075 0.021343916 -0.313698219
0.515426266 0.660502913 0.325056808 -0.563323661
0.457899661 0.374433138 -0.15029391 0.659909045 -
0.140100776 0.393346526 -0.557457794 0.417395433 0.44065486
0.44643047 0.267092383 0.358247309 0.560483769
0.397894586 0.481418079 0.608086975 0.114992651 -
0.417520793 -0.513180212 0.666231133 -0.480284054 0.470904848
0.30426724 0.60779784 -0.379702036 0.470409652
0.205934977 0.717368333 0.564852626 -0.372002082

28434 0.48505503 -0.013061663 -0.088141033 -0.308959976 -
28435 0.021174932 -0.37975506 0.314198643 0.599504225 -0.342664556
28436 -0.038961131 0.627803455 -0.516089315 0.137283152 -
28437 0.52379062 -0.141470957 -0.569441351 -0.286469358 -0.067343744
28438 -0.3828903 -0.530134129 -0.488021524 0.470789065 0.284821195
28439 0.195150469 -0.1251037 -0.074489277 0.103591513 -0.474510891
28440 -0.381448928 -0.559583111 0.129886387 0.154077125 -
28441 0.503342756 -0.354029839 -0.37042153 0.233668907 0.561371194
28442 0.384932845 0.608333627 -0.209053198 0.593387233 -
28443 0.390249761 0.055826701 0.531576881 -0.133066875 -0.194000917
28444 -0.221028458 -0.407394358 -0.543868298 0.373557636 -
28445 0.559901718 0.555514959 -0.58239854 0.563291348 -0.36417485
28446 0.320892196 0.070016873 0.168349106 0.062811173 -
28447 0.537210062 -0.067995206 0.384443068 0.388101344 0.212917349
28448 0.200822282 -0.313823201 0.168164033 -0.532304076 -
28449 0.114217005 -0.632986344 -0.58965655 0.102555593 -0.095722173
28450 -0.29072272 -0.219269793 0.311942391 0.459895795
28451 0.267716322 -0.255652401 0.036668504 0.10403206
28452 0.61570508 -0.323913476 -0.315383314 -0.048042889
28453 0.508168319 0.367361523 -0.513832299 0.596147983
28454 0.558449913 -0.313815944 0.219608102 -0.364149931 -
28455 0.566899175 0.132227123 -0.410780885 -0.406059463 -0.075841791

0.647719945 0.312103129 -0.395639846 -0.435902353
0.051103960.407688119 -0.427738369 -0.31744947 -0.188353854
-0.22999557 -0.198284811 -0.219405237 0.211982945
0.340332571 0.118360068 0.38055354 0.136435317 -
0.307998152 0.400408015 -0.2712095 0.666586495 -0.227242764 -
0.545593957 -0.350161674 0.391870669 -0.423656628 0.247200294
0.47535947 -0.327258251 -0.604135934 0.282317504
0.521509044 0.026836168 0.260320658 -0.470547047
0.044959545 0.350576202 -0.462014923 -0.565869561 -
0.642162909 -0.366576896 -0.583407382 0.301490064 0.157560959
0.33543614 0.133235522 0.221628674 0.578153086 -
0.444279358 0.005051981 0.18577957 -0.399073865 -0.603476402
0.449117707 0.637927922 -0.409641786 -0.254472017 -
0.558978499 -0.180630001 -0.532329249 0.348693953 0.65190846
0.576300587 -0.281396209 -0.103798686 0.435358862 -
0.337269077 0.543619428 -0.620524399 0.083794618 -0.240552318
0.042991538 -0.32665108 -0.314099867 -0.496398784 -
0.287805408 0.194592008 -0.249163776 0.504233987 0.171390085
-0.466445072 0.194842682 0.112478062 0.278387121
0.476187914 0.209043983 -0.413359144 -0.007418289 -
0.444813383 -0.667164499 -0.117917536 0.003385068 -0.308652491
0.528737358 -0.382348102 -0.020875481 -0.321972253

0.61546845 -0.304556642 0.648252938 -0.179514906 -
0.482064396 0.065554073 -0.047035796 -0.437702358 -0.289158258
0.612446667 -0.231002242 0.537539774 0.177380194
0.152054812 -0.154458723 0.072115933 -0.100795798 -
0.621170707 0.248426184 0.132751005 -0.426956004 0.279623634
0.434700998 0.092807878 -0.255561969 -0.069923855 -
0.37244421 -0.396026628 -0.257298056 -0.177972667 0.075478427
-0.098620184 -0.001853905 0.246236824 -0.533769105 -
0.173003048 -0.15696007 -0.45229475 0.04844487 -0.272554441
-0.392710055 -0.270596188 -0.215554374 -0.371467968 -
0.027894296 -0.029440166 0.339914031 0.409368235 0.187858125
0.07307086 -0.361720784 0.210061035 -0.220150668
0.314904016 0.103470673 -0.340981285 0.385779315
0.097122616 0.029415282 -0.476892823 0.271292578
0.08373144 0.345060657 -0.366086645 -0.00610856
0.090645339 0.487018754 0.052674685 -0.188744981
0.270401479 0.295637042 0.250741257
KEGG_ALLOGRAFT_REJECTION -0.566712229 0.610294129 -
0.659988663 -0.143696818 0.211873012 0.157350004 -0.294747641
0.27871303 0.355106284 0.484843418 0.462959879 -
0.544299383 0.240961758 -0.706414605 0.608109758 -0.384688941
-0.52719707 0.619301808 0.162377398 0.767957563 -

28500 0.656411455 0.629583444 0.47878074 -0.726819613 0.594390738

28501 0.092934257 -0.041718895 0.161101570.252164115 -0.380393564

28502 -0.630046171 0.483388821 0.459104586 0.756048345

28503 0.397268109 -0.364157289 -0.344760154 0.474746465 -

28504 0.610332295 0.443501682 -0.607429881 0.134648798 -0.407680868

28505 0.726957538 -0.182748047 -0.256080327 -0.34562667 -

28506 0.479321466 -0.516189727 0.590223685 -0.45542154 -0.340669547

28507 0.216484435 -0.558221376 -0.473577008 -0.640230507 -

28508 0.380581388 0.257877562 0.266887685 -0.493987465 0.289033298

28509 0.576168113 -0.174122784 -0.491767369 -0.512354284 -

28510 0.022303652 0.293805159 -0.779498418 0.467495691 0.654274398

28511 0.631372634 0.723209701 -0.535911319 0.412259002 -

28512 0.533776893 0.525621703 0.590789946 -0.41962577 0.48455591

28513 -0.476132161 -0.53386432 0.416449457 0.843306654 -

28514 0.269331768 0.418214949 0.295292565 -0.713094182 -0.190871309

28515 0.745409774 -0.201451045 -0.623954335 0.037330799

28516 0.057230703 0.348322489 0.690732318 0.756073843 -

28517 0.379683028 0.676356129 0.324903631 -0.443582215 0.629372374

28518 0.5455723 0.231713702 0.563889601 -0.029719257 0.191278512

28519 -0.595460894 -0.486088524 -0.504126784 0.695157916

28520 0.189482231 0.168328273 -0.275643498 -0.631060938

28521 0.003806886 0.636224887 0.548224287 0.351003691

28522 0.001005969 -0.553274094 0.183034269 -0.005653102 -
28523 0.349619354 -0.698100722 0.343097514 0.138291687 -0.445489491
28524 0.345517683 -0.20823516 0.741654686 0.69938804
28525 0.689024405 -0.501138174 0.632926867 0.51545295 -
28526 0.035729269 -0.02818702 -0.581854501 -0.404629883 -0.457914972
28527 -0.036081552 -0.356297319 -0.655964823 -0.429231629
28528 0.202909593 -0.645866106 0.542312599 0.419718204
28529 0.210326887 -0.060817789 0.662759855 0.202869122
28530 0.674536723 0.644187703 0.271333979 -0.413624538
28531 0.039011155 0.556060514 -0.454345222 0.656346752 -
28532 0.478912924 -0.120362006 -0.02485156 0.264632981 -0.348389625
28533 -0.567938475 0.548462725 0.251350591 0.333075036 -
28534 0.142308856 -0.648068358 -0.491144962 0.249497169 -0.548641926
28535 0.024561851 -0.760020093 0.250252836 -0.336709023 -
28536 0.255735231 0.607324625 0.519955873 -0.50872716 0.621372478
28537 0.095027033 -0.449935406 0.121509585 -0.657045193
28538 0.173951086 0.698897276 -0.139064314 -0.227930103
28539 0.386052407 -0.289930191 -0.555742444 0.579766189 -
28540 0.314905791 0.417295588 0.267867696 -0.151772899 -0.416608453
28541 0.169530464 0.509602045 -0.007295575 -0.389259301 -
28542 0.069474688 0.607486307 -0.465384348 -0.281991169 -0.658059365
28543 0.387098641 0.55561092 -0.665365469 -0.44416894 -

0.0087515 0.304244315 0.096969924 0.278514121 0.451466221 -
0.25656135 0.509585871 0.619331196 -0.298225043 -0.205182536
0.09742878 0.381496039 -0.006656516 0.218104888
0.232229682 -0.697048624 0.507559442 -0.363222075
0.524559778 0.453060291 0.525038553 0.339579503 -
0.667430911 0.652925904 -0.297393884 0.586793157 0.521499573
0.133393551 -0.098486452 -0.521516851 0.100404269 -
0.660197441 -0.117511324 -0.268397916 -0.022925356 0.600191216
0.38087724 -0.267201378 -0.072365528 -0.163131482
0.033379928 -0.065038324 -0.288308035 -0.358827461
0.153217668 0.085912107 -0.344167114 0.409137075
0.70288639 0.261565992 -0.544244661 0.515992967 -
0.355276877 -0.228148568 -0.453923276 0.63934219 0.642513284
0.457391089 -0.673438271 0.604921564 0.441502186 -
0.174122698 0.737233657 -0.201075054 0.50498245 -0.68378595
0.483870546 0.46206346 0.531781508 0.412198265
0.033862044 0.562293613 0.310667881 0.445189092
0.722866235 -0.003451432 -0.64102367 -0.583638413
0.736179289 -0.58816343 0.543318379 0.262775839
0.607287873 -0.39716571 0.576005351 0.347459631
0.774208849 0.635659786 -0.461978245 0.525920848 -
0.170510281 -0.190478859 -0.310524287 -0.06834449 -0.379181268

0.217769318 0.58903792 -0.371951801 -0.134459895
0.709858842 -0.633414397 0.104839528 -0.568392292 -
0.354398076 -0.633629642 -0.29093012 0.010830997 -0.624697138
-0.563505697 -0.527268283 0.473673066 0.26555897
0.185977866 -0.12189038 -0.215380754 -0.132006368 -
0.618673527 -0.372146956 -0.583576411 0.009259355 0.146491101
-0.577065686 -0.33073479 -0.232238207 0.258020218
0.735724788 0.561641356 0.725802829 -0.502402025
0.715625111 -0.438437372 -0.254757638 0.576053355 -
0.067643666 -0.266506342 -0.296315223 -0.4510295 -0.52749818
0.487491541 -0.613534112 0.589632879 -0.73864174
0.508163167 -0.542854242 0.460437509 0.106865361 -
0.024193459 0.029876292 -0.617870965 -0.163912971 0.423275964
0.539007447 0.228465837 0.271521195 -0.396996504
0.116696295 -0.570996214 -0.031978248 -0.631745416 -
0.655145448 0.252015036 -0.108926784 -0.404234614 -0.370661479
0.336668899 0.541887406 0.642657871 -0.42756273
0.069148795 0.231170937 0.673346978 -0.344633899 -
0.274351028 -0.125330955 0.591187317 0.499554935 -0.562185602
0.682189269 0.68005831 -0.416888813 0.395091389 -
0.359035079 -0.60258837 -0.201589261 -0.511873926 -0.565178583
-0.165333643 0.651972936 0.296525598 -0.345630905 -

0.499809302 -0.041139363 0.467091627 -0.471367394 -0.41577489
-0.167706321 -0.192401909 -0.253670737 -0.230870817
0.079516467 0.243756162 0.091475844 0.344858273
0.22837274 -0.356666921 0.450024848 -0.23001382
0.751357126 -0.356686054 -0.690080568 -0.204231357
0.402924468 -0.425773798 0.01772582 0.604438653 -
0.268592661 -0.738183299 0.091545121 0.573624859 -0.059571778
0.044604865 -0.587330274 0.062278852 0.478238427 -
0.609463353 -0.664979353 -0.782463054 -0.288312088 -0.724839698
0.477395768 -0.139561988 0.508490148 0.257869079
0.09766722 0.620617375 -0.631144812 0.060929479
0.250418664 -0.586182936 -0.70219391 0.457234687
0.70414271 -0.474916939 -0.247869355 -0.64422885 -
0.38659775 -0.560067444 0.500804726 0.768081993 0.590473625
-0.265521481 0.04342553 0.416317252 -0.267442325
0.611919663 -0.665966311 -0.131485385 -0.280815165 -
0.126048894 -0.268370085 -0.36035059 -0.533541004 -0.271704075
0.05069351 -0.325408122 0.567446317 0.13616691 -
0.421012999 -0.019985033 0.316807342 0.045768267 0.545615904
0.289421397 -0.40054549 -0.014972477 -0.512168555 -
0.754429746 -0.324282195 -0.364660149 -0.254264152 0.667297174
-0.343345021 -0.326559648 -0.470874996 0.70952789 -

0.417398564 0.783632583 -0.232357086 -0.60469735 0.086295331
-0.065558253 -0.480674346 -0.286088297 0.683497624 -
0.207557855 0.438204008 0.329331643 0.240739043 -0.193100225
0.20584232 -0.419615451 -0.677212736 0.263719404 -
0.163632239 -0.421851457 0.304606212 0.541856788 -0.016874574
-0.460074673 0.044540428 -0.51972741 -0.490692836 -
0.35813717 -0.150156617 0.157737605 -0.02132434 -0.03960366
-0.038963197 -0.668494687 0.101466142 0.179376877 -
0.646554898 -0.14759165 -0.463986342 -0.37664472 -0.343246943
-0.302913068 -0.486442777 0.002694764 -0.010881314
0.606524202 0.303581598 0.053042567 0.256140538 -
0.380674051 0.082054549 -0.37626953 0.560647141 0.309494986
-0.495124185 0.526423475 0.061726836 -0.046973548 -
0.293756784 0.081091633 0.276932209 0.441609406 -0.521444937
-0.17830342 -0.101449259 0.492508579 0.190465468 -
0.42407741 0.295510955 0.352893056 0.234428014
KEGG_GRAFT_VERSUS_HOST_DISEASE -0.519340991 0.637998359
-0.674513246 -0.294499853 0.313009205 -0.018699613
0.025827514 0.297552349 0.358359447 0.434925693
0.438710132 -0.516057909 0.213421322 -0.73626768
0.540542494 -0.388007125 -0.495249182 0.657436237
0.20383879 0.709028904 -0.597333351 0.592103256

28632 0.570455358 -0.630695272 0.653445012 0.118630814

28633 0.012126471 0.2136015 0.182494876 -0.365434406 -0.57955362

28634 0.556483449 0.499242808 0.735294841 0.484920382 -

28635 0.430103615 -0.322170565 0.511813996 -0.719600604 0.046353203

28636 -0.535604776 0.445208374 -0.392999772 0.68603187 -

28637 0.193455234 -0.321700437 -0.452065746 -0.533125272 -0.491417409

28638 0.529837495 -0.453948334 -0.325426949 0.328570663 -

28639 0.605863223 -0.418890284 -0.623048094 -0.341997799 0.259125383

28640 0.257818215 -0.337377311 -0.102067945 0.544765654 -

28641 0.044448261 -0.541785408 -0.445321229 -0.005501395 0.479671347

28642 -0.789237264 0.489576275 0.619238068 0.643667251

28643 0.739975831 -0.558587119 0.343739699 -0.59433863

28644 0.54337216 0.564735421 -0.530737379 0.535781879 -

28645 0.451364873 -0.409275095 0.380176925 0.807269466 -0.258291877

28646 0.484856548 0.270620805 -0.721451646 -0.307597589

28647 0.727802939 -0.085310512 -0.628817248 0.245298015

28648 0.014956917 0.406340451 0.510986743 0.634132775 -

28649 0.324783934 0.666772882 0.293569483 -0.437228049 0.670985368

28650 0.4198781 0.078846223 0.523141385 -0.041260094 0.104145118

28651 -0.582039566 -0.492551681 -0.491771985 0.656183611

28652 0.26817086 0.247141435 -0.253528613 -0.681336489

28653 0.032386864 0.672194252 0.457527675 0.335388586 -

0.062771768 -0.503087172 0.11839327-0.088546463 -0.168296178 -
0.722668034 0.189914457 0.117210186 -0.465569885 0.288542077
-0.273541688 0.722244915 0.663760234 0.660960032 -
0.543219731 0.673200965 0.502325113 -0.191425249 -0.141683636
-0.59392178 -0.39661438 -0.47476279 -0.200064047 -
0.509103155 -0.648529692 -0.46988959 0.182578072 -0.659217116
0.543666967 0.481029508 0.235220883 0.111571277
0.659001165 0.258037603 0.668497034 0.629296993
0.37493229 -0.364306351 -0.020975676 0.519626659 -
0.48723322 0.709466793 -0.397887176 -0.119563992 -0.23456764
0.259534216 -0.269915004 -0.515817045 0.612650127
0.308605108 0.306688735 -0.118289738 -0.628879741 -
0.517938493 0.36277318 -0.54573345 -0.038557319 -0.769493499
0.004343032 -0.319316244 -0.359755975 0.657206554
0.652361305 -0.49069317 0.570471754 0.329083426 -
0.404889925 0.238702154 -0.629024551 0.144437409 0.671609687
-0.01578677 -0.252260888 0.385372099 -0.290030031 -
0.552662645 0.603553148 -0.015675699 0.427148428 0.181182014
-0.266335459 -0.404253381 0.101353311 0.496238793 -
0.062406627 -0.399372127 0.105361815 0.501636172 -0.430466415
-0.322639623 -0.6397175 0.417120443 0.488854318 -0.618322125
-0.473853294 0.051042556 0.277846202 -0.094521272

0.255377277 0.409203609 -0.370860986 0.315279999

0.661972099 -0.372999193 -0.080210978 0.136951238

0.403838438 0.045026828 0.244462457 0.281601932 -

0.651152263 0.541782433 -0.374763043 0.601223911 0.448041502

0.602978016 0.206448685 -0.650836356 0.659335613 -

0.226746946 0.493425064 0.538028603 -0.043510926 -0.056590445

-0.590294951 0.029520638 -0.715463502 0.101811134 -

0.222576887 -0.003244025 0.592319666 0.439004981 -0.319275291

0.020347014 -0.22925198 0.022278659 0.019444325 -

0.163080689 -0.420953473 0.30375482 -0.090111147 -0.278002019

0.418012321 0.740962005 0.282944282 -0.574225656

0.591470197 -0.370373129 -0.070050919 -0.452466864

0.590657142 0.634994943 0.42962997 -0.555210207

0.616221697 0.44885991 -0.190590343 0.719878284 -

0.050673369 0.457125741 -0.749089837 0.479717639 0.405502495

0.482137712 0.434890161 0.208539691 0.552243521

0.326288546 0.362213029 0.672703459 0.122149301 -

0.614260385 -0.550449574 0.773089472 -0.554958824 0.497305582

0.193216266 0.658549126 -0.4142728 0.322320323 0.276869899

0.790705584 0.458955644 -0.458554285 0.537095813 -

0.154389401 -0.247600974 -0.26128544 -0.252696541 -0.335408627

0.227822772 0.52124504 -0.400189988 -0.05897039

0.621986369 -0.580363104 0.156631176 -0.524604044 -
0.313043031 -0.590478775 -0.290256864 0.17660247 -0.683950702
-0.555215466 -0.341882878 0.433398255 0.223641575
0.503463355 -0.082846594 -0.270067496 -0.060984799 -
0.686683404 -0.375332 -0.625357261 -0.006748181 0.152382243 -
0.418261448 -0.320617578 -0.248863826 0.46104574 0.5993092
0.416947198 0.729591815 -0.518172563 0.711448842 -
0.279411958 -0.394687115 0.553023434 -0.290157731 -0.26594612
-0.052059557 -0.431571603 -0.472185695 0.433874612 -
0.607738393 0.500777885 -0.726571059 0.522864781 -0.550462823
0.426945414 0.142148904 -0.060642017 -0.064195028 -
0.649165025 0.003434263 0.479985839 0.526008307 0.1991521
0.058015707 -0.395668505 -0.00508187 -0.583584647 -
0.084120862 -0.606882602 -0.624068756 0.345488219 0.164391133
-0.393669009 -0.349946484 0.286203981 0.590227882
0.642555569 -0.357026618 0.180570675 0.190900817
0.646340403 -0.367069217 -0.297259461 -0.121787523
0.615816134 0.428266293 -0.536140591 0.63886392
0.68831014 -0.386416688 0.431449584 -0.251202617 -
0.602689384 -0.214607729 -0.477677338 -0.532596725 -0.127041249
0.557260449 0.24448652 -0.092284304 -0.485547288 -
0.059794246 0.408584993 -0.450439095 -0.373873788 -0.103164141

-0.18574858 -0.32278622 -0.343285149 0.283446568
0.257320144 0.128759941 0.492107853 0.199240047 -
0.333323551 0.4840047 -0.256792855 0.683607252 -0.283852677 -
0.702935023 -0.084334789 0.475048711 -0.456922738 0.03043636
0.708127212 -0.131566596 -0.676879976 0.156236233
0.512747683 -0.003211933 0.010568807 -0.596420641
0.139948701 0.469593828 -0.576236009 -0.71281715 -
0.786938354 -0.283288217 -0.717931827 0.57340746 -0.174485502
0.398808671 0.155416801 0.168224228 0.560164012 -
0.664415893 0.019336798 0.301481139 -0.548998831 -0.688111279
0.517702904 0.692140414 -0.425277454 -0.151763294 -
0.62606279 -0.501492959 -0.422802287 0.506182512 0.735829046
0.556561079 -0.087653963 0.183189457 0.462036702 -
0.301154892 0.624694363 -0.63984911 -0.17106575 -0.153349847
-0.11157343 -0.162285003 -0.47299888 -0.648484728 -
0.170808765 0.284160119 -0.339374211 0.447673452 -0.02715932
-0.405567404 -0.087619124 0.305534906 0.124137311
0.59267594 0.208999786 -0.502203786 -0.014972477 -
0.484799618 -0.759317331 -0.355087981 -0.342148054 -0.202
0.526178741 -0.323657441 -0.386406261 -0.405518524
0.744233975 -0.419001818 0.776946718 0.026352146 -
0.566956167 0.37588265 0.069431343 -0.539966745 -0.276691482

0.687029891 -0.207661583 0.460715958 -0.066116087
0.296073417 -0.103689638 0.257269673 -0.417257282 -
0.721616983 0.431573132 -0.204198686 -0.502520207 0.49142568
0.421628922 -0.234744078 -0.454791732 -0.035379131 -
0.387385532 -0.560428095 -0.382243338 0.098771922 0.165520483
-0.13600649 -0.291186543 -0.284516288 -0.692580316
0.35428685 -0.045556269 -0.563440531 -0.183880877 -
0.538032761 -0.438247253 -0.298216057 -0.385715857 -0.416288207
0.046088315 0.037760584 0.566671323 0.40154456
0.348329591 0.360927682 -0.289820204 0.088834146 -
0.403307994 0.490175824 0.289650558 -0.421045239 0.608065905
0.073690299 -0.11616959 -0.286808537 0.093218669
0.290029186 0.524752631 -0.486850966 -0.407318927 -
0.089919821 0.54864509 0.053210583 -0.285364038 0.3247046
0.220091146 0.142456394
KEGG_VIRAL_MYOCARDITIS -0.179940714 0.399361618 -0.558084653
0.060980968 -0.093315597 -0.242513812 -0.251284157
0.053903254 0.278524567 0.271514484 0.188072331 -
0.462579862 0.2085805 -0.424337086 0.264764176 -0.281278289 -
0.265769485 0.435373537 0.22126062 0.461839899 -0.36192914
0.412100207 0.332914501 -0.383627979 0.479901046 -
0.130130051 -0.261760727 -0.060545383 -0.035931861 -0.408105738

-0.359153738 0.246691166 -0.023951398 0.444187919
0.406887572 -0.471682331 0.091662977 0.288223769 -
0.316035341 -0.066960884 -0.425060074 0.309063862 -0.275329783
0.408844755 -0.166079793 0.201359988 -0.236682832 -
0.392963544 -0.319793745 0.259799125 -0.347996239 -0.112531607
0.295074527 -0.363253657 -0.012493734 -0.276548998 -
0.20334149 0.357245451 -0.061353704 -0.025684546 0.035588634
0.431370339 -0.156696914 -0.151491593 -0.326566663 -
0.006035373 0.187411651 -0.390696379 0.288626994 0.230280619
0.437189152 0.492651139 -0.444751676 0.154172482 -
0.220648683 0.24214378 0.348300415 -0.258597988 0.295045061
-0.31907071 -0.319116547 0.455637585 0.541234711
0.108894792 0.300489636 0.172818014 -0.441123393 -
0.055983043 0.488519818 -0.36138638 -0.399173633 0.14644342
-0.054507398 0.448642159 0.393888025 0.430159854 -
0.346635694 0.379412669 0.15779417 -0.102401712 0.446918751
0.10477112-0.022335342 0.476284582 0.271943313 -0.093959969
-0.409345141 -0.307253201 0.011036599 0.452531451
0.146303589 -0.08323231 -0.214259194 -0.22368783 -
0.247905892 0.297985146 0.025740521 -0.082393445 -0.264213301
-0.324967792 -0.173949705 -0.227049445 -0.035653652 -
0.507049214 -0.13339819 -0.044478079 -0.438533934 0.139228336

28786 -0.020132287 0.429051181 0.378899192 0.300800228 -

28787 0.373881438 0.320724736 0.434986973 0.277235116 0.08973941

28788 -0.184733949 -0.223630698 -0.014360232 -0.205459811 -

28789 0.209950799 -0.465462293 -0.392571451 0.183401017 -0.293683469

28790 0.195760863 0.157775982 0.156758407 0.216313596

28791 0.531104803 0.291456708 0.499267999 0.466881301

28792 0.082432412 -0.142943593 -0.035761964 0.218944143 -

28793 0.16287632 0.397250664 0.03100413 -0.046428659 -0.12091946

28794 0.013490759 -0.186145192 -0.232987488 0.345021375

28795 0.153767964 0.143175382 -0.078390144 -0.4545309 -0.379326585

28796 0.121660311 -0.334835406 0.234049891 -0.262266399

28797 0.123165979 -0.373353394 -0.095957685 0.435379584 0.3666108

28798 -0.417890949 0.223433462 0.229653584 -0.090758016

28799 0.149142754 -0.427967664 -0.071200745 0.48709879

28800 0.090932519 0.04238094 0.134730077 -0.248739274 -

28801 0.321754476 -0.073696104 0.054747624 0.05468718 0.160972767

28802 -0.339199279 -0.334658231 0.328764327 0.26834525

28803 0.112272908 -0.186809536 0.009565046 0.142167389 -

28804 0.398971508 -0.310778521 -0.379050396 -0.19750091 0.083160746

28805 -0.526674003 -0.159765389 0.094379509 0.164533263 -

28806 0.045314491 0.039002679 0.221168785 -0.327428382 0.309606177

28807 0.490959512 -0.073604942 -0.137887375 0.179225301

28808 0.151871394 0.113998452 0.204868544 0.052899261 -

28809 0.253213469 0.449690697 -0.420278135 0.251624362 0.119306711

28810 0.465973792 0.015434459 -0.275313576 0.444163698 -

28811 0.16030172 0.347210335 0.32660821 0.185130553 -0.134435326

28812 -0.439555702 0.349706415 -0.486952259 0.03399436 -

28813 0.08879551 0.138104716 0.333866828 0.070679807 -0.011860083

28814 -0.029652808 -0.182242857 0.234817559 -0.076685859

28815 0.10403546 -0.13093029 0.050300417 0.240597693 -

28816 0.270525766 -0.098998126 0.293060031 -0.164763671 -0.379435931

28817 0.265094153 -0.314830262 0.242959287 -0.293492472

28818 0.354557114 0.328134955 -0.116206791 -0.139461789

28819 0.388390452 0.199042982 -0.108157778 0.363652563 -

28820 0.117316681 0.177212345 -0.488203647 0.398782218 0.326669929

28821 0.365366636 0.239176947 0.075019384 0.34100092

28822 0.059260816 0.047619167 0.319322223 0.3330985 -0.268910474

28823 -0.427560159 0.52402477 -0.431596468 0.476432487

28824 0.080091013 0.37407039 -0.382776644 0.383936485

28825 0.201941959 0.406985647 0.343311012 -0.27825637

28826 0.19881587 0.011718007 -0.065728577 -0.09920248

28827 0.250920417 -0.174329922 0.357190085 0.236605111 -

28828 0.174315121 0.020136063 0.458292246 -0.336069341 0.246859624

28829 -0.29491051 -0.066316811 -0.484244118 -0.0583518 0.072623035

-0.436387707 -0.293153431 -0.188360386 0.330666517
0.124577939 0.282355442 0.00232847 -0.259574612 -
0.0602705 -0.398960229 -0.245266794 -0.324224088 -0.016822736
0.311066063 -0.124442601 -0.130420505 0.076098611
0.32490926 0.310997249 0.167154193 0.368014464 -
0.411305408 0.231275528 -0.12295571 -0.087933595 0.10204014
-0.157708048 0.188576591 -0.128215617 -0.325030169 -
0.132891071 0.209140944 -0.316119341 0.215032885 -0.488287243
0.143374532 -0.247096699 0.256502405 -0.27733598 -
0.280106346 0.012317682 -0.42731669 -0.145730747 0.373814412
0.261394438 0.210163888 0.027750451 -0.178513143 -
0.047654428 -0.345188904 0.067570125 -0.28689075 -0.233789537
0.345611459 -0.096974558 -0.348787862 -0.299408232
0.005383821 0.418883144 0.354232516 -0.349114328
0.031105859 0.009615966 0.400497622 -0.073658886 -
0.053893768 -0.062242235 0.534241407 0.346986451 0.000988026
0.382030352 0.449194532 -0.272621942 0.369720447
0.096567701 -0.12000137 -0.17609958 -0.254845103 -
0.360196403 -0.1201476 0.13102875 0.340379311 -0.091737723 -
0.419293163 -0.111318787 -0.002988919 -0.410457659 -0.278408839
0.129183944 -0.192398579 -0.260183257 -0.230249006 -
0.211217727 0.270903356 0.147617843 0.292364726 0.063501881

-0.216046905 0.464189612 -0.002530072 0.515808441 -
0.158040571 -0.450951705 0.153549301 0.337811393 -0.155457307
-0.046047463 0.502464971 -0.068153025 -0.403852589 -
0.244234123 0.400361777 0.148457637 -0.119358443 -0.317312964
0.213972258 0.381362711 -0.311204362 -0.421128475 -
0.424307314 -0.348330475 -0.264247701 0.005193788 0.021099699
0.302178544 0.065820452 0.108282512 0.318846633 -
0.286827609 -0.04154726 0.14668078 -0.414845724 -0.33788993
0.338161929 0.344775644 -0.214033468 -0.375653341 -
0.20132692 -0.069248478 -0.346226513 0.408475296 0.457587036
0.350030535 0.164437601 0.172585641 0.321652132 -
0.198987152 0.437083472 -0.372852465 -0.047018283 -0.368966122
0.019994624 -0.118980323 -0.089279124 -0.403047224 -
0.195648106 0.102456815 -0.203602407 0.062714197 -0.006322294
0.16713472 -0.066915717 -0.040045271 0.16905803
0.314701341 -0.034803093 0.011997248 -0.007184213 -
0.39834949 -0.392322308 -0.263939324 -0.190867191 0.07005137
0.390498209 -0.273402635 -0.344898499 -0.180790873
0.525308468 -0.236470907 0.498988626 0.169055792 -
0.468556051 0.171499728 0.220608964 0.052882915 -0.234319173
0.407431273 -0.035898316 0.305318771 -0.08284742 -
0.082675484 0.025675765 0.387319597 -0.141565514 -0.292637305

0.246126448 0.074914058 -0.28185695 0.139013372
0.281254075 -0.107033451 -0.452898758 0.023773478 -
0.248111207 -0.222024826 -0.002458456 0.143334551 0.071203215
0.312495495 0.155508049 -0.031667072 -0.507497987
0.000568983 -0.252517009 -0.456028677 -0.041411672 -
0.276738813 -0.270276398 -0.165696048 -0.286841106 -0.278115493
0.272842897 0.056749206 0.372068389 0.310145439 -
0.040275379 0.01904996 -0.348847299 -0.139292873 -0.337437751
0.317877455 0.123982601 -0.261490776 0.435836361 -
0.030445919 -0.143285555 0.0203646 -0.019120049 -0.151972471
0.193469914 -0.37291991 -0.09771197 -0.050876634
0.412798791 0.150030663 -0.188659054 0.229528399
0.216058204 -0.035292611

Table-S5: Prognostic analysis of m6A phenotype-related genes using a univariate Cox regression model

id	HR	HR.95L	HR.95H	pvalue
SERINC3	0.666509462	0.535671553	0.829304563	2.74E-04
PARVA	0.706871416	0.56323469	0.887138537	2.76E-03
ROCK2	0.747428042	0.625013676	0.893818327	1.42E-03
BMPR2	0.700349339	0.593579191	0.826324783	2.44E-05
VIRMA	0.705531677	0.563590881	0.883220372	2.34E-03
KCTD10	0.717057565	0.566485445	0.907651832	5.68E-03

28896	KCTD20	0.781461425	0.653231327	0.934863246	7.01E-03
28897	ADAM17	0.82211799	0.683675708	0.988594421	3.74E-02
28898	PHF20	0.729684138	0.568143187	0.937156254	1.36E-02
28899	SPRED1	0.799579614	0.67284795	0.950181328	1.11E-02
28900	HEG1	0.778244483	0.686171376	0.882672312	9.52E-05
28901	CDC27	0.760464398	0.606966085	0.952781572	1.73E-02
28902	RAB6A	0.615518532	0.490189042	0.772891743	2.94E-05
28903	AKAP13	0.745821457	0.625804964	0.888854638	1.05E-03
28904	METAP2	0.655372357	0.527737555	0.813875994	1.32E-04
28905	PRKD3	0.777883325	0.64702337	0.935209602	7.52E-03
28906	NUP98	0.719111624	0.575828648	0.898047587	3.63E-03
28907	SH3GLB1	0.675345475	0.550862615	0.82795873	1.59E-04
28908	BACE1	0.741803221	0.608328465	0.904563982	3.17E-03
28909	ITGB1	0.823588316	0.722090979	0.939352151	3.82E-03
28910	VCL	0.790934718	0.66608761	0.939182353	7.45E-03
28911	RAPH1	0.589189797	0.45533344	0.762396492	5.74E-05
28912	CSNK1A1	0.762387663	0.6399539	0.90824503	2.39E-03
28913	RAB3GAP1	0.687954906	0.556350517	0.850690237	5.55E-04
28914	ANKRD50	0.720684978	0.60646988	0.856409947	1.99E-04
28915	YAP1	0.816684353	0.672819612	0.991310777	4.05E-02
28916	TMEM30A	0.732274353	0.606640147	0.883927204	1.18E-03
28917	ZNFX1	0.739163491	0.603465826	0.90537466	3.49E-03

28918	TSN	0.717142638	0.563996644	0.911873447	6.67E-03
28919	IL6ST	0.752849185	0.644387893	0.879566332	3.48E-04
28920	NSD1	0.733685059	0.605709012	0.888700275	1.54E-03
28921	KLHL20	0.738784306	0.617475994	0.883924647	9.39E-04
28922	IPO9	0.77119801	0.61257427	0.970896755	2.70E-02
28923	LSM11	0.749572107	0.582133982	0.965170152	2.54E-02
28924	TRIP12	0.703905655	0.58362857	0.848970043	2.40E-04
28925	ATP11B	0.745779593	0.626687937	0.887502643	9.52E-04
28926	MAN1A2	0.697884037	0.582427568	0.83622781	9.69E-05
28927	IQGAP1	0.723120444	0.604062119	0.865644709	4.13E-04
28928	MSN	0.78107002	0.658053938	0.92708263	4.72E-03
28929	YWHAQ	0.771194143	0.624626538	0.952153599	1.57E-02
28930	KIF5B	0.740868258	0.602471802	0.911056375	4.47E-03
28931	SP3	0.758547985	0.621541004	0.925755567	6.55E-03
28932	WDFY1	0.795299997	0.65381448	0.967402994	2.19E-02
28933	ZFR	0.693393205	0.566930981	0.848064672	3.65E-04
28934	EIF2AK4	0.74710848	0.573736271	0.97287048	3.05E-02
28935	CAND1	0.707183536	0.576806454	0.867030093	8.61E-04
28936	PANK2	0.704627567	0.520795416	0.953349423	2.32E-02
28937	SLC38A2	0.77739307	0.626214084	0.965069296	2.25E-02
28938	HIPK1	0.73433091	0.613192391	0.87940081	7.88E-04
28939	ATF6	0.666592662	0.548831411	0.809621621	4.33E-05

28940	PTPN14	0.759477453	0.642015354	0.898430229	1.33E-03
28941	PAPSS1	0.67857179	0.542690379	0.848475837	6.71E-04
28942	EIF2AK2	0.789835793	0.639479639	0.975544085	2.85E-02
28943	TM9SF3	0.686002282	0.547106644	0.86015978	1.09E-03
28944	ADNP	0.713612849	0.544882011	0.934593707	1.42E-02
28945	GEMIN5	0.668405296	0.544515778	0.82048245	1.17E-04
28946	TGOLN2	0.739974198	0.631549598	0.867013162	1.95E-04
28947	FAM20B	0.713811289	0.566907873	0.898781939	4.13E-03
28948	NDST1	0.748649832	0.635576722	0.881839361	5.30E-04
28949	SRP72	0.637008956	0.517338824	0.784361026	2.16E-05
28950	SF3B3	0.740146418	0.594554122	0.921390837	7.09E-03
28951	MGAT5	0.722072466	0.611852387	0.852147768	1.17E-04
28952	STAT3	0.814663846	0.664343168	0.998997527	4.89E-02
28953	KRR1	0.654010379	0.502406743	0.851361136	1.60E-03
28954	XRCC5	0.670812742	0.532871857	0.844461437	6.76E-04
28955	NUAK1	0.804325785	0.682830816	0.947438156	9.16E-03
28956	TGFBRAP1	0.767403099	0.615191431	0.957275227	1.89E-02
28957	FEZ2	0.683912741	0.541968022	0.863033644	1.37E-03
28958	CSNK1G3	0.661588799	0.514039362	0.851490706	1.33E-03
28959	ANAPC1	0.738061112	0.575165253	0.947091644	1.70E-02
28960	NUP155	0.683278885	0.540100408	0.864413408	1.50E-03
28961	YWHAB	0.621084019	0.486420423	0.793028706	1.34E-04

28962	PELO	0.645957819	0.507799119	0.821705845	3.72E-04
28963	SHOC2	0.673385919	0.536710328	0.844866538	6.35E-04
28964	STARD13	0.793390623	0.66872917	0.941290898	7.96E-03
28965	BTBD10	0.738193242	0.577855921	0.943019259	1.51E-02
28966	SIPA1L2	0.746925872	0.637664004	0.874909443	2.99E-04
28967	TMED10	0.714562332	0.584006704	0.874303879	1.10E-03
28968	RNF103	0.683122146	0.51703919	0.902554149	7.33E-03
28969	MYO1B	0.842333688	0.724115559	0.97985195	2.62E-02
28970	QKI	0.798754667	0.661245924	0.964858905	1.97E-02
28971	WBP11	0.7248527	0.571352535	0.919592378	8.04E-03
28972	SCARB2	0.664607299	0.536885155	0.822713867	1.75E-04
28973	CNOT8	0.644696213	0.510949399	0.813452776	2.15E-04
28974	MFAP3	0.735093687	0.644964073	0.837818339	4.00E-06
28975	PGM2L1	0.766697464	0.649212523	0.905443102	1.75E-03
28976	CEP350	0.753483092	0.619552208	0.916366308	4.59E-03
28977	BZW1	0.757169705	0.596410503	0.961260673	2.23E-02
28978	TNPO1	0.765207829	0.621362529	0.942353287	1.18E-02
28979	WDR3	0.694822773	0.557539426	0.865909501	1.19E-03
28980	AZI2	0.655271593	0.526553947	0.815454643	1.52E-04
28981	ARCN1	0.697652754	0.564068509	0.862872786	9.00E-04
28982	USP9X	0.729516598	0.605846396	0.878431349	8.76E-04
28983	MSH2	0.729037296	0.596484357	0.891046634	2.02E-03

28984	RAI14	0.819174407	0.688142296	0.975156901	2.49E-02
28985	CNOT2	0.721221875	0.52305234	0.994472165	4.62E-02
28986	CUL4B	0.738460539	0.59100217	0.9227106	7.64E-03
28987	ATRN	0.696554274	0.577012862	0.840861424	1.67E-04
28988	COPA	0.759889967	0.61739254	0.935276544	9.56E-03
28989	SMARCC1	0.742973103	0.598873913	0.921744994	6.92E-03
28990	VKORC1L1	0.747182899	0.594922836	0.938411255	1.22E-02
28991	ZDHHC20	0.795685195	0.654951245	0.966659631	2.14E-02
28992	GNAI3	0.697314513	0.547850818	0.887554628	3.40E-03
28993	ADAM10	0.66983795	0.54180417	0.828127401	2.14E-04
28994	NOLC1	0.80735454	0.659999118	0.987609431	3.74E-02
28995	ACBD3	0.779935842	0.653935629	0.930213755	5.70E-03
28996	TAOK1	0.768140399	0.627422477	0.940418449	1.06E-02
28997	TP53BP2	0.783132902	0.631354733	0.971398661	2.62E-02
28998	PANK3	0.709571114	0.589025956	0.854786043	3.04E-04
28999	HS2ST1	0.73109272	0.600081633	0.890706424	1.88E-03
29000	ZMYM4	0.777882083	0.623619474	0.970304103	2.59E-02
29001	PEX13	0.712483182	0.546362661	0.92911233	1.23E-02
29002	LAMC1	0.811807318	0.698365468	0.943676558	6.63E-03
29003	MAPK14	0.792656325	0.641534741	0.979376501	3.13E-02
29004	TMEM87A	0.722391392	0.569048898	0.917055328	7.56E-03
29005	MAPRE1	0.713725687	0.561104532	0.907859994	6.01E-03

29006	TRIM24	0.638164192	0.492054392	0.827659588	7.10E-04
29007	ETF1	0.731830254	0.591950397	0.90476419	0.003918179
29008	ROBO1	0.847968287	0.729979087	0.985028514	0.030982756
29009	SLC30A1	0.78889298	0.673581544	0.923944754	0.0032706
29010	SEL1L	0.732215311	0.609371483	0.879823355	0.000879738
29011	ZFYVE16	0.691690451	0.55798356	0.857436876	0.000770058
29012	EI24	0.714809173	0.537505396	0.950599115	0.020983464
29013	SLC30A6	0.775121137	0.623927007	0.962953631	0.021394586
29014	NFATC3	0.677211649	0.560491218	0.818238723	5.38E-05
29015	MTDH	0.775647952	0.641741357	0.937495673	0.008602124
29016	DDX21	0.801099191	0.680735844	0.942744414	0.007590908
29017	CDKL5	0.69339436	0.582686894	0.825135667	3.70E-05
29018	PAFAH1B2	0.690827721	0.517194517	0.922753287	0.012269587
29019	PAK2	0.783558738	0.623569568	0.984596312	0.036332706
29020	ACTR2	0.80105856	0.669400723	0.95861088	0.015461712
29021	RNF2	0.766788771	0.610087078	0.963739505	0.022809147
29022	ISG20L2	0.76060265	0.603703958	0.95827828	0.020259109
29023	ZNF200	0.665409704	0.460935762	0.960589545	0.029659288
29024	RIOK2	0.6521929	0.505168936	0.842006601	0.001040232
29025	CDC73	0.676643167	0.567429692	0.806877014	1.37E-05
29026	STK38L	0.772777657	0.632605486	0.944009054	0.011595614
29027	THADA	0.659783574	0.484214725	0.899011001	0.008428736

29028	WDR44	0.733588682	0.596430204	0.902288904	0.003350806
29029	MCFD2	0.726817201	0.61223641	0.862841926	0.000267014
29030	SERINC5	0.79167674	0.660923844	0.948296943	0.011200812
29031	BCL10	0.634520065	0.510978289	0.787931156	3.83E-05
29032	EIF4G2	0.688281438	0.569380706	0.832011576	0.000113104
29033	HAT1	0.70587646	0.562149019	0.886351412	0.002712861
29034	ASCC1	0.633981569	0.504225305	0.797129033	9.60E-05
29035	CHML	0.827101052	0.700571769	0.976482612	0.025032531
29036	SSRP1	0.766296794	0.598765339	0.980702686	0.034449166
29037	NRP1	0.793492911	0.698793756	0.901025509	0.00036072
29038	MYO1E	0.765403754	0.6336853	0.924501336	0.005525716
29039	TTC27	0.694995226	0.5481424	0.881191393	0.00266181
29040	BCL2L11	0.781169509	0.634697825	0.961443032	0.019747614
29041	CAP1	0.668177091	0.532125731	0.83901341	0.000518465
29042	AGGF1	0.616259113	0.496655438	0.764665531	1.10E-05
29043	CCDC117	0.654826992	0.514508855	0.833413042	0.000579639
29044	RRM1	0.78126796	0.634209964	0.96242516	0.020345191
29045	KALRN	0.653399606	0.532343198	0.801984599	4.69E-05
29046	RECQL	0.794446919	0.685057286	0.921303837	0.002331614
29047	WDR36	0.75485459	0.589983076	0.965799656	0.025303803
29048	PDLIM5	0.63942555	0.517557042	0.789990285	3.40E-05
29049	SETD7	0.799227856	0.676929863	0.943620899	0.008173664

29050	SEC63	0.757305396	0.617205966	0.929205962	0.007734132
29051	MIA3	0.795170325	0.633124286	0.998691503	0.048698137
29052	ATP2B4	0.842811508	0.713100382	0.996116753	0.044899519
29053	KPNA3	0.792794731	0.633989402	0.991378538	0.041761799
29054	SLC4A1AP	0.739022747	0.565247622	0.966221882	0.027021908
29055	USP32	0.756737273	0.61978991	0.923944212	0.00620815
29056	DCTN5	0.652643105	0.491022533	0.867461255	0.00328896
29057	AP3M1	0.747296965	0.604273925	0.924171524	0.007198514
29058	TOP1	0.802262999	0.652500294	0.986399433	0.036630463
29059	YIPF5	0.701013428	0.587562422	0.836370415	8.02E-05
29060	RAB23	0.823832103	0.682418173	0.994550497	0.043712816
29061	UBE2G1	0.727875915	0.582386172	0.909711414	0.005243725
29062	REEP3	0.702710542	0.576570507	0.856447044	0.000473895
29063	ATF2	0.712609039	0.570774754	0.889688339	0.002769917
29064	LMBRD2	0.669067383	0.559592269	0.799959519	1.04E-05
29065	DNAJC3	0.68753242	0.572570101	0.825577214	5.99E-05
29066	CCDC50	0.792984209	0.643440845	0.977283243	0.029593999
29067	SENP2	0.738304145	0.571904259	0.95311934	0.019887712
29068	GNA13	0.745013712	0.623309989	0.890480564	0.001218092
29069	MAP4	0.782084721	0.615697854	0.993436157	0.044017643
29070	TMTC3	0.67849556	0.544610881	0.845293843	0.00054296
29071	ANKH	0.797006296	0.666002283	0.953779067	0.013268238

29072	PIGN	0.716137406	0.574788746	0.892245696	0.002917426
29073	GSPT1	0.658124863	0.522426749	0.829069981	0.000383703
29074	NRAS	0.714157834	0.596262137	0.855364413	0.0002551
29075	NUP205	0.752653711	0.61262333	0.924691537	0.006821838
29076	ACVR1	0.771363503	0.62299527	0.955066086	0.017229152
29077	YTHDF3	0.716738803	0.582636528	0.881706669	0.0016263
29078	TOR1B	0.674920936	0.506666663	0.899049224	0.007201982
29079	NR3C1	0.788869618	0.664388361	0.936673955	0.006797855
29080	ASCC3	0.799185051	0.652474183	0.978884318	0.030295836
29081	DCTN4	0.65961503	0.514914217	0.844979557	0.000991138
29082	GGCX	0.773652346	0.617471211	0.969337422	0.025703979
29083	EGLN1	0.661300062	0.553555502	0.790016125	5.18E-06
29084	GOSR2	0.737849716	0.548234103	0.99304695	0.044855928
29085	MAP2K1	0.742944401	0.583580546	0.94582725	0.015861343
29086	MAP3K2	0.780019505	0.620344468	0.980794477	0.033510988
29087	SPARC	0.813599151	0.701745394	0.943281685	0.006261474
29088	MIER1	0.704162329	0.567645882	0.873510407	0.001423419
29089	OS9	0.679057268	0.543440197	0.848517971	0.000661444
29090	PPP3CA	0.643807434	0.512465963	0.808810813	0.000155136
29091	COL4A1	0.840139649	0.742802843	0.95023146	0.005562487
29092	PIGK	0.654184797	0.525223186	0.814811227	0.000151802
29093	ARHGAP21	0.752279094	0.600205639	0.942883236	0.013496761

29094	COL15A1	0.800820584	0.715531628	0.896275697	0.000110678
29095	GOLPH3	0.662695966	0.514688781	0.853265041	0.001420381
29096	TAF2	0.780485889	0.637303053	0.955837603	0.016540723
29097	BCAP29	0.655042117	0.510251962	0.840918228	0.000902048
29098	NID1	0.867839997	0.764371063	0.985314982	0.028643537
29099	SEC23IP	0.740894802	0.591130472	0.928602285	0.009244512
29100	BRAP	0.685862987	0.493794514	0.952639253	0.024487286
29101	ADIPOR1	0.612048963	0.461435174	0.811823534	0.000658066
29102	EAF1	0.74023254	0.576532267	0.950413783	0.018334687
29103	RNGTT	0.723230496	0.570624865	0.916648367	0.007368467
29104	NEDD4	0.84228379	0.716353895	0.990351261	0.03777314
29105	CREB3L2	0.746983655	0.622099852	0.896937331	0.00177643
29106	DCP2	0.787674918	0.622727463	0.996313498	0.046506145
29107	ARF3	0.675272947	0.515142673	0.885179149	0.004467378
29108	YPEL5	0.589336719	0.448674894	0.77409673	0.000144505
29109	UBLCP1	0.704233162	0.543342483	0.912765636	0.008056205
29110	GFPT1	0.727542104	0.590187588	0.896863174	0.002885979
29111	ARF6	0.76221355	0.608393445	0.954923989	0.018224651
29112	SLFN5	0.851980345	0.731949643	0.991694599	0.038676694
29113	EVI5	0.73715879	0.578855194	0.938754782	0.013422519
29114	TMCO3	0.716305667	0.597467963	0.858780453	0.000312359
29115	NID2	0.879970091	0.781104577	0.991349153	0.035478797

29116	RNF4	0.745979923	0.569678299	0.976842626	0.033148617
29117	PWP1	0.63898032	0.481018136	0.848815915	0.001992834
29118	NRIP1	0.810692909	0.697437418	0.942339736	0.006266248
29119	RB1CC1	0.754170654	0.602842175	0.943486369	0.013547037
29120	IDS	0.731477091	0.592575023	0.902938386	0.003611673
29121	STAU1	0.672962771	0.529128324	0.855896142	0.001245287
29122	SNAP23	0.751801964	0.581127055	0.972603476	0.029900966
29123	CLTC	0.692772803	0.571896019	0.839198283	0.000175487
29124	IPO7	0.762902604	0.632385749	0.920356577	0.004699979
29125	RAB10	0.679790852	0.533904799	0.865539332	0.001738706
29126	AZIN1	0.750668315	0.606316568	0.929387301	0.008487626
29127	RHBDD1	0.652246828	0.514529144	0.826825708	0.000413296
29128	DOCK7	0.747592522	0.619451947	0.90224041	0.002426719
29129	SLC30A5	0.675052511	0.535684255	0.85068002	0.000866451
29130	CLCC1	0.756246948	0.619173564	0.923665801	0.006177894
29131	RAD1	0.633347392	0.47486905	0.844714809	0.001880381
29132	AP3B1	0.579250399	0.464617306	0.722166437	1.22E-06
29133	KPNA4	0.717065352	0.543461165	0.946125965	0.018697029
29134	FBXO28	0.680750176	0.557170631	0.831739464	0.000168206
29135	RAB8B	0.756919592	0.622597949	0.920220296	0.00520426
29136	ATP2C1	0.741317921	0.600412969	0.915290456	0.005387602
29137	GUCY1A2	0.825927227	0.704820065	0.967843876	0.018080411

29138	RNF139	0.65849606	0.514951958	0.842053428	0.000867575
29139	ZRANB3	0.656708109	0.442535786	0.974532578	0.036792424
29140	PCDHGC3	0.822987493	0.729953155	0.927879288	0.001457818
29141	PLAA	0.665642436	0.522782859	0.847540895	0.000960093
29142	ZNF436	0.794508991	0.650429897	0.970503568	0.024244056
29143	PSMD10	0.688963432	0.526908747	0.900859236	0.006468026
29144	PTPN11	0.751814721	0.623300051	0.906827094	0.002858809
29145	FRMD4A	0.777591965	0.645327571	0.93696487	0.008183153
29146	CAST	0.764528323	0.614052375	0.951878994	0.016351461
29147	AHCYL1	0.674030426	0.531336748	0.85504535	0.001153043
29148	PPP1R3D	0.588403399	0.45985443	0.752887298	2.48E-05
29149	EIF2S3	0.763101365	0.620491997	0.938487034	0.010424144
29150	EXOC4	0.691400623	0.550670934	0.868095248	0.001482019
29151	GCLM	0.709049628	0.589009816	0.853553475	0.000279938
29152	COPB1	0.730407122	0.592691527	0.900121799	0.003207832
29153	DDX23	0.709306571	0.551467322	0.912322073	0.007484242
29154	RAP1B	0.7372667	0.560131364	0.970419123	0.02969426
29155	STT3A	0.775762202	0.611560143	0.984052019	0.036398323
29156	CCT8	0.716625994	0.542596288	0.946473145	0.018897173
29157	CD99L2	0.777829083	0.608864609	0.993682459	0.044359041
29158	STAMBP	0.730903864	0.56186378	0.950800671	0.019496019
29159	SSBP2	0.801432829	0.647830346	0.991454914	0.041448455

29160	MTPN	0.731904069	0.604664402	0.885918807	0.001359532
29161	SGTB	0.664866656	0.533893316	0.82797004	0.000265889
29162	SCFD2	0.512785497	0.387027179	0.679406976	3.28E-06
29163	ATF1	0.723569542	0.590323919	0.88689085	0.001833879
29164	DNAJC14	0.698894405	0.569236874	0.858084589	0.000622031
29165	PAM	0.754004575	0.645179948	0.881185501	0.00038454
29166	GDI2	0.670095874	0.549449059	0.817234051	7.73E-05
29167	TEX2	0.718917067	0.601900904	0.858682461	0.000271753
29168	XRN2	0.744269054	0.614073074	0.902069229	0.002608377
29169	UXS1	0.703698454	0.549071975	0.901869949	0.005505977
29170	SLC35E1	0.731560139	0.587335104	0.911200835	0.005270928
29171	ZNF217	0.786028709	0.633198688	0.975746069	0.029069753
29172	MBTPS2	0.751663769	0.615968338	0.917252375	0.004950243
29173	DAAM2	0.86843266	0.755671105	0.998020541	0.046824289
29174	F2R	0.873936868	0.774376881	0.986297071	0.028995118
29175	ZFP64	0.627009013	0.478326662	0.821907566	0.000724414
29176	HEPH	0.749804399	0.646959112	0.868998715	0.000130563
29177	CDV3	0.822082994	0.67872051	0.995727166	0.045095871
29178	CCT4	0.693350536	0.562518336	0.854612081	0.00059804
29179	LRP4	0.793517851	0.669069407	0.94111399	0.007877634
29180	VCPIP1	0.684992914	0.531185768	0.883335588	0.003544749
29181	CCT7	0.626943899	0.457447902	0.859242441	0.003692498

29182	ABI1	0.696649055	0.553386778	0.87699946	0.002088701
29183	SMAD1	0.718621218	0.600062653	0.860604227	0.00032833
29184	DAG1	0.785333784	0.619836327	0.995019371	0.045358278
29185	C5orf22	0.738003699	0.568561759	0.957942476	0.022440648
29186	INSIG2	0.818417302	0.682236145	0.98178158	0.030930029
29187	H6PD	0.797828757	0.675121282	0.942839076	0.008031266
29188	IFT52	0.678813707	0.529252224	0.870639796	0.002281752
29189	ZBTB41	0.795620284	0.655571243	0.965587865	0.0206429
29190	LRP10	0.752008155	0.604555804	0.935424425	0.010484138
29191	TCP11L1	0.711642523	0.586346999	0.863712241	0.000575797
29192	SNX6	0.74966189	0.617468305	0.910156756	0.003602019
29193	PPAT	0.792077704	0.63299617	0.991138839	0.04157239
29194	PRPS1	0.755736156	0.614093615	0.930048976	0.008174097
29195	CANX	0.667115077	0.560560908	0.793923586	5.14E-06
29196	CTBS	0.683558311	0.548487941	0.851891044	0.000706398
29197	PCDH1	0.684184971	0.581306215	0.805271064	5.00E-06
29198	ARL13B	0.796791991	0.639933028	0.992099874	0.042267853
29199	ECM2	0.870114269	0.757624561	0.999306094	0.048862259
29200	NEK7	0.80758773	0.677194669	0.963087826	0.0173794
29201	CD302	0.761851979	0.652576027	0.889426541	0.000574546
29202	CSNK2A1	0.768363285	0.593036549	0.995524034	0.046161814
29203	GNPTAB	0.720223786	0.567604358	0.913880056	0.006909813

29204	CALD1	0.852879341	0.747119789	0.973609829	0.018478059
29205	MYO1D	0.677766211	0.57008754	0.80578333	1.05E-05
29206	PAIP1	0.704205189	0.556070312	0.891802597	0.003611271
29207	MALL	0.745245726	0.644012336	0.862392163	7.90E-05
29208	RAB1A	0.766203867	0.596517489	0.98415952	0.037071543
29209	PIK3R1	0.765947514	0.659708346	0.889295395	0.000465227
29210	RPS6KA3	0.76317344	0.642729108	0.906188459	0.002042158
29211	LRRC42	0.758579059	0.612198466	0.939960192	0.011536363
29212	PRIM1	0.729040782	0.586625356	0.906030494	0.004373793
29213	RXRA	0.727288288	0.589095796	0.897898538	0.003060182
29214	CCDC6	0.70035785	0.561020358	0.874301815	0.001650668
29215	BFAR	0.578518095	0.436344955	0.76701514	0.000142803
29216	DYNLT3	0.691898882	0.553360331	0.865121761	0.001233941
29217	RASSF3	0.804393336	0.664712654	0.973426088	0.025304027
29218	MAN2B2	0.734207058	0.571289164	0.943585206	0.015796292
29219	COL4A2	0.859406477	0.753320449	0.980432025	0.02419946
29220	TRIM5	0.748724739	0.596439989	0.939891263	0.012621185
29221	ARFGEF1	0.691965289	0.561760489	0.852348948	0.000536104
29222	PIGS	0.768905882	0.632820153	0.934256366	0.008187296
29223	SNX7	0.708320133	0.606039143	0.827863045	1.46E-05
29224	STS	0.733644385	0.619399858	0.868960619	0.000335432
29225	PPP1CC	0.700124865	0.53360567	0.91860873	0.010093968

29226	SMARCAL1	0.730532651	0.56013005	0.952775081	0.020506705
29227	CAPN2	0.719908793	0.565856353	0.915901477	0.007472323
29228	COG5	0.714661737	0.575618788	0.88729105	0.002340628
29229	ARFGAP3	0.77613765	0.61995618	0.971664887	0.027055955
29230	PCDHAC2	0.516455926	0.366303593	0.72815754	0.00016329
29231	ITGAV	0.755893401	0.656578118	0.870231306	9.86E-05
29232	TFIP11	0.73589749	0.593330022	0.91272158	0.005251745
29233	IDE	0.741105435	0.578657471	0.949157824	0.017630197
29234	CASK	0.789275855	0.638767045	0.975248145	0.028369283
29235	RAB43	0.644245457	0.459194306	0.903870547	0.010928241
29236	ATP2B1	0.786590494	0.64015571	0.966522045	0.022374715
29237	TMED7	0.74363471	0.624371019	0.885679452	0.000896517
29238	OSBPL10	0.767378755	0.618595868	0.951946472	0.016048976
29239	NUDCD3	0.652499979	0.480403564	0.886247011	0.006276488
29240	P2RY1	0.761083344	0.645906057	0.896798924	0.001110179
29241	COPS8	0.675317597	0.508438996	0.896968684	0.006712132
29242	MYO5A	0.710220094	0.577667494	0.873188448	0.001168123
29243	TBC1D23	0.744700964	0.599512008	0.925051574	0.007720641
29244	CTNNBL1	0.709955342	0.515254417	0.978228561	0.036210396
29245	PDE3A	0.831156598	0.721009225	0.958131002	0.01078405
29246	VPS37A	0.767631498	0.601721354	0.97928736	0.033302028
29247	NBN	0.787869694	0.653984758	0.949163795	0.012107029

29248	NIPA2	0.790817379	0.654497966	0.955529519	0.015048722
29249	RABIF	0.696669915	0.508099918	0.955223478	0.024804986
29250	KLHL2	0.689272318	0.546724438	0.868986817	0.001644533
29251	ZMAT3	0.753804348	0.617209033	0.920629746	0.005593823
29252	SLC30A7	0.77303466	0.611800714	0.976760196	0.031006773
29253	CBX5	0.80936076	0.656340146	0.998056943	0.047911323
29254	RP2	0.75112287	0.623934952	0.904237796	0.002499352
29255	PRKAB2	0.754360829	0.621089829	0.916228592	0.004482238
29256	TSPAN9	0.778101215	0.632516113	0.95719538	0.017602056
29257	BTF3	0.674609185	0.524413141	0.867822558	0.002189659
29258	RPE	0.702969562	0.543225793	0.909688404	0.007370774
29259	TGFBR1	0.744741606	0.601378682	0.922280881	0.006900601
29260	SPCS3	0.668436817	0.518255646	0.862137792	0.001919023
29261	PGK1	0.715368521	0.615950849	0.830832723	1.15E-05
29262	VBP1	0.716287222	0.558606988	0.918476488	0.008530523
29263	PTGES3	0.712912981	0.556888616	0.91265094	0.007247308
29264	MOCS3	0.554450435	0.40647389	0.756297742	0.000196595
29265	M6PR	0.76512963	0.657344792	0.89058795	0.000548862
29266	RAB30	0.68337669	0.521251759	0.895927337	0.005863521
29267	NDRG3	0.74686511	0.560798483	0.994666552	0.045874617
29268	RHOU	0.688956892	0.588278809	0.806865031	3.79E-06
29269	LMAN1	0.830221242	0.692752159	0.99496956	0.043949962

29270	UNC5C	0.783311447	0.624284544	0.982848012	0.034910722
29271	SCAMP1	0.638690741	0.52700964	0.774038711	4.83E-06
29272	STRAP	0.76787341	0.607428871	0.970697315	0.027198695
29273	ATG7	0.620600149	0.457774978	0.841340317	0.002121652
29274	FBXL7	0.845669062	0.7165202	0.998096302	0.047423288
29275	PSME3	0.759588207	0.584823084	0.986579122	0.039279345
29276	RRM2B	0.734270798	0.610169239	0.883613217	0.001075973
29277	POT1	0.701562456	0.562621654	0.874815031	0.001645739
29278	GNPNAT1	0.742102512	0.615394524	0.894899314	0.001793614
29279	TXNDC9	0.75337426	0.586743124	0.967327527	0.026390581
29280	NR5A2	0.754918434	0.616411139	0.924548254	0.006557144
29281	CLNS1A	0.720316499	0.52008114	0.997644058	0.048363803
29282	IFNGR1	0.781835363	0.629380662	0.971219124	0.0261591
29283	SUMO3	0.771449673	0.60558454	0.982744041	0.035650159
29284	ZNF622	0.581629767	0.454622929	0.744118177	1.62E-05
29285	ARL10	0.843124943	0.715670904	0.993277307	0.041287624
29286	MLH1	0.682290191	0.511381186	0.910318796	0.009359073
29287	RBM3	0.627703096	0.491724403	0.801284569	0.000185178
29288	HFE	0.761057263	0.608255811	0.952244347	0.016944528
29289	NXT2	0.779715774	0.611754496	0.993791942	0.044402038
29290	NSUN3	0.653280703	0.49304829	0.865585959	0.003023295
29291	RAB3D	0.806472594	0.679056347	0.957796872	0.014229577

29292	ARL2BP	0.735738967	0.634919734	0.852567339	4.48E-05
29293	MAP1LC3B	0.590636047	0.445726372	0.782657167	0.000246136
29294	STT3B	0.726320114	0.574415191	0.918396512	0.00756183
29295	MAN2A1	0.70861863	0.588268871	0.853589893	0.000286853
29296	GOLPH3L	0.643965651	0.521161977	0.795706093	4.56E-05
29297	PALLD	0.819505037	0.711572274	0.943809266	0.005734806
29298	XRCC4	0.75596402	0.604284216	0.945716575	0.014348267
29299	MINPP1	0.77109922	0.625968032	0.949879189	0.014553995
29300	APLP2	0.665903419	0.535153418	0.828598582	0.000266549
29301	ST3GAL1	0.745148014	0.60598929	0.916262997	0.005285238
29302	AHR	0.776589333	0.664999764	0.90690407	0.001400184
29303	MANBA	0.713008653	0.559443757	0.90872645	0.006268957
29304	FAM3C	0.756883109	0.611718095	0.936496803	0.01035158
29305	C12orf29	0.729750447	0.54957408	0.968997144	0.029432232
29306	EGFR	0.865190515	0.764818282	0.97873527	0.021357746
29307	GALNT2	1.269240035	1.077977125	1.494438266	0.004223211
29308	LAMP2	0.676186504	0.552574924	0.827450121	0.000145382
29309	CDADC1	0.493955448	0.389808886	0.625927201	5.28E-09
29310	SCCPDH	0.708694948	0.568469753	0.88350968	0.002206182
29311	NSF	0.771984221	0.596695493	0.998766783	0.048912416
29312	CFH	1.124141828	1.011139834	1.249772589	0.030394121
29313	SLC33A1	0.673083255	0.537239934	0.843275116	0.000577311

29314	ARMC1	0.747727543	0.572512975	0.976565601	0.032839982
29315	MRPS27	0.639079195	0.490844988	0.832079837	0.000883452
29316	MFSD1	0.679926353	0.561966622	0.822646447	7.24E-05
29317	PHLDA1	0.832879045	0.719544603	0.964064633	0.014271751
29318	ITPR1	0.79575014	0.676872026	0.935506656	0.005648463
29319	HSPA8	0.73314289	0.635485239	0.845807998	2.08E-05
29320	FZD7	0.841030377	0.708711897	0.998053083	0.047448186
29321	KCTD12	0.773044895	0.666699829	0.896353027	0.000651889
29322	PPP1R13B	0.634541967	0.523660788	0.768901391	3.45E-06
29323	PGRMC2	0.518145092	0.402136442	0.667620013	3.69E-07
29324	ARSB	0.785310825	0.676763075	0.911268824	0.00145157
29325	SLC35D1	0.79589077	0.672254468	0.942265389	0.008040655
29326	MOSPD1	0.689686107	0.511175406	0.930535626	0.015054308
29327	WDR31	0.60922679	0.506197591	0.733226091	1.58E-07
29328	CYB5R4	0.675134367	0.527173619	0.864622957	0.001855523
29329	DTX3L	0.779847794	0.626268901	0.971088586	0.02627371
29330	ATP1A1	0.611341329	0.502845652	0.743246399	7.94E-07
29331	SCN4A	0.659447158	0.558334355	0.778871209	9.45E-07
29332	RNF130	0.610872265	0.450194706	0.82889674	0.001550481
29333	STXBP1	0.767126833	0.67462011	0.872318463	5.27E-05
29334	TMED5	0.672555424	0.567511869	0.797042005	4.69E-06
29335	EMCN	0.697727934	0.632351226	0.769863724	7.48E-13

29336	IL17RD	0.677719019	0.589355064	0.779331675	4.82E-08
29337	TGFA	0.858350025	0.787455095	0.935627657	0.000515137
29338	INTS8	1.454748578	1.046137144	2.022959837	0.025875663
29339	RPS23	0.72332971	0.555575081	0.941737466	0.016134542
29340	FRMD3	0.711035598	0.634789395	0.79643993	3.80E-09
29341	RUNX1	1.363758887	1.16002473	1.6032747	0.000171229
29342	SETD3	0.510249987	0.412239401	0.631562748	6.30E-10
29343	SECISBP2	0.71332785	0.580577567	0.876431764	0.001302723
29344	SORBS2	0.608408185	0.539112218	0.686611259	8.00E-16
29345	VPS29	0.610381587	0.422136619	0.882571341	0.008692913
29346	F3	1.200948438	1.091256693	1.321666259	0.000178975
29347	CLCN6	0.793471567	0.651515847	0.966357351	0.021434848
29348	FANCC	0.646710865	0.538960854	0.77600245	2.77E-06
29349	AFG3L2	0.608470519	0.509174284	0.727130933	4.62E-08
29350	TMEM25	0.617478589	0.526553591	0.724104468	2.99E-09
29351	HMBOX1	0.72459178	0.570647111	0.920066426	0.008202388
29352	PPP2R2D	0.641297725	0.451704284	0.910469052	0.012972991
29353	PLCL2	0.596974672	0.508115828	0.701373071	3.52E-10
29354	MCF2L	0.704207944	0.601318065	0.824703026	1.35E-05
29355	SEMA4B	1.292940522	1.092934679	1.529547215	0.002731921
29356	PER3	0.65786966	0.564955639	0.766064554	7.03E-08
29357	BCL2	0.664893261	0.585273692	0.755344131	3.57E-10

29358	PLOD2	1.253246269	1.087424919	1.444353705	0.001824515
29359	GARNL3	0.549811632	0.41723298	0.724518063	2.15E-05
29360	STK32B	0.717325356	0.643982649	0.79902101	1.57E-09
29361	MRPL50	0.583910291	0.471212763	0.723561106	8.77E-07
29362	SERPINE1	1.116117953	1.022037807	1.218858322	0.014479327
29363	ARL4C	1.183325773	1.05413077	1.328355006	0.004321862
29364	NR3C2	0.598806155	0.516496324	0.694233036	1.07E-11
29365	C1orf21	1.198695489	1.008225062	1.425148937	0.040097408
29366	FAM107A	0.683179506	0.60074844	0.776921264	6.34E-09
29367	CRY2	0.598448888	0.508768432	0.703937292	5.71E-10
29368	HABP4	0.618392938	0.515463151	0.741876166	2.29E-07
29369	ECM1	1.236090676	1.078167141	1.417145914	0.002372665
29370	DDAH1	0.647999976	0.569182479	0.737731719	5.49E-11
29371	ALDH6A1	0.647705415	0.569331227	0.736868602	4.11E-11
29372	CYB5D2	0.446697076	0.346434727	0.575976546	5.17E-10
29373	MRPS31	0.603148364	0.45159268	0.805566532	0.000616311
29374	LMO7	0.655969043	0.556817955	0.772775702	4.59E-07
29375	NOSTRIN	0.701986018	0.609358608	0.808693538	9.54E-07
29376	ENPP5	0.668083375	0.590387966	0.756003547	1.61E-10
29377	CABLES1	0.709786172	0.617604209	0.815726969	1.37E-06
29378	CBX7	0.653628789	0.555226604	0.769470682	3.26E-07
29379	PCCA	0.604042158	0.517785338	0.704668329	1.43E-10

29380	MMP19	1.24874848	1.091286646	1.428930494	0.001236736
29381	ALDH3A2	0.636088652	0.552198073	0.732723986	3.62E-10
29382	COL4A4	0.625868346	0.538540249	0.72735731	9.85E-10
29383	CD44	1.302853296	1.120921116	1.514314154	0.00056584
29384	NTN4	0.720199154	0.649415333	0.798698144	5.03E-10
29385	SH3YL1	0.613879485	0.516692722	0.729346488	2.87E-08
29386	CBR4	0.55416961	0.431395171	0.711885478	3.85E-06
29387	FRAS1	0.749196139	0.661418133	0.848623325	5.58E-06
29388	AGTR1	0.78018357	0.707975437	0.859756386	5.46E-07
29389	ARHGAP24	0.607844332	0.523837543	0.705323123	5.37E-11
29390	TSPAN7	0.692651897	0.622062071	0.771252055	2.14E-11
29391	ZNF684	0.544145675	0.418755367	0.707082317	5.27E-06
29392	CIRBP	0.657420759	0.53323807	0.810523627	8.61E-05
29393	ZNF711	0.859642973	0.767120015	0.9633252	0.009239413
29394	TMEM38B	0.686018406	0.603726914	0.779526707	7.46E-09
29395	HLF	0.679267901	0.603490009	0.764560928	1.47E-10
29396	RAB3IP	0.696283505	0.611121556	0.793313072	5.38E-08
29397	BPHL	0.577496206	0.47268356	0.705549962	7.73E-08
29398	MAPK10	0.587466643	0.46844588	0.7367277	4.12E-06
29399	CX3CL1	0.661225778	0.579885926	0.753975066	6.55E-10
29400	CYP1B1	1.11624772	1.012178308	1.231017266	0.027638416
29401	NPR3	0.778782331	0.724508234	0.83712219	1.17E-11

29402	P4HA3	1.291646688	1.142832064	1.459839306	4.17E-05
29403	IL2RA	1.244424961	1.094893528	1.41437815	0.000814124
29404	POMT1	0.666920516	0.514298661	0.864834014	0.002248931
29405	TMEM88	0.71928694	0.636163815	0.813271187	1.45E-07
29406	ZNF577	0.803953238	0.67419553	0.958684505	0.015108042
29407	ZNF433	0.646500933	0.53808242	0.776764751	3.20E-06
29408	KL	0.763401763	0.703715837	0.828149973	8.05E-11
29409	TMEM45A	1.167111866	1.058402241	1.286987172	0.001949615
29410	ERBB2	0.66637467	0.563665375	0.787799323	2.01E-06
29411	IRF6	0.710723906	0.639329083	0.790091494	2.58E-10
29412	NDRG2	0.658721561	0.566191184	0.76637381	6.47E-08
29413	MLLT11	1.516413302	1.317674752	1.745126634	6.29E-09
29414	SIRT5	0.597843045	0.455916722	0.783950861	0.000199016
29415	TCTA	0.535689856	0.42535458	0.674645661	1.13E-07
29416	AJAP1	0.556472778	0.464996675	0.665944445	1.59E-10
29417	MGAM	0.765176746	0.704032539	0.83163124	3.00E-10
29418	RNF152	0.663711455	0.582380265	0.756400794	7.96E-10
29419	HIBCH	0.544998124	0.449804724	0.660337564	5.75E-10
29420	DNASE1L3	0.65343893	0.567063997	0.752970453	4.05E-09
29421	FNDC4	1.158134403	1.036410732	1.294154193	0.009564587
29422	ADCY5	0.725264984	0.654817499	0.803291448	7.21E-10
29423	MAP7	0.709439849	0.639891167	0.78654766	6.99E-11

29424	LEF1	1.166683665	1.034108774	1.316254932	0.012247434
29425	BTNL9	0.828491859	0.752780585	0.91181783	0.000119098
29426	PAQR5	0.628210799	0.553297078	0.713267471	7.21E-13
29427	CCL13	1.239148454	1.086620046	1.413087211	0.001376586
29428	DGKG	1.38052787	1.096453324	1.738201853	0.006082038
29429	PLEKHG3	0.737469649	0.607369084	0.895438206	0.002103315
29430	SLC25A23	0.531686727	0.436200916	0.648074697	3.98E-10
29431	RCC1	1.393514084	1.111808659	1.74659685	0.003979465
29432	FKBP10	1.355622984	1.189692386	1.544696508	4.94E-06
29433	SOX6	0.651189458	0.553746411	0.765779609	2.14E-07
29434	CCNB1	1.438402068	1.196007014	1.729923391	0.00011296
29435	TGFBI	1.097950292	1.028355031	1.172255504	0.005160748
29436	TPPP	0.789395255	0.699239427	0.891175247	0.000132377
29437	CA4	0.780619792	0.707825245	0.860900716	7.09E-07
29438	SRPX2	1.254245483	1.146783814	1.371777062	7.16E-07
29439	ZNF540	0.670368491	0.521105563	0.862385561	0.001858014
29440	CAMTA1	0.483639272	0.337917318	0.692201711	7.16E-05
29441	ETFDH	0.519135619	0.421892162	0.638793074	5.83E-10
29442	CETP	0.716450832	0.607073503	0.845534836	7.98E-05
29443	SLC44A3	0.657690496	0.56881259	0.760455721	1.54E-08
29444	KCTD17	1.563383142	1.283226925	1.904703525	9.20E-06
29445	PANK1	0.633620414	0.553507172	0.725329045	3.69E-11

29446	ADAM12	1.274142108	1.144520732	1.418443604	9.60E-06
29447	ACAA2	0.644964094	0.561652402	0.740633676	5.14E-10
29448	ELOVL3	1.668995204	1.159518818	2.402328404	0.005843686
29449	PCSK1	1.231218835	1.06210451	1.427260506	0.005793892
29450	CCNB1IP1	0.692647542	0.565814848	0.84791097	0.000372675
29451	MAPT	0.799346874	0.726180618	0.879884989	4.82E-06
29452	CGN	0.673438024	0.590612819	0.767878307	3.54E-09
29453	TRPM3	0.676619729	0.583969529	0.783969427	2.00E-07
29454	TUB	0.833127878	0.737286361	0.94142805	0.003412057
29455	IL1R2	1.138756903	1.052940779	1.23156716	0.00115229
29456	DMRTA1	0.594323191	0.509983782	0.69261037	2.67E-11
29457	TMEM132A	1.43633814	1.230111321	1.677138661	4.67E-06
29458	CLEC2B	1.421493556	1.199812174	1.684133546	4.79E-05
29459	GIPC2	0.765641658	0.702824389	0.83407343	9.72E-10
29460	C1QTNF1	1.221556419	1.075247523	1.387773563	0.002107987
29461	ANK3	0.635553437	0.559278433	0.72223091	3.69E-12
29462	ROR2	1.193112627	1.069767848	1.330679123	0.001517701
29463	VPS37D	0.604201786	0.511529237	0.713663603	3.01E-09
29464	LIN7A	0.753587385	0.68381088	0.830483931	1.15E-08
29465	RHBDL2	1.823540569	1.379025825	2.411340053	2.50E-05
29466	MYEF2	0.78033747	0.636600802	0.956528118	0.01694722
29467	CDC42BPG	0.586626541	0.493716051	0.697021492	1.34E-09

29468	DNTTIP1	1.66976503	1.350687831	2.06421883	2.16E-06
29469	PKHD1	0.753590488	0.684170881	0.830053777	9.60E-09
29470	EMX2OS	0.726091806	0.662217436	0.796127197	9.57E-12
29471	C4orf19	0.599647904	0.521987518	0.688862465	4.94E-13
29472	HM13	1.526311712	1.127752248	2.065726266	0.006169967
29473	AQP1	0.77649271	0.717970162	0.839785496	2.49E-10
29474	KMO	1.113248615	1.003184192	1.235388764	0.043402283
29475	IGFBP1	1.121325122	1.068030216	1.177279455	4.05E-06
29476	TAL2	0.731956097	0.653454486	0.819888362	7.01E-08
29477	SEMA3C	1.127458579	1.021503957	1.244403253	0.017195479
29478	IMPA2	0.650481431	0.575291964	0.735498005	6.80E-12
29479	ADA	1.311921116	1.138011431	1.512407493	0.00018274
29480	PTPN3	0.707994417	0.606396642	0.826614233	1.25E-05
29481	PPARG	0.564289742	0.459909609	0.692359773	4.18E-08
29482	GAS8	0.677473722	0.536293226	0.855820329	0.001091622
29483	RRM2	1.449195792	1.245362169	1.686391716	1.61E-06
29484	PDGFRL	1.316908585	1.187242853	1.46073587	1.94E-07
29485	SLC6A19	0.826223881	0.771591078	0.884724982	4.53E-08
29486	CLCN5	0.695780503	0.612732451	0.790084656	2.23E-08
29487	CLDN10	0.751068684	0.682525909	0.826494878	4.55E-09
29488	TPX2	1.521668886	1.311451051	1.765583395	3.12E-08
29489	SLC25A4	0.508849275	0.418451274	0.618775949	1.29E-11

29490	HMGCL	0.532947748	0.412043558	0.689328341	1.63E-06
29491	TPST2	1.256946822	1.064603345	1.484041282	0.006960274
29492	GBA2	0.73895767	0.596157413	0.915963512	0.005759838
29493	TRUB2	0.473995561	0.354171945	0.634357957	5.14E-07
29494	UCK1	0.609520965	0.45988376	0.807847198	0.000571902
29495	EFHD1	0.720882218	0.632990999	0.820977192	8.08E-07
29496	ARPC3	2.024837664	1.382243448	2.966168927	0.000292568
29497	NRG1	0.742103536	0.626280337	0.879346876	0.000570969
29498	HSD11B1	1.235521826	1.105693605	1.380594204	0.000188674
29499	CDS1	0.643472087	0.556890482	0.74351482	2.24E-09
29500	CTHRC1	1.172120335	1.067476428	1.287022405	0.000873178
29501	CACNA2D4	1.2542184	1.059971038	1.484062997	0.008331243
29502	PLTP	1.26926144	1.153633736	1.396478409	9.96E-07
29503	SLC27A2	0.795373947	0.737323008	0.857995353	3.20E-09
29504	CYP4V2	0.675234824	0.57278519	0.796008828	2.91E-06
29505	STX3	0.661689855	0.543631846	0.805385974	3.81E-05
29506	ACSL1	0.695977321	0.605584569	0.799862574	3.29E-07
29507	C8G	1.333051794	1.17021379	1.518549091	1.53E-05
29508	LRRC3	1.200132768	1.004449153	1.433938846	0.044553357
29509	FBXL16	0.831500856	0.761812493	0.907564105	3.60E-05
29510	CHDH	0.712411323	0.63424869	0.80020645	1.07E-08
29511	HMMR	1.491052137	1.245713427	1.78470941	1.33E-05

29512	SLC5A8	0.866988166	0.813858127	0.923586623	9.71E-06
29513	TSGA10	0.539642717	0.373053572	0.780623169	0.001057449
29514	RLN2	0.557898012	0.415087379	0.749842583	0.000109616
29515	PMPCA	0.67024952	0.488075689	0.920419577	0.013421274
29516	FOXM1	1.587984857	1.375942283	1.832704713	2.55E-10
29517	RIN1	1.534052139	1.279657784	1.839019771	3.74E-06
29518	KIF14	2.022449994	1.599076601	2.557916223	4.18E-09
29519	ILVBL	0.5640297	0.454698125	0.69964991	1.90E-07
29520	APLP1	1.280510918	1.140730093	1.437419965	2.76E-05
29521	GRHPR	0.576788543	0.464752033	0.715833389	5.92E-07
29522	AUH	0.584673908	0.49030956	0.697199498	2.28E-09
29523	TMEM159	0.61400833	0.511444568	0.737139962	1.69E-07
29524	INSL3	1.450264573	1.19818876	1.755372276	0.00013563
29525	PIP5K1B	0.669239681	0.547516406	0.818024347	8.82E-05
29526	CEBPB	1.42239477	1.221692051	1.656069449	5.62E-06
29527	FLNC	1.234068525	1.120452785	1.359205087	1.97E-05
29528	ARVCF	0.827702888	0.686371545	0.998135887	0.047760236
29529	CCDC91	1.183131042	1.048605033	1.334915451	0.006321471
29530	TINAGL1	0.680651886	0.570523822	0.812037943	1.94E-05
29531	CPNE7	1.408753483	1.259916911	1.575172425	1.79E-09
29532	HSD17B8	0.54760127	0.453812845	0.660772726	3.32E-10
29533	CD82	1.268853022	1.090889008	1.475849494	0.002013567

29534	ASTN2	0.525427206	0.429176399	0.64326405	4.56E-10
29535	CDCA8	1.636164969	1.376018311	1.945494317	2.51E-08
29536	APCDD1L	1.302286329	1.185504925	1.430571604	3.59E-08
29537	STEAP3	1.29979889	1.185419888	1.425214113	2.42E-08
29538	CCL26	1.215532163	1.057702529	1.396913025	0.005950142
29539	PRKAB1	0.670741054	0.546362659	0.823433948	0.000135384
29540	MRRF	0.731373594	0.549353487	0.973703355	0.032155603
29541	NEDD4L	0.633502942	0.529452759	0.758001485	6.14E-07
29542	ABHD6	0.605918538	0.512156371	0.716846055	5.19E-09
29543	GBA3	0.807711428	0.758860934	0.859706598	1.96E-11
29544	C11orf71	0.612079655	0.469288054	0.79831886	0.000292489
29545	KIF20A	1.619034311	1.39647278	1.877066376	1.70E-10
29546	LHFPL3	0.771241541	0.598803784	0.993336265	0.044246703
29547	TDO2	1.26583396	1.093565226	1.465240093	0.001586797
29548	RTN4RL1	1.13415195	1.00671289	1.27772343	0.03845443
29549	CHEK2	2.001517349	1.530212295	2.617984259	4.08E-07
29550	DBNDD1	0.77632425	0.666898809	0.903704329	0.001090182
29551	TMEM158	1.443263352	1.274400519	1.634501141	7.50E-09
29552	WNT5A	1.309306509	1.098232453	1.560947804	0.002658796
29553	OR7E47P	0.85355257	0.757558139	0.961710993	0.009286267
29554	FABP5	1.322343265	1.121446135	1.559229335	0.000889784
29555	C1orf210	0.767859577	0.706340044	0.834737228	5.66E-10

29556	LY96	1.227688188	1.054341043	1.429535819	0.008258451
29557	PLAUR	1.503171125	1.298003452	1.740768429	5.22E-08
29558	WDR72	0.697582154	0.635177307	0.766118147	5.00E-14
29559	SLC12A5	1.918497391	1.37729038	2.672371993	0.000116648
29560	BUB1	1.603521642	1.351799782	1.90211723	5.97E-08
29561	TMEM125	0.759606645	0.689763424	0.836521966	2.31E-08
29562	COL10A1	1.223912176	1.084787283	1.380879954	0.001031299
29563	CASP5	1.57399413	1.220341462	2.030134678	0.000476551
29564	CCNB2	1.742884272	1.471698695	2.064040415	1.21E-10
29565	WT1	1.220549187	1.100171643	1.354098088	0.000168583
29566	OLFM2	1.190637059	1.037054861	1.366963948	0.013273836
29567	TIMP1	1.388759229	1.203412458	1.602652677	7.01E-06
29568	CEP55	1.636807735	1.400360129	1.913178978	6.01E-10
29569	ID4	0.808031211	0.728252848	0.896549103	5.85E-05
29570	ACSS1	0.758718342	0.620228544	0.928131297	0.007247325
29571	BSPRY	0.706611305	0.628479805	0.794455974	6.30E-09
29572	DENND1C	0.646648212	0.559079149	0.747933295	4.30E-09
29573	LGALS3BP	1.431038487	1.187384611	1.724690663	0.000167535
29574	C1R	1.265507713	1.138730753	1.406398981	1.23E-05
29575	SLC10A2	0.773733757	0.71446527	0.837918863	2.81E-10
29576	AMDHD1	0.708489068	0.618353722	0.811763141	6.91E-07
29577	NRXN2	0.832111723	0.738766697	0.937251127	0.002466319

29578	HAO2	0.833230146	0.777407338	0.893061388	2.51E-07
29579	ARHGEF10L	0.780609113	0.646053272	0.943189385	0.010291454
29580	TRIP13	1.848514398	1.519351534	2.248989391	8.22E-10
29581	SLC5A1	0.848678645	0.793004334	0.908261672	2.14E-06
29582	GLYATL1	0.837886001	0.774239197	0.90676493	1.14E-05
29583	SLC7A5	1.295462733	1.178129031	1.424482081	9.09E-08
29584	DHDH	0.788502734	0.713331378	0.871595699	3.34E-06
29585	CDCP1	1.15567341	1.048284315	1.274063736	0.003642092
29586	GRIK4	1.387780689	1.160620561	1.659401278	0.000326617
29587	SLC16A12	0.752244123	0.702661646	0.805325329	2.76E-16
29588	TMEM44	1.57700955	1.307481909	1.902098302	1.90E-06
29589	FAHD1	0.556346868	0.456686734	0.677755263	5.81E-09
29590	AURKA	1.645753227	1.355892544	1.997579894	4.65E-07
29591	MT2A	1.267250644	1.135139487	1.414737319	2.48E-05
29592	BDH2	0.586414623	0.483515613	0.711212008	5.90E-08
29593	ARPC1B	1.405282283	1.127681025	1.751220648	0.002444516
29594	PLXNB3	1.605673074	1.399782778	1.841847222	1.35E-11
29595	BAG1	0.483201178	0.383377765	0.609016483	7.27E-10
29596	PTPRN	1.177230802	1.065591602	1.300566144	0.001328753
29597	UNC5D	0.769928141	0.641598761	0.923925324	0.00494658
29598	LRP8	2.269045779	1.787832135	2.879783089	1.61E-11
29599	TREM1	1.309156813	1.158346383	1.479601946	1.60E-05

29600	EMX2	0.741130197	0.683099853	0.804090304	5.96E-13
29601	MT1X	1.166581898	1.071646503	1.269927463	0.000374057
29602	KLHDC7A	0.719152403	0.640248125	0.807780855	2.70E-08
29603	NFE2L3	1.351466515	1.141544812	1.599991277	0.000470511
29604	GKAP1	0.732742843	0.561757179	0.95577252	0.021812582
29605	ATOH8	0.707550508	0.626757807	0.798757856	2.24E-08
29606	SPOCK1	1.099065861	1.017617354	1.18703338	0.016194048
29607	HYAL1	0.614425148	0.535014542	0.705622432	5.27E-12
29608	KIF2C	1.679139016	1.416414618	1.990594986	2.37E-09
29609	ARHGEF16	0.758544451	0.673625566	0.854168417	5.06E-06
29610	SLC16A9	0.837920192	0.779713002	0.900472669	1.48E-06
29611	CIT	0.8906364	0.797618849	0.994501569	0.039596608
29612	GRAMD1C	0.591218309	0.497871805	0.702066446	2.04E-09
29613	CILP2	1.374590229	1.217893719	1.55144761	2.58E-07
29614	TMED3	1.346662827	1.011219161	1.793380544	0.041720758
29615	DPY19L1P1	1.754100905	1.398049963	2.200829775	1.21E-06
29616	AMZ1	1.222120159	1.040296544	1.435723008	0.014660755
29617	EXPH5	0.655540459	0.536672287	0.800736881	3.52E-05
29618	SLC17A4	0.871723257	0.812225635	0.935579233	0.000141155
29619	GTSE1	1.897624851	1.575263323	2.28595437	1.54E-11
29620	SLC22A12	0.849435957	0.796278045	0.906142584	7.46E-07
29621	TNFSF13B	1.215517663	1.058783603	1.39545341	0.005589495

"Table-S6: Functional annotation for m6A phenotype -related genes (Gene Ontology-Biological process)"

ONTOLOGY ID Description GeneRatio BgRatio pvalue p.adjust qvalue geneID Count

BP GO:0048193 Golgi vesicle transport 55/1105 374/18866 2.54E-10 7.86E-07 6.40E-07 RAB6A/KLHL20/SEC23A/TMED10/MYO1B/TBC1D20/ARCN1/COPA/SAR1A/MCFD2/MIA3/DCTN5/YIPF5/SPAST/ANKRD28/DCTN4/GOSR2/GOLPH3/BCAP29/SEC23IP/CREB3L2/ARF3/SNAP23/RAB10/RAB8B/RNF139/NRB

P1/EXOC4/COPB1/KIF3C/RAB31/STX6/RAB1A/COG5/ARFGAP3/SEC22B/COPB2/TMED7/GBF1/MYO5A/RABIF/RP2/LMAN1/SCAMP1/SURF4/LMAN2L/GOLPH3L/NSF/TMED5/TGFA/RINT1/ANK3/CCDC91/KIF2C/TMED3 55

BP GO:1901796 regulation of signal transduction by p53 class mediator 35/1105 180/18866 2.83E-10 7.86E-07 6.40E-07 PHF20/SPRED1/HIPK1/NUAK1/CHD4/TP53BP2/MAPK14/TRIM24/SSRP1/TAF2/HDAC1/RAD1/PSMD10/TOPBP1/CSNK2A1/CSNK2A2/CDK2/NBN/TP53RK/PRKAB2/RRM2B/RNF34/RBBP7/BRCA1/PML/PPP1R13B/TAF13/CHD3/RFC5/BCL2/CD44/TPX2/PRKAB1/CHEK2/AURKA 35

BP GO:1903829 positive regulation of cellular protein localization 50/1105 338/18866 1.36E-09 2.52E-06 2.05E-06 ROCK2/SH3GLB1/ITGB1/TMEM30A/IQGAP1/MSN/YWHAQ/KIF5B/PPP3R1/YWHAB/TP53BP2/SAR1A/MAPK14/RIOK2/SSH1/EDEM1/ATP2B4/ARF6/EIF2AK3/YWHAZ/CCT8/NPEPPS/CCT4/LRP4/CCT7/FLNA/SAE1/PIK3R1/CSNK2A2/HDAC3/RHOU/DKC1/PRNP/CCT2/EPHA3/NMT1/CYLD/EGFR/PPP1R13B/DTX3L/PARP9/BCL2/EDEM2/ERBB2/MAPT/ANK3/GAS8/STX3/ARHGEF16/GTSE1 50

BP GO:0072331 signal transduction by p53 class mediator 42/1105 267/18866 4.51E-09 6.28E-06 5.11E-06 PHF20/SPRED1/HIPK1/NUAK1/CNOT8/MSH2/CNOT2/CHD4/TP53BP2/MAPK14/TRIM24/SSRP1/TAF2/HDAC1/RAD1/PSMD10/TOPBP1/CSNK2A1/CSNK2A2/CDK2/NBN/TP53RK/PRKAB2/SP100/RRM2B/RNF34/RBBP7/BR

CA1/PML/PPP1R13B/TAF13/CHD3/RFC5/BCL2/CD44/CCNB1/TPX2/FOXM1/PRKAB1/CHEK2/AURKA/GTSE1 42

BP GO:0006888 endoplasmic reticulum to Golgi vesicle-mediated transport 35/1105 206/18866 1.17E-08 1.09E-05 8.85E-06 SEC23A/TMED10/TBC1D20/ARCN1/COPA/SAR1A/MCFD2/MIA3/DCTN5/YIPF5/SPAST/ANKRD28/DCTN4/GOSR2/BCAP29/SEC23IP/CREB3L2/RNF139/NRBP1/COPB1/RAB1A/COG5/ARFGAP3/SEC22B/COPB2/TMED7/GBF1/LMAN1/LMAN2L/NSF/TMED5/TGFA/RINT1/ANK3/TMED3 35

BP GO:0010498 proteasomal protein catabolic process 61/1105 483/18866 1.17E-08 1.09E-05 8.85E-06 KCTD10/CDC27/USP13/CSNK1A1/KLHL20/EDEM3/ANAPC1/RNF103/CUL4B/SMARCC1/SENP1/SEL1L/GNA12/PSMD12/UBE2J1/EDEM1/DNAJC10/UBE2G1/TMTC3/OS9/NEDD4/RNF4/CUL2/RHBDD1/RNF139/PLAA/CUL1/PSMD10/PSMD1/SGTB/COP1/BFAR/ENC1/BIRC2/PSMF1/PSMD11/SUMO2/CDK2/UBQLN4/PSME4/UBE2A/FBXW8/PSMD2/FBXL7/PSME3/UBE2E1/HFE/RNF34/STT3B/PSMD3/PML/SDCBP/EDEM2/CCNB1/KCTD17/HM13/ANAPC2/FBXL16/KIF14/NEDD4L/AURKA 61

BP GO:0051656 establishment of organelle localization 56/1105 432/18866 1.88E-08 1.43E-05 1.17E-05 RAB6A/CLASP1/ITGB1/SEC23A/KIF5B/TMED10/MYO1B/TBC1D20/SAR1A/ACTR2/RIOK2/MCFD2/MYO1E/AP3M1/SPAST/MAP4/ANKRD28/GOSR2/ACTR3/ARHGAP21/KPNB1/SEC23IP/SNAP23/DOCK7/MAP1B/AP3B1/EXO

C4/YWHAZ/KIF3C/ARHGEF2/TERF1/MYO1D/RAB1A/INCENP/C12orf4/ARF
GAP3/SEC22B/GBF1/MYO5A/TBC1D23/RHOT1/LMAN1/MLH1/MIS12/LYN/
NSF/IL4R/IKBKG/STXBP1/TGFA/CCNB1/MAPT/LIN7A/KIF14/CDCA8/KIF2C
56
BP GO:0051056 regulation of small GTPase mediated signal transduction
46/1105 323/18866 2.06E-08 1.43E-05 1.17E-05
KCTD10/MAP4K4/HEG1/AKAP13/ITGB1/ABL2/SHOC2/STARD13/SIPA1
L2/CRKL/ROBO1/NRP1/KALRN/NOTCH2/GNA13/ARHGAP21/ARF6/STAMB
P/GDI2/TRIO/F2R/ARHGEF2/CDC42SE1/ARFGEF1/ITSN1/RAF1/OCRL/GB
F1/RASA3/RHOT1/RHOU/ARHGEF6/ARHGAP20/RALGPS2/PLEKHG2/LYN/
GRB2/RAC1/MCF2L/GARNL3/ARHGAP24/IRS2/NRG1/FOXM1/KIF14/ARHG
EF16 46
BP GO:0043161 proteasome-mediated ubiquitin-dependent protein
catabolic process 55/1105 424/18866 2.47E-08 1.52E-05 1.24E-05
KCTD10/CDC27/CSNK1A1/KLHL20/EDEM3/ANAPC1/RNF103/CUL4B/S
MARCC1/SENP1/SEL1L/GNA12/PSMD12/UBE2J1/EDEM1/DNAJC10/UBE2
G1/OS9/NEDD4/RNF4/CUL2/PLAA/CUL1/PSMD10/PSMD1/SGTB/COP1/BF
AR/BIRC2/PSMF1/PSMD11/SUMO2/CDK2/UBQLN4/PSME4/UBE2A/FBXW8
/PSMD2/FBXL7/PSME3/UBE2E1/HFE/RNF34/STT3B/PSMD3/PML/SDCBP/
EDEM2/CCNB1/KCTD17/ANAPC2/FBXL16/KIF14/NEDD4L/AURKA 55
BP GO:0032386 regulation of intracellular transport 48/1105 358/18866
6.93E-08 3.76E-05 3.06E-05

SH3GLB1/TMEM30A/MSN/YWHAQ/KIF5B/PPP3R1/PTPN14/YWHAB/TBC1D20/NOLC1/TP53BP2/SAR1A/MAPK14/RIOK2/UBE2J1/EDEM1/YIPF5/RAB23/MAP2K1/OS9/NEDD4/MAP1B/RNF139/PTPN11/YWHAZ/NPEPPS/FLNA/SAE1/PIK3R1/CSNK2A2/PPP1CC/HDAC3/RHOT1/SP100/NCBP2/RHOU/LMAN1/NSUN2/PRNP/NMT1/LDLRAP1/PPP1R13B/RINT1/BCL2/EDEM2/ERBB2/ANK3/GTSE1 48

BP GO:0009100 glycoprotein metabolic process 53/1105 415/18866 7.44E-08 3.76E-05 3.06E-05

BMPR2/DPY19L3/EXT2/GANAB/MAN1A2/EDEM3/FAM20B/NDST1/CHSY1/MGAT5/RNF103/HS2ST1/SEL1L/MCFD2/UBE2J1/EDEM1/TMTC3/OS9/GOLPH3/GFPT1/IDS/GALNT1/RNF139/FUT4/STT3A/ST3GAL2/CANT1/NCSTN/GNPTAB/RAB1A/ARFGEF1/EXT1/LMAN1/SULF1/PGM3/STT3B/MAN2A1/B4GALT3/DPM1/ST3GAL1/ADAMTS9/MANBA/GALNT2/GALNT10/DPAGT1/PHLDA1/ARSB/SLC35D1/SULF2/BCL2/EDEM2/POMT1/HYAL1 53

BP GO:0006890 retrograde vesicle-mediated transport, Golgi to endoplasmic reticulum 20/1105 87/18866 1.05E-07 4.89E-05 3.98E-05

RAB6A/TMED10/ARCN1/COPA/GOLPH3/ARF3/COPB1/KIF3C/RAB1A/ARFGAP3/SEC22B/COPB2/TMED7/GBF1/SURF4/GOLPH3L/NSF/RINT1/KIF2C/TMED3 20

BP GO:0010256 endomembrane system organization 57/1105 468/18866 1.25E-07 5.35E-05 4.35E-05

CLASP1/SH3GLB1/CSNK1A1/RAB3GAP1/NUP155/TMED10/SCARB2/TBC1D20/ATP2A2/MIA3/REEP3/SPAST/TOR1B/MAP2K1/ARHGAP21/GOLPH3/SEC23IP/EIF2AK3/RAB10/AP3B1/YWHAZ/ATP8B2/F2R/VCPIP1/STX6/RAB1A/PARP11/ARFGEF1/RAB43/TMEM43/TMED7/GBF1/MYO5A/VPS37A/FBXW8/RAB30/LMAN1/SURF4/NUP93/VAMP4/TOR1AIP2/LMAN2L/GOLPH3L/CORO1C/SMPD4/PI4K2B/STX2/TMED5/TMEM38B/CCNB1/ANK3/VPS37D/SERPINE2/CCNB2/PTPRN/TMED3/HOOK2 57

BP GO:1901990 regulation of mitotic cell cycle phase transition 55/1105 448/18866 1.60E-07 6.35E-05 5.17E-05 ADAM17/CDC27/CLASP1/LSM11/USP47/ANAPC1/NEDD1/CNOT8/CNOT2/CUL4B/TAOK1/PRKDC/MAPRE1/RIOK2/CDC73/PSMD12/SENP2/CKAP5/CUL1/PSMD10/PSMD1/MTA3/TOPBP1/PSMF1/PSMD11/RBL1/RAD51C/CDK2/NBN/EIF4G1/PSME4/PSMD2/ZFP36L1/FBXL7/PSME3/UBE2E1/PSMD3/TUBB/BRCA1/PML/EGFR/RINT1/BCL2/FAM107A/CCNB1/ANAPC2/TPX2/HMMR/KIF14/CHEK2/BUB1/TRIP13/AURKA/HYAL1/GTSE1 55

BP GO:0007030 Golgi organization 27/1105 151/18866 1.85E-07 6.88E-05 5.60E-05 CLASP1/CSNK1A1/TMED10/TBC1D20/MAP2K1/ARHGAP21/GOLPH3/SEC23IP/YWHAZ/ATP8B2/VCPIP1/STX6/RAB1A/ARFGEF1/RAB43/TMED7/GBF1/FBXW8/RAB30/LMAN1/SURF4/VAMP4/LMAN2L/GOLPH3L/PI4K2B/TMED5/TMED3 27

BP GO:0000086 G2/M transition of mitotic cell cycle 37/1105 254/18866

2.78E-07 9.66E-05 7.86E-05 CLASP1/USP47/NEDD1/TAOK1/MAPRE1/PSMD12/CKAP5/PPME1/TAF2/CUL1/PSMD10/PSMD1/MTA3/TOPBP1/PSMF1/PSMD11/RAD51C/CDK2/NBN/PSME4/PSMD2/FBXL7/PSME3/MASTL/PSMD3/TUBB/BRCA1/PHLDA1/RINT1/CCNB1/TPX2/HMMR/FOXM1/KIF14/CHEK2/CCNB2/AURKA 37

BP GO:0046578 regulation of Ras protein signal transduction 31/1105 194/18866 3.27E-07 0.000107126 8.72E-05 KCTD10/MAP4K4/HEG1/AKAP13/ITGB1/ABL2/SHOC2/STARD13/CRKL/ROBO1/NRP1/KALRN/NOTCH2/ARF6/STAMBP/F2R/ARHGEF2/CDC42SE1/ARFGEF1/RAF1/GBF1/RASA3/RALGPS2/LYN/GRB2/RAC1/ARHGAP24/IRS2/NRG1/FOXM1/KIF14 31

BP GO:0007265 Ras protein signal transduction 45/1105 346/18866 4.26E-07 0.000131842 0.000107334 ROCK2/KCTD10/MAP4K4/HEG1/AKAP13/ITGB1/ABL2/SHOC2/STARD13/MAPK14/CRKL/ROBO1/NRP1/GNA12/KALRN/NOTCH2/GNA13/NRAS/KPNB1/BRAP/ARF6/RAP1B/STAMBP/F2R/ARHGEF2/CDC42SE1/ARFGEF1/RAF1/GBF1/CDK2/RASA3/RAB30/RHOU/DNMT1/RALGPS2/LYN/GRB2/RAC1/AGTR1/ARHGAP24/RCC1/IRS2/NRG1/FOXM1/KIF14 45

BP GO:0034976 response to endoplasmic reticulum stress 40/1105 294/18866 5.78E-07 0.000169388 0.000137901 SERINC3/USP13/EIF2AK4/ATF6/EIF2AK2/EDEM3/RNF103/ATP2A2/SEL1L/BCL2L11/UBE2J1/EDEM1/DNAJC10/DNAJC3/TMTC3/GOSR2/OS9/CRE

B3L2/GFPT1/EIF2AK3/RHBDD1/SSR1/RNF139/APAF1/SGTB/MBTPS2/CANX/PIK3R1/BFAR/EIF4G1/STT3B/PML/ITPR1/BCL2/EDEM2/P4HB/HM13/IGFBP1/CXXC1/CEBPB 40

BP GO:0044839 cell cycle G2/M phase transition 38/1105 273/18866 6.21E-07 0.000172665 0.000140569 CLASP1/USP47/NEDD1/TAOK1/MAPRE1/PSMD12/CKAP5/PPME1/TAF2/CUL1/PSMD10/PSMD1/MTA3/TOPBP1/PSMF1/PSMD11/RAD51C/CDK2/NBN/PSME4/PSMD2/FBXL7/PSME3/MASTL/PSMD3/TUBB/BRCA1/PHLDA1/RINT1/CCNB1/TPX2/HMMR/FOXM1/KIF14/CHEK2/CCNB2/AURKA/GTSE1 38

BP GO:0048194 Golgi vesicle budding 18/1105 81/18866 7.76E-07 0.00020575 0.000167503 SEC23A/TMED10/TBC1D20/SAR1A/MCFD2/MIA3/ANKRD28/GOSR2/GOLPH3/SEC23IP/RAB1A/ARFGAP3/SEC22B/GBF1/LMAN1/GOLPH3L/NSF/TGFA 18

BP GO:1901987 regulation of cell cycle phase transition 56/1105 486/18866 9.84E-07 0.000249014 0.000202725 ADAM17/CDC27/CLASP1/LSM11/USP47/ANAPC1/NEDD1/CNOT8/CNOT2/CUL4B/TAOK1/PRKDC/MAPRE1/RIOK2/CDC73/PSMD12/ATP2B4/SENP2/CKAP5/CUL1/PSMD10/PSMD1/MTA3/TOPBP1/PSMF1/PSMD11/RBL1/RAD51C/CDK2/NBN/EIF4G1/PSME4/PSMD2/ZFP36L1/FBXL7/PSME3/UBE2E1/PSMD3/TUBB/BRCA1/PML/EGFR/RINT1/BCL2/FAM107A/CCNB1/ANAPC2/

TPX2/HMMR/KIF14/CHEK2/BUB1/TRIP13/AURKA/HYAL1/GTSE1 56

BP GO:0001701 in utero embryonic development 47/1105 383/18866 1.28E-06 0.000309843 0.000252247 HEG1/ITGB1/SP3/SH3PXD2A/PELO/MSH2/CNOT2/ADAM10/SLC30A1/MYO1E/BCL2L11/GNA12/NOTCH2/SENP2/GNA13/ACVR1/EGLN1/MAP2K1/SKIL/RXRA/CAPN2/OCRL/NBN/UBE2A/FBXW8/MBNL1/BTF3/TGFBR1/EDNRA/NSUN2/ZFP36L1/ZFPM2/COPS3/MAN2A1/NMT1/TGFB3/EGFR/SMPD4/GRB2/LEF1/SOX6/CCNB1/CCNB1IP1/HM13/ADA/CEBPB/CCNB2 47

BP GO:0048732 gland development 52/1105 443/18866 1.38E-06 0.000319315 0.000259958 MSN/SP3/SMARCC1/PRKDC/CRKL/ROBO1/NRP1/BCL2L11/KALRN/SEC63/NOTCH2/MAP2K1/ARF6/PLXND1/RB1CC1/PAM/DAG1/PPAT/CSNK2A2/RXRA/RAF1/CEBPG/TGFBR1/GNPNAT1/ETV5/SULF1/HFE/MAN2A1/TGFB3/PML/EGFR/OAS2/TWSG1/NCOR2/SULF2/BCL2/NTN4/IRF6/LEF1/IRS2/SERPINE2/SEMA3C/ADA/PTPN3/HMGCL/NRG1/CEBPB/WNT5A/CCNB2/WT1/ID4/AURKA 52

BP GO:0043687 post-translational protein modification 45/1105 363/18866 1.63E-06 0.000362193 0.000294865 KLHL20/CAND1/TGOLN2/CUL4B/ADAM10/LAMC1/FBN1/CHML/PSMD12/MIA3/DNAJC3/CUL2/RHBDD1/CUL1/PSMD10/PSMD1/STT3A/COP1/RAB1A/PSMF1/PSMD11/COPS8/KLHL2/PSME4/FBXW8/CRTAP/PSMD2/ATG7/FBXL7/PSME3/ATG12/COPS3/STT3B/ATG5/PSMD3/RBBP7/APLP2/CP/LMO

7/P4HB/TMEM132A/SCG2/IGFBP1/FBXL16/TIMP1 45

BP GO:0031647 regulation of protein stability 39/1105 296/18866 1.80E-06 0.000368648 0.000300121 USP13/SH3GLB1/USP47/USP9X/PRKDC/TRIM24/SEL1L/CDC73/SENP2/RNF139/CCT8/GTPBP4/CCT4/CCT7/FLNA/PRKRA/PIK3R1/HDAC3/PTGES3/CRTAP/ATF7IP/PRNP/CMTM6/CCT2/LAMP2/HSPA8/STXBP1/TMEM25/PER3/BCL2/FAM107A/TMEM88/TSC1/NEDD4L/GBA3/CHEK2/AURKA/BAG1/GTSE1 39

BP GO:0006623 protein targeting to vacuole 12/1105 40/18866 1.86E-06 0.000368648 0.000300121 SCARB2/ZFYVE16/AP3M1/GOSR2/NEDD4/AP3B1/GNPTAB/VPS37A/M6PR/LAMP2/HSPA8/VPS37D 12

BP GO:0006622 protein targeting to lysosome 9/1105 22/18866 1.95E-06 0.000368648 0.000300121 SCARB2/ZFYVE16/AP3M1/NEDD4/AP3B1/GNPTAB/M6PR/LAMP2/HSPA8 9

BP GO:0010389 regulation of G2/M transition of mitotic cell cycle 30/1105 200/18866 1.97E-06 0.000368648 0.000300121 CLASP1/USP47/NEDD1/TAOK1/MAPRE1/PSMD12/CKAP5/CUL1/PSMD10/PSMD1/MTA3/TOPBP1/PSMF1/PSMD11/RAD51C/CDK2/NBN/PSME4/PSMD2/FBXL7/PSME3/PSMD3/TUBB/BRCA1/RINT1/CCNB1/TPX2/HMMR/KIF14/AURKA 30

BP GO:0033157 regulation of intracellular protein transport 36/1105 264/18866 1.99E-06 0.000368648 0.000300121 SH3GLB1/TMEM30A/YWHAQ/KIF5B/PPP3R1/PTPN14/YWHAB/NOLC1/TP53BP2/SAR1A/MAPK14/RIOK2/UBE2J1/EDEM1/RAB23/OS9/PTPN11/YWHAZ/NPEPPS/FLNA/SAE1/PIK3R1/CSNK2A2/HDAC3/SP100/RHOU/LMAN1/NSUN2/PRNP/NMT1/PPP1R13B/BCL2/EDEM2/ERBB2/ANK3/GTSE1 36

BP GO:0006903 vesicle targeting 19/1105 95/18866 2.12E-06 0.00038036 0.000309655 CLASP1/SEC23A/TMED10/TBC1D20/SAR1A/MCFD2/ANKRD28/GOSR2/SEC23IP/SNAP23/EXOC4/RAB1A/ARFGAP3/SEC22B/GBF1/TBC1D23/LMAN1/NSF/TGFA 19

BP GO:0032388 positive regulation of intracellular transport 32/1105 222/18866 2.20E-06 0.000382494 0.000311392 SH3GLB1/TMEM30A/MSN/YWHAQ/KIF5B/PPP3R1/YWHAB/TBC1D20/TP53BP2/SAR1A/MAPK14/RIOK2/EDEM1/NEDD4/YWHAZ/NPEPPS/FLNA/SAE1/PIK3R1/CSNK2A2/HDAC3/NCBP2/RHOU/PRNP/NMT1/LDLRAP1/PPP1R13B/BCL2/EDEM2/ERBB2/ANK3/GTSE1 32

BP GO:0034504 protein localization to nucleus 37/1105 277/18866 2.37E-06 0.000399361 0.000325124 NUP98/IPO9/STAT3/NUP155/TNPO1/CSE1L/NOLC1/MAPK14/KPNA3/RAB23/TOR1B/PPP3CA/KPNB1/EIF2AK3/IPO7/KPNA4/CCT8/CCT4/CCT7/FLNA/PIK3R1/CBLB/HDAC3/SP100/DKC1/NUP93/CCT2/SUMO3/TOR1AIP2/N

XT2/NUP107/ARL2BP/PML/TNPO3/DTX3L/PARP9/GTSE1 37
BP GO:0016049 cell growth 55/1105 490/18866 2.76E-06
0.000451378 0.000367472
BMPR2/ADAM17/AKAP13/ITGB1/VCL/RAPH1/IQGAP1/EIF2AK4/USP47/
ADNP/USP9X/ADAM10/CRKL/EI24/CDKL5/CDC73/EIF4G2/NRP1/WDR36/P
DLIM5/RYK/ADIPOR1/MAP1B/PLAA/PSMD10/MTPN/CSNK2A1/RPS6KA3/E
IF4G1/TGFBR1/NDRG3/RBBP7/PML/EGFR/SDCBP/SORBS2/SEMA4B/BCL
2/FAM107A/AGTR1/ERBB2/LEF1/MAPT/SERPINE2/IGFBP1/SEMA3C/PPAR
G/ANAPC2/KIF14/NEDD4L/WNT5A/WT1/AURKA/SPOCK1/HYAL1 55
BP GO:0042176 regulation of protein catabolic process 47/1105
397/18866 3.48E-06 0.000553597 0.000450689
USP13/CSNK1A1/MSN/SF3B3/CUL4B/SMARCC1/SENP1/GNA12/UBE2
J1/EDEM1/TMTC3/EGLN1/OS9/NEDD4/AZIN1/RNF139/PSMD10/PSMD1/FL
NA/COP1/CSNK2A1/CSNK2A2/PSMF1/IDE/SEC22B/SUMO2/UBQLN4/FBX
W8/ADAM9/PSMD2/ATG7/PSME3/HFE/PSMD3/PML/EGFR/NSF/SDCBP/ED
EM2/SERPINE2/PTPN3/NRG1/NEDD4L/CHEK2/WNT5A/TIMP1/AURKA 47
BP GO:1902749 regulation of cell cycle G2/M phase transition 31/1105
217/18866 3.81E-06 0.000575511 0.00046853
CLASP1/USP47/NEDD1/TAOK1/MAPRE1/PSMD12/CKAP5/CUL1/PSMD
10/PSMD1/MTA3/TOPBP1/PSMF1/PSMD11/RAD51C/CDK2/NBN/PSME4/PS
MD2/FBXL7/PSME3/PSMD3/TUBB/BRCA1/RINT1/CCNB1/TPX2/HMMR/KIF
14/AURKA/GTSE1 31

BP GO:0000209 protein polyubiquitination 42/1105 340/18866 3.93E-06 0.000575511 0.00046853 CDC27/KLHL20/TRIP12/ANAPC1/PSMD12/UBE2J1/UBE2G1/NEDD4/RNF4/FBXO28/LNPEP/PLAA/CUL1/PSMD10/PSMD1/NPEPPS/UBE2V2/UBE2N/BFAR/TRIM5/BIRC2/PSMF1/PSMD11/PSME4/UBE2A/FBXW8/CBFB/PSMD2/FBXL7/PSME3/UBE2O/UBE2E1/RNF34/PSMD3/BRCA1/DTX3L/BCL2/LMO7/RNF152/ANAPC2/FBXL16/NEDD4L 42

BP GO:0016050 vesicle organization 42/1105 340/18866 3.93E-06 0.000575511 0.00046853 SEC23A/KIF5B/TGFBRAP1/TMED10/SCARB2/TBC1D20/GNAI3/SAR1A/ZFYVE16/MCFD2/MIA3/ANKRD28/GOSR2/GOLPH3/SEC23IP/SNAP23/AP3B1/RAB8B/F2R/STX6/RAB1A/ARFGAP3/SEC22B/GBF1/VPS37A/SAMD9/LMAN1/VAMP4/GOLPH3L/CORO1C/NSF/PI4K2B/STXBP1/STX2/SDCBP/TGFA/BCL2/VPS37D/SERPINE2/STX3/PTPRN/HOOK2 42

BP GO:0002474 antigen processing and presentation of peptide antigen via MHC class I 19/1105 99/18866 4.04E-06 0.000575849 0.000468805 SEC23A/PSMD12/SNAP23/LNPEP/ITGB5/PSMD10/PSMD1/CANX/PSMF1/PSMD11/ITGAV/IDE/SEC22B/PSME4/PSMD2/PSME3/HFE/PSMD3/IKBKG 19

BP GO:1905477 positive regulation of protein localization to membrane 22/1105 127/18866 4.26E-06 0.000593326 0.000483033

ITGB1/YWHAQ/KIF5B/PPP3R1/YWHAB/TP53BP2/SSH1/ATP2B4/ARF6/YWHAZ/LRP4/PIK3R1/PRNP/EPHA3/NMT1/EGFR/PPP1R13B/BCL2/ERBB2/ANK3/STX3/ARHGEF16 22

BP GO:2000736 regulation of stem cell differentiation 21/1105 118/18866 4.51E-06 0.00060746 0.00049454 YAP1/EIF2AK2/STAT3/PRKDC/PSMD12/PWP1/PSMD10/PSMD1/TCF12/PSMF1/PSMD11/PSME4/CBFB/NSUN2/PSMD2/PSME3/PSMD3/SOX5/RUNX1/SOX6/SEMA3C 21

BP GO:2001233 regulation of apoptotic signaling pathway 48/1105 413/18866 4.58E-06 0.00060746 0.00049454 SERINC3/YAP1/YWHAQ/PPP3R1/USP47/YWHAB/GNAI3/TP53BP2/PAK2/BCL10/NRP1/BCL2L11/ACVR1/RB1CC1/EIF2AK3/HDAC1/PSMD10/GCLM/YWHAZ/APAF1/SKIL/ARHGEF2/PRKRA/CSNK2A1/CSNK2A2/RAF1/ITGAV/TGFBR1/SP100/PHIP/PSME3/RRM2B/RNF34/NMT1/BRCA1/PLAGL2/CYLD/PML/PPP1R13B/BCL2/SERPINE1/CD44/CX3CL1/P4HB/MLLT11/ACAA2/SCG2/PLAUR 48

BP GO:0031589 cell-substrate adhesion 43/1105 359/18866 6.69E-06 0.000842461 0.000685856 PARVA/ROCK2/MAP4K4/CLASP1/ITGB1/VCL/IQGAP1/ATRN/LAMC1/CRKL/NRP1/BCL2L11/NID1/RSU1/NID2/ITGB5/FLNA/DAG1/ECM2/RAB1A/PIK3R1/ITGAV/CASK/ADAM9/EPHA3/EMP2/ADAMTS9/FMN1/CORO1C/FZD7/RAC1/BCL2/SERPINE1/FAM107A/CD44/NTN4/CX3CL1/P4HB/AJAP1/TSC1/

LAMB3/KIF14/SPOCK1 43

BP GO:0009113 purine nucleobase biosynthetic process 6/1105 10/18866 6.82E-06 0.000842461 0.000685856 GMPS/PPAT/PRPS1/GART/PAICS/ADA 6

BP GO:0032836 glomerular basement membrane development 6/1105 10/18866 6.82E-06 0.000842461 0.000685856 MYO1E/NID1/SULF1/SULF2/COL4A4/WT1 6

BP GO:0097193 intrinsic apoptotic signaling pathway 37/1105 290/18866 6.96E-06 0.000842461 0.000685856 SERINC3/MSH6/HIPK1/USP47/MSH2/TP53BP2/PRKDC/BCL2L11/DNAJC10/EIF2AK3/HDAC1/CUL2/CUL1/APAF1/SKIL/ARHGEF2/PRKRA/PIK3R1/NBN/RRM2B/ZNF622/MLH1/BRCA1/PLAGL2/CYLD/DIABLO/PML/ITPR1/PPP1R13B/BCL2/CD44/CYP1B1/P4HB/MLLT11/CEBPB/CHEK2/PLAUR 37

BP GO:0030307 positive regulation of cell growth 26/1105 171/18866 7.27E-06 0.000860574 0.000700602 BMPR2/ADAM17/USP47/ADNP/ADAM10/CDKL5/EIF4G2/NRP1/MAP1B/PLAA/PSMD10/MTPN/CSNK2A1/RPS6KA3/EIF4G1/TGFBR1/EGFR/SDCBP/BCL2/ERBB2/LEF1/MAPT/IGFBP1/ANAPC2/NEDD4L/HYAL1 26

BP GO:0090316 positive regulation of intracellular protein transport 27/1105 182/18866 7.80E-06 0.000904164 0.00073609 SH3GLB1/TMEM30A/YWHAQ/KIF5B/PPP3R1/YWHAB/TP53BP2/SAR1A/MAPK14/RIOK2/EDEM1/YWHAZ/NPEPPS/FLNA/SAE1/PIK3R1/CSNK2A2/

HDAC3/RHOU/PRNP/NMT1/PPP1R13B/BCL2/EDEM2/ERBB2/ANK3/GTSE1 27

BP GO:1905475 regulation of protein localization to membrane 28/1105 195/18866 1.00E-05 0.001118312 0.00091043 ITGB1/YWHAQ/KIF5B/PPP3R1/YWHAB/ADAM10/TP53BP2/CRKL/SSH1/ATP2B4/ARF6/CLTC/YWHAZ/LRP4/DAG1/PIK3R1/PRNP/EPHA3/NMT1/LDLRAP1/EGFR/PPP1R13B/ZDHHC7/BCL2/ERBB2/ANK3/STX3/ARHGEF16 28

BP GO:0010972 negative regulation of G2/M transition of mitotic cell cycle 18/1105 96/18866 1.01E-05 0.001118312 0.00091043 USP47/TAOK1/PSMD12/CUL1/PSMD10/PSMD1/TOPBP1/PSMF1/PSMD11/NBN/PSME4/PSMD2/FBXL7/PSME3/PSMD3/BRCA1/RINT1/AURKA 18

BP GO:0006901 vesicle coating 15/1105 70/18866 1.02E-05 0.001118312 0.00091043 SEC23A/TMED10/TBC1D20/SAR1A/MCFD2/ANKRD28/GOSR2/SEC23IP/RAB1A/ARFGAP3/SEC22B/GBF1/LMAN1/NSF/TGFA 15

BP GO:1901991 negative regulation of mitotic cell cycle phase transition 33/1105 251/18866 1.15E-05 0.001227415 0.000999252 USP47/CNOT8/CNOT2/TAOK1/PRKDC/CDC73/PSMD12/CUL1/PSMD10/PSMD1/TOPBP1/PSMF1/PSMD11/RBL1/CDK2/NBN/PSME4/PSMD2/ZFP36L1/FBXL7/PSME3/PSMD3/BRCA1/PML/RINT1/BCL2/FAM107A/CCNB1/CHEK2/BUB1/TRIP13/AURKA/GTSE1 33

BP GO:0002283 neutrophil activation involved in immune response 53/1105 490/18866 1.21E-05 0.001271423 0.001035079 RAB6A/VCL/TMEM30A/ATP11B/IQGAP1/CAND1/XRCC5/KCMF1/ADAM10/MAPK14/PAFAH1B2/ACTR2/CAP1/PSMD12/DNAJC3/NRAS/KPNB1/YPEL5/SNAP23/RAB10/COPB1/PSMD1/RAP1B/APAF1/CCT8/GDI2/RAB31/CANT1/NCSTN/PSMD11/ITGAV/TXNDC5/SCAMP1/PSMD2/ATG7/SURF4/CMTM6/CCT2/RAB3D/PSMD3/TUBB/MANBA/LAMP2/HSPA8/ARSB/RAC1/SDCBP/CD44/MGAM/DNASE1L3/SLC27A2/FABP5/PLAUR 53

BP GO:0018209 peptidyl-serine modification 40/1105 333/18866 1.30E-05 0.001336829 0.001088327 ROCK2/CSNK1A1/NSD1/HIPK1/CSNK1G3/RPS6KB1/PRKDC/MAPK14/PAK2/STK38L/ATP2B4/TOP1/WNK1/VRK2/PWP1/EIF2AK3/GALNT1/DOCK7/SRPK2/CSNK2A1/RPS6KA3/CSNK2A2/TBK1/RAF1/CDK2/EIF4G1/TGFBR1/MASTL/TLK1/EGFR/GALNT2/PLCL2/BCL2/STK32B/CD44/STK39/CCNB1/CHEK2/WNT5A/AURKA 40

BP GO:0018105 peptidyl-serine phosphorylation 38/1105 310/18866 1.34E-05 0.001345151 0.001095102 ROCK2/CSNK1A1/NSD1/HIPK1/CSNK1G3/RPS6KB1/PRKDC/MAPK14/PAK2/STK38L/ATP2B4/TOP1/WNK1/VRK2/PWP1/EIF2AK3/DOCK7/SRPK2/CSNK2A1/RPS6KA3/CSNK2A2/TBK1/RAF1/CDK2/EIF4G1/TGFBR1/MASTL/TLK1/EGFR/PLCL2/BCL2/STK32B/CD44/STK39/CCNB1/CHEK2/WNT5A/AURKA 38

BP GO:0036507 protein demannosylation 8/1105 21/18866 1.38E-05 0.001345151 0.001095102 MAN1A2/EDEM3/RNF103/SEL1L/EDEM1/OS9/RNF139/EDEM2 8

BP GO:0036508 protein alpha-1,2-demannosylation 8/1105 21/18866 1.38E-05 0.001345151 0.001095102 MAN1A2/EDEM3/RNF103/SEL1L/EDEM1/OS9/RNF139/EDEM2 8

BP GO:1904951 positive regulation of establishment of protein localization 43/1105 370/18866 1.42E-05 0.001345151 0.001095102 SH3GLB1/TMEM30A/YWHAQ/KIF5B/PPP3R1/YWHAB/TP53BP2/SAR1A/MAPK14/RIOK2/EDEM1/GOLPH3/ACSL4/ARF6/FRMD4A/YWHAZ/CCT8/NPEPPS/CCT4/CCT7/FLNA/SAE1/PIK3R1/CSNK2A2/HDAC3/ADAM9/RHOU/DKC1/PRNP/CCT2/NMT1/GOLPH3L/TGFB3/EGFR/PPP1R13B/RAC1/BCL2/EDEM2/ERBB2/ANK3/IRS2/EXPH5/GTSE1 43

BP GO:0070203 regulation of establishment of protein localization to telomere 6/1105 11/18866 1.43E-05 0.001345151 0.001095102 CCT8/CCT4/CCT7/TERF1/DKC1/CCT2 6

BP GO:0051054 positive regulation of DNA metabolic process 28/1105 200/18866 1.62E-05 0.001504323 0.001224686 XRCC5/USP9X/MSH2/PRKDC/ACTR2/CCT8/CCT4/CCT7/NEK7/UBE2N/CDK2/NBN/CEBPG/PTGES3/DKC1/POT1/CCT2/MLH1/BRCA1/PML/EGFR/DTX3L/RFC5/PARP9/HMBOX1/ZNF711/FOXM1/WT1 28

BP GO:1902036 regulation of hematopoietic stem cell differentiation

15/1105 73/18866 1.75E-05 0.001572058 0.001279829 YAP1/EIF2AK2/PRKDC/PSMD12/PSMD10/PSMD1/TCF12/PSMF1/PSMD11/PSME4/CBFB/PSMD2/PSME3/PSMD3/RUNX1 15

BP GO:1903747 regulation of establishment of protein localization to mitochondrion 15/1105 73/18866 1.75E-05 0.001572058 0.001279829 SH3GLB1/YWHAQ/PPP3R1/YWHAB/TP53BP2/YWHAZ/NPEPPS/SAE1/CSNK2A2/RHOU/LMAN1/NMT1/PPP1R13B/BCL2/MAPT 15

BP GO:0006900 vesicle budding from membrane 19/1105 110/18866 1.97E-05 0.001732528 0.00141047 SEC23A/TMED10/TBC1D20/SAR1A/MCFD2/MIA3/ANKRD28/GOSR2/GOLPH3/SEC23IP/AP3B1/RAB1A/ARFGAP3/SEC22B/GBF1/LMAN1/GOLPH3L/NSF/TGFA 19

BP GO:0071559 response to transforming growth factor beta 33/1105 258/18866 2.04E-05 0.001732528 0.00141047 ROCK2/ADAM17/SPRED1/TGFBRAP1/USP9X/CRKL/FBN1/ACVR1/NR3C1/ITGB5/APAF1/SKIL/SMAD1/SNX6/COL4A2/BCL9L/PDE3A/ADAM9/TGFBR1/USP15/ZFP36L1/STRAP/TGFB3/PML/TWSG1/SOX5/IL17RD/SDCBP/LOX/FNDC4/SOX6/CGN/WNT5A 33

BP GO:0048199 vesicle targeting, to, from or within Golgi 15/1105 74/18866 2.08E-05 0.001732528 0.00141047 SEC23A/TMED10/TBC1D20/SAR1A/MCFD2/ANKRD28/GOSR2/SEC23I

P/RAB1A/ARFGAP3/SEC22B/GBF1/LMAN1/NSF/TGFA 15

BP GO:0043312 neutrophil degranulation 52/1105 487/18866 2.08E-05 0.001732528 0.00141047 RAB6A/VCL/TMEM30A/ATP11B/IQGAP1/CAND1/XRCC5/KCMF1/ADAM10/MAPK14/PAFAH1B2/ACTR2/CAP1/PSMD12/DNAJC3/NRAS/KPNB1/YPEL5/SNAP23/RAB10/COPB1/PSMD1/RAP1B/APAF1/CCT8/GDI2/RAB31/CANT1/NCSTN/PSMD11/ITGAV/TXNDC5/SCAMP1/PSMD2/ATG7/SURF4/CMTM6/CCT2/RAB3D/PSMD3/TUBB/MANBA/LAMP2/HSPA8/ARSB/RAC1/SDCBP/CD44/MGAM/SLC27A2/FABP5/PLAUR 52

BP GO:0006606 protein import into nucleus 23/1105 150/18866 2.09E-05 0.001732528 0.00141047 NUP98/IPO9/STAT3/NUP155/TNPO1/CSE1L/NOLC1/MAPK14/KPNA3/RAB23/PPP3CA/KPNB1/IPO7/KPNA4/FLNA/PIK3R1/CBLB/HDAC3/NUP93/NXT2/NUP107/PML/TNPO3 23

BP GO:0051170 import into nucleus 25/1105 171/18866 2.16E-05 0.001732528 0.00141047 NUP98/IPO9/GEMIN5/STAT3/NUP155/TNPO1/CSE1L/NOLC1/MAPK14/KPNA3/RAB23/PPP3CA/KPNB1/IPO7/KPNA4/FLNA/PIK3R1/CBLB/HDAC3/NUP93/NXT2/NUP107/PML/TNPO3/HTATIP2 25

BP GO:0031145 anaphase-promoting complex-dependent catabolic process 16/1105 83/18866 2.16E-05 0.001732528 0.00141047 CDC27/ANAPC1/PSMD12/PSMD10/PSMD1/PSMF1/PSMD11/CDK2/PS

ME4/PSMD2/PSME3/UBE2E1/PSMD3/CCNB1/ANAPC2/AURKA 16

BP GO:0009112 nucleobase metabolic process 10/1105 35/18866 2.18E-05 0.001732528 0.00141047 RRM1/GMPS/PPAT/PRPS1/GART/CTPS2/PAICS/DPYD/ALDH6A1/ADA 10

BP GO:0000077 DNA damage checkpoint 23/1105 151/18866 2.34E-05 0.001830705 0.001490397 EIF2AK4/CNOT8/MSH2/CNOT2/TAOK1/PRKDC/MAPK14/ATF2/RAD1/PTPN11/TIPRL/TOPBP1/CDK2/NBN/THOC5/BRCA1/PML/WDR76/RINT1/CCNB1/CHEK2/AURKA/GTSE1 23

BP GO:1903749 positive regulation of establishment of protein localization to mitochondrion 13/1105 58/18866 2.38E-05 0.001837966 0.001496308 SH3GLB1/YWHAQ/PPP3R1/YWHAB/TP53BP2/YWHAZ/NPEPPS/SAE1/CSNK2A2/RHOU/NMT1/PPP1R13B/BCL2 13

BP GO:0006517 protein deglycosylation 9/1105 29/18866 2.71E-05 0.002011231 0.001637365 MAN1A2/EDEM3/RNF103/SEL1L/EDEM1/OS9/RNF139/MAN2A1/EDEM2 9

BP GO:0019048 modulation by virus of host process 9/1105 29/18866 2.71E-05 0.002011231 0.001637365 EIF2AK4/EIF2AK2/BCL2L11/KPNA3/KPNB1/KPNA4/DAG1/RXRA/ATG7

9

BP GO:0070202 regulation of establishment of protein localization to chromosome 6/1105 12/18866 2.71E-05 0.002011231 0.001637365 CCT8/CCT4/CCT7/TERF1/DKC1/CCT2 6

BP GO:0043487 regulation of RNA stability 27/1105 195/18866 2.79E-05 0.002041387 0.001661915 ROCK2/YWHAB/TNPO1/MAPK14/PSMD12/YTHDF3/FXR1/DCP2/PSMD10/YWHAZ/PSMD1/PAIP1/PSMF1/KHSRP/PSMD11/EIF4G1/PSME4/NSUN2/DKC1/PSMD2/ZFP36L1/PSME3/PSMD3/MYD88/HSPA8/CIRBP/MYEF2 27

BP GO:0000082 G1/S transition of mitotic cell cycle 35/1105 287/18866 3.22E-05 0.002312268 0.001882442 ADAM17/ITGB1/LSM11/RPS6KB1/CNOT8/CNOT2/CUL4B/PRKDC/CDC73/SENP2/GSPT1/ACVR1/PPP3CA/CUL2/CUL1/PPAT/PRIM1/RBL1/CDK2/EIF4G1/RHOU/PSME3/PML/EGFR/BCL2/FAM107A/RCC1/CCNB1/RRM2/KIF14/CHEK2/ID4/AURKA/HYAL1/GTSE1 35

BP GO:0072665 protein localization to vacuole 14/1105 68/18866 3.24E-05 0.002312268 0.001882442 ROCK2/SH3GLB1/SCARB2/ZFYVE16/AP3M1/GOSR2/NEDD4/AP3B1/GNPTAB/VPS37A/M6PR/LAMP2/HSPA8/VPS37D 14

BP GO:0007160 cell-matrix adhesion 30/1105 230/18866 3.29E-05 0.002316698 0.001886048 ROCK2/MAP4K4/CLASP1/ITGB1/VCL/IQGAP1/NRP1/BCL2L11/NID1/NI

D2/ITGB5/DAG1/ECM2/PIK3R1/ITGAV/CASK/ADAM9/EPHA3/EMP2/ADAMTS9/FMN1/CORO1C/RAC1/BCL2/SERPINE1/FAM107A/CD44/CX3CL1/AJAP1/TSC1 30

BP GO:0010948 negative regulation of cell cycle process 41/1105 359/18866 3.36E-05 0.002336644 0.001902287 USP47/CNOT8/CNOT2/TAOK1/PRKDC/RNF2/CDC73/PSMD12/RAD1/PCGF6/CUL1/PSMD10/PSMD1/APAF1/TOPBP1/TERF1/PSMF1/PSMD11/RBL1/CDK2/NBN/PSME4/CBX5/PSMD2/ZFP36L1/FBXL7/PSME3/PSMD3/RBBP7/BRCA1/PML/RINT1/BCL2/FAM107A/CCNB1/RRM2/CHEK2/BUB1/TRIP13/AURKA/GTSE1 41

BP GO:0072666 establishment of protein localization to vacuole 12/1105 52/18866 3.57E-05 0.002455053 0.001998685 SCARB2/ZFYVE16/AP3M1/GOSR2/NEDD4/AP3B1/GNPTAB/VPS37A/M6PR/LAMP2/HSPA8/VPS37D 12

BP GO:0001844 protein insertion into mitochondrial membrane involved in apoptotic signaling pathway 9/1105 30/18866 3.66E-05 0.002487182 0.002024842 YWHAQ/PPP3R1/YWHAB/TP53BP2/BCL2L11/YWHAZ/NMT1/PPP1R13B/BCL2 9

BP GO:0001952 regulation of cell-matrix adhesion 20/1105 125/18866 3.81E-05 0.002555276 0.002080278 ROCK2/MAP4K4/CLASP1/VCL/IQGAP1/NRP1/DAG1/PIK3R1/CASK/EP

HA3/EMP2/FMN1/CORO1C/RAC1/BCL2/SERPINE1/FAM107A/CX3CL1/AJAP1/TSC1 20

BP GO:0060218 hematopoietic stem cell differentiation 16/1105 87/18866 3.97E-05 0.002632299 0.002142983 YAP1/EIF2AK2/XRCC5/PRKDC/PSMD12/PSMD10/PSMD1/TCF12/PSMF1/PSMD11/PSME4/CBFB/PSMD2/PSME3/PSMD3/RUNX1 16

BP GO:0046112 nucleobase biosynthetic process 7/1105 18/18866 4.17E-05 0.002697658 0.002196193 GMPS/PPAT/PRPS1/GART/CTPS2/PAICS/ADA 7

BP GO:0070200 establishment of protein localization to telomere 7/1105 18/18866 4.17E-05 0.002697658 0.002196193 CCT8/CCT4/CCT7/TERF1/DKC1/POT1/CCT2 7

BP GO:0017038 protein import 27/1105 200/18866 4.38E-05 0.002802879 0.002281854 NUP98/IPO9/STAT3/NUP155/TNPO1/CSE1L/NOLC1/PEX13/MAPK14/KPNA3/RAB23/PPP3CA/KPNB1/IPO7/KPNA4/RAB8B/FLNA/PIK3R1/CBLB/HDAC3/NUP93/NXT2/NUP107/PML/TNPO3/LAMP2/HSPA8 27

BP GO:0051650 establishment of vesicle localization 28/1105 212/18866 4.75E-05 0.002988104 0.002432647 CLASP1/SEC23A/KIF5B/TMED10/MYO1B/TBC1D20/SAR1A/MCFD2/MYO1E/AP3M1/ANKRD28/GOSR2/SEC23IP/SNAP23/AP3B1/EXOC4/MYO1D/RAB1A/ARFGAP3/SEC22B/GBF1/MYO5A/TBC1D23/LMAN1/NSF/IKBKG/TG

FA/LIN7A 28

BP GO:0044003 modulation by symbiont of host process 10/1105 38/18866 4.78E-05 0.002988104 0.002432647 EIF2AK4/EIF2AK2/BCL2L11/KPNA3/KPNB1/KPNA4/DAG1/RXRA/ATG7/ATG5 10

BP GO:0019058 viral life cycle 39/1105 341/18866 5.02E-05 0.003101926 0.002525312 ROCK2/NUP98/ITGB1/EIF2AK4/EIF2AK2/NUP155/SCARB2/TBC1D20/KPNA3/ADAR/NUP205/KPNB1/NEDD4/STAU1/ITGB5/SRPK1/SRPK2/DAG1/RAB1A/TRIM5/MORC2/ITGAV/IDE/RAB43/VPS37A/NR5A2/NUP93/NUP107/ATG5/PML/EGFR/OAS3/OAS2/HSPA8/BCL2/P4HB/VPS37D/NEDD4L/HYAL1 39

BP GO:1901988 negative regulation of cell cycle phase transition 33/1105 270/18866 5.12E-05 0.00313376 0.002551227 USP47/CNOT8/CNOT2/TAOK1/PRKDC/CDC73/PSMD12/CUL1/PSMD10/PSMD1/TOPBP1/PSMF1/PSMD11/RBL1/CDK2/NBN/PSME4/PSMD2/ZFP36L1/FBXL7/PSME3/PSMD3/BRCA1/PML/RINT1/BCL2/FAM107A/CCNB1/CHEK2/BUB1/TRIP13/AURKA/GTSE1 33

BP GO:1902750 negative regulation of cell cycle G2/M phase transition 18/1105 108/18866 5.29E-05 0.003197947 0.002603483 USP47/TAOK1/PSMD12/CUL1/PSMD10/PSMD1/TOPBP1/PSMF1/PSMD11/NBN/PSME4/PSMD2/FBXL7/PSME3/PSMD3/BRCA1/RINT1/AURKA 18

BP GO:0042590 antigen processing and presentation of exogenous peptide antigen via MHC class I 15/1105 80/18866 5.44E-05 0.003255211 0.002650102 PSMD12/SNAP23/LNPEP/ITGB5/PSMD10/PSMD1/PSMF1/PSMD11/ITGAV/SEC22B/PSME4/PSMD2/PSME3/PSMD3/IKBKG 15

BP GO:1903311 regulation of mRNA metabolic process 39/1105 344/18866 6.08E-05 0.00359983 0.00293066 ROCK2/VIRMA/NUP98/YWHAB/QKI/CNOT8/TNPO1/MAPK14/CDC73/PSMD12/YTHDF3/FXR1/DCP2/SART3/PSMD10/AHCYL1/YWHAZ/PSMD1/SRPK1/SRPK2/PAIP1/PSMF1/KHSRP/PSMD11/EIF4G1/PSME4/MBNL1/NCBP2/PSMD2/ZFP36L1/PSME3/RBM3/PSMD3/MYD88/HSPA8/SECISBP2/CIRBP/CCNB1/MYEF2 39

BP GO:0000723 telomere maintenance 23/1105 161/18866 6.55E-05 0.003797 0.003091179 XRCC5/PRKDC/DCLRE1B/DCP2/CCT8/CCT4/CCT7/NEK7/TERF1/PRIM1/POLD3/SMARCAL1/TFIP11/RAD51C/NBN/SP100/PTGES3/DKC1/POT1/CCT2/PML/RFC5/HMBOX1 23

BP GO:0031570 DNA integrity checkpoint 23/1105 161/18866 6.55E-05 0.003797 0.003091179 EIF2AK4/CNOT8/MSH2/CNOT2/TAOK1/PRKDC/MAPK14/ATF2/RAD1/PTPN11/TIPRL/TOPBP1/CDK2/NBN/THOC5/BRCA1/PML/WDR76/RINT1/CCNB1/CHEK2/AURKA/GTSE1 23

BP GO:0030198 extracellular matrix organization 43/1105 395/18866 6.78E-05 0.003849021 0.00313353 CLASP1/ITGB1/SH3PXD2A/ADAM10/LAMC1/FBN1/MYO1E/DDR2/SPARC/COL4A1/COL15A1/NID1/LAMA4/NID2/ITGB5/P4HA1/DAG1/ECM2/COL4A2/CAPN2/ITGAV/TGFBR1/COL12A1/CRTAP/SULF1/ADAMTS9/SULF2/SERPINE1/MMP19/LOX/COL4A4/CD44/NTN4/TMEM38B/CYP1B1/POMT1/TGFBI/ADAM12/LAMB3/WDR72/COL10A1/WT1/TIMP1 43

BP GO:0001558 regulation of cell growth 45/1105 420/18866 6.78E-05 0.003849021 0.00313353 BMPR2/ADAM17/USP47/ADNP/ADAM10/CRKL/EI24/CDKL5/CDC73/EIF4G2/NRP1/WDR36/RYK/ADIPOR1/MAP1B/PLAA/PSMD10/MTPN/CSNK2A1/RPS6KA3/EIF4G1/TGFBR1/NDRG3/RBBP7/PML/EGFR/SDCBP/SEMA4B/BCL2/FAM107A/AGTR1/ERBB2/LEF1/MAPT/SERPINE2/IGFBP1/SEMA3C/PPARG/ANAPC2/KIF14/NEDD4L/WNT5A/WT1/SPOCK1/HYAL1 45

BP GO:0050808 synapse organization 46/1105 433/18866 6.94E-05 0.003899688 0.003174778 ADNP/IL1RAP/ADAM10/MAPK14/CRKL/CDKL5/ACTR2/SSH1/NRP1/KALRN/PDLIM5/RYK/EGLN1/SPARC/COL4A1/NEDD4/ARF6/PLXND1/STAU1/MAP1B/PCDHGC3/CAST/YWHAZ/F2R/LRP4/DAG1/ITSN1/MYO5A/EIF4G1/ETV5/PRNP/GNPAT/TUBB/HSPA8/AFG3L2/CX3CL1/ERBB2/SRPX2/TSC1/MAPT/ANK3/ANAPC2/NRG1/WNT5A/NRXN2/LRP8 46

BP GO:0044843 cell cycle G1/S phase transition 36/1105 310/18866

7.03E-05 0.003900482 0.003175425 ADAM17/ITGB1/LSM11/RPS6KB1/CNOT8/CNOT2/CUL4B/PRKDC/CDC73/ATP2B4/SENP2/GSPT1/ACVR1/PPP3CA/CUL2/CUL1/PPAT/PRIM1/RBL1/CDK2/EIF4G1/RHOU/PSME3/PML/EGFR/BCL2/FAM107A/RCC1/CCNB1/RRM2/KIF14/CHEK2/ID4/AURKA/HYAL1/GTSE1 36

BP GO:0043062 extracellular structure organization 43/1105 396/18866 7.18E-05 0.003900482 0.003175425 CLASP1/ITGB1/SH3PXD2A/ADAM10/LAMC1/FBN1/MYO1E/DDR2/SPARC/COL4A1/COL15A1/NID1/LAMA4/NID2/ITGB5/P4HA1/DAG1/ECM2/COL4A2/CAPN2/ITGAV/TGFBR1/COL12A1/CRTAP/SULF1/ADAMTS9/SULF2/SERPINE1/MMP19/LOX/COL4A4/CD44/NTN4/TMEM38B/CYP1B1/POMT1/TGFBI/ADAM12/LAMB3/WDR72/COL10A1/WT1/TIMP1 43

BP GO:0010810 regulation of cell-substrate adhesion 28/1105 217/18866 7.20E-05 0.003900482 0.003175425 ROCK2/MAP4K4/CLASP1/VCL/IQGAP1/CRKL/NRP1/NID1/RSU1/FLNA/DAG1/ECM2/PIK3R1/CASK/EPHA3/EMP2/FMN1/CORO1C/FZD7/RAC1/BCL2/SERPINE1/FAM107A/CX3CL1/P4HB/AJAP1/TSC1/SPOCK1 28

BP GO:2001236 regulation of extrinsic apoptotic signaling pathway 23/1105 162/18866 7.22E-05 0.003900482 0.003175425 YAP1/PAK2/BCL10/NRP1/ACVR1/RB1CC1/GCLM/SKIL/ARHGEF2/RAF1/ITGAV/TGFBR1/SP100/PHIP/PSME3/RNF34/BRCA1/CYLD/PML/BCL2/SERPINE1/CX3CL1/SCG2 23

BP GO:0090114 COPII-coated vesicle budding 14/1105 73/18866 7.40E-05 0.003957967 0.003222223 SEC23A/TMED10/TBC1D20/SAR1A/MCFD2/MIA3/ANKRD28/GOSR2/SEC23IP/RAB1A/SEC22B/LMAN1/NSF/TGFA 14

BP GO:0071560 cellular response to transforming growth factor beta stimulus 31/1105 252/18866 7.57E-05 0.003996891 0.003253912 ADAM17/SPRED1/TGFBRAP1/USP9X/CRKL/FBN1/ACVR1/NR3C1/ITGB5/APAF1/SKIL/SMAD1/SNX6/COL4A2/BCL9L/PDE3A/ADAM9/TGFBR1/USP15/ZFP36L1/STRAP/TGFB3/PML/TWSG1/SOX5/IL17RD/SDCBP/LOX/SOX6/CGN/WNT5A 31

BP GO:0042493 response to drug 43/1105 397/18866 7.61E-05 0.003996891 0.003253912 ADAM17/TMEM30A/USP47/SRP72/STAT3/XRCC5/ADAM10/CRKL/GNA12/TOP1/PPP3CA/EFTUD2/MAP1B/GCLM/RAP1B/PAM/PPAT/TERF1/PDE3A/ADAM9/PRNP/GNPAT/ATG5/EGFR/LYN/PDE4B/ATP1A1/BCL2/LOX/CCNB1/AQP1/SEMA3C/ADA/PPARG/ACSL1/TDO2/CHEK2/SLC12A5/MT2A/LRP8/EMX2/SLAMF8/SLC22A12 43

BP GO:1904814 regulation of protein localization to chromosome, telomeric region 6/1105 14/18866 7.96E-05 0.004133136 0.00336483 CCT8/CCT4/CCT7/TERF1/DKC1/CCT2 6

BP GO:0016574 histone ubiquitination 11/110548/18866 8.06E-05 0.004133136 0.00336483

TRIP12/CUL4B/RNF2/CDC73/PCGF6/WDR70/UBE2N/UBE2A/UBE2E1/ATXN7L3/DTX3L 11

BP GO:1900739 regulation of protein insertion into mitochondrial membrane involved in apoptotic signaling pathway 8/1105 26/18866 8.17E-05 0.004133136 0.00336483 YWHAQ/PPP3R1/YWHAB/TP53BP2/YWHAZ/NMT1/PPP1R13B/BCL2 8

BP GO:1900740 positive regulation of protein insertion into mitochondrial membrane involved in apoptotic signaling pathway 8/1105 26/18866 8.17E-05 0.004133136 0.00336483 YWHAQ/PPP3R1/YWHAB/TP53BP2/YWHAZ/NMT1/PPP1R13B/BCL2 8

BP GO:0150115 cell-substrate junction organization 17/1105 102/18866 8.46E-05 0.004239028 0.003451038 ROCK2/MAP4K4/CLASP1/VCL/IQGAP1/LAMC1/NRP1/ARF6/EPHA3/DST/FMN1/CORO1C/RAC1/BCL2/FAM107A/TSC1/LAMB3 17

BP GO:0061013 regulation of mRNA catabolic process 27/1105 208/18866 8.68E-05 0.004314361 0.003512368 ROCK2/YWHAB/CNOT8/TNPO1/MAPK14/PSMD12/YTHDF3/FXR1/DCP2/PSMD10/YWHAZ/PSMD1/PAIP1/PSMF1/KHSRP/PSMD11/EIF4G1/PSME4/PSMD2/ZFP36L1/PSME3/PSMD3/MYD88/HSPA8/SECISBP2/CIRBP/MYEF2 27

BP GO:0043488 regulation of mRNA stability 25/1105 186/18866 8.90E-05 0.004363898 0.003552696

ROCK2/YWHAB/TNPO1/MAPK14/PSMD12/YTHDF3/FXR1/DCP2/PSMD10/YWHAZ/PSMD1/PAIP1/PSMF1/KHSRP/PSMD11/EIF4G1/PSME4/PSMD2/ZFP36L1/PSME3/PSMD3/MYD88/HSPA8/CIRBP/MYEF2 25

BP GO:0006144 purine nucleobase metabolic process 7/1105 20/18866 9.16E-05 0.004363898 0.003552696 GMPS/PPAT/PRPS1/GART/PAICS/DPYD/ADA 7

BP GO:0008356 asymmetric cell division 7/1105 20/18866 9.16E-05 0.004363898 0.003552696 ACTR2/ACTR3/RAB10/DOCK7/ARHGEF2/ETV5/SOX5 7

BP GO:0046931 pore complex assembly 7/1105 20/18866 9.16E-05 0.004363898 0.003552696 NUP98/ADAM10/AHCTF1/NUP205/CCT8/NUP93/NUP107 7

BP GO:0007163 establishment or maintenance of cell polarity 28/1105 220/18866 9.17E-05 0.004363898 0.003552696 PARVA/ROCK2/CLASP1/ITGB1/MSN/CRKL/ACTR2/CAP1/MAP4/CKAP5/ACTR3/GOLPH3/ARF6/RAB10/DOCK7/MAP1B/FRMD4A/RAP1B/ARHGEF2/ARFGEF1/GBF1/DST/RAC1/MAP7/LIN7A/AQP1/WNT5A/KIF2C 28

BP GO:0070534 protein K63-linked ubiquitination 12/1105 57/18866 9.34E-05 0.004404895 0.003586073 TRIP12/UBE2G1/NEDD4/RNF4/PLAA/UBE2V2/UBE2N/BFAR/TRIM5/BIRC2/UBE2O/RNF152 12

BP GO:0019882 antigen processing and presentation 29/1105

232/18866 9.58E-05 0.004481643 0.003648554
RAB6A/SEC23A/PSMD12/DCTN5/DCTN4/SNAP23/CLTC/RAB10/AP3B1/RAB8B/LNPEP/ITGB5/PSMD10/PSMD1/KIF3C/CANX/PSMF1/PSMD11/ITGAV/IDE/SEC22B/PSME4/PSMD2/PSME3/HFE/ATG5/PSMD3/IKBKG/KIF2C
29

BP GO:0048207 vesicle targeting, rough ER to cis-Golgi 13/1105
66/18866 9.97E-05 0.00454629 0.003701183
SEC23A/TMED10/TBC1D20/SAR1A/MCFD2/ANKRD28/GOSR2/SEC23IP/RAB1A/SEC22B/LMAN1/NSF/TGFA 13

BP GO:0048208 COPII vesicle coating 13/1105 66/18866 9.97E-05
0.00454629 0.003701183
SEC23A/TMED10/TBC1D20/SAR1A/MCFD2/ANKRD28/GOSR2/SEC23IP/RAB1A/SEC22B/LMAN1/NSF/TGFA 13

BP GO:0150116 regulation of cell-substrate junction organization
13/1105 66/18866 9.97E-05 0.00454629 0.003701183
ROCK2/MAP4K4/CLASP1/VCL/IQGAP1/NRP1/ARF6/EPHA3/FMN1/CORO1C/RAC1/FAM107A/TSC1 13

BP GO:0002479 antigen processing and presentation of exogenous peptide antigen via MHC class I, TAP-dependent 14/1105 75/18866
0.000100553 0.004549415 0.003703728
PSMD12/SNAP23/ITGB5/PSMD10/PSMD1/PSMF1/PSMD11/ITGAV/SEC22B/PSME4/PSMD2/PSME3/PSMD3/IKBKG 14

BP GO:0051052 regulation of DNA metabolic process 40/1105 365/18866 0.000103886 0.004662283 0.003795615 BMPR2/MSH6/TRIP12/XRCC5/USP9X/MSH2/PRKDC/ACTR2/DCP2/ZRANB3/APAF1/CCT8/WDR70/CCT4/UBE2V2/CCT7/NEK7/TERF1/UBE2N/TFIP11/CDK2/UBQLN4/NBN/CEBPG/PTGES3/DKC1/POT1/CCT2/MLH1/BRCA1/PML/EGFR/DTX3L/RFC5/PARP9/HMBOX1/ZNF711/PPARG/FOXM1/WT1 40

BP GO:0022612 gland morphogenesis 19/1105 124/18866 0.00010747 0.004724932 0.003846618 MSN/NRP1/NOTCH2/PLXND1/DAG1/RXRA/ETV5/SULF1/TGFB3/PML/EGFR/TWSG1/SULF2/BCL2/NTN4/SEMA3C/CEBPB/WNT5A/ID4 19

BP GO:0045930 negative regulation of mitotic cell cycle 38/1105 341/18866 0.000108183 0.004724932 0.003846618 USP47/CNOT8/MSH2/CNOT2/TAOK1/PRKDC/CDC73/PSMD12/DCLRE1B/ATF2/CUL1/PSMD10/PSMD1/TOPBP1/PSMF1/PSMD11/RBL1/CDK2/NBN/PSME4/PSMD2/ZFP36L1/FBXL7/PSME3/PSMD3/BRCA1/PML/EGFR/RINT1/BCL2/FAM107A/CCNB1/PTPN3/CHEK2/BUB1/TRIP13/AURKA/GTSE138

BP GO:0006892 post-Golgi vesicle-mediated transport 17/1105 104/18866 0.000108409 0.004724932 0.003846618 KLHL20/MYO1B/GOSR2/GOLPH3/SNAP23/RAB10/EXOC4/RAB31/GBF1/MYO5A/RABIF/RP2/SCAMP1/GOLPH3L/NSF/ANK3/CCDC91 17

BP GO:0032212 positive regulation of telomere maintenance via telomerase

9/1105 34/18866 0.000108678 0.004724932 0.003846618 XRCC5/CCT8/CCT4/CCT7/NEK7/DKC1/POT1/CCT2/HMBOX1 9

BP GO:0001738 morphogenesis of a polarized epithelium 21/1105 145/18866 0.000109769 0.004735375 0.00385512 MSN/PSMD12/RYK/CLTC/RAB10/PSMD10/PSMD1/PSMF1/PSMD11/PSME4/PSMD2/PSME3/PSMD3/FZD7/RAC1/ZDHHC7/AJAP1/ROR2/CTHRC1/ASTN2/WNT5A 21

BP GO:0006913 nucleocytoplasmic transport 39/1105 354/18866 0.000112766 0.004827232 0.003929902 AKAP13/NUP98/IPO9/PTPN14/GEMIN5/STAT3/NUP155/TNPO1/CSE1L/NOLC1/MAPK14/RIOK2/KPNA3/RAB23/NUP205/PPP3CA/KPNB1/NEDD4/IPO7/KPNA4/PTPN11/AHCYL1/FLNA/PIK3R1/PPP1CC/CBLB/HDAC3/THOC5/SP100/NCBP2/NSUN2/NUP93/NXT2/NUP107/PML/TNPO3/TSC1/HTATIP2/GTSE1 39

BP GO:0033866 nucleoside bisphosphate biosynthetic process 13/1105 67/18866 0.000117116 0.004900398 0.003989467 SLC26A2/PANK2/PAPSS1/PANK3/ACSL4/ELOVL5/ELOVL1/CBR4/PANK1/ELOVL3/ACSL1/HSD17B8/ACSS1 13

BP GO:0034030 ribonucleoside bisphosphate biosynthetic process 13/1105 67/18866 0.000117116 0.004900398 0.003989467 SLC26A2/PANK2/PAPSS1/PANK3/ACSL4/ELOVL5/ELOVL1/CBR4/PANK1/ELOVL3/ACSL1/HSD17B8/ACSS1 13

BP GO:0034033 purine nucleoside bisphosphate biosynthetic process 13/1105 67/18866 0.000117116 0.004900398 0.003989467 SLC26A2/PANK2/PAPSS1/PANK3/ACSL4/ELOVL5/ELOVL1/CBR4/PANK1/ELOVL3/ACSL1/HSD17B8/ACSS1 13

BP GO:0007409 axonogenesis 49/1105 482/18866 0.000121969 0.005065364 0.004123768 BMPR2/ITGB1/VCL/RAPH1/KIF5B/ADNP/FEZ2/USP9X/ROBO1/CDKL5/PAK2/NRP1/WDR36/NOTCH2/SPAST/RYK/MAP2K1/PLXND1/RAB10/DOCK7/MAP1B/PTPN11/SKIL/TRIO/LRP4/DAG1/PIK3R1/EXT1/UNC5C/NOTCH3/EPHA3/ENAH/PALLD/GRB2/RAC1/STXBP1/AFG3L2/SEMA4B/BCL2/NTN4/ERBB2/MAPT/ANK3/IRS2/SEMA3C/ANAPC2/WNT5A/PLXNB3/UNC5D 49

BP GO:0006605 protein targeting 46/1105 444/18866 0.000125648 0.005157973 0.004199162 SH3GLB1/YWHAQ/SRP72/YWHAB/SCARB2/USP9X/ZDHHC20/PEX13/ZFYVE16/AP4E1/SEC63/AP3M1/GOSR2/OS9/NEDD4/AP3B1/SSR1/RAB8B/EXOC4/YWHAZ/SGTB/NPEPPS/SAE1/GNPTAB/CSNK2A2/IDE/VPS37A/SPCS3/M6PR/RHOU/LMAN1/PRNP/GNPAT/LAMP2/HSPA8/SDCBP/RPS23/ZDHHC7/RAB3IP/ERBB2/ANK3/VPS37D/HMGCL/SLC27A2/PMPCA/HAO2 46

BP GO:0071474 cellular hyperosmotic response 6/1105 15/18866 0.000126053 0.005157973 0.004199162 XRCC5/ARHGEF2/MAPK10/SLC25A23/AQP1/EFHD1 6

BP GO:1901532 regulation of hematopoietic progenitor cell differentiation

15/1105 86/18866 0.000128509 0.005220101 0.00424974 YAP1/EIF2AK2/PRKDC/PSMD12/PSMD10/PSMD1/TCF12/PSMF1/PSMD11/PSME4/CBFB/PSMD2/PSME3/PSMD3/RUNX1 15

BP GO:0018210 peptidyl-threonine modification 19/1105 126/18866 0.000133584 0.005320172 0.004331209 ROCK2/SPRED1/HIPK1/PRKDC/WNK1/ACVR1/MAP2K1/GALNT1/CSNK2A1/CSNK2A2/TBK1/EIF4G1/TGFBR1/GALNT2/BCL2/STK39/CDC42BPG/WNT5A/CIT 19

BP GO:0098722 asymmetric stem cell division 5/1105 10/18866 0.00013435 0.005320172 0.004331209 RAB10/DOCK7/ARHGEF2/ETV5/SOX5 5

BP GO:1904851 positive regulation of establishment of protein localization to telomere 5/1105 10/18866 0.00013435 0.005320172 0.004331209 CCT8/CCT4/CCT7/DKC1/CCT2 5

BP GO:0051169 nuclear transport 39/1105 357/18866 0.000134797 0.005320172 0.004331209 AKAP13/NUP98/IPO9/PTPN14/GEMIN5/STAT3/NUP155/TNPO1/CSE1L/NOLC1/MAPK14/RIOK2/KPNA3/RAB23/NUP205/PPP3CA/KPNB1/NEDD4/IPO7/KPNA4/PTPN11/AHCYL1/FLNA/PIK3R1/PPP1CC/CBLB/HDAC3/THOC5/SP100/NCBP2/NSUN2/NUP93/NXT2/NUP107/PML/TNPO3/TSC1/HTATIP2/GTSE1 39

BP GO:0032206 positive regulation of telomere maintenance 11/1105

51/18866 0.000144487 0.005662481 0.004609887 XRCC5/CCT8/CCT4/CCT7/NEK7/NBN/DKC1/POT1/CCT2/PML/HMBOX1 11

BP GO:2001020 regulation of response to DNA damage stimulus 28/1105 226/18866 0.00014626 0.005691884 0.004633824 SPRED1/TRIP12/USP47/PRKDC/ACTR2/FXR1/PSMD10/SKIL/WDR70/UBE2V2/UBE2N/TFIP11/UBQLN4/CEBPG/THOC5/POT1/BRCA1/PML/EGFR/WDR76/DTX3L/PARP9/RINT1/BCL2/CD44/MAPT/FOXM1/CHEK2 28

BP GO:0061462 protein localization to lysosome 10/1105 43/18866 0.000148371 0.005712963 0.004650984 ROCK2/SCARB2/ZFYVE16/AP3M1/NEDD4/AP3B1/GNPTAB/M6PR/LAMP2/HSPA8 10

BP GO:0022604 regulation of cell morphogenesis 50/1105 499/18866 0.000148855 0.005712963 0.004650984 PARVA/BMPR2/MSN/PRPF40A/ADNP/ADAM10/PRKDC/ZMYM4/CRKL/ROBO1/CDKL5/PAK2/ACTR2/NRP1/GNA12/KALRN/WDR36/PDLIM5/GNA13/RYK/MAP2K1/SPARC/PPP3CA/NEDD4/PLXND1/MAP1B/PLAA/SKIL/LRP4/FLNA/DAG1/CDC42SE1/BCL9L/P2RY1/FBXW8/PHIP/RHOU/CORO1C/RAC1/SEMA4B/CD44/P4HB/CCL13/MAPT/SEMA3C/ANAPC2/NEDD4L/WNT5A/PLXNB3/LRP8 50

BP GO:2001237 negative regulation of extrinsic apoptotic signaling pathway 17/1105 107/18866 0.000155241 0.005917218 0.004817271

YAP1/NRP1/ACVR1/RB1CC1/GCLM/ARHGEF2/RAF1/ITGAV/TGFBR1/PHIP/PSME3/RNF34/BRCA1/BCL2/SERPINE1/CX3CL1/SCG2 17

BP GO:0045927 positive regulation of growth 32/1105 274/18866 0.000156468 0.005923436 0.004822333 BMPR2/ADAM17/YAP1/USP47/ADNP/ADAM10/PRKDC/MAPK14/CDKL5/EIF4G2/NRP1/MAP1B/PLAA/PSMD10/MTPN/CSNK2A1/RPS6KA3/EIF4G1/TGFBR1/ZFPM2/EGFR/SDCBP/BCL2/ERBB2/LEF1/CCNB1/MAPT/IGFBP1/ANAPC2/NEDD4L/WT1/HYAL132

BP GO:0006513 protein monoubiquitination 13/1105 69/18866 0.000160038 0.005999802 0.004884503 CUL4B/RNF2/CDC73/NEDD4/PCGF6/CUL1/BIRC2/RAD18/UBE2O/UBE2E1/ATXN7L3/DTX3L/NEDD4L 13

BP GO:0070997 neuron death 39/1105 360/18866 0.000160642 0.005999802 0.004884503 BACE1/ADNP/STAT3/BTBD10/MSH2/TP53BP2/SSH1/NRP1/BCL2L11/ATF2/EGLN1/GCLM/APAF1/STAMBP/F2R/SRPK2/SNX6/NCSTN/TBK1/ITSN1/CAPN2/EIF4G1/ATG7/PRNP/TGFB3/DIABLO/PPP1R13B/IKBKG/STXBP1/ZNF746/BCL2/CX3CL1/TSC1/MAPT/KMO/KIF14/CEBPB/WNT5A/CIT 39

BP GO:0009895 negative regulation of catabolic process 36/1105 323/18866 0.000161842 0.006004331 0.004888191 SF3B3/STAT3/FEZ2/SMARCC1/SENP1/MAPK14/EIF4G2/UBE2J1/ATP2B4/OS9/AZIN1/FLNA/CSNK2A1/PAIP1/CSNK2A2/PSMF1/SEC22B/UBQLN4/

EIF4G1/NSUN2/DKC1/ATG7/HFE/PML/EGFR/MYD88/SDCBP/ZDHHC7/SECISBP2/BCL2/CIRBP/TSC1/SERPINE2/PTPN3/NRG1/TIMP1 36

BP GO:0070646 protein modification by small protein removal 34/1105 300/18866 0.000177051 0.006525076 0.005312135 USP13/USP47/USP9X/SENP1/JOSD1/PSMD12/USP32/SENP2/TRRAP/SART3/PSMD10/PSMD1/STAMBP/VCPIP1/SMAD1/BIRC2/PSMF1/PSMD11/COPS8/PSME4/RHOT1/TGFBR1/USP15/PSMD2/PSME3/COPS3/ATXN7L3/PSMD3/BRCA1/CYLD/USP54/USP18/IKBKG/SPATA2 34

BP GO:0051648 vesicle localization 28/1105 229/18866 0.000183124 0.006696114 0.005451378 CLASP1/SEC23A/KIF5B/TMED10/MYO1B/TBC1D20/SAR1A/MCFD2/MYO1E/AP3M1/ANKRD28/GOSR2/SEC23IP/SNAP23/AP3B1/EXOC4/MYO1D/RAB1A/ARFGAP3/SEC22B/GBF1/MYO5A/TBC1D23/LMAN1/NSF/IKBKG/TGFA/LIN7A 28

BP GO:0033619 membrane protein proteolysis 12/1105 61/18866 0.000185301 0.006696114 0.005451378 ADAM17/BACE1/ADAM10/RHBDD1/MBTPS2/DAG1/NCSTN/ADAM9/AFG3L2/HM13/PTPN3/TIMP1 12

BP GO:1902110 positive regulation of mitochondrial membrane permeability involved in apoptotic process 12/1105 61/18866 0.000185301 0.006696114 0.005451378 YWHAQ/PPP3R1/YWHAB/TP53BP2/BCL2L11/ATF2/YWHAZ/RHOT1/NM

T1/PPP1R13B/BCL2/ACAA2 12

BP GO:0019511 peptidyl-proline hydroxylation 6/1105 16/18866 0.00019171 0.006883016 0.005603537 EGLN1/OGFOD1/P4HA1/CRTAP/P4HA3/P4HB 6

BP GO:0071496 cellular response to external stimulus 36/1105 326/18866 0.000194321 0.006932043 0.005643451 BMPR2/SH3GLB1/EIF2AK4/SLC38A2/EIF2AK2/ADNP/NUAK1/TRIM24/BCL10/MAP3K2/EIF2AK3/PTPN11/MTPN/DAG1/ATP2B1/EIF4G1/ATG7/HFE/MAP1LC3B/ATG5/EGFR/LAMP2/LYN/MYD88/HSPA8/RAC1/ATP1A1/BCL2/FAM107A/HABP4/RNF152/TSC1/AQP1/PPARG/GLRX2/CASP536

BP GO:0007044 cell-substrate junction assembly 16/1105 99/18866 0.00019598 0.006943358 0.005652662 ROCK2/MAP4K4/CLASP1/VCL/IQGAP1/LAMC1/NRP1/EPHA3/DST/FMN1/CORO1C/RAC1/BCL2/FAM107A/TSC1/LAMB3 16

BP GO:0097191 extrinsic apoptotic signaling pathway 28/1105 230/18866 0.000197134 0.006943358 0.005652662 YAP1/HIPK1/PAK2/BCL10/NRP1/BCL2L11/ACVR1/RB1CC1/GCLM/SKIL/ARHGEF2/PIK3R1/RAF1/ITGAV/TGFBR1/SP100/PHIP/PSME3/RNF34/BRCA1/CYLD/DIABLO/PML/BCL2/SERPINE1/CX3CL1/MLLT11/SCG2 28

BP GO:0009165 nucleotide biosynthetic process 31/1105 266/18866 0.000204474 0.007156574 0.005826244 SLC26A2/PANK2/PAPSS1/STAT3/ADCY3/PANK3/RRM1/ACSL4/GUCY1

A2/GMPS/PPAT/PRPS1/GART/CTPS2/RRM2B/PAICS/ELOVL5/ELOVL1/PARP9/CBR4/ADCY5/PANK1/ELOVL3/KMO/ADA/RRM2/UCK1/ACSL1/SLC5A8/HSD17B8/ACSS1 31

BP GO:0000075 cell cycle checkpoint 27/1105 219/18866 0.000206674 0.00717883 0.005844362 EIF2AK4/CNOT8/MSH2/CNOT2/TAOK1/PRKDC/MAPK14/DCLRE1B/ATF2/RAD1/PTPN11/TIPRL/TOPBP1/CDK2/NBN/THOC5/NSUN2/BRCA1/PML/WDR76/RINT1/CCNB1/CHEK2/BUB1/TRIP13/AURKA/GTSE1 27

BP GO:0032210 regulation of telomere maintenance via telomerase 11/110553/18866 0.000207689 0.00717883 0.005844362 XRCC5/DCP2/CCT8/CCT4/CCT7/NEK7/TERF1/DKC1/POT1/CCT2/HMBOX1 11

BP GO:1990778 protein localization to cell periphery 37/1105 340/18866 0.000213867 0.007279357 0.005926202 ROCK2/ITGB1/SEC23A/KIF5B/ADAM10/ATP2B4/GOLPH3/ARF6/CLTC/RAB10/RAB8B/RAB31/FLNA/DAG1/ARL13B/PIK3R1/P2RY1/MYO5A/PRNP/EPHA3/EMP2/RAB3D/GOLPH3L/LDLRAP1/EGFR/NSF/STXBP1/ZDHHC7/MAPK10/TMEM88/MAP7/TUB/ANK3/LIN7A/STX3/WDR72/ARHGEF16 37

BP GO:0032200 telomere organization 23/1105 174/18866 0.000215246 0.007279357 0.005926202 XRCC5/PRKDC/DCLRE1B/DCP2/CCT8/CCT4/CCT7/NEK7/TERF1/PRIM1/POLD3/SMARCAL1/TFIP11/RAD51C/NBN/SP100/PTGES3/DKC1/POT1/C

CT2/PML/RFC5/HMBOX1 23

BP GO:0007029 endoplasmic reticulum organization 13/1105 71/18866 0.00021583 0.007279357 0.005926202 RAB3GAP1/MIA3/REEP3/TOR1B/EIF2AK3/RAB10/VCPIP1/MYO5A/LMAN1/TOR1AIP2/LMAN2L/SMPD4/TMEM38B 13

BP GO:0033627 cell adhesion mediated by integrin 13/1105 71/18866 0.00021583 0.007279357 0.005926202 ADAM17/ITGB1/CRKL/FBN1/WNK1/ITGB5/PTPN11/ITGAV/ADAM9/LYN/SERPINE1/CYP1B1/ADA 13

BP GO:1904358 positive regulation of telomere maintenance via telomere lengthening 9/1105 37/18866 0.000219932 0.007373009 0.006002446 XRCC5/CCT8/CCT4/CCT7/NEK7/DKC1/POT1/CCT2/HMBOX1 9

BP GO:0051222 positive regulation of protein transport 38/1105 354/18866 0.000233388 0.007719343 0.0062844 SH3GLB1/TMEM30A/YWHAQ/KIF5B/PPP3R1/YWHAB/TP53BP2/SAR1A/MAPK14/RIOK2/EDEM1/GOLPH3/ACSL4/ARF6/FRMD4A/YWHAZ/NPEPPS/FLNA/SAE1/PIK3R1/CSNK2A2/HDAC3/ADAM9/RHOU/PRNP/NMT1/GOLPH3L/TGFB3/EGFR/PPP1R13B/RAC1/BCL2/EDEM2/ERBB2/ANK3/IRS2/EXPH5/GTSE1 38

BP GO:1904869 regulation of protein localization to Cajal body 5/1105 11/18866 0.000234424 0.007719343 0.0062844 CCT8/CCT4/CCT7/DKC1/CCT2 5

BP GO:1904871 positive regulation of protein localization to Cajal body 5/1105 11/18866 0.000234424 0.007719343 0.0062844 CCT8/CCT4/CCT7/DKC1/CCT2 5

BP GO:0009101 glycoprotein biosynthetic process 37/1105 342/18866 0.000240281 0.007865685 0.006403538 BMPR2/DPY19L3/EXT2/MAN1A2/EDEM3/FAM20B/NDST1/CHSY1/MGAT5/HS2ST1/MCFD2/UBE2J1/TMTC3/GOLPH3/GFPT1/GALNT1/FUT4/STT3A/ST3GAL2/CANT1/NCSTN/ARFGEF1/EXT1/LMAN1/PGM3/STT3B/MAN2A1/B4GALT3/DPM1/ST3GAL1/GALNT2/GALNT10/DPAGT1/PHLDA1/SLC35D1/BCL2/POMT1 37

BP GO:0070198 protein localization to chromosome, telomeric region 8/1105 30/18866 0.00024824 0.0080104 0.006521353 XRCC5/CCT8/CCT4/CCT7/TERF1/DKC1/POT1/CCT2 8

BP GO:0071711 basement membrane organization 8/1105 30/18866 0.00024824 0.0080104 0.006521353 CLASP1/ITGB1/COL4A1/NID1/NID2/DAG1/NTN4/LAMB3 8

BP GO:1901293 nucleoside phosphate biosynthetic process 31/1105 269/18866 0.000249771 0.0080104 0.006521353 SLC26A2/PANK2/PAPSS1/STAT3/ADCY3/PANK3/RRM1/ACSL4/GUCY1A2/GMPS/PPAT/PRPS1/GART/CTPS2/RRM2B/PAICS/ELOVL5/ELOVL1/PARP9/CBR4/ADCY5/PANK1/ELOVL3/KMO/ADA/RRM2/UCK1/ACSL1/SLC5A8/HSD17B8/ACSS1 31

BP GO:0043547 positive regulation of GTPase activity 42/1105 406/18866 0.000250932 0.0080104 0.006521353 MAP4K4/ITGB1/RAB3GAP1/BNIP2/SEC23A/IQGAP1/TBC1D2B/STARD13/SIPA1L2/TBC1D20/CRKL/CDKL5/CHML/NRP1/KALRN/ARHGAP21/RSU1/EVI5/DOCK7/GDI2/F2R/ARFGEF1/ARFGAP3/OCRL/RASA3/RP2/ARHGEF6/ARHGAP20/STXBP5/CORO1C/TBC1D22A/GARNL3/ARHGAP24/CX3CL1/ERBB2/CCL13/TSC1/RIN1/CCL26/WNT5A/ARHGEF10L/ARHGEF16 42

BP GO:0051893 regulation of focal adhesion assembly 12/1105 63/18866 0.000254778 0.0080104 0.006521353 ROCK2/MAP4K4/CLASP1/VCL/IQGAP1/NRP1/EPHA3/FMN1/CORO1C/RAC1/FAM107A/TSC1 12

BP GO:0090109 regulation of cell-substrate junction assembly 12/1105 63/18866 0.000254778 0.0080104 0.006521353 ROCK2/MAP4K4/CLASP1/VCL/IQGAP1/NRP1/EPHA3/FMN1/CORO1C/RAC1/FAM107A/TSC1 12

BP GO:1902686 mitochondrial outer membrane permeabilization involved in programmed cell death 12/1105 63/18866 0.000254778 0.0080104 0.006521353 YWHAQ/PPP3R1/YWHAB/TP53BP2/BCL2L11/ATF2/YWHAZ/RHOT1/NMT1/PPP1R13B/BCL2/ACAA2 12

BP GO:0071214 cellular response to abiotic stimulus 36/1105 331/18866 0.000261613 0.008133396 0.006621485

METAP2/YAP1/EIF2AK4/SLC38A2/USP47/XRCC5/CUL4B/MAPK14/BCL10/MAP3K2/NEDD4/RHBDD1/RAD1/PTPN11/MTPN/DAG1/ARHGEF2/PIK3R1/POLD3/ZFP36L1/NMT1/EGFR/MYD88/GRB2/RAC1/ATP1A1/CRY2/HABP4/STK39/MAPK10/SLC25A23/AQP1/EFHD1/CHEK2/CASP5/HYAL1 36

BP GO:0104004 cellular response to environmental stimulus 36/1105 331/18866 0.000261613 0.008133396 0.006621485 METAP2/YAP1/EIF2AK4/SLC38A2/USP47/XRCC5/CUL4B/MAPK14/BCL10/MAP3K2/NEDD4/RHBDD1/RAD1/PTPN11/MTPN/DAG1/ARHGEF2/PIK3R1/POLD3/ZFP36L1/NMT1/EGFR/MYD88/GRB2/RAC1/ATP1A1/CRY2/HABP4/STK39/MAPK10/SLC25A23/AQP1/EFHD1/CHEK2/CASP5/HYAL1 36

BP GO:0050769 positive regulation of neurogenesis 48/1105 485/18866 0.000266894 0.008251472 0.006717612 BMPR2/TMEM30A/IL6ST/IQGAP1/ADNP/ABL2/XRCC5/SCARB2/CRKL/ROBO1/CDKL5/ACTR2/NRP1/KALRN/MAP2K1/CREB3L2/PLXND1/HDAC1/MAP1B/PLAA/TCF12/ATF1/SKIL/FLNA/DAG1/ARHGEF2/ENC1/ITSN1/PPP1CC/EIF4G1/FBXW8/ETV5/EPHA3/MAN2A1/LYN/ARSB/BCL2/CYB5D2/CX3CL1/MAPT/ROR2/SERPINE2/PPARG/ANAPC2/NEDD4L/WNT5A/PLXNB3/LRP8 48

BP GO:0006997 nucleus organization 19/1105 133/18866 0.000274141 0.008382403 0.006824205 NUP98/NUP155/NOLC1/AHCTF1/REEP3/SPAST/NUP205/TOR1B/SRPK1/SRPK2/PARP11/TMEM43/PSME4/NUP93/NUP107/PML/HABP4/CCNB1/C

CNB2 19

BP GO:0042770 signal transduction in response to DNA damage 19/1105 133/18866 0.000274141 0.008382403 0.006824205 SPRED1/CNOT8/CNOT2/PRKDC/MAPK14/PSMD10/CDK2/NBN/SP100/BRCA1/PML/GRB2/RINT1/CD44/CCNB1/FOXM1/CHEK2/AURKA/GTSE1 19

BP GO:0002218 activation of innate immune response 20/1105 144/18866 0.000280889 0.008477495 0.00690162 XRCC5/PRKDC/PAK2/BCL10/PSMD12/NRAS/CUL1/PSMD10/PSMD1/TBK1/TRIM5/PSMF1/RAF1/PSMD11/PSME4/PSMD2/PSME3/PSMD3/LYN/IKBKG 20

BP GO:1904380 endoplasmic reticulum mannose trimming 6/1105 17/18866 0.000281644 0.008477495 0.00690162 RNF103/SEL1L/EDEM1/OS9/RNF139/EDEM2 6

BP GO:0007015 actin filament organization 44/1105 434/18866 0.000281822 0.008477495 0.00690162 ROCK2/CLASP1/MYO1B/PAK2/ACTR2/SSH1/NRP1/MYO1E/CAP1/ACTR3/ARF6/TPM3/ITGB5/MTPN/ABI1/FLNA/ARHGEF2/MICAL2/MYO1D/PIK3R1/ARFGEF1/MYO5A/TGFBR1/ENAH/EMP2/TGFB3/PLEKHG2/FMN1/CORO1C/PLS3/GRB2/ELMO2/RAC1/SORBS2/BCL2/FAM107A/CX3CL1/TSC1/GBA2/ARPC3/PLEKHH2/CCL26/ARHGEF10L/ARPC1B 44

BP GO:0097345 mitochondrial outer membrane permeabilization 11/110555/18866 0.000292844 0.008761692 0.007132987

YWHAQ/PPP3R1/YWHAB/TP53BP2/BCL2L11/YWHAZ/RHOT1/NMT1/PPP1R13B/BCL2/ACAA2 11

BP GO:1901214 regulation of neuron death 35/1105 321/18866 0.000301803 0.008981476 0.007311917 BACE1/ADNP/STAT3/BTBD10/MSH2/TP53BP2/SSH1/NRP1/BCL2L11/ATF2/EGLN1/GCLM/STAMBP/F2R/SRPK2/SNX6/TBK1/ITSN1/CAPN2/EIF4G1/ATG7/PRNP/TGFB3/PPP1R13B/IKBKG/STXBP1/ZNF746/BCL2/CX3CL1/TSC1/MAPT/KMO/KIF14/CEBPB/WNT5A 35

BP GO:0007178 transmembrane receptor protein serine/threonine kinase signaling pathway 38/1105 359/18866 0.000308929 0.009144631 0.007444743 BMPR2/ADAM17/SPRED1/TGFBRAP1/PELO/USP9X/MAPK14/FBN1/ZFYVE16/NOTCH2/ACVR1/LNPEP/ITGB5/SKIL/SMAD1/SNX6/BCL9L/ADAM9/TGFBR1/USP15/STRAP/RGMB/UBE2O/SULF1/NUP93/HFE/TGFB3/PML/SLC33A1/TWSG1/IL17RD/SDCBP/LOX/LEF1/CGN/ROR2/WNT5A/ATOH8 38

BP GO:0070498 interleukin-1-mediated signaling pathway 16/1105 103/18866 0.000312548 0.009201759 0.007491251 IL1RAP/PSMD12/VRK2/CUL1/PSMD10/PSMD1/UBE2N/PSMF1/PSMD11/PSME4/PSMD2/PSME3/PSMD3/MYD88/IKBKG/IL1R2 16

BP GO:0031146 SCF-dependent proteasomal ubiquitin-dependent protein catabolic process 15/1105 93/18866 0.000314166 0.009201759 0.007491251

CUL4B/PSMD12/CUL2/CUL1/PSMD10/PSMD1/PSMF1/PSMD11/PSME4/PSMD2/FBXL7/PSME3/PSMD3/FBXL16/KIF14 15

BP GO:0010769 regulation of cell morphogenesis involved in differentiation 34/1105 310/18866 0.000327999 0.009556608 0.007780137 BMPR2/ADNP/ADAM10/PRKDC/CRKL/ROBO1/CDKL5/PAK2/ACTR2/NRP1/KALRN/WDR36/PDLIM5/RYK/MAP2K1/PPP3CA/NEDD4/PLXND1/MAP1B/SKIL/LRP4/FLNA/FBXW8/CORO1C/RAC1/SEMA4B/P4HB/MAPT/SEMA3C/ANAPC2/NEDD4L/WNT5A/PLXNB3/LRP8 34

BP GO:0010770 positive regulation of cell morphogenesis involved in differentiation 21/1105 157/18866 0.000338063 0.009798545 0.007977101 BMPR2/ADNP/PRKDC/CRKL/ROBO1/CDKL5/ACTR2/NRP1/KALRN/MAP2K1/PLXND1/MAP1B/SKIL/FLNA/FBXW8/RAC1/P4HB/MAPT/ANAPC2/PLXNB3/LRP8 21

BP GO:0035794 positive regulation of mitochondrial membrane permeability 12/1105 65/18866 0.000345232 0.009954492 0.008104059 YWHAQ/PPP3R1/YWHAB/TP53BP2/BCL2L11/ATF2/YWHAZ/RHOT1/NMT1/PPP1R13B/BCL2/ACAA2 12

BP GO:0071472 cellular response to salt stress 5/1105 12/18866 0.000382508 0.010805378 0.008796774 XRCC5/ZFP36L1/SLC25A23/AQP1/EFHD1 5

BP GO:1903405 protein localization to nuclear body 5/1105 12/18866

0.000382508 0.010805378 0.008796774 CCT8/CCT4/CCT7/DKC1/CCT2 5

BP GO:1904816 positive regulation of protein localization to chromosome, telomeric region 5/1105 12/18866 0.000382508 0.010805378 0.008796774 CCT8/CCT4/CCT7/DKC1/CCT2 5

BP GO:1904867 protein localization to Cajal body 5/1105 12/18866 0.000382508 0.010805378 0.008796774 CCT8/CCT4/CCT7/DKC1/CCT2 5

BP GO:0051972 regulation of telomerase activity 10/1105 48/18866 0.000387757 0.010898331 0.008872449 XRCC5/CCT4/NEK7/TERF1/PTGES3/DKC1/POT1/CCT2/HMBOX1/PPARG 10

BP GO:0006970 response to osmotic stress 14/1105 85/18866 0.000395048 0.010955994 0.008919392 XRCC5/NOLC1/ATF2/ARHGEF2/ZFP36L1/EGFR/STK39/MAPK10/MAP7/SLC25A23/AQP1/KMO/EFHD1/SLC12A5 14

BP GO:0048863 stem cell differentiation 30/1105 264/18866 0.000396396 0.010955994 0.008919392 YAP1/EIF2AK2/STAT3/XRCC5/NOLC1/PRKDC/NRP1/PSMD12/ACVR1/ZNF281/PWP1/PSMD10/PSMD1/TCF12/PSMF1/PSMD11/PSME4/CBFB/EDNRA/NSUN2/PSMD2/PSME3/PSMD3/CORO1C/SOX5/RUNX1/SEMA4B/SOX6/SEMA3C/NRG1 30

BP GO:0009896 positive regulation of catabolic process 45/1105 454/18866 0.000396431 0.010955994 0.008919392 ROCK2/USP13/SH3GLB1/CSNK1A1/RAB3GAP1/MSN/EIF2AK4/CNOT8/CUL4B/GNAI3/MTDH/PAFAH1B2/BCL2L11/EDEM1/TMTC3/YTHDF3/NEDD4/RNF139/PSMD10/COP1/CSNK2A1/TBK1/TRIM5/KHSRP/IDE/SEC22B/SUMO2/FBXW8/ADAM9/ZFP36L1/ATG7/CAMKK2/ATG5/NSF/PFKFB3/IKBKG/DTX3L/EDEM2/RNF152/TSC1/IRS2/MEFV/NEDD4L/WNT5A/AURKA 45

BP GO:0045785 positive regulation of cell adhesion 43/1105 428/18866 0.000398438 0.010955994 0.008919392 MAP4K4/IL6ST/IQGAP1/CRKL/PAK2/BCL10/NRP1/PPP3CA/NID1/RSU1/AP3B1/PTPN11/FUT4/FLNA/DAG1/ECM2/PIK3R1/ITGAV/ADAM9/CBFB/EMP2/CYLD/FMN1/LYN/GRB2/TFE3/IL4R/RAC1/RUNX1/CD44/CX3CL1/IL2RA/ERBB2/P4HB/LEF1/TSC1/ANK3/ADA/STX3/WNT5A/PLAUR/HYAL1/TNFSF13B 43

BP GO:1902108 regulation of mitochondrial membrane permeability involved in apoptotic process 12/1105 66/18866 0.000399802 0.010955994 0.008919392 YWHAQ/PPP3R1/YWHAB/TP53BP2/BCL2L11/ATF2/YWHAZ/RHOT1/NMT1/PPP1R13B/BCL2/ACAA2 12

BP GO:1904872 regulation of telomerase RNA localization to Cajal body 6/1105 18/18866 0.000401621 0.010955994 0.008919392 DCP2/CCT8/CCT4/CCT7/DKC1/CCT2 6

BP GO:0043087 regulation of GTPase activity 47/1105 481/18866 0.000409528 0.011117187 0.009050621 MAP4K4/ITGB1/RAB3GAP1/BNIP2/SEC23A/IQGAP1/TBC1D2B/STARD13/SIPA1L2/TBC1D20/CRKL/CDKL5/CHML/NRP1/KALRN/WNK1/ARHGAP21/RSU1/EVI5/PLXND1/DOCK7/GDI2/F2R/CDC42SE1/ARFGEF1/ARFGAP3/OCRL/RASA3/RP2/EPHA3/ARHGEF6/ARHGAP20/STXBP5/CORO1C/TBC1D22A/GARNL3/ARHGAP24/CX3CL1/ERBB2/CCL13/TSC1/RIN1/CCL26/WNT5A/ARHGEF10L/PLXNB3/ARHGEF16 47

BP GO:0050807 regulation of synapse organization 27/1105 229/18866 0.000425335 0.011490250.009354336 ADNP/IL1RAP/ADAM10/MAPK14/CRKL/CDKL5/ACTR2/SSH1/KALRN/PDLIM5/EGLN1/SPARC/NEDD4/ARF6/YWHAZ/LRP4/DAG1/ITSN1/EIF4G1/ETV5/PRNP/TUBB/HSPA8/SRPX2/ANAPC2/WNT5A/LRP8 27

BP GO:0046390 ribose phosphate biosynthetic process 24/1105 194/18866 0.00043193 0.011545068 0.009398964 SLC26A2/PANK2/PAPSS1/STAT3/ADCY3/PANK3/ACSL4/GUCY1A2/GMPS/PPAT/PRPS1/GART/CTPS2/PAICS/ELOVL5/ELOVL1/CBR4/ADCY5/PANK1/ELOVL3/UCK1/ACSL1/HSD17B8/ACSS1 24

BP GO:0048002 antigen processing and presentation of peptide antigen 24/1105 194/18866 0.00043193 0.011545068 0.009398964 SEC23A/PSMD12/DCTN5/DCTN4/SNAP23/CLTC/LNPEP/ITGB5/PSMD10/PSMD1/KIF3C/CANX/PSMF1/PSMD11/ITGAV/IDE/SEC22B/PSME4/PSMD

2/PSME3/HFE/PSMD3/IKBKG/KIF2C 24

BP GO:0034446 substrate adhesion-dependent cell spreading 16/1105 106/18866 0.000435416 0.011545068 0.009398964 PARVA/ATRN/LAMC1/CRKL/NRP1/FLNA/RAB1A/PIK3R1/ITGAV/CORO1C/FZD7/RAC1/NTN4/P4HB/LAMB3/KIF14 16

BP GO:0010976 positive regulation of neuron projection development 32/1105 290/18866 0.000435663 0.011545068 0.009398964 BMPR2/TMEM30A/IQGAP1/ADNP/ABL2/SCARB2/ROBO1/CDKL5/ACTR2/NRP1/KALRN/MAP2K1/CREB3L2/PLXND1/MAP1B/PLAA/ATF1/SKIL/ENC1/ITSN1/FBXW8/EPHA3/LYN/ARSB/CX3CL1/MAPT/ROR2/ANAPC2/NEDD4L/WNT5A/PLXNB3/LRP8 32

BP GO:0035023 regulation of Rho protein signal transduction 14/1105 86/18866 0.000446876 0.011786077 0.009595172 KCTD10/HEG1/AKAP13/ITGB1/ABL2/STARD13/ROBO1/NRP1/KALRN/F2R/ARHGEF2/CDC42SE1/RAF1/RAC1 14

BP GO:2000027 regulation of animal organ morphogenesis 29/1105 254/18866 0.000454694 0.011846631 0.009644469 BMPR2/ROBO1/PSMD12/ACVR1/RYK/CLTC/PSMD10/PSMD1/RXRA/PSMF1/PSMD11/PSME4/TGFBR1/PSMD2/PSME3/ETV5/SULF1/PSMD3/FZD7/RAC1/SULF2/BCL2/NTN4/AJAP1/ROR2/SEMA3C/CTHRC1/WNT5A/WT1 29

BP GO:1901342 regulation of vasculature development 44/1105

444/18866 0.000459563 0.011846631 0.009644469 ROCK2/SPRED1/ITGB1/HIPK1/STAT3/STARD13/ADAM10/NFATC3/MTDH/NRP1/AGGF1/ATP2B4/ATF2/EGLN1/SPARC/PLXND1/SMAD1/COL4A2/SP100/PGK1/SULF1/DNMT1/EMP2/BRCA1/ADAMTS9/PML/RUNX1/F3/SERPINE1/ECM1/DDAH1/AGTR1/CX3CL1/CYP1B1/ERBB2/SRPX2/ADAM12/AQP1/PPARG/RLN2/WNT5A/WT1/HTATIP2/HYAL1 44

BP GO:0018107 peptidyl-threonine phosphorylation 17/1105 117/18866 0.000461384 0.011846631 0.009644469 ROCK2/SPRED1/HIPK1/PRKDC/WNK1/ACVR1/MAP2K1/CSNK2A1/CSNK2A2/TBK1/EIF4G1/TGFBR1/BCL2/STK39/CDC42BPG/WNT5A/CIT 17

BP GO:1905710 positive regulation of membrane permeability 12/1105 67/18866 0.000461479 0.011846631 0.009644469 YWHAQ/PPP3R1/YWHAB/TP53BP2/BCL2L11/ATF2/YWHAZ/RHOT1/NMT1/PPP1R13B/BCL2/ACAA2 12

BP GO:0090151 establishment of protein localization to mitochondrial membrane 10/1105 49/18866 0.000461944 0.011846631 0.009644469 YWHAQ/PPP3R1/YWHAB/TP53BP2/BCL2L11/AP3B1/YWHAZ/NMT1/PPP1R13B/BCL2 10

BP GO:0097720 calcineurin-mediated signaling 10/1105 49/18866 0.000461944 0.011846631 0.009644469 PPP3R1/NFATC3/ATP2B4/PPP3CA/LMCD1/PRNP/NR5A2/FHL2/CAMTA

1/NRG1 10

BP GO:0046902 regulation of mitochondrial membrane permeability 13/1105 77/18866 0.000493011 0.012585346 0.010245866 YWHAQ/PPP3R1/STAT3/YWHAB/TP53BP2/BCL2L11/ATF2/YWHAZ/RHOT1/NMT1/PPP1R13B/BCL2/ACAA2 13

BP GO:1901998 toxin transport 9/1105 41/18866 0.00050149 0.01274333 0.010374482 ADAM10/NRP1/CCT8/CCT4/CCT7/ANTXR2/COPB2/RAB43/CCT2 9

BP GO:0001655 urogenital system development 35/1105 330/18866 0.000505213 0.01277959 0.010404002 YAP1/IQGAP1/TMED10/SMARCC1/CRKL/FBN1/NRP1/MYO1E/BCL2L11/NOTCH2/COL4A1/NID1/APAF1/LRP4/SMAD1/PPAT/RXRA/TGFBR1/NOTCH3/RRM2B/SULF1/NUP107/FMN1/SGPL1/SULF2/BCL2/COL4A4/FRAS1/AGTR1/TSC1/PKHD1/WNT5A/WT1/ID4/EMX2 35

BP GO:0019884 antigen processing and presentation of exogenous antigen 23/1105 185/18866 0.00052512 0.01315818 0.010712216 SEC23A/PSMD12/DCTN5/DCTN4/SNAP23/CLTC/AP3B1/LNPEP/ITGB5/PSMD10/PSMD1/KIF3C/CANX/PSMF1/PSMD11/ITGAV/SEC22B/PSME4/PSMD2/PSME3/PSMD3/IKBKG/KIF2C 23

BP GO:0035567 non-canonical Wnt signaling pathway 20/1105 151/18866 0.000526811 0.01315818 0.010712216 PPP3R1/PSMD12/RYK/PPP3CA/CLTC/PSMD10/PSMD1/DAAM2/PSMF1

/PSMD11/PSME4/PSMD2/PSME3/PSMD3/FZD7/RAC1/LEF1/ROR2/CTHRC1/WNT5A 20

BP GO:0007266 Rho protein signal transduction 19/1105 140/18866 0.000529637 0.01315818 0.010712216 ROCK2/KCTD10/HEG1/AKAP13/ITGB1/ABL2/STARD13/ROBO1/NRP1/GNA12/KALRN/GNA13/F2R/ARHGEF2/CDC42SE1/RAF1/RHOU/RAC1/AGTR1 19

BP GO:0009411 response to UV 19/1105 140/18866 0.000529637 0.01315818 0.010712216 MSH6/EIF2AK4/USP47/MSH2/CUL4B/NEDD4/RHBDD1/ZRANB3/PIK3R1/POLD3/RAD18/UBE2A/PML/EGFR/HMGN1/BCL2/CIRBP/AQP1/HYAL1 19

BP GO:0072659 protein localization to plasma membrane 31/1105 281/18866 0.000533316 0.013190678 0.010738673 ROCK2/ITGB1/SEC23A/KIF5B/ATP2B4/GOLPH3/ARF6/CLTC/RAB10/RAB8B/RAB31/FLNA/PIK3R1/P2RY1/MYO5A/PRNP/EPHA3/EMP2/RAB3D/GOLPH3L/LDLRAP1/EGFR/NSF/STXBP1/ZDHHC7/TMEM88/MAP7/ANK3/STX3/WDR72/ARHGEF16 31

BP GO:0060071 Wnt signaling pathway, planar cell polarity pathway 16/1105 108/18866 0.000538645 0.013221522 0.010763783 PSMD12/RYK/CLTC/PSMD10/PSMD1/PSMF1/PSMD11/PSME4/PSMD2/PSME3/PSMD3/FZD7/RAC1/ROR2/CTHRC1/WNT5A 16

BP GO:0007528 neuromuscular junction development 10/1105

50/18866 0.000547534 0.013221522 0.010763783 CRKL/KALRN/COL4A1/NEDD4/F2R/LRP4/ETV5/AFG3L2/ERBB2/ANK3 10

BP GO:0007568 aging 34/1105 319/18866 0.000552481 0.013221522 0.010763783 MSH6/STAT3/NUAK1/MSH2/PRKDC/MAPK14/SEC63/MAP2K1/PPP3CA/GCLM/APAF1/DAG1/CANX/COL4A2/RBL1/IDE/ATP2B1/P2RY1/ATG7/PRNP/TGFB3/PML/GRB2/BCL2/SERPINE1/CX3CL1/KL/KMO/IGFBP1/ADA/FOXM1/GLRX2/CHEK2/TIMP1 34

BP GO:0048008 platelet-derived growth factor receptor signaling pathway 11/110559/18866 0.000552825 0.013221522 0.010763783 IQGAP1/PTPN12/NRP1/MYO1E/PTPN11/PHF14/CBLB/SGPL1/F3/LOX/PDGFRL 11

BP GO:0019054 modulation by virus of host cellular process 6/1105 19/18866 0.000558058 0.013221522 0.010763783 EIF2AK4/BCL2L11/KPNA3/KPNB1/KPNA4/ATG7 6

BP GO:0090670 RNA localization to Cajal body 6/1105 19/18866 0.000558058 0.013221522 0.010763783 DCP2/CCT8/CCT4/CCT7/DKC1/CCT2 6

BP GO:0090671 telomerase RNA localization to Cajal body 6/1105 19/18866 0.000558058 0.013221522 0.010763783 DCP2/CCT8/CCT4/CCT7/DKC1/CCT2 6

BP GO:0090672 telomerase RNA localization 6/1105 19/18866 0.000558058 0.013221522 0.010763783 DCP2/CCT8/CCT4/CCT7/DKC1/CCT2 6

BP GO:0090685 RNA localization to nucleus 6/1105 19/18866 0.000558058 0.013221522 0.010763783 DCP2/CCT8/CCT4/CCT7/DKC1/CCT2 6

BP GO:2001234 negative regulation of apoptotic signaling pathway 27/1105 233/18866 0.000558475 0.013221522 0.010763783 YAP1/USP47/GNAI3/NRP1/ACVR1/RB1CC1/HDAC1/PSMD10/GCLM/ARHGEF2/CSNK2A1/CSNK2A2/RAF1/ITGAV/TGFBR1/PHIP/PSME3/RRM2B/RNF34/BRCA1/BCL2/SERPINE1/CD44/CX3CL1/ACAA2/SCG2/PLAUR 27

BP GO:0010822 positive regulation of mitochondrion organization 17/1105 119/18866 0.000563449 0.013221522 0.010763783 SH3GLB1/YWHAQ/PPP3R1/YWHAB/TP53BP2/BCL2L11/YWHAZ/NPEPPS/SAE1/CSNK2A2/RHOU/CAMKK2/NMT1/PPP1R13B/BCL2/MLLT11/PLAUR 17

BP GO:0009260 ribonucleotide biosynthetic process 23/1105 186/18866 0.000566797 0.013221522 0.010763783 SLC26A2/PANK2/PAPSS1/STAT3/ADCY3/PANK3/ACSL4/GUCY1A2/GMPS/PPAT/GART/CTPS2/PAICS/ELOVL5/ELOVL1/CBR4/ADCY5/PANK1/ELOVL3/UCK1/ACSL1/HSD17B8/ACSS1 23

BP GO:0090559 regulation of membrane permeability 14/1105

88/18866 0.00056807 0.013221522 0.010763783 HEG1/YWHAQ/PPP3R1/STAT3/YWHAB/TP53BP2/BCL2L11/ATF2/YWHAZ/RHOT1/NMT1/PPP1R13B/BCL2/ACAA2 14

BP GO:0001783 B cell apoptotic process 7/1105 26/18866 0.0005702 0.013221522 0.010763783 CRKL/BCL10/BCL2L11/SLC39A10/LYN/IRS2/ADA 7

BP GO:0044068 modulation by symbiont of host cellular process 7/1105 26/18866 0.0005702 0.013221522 0.010763783 EIF2AK4/BCL2L11/KPNA3/KPNB1/KPNA4/ATG7/ATG5 7

BP GO:0007004 telomere maintenance via telomerase 12/1105 69/18866 0.000609074 0.014057992 0.011444763 XRCC5/DCP2/CCT8/CCT4/CCT7/NEK7/TERF1/PTGES3/DKC1/POT1/CCT2/HMBOX1 12

BP GO:0050821 protein stabilization 23/1105 187/18866 0.000611327 0.014057992 0.011444763 USP13/USP9X/SEL1L/CCT8/GTPBP4/CCT4/CCT7/FLNA/PRKRA/PIK3R1/PTGES3/CRTAP/ATF7IP/CCT2/LAMP2/STXBP1/PER3/TMEM88/TSC1/GBA3/CHEK2/BAG1/GTSE1 23

BP GO:0051817 modulation of process of other organism involved in symbiotic interaction 15/1105 99/18866 0.00062409 0.014292421 0.011635614 EIF2AK4/EIF2AK2/TBC1D20/BCL2L11/KPNA3/KPNB1/HDAC1/KPNA4/D

AG1/RXRA/ATG7/ATG5/HSPA8/LEF1/AQP1 15

BP GO:0071260 cellular response to mechanical stimulus 13/1105 79/18866 0.000635496 0.014493991 0.011799714 SLC38A2/BCL10/MAP3K2/PTPN11/MTPN/DAG1/EGFR/MYD88/RAC1/ATP1A1/HABP4/AQP1/CASP5 13

BP GO:0016482 cytosolic transport 21/1105 165/18866 0.000661338 0.0149793 0.01219481 RAB6A/KLHL20/MSN/KIF5B/TMEM87A/ACTR2/GOSR2/MAP2K1/EVI5/CLTC/SNX6/MYO1D/STX6/VPS26B/GBF1/TBC1D23/UBE2O/EMP2/VPS29/CCDC91/HOOK2 21

BP GO:0090175 regulation of establishment of planar polarity 16/1105 110/18866 0.000662158 0.0149793 0.01219481 PSMD12/RYK/CLTC/PSMD10/PSMD1/PSMF1/PSMD11/PSME4/PSMD2/PSME3/PSMD3/FZD7/RAC1/ROR2/CTHRC1/WNT5A 16

BP GO:0002478 antigen processing and presentation of exogenous peptide antigen 22/1105 177/18866 0.000689402 0.015532482 0.012645161 SEC23A/PSMD12/DCTN5/DCTN4/SNAP23/CLTC/LNPEP/ITGB5/PSMD10/PSMD1/KIF3C/CANX/PSMF1/PSMD11/ITGAV/SEC22B/PSME4/PSMD2/PSME3/PSMD3/IKBKG/KIF2C22

BP GO:0010565 regulation of cellular ketone metabolic process 23/1105 189/18866 0.000709597 0.015874629 0.012923706 PANK2/PSMD12/ATP2B4/ADIPOR1/AZIN1/PSMD10/PSMD1/INSIG2/H6

PD/PSMF1/PSMD11/PSME4/PRKAB2/PSMD2/PSME3/PSMD3/BRCA1/ELOVL5/NCOR2/SIRT5/IRS2/PPARG/FABP5 23

BP GO:0019693 ribose phosphate metabolic process 42/1105 427/18866 0.000711831 0.015874629 0.012923706 NUP98/SLC26A2/PANK2/PAPSS1/STAT3/NUP155/PGM2L1/ADCY3/GNAI3/PANK3/RAB23/NUP205/ACSL4/GUCY1A2/NT5E/ACOT9/GMPS/PPAT/PRPS1/GART/CASK/PGK1/CTPS2/NUP93/PAICS/NUP107/PDE4B/PFKFB3/ELOVL5/ELOVL1/CBR4/ADCY5/PDE10A/PANK1/ELOVL3/HMGCL/TPST2/UCK1/ACSL1/HSD17B8/TDO2/ACSS1 42

BP GO:0006029 proteoglycan metabolic process 14/1105 90/18866 0.000716083 0.015874629 0.012923706 BMPR2/EXT2/FAM20B/NDST1/CHSY1/HS2ST1/IDS/CANT1/EXT1/SULF1/ARSB/SLC35D1/SULF2/HYAL1 14

BP GO:0032204 regulation of telomere maintenance 13/1105 80/18866 0.000718851 0.015874629 0.012923706 XRCC5/DCP2/CCT8/CCT4/CCT7/NEK7/TERF1/NBN/DKC1/POT1/CCT2/PML/HMBOX1 13

BP GO:1900182 positive regulation of protein localization to nucleus 13/1105 80/18866 0.000718851 0.015874629 0.012923706 MAPK14/EIF2AK3/CCT8/CCT4/CCT7/FLNA/PIK3R1/HDAC3/DKC1/CCT2/DTX3L/PARP9/GTSE1 13

BP GO:0006829 zinc ion transport 7/1105 27/18866 0.000731212

0.015957626 0.012991275
SLC30A1/SLC30A6/SLC39A10/SLC30A5/SLC30A7/SLC39A6/SLC39A11 7

BP GO:0018126 protein hydroxylation 7/1105 27/18866 0.000731212 0.015957626 0.012991275
EGLN1/OGFOD1/P4HA1/CRTAP/PLOD2/P4HA3/P4HB 7

BP GO:0070199 establishment of protein localization to chromosome 7/1105 27/18866 0.000731212 0.015957626 0.012991275
CCT8/CCT4/CCT7/TERF1/DKC1/POT1/CCT2 7

BP GO:0030509 BMP signaling pathway 20/1105 155/18866 0.000738089 0.01604478 0.013062228
BMPR2/PELO/USP9X/FBN1/ZFYVE16/NOTCH2/ACVR1/SKIL/SMAD1/USP15/RGMB/UBE2O/SULF1/HFE/TGFB3/SLC33A1/TWSG1/LEF1/ROR2/WNT5A 20

BP GO:1904356 regulation of telomere maintenance via telomere lengthening 11/1105 61/18866 0.000742057 0.016045711 0.013062986
XRCC5/DCP2/CCT8/CCT4/CCT7/NEK7/TERF1/DKC1/POT1/CCT2/HMBOX1 11

BP GO:0016569 covalent chromatin modification 46/1105 481/18866 0.000743898 0.016045711 0.013062986
PHF20/NSD1/TRIP12/TAF5L/CUL4B/RNF2/CDC73/HAT1/BAZ1B/SETD7/ATF2/TRRAP/MIER1/PWP1/HDAC1/SART3/PCGF6/PHF20L1/HLCS/WDR7

0/MTA3/YEATS2/SUV39H2/PHF14/UBE2N/MORF4L2/INCENP/MORC2/CDK2/HDAC3/UBE2A/USP15/ATF7IP/PPHLN1/UBE2E1/DNMT1/ATXN7L3/ATG5/BRCA1/DTX3L/SETD3/ZNF711/LEF1/CCNB1/CXXC1/AURKA 46

BP GO:0006401 RNA catabolic process 41/1105 415/18866 0.000751018 0.016136744 0.013137097 ROCK2/YWHAB/PELO/CNOT8/TNPO1/CNOT2/MAPK14/ETF1/PSMD12/GSPT1/YTHDF3/FXR1/DCP2/RNASEH1/PSMD10/YWHAZ/PSMD1/XRN2/PAIP1/PSMF1/KHSRP/PSMD11/EIF4G1/PSME4/NCBP2/NSUN2/DCP1B/DKC1/PSMD2/ZFP36L1/PSME3/MLH1/ZHX2/PSMD3/MYD88/OAS2/HSPA8/RPS23/SECISBP2/CIRBP/MYEF2 41

BP GO:0043555 regulation of translation in response to stress 6/1105 20/18866 0.000757984 0.016174042 0.013167462 EIF2AK4/EIF2AK2/DNAJC3/EIF2AK3/RPS6KA3/EIF4G1 6

BP GO:0042177 negative regulation of protein catabolic process 18/1105 133/18866 0.000758567 0.016174042 0.013167462 SF3B3/SMARCC1/SENP1/UBE2J1/OS9/AZIN1/FLNA/CSNK2A1/CSNK2A2/PSMF1/HFE/PML/EGFR/SDCBP/SERPINE2/PTPN3/NRG1/TIMP1 18

BP GO:0070507 regulation of microtubule cytoskeleton organization 23/1105 190/18866 0.000763679 0.016211137 0.013197661 ROCK2/CLASP1/TAOK1/MAPRE1/SPAST/CKAP5/RNF4/CLTC/MAP1B/ARHGEF2/EPHA3/BRCA1/CYLD/RAC1/CKAP2/FAM107A/RCC1/TPPP/MAPT/PKHD1/TPX2/GBA2/AURKA 23

BP GO:0007435 salivary gland morphogenesis 8/1105 35/18866 0.000769046 0.016211137 0.013197661 NRP1/PLXND1/DAG1/TGFB3/EGFR/TWSG1/NTN4/SEMA3C 8

BP GO:1901030 positive regulation of mitochondrial outer membrane permeabilization involved in apoptotic signaling pathway 8/1105 35/18866 0.000769046 0.016211137 0.013197661 YWHAQ/PPP3R1/YWHAB/TP53BP2/YWHAZ/NMT1/PPP1R13B/BCL2 8

BP GO:0044773 mitotic DNA damage checkpoint 15/1105 101/18866 0.000773189 0.016236977 0.013218697 CNOT8/MSH2/CNOT2/TAOK1/PRKDC/ATF2/TOPBP1/CDK2/NBN/PML/RINT1/CCNB1/CHEK2/AURKA/GTSE1 15

BP GO:0009259 ribonucleotide metabolic process 41/1105 416/18866 0.000787392 0.016473069 0.013410902 NUP98/SLC26A2/PANK2/PAPSS1/STAT3/NUP155/PGM2L1/ADCY3/GNAI3/PANK3/RAB23/NUP205/ACSL4/GUCY1A2/NT5E/ACOT9/GMPS/PPAT/GART/CASK/PGK1/CTPS2/NUP93/PAICS/NUP107/PDE4B/PFKFB3/ELOVL5/ELOVL1/CBR4/ADCY5/PDE10A/PANK1/ELOVL3/HMGCL/TPST2/UCK1/ACSL1/HSD17B8/TDO2/ACSS1 41

BP GO:0045765 regulation of angiogenesis 40/1105 403/18866 0.000791585 0.016494713 0.013428523 ROCK2/SPRED1/ITGB1/HIPK1/STAT3/STARD13/MTDH/NRP1/AGGF1/ATP2B4/ATF2/EGLN1/SPARC/PLXND1/SMAD1/COL4A2/SP100/PGK1/SULF1

/EMP2/BRCA1/ADAMTS9/PML/RUNX1/F3/SERPINE1/ECM1/DDAH1/AGTR1/CX3CL1/CYP1B1/ERBB2/SRPX2/ADAM12/AQP1/PPARG/RLN2/WNT5A/HTATIP2/HYAL1 40

BP GO:0046579 positive regulation of Ras protein signal transduction 12/1105 71/18866 0.000794355 0.016494713 0.013428523 MAP4K4/AKAP13/SHOC2/CRKL/ROBO1/NOTCH2/F2R/LYN/GRB2/RAC1/IRS2/NRG1 12

BP GO:0010833 telomere maintenance via telomere lengthening 13/1105 81/18866 0.000811216 0.016782217 0.013662583 XRCC5/DCLRE1B/DCP2/CCT8/CCT4/CCT7/NEK7/TERF1/PTGES3/DKC1/POT1/CCT2/HMBOX1 13

BP GO:0010821 regulation of mitochondrion organization 23/1105 191/18866 0.0008213 0.016927914 0.013781196 SH3GLB1/YWHAQ/PPP3R1/YWHAB/TP53BP2/BCL2L11/GOLPH3/PSMD10/YWHAZ/NPEPPS/SAE1/CSNK2A2/RHOT1/RHOU/LMAN1/CAMKK2/NMT1/PPP1R13B/BCL2/MLLT11/ACAA2/MAPT/PLAUR 23

BP GO:0033173 calcineurin-NFAT signaling cascade 9/1105 44/18866 0.000866515 0.017793925 0.014486226 PPP3R1/NFATC3/ATP2B4/PPP3CA/LMCD1/PRNP/FHL2/CAMTA1/NRG1 9

BP GO:0006999 nuclear pore organization 5/1105 14/18866 0.000876178 0.017860552 0.014540467

NUP98/AHCTF1/NUP205/NUP93/NUP107 5

BP GO:1990173 protein localization to nucleoplasm 5/1105 14/18866 0.000876178 0.017860552 0.014540467 CCT8/CCT4/CCT7/DKC1/CCT2 5

BP GO:0050803 regulation of synapse structure or activity 27/1105 240/18866 0.000880719 0.017887598 0.014562486 ADNP/IL1RAP/ADAM10/MAPK14/CRKL/CDKL5/ACTR2/SSH1/KALRN/PDLIM5/EGLN1/SPARC/NEDD4/ARF6/YWHAZ/LRP4/DAG1/ITSN1/EIF4G1/ETV5/PRNP/TUBB/HSPA8/SRPX2/ANAPC2/WNT5A/LRP8 27

BP GO:0002223 stimulatory C-type lectin receptor signaling pathway 16/1105 113/18866 0.000892377 0.018058456 0.014701583 PAK2/BCL10/PSMD12/NRAS/CUL1/PSMD10/PSMD1/PSMF1/RAF1/PSMD11/PSME4/PSMD2/PSME3/PSMD3/LYN/IKBKG 16

BP GO:0031330 negative regulation of cellular catabolic process 29/1105 265/18866 0.000901952 0.018090697 0.014727831 STAT3/FEZ2/SMARCC1/SENP1/MAPK14/EIF4G2/UBE2J1/ATP2B4/OS9/CSNK2A1/PAIP1/CSNK2A2/PSMF1/SEC22B/UBQLN4/EIF4G1/NSUN2/DKC1/ATG7/HFE/PML/MYD88/SDCBP/SECISBP2/BCL2/CIRBP/TSC1/PTPN3/TIMP1 29

BP GO:0038095 Fc-epsilon receptor signaling pathway 21/1105 169/18866 0.000905611 0.018090697 0.014727831 PPP3R1/NFATC3/PAK2/BCL10/PSMD12/PPP3CA/CUL1/PSMD10/PSMD

1/PIK3R1/PSMF1/PSMD11/PSME4/PSMD2/PSME3/PSMD3/LYN/GRB2/RAC1/IKBKG/MAPK10 21

BP GO:0001736 establishment of planar polarity 17/1105 124/18866 0.000906973 0.018090697 0.014727831 PSMD12/RYK/CLTC/PSMD10/PSMD1/PSMF1/PSMD11/PSME4/PSMD2/PSME3/PSMD3/FZD7/RAC1/ROR2/CTHRC1/ASTN2/WNT5A 17

BP GO:0007164 establishment of tissue polarity 17/1105 124/18866 0.000906973 0.018090697 0.014727831 PSMD12/RYK/CLTC/PSMD10/PSMD1/PSMF1/PSMD11/PSME4/PSMD2/PSME3/PSMD3/FZD7/RAC1/ROR2/CTHRC1/ASTN2/WNT5A 17

BP GO:0016925 protein sumoylation 13/1105 82/18866 0.000913341 0.018152653 0.01477827 NUP98/NUP155/SENP1/SENP2/NUP205/RNF4/SAE1/SUMO2/PIAS3/NUP93/SUMO3/NUP107/UBE2I 13

BP GO:0045666 positive regulation of neuron differentiation 38/1105 380/18866 0.000920545 0.018230713 0.014841819 BMPR2/TMEM30A/IQGAP1/ADNP/ABL2/SCARB2/ROBO1/CDKL5/ACTR2/NRP1/KALRN/MAP2K1/CREB3L2/PLXND1/MAP1B/PLAA/TCF12/ATF1/SKIL/ARHGEF2/ENC1/ITSN1/EIF4G1/FBXW8/ETV5/EPHA3/LYN/ARSB/BCL2/CYB5D2/CX3CL1/MAPT/ROR2/ANAPC2/NEDD4L/WNT5A/PLXNB3/LRP8 38

BP GO:0008361 regulation of cell size 22/1105 181/18866 0.000931645 0.01832015 0.014914631

BMPR2/MSN/ADNP/CDKL5/NRP1/WDR36/RYK/RB1CC1/MAP1B/MTPN/TMEM123/CLNS1A/RAC1/CLCN6/SEMA4B/TSC1/MAPT/AQP1/SEMA3C/ANAPC2/WNT5A/SLC12A5 22

BP GO:1905330 regulation of morphogenesis of an epithelium 22/1105 181/18866 0.000931645 0.01832015 0.014914631 CLASP1/PSMD12/RYK/CLTC/PSMD10/PSMD1/RXRA/PSMF1/PSMD11/PSME4/PSMD2/PSME3/ETV5/SULF1/PSMD3/FZD7/RAC1/NTN4/AJAP1/ROR2/CTHRC1/WNT5A 22

BP GO:0038093 Fc receptor signaling pathway 27/1105 241/18866 0.00093796 0.018328155 0.014921148 PPP3R1/NFATC3/PAK2/ACTR2/BCL10/PSMD12/PPP3CA/ACTR3/CUL1/PSMD10/PSMD1/ABI1/PIK3R1/PSMF1/PSMD11/PSME4/PSMD2/PSME3/PSMD3/LYN/GRB2/ELMO2/RAC1/IKBKG/MAPK10/ARPC3/ARPC1B 27

BP GO:0051973 positive regulation of telomerase activity 8/1105 36/18866 0.000938639 0.018328155 0.014921148 XRCC5/CCT4/NEK7/PTGES3/DKC1/POT1/CCT2/HMBOX1 8

BP GO:0090150 establishment of protein localization to membrane 35/1105 342/18866 0.000960733 0.018670749 0.015200057 RAB3GAP1/YWHAQ/PPP3R1/SRP72/YWHAB/ZDHHC20/TP53BP2/BCL2L11/SEC63/GOLPH3/RAB10/AP3B1/SSR1/RAB8B/EXOC4/YWHAZ/SGTB/RAB31/VPS37A/PRNP/NMT1/GOLPH3L/EGFR/NSF/PPP1R13B/SDCBP/RPS23/ZDHHC7/BCL2/RAB3IP/MAPK10/ERBB2/ANK3/VPS37D/STX3 35

BP GO:0031346 positive regulation of cell projection organization 39/1105 394/18866 0.000962894 0.018670749 0.015200057 BMPR2/TMEM30A/IQGAP1/ADNP/ABL2/SCARB2/ROBO1/CDKL5/ACTR2/NRP1/KALRN/MAP2K1/ACTR3/CREB3L2/PLXND1/MAP1B/RAB8B/PLAA/ATF1/SKIL/ENC1/ITSN1/FBXW8/TGFBR1/EPHA3/TGFB3/CORO1C/LYN/ARSB/RAC1/CX3CL1/KCTD17/MAPT/ROR2/ANAPC2/NEDD4L/WNT5A/PLXNB3/LRP8 39

BP GO:0009314 response to radiation 43/1105 447/18866 0.000969871 0.018740734 0.015257033 METAP2/BACE1/ITGB1/MSH6/YAP1/EIF2AK4/USP47/XRCC5/MSH2/CUL4B/PRKDC/MAPK14/RRM1/THBD/NEDD4/RHBDD1/RAD1/ZRANB3/TOPBP1/COP1/PIK3R1/POLD3/RAD18/PPP1CC/UBE2A/COPS3/NMT1/XRCC4/BRCA1/PML/EGFR/GRB2/HMGN1/PER3/BCL2/CRY2/CIRBP/MAPK10/AQP1/CDS1/CACNA2D4/CHEK2/HYAL1 43

BP GO:0006521 regulation of cellular amino acid metabolic process 11/110563/18866 0.000982159 0.018855474 0.015350444 PSMD12/ATP2B4/AZIN1/PSMD10/PSMD1/PSMF1/PSMD11/PSME4/PSMD2/PSME3/PSMD3 11

BP GO:0034329 cell junction assembly 42/1105 434/18866 0.000982585 0.018855474 0.015350444 ROCK2/MAP4K4/HEG1/CLASP1/VCL/IQGAP1/HIPK1/ADNP/IL1RAP/LAMC1/CRKL/MTDH/PAK2/NRP1/PDLIM5/RYK/ARF6/PLXND1/MAP1B/RAP1B

/LRP4/FLNA/EIF4G1/CBFB/EPHA3/GNPAT/DST/FMN1/CORO1C/RAC1/RUNX1/BCL2/FAM107A/SRPX2/TSC1/CLDN10/NRG1/LAMB3/FLNC/ARVCF/WNT5A/NRXN2 42

BP GO:0008637 apoptotic mitochondrial changes 17/1105 125/18866 0.000993732 0.019003852 0.01547124 YWHAQ/PPP3R1/YWHAB/TP53BP2/BCL2L11/ATF2/PSMD10/GCLM/YWHAZ/RHOT1/NMT1/PPP1R13B/BCL2/MLLT11/ACAA2/SLC25A4/PLAUR 17

BP GO:2001252 positive regulation of chromosome organization 22/1105 182/18866 0.001002588 0.019064007 0.015520213 CDC27/XRCC5/MIER1/SART3/CCT8/CCT4/CCT7/NEK7/TERF1/UBE2N/MORC2/NBN/ATF7IP/PPHLN1/DKC1/POT1/CCT2/DNMT1/BRCA1/PML/HMBOX1/CCNB1 22

BP GO:0071375 cellular response to peptide hormone stimulus 34/1105 330/18866 0.001003729 0.019064007 0.015520213 ROCK2/STAT3/RPS6KB1/ADCY3/SMARCC1/PRKDC/FBN1/ADIPOR1/RAB10/MAP1B/RAB8B/PTPN11/AHCYL1/RAP1B/RAB31/INSIG2/PPAT/PIK3R1/IDE/ATP2B1/MYO5A/PHIP/ZFP36L1/LYN/GRB2/ZDHHC7/AGTR1/KL/ADCY5/TSC1/IRS2/IGFBP1/PPARG/GKAP1 34

BP GO:0072001 renal system development 31/1105 292/18866 0.001011804 0.019127042 0.015571531 YAP1/IQGAP1/TMED10/FBN1/NRP1/MYO1E/BCL2L11/NOTCH2/COL4A1/NID1/APAF1/LRP4/SMAD1/PPAT/TGFBR1/NOTCH3/RRM2B/SULF1/NUP1

07/FMN1/SGPL1/SULF2/BCL2/COL4A4/FRAS1/AGTR1/TSC1/PKHD1/WNT5A/WT1/EMX2 31

BP GO:0033044 regulation of chromosome organization 36/1105 356/18866 0.001015533 0.019127042 0.015571531 CDC27/NSD1/TRIP12/XRCC5/ANAPC1/RIOK2/SSRP1/SETD7/MIER1/DCP2/RNF4/SART3/CCT8/WDR70/CCT4/CCT7/NEK7/TERF1/UBE2N/MORC2/NBN/ATF7IP/PPHLN1/DKC1/POT1/CCT2/DNMT1/ATG5/BRCA1/TLK1/PML/HMBOX1/CCNB1/MAPT/BUB1/TRIP13 36

BP GO:0001570 vasculogenesis 13/1105 83/18866 0.001026019 0.019127042 0.015571531 SPRED1/HEG1/YAP1/QKI/MYO1E/AGGF1/ITGAV/ZFP36L1/ZFPM2/EMP2/SGPL1/FOXM1/WT1 13

BP GO:0048041 focal adhesion assembly 13/1105 83/18866 0.001026019 0.019127042 0.015571531 ROCK2/MAP4K4/CLASP1/VCL/IQGAP1/NRP1/EPHA3/FMN1/CORO1C/RAC1/BCL2/FAM107A/TSC1 13

BP GO:0051204 protein insertion into mitochondrial membrane 9/1105 45/18866 0.00102767 0.019127042 0.015571531 YWHAQ/PPP3R1/YWHAB/TP53BP2/BCL2L11/YWHAZ/NMT1/PPP1R13B/BCL2 9

BP GO:1901028 regulation of mitochondrial outer membrane permeabilization involved in apoptotic signaling pathway 9/1105 45/18866

0.00102767 0.019127042 0.015571531 YWHAQ/PPP3R1/YWHAB/TP53BP2/YWHAZ/NMT1/PPP1R13B/BCL2/ACAA2 9

BP GO:0043254 regulation of protein-containing complex assembly 43/1105 449/18866 0.001059719 0.019657794 0.016003621 CLASP1/SH3GLB1/MSN/CAND1/EIF2AK2/CUL4B/SAR1A/MAPRE1/ACTR2/SSH1/BCL2L11/CKAP5/ACTR3/ARF6/RNF4/MAP1B/PTPN11/GTF2H3/RAP1B/MTPN/ARHGEF2/TERF1/ARFGEF1/BIRC2/RAF1/TFIP11/EIF4G1/ATF7IP/PLEKHG2/FMN1/GRB2/HSPA8/RAC1/STXBP1/TPPP/MAPT/GBA2/ARPC3/NRG1/KIF14/MEFV/CCL26/ARPC1B 43

BP GO:0006986 response to unfolded protein 22/1105 183/18866 0.001078136 0.019853671 0.016163087 ATF6/EIF2AK2/EDEM3/BCL2L11/EDEM1/DNAJC3/TOR1B/GOSR2/CREB3L2/GFPT1/EIF2AK3/RHBDD1/SSR1/MBTPS2/CANX/PIK3R1/BFAR/STT3B/HSPA8/EDEM2/IGFBP1/CXXC1 22

BP GO:0071347 cellular response to interleukin-1 22/1105 183/18866 0.001078136 0.019853671 0.016163087 IL1RAP/PSMD12/VRK2/CUL1/PSMD10/PSMD1/UBE2N/PSMF1/PSMD11/PSME4/PSMD2/PSME3/PSMD3/MYD88/IKBKG/CX3CL1/CCL13/IL1R2/KMO/CEBPB/CCL26/HYAL1 22

BP GO:0043620 regulation of DNA-templated transcription in response to stress 16/1105 115/18866 0.001080982 0.019853671 0.016163087

ATF6/PSMD12/EGLN1/NEDD4/CUL2/PSMD10/PSMD1/MBTPS2/RPS6KA3/PSMF1/PSMD11/PSME4/PSMD2/PSME3/PSMD3/CEBPB 16

BP GO:1901136 carbohydrate derivative catabolic process 23/1105 195/18866 0.001091069 0.019973026 0.016260255 EDEM3/EDEM1/IDS/NT5E/CTBS/TDG/STT3B/DPYD/UNG/ADAMTS9/MANBA/NAGA/CDADC1/PDE4B/ARSB/EDEM2/CD44/PDE10A/ADA/GBA2/HMMR/GBA3/HYAL1 23

BP GO:1990138 neuron projection extension 21/1105 172/18866 0.001136492 0.020736319 0.01688166 BMPR2/ITGB1/VCL/RAPH1/IQGAP1/EIF2AK4/ADNP/USP9X/CDKL5/NRP1/WDR36/RYK/MAP1B/PLAA/SEMA4B/MAPT/SEMA3C/ANAPC2/NEDD4L/WNT5A/AURKA 21

BP GO:0010390 histone monoubiquitination 7/1105 29/18866 0.001159817 0.020874607 0.016994242 CUL4B/RNF2/CDC73/PCGF6/UBE2E1/ATXN7L3/DTX3L 7

BP GO:0030970 retrograde protein transport, ER to cytosol 7/1105 29/18866 0.001159817 0.020874607 0.016994242 SEL1L/UBE2J1/EDEM1/OS9/RHBDD1/EDEM2/HM13 7

BP GO:0150117 positive regulation of cell-substrate junction organization 7/1105 29/18866 0.001159817 0.020874607 0.016994242 MAP4K4/IQGAP1/NRP1/ARF6/FMN1/RAC1/TSC1 7

BP GO:1903513 endoplasmic reticulum to cytosol transport 7/1105

29/18866 0.001159817 0.020874607 0.016994242 SEL1L/UBE2J1/EDEM1/OS9/RHBDD1/EDEM2/HM13 7

BP GO:1901653 cellular response to peptide 39/1105 398/18866 0.001162826 0.020874607 0.016994242 ROCK2/BACE1/STAT3/RPS6KB1/ADCY3/SMARCC1/PRKDC/FBN1/BCL2L11/ADIPOR1/RAB10/MAP1B/RAB8B/PTPN11/AHCYL1/RAP1B/RAB31/INSIG2/SNX6/ARHGEF2/PPAT/PIK3R1/IDE/ATP2B1/MYO5A/PHIP/ZFP36L1/PRNP/LYN/GRB2/ZDHHC7/AGTR1/KL/ADCY5/TSC1/IRS2/IGFBP1/PPARG/GKAP1 39

BP GO:0072522 purine-containing compound biosynthetic process 24/1105 208/18866 0.001170531 0.020945351 0.017051835 SLC26A2/PANK2/PAPSS1/STAT3/ADCY3/PANK3/ACSL4/GUCY1A2/NT5E/GMPS/PPAT/PRPS1/GART/PAICS/ELOVL5/ELOVL1/CBR4/ADCY5/PANK1/ELOVL3/ADA/ACSL1/HSD17B8/ACSS1 24

BP GO:0002220 innate immune response activating cell surface receptor signaling pathway 16/1105 116/18866 0.001187279 0.021132226 0.017203973 PAK2/BCL10/PSMD12/NRAS/CUL1/PSMD10/PSMD1/PSMF1/RAF1/PSMD11/PSME4/PSMD2/PSME3/PSMD3/LYN/IKBKG 16

BP GO:0001704 formation of primary germ layer 17/1105 127/18866 0.001188569 0.021132226 0.017203973 BMPR2/ITGB1/EXT2/CDC73/ACVR1/ITGB5/EXOC4/SMAD1/COL4A2/IT

GAV/COL12A1/TWSG1/FZD7/LEF1/LAMB3/WNT5A/ATOH8 17

BP GO:0001503 ossification 40/1105 412/18866 0.001205949 0.021372947 0.017399946 BMPR2/EXT2/SLC26A2/IL6ST/SP3/CHSY1/MAPK14/DDX21/ADAR/DDR2/ANKH/ACVR1/SPARC/BCAP29/EIF2AK3/CLTC/PTPN11/GTPBP4/LRP4/SMAD1/EXT1/ATP2B1/CBFB/TGFB3/MINPP1/EGFR/TWSG1/RUNX1/BCL2/ECM1/LOX/TMEM38B/FHL2/KL/LEF1/ROR2/CTHRC1/CEBPB/WNT5A/ID4 40

BP GO:0007019 microtubule depolymerization 9/1105 46/18866 0.001212257 0.021416541 0.017435436 CLASP1/TAOK1/SPAST/CKAP5/MAP1B/ARHGEF2/CKAP2/TPX2/KIF2C 9

BP GO:0009152 purine ribonucleotide biosynthetic process 21/1105 173/18866 0.001223907 0.021512274 0.017513373 SLC26A2/PANK2/PAPSS1/STAT3/ADCY3/PANK3/ACSL4/GUCY1A2/GMPS/PPAT/GART/PAICS/ELOVL5/ELOVL1/CBR4/ADCY5/PANK1/ELOVL3/ACSL1/HSD17B8/ACSS1 21

BP GO:1902903 regulation of supramolecular fiber organization 37/1105 373/18866 0.001234616 0.021512274 0.017513373 ROCK2/AKAP13/CLASP1/TAOK1/MAPRE1/PAK2/ACTR2/SSH1/NRP1/SPAST/CKAP5/ACTR3/ARF6/MAP1B/MTPN/FLNA/ARHGEF2/PIK3R1/ARFGEF1/TGFBR1/TGFB3/PLEKHG2/FMN1/GRB2/HSPA8/RAC1/CKAP2/CX3CL1/TSC1/MAPT/TPX2/GBA2/ARPC3/PLEKHH2/CCL26/ARHGEF10L/ARPC1B

37

BP GO:0010506 regulation of autophagy 35/1105 347/18866 0.001238184 0.021512274 0.017513373 USP13/SH3GLB1/RAB3GAP1/EIF2AK4/ABL2/STAT3/FEZ2/GNAI3/MTDH/PAFAH1B2/EIF4G2/BCL2L11/NEDD4/RB1CC1/EXOC4/SNX6/CSNK2A2/TBK1/TRIM5/VPS26B/SEC22B/UBQLN4/EIF4G1/PRKAB2/ATG7/CAMKK2/ATG5/ITPR1/IKBKG/BCL2/RNF152/TSC1/MAPT/MEFV/PRKAB1 35

BP GO:0016579 protein deubiquitination 30/1105 283/18866 0.001238369 0.021512274 0.017513373 USP13/USP47/USP9X/JOSD1/PSMD12/USP32/TRRAP/SART3/PSMD10/PSMD1/STAMBP/VCPIP1/SMAD1/BIRC2/PSMF1/PSMD11/PSME4/RHOT1/TGFBR1/USP15/PSMD2/PSME3/ATXN7L3/PSMD3/BRCA1/CYLD/USP54/USP18/IKBKG/SPATA2 30

BP GO:2000045 regulation of G1/S transition of mitotic cell cycle 22/1105 185/18866 0.001244021 0.021512274 0.017513373 ADAM17/LSM11/CNOT8/CNOT2/CUL4B/PRKDC/CDC73/SENP2/RBL1/CDK2/EIF4G1/PSME3/PML/EGFR/BCL2/FAM107A/CCNB1/KIF14/CHEK2/AURKA/HYAL1/GTSE1 22

BP GO:0055057 neuroblast division 5/1105 15/18866 0.001251241 0.021512274 0.017513373 RAB10/DOCK7/ARHGEF2/SOX5/LEF1 5

BP GO:0090110 COPII-coated vesicle cargo loading 5/1105 15/18866

0.001251241 0.021512274 0.017513373 SEC23A/TBC1D20/SAR1A/MIA3/RAB1A 5

BP GO:1904874 positive regulation of telomerase RNA localization to Cajal body 5/1105 15/18866 0.001251241 0.021512274 0.017513373 CCT8/CCT4/CCT7/DKC1/CCT2 5

BP GO:0006164 purine nucleotide biosynthetic process 23/1105 197/18866 0.001252467 0.021512274 0.017513373 SLC26A2/PANK2/PAPSS1/STAT3/ADCY3/PANK3/ACSL4/GUCY1A2/GMPS/PPAT/PRPS1/GART/PAICS/ELOVL5/ELOVL1/CBR4/ADCY5/PANK1/ELOVL3/ADA/ACSL1/HSD17B8/ACSS1 23

BP GO:0002758 innate immune response-activating signal transduction 16/1105 117/18866 0.001302272 0.022298901 0.018153775 PAK2/BCL10/PSMD12/NRAS/CUL1/PSMD10/PSMD1/PSMF1/RAF1/PSMD11/PSME4/PSMD2/PSME3/PSMD3/LYN/IKBKG 16

BP GO:0009150 purine ribonucleotide metabolic process 39/1105 401/18866 0.001335813 0.022803069 0.018564224 NUP98/SLC26A2/PANK2/PAPSS1/STAT3/NUP155/PGM2L1/ADCY3/GNAI3/PANK3/RAB23/NUP205/ACSL4/GUCY1A2/NT5E/ACOT9/GMPS/PPAT/GART/CASK/PGK1/NUP93/PAICS/NUP107/PDE4B/PFKFB3/ELOVL5/ELOVL1/CBR4/ADCY5/PDE10A/PANK1/ELOVL3/HMGCL/TPST2/ACSL1/HSD17B8/TDO2/ACSS1 39

BP GO:0006303 double-strand break repair via nonhomologous end joining

14/1105 96/18866 0.001369102 0.023299851 0.018968659 XRCC5/PRKDC/DCLRE1B/UBE2V2/UBE2N/SMARCAL1/TFIP11/NBN/POT1/MLH1/XRCC4/BRCA1/DTX3L/PARP9 14

BP GO:0072655 establishment of protein localization to mitochondrion 18/1105 140/18866 0.001384291 0.023486528 0.019120635 SH3GLB1/YWHAQ/PPP3R1/YWHAB/TP53BP2/BCL2L11/AP3B1/YWHAZ/NPEPPS/SAE1/CSNK2A2/RHOU/LMAN1/NMT1/PPP1R13B/BCL2/MAPT/PMPCA 18

BP GO:0042180 cellular ketone metabolic process 28/1105 260/18866 0.001397842 0.023644346 0.019249116 PANK2/PSMD12/ATP2B4/ADIPOR1/AZIN1/PSMD10/PSMD1/INSIG2/H6PD/PSMF1/PSMD11/PSME4/PRKAB2/PSMD2/PSME3/NMT1/PSMD3/BRCA1/ELOVL5/NCOR2/CBR4/SIRT5/IRS2/KMO/PPARG/TDO2/FABP5/COQ6 28

BP GO:0060249 anatomical structure homeostasis 44/1105 469/18866 0.001406125 0.023712383 0.019304506 ITGB1/VCL/YAP1/XRCC5/TAOK1/PRKDC/LAMC1/WDR36/DCLRE1B/DCP2/PTPN11/CCT8/F2R/CCT4/CCT7/NEK7/TERF1/PRIM1/POLD3/SMARCAL1/TFIP11/ATP2B1/RAD51C/P2RY1/NBN/SP100/PTGES3/DKC1/STRAP/POT1/CCT2/RAB3D/PML/EGFR/LAMP2/RAC1/RFC5/HMBOX1/BCL2/LOX/STK39/CX3CL1/TUB/VSIG1 44

BP GO:0030330 DNA damage response, signal transduction by p53 class mediator 15/1105 107/18866 0.001413462 0.023764089 0.0193466

SPRED1/CNOT8/CNOT2/PSMD10/CDK2/NBN/SP100/BRCA1/PML/CD44/CCNB1/FOXM1/CHEK2/AURKA/GTSE1 15

BP GO:0006402 mRNA catabolic process 37/1105 376/18866 0.001424111 0.023871015 0.01943365 ROCK2/YWHAB/PELO/CNOT8/TNPO1/CNOT2/MAPK14/ETF1/PSMD12/GSPT1/YTHDF3/FXR1/DCP2/PSMD10/YWHAZ/PSMD1/XRN2/PAIP1/PSMF1/KHSRP/PSMD11/EIF4G1/PSME4/NCBP2/DCP1B/PSMD2/ZFP36L1/PSME3/MLH1/ZHX2/PSMD3/MYD88/HSPA8/RPS23/SECISBP2/CIRBP/MYEF2 37

BP GO:0009791 post-embryonic development 13/1105 86/18866 0.001435913 0.02394705 0.019495551 HEG1/FBN1/MYO1E/BCL2L11/TGFBR1/PLAGL2/ITPR1/SGPL1/HMGN1/BCL2/SOX6/TAL2/SEMA3C 13

BP GO:0018279 protein N-linked glycosylation via asparagine 7/1105 30/18866 0.001437253 0.02394705 0.019495551 MGAT5/MCFD2/UBE2J1/STT3A/LMAN1/STT3B/DPM17

BP GO:0032922 circadian regulation of gene expression 11/110566/18866 0.001459697 0.02417624 0.019682137 TOP1/GFPT1/NRIP1/HDAC1/PPP1CC/HDAC3/AHR/PML/PER3/CRY2/ID4 11

BP GO:0034394 protein localization to cell surface 11/110566/18866 0.001459697 0.02417624 0.019682137 VCL/PIGK/ARF6/AP3B1/FLNA/GBF1/HFE/EMP2/STX3/ASTN2/NEDD4L

11

BP GO:0006278 RNA-dependent DNA biosynthetic process 12/1105 76/18866 0.001471484 0.024299141 0.019782192 XRCC5/DCP2/CCT8/CCT4/CCT7/NEK7/TERF1/PTGES3/DKC1/POT1/CCT2/HMBOX1 12

BP GO:0044774 mitotic DNA integrity checkpoint 15/1105 108/18866 0.001554561 0.025595064 0.020837217 CNOT8/MSH2/CNOT2/TAOK1/PRKDC/ATF2/TOPBP1/CDK2/NBN/PML/RINT1/CCNB1/CHEK2/AURKA/GTSE1 15

BP GO:0048016 inositol phosphate-mediated signaling 10/1105 57/18866 0.001587988 0.02599162 0.021160058 PPP3R1/NFATC3/ATP2B4/PPP3CA/LMCD1/PRNP/ITPR1/FHL2/CAMTA1/NRG1 10

BP GO:0070936 protein K48-linked ubiquitination 10/1105 57/18866 0.001587988 0.02599162 0.021160058 UBE2G1/RNF4/BFAR/BIRC2/UBE2A/UBE2E1/RNF34/DTX3L/RNF152/NEDD4L 10

BP GO:0072521 purine-containing compound metabolic process 44/1105 472/18866 0.001593014 0.025997434 0.02116479 NUP98/SLC26A2/PANK2/PAPSS1/STAT3/NUP155/PGM2L1/ADCY3/GNAI3/PANK3/RAB23/NUP205/ACSL4/GUCY1A2/NT5E/ACOT9/GMPS/PPAT/PRPS1/GART/CASK/PGK1/NUP93/PAICS/NUP107/DPYD/PDE4B/PFKFB3/EL

OVL5/ELOVL1/CBR4/ADCY5/PDE10A/PANK1/ELOVL3/ADA/HMGCL/TPST2/ACSL1/HSD17B8/TDO2/ACSS1/SLC16A9/SLC22A12 44

BP GO:0048013 ephrin receptor signaling pathway 13/1105 87/18866 0.00159956 0.026027932 0.021189619 ROCK2/ACTR2/KALRN/ACTR3/PTPN11/NCSTN/ITSN1/EPHA3/LYN/RAC1/SDCBP/ARPC3/ARPC1B 13

BP GO:0007431 salivary gland development 8/1105 39/18866 0.001633013 0.026494803 0.021569704 NRP1/PLXND1/DAG1/TGFB3/EGFR/TWSG1/NTN4/SEMA3C 8

BP GO:0007369 gastrulation 22/1105 189/18866 0.001642283 0.026567742 0.021629084 BMPR2/CLASP1/ITGB1/EXT2/RNF2/CDC73/WNK1/ACVR1/ITGB5/EXOC4/SMAD1/DAG1/COL4A2/EXT1/ITGAV/COL12A1/TWSG1/FZD7/LEF1/LAMB3/WNT5A/ATOH8 22

BP GO:0007162 negative regulation of cell adhesion 31/1105 301/18866 0.001648126 0.026584982 0.021643119 MAP4K4/ADAM10/MIA3/WNK1/PLXND1/PTPN11/GTPBP4/PIK3R1/CASK/PAG1/CBFB/PRNP/HFE/CORO1C/TWSG1/IL4R/FZD7/RUNX1/SERPINE1/FAM107A/CX3CL1/CYP1B1/IL2RA/ERBB2/AJAP1/TGFBI/C1QTNF1/SERPINE2/CEBPB/PLXNB3/SPOCK1 31

BP GO:0035561 regulation of chromatin binding 6/1105 23/18866 0.001697352 0.027221229 0.022161095

SENP2/PPP3CA/TDG/DTX3L/PARP9/HMBOX1 6

BP GO:0071577 zinc ion transmembrane transport 6/1105 23/18866 0.001697352 0.027221229 0.022161095 SLC30A1/SLC30A6/SLC39A10/SLC30A5/SLC39A6/SLC39A11 6

BP GO:0043618 regulation of transcription from RNA polymerase II promoter in response to stress 15/1105 109/18866 0.001707255 0.027223135 0.022162647 ATF6/PSMD12/EGLN1/NEDD4/CUL2/PSMD10/PSMD1/MBTPS2/PSMF1/PSMD11/PSME4/PSMD2/PSME3/PSMD3/CEBPB 15

BP GO:2000278 regulation of DNA biosynthetic process 15/1105 109/18866 0.001707255 0.027223135 0.022162647 BMPR2/XRCC5/DCP2/CCT8/CCT4/CCT7/NEK7/TERF1/PTGES3/DKC1/POT1/CCT2/RFC5/HMBOX1/PPARG 15

BP GO:0051098 regulation of binding 36/1105 367/18866 0.001731521 0.027395693 0.022303128 CSNK1A1/NSD1/ADNP/ATP2A2/MAPRE1/NRP1/SENP2/PPP3CA/PLXND1/GTPBP4/TFIP11/IDE/P2RY1/EIF4G1/CEBPG/TGFBR1/SP100/TDG/SUMO3/HFE/CYLD/LDLRAP1/DTX3L/PARP9/HMBOX1/PLCL2/HABP4/LOX/LEF1/ZBTB7C/PPARG/GAS8/CTHRC1/WNT5A/PLAUR/AURKA 36

BP GO:0010820 positive regulation of T cell chemotaxis 5/1105 16/18866 0.001732845 0.027395693 0.022303128 ADAM17/ADAM10/WNK1/STK39/WNT5A 5

BP GO:0060628 regulation of ER to Golgi vesicle-mediated transport 5/1105 16/18866 0.001732845 0.027395693 0.022303128 TBC1D20/SAR1A/YIPF5/RNF139/RINT1 5

BP GO:2000116 regulation of cysteine-type endopeptidase activity 26/1105 239/18866 0.001761779 0.02764995 0.022510122 USP47/SENP1/ROBO1/PAK2/BCL10/BCL2L11/EIF2AK3/UACA/CAST/APAF1/F2R/CSNK2A1/RPS6KA3/BIRC2/RAF1/RNF34/DIABLO/PML/F3/CD44/LEF1/MAPT/AQP1/PPARG/MEFV/PLAUR 26

BP GO:0018196 peptidyl-asparagine modification 7/1105 31/18866 0.001763833 0.02764995 0.022510122 MGAT5/MCFD2/UBE2J1/STT3A/LMAN1/STT3B/DPM17

BP GO:0031114 regulation of microtubule depolymerization 7/1105 31/18866 0.001763833 0.02764995 0.022510122 CLASP1/TAOK1/SPAST/MAP1B/ARHGEF2/CKAP2/TPX2 7

BP GO:0060485 mesenchyme development 30/1105 290/18866 0.001812952 0.028340102 0.023071982 BMPR2/SPRED1/CLASP1/YAP1/NOLC1/ROBO1/NRP1/ACVR1/ADIPOR1/NEDD4/EXOC4/DAG1/BCL9L/TGFBR1/EDNRA/ZFP36L1/STRAP/ZFPM2/EPHA3/TGFB3/CORO1C/IL17RD/SDCBP/SEMA4B/BCL2/LEF1/SEMA3C/NRG1/WNT5A/WT1 30

BP GO:0043281 regulation of cysteine-type endopeptidase activity involved in apoptotic process 24/1105 215/18866 0.001839807 0.028370984

0.023097123 USP47/SENP1/ROBO1/PAK2/BCL10/BCL2L11/EIF2AK3/UACA/APAF1/F2R/CSNK2A1/RPS6KA3/BIRC2/RAF1/RNF34/DIABLO/PML/F3/CD44/LEF1/MAPT/AQP1/PPARG/PLAUR 24

BP GO:0010998 regulation of translational initiation by eIF2 alpha phosphorylation 4/1105 10/18866 0.001848241 0.028370984 0.023097123 EIF2AK4/EIF2AK2/DNAJC3/EIF2AK3 4

BP GO:0021932 hindbrain radial glia guided cell migration 4/1105 10/18866 0.001848241 0.028370984 0.023097123 ITGB1/FLNA/ARL13B/LEF1 4

BP GO:0036490 regulation of translation in response to endoplasmic reticulum stress 4/1105 10/18866 0.001848241 0.028370984 0.023097123 EIF2AK4/DNAJC3/EIF2AK3/EIF4G1 4

BP GO:0099532 synaptic vesicle endosomal processing 4/1105 10/18866 0.001848241 0.028370984 0.023097123 AP3B1/STX6/ITSN1/VAMP4 4

BP GO:1902946 protein localization to early endosome 4/1105 10/18866 0.001848241 0.028370984 0.023097123 ROCK2/MSN/NRP1/DTX3L 4

BP GO:0051057 positive regulation of small GTPase mediated signal transduction 12/1105 78/18866 0.001850614 0.028370984 0.023097123

MAP4K4/AKAP13/SHOC2/CRKL/ROBO1/NOTCH2/F2R/LYN/GRB2/RAC1/IRS2/NRG1 12

BP GO:0070585 protein localization to mitochondrion 18/1105 144/18866 0.001907661 0.029165196 0.023743699 SH3GLB1/YWHAQ/PPP3R1/YWHAB/TP53BP2/BCL2L11/AP3B1/YWHAZ/NPEPPS/SAE1/CSNK2A2/RHOU/LMAN1/NMT1/PPP1R13B/BCL2/MAPT/PMPCA 18

BP GO:0032527 protein exit from endoplasmic reticulum 9/1105 49/18866 0.001931427 0.029435706 0.023963925 TMEM30A/SAR1A/SEL1L/UBE2J1/EDEM1/OS9/RHBDD1/EDEM2/HM13 9

BP GO:0006631 fatty acid metabolic process 38/1105 396/18866 0.001941223 0.029435706 0.023963925 PANK2/QKI/PEX13/MAPK14/ACSL4/ADIPOR1/PLAA/PAM/INSIG2/MORC2/MYO5A/PRKAB2/PTGES3/GNPAT/BRCA1/ELOVL5/ELOVL1/SGPL1/PCCA/ALDH3A2/CBR4/CYP1B1/ETFDH/ACAA2/ELOVL3/IRS2/PPARG/SLC27A2/CYP4V2/ACSL1/ILVBL/AUH/HSD17B8/PRKAB1/FABP5/ACSS1/HAO2/BDH2 38

BP GO:0070482 response to oxygen levels 38/1105 396/18866 0.001941223 0.029435706 0.023963925 ROCK2/ADAM17/PSMD12/EGLN1/CUL2/PSMD10/PSMD1/APAF1/PAM/NPEPPS/SUV39H2/BIRC2/PSMF1/CAPN2/RAF1/PSMD11/PSME4/PGK1/ED

NRA/PSMD2/ZFP36L1/ATG7/PSME3/PSMD3/TGFB3/PML/ITPR1/CYB5R4/BCL2/PLOD2/DDAH1/P4HB/CCNB1/ACAA2/TSC1/AQP1/ADA/PPARG 38

BP GO:0071772 response to BMP 20/1105 168/18866 0.001999546 0.030155752 0.024550122 BMPR2/PELO/USP9X/FBN1/ZFYVE16/NOTCH2/ACVR1/SKIL/SMAD1/USP15/RGMB/UBE2O/SULF1/HFE/TGFB3/SLC33A1/TWSG1/LEF1/ROR2/WNT5A 20

BP GO:0071773 cellular response to BMP stimulus 20/1105 168/18866 0.001999546 0.030155752 0.024550122 BMPR2/PELO/USP9X/FBN1/ZFYVE16/NOTCH2/ACVR1/SKIL/SMAD1/USP15/RGMB/UBE2O/SULF1/HFE/TGFB3/SLC33A1/TWSG1/LEF1/ROR2/WNT5A 20

BP GO:0036503 ERAD pathway 14/1105 100/18866 0.002035858 0.03062041 0.024928405 USP13/EDEM3/RNF103/SEL1L/UBE2J1/EDEM1/DNAJC10/OS9/RHBDD1/RNF139/SGTB/STT3B/EDEM2/HM13 14

BP GO:0006446 regulation of translational initiation 12/1105 79/18866 0.002068247 0.030928394 0.025179138 EIF2AK4/EIF2AK2/RPS6KB1/EIF4G2/DNAJC3/YTHDF3/EIF2AK3/PAIP1/RXRA/EIF4G1/NCBP2/HABP4 12

BP GO:0072413 signal transduction involved in mitotic cell cycle checkpoint 10/1105 59/18866 0.002078566 0.030928394 0.025179138

CNOT8/CNOT2/PRKDC/CDK2/PML/RINT1/CCNB1/CHEK2/AURKA/GTSE1 10

BP GO:1902402 signal transduction involved in mitotic DNA damage checkpoint 10/1105 59/18866 0.002078566 0.030928394 0.025179138 CNOT8/CNOT2/PRKDC/CDK2/PML/RINT1/CCNB1/CHEK2/AURKA/GTSE1 10

BP GO:1902403 signal transduction involved in mitotic DNA integrity checkpoint 10/1105 59/18866 0.002078566 0.030928394 0.025179138 CNOT8/CNOT2/PRKDC/CDK2/PML/RINT1/CCNB1/CHEK2/AURKA/GTSE1 10

BP GO:2000573 positive regulation of DNA biosynthetic process 11/1105 69/18866 0.002112522 0.031349827 0.025522231 XRCC5/CCT8/CCT4/CCT7/NEK7/PTGES3/DKC1/POT1/CCT2/RFC5/HMBOX1 11

BP GO:0046949 fatty-acyl-CoA biosynthetic process 7/1105 32/18866 0.002145185 0.031665663 0.025779357 ACSL4/ELOVL5/ELOVL1/CBR4/ELOVL3/ACSL1/HSD17B8 7

BP GO:0097421 liver regeneration 7/1105 32/18866 0.002145185 0.031665663 0.025779357 CSNK2A2/HFE/EGFR/SULF2/PTPN3/CEBPB/AURKA 7

BP GO:0032469 endoplasmic reticulum calcium ion homeostasis 6/1105 24/18866 0.00215229 0.031686494 0.025796315 ATP2A2/THADA/PML/ITPR1/BCL2/KCTD17 6

BP GO:0006457 protein folding 25/1105 230/18866 0.002160646 0.031725579 0.025828135 GANAB/ATF6/FKBP9/GNAI3/DNAJC10/DNAJC3/TOR1B/CCT8/SGTB/CCT4/CCT7/CANX/CSNK2A1/CSNK2A2/NUDCD3/RP2/VBP1/PTGES3/CRTAP/LMAN1/CCT2/LMAN2L/HSPA8/P4HB/BAG1 25

BP GO:0052547 regulation of peptidase activity 43/1105 466/18866 0.002169736 0.031775216 0.025868545 ROCK2/USP47/STAT3/SENP1/MAPK14/ROBO1/PAK2/BCL10/BCL2L11/EIF2AK3/HDAC1/UACA/CAST/APAF1/F2R/CSNK2A1/RPS6KA3/BIRC2/PSMF1/RAF1/PSME4/UBE2Z/PSME3/UBE2O/PRNP/RNF34/APLP2/DIABLO/PML/LYN/F3/SERPINE1/ECM1/CD44/LEF1/MAPT/SERPINE2/AQP1/PPARG/MEFV/PLAUR/TIMP1/SPOCK1 43

BP GO:0048675 axon extension 16/1105 123/18866 0.002207463 0.032242862 0.02624926 BMPR2/ITGB1/VCL/RAPH1/ADNP/USP9X/CDKL5/NRP1/WDR36/RYK/MAP1B/SEMA4B/MAPT/SEMA3C/ANAPC2/WNT5A 16

BP GO:0050673 epithelial cell proliferation 42/1105 453/18866 0.002222725 0.032348592 0.026335336 BMPR2/ADAM17/YAP1/STAT3/BTBD10/PRKDC/LAMC1/ROBO1/CDC73/

NRP1/AGGF1/NOTCH2/ATF2/NRAS/MAP2K1/SPARC/IFT52/NCSTN/CASK/TGFBR1/ZFP36L1/STRAP/SULF1/EGFR/FZD7/HMGN1/TGFA/F3/SULF2/ECM1/AGTR1/ERBB2/IRF6/SCG2/PPARG/CEBPB/CCL26/WNT5A/PLXNB3/PTPRN/ATOH8/HYAL1 42

BP GO:0034620 cellular response to unfolded protein 18/1105 146/18866 0.002226327 0.032348592 0.026335336 ATF6/EIF2AK2/BCL2L11/EDEM1/DNAJC3/GOSR2/CREB3L2/GFPT1/EIF2AK3/RHBDD1/SSR1/MBTPS2/CANX/PIK3R1/BFAR/HSPA8/IGFBP1/CXXC1 18

BP GO:0046822 regulation of nucleocytoplasmic transport 15/1105 112/18866 0.002242314 0.03242802 0.026399999 PTPN14/NOLC1/MAPK14/RIOK2/RAB23/NEDD4/PTPN11/FLNA/PIK3R1/PPP1CC/HDAC3/SP100/NCBP2/NSUN2/GTSE1 15

BP GO:0045088 regulation of innate immune response 31/1105 307/18866 0.002243448 0.03242802 0.026399999 XRCC5/PRKDC/PAK2/BCL10/PSMD12/ADAR/NRAS/YTHDF3/CUL1/PSMD10/PTPN11/PSMD1/TBK1/TRIM5/BIRC2/PSMF1/RAF1/PSMD11/PSME4/PSMD2/PSME3/IFNGR1/PSMD3/LYN/USP18/IKBKG/PARP9/PPARG/WNT5A/LRP8/SLAMF8 31

BP GO:0050852 T cell receptor signaling pathway 23/1105 206/18866 0.002257507 0.0324626 0.026428152 CRKL/PAK2/BCL10/PSMD12/WNK1/CUL1/PSMD10/PSMD1/UBE2N/PIK

3R1/PSMF1/PSMD11/PAG1/PSME4/PSMD2/PSME3/PRNP/PSMD3/CYLD/PDE4B/IKBKG/BTNL9/ADA 23

BP GO:1902806 regulation of cell cycle G1/S phase transition 23/1105 206/18866 0.002257507 0.0324626 0.026428152 ADAM17/LSM11/CNOT8/CNOT2/CUL4B/PRKDC/CDC73/ATP2B4/SENP2/RBL1/CDK2/EIF4G1/PSME3/PML/EGFR/BCL2/FAM107A/CCNB1/KIF14/CHEK2/AURKA/HYAL1/GTSE1 23

BP GO:0071346 cellular response to interferon-gamma 21/1105 182/18866 0.002304747 0.033056481 0.026911636 KIF5B/ACTR2/ACTR3/TRIM5/VPS26B/RAB43/SP100/IFNGR1/PML/OAS3/OAS2/STXBP1/PARP9/CD44/CX3CL1/IRF6/CCL13/PPARG/CCL26/WNT5A/MT2A 21

BP GO:0002566 somatic diversification of immune receptors via somatic mutation 5/1105 17/18866 0.002337513 0.033354506 0.027154262 MSH6/MSH2/ADAR/MLH1/UNG 5

BP GO:0010819 regulation of T cell chemotaxis 5/1105 17/18866 0.002337513 0.033354506 0.027154262 ADAM17/ADAM10/WNK1/STK39/WNT5A 5

BP GO:0016570 histone modification 43/1105 468/18866 0.002350819 0.033426354 0.027212754 PHF20/NSD1/TRIP12/TAF5L/CUL4B/RNF2/CDC73/HAT1/BAZ1B/SETD7/ATF2/TRRAP/MIER1/PWP1/HDAC1/SART3/PCGF6/PHF20L1/HLCS/WDR7

0/MTA3/YEATS2/SUV39H2/PHF14/UBE2N/MORF4L2/INCENP/CDK2/HDAC3/UBE2A/USP15/UBE2E1/DNMT1/ATXN7L3/ATG5/BRCA1/DTX3L/SETD3/ZNF711/LEF1/CCNB1/CXXC1/AURKA 43

BP GO:0016197 endosomal transport 26/1105 244/18866 0.002354561 0.033426354 0.027212754 RAB6A/ANKRD50/TMEM87A/ZFYVE16/GOSR2/ARF6/EVI5/CLTC/RAB10/AP3B1/SNX6/STX6/VPS26B/ITSN1/GBF1/TBC1D23/VPS37A/UBE2O/CMTM6/VAMP4/CORO1C/VPS29/ARL4C/VPS37D/TINAGL1/DENND1C 26

BP GO:0035821 modulation of process of other organism 15/1105 113/18866 0.002448937 0.034526363 0.028108283 EIF2AK4/EIF2AK2/TBC1D20/BCL2L11/KPNA3/KPNB1/HDAC1/KPNA4/DAG1/RXRA/ATG7/ATG5/HSPA8/LEF1/AQP1 15

BP GO:0001959 regulation of cytokine-mediated signaling pathway 21/1105 183/18866 0.002463752 0.034526363 0.028108283 ADAM17/HIPK1/ROBO1/ADAR/YTHDF3/VRK2/PTPN11/TBK1/BIRC2/CBFB/IFNGR1/CYLD/USP18/IKBKG/PARP9/SPATA2/RUNX1/ECM1/IL1R2/PPARG/WNT5A 21

BP GO:0007051 spindle organization 21/1105 183/18866 0.002463752 0.034526363 0.028108283 CLASP1/MAPRE1/SPAST/MAP4/CKAP5/KPNB1/RNF4/CLTC/FLNA/NEK7/INCENP/HDAC3/MLH1/TUBB/CHD3/RCC1/CCNB1/TPPP/TPX2/CHEK2/AURKA 21

BP GO:0030177 positive regulation of Wnt signaling pathway 21/1105 183/18866 0.002463752 0.034526363 0.028108283 YAP1/USP47/CSNK1G3/CDC73/PSMD12/WNK1/PSMD10/PSMD1/DAAM2/CSNK2A1/PSMF1/PSMD11/PSME4/PSMD2/PSME3/SULF1/PSMD3/EGFR/SULF2/ROR2/WNT5A 21

BP GO:2001235 positive regulation of apoptotic signaling pathway 21/1105 183/18866 0.002463752 0.034526363 0.028108283 SERINC3/YWHAQ/PPP3R1/YWHAB/TP53BP2/PAK2/BCL10/BCL2L11/YWHAZ/APAF1/SKIL/PRKRA/TGFBR1/NMT1/PLAGL2/CYLD/PML/PPP1R13B/BCL2/MLLT11/PLAUR 21

BP GO:0006163 purine nucleotide metabolic process 41/1105 442/18866 0.002469271 0.034526363 0.028108283 NUP98/SLC26A2/PANK2/PAPSS1/STAT3/NUP155/PGM2L1/ADCY3/GNAI3/PANK3/RAB23/NUP205/ACSL4/GUCY1A2/NT5E/ACOT9/GMPS/PPAT/PRPS1/GART/CASK/PGK1/NUP93/PAICS/NUP107/PDE4B/PFKFB3/ELOVL5/ELOVL1/CBR4/ADCY5/PDE10A/PANK1/ELOVL3/ADA/HMGCL/TPST2/ACSL1/HSD17B8/TDO2/ACSS1 41

BP GO:1902905 positive regulation of supramolecular fiber organization 23/1105 208/18866 0.00255607 0.035650451 0.029023416 ROCK2/CLASP1/MAPRE1/ACTR2/NRP1/SPAST/CKAP5/ACTR3/ARF6/MAP1B/FLNA/TGFBR1/TGFB3/FMN1/GRB2/RAC1/CX3CL1/TSC1/MAPT/ARPC3/CCL26/ARHGEF10L/ARPC1B23

BP GO:0009161 ribonucleoside monophosphate metabolic process 9/1105 51/18866 0.002575788 0.035835652 0.029174189 NT5E/GMPS/PPAT/PRPS1/GART/CASK/PAICS/ADA/UCK1 9

BP GO:0050685 positive regulation of mRNA processing7/1105 33/18866 0.002587178 0.035904356 0.029230122 NUP98/CDC73/NCBP2/RBM3/HSPA8/CIRBP/CCNB1 7

BP GO:0016236 macroautophagy 31/1105 310/18866 0.002604786 0.035993969 0.029303077 SH3GLB1/RAB3GAP1/FEZ2/GNAI3/EI24/PAFAH1B2/RAB23/NEDD4/RB1CC1/PLAA/EXOC4/SNX6/CSNK2A1/RAB1A/CSNK2A2/TBK1/VPS26B/SEC22B/RAB43/UBQLN4/VPS37A/PRKAB2/ATG7/ATG12/MAP1LC3B/ATG5/LAMP2/IKBKG/TSC1/VPS37D/PRKAB1 31

BP GO:0030968 endoplasmic reticulum unfolded protein response 16/1105 125/18866 0.002606571 0.035993969 0.029303077 ATF6/EIF2AK2/BCL2L11/EDEM1/DNAJC3/GOSR2/CREB3L2/GFPT1/EIF2AK3/SSR1/MBTPS2/CANX/PIK3R1/BFAR/IGFBP1/CXXC1 16

BP GO:0035690 cellular response to drug 11/110571/18866 0.002666574 0.036324563 0.029572217 CRKL/PPP3CA/EFTUD2/RAP1B/PPAT/PRNP/EGFR/PDE4B/CHEK2/MT2A/SLAMF8 11

BP GO:0003279 cardiac septum development 15/1105 114/18866 0.002671071 0.036324563 0.029572217

PARVA/BMPR2/HEG1/NDST1/ROBO1/NRP1/NOTCH2/DCTN5/ACVR1/EGLN1/PLXND1/TGFBR1/ZFPM2/SEMA3C/WNT5A15

BP GO:0021537 telencephalon development27/1105 259/18866 0.002680503 0.036324563 0.029572217 KIF5B/SLC38A2/PEX13/CRKL/ROBO1/ATP2B4/RYK/HDAC1/XRN2/FLNA/ARL13B/CSNK2A2/GART/EXT1/EGFR/CORO1C/SECISBP2/LEF1/TSC1/AQP1/NRG1/KIF14/RTN4RL1/WNT5A/ID4/LRP8/EMX2 27

BP GO:0006509 membrane protein ectodomain proteolysis 8/1105 42/18866 0.002681894 0.036324563 0.029572217 ADAM17/BACE1/ADAM10/DAG1/NCSTN/ADAM9/PTPN3/TIMP1 8

BP GO:0071470 cellular response to osmotic stress 8/1105 42/18866 0.002681894 0.036324563 0.029572217 XRCC5/ARHGEF2/ZFP36L1/STK39/MAPK10/SLC25A23/AQP1/EFHD1 8

BP GO:0031331 positive regulation of cellular catabolic process 37/1105 390/18866 0.002682656 0.036324563 0.029572217 ROCK2/USP13/SH3GLB1/CSNK1A1/RAB3GAP1/MSN/EIF2AK4/CNOT8/GNAI3/MTDH/PAFAH1B2/BCL2L11/EDEM1/TMTC3/YTHDF3/RNF139/PSMD10/COP1/TBK1/TRIM5/KHSRP/SUMO2/FBXW8/ADAM9/ZFP36L1/ATG7/CAMKK2/ATG5/PFKFB3/IKBKG/DTX3L/EDEM2/RNF152/TSC1/IRS2/MEFV/AURKA 37

BP GO:1903078 positive regulation of protein localization to plasma

membrane 10/1105 61/18866 0.002684357 0.036324563 0.029572217 ITGB1/KIF5B/ATP2B4/ARF6/PIK3R1/PRNP/EPHA3/EGFR/STX3/ARHGEF1610

BP GO:0036010 protein localization to endosome 6/1105 25/18866 0.00269343 0.036324563 0.029572217 ROCK2/TMEM30A/MSN/NRP1/ARF6/DTX3L 6

BP GO:0051894 positive regulation of focal adhesion assembly 6/1105 25/18866 0.00269343 0.036324563 0.029572217 MAP4K4/IQGAP1/NRP1/FMN1/RAC1/TSC1 6

BP GO:0008630 intrinsic apoptotic signaling pathway in response to DNA damage 14/1105 103/18866 0.002695785 0.036324563 0.029572217 MSH6/HIPK1/USP47/MSH2/PRKDC/BCL2L11/SKIL/PIK3R1/MLH1/BRCA1/PML/BCL2/CD44/CHEK2 14

BP GO:0009612 response to mechanical stimulus 23/1105 209/18866 0.002717465 0.036528238 0.029738031 BACE1/SLC38A2/MAPK14/BCL10/MAP3K2/MAP1B/PTPN11/MTPN/DAG1/RAF1/P2RY1/EGFR/MYD88/RAC1/ATP1A1/CLCN6/HABP4/CCNB1/SERPINE2/AQP1/PPARG/CASP5/NRXN2 23

BP GO:0018401 peptidyl-proline hydroxylation to 4-hydroxy-L-proline 4/1105 11/18866 0.002770422 0.03697218 0.030099449

EGLN1/P4HA1/P4HA3/P4HB 4

BP GO:0070417 cellular response to cold 4/1105 11/18866 0.002770422 0.03697218 0.030099449 EIF2AK4/DNAJC3/EIF2AK3/RNF34 4

BP GO:0090309 positive regulation of DNA methylation-dependent heterochromatin assembly 4/1105 11/18866 0.002770422 0.03697218 0.030099449 MORC2/ATF7IP/PPHLN1/DNMT1 4

BP GO:0030900 forebrain development 37/1105 391/18866 0.002801139 0.037292674 0.030360367 RAB3GAP1/KIF5B/SLC38A2/NDST1/PEX13/CRKL/ROBO1/NRP1/RRM1/ATP2B4/RYK/HDAC1/DOCK7/APAF1/XRN2/FLNA/ARL13B/MYO1D/CSNK2A2/GART/EXT1/NOTCH3/EGFR/CORO1C/TWSG1/SECISBP2/LEF1/TSC1/AQP1/TAL2/NRG1/KIF14/RTN4RL1/WNT5A/ID4/LRP8/EMX2 37

BP GO:1903313 positive regulation of mRNA metabolic process 12/1105 82/18866 0.002849786 0.037849782 0.030813914 ROCK2/NUP98/CNOT8/CDC73/YTHDF3/KHSRP/NCBP2/ZFP36L1/RBM3/HSPA8/CIRBP/CCNB1 12

BP GO:0071453 cellular response to oxygen levels 25/1105 235/18866 0.00288699 0.038252623 0.031141872 ROCK2/PSMD12/EGLN1/CUL2/PSMD10/PSMD1/NPEPPS/SUV39H2/PSMF1/PSMD11/PSME4/PGK1/PSMD2/ZFP36L1/ATG7/PSME3/PSMD3/BCL2/DDAH1/P4HB/CCNB1/ACAA2/TSC1/AQP1/PPARG 25

BP GO:0033865 nucleoside bisphosphate metabolic process 17/1105 138/18866 0.002934449 0.038514646 0.031355187 SLC26A2/PANK2/PAPSS1/PANK3/ACSL4/ACOT9/ELOVL5/ELOVL1/CBR4/PANK1/ELOVL3/HMGCL/TPST2/ACSL1/HSD17B8/TDO2/ACSS1 17

BP GO:0033875 ribonucleoside bisphosphate metabolic process 17/1105 138/18866 0.002934449 0.038514646 0.031355187 SLC26A2/PANK2/PAPSS1/PANK3/ACSL4/ACOT9/ELOVL5/ELOVL1/CBR4/PANK1/ELOVL3/HMGCL/TPST2/ACSL1/HSD17B8/TDO2/ACSS1 17

BP GO:0034032 purine nucleoside bisphosphate metabolic process 17/1105 138/18866 0.002934449 0.038514646 0.031355187 SLC26A2/PANK2/PAPSS1/PANK3/ACSL4/ACOT9/ELOVL5/ELOVL1/CBR4/PANK1/ELOVL3/HMGCL/TPST2/ACSL1/HSD17B8/TDO2/ACSS1 17

BP GO:0055076 transition metal ion homeostasis 17/1105 138/18866 0.002934449 0.038514646 0.031355187 CAND1/SLC30A1/SLC39A10/EGLN1/SLC30A5/CUL1/HEPH/SLC30A7/SLC39A6/PRNP/HFE/CYBRD1/CP/STEAP3/MT2A/BDH2/MT1X 17

BP GO:0000726 non-recombinational repair 14/1105 104/18866 0.002951347 0.038527535 0.03136568 XRCC5/PRKDC/DCLRE1B/UBE2V2/UBE2N/SMARCAL1/TFIP11/NBN/POT1/MLH1/XRCC4/BRCA1/DTX3L/PARP9 14

BP GO:0006998 nuclear envelope organization 9/1105 52/18866 0.002956201 0.038527535 0.03136568

NUP155/REEP3/SPAST/TOR1B/PARP11/TMEM43/NUP93/CCNB1/CCNB2 9

BP GO:0090329 regulation of DNA-dependent DNA replication 9/1105 52/18866 0.002956201 0.038527535 0.03136568 SENP2/ZRANB3/TERF1/SMARCAL1/CDK2/NBN/ATG7/RFC5/CHEK2 9

BP GO:0006403 RNA localization 25/1105 236/18866 0.003054689 0.039555389 0.032202468 NUP98/NUP155/QKI/AHCTF1/RIOK2/SENP2/NUP205/CKAP5/DCP2/CCT8/CCT4/CCT7/TERF1/PARP11/KHSRP/THOC5/NCBP2/NSUN2/DKC1/ZFP36L1/NUP93/CCT2/NXT2/NUP107/TSC1 25

BP GO:1900180 regulation of protein localization to nucleus 16/1105 127/18866 0.003063781 0.039555389 0.032202468 NOLC1/MAPK14/RAB23/EIF2AK3/CCT8/CCT4/CCT7/FLNA/PIK3R1/HDAC3/DKC1/CCT2/SUMO3/DTX3L/PARP9/GTSE1 16

BP GO:0050679 positive regulation of epithelial cell proliferation 23/1105 211/18866 0.003066183 0.039555389 0.032202468 BMPR2/ADAM17/YAP1/STAT3/LAMC1/NRP1/AGGF1/NOTCH2/NRAS/TGFBR1/EGFR/FZD7/TGFA/F3/ECM1/AGTR1/ERBB2/SCG2/CCL26/WNT5A/PLXNB3/PTPRN/HYAL1 23

BP GO:0010766 negative regulation of sodium ion transport 5/1105 18/18866 0.003082086 0.039555389 0.032202468 WNK1/NEDD4/STK39/SERPINE2/NEDD4L 5

BP GO:0031293 membrane protein intracellular domain proteolysis 5/1105 18/18866 0.003082086 0.039555389 0.032202468 ADAM17/ADAM10/RHBDD1/MBTPS2/NCSTN 5

BP GO:0006891 intra-Golgi vesicle-mediated transport 7/1105 34/18866 0.003095897 0.039555389 0.032202468 RAB6A/COPA/GOSR2/COPB1/COG5/COPB2/NSF 7

BP GO:0009156 ribonucleoside monophosphate biosynthetic process 7/1105 34/18866 0.003095897 0.039555389 0.032202468 GMPS/PPAT/PRPS1/GART/PAICS/ADA/UCK1 7

BP GO:0031112 positive regulation of microtubule polymerization or depolymerization 7/1105 34/18866 0.003095897 0.039555389 0.032202468 CLASP1/MAPRE1/SPAST/CKAP5/MAP1B/RAC1/MAPT 7

BP GO:0052548 regulation of endopeptidase activity 40/1105 434/18866 0.003099039 0.039555389 0.032202468 ROCK2/USP47/STAT3/SENP1/ROBO1/PAK2/BCL10/BCL2L11/EIF2AK3/HDAC1/UACA/CAST/APAF1/F2R/CSNK2A1/RPS6KA3/BIRC2/PSMF1/RAF1/UBE2Z/PSME3/UBE2O/PRNP/RNF34/APLP2/DIABLO/PML/LYN/F3/SERPINE1/CD44/LEF1/MAPT/SERPINE2/AQP1/PPARG/MEFV/PLAUR/TIMP1/SPOCK1 40

BP GO:0006520 cellular amino acid metabolic process 33/1105 340/18866 0.003109408 0.039596929 0.032236285

SERINC3/SERINC5/PSMD12/ATP2B4/SLC16A2/GFPT1/AZIN1/PSMD10/GCLM/PSMD1/GMPS/PPAT/GART/PSMF1/PSMD11/PSME4/CTPS2/PSMD2/PSME3/DPYD/PSMD3/PLOD2/DDAH1/ALDH6A1/BPHL/HIBCH/KMO/HMGCL/ILVBL/AUH/TDO2/AMDHD1/GLYATL1 33

BP GO:0046916 cellular transition metal ion homeostasis 15/1105 116/18866 0.003165372 0.040217574 0.032741559 CAND1/SLC30A1/SLC39A10/EGLN1/SLC30A5/CUL1/HEPH/SLC30A7/SLC39A6/PRNP/HFE/CYBRD1/CP/MT2A/MT1X 15

BP GO:0050678 regulation of epithelial cell proliferation 37/1105 395/18866 0.003321152 0.041734724 0.033976687 BMPR2/ADAM17/YAP1/STAT3/PRKDC/LAMC1/ROBO1/CDC73/NRP1/AGGF1/NOTCH2/ATF2/NRAS/SPARC/IFT52/CASK/TGFBR1/ZFP36L1/STRAP/SULF1/EGFR/FZD7/HMGN1/TGFA/F3/SULF2/ECM1/AGTR1/ERBB2/SCG2/PPARG/CCL26/WNT5A/PLXNB3/PTPRN/ATOH8/HYAL1 37

BP GO:0033522 histone H2A ubiquitination 6/1105 26/18866 0.003330399 0.041734724 0.033976687 TRIP12/CUL4B/RNF2/PCGF6/UBE2A/DTX3L 6

BP GO:0006487 protein N-linked glycosylation 11/110573/18866 0.003332288 0.041734724 0.033976687 MGAT5/MCFD2/UBE2J1/GFPT1/STT3A/LMAN1/PGM3/STT3B/DPM1/ST3GAL1/DPAGT1 11

BP GO:0072401 signal transduction involved in DNA integrity checkpoint

11/110573/18866 0.003332288 0.041734724 0.033976687 CNOT8/CNOT2/PRKDC/CDK2/BRCA1/PML/RINT1/CCNB1/CHEK2/AURKA/GTSE1 11

BP GO:0072422 signal transduction involved in DNA damage checkpoint 11/110573/18866 0.003332288 0.041734724 0.033976687 CNOT8/CNOT2/PRKDC/CDK2/BRCA1/PML/RINT1/CCNB1/CHEK2/AURKA/GTSE1 11

BP GO:0030705 cytoskeleton-dependent intracellular transport 22/1105 200/18866 0.003337278 0.041734724 0.033976687 RAB6A/KIF5B/MYO1B/TNPO1/MYO1E/AP3M1/SPAST/ARHGAP21/MAP1B/AP3B1/KIF3C/IFT52/MYO1D/RAB1A/MYO5A/RHOT1/DST/TUBB/HSPA8/MAPT/TUB/HOOK2 22

BP GO:1901888 regulation of cell junction assembly 22/1105 200/18866 0.003337278 0.041734724 0.033976687 ROCK2/MAP4K4/CLASP1/VCL/IQGAP1/ADNP/IL1RAP/NRP1/PDLIM5/ARF6/RAP1B/EIF4G1/CBFB/EPHA3/FMN1/CORO1C/RAC1/RUNX1/FAM107A/SRPX2/TSC1/WNT5A 22

BP GO:1903050 regulation of proteolysis involved in cellular protein catabolic process 24/1105 225/18866 0.003345555 0.041744429 0.033984588 USP13/CSNK1A1/SMARCC1/SENP1/GNA12/UBE2J1/EDEM1/TMTC3/OS9/RNF139/PSMD10/COP1/CSNK2A1/CSNK2A2/PSMF1/SUMO2/UBQLN4/

FBXW8/PSME3/HFE/PML/SDCBP/EDEM2/AURKA 24
BP GO:0019395 fatty acid oxidation 14/1105 106/18866 0.003522135 0.043849401 0.035698269 PEX13/MAPK14/ADIPOR1/ALDH3A2/ETFDH/ACAA2/IRS2/PPARG/SLC27A2/CYP4V2/ILVBL/AUH/HAO2/BDH2 14
BP GO:0032869 cellular response to insulin stimulus24/1105 226/18866 0.003541338 0.043990063 0.035812783 RPS6KB1/SMARCC1/PRKDC/ADIPOR1/RAB10/RAB8B/PTPN11/RAB31/INSIG2/PPAT/PIK3R1/IDE/ATP2B1/MYO5A/PHIP/ZFP36L1/GRB2/ZDHHC7/KL/TSC1/IRS2/IGFBP1/PPARG/GKAP1 24
BP GO:2000404 regulation of T cell migration 8/1105 44/18866 0.003631578 0.045010536 0.036643561 ADAM17/ADAM10/CRKL/WNK1/CD99L2/ECM1/STK39/WNT5A 8
BP GO:0006486 protein glycosylation 26/1105 252/18866 0.003654072 0.045041182 0.036668511 DPY19L3/EXT2/MAN1A2/EDEM3/MGAT5/MCFD2/UBE2J1/TMTC3/GFPT1/GALNT1/FUT4/STT3A/ST3GAL2/ARFGEF1/EXT1/LMAN1/PGM3/STT3B/MAN2A1/B4GALT3/DPM1/ST3GAL1/GALNT2/GALNT10/DPAGT1/POMT1 26
BP GO:0043413 macromolecule glycosylation 26/1105 252/18866 0.003654072 0.045041182 0.036668511 DPY19L3/EXT2/MAN1A2/EDEM3/MGAT5/MCFD2/UBE2J1/TMTC3/GFPT1/GALNT1/FUT4/STT3A/ST3GAL2/ARFGEF1/EXT1/LMAN1/PGM3/STT3B/

MAN2A1/B4GALT3/DPM1/ST3GAL1/GALNT2/GALNT10/DPAGT1/POMT1 26

BP GO:0001889 liver development 17/1105 141/18866 0.003673826 0.045041182 0.036668511 SP3/SEC63/NOTCH2/ARF6/RB1CC1/CSNK2A2/CEBPG/GNPNAT1/HFE/MAN2A1/EGFR/SULF2/ADA/PTPN3/HMGCL/CEBPB/AURKA 17

BP GO:0030010 establishment of cell polarity 17/1105 141/18866 0.003673826 0.045041182 0.036668511 ROCK2/CLASP1/ITGB1/MSN/CRKL/MAP4/GOLPH3/ARF6/RAB10/DOCK7/MAP1B/FRMD4A/RAP1B/ARHGEF2/ARFGEF1/GBF1/WNT5A 17

BP GO:0070085 glycosylation 27/1105 265/18866 0.003681694 0.045041182 0.036668511 DPY19L3/EXT2/MAN1A2/EDEM3/MGAT5/MCFD2/UBE2J1/TMTC3/GFPT1/GALNT1/FUT4/STT3A/ST3GAL2/ARFGEF1/EXT1/LMAN1/PGM3/STT3B/MAN2A1/B4GALT3/DPM1/ST3GAL1/GALNT2/GALNT10/DPAGT1/POMT1/GBA2 27

BP GO:0030324 lung development 20/1105 177/18866 0.003682612 0.045041182 0.036668511 BMPR2/HEG1/YAP1/MAN1A2/SP3/MAP2K1/SPARC/AP3B1/YWHAZ/DAG1/PHF14/ZFPM2/MAN2A1/TGFB3/EGFR/LOX/TMEM38B/ADA/HSD11B1/WNT5A 20

BP GO:0072395 signal transduction involved in cell cycle checkpoint 11/110574/18866 0.003711782 0.045298397 0.036877912

CNOT8/CNOT2/PRKDC/CDK2/BRCA1/PML/RINT1/CCNB1/CHEK2/AURKA/GTSE1 11

BP GO:0036293 response to decreased oxygen levels 35/1105 371/18866 0.00376416 0.045658701 0.03717124 ROCK2/ADAM17/PSMD12/EGLN1/CUL2/PSMD10/PSMD1/APAF1/PAM/NPEPPS/SUV39H2/BIRC2/PSMF1/CAPN2/RAF1/PSMD11/PSME4/PGK1/EDNRA/PSMD2/ZFP36L1/PSME3/PSMD3/TGFB3/PML/ITPR1/BCL2/PLOD2/DDAH1/P4HB/CCNB1/ACAA2/TSC1/AQP1/ADA 35

BP GO:0007179 transforming growth factor beta receptor signaling pathway 22/1105 202/18866 0.00376592 0.045658701 0.03717124 ADAM17/SPRED1/TGFBRAP1/USP9X/FBN1/ACVR1/ITGB5/SKIL/SMAD1/SNX6/BCL9L/ADAM9/TGFBR1/USP15/STRAP/TGFB3/PML/TWSG1/IL17RD/SDCBP/LOX/CGN 22

BP GO:0034341 response to interferon-gamma 22/1105 202/18866 0.00376592 0.045658701 0.03717124 KIF5B/ACTR2/ACTR3/TRIM5/VPS26B/RAB43/SP100/IFNGR1/PML/OAS3/OAS2/STXBP1/PARP9/CD44/CX3CL1/IRF6/CCL13/PPARG/MEFV/CCL26/WNT5A/MT2A 22

BP GO:0032886 regulation of microtubule-based process 25/1105 240/18866 0.003810233 0.045844207 0.037322261 ROCK2/CLASP1/TAOK1/MAPRE1/SPAST/CKAP5/RNF4/CLTC/MAP1B/ARHGEF2/RHOT1/EPHA3/BRCA1/CYLD/RAC1/CKAP2/FAM107A/ERBB2/RC

C1/TPPP/MAPT/PKHD1/TPX2/GBA2/AURKA 25

BP GO:0090174 organelle membrane fusion 14/1105 107/18866 0.00383957 0.045844207 0.037322261 KIF5B/TGFBRAP1/GNAI3/GOSR2/SNAP23/RAB8B/VCPIP1/STX6/SEC22B/SAMD9/VAMP4/STXBP1/STX2/STX3 14

BP GO:0099175 regulation of postsynapse organization 14/1105 107/18866 0.00383957 0.045844207 0.037322261 IL1RAP/ADAM10/CRKL/CDKL5/ACTR2/SSH1/KALRN/PDLIM5/EGLN1/LRP4/PRNP/HSPA8/WNT5A/LRP8 14

BP GO:0031050 dsRNA processing 9/1105 54/18866 0.003849197 0.045844207 0.037322261 TSN/STAT3/ADAR/MAP2K1/PPP3CA/SMAD1/PRKRA/EGFR/NCOR2 9

BP GO:0070918 production of small RNA involved in gene silencing by RNA 9/1105 54/18866 0.003849197 0.045844207 0.037322261 TSN/STAT3/ADAR/MAP2K1/PPP3CA/SMAD1/PRKRA/EGFR/NCOR2 9

BP GO:0032835 glomerulus development 10/1105 64/18866 0.003849469 0.045844207 0.037322261 IQGAP1/MYO1E/NOTCH2/NID1/NOTCH3/SULF1/SULF2/BCL2/COL4A4/WT1 10

BP GO:2000401 regulation of lymphocyte migration 10/1105 64/18866 0.003849469 0.045844207 0.037322261 ADAM17/MSN/ADAM10/CRKL/MIA3/WNK1/CD99L2/ECM1/STK39/WNT

5A 10

BP GO:0048511 rhythmic process 30/1105 305/18866 0.003853084 0.045844207 0.037322261 ROCK2/ADNP/USP9X/PRKDC/TOP1/GFPT1/NRIP1/HDAC1/PAM/SUV39H2/CSNK2A1/PPP1CC/HDAC3/ATG7/TGFB3/AHR/PML/EGFR/NCOR2/SGPL1/PER3/SERPINE1/CRY2/MMP19/HLF/MAPK10/ADA/PPARG/ID4/PTPRN 30

BP GO:0031110 regulation of microtubule polymerization or depolymerization 12/1105 85/18866 0.003855362 0.045844207 0.037322261 CLASP1/TAOK1/MAPRE1/SPAST/CKAP5/MAP1B/ARHGEF2/RAC1/CKAP2/MAPT/TPX2/GBA2 12

BP GO:0001667 ameboidal-type cell migration 43/1105 481/18866 0.003878942 0.046026247 0.037470463 ROCK2/BMPR2/ADAM17/MAP4K4/SPRED1/CLASP1/ITGB1/IQGAP1/STARD13/ROBO1/NRP1/CAP1/GNA12/MIA3/ATP2B4/DDR2/ACVR1/SPARC/ADIPOR1/ARF6/PLXND1/PTPN11/ADAM9/TGFBR1/SP100/STRAP/EMP2/ADAMTS9/PML/CORO1C/ARSB/SGPL1/SEMA4B/CYP1B1/SRPX2/SCG2/SEMA3C/PPARG/INSL3/WNT5A/TIMP1/ATOH8/HYAL1 43

BP GO:0030157 pancreatic juice secretion 4/1105 12/18866 0.003964379 0.046572092 0.037914841 COPA/STK39/NPR3/AQP1 4

BP GO:0033523 histone H2B ubiquitination 4/1105 12/18866 0.003964379 0.046572092 0.037914841 CDC73/WDR70/UBE2E1/DTX3L 4

BP GO:0033629 negative regulation of cell adhesion mediated by integrin 4/1105 12/18866 0.003964379 0.046572092 0.037914841 WNK1/PTPN11/SERPINE1/CYP1B1 4

BP GO:0007093 mitotic cell cycle checkpoint 19/1105 166/18866 0.00397093 0.046572092 0.037914841 CNOT8/MSH2/CNOT2/TAOK1/PRKDC/DCLRE1B/ATF2/TOPBP1/CDK2/NBN/BRCA1/PML/RINT1/CCNB1/CHEK2/BUB1/TRIP13/AURKA/GTSE1 19

BP GO:0035338 long-chain fatty-acyl-CoA biosynthetic process 5/1105 19/18866 0.003983525 0.046572092 0.037914841 ACSL4/ELOVL5/ELOVL1/ELOVL3/ACSL1 5

BP GO:0150146 cell junction disassembly 5/1105 19/18866 0.003983525 0.046572092 0.037914841 MAP4K4/ARF6/TGFBR1/TGFB3/CX3CL1 5

BP GO:2000104 negative regulation of DNA-dependent DNA replication 5/1105 19/18866 0.003983525 0.046572092 0.037914841 ZRANB3/TERF1/SMARCAL1/ATG7/CHEK2 5

BP GO:0001666 response to hypoxia 34/1105 359/18866 0.004004202 0.046715685 0.038031741 ROCK2/ADAM17/PSMD12/EGLN1/CUL2/PSMD10/PSMD1/APAF1/PAM/

NPEPPS/SUV39H2/BIRC2/PSMF1/CAPN2/RAF1/PSMD11/PSME4/PGK1/EDNRA/PSMD2/ZFP36L1/PSME3/PSMD3/TGFB3/PML/ITPR1/BCL2/PLOD2/DDAH1/P4HB/CCNB1/ACAA2/AQP1/ADA 34

BP GO:0003148 outflow tract septum morphogenesis 6/1105 27/18866 0.004073024 0.046928318 0.038204848 PARVA/BMPR2/ROBO1/NRP1/ZFPM2/SEMA3C 6

BP GO:0006516 glycoprotein catabolic process 6/1105 27/18866 0.004073024 0.046928318 0.038204848 EDEM3/EDEM1/STT3B/ADAMTS9/MANBA/EDEM2 6

BP GO:0007026 negative regulation of microtubule depolymerization 6/1105 27/18866 0.004073024 0.046928318 0.038204848 CLASP1/TAOK1/MAP1B/ARHGEF2/CKAP2/TPX2 6

BP GO:0009651 response to salt stress 6/1105 27/18866 0.004073024 0.046928318 0.038204848 XRCC5/ZFP36L1/SLC25A23/AQP1/KMO/EFHD1 6

BP GO:0035459 vesicle cargo loading 6/1105 27/18866 0.004073024 0.046928318 0.038204848 SEC23A/TMED10/TBC1D20/SAR1A/MIA3/RAB1A 6

BP GO:1900120 regulation of receptor binding 6/1105 27/18866 0.004073024 0.046928318 0.038204848 ATP2A2/NRP1/HFE/PLCL2/LOX/LEF1 6

BP GO:0003206 cardiac chamber morphogenesis 16/1105 131/18866

0.004177714 0.047776591 0.038895437 PARVA/BMPR2/HEG1/ROBO1/NRP1/NOTCH2/ACVR1/EGLN1/RXRA/TGFBR1/ZFPM2/CPE/FHL2/SEMA3C/NRG1/WNT5A 16

BP GO:0010508 positive regulation of autophagy 16/1105 131/18866 0.004177714 0.047776591 0.038895437 SH3GLB1/RAB3GAP1/EIF2AK4/GNAI3/MTDH/PAFAH1B2/BCL2L11/TBK1/TRIM5/ATG7/CAMKK2/ATG5/IKBKG/RNF152/TSC1/MEFV 16

BP GO:0002062 chondrocyte differentiation 14/1105 108/18866 0.00417989 0.047776591 0.038895437 BMPR2/CHSY1/MAPK14/CREB3L2/EIF2AK3/PTPN11/TGFBR1/SULF1/SOX5/RUNX1/SULF2/ECM1/SOX6/TGFBI 14

BP GO:0032956 regulation of actin cytoskeleton organization 34/1105 360/18866 0.004180988 0.047776591 0.038895437 ROCK2/AKAP13/CLASP1/IQGAP1/ABL2/TAOK1/PAK2/ACTR2/SSH1/NRP1/NOTCH2/ACTR3/ARF6/MTPN/PAM/FLNA/PIK3R1/ARFGEF1/TGFBR1/EPHA3/TGFB3/PLEKHG2/FMN1/GRB2/RAC1/FAM107A/CX3CL1/TSC1/GBA2/ARPC3/PLEKHH2/CCL26/ARHGEF10L/ARPC1B 34

BP GO:0060541 respiratory system development 22/1105 204/18866 0.00423963 0.048330331 0.039346242 BMPR2/HEG1/YAP1/MAN1A2/SP3/MAP2K1/SPARC/AP3B1/YWHAZ/DAG1/PHF14/ZFPM2/MAN2A1/TGFB3/EGFR/LOX/TMEM38B/LEF1/ADA/HSD11B1/WNT5A/WT1 22

BP GO:0050772 positive regulation of axonogenesis 12/1105 86/18866 0.004247705 0.048330331 0.039346242 BMPR2/ADNP/ROBO1/CDKL5/NRP1/MAP2K1/PLXND1/MAP1B/SKIL/MAPT/ANAPC2/PLXNB3 12

BP GO:1901216 positive regulation of neuron death 13/1105 97/18866 0.004255501 0.048330331 0.039346242 BACE1/TP53BP2/SSH1/BCL2L11/ATF2/SRPK2/CAPN2/PRNP/PPP1R13B/ZNF746/MAPT/KMO/WNT5A 13

BP GO:1990845 adaptive thermogenesis 18/1105 155/18866 0.004265457 0.048344738 0.039357971 ADAM17/THADA/ADIPOR1/LAMA4/DOCK7/LNPEP/HDAC3/PRKAB2/TFE3/IL4R/PLCL2/NPR3/ELOVL3/ACSL1/CEBPB/PRKAB1/ABHD6/FABP5 18

BP GO:0048278 vesicle docking 10/1105 65/18866 0.004316091 0.048720177 0.03966362 RAB10/RAB8B/EXOC4/SCFD2/STX6/RAB3D/NSF/STXBP1/STX2/STX3 10

BP GO:0051205 protein insertion into membrane 10/1105 65/18866 0.004316091 0.048720177 0.03966362 YWHAQ/PPP3R1/YWHAB/TP53BP2/BCL2L11/YWHAZ/NMT1/EGFR/PPP1R13B/BCL2 10

BP GO:0001569 branching involved in blood vessel morphogenesis 7/1105 36/18866 0.00433874 0.048876697 0.039791045

NRP1/GNA13/ACVR1/COL4A1/PLXND1/EDNRA/LEF1 7

BP GO:0001954 positive regulation of cell-matrix adhesion 9/1105 55/18866 0.004368357 0.049110919 0.039981727 MAP4K4/IQGAP1/NRP1/DAG1/EMP2/FMN1/RAC1/CX3CL1/TSC19

BP GO:0048608 reproductive structure development 40/1105 443/18866 0.004413539 0.049518843 0.040313822 BMPR2/SP3/TBC1D20/MSH2/SMARCC1/MAPK14/CRKL/BCL2L11/RRM1/NOTCH2/SENP2/EGLN1/MAP2K1/NRIP1/PTPN11/FLNA/RXRA/BIRC2/FBXW8/TGFBR1/ZFP36L1/SULF1/ZFPM2/NUP107/EGFR/GRB2/SGPL1/BCL2/MMP19/LEF1/DMRTA1/ROR2/SERPINE2/ADA/PPARG/CEBPB/WNT5A/WT1/ID4/PTPRN 40

BP GO:0030111 regulation of Wnt signaling pathway 35/1105 375/18866 0.004458947 0.049927644 0.040646632 CSNK1A1/YAP1/USP47/CSNK1G3/MAPK14/CDC73/PSMD12/SENP2/WNK1/HDAC1/PSMD10/PSMD1/DAAM2/LRP4/CSNK2A1/PSMF1/PSMD11/PSME4/CBFB/PSMD2/PSME3/SULF1/PSMD3/CYLD/EGFR/FZD7/RUNX1/SULF2/TMEM88/LEF1/ROR2/IGFBP1/CTHRC1/APCDD1L/WNT5A 35

BP GO:0035966 response to topologically incorrect protein 22/1105 205/18866 0.004494487 0.0501216 0.040804533 ATF6/EIF2AK2/EDEM3/BCL2L11/EDEM1/DNAJC3/TOR1B/GOSR2/CREB3L2/GFPT1/EIF2AK3/RHBDD1/SSR1/MBTPS2/CANX/PIK3R1/BFAR/STT3B/HSPA8/EDEM2/IGFBP1/CXXC1 22

BP GO:0006282 regulation of DNA repair 16/1105 132/18866 0.004502655 0.0501216 0.040804533 TRIP12/PRKDC/ACTR2/WDR70/UBE2V2/UBE2N/TFIP11/UBQLN4/CEBPG/POT1/BRCA1/PML/EGFR/DTX3L/PARP9/FOXM1 16

BP GO:0048872 homeostasis of number of cells 26/1105 256/18866 0.004503288 0.0501216 0.040804533 ADAM17/SP3/STAT3/SENP1/PRKDC/MAPK14/BCL10/BCL2L11/ADAR/SART3/PTPN11/SKIL/F2R/NCSTN/CEBPG/ZFP36L1/CYLD/LYN/PDE4B/FANCC/BCL2/IL2RA/SOX6/ADA/CCNB2/TNFSF13B 26

BP GO:0061008 hepaticobiliary system development 17/1105 144/18866 0.004560503 0.050455663 0.041076498 SP3/SEC63/NOTCH2/ARF6/RB1CC1/CSNK2A2/CEBPG/GNPNAT1/HFE/MAN2A1/EGFR/SULF2/ADA/PTPN3/HMGCL/CEBPB/AURKA 17

BP GO:0106106 cold-induced thermogenesis 17/1105 144/18866 0.004560503 0.050455663 0.041076498 ADAM17/ADIPOR1/LAMA4/DOCK7/LNPEP/HDAC3/PRKAB2/TFE3/IL4R/PLCL2/NPR3/ELOVL3/ACSL1/CEBPB/PRKAB1/ABHD6/FABP5 17

BP GO:0120161 regulation of cold-induced thermogenesis 17/1105 144/18866 0.004560503 0.050455663 0.041076498 ADAM17/ADIPOR1/LAMA4/DOCK7/LNPEP/HDAC3/PRKAB2/TFE3/IL4R/PLCL2/NPR3/ELOVL3/ACSL1/CEBPB/PRKAB1/ABHD6/FABP5 17

BP GO:0036294 cellular response to decreased oxygen levels 23/1105

218/18866 0.004597568 0.050764817 0.041328183 ROCK2/PSMD12/EGLN1/CUL2/PSMD10/PSMD1/NPEPPS/SUV39H2/PSMF1/PSMD11/PSME4/PGK1/PSMD2/ZFP36L1/PSME3/PSMD3/BCL2/DDAH1/P4HB/CCNB1/ACAA2/TSC1/AQP1 23

BP GO:0001822 kidney development 28/1105 283/18866 0.004725319 0.052072075 0.042392436 YAP1/IQGAP1/TMED10/FBN1/NRP1/MYO1E/BCL2L11/NOTCH2/NID1/APAF1/LRP4/SMAD1/PPAT/TGFBR1/NOTCH3/RRM2B/SULF1/NUP107/FMN1/SGPL1/SULF2/BCL2/COL4A4/FRAS1/AGTR1/TSC1/PKHD1/WT128

BP GO:0030323 respiratory tube development 20/1105 181/18866 0.004741967 0.052152263 0.042457718 BMPR2/HEG1/YAP1/MAN1A2/SP3/MAP2K1/SPARC/AP3B1/YWHAZ/DAG1/PHF14/ZFPM2/MAN2A1/TGFB3/EGFR/LOX/TMEM38B/ADA/HSD11B1/WNT5A 20

BP GO:0071383 cellular response to steroid hormone stimulus 22/1105 206/18866 0.004761952 0.052268764 0.042552563 AKAP13/YAP1/CNOT2/BCL2L11/NR3C1/NEDD4/HDAC1/RXRA/ATP2B1/CBFB/ZFP36L1/BRCA1/EGFR/PGRMC2/NCOR2/ATP1A1/RUNX1/NR3C2/FAM107A/CRY2/LEF1/AQP1 22

BP GO:0048538 thymus development 8/1105 46/18866 0.004822443 0.052828538 0.043008281 PRKDC/CRKL/BCL2L11/MAP2K1/RAF1/TGFBR1/BCL2/CCNB2 8

BP GO:0018212 peptidyl-tyrosine modification 35/1105 377/18866 0.004845262 0.052974231 0.043126891 ADAM17/IL6ST/IQGAP1/HIPK1/EIF2AK2/ADNP/ABL2/STAT3/PAK2/BAZ1B/NRP1/DDR2/ACVR1/RYK/MAP2K1/PTPN11/LRP4/ABI1/SNX6/ARHGEF2/CBLB/PRNP/EPHA3/CAMKK2/ARL2BP/EGFR/LYN/TGFA/PARP9/CD44/ERBB2/ROR2/TPST2/NRG1/LRP8 35

BP GO:0006972 hyperosmotic response 6/1105 28/18866 0.004931259 0.053419092 0.043489057 XRCC5/ARHGEF2/MAPK10/SLC25A23/AQP1/EFHD1 6

BP GO:0010575 positive regulation of vascular endothelial growth factor production 6/1105 28/18866 0.004931259 0.053419092 0.043489057 IL6ST/EIF2AK3/SULF1/BRCA1/SULF2/CYP1B1 6

BP GO:0010818 T cell chemotaxis 6/1105 28/18866 0.004931259 0.053419092 0.043489057 ADAM17/ADAM10/WNK1/STK39/CCL26/WNT5A 6

BP GO:0031440 regulation of mRNA 3'-end processing 6/1105 28/18866 0.004931259 0.053419092 0.043489057 VIRMA/CDC73/AHCYL1/NCBP2/ZFP36L1/CCNB1 6

BP GO:0007041 lysosomal transport 14/1105 110/18866 0.004933947 0.053419092 0.043489057 TGFBRAP1/SCARB2/ZFYVE16/AP3M1/NEDD4/AP3B1/GNPTAB/M6PR/LAMP2/HSPA8/ARSB/DTX3L/CCDC91/HOOK2 14

BP GO:0007411 axon guidance 28/1105 284/18866 0.004958476 0.05358043 0.043620404 BMPR2/KIF5B/FEZ2/ROBO1/NRP1/NOTCH2/RYK/PLXND1/PTPN11/TRIO/DAG1/PIK3R1/EXT1/UNC5C/NOTCH3/EPHA3/ENAH/PALLD/GRB2/RAC1/SEMA4B/NTN4/ERBB2/IRS2/SEMA3C/WNT5A/PLXNB3/UNC5D 28

BP GO:0008654 phospholipid biosynthetic process 27/1105 271/18866 0.004981641 0.053705265 0.043722033 SERINC5/PIGN/PIGK/ARF3/PCYT1A/SYNJ2/CSNK2A1/MBOAT2/PIK3R1/CSNK2A2/PIGS/CAPN2/OCRL/PLEKHA8/GNPAT/DPM1/DPAGT1/ABHD4/PI4K2B/SH3YL1/TMEM38B/DGKG/SLC44A3/CDS1/PIP5K1B/CPNE7/FABP5 27

BP GO:0032970 regulation of actin filament-based process 37/1105 405/18866 0.00499481 0.053705265 0.043722033 ROCK2/AKAP13/CLASP1/IQGAP1/ABL2/ATP2A2/TAOK1/PAK2/ACTR2/SSH1/NRP1/NOTCH2/ACTR3/ARF6/MTPN/PAM/FLNA/PIK3R1/ARFGEF1/TGFBR1/EPHA3/TGFB3/PLEKHG2/FMN1/PDE4B/GRB2/RAC1/ATP1A1/FAM107A/CX3CL1/TSC1/GBA2/ARPC3/PLEKHH2/CCL26/ARHGEF10L/ARPC1B 37

BP GO:0002902 regulation of B cell apoptotic process 5/1105 20/18866 0.005058705 0.053705265 0.043722033 BCL10/SLC39A10/LYN/IRS2/ADA 5

BP GO:0006346 DNA methylation-dependent heterochromatin assembly

5/1105 20/18866 0.005058705 0.053705265 0.043722033 HDAC1/MORC2/ATF7IP/PPHLN1/DNMT1 5

BP GO:0009168 purine ribonucleoside monophosphate biosynthetic process 5/1105 20/18866 0.005058705 0.053705265 0.043722033 GMPS/PPAT/GART/PAICS/ADA 5

BP GO:0060411 cardiac septum morphogenesis 11/110577/18866 0.005061083 0.053705265 0.043722033 PARVA/BMPR2/ROBO1/NRP1/NOTCH2/ACVR1/EGLN1/TGFBR1/ZFPM2/SEMA3C/WNT5A11

BP GO:0061418 regulation of transcription from RNA polymerase II promoter in response to hypoxia 11/110577/18866 0.005061083 0.053705265 0.043722033 PSMD12/EGLN1/CUL2/PSMD10/PSMD1/PSMF1/PSMD11/PSME4/PSMD2/PSME3/PSMD3 11

BP GO:0010717 regulation of epithelial to mesenchymal transition 13/1105 99/18866 0.00507371 0.053705265 0.043722033 SPRED1/CLASP1/ACVR1/ADIPOR1/DAG1/BCL9L/TGFBR1/STRAP/EPHA3/TGFB3/IL17RD/SDCBP/LEF1 13

BP GO:0006882 cellular zinc ion homeostasis 7/1105 37/18866 0.005085835 0.053705265 0.043722033 SLC30A1/SLC39A10/SLC30A5/SLC30A7/SLC39A6/MT2A/MT1X 7

BP GO:0009167 purine ribonucleoside monophosphate metabolic process

7/1105 37/18866 0.005085835 0.053705265 0.043722033 NT5E/GMPS/PPAT/GART/CASK/PAICS/ADA 7

BP GO:0033120 positive regulation of RNA splicing 7/1105 37/18866 0.005085835 0.053705265 0.043722033 NUP98/SLC38A2/PIK3R1/RBM3/HSPA8/HABP4/CIRBP 7

BP GO:0045070 positive regulation of viral genome replication 7/1105 37/18866 0.005085835 0.053705265 0.043722033 TBC1D20/ADAR/STAU1/SRPK1/SRPK2/NR5A2/HSPA8 7

BP GO:0010811 positive regulation of cell-substrate adhesion 15/1105 122/18866 0.005116401 0.053925699 0.043901491 MAP4K4/IQGAP1/CRKL/NRP1/NID1/RSU1/FLNA/DAG1/ECM2/EMP2/FMN1/RAC1/CX3CL1/P4HB/TSC1 15

BP GO:0061458 reproductive system development 40/1105 447/18866 0.005135198 0.054021507 0.04397949 BMPR2/SP3/TBC1D20/MSH2/SMARCC1/MAPK14/CRKL/BCL2L11/RRM1/NOTCH2/SENP2/EGLN1/MAP2K1/NRIP1/PTPN11/FLNA/RXRA/BIRC2/FBXW8/TGFBR1/ZFP36L1/SULF1/ZFPM2/NUP107/EGFR/GRB2/SGPL1/BCL2/MMP19/LEF1/DMRTA1/ROR2/SERPINE2/ADA/PPARG/CEBPB/WNT5A/WT1/ID4/PTPRN 40

BP GO:0097485 neuron projection guidance 28/1105 285/18866 0.005201196 0.05461256 0.044460672 BMPR2/KIF5B/FEZ2/ROBO1/NRP1/NOTCH2/RYK/PLXND1/PTPN11/TRI

O/DAG1/PIK3R1/EXT1/UNC5C/NOTCH3/EPHA3/ENAH/PALLD/GRB2/RAC1/SEMA4B/NTN4/ERBB2/IRS2/SEMA3C/WNT5A/PLXNB3/UNC5D 28

BP GO:0045766 positive regulation of angiogenesis 22/1105 208/18866 0.005336586 0.055823494 0.045446506 ITGB1/HIPK1/STAT3/MTDH/NRP1/AGGF1/SMAD1/BRCA1/RUNX1/F3/SERPINE1/ECM1/DDAH1/AGTR1/CX3CL1/CYP1B1/SRPX2/ADAM12/AQP1/RLN2/WNT5A/HYAL1 22

BP GO:0071456 cellular response to hypoxia 22/1105 208/18866 0.005336586 0.055823494 0.045446506 ROCK2/PSMD12/EGLN1/CUL2/PSMD10/PSMD1/NPEPPS/SUV39H2/PSMF1/PSMD11/PSME4/PGK1/PSMD2/ZFP36L1/PSME3/PSMD3/BCL2/DDAH1/P4HB/CCNB1/ACAA2/AQP1 22

BP GO:0034440 lipid oxidation 14/1105 111/18866 0.005350134 0.055860219 0.045476404 PEX13/MAPK14/ADIPOR1/ALDH3A2/ETFDH/ACAA2/IRS2/PPARG/SLC27A2/CYP4V2/ILVBL/AUH/HAO2/BDH2 14

BP GO:0070613 regulation of protein processing 10/1105 67/18866 0.005382369 0.055986703 0.045579376 RHBDD1/RNF139/CAST/IFT52/BIRC2/PRNP/LDLRAD3/SERPINE1/IL1R2/SERPINE2 10

BP GO:0072678 T cell migration 10/1105 67/18866 0.005382369 0.055986703 0.045579376

ADAM17/MSN/ADAM10/CRKL/WNK1/CD99L2/ECM1/STK39/CCL26/WNT5A 10

BP GO:0002903 negative regulation of B cell apoptotic process 4/1105 13/18866 0.005463358 0.056407399 0.04592187 BCL10/SLC39A10/IRS2/ADA 4

BP GO:0009313 oligosaccharide catabolic process 4/1105 13/18866 0.005463358 0.056407399 0.04592187 CTBS/MAN2B2/MANBA/GBA3 4

BP GO:0031453 positive regulation of heterochromatin assembly 4/1105 13/18866 0.005463358 0.056407399 0.04592187 MORC2/ATF7IP/PPHLN1/DNMT1 4

BP GO:0036109 alpha-linolenic acid metabolic process 4/1105 13/18866 0.005463358 0.056407399 0.04592187 ELOVL5/ELOVL1/ELOVL3/ACSL1 4

BP GO:0050919 negative chemotaxis 8/1105 47/18866 0.005519828 0.05654656 0.046035163 ROBO1/RYK/ITGAV/UNC5C/SEMA4B/SEMA3C/NRG1/WNT5A 8

BP GO:0120163 negative regulation of cold-induced thermogenesis 8/1105 47/18866 0.005519828 0.05654656 0.046035163 ADAM17/LAMA4/DOCK7/LNPEP/TFE3/PLCL2/NPR3/ABHD6 8

BP GO:1904375 regulation of protein localization to cell periphery 15/1105 123/18866 0.00552042 0.05654656 0.046035163

ITGB1/KIF5B/ADAM10/ATP2B4/ARF6/CLTC/DAG1/PIK3R1/PRNP/EPHA3/LDLRAP1/EGFR/ZDHHC7/STX3/ARHGEF16 15

BP GO:0060759 regulation of response to cytokine stimulus 21/1105 196/18866 0.005523475 0.05654656 0.046035163 ADAM17/HIPK1/ROBO1/ADAR/YTHDF3/VRK2/PTPN11/TBK1/BIRC2/CBFB/IFNGR1/CYLD/USP18/IKBKG/PARP9/SPATA2/RUNX1/ECM1/IL1R2/PPARG/WNT5A 21

BP GO:0045069 regulation of viral genome replication 13/1105 100/18866 0.005527642 0.05654656 0.046035163 EIF2AK4/EIF2AK2/TBC1D20/ADAR/STAU1/SRPK1/SRPK2/MORC2/NR5A2/OAS3/OAS2/HSPA8/BCL2 13

BP GO:0072431 signal transduction involved in mitotic G1 DNA damage checkpoint 9/1105 57/18866 0.005569081 0.056658022 0.046125904 CNOT8/CNOT2/PRKDC/CDK2/PML/CCNB1/CHEK2/AURKA/GTSE1 9

BP GO:0075733 intracellular transport of virus 9/1105 57/18866 0.005569081 0.056658022 0.046125904 NUP98/NUP155/KPNA3/NUP205/KPNB1/VPS37A/NUP93/NUP107/VPS37D 9

BP GO:1902400 intracellular signal transduction involved in G1 DNA damage checkpoint 9/1105 57/18866 0.005569081 0.056658022 0.046125904 CNOT8/CNOT2/PRKDC/CDK2/PML/CCNB1/CHEK2/AURKA/GTSE1 9

BP GO:0006417 regulation of translation 40/1105 450/18866 0.005739981 0.05829014 0.04745463 ROCK2/EIF2AK4/EIF2AK2/GEMIN5/STAT3/RPS6KB1/QKI/CNOT8/CNOT2/NOLC1/PRKDC/ETF1/EIF4G2/DNAJC3/GSPT1/YTHDF3/FXR1/EIF2AK3/RNF139/OGFOD1/EIF2S3/MTPN/PAIP1/RPS6KA3/RXRA/ENC1/KHSRP/EIF4G1/NCBP2/ZFP36L1/RBM3/NSUN3/MRPS27/SECISBP2/HABP4/CIRBP/ERBB2/ZNF540/TSC1/TRUB2 40

BP GO:0002244 hematopoietic progenitor cell differentiation 19/1105 172/18866 0.005834905 0.059095194 0.048110033 YAP1/EIF2AK2/XRCC5/PRKDC/PSMD12/ADAR/PSMD10/PSMD1/TCF12/PSMF1/PSMD11/PSME4/CBFB/PSMD2/PSME3/PSMD3/C12orf29/RUNX1/BCL2 19

BP GO:0002831 regulation of response to biotic stimulus 37/1105 409/18866 0.005840495 0.059095194 0.048110033 EIF2AK4/XRCC5/PRKDC/PAK2/BCL10/PSMD12/ADAR/NRAS/YTHDF3/CUL1/PSMD10/PTPN11/PSMD1/TBK1/TRIM5/BIRC2/PSMF1/RAF1/PSMD11/PSME4/USP15/PSMD2/PSME3/IFNGR1/PSMD3/LYN/USP18/IKBKG/DTX3L/PARP9/CX3CL1/IL2RA/PPARG/WNT5A/LY96/LRP8/SLAMF8 37

BP GO:0001782 B cell homeostasis 6/1105 29/18866 0.005915114 0.059522712 0.04845808 BCL10/BCL2L11/LYN/BCL2/ADA/TNFSF13B 6

BP GO:0070861 regulation of protein exit from endoplasmic reticulum

6/1105 29/18866 0.005915114 0.059522712 0.04845808 TMEM30A/SAR1A/UBE2J1/EDEM1/OS9/EDEM2 6

BP GO:0097064 ncRNA export from nucleus 7/1105 38/18866 0.005925531 0.059522712 0.04845808 NUP98/NUP155/NUP205/NCBP2/NUP93/NUP107/TSC1 7

BP GO:1905898 positive regulation of response to endoplasmic reticulum stress 7/1105 38/18866 0.005925531 0.059522712 0.04845808 SERINC3/USP13/ATF6/BCL2L11/EDEM1/PIK3R1/EDEM2 7

BP GO:0010812 negative regulation of cell-substrate adhesion 10/1105 68/18866 0.00598746 0.059928448 0.048788394 MAP4K4/PIK3R1/CASK/CORO1C/FZD7/SERPINE1/FAM107A/CX3CL1/AJAP1/SPOCK1 10

BP GO:1904377 positive regulation of protein localization to cell periphery 10/1105 68/18866 0.00598746 0.059928448 0.048788394 ITGB1/KIF5B/ATP2B4/ARF6/PIK3R1/PRNP/EPHA3/EGFR/STX3/ARHGEF1610

BP GO:0006906 vesicle fusion 13/1105 101/18866 0.006013457 0.059990361 0.048838798 KIF5B/TGFBRAP1/GNAI3/GOSR2/SNAP23/RAB8B/STX6/SEC22B/SAMD9/VAMP4/STXBP1/STX2/STX3 13

BP GO:0045727 positive regulation of translation 16/1105 136/18866 0.006015206 0.059990361 0.048838798

EIF2AK4/RPS6KB1/PRKDC/DNAJC3/YTHDF3/FXR1/EIF2S3/PAIP1/RXRA/EIF4G1/RBM3/MRPS27/HABP4/CIRBP/ERBB2/TRUB2 16

BP GO:0016485 protein processing 23/1105 223/18866 0.006043675 0.060166456 0.048982158 METAP2/ADAM10/RHBDD1/RNF139/CAST/IFT52/NCSTN/ECE1/BIRC2/SPCS3/PGK1/PRNP/LDLRAD3/CPE/F3/AFG3L2/SERPINE1/PCSK1/IL1R2/HM13/SERPINE2/PMPCA/C1R 23

BP GO:0006302 double-strand break repair 26/1105 262/18866 0.006082836 0.060448181 0.049211514 TRIP12/XRCC5/MSH2/TDP1/PRKDC/ACTR2/RECQL/DCLRE1B/WDR70/UBE2V2/UBE2N/SMARCAL1/TFIP11/RAD51C/UBQLN4/NBN/POT1/MLH1/XRCC4/BRCA1/PML/DTX3L/PARP9/FOXM1/CHEK2/TRIP13 26

BP GO:0007492 endoderm development 11/110579/18866 0.006157575 0.060864836 0.049550717 PELO/LAMC1/CDC73/HDAC1/ITGB5/COL4A2/EXT1/ITGAV/COL12A1/ZFP36L1/LAMB3 11

BP GO:0050000 chromosome localization 11/110579/18866 0.006157575 0.060864836 0.049550717 ACTR2/ACTR3/KPNB1/TERF1/INCENP/MLH1/MIS12/CCNB1/KIF14/CDCA8/KIF2C 11

BP GO:0051303 establishment of chromosome localization 11/1105 79/18866 0.006157575 0.060864836 0.049550717

ACTR2/ACTR3/KPNB1/TERF1/INCENP/MLH1/MIS12/CCNB1/KIF14/CDCA8/KIF2C 11

BP GO:0032535 regulation of cellular component size 35/1105 383/18866 0.006178014 0.0609586 0.049627051 BMPR2/MSN/ADNP/CDKL5/ACTR2/SSH1/NRP1/WDR36/RYK/ACTR3/ARF6/RB1CC1/MAP1B/MTPN/TMEM123/ARFGEF1/CLNS1A/PLEKHG2/FMN1/GRB2/RAC1/CLCN6/SEMA4B/TSC1/MAPT/AQP1/SEMA3C/ANAPC2/GBA2/ARPC3/PLEKHH2/CCL26/WNT5A/SLC12A5/ARPC1B 35

CC GO:0005925 focal adhesion 65/1122 415/19559 1.26E-13 7.82E-11 5.80E-11 PARVA/ADAM17/MAP4K4/CLASP1/ITGB1/VCL/IQGAP1/MSN/YWHAQ/PTPN12/YWHAB/SCARB2/ADAM10/SENP1/MAPRE1/ACTR2/NRP1/CAP1/GNA12/DDR2/GNA13/DCTN4/MAP2K1/ACTR3/RSU1/ARF6/SNAP23/CLTC/RAB10/DOCK7/ITGB5/YWHAZ/CD99L2/GDI2/FLNA/DAG1/ARHGEF2/NCSTN/PPP1CC/CAPN2/ITGAV/CASK/TSPAN9/ADAM9/RHOU/ENAH/DST/PALLD/EGFR/CORO1C/HSPA8/RAC1/LMLN/SDCBP/SORBS2/FAM107A/LMO7/CD44/ARHGAP24/FHL2/P4HB/ARPC3/FLNC/PLAUR/ARPC1B 65

CC GO:0030055 cell-substrate junction 65/1122 423/19559 3.12E-13 9.65E-11 7.15E-11 PARVA/ADAM17/MAP4K4/CLASP1/ITGB1/VCL/IQGAP1/MSN/YWHAQ/PTPN12/YWHAB/SCARB2/ADAM10/SENP1/MAPRE1/ACTR2/NRP1/CAP1/GNA12/DDR2/GNA13/DCTN4/MAP2K1/ACTR3/RSU1/ARF6/SNAP23/CLTC/R

AB10/DOCK7/ITGB5/YWHAZ/CD99L2/GDI2/FLNA/DAG1/ARHGEF2/NCSTN/PPP1CC/CAPN2/ITGAV/CASK/TSPAN9/ADAM9/RHOU/ENAH/DST/PALLD/EGFR/CORO1C/HSPA8/RAC1/LMLN/SDCBP/SORBS2/FAM107A/LMO7/CD44/ARHGAP24/FHL2/P4HB/ARPC3/FLNC/PLAUR/ARPC1B 65

CC GO:0031984 organelle subcompartment 53/1122 392/19559 5.44E-09 9.67E-07 7.17E-07 RAB6A/BACE1/RAB3GAP1/KLHL20/ATP11B/TGOLN2/CHSY1/MYO1B/ADAM10/TMEM87A/AP4E1/REEP3/KPNB1/GOLPH3/CLTC/RAB10/GALNT1/ATP2C1/FUT4/ATP8B2/PAM/UXS1/ST3GAL2/VCPIP1/RAB31/CANT1/STX6/ARFGEF1/COG5/OCRL/RAB43/PLEKHA8/GBF1/TBC1D23/M6PR/RAB30/SCAMP1/SULF1/VAMP4/MAN2A1/B4GALT3/GOLPH3L/ST3GAL1/GALNT2/NSF/SMPD4/PI4K2B/RAC1/SULF2/TGFBI/CA4/CCDC91/TMED3 53

CC GO:0005635 nuclear envelope 60/1122 473/19559 6.25E-09 9.67E-07 7.17E-07 PHF20/NUP98/DPY19L3/IPO9/BNIP2/ATP11B/ATF6/NUP155/TBC1D20/WDR3/CSE1L/SENP1/AHCTF1/SLC30A1/EI24/MTDH/RRM1/KPNA3/DCTN5/SPAST/SENP2/NUP205/TOR1B/KPNB1/BRAP/VRK2/RB1CC1/IPO7/CLCC1/KPNA4/TEX2/PCYT1A/ARL6IP6/GTPBP4/SMAD1/PARP11/ITSN1/TMEM43/CBX5/PRNP/NUP93/EPHA3/TOR1AIP2/NXT2/NUP107/DST/TUBB/EGFR/TNPO3/SMPD4/UBE2I/ITPR1/PGRMC2/SDCBP/BCL2/LMO7/TMEM38B/RCC1/AQP1/HTATIP2 60

CC GO:0098791 Golgi apparatus subcompartment 50/1122 368/19559

1.25E-08 1.55E-06 1.15E-06

RAB6A/BACE1/KLHL20/ATP11B/TGOLN2/CHSY1/MYO1B/ADAM10/TMEM87A/AP4E1/GOLPH3/CLTC/RAB10/GALNT1/ATP2C1/FUT4/ATP8B2/PAM/UXS1/ST3GAL2/VCPIP1/RAB31/CANT1/STX6/ARFGEF1/COG5/OCRL/RAB43/PLEKHA8/GBF1/TBC1D23/M6PR/RAB30/SCAMP1/SULF1/VAMP4/MAN2A1/B4GALT3/GOLPH3L/ST3GAL1/GALNT2/NSF/SMPD4/PI4K2B/RAC1/SULF2/TGFBI/CA4/CCDC91/TMED3 50

CC GO:0005788 endoplasmic reticulum lumen 43/1122 308/19559

5.96E-08 6.15E-06 4.56E-06

BACE1/GANAB/TGOLN2/EDEM3/ADAM10/LAMC1/FBN1/FKBP7/MIA3/DNAJC10/DNAJC3/TOR1B/OS9/COL4A1/COL15A1/P4HA1/VCPIP1/DAG1/H6PD/CANX/COL4A2/STS/TXNDC5/GBF1/COL12A1/CRTAP/MINPP1/APLP2/ARSB/CP/EDEM2/COL4A4/P4HA3/P4HB/FKBP10/TMEM132A/SCG2/IGFBP1/SLC27A2/WNT5A/PLAUR/COL10A1/TIMP1 43

CC GO:0005802 trans-Golgi network35/1122 245/19559 5.76E-07

4.93E-05 3.65E-05

RAB6A/BACE1/KLHL20/ATP11B/TGOLN2/MYO1B/ADAM10/AP4E1/GOLPH3/CLTC/RAB10/ATP2C1/FUT4/ATP8B2/PAM/RAB31/STX6/ARFGEF1/COG5/OCRL/RAB43/PLEKHA8/GBF1/TBC1D23/M6PR/RAB30/SCAMP1/VAMP4/GOLPH3L/SMPD4/PI4K2B/RAC1/TGFBI/CA4/CCDC91 35

CC GO:0031965 nuclear membrane 40/1122 301/19559 6.37E-07

4.93E-05 3.65E-05

PHF20/NUP98/DPY19L3/ATP11B/NUP155/TBC1D20/WDR3/SENP1/AHCTF1/SLC30A1/EI24/MTDH/DCTN5/SPAST/SENP2/NUP205/TOR1B/KPNB1/BRAP/RB1CC1/CLCC1/KPNA4/TEX2/ARL6IP6/GTPBP4/SMAD1/TMEM43/PRNP/NUP93/EPHA3/TOR1AIP2/NUP107/EGFR/SMPD4/ITPR1/SDCBP/BCL2/TMEM38B/RCC1/AQP1 40

CC GO:0030135 coated vesicle 39/1122 298/19559 1.29E-06 8.12E-05 6.02E-05

SEC23A/TGOLN2/TMED10/SCARB2/ARCN1/COPA/ADAM10/SAR1A/MCFD2/MYO1E/YIPF5/GOSR2/SEC23IP/CLTC/AP3B1/RAB8B/COPB1/STX6/ECE1/MALL/OCRL/SEC22B/COPB2/TMED7/M6PR/LMAN1/SCAMP1/VAMP4/LMAN2L/LDLRAP1/EGFR/HSPA8/TMED5/TGFA/ROR2/ASTN2/WNT5A/DENND1C/TMED3 39

CC GO:0005604 basement membrane 20/1122 103/19559 1.36E-06 8.12E-05 6.02E-05

ATRN/LAMC1/FBN1/SPARC/COL4A1/COL15A1/NID1/LAMA4/NID2/DAG1/COL4A2/CASK/DST/COL4A4/NTN4/FRAS1/TGFBI/LAMB3/APLP1/TIMP1 20

CC GO:0030027 lamellipodium 30/1122 201/19559 1.44E-06 8.12E-05 6.02E-05

PARVA/ITGB1/RAPH1/ACTR2/SSH1/ACTR3/PLXND1/NRBP1/ABI1/DAG1/ITSN1/ITGAV/KLHL2/SLC39A6/UNC5C/ARHGEF6/ENAH/PALLD/CORO1C/RAC1/STX2/SORBS2/CD44/RAB3IP/P4HB/TSC1/ARPC3/PLEKHH2/STX3/

MEFV 30

CC GO:0098687 chromosomal region 43/1122 350/19559 2.07E-06 0.000106694 7.91E-05 CLASP1/NUP98/CSNK1A1/XRCC5/MSH2/PRKDC/AHCTF1/CDC73/HAT1/BAZ1B/DCLRE1B/DCTN5/DCTN4/CKAP5/FBXO28/TERF1/SUV39H2/INCENP/DYNLT3/PPP1CC/TFIP11/CDK2/NBN/CBX5/THOC5/SP100/PTGES3/POT1/SUMO3/DNMT1/NUP107/MIS12/PML/CENPP/HMBOX1/CCNB1/PKHD1/CEBPB/CDCA8/CHEK2/BUB1/AURKA/KIF2C 43

CC GO:0044322 endoplasmic reticulum quality control compartment 9/1122 24/19559 3.89E-06 0.000179803 0.000133312 EDEM3/RNF103/SEL1L/EDEM1/OS9/RHBDD1/RNF139/CANX/EDEM2 9

CC GO:1905368 peptidase complex 18/1122 92/19559 4.07E-06 0.000179803 0.000133312 PSMD12/TRRAP/PIGK/PSMD10/PSMD1/PIGS/PSMF1/PSMD11/IDE/UBQLN4/PSME4/SPCS3/PSMD2/PSME3/ATXN7L3/PSMD3/F3/AFG3L2 18

CC GO:0042470 melanosome 19/1122 105/19559 7.38E-06 0.000285502 0.000211681 ITGB1/GANAB/YWHAB/TMED10/GNA13/CLTC/YWHAZ/CCT4/CANX/NCSTN/RAB1A/SEC22B/MYO5A/HSPA8/RAC1/ATP1A1/SDCBP/P4HB/STX3 19

CC GO:0048770 pigment granule 19/1122 105/19559 7.38E-06 0.000285502 0.000211681

ITGB1/GANAB/YWHAB/TMED10/GNA13/CLTC/YWHAZ/CCT4/CANX/NCSTN/RAB1A/SEC22B/MYO5A/HSPA8/RAC1/ATP1A1/SDCBP/P4HB/STX3 19

CC GO:0005667 transcription regulator complex 46/1122 413/19559 1.30E-05 0.000471712 0.000349743 YAP1/SP3/GEMIN5/STAT3/YWHAB/TAF5L/CHD4/PRKDC/NFATC3/ASCC1/TRRAP/ASCC3/MIER1/GTF3C1/TAF2/LIN9/HDAC1/GTF2H3/TCF12/ATF1/SKIL/MTA3/SMAD1/CSNK2A1/RXRA/RBL1/BCL9L/CDK2/HDAC3/GTF2E1/CBX5/TCEA1/CBFB/ATF7IP/TAF1A/NFYB/NR5A2/RBBP7/AHR/TAF13/CHD3/NCOR2/RUNX1/LEF1/PPARG/CEBPB 46

CC GO:0030662 coated vesicle membrane 26/1122 183/19559 1.74E-05 0.000573416 0.00042515 SEC23A/TGOLN2/TMED10/SCARB2/ARCN1/COPA/SAR1A/MCFD2/GOSR2/SEC23IP/CLTC/AP3B1/COPB1/SEC22B/COPB2/TMED7/M6PR/LMAN1/VAMP4/LDLRAP1/EGFR/HSPA8/TGFA/ROR2/WNT5A/TMED3 26

CC GO:0034399 nuclear periphery 21/1122 131/19559 1.76E-05 0.000573416 0.00042515 NUP98/PRPF40A/AHCTF1/HAT1/NUP205/SPARC/SRPK1/HLCS/DAG1/ENC1/ARFGEF1/MORC2/CASK/NUP93/NUP107/PML/NCOR2/GMCL1/MAPT/CXXC1/CEBPB 21

CC GO:0005766 primary lysosome 23/1122 155/19559 2.59E-05 0.000764421 0.000566768 TMEM30A/ATP11B/ACTR2/CAP1/DNAJC3/SNAP23/PSMD1/RAP1B/CC

T8/GDI2/NCSTN/TXNDC5/SURF4/CMTM6/CCT2/RAB3D/TUBB/MANBA/LAMP2/ARSB/SDCBP/STX3/FABP5 23

CC GO:0042582 azurophil granule 23/1122 155/19559 2.59E-05 0.000764421 0.000566768 TMEM30A/ATP11B/ACTR2/CAP1/DNAJC3/SNAP23/PSMD1/RAP1B/CCT8/GDI2/NCSTN/TXNDC5/SURF4/CMTM6/CCT2/RAB3D/TUBB/MANBA/LAMP2/ARSB/SDCBP/STX3/FABP5 23

CC GO:0031983 vesicle lumen 38/1122 328/19559 3.04E-05 0.000853992 0.000633178 BACE1/VCL/CAND1/XRCC5/MAPK14/PAFAH1B2/ACTR2/CAP1/PSMD12/DNAJC3/SPARC/KPNB1/PSMD1/APAF1/CCT8/GDI2/CANT1/PSMD11/TXNDC5/PSMD2/ATG7/CCT2/PSMD3/TUBB/TGFB3/FAM3C/EGFR/SCCPDH/HSPA8/ARSB/SDCBP/SERPINE1/ECM1/PCSK1/ADA/FABP5/TIMP1/LGALS3BP 38

CC GO:0030126 COPI vesicle coat 6/1122 13/19559 4.26E-05 0.001142403 0.000847016 ARCN1/COPA/COPB1/COPB2/TMED7/TMED3 6

CC GO:0031252 cell leading edge 45/1122 421/19559 4.43E-05 0.001142403 0.000847016 PARVA/ADAM17/ITGB1/RAPH1/IQGAP1/CDKL5/ACTR2/SSH1/ACTR3/ARF6/PLXND1/NRBP1/ABI1/DAG1/ARHGEF2/MYO1D/ITSN1/ITGAV/ATP2B1/GBF1/MYO5A/KLHL2/SLC39A6/UNC5C/ARHGEF6/ENAH/DST/PALLD/COR

O1C/RAC1/STX2/SORBS2/FAM107A/CD44/SH3YL1/RAB3IP/P4HB/TSC1/MAPT/CDC42BPG/ARPC3/PLEKHH2/STX3/MEFV/BSPRY 45

CC GO:0090575 RNA polymerase II transcription regulator complex 23/1122 161/19559 4.78E-05 0.001162907 0.000862218 YAP1/GEMIN5/STAT3/TAF5L/TRRAP/ASCC3/TAF2/GTF2H3/TCF12/ATF1/SMAD1/RXRA/BCL9L/GTF2E1/TCEA1/CBFB/NFYB/NR5A2/TAF13/RUNX1/LEF1/PPARG/CEBPB 23

CC GO:0000151 ubiquitin ligase complex 34/1122 287/19559 4.98E-05 0.001162907 0.000862218 KCTD10/CDC27/KLHL20/CAND1/USP47/ANAPC1/CUL4B/SEL1L/RNF2/OS9/BRAP/NEDD4/YPEL5/WDR26/CUL2/PCGF6/CUL1/COP1/UBE2N/ENC1/RAD18/KLHL2/UBE2A/FBXW8/CBX2/FBXL7/UBE2E1/BRCA1/IKBKG/LMO7/CBX7/KCTD17/ANAPC2/FBXL1634

CC GO:0005793 endoplasmic reticulum-Golgi intermediate compartment 20/1122 130/19559 5.07E-05 0.001162907 0.000862218 TMED10/TBC1D20/ROBO1/MCFD2/GOSR2/SEC23IP/COPB1/SEC22B/TMED7/GBF1/LMAN1/RGMB/SURF4/GNPNAT1/LMAN2L/TMED5/TGFA/P4HB/CA4/TMED3 20

CC GO:0000775 chromosome, centromeric region 26/1122 196/19559 5.78E-05 0.001272303 0.000943328 CLASP1/NUP98/CSNK1A1/AHCTF1/BAZ1B/DCTN5/DCTN4/CKAP5/FBXO28/SUV39H2/INCENP/DYNLT3/PPP1CC/CBX5/SUMO3/DNMT1/NUP107/M

IS12/CENPP/CCNB1/PKHD1/CEBPB/CDCA8/BUB1/AURKA/KIF2C 26

CC GO:0060205 cytoplasmic vesicle lumen 37/1122 326/19559 5.96E-05 0.001272303 0.000943328 BACE1/VCL/CAND1/XRCC5/MAPK14/PAFAH1B2/ACTR2/CAP1/PSMD12/DNAJC3/SPARC/KPNB1/PSMD1/APAF1/CCT8/GDI2/CANT1/PSMD11/TXNDC5/PSMD2/ATG7/CCT2/PSMD3/TUBB/TGFB3/FAM3C/SCCPDH/HSPA8/ARSB/SDCBP/SERPINE1/ECM1/PCSK1/ADA/FABP5/TIMP1/LGALS3BP 37

CC GO:0016605 PML body 17/1122 102/19559 6.56E-05 0.001353739 0.001003707 KLHL20/SP3/HIPK1/SENP2/RNF4/TOPBP1/SUMO2/NBN/CBX5/SP100/TDG/SUMO3/ELF4/PML/UBE2I/HMBOX1/CHEK2 17

CC GO:0005874 microtubule45/1122 431/19559 7.75E-05 0.001544854 0.001145406 CDC27/CLASP1/IQGAP1/KIF5B/MAPRE1/BCL10/REEP3/SPAST/MAP4/CKAP5/RNF4/CLTC/MAP1B/CCT8/CCT4/KIF3C/CEP170/CCT7/TCP11L1/ARHGEF2/NEK7/INCENP/DYNLT3/RASSF3/MYO5A/CCT2/DST/RAB3D/MAP1LC3B/TUBB/CYLD/CKAP2/MAP7/TPPP/MAPT/ROR2/GAS8/TPX2/KIF14/MEFV/KIF20A/AURKA/KIF2C/HOOK2/GTSE1 45

CC GO:0030667 secretory granule membrane 35/1122 306/19559 7.99E-05 0.001544854 0.001145406 RAB6A/TMEM30A/ATP11B/IQGAP1/TMED10/ADAM10/NRAS/SPARC/SNAP23/RAB10/SLC30A5/COPB1/RAP1B/PAM/RAB31/NCSTN/ITGAV/SCAM

P1/SURF4/CMTM6/RAB3D/APLP2/MANBA/LAMP2/ITPR1/RAC1/CPE/CD44/MGAM/CA4/SLC27A2/STX3/DMBT1/FABP5/PLAUR 35

CC GO:0030133 transport vesicle 43/1122 410/19559 0.000100215 0.001830193 0.001356967 RAB6A/BACE1/TMEM30A/SEC23A/TGOLN2/TMED10/ARCN1/COPA/ADAM10/SAR1A/MCFD2/GOSR2/SEC23IP/CLTC/RAB10/RAB8B/COPB1/PAM/NCSTN/STX6/RAB1A/SEC22B/COPB2/TMED7/M6PR/LMAN1/SCAMP1/SURF4/VAMP4/RAB3D/STXBP5/PDE4B/ITPR1/STX2/TGFA/CPE/RAB3IP/CA4/PCSK1/CLCN5/STX3/PTPRN/TMED3 43

CC GO:0000118 histone deacetylase complex 14/1122 77/19559 0.000108614 0.001830193 0.001356967 CHD4/TRRAP/NRIP1/HDAC1/ZNF217/MTA3/CSNK2A1/HDAC3/CBX5/RBBP7/PHF21A/CHD3/NCOR2/DNTTIP1 14

CC GO:0005798 Golgi-associated vesicle 24/1122 181/19559 0.000110483 0.001830193 0.001356967 BACE1/SEC23A/TGOLN2/TMED10/ARCN1/COPA/ADAM10/SAR1A/MCFD2/YIPF5/GOSR2/SEC23IP/CLTC/RAB8B/COPB1/OCRL/SEC22B/COPB2/TMED7/LMAN1/LMAN2L/TMED5/TGFA/TMED3 24

CC GO:0016581 NuRD complex 6/1122 15/19559 0.000112354 0.001830193 0.001356967 CHD4/HDAC1/MTA3/CSNK2A1/RBBP7/CHD3 6

CC GO:0042588 zymogen granule 6/1122 15/19559 0.000112354

0.001830193 0.001356967 TMED10/GNAI3/SCAMP1/RAB3D/STX3/DMBT1 6

CC GO:0090545 CHD-type complex 6/1122 15/19559 0.000112354 0.001830193 0.001356967 CHD4/HDAC1/MTA3/CSNK2A1/RBBP7/CHD3 6

CC GO:0030137 COPI-coated vesicle 8/1122 28/19559 0.000126725 0.002011350.001491282 TMED10/ARCN1/COPA/COPB1/SEC22B/COPB2/TMED7/TMED3 8

CC GO:1905369 endopeptidase complex 13/1122 69/19559 0.000130295 0.00201631 0.00149496 PSMD12/PIGK/PSMD10/PSMD1/PIGS/PSMF1/PSMD11/IDE/UBQLN4/PSME4/PSMD2/PSME3/PSMD3 13

CC GO:0005795 Golgi stack 21/1122 151/19559 0.000147928 0.002233345 0.001655877 CHSY1/TMEM87A/GOLPH3/GALNT1/FUT4/UXS1/ST3GAL2/VCPIP1/CANT1/OCRL/GBF1/RAB30/SULF1/MAN2A1/B4GALT3/GOLPH3L/ST3GAL1/GALNT2/NSF/SULF2/TMED3 21

CC GO:0045121 membrane raft 36/1122 329/19559 0.000156345 0.002304223 0.001708428 BMPR2/ADAM17/SPRED1/BACE1/ITGB1/IQGAP1/GNAI3/BCL10/ATP2B4/F2R/LRP4/DAG1/SYNJ2/MYO1D/MALL/BIRC2/CAPN2/CBLB/ATP2B1/PAG1/TGFBR1/PGK1/RGMB/PRNP/SULF1/EMP2/TUBB/EGFR/CORO1C/LAMP2

/LYN/ATP1A1/SDCBP/MAPT/RTN4RL1/LRP8 36

CC GO:0098857 membrane microdomain 36/1122 330/19559 0.000166055 0.002390416 0.001772335 BMPR2/ADAM17/SPRED1/BACE1/ITGB1/IQGAP1/GNAI3/BCL10/ATP2B4/F2R/LRP4/DAG1/SYNJ2/MYO1D/MALL/BIRC2/CAPN2/CBLB/ATP2B1/PAG1/TGFBR1/PGK1/RGMB/PRNP/SULF1/EMP2/TUBB/EGFR/CORO1C/LAMP2/LYN/ATP1A1/SDCBP/MAPT/RTN4RL1/LRP8 36

CC GO:0098589 membrane region 37/1122 343/19559 0.000170287 0.002395635 0.001776204 BMPR2/ADAM17/SPRED1/BACE1/ITGB1/IQGAP1/GNAI3/BCL10/ATP2B4/F2R/LRP4/DAG1/SYNJ2/MYO1D/MALL/BIRC2/CAPN2/CBLB/ATP2B1/PAG1/TGFBR1/PGK1/RGMB/PRNP/SULF1/EMP2/TUBB/EGFR/TNPO3/CORO1C/LAMP2/LYN/ATP1A1/SDCBP/MAPT/RTN4RL1/LRP8 37

CC GO:0016323 basolateral plasma membrane 29/1122 246/19559 0.000188645 0.002594923 0.001923963 BMPR2/MSN/ATP2B4/HEPH/DAG1/MYO1D/IDE/CASK/ATP2B1/P2RY1/ADAM9/DST/LDLRAP1/EGFR/ATP1A1/STX2/TGFA/CNNM4/CD44/STK39/ERBB2/AJAP1/MAP7/ANK3/LIN7A/AQP1/VSIG1/SLC7A5/SLC16A12 29

CC GO:0034774 secretory granule lumen 35/1122 322/19559 0.000218034 0.002933985 0.002175355 VCL/CAND1/XRCC5/MAPK14/PAFAH1B2/ACTR2/CAP1/PSMD12/DNAJC3/SPARC/KPNB1/PSMD1/APAF1/CCT8/GDI2/CANT1/PSMD11/TXNDC5/P

SMD2/ATG7/CCT2/PSMD3/TUBB/TGFB3/FAM3C/SCCPDH/HSPA8/ARSB/SDCBP/SERPINE1/ECM1/PCSK1/FABP5/TIMP1/LGALS3BP 35
CC GO:0101002 ficolin-1-rich granule 18/1122 124/19559 0.000250635 0.003232149 0.002396424 VCL/CAND1/KCMF1/MAPK14/PAFAH1B2/ACTR2/PSMD12/KPNB1/YPEL5/APAF1/CCT8/CANT1/PSMD11/PSMD2/ATG7/PSMD3/HSPA8/ARSB 18
CC GO:1904813 ficolin-1-rich granule lumen 18/1122 124/19559 0.000250635 0.003232149 0.002396424 VCL/CAND1/KCMF1/MAPK14/PAFAH1B2/ACTR2/PSMD12/KPNB1/YPEL5/APAF1/CCT8/CANT1/PSMD11/PSMD2/ATG7/PSMD3/HSPA8/ARSB 18
CC GO:0022624 proteasome accessory complex 7/1122 24/19559 0.000292891 0.003699993 0.002743299 PSMD12/PSMD10/PSMD1/PSMD11/PSMD2/PSME3/PSMD3 7
CC GO:0017053 transcription repressor complex13/1122 76/19559 0.000354939 0.004358597 0.00323161 SP3/YWHAB/CHD4/MIER1/LIN9/HDAC1/MTA3/CSNK2A1/HDAC3/CBX5/RBBP7/CHD3/NCOR2 13
CC GO:0030663 COPI-coated vesicle membrane 6/1122 18/19559 0.000359109 0.004358597 0.00323161 ARCN1/COPA/COPB1/COPB2/TMED7/TMED3 6
CC GO:0101031 chaperone complex7/1122 25/19559 0.000386789 0.004604276 0.003413765

CCT8/CCT4/CCT7/PTGES3/CCT2/HSPA8/TSC1 7

CC GO:0000793 condensed chromosome 26/1122 222/19559 0.000438368 0.005119806 0.003795996 CLASP1/CSNK1A1/AHCTF1/BAZ1B/DCTN5/CKAP5/RAD1/FBXO28/TOPBP1/INCENP/DYNLT3/PPP1CC/CDK2/SMC4/MLH1/NUP107/BRCA1/MIS12/UBE2I/RCC1/CCNB1/CCNB1IP1/CEBPB/BUB1/AURKA/KIF2C 26

CC GO:0045178 basal part of cell 12/1122 69/19559 0.000506523 0.005806253 0.004304951 BMPR2/CLASP1/DOCK7/DAG1/HFE/DST/LDLRAP1/EGFR/ERBB2/ANK3/AQP1/SLC7A5 12

CC GO:0005819 spindle 37/1122 367/19559 0.000633119 0.007125471 0.005283063 CDC27/CLASP1/NEDD1/CEP350/MAPK14/MAPRE1/SPAST/MAP4/NR3C1/DCTN4/CKAP5/YPEL5/EVI5/CLTC/CEP170/YEATS2/ARHGEF2/TOPBP1/NEK7/TERF1/INCENP/DYNLT3/HDAC3/NSUN2/ARL2BP/CYLD/CKAP2/IKBKG/CCNB1/TPPP/PKHD1/TPX2/KIF14/CDCA8/KIF20A/AURKA/KIF2C 37

CC GO:0030660 Golgi-associated vesicle membrane 16/1122 112/19559 0.000648517 0.007168426 0.005314911 SEC23A/TMED10/ARCN1/COPA/SAR1A/MCFD2/GOSR2/SEC23IP/CLTC/COPB1/SEC22B/COPB2/TMED7/LMAN1/TGFA/TMED3 16

CC GO:0005657 replication fork 12/1122 71/19559 0.000662063 0.007189769 0.005330736

BAZ1B/TOP1/ZRANB3/PRIM1/POLD3/RAD18/SMARCAL1/RAD51C/NBN/WDHD1/DNMT1/RFC5 12

CC GO:0090734 site of DNA damage 13/1122 82/19559 0.000754402 0.008051288 0.005969495 XRCC5/ACTR2/ATF2/ACTR3/WDR70/RAD18/SMARCAL1/UBQLN4/NBN/CBX5/WDR76/PARP9/ARPC3 13

CC GO:0030136 clathrin-coated vesicle 23/1122 194/19559 0.000771516 0.008094375 0.006001441 TGOLN2/TMED10/SCARB2/ADAM10/MYO1E/CLTC/AP3B1/RAB8B/STX6/ECE1/MALL/OCRL/M6PR/SCAMP1/VAMP4/LDLRAP1/EGFR/HSPA8/TGFA/ROR2/ASTN2/WNT5A/DENND1C 23

CC GO:0000502 proteasome complex 11/112263/19559 0.000828782 0.008550266 0.006339454 PSMD12/PSMD10/PSMD1/PSMF1/PSMD11/IDE/UBQLN4/PSME4/PSMD2/PSME3/PSMD3 11

CC GO:0000776 kinetochore 18/1122 137/19559 0.00085085 0.008634038 0.006401565 CLASP1/NUP98/CSNK1A1/AHCTF1/DCTN5/DCTN4/CKAP5/FBXO28/INCENP/DYNLT3/PPP1CC/CBX5/SUMO3/NUP107/MIS12/CCNB1/BUB1/KIF2C 18

CC GO:0005838 proteasome regulatory particle 6/1122 21/19559 0.000905036 0.008910009 0.00660618

PSMD12/PSMD10/PSMD1/PSMD11/PSMD2/PSMD3 6

CC GO:0098978 glutamatergic synapse 36/1122 361/19559 0.000906835 0.008910009 0.00660618 ITGB1/STAT3/IL1RAP/ADAM10/MAPK14/CDKL5/PAK2/NRP1/ATP2B4/EGLN1/SPARC/PPP3CA/FXR1/NEDD4/ARF6/STAU1/YWHAZ/FLNA/DAG1/ARHGEF2/CANX/ITSN1/PPP1CC/ATP2B1/P2RY1/MYO5A/LYN/HSPA8/RAC1/STXBP1/MAPK10/NRG1/STX3/ABHD6/WNT5A/OLFM2 36

CC GO:0030134 COPII-coated ER to Golgi transport vesicle 14/1122 95/19559 0.001012877 0.009737831 0.007219954 SEC23A/TMED10/SAR1A/MCFD2/YIPF5/GOSR2/SEC23IP/SEC22B/TMED7/LMAN1/LMAN2L/TMED5/TGFA/TMED3 14

CC GO:0002102 podosome 7/1122 29/19559 0.001026967 0.009737831 0.007219954 VCL/SH3PXD2A/PTPN12/ACTR2/ARHGEF2/RHOU/PALLD 7

CC GO:0005769 early endosome 37/1122 377/19559 0.001038282 0.009737831 0.007219954 BACE1/ATP11B/WDFY1/RPS6KC1/TGFBRAP1/MYO1B/ZFYVE16/NRP1/MAP2K1/ARF6/LNPEP/STAMBP/F2R/RAB31/SNX6/NCSTN/MYO1D/STX6/RAB1A/VPS26B/OCRL/MYO5A/NIPA2/CMTM6/EPHA3/AOC3/HFE/LDLRAP1/PML/EGFR/DTX3L/VPS29/ERBB2/CLCN5/ASTN2/C1orf210/SLC5A1 37

CC GO:0033116 endoplasmic reticulum-Golgi intermediate compartment membrane 12/1122 75/19559 0.001094493 0.009991136

0.007407763 TMED10/TBC1D20/ROBO1/MCFD2/GOSR2/SEC22B/TMED7/LMAN1/SURF4/TMED5/TGFA/TMED3 12

CC GO:0045335 phagocytic vesicle 18/1122 140/19559 0.001097572 0.009991136 0.007407763 KIF5B/RAB23/SNAP23/RAB10/RAB8B/ITGB5/RAB31/STX6/VPS26B/OCRL/ITGAV/SEC22B/RAB43/ATG12/ATG5/LAMP2/STXBP1/DMBT1 18

CC GO:0030670 phagocytic vesicle membrane 12/1122 77/19559 0.001386055 0.012317205 0.009132389 RAB23/SNAP23/RAB10/RAB8B/RAB31/OCRL/SEC22B/RAB43/ATG12/ATG5/LAMP2/DMBT1 12

CC GO:0016363 nuclear matrix 15/1122 109/19559 0.001392899 0.012317205 0.009132389 PRPF40A/AHCTF1/HAT1/SPARC/SRPK1/HLCS/ENC1/ARFGEF1/MORC2/CASK/PML/NCOR2/GMCL1/CXXC1/CEBPB 15

CC GO:0035371 microtubule plus-end 6/1122 23/19559 0.001525615 0.013300784 0.009861647 CLASP1/MAPRE1/CKAP5/MYO5A/DST/KIF2C 6

CC GO:0048787 presynaptic active zone membrane 7/1122 31/19559 0.001565087 0.013455403 0.009976287 ATP2B4/CANX/ATP2B1/P2RY1/STXBP1/STX2/STX3 7

CC GO:0000779 condensed chromosome, centromeric region 16/1122

122/19559 0.001643553 0.013936432 0.010332938 CLASP1/CSNK1A1/AHCTF1/DCTN5/CKAP5/FBXO28/INCENP/DYNLT3/PPP1CC/NUP107/MIS12/CCNB1/CEBPB/BUB1/AURKA/KIF2C 16

CC GO:0000784 nuclear chromosome, telomeric region 16/1122 124/19559 0.001949565 0.016307849 0.012091186 XRCC5/MSH2/PRKDC/CDC73/HAT1/DCLRE1B/TERF1/PPP1CC/TFIP11/NBN/CBX5/THOC5/SP100/POT1/PML/HMBOX1 16

CC GO:0030426 growth cone 21/1122 184/19559 0.002058168 0.016986749 0.012594546 IQGAP1/KIF5B/USP9X/COPA/CDKL5/SSH1/NRP1/FXR1/DOCK7/MAP1B/EXOC4/ABI1/FLNA/MYO5A/UNC5C/PALLD/NDRG2/TSC1/MAPT/ARPC3/STX3 21

CC GO:0009925 basal plasma membrane 9/1122 51/19559 0.00223798 0.018227755 0.013514669 BMPR2/DAG1/DST/LDLRAP1/EGFR/ERBB2/ANK3/AQP1/SLC7A5 9

CC GO:0032580 Golgi cisterna membrane 13/1122 93/19559 0.002455787 0.019741978 0.014637365 CHSY1/TMEM87A/GOLPH3/GALNT1/FUT4/UXS1/ST3GAL2/CANT1/B4GALT3/GOLPH3L/ST3GAL1/GALNT2/TMED3 13

CC GO:0005832 chaperonin-containing T-complex 4/1122 11/19559 0.002567149 0.019863317 0.01472733 CCT8/CCT4/CCT7/CCT2 4

CC GO:0005885 Arp2/3 protein complex 4/1122 11/19559 0.002567149 0.019863317 0.01472733 ACTR2/ACTR3/ARPC3/ARPC1B 4

CC GO:0042589 zymogen granule membrane 4/1122 11/19559 0.002567149 0.019863317 0.01472733 TMED10/SCAMP1/STX3/DMBT1 4

CC GO:0012507 ER to Golgi transport vesicle membrane 10/1122 62/19559 0.002613672 0.01997362 0.014809112 SEC23A/TMED10/SAR1A/MCFD2/GOSR2/SEC23IP/SEC22B/TMED7/LMAN1/TGFA 10

CC GO:0000152 nuclear ubiquitin ligase complex 8/1122 43/19559 0.002752833 0.02016268 0.014949287 CDC27/ANAPC1/RNF2/PCGF6/CBX2/BRCA1/CBX7/ANAPC2 8

CC GO:1990752 microtubule end 7/1122 34/19559 0.00275569 0.02016268 0.014949287 CLASP1/MAPRE1/CKAP5/RNF4/MYO5A/DST/KIF2C 7

CC GO:0000781 chromosome, telomeric region 19/1122 164/19559 0.002768704 0.02016268 0.014949287 XRCC5/MSH2/PRKDC/CDC73/HAT1/DCLRE1B/TERF1/PPP1CC/TFIP11/CDK2/NBN/CBX5/THOC5/SP100/PTGES3/POT1/PML/HMBOX1/CHEK2 19

CC GO:0030666 endocytic vesicle membrane 19/1122 164/19559 0.002768704 0.02016268 0.014949287 GPR161/SCARB2/RAB23/SNAP23/CLTC/RAB10/RAB8B/RAB31/OCRL/

SEC22B/RAB43/ENTPD7/ATG12/ATG5/LAMP2/NOSTRIN/ROR2/DMBT1/WNT5A 19

CC GO:0031985 Golgi cisterna 15/1122 117/19559 0.002830811 0.020375254 0.015106897 CHSY1/TMEM87A/GOLPH3/GALNT1/FUT4/UXS1/ST3GAL2/CANT1/RAB30/MAN2A1/B4GALT3/GOLPH3L/ST3GAL1/GALNT2/TMED3 15

CC GO:0000777 condensed chromosome kinetochore 14/1122 106/19559 0.002924705 0.020572638 0.015253244 CLASP1/CSNK1A1/AHCTF1/DCTN5/CKAP5/FBXO28/INCENP/DYNLT3/PPP1CC/NUP107/MIS12/CCNB1/BUB1/KIF2C 14

CC GO:0030175 filopodium 14/1122 106/19559 0.002924705 0.020572638 0.015253244 ITGB1/RAPH1/MSN/MYO1B/FXR1/ARF6/ABI1/DAG1/CD302/ITGAV/MYO5A/UNC5C/ENAH/ARL4C 14

CC GO:0016324 apical plasma membrane 34/1122 361/19559 0.003174156 0.022076434 0.016368209 BMPR2/ADAM17/SLC26A2/TMEM30A/MSN/MTDH/DDR2/SLC16A2/SLC30A5/AHCYL1/ATP2B1/P2RY1/EMP2/EGFR/ATP1A1/SORBS2/LMO7/CD44/STK39/KL/ERBB2/AJAP1/MGAM/CA4/PKHD1/AQP1/SLC6A19/STX3/SLC5A8/SLC10A2/SLC5A1/SLC7A5/SLC17A4/SLC22A12 34

CC GO:0030427 site of polarized growth 21/1122 191/19559 0.003231956 0.022193195 0.01645478

IQGAP1/KIF5B/USP9X/COPA/CDKL5/SSH1/NRP1/FXR1/DOCK7/MAP1B/EXOC4/ABI1/FLNA/MYO5A/UNC5C/PALLD/NDRG2/TSC1/MAPT/ARPC3/STX3 21

CC GO:0005643 nuclear pore 12/1122 85/19559 0.003262651 0.022193195 0.01645478 NUP98/NUP155/AHCTF1/KPNA3/SENP2/NUP205/KPNB1/IPO7/PARP11/NUP93/NXT2/NUP107 12

CC GO:0045177 apical part of cell 39/1122 433/19559 0.003624384 0.024243284 0.017974784 BMPR2/ADAM17/SLC26A2/TMEM30A/MSN/MYO1B/NEDD1/MTDH/DDR2/SLC16A2/ACVR1/SLC30A5/AHCYL1/ATP2B1/P2RY1/HFE/EMP2/EGFR/ATP1A1/SORBS2/LMO7/CD44/STK39/KL/ERBB2/AJAP1/MGAM/CA4/PKHD1/AQP1/SLC6A19/CLCN5/STX3/SLC5A8/SLC10A2/SLC5A1/SLC7A5/SLC17A4/SLC22A12 39

CC GO:0043034 costamere 5/1122 19/19559 0.003642367 0.024243284 0.017974784 VCL/FXR1/DAG1/ANK3/FLNC 5

CC GO:0018995 host cellular component 10/1122 65/19559 0.00372746 0.024287343 0.018007451 NUP98/NUP155/NUP205/KPNB1/VPS37A/LMAN1/NUP93/NUP107/VPS37D/AQP1 10

CC GO:0043657 host cell 10/1122 65/19559 0.00372746 0.024287343 0.018007451

NUP98/NUP155/NUP205/KPNB1/VPS37A/LMAN1/NUP93/NUP107/VPS37D/AQP1 10

CC GO:0030139 endocytic vesicle 30/1122 313/19559 0.004175473 0.026923101 0.01996169 KIF5B/GPR161/SCARB2/MYO1E/RAB23/SPARC/ARF6/SNAP23/CLTC/RAB10/RAB8B/ITGB5/RAB31/STX6/VPS26B/ITSN1/OCRL/ITGAV/SEC22B/RAB43/ENTPD7/ATG12/ATG5/EGFR/LAMP2/STXBP1/NOSTRIN/ROR2/DMBT1/WNT5A 30

CC GO:0030176 integral component of endoplasmic reticulum membrane 18/1122 158/19559 0.004219831 0.026928609 0.019965774 ATF6/EDEM1/PIGK/EIF2AK3/RHBDD1/INSIG2/CANX/BFAR/PIGS/EXT1/DPAGT1/ELOVL5/ELOVL1/SGPL1/ELOVL3/HM13/HSD11B1/SLC27A2 18

CC GO:0030120 vesicle coat9/1122 56/19559 0.004315373 0.027257307 0.020209482 SEC23A/ARCN1/COPA/SAR1A/CLTC/COPB1/COPB2/TMED7/TMED3 9

CC GO:0000792 heterochromatin 11/112278/19559 0.004792474 0.029965064 0.022217104 RNF2/BAZ1B/HDAC1/INCENP/MORC2/CBX5/UBE2A/CBX2/DNMT1/PML/WDR76 11

CC GO:0031253 cell projection membrane 32/1122 344/19559 0.004953923 0.030630167 0.022710233

ADAM17/ITGB1/SLC26A2/MSN/GPR161/MAPRE1/CDKL5/GNA12/GNA13/ARF6/PLXND1/ARHGEF2/ARL13B/MYO1D/ITGAV/CASK/ATP2B1/SLC39A6/CYBRD1/CORO1C/RAC1/FAM107A/CD44/SH3YL1/CA4/MAPT/AQP1/SLC6A19/PLAUR/SLC5A1/SLC7A5/SLC22A12 32

CC GO:0005775 vacuolar lumen 19/1122 173/19559 0.004997814 0.030630167 0.022710233 SCARB2/ACTR2/CAP1/DNAJC3/IDS/PSMD1/CCT8/GDI2/MAN2B2/TXNDC5/CCT2/TUBB/MANBA/LAMP2/HSPA8/ARSB/SDCBP/FABP5/HYAL1 19

CC GO:0035577 azurophil granule membrane 9/1122 58/19559 0.005477438 0.03324053 0.024645644 TMEM30A/ATP11B/RAP1B/NCSTN/SURF4/CMTM6/RAB3D/MANBA/LAMP2 9

CC GO:0035578 azurophil granule lumen 12/1122 91/19559 0.00571946 0.034372287 0.025484767 ACTR2/CAP1/DNAJC3/PSMD1/CCT8/GDI2/TXNDC5/CCT2/TUBB/ARSB/SDCBP/FABP5 12

CC GO:0042827 platelet dense granule 5/1122 21/19559 0.005794182 0.034486527 0.025569468 FAM3C/LAMP2/ITPR1/ECM1/LGALS3BP 5

CC GO:0000794 condensed nuclear chromosome 13/1122 103/19559 0.005988875 0.035305845 0.026176938 RAD1/TOPBP1/INCENP/SMC4/MLH1/BRCA1/MIS12/UBE2I/RCC1/CCN

B1/CCNB1IP1/BUB1/AURKA 13

CC GO:0032040 small-subunit processome 7/1122 39/19559 0.006141898 0.035866368 0.026592528 KRR1/XRCC5/WDR3/PRKDC/WDR36/NOL10/HEATR1 7

CC GO:0005902 microvillus 12/1122 93/19559 0.006804487 0.039364275 0.029185994 SLC26A2/MSN/MYO1B/MYO1E/EXOC4/CD302/MYO1D/ITGAV/AOC3/CD44/SLC10A2/SLC7A5 12

CC GO:0031227 intrinsic component of endoplasmic reticulum membrane 18/1122 166/19559 0.007041071 0.039919645 0.029597764 ATF6/EDEM1/PIGK/EIF2AK3/RHBDD1/INSIG2/CANX/BFAR/PIGS/EXT1/DPAGT1/ELOVL5/ELOVL1/SGPL1/ELOVL3/HM13/HSD11B1/SLC27A2 18

CC GO:1990391 DNA repair complex 7/1122 40/19559 0.00708416 0.039919645 0.029597764 MSH6/XRCC5/MSH2/PRKDC/MLH1/XRCC4/PHF21A 7

CC GO:0001650 fibrillar center 15/1122 129/19559 0.007102183 0.039919645 0.029597764 NUAK1/BTBD10/RAI14/NOLC1/MTDH/PAFAH1B2/TOP1/HEATR1/NRIP1/TERF1/UBE2N/UTP15/DKC1/HERC4/SPATA2 15

CC GO:0031201 SNARE complex 8/1122 50/19559 0.00715845 0.039919645 0.029597764 GOSR2/SNAP23/STX6/SEC22B/VAMP4/STXBP5/STX2/STX3 8

CC GO:0030117 membrane coat 12/1122 94/19559 0.007404593 0.040205642 0.029809812 SEC23A/ARCN1/COPA/SAR1A/AP4E1/AP3M1/CLTC/AP3B1/COPB1/COPB2/TMED7/TMED3 12

CC GO:0032588 trans-Golgi network membrane 12/1122 94/19559 0.007404593 0.040205642 0.029809812 RAB6A/MYO1B/AP4E1/CLTC/RAB31/STX6/COG5/RAB43/M6PR/SCAMP1/VAMP4/GOLPH3L 12

CC GO:0048475 coated membrane 12/1122 94/19559 0.007404593 0.040205642 0.029809812 SEC23A/ARCN1/COPA/SAR1A/AP4E1/AP3M1/CLTC/AP3B1/COPB1/COPB2/TMED7/TMED3 12

CC GO:0005903 brush border 13/1122 106/19559 0.007609221 0.040957459 0.030367235 SLC38A2/MYO1B/MYO1E/GNA12/GNA13/ACTR3/MYO1D/CYBRD1/CA4/AQP1/SLC6A19/SLC5A1/SLC22A12 13

CC GO:0036464 cytoplasmic ribonucleoprotein granule 23/1122 233/19559 0.007971298 0.042536494 0.031537984 ROCK2/IQGAP1/CNOT8/TAF5L/CNOT2/TOP1/KPNB1/FXR1/DCP2/STAU1/OGFOD1/TRIM5/KHSRP/EIF4G1/MBNL1/NSUN2/DCP1B/ZFP36L1/TUBB/RAC1/HABP4/CIRBP/MAPT 23

CC GO:0098858 actin-based cell projection 22/1122 220/19559

0.00805642 0.04262328 0.03160233 ITGB1/RAPH1/SLC26A2/MSN/MYO1B/MYO1E/FXR1/ARF6/EXOC4/ABI1/DAG1/CD302/MYO1D/ITGAV/MYO5A/UNC5C/ENAH/AOC3/ARL4C/CD44/SLC10A2/SLC7A5 22

CC GO:0043596 nuclear replication fork 7/1122 41/19559 0.008129379 0.042644794 0.031618281 BAZ1B/TOP1/ZRANB3/PRIM1/POLD3/SMARCAL1/WDHD1 7

CC GO:0030496 midbody 19/1122 182/19559 0.008528913 0.044364682 0.032893463 SH3GLB1/IQGAP1/GNAI3/SSH1/SPAST/YPEL5/ARF6/EXOC4/INCENP/PPP1CC/TXNDC9/ARL2BP/CYLD/SCCPDH/KIF14/CDCA8/KIF20A/CEP55/AURKA 19

CC GO:0035102 PRC1 complex 4/1122 15/19559 0.008834467 0.045194504 0.033508722 RNF2/PCGF6/CBX2/CBX7 4

CC GO:0099059 integral component of presynaptic active zone membrane 4/1122 15/19559 0.008834467 0.045194504 0.033508722 ATP2B4/CANX/ATP2B1/P2RY1 4

CC GO:0030658 transport vesicle membrane 21/1122 209/19559 0.009001098 0.045669508 0.033860905 TMEM30A/SEC23A/TMED10/SAR1A/MCFD2/GOSR2/SEC23IP/CLTC/PAM/STX6/RAB1A/SEC22B/TMED7/LMAN1/SCAMP1/VAMP4/ITPR1/TGFA/CPE/CA4/PTPRN 21

CC GO:0005875 microtubule associated complex 17/1122 159/19559 0.009902943 0.04983676 0.036950646 KIF5B/MAP4/DCTN4/STAU1/MAP1B/KIF3C/INCENP/DYNLT3/NUDCD3/MAP7/KIF14/CDCA8/MEFV/KIF20A/AURKA/LRP8/KIF2C 17

CC GO:0031233 intrinsic component of external side of plasma membrane 5/1122 24/19559 0.010495365 0.052392185 0.038845324 ADAM9/PRNP/F3/CA4/PKHD1 5

CC GO:0042383 sarcolemma 15/1122 135/19559 0.010643094 0.052704604 0.039076962 ITGB1/VCL/PPP3R1/SLC38A2/SLC30A1/ATP2B4/PPP3CA/DAG1/NCSTN/TGFB3/CORO1C/ATP1A1/ANK3/AQP1/FLNC 15

CC GO:0061695 transferase complex, transferring phosphorus-containing groups 24/1122 253/19559 0.010912008 0.053607407 0.03974633 ZNFX1/TAF5L/CDC73/ACVR1/TRRAP/TAF2/POLR3A/RB1CC1/GTF2H3/PRPS1/PIK3R1/PRIM1/POLD3/CDK2/GTF2E1/PRKAB2/TGFBR1/TCEA1/CCNJ/TAF13/IKBKG/CCNB1/PRKAB1/CCNB2 24

CC GO:0044233 mitochondria-associated endoplasmic reticulum membrane 4/1122 16/19559 0.011252738 0.053874339 0.039944242 ACSL4/CLCC1/CANX/ATG5 4

CC GO:0071437 invadopodium 4/1122 16/19559 0.011252738 0.053874339 0.039944242 ITGB1/MSN/ACTR2/PLAUR 4

CC GO:0031594 neuromuscular junction 10/1122 76/19559

0.011297499 0.053874339 0.039944242 ITGB1/CRKL/ASCC1/F2R/LRP4/STXBP5/ANK3/SERPINE2/NRG1/SPOCK1 10

CC GO:0032154 cleavage furrow 8/1122 54/19559 0.0113487 0.053874339 0.039944242 ITGB1/SSH1/ARF6/STAMBP/PPP1CC/MASTL/KIF20A/CEP55 8

CC GO:0030016 myofibril 22/1122 227/19559 0.011401516 0.053874339 0.039944242 PARVA/VCL/PDLIM5/ATP2B4/DCTN4/PPP3CA/FXR1/TPM3/FLNA/DAG1/CALD1/ABCC9/LMAN1/DST/PALLD/CORO1C/PDE4B/SORBS2/HABP4/FHL2/ANK3/FLNC 22

CC GO:0031461 cullin-RING ubiquitin ligase complex 17/1122 162/19559 0.011804167 0.055354391 0.041041603 KCTD10/CDC27/KLHL20/CAND1/USP47/ANAPC1/CUL4B/CUL2/CUL1/COP1/ENC1/KLHL2/FBXW8/FBXL7/KCTD17/ANAPC2/FBXL16 17

CC GO:0031301 integral component of organelle membrane 32/1122 367/19559 0.012324862 0.057361576 0.042529797 ATF6/TBC1D20/ZDHHC20/PEX13/EDEM1/PIGK/EIF2AK3/RHBDD1/INSIG2/CASD1/CANX/STX6/BFAR/PIGS/EXT1/TMEM43/RHOT1/VAMP4/GALNT2/LAMP2/DPAGT1/ELOVL5/ELOVL1/SGPL1/AFG3L2/RNF152/ETFDH/ELOVL3/HM13/SLC25A4/HSD11B1/SLC27A2 32

CC GO:0035770 ribonucleoprotein granule 23/1122 243/19559

0.01282571 0.059247124 0.043927805 ROCK2/IQGAP1/CNOT8/TAF5L/CNOT2/TOP1/KPNB1/FXR1/DCP2/STAU1/OGFOD1/TRIM5/KHSRP/EIF4G1/MBNL1/NSUN2/DCP1B/ZFP36L1/TUBB/RAC1/HABP4/CIRBP/MAPT 23

CC GO:0005881 cytoplasmic microtubule 10/1122 78/19559 0.013445982 0.061198993 0.045374987 CLASP1/MAPRE1/BCL10/REEP3/DYNLT3/RAB3D/CYLD/CKAP2/KIF2C/GTSE1 10

CC GO:0030684 preribosome 10/1122 78/19559 0.013445982 0.061198993 0.045374987 KRR1/XRCC5/WDR3/PRKDC/RIOK2/WDR36/NOL10/HEATR1/ZNF622/NIP7 10

CC GO:0062023 collagen-containing extracellular matrix 36/1122 427/19559 0.013601978 0.061457112 0.045566365 ATRN/LAMC1/FBN1/SPARC/COL4A1/COL15A1/NID1/LAMA4/NID2/DAG1/ECM2/COL4A2/CASK/COL12A1/LMAN1/SULF1/DST/TGFB3/ADAMTS9/F3/SERPINE1/ECM1/COL4A4/NTN4/FRAS1/TGFBI/SRPX2/SERPINE2/CTHRC1/LAMB3/APLP1/TINAGL1/WNT5A/COL10A1/TIMP1/LGALS3BP36

MF GO:0004674 protein serine/threonine kinase activity 57/1113435/18352 3.18E-08 3.07E-05 2.69E-05 ROCK2/BMPR2/MAP4K4/AKAP13/PRKD3/CSNK1A1/RPS6KC1/EIF2AK4/HIPK1/EIF2AK2/NUAK1/CSNK1G3/RPS6KB1/TAOK1/PRKDC/MAPK14/CD

KL5/PAK2/RIOK2/STK38L/KALRN/TOP1/WNK1/ACVR1/MAP2K1/MAP3K2/VRK2/EIF2AK3/SRPK1/TRIO/SRPK2/NEK7/CSNK2A1/RPS6KA3/CSNK2A2/TBK1/RAF1/CASK/CDK2/STK35/TP53RK/PRKAB2/TGFBR1/CAMKK2/MASTL/TLK1/EGFR/STK32B/STK39/MAPK10/CDC42BPG/PRKAB1/CHEK2/BUB1/HTATIP2/AURKA/CIT 57

MF GO:0044389 ubiquitin-like protein ligase binding 42/1113316/18352 1.41E-06 0.000680311 0.000595898 USP13/VCL/ATF6/XRCC5/CUL4B/BCL10/UBE2J1/UBE2G1/CCDC50/CUL2/CUL1/YWHAZ/PSMD1/SMAD1/UBE2N/RAD18/SUMO2/UBE2A/CCT2/SUMO3/RNF34/MAP1LC3B/TUBB/BRCA1/PML/EGFR/LYN/HSPA8/IKBKG/DTX3L/PARP9/ZNF746/PER3/BCL2/CCNB1/HM13/ANAPC2/CEBPB/CHEK2/AURKA/BAG1/PTPRN 42

MF GO:0017016 Ras GTPase binding 48/1113415/18352 1.30E-05 0.003378056 0.002958908 ROCK2/KCTD10/AKAP13/RAB3GAP1/IPO9/IQGAP1/TBC1D2B/TGFBRAP1/TBC1D20/TNPO1/CSE1L/CDKL5/PAK2/WDR44/CHML/KALRN/AP3M1/KPNB1/EVI5/IPO7/DOCK7/EXOC4/GDI2/DAAM2/TRIO/FLNA/ARHGEF2/CDC42SE1/OCRL/MYO5A/ARHGEF6/STXBP5/PLEKHG2/TNPO3/CORO1C/NSF/ELMO2/TBC1D22A/RAC1/RAB3IP/PLEKHG3/RCC1/GAS8/RIN1/DENND1C/ARHGEF10L/ARHGEF16/EXPH5 48

MF GO:0031267 small GTPase binding 49/1113428/18352 1.40E-05 0.003378056 0.002958908

ROCK2/KCTD10/AKAP13/RAB3GAP1/IPO9/IQGAP1/TBC1D2B/TGFBRAP1/TBC1D20/TNPO1/CSE1L/CDKL5/PAK2/WDR44/CHML/KALRN/AP3M1/KPNB1/EVI5/IPO7/DOCK7/EXOC4/GDI2/DAAM2/TRIO/FLNA/ARHGEF2/CDC42SE1/OCRL/MYO5A/ARHGEF6/STXBP5/PLEKHG2/TNPO3/CORO1C/NSF/ELMO2/TBC1D22A/RAC1/RAB3IP/RNF152/PLEKHG3/RCC1/GAS8/RIN1/DENND1C/ARHGEF10L/ARHGEF16/EXPH5 49

MF GO:0015923 mannosidase activity 7/1113 16/18352 2.09E-05 0.003421675 0.002997115 MAN1A2/EDEM3/EDEM1/MAN2B2/MAN2A1/MANBA/EDEM2 7

MF GO:0047485 protein N-terminus binding 19/1113107/18352 2.13E-05 0.003421675 0.002997115 YWHAQ/SMARCC1/PDLIM5/DCTN4/MAP2K1/ARF6/HDAC1/EXOC4/GTF2H3/CSNK2A1/CSNK2A2/BIRC2/PPP1CC/PIAS3/NBN/NCOR2/STXBP1/SDCBP/TSC1 19

MF GO:0045296 cadherin binding 40/1113332/18352 2.66E-05 0.003673666 0.003217839 PARVA/BMPR2/SH3GLB1/ITGB1/VCL/IQGAP1/KIF5B/YWHAB/MYO1B/BZW1/CRKL/MAPRE1/LRRFIP1/PAK2/EIF4G2/PDLIM5/ACVR1/CKAP5/PPME1/RAB10/PTPN11/CAST/EIF2S3/YWHAZ/CCT8/HDLBP/ABI1/FLNA/CALD1/RAB1A/NOTCH3/TXNDC9/PAICS/EGFR/HSPA8/CGN/ANK3/ARVCF/CCNB2/ARHGEF16 40

MF GO:0019902 phosphatase binding 27/1113194/18352 4.63E-05

0.005579657 0.004887335 SPRED1/CDC27/IQGAP1/PPP3R1/STAT3/SHOC2/MAPK14/ATP2B4/WNK1/PPME1/EIF2AK3/STAU1/AP3B1/PPP1R3D/PIK3R1/TBK1/PPP1CC/MASTL/EGFR/GRB2/BCL2/CRY2/SH3YL1/ERBB2/MAPT/IRS2/PPARG 27

MF GO:0031625 ubiquitin protein ligase binding 36/1113297/18352 5.83E-05 0.006255211 0.005479067 USP13/VCL/ATF6/XRCC5/CUL4B/BCL10/UBE2J1/UBE2G1/CCDC50/CUL2/CUL1/YWHAZ/PSMD1/SMAD1/UBE2N/RAD18/SUMO2/UBE2A/CCT2/RNF34/MAP1LC3B/TUBB/BRCA1/PML/EGFR/LYN/HSPA8/IKBKG/ZNF746/PER3/BCL2/HM13/ANAPC2/CHEK2/AURKA/BAG1 36

MF GO:0019903 protein phosphatase binding 22/1113149/18352 9.41E-05 0.009083987 0.007956849 CDC27/IQGAP1/STAT3/SHOC2/MAPK14/ATP2B4/PPME1/EIF2AK3/STAU1/AP3B1/PPP1R3D/PIK3R1/TBK1/PPP1CC/MASTL/EGFR/GRB2/BCL2/ERBB2/MAPT/IRS2/PPARG 22

MF GO:0003684 damaged DNA binding 13/111365/18352 0.000119933 0.010521405 0.009215913 MSH6/XRCC5/MSH2/CUL4B/DCLRE1B/RAD1/RAD18/NBN/POT1/TDG/BRCA1/UNG/CRY2 13

MF GO:0004559 alpha-mannosidase activity 6/1113 15/18352 0.000152809 0.011584249 0.010146879 MAN1A2/EDEM3/EDEM1/MAN2B2/MAN2A1/EDEM2 6

MF GO:0015924 mannosyl-oligosaccharide mannosidase activity 5/1113 10/18352 0.00015849 0.011584249 0.010146879 MAN1A2/EDEM3/EDEM1/MAN2A1/EDEM2 5

MF GO:0003725 double-stranded RNA binding 14/111376/18352 0.000168062 0.011584249 0.010146879 MSN/ZFR/EIF2AK2/LRRFIP1/MTDH/DDX21/ADAR/FXR1/STAU1/CLTC/PRKRA/MBNL1/OAS3/OAS2 14

MF GO:0031543 peptidyl-proline dioxygenase activity 5/1113 12/18352 0.000449632 0.02892633 0.025337162 EGLN1/OGFOD1/P4HA1/P4HA3/P4HB 5

MF GO:0019787 ubiquitin-like protein transferase activity 46/1113 457/18352 0.000517836 0.030885405 0.027053156 KCTD10/KLHL20/TRIP12/ANAPC1/KCMF1/RNF103/CUL4B/TRIM24/SEL1L/RNF2/UBE2J1/UBE2G1/BRAP/NEDD4/RNF4/CUL2/RNF24/RNF139/CUL1/UBE2V2/COP1/UBE2N/BFAR/TRIM5/BIRC2/RAD18/CBLB/PIAS3/UBE2A/FBXW8/UBE2Z/UBE2O/UBE2E1/HERC4/RNF34/BRCA1/PML/UBE2I/DTX3L/RNF130/LMO7/RNF152/CCNB1IP1/MEFV/NEDD4L/BSPRY 46

MF GO:0050840 extracellular matrix binding 11/1113 57/18352 0.000544095 0.030885405 0.027053156 ITGB1/SPARC/NID1/DAG1/ITGAV/ADAM9/ECM1/NTN4/TGFBI/DMBT1/TINAGL1 11

MF GO:0004842 ubiquitin-protein transferase activity44/1113435/18352

0.000612694 0.032516754 0.028482087 KCTD10/KLHL20/TRIP12/ANAPC1/KCMF1/RNF103/CUL4B/TRIM24/SEL1L/RNF2/UBE2J1/UBE2G1/BRAP/NEDD4/RNF4/CUL2/RNF24/RNF139/CUL1/UBE2V2/COP1/UBE2N/BFAR/TRIM5/BIRC2/RAD18/CBLB/UBE2A/FBXW8/UBE2Z/UBE2O/UBE2E1/HERC4/RNF34/BRCA1/PML/DTX3L/RNF130/LMO7/RNF152/CCNB1IP1/MEFV/NEDD4L/BSPRY 44

MF GO:0004553 hydrolase activity, hydrolyzing O-glycosyl compounds 15/111396/18352 0.000641143 0.032516754 0.028482087 GANAB/MAN1A2/EDEM3/EDEM1/CTBS/MAN2B2/MAN2A1/MANBA/NAGA/EDEM2/KL/MGAM/GBA2/GBA3/HYAL1 15

MF GO:0003779 actin binding 44/1113437/18352 0.000673922 0.032516754 0.028482087 PARVA/ITGB1/VCL/KLHL20/IQGAP1/MSN/ABL2/MYO1B/RAI14/ACTR2/STK38L/SSH1/MYO1E/CAP1/PDLIM5/ACTR3/MAP1B/TPM3/DAAM2/FLNA/DAG1/MICAL2/CALD1/MYO1D/ENC1/MYO5A/KLHL2/ENAH/DST/PALLD/EGFR/FMN1/CORO1C/PLS3/SETD3/FAM107A/P4HB/MAPT/CGN/ARPC3/PLEKHH2/FLNC/MEFV/ARPC1B 44

MF GO:0017048 Rho GTPase binding 21/1113162/18352 0.000809556 0.035058262 0.030708246 ROCK2/KCTD10/AKAP13/IQGAP1/CDKL5/PAK2/KALRN/DOCK7/DAAM2/TRIO/FLNA/ARHGEF2/CDC42SE1/OCRL/ARHGEF6/PLEKHG2/CORO1C/ELMO2/PLEKHG3/ARHGEF10L/ARHGEF16 21

MF GO:0008139 nuclear localization sequence binding 7/1113 27/18352 0.000898981 0.035058262 0.030708246 NUP98/TNPO1/NOLC1/KPNA3/KPNB1/BRAP/KPNA4 7

MF GO:0031418 L-ascorbic acid binding 6/1113 20/18352 0.000910558 0.035058262 0.030708246 EGLN1/OGFOD1/PAM/P4HA1/PLOD2/P4HA3 6

MF GO:0061608 nuclear import signal receptor activity 6/1113 20/18352 0.000910558 0.035058262 0.030708246 IPO9/TNPO1/KPNA3/KPNB1/KPNA4/TNPO3 6

MF GO:0017124 SH3 domain binding 18/1113131/18352 0.000944031 0.035058262 0.030708246 ADAM17/PTPN12/QKI/ADAM10/TP53BP2/ABI1/SYNJ2/CCDC6/CBLB/ADAM9/ENAH/FMN1/LYN/GRB2/ELMO2/ADAM12/MAPT/CIT 18

MF GO:0005518 collagen binding 12/111370/18352 0.000944575 0.035058262 0.030708246 ITGB1/DDR2/SPARC/NID1/NID2/ECM2/ADAM9/CRTAP/LOX/CD44/TGFBI/C1QTNF1 12

MF GO:0015926 glucosidase activity 5/1113 14/18352 0.00102629 0.03668038 0.032129092 GANAB/KL/MGAM/GBA2/GBA3 5

MF GO:0061650 ubiquitin-like protein conjugating enzyme activity 9/1113 44/18352 0.001108887 0.037176489 0.032563644 UBE2J1/UBE2G1/UBE2V2/UBE2N/UBE2A/UBE2Z/UBE2O/UBE2E1/UBE

2I 9

MF GO:0016798 hydrolase activity, acting on glycosyl bonds 18/1113 133/18352 0.001128029 0.037176489 0.032563644 GANAB/MAN1A2/EDEM3/IL1RAP/EDEM1/CTBS/MAN2B2/TDG/MAN2A1/UNG/MANBA/NAGA/EDEM2/KL/MGAM/GBA2/GBA3/HYAL1 18

MF GO:0045309 protein phosphorylated amino acid binding 10/1113 53/18352 0.001155746 0.037176489 0.032563644 ABL2/YWHAB/CRKL/NEDD4/PTPN11/PIK3R1/CBLB/LDLRAP1/GRB2/PTPN3 10

MF GO:0005385 zinc ion transmembrane transporter activity 6/1113 21/18352 0.001209928 0.037663879 0.032990559 SLC30A1/SLC30A6/SLC39A10/SLC30A5/SLC39A6/SLC39A11 6

MF GO:0043236 laminin binding 7/1113 29/18352 0.001420718 0.042843531 0.037527521 ITGB1/NID1/DAG1/ADAM9/ECM1/NTN4/TINAGL1 7

Table-S7: The changes of m6Aclusters, survival time and m6Ascore

id	futime	fustat	m6Ascore	sampleType	group
TCGA-CZ-4859	4.895890411	0	-2.023452241	TCGA	High
TCGA-CW-5587	6.098630137	0	-13.80938829	TCGA	High
TCGA-BP-4332	3.104109589	0	-3.764098061	TCGA	High
TCGA-CJ-4643	4.912328767	0	-8.975352216	TCGA	High
TCGA-B0-4817	2.791780822	1	29.68698721	TCGA	High
TCGA-BP-4964	5.101369863	0	1.547080697	TCGA	High
TCGA-B0-5402	3.534246575	0	-4.567357016	TCGA	High
TCGA-CJ-4891	2.243835616	1	18.00567696	TCGA	High
TCGA-B0-4690	0.117808219	1	-40.09512524	TCGA	Low

33120	TCGA-B0-4815	4.350684932	1	-6.341618915	TCGA	High
33121	TCGA-B2-5635	2.068493151	0	-5.709237552	TCGA	High
33122	TCGA-A3-3328	3.794520548	0	18.40760821	TCGA	High
33123	TCGA-CJ-4872	3.931506849	0	-1.86337382	TCGA	High
33124	TCGA-B8-A54K	1.284931507	0	-5.901755596	TCGA	High
33125	TCGA-CJ-4873	4.865753425	0	31.6044979	TCGA	High
33126	TCGA-G6-A8L7	5.843835616	0	35.771515	TCGA	High
33127	TCGA-CZ-5467	0.2	1	-14.37661907	TCGA	High
33128	TCGA-B2-5641	1.797260274	0	-12.01679003	TCGA	High
33129	TCGA-CJ-4882	5.158904110		4.792552202	TCGA	High
33130	TCGA-B8-A54G	0.145205479	0	2.255643071	TCGA	High
33131	TCGA-BP-4961	5.301369863	0	-19.95159713	TCGA	High
33132	TCGA-B0-5698	9.947945205	0	2.661109862	TCGA	High
33133	TCGA-BP-5199	3.712328767	0	-7.698345844	TCGA	High
33134	TCGA-CW-6093	8.619178082	0	-20.16037644	TCGA	High
33135	TCGA-B0-4700	5.424657534	1	-7.194080729	TCGA	High
33136	TCGA-AK-3460	6.871232877	0	6.593729761	TCGA	High
33137	TCGA-AK-3458	3.2	0	22.03875731	TCGA	High
33138	TCGA-CZ-5984	5.663013699	0	-17.13735172	TCGA	High
33139	TCGA-A3-3324	3.249315068	0	2.327424835	TCGA	High
33140	TCGA-CJ-4912	4.539726027	0	33.91237238	TCGA	High
33141	TCGA-CZ-5453	6.62739726	1	18.64852242	TCGA	High

33142	TCGA-A3-3378	1.726027397	0	-15.290841	TCGA	High
33143	TCGA-A3-3343	2.589041096	0	4.406498885	TCGA	High
33144	TCGA-CJ-4887	2.553424658	1	24.33964206	TCGA	High
33145	TCGA-DV-5574	5.523287671	0	-17.92473394	TCGA	High
33146	TCGA-CJ-4920	0.380821918	1	-12.34503028	TCGA	High
33147	TCGA-BP-5177	0.802739726	0	11.4876644	TCGA	High
33148	TCGA-B0-4842	4.723287671	1	28.13712779	TCGA	High
33149	TCGA-BP-4355	2.610958904	1	10.01527823	TCGA	High
33150	TCGA-A3-3359	6.860273973	0	-4.341108039	TCGA	High
33151	TCGA-AK-3433	9.339726027	0	32.79364715	TCGA	High
33152	TCGA-B0-4827	2.424657534	1	-6.805061876	TCGA	High
33153	TCGA-B2-A4SR	1.389041096	0	-13.43451088	TCGA	High
33154	TCGA-CJ-4874	6.254794521	0	-1.219165093	TCGA	High
33155	TCGA-B0-4699	0.301369863	1	-25.98018927	TCGA	Low
33156	TCGA-B8-5550	4.043835616	0	-24.61282393	TCGA	Low
33157	TCGA-A3-A8OW	0.884931507	0	-0.653370263	TCGA	High
33158	TCGA-B2-3923	2.717808219	0	10.28813789	TCGA	High
33159	TCGA-BP-4967	0.561643836	0	-4.346147205	TCGA	High
33160	TCGA-BP-5201	2.605479452	0	19.83594628	TCGA	High
33161	TCGA-AK-3440	7.849315068	0	30.45597769	TCGA	High
33162	TCGA-A3-3365	2.391780822	0	-13.70748936	TCGA	High
33163	TCGA-DV-5569	0.97260274	0	-7.117178761	TCGA	High

33164	TCGA-BP-4974	0.578082192	1	-11.49093149	TCGA	High
33165	TCGA-CW-5584	0.449315068	1	-10.85240414	TCGA	High
33166	TCGA-B4-5838	0.454794521	0	-23.39525006	TCGA	Low
33167	TCGA-AK-3454	2.394520548	0	25.44720232	TCGA	High
33168	TCGA-BP-4782	0.969863014	0	-9.469661475	TCGA	High
33169	TCGA-CZ-5451	5.284931507	0	3.324904501	TCGA	High
33170	TCGA-CJ-4907	4.106849315	0	-14.59750227	TCGA	High
33171	TCGA-BP-5196	2.789041096	0	-19.03294095	TCGA	High
33172	TCGA-CJ-4889	5.331506849	0	-9.341206067	TCGA	High
33173	TCGA-AK-3428	10.21369863	0	-12.45984927	TCGA	High
33174	TCGA-A3-A8CQ	0.008219178	0	-9.30405966	TCGA	High
33175	TCGA-A3-3380	1.553424658	0	3.006890326	TCGA	High
33176	TCGA-A3-3323	3.030136986	0	-14.01380611	TCGA	High
33177	TCGA-CJ-4895	3.287671233	1	-13.21324509	TCGA	High
33178	TCGA-CW-5591	6.221917808	0	-20.88303772	TCGA	High
33179	TCGA-BP-4799	3.104109589	1	18.4519321	TCGA	High
33180	TCGA-B0-4847	2.17260274	1	53.63127685	TCGA	High
33181	TCGA-CW-6087	0.112328767	1	-39.19346795	TCGA	Low
33182	TCGA-BP-4173	5.18630137	0	-15.47277537	TCGA	High
33183	TCGA-B8-4619	1.432876712	0	18.70484823	TCGA	High
33184	TCGA-BP-4176	5.356164384	0	-19.76386221	TCGA	High
33185	TCGA-BP-4768	1.095890411	0	5.153469633	TCGA	High

33186	TCGA-B8-A54I	0.410958904	0	31.38400283	TCGA	High
33187	TCGA-CJ-4884	4.819178082	0	11.32074159	TCGA	High
33188	TCGA-BP-4164	2.717808219	1	-2.717877403	TCGA	High
33189	TCGA-CZ-5466	1.876712329	0	4.795856084	TCGA	High
33190	TCGA-AK-3450	4.131506849	0	1.516432439	TCGA	High
33191	TCGA-BP-4327	0.298630137	1	19.23111386	TCGA	High
33192	TCGA-BP-5200	2.912328767	0	-10.56267214	TCGA	High
33193	TCGA-BP-4160	7.893150685	0	-10.95547921	TCGA	High
33194	TCGA-BP-5170	6.608219178	0	1.925937993	TCGA	High
33195	TCGA-CJ-6027	9.904109589	1	-16.17334028	TCGA	High
33196	TCGA-BP-4983	3.871232877	0	-38.4550807	TCGA	Low
33197	TCGA-B4-5378	0.479452055	0	-22.91568073	TCGA	Low
33198	TCGA-A3-3317	4.084931507	0	-14.0735121	TCGA	High
33199	TCGA-BP-4771	0.443835616	1	7.983519929	TCGA	High
33200	TCGA-A3-3352	1.536986301	1	-0.613346389	TCGA	High
33201	TCGA-B0-5084	0.608219178	1	8.578827912	TCGA	High
33202	TCGA-BP-4159	7.126027397	1	26.12822095	TCGA	High
33203	TCGA-B8-A54J	1.446575342	0	3.419901934	TCGA	High
33204	TCGA-CJ-4644	0.920547945	1	-7.28076717	TCGA	High
33205	TCGA-CZ-4863	5.282191781	0	-12.39216476	TCGA	High
33206	TCGA-BP-4330	5.17260274	0	-3.504283204	TCGA	High
33207	TCGA-BP-4995	3.756164384	0	-1.620549048	TCGA	High

TCGA-BP-4798	0.915068493	1	-4.592499495	TCGA	High
TCGA-B0-5700	4.904109589	0	22.35612657	TCGA	High
TCGA-AK-3456	3.131506849	0	11.33768454	TCGA	High
TCGA-CJ-4641	4.550684932	1	-14.16930205	TCGA	High
TCGA-BP-4335	1.301369863	1	27.29001473	TCGA	High
TCGA-B8-4146	1.4	0	-32.13106729	TCGA	Low
TCGA-B0-4694	0.290410959	1	-6.301029234	TCGA	High
TCGA-B0-4836	3.391780822	1	-23.27820947	TCGA	Low
TCGA-CJ-4635	3.879452055	0	-14.05872313	TCGA	High
TCGA-BP-4975	3.926027397	0	-3.642635302	TCGA	High
TCGA-B8-A54E	2.490410959	0	21.58145532	TCGA	High
TCGA-A3-3376	4.646575342	1	-24.56955283	TCGA	Low
TCGA-BP-4758	6.049315068	0	37.17255619	TCGA	High
TCGA-B0-5121	4.068493151	0	41.03344948	TCGA	High
TCGA-BP-5180	6.2	0	11.5441746	TCGA	High
TCGA-DV-5566	3.830136986	0	4.280311108	TCGA	High
TCGA-BP-4784	5.079452055	0	-24.07801087	TCGA	Low
TCGA-A3-3331	3.443835616	0	-3.23034145	TCGA	High
TCGA-CZ-5455	1.536986301	1	-22.15266003	TCGA	Low
TCGA-A3-3383	2.358904110		26.02904534	TCGA	High
TCGA-CJ-4875	9.736986301	1	45.22274276	TCGA	High
TCGA-B0-4811	3.882191781	1	54.76699865	TCGA	High

33230	TCGA-T7-A92I	0.975342466	0	-0.856282923	TCGA	High
33231	TCGA-B0-5085	2.109589041	1	68.26909484	TCGA	High
33232	TCGA-CZ-4853	2.120547945	0	13.49355548	TCGA	High
33233	TCGA-A3-A6NI	2.789041096	0	1.370788868	TCGA	High
33234	TCGA-AK-3465	1.010958904	0	34.10641994	TCGA	High
33235	TCGA-B2-4101	1.775342466	0	-8.874667108	TCGA	High
33236	TCGA-BP-5185	3.101369863	0	82.87749373	TCGA	High
33237	TCGA-AK-3427	9.816438356	0	37.26073904	TCGA	High
33238	TCGA-BP-4998	2.553424658	0	-19.1305058	TCGA	High
33239	TCGA-BP-5202	0.079452055	0	-1.251404581	TCGA	High
33240	TCGA-B8-5164	0.071232877	0	-16.87141221	TCGA	High
33241	TCGA-DV-5573	3.095890411	0	-6.095671059	TCGA	High
33242	TCGA-BP-4989	0.323287671	0	19.05465577	TCGA	High
33243	TCGA-G6-A8L8	2.989041096	1	23.83022651	TCGA	High
33244	TCGA-BP-4162	8.421917808	0	-9.744395525	TCGA	High
33245	TCGA-BP-5173	0.169863014	1	0.402496489	TCGA	High
33246	TCGA-B8-A8YJ	1.180821918	0	-22.89964651	TCGA	Low
33247	TCGA-B0-5080	0.936986301	1	-24.12330238	TCGA	Low
33248	TCGA-BP-4976	4.471232877	0	-10.33172306	TCGA	High
33249	TCGA-BP-4163	7.778082192	0	-11.11543431	TCGA	High
33250	TCGA-CJ-4899	4.18630137	0	-20.50606626	TCGA	High
33251	TCGA-BP-4977	1.243835616	0	3.810857207	TCGA	High

33252	TCGA-A3-3335	5.167123288	0	14.2868008	TCGA	High
33253	TCGA-CJ-4634	9.583561644	0	-10.10465274	TCGA	High
33254	TCGA-A3-3325	3.205479452	1	1.939540924	TCGA	High
33255	TCGA-CW-6090	6.991780822	0	-22.2385367	TCGA	Low
33256	TCGA-BP-4177	4.575342466	0	-10.92697738	TCGA	High
33257	TCGA-BP-4170	6.419178082	1	-1.692102315	TCGA	High
33258	TCGA-A3-3357	7.364383562	0	3.724783132	TCGA	High
33259	TCGA-BP-4960	5.950684932	0	34.13012232	TCGA	High
33260	TCGA-CJ-5677	2.142465753	1	-3.310118668	TCGA	High
33261	TCGA-BP-4167	7.446575342	0	18.32260071	TCGA	High
33262	TCGA-CW-6097	1.564383562	1	-39.73368875	TCGA	Low
33263	TCGA-B8-5162	0.098630137	0	-11.80206539	TCGA	High
33264	TCGA-CJ-6031	5.221917808	0	1.183328202	TCGA	High
33265	TCGA-CJ-4639	8.846575342	0	-15.37205155	TCGA	High
33266	TCGA-BP-4759	6.498630137	0	31.02580909	TCGA	High
33267	TCGA-BP-4986	2.150684932	0	-30.63735836	TCGA	Low
33268	TCGA-B0-5075	1.745205479	1	10.25296819	TCGA	High
33269	TCGA-BP-4775	5.049315068	0	-8.839725272	TCGA	High
33270	TCGA-CJ-4888	4.293150685	1	-2.344207862	TCGA	High
33271	TCGA-AK-3431	6.139726027	1	-24.74096666	TCGA	Low
33272	TCGA-BP-4338	7.832876712	0	-11.99867743	TCGA	High
33273	TCGA-B0-4701	0.652054795	1	-4.827415165	TCGA	High

33274	TCGA-B0-4718	4.871232877	0	23.15201773	TCGA	High
33275	TCGA-B0-5696	7.147945205	0	4.085950722	TCGA	High
33276	TCGA-BP-4326	4.452054795	1	-12.91126719	TCGA	High
33277	TCGA-CZ-4856	0.049315068	0	0.697155993	TCGA	High
33278	TCGA-BP-4972	4.115068493	0	-11.67453072	TCGA	High
33279	TCGA-BP-5169	0.528767123	0	49.28935929	TCGA	High
33280	TCGA-B2-5633	2.638356164	0	-15.35360559	TCGA	High
33281	TCGA-B0-5693	11.16164384	0	-11.64219367	TCGA	High
33282	TCGA-BP-4969	4.915068493	0	40.08717476	TCGA	High
33283	TCGA-BP-4165	8.320547945	0	32.94697934	TCGA	High
33284	TCGA-CJ-4870	4.104109589	0	13.33522336	TCGA	High
33285	TCGA-B8-5552	2.865753425	0	-21.14254749	TCGA	High
33286	TCGA-BP-4760	6.468493151	0	-23.97093903	TCGA	Low
33287	TCGA-B0-5113	3.219178082	0	-16.17974124	TCGA	High
33288	TCGA-BP-4340	1.539726027	1	4.303501646	TCGA	High
33289	TCGA-B8-A7U6	1.356164384	0	-5.876306259	TCGA	High
33290	TCGA-B8-5551	0.043835616	0	-28.19496362	TCGA	Low
33291	TCGA-B0-5400	4.747945205	0	12.49037601	TCGA	High
33292	TCGA-BP-4994	3.583561644	0	2.732926211	TCGA	High
33293	TCGA-B0-4824	4.539726027	1	11.08467863	TCGA	High
33294	TCGA-CJ-4871	6.638356164	0	-12.91280713	TCGA	High
33295	TCGA-B0-5690	9.293150685	0	-10.45297478	TCGA	High

33296	TCGA-B8-5549	0.531506849	0	-10.05602359	TCGA	High
33297	TCGA-DV-A4W0	6.767123288	0	-9.802496048	TCGA	High
33298	TCGA-CZ-5989	5.219178082	0	-27.0007651	TCGA	Low
33299	TCGA-B0-5081	0.991780822	1	11.28663849	TCGA	High
33300	TCGA-A3-3373	4.44109589	0	-2.599014867	TCGA	High
33301	TCGA-CZ-4860	0.564383562	1	-40.47591551	TCGA	Low
33302	TCGA-AK-3451	7.857534247	0	39.26391685	TCGA	High
33303	TCGA-BP-4351	2.657534247	0	7.529155356	TCGA	High
33304	TCGA-B2-4099	2.663013699	0	7.6345319	TCGA	High
33305	TCGA-B0-5694	1.315068493	1	13.61776285	TCGA	High
33306	TCGA-CJ-4903	4.273972603	0	5.357065636	TCGA	High
33307	TCGA-CZ-5457	7.545205479	0	-0.028132634	TCGA	High
33308	TCGA-BP-4158	9.252054795	0	14.30055191	TCGA	High
33309	TCGA-A3-3320	4.131506849	0	-8.693778247	TCGA	High
33310	TCGA-DV-5568	1.01369863	0	1.873931492	TCGA	High
33311	TCGA-CZ-5985	5.471232877	0	-19.74649105	TCGA	High
33312	TCGA-BP-4166	0.035616438	0	1.817706413	TCGA	High
33313	TCGA-AS-3777	3.391780822	0	39.08960584	TCGA	High
33314	TCGA-CZ-5456	6.635616438	0	-26.85341156	TCGA	Low
33315	TCGA-BP-5183	3.536986301	0	6.356385555	TCGA	High
33316	TCGA-CZ-4854	3.846575342	1	-10.66597266	TCGA	High
33317	TCGA-A3-3306	3.068493151	0	-4.271362779	TCGA	High

33318	TCGA-BP-4789	4.079452055	0	-16.17284997	TCGA	High
33319	TCGA-CZ-5462	0.852054795	1	-15.56990986	TCGA	High
33320	TCGA-B0-5705	12.43013699	0	1.556527245	TCGA	High
33321	TCGA-B0-4710	4.808219178	0	-7.571037903	TCGA	High
33322	TCGA-BP-5004	3.084931507	0	-11.65629101	TCGA	High
33323	TCGA-A3-3387	1.690410959	0	-25.37668819	TCGA	Low
33324	TCGA-BP-5175	2.553424658	0	47.19259227	TCGA	High
33325	TCGA-BP-4331	6.723287671	1	-7.846217634	TCGA	High
33326	TCGA-BP-4174	5.147945205	0	-15.77894048	TCGA	High
33327	TCGA-CJ-6028	4.452054795	1	-24.09265582	TCGA	Low
33328	TCGA-A3-3367	6.219178082	0	1.716431154	TCGA	High
33329	TCGA-A3-3351	2.493150685	0	-16.13839784	TCGA	High
33330	TCGA-CJ-4890	9.64109589	0	-13.4968234	TCGA	High
33331	TCGA-CW-5581	7.668493151	0	-6.404922832	TCGA	High
33332	TCGA-B8-4153	2.087671233	0	36.65525017	TCGA	High
33333	TCGA-B0-4698	0.115068493	1	-36.66308935	TCGA	Low
33334	TCGA-B0-5106	4.378082192	1	2.414703966	TCGA	High
33335	TCGA-BP-4329	2.315068493	1	6.528561906	TCGA	High
33336	TCGA-B0-4712	3.663013699	1	-17.41509906	TCGA	High
33337	TCGA-B0-4691	0.380821918	1	14.14080285	TCGA	High
33338	TCGA-CZ-5982	6.682191781	0	-18.10606573	TCGA	High
33339	TCGA-BP-4962	4.890410959	0	16.20871309	TCGA	High

33340	TCGA-DV-A4VX	4.454794521	1	23.81601612	TCGA	High
33341	TCGA-CJ-6030	6.298630137	1	-14.45927739	TCGA	High
33342	TCGA-B0-4818	1.397260274	1	12.42500425	TCGA	High
33343	TCGA-B0-5706	8.780821918	0	2.922301511	TCGA	High
33344	TCGA-BP-4973	3.791780822	0	5.302508031	TCGA	High
33345	TCGA-B8-5165	2.019178082	0	-15.84312603	TCGA	High
33346	TCGA-CJ-4901	3.97260274	0	3.355037923	TCGA	High
33347	TCGA-B0-5110	5.504109589	0	-15.32435491	TCGA	High
33348	TCGA-B0-4838	2.284931507	1	-1.483772179	TCGA	High
33349	TCGA-BP-4968	4.783561644	0	13.70763783	TCGA	High
33350	TCGA-A3-A6NN	0.008219178	0	-10.73834994	TCGA	High
33351	TCGA-B0-4814	0.460273973	1	4.473025299	TCGA	High
33352	TCGA-B0-4823	1.243835616	1	-12.02139941	TCGA	High
33353	TCGA-B0-5098	4.339726027	1	-17.56419139	TCGA	High
33354	TCGA-B0-5711	10.92876712	0	-11.0162115	TCGA	High
33355	TCGA-A3-3319	3.095890411	0	-14.2342474	TCGA	High
33356	TCGA-BP-5007	3.123287671	0	3.9990891	TCGA	High
33357	TCGA-B8-A54D	2.273972603	0	19.3342156	TCGA	High
33358	TCGA-B0-4833	6.536986301	1	31.20994965	TCGA	High
33359	TCGA-BP-4347	3.745205479	0	-0.219466289	TCGA	High
33360	TCGA-CW-5590	2.945205479	1	-7.906136115	TCGA	High
33361	TCGA-B0-4822	3.043835616	1	27.87796241	TCGA	High

33362	TCGA-B0-4703	0.498630137	1	-39.33334768	TCGA	Low
33363	TCGA-B0-5702	5.950684932	0	58.69491658	TCGA	High
33364	TCGA-CJ-4918	0.254794521	1	-12.03435396	TCGA	High
33365	TCGA-CJ-4897	9.153424658	0	-0.038417573	TCGA	High
33366	TCGA-BP-4776	1.126027397	0	36.22614635	TCGA	High
33367	TCGA-B4-5377	1	0	-12.28499668	TCGA	High
33368	TCGA-BP-4774	5.164383562	0	13.60202953	TCGA	High
33369	TCGA-B8-4143	1.942465753	1	-14.8756237	TCGA	High
33370	TCGA-CJ-5675	10.78356164	0	2.034585471	TCGA	High
33371	TCGA-B0-5399	3.865753425	0	2.880909725	TCGA	High
33372	TCGA-B0-4707	1.643835616	1	25.00834436	TCGA	High
33373	TCGA-CZ-4865	0.454794521	1	-12.68744776	TCGA	High
33374	TCGA-BP-4344	4.564383562	0	-9.843651307	TCGA	High
33375	TCGA-DV-5565	3.64109589	0	10.78100275	TCGA	High
33376	TCGA-AK-3436	9.126027397	0	-2.889255633	TCGA	High
33377	TCGA-A3-A6NJ	1.282191781	0	1.136136706	TCGA	High
33378	TCGA-BP-5191	2.649315068	0	60.6452657	TCGA	High
33379	TCGA-B8-4154	3.780821918	0	15.1798907	TCGA	High
33380	TCGA-B0-4688	0.276712329	1	-47.72753536	TCGA	Low
33381	TCGA-B0-5697	7.205479452	0	-4.545748524	TCGA	High
33382	TCGA-BP-4343	5.238356164	1	-30.23069269	TCGA	Low
33383	TCGA-MW-A4EC	1.364383562	0	7.808942348	TCGA	High

33384	TCGA-CZ-5452	4.901369863	0	9.150860529	TCGA	High
33385	TCGA-B0-5699	10.52328767	0	-2.099134656	TCGA	High
33386	TCGA-CZ-5460	7.871232877	0	17.41961089	TCGA	High
33387	TCGA-B0-4845	5.44109589	1	12.59828636	TCGA	High
33388	TCGA-MM-A563	1.619178082	0	5.989565862	TCGA	High
33389	TCGA-BP-4971	4.073972603	0	23.20842292	TCGA	High
33390	TCGA-AK-3425	9.158904110		-7.892380889	TCGA	High
33391	TCGA-CZ-4858	5.767123288	1	-36.28649669	TCGA	Low
33392	TCGA-B0-4841	0.558904111		67.54958357	TCGA	High
33393	TCGA-B0-4819	0.501369863	1	20.42295419	TCGA	High
33394	TCGA-G6-A5PC	0.663013699	1	63.7714691	TCGA	High
33395	TCGA-BP-4992	1.37260274	0	62.78341294	TCGA	High
33396	TCGA-CZ-4864	3.602739726	1	-4.959082992	TCGA	High
33397	TCGA-BP-5198	1.652054795	0	-14.11620902	TCGA	High
33398	TCGA-BP-4349	1.019178082	0	16.71062605	TCGA	High
33399	TCGA-CJ-4878	5.989041096	0	7.173953273	TCGA	High
33400	TCGA-CJ-5672	5.402739726	1	13.48070229	TCGA	High
33401	TCGA-BP-4765	5.983561644	0	-9.158992073	TCGA	High
33402	TCGA-BP-4970	1.18630137	0	-21.32954636	TCGA	Low
33403	TCGA-BP-5009	2.991780822	1	16.93712019	TCGA	High
33404	TCGA-CJ-4900	4.695890411	1	23.6843491	TCGA	High
33405	TCGA-BP-5186	1.898630137	0	-2.893112471	TCGA	High

33406	TCGA-A3-3370	6.230136986	0	-6.145004524	TCGA	High
33407	TCGA-B0-4810	1.309589041	1	-11.5008022	TCGA	High
33408	TCGA-CJ-4916	3.761643836	0	20.37917545	TCGA	High
33409	TCGA-B0-4846	3.287671233	1	-8.988154334	TCGA	High
33410	TCGA-BP-4756	1.024657534	0	22.93201048	TCGA	High
33411	TCGA-B0-5088	1.542465753	1	26.76391265	TCGA	High
33412	TCGA-B0-4837	3.775342466	1	5.226545368	TCGA	High
33413	TCGA-BP-5174	6.183561644	0	28.63050878	TCGA	High
33414	TCGA-BP-4803	0.558904110		-6.921817199	TCGA	High
33415	TCGA-B0-5097	1.821917808	0	-35.05992979	TCGA	Low
33416	TCGA-B0-5119	4.252054795	0	-3.936780205	TCGA	High
33417	TCGA-BP-4345	4.153424658	0	-13.58402875	TCGA	High
33418	TCGA-AK-3453	6.934246575	0	47.50415101	TCGA	High
33419	TCGA-BP-4342	6.180821918	1	-13.12443151	TCGA	High
33420	TCGA-B0-4844	0.857534247	1	11.11754797	TCGA	High
33421	TCGA-BP-4804	3.997260274	0	1.796185516	TCGA	High
33422	TCGA-CW-6088	8.82739726	0	-20.32199868	TCGA	High
33423	TCGA-B0-5701	6.742465753	0	-12.85309987	TCGA	High
33424	TCGA-CJ-5681	1.512328767	1	-21.7616947	TCGA	Low
33425	TCGA-B0-4843	0.876712329	1	26.56713046	TCGA	High
33426	TCGA-BP-4766	4.005479452	0	-1.52780981	TCGA	High
33427	TCGA-B8-A54H	0.701369863	0	-2.209431694	TCGA	High

33428	TCGA-B4-5836	0.38630137	0	-19.9512289	TCGA	High
33429	TCGA-B0-5117	4.405479452	0	59.76044292	TCGA	High
33430	TCGA-CJ-5680	2.104109589	1	-2.178769334	TCGA	High
33431	TCGA-CW-5589	6.515068493	0	-8.248801006	TCGA	High
33432	TCGA-BP-5000	1.542465753	0	32.99393595	TCGA	High
33433	TCGA-A3-3346	0.375342466	1	-20.20009382	TCGA	High
33434	TCGA-3Z-A93Z	1.054794521	0	6.752051485	TCGA	High
33435	TCGA-CJ-4638	1.180821918	1	47.4404416	TCGA	High
33436	TCGA-BP-5184	3.104109589	0	-1.865136806	TCGA	High
33437	TCGA-B0-5116	3.490410959	0	-5.986149484	TCGA	High
33438	TCGA-CZ-4857	3.923287671	1	-24.89831036	TCGA	Low
33439	TCGA-BP-5192	1.956164384	0	5.230900929	TCGA	High
33440	TCGA-B8-4620	2.128767123	0	-15.36331078	TCGA	High
33441	TCGA-BP-4959	7.287671233	0	4.350979177	TCGA	High
33442	TCGA-CJ-4905	4.098630137	0	-18.13819719	TCGA	High
33443	TCGA-BP-5181	4.095890411	0	-1.458519311	TCGA	High
33444	TCGA-EU-5906	0.564383562	0	-19.00590408	TCGA	High
33445	TCGA-CJ-4868	1.769863014	1	-24.32905947	TCGA	Low
33446	TCGA-B8-4148	4.164383562	0	-6.58500824	TCGA	High
33447	TCGA-CJ-4886	5.347945205	0	-13.10257209	TCGA	High
33448	TCGA-AK-3429	9.117808219	0	6.160893102	TCGA	High
33449	TCGA-CZ-5465	7.024657534	1	-2.479234947	TCGA	High

33450	TCGA-B0-4714	0.271232877	1	32.53669719	TCGA	High
33451	TCGA-AK-3461	6.073972603	0	-11.96505101	TCGA	High
33452	TCGA-B8-5159	1.978082192	0	-12.30043641	TCGA	High
33453	TCGA-A3-3358	3.580821918	0	3.633989331	TCGA	High
33454	TCGA-A3-3372	2.01369863	0	-17.37362228	TCGA	High
33455	TCGA-CJ-6032	9.969863014	0	-7.164779637	TCGA	High
33456	TCGA-B0-5703	6.153424658	0	21.18362201	TCGA	High
33457	TCGA-B8-A54F	1.421917808	0	-16.57537626	TCGA	High
33458	TCGA-B0-5095	0.671232877	1	-32.85502154	TCGA	Low
33459	TCGA-CZ-4861	1.221917808	1	-13.55370509	TCGA	High
33460	TCGA-A3-3349	3.794520548	0	-15.4820587	TCGA	High
33461	TCGA-AK-3455	1.871232877	1	5.060446373	TCGA	High
33462	TCGA-CW-5585	7.147945205	0	-0.084098905	TCGA	High
33463	TCGA-BP-4981	3.005479452	1	50.2772793	TCGA	High
33464	TCGA-BP-4337	0.005479452	1	17.62271836	TCGA	High
33465	TCGA-CJ-5686	5.583561644	0	-11.13611632	TCGA	High
33466	TCGA-A3-A8OV	0.931506849	0	19.35035813	TCGA	High
33467	TCGA-AK-3447	3.334246575	0	74.35379858	TCGA	High
33468	TCGA-CJ-4876	5.356164384	0	14.46133683	TCGA	High
33469	TCGA-B0-4834	5.726027397	1	26.77594504	TCGA	High
33470	TCGA-B0-5713	7.621917808	0	-4.417890342	TCGA	High
33471	TCGA-CJ-5671	10.92328767	0	-16.02851589	TCGA	High

33472	TCGA-CZ-5454	1.978082192	1	-3.22110052	TCGA	High
33473	TCGA-BP-4790	3.043835616	1	-21.24647463	TCGA	Low
33474	TCGA-B0-5107	2.539726027	1	15.10755423	TCGA	High
33475	TCGA-B4-5834	0.104109589	0	-17.4492049	TCGA	High
33476	TCGA-G6-A8L6	0.857534247	1	23.70305688	TCGA	High
33477	TCGA-BP-4991	3.871232877	0	-20.16038088	TCGA	High
33478	TCGA-A3-3347	4.410958904	1	-35.53368858	TCGA	Low
33479	TCGA-B2-5636	2.517808219	0	-32.89875423	TCGA	Low
33480	TCGA-B4-5844	0.019178082	0	-14.24225662	TCGA	High
33481	TCGA-BP-4801	3.079452055	0	-8.041163269	TCGA	High
33482	TCGA-A3-3307	3.934246575	0	-9.119540695	TCGA	High
33483	TCGA-B0-4821	3.369863014	1	13.50293316	TCGA	High
33484	TCGA-B0-4813	0.049315068	1	20.31784778	TCGA	High
33485	TCGA-CZ-5459	4.610958904	0	12.77921254	TCGA	High
33486	TCGA-B0-5108	4.882191781	0	-20.61475308	TCGA	High
33487	TCGA-CZ-4862	8.961643836	0	-23.95354445	TCGA	Low
33488	TCGA-MM-A564	1.663013699	0	30.57694264	TCGA	High
33489	TCGA-CJ-4881	5.517808219	0	-5.7915318	TCGA	High
33490	TCGA-B0-5077	3.608219178	1	7.562036078	TCGA	High
33491	TCGA-B0-5092	1.257534247	1	13.9388282	TCGA	High
33492	TCGA-EU-5905	0.326027397	0	-28.98876376	TCGA	Low
33493	TCGA-BP-5168	4.008219178	1	8.855881262	TCGA	High

33494	TCGA-B4-5832	0.424657534	0	-46.50043004	TCGA	Low
33495	TCGA-B8-5163	2.252054795	0	-39.18931891	TCGA	Low
33496	TCGA-DV-5575	4.736986301	0	-10.92056992	TCGA	High
33497	TCGA-CW-5580	5.380821918	1	-25.65339911	TCGA	Low
33498	TCGA-B0-5692	10.80547945	0	13.10711373	TCGA	High
33499	TCGA-CJ-4640	9.534246575	0	-7.94353473	TCGA	High
33500	TCGA-A3-3316	4.090410959	0	-14.51140198	TCGA	High
33501	TCGA-BP-4797	3.032876712	0	-7.684821297	TCGA	High
33502	TCGA-BP-5195	2.052054795	0	-9.405880711	TCGA	High
33503	TCGA-B0-5691	9.4	0	-19.05518209	TCGA	High
33504	TCGA-BP-5006	2.301369863	0	8.604632834	TCGA	High
33505	TCGA-CZ-5469	2.591780822	1	7.710767735	TCGA	High
33506	TCGA-BP-4999	3.468493151	0	-6.766453683	TCGA	High
33507	TCGA-EU-5904	1.509589041	0	-6.133227809	TCGA	High
33508	TCGA-B2-3924	2.991780822	0	3.593162544	TCGA	High
33509	TCGA-BP-5189	2.252054795	1	-20.55155647	TCGA	High
33510	TCGA-B0-5812	10.50410959	0	-19.45503301	TCGA	High
33511	TCGA-A3-3385	5.460273973	0	13.69141494	TCGA	High
33512	TCGA-BP-4787	1.315068493	1	-16.00024359	TCGA	High
33513	TCGA-BP-4325	8.120547945	0	7.01942085	TCGA	High
33514	TCGA-A3-3322	4.049315068	0	8.683087921	TCGA	High
33515	TCGA-BP-5194	1.117808219	0	-5.328202573	TCGA	High

33516	TCGA-B0-5096	0.18630137	1	-34.38589381	TCGA	Low
33517	TCGA-CZ-5986	1.021917808	0	-15.17596432	TCGA	High
33518	TCGA-B8-4151	3.558904110		13.82152615	TCGA	High
33519	TCGA-BP-4982	2.778082192	0	-15.48768141	TCGA	High
33520	TCGA-B0-4713	0.553424658	1	24.19626588	TCGA	High
33521	TCGA-CJ-4902	4.164383562	0	-7.24065285	TCGA	High
33522	TCGA-MM-A84U	1.917808219	0	27.02344843	TCGA	High
33523	TCGA-B0-4816	3.756164384	1	0.324987309	TCGA	High
33524	TCGA-A3-3311	3.263013699	1	-9.196942616	TCGA	High
33525	TCGA-CZ-4866	8.950684932	0	-5.871955029	TCGA	High
33526	TCGA-BP-5182	3.191780822	0	-6.455797544	TCGA	High
33527	TCGA-CJ-6033	0.61369863	1	-12.57160055	TCGA	High
33528	TCGA-CJ-4637	6.101369863	1	-19.34009671	TCGA	High
33529	TCGA-CJ-4885	9.454794521	0	5.241234013	TCGA	High
33530	TCGA-B8-5546	1.383561644	0	3.43154154	TCGA	High
33531	TCGA-B8-5545	4.178082192	0	-29.08687209	TCGA	Low
33532	TCGA-CJ-5676	11.14246575	0	16.55391972	TCGA	High
33533	TCGA-B0-5707	10.25753425	0	6.269160985	TCGA	High
33534	TCGA-BP-5190	2.769863014	0	39.08071549	TCGA	High
33535	TCGA-B0-5709	10.88767123	0	-26.00660087	TCGA	Low
33536	TCGA-B0-5109	1.608219178	1	-12.03029555	TCGA	High
33537	TCGA-B4-5843	0.030136986	0	-18.45862317	TCGA	High

33538	TCGA-B2-4098	0.139726027	1	21.74430954	TCGA	High
33539	TCGA-CJ-4893	2.054794521	0	-4.593549661	TCGA	High
33540	TCGA-BP-4985	2.608219178	1	-16.60106646	TCGA	High
33541	TCGA-BP-5001	3.224657534	0	55.19908512	TCGA	High
33542	TCGA-A3-3313	2.01369863	1	18.85279325	TCGA	High
33543	TCGA-BP-4169	1.920547945	1	-24.04959551	TCGA	Low
33544	TCGA-B8-5553	1.191780822	0	-18.04924577	TCGA	High
33545	TCGA-AK-3443	3.898630137	0	22.60317137	TCGA	High
33546	TCGA-B0-4696	2.37260274	1	-39.76767138	TCGA	Low
33547	TCGA-DV-5576	1.991780822	1	-13.40827384	TCGA	High
33548	TCGA-BP-4987	3.079452055	0	-2.437641513	TCGA	High
33549	TCGA-BP-4769	5.139726027	0	-21.92867275	TCGA	Low
33550	TCGA-BP-4161	7.523287671	0	-8.272330616	TCGA	High
33551	TCGA-BP-5010	2.405479452	1	33.09514077	TCGA	High
33552	TCGA-AK-3445	6.553424658	0	-16.93126288	TCGA	High
33553	TCGA-B2-4102	2.608219178	0	-7.613615887	TCGA	High
33554	TCGA-B0-5083	2.863013699	1	25.22422241	TCGA	High
33555	TCGA-B0-4697	1.583561644	1	21.68180559	TCGA	High
33556	TCGA-DV-A4VZ	1	0	12.49805847	TCGA	High
33557	TCGA-BP-4963	5.024657534	0	-4.659453317	TCGA	High
33558	TCGA-BP-4354	2.832876712	1	-49.72085662	TCGA	Low
33559	TCGA-BP-4341	4.353424658	1	30.19716266	TCGA	High

33560	TCGA-B0-5099	1.328767123	1	7.5671626	TCGA	High
33561	TCGA-B0-4839	4.490410959	1	35.01241538	TCGA	High
33562	TCGA-CJ-5678	1.57260274	1	18.73564164	TCGA	High
33563	TCGA-A3-3308	0.043835616	0	-20.7466422	TCGA	High
33564	TCGA-B0-5120	3.202739726	0	-11.26817425	TCGA	High
33565	TCGA-B0-5100	5.24109589	1	4.97512597	TCGA	High
33566	TCGA-B0-4945	5.876712329	1	-5.547613467	TCGA	High
33567	TCGA-BP-4781	5.698630137	0	-25.87260938	TCGA	Low
33568	TCGA-CJ-5684	6.112328767	0	5.173225398	TCGA	High
33569	TCGA-BP-4346	4.090410959	1	5.712719572	TCGA	High
33570	TCGA-AK-3426	2.424657534	1	30.66950686	TCGA	High
33571	TCGA-CJ-5682	10.23561644	0	-1.679015987	TCGA	High
33572	TCGA-A3-A6NL	1.887671233	0	-10.93054487	TCGA	High
33573	TCGA-CZ-5987	1.219178082	1	7.731010284	TCGA	High
33574	TCGA-A3-3382	1.57260274	0	-41.1180635	TCGA	Low
33575	TCGA-CJ-4636	5.271232877	0	7.178256997	TCGA	High
33576	TCGA-CW-5583	6.819178082	0	-16.66263779	TCGA	High
33577	TCGA-B0-4849	0.189041096	1	7.329549895	TCGA	High
33578	TCGA-B0-4852	3.071232877	1	0.362736099	TCGA	High
33579	TCGA-B8-4621	2.158904110	13.48934015	TCGA	High	
33580	TCGA-BP-4807	0.578082192	0	-13.79080528	TCGA	High
33581	TCGA-CJ-4908	4.194520548	0	-16.81910197	TCGA	High

33582	TCGA-BP-4352	0.942465753	1	5.066278909	TCGA	High
33583	TCGA-AK-3434	5.717808219	0	11.48522714	TCGA	High
33584	TCGA-CJ-5683	5.175342466	0	6.685787339	TCGA	High
33585	TCGA-A3-3326	3.115068493	0	8.328263055	TCGA	High
33586	TCGA-BP-4353	1.02739726	1	-29.31450444	TCGA	Low
33587	TCGA-GK-A6C7	0.167123288	0	-14.76142914	TCGA	High
33588	TCGA-BP-4761	0.498630137	0	23.43184355	TCGA	High
33589	TCGA-A3-3329	4.449315068	0	4.487478214	TCGA	High
33590	TCGA-A3-3374	3.6	0	42.88876287	TCGA	High
33591	TCGA-CZ-5470	1.057534247	0	9.02612566	TCGA	High
33592	TCGA-CZ-5463	1.81369863	0	-11.98690881	TCGA	High
33593	TCGA-BP-4965	5.126027397	0	-19.82139847	TCGA	High
33594	TCGA-B4-5835	0.043835616	0	-0.362159597	TCGA	High
33595	TCGA-B0-5094	0.912328767	1	-16.44459358	TCGA	High
33596	TCGA-DV-5567	5.490410959	0	-19.6636187	TCGA	High
33597	TCGA-BP-4334	1.767123288	1	70.81189169	TCGA	High
33598	TCGA-BP-5187	1.112328767	0	-11.76724012	TCGA	High
33599	TCGA-CZ-5988	1.898630137	0	-32.40021036	TCGA	Low
33600	TCGA-CJ-4894	2.304109589	1	-6.427664898	TCGA	High
33601	TCGA-BP-4763	3.479452055	1	-27.53277954	TCGA	Low
33602	TCGA-B0-4828	0.84109589	1	25.66603566	TCGA	High
33603	TCGA-CJ-5679	1.860273973	1	6.239888849	TCGA	High

33604	TCGA-CJ-4869	6.997260274	0	9.815263319	TCGA	High
33605	TCGA-A3-3363	0.873972603	0	3.101787906	TCGA	High
33606	TCGA-BP-4777	4.742465753	0	10.51623198	TCGA	High
33607	TCGA-B8-5158	3.336986301	0	-27.39951229	TCGA	Low
33608	TCGA-B0-5695	5.890410959	0	1.024012672	TCGA	High
33609	TCGA-BP-5176	4.356164384	1	16.49494129	TCGA	High
33610	TCGA-BP-4993	0.484931507	0	1.354889448	TCGA	High
33611	TCGA-BP-4795	1.698630137	0	-22.30881213	TCGA	Low
33612	TCGA-CW-5588	5.526027397	0	-24.0670698	TCGA	Low
33613	TCGA-CZ-5464	5.830136986	0	-3.252829829	TCGA	High
33614	TCGA-BP-4762	3.679452055	1	-19.34635932	TCGA	High
33615	TCGA-BP-5178	5.238356164	1	21.24856237	TCGA	High
33616	TCGA-B0-5115	4.394520548	0	-5.28855646	TCGA	High
33617	TCGA-B8-4622	4.178082192	0	-5.234808137	TCGA	High
33618	TCGA-B0-5102	7.57260274	1	14.71253349	TCGA	High
33619	TCGA-BP-5008	2.934246575	0	-11.58607134	TCGA	High
33620	TCGA-CJ-4642	8.780821918	0	25.23830214	TCGA	High
33621	TCGA-A3-3362	4.271232877	0	-2.325570565	TCGA	High
33622	TCGA-B2-5639	2.747945205	1	2.539948021	TCGA	High
33623	TCGA-CZ-5458	7.64109589	0	-4.992880041	TCGA	High
33624	TCGA-AS-3778	0.117808219	0	-4.578735853	TCGA	High
33625	TCGA-B0-4693	0.210958904	1	-2.957817334	TCGA	High

33626	TCGA-CJ-4892	4.167123288	0	7.465764945	TCGA	High
33627	TCGA-B0-5712	7.457534247	0	13.46656015	TCGA	High
33628	TCGA-B0-4706	0.178082192	1	18.7587185	TCGA	High
33629	TCGA-CZ-5461	0.904109589	1	-19.0649563	TCGA	High
33630	TCGA-6D-AA2E	0.991780822	0	25.94376674	TCGA	High
33631	TCGA-EU-5907	0.347945205	0	-19.63957802	TCGA	High
33632	TCGA-BP-4770	0.901369863	1	-30.46317096	TCGA	Low
33633	TCGA-B0-4848	2.419178082	1	-25.81472334	TCGA	Low
33634	TCGA-CZ-5468	0.161643836	1	-22.01721856	TCGA	Low
33635	TCGA-CJ-4904	9.046575342	0	-14.23749122	TCGA	High
33636	TCGA-B0-5710	6.657534247	0	-19.56032124	TCGA	High
33637	GSM733580	3.287671233	1	-8.776805119	GSE29609	High
33638	GSM733582	0.082191781	1	8.276080379	GSE29609	High
33639	GSM733584	3.698630137	1	6.249172939	GSE29609	High
33640	GSM733588	3.863013699	0	-7.721441929	GSE29609	High
33641	GSM733589	2.547945205	0	19.4087866	GSE29609	High
33642	GSM733590	3.534246575	0	-12.5581714	GSE29609	High
33643	GSM733591	5.095890411	0	-1.28153985	GSE29609	High
33644	GSM733593	3.616438356	0	1.861836067	GSE29609	High
33645	GSM733594	3.205479452	0	19.75352128	GSE29609	High
33646	GSM733595	0.575342466	1	3.311613271	GSE29609	High
33647	GSM733596	4.438356164	1	-12.69757141	GSE29609	High

33648	GSM733597	5.589041096	0	0.441564518	GSE29609 High
33649	GSM733598	0.821917808	1	2.215409378	GSE29609 High
33650	GSM733599	0.493150685	1	7.119794398	GSE29609 High
33651	GSM733600	4.356164384	1	2.147979521	GSE29609 High
33652	GSM733601	4.356164384	0	-11.37698085	GSE29609 High
33653	GSM733602	2.383561644	0	-12.66785971	GSE29609 High
33654	GSM733603	4.602739726	0	0.860790754	GSE29609 High
33655	GSM733604	2.301369863	1	-4.885648974	GSE29609 High
33656	GSM733605	3.369863014	0	-4.960259656	GSE29609 High
33657	GSM733606	4.191780822	0	-11.77274171	GSE29609 High
33658	GSM733607	1.643835616	1	1.493304229	GSE29609 High
33659	GSM733608	5.01369863	0	-14.39414194	GSE29609 High
33660	GSM733609	4.109589041	0	-13.31652231	GSE29609 High
33661	GSM733610	1.479452055	1	10.59454556	GSE29609 High
33662	GSM733612	0.164383562	1	1.803292115	GSE29609 High
33663	GSM733613	4.684931507	0	-0.064434522	GSE29609 High
33664	GSM733614	1.561643836	1	-1.402124028	GSE29609 High
33665	GSM733615	6.246575342	0	9.943691961	GSE29609 High
33666					
